# High density linkage mapping of genomic and transcriptomic SNPs for synteny analysis and anchoring the genome sequence of chickpea

**DOI:** 10.1038/srep13387

**Published:** 2015-08-25

**Authors:** Rashmi Gaur, Ganga Jeena, Niraj Shah, Shefali Gupta, Seema Pradhan, Akhilesh K Tyagi, Mukesh Jain, Debasis Chattopadhyay, Sabhyata Bhatia

**Affiliations:** 1 National Institute of Plant Genome Research, Aruna Asaf Ali Marg, Post Box No. 10531, New Delhi 110067, India

## Abstract

This study presents genome-wide discovery of SNPs through next generation sequencing of the genome of *Cicer reticulatum.* Mapping of the *C. reticulatum* sequenced reads onto the draft genome assembly of *C. arietinum* (desi chickpea) resulted in identification of 842,104 genomic SNPs which were utilized along with an additional 36,446 genic SNPs identified from transcriptome sequences of the aforementioned varieties. Two new chickpea Oligo Pool All (OPAs) each having 3,072 SNPs were designed and utilized for SNP genotyping of 129 Recombinant Inbred Lines (RILs). Using Illumina GoldenGate Technology genotyping data of 5,041 SNPs were generated and combined with the 1,673 marker data from previously published studies, to generate a high resolution linkage map. The map comprised of 6698 markers distributed on eight linkage groups spanning 1083.93 cM with an average inter-marker distance of 0.16 cM. Utility of the present map was demonstrated for improving the anchoring of the earlier reported draft genome sequence of desi chickpea by ~30% and that of kabuli chickpea by 18%. The genetic map reported in this study represents the most dense linkage map of chickpea , with the potential to facilitate efficient anchoring of the draft genome sequences of desi as well as kabuli chickpea varieties.

High density linkage maps are fundamental for facilitating molecular breeding applications and elucidating genetic mechanisms for agronomically important traits. Currently, with the large number of plant genomes being sequenced, one of the most important applications of high density maps is for anchoring and orienting scaffolds arising from whole genome sequence data. Hence recently, for a wide variety of species, high density maps have been developed utilizing large numbers of molecular markers ranging from 1,000 to about 15,000, primarily simple sequence repeats (SSRs) and single nucleotide polymorphisms (SNPs) in species such as potato (1; 10,000 loci), *Brassica* (2; 13,551 loci), cotton (3; 8,254 loci), sunflower (4; 10,080 loci), and lettuce (5; 13,943 loci). High density maps have now become possible due to the recent advances in sequencing technologies that have accelerated the discovery of sequence variations such as SNPs in large numbers at the whole genome scale. Recently, SNPs ranging from 14,000 to 3 million, have been identified in different crops including soybean^6^, rice^7^, *Medicago*^8^ and Quinoa^9^ and have been successfully utilized in genome-wide association studies^10^, evaluation of allelic variation in breeding germplasm^11^,^12^,^13^, generation of high density genetic maps^14^,^15^, anchoring and orienting scaffolds arising out of whole genome sequencing^16^,^17^,^18^,^19^ and comparisons of synteny^20^,^21^.

Chickpea (*Cicer arietinum* L.), with a genome size of 740 Mb (2n = 2x = 16), is the third most important legume crop and is comprised of two main types i.e. the desi and the kabuli. These two types are different in their morphology as desi chickpea, which is the progenitor of kabuli, has purple flowers and small, dark and angular seeds, while kabuli chickpea has white flowers and large, cream-coloured seeds. Development of high throughput genomic resources to complement the ongoing efforts on genetic enhancement is required to improve the productivity, nutritional quality and stress tolerance of this important legume crop. Chickpea genomics has witnessed rapid advances in the current decade where assessment of genetic variation for the development of various kinds of molecular markers was carried out. Initially SSR markers gained more importance and were considered as one of the most reliable markers for diversity analysis^22^, QTL identification^23^ and construction of genetic maps^24^,^25^,^26^,^27^,^28^. However, recent advancements in chickpea have reported the large scale discovery and genotyping of SNPs in chickpea^29^,^30^,^31^. These advancements were complemented with the release of the draft genome sequences of two major chickpea types i.e. desi [*Cicer arietinum* ICC4958]^32^ and kabuli [*C. arietinum* CDC Frontier]^33^. The draft assemblies of these two varieties covered similar genome fractions (~60%) of the estimated genome length^34^. However, in case of the kabuli assembly, 65.23% of the sequenced genome could be placed into eight pseudomolecules whereas in the desi assembly, only 23.93% of the sequenced genome was anchored to the eight pseudomolecules. The desi assembly previously reported had been based on the genetic map reported earlier by our group^29^ which was a low marker resolution map with only 1063 markers. Therefore, for improving the percentage of the anchored genome of desi cultivar, there was an urgent need to develop and utilize a high density linkage map of chickpea.

This study was undertaken with the objective of identifying a large number of SNPs from the genome sequence of 2 genotypes i.e. the cultivated *C. arietinum* ICC4958 and the wild species *C. reticulatum* PI489777, parents of the reference mapping population. Further, conversion of the genomic SNPs generated here and the transcriptomic SNPs reported earlier^29^ in to successful genotyping assays by developing two new chickpea Illumina based oligo pool all assays (OPAs; CpOPA-II and CpOPA-III) was demonstrated. Next, the SNP resources were used to construct the most advanced high-density linkage map of chickpea with increased genome coverage and marker density. Moreover, the utility of the present map was established for improving the anchoring of scaffolds from the draft chickpea genome sequence^32^,^33^.

## Results

### Identification of SNPs from chickpea whole genome and transcriptome sequences

In this study, large scale identification of SNPs was carried out both from the genome as well as the transcriptome sequences of chickpea. For identification of genomic SNPs, the whole genome sequences of two chickpea genotypes namely the cultivated *C. arietinum* ICC4958 and the wild progenitor *C. reticulatum* PI489777 were compared. Sequencing of the wild progenitor *C. reticulatum* PI489777 was carried out using SOLiD platform and the generated reads were trimmed and filtered to obtain 61,47,031 filtered reads comprising of 1.833 Gb sequence data. For SNP identification, the *C. reticulatum* reads were aligned to the published draft reference assembly of the desi chickpea (ICC4958)^32^. Only 57.82% of total reads were uniquely mapped and were retained, whereas the reads mapping to multiple sites were discarded. A total of 275,934,783 bases were mapped to unique positions on the reference desi genome (ICC4958) and were used for SNP calling. This resulted in the detection of a total of 8,42,104 unique SNPs at non-repetitive sites between ICC4958 and PI489777 genome sequences. An average SNP density of 1.13 SNPs per kb was observed in this study, when considering the chickpea genome size to be 740 Mb. Moreover, the frequency of transitions [432656; 63.5%] was significantly higher than transversions [248270; 36.5%] with SNP transition to transversion ratio of 1.74.

Further, transcriptomic SNPs were also utilized for linkage mapping in this study. An earlier study^35^ comparing the transcriptomes of the same cultivars used above had reported 36,446 SNPs (distributed in 10,880 transcripts) of which, 3,113 SNPs were selected for genotyping by GoldenGate assay in this study.

### SNP genotyping using Illumina GoldenGate Genotyping Technology (GGGT)

Genotyping of 129 recombinant inbred lines (RILs) of the interspecific reference mapping population [*C. arietinum* ICC4958 x *C. reticulatum* PI489777] of chickpea was carried out using the GGGT from Illumina. For the purpose of genotyping, both genomic and transcriptomic SNPs were utilized by designing an Oligo Pool All (OPA) assay for each of them. Hence, for designing the CpOPA-II, 3237 genomic SNPs were evaluated by ADT and finally 3072 SNPs having ADT scores above 0.4 were selected thereby revealing a successful conversion rate of 94.9%. Similarly, 3072 out of 3113 high quality transcriptomic SNPs were utilized to design the CpOPA-III. The genomic SNPs were designated as CaSNP1919-CaSNP5155 whereas the transcriptomic SNPs ranged from CaTSNP6001-CaTSNP9160 and are described in Table S1.

Genotyping data of 129 RILs at 6144 SNP loci (3072 from each of CpOPA II and III) were generated and downstream analysis was carried out using Illumina’s Genome Studio software (Illumina, San Diego, CA). In CpOPA-II, 2506 polymorphic SNPs were obtained after excluding 385 homozygous and 181 technically unsatisfactory SNPs since they had GenTrain and GenCall50 score <0.40, and ‘no-call’ frequencies >5%. Similarly in CpOPA-III, 2535 polymorphic SNPs were obtained after excluding 101 homozygous and 286 technically unsatisfactory SNPs. In total, 5041 (2506 genomic and 2535 transcriptomic) SNPs were found to be successfully polymorphic thereby demonstrating an overall success rate of 82.05% for the Illumina GGGT in chickpea.

### Construction of the chickpea genetic linkage map

**Figure 1:**
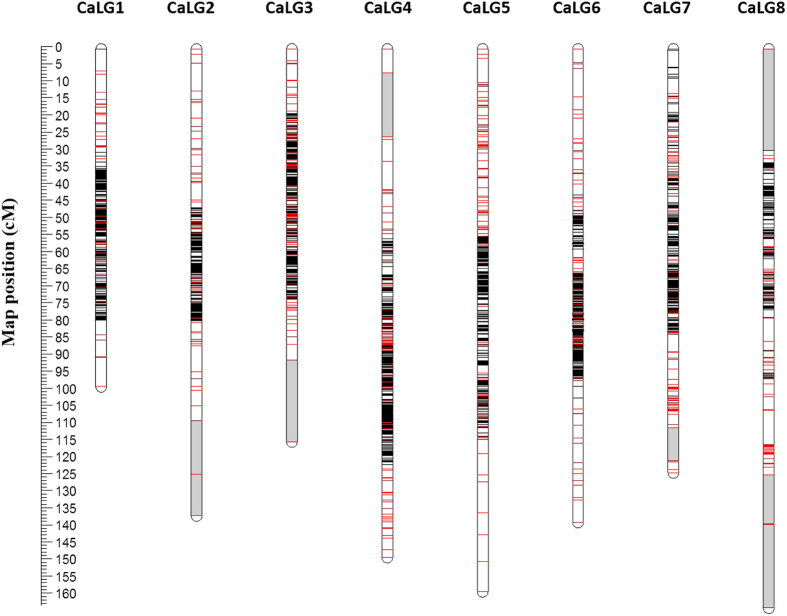
The high density genetic linkage map of chickpea: The inter-specific linkage map of chickpea based on RILs of *C. arietinum* (ICC4958) × *C. reticulatum* (PI489777) harbouring 6698 loci. The name of the linkage groups is mentioned at the top of each LG. SNP markers are represented in black, while red colour is shown for markers other than SNPs. Large gaps with >10 cM length were observed at the proximal ends of different LGs (CaLG2, CaLG3, CaLG4, CaLG7 and CaLG8) and are represented in grey shade.

The 5041 polymorphic SNPs were utilized for construction of the genetic linkage map of chickpea. Of these, loci that contained >25% missing data were excluded and hence data of 5013 high quality SNPs were considered for further analysis. Along with this, the genotyping data of 1065 markers (including 697 SNPs from chickpea CpOPA-I, 238 genomic SSRs and 130 genic markers) from our previous study^29^ and 636 SSR markers from Khajuria *et al.*^36^ were also included in the linkage analysis (Table 1). Hence, genotyping data of a total of 6714 markers across 129 RILs involving 866,106 (~0.8 million) data points were employed to construct the linkage map. The resulting map of chickpea defined map positions of 6698 (99.76%) markers distributed over 8 linkage groups at 5030 unique positions (Fig. 1; Table S2) thereby validating the high quality of the data utilized in the present study.

The present map spanned 1083.93 cM with an average inter-marker distance of 0.16 cM. The map contained an average of 9.05 map positions per Mbp of genome (6698 map positions/740 Mbp) and represented an average physical interval of 110.48 kb/marker (740 Mbp/6698). The LGs of the present map were designated as CaLG1 to CaLG8 based on previously mapped markers^24^,^28^,^29^ to maintain consistency with the published maps of chickpea. The genetic length of the LGs ranged from 98.798 cM (CaLG1) to 163.633 cM (CaLG8) (Table 2). Most of the LGs (CaLG1, CaLG3, CaLG4, CaLG5, CaLG6 and CaLG7) were highly saturated having average marker density below 0.2 cM with number of markers ranging from 778 to 1050. On an average, one linkage group contained 837.25 markers and spanned an average of 135.49 cM of genetic length. Moreover, all categories of markers including SSRs, ITPs, ESTPs and SNPs were found on each of the LGs. The number of SNP markers in each of the LGs varied from 323 to 942 (Table 2). Moreover, of the mapped loci, 35.32% (2366) were assigned into 726 bins leaving 64.67% (4332) as singletons or individual loci. The average number of loci per bin was 3.25 (Table 2).

**Figure 2:**
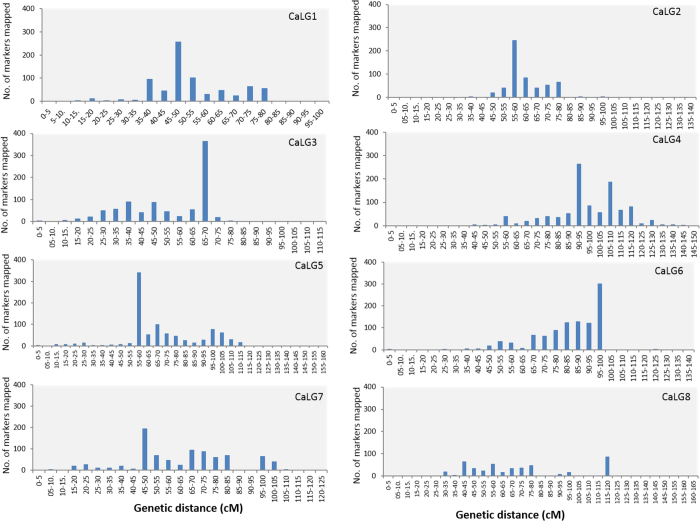
Graphical representation of frequency distribution of loci at intervals of 5 cM of genetic distance on LGs of the present chickpea map. Major hot spot regions ranging from 88 to 367 loci per 5 cM are represented as bar lengths.

The distribution of markers was not uniform across the LGs as some high and low marker density regions were observed in the present map. Despite having such a high density, eight large gaps of >8 cM including 4 gaps with >15 cM length were observed at the proximal ends of linkage groups CaLG2, CaLG3, CaLG4, and CaLG8 (Fig. 1; Table 2). In contrast, several regions with high marker density were observed on different LGs (CaLG3, CaLG4, CaLG5, CaLG6 and CaLG7). A total of 219 clusters were identified on the 8 LGs that contained >5 markers per cM. These included 45 highly dense regions harbouring >50 markers per 5 cM ranging from 51 to 367 markers (CaLG3). The marker density across LGs expressed as markers per 5 cM of genetic distance is depicted in Fig. 2 which revealed major hot spot regions on CaLG1 to CaLG7 with the highest having up to 367 markers within 5 cM (Fig. 2).

In the χ^2^ test analysis, significant deviations from the expected 1:1 Mendelian ratios in haploid progeny were observed which revealed that 38.9% (2615) of the markers showed distortions (*P* < 0.05). However, 2599 (99.4%) of these markers were finally integrated into the map so that the loss of genetic information related to these markers was minimized. Majority (74.8%) of them were skewed towards the wild parent, PI489777 (female) and were distributed mainly across CaLG1, 2, 5 and 6. These distorted markers were distributed throughout all the LGs, although the ratios varied from one LG to another. Moreover, these markers were found in clusters comprising of 2 to 401 markers in regions ranging from 0 to 10.288 cM in size.

### Anchoring scaffolds of the desi chickpea genome assembly

Our group had recently published the draft genome assembly of the desi chickpea^32^ in which the 15X coverage assembly spanned 520 Mb distributed across 181,462 scaffolds. This assembly had been anchored using a 1063 marker linkage map^26^ which had facilitated anchoring of 532 scaffolds spanning only 124.38 Mb (23.93%) of the genome (v1.0). Therefore, the currently generated high resolution 6698 marker map was utilized to greatly improve the anchoring of the scaffolds. For this, the sequences of mapped loci were aligned to the desi chickpea genome assembly and 5887 marker sequences found hits, of which 2598 (44.13%) were positioned on the 8 chickpea pseudomolecules, while 3289 (55.86%) were dispersed in 1901 unassembled scaffolds. Utilizing this information for anchoring resulted in the successful alignment of 2674 scaffolds covering 279 Mb (53.67%) of the assembled genome (Table 3). The numbers of scaffolds anchored to each of the LGs was significantly improved and ranged in numbers from 119 (CaLG8) to 430 (CaLG6) (Table 3).

### Comparative analysis of desi chickpea with kabuli chickpea and related legumes

**Figure 3:**
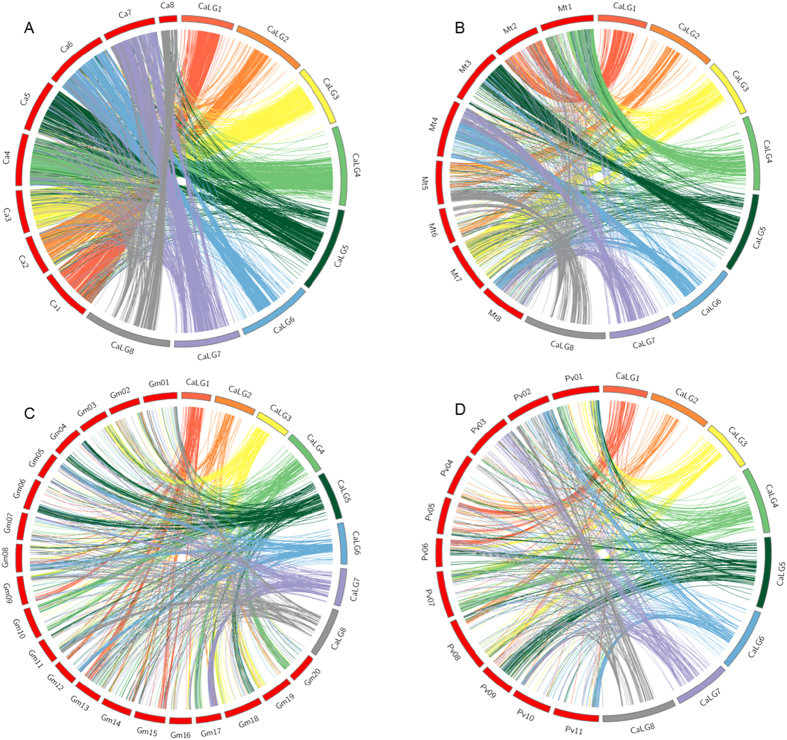
Syntenic relationship of desi chickpea with (**A**) Kabuli chickpea (**B**) *Medicago truncatula* (**C**) *Glycine max* and (**D**) *Phaseolus vulgaris*. Links connect the locations of homeologs between genomes, based on the comparison of sequence information of mapped markers on chickpea linkage map with genome sequence of *Medicago*, soybean and *P. vulgaris*.

The sequence information of the 6698 markers mapped in this study was utilized in BLASTN analysis with the kabuli genome^33^ which revealed that 6548 (97.76%) markers mapped to the kabuli genome. The mapped markers included 5742 (87.69%) markers that mapped to the eight kabuli pseudomolecules and 806 (12.3%) that mapped to 344 unassembled scaffolds. The unassembled scaffolds spanned a total of 96.0 Mb of the kabuli genome which could be anchored and placed into the pseudomolecules thereby greatly improving the coverage of the published draft assembly of kabuli genome from 347.247 Mb (65.22%) to 443.247 Mb (83.27%) of the sequenced genome fraction. Further, chromosome wise distribution of the 5742 mapped markers across the pseudomolecules of desi (CaLG1-8; Table 4) and kabuli (Ca1-8; Table 4) revealed that, 5062 (88.15%) markers had common locations on the corresponding pseudomolecules of the desi and kabuli genomes, respectively (Fig. 3A; Table 4). For example, in the case of the mapped markers on pseudomolecule1, 689 markers were commonly present both in the desi CaLG1 as well as in the kabuli Ca1. Moreover, the Circos plot depicting synteny between desi and kabuli chickpea (Fig. 3A) showed near-perfect synteny between the two genotypes with about 95% syntenic loci in the case of pseudomolecules 1 (CaLG1-Ca1) and 6 (CaLG6-Ca6) (Table 4). However only in the case of pseudomolecule 8 (CaLG8-Ca8) synteny levels were low (69.17%). The Circos plot also revealed that despite the high synteny, certain regions of same pseudomolecules did not find a match with the corresponding pseudomolecules. For example, markers from the desi CaLG5 and CaLG7 found syntenic locations on other non-corresponding pseudomolecules of kabuli. Inverted regions were also observed in some pseudomolecules such as CaLG3, CaLG4 and CaLG7.

The level of synteny between chickpea and three other legumes i.e. *Medicago truncatula, Glycine max* and *Phaseolus vulgaris* was assessed. From BLAST analysis, maximum hits were obtained with *M. truncatula*, followed by *G. max* and *P. vulgaris*. In chickpea vs. *Medicago* synteny (Fig. 3B; Table S3), BLAST hits were obtained for 29.5% (1936) of the sequences. Majority of the markers on CaLG1 found hits on Mt2 (Fig. 3B; Table S3). In chickpea vs. soybean synteny (Fig. 3C; Table S3), BLAST hits were obtained for 16.24% (1066) of the sequences, of which majority of the markers belonged to CaLG4 and found hits on Gm10, Gm17 and Gm20. Further, in the chickpea vs. *P. vulgaris* synteny, hits were found against 783 (11.93%) markers of which maximum hits were obtained for markers located on CaLG4, CaLG5, CaLG6 and CaLG7 (Fig. 3D; Table S3).

## Discussion

The advent of NGS technologies has enabled the development of sequence-based markers by comparison of two or more genome sequences. SNPs represent the most abundant DNA sequence variation and are well chronicled for use in high-resolution genetic mapping and association studies. In the present study, we detected ~0.8 million SNPs between cultivated (ICC4958) and wild (PI489777) chickpea genome sequences. Genome wide identification of SNPs in large numbers, ranging from 27,658 to 6,318,109, has been well documented in plant species^6^,^7^,^8^,^15^. The average SNP density of 1.13 SNPs per kb observed in this study was higher than in tomato (0.6 SNPs/kb^37^) and flax (0.17 SNPs/kb^38^) and lesser than potato (11.5 SNPs/kb^39^), maize (8.9 SNPs/kb^40^) and rice (6.78 SNPs/kb^41^). Such large variations in SNP density in different genomes may be attributed to a number of factors, primarily including the methodology used for SNP discovery, criteria used for SNP mining and the nature and genetic proximity of genotypes/accessions compared for SNP identification. An analysis of the transitions and transversions revealed a higher frequency of transitions (63.5%) than transversions (36.5%) with SNP transition to transversion ratio of 1.74. This phenomenon, known as transition bias, is a general property of DNA sequence evolution^42^ which favours tolerance of transitions during natural selection^41^ that are less likely to result in amino acid substitutions (due to “wobble”), and therefore persist as “silent substitutions” in populations as SNPs^41^,^42^.

The availability of large numbers of SNPs has necessitated the simultaneous development of various high-throughput genotyping platforms that allow cost-effective genotyping of thousands of SNPs in parallel to facilitate construction of high-density linkage maps. Illumina’s GGGT is one such platform which was used for genotyping 129 RILs in chickpea. A conversion rate of 98.68% was obtained which was comparable with 89.0 to 92.0% conversion rate in barley, soybean and maize^43^,^44^,^45^ and was considerably higher than the 66.9% in *Picea*^46^ and 82% in *Pinus spp.*^47^. After downstream analysis, an overall success rate of 82% (polymorphic SNPs) was obtained. The success rate obtained in this study was low as compared to the 90.75% success reported in our previous study of chickpea^29^ but was comparable with the 81% success rate reported in another study of chickpea in which KASPar assay was utilized for high-throughput SNP genotyping^30^.

Genetic linkage maps, based on pair-wise distance estimates, have emerged as pivotal tools for locating genes or QTLs. The analysis of recombination events from marker segregation data is especially helpful when a large number of markers segregate in a single mapping population. But mapping larger number of markers also exponentially increases the potential orders of these loci on a chromosome. Hence, advanced and efficient algorithms are required to achieve near perfect ordering of large number of loci. REcombination Counting and ORDering (RECORD)^48^ is a faster, more accurate method for ordering of loci on genetic linkage maps and performs especially well in regions of map with high marker density. Therefore, in this study, utilizing the RECORD, the best marker order in each LG was obtained and generated a highly dense map of chickpea with average marker density of 0.16 cM and average of 9.05 map positions per Mb of chickpea genome. In the past, several genetic maps of chickpea^24^,^25^,^26^,^27^,^28^,^29^,^30^,^31^ have been developed but a linkage map of such high resolution, as reported here, was not available. Thus, it represented the most saturated map of chickpea till date especially with respect to the total number of markers mapped and the average marker density in comparison to the published chickpea maps [(303 markers; AMD of 6.8 cM^24^), (521 markers; AMD of 4.99 cM^25^), (406 markers; AMD of 3.68 cM^28^), (1291 markers; AMD of 0.65 cM^27^), (1063 markers; AMD of 1.70 cM^29^), (1328 markers; AMD of 0.59 cM^30^)] and (1336 markers; AMD of 0.5 cM^31^)].

In addition, the present map revealed locations of several candidate genes as 3112 (46.46%) of the mapped markers were derived from the transcriptome and included 2584 (83.03%) SNPs and 528 (16.96%) EST-SSR and ESTP markers. Functional annotation of these sequences revealed that the most represented classes in each GO category were ‘cellular processes’, ‘cell part’ and ‘binding activity’ (Fig. S1). Specifically, in biological processes, 167 transcripts were found to be involved in developmental processes whereas in molecular function category, 91 putative transcription factors belonging to different TF families (NAC, followed by ERF, B3 domain containing protein, SCARECROW-like protein and homeobox-leucine zipper) were identified.

An uneven distribution of markers along LGs was observed where both large gaps (>10 cM in length) as well as marker hot spots (accommodating upto 367 markers within 5 cM) existed due to the uneven distribution of DNA polymorphism along chromosomes or due to the variation in rates of recombination along the chromosomes i.e.. higher recombination rates at the telomeric regions and suppression of recombination near the heterochromatin region. This was evident from the current map of chickpea (Fig. 1) as well as the plots of LGs (Fig. 2) and also consistent with the previously published maps of chickpea and other plant species such as tomato^49^,^50^, barley^51^ etc.

Segregation distortion is a general phenomenon that arises during linkage analysis and could be the result of recombination suppression at meiosis or translocations and inversions that are common in inter-specific or wide crosses. In this study, 38.9% of the markers showed distortion from the expected Mendelian ratio similar to the 42%, 41.3% and 38% distortion reported earlier for the same mapping population^24^,^28^,^29^. However, 99.4% of the distorted markers were finally integrated into the map so that the loss of genetic information related to these markers was minimized as has been done in several earlier studies^52^,^53^,^54^,^55^.

In the modern genomics era where complete genome sequences have become available, genetic mapping has become of secondary importance. However, trait discovery and crop improvement that depend on recombination necessitate the construction of genetic maps. Moreover, the generation of good whole genome assemblies *per se* depends on high density linkage maps that serve as a useful platform for assembling and improving the quality of sequenced genomes. The present map of 6698 markers was utilized in order to improve the previously reported genome assembly (v1.0) of desi variety^32^. This draft had been anchored based on the map positions of only 835 markers^29^ which anchored 532 scaffolds spanning 124.38 Mb (23.8%) of the desi genome. However, based on the present map, anchoring of an additional set of 2143 scaffolds that had earlier remained unanchored was carried out, thereby leading to a coverage of ~279 Mb (53.67%) of the assembled genome which was more than double of the 124.38 Mb previously assembled (v1.0) sequence. Moreover, the map was further utilized to improve the anchoring of the kabuli assembly^33^ wherein 96 Mb of sequence data could be added to the pseudomolecules leading to an 18% improvement of the current kabuli assembly^33^. Various studies have reported 67% to 97% of anchored assembly sequences in different crops such as grape^56^, cucumber^57^, apple^58^, soybean^16^, *Brassica*^17^, watermelon^18^ and banana^19^.

The Circos plot of desi vs kabuli revealed that markers from some of the desi LGs (CaLG5 and 7) found syntenic locations on non-corresponding pseudomolecules of kabuli genome. This could be a result of differences in methods of sequencing, assembly and anchoring of the desi and kabuli genomes since different sequencing technologies, assembly algorithms and linkage maps were used to assemble the two genomes. These anomalies are bound to disappear once advanced sequence assemblies based on denser genetic linkage maps become available. Moreover, a comparative study was recently reported by Ruperao *et al.*^59^ which was conducted to assess the quality of assembly of the two published genome sequences of chickpea i.e. desi^32^ and kabuli^33^. This study reported differences in the position of regions within and between pseudomolecules of the two genome assemblies. For example, they highlighted that some regions of desi pseudomolecules CaLG2, CaLG3 and CaLG8 matched with kabuli pseudomolecules Ca6, Ca8 and Ca7, respectively. This could be attributed to the fact that in the desi assembly v1.0^32^ only 124.38 Mb was placed into pseudomolecules in comparison to 347.247 Mb of kabuli genome^33^. This discrepancy has now been resolved by utilizing the present high density map based on the 6698 new marker positions for aligning a tremendously large number of unassembled scaffolds. Almost 87.69% of the marker sequences mapped in this study found matches with the pseudomolecules of kabuli (Table 4). Pseudomolecule-wise distribution of the markers between desi and kabuli showed only 30, 3 and 22 marker mismatches between CaLG2-Ca6, CaLG3-Ca8 and CaLG8-Ca7, respectively (Table 4). Moreover, Ruperao *et al.*^59^ also reported that numerous small regions of kabuli pseudomolecules were misplaced as 46 regions representing 16,164 kbp (ranging from 57 to 1371 kbp) were placed in wrong pseuodomolecules. This could also be a reason for the few dissimilarities in genome assemblies of desi and kabuli varieties.

In conclusion, this study reports the utilization of high-throughput technologies for identification and genotyping of SNPs. The large body of genotyping data generated were further utilized for linkage analysis in order to generate a very high density linkage map which represents the highest resolution map so far reported in chickpea with an average inter marker distance of 0.16 cM. The present map also revealed map positions of 3112 candidate transcripts which may serve as a promising starting point for QTL fine mapping, association mapping, and map-based cloning. Moreover, the utility of this map was demonstrated for generating an improved anchoring (more than double) of the desi genome sequence encompassing ~279 Mb (53.67%) of the assembled genome which was better aligned with the *Kabuli* genome thereby overcoming some of the earlier reported inconsistencies. The map also facilitated 18% improvement of the earlier assembled kabuli genome. Comparative analysis of chickpea with three important legumes revealed different levels of synteny that would facilitate information transfer among legumes for better understanding of the genetics of legumes.

## Methods

### Plant material and DNA isolation

A population of 129 F_10_ recombinant inbred lines (RILs) from a cross between *C. arietinum* var. ICC4958 (desi, *Fusarium* wilt resistant and drought tolerant) and *C. reticulatum* var. PI489777 (wild, *Fusarium* wilt susceptible) was utilized. This is an internationally accepted, reference mapping population, developed at the Washington State University, USDA, USA. Genomic DNA was isolated from fresh young leaf tissues of the two mapping parents and the RILs using the GenElute^TM^ plant genomic DNA Miniprep kit (Sigma). DNA quality was checked by electrophoresis on 0.8% agarose-gels. For high-throughput SNP genotyping, the DNA was quantified using Quant-iT™ Pico Green^®^ dsDNA Kit (Invitrogen) and the fluorescence was measured with Microtiter plate reader (Varioscan from Thermo Scientific).

### High-throughput SNP discovery

Whole genome sequencing of *C. reticulatum* cv. PI489777, the wild progenitor of the cultivated chickpea i.e. *C. arietinum* cv. ICC4958 was performed. A WGS library of 3 kb average insert size of *C. reticulatum* PI489777 was constructed using SOLiDOpti Mate-paired library kit following manufacturer’s protocol and sequenced using the SOLiD 4.0 platform (ABI). For genome-wide detection of SNPs in chickpea, the filtered reads of *C. reticulatum* PI489777 were mapped onto the previously published draft assembly of desi chickpea (ICC4958) (http://nipgr.res.in/CGAP/download/genome_sequencing/genome_sequence/)^32^ using LifeScope 2.5 (www.appliedbiosystems.com/lifescope). Stringent mapping criteria used for selection of SNPs were 3X minimum coverage of non-duplicate reads, minimum Phred QV of the bases 20 and minimum non-reference colour QV as 10. More than 65% or at least 2 reads, whichever was higher, containing non-reference alleles were considered. In addition, reads with variant allele not having additional sequence polymorphism within 20 bases up or downstream were only considered. In addition to the genomic SNPs, genic SNPs were also utilized that had been identified in an earlier study^35^ by comparison of transcriptome sequences of the same varieties mentioned above.

### SNP genotyping assay

High-throughput SNP genotyping was carried out using Illumina’s GoldenGate Genotyping Technology (GGGT) as described earlier^29^. The identified SNPs, one per scaffold, along with the 30 bp flanking sequence on either side were submitted to Illumina for processing by Illumina’s Array Design Tool (ADT) to obtain a designability rank score ranging from 0 to 1. SNPs having ADT score >0.4 were selected for designing Illumina’s custom Oligo Pool All (OPA) Assay. Two chickpea OPAs (CpOPA-II and CpOPA-III) were designed.

Genotyping of SNPs was performed using Illumina’s BeadArray Express according to the standard manufacturer’s protocol^60^ and as described in Gaur *et al.*^29^. The automatic allele calling for each locus was inferred with the GenomeStudio Software V2011.1 (Illumina, San Diego, CA). The quality of each SNP was checked manually and based on the GenTrain and GenCall score (>0.4) and call rate (>95%), high quality SNPs were extracted. For linkage analysis, the high quality genotyping data from CpOPA-II and CpOPA-III were transformed into ABH data format: A = parent 1 (*C. arietinum* ICC4958), B = parent 2 (*C. reticulatum* PI489777) and H = heterozygous (AB).

### Construction of genetic linkage map and anchoring scaffolds from genome assembly

Linkage analysis was performed using JoinMap 4.1 software^61^. The markers were clustered into linkage groups (LGs) using the Independence LOD parameter with LOD >6. The remaining ungrouped loci were assigned to the existing LGs based on their SCL (strongest cross link) group information at the same LOD. The program Recombination Counting and ORDering (RECORD)^48^ was used for determining the best order of loci on each LG. In JoinMap, regression mapping algorithm was used to order the loci in each LG using recombination frequency smaller than 0.49, LOD threshold >0.01, value of 5 for the jump and ripple value of 1. Recombination frequencies were converted into map distances using Kosambi mapping function^62^. Clear images of the LGs were drawn using the MapChart v2.0 software^63^. Genetic bins were counted as the group of markers showing zero recombination among them and the singletons were represented as the markers exhibiting recombination with all other markers in the same LG. Segregation distortion was analysed to evaluate its deviation from the expected 1:1 Mendelian segregation ratio.

Sequence information of the mapped markers was used to BLAST the draft genome assemblies of the desi^32^ as well as the kabuli^33^ chickpeas. Unique scaffold sequences were identified from the sequence assemblies that showed matches with the marker sequences based on the criteria of >95% identity and query coverage.

### Comparative analysis

Synteny analysis was carried out using sequence information of the markers located on the present map of chickpea. The sequences of the mapped markers were searched against genomes of legumes including chickpea (desi^32^ and kabuli^33^), *M. truncatula*, soybean and *P. vulgaris* (Phytozome v10.0) using Blastn with an e-value cut-off of 1e-05 (ftp://ftp.ncbi.nlm.nih.gov/blast/executables/blast+/2.2.25/). To generate the figures, cM distances on the LGs of present map of chickpea were scaled up by a factor of 500000 to match similar base pair lengths of the chromosomes of other genomes. Figures were visualized using Circos 0.67^64^ in order to identyify syntenic regions between chickpea (genetic position in cM) and genomes of *M. truncatula, G. max* and *P. vulgaris* (physical position in Mb).

## Additional Information

**How to cite this article**: Gaur, R. *et al.* High density linkage mapping of genomic and transcriptomic SNPs for synteny analysis and anchoring the genome sequence of chickpea. *Sci. Rep.* **5**, 13387; doi: 10.1038/srep13387 (2015).

## Supplementary Material

Supplementary Information

## Figures and Tables

**Table 1:** Markers utilized for construction of the high-density linkage map of chickpea (*C. arietinum* ICC4958 x *C. reticulatum* PI489777).

	**Markers analysed**	**Polymorphic markers used for mapping (%)**
CpOPAI^29^	768 SNPs	677 SNPs
CpOPA-II	3072 SNPs	2506 SNPs
CpOPA-III	3072 SNPs	2535 SNPs
Total SNPs	6912 SNPs	5718 (91.8%) SNPs
Other markers^29^,^36^	996 (SSRs, ITPs and ESTPs )	
Total markers utilized	6714	

**Table 2:** Distribution of markers on the eight linkage groups of the 6698 loci genetic map of chickpea.

**LGs**	**Genetic distance (cM)**	**No. of markers mapped**	**Average marker density (cM)**	**Unique positions**	**No. Bins (Markers)**	**No. Singletons**	**SNP markers**	**Largest Gap (cM)**
CaLG1	98.798	778	0.13	620	88 (246)	532	708	8.4
CaLG2	136.623	599	0.23	459	59 (199)	400	535	15.8
CaLG3	114.853	912	0.13	580	96 (428)	484	768	23.8
CaLG4	148.771	1050	0.14	812	126 (364)	686	903	18.8
CaLG5	158.788	942	0.17	721	120 (342)	601	819	8.93
CaLG6	138.482	1040	0.13	730	132 (442)	598	942	8.17
CaLG7	123.982	897	0.14	689	111 (319)	578	704	9.62
CaLG8	163.633	480	0.34	402	46 (124)	356	323	24.4
Total	1083.93	6698	0.16	5030	726 (2366)	4332	5702	**—**

**Table 3:** Summary of markers used for anchoring of scaffolds.

	**Total markers on LGs**		**No. of matched Scaffolds**		**Anchored lengths (nt)**	
	**v1.0**	**v1.1**	**v1.0**	**v1.1**	**v1.0**	**v1.1**
CaLG1	119	778	62	322	14791696	34198654
CaLG2	138	599	87	314	17304114	27556814
CaLG3	205	912	105	355	23376002	37128598
CaLG4	168	1050	84	404	22093647	44969074
CaLG5	141	942	65	379	16301343	38712624
CaLG6	108	1040	50	430	11482212	45233197
CaLG7	100	897	47	351	8461617	37542609
CaLG8	84	480	32	119	10574966	14354873
Total	1063	6698	532	2674	124,385,597	279,696,443

v1.0 represents the already available draft sequence^32^ and v1.1 represents the highly anchored improved sequence.

**Table 4:** Mapping of markers on LGs of desi and kabuli genome sequences as well as on the unassembled scaffolds of kabuli genome.

	**LGs**	***Kabuli*** **pseudomolecules**									**Unassembled kabuli scaffolds**
		**Ca1**	**Ca2**	**Ca3**	**Ca4**	**Ca5**	**Ca6**	**Ca7**	**Ca8**	**Total**	**No. of Scaffolds (size in Kb)**
Desi LGs	CaLG1	**689**	5	3	9	3	8	8	1	726	20 (4,324,571)
	CaLG2	7	**443**	5	4	3	30	4	1	497	44 (10,883,699)
	CaLG3	11	41	**631**	23	13	7	11	3	740	69 (19,034,154)
	CaLG4	5	8	18	**834**	13	21	5	8	912	49 (13,885,709)
	CaLG5	9	5	7	26	**704**	27	17	7	802	53 (17,316,010)
	CaLG6	5	4	4	11	7	**852**	6	10	899	53 (13,143,015)
	CaLG7	16	13	15	25	30	23	**624**	8	754	34 (10,268,573)
	CaLG8	17	11	13	25	20	19	22	**285**	412	22 (7,151,916)
	Total	759	530	696	957	793	987	697	323	**5742**	**344 (96,007,647)**
