# Supplementary material for: High density linkage mapping of genomic and transcriptomic SNPs for synteny analysis and anchoring the genome sequence of chickpea
Source: Sci Rep. 2015 Aug 25;5:13387. doi: 10.1038/srep13387 (PMC4548218; doi:10.1038/srep13387)
Supplement: Supplementary Information [file srep13387-s1.pdf]

High density linkage mapping of genomic and transcriptomic SNPs for synteny analysis and anchoring the genome sequence of chickpea

Rashmi Gaur, Ganga Jeena, Niraj Shah, Shefali Gupta, Seema Pradhan, Akhilesh K Tyagi, Mukesh Jain, Debasis Chattopadhyay, Sabhyata Bhatia*

National Institute of Plant Genome Research, Aruna Asaf Ali Marg, Post Box No. 10531, New Delhi 110067, India

*To whom correspondence should be addressed.

Tel. +91-11-26735159; Fax. +91-11-26741658

E-mail: sabhyatabhatia@nipgr.ac.in

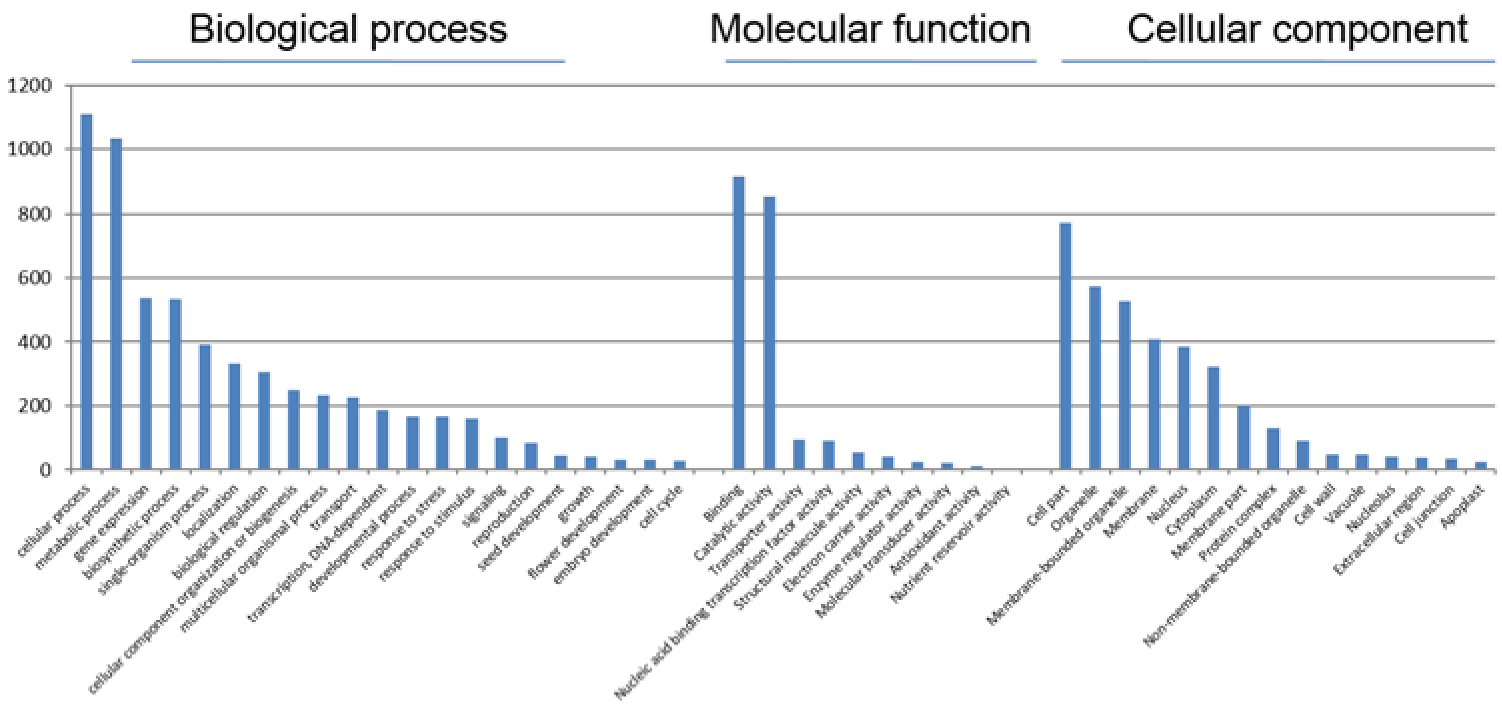

Figure S1. Functional annotation of the gene derived markers based on GO terms

Table S1: Characteristics of the 6144 SNPs which were used to design the chickpea Oligo Pool All (CpOPA-II and CpOPA-III) for use in the Illumina's GoldenGate Assay. Locus name, target SNP and sequence flanking the queried SNPs (in brackets) are mentioned.

S. No.	Locus	Sequence
1	CaSNP1919	CCTCACAACAGAATCTCTGACTAAAGGGCAATTGTTGTTCAAATAATCTCAAAGGATGCT[A/G]GTTACAAACAAAATAGTGATCCTACTCTGTAATCCGCTTATATAGATACAAACAACAAAC
2	CaSNP1920	TCTTTTTTTATCAACAATTCTCATGTCCATTTATGCATCATGCACAAACTAAACTACCTA[A/T]GACAGTTGGACAGTGTTACATTGCTCAATAACTTTTTATCCGATATATATTTTATCTGAT
3	CaSNP1921	TTAATTTATAATTAACCCAAAAGAAAAATGGAAAACAGAATATGACACAAGCGACACTGT[A/T]CACTCATATAGAAGAATTGAAGTGTCCAATGAATTGTACCTAATTATATGTATGTATGTA
4	CaSNP1922	CTCAGTTTCAAACGGTCCAACATCAATACAGTTCCTAGATACTTCCGGCCTACACTTTGT[A/G]TCTCGGGAATTCCACGCGAATTGACTAACCGGTTGAAATATTAACCTTTGCATCCAACTT
5	CaSNP1923	TGTTTATGCATAGGTTACATGAGGTGGATGAAACATATCTTTGCCTAATTTAGGAACCAA[A/T]CGTTAAATAATTTTCAGGCATTCTATCTTCCGTGGGCTATGCTTGCTTTGGATGTCATTT
6	CaSNP1924	GATAGCTAGCCAATAATCACGACGAGCGTGACCTTTAAGCTCTGGAAATTCTAGTACAGC[A/T]GGTTCTGATCTACATGAGAAATAACATTTGAAAGTTGATAGCAAGATAAACTTCTGCAGT
7	CaSNP1925	GATATTTTCTAGATCAAATACTTAATTAATGCCAAAACCTGAGTATATGCAAATTGATAC[A/G]TGTACTCTCGAATAGATTAATTGAATCAGTGTGGACTGTGAAGAGTTTATGCTTGCCAAT
8	CaSNP1926	GGATCCATCAACTGCATTTTCTGTCTTTGCGGTATTATTTATTTTCTGGTTTACTTCCTG[T/C]ATTTATTTGAGTTGAGCAATTTAGTTTTAGGTCTTGTTTGGATAAACAACTATTAAGAGC
9	CaSNP1927	ATTAATCGGAGCTAGCTTTTTGTGTTTTCTTCTGGTCGCCAAATATATTGTATGACATTA[T/G]ACTCCCTATTTCATCTTACTATTATTCCTTAGTAGTTAGGTATATAAAGTGTTTTTCTAA
10	CaSNP1928	TTTTTATATCAATGTAGAATTCAAATTTGAATTATTATAGAATTTGAGTTGGAAATAGGC[A/G]AATACACACTAATTATTCTTTTATTCTTTTGCATTCAAATCTTCTAGTATTGAAACATGT
11	CaSNP1929	TCTAAAATTACAACGGTTTACAACATGCGAGCTATATTTCACTAGGAGTTCAAAAACACA[T/G]TCACATCGAAACAAGTGTAACCAAAGGAGTCGATGAGTATCACAAAGCAGAGAACATACT
12	CaSNP1930	AATTATGTCTAATGCACGTGTCTCACATGTATAATAAGCATCCAGCGAGTGTCAAGTTAC[A/T]ACAAATTTAGGAAACTTCGTCCAAATCAAGTATTTAACATAAGTTGCAAATGGACATGAC
13	CaSNP1931	AATGCTGACAAGGAGCACCTCCACTTCTGCAGCGCGAAAAAGCTCATGCTACGAGGTAAT[A/G]GGATAATAGAATATAACAAGTAGAGGGAGGCATATACCTTACCTTGTTTGCGGCCAGAAA
14	CaSNP1932	GTTTTAGGTAACATATTTCCTAAAGACATTACATCATTTCTATGACAGCTGATTGGGAGC[A/G]TTGTGGATTGTTCAGTTCATCAGAAATGGTTACACTCTCCAACCTTGGCGAATTTCGACG
15	CaSNP1933	TGTTATTCTTGAAAGTGATTCTTAGGTCACTATTGATGTCGTCACCTATAAAACTAAGTT[A/G]GGTCGGTTGTTAGCCATACTAAGAACATTATTTGTAGTAACTCTAACTTTATGGTAAAGT
16	CaSNP1935	AAAAGGAGCTAGCGATGGTAAATTAGTAGCTAGTGTGACTGCAGGAAGTGATACAGTTGG[T/C]GCCTTGTTTGGAACTGATACTTGGAAAGTTTCAGGAGCTAGTGTAGCCGAAGGTGCTGGA
17	CaSNP1936	GTACCTAAACCAATCATAAAATGACAAAATCCTTATTATTGCTGGTTAAAGTTCAGAAAC[T/C]AACTTAAAGTTGGTCATCATATACTGATGGTATGGAAATAGAAAATTGAAAGTATAAGAA
18	CaSNP1937	AACTAACTCATGAATTGAGAGAATCTATCCCAAGACCCAATTACCTTCTTATGTTTAACT[T/G]CTTTTACCTACTAATTCTAGCTTGCACATAATTCATGTTTTGCTTTTTTGTATGATGGAT
19	CaSNP1938	TCGATCTAGTTGGATATAATAATATAACTAACAAAATATTCGTACATGGCTATGGATCAC[A/G]TATGTGGGTCGTGCGAAGTTGATTATTATACGAAGATAAACAATAGTAAACGAATAAATC
20	CaSNP1939	CACCTGCACAAGCAAATAATTGCTTAATATTAATCAACTCTACGATGTATCAATAGTTTT[T/G]CTCCGTTTTTCCTCTACCCTCCTTAATATGCAATGTTTCTTCCAAGATAAATACTAACAA
21	CaSNP1940	CATGAACCCGGATTATCTATTCAATATAATTATTTACATTATTTTTCTGGGATTCTCACC[T/G]GAATTACACGATACAACATCTCAGCACCAGCCCCTTTTGGTCAATTCTTCCATCTTCATT
22	CaSNP1941	CATTTGCATCTTTCAGCTGTACAACGAATCATCAAATACTTATTGGGTACTCCTAGTCGC[A/G]ATATCTTCTTCCCTAATAGTTCAACAATACAACTTCAAGCATACAATGATGCTGATTGGG
23	CaSNP1942	CAATTCCTAAAAATAAAAATCAAGAAAATGCATCTTAAATTGTATTCTATAATAACACCA[T/C]TAATTCAAAACTTTGATTGAAATTAGATTTTACCTATCACTACTTGATTAGCAAGAGTAG
24	CaSNP1943	TATGCATGTAACACAACTAAGAAGTTAGGAAGAACAATAAGATGGTGGCGAAGAGGGAAG[A/G]GAACAGGATTCATTAATAAAGTGAAAAATATAAACTGATCTCACTAGAAGCAATTATCAT
25	CaSNP1944	CTAGAGAACTCAATTGCTTTGGCCTGGTCTTCGTTGATATTTCAACCTTTGCATCACACC[A/G]TGTTCTTTGCAGAAACCTTTTAACCTTGAAGTTATTTATTTTAAGCAGATTCCGGCATAG
26	CaSNP1945	CATTTGATTTGTGTGTGTGTGTTTAAAAACATGGCAACTTGAAACACTAGAAACCATTAA[T/C]AAGATTAAATTGATGGAATGAGAAAAACCTTGCTGTTCTGGAACTTTCCTGCAGCACCAT

27	CaSNP1946	TGAAAGAGTTTCAGTCTCACAACACCGATTCACCAAAAATCAACAATTCAAAAACTATCT[T/C]CATTCGTTCATTGAGAAAGAAACTTCATCCAGAAACCTGTTTGTTTCGAAAGCATTTTAT
28	CaSNP1947	AAATAACCTTATAACCATCAGACTAACATCTATATCTGCATGTGTTTTCATGACCTTGCA[A/T]TGAAATTTTGCACAAGGTTGCACATCAAGATTGATAAACTAAATAGAGACAAACTACAGG
29	CaSNP1948	CAAGTGATTAAGTATCCAATCACATAGTTTTGTTCGATGCACTAATTTGTCTATATGCCA[T/C]CGTCGTATCATAATATTGCTTCTCAAAATCCTAGCCCAACAATTTTGTATATAGATATAA
30	CaSNP1949	GCCAATCCAAGTTTGCAGCCACAAATAGTACGATTCGTACTGAGTTAAGTTTGGCTACTG[A/G]AGCAAATGTCTCTTCATAGTCTATGCCAGATGATTGGGTGAATCCCTTTGCTACTAATCG
31	CaSNP1950	GACACGCTTTGCACCCACTGATATCAGTAAGGTTGGATTATCTTCATCCTCGTCAGCAGC[A/C]TCTAGTTCACTCCTCCGATCTGGTACATCGGTTGTAGCTGATGGAATGGTGTGTAGTTTT
32	CaSNP1951	TGCTCTAAAACATAACTGAAGCTAGCTGCTTAAAAGAAGCTAAAAAATTGAAGGGCAACT[A/G]TTAAATAGTATTGTCTCCGTAAGTTATCACATTATTAGGATTGAGTTCAGAGCAAAGGAA
33	CaSNP1952	TCAAATCATAAGACAAATGCACAGGCAGAGTCTCTACTCCTGGCACATTAGGAGATGCGA[A/C]TTTGTGATCTTTCAGCTTGCATCATTTCATAATCTGATTGTGAGATTGATGTTACACTTT
34	CaSNP1953	ACAATACCTTAATTTCTTCATTAACGGACTCATCGTTAGAATCAGAGTTAGGGTGAGTAT[T/C]TGTCATCACAACCATGTTTGCATGCTCTTCATCTTCATCAGATGATTCATCAGAGTCATC
35	CaSNP1954	ATTTTCTTGACAACACTGACTGCTGTGATATTAGAAATCGACTCTTACCAGCGTACCAAC[A/G]GAAAGAATGTGCGAGAGCATGTGCACATTAAAAAGCCCGGCCAAAATACTGGCAATACAG
36	CaSNP1955	AATCCAAATATCAACAAAAAGAACCAAAACATTTTCCATTAGAAAAGTTGGTTAATTGGA[T/C]CAAATGGCATCATGTCCTATTGTATATTACGCCTAGAGAAAATTCATTTAAATAATGCTT
37	CaSNP1956	AACAGAATATTTGAAACAATAGCTGCTTGAGATATAACATTACAGCAAACATAACAAAAT[A/G]AAGACACTAATAAATGAGTGCAGTACAACGGACTATGTATATCATTCAATAATCAAGATA
38	CaSNP1957	TCAATAATATTTTCTGATGTGATATATTAAGAATCATACTTGATGCCTTCCCCAAGTAAT[T/G]AATTGAATTAGATTATGATGCTATTTGTTACTAATGTTGTTTGCCTTGACAGTTAAGCCG
39	CaSNP1958	CAAAAAAACCAATTACTTAAAGTAATTTGGAGGGGTGACTCCTCTTACTTTATATTGAGC[A/C]TAATTCATTTTTACAAAACTAACTTGTGAAATGATGGGTGTCACTATTTATAAATTATTA
40	CaSNP1959	TGAACATGCTGCACATGCTAGTGTTAGAAGTTAATCATTATTACAACCAAACATGGTCTT[T/C]AAAGGAAGATTCTTCTCTTCCAAAAAATCTGATACTTCAAGTCCAGATGCTTCTTCAAAC
41	CaSNP1960	TTAGGTTTTGCATAGTAAATAATCACTGTTTGAATAATCCTTAGAAGTATTAATATCTTT[T/C]TTGTGATTTGGTGTACTAAGAGGATCATAAAATTAACATAATTGAGCTAAACATCGAAAT
42	CaSNP1961	AAACGTGTATATATGCAGATCTTGATATAGAGACAAATGGGAGATCACTTGCTTGCAGAA[T/C]TATCATACAACCATCTCCAGCATTTTCTGATGAATTAGTAGCTTTGTTTAGTAGCAAGAC
43	CaSNP1962	GAACATTATTACACTGGCCAGTATTGTAAGTGTTGTAAACATGACGTAGTATATAGGAGA[A/C]ACAATTGCTGTGTTGAAAGTGTCAAGAGCCTGAAATGTTATAATCATGTCAGCGAAGCAT
44	CaSNP1963	TACTATGCTGCCACGTGTTATTATATCAATTAATTAATCCATGTATGTATGTCCCCGGCG[T/G]ATTAAATTTCCATATAACCGTTATGATTTGGGCTCCACCACATAAAATGGGCTTCTATTC
45	CaSNP1964	TATATAGATTTCTTCTCTACAAGCATACAGGTAACTCTATAATACATCAAGGCATCGTCT[T/C]CATGAGTAATGGAAGTTTATTTTTCGCCCATCCTTAGTGAAGAATAATTTTCTCGTTTTA
46	CaSNP1965	CCATACATTTCAATTCTTTATTTTGATTTAATTGCCAAAGGGATAGCAATTCTCTGCAAC[A/T]CTTCTGCATAACCTTGATGTAGTCAATGGCAAAAAGCAGTGTGCATAAATATATAAGAAT
47	CaSNP1966	AATCTAATTCAAACATCAGAAATGGTGAAATATAGAAATATTAACCGGACAACATGATGA[T/C]ACAAGATACTGGAGAAAAGTAGGATGCCTATATTTCATACGGAACAAACTGAGCCAGATT
48	CaSNP1967	TCAGAATGTTGCTCCTGCACATGCTCCGGATATTGTCATATTTATAGATGCCATTACAAT[T/C]ATATTTGACGAAGCTACAAAAACTGGACCACCATCATCATTACCATTTGCATGTATTGAA
49	CaSNP1968	ACGTAATTTTGTGTTTGGTATCTAATTTTGATTGGAACCTTCGAAACTCTCATGGCACTG[A/G]TGGTTAGGTGAAAGTGCGAATAGTATTATTTGACGTGAAAACAAATATTTCGTCTTGTAC
50	CaSNP1969	TCGAATATAGTTTTTAAAAATACAACTTAAGTTAAGTCCCGTGCAAGGCAAAGATTCATA[C/G]ACTAGTATAAAATAATGTCTCTAATTGATATGTGTATTAACAAATATGTACTGATAAGCG
51	CaSNP1970	ATTGCTCTTAGCCCGAAATGATGATCCCCAAATTCCTTCGTGATTATCACTGAGCACAAT[A/G]AGGATAACATGGTTTGGCTAGGTGACAAGATGGATTGTTTATCGATCTCATCTACTTATA
52	CaSNP1971	TAACTATCATCTTGTCCTGCATAATTGTTATTATTTGTTCCATTGTTGGAAATGCACCCA[A/G]TTATTGTGTACACGTCTTTTGATATGCAATAGCAATTTGACTTTGATTTGTTTCCCTGTG
53	CaSNP1972	CCCATCCGGTTGACATGCCTTCATAAACAAAATGCCATGAAATCGAAAAAGATTCTTGGA[A/T]ATGGTCTGCAATATTGATATGATATGATCCAACATAGTTGCCAAAAAATTCATCATGTAA
54	CaSNP1973	TCAGCCCATGGCATACTTCTGAGTCCAGCTGCGTGCAGTGGCTTCGTACTTAGCCCGGTC[T/C]GTTTTGTACATATGAGCAATCTCGGGTACAAGCGGGTCATCAGGATTTGGATCAGTCAGC
55	CaSNP1974	AATCTCAAGTAGTATATACTTTCAGCATATAAGCCATAGTAGTACATTACAGCCTATAAC[A/C]ACTCACTTTTGCTTTGAATGGTTATTAATAACCAAATGCAAATGGTTGTGTTACATTTCT
56	CaSNP1975	AGGTAAATTTAGCAAAATGTTGCCAAAATCACCATAGCACACAATGATTCTGTCAAACTC[A/G]CCGCGTAGCAAATTGATTCCAAATAATATAATGTAACTTAACAATAATGTTGAATGCAAG

57	CaSNP1976	ATAAGAATAGTATACGAATTACTATATGTGAATGGCAAACTAGGTAGCTTCATCAGACAG[A/G]TTTATGTTAGAATTTAGTAAAAACAACTGAAGTATAGACACCCTGTATAAATGGCATATA
58	CaSNP1977	GAATTATAATCAATTATCTACATCCTATTATGCTTTTGTGGCTTCCTTGGATTCTGTGTC[T/C]ATTCCGAAAACTACATGTGAAGCTATGTCTGATCCTTGCTGGCGACAAGCAATGATAGAT
59	CaSNP1978	GAAGAGTTACTGCAGAGAGACGTCGAAAAAGATGATTTTCGAATGGAGACTCGGCGAGAC[A/G]GTCGACGATGATGACAAAGAAGCGGACCATGGAGGGACGTTGTCACTTTCTTTAGACCGA
60	CaSNP1979	GAGGAGTCTGTTTGAAGAAATGATTGACTCAGGGATAGCCCCGAATGAGAAGACTCTAAC[A/C]GCAGTAATTAAGATATATGGAAAGGCTAGATGGTCTAGAGATGCTCTAGAATTGTGGAAA
61	CaSNP1980	TGAAAAAGAATCTCCCATTTACGTATATAAGAATCTACCATATACGAAAAAGAATCCTTG[T/C]AGGTGTGAATCGTCGTCCTCTTCAATCGGATGCTACTACTCCCGTGTTTGTGTGTCTCTG
62	CaSNP1981	CTATTGCGGATGTTGTTAGGAAAATACTTGATTAAGAACATGTTTCTGAATACAACCCAA[A/G]TAATTGCAGTCCCTGCATTATCTAAACACTGTCATGCATTATCCCACCATTGTTCAGCTT
63	CaSNP1982	CCATTACTTGGCATCTACACGATCCTCATAACAATACACTTTATGAAGGCTTTTTACCTA[A/C]CTCAAAAATATTTGTTTTTCTTGTAGTAAATTGTACGTAAACATTCTGAATCTATCTTTT
64	CaSNP1983	AATTTAAATTTATTTATCTACTACATGACTACTGCGATTGATGTTATATTTTGAAGTTTA[T/G]CCTTTAGAATAAAATATTAATGTATTTTCATCATTTTAATATACTAATTTATTGTTTGTT
65	CaSNP1984	AAAACACAATCTATGCCGTAGTATCATAGAAACTTCAATTCTGGTCATATGTTGCACATC[A/G]TAGACACCAAAATTACAATAAGATGAAATACTTGTATCAAGATGAGTGTGATGCATATAG
66	CaSNP1985	CATAATAGAATTCTATATTAAAATATTCAAGGAAATAATATCACATTTTAACACCAAAAT[T/C]ACTCAAGTAAAATCAATATCTCATTGTAATATCAAATCATCTAAAAGATATTATCATCAC
67	CaSNP1986	CAACACCAAGCCTATTGCACCAACCATTGTCAACCTATTCGTCGACTCATCACCAAATGA[A/C]TCCTATGACTTTTCTCTCCCACTTTATCATTTTGGCAATTGTCCACTCCACCCAGCCTTC
68	CaSNP1987	CTGGGAAACTGCAGGACTTGATGCCTGATGAGAGATCCTCCCTGGAAAAAGATACCGAGT[T/C]GTTGCAAGAGGGTTTGGGTGCAATACACAGTTCTATTGATTCATTTGACCAAAGTACCTC
69	CaSNP1988	AACAAAGTCACCATCAAAAACACGTATCCATTGCCACACATTGATGACTTGATGGACCAA[T/C]TCAAAGGAGCAATCATATTTTCAACGATTGATCTGAAGTCGGGGTACCATCAGATTCGGT
70	CaSNP1989	CTCATATGTTAATTACTGCACAATCTACTATACATTGCTGAATTGTCTCTGAAATTCTAT[T/C]CTGTTTTGCATTTATGCAGGAAATTGTATAAAATGTCTCTTCTTGGATTTCTTGGAATTA
71	CaSNP1990	TCTTCTTAGAGGATTGAGATGGTTGGGAATTAGGATATTCACAATATGTTGCATTAACAC[A/G]TTGAAAGTAAGAAGGGTCTCGATATACATCATATCCATCCGACTTCTTACACGTACTCTT
72	CaSNP1991	CCATCATCTGCATCATACGCTTCATCTGCATTTGTTTGTGCATCTACATCTGATCTACAT[A/C]TGTTTGTTGCATCTAAGTGTGGTTTTGCATAACATTTGAATAGATGACAAGTACTTGCAA
73	CaSNP1992	CCCGCAGAAGGCGCCCGGCACTCTGAAAGAGAGAAACAAAATACATGAATCAGCTAAAAG[A/G]AATACAAGGTGAACACCACGTCCAATAACATTACACAGGTAAAGAAACTGAGTCATAAAA
74	CaSNP1993	GATGATGCATAAGTGTCCTTCCTATAGTATATAGTATTCTGCTCGAGGTACATGAAAAGT[T/C]TTTACAAGACCGCGACACATAATGATTCAGCGTGGAAGCTCAAATAACATTCATGCTTGT
75	CaSNP1994	ATTACTTATGGATGGTCAAGTGACAAGGTATGAACGAGTTTGGGCCAAAACCCCCAATCT[A/G]GCCTTTGAAAAGTATTACATATGCAACAATATTAAGTAGAATATTACTTGCGTGTTTTAT
76	CaSNP1995	GTTCACTTTAGGAGAGTTGATCCCTAATTTGGTAGTTTGAGTATCTGAGAGATAATCGTC[A/G]GGGGAATAAGAGGTCATACATTCAACTCTTCCACTAACAAAGGTAACTATTAATGTGATA
77	CaSNP1996	CCTTCTTGTTCCTCCCTACCAGTCACAGTCTACTCTGATGCATATTGGGGTAGTTAGTTT[T/C]GTATCTTGCTGATCCACATCAGGTTTTTGCAGTTTTTAGGCTCATTTCTCACTGATTGAA
78	CaSNP1997	TCTCTAAAGAGCCGCAATTCCAAGTTTAACAATACTATGTTGAAAATGGCCAAGTCCATT[A/G]AAGGTTAATTCACCAAACAAGAATGAAGTATGCTGGTTGAAAGAGAGGCGACCAAGTCAC
79	CaSNP1998	ATCAATCTGAAGAACTTACAATCTTAATCTAAACTAAAAGGAACATAATCAATTTAAAAA[A/T]TTTACAAATGCAATATGAACAAAATACAATTCAAAATTAAACAAGGATAAAATTGAATCT
80	CaSNP1999	AAAGTAGAAATGTGGGCCCCATCATTCATGTTCGATGCTTATCGGAAAACTTAATGTCGA[A/G]TGCGACCAATACAGTAAGGCTCATATTGAATTGAATGGATGTCTTCTAATTGATTTTTAA
81	CaSNP2000	TGAGTATCTCCTGGTTGAGAAGCTTGAGAATCAGACTCATTGGACTTGCTGAGGCACAAA[A/G]TTGAACTTCTTGTGTTGCGGAGTTTTGTGCAGTGGACATTATACTTTATGTTGGTATGTC
82	CaSNP2001	ACTTACAATACATGTCCAACAATGCATTCCCCATAATGGTCGTGAAATCCAATTCATTTG[T/C]GATATAACTGTGAATCTCCTTGCCAAGCTCCACATGCCTCAATGCCGCACAAGCGGTTAG
83	CaSNP2002	CCTAGACTTTTGAGGAACTAACCAACTTGACCCTACAAGCCAAGTCTTCTTCTAACAACC[A/T]GACAACTCTAGATTGAATAAGGAACTAGACCAATATTAGGTTGGATACAAAAGATTGGAA
84	CaSNP2003	AGTGTCTGTTTTGGCATCCTGCTGCACTTTTAGTTTCATATGTTCCTTTTGTTTGGTTGT[A/G]TTTTAGTTGTTTAGTTTTTTCAGCTCTTAAGGTTATCTTATCCTCACCTGTGGTTTGCTT
85	CaSNP2004	CATCAGACAAATATAAAGACTGAATGTCTTAGAATCACGCGGCCACCCTGTTTATGTCTC[T/C]GTATTGCTTTCATTCACCTTGTGTTACTGGCCATCTCTAGTTTGATTAGTTTTTGAATCA
86	CaSNP2005	GTATTTTCTGTAATTTGATTGTATTAATATATACCATATTACACAATTTTGATTATATAA[A/G]CAGTAGGCTGTTTGATGTCACGGTACTCTATTTATGTTATTATTTGACTGTTGCAGGTCT

87	CaSNP2006	ACACCTTTATCTTTCCCACCAGAATTCTGAACTTTGTGGAGTCAAGAGTATCTAGAGTCA[T/G]ATAGAGCCTTCGCTTTCTTAACAACCCTGTTGTTTCCTTTCCTAAAAACATTGTAATAGT
88	CaSNP2007	TATTCTACTTCTGTTGTAGACAAAGCAATAATCTACTCATTCCACAATAGTTGCTGAAGA[T/C]AGAGCAATAGTATACTCCTTCCACTATGTGCAGACAATTCCTCTATCTATGACATAATAT
89	CaSNP2008	ATAGGATTAATTTACTTCAGTAAATTATGACAGACATATGAAGAGCTCCAATTAATTCCA[T/C]CGCCAAAAAGCTACATAGGGATATTATTATTTTTTAATTAAACAAAAGGCAGTAATTGGC
90	CaSNP2009	GTTGTTATTAATATCATCCTATCATATAATGCAAAACGGCTAACAACTAAAGCAAAATCG[A/C]TTGACGGTTAAGTCACGTGGGTATCAAGTCTAAATGGGTATGAAGACCAATAAAGACTCT
91	CaSNP2010	ATTGAAGCTGTTGCAGAGTCAATGGGTCTTAAGGCCCATTCTCGCAGTTACAAGGTATGA[A/C]TTTGCAATACATACTTTACACATAATTCATTCAGAGAAACTAATTTTGTGATTAAATCCT
92	CaSNP2011	ATGACATACAAGTGTTAGTATCAAAATTCAGCGTGCCTGAGCCTAGGCCATGTGGCAAAG[A/G]TTTACTACAGAGATGTCAGTATAAAAAGGGTCAATTCTTAGAATTAGAAACCAAACAAAC
93	CaSNP2012	TATCAGAAAACAAATTAAGAATCATGACAAACTTTATGCCAATTTAATTTAGACCATAGG[A/C]GAAGAAAAACGTACCAAGAATAATGAAATAGACGATGAGGATACCAAGGTTAGTTGTGAT
94	CaSNP2013	AACCAATCCAATTCCAATTCTTTGAAGGTGTCGAATTCCGGTTGGTATACCTGTGATTCT[A/T]CGAGCGAGCGGGACAAAAAACCGGTCGTATAAAGGGATTAGGACAAACATGAACAATAGA
95	CaSNP2014	TGAGTATCTATTTATGGTCAACAAATACCTCTTCCAAATATAATTATAAAAGATATGTTC[T/C]GATAATATGTCCATTAGCCAATTCTTTATTTCTGAATCTGATATGAGCAACTCTTCCAAA
96	CaSNP2017	AATTGGGAACTTAGCTGAATTCAATGGTGGAGGTAAGTGTACTGCCATTGGATGTTAATT[T/G]TTGGATAGGTAAATGTTAGTTGTTAGTATGTTATGTTAGCTTCACTGATTTGTAATCTTT
97	CaSNP2018	CAGTCTCCTAGAGCTTGGTTTGGCAGGTTCAGCACGGTAGTACAAGAGTTTGGTATGATT[T/C]GTATTGAAGTCGATTACTTTGTATTTTATCGTCACTTAACCCAAAGGTGCATCTATCTTA
98	CaSNP2019	AGACTTTGAAAGCCACAAAACAGGAAATGAACTTTCAGAAATAACCGAATTTGGTGAGGA[T/C]TGGATGGTGTCTGTCTTCTCACATAATCCCACTAATATATATCTTAAGGCCTTCCGGCAA
99	CaSNP2020	CCTAGTCAAGCAACTTTGGCTAATGTCGCACCGTGGATAACCACTGTAGGCGCTGGAACT[A/C]TAGACCGTGATTTCCCTGCCTATATCACCCTTGGAAATGGGAAGAGATACAACGGAGTGT
100	CaSNP2021	TAATACATCGATCATAAACTAAATAGTGATGTGTATATTTATTGTCAACTCACAACCATA[A/G]ATTTGTGAATTTGTTATCATAACTAATTAGAATACAAGATCATAACTTCTAAATTCTACT
101	CaSNP2022	CACTTTTATATAGTGCTCTTAATGTAACTAACCTAACAAATTTCATATAACCATGAGTAT[T/C]TTTTAACTACCTCTAATAGTCCCCAGCAAGTTGAGACTGTGTAACAATATTTCCAACTTA
102	CaSNP2023	AAGGTAGGAGTAGTAATAGAGAAGATGCAATCATCTCGTATTAGGTGATTTGGACATGCA[T/C]GGAGAAGACTTGTAGAAACAATGTGTTAGAATTCAAAGTATGATTTATTATTCTTTGACT
103	CaSNP2024	TAAGATATCTGTTTTTGGACACCGCATATTTCTAATTTATGCAGTAAGTGTGAAGCCACA[A/G]TATCCCTCTAAGTTACTCTTCAGCAGTGAACATAAATTTCTATGGTGTAACAAATGATTA
104	CaSNP2025	AAGATGGTAAAAAAAGAGCAAGTGAAGAACTGTTAAAACAACTAGCTAGCCTGTAATGAG[A/C]TTTATCGAGCCGGGCTTAAAAGCCGTCTTTGAAAACGGGCTTAAGAATTAGTATCTGAGC
105	CaSNP2026	AACACTATAGAATAAACACATGACACATTTTAATCTTAGTCATTTATACACACCTAAATA[T/C]AAAGTGGCAACACTACACTCTACAAAAATTAATTTCACATAAAAAGATAAATAAAAAGGT
106	CaSNP2028	GCATACTAGATAATATAATTATATACATATGCTTAACATGTTCATGAATTTTCCTTGTTG[A/G]TTTTGCAGGCAATTGTGTCAGAGGAGGATGGGATGGAGTCAATAGCTCACAGATTTCTTT
107	CaSNP2029	CAAATAGAAAACTATAGACACATTTCTAGGAGTTTCTTAACTAATACCCACTTCTAGATA[T/C]TTCCCATATTTTCTAGATTCTTACATAGAAGTTCTACATGGTTCTAGACTTATCTAATTT
108	CaSNP2030	TAGCTCATTAAGATCATATGAAATTGAGCTTACAGAGGATGAGCCTAAGCATAAGTAGAA[T/G]ACTTTGGCTTTGAAATCCAAATAGAAGAAGGCTATGAAGATAACAACAAAATCAGAGGAT
109	CaSNP2031	TTACAAAAATATATCTAAAATTTAAAGTGAAAACATGAATCATTTGATCAAAGATCAAAA[C/G]ATCTAGATCTGCAATAGTTGTGATTGTGTGACCTTAGATAATCTATTTCTTGTTAATAAA
110	CaSNP2032	CCAAGATTCGCACGGTATTACATTACAGACTCGTGAAACTCTATGAAGCATTCCTATTAA[A/G]GAAGATGGGAAATTTCTATTAAGCAAGATGGAAATATATTTACGCTTTCATCTTGAATGA
111	CaSNP2033	TGTAAATTTGTTTTTAGAAGTATTACATAAGTGTAGATTTTGAAAATTAACCATTGTAGT[A/G]TACAATCGTATACTACTTTGGAGAAGGAAATTTGATTATAAAGTAGTATGTTTAGTGTAC
112	CaSNP2034	TGTATCCATGCTTTTTGGCACCGTTTTCAATTACTTTACAGAAAGATCAATGAATAAAAA[T/C]TTGAGTCAAGGTGCAACATCTAAGTAACTTGCCTTAAGAAGTGATCGCATAAATGCAGTC
113	CaSNP2035	TTCATGTTAAACATGAAACAGTCCATTCACAATTGCAAAACCTAATACATAAAGAATGTC[A/C]GGAGCTTTCAGCTGTGCAAATAAGTCCCAACAACCAAAAATGCAAAAATTGCATGCAATT
114	CaSNP2036	TCGCCCCAGTCCCTTTGTTTATATTTGACCGGACTATCATTTTAGATATTCGACTGAATC[A/G]TTGTCAGCTGTATAGCGAAAAAGGGATCCATTGGGAGACTCAAGTATATATATCTCAAAA
115	CaSNP2037	GCTTCAACCTGCATGTTTTATTTGTTCTTTGGTGCATGCTGTGTTAATAAGTTGGTGAAT[T/G]GTGATTTATTACATTATGAATAGGAAACTTTGCATGAGGGAAGATGGACGGCAGCAAGTT
116	CaSNP2038	CTGTGATATATAATTAAAAGGAGATGATTTAGAAAATCATTAAGTTGTATGACGAACAAA[A/C]GATAACTCTTTTAGAGATATAAAACACTACATACCTAATGGCAGATCGAAGATAAAACAA

117	CaSNP2039	ACGTATTACCTGCGGAATTAATTGCTTCGGAACATCCGCAGGTAAAACAAACATTTATAG[A/T]AGTGATATTAGTATATAACTTTTTGTAATTATGATTTTATTGGTCCAAATTAATTTTATG
118	CaSNP2040	AATTTGTCAAGTTTGACTAAAATATAGTAGTTATTTGCATCTTCATTGGTACTGCTGATA[C/G]CCTTTATACCTTACGCATAATTGTTGCTCAATTGTTGTCTGTTTCTTGTGATTGATCTTT
119	CaSNP2041	AAACTTTGAACCAGAAAGCCAAGCACCAACGCCTGCTGAAGTTGGTAGAGATATGGTTAG[A/T]GAGTTTGCCAGAGTTGTATATGCTGATGCCTCAGTTCTTCAAAGTTCCATTTACATTTGT
120	CaSNP2042	AAATGATATAGAATGCCTTGCACCATTGCCTTATTGTATCCTTCGGTGTAATTGACTTGG[A/G]TGGTAATGCTTGCAGTGACTGTTGCAACGTTGACAGATCTTGGTTTCTTATGGTTTAGAG
121	CaSNP2043	AGATCATAAGTATTGTTACTGTGTACCATTTTGGGATGAAAAAACTGACGCAAAATTCGA[T/C]GAGGACGGTTACTTCGGTTGTGTCTAGTGCGTCGATTGAACGAACAAGGTAGAGTAGAGG
122	CaSNP2044	CTTCGTCTACACTCATCCCTTCCTTTTAAATAGTGCATGTCTTTCTTTTTCTCTAATACA[T/C]CCATTCCTAATTTTATCTTAATTTCATATCTCTTCCAGTCCTCTATCACAACACTCCTAA
123	CaSNP2045	AAAGAAATGACTTGTACATTTTAGACGGTTCTACCATAATTGCACAAGCTTCAATTGCTA[A/G]CTCTAGTTTGCATTTATCTACTAGAATATGACATATGAGGTTAGGACATGTAAGTGAAAA
124	CaSNP2046	AATATTCAGGTGGTTTTTAGTGCCGCAATATAATCCAACAATTGATAACATATATTCAAC[A/C]TTTGTAAATGATATGTCATGTTCAGGTGGTTTTTAGTGCCGCAATATAATCTAAGGGCTT
125	CaSNP2047	GATATCGGAGGGGGGGGGGGAAACCCTCTATGTATGGAGTGGTTGGACTTCGTTATGTTG[T/C]TATTATTTTTAAGATGACTGTTGATACAATGTGGAGTTGTCATTACTTAAGACATAATTG
126	CaSNP2048	CAAAACCTCAAACAGATTGGCAGAAACGTTCTACAAACTTGCTTCAACTTTCCTATTTAT[A/T]TCATGTTACATCATGTTATTCAAAAAAATTAGTGCATCAAGTCATTAACCTGTTAAAATT
127	CaSNP2049	ATATGTGTAGAGGATCCAATTTTAGGTTCAACCATGTGCACCACAATGCTGATTTCACTT[A/G]CCGAACTACGTGATAACCGAACATTGCAATGAACAGGCAACGGTAGAGGAAGATTTTACT
128	CaSNP2050	ATAGCTATATTATAAGTAGTTGACACTAGTCAGCTATTAAATTGTGGAGCCATAAAGCAA[T/G]CACAAAGCGAACTATTCTTTCGCCTTTTACTAAAGAATACCGTGTGCTTACTTTCATAAG
129	CaSNP2051	GATATTTTAGGTTGGTCCTTTGAGATGTTGGCCTGGTGGCTTGTGCCTTTCACGATCTAT[T/C]GGAGATATGGATATTGGTGAATTTGTTGTCCCTGTACCATATGTAAAACAAGTGAAGGTT
130	CaSNP2052	TTTATTAGTCATTCTCAGTGCATAATCAATCTTAGCAATTTTCAATGCACTATTAATTTT[A/G]ATTTATTTTCATTGCATCATTAATGCCTCATTAATGCACCATTAATATTCAGTCATTCTC
131	CaSNP2054	TGTATCTGTAATGACAGAGAACAAAGAAACAGAATCATAGCAGAATACAGAAGAATAATG[C/G]AGACAATATCAATATAAAATGTCATGCACATAGAAAATATGAAGCACCTGAATTTTGGGA
132	CaSNP2055	AACCTGAATATATCGTTCCCATGGCTGCAATTGCAAAAACTGGTTGTCACATTTACACAA[A/T]ATCATGACTGTGCAGGAGAATAAACTTTCTGCAGTTTGCGAGAAAGTGTTTGACCGGTGC
133	CaSNP2056	TCCAATGGCCGAGATCTCTAACTGCTGTGGCGGCATGGAGTTTCGACGCCAGAGACAATC[T/C]GAATATGGGCGGCTAGTACTGTATATAACATGATTTCATATCATTTTGATACATTTATC
134	CaSNP2057	ATTTTGAATCTAATCCAGGACATGCAATTTGCTTGTTTTTTATTCACATCTACCACACTT[T/C]CAGGTATACTGTCTGATTTCACATGTTGGATGTGGTGTTTGTGACTTAAACCAACCTAAA
135	CaSNP2058	ATTGTGGAGAAGATAATAGAATCTCACAGTAAGTGATTTGGGCAAGTGTAGAAGCCGCCT[T/C]GTATGGAGAGTAGATTAAATGAAGAAGGGAAGTCTACTAGTTAGAGGTAGAGGGAGACAT
136	CaSNP2059	ATGCGTAATTTCTTATCTATGAGTAGACAACAACAAAACTGATTAGCTTTAGTAATGTGA[A/T]AGGCATAATCGACTTTGGTATTTGATGAAGTATTATCACTCCCTTTGGCAAGCCTTAGGG
137	CaSNP2060	TGCAATTATTAATCTCGCTACATGACAGATACAGATATCACATAATGAACCATCCGTACG[C/G]AAATTGTTCAATCCCACTACAAGATAAATTCAGACATCATGTAAAGGATCATCTATACGA
138	CaSNP2061	TTCTCTCTTTTCCAAAGTCTTTCCCTCCCTCCCTCTAAAGATGCTATTATGAATTAAATG[A/C]AATACGTTTGTTATAGCTAGTAAAAAACACAGGAAAGAATTGGATTTGTTTAAAGTTCCA
139	CaSNP2062	AAAATAAACCGATAAACTGCTACAAAACCAATAAAAACCGACAAAACCGATGGTTCAAGC[A/G]ATTAGACCGTGTTTTCTCATTTGTTTGCGATGCATCTTGATGGCGTAGAAGAAGCTATTG
140	CaSNP2063	AATATTCAAATGAGTAAACATCGTGCAGAGAATTGCTTACTTTGGACCGTGAACCAGAAT[A/G]GCGAGATCTTTGGTCATCTTTCCTGACTCCACTGTCTCAACACATGCAGCCTCTAACTTA
141	CaSNP2064	CACTTGACTTTTATTATATTTATAGACATAAATATGTTGTTGATTTGTGCTATATATTAG[T/C]TTGAATATTGTAAATTTGTTTACAAATTAATTCTTTTTGATTTTGGTGACTTTTAATTTG
142	CaSNP2065	TTGGAACTCCATGGTCAGGCCACTCAGGATACTGAATATGCAAAACAGATAATGGCGCAT[T/C]TTCTACCTGCAACAATATCAATCAAAAATTAATATGAACCAAAATGCGGACATCTTGATG
143	CaSNP2066	TGATAGAGGAAATTAAAAATCTAATGGAAAAAGGAGAGAAATTTGAAAATGTTGAACTTG[T/C]TCGTCGTCTTCCAGATGTTGAGCGTTAACGGAAGAAGATTCATGGATTTAGTTATGATTA
144	CaSNP2067	GGTGAGAAAGTATCACTGTAATCAAGACCAAAAACCTGAGTATAACCTTTAGCTACCAGA[T/C]GAGCTTTCAGACAATCAATATGTCAATATGACCAACTTTGACTGTGTATACCCAACGACA
145	CaSNP2068	GAGTGCTAATCCTTGAGCATGACTTCTCAGAAGATAATTTTGAAGGGACACTCTGGTTCA[A/G]AGCTATTTCCGAGCTCCTATTGTTCAAGTTTCTGGGAGTTCCATCACTAGAGCCCAGAAC
146	CaSNP2069	AGGGTTTACATCTCATTGATAACCATCTCCTCTTAAGGATACATTTCTCATTGGGGTGAC[A/C]TCCATCTAACAGGTAACTCACCCAAATAAAACGATGTAGCTAGGTGCACCTACCTTTGCT

147	CaSNP2070	CGACTAGCTTACACAAGTCAAACAATTTGTGTATCATACAAACAAAAATGTACTCACAGA[A/C]ACTATCCAATCTTACCAGTAACATGAAATACATTTAACCCATGAACGTATTTTTTTACAA
148	CaSNP2071	GATGCAGACTAAGAAGGATAGGAATTATTAGAACTATAATCATAATCCATATTGTTTCTT[T/C]ATTTTGATTTTGGAGTTGCTCTAATTATGGAAGCACTTTCCCTTTCCCTTCCCCTTCCCC
149	CaSNP2072	TAATCTCTTTTCAATACACTACTACTTATAGTTGAGTTCCACATGGGTCACTCATGCTAA[A/G]TAACATTTTGCGACCTACTCTCTATTTAGTGGGACCCATTGGAGAATCTTGCTTTGGAAG
150	CaSNP2073	CTACTTGCTCCTCATATGTTCTCCGAGATTAATCAATGAGAACTGCTCTAGAGTCAATAG[A/G]CACATACATCCAACATTCGTCAATAACTTGAAATAGCTTATTCCAAACAAAAGTGGTCGC
151	CaSNP2074	ATTTTCTGATTAAGCTAGTCTAATTTTATTATAAATAAATAGGTGGTTAGTATTTCACCT[T/C]CGTACAATCATTGCAACACGTCCACAAGAATCATCTTGTGATGTTTACATTACAGCTCTT
152	CaSNP2075	ATCCACTTCATCTTCTCTAGGAGCTTGAATATTGAGATCTACCGAACACCTAGAAAATCA[A/T]TAGCACTGGTACGAGCATTAGCAACCATAGTATTGGGAAAGTGTACCTTCTTGTGTCCAC
153	CaSNP2076	AACAATCAAAATAAGGTACATCGCACACCCTACCAACCAAGCACAATCTAATAAATTGTT[C/G]GGGAACAACTTGGGGTAGATGATGGTCCTCTGCATCCAGTCACAGGATCTTGGATTTTAA
154	CaSNP2077	GTGATGACCACTCAAGAAACTCACAATGAACAAATTAAGAGACAATGTCACAATGCAATG[T/G]AGATCATAGTACTCGTCAATTTCCTGTCTTGCCAATCTGCTGTTGTCAAATTTAGTTCTC
155	CaSNP2078	GCAATTTCTAAATTATTATATTTATATACATGAGACGCTGGGCCTAGACATAACAATACA[T/C]GTATCAAATGCCAAGATGAACTTTCTACAACAGAGGACTTTGCACTTTCAATATTCCTAC
156	CaSNP2079	TTAATAAAAACACCGACAGCATGATCCCAATACTGTAAGAAAAGTGCATTTATGCATTTA[A/C]AAATTTAATGGTATGACTTATGTGTCTCTTTGTATTATGTTTGACTAAGTCTAAATGAAG
157	CaSNP2080	ATACAGTTATGTTATTTCATTATGTCATATAACTATTCTAGTAGTTGTTCCTTACTATGA[A/T]TACTGCTCTCTGATTTTGTAATGTTTGGTGTGTTACAGCTATCAGAAATTGTAAAACAAG
158	CaSNP2081	GGACATTTTATGGCAATTTTCTTGCTATGATAAAGAATGGTTGATGTTCAACACAACAAC[A/G]AATCTTCAGTGATTTCCGTGAGATATTTATAATGTCATGCTCATTTGTTCTTTTTGTGAT
159	CaSNP2082	TTCTCTTCTTCACCATAACCACCATCGCTTCCTTTTCTCTTTCCGTTCCAAACCTTCACT[T/G]TCCGATAATTCTAACTCACACTCACAATCACAATCTATCTCTTTCTCTAAATCGCTATCT
160	CaSNP2083	TTACCGCCACAAATTCTAGTTTTCTTGTAATGTATGAAACTGGAACTTGTGATTCTTCTT[A/G]TTCGAGTTGGGAGTGATTGAACTTGTAGATGAGAATCACTTTTTATTTGGAAGCAAAACT
161	CaSNP2084	GTTAGGTAAACCCTCTACCTGTTTACTTGAGTCACAAACCACTTCAAAATTAAATATTGC[T/C]AACCATTGTTGGTTTGATAGCGGCTACATTTATATTGATAGTGTACTCAAATCTATAGGA
162	CaSNP2085	TACTAAGCCCCCTTAGGAAATTAATCTTGAACCTCCTCAGTTAGTTATCTCTATTTCGCC[T/C]AGTCTTGTTCCTCTCAAACCTGTTGAGTTTGCCCCTTAGCTACCTCTACGGTTGATCCCA
163	CaSNP2086	AAAATGCAATAAACGCCAGACATAAGATGTTGTGCATTGGAGCTCAAGTTCATGTACCTA[A/G]GGGTCATCTCTGCAATATTTTGTTAATTTCTTTCTGTCTAGGAGGGTTTGAGCATTCGTG
164	CaSNP2087	GGTTAGACTCCTCTCTATATATGTATATAAATAGCGATAATTGTGCGGTGAGTTTGGTGC[A/G]ACGGCGTCAAGATAGCAGTGAGGTGGGAATGGCAGATTGGAGCAGTTAGAAGTTGTGGGA
165	CaSNP2088	TTTATGCTCAGCTAGAAACTACATTTATTATATTTGAATTACTCTAAATCAAACATATCT[A/T]TTCCTAGAACAAAATCTCTCATTCAGGTCTGGTCATTTCTACCCAGTTAAACTACATACT
166	CaSNP2089	TCACAATATCCCTTATTTTCCTCTTTGATATGGATTTTTACTTTTAATTTAATCAGGAGA[A/G]TATTATTAATTAAAGAAACTGTTGAGCAGGCATTACGCACCTGATATAATACAATCCACA
167	CaSNP2090	GTTTTGTAGTGGTGATGATGGAGGTATGAGATGGTGGTCAATAGTGATGGCAGTGGTCAG[A/G]GGTGGATGATCCGAGATTGTGATGGCAGTTGTCAAAGTTGGGTGGTTAGTGGTGCTGGTA
168	CaSNP2091	TAACAAGAATATAAAGGAGAAACTAAAATCTTTGTTTTTGGGATCGAGAATCAAAAAACA[C/G]GATAGTGTTGGACAACTACTTGTTTGTGGCAGTGCCAGCAAATTTGAGGATGTTCAACTT
169	CaSNP2092	GATGAATCAATATTCGTAAGTCCTCTTCATTTAGAAATCCTAATTCTCAAATTATTTAAT[A/G]TATATAATACGTAGTTGGTTTGTCTTCGTTTTTGCAAATTGATAAACCCATATTAGTACA
170	CaSNP2093	TCTCTTCCTCTCAATCCTCTAATTCGCATATGTTGATCGGTCTCAAGACAACGTTGTATT[A/C]CATGTGATGCTCGTTTCTCTGTCTCAGCCCCTAGGCAATCTCCACGCGTTAGTTTCTCAT
171	CaSNP2094	GCCACTTAAAAATCAATGAAGAAAAAAAAATGATGTAGAAAGAGCGAGATAGATAACAAA[A/G]AGAAATGATAGAAATACATAAGAGGGAGATAGAAAATAGAGAGATAAATGTGAGGGAAAG
172	CaSNP2095	TTTTCCCACCCCTACGCATAACCTACAAGTTAATAAATGTTGCCCTATTATCTGCATTTG[T/C]CGAACAATGGCATAAGGAGACTAGCTTCTTTCATCTTCCTATTGGTGATATGACAATGAC
173	CaSNP2096	TAACAAATAAGCAAGCAGCCATGTAGCCATGTGATTCTATGGTTTTATTCCCATTCCCAC[A/C]AACATGTGACCATCGTCCCCTGTACGTATTCGTTAACCACATAAACCTTTTTCCAAGTCC
174	CaSNP2097	TTGGCTTGTTGCTCCTAAGTTCTATACACGTGCAATGTCTTCTAATGTAGTTAATGAGTT[A/G]GTTGACTTTACTTTTAGAGTTCTATCCTTATTAGCAATGAGAATTACTCCTATGTTAGCT
175	CaSNP2098	GTTTGATGTACTTTAGAAAGATTCTAAGGAATAAAGAAGTCAAAGCAAAATATGCGTCTC[A/C]TAACAATAGGAATCAACAAAACTATGTTTGTTTGAGAGTTCAGCAGAGGAGAGTAGAGGG
176	CaSNP2099	TATCTTGGTATGCTTAGTTCTCATAAAAGATTGAATTTGATGCAAAACAACTTGACTATG[T/C]CGAATGAGTGTCAACTCTGTACCATTTTCAAATCTAAGCTCTTGAAATAACACTTAAAAA

177	CaSNP2100	AAAGTGAAAAGCTGTCACTATGTATTAGACTTCAAACAATTTTATAGTGATCATAAGAAA[A/T]ACTTCCTGAGTGTATGACAAATTCAAATATTCAACCACACTAACTTCAATTAAGTTATCA
178	CaSNP2101	ATGCCAAAGAGAGAGCAAAACAAGAACCAACTAATAAGGCTGATGGAACTGCTTACACTG[C/G]CCAGGAAGAGTGTACAGCAAGAAACAAACAGGACAGTAGAGTTGGGCAAAATGAGGTTTT
179	CaSNP2102	TTGCAGGAAATAATGCCTCTAAGACGCATTTCCGGTGCTAGTGATGCCACTAATGGGATC[A/C]ATGAAACAGGGATGGTGCAAATGGCTCAAGCTATGGTACAGATGGCTGTTGCTATCGCCG
180	CaSNP2103	ATGGATATGATTGTTACGTCACGAAGGAAAGCTTGAAACAATGGGATTATTGAACTCAGA[T/G]TAACTAAATATGTGATTTAGATACTACATTTTTACCAAAAAATTTAAAATATTAAGTCAT
181	CaSNP2104	TTGAGGTGGTTCCGACCAAATAAACACCATGTCCACCTAAACCTAGATTCAGTCACCCTA[T/G]GTATAATTATGAAAACAATGATGCTATATGATATGAAGATATTTTACATGAGACTATATA
182	CaSNP2105	ATATTCCCACTCAATTTTCGTTAAGAAGAGAAGAGACTTGGACTCCAAAAGTCAAATGTC[T/C]AATTTGAGGGATAATCATCCCAATCTTTTAGATGGGCCCGTTAAGAAGATTGCAAGAGAT
183	CaSNP2106	CTTAAAATGGGGGACTTAGGGTGCACTATATGGAACATTTTCTAAGGGTATAATGTTTTC[A/G]GGTGGCACGTAGCCTAGAGGAGATGGGATTTGTGATTGGTTTAGGTTTTGGGGGTTGATA
184	CaSNP2107	TTTAGTTTGATGCATATTGGTTCACATATATATTGTTACTACCTATGAAGCACAAATACT[A/G]ACATGGACACCAGATACGACATTGACACGTCAACATTGGTAATCATATGAGAAAATGATT
185	CaSNP2108	TTAACTTGGAGAGGTACTATAAAAATTCTACTTAAGAGACAAGTGAAGCCAAACTTCTTA[T/C]GAAATCGAGATGCTATACTTTTGGTTGAATGTTGAAACATTATAATATAGAGGTGCTTGA
186	CaSNP2109	ACGCGGGGAATAACATTATTGGAGCATCACTTCAGCATAGGTGTATGCCGCGAGTACCCA[C/G]GGTTATGAGCAATGTCTCAATGGTCTCATATTCTGATGTATTTACTAGTTGTATACTTTG
187	CaSNP2110	GCAAACTCTGTATCATTCGTCTCGCACAATATTCCTTCTACAGACGTATTCGGAGCAGAT[A/T]TGGTCAATCGCAGGGACAAAAAAGGCATAGCTATAGAATGAGAAATCACTTGCCAGATCT
188	CaSNP2111	GATTACTACTAATAGCAAGATTAAAATAGATAACCACCCTAATAAACTGACAAATGTCAA[A/G]CTTGCCAGAAAATTAACTTTGTTTTATTCTAATGTAAGCCTCCTAATATGCACTAATTTC
189	CaSNP2112	TAACCTTTGTTTATTTTCATCAAACATGTTGTAGTTATATTAGGGCTTAGTTGAGGATGG[A/T]GCATACCTACAAGATCAGCTCGTGCCTTACAATTATCTTTGGATTTTCCCTCTTTTGTTT
190	CaSNP2113	GTGAAAGCTTCACTAAATAGAAAGGCCAATAGACCATTTCATCATAAGAGTATCCATATA[A/G]GCCACCAAAAGAATACCTCATATATTTACATTCATTCATAACTCACAACAATAATTCTAC
191	CaSNP2114	TGCGTTGGCAAATCGCACGGCCCTCCAACACAACTGTGACAGCAGGAACAACTGCGGATA[A/C]AAGATCTTCATCTTCTACTCCAAGGAACCCTTCAATTTTGTTATACTTTCCCGTATTTGT
192	CaSNP2115	TCGCCTCTTAGGTGAACCTGGAGTTGCCTTTTTTACACTATCCTTATTTACACAAATTTC[C/G]GGAGTACTACTCTGCAACTTATCAGTAGATGATTTCCCAGTATTTGTGCACTTGTTGATA
193	CaSNP2116	CCAGTGCATCTCTTGATCGCACCATAAAGGTTTAGACGTGTTCTTTTTGTCAGCTGTTCC[T/C]GGCATTTTCTTTATTTATTTCCTTACACCTATACAATAGTATTATGCATAAACACTGTTA
194	CaSNP2117	AGTAATGCACAAATTGCAGGAACAAGACCTAAGCCCAACCTTGTGATTGAAGGAGGAAAC[A/T]GAACTAGCTTGAGCTGTGGAGAGACATCTGCATTCATCATTTAAATTGTATTGTATGAGA
195	CaSNP2118	CTTCACAAAATTGAAATAGCGGTTGGCTCAAAAACTGCCACTGCAATCCATCATGGCACC[A/G]CCATCGCCACTTTTTAACATTACTTGTGTGCGTTGCACAGTTGCAGACTAACACAAGTAT
196	CaSNP2119	ATGTGTTGTGCATATGTTTCATATTAACGTTAGCACAAGTAAAGATAAAGTGATTGGTAC[T/C]GTGAACACACATGTTTTCTTTTACTCATGAGTCACGATTAATGTTCAAACATGGAAAATG
197	CaSNP2120	AACCACACCTCATTCTTAAGCTTAAACCAACAACAATTTGACCAGGGCTTTAAGGCTCTT[T/C]GCTCTAATTTTATAAGTGGAAAACTCTATAAGCTCTTAAGTCCCTTGTTTCGTCCACATT
198	CaSNP2121	TAATACACCACATAATTCAATCATGCAACCTAAGCCTAAATTCTTGAAAACTAATTTGTT[T/G]TCTGTTTGCAGCACTCTGCATCAGAAAGATAATGCTGACAATTGTCTTAGGCAAGACCAG
199	CaSNP2122	AATGACCCAATCCCTCTACTCCACCACTAATGAACATGAAATATATCTTCAGTGTCACAC[T/C]GAGTGCTGTTAGGGTTAGGTCACATGCCAAAAGCTAAAATTATAAATACACAAGCCACTT
200	CaSNP2124	AGTCATTTTGAACTATAGTGAAACTCACGAGTTTGTGAAGAACTTGACGTAACCCATTTT[C/G]GGTGAACCAATATAAATTTACGCATGTCCTCTTTATATTTTTAGTTTTTCTGTTTCATAC
201	CaSNP2125	AGATGATCGACCATCTGACAAGACAATTTTGCAAGGATATTCTCTCCCATCAACTGATAG[A/G]AATTCTTCGTAAAAACTTACATCCCTCCCATATATATATGCCAAAGAAGATGATGTTATC
202	CaSNP2126	CCGAGCCTCACCTGTGCTACAAGAAATGTCTTCATTGCACTTATCGGAAGTTTGTGCTAC[T/C]GAGTCCTGAGAATTAAATCAACCAAATGAAGGAAACTGAATAGCATATAAACAACTGAAG
203	CaSNP2127	TTGCAGAAGTTGTGAGCGAAAAGCGAATAGAAAGGGGGAAAATAGGAAATAGCGAGGGTT[C/G]GGGTATACCTTGGCGGTGATAATGTTACGAGCGGCGAAGATATTAGCGATGGATTTGGGC
204	CaSNP2129	CTTCTCCTATGTAGAGTTTCTTATCCATGTTCATAGGCAAATCCAGAATAAAATGGCATC[A/G]TAATGAGTATATATGCCAGCTTCATTTTTGCAAGTTTTTAGACCTAAATGATAGAGCAAA
205	CaSNP2130	AAAGTCATCCATGGAGAATGTGGGAGGATCATCACGAGAAATAAACTGATAACTGGTTGA[T/C]ATGGGAAGAGCATAATTGGAAGTAGCTTCAGCCTTTAATTTTTCCTTCAGTTCCTACAAA
206	CaSNP2131	ACACATTAACACCTATGGTTTTACACTTTAGGTCAGGTTTGTCACCAACGAAATTTTCAA[A/T]GACACTTGCACAAGTTTGACACAATCCACATTAAACGAGTTGTGATCTCTTCCATTTAAT

207	CaSNP2132	ATTTATGTTAAATAACCAAATGCATATTTACCTTACCATGTCACAAACAGAAGATTGCAG[T/C]GGCACATATACCATAGAATATTACACATCAACAAAAACATGACTAACAGAAAAAGGGTAG
208	CaSNP2133	AAATATAAAACATGTACAAGTGCAATAGAAAGAAAGAGATATTAAGAGGGTGAGAGAATA[A/T]TAAGCTCACTTTTTTGACAGTTAAACAAGTAATTATTTATGTTCAGCTATATACCTGTGA
209	CaSNP2134	TTAATGAGTTGTCACACATTGTTATATTCATTAACCATCAAGTGAATGTAATTAACCACA[T/G]ATAAATAACGTTAACCATCTAAATTGTTGTCAAATTTTCTTATCATTTCTCTTGGGAAGA
210	CaSNP2135	AGTTGTTAGACACAAAAGAAAGATCTGCTATACAATATACAGAAAAATAATAGGCTGGAC[A/C]ATAAGGAAAATGTAGCTTTAGTCACCAACATAATTACTTATAGAAGTTAATTTCCTACAC
211	CaSNP2136	AGACCATATAATTGAGTATGTAATCATTAACAACTTTAACTCTCTTACTTATTATAGTTT[T/C]TATTTCTCCTTGTATGTTGCTTTCAGTGTTCCTCGCCACAAGAACATGACTTGGTTCGCT
212	CaSNP2137	AGATTAAGCAATTATTTGAATTGACAGTGAATTTGGTAGAATCACGGATTCGCAATGTAT[A/T]ACACGGGGATTCTGGCAAAAGCTAAACTTTGGAGCTTCAGCAAAATTGCTCTATCCACCT
213	CaSNP2138	AATTGTGTCGAAAACATAGTGGTAGAGTCAGATCGAAATAGAGGGAGCAAATCGGTATCA[A/G]TAGCAAAACGGGTCATACAAAGCTCTTTACAGTAGATCACTTTACAGTAGATCCTAAAAA
214	CaSNP2139	CTACACCATATAAATGTCAATTTTATCGAAATTATTCACACACCAAATGCATGAACCGTC[T/G]GTCGATTTAGTTATTTAGATGGTGCAATAGTAGTAGAACCATCAAATCTAGTGAGAGAAA
215	CaSNP2140	TTAATCACCATTAGTTATCTAAGTATATATATATCCCTTTTTTCCAACTAAGTCCATACA[A/G]TATATAAAATAATAAGTATTTCTTAAACTATATGAAATATGTCAAATTAGTCTCTACACC
216	CaSNP2141	CGGGAGAGAAATGGGACATGGAGTTGGGATAGGATTGAAGTATTTAAAGAGCTGTAGTTC[C/G]TTGCTTGAAAAGTTGATGAAACACAAGCATTGTTGGGTGTTTAATACTCCAGTTGATGTT
217	CaSNP2142	TCTGGGACAATTTTCTATGTTGTAGTACTATTTATTAATGGCATGTTTGATGCATGTATT[A/G]AGAAAGTTGCAAAATTGATGATTTCTTCTGTAAATGGTGAATGGGTTGAAGGCTTCTTAG
218	CaSNP2143	AATGGATAGGGAGAAGGAGTCTATTGCAACAACGTTCAATAGAAACGATGGTAAATACTC[A/G]GAAATTTACTCAATTATAGATAATAGATGAGAGTGTCAACTTCATTGTTCATTGCATACA
219	CaSNP2144	AAAGCACTCTCTATATTGTTCTGTAAGTTGATTGTACTTTATATTTTGATTATATTTGGG[A/T]ATCAGTAGAGTTAGCATTTATTTTTATCATTCCTAGTTTTGAGTACATAACTGTTGATTC
220	CaSNP2145	TCATATCTGAGCTAAGAGCTTGCGAACGAACTTGGTTATCTGACAACTCACACTGAAATA[A/G]TTCCTTCGCTAGCATAGGAGACAGAGAGATACCATGTCCATGATTGTATTCATACTCCGA
221	CaSNP2146	TCTTGATGCACTGCTTGGTCCTACTCAAACTCAAGTTGTACATTATCCTCATGTTGTTCA[A/G]CCACATAATGTTCCTCTTGATGCTCAATCTCAAGTTGTGTCTCTTGTTGCATCTCTTCGT
222	CaSNP2147	GCCTCAACTACTTACCCGATTAGTTGCAGAATTGGGCCACTTATTCCTTCAGGTATAAAC[A/C]CTGTTAATTGGGGGGGTCGTCTAACAAGGTCCCACTAATTGGGATACATGATTCAGTTGA
223	CaSNP2148	ACTTGAATAAAATTGCTGATAACTATTTTGCTTAAGGAAATAAGAAACAACACTTGCAGT[A/G]CAAATACCTAAACAACCATGGTTTATGATGTCTTTGCAACCGCGTGTCGGACGCATCATT
224	CaSNP2149	GCAGACCACCACGATCAAGCTCATTCAGTTGTACTTTTCCAGAATCTTTATGCTTGTAAG[A/G]CCCTACCTATATGATGACAAAATAATATCCACTTCACAAATTCTCAATATTTATTTTCTC
225	CaSNP2150	CCACGGTCTTTAACTTCAAATTCCTTAGCAAGTGCTTTAATTCTCTAATTCCATTCTGAT[A/C]ATCGTTAGTGATGATGATGTCATCTACATAAACAATGAAGATAGTCATCCTTCCTTCTTT
226	CaSNP2151	GTGAAAATTCATCCCATTAGTCCCTAACATCTTAACACCAGGAATAACACGACGAAGTTT[T/C]GTATATTTTAAGAACATGTATACTTATATGGACAAAGTAAAAGAGAAGTAATACCAGAGT
227	CaSNP2152	GGCCTATGCCATGATGAGAAATAATCACTCTTATAATAAGCTGGTTGCCCGTAAACTTTC[T/C]GATAGACAAAGAAGTGAAGTTTCAGTTAAAATTGACAATGAGATAGAGCACCCATCACTG
228	CaSNP2153	TGCAAAATGCCCCCATTTTTTACAATTGTAACATTGGATCCCTTTGTTGTTGAATTTCTT[T/C]TTGCCATTCGAATTCTTGTTGTTATCACTTCTGTTTTGGTTTCTTGAATTTCCATCCCCT
229	CaSNP2154	TCCCCATTAGTAAAGGGCGGCTTTTCTAATTATGGATGTACTTTGTTGCACATCCACCTA[T/C]GGATTTTGGTCCTGCATGTGGATTTGTTTGATTTCAATTAATTTTGGGACATTATAAAAC
230	CaSNP2155	ATTTTTCATTTAAATTTTGATACTCAGTCATCCACAATGAGTGCACTGCTGAGCTGAAAA[T/C]CCTAATAGGCATCACCAGAAGCTTTAATCTCCCGTCTGTATTCTTAGAATAATCTAACCT
231	CaSNP2157	GTACCCTATAATATTATAAATCATGTCCATTTCATTTCTTCTCTTTCAATTCCTATCGAC[T/C]AGACCAGTTTTCTCGATTTCCACTGATTAAGGTAAGATGATTGGTGTGTGGTAAGAGTTG
232	CaSNP2158	TGGATTCCAGGGCTCTCTATACGCTTGCATGCAAGATTCGTCTTCGTTTTTGCACAATGC[A/G]GGGCAAATCGATCCACAAAACCAGAACCCAACATTTGTCAAGGTATGATTTTTAAGTAAT
233	CaSNP2159	GGAATCACTCGCTTTGCATGAGATGGTGGCAGCAAGTGGTGGGGATCCAGGGGCACGCAT[T/C]GAGAAGATGTCAATGTTATTGAAGAAGATAAAGGATCATGTGCAAATAGAAAACCTAGTC
234	CaSNP2160	TATTGTGATTCATGAAGGAGATTTTTGATATGAATCAAAATATAATTCTAGAAACGGTAC[A/G]TTATTTTTATTATATTTTGATTTTCATATATAAACTATATATATATATATATATATATAT
235	CaSNP2161	ACTTCTTCATGCTTATCGGAGTTTCATATGGTATTTTCAATTGATGTTGAACTATAATTA[C/G]TTTTCATTGTTAGATATGGTAATAGCCTATTACAGTGCTCTTTGAAAGAGCGCTGTAAAA
236	CaSNP2162	TACTTATGATAGGAATTTTATGGATTTGTCGTCGATTTGAATTGTCTAGTCAAGATCAGA[T/C]GGCCCACAATTTGATTTAATATCTTGGTCCTGATTTACAATTTCGAAACAATGATTGATT

237	CaSNP2163	TAATACCAAATCATTTTAAGCAAATCATTTTAGGACAAGTTAGAGAGAGAGAGATTATAT[T/G]CTCCTAACTAGTTAAGCTTAGAGCCACAATGCATGAACAACATATGCTAACCTAATTATT
238	CaSNP2164	ATTATATAAAACAATTCATTTGCTACCATCTGATTTCTTCTGCAGTCCTGGAACATCTGT[A/C]GCAGCAAGTTCAGTATTGTTGGGTCTGATACTTCTTGGAAGAGCGGCGTTTGTTTTCCCC
239	CaSNP2165	TGTTTGTGTGTGTCAGAGACTCATTAATGAATTATTGACTTTGATGTGGGTGTAGAGGAA[T/C]AACTCATCCAAGTGGAAACAAAGCATAATGGAAGCAATGGCGCATGACTTATCATGGGAT
240	CaSNP2166	AGAATGGATCTGAATTTTACAGATTAATTGCACAACATGTGTTTTAATTAGTGCGAGGTA[C/G]TTTACACACTTTGTAGATTACAAAATTTATACAGACTTTGTATTTTCTATAACTACTAAA
241	CaSNP2167	ATGACATGCATCATATAGATTGGTAGTGTAGATCACATATAGATTGGTACGTGAATTAAT[A/C]TAACCAAATGACATACATCATGTAGATTAGTACGTGTAGATCACATATACCTCTCTATGA
242	CaSNP2168	CCGAAGTATAGTGGTATGGTGGAGGATTGTTGTTGTGATTACGAAATTGTGGATCGGCTT[A/C]ACAAGGAAGTGTTGTATCCATCTCTACAAGAACTTGTGAAAACCTCATTCTTTCGGTATT
243	CaSNP2169	GAATGAGATTGAAGGTGCTTGGTGGTGTAACTGCACCGGTTGGTTAATTCCGTTACGATA[A/G]CCCTAACATTAGAGAGAGAGTGGATTGTGATGATGAAATGGAATGAATGGATGAAAGAAA
244	CaSNP2170	TTGAGAATACAAACCAAAGTAACTAAGAGTCAATAAACCAAGAGGAAAGGTTATGCCAGG[A/G]ACTATCAATGAGGTAGAATCCCACTTACAATGAACCAATTCAAATAGCATGAGAATTTCC
245	CaSNP2171	CATATTCTCCACTTTCTATGATGCAGTCACCCTCCAAGCCCAACAAGAATATCCTCCTGA[T/C]ATGCAATGCAAAGATAAGTTTCTCATACAAAGTACAGCAGTGGATTCAAACACTGATGTT
246	CaSNP2172	TATCATAGCAATGAATTACTCTTCTGTCATGTTAGCCTGATTAGAAGGGTCAGGCTTGCT[A/T]CTACACTTGTGTGCCATATGACCCTCTTTCCCACAATAAAAACAGAGAAAAGGTGGGCGA
247	CaSNP2173	CAGTCTAAATACAAATCAAAGCCCAAATAAACACAAACTATATTGAGACTGCATAGAAAA[T/C]TATATCCCACACCCCCAATTGTACCTCTCACCTCTATTTTTCATTAAAGACTATTTTGTC
248	CaSNP2174	GTTGGTGCCAGCAAAAGTCTCCGGAGATGCTAATGTCTGGTACCCCACTGTTAATCCAAA[T/G]GAAGAAACAGAAAAGATACATGTAATTAGAATGATCATGTTGACAGCATATTGAAGTAAA
249	CaSNP2176	ATTATAGCTGTAACTAGTGTGATGTGAAACATAATACGTTGATGGTGTTTTTGGTCTATA[T/G]GGAGTGGTAGTTGTGAGAAGCTGCTGGCGGTAACGATGAGAATGATCTTTTTCAGCAAAG
250	CaSNP2177	CACTTACCAATTACGGCACAATCACAAACAACAACTGAGTATGTATATACAATCATCACA[A/G]TATATAAAACATGACTCGCGTGCCAAACACCCTATTGCAATGCGTATATGCCAAAATGCA
251	CaSNP2178	AGAGAGGGAAATTGAGAGAAACCTTCCACCTTATTTGAGAGAGTGATTTTGTAGAGAATG[A/G]TTAGATAATTCTTTTGATTAATATTTTATTTTTAGAGTATTATAACATGGAAAGGATTTG
252	CaSNP2179	TACACAATCAGCATTAACTGTAGCACTGCGAACATTTTGCATCATCATTGAGTCTAACTT[A/G]TGCCTTTGCTCTCGTAGAACACCAGGAGTATCATAAAGTATCATCTGCAAGAGATTCACA
253	CaSNP2180	AATTTCAATTCAATATAACCTACAATCATTTCAATTTGCAAAATGCAAAGCAAGAAACAC[A/T]AGAGTAGTTAGTTACAAAGCATACCTACGATACTGAAGAGGCATGTTACCAGCTTTCCAT
254	CaSNP2181	ACGAAGATAAGAGGAATTCAACAAGAGAGAGGTGGCTAGATTATGACATAACCTTGTTTG[A/T]AGCATTTGAAGTTGAAATTAGTTTTATTTTATATTAGAAATTGACAATAGTAACTACAAT
255	CaSNP2182	TACGGGACCATATATTATATGACATACTTATATATCAAATAAAGTTATGGTACATATGAG[C/G]ACATGCACACACATTTTCTCGATTGACTAGAAGGAAACGCCCCATTTCTACTTTCCATCA
256	CaSNP2183	AAGTTTTCAAGACCAATCCCAATCTAAAGAAAAATAAGGAATAAATTGACAACCATGTGT[T/G]TGTGTGTGTGATATCAATAAATTCCCAAATTGAGAAACATATAGAGCTGTCATAAATAAA
257	CaSNP2184	TTACAGTATGCGGCGACATATTGGTCAAAGTGTGAAATGCAACACTAATTGCAGAGTACA[A/G]TTGTACAAATAGCCTTATAATACCATTGCATTCAAGAGATTAGACAGCAATTTCAATTTT
258	CaSNP2185	AGCATGTGGTGAAGTTGAATCCAACACACTAATAATGATGTTATTAAATTTTGCAGGCTG[A/C]GAGAGCTACACACACTCAAGGGACATGTTGAATCAGTTGTCAAGTTGAAGGGTCTTGACA
259	CaSNP2186	CATTACTGATAGTTTGAAGAAAACAAATGAACTTTTGAATCAAGAAGTGCTGAAGTTGCG[T/C]TCTCAGGTATATTATGCTTCCCTCGTTTCCCTCTTAAAACCATGTAAATCATATATAGCT
260	CaSNP2187	AAATGGAAAAGATTCACCAAATCAACAATTTGCAGTACATCTCTGCATCAAAAACCTATG[T/C]GATTAGTAAACAAGGTCATATTATGAGACCAACCTTGGCAAATTTATTAAAGTCACGGAG
261	CaSNP2188	AAATTCCTCTATGGTACAGTGCTATAGCGCAAGCTGCACAAGTAAAGCCGCTATTTGAAA[A/C]CATTTTGTACTAAATATCGTTTCACAGAACAATAGTGTTTGTTCAGATAACGTTACGCTG
262	CaSNP2189	AATGTATATGTGTTTAAGTGATTTATTGTGTCGTGTTAGCTTATCATGTTAAGTTAAATG[A/C]TTTTTGACATGTTGTGCTTAATTGGAGAGTGAATGTCTTCTATATATTTATAGACTAAGT
263	CaSNP2190	AGAAGTTGCGACATACTTATCTGTTTTTGGATATGCAGGACTTTGAGGCACGTGTTGTAA[T/C]CCAATTGTTGTGTCTGTAGGATATATACACAAAATCGATGGGTCTATTTTTATTCAATTC
264	CaSNP2191	ACCGTGCTTGTGATAGGGGATACACTTTATGTTGCTAATGTTGGCGATTCAAGGGCCGTG[C/G]TAGCTGTTAAGGATGGTAATAGAATTGTTGCTGAGGATTTGTCCTCTGATCAGACACCAT
265	CaSNP2192	GAGACATTTAGTTTTATGAACTCTTTTGACAATAGATTCTCCAACTGATTTTTCAACTCT[A/G]AAAATTTGGATGGAGACATTGTATATGGGGTAATAAAAATAGACCTTGTACCAAGAGTCA
266	CaSNP2193	ATATGGAAGAGGATGTCCCTGAGCCTCCATATATGGTGCAGGCTGAGGAGTTGTATGTTG[A/C]ACATGTTGATGATGCTGATGAGCAGAATCAAGATCCCAGATTCCTCGGAGGATCGGTGGT

267	CaSNP2194	CGTCGAGTGGAGTTGGGGTTTCTAGAGTTGACATTGAATACAGTGTTATCAGGTGGAACT[T/G]GTCAGATAGTTGGTTCTTGTTGAGCCACTAGAGAATAAACCTGAGTAATATAGGGTAATG
268	CaSNP2195	AAGTTGTATTCATATAAGGAATGGAGTTACAATTTTTACTTCTGAATAATCAATTTGGTC[T/C]CTTGATAAAGAATAACCTGATATACTCAAGGATGCGGTCAACACACTTAGGACTACATTC
269	CaSNP2196	CAAGTCCATTATAAGCAAGTGGTGCGTACATCCACATATCATCAGAGCTCAAAAGCTACA[T/C]AAAAAGAACAATGAAAAAATGGTTATGATAAGTAATAGTTTTTAACAAATAAATATATTA
270	CaSNP2197	GTGGTGGGTAGTCACCCTAAAAGCCAGTTTTGTGGGGTTGTGTTAGTCTCAAACCAAATT[T/C]TAAGATGGTATCTGTTAGAATAATAGAAAATATCTTATAATATCTATTATATTATATTAG
271	CaSNP2198	AGCAAATACGTAATTAAGCCTTTTTTTATGGAGCTTTTGAAAAATTATCATCTTTACTAT[A/G]TAACAGGTTCCGATGTTGATACTAGGATATTATAAACTTTACTTGAGCTTTAAAAGAGCT
272	CaSNP2199	TAGGTTTTGCTAACCTCGATCTACATGTGCTTTAGACTTGAATTGATTTGTATTCTCCTC[A/G]GCCAATGAAAGCACCTTCTTACCCTCTCCATTTTTTCTGGTCTGGAAATTTTCCAAATTG
273	CaSNP2200	ACTTTACCAGACTTCATGAAGCTTAAACCTCATACTTTTTCTAGGTCTAGCGCAACTGGA[T/G]ACCCACAACAATTCATTGATAGTCTAGAGTGGCTTTGGAGAGCATTGAGTTGTTCTGATG
274	CaSNP2201	GTGGTTAAATTCAACTACTTCTATTTTCTCAAATTATTATCGGTATCTGAGTAACTTAGA[T/C]CAGTCATTTTACTTATTTAACTATCAACAATATCACATCTGATACTCATAACAAAAATGT
275	CaSNP2202	CATTTCATTATTATGAATGTTTTTCATTTTTTTAATCAAAGATTTTTTTAACCCTCCGG[A/G]TCATAAGGAAAAAGGTCATAGTAATATGGAGTTCAGCCAGGATTGGAACTCTTGAGTTTT
276	CaSNP2203	CACCATAATTTAACATGAACTATTTTATCTGAATGTAACAAGAACCACCCAACCTTCTCT[C/G]ACTAAAATTCTATTATCTCCACTTGATTCTATTGCTCTTAATTTTTCTCGCTTTCTTCAA
277	CaSNP2204	CCTTGTGAAAGACTCAAGTTATTACCTCTATTTACATTTTATCAATTGTATTTGACCCGT[A/G]TGCGACAGCGGATCACTAGTAGATCTTTTATTTTATATATTTATTTATTTATTTTGTATA
278	CaSNP2205	CCTCTGGTTCTAGTTTTGAATTAGGAACTTAGGATTAAATTATTATGGATTTAGTATATT[T/G]CTAAGTTATTTAAAATTGTTGTGGATGAAGGATGTATTGCATACATGACAAAATAAAGAC
279	CaSNP2207	TACCAGTTTCTCCGTTGTACGACGTGTTACTCAGAAGTGGTTCAAGAGGGAAATTCGCAG[A/G]ATTGTGAGATGCTTCAGCACCAACCGGTAAATAATATGATTCAAAGGGATTTGGACCTCT
280	CaSNP2208	ATTTGCCATAAGAATGAAGTCAAATACAACACAATAAATTAGTGATTTGAACCAATCTGA[T/C]TTCTAATTGTTACAAAATTGTAAATAAATTATATGTGTAGCCATGCTATGCTGGCTTTGC
281	CaSNP2209	TGTTGTTGTTATAATTATAATTATTATTTAATTTGATTAGATTAACCGAGTATTTGAGAC[C/G]CCAAATATATTTCTAGCTTGCATTTTAGGTGAACTCTGATGGCAATGCTTGTATAATTTG
282	CaSNP2210	AGGTGTCAGTGTCTGAGTCATGTCCGACATGACTCAGACACTAGGACACACATTTGACTA[A/G]AGGTGTCAATGTTAGAGAGTCCAGATCCCATAGAATTGAGTAACACTGAAGAACATAGTA
283	CaSNP2211	CATTGCTTCTTTCCATAAGGGTCTTGTGTGAGCTTCCTTGGCATTTATAGTTTCCATTGT[A/G]TCTTTGTAGGTCTCAATGAGTCCAATGTAAGGTAGCTTTGGTTTGTAAATGCTTGACTTG
284	CaSNP2212	TATTCTGGGCTTAAGAAAAGTCCATAGGTAACACATCAGATGAATTCTTAGGAAGGCCTG[T/G]TTATCAATGATATTAAAATCAATAAGTTGACAAACATAACAAAACTGTTTAACAGAAAGA
285	CaSNP2213	TACAGTGCAATTTAAAACTCTCAGGTATATTAATTATAAAACACAATGAATATAATAATT[A/G]GCATGTACGCTCTATAATGTCCCATAAAAAAAGTAAATATTATCGTTATTAACTCACATC
286	CaSNP2214	TATATTAGTTTCTCAATCTTACTCTTAATGTCACTAAGTTTCCCATCTCCCAACCTTTAC[T/C]ATTTGTTAATATTGTATCCAATGGTAATATTGGTTCTGGTAATGACTTATCTGCTAGTTC
287	CaSNP2215	TATTTATTTTTGAGACAATTTCCTAATGTTATTCAACCATACATTGATGACATATTTAAT[A/G]TCATAACAGATGGAAATTGTGGCTATCACACTATTGCATCATTGCTTGAACACAATGAAG
288	CaSNP2216	GAAAACTCCCAGTGCGAGGAACATTATACCTAGAGTTCACATTATTAACAAGTGAAGAAG[T/C]ATTACTATCAGCATCAGCATTCACATCCTTACCCTTCTTCTGTTCAACAGATACATTCTC
289	CaSNP2217	TAACTTTGTGATCAAATTTCTTGTGATCTGTTTTCTTTTGAATCAGTGCGACTGTTCTAG[T/C]ACTTTGTATTTGCAACAAGCTAATGAAACATGTTAATTCTACTTATTAATACAGGGCTAT
290	CaSNP2218	AGCATTATTTACACTAACTTCTCGTCCATGCGAGTCTGCAATTTTTCCTGTCTTCATGGC[T/C]TGAGACAAACTACTTTGAGCTACTATATCCGCTCTGTCAACATTTTCAATGAAAACAACT
291	CaSNP2220	GTTTTATTTGGATAATCGGTTTAATCAACTACATATGTGTTAAGAGTCTCACATGAGATA[A/T]ATAAGGTCTGATAAAAGGTTTATAAGTAGTAGGTAGTTTAGTGGGAGTTATGTATGGCCC
292	CaSNP2221	CTATACTAGCACTTCTATGTAGAAAGTTATGCATGCTTCTTATCACATGTACCAACTGAC[T/G]TTTTACACCTCATTATCACACTTATTCACTTTTTTCTTACCAACAGACTTTCGACAGCTC
293	CaSNP2222	CCACGAAATAAGACATCCACCGCCACTTTCTTTCCCGTTTTCAATTTAGAACATGCAACA[A/G]TTAGCATTACAGTACAACACAAGATCCAGCAAATCTAAACTTTAGGCAGATTATAAAAAC
294	CaSNP2223	AACAAAAATAATTGCAATCCAAATTGACAGACTTCATAACCATGTATAACTATAGTCCAG[T/C]AAACTTGCGTATATGTTAAGAAATGGTGTGATGAGTGCTATTCATGTCGCTAATGAAAAA
295	CaSNP2224	TGCCAAGTTGAAATGGTAGGTGAAAGACCTTACATTGTCCTGCTGAAAGATAAAGACAAA[C/G]TGAAGGAGAAAGAAAAGGAGAAGGATAAATCATTGGAGAAAGACAAAATACAGAATAGTG
296	CaSNP2225	GACAAGTTCTCATACCCTCAATACTTAAACAAGTTCCCAACTTGGTATCATTCTCTTGTG[C/G]CAATGGTTTAACCTCATCTTCAATTATAAGTGGACTTTTTACATAATTGTTTAGACCAAT

297	CaSNP2226	CACTATCAAACATATTTATTTTACCATGATTCATCTTCATTGAGTATCCCATGTAGTTAC[T/C]TTCCTATTACCTGCAAATTTGGTCTCTGTTTTTACCTTCTCATTAATGCTTACAAACCAA
298	CaSNP2227	TCGATACAGGCATGTATATGATAAAAAAGAAACAACTAATCCAACAATACTAACACAACA[T/C]GGAAAGTACCCATAGCTCATTATATGCATTGAACAGTAATTAATTATGAAAAAGAGGATG
299	CaSNP2228	TTGGATTCTATTAGAGTATATGCCAATTGCTACATCACTCTGCTTTCTCATTTATATTTC[A/T]TTGTATTAGTATTTCCCTTCAGATAGTCTAGAGTATTCTATTTTCAATTATTGCTTCTGT
300	CaSNP2229	CAATTCCTCCCTAACCCTAACAATCCTCGCTTCCTTCTTAATCGCTCACACTACTTTCCT[A/C]ATTGGCGTTTGGTCTTCTCTTCAATTCAAATTCCTCCTAATGGAAAACCCCTCTGTCGTC
301	CaSNP2230	AGAGTTGTACGAGTTTAATATATACATACGTCTACCTGAAGGATGTCGGGGATGAGAGAA[C/G]TTGCACGGTGAAAAATGTCTTGAACAACATTCAATAAAGGTCTAACATCAACATGCTCTT
302	CaSNP2231	ATAATATGTTGATGGATATTGCTAGTGTTTTGGATCCTAGGTGCAAATTTCATATGGTAC[A/G]TATATGTTTTCCCTTGATGTATCAATCTAAAGAAGTTGTTGATGAGAATATAAAAAAGGT
303	CaSNP2232	TCATCAAAATCATGAGGGCTAGCTAAGCCTGAAGCCACTCTTGATAAGGTATCAACAATT[A/G]CATTAGCCCATTTCTTAGGGAACTCTACCGGCAATAAAATTTGCACACTCTGAGTTTACA
304	CaSNP2233	TACTCACCGATTATCCATGGACACAGGTTCATAGCTATTTCGCTCGTAGAAAAATATTTA[T/C]CTACAGTAAAAACAGTGGGATACATTTACTTAAGGCATATCCATGTATAGTATGACTCAC
305	CaSNP2234	CATGCATTTCAGGAACATTCTCTGATGCATGCTTTGTCTTTATGTTTAATCTAAGCAGAA[T/C]TTCAGACAATATCAGTGGACTTGACATTAGTATCAAACTAAGATTAACGAGATATCTTAA
306	CaSNP2235	GTATGATAAGTTATATAAAATATTATAATAGTATAGTATGCATGTTGATTGGGGGATCAG[A/G]CTAACCCTCTTACACTAGCCCTCGGTGTGGCAATAATATCTGAGTTGACAGGAAAAATAA
307	CaSNP2236	AGCAATTTCCAAGGCTCTGAACATGAAATCTCCAGAGAGAGAGAAGCGACACAGACACAA[T/C]TTTCATCATGTAACAACCCACACTGCTCAGGAATGGGCAGAAACTTTTGTCAGGTATACT
308	CaSNP2237	CATGAACCGAGTGTGAGTGCATGAGAAGTGGTAGAAACATGGAGGAAGGTGTCGCACGTT[A/G]CGGTCTTGAATCATGACTTATATATATATATATATATAAAAGATTAGAAGTATAAGTCAT
309	CaSNP2238	AGTTACATAATCCACCCACCAAACTGAAATAGCTGTGAAATCACACACAATCATACTGAG[A/G]TATAAATGCTGGGAATTCAGGTGTAAGTGGTAGAAATTCTCCTGTTTGTTAAATAGGAAT
310	CaSNP2239	AATAAACATAGTTGCTTCGGCGCCAAGAAAGTCCATAGCAGCAAAATTGTTGGTGAAGCG[T/C]GCCGTTCGAGCATGGAAGTACAAGTATCCAGGATCAAAGGTTGATGATTGTGCAGTCATA
311	CaSNP2240	AATAACACAAATGCATATGCAAACCGAACAGGCGAAAGCTTTCCATCGCACACTCTACTT[A/C]ATGCATATCTCCCTTATTACTCAACTCTTCCACTTACTGACTCCGCCTACTATGTCCATA
312	CaSNP2241	AGTTGAGTAGATGGTTAATTTAGATCAAGTTGAAAATTATGATTAATTATGCAGTTGAAT[T/G]ACCACATTTTTGTCGTGATTTGAATTAACCATTTACTCAACATTAACCACATTTTTGTAC
313	CaSNP2242	ATACTAACACGTGTTGAACCACTGATATATATTTTCGATCAAAAGTGTCAGTACTACAGA[T/G]AAATCTATAAAATTGGCCAAGCAATTACCATCACGACATTAGTGAAATCTATAAAATCGG
314	CaSNP2243	TCCATTTATAGAAAAGCTTTTAGTCCATTAGGGTTTTTGACTGTTGTTGTAATTGCATGA[A/G]ATCAACATATGAAATAATAACTCGTATCAAATCTTCAAATATATTGAGTCAACATTAACA
315	CaSNP2244	GCCCATAACTCCTGAAAGTGCGGTAAACTCAGTGACAACCGATGTGGATAGATCAGACAT[A/G]GCAAGTGAGGCAGCTTCCTGGATAATTTGTTGCATCTCTTCATCAATATCCAACATGCAA
316	CaSNP2245	TTCCATAAACAAATCTAACAGATCAACTTATCGTGATGTGCTAGCTAATGAAATGTGTAC[T/C]TATTTATAATCATCAGAAGAATCAATCTCATAATTAAATATATTTGACTATATTAAAATA
317	CaSNP2246	TATTACATACTACAATTATTAGCGAAAGAGAGTAGCTGAACTCATTGAACAGATACACTA[T/C]TACACAAAAATGTTGGACACTGCAATATCAAGAGATGCAAATAAAAATGAGGTTAGGGTG
318	CaSNP2247	TGCCTAAAGAATGGCTTCTTTTCCCATGTTGTCACTTCAGTTCCCATGCAATGTAATGCA[A/G]AATATAAAGAATTTGGTCTCCTATAAAATGAGAATAGTTATATTCCATCACATCTATGAT
319	CaSNP2248	TTCATAATTAGGGCATTTGACACGAAGTATTCTCACAAATTTAGGTCATTTTGACCCGTG[A/C]GGGACTAATATTCACTCGGATGATTAGGTCACAGCTTAAGATTTTTTGCCTCTCCACAAG
320	CaSNP2249	TGTCAGCATTGATAGCCATTCTATTGGTTCTGTTATGATTGATGAGAATATGAGTTTGTT[A/G]GTGTTGTTCTTTGTTTTCTTTGTTGGGTTAGGTGAATGATCTTCTTCTTGAGCTGTTGAC
321	CaSNP2250	GTCTTTATTCGGGTTGTAACATGGCCAAGGTTATGGTGAGATCATCCTAAGGAGACTAGG[T/G]CTGAAAATCAAAGAGGATAAATGAGTAATGATTAAGAGAGAAAAACAGGATTAAGAAATA
322	CaSNP2251	ACTCAAATCTCACCATTTGAATTATCTTTCTTTGTAATTAGCGTTGCATTTGCTAGACAC[A/G]TTTACATCTAACAGTAGTCACTCAAAAAAGTTCACCATATGAGCAATGATGGCTCACCTG
323	CaSNP2252	CACCCAATGATTAGGGAGTTCTCTGACTTCCATTTTTGATATGTTGGATTAGTCTACTCT[A/G]GTTGAGGAGTGTCTCCAGCCAAGTAGCTTAACTTTCCCTTGCCATCAAGAGCTAACTTCA
324	CaSNP2253	AGTGGATTGTTTTGAATATTTTTCTCCTCTAACACTAGATCACTCTAAGAACCAACAATT[T/C]AGAAATCTAGATATTGATGATCCTCATATACATGATTTGTGTTAGCTGAACTAGATAAGC
325	CaSNP2254	CCTCAACAAAACGGTGTCGCTAAGAGAAAGAACCGACACTTGGTGGAAACGGCTCGTACC[A/G]TGCTCCTTCACAATAATGTACCTCCTCGTTTTTGGGGAGATGCAATTCTCACAGCCTGCT
326	CaSNP2255	CAAGAATGATACTTGTCAGTTTATTTATTAGAAGTCAGTCAAAGAGTCATTCTCATACTT[A/C]GTGGAAAACATGAGAGTGCAGGTGGAAGAAAACTGTTCCTATAATTTGGATGTTTTCGTT

327	CaSNP2256	TCATTCAGGGACTATTGTAACATTGTTTTGCACATTTAGGGACCAAAATGACCGTTTACT[T/C]GTATCCGGATGCTAGTTTTCTGATACATTTCACTTTTAATGGAATGTATTCATTCTGATT
328	CaSNP2257	AATAATCTTTTTCAAAAGGAAAATGGTAGGGGAAATAAGCAGAGATTGTAATATTGCAAA[A/G]CATCTAAAATTTGCAGCACTAATTTTTGTTAGGGCCCGGGTTATAGGGATTTGTATTGAG
329	CaSNP2258	CAGCCTAACAATCAATATGGGCTGCACCTATACTACATTACCTTATTTGTGCAAGCTCCA[T/C]TATGCAATCGTGTTTGTTCTTGTGGGATTTGAACTGAACTGTTTCTGATTGCTTGCCTGG
330	CaSNP2259	AATAGAAGGATATTTGGCTGCCATTTACTACAACAACTACTTCCATTTAAAATTGATGGT[A/G]ATACCAACAAATCTTTACAACTAAATTTCATTCTTGAGCGTGCAACCATTACTGTAAGGA
331	CaSNP2260	TGTGGGCTCTCCAATTAAATTGGATCATATGTGTGAACTGCTTTAGTTATTTAAAGAAGT[A/G]GTCAATTTTGTTACTTCATTTCTCATTATTTACGAGACTTTATGGCAATTTTTACGGATA
332	CaSNP2261	ATACTTATTTTTCGTGCATCTTTTTATACAGGTACATAATGCACCAAGAAGCTTTTTTGG[T/C]GTAGAGGATTTCATCGATGATGATAACAGCCGACCTTATACATACCAAAAAGGGAAGAAG
333	CaSNP2262	TAAAAACAATTCATACAAGTGTCATGCACATTATTGTTTTTTAATAGTGCAATTTATAAG[A/G]ACATGTTCTAATGTCTAAGAACTCTATTAAGAATTGTCATCTGTTACGAAAATGCAATGT
334	CaSNP2263	TTCAAAAGACATGCCAAAATTCATAATAATTATAAAAGGTCGTTTAATTTCAATCGAACT[C/G]GTGTATAGATGGTATAGTTGGAAAAATAATTAATATTACATAAAAAATAGCATTCCTTTT
335	CaSNP2264	AAGGTATTACATTTACATTGCTATCATCAAATTCCATGGATATAGTTCCAATATTAAAAT[A/C]TATTGTTGTTTTAGCAGTTTTCAAGAATGATCTACCTAAGATGATAGGCACCTTGTTGAA
336	CaSNP2265	GGGTGTCGAATGTCTCGTCCTGCACAGGTTGAACAAACAATCTCGACCCTTCTCCTATAA[A/G]TAATAAATAAAATGAGTGTGTTACTGTGCCATATATAGAATAATATAATTGAAATGTCCA
337	CaSNP2266	CAATTAATGAACACGCATACAAATACTACTGTAGTGAAATCGACATAGTAAACCGAAAGA[A/C]TATAAGATGTCTAGACAACATACCACGAGAAAATGGGATTTTGATGGAGATAACCGTTGG
338	CaSNP2267	GTGTACAAATGATGGGTTAGGTCGGATTTAATGTCTCTTACCCGGTCTCAATATTTGATC[A/G]GACCAATTTTTAGACTCTAACTCATTCCTTAACCCGATCAAACTCGATTAGTTATGGGTC
339	CaSNP2268	CTAATTAACCTTCATTTTTATGAAACAAAGAGTTATCTATGAACTTTTGTATTTTAAAAT[T/C]ACTTGGAAAGTATATTATGGTATCACAACTCCAAAGTGTTGGAAATGTCACTTTATTGTA
340	CaSNP2269	TATATATCTCTTTGCATCAATTTTTATTTGTTGATTCCTGCGGCGATGTTATTGGATTTA[A/T]TTTTATTATTGCATGCTGCTCTGGCAGGACACTTTCCACTGACGTTCAGAACTCCTCCAG
341	CaSNP2270	CTTCTCGCTCTCTCCGATTATGACTATGACATATATTCCATTTATGCATGGCCTCAACTA[T/C]TAATCTTGCATCGGCCTGTGGAACAAACTCATAAGCAGGGGACAATCCAGAAACAACAGC
342	CaSNP2271	TCTTCTATATCTCCATGGAGGAAGACATTTTTTACATCAAATTGGTGCAAGTTCCAATTG[A/T]AATTAGATGCTAGAGATTATATCACCTTAACAATATTCATTTTTGCAATTAGAGCAAATG
343	CaSNP2272	TCCTAGGTGATCACAGAGAGAAACAGTCAGACAATCCCTTGAAATTGACCAATTAAAGTA[C/G]CTTTTACAATTAAAAAGTTGCAATTGAAAGTGATATTGGCAACTCAAACATTACATAACA
344	CaSNP2273	AATATTGGAGCTCTTGCTGTTCCTTGCACTCTCCAATTCACTAGTAATGATAGCAGGGTT[A/G]CAAGTATTGCAGGAAGACACTGATTTATAATCCTCTAAACTGTTTCTTCTCTTACACTTG
345	CaSNP2274	TTGGTTTTAAGCCACCTTCCTATGATCAAATCAAAACAAAGTACTTGAAGCAAAAGGTGG[A/G]GAAGACAACTAAGGTCAATGAAGAACACAAACTAACTTGGAATAAGACAGGTTGCACTAT
346	CaSNP2275	TTAGTCATGCTTCCCAACAGCTAGATAACTTTATCCATGACTACCAACAAGTTGCAAACA[A/C]ATAAATAGTCAAGTGAAACATCTTTTGTATTTTAGGGAAATGAATCATGTTGAGATTATG
347	CaSNP2276	AGAAAAGGGAAGGAGATTTAAATGGGTTCGATCAGATAAAGGTGGTGAATACATAGGTTC[A/G]TTTGAAGAGTATTGTAGAGAACACGAGGTCAAGCTTGAGAAAAAAGTTTCAAATACACAT
348	CaSNP2278	ATAAGGTGAAAGTGGGTCACCTTGTATAACTTCTTGCTATGGTATGATTGTTGTGCTTGG[T/C]TATCCATTAGATAAAACCTGAATTGATATATTTGGGGAAAAACCCGAGCAATGTATGGGC
349	CaSNP2279	ATTGAATGAAAAATGCAAAACAATCTGCCACTTTCCTCTGGCATTTGCCATAGCATGATA[A/G]TTGATGACTGACCCTCCCCATGACTAACCCCCTGACACCGCCAACTGAAGACACTGAATG
350	CaSNP2280	GTGAAAATGGATCTTCTCCATAAAGCACAATGCTGATAGGAGTGTGAATAGGTACAAAAC[T/C]CGCTTTGTGGCAAAGGGGTTCACCCAATCATATGGGGTGGACTATGAAAAAACATTTGCT
351	CaSNP2281	AAACACACAAGGCAAACAAAATAGAGTAGTAAATCTGACCTTTACAGTACTATCCAACAG[A/G]GCAACAGCAATATATTTGGCATCAGGGCTAATTGCAACCACAAGAACATCATCATTCATT
352	CaSNP2282	GTATGATATTAGAATTGGTTATTACTGAGTTATAAAATATAGAATCTATTTCTCAAACAA[A/G]AGTTAAGCAATACTCAGATTCTAACAATCTCAAAATCATTACTTTGTAACTCAAGGCTAT
353	CaSNP2283	AGCGGCTATAGTGCTACAGCATAATGAAATTTGGACAAATTGCAATTGTTCCGTGATACA[C/G]GATTTAGTACAAAGTGTTGTGAAATTGCAGCTATAACGCTATAGCCTAGCAAAATTTGAC
354	CaSNP2284	TGTGAAAGTTGATATTTCAGAGAGCTGTCGTAAAATGGAATTGGTACATAGATCTTTTCT[A/T]TTGTCAATTGTCACTGTACTCAGTTAAAATTTGAAGCTGTTGTTGTTATTCACATTGTCT
355	CaSNP2285	TCTTTTTATATAAGAAACATTATAGCACGGTACCTTCTATTATTGGGTGGAAGCCACACG[A/G]ATCCCACATAAATTTCACTCAATGATAAAAAATGTGTTGAAAAGAATGTGGTAGAGAACA
356	CaSNP2286	ACAAACACATTGCATATCATTCACAAACATTACCAATTATCATGCCAATAATTAGTCCAG[A/C]AAACAATTTTAGCATGCATATAATAATATTTAACATATATACATCATCATGCATATATAT

357	CaSNP2287	GAATGCAGTACTCACAGTTCTAATACTGATTCTAAAAGTAATAGTGACACTACTCGTGTT[T/C]ATTCTAGCAGGGCAAACTGGGATCTGAATACTACGATGGATGCATGGGATGAAGCAAGTG
358	CaSNP2288	ATGAGAAGCTTCAGCTTGTGATTCTTCAAATCAGATCCTCTTAGTCCTTGAACTTAGTTT[A/C]TGGACTTGATTTGCAGCATATACAAACAGTGCTTGAATTTGTTAAGTGGACAGGTTTTGT
359	CaSNP2289	CCCTCTAGTGTATTTCCCGATTTTCTGAGTCTAAATCCTTGCTTTGTACAACCTTTTATG[T/C]ATTGCTCTCTTTATATCTGCCATTTCCTTCCTTGTTATATTCTGTTCAATTATTTCTTTA
360	CaSNP2290	TTTAATGACAAAATATTTAATTCCAAAGTAACTCTTAATTACTTAGTAGATTTAAGATTA[T/G]AATTAAGTTCAAATGTGCAAGAATTCTCGTAATGATCTATTGTCTCTACAAATATCTCAA
361	CaSNP2291	AAATTGATGTTATCATTCTGGAGATTGATGTTTAACATGTTGGAGAATGATGTTAGCATG[T/C]TATGATTAATGTTAATATGTTGGAGAATGATGTTAGCATGTTGGAGATTAATGTCAACAT
362	CaSNP2292	TTCTAATGGCTCTTCGTGATGATTTTGAGGGTCTTCGTGGGGGTATTGTACATCGTTCCC[T/C]TCTTTCTAATGTTGAATTAGTAGTTAGTGAATTGCTGGCAGAAGAAATCAGACTTAAGAC
363	CaSNP2293	TGTTCAGATTTTGCGGTGGAATTGAGGTTGCATCTTTTGTCACAAGCTCTCGCATCCTTT[A/G]TAAATCATGGAGTACTCTCTCATCTCATAATCAACACGACGCCGTTTCATATGATGGTGT
364	CaSNP2294	GAAAGCTTAGACCACGTGGCAAACCTGGTATTGCTGATTGGAATCACTAGTTTTTGGGAC[A/C]TGGATTCTCGGTAGTTAGACAAAACCAGCAGTCGTGCAGTAGGACATTATAAGCCGTATG
365	CaSNP2295	TTCACTTAAATGAAAGTTGGAAAATACAAGGTTGTTGACTATGTAGCTCAACATAATCAC[T/C]GTCTTCAACCGTTAGGGTATGTTCATACAATTCACTCTCATTGACACATATTTGAGACTC
366	CaSNP2296	ATCAAAATACAACTCTTCACCATCCTCCATAAGCTGTGGAGGATACGCCACGTATGAAGA[A/T]GATTGGAAGCTACTTGATCATGGAGTTTTGGAGGACAGTGGTGCATGTGATATTACACTA
367	CaSNP2297	GTGTACCAGAATGGCACCATCCCAGTTTAATTGAAACAAATATTGATCTATTGCACTAGG[T/C]GTTGGGAAATGTAATTCACTTTATAACAGTGAAATACCATGTGGCCCATAGTGCCTCACA
368	CaSNP2298	CAGGCCATAGGACCCTGACAGACGGCCTAGCCTATTCTCATCCCTACTCACGAATTATGT[T/G]TTTAAGAGTTCGTGTCGCTCGGACCTCAGATTCTTGAAGTGTCTCAAATGGTTTCTAATT
369	CaSNP2299	CATATAACACGTAAGTAGAAGGGGACATTTTATTAATTTCGAACATTTAATTTAAACATG[T/C]CATGAGTGACGTACCATTTCCCAACTAAAATATCATATTTAGAATGAGTATACAAATATG
370	CaSNP2300	CTTTTCATCAATCTCCAAATATCAATGACTCTCCAACTCTTCCAACAGTTAGTCATCCCA[A/C]TGAGGAGGTATGTCCTCCCACTGAAGAAGTATGTCCTCCATCTGAGGATGCGCCACATGA
371	CaSNP2301	GGTAGAACCGTAGAAGGTATGACTTTTCATGTTTCTAGGTATGGTTATAGAGAAAGTTTA[T/C]GTAAGGCTTGAATATCAATTTTAGAGATAAATAGTGAGGATTCAAATAATTAACGGTGTA
372	CaSNP2302	TGGTTTAGAAAAATGGGAAATCTATAGCATCATCTTTATATGAGGTTGATCATCAAGTTT[T/C]AGAATTTATACCTCATTGCATGTTCTGCACAAAAGGTGCACCTAATGATTTGGTAGACAT
373	CaSNP2303	GAGATGAAGTCTTTAATCTCCAATCAAACATGGGAACTAACTGAGTTACCCATAGGAAAG[A/G]AAGCACTTGACAACAAATTAGTGTATCGAGTTAATAAGGATCATGATGGCTCCAAAAGAT
374	CaSNP2304	AACCTGATAACTCCTAAAACAAACTTACATTCACTAAAGTATTCGACATAGCATTTAATC[A/C]TTCAGCTGCAATAAAGAACAAGAAATGAGATAAATGATCATTTTGTCTTAACCCTCTCTC
375	CaSNP2305	TACTAAATTTTGCAGATCCTAGGCTGAAGTTGGGCATTAATTTTCAGTTAATCGGGCCTT[A/G]TATTAGTTTATATTCTAAGACTTTGTGATTTGTTTCATTCATGGTCCTATCTTTTCTCAT
376	CaSNP2306	CATATCATTCACACAGTACCAATCAACATAATATTCATAAAATTAGACGGCATACAATCT[A/G]GATATAAATAAATTCACGGCAATTCTATAGGTTGCTAAATTATATTTAATCCTCACTATT
377	CaSNP2307	GAACGAAGATTCCAATGGACCGTTACCCAACAATAGGGAATGGATGTTGTATCTTCCCAA[A/G]GCTTATCATCCTTTATTGCTTCAGAGTCATAGGGCGAATTTACAAAAGGCCAAAGAGGAT
378	CaSNP2308	CTTAAGGTGAAAGTTTTGGTGGTTGTAGATTACATGACACACTTATCAACCCTTGCAATC[A/G]CATACTAATTTTTAATCCTACATTCAATGAGTACCTTCCTTCACTCATTTTGTGGTTACG
379	CaSNP2309	TCTCTTTCTATTTCTCTCTAATTTTACTTTTCGGATTTTCCTTACCCTGTCCTGCATCGC[A/G]CGAGGATGCGATTCCAACGAGTAGAACCTATTTGGATGATGCTTTTTATTTTTTTACCAT
380	CaSNP2310	GGCCTGCCATCCATGTATGTGGCATTTGTTTATGTTGAATAGATAGTACTTGTTATTTTC[T/C]GGAGCGAATGTTTTAGATCTATACCGTATCAAGTTGGAATAGGTAGGACTTATTTTGTTT
381	CaSNP2311	AAATTATACGGAAGGACTAGTAAATGATAAAAGATATAGCAGCGATTTGTAAATAGTCCA[A/C]AACAACTTATTACAATAATGTTAGTTTCAATCATTGTGATATTTCAAGTAAGACAACAAT
382	CaSNP2312	ATGTTAGGAGGAGATAGCTATCAACGAGATGAAAGCTTTTGTGTTTCTCTTCTAGTTGAC[A/G]GTGGTTGTTGTCTTCTCACTAAGTCTTTGTGACTTACCCTTCATGTTGTTTATTTTTTTT
383	CaSNP2313	TGACTCAAATTACAATTCAATTAGTGTTGAACCTGAATTCGTGACCAAAAATGTTGACAC[T/C]AACTCCAATTGCTATGATTTGTTGTACAATGGGGACCAAGGGAGTGTGTTTCAGCAAGCA
384	CaSNP2314	TTAATCTTTTCAATAATTTAAGCATATTAGCCTACATAAGTTTTTGGCATATCATAGGTA[T/C]TATTATAAAATGATCATGTTAGATATCAAAATAGAATTTAATCTCATTGACCTATAGTTA
385	CaSNP2315	AAAAACCGATTCGGTTATCCAACTAACTCAAGTACACTATATCTAACCTAACCGCACAGT[T/C]AGAATGAAGTTAGTTTAGCTGATACAAGGATAGCTAATTGTATACTCTAATAATAATAGT
386	CaSNP2316	AACATAGTGTTCAAACTAAGAATATCGGTATTAGTCAATTGAGCTAAAACTTAAAGGACA[A/G]TTGTATGCAATTTATTATGTATGACTTTGTTGTTTGGTGGTTCTAATTTGAAGGACTAAT

387	CaSNP2317	AATAGAAGATTTATAAATCTAATGTTTGAAATGAATGTTTTATTGGTTGGTAAAGTTGAT[A/G]CAAGTATAATGATGAGTAACACACTTGAGGAGAGATTGATGGAAATTCGAGTGTGAGTTC
388	CaSNP2320	TCTGATCTATCACTGCCTCATTTACTCATCTATGTCACATCCGTAACAATTTATCTCTCC[A/G]TGTCTCAATTTTCCCTTTTCTTTCCTTTTCCTTATTCAAATGAGTAGACACCTTTCATTA
389	CaSNP2321	TTCTAAATGGAATAAACAGGGGTCAGTAATGCCTCCTGGTATGGTTGATGGCCTTGGTTC[A/G]CAGCTTCTTCAGCGCCGACAGCTTTATGCTGGTCCCATGTCTAAGTTGAGAATGCTTATG
390	CaSNP2322	GTCCCTTAAGCTAACACCTCACGGACAAATACTAGTATTGATTGTTTAATTGTTTATTAC[A/T]AAAGAATGCACTACAAGAGAATAGTATCAAGCTGATTCTTTATTTAGTGGATATGCTCTC
391	CaSNP2323	CAGCATTAAGGACCAACTTTATCATAAAACTAAAACCCATTGGATATAATAACAGTGCCA[A/T]TCAAACTACCAAAAGACATGAGATATCTTGGTACTTGAGTTTGAAAATTACCAATTAGAG
392	CaSNP2324	TCCACATAAACAACAAGATAGATGCACCCATGGGTTAGGTGACGATAAAATACAAAGTGA[T/C]CAGCTTCAGTGTGAATATTTAGAGGCATTTTCGAACATAGTCCCTAATAAAGTGATGTTT
393	CaSNP2325	GAGGTCATGTGAAATGAACACCTTGAGTCAAGTACACAATCACCATCCATGATTTCAGTT[C/G]AACTTGCCATAAGAACTTCTGTACTTTCATATCCTTCAGAGGCTATAGATGCATTTCCAT
394	CaSNP2326	TTGCACCAAAAATCTTCACTTATTCCTAAACTTCTCATTGCAATCAAAACAAAACAAAAA[T/G]GTAGTAAAATAGAACTAAAATTGAATTAAAAACATCAAATTAACCTATCAAATAAAACTA
395	CaSNP2327	ACCATGATATGTTTCTTTCTAGTCTCTTGCGTATTTACTTAACTTGACGATCTTGTGTCT[T/C]TGTTTATGCTGATTTTTTGGTACTCAATATTAGGTAGCGATCATGGAATCAAAGCACAAG
396	CaSNP2328	TCCTATGATCGGGCCACCCACCACTTATATCCACACACCAAGTCCAATAGTGTTAGGCTT[T/G]AGGGGGTGGGGTGAATTGGGAAAACCCCAAGTCTCACATAGAATAAAGATAAGTCACAAG
397	CaSNP2329	GTTTGAACAGGGAGCTCCCTTTCCTTACTCTTATTTATACTTGTTACTTTCATCGAGGA[T/C]AATGTGTAATTTAAGTGTGGGGGTATTATTTAGGATTTTATTTGTTATATTTAAAAAATT
398	CaSNP2330	TGATGATCTTGTTTTTCTTCATGACTATGAGTCAATTAATCTTGTATCTGATGCGAGCAT[A/G]TGGATAGTTGATAGTGATGCTATACTACATGTTACACCGAGGAAAGAGTTATTCACATCT
399	CaSNP2332	CTCGAGCCGTTGAGAGCCTGTACCAGGTCTCAAGATACTTGCATCGCCCCAAGTTAAACA[T/C]GTGTTGTTCCCAGGCTCGCCTAAGCACCATCCACCAGGAACAACTGATCCAGGACTTCGA
400	CaSNP2333	AGTCTTAAAGGAAACTAACAACAATTACAGATGTAAGGTCATAAGACACAAATCTCTTCT[A/G]GCATAACATAAACTGAGTCTAGAGTTCATATCTGAACCTAAAAATGCTGTTGCATTACAA
401	CaSNP2334	GTTGTTATTGAGTTCTACTAATATAGCATCTTGGTTCTATCATGGCTTTTCCTGGTGAAT[A/G]GTTTGAAACTCCAAAGGACTATCTGTAATGATTTATTGAGTTTTACGCACTTACCGTCCT
402	CaSNP2335	ACTCATAACTGAATCTTATATTTTTTCTTTTCTAAGCTTATAGGGACCAACAGTATTTGC[A/T]TAAAATATTATCATCATTGTTGACTCAGACTGTGGCAAAAAAATTCATGCTATTATATTGT
403	CaSNP2336	TTTACCCCCTCATCACCTTCAAGCATCTTAAAGTTTGAAAACTGTGTCCTTAAGTCATCA[T/C]CCTCATCACTATTTGGAGGTGTTTCAAGTTGATCAAAATCATCTTCACAATCTGCTACTT
404	CaSNP2337	ACTGCTTCCCAACTTAGAGGAGATTTCCATAGAAAATTGCTTCCTCTCAACGAAACAGCG[A/G]ATGAGCACAAGTATCCCAGTTGACCATTGAAGAATAAACTGCAATCCCAAAGACCAAGGT
405	CaSNP2338	AAAAAGGAAAAATAAATAAGAAATGTTTCAATAAGATTAATCCAAAAACACTCATAACAG[T/C]CTGTTGCTTCTTGACCTCCAAACTTGGGCACTACCAACAACTATAAAATAGTCTGCAAAT
406	CaSNP2339	TTTAGTGCATGATCTAACAAGTTTTCTAATTGCAATAGGAAGGTCTATATCAAGATACTC[A/G]ACATGACTCTTAAGAACAGAAGAAGACTTACCTGTATCAAGATGATATGTTGGATTCTCT
407	CaSNP2340	GCGTGCAACTTTATTTCTTTGGTCCCTGTATTGTTCAGTGGAATCTCTAAATGCTTGAGA[C/G]TTCAGTTGCAGCTCGTCATTCTCAGAGAAGCTACTCAAAACAGGAACGTTCAAGCTCCAG
408	CaSNP2341	CGTAAAATACTTACAATTACAATTTGATAAGATAATTGGTTAATATACTTTTAACAAACT[T/C]AATTATTTGTATTTTCTAGACCAGGTTTGTTACCACATGATGAACTTGAGTTTCTCCACC
409	CaSNP2342	GCATTGATTGCTGAATCAGATGTTATTTCATATTGGTTTAATTTGATTGAGGGTTGTCTG[A/G]TTCTCCCAGAACGCCTCACTGGGTCTGGAGCTGACTGAGGTTGTCTGATTCTCCCATTAC
410	CaSNP2343	ATCTGTAGTATCATCCCATTCAGGTGAATCCTCAAATGAGATAGGTTGTCTTATTGAACC[C/G]GGTTTACGCAGATAGAAAAAGTTGCTTGATGGCACCCTACTTGCCGGGCTACTAGCCGAA
411	CaSNP2344	TTTTTCCATGAGTTCTTTTGGAGAAGTTTATCCAAATTGGCTCTTAGTAACTTTGCAGAA[T/G]GAAATTGGAGTTAATATATATTTTTTGAGCTCTTAAGTCAGTGGTTTGGCTAGAGATATGA
412	CaSNP2345	TCATATACATTATCACAATGATTCACATAATTCCACAGCCTACAAATTTGTTTACTACAA[A/C]GGTTGACATCATTATAGCCTTTATGCACATCCATCCAGTAACATTAAGATTGGTCTTTTT
413	CaSNP2346	TCACAAAGTAAACAATAAAACATGTAAACTGATGGAAATCTACCTGCAAAACTATATTGC[A/G]GAGAAATGTCAAAATGATATACTCTAACCTGTGCAAGTTATCTGAGGACCCGTCTCTTAG
414	CaSNP2347	TTCAACATTATCTCGGTATTACAAATGATTTGGCAACACATGATTTGATTGATTATATTA[A/G]ATACCACCTATAGTATACGTAAATGAACACTACCAAATATTTTTTTAGGAAGTTAGATTA
415	CaSNP2349	TCACCAAAGGAAGTAAAAAGTTCGTGCAATTGCCTCTAATAGCGTGTTTTTGGTAGTTCA[A/G]CATGTTTCTACATCTGACTCTGAAGACTCAAGTATAGAGAAGACTTGACTTATAGGGCTA
416	CaSNP2350	GTCAGAGGAAATATATTAGAAACCTGAGAACTTAACAGCGTAATCCCATTGAGAGCTTAG[A/C]AGCGTAATTACATTGAGAGTTTAGCATAATACTTCTCATCTCCAAATATGAATTCCATTT

417	CaSNP2351	AAGTGGTGTTTTATCTCTATGTGTTTAGCTCTACAATGTAGGATATGGTTTTTGGTTAGG[A/C]AAATGACAGAAGTGTTATCGTTATAAATTGGAATGTTGGATTCTAGGATTTTGTAATCAT
418	CaSNP2352	AAATCCATGAAGATGACATATATTTTACCTATGAGAACGACTGCTTCTCTCATTTAGAGC[A/T]GTAGCCCCTACAGCACGGTTTTTCTGACCGATTCTCATTAAATTTACAACATCTCGAGTA
419	CaSNP2353	TTGACGACGAAATAATGGCCCAGAAGCATATGAAATGACAAAACCAAACAACAGGTCAAA[A/C]CGGTCACCTAGGTAACAAACAAATAACCACACACAAGAATAAGTCCTCAAATTTTTTTCC
420	CaSNP2354	GTCTCGGTCATTGTAAAATTATTATGCAAGAGCTCTAACTGCTAGCAGAGCACCCTTTAC[A/C]GAGCTTCCATATGCTCTATTAGGTCGAGTTTACTTGTCTTAGGCTGTTGTTTAAGCTCAG
421	CaSNP2355	CAGAATAACTGGGGAAGAATCAAGCATCCCTGGTACAAGTTGCAAATGATGTTGCTGACA[T/G]CCAGATGCAAAGGAAGGTTAAGAGAGGTTGAGAGTATCAAAGATCGAGATTCTTGATAGA
422	CaSNP2356	ACAATTAAAGTTTAAAATAACCGCTCTTCCCCAACCAAACTCTATACAAGTTTATGGCCT[C/G]CATTTAGCATTTTCTTTTTCCATTTTGAATATAGATTTGACAGTCACTTAATTTTACTTA
423	CaSNP2357	TTTCTGCTTCTGTTTTTTTCTAAATGTTCGCCTAAATATTAATCAAATTTCCCCAAACTC[A/G]TCATTTAAAACTAATAACCCCATTTTGTCCTTCGTAAGTTTTGATGTTCTGGTCAATTGG
424	CaSNP2358	GATATCAGTTTTCTGAACACAAAATTGAATAACACTGTACTGCTCTTACTTTCTACTTCT[A/G]ACTATTAACTTGCAAAATTGCAGGAGAAAAATTTGGCGGCTTCAACCCCACAAAAGTAAT
425	CaSNP2359	TGGTGGCGTTGGAGGAGAGTCTGTAATGGGAATAGGAACAGGTACAACGGAGGGCGAGCG[T/C]CGACGATGATATGTTTGTAGTGGTCGTGGAGGTTAGGGGCAGACTCGACAGGTTCAATAG
426	CaSNP2360	GTGAATTGTGACTTGTGAACGATTTTAGTGTAATAATTGAGATTGTGAACTGTAGTGTGT[T/G]AATTGTGAACGATTATTCTGATTAGTGTAGTGTGGATATGAGATTATGAATTTGTGAATT
427	CaSNP2361	TCTGATATACTTAAAATCTAAGAATTACTTCTCACTCTTTGTCAATGCAAAAAGGAATAA[T/G]AGAATAAAATAAACTAATTCAGAAAGATAGCGATGAGGAAGATCCCTCAAATGATGAAGA
428	CaSNP2362	TAATATGTGACATAAAAGTTGATATCCACAAGAACCACTACCCAAATATTTAAAATAACA[A/T]ATACAAAGAAAAACAGTTAAGAAACATTTATGTAACTAGGCGAGGATAAGAGCAAAAGAG
429	CaSNP2363	TTTTTTTTTTTTTTTTATTTTCGAGGGTGGATGCGTTGGGGTTCGAAGATGTATGCTCAT[A/C]TGTCAAATAGGGTATTGCGTTAATACAGACATGTCCAAAGTATTCCTAGCTCACCGTTAG
430	CaSNP2364	GAGGACATTCTACACAAAAATTGCGTGAAATAATAAGTTAATTGTATAGCTTTTATATGT[A/T]GTGTAAGACATGCATCGGTGACAAAAATGGTAAACTGAGATTAAGACTGTTTCCCTTTCT
431	CaSNP2365	AATCACATTCAGAGGTAGACAAACCAATTTTGTCAACTGGTTCATCTATTCTCTCGCGAG[T/C]ACTGACCATTGAAATGTCTCCATGTGCCACCTCGTACGATTCAGAAACTCGTCTTTTCAT
432	CaSNP2366	CAAAGTTAGGTCTTCGAACATAGACTTTTCTCTCGTCATATGTCGCGAGCCTCCATTGTC[C/G]ATTTACCATGACAGATGTTTCAACTTAGCTGTTGAGAACATTTGCAACATAGTTAATTTT
433	CaSNP2367	TAAATACCAGATTACATGTCTGTAATACACACACTCATTCAGATTTTTAAATATGAACAA[C/G]GGAAGAACATATTAGTTACTTCGGAGTTCAGAACAGATTAAAGTCGATATCATGGTGATT
434	CaSNP2368	ACTTTCATCGTTCATTGTTTCGTAACAATCAACATTTTCAATGAAAGAAGAAAACTGACG[A/C]ACCAATATCTAACTTGGTATCTTTCCTATATTGCTAATCACTGTATTAGCCTTTCATGTC
435	CaSNP2369	ATACCCATGCCTCATACTAGATTCTCACTGGGGAAAATATCTGCTTCATTATGTAAACTA[T/C]AGTGATTTTTGAAATCGTAAGTGTGATAGTTTTGTTATTTGATAGACTTGTCTAAGTGAT
436	CaSNP2370	AAGAGGGTGGACATAGCATGATTTATCTGATTCTTTATTTTCAGGAACACCAAGCAGCAA[A/G]TTCAATGCACCAGCTATTGATAAAGCCATTTTCTCCTTGTCAACCACAGCAGAAATCACT
437	CaSNP2371	AGAATTGTTGACTACATTCATTTCATTTCTCTCTCTTCTTCTCATTATTTTTTATTTAAG[T/C]TTCTCCTTTGACTAATGAACCGAGAAAAATGGTCGAAGATGTTTGAGACATAATCTAATT
438	CaSNP2372	TCAAATTGTTTTCACAAAGAAGGCCTCCACACTTAAAAAGATTAATCGAGTTTATGTGTG[A/T]GTCGTCGCTAATGAGTTTGATTCATCCTTCTTACGGTCTCTTCCAGCCAACGAAGACAAT
439	CaSNP2373	AGCACAACAAAATAATAGTTTTGTCTTCGTCATCAATCGTCACCCTAAACCTCACTAAAT[T/C]GATGATTATTCCATTGAAGTTATTGACATGTTGCAAATAAGATCATTCCTACATCTTCAA
440	CaSNP2374	GGTGTAATATATATTTTCATATAAGCTAATCTTTTCTTCACCAACACCAACATCCCCACA[T/C]GCCTCTATATTTTTCATTTACCAAGTTGTTTGAGAAAATCACTTTAACCGTTTTCATTAT
441	CaSNP2375	ATCAATCATATCTACATCATTCACATCCCTTTACATGATTTATTGTAGATCCAATATATA[T/C]CGTTATGATTGTCGTGTTACAAATAATGTTTGATGACCAAGAAGTATACAACTCTTTACA
442	CaSNP2376	GGAGCCGCCGACTAGAATAACCTATTTGGGCTCATAGAACAACTTGCGAAACACCAATAA[A/G]GATGTCCCGTTATCGACTTGTGTTTGGTAAGGCATGCCAACTCCTAGTGGAATTACAACA
443	CaSNP2377	TTTTCCTATCAGCTATAGTGTTTCTTTTCATGCTTTTGATCTTGTTCACATGGATATTTA[A/G]GGTCCGTTTAATACTTGTTCTTTGGATGGTTTTAAATATTTTTTGACCATTATTGACGAC
444	CaSNP2378	CGGGAAATAACTTGTTCCTTATCACGCACATCCATGTTCTCTGAGCCTTCCTCCACCAGT[T/C]CGTCTAATATTTCAACTGTCTAAAACATGACAAGGAGTTAGAGAGAGATAGGAAGTATGA
445	CaSNP2379	ACCTGCATCAGTCAAGTAAGAAGCGCAAGCGTGTTGTAAAACGAGGTACATCAAGATCTG[T/G]GACAAATACTTCAAGAGGTTCTGCAAAGGTAATAATAGCTCACTACTTTAACTGCATCTA
446	CaSNP2380	TCTTGCATGATAATTAAATTATTAACCAATAGCATTGGATGTAGGGTTTGTTATTTTATA[A/G]TTTTTTGGGTATGTATAAGTTAGGTTTATTTCAAACCTGAGCATAATATGTGTTAGGGGC

447	CaSNP2381	CTAATTTGACGATTTGCTTCGTTTCGTCGTCTATTTGATGTTGTTGGTGGAATTCTCTCT[A/G]CACCATCACCACCTCTATCACCACCTATAATGTTGAGAATAATTTGGCCGAAGGCACCTC
448	CaSNP2382	ATCCTAAAATTGATCATTGAAATTTGCAAAGAAAGTTTTGAGGTATCTGCAAGGAACTAA[A/G]GAGTATATGTTAATGTATAGAAGATCTAATCAATTAGAAATAATTGGATATACCAACTCA
449	CaSNP2383	CATGTTTTGTATGGGTTTAATTTTCATGCATTGACAGTGCAATTATTTTTACACATTTGA[T/C]AAATCATGAGTAATCATGTAGATGATTTTGTAAATAATTATGACGAAAATTAAATATTAT
450	CaSNP2384	GTTCTCAAAATTGGAATTATTGGAACATAGTTCATCATGAAGTTCCTAGAATGTGCTCCT[T/C]TGGACAGGTAATTGTAATTTCAAACGATTTTGAATTGAATCGTGTGATTTTGCTGTATAA
451	CaSNP2385	TAAAATAATTTAGATTGATTGAAAAAAGGTGGGGAAAAATATGCGGTGAATCAAGTATTG[A/C]AGACTTGTGCTCAAAAGGATGGTAGTGATCATAAGAAGAAGTGGAAGAAAGGTGGAGCAA
452	CaSNP2386	AGACATCTAACACTACAAAAACAGAACAGCAAAACATGCCAAGCAGAGGCAGAACATTAG[T/C]TCAGCAATAAACTAGAGCAAATCCCAATTAAGAACAATGTCATGGCCGATAGCGAGAAGT
453	CaSNP2387	AAGTCCTAACCTTTGCTTGAATGAACTCGATCGAACAGTACTTCTTGATGCATTACTTGT[T/C]GATGAAGACGCTGCTGTATTGCCAGGCATTGGAGGAAACTCTATGCCAGTCTCTTGTGCT
454	CaSNP2388	TCCATATTTTAATCTCACAATTCCCATATTTTTATATTAATCAATTTATGACTCAAAGGG[T/C]TACTGCCGTACGCAGCGAGCCATGAATGAATTGTTGGGAAATACAGTTTTTCCGTTCATC
455	CaSNP2389	ATTGTGATCACTTGGTCTTAAACAGACATTGTCTCTCGCTCTTCTACAGAAGTTGAGTAT[T/C]GTGTTATGACATCCACTACTGCTGAAATAATTTGATTGTGTTGGCTTCTATCTGATATCG
456	CaSNP2390	TATTCAATCTCTTCATTTTCAGACAGTATAAATACACAAGTGATACTGTTAAAACTGTAA[T/C]GAAAAAAGCACTTCCTATACTAATACCAATTATAACAGGAAGTTGAATGCTTCTCTTTCT
457	CaSNP2391	ATGCTTTCTCTATCTCCAGATTACAACTCATGACCGCCCAATAAGCACGATGCTCTATTT[T/C]CATTGGGAGGTGACATGCCTTACCAAACACAAGTCGATAAGGAGACATCTCTATTGGTGT
458	CaSNP2392	AAGCTCGTAGAAATGTGTCAAGAGTTATATATTTGTACTATAAATACCGCCTTGTACTTG[C/G]CATTAAACATCATGAAAATGATGAGTTTTCTATTAGAAATACTTTTCTGCCTCTATAAAG
459	CaSNP2393	TCATTGAGAAAGGTTATTTATTGTACTTGAAATTATATTAGACTGTTATCATTGTATTCA[A/T]TTGTTATACACTCTTGATCTCAGTCAAACTCATTATTGTAAACCATTCAAGTTATTGTGT
460	CaSNP2394	TTCCATTTCATAACAACTTAGTTTAGTTAAATGTCAACATTCATAATTTATAGCTAGTTT[T/G]CCCTTATTTTAGTTAGAGACTGTCTTCAAAGCACTGACCATATTCTGCTTTCTCATTGAA
461	CaSNP2396	AGATCAACATGATTTTCTAGAATGCAATCGCTCACAGATCAACACATTCTAGAGTGCAGC[C/G]TCTCATAGACCAACACTAATTATCAAGGTGCAATCTCTCATAGATCAGCACGACGCGATA
462	CaSNP2397	TATTCGTGAAAATCCCTCATAAATATTAGTAATATCCTGCTCTCTTTTTATATTAATTGT[C/G]CATTTATTCTTAAGGTTTTTTTCGCACACAAATTAAAATTAAATAAATTATTGGTTGAAT
463	CaSNP2398	TTTAATGCACGTTGTAGAATTGTTGTGCCTATAAATGACAATGAATCAATGGTGTTATTC[C/G]TTGTATTGAGAAAGTACTACATAGTTGTGTGGGAATGGCGGTCTAATGTTGGCAATTGCA
464	CaSNP2399	GCTGATTATTCAAACAGAGCCTTGATTGAGTAATTAACAAACGACGGATTTCTCTCGTGA[T/G]ATGAACATAAAGCAAGATCACCGATCAATCTTCACAGCTATTCTCGAATCGAAACCATAA
465	CaSNP2400	AACTTGCTCAAATTTTCATTAGATAATTAGACAAGCCCTTTCATGGTGTGCAGGTTAGTT[C/G]CTCACCAACCATTATTCTTATTCTGTCTTCAGATAAGCACCTCAAAAGCATCGTTGTTAG
466	CaSNP2401	GCATTCTCAAATAGCAAAACAATAAAATTATCGAATAGTAGACATGTCCTTGAGCCAAAC[A/G]TTTAATGGATATACAGAGAAAACATAAACATAAAAAGCATACCATTTGAATGGTATGCTC
467	CaSNP2402	ATTCGGTTTTCATGAGAGGAACGAGCACATTGAGACAGATTGTCACTTTGTACGGGAGAA[C/G]ATCGAATCAGGCGACATCACTGCTAGCTGTGTTAATTCCAATGATTAATTGTCAAATGTT
468	CaSNP2403	TTACAACTTACAAGAGCTGTAATTATGTGAAGGCAAAATATTAGCCAACAAACAATGGTG[A/G]ATAATTTGTTATGAGGTGATGCCATGGTTGTGGAACTTTGATTTCACTCTTTTACTTCAA
469	CaSNP2404	TCATCCCAACTCTTATATCCTGTACAATTTCGAAGACCCAATAAAAATGCAAGGGTCCAC[T/C]CTTTCTCCCCATTATTCAAAAGAATTAACTGCTATTTCCACTGCAGAAAGAAAAGCAAAA
470	CaSNP2405	ATATGGGTAACCGTGAAAAGGTGTGACAAGAGCTCCGAGACTTGAGTATGTCCATTTGAA[T/G]TCCTTGGTTTCTTGGAGGTCATTTCAACACAATCTTCTTCGATCATGAAAAAGTTGGAGG
471	CaSNP2406	TAAGTACAATTTATGGTTATATTGGATAGTCAAAGCTATAATATGGACCACCATCTTGGG[T/C]GGTCTATGGTGTACTATAATTTTTAGCCATTAGATTAATTTGTACTCATAATATTATGGT
472	CaSNP2407	GACACAGGCTTGTTCAGAAAATGGAACGGCAACGACGAAGACTATTTAAGAACACCAACA[T/G]CACGCGATTCTCTCCCAGCAGACGTATCAGGTACGAGGAACATCACAGTAAATCCTGATT
473	CaSNP2408	TTTACTCAGCTCAATTTCACAACATAAGCAATGTTTGCTTTTGCTATCAACTCATTAGTC[A/C]CTAGATGAGAAGAAGTTGAGATATTAATAATGGGTCCTCTCATCAGTCTCTAACTGCAAA
474	CaSNP2409	TTCCAGCACCTTCATTTGGTATTGGAATTTAGTATCTTGATTGAGCACTTTACTATGTAC[A/G]AGTAAAGATCCATTACTACTGGAAATACTAGTGAAATAGTTTGAATTAGTGTATGTTTTT
475	CaSNP2410	GTAAGTTATGTACGGGTACACAATTTACCAAGAAGAATATGTTATCACCCTATTTCTGTA[A/G]CTATCAGGACCTACGACTGAACTCTGTTACCAACTTTGTCACAAACCGAAAACCAACACC
476	CaSNP2411	GAACCACTGAAAAATCATAAGAAATTAAATTAAACTTCAAAGGAGTGCAGATTTCATCGA[A/G]TGTCTAATCAAAAAGCCTAAATCTCTTTTTTGAATCCAAAGCAATCACAGGCTTGAAACA

477	CaSNP2412	CAGTAATCTTTAAGATTTATAGTACTCATGATCTCCATATTCAACTGAACTTTCCATGTG[T/C]AGGCCATAATGCATAAGCTAAGTTATGTGCATAAAATACAATTATAAGGCACCAAAGTAG
478	CaSNP2413	CATTCACTCCAATATATGAACGCATTGTAATACCTTATTTTGATTCCAACGGAGGGACAA[C/G]AGTCATCCTGCGGTCCTTTTAACAACAAAAAGCCATTCGGTTAAGCTAGTGCGCTGACTT
479	CaSNP2414	TTTTGACATTGCATGTTGATTGTGAACTAGTTCTGCCATTGCTCGTTCTATTGTGTAGGA[T/C]GTTGTATCGGTACCCGCAACAAATAAATCCTATTATAAAGTCACAAAATGAAATATAAAT
480	CaSNP2415	GTTATCATTTCTATTAACTTATGTTATTAGTATCTTCACCCTACTTTTGAGCATATGCCT[T/C]TCTTTCTATATTTCTAGGTAGGACAAGAGGTCTAATAGAATGGTACTGAACAAAGTCCGT
481	CaSNP2416	TTCATGCGGTTAGACTAGTGACCTAGTAGCCTAACCAAGTTGATGGCAAACCCTAGTCTT[A/C]AAACACTAGAACCTACCATAGGAATAAGAAGTGTTCATCTGTAGGGGCCAAATTATTGCA
482	CaSNP2417	CCTATACTTGTTGGCTTTGTAGGTGTTATCCATAATCAATACAATAGGAAACATGTTCAA[T/C]AAGGTTATGGAATCTGGGTCTGTCCAAAACATGTCATTCACAAAGTTGGAGTCTTCATGA
483	CaSNP2418	CTGGTTGAGACCAAGGCATTGAGGGTCACAAACAGGGTGTAATGAATTCAAACTATGAGC[A/G]TGTGTTGTTGCCAGCTTCATCCAGCAATTGTGGACAACCTATGGCGGGGAATATGCTTGC
484	CaSNP2419	GTATTACAAAAACCATACCTCAATCGCACTTGTCCATTATAACGCCACCTTATGCTTCTA[A/C]ACCTACGTTTCCAGGTACTTATTTTGTTTCTCTTTTTATGTTTTTTTCCTTCTAACTTTC
485	CaSNP2420	ATTACAACCAACATAATGAATTATCTCGCGCAAGCGATCGAGCCTGCTCAACCATCGCCA[T/C]CACCTCCACGAGATGTCACAAACAAATTGTTTTATGACTTTCAGTAGCTTTTCTAGGGAG
486	CaSNP2421	TTCAAATATGAAACAAATAGTCACATTTTCTACATCCTAAGTAGTGACATGTGAAATCTC[T/C]GGTAAAGGTACGACGTGAGTTTAACTCTTGATATATATTAAACTGATGAAATAGATTTGT
487	CaSNP2422	CATCTGCTTAACGAGGTCCTTTAATGAAGGTGCATTGTTTTGCTGTTGTGGAATATTTTG[A/C]TGCTGCTGCTTCCTAGGTTGCTGTTGTGCAGATGAATTCCCCCATCTCAGGTTGGAATGG
488	CaSNP2423	ATTAGCTGTGTAATTCTGTAAACTGCATTAATTCTATATACTGCATTTAACTGTGTAATT[T/C]TGTAAACTGCATTAATTGATTTGTTAAGTACTACTACGACTACTATATTTCTTGTTCCTC
489	CaSNP2424	TCCTTTTCATTTTCATATGTAGTTTTACAAAATCACATTCATACCATTTTACATTTAATC[T/G]TACAAGTCTCTGAGGTGTAGCTCAAGTGGTTGACCTGGGGCATCTGAATGAGTTTGAGAA
490	CaSNP2425	AAATCCATTCAACATGCAAAAGTTAAAAGATAGCGTATAGCCTCGAGTTGGGCAACTGGC[A/G]CATAAGTTTTCTCATAGTCGATTCCTTCCTCTTGGTTATATTCTTTTGCCACCAATCTAA
491	CaSNP2426	CTTGTCTATAACTAAGGGCAATAAAGTAAACTTGTAGTATCAATATCTCATGATAATATA[T/C]AAACATCAGGGAAGTTGGTTCTACTTACAGATTGGTAGAAGCAGCAACCTTGATTTTAGC
492	CaSNP2427	AGCCACACCAGTCCTCAGAGCCTTCTTACCCGTATTCATTTTTGGATCCGAGACGTTATC[A/G]TTAGATGCAATAATAGATTGATACTCTTCTTCAACATTGCAACCAACATGGGTAGCTAAG
493	CaSNP2429	CGTAATTCTGCCAAACTTACCGTTAATTCAAACATGCTAAGTATCTAGTAGCAAGAATGA[A/T]TTTTGAAATTCGGAAAGTAGTAATTACAAGCTAACATAACAATTAGAGTTATAAAGAACA
494	CaSNP2430	TAATGTAAATTAACTAGAACATTAATTGCATCCGTTAGTAAAAAGAATGTATGACCTACA[A/G]ATCTAGAGTATTACATCCGTAACAATTAATTCCTTTTTTCAGAGGATTAGTAACTATTAA
495	CaSNP2431	GTAGGTGTTTATGGAATGGTGGAGTTGATAGGTGTTTGTGGAATGTTAGTGTAAGTAGTT[T/G]ATGTTGTTTGGTTTGGAGTGTTGGTCTTGGTGGGAAGAGGATGTCTTTTACATCTGAGGG
496	CaSNP2432	ATAAACATGTTGCTGTTTAGCATATGATTGCTTCACGGAAAATATATATATCGAAGGTTT[T/C]GAGGGGCTATTGCTTTTTTAGTAGACAAATTTATTAAAGTCACAACCACAACATACCATC
497	CaSNP2433	GTAAATAATCAAACCTAAATAAAAAGAATCAAGAGAATTGACTACTTGAATAGAGAGGTG[T/C]GTAATGACACCACAACATTTCGATATGTGGTTATGGCTCCCCTTAAAGTTGTATGGACCC
498	CaSNP2434	CGATATTGGAATGAAATTTGGAAATGGAGCATATAACACTATGGACAATGGGGTTCTGAG[A/G]TTTGAACATGTACGAATTCCAAGGAATCAAATGTTAATGAGGTTAGTTTATCACTTTATC
499	CaSNP2435	AGAGTCTATCAGCCTCATCCAAAATCAAAATCTGCAAGCTAAATCACAATTAAAATCCTA[C/G]CCTTACTAATACACAAATGTACATTGTCCCTACTTTTGATAAACAACTTAATTAAACACT
500	CaSNP2436	ATAAATTATGAGTGTATAAACTAATATTTCATGTAGCAATTTTAAATTATTTGAATAAAA[A/G]TAAAGATGACAATTTCTTCGGTCCAATATGGTTTAGTTCATATTGATCAATTATATGTAG
501	CaSNP2437	CAATGTAGCATTGTTATCTATATTGAGTCAAACTTTCTTTATTGGATTTGATATTCTTTT[T/C]GAAAGTATACACAAATGTTGATTATATTGCTTTGGAGAGAACATGTCAGATGAATTTCTT
502	CaSNP2438	TCGTATGATAATTGATGGAATCAGTTTGATGAATGTTTTCTGATCTTACTACTCTAAAGT[C/G]ATCTCATCCCTTTAAATGAGTTAAATGTTATTCTCTTTCATGTTATTTGGCACCTATCAT
503	CaSNP2439	AGAGGAGGGAATCAAACTCGCCGATGAATTAACTATACATTCTAAACACAATTGTTGATG[A/G]TTAATTTTATTTTTAAAATATTAGTGCTTCACTTTTGAGTAGATACACTCACAAAATATG
504	CaSNP2440	TATGGCTGAGATGTAGATTGTGGGTTGGTATTGATTGATTGATCGGGATTGTTATTGAAT[C/G]GATTGCAATGTATGATTTGAAACTTTGCTCATAGTAGTATTAGCTATTTCTTGCGGTTCA
505	CaSNP2441	GCAGTGTATTGACCAACTAAGCTGTGTACTCAACTGATTTTATTAGCAAACTTATTGGTT[A/T]TTTTCAGTGATTTGTTTGATGAAAGAAAACCAGCCTTGTAAATTTCCTACCAATTTGATG
506	CaSNP2442	AAAATACTTTGTACTTGTATTGACCTTGTTAGAAGAGTTTTCAACAAGTAGCTAAAGTTA[T/C]GATCAACTAGACTTTGTTTTGGATAATACAAGGCTAAGAGCCTTGGGTTTTAAGAATATA

507	CaSNP2443	GCATAAGGTCATCTTGAATCTTTCCATTTGACAATATTACTCACAAATCACAAGAAACGA[A/C]TTCCTTGTAAGAGATTAATGAATAGATTTGAATGTGGACAGGAGAGCTATCATGAGTAGA
508	CaSNP2444	GTCTTTGATACCATGTTGAGAGACAATCAAACTCAAATCCAGGCTCCAATTTATTTTTGT[A/C]TGGAACGGGGTATCAAATTTGCCCAGTTGAAAGTTACACAGTGCATATATTCTCAAATTT
509	CaSNP2445	TGACCAGATAGAAGTCACAAACCATGTTTTAAAACCCTAATGGACATACTAGTAATGAAC[A/T]ATACCTGTGATTGAGCCAACAATGATCAAGCGCTTTGAAGGGTAATCAGATTTCTGCAAG
510	CaSNP2446	TGGAAGATAGTAGGGCATTTACTTTGAAATTAAGTGGGAATATACATCTCGGTTTGACTC[A/G]CATTGTAGGTTCTTACCTTACAATAATCCGTTTAGAAGAAATAAAAATACATTTATTAAG
511	CaSNP2447	TTATATGGAATGAAATTTTATAAGGAATGCAATATGTAATATAAGTATTTGACGTGCCTT[T/C]ACTATCAAGGTGTCTGCTTACTGTTTACATCATTTTGCGCATCTGTTTTTTACCACCATA
512	CaSNP2448	GTCATGTTAATCGGTACAAGTGAAGGTTGTTGACAGGTGCTTTGAAGCCAATAAATGTGT[A/C]GAATAATAATATTATGTTAGTCAGGATTATAAAGATTGTTTTGGTATAGATGCATGAACC
513	CaSNP2449	CAACATTTTCAATAAATTATGGCTTATTTGACGTTATCATCTTTTTTTAGGCAACTTCGT[A/T]TTATTAACCTCTCATTCTCATTAGTATTAATTCATATCTCTTAATGAAATACTAGTGATT
514	CaSNP2450	TTAATCCGACAGCCCAACTGTTTTAATTTCTAGGTTGAGCTTTGTGCTGTAAACCGAGTT[T/C]GGTTGATCGGTTAATGCGTGACATCGTGTAAGAGATTGTCTGTTGTAAAGTGACACTATC
515	CaSNP2451	CGTTTCACTTGAGTCCTTCACTGGAAGATCTAGGCAACTTTTGTCATTCAACAATTCTGA[A/T]TCAGGATTGGGTGAACATGGCTGTTTTGAATCCTACAAATAAGTGGAGACTTGGAATTAG
516	CaSNP2452	CAATAGACTATAAGTTCTTCTTCAACCCTTTACATGACATACCCCTTGTATTTTGTAGTA[C/G]TACCATCATGTAGTTTTCATTTAATTGTACGGACTCCAACAATCTCTAAGGCGTAGTCAT
517	CaSNP2453	TGGTGATATGTATCTTTGAGCATCATTATCATTTTATGACATATCATATGCATCTTTGTG[A/G]ACGCCTGTGTGGCTGGTTTAATTTTCCACATTGGTATGGGTGACTTCTGTGTGGACGCCT
518	CaSNP2454	GAGTGAAATAGTTTGACTTTGCATGTATATAACGATATTAGTGAGACTTCTAACTTCACA[T/G]TGTCTAACTTTATTGCATGAGACTAATGGTTTCCTTTGACAATTATACCATTTACTAGTA
519	CaSNP2455	ATCTGAATTTCATTACAATTTATATTATTTGTTTATCTAATTAACGTTATGCATCAAGTT[A/G]TAGCTTACCATTTGTGTGTCTCTGCATGCTCCTGAGCAACCACATTGTTGTTTCTGTCTG
520	CaSNP2456	CCCAAAACTCAAGTTAGAAGCCTAAAGAAGAAAAACAGCCTCCAAATTGATGACCAAGTC[C/G]ATCTCTAGACTGATGAGTTGTGAGCAAGGACAACAAGGACGAAAGAACATTGGTGCTGCA
521	CaSNP2457	CAAATAATTTCCTCTGGGAAACTATCAGATCCACTCTTTGAATAGGAAGAAACCAGATAA[C/G]AGGATGGTAAGAATATCATATGAAGAGAAAACTGTATCCTCCAAGACTACAAGCAGACAA
522	CaSNP2458	CCTTGGCGACATTAAATTATAGCGGATTCGGATTTTCTCTAATAACATTATGTAGTGATG[A/G]GTTTAGATTCGCTCTGATGACATTACGTTGCAATGGATTTGAATTATCCTCGGTGATAAA
523	CaSNP2459	TGATGTAACATGAGGTGGGAACATAATACATGCTGGTGTTTGCAACACTTTAGACAGTGG[C/G]GTGACGATAATACCATGCTTGGCGACGAATGAGATACTATGCTTTTCGGGTTGACACTAG
524	CaSNP2461	GTAACATAGCTCTCTATCACTTATATATATTTTTATGCTTTGTGCAGTATTTTTGTGACC[C/G]TACTTCTTGTGTTGTGTTGTGATTCTGATTATGTAACAGGTGATTTTATTCTCAGACATT
525	CaSNP2462	AACGTTAACCTAGGTACTTTCCACCTTTAATAGCTAATTATATTCAAATTGAAGTGGCTG[T/C]AATTTGCGTAGAGATATTTTCTTCCAACTGTATGATTTGGGGTTGGGGAAACCTATTAAT
526	CaSNP2463	TGGTGACTTACTCGAATTCCGAGGCAGCAAGCAATGAAAACATAAATAATTTGACACATA[T/G]ATTTAACTTGGTTTACCACTAACCTCGCTACATACAGTTCCCTCATTCAGAAGCCTTTAA
527	CaSNP2464	TGCTTTCTAATGTCTATTTAAAATATAATCATTCATTACATTGTGTAGTAATGTATAATA[A/G]TATGGTGTCTTGATTTTCGTGTTCGAGATTTTTAAATGTTTTATTGCTTTCAGGCACTGG
528	CaSNP2465	TAAATTGGTGGTGTGTTGGATTCTAATTCCTGTGCATAGGCGTATTCATAGATTGAATCA[C/G]TAGAGTTTGAAGCGTCAAGGGAAATTACCTCATGTAAGGTCGAATTGAAGTCATGGATGT
529	CaSNP2466	CATAAATTTCCATTTGAAAACTTGAATGATTGTGAAAATTGCTTATTGGTTAGATAAACA[T/C]GATAGTTGAAAATAATCTAGGAGACTAGGAGTATATTGAATATTACAGGCCAAAAGGCAT
530	CaSNP2467	CATACTATTTGTACCTCTTTCTTATTACATAAATTAAATTAACCAAATGGTATGAAATGA[A/T]TGGACACCCCTTTGTAAATCAATTAGTGTGTTTATCATATTAGTCCTATAATATAAATAAG
531	CaSNP2468	GTAAGAGGAATATGCTAATTGTAGCATTCTTTTCTTTGGTGATCAGAGGATCAATTCTTG[A/G]CTTATATTTCTTTTGTCTTCAACGCTTTGACTCAGTACGTAGATTATCAGATTCTTGATG
532	CaSNP2469	ACGGGCTATCCCGCCCTGTGATTAGCCCGCCAAAAAGCACATGTATCAGTGTCATGCACT[A/G]AATACGCCGTATGAAATGTTGGTGGTGTACCCTTTAGGAGGTGAATACAACCTTCAATCA
533	CaSNP2470	GAAGCATAGTTCTGGTAACTAGAGAAAATGCAAACCTACTCTTTCATGTATTCTAATTCT[A/T]ACATGTAAAATCCTATGGTATTTCTGTTTCCAATTGGATTGATTTAATTGGTGTTTTCTC
534	CaSNP2471	TTTCTTGCATGAGGATAATAACAACGATAACCCTTTTGACCATCCTCATAACCAAGAAAA[A/G]CACACATGGTTGAATGAGAAGACAACTTACTGCGATCTACTTGAGGGCGAAGAACAAAAC
535	CaSNP2472	GGATGTATCATTCATGTAAAATATAGGGGGACTCGTATCCAGACAGACAGCATGAAACTG[A/G]TTGCTATCGGTACAGGTGAGTTGTGAAAAAGATGCAAAATCACGGTTTCTGATGGCTTCT
536	CaSNP2473	TGTAGTTGGGCCAGAAAGAGAAGGTATCCAAAAGAAGCCTTCATTAAGGAGTCCCTTAGA[A/T]TATTATTAGACAAAAACATAACATCAGCCATGGAAAATAAAAAGATATGTATATATCTGA

537	CaSNP2474	TCTTCTCTAAGCATTATGCAGGACTTGAGTATATGCACTTTTGATGAAGAAGATATACAC[T/C]CGAATGTTAGAAATTATAGATAAAATGCTCTAGGAAATTTATTATCTATAGACAAAATTT
538	CaSNP2475	TAACCCACATTTAGTCTTGGTAAAATTAAACCCACCAGAGTTGGTAAAGGTACATCAGGT[T/C]GTGGCAGGTGTGTTGAGGGATTAGTCGATCCATGCAGGTAATAACCATGTGGTTGGTGAG
539	CaSNP2476	TTTAAATATACATATTTCTTCTTTATAAATAGGCCAAGTTTATGATCATATTGCTTCACT[T/C]TTCTTTATATTTCTTCACAACTTCACTCTTCTTTACATTTTCACTCTTCTACTCTTTATA
540	CaSNP2477	GCCAATGACTATGGACCTTCTTGGGATCCAATTACTAATAATACCTTCAAAAGGAAAACC[T/C]GCAAGAAGTGATTAATTAGATCCTCTACCATAGCACCCGGTGACAGTTTGTTATGGGGAG
541	CaSNP2478	TATGTAGGATATATCCTCTAATGATCAGATTAATTCAAAGAAAAAGACTTGGTACATTCC[A/G]CATGTCTAATATAGGGATAAGGGCCTCTTATATATTGCCTCTAATGGTACAAACAAAGGA
542	CaSNP2479	TTGATGGTAGCTAATATGTGAAGATGCACAAATTCATCCTTTTAAATATGACATTGTTGA[T/C]ACTACAATTCTAACTCCAATGTTGTATGTGTTGATTTCCAAAAACAAGATTTAATGTTTA
543	CaSNP2480	GGATGAAAGAGTTACTAACCCAAGTGCCATACGAACTTTAAGTTGCACTCCGTTTGTAAA[A/G]TTGAGAACATAGGCATCATTGACAAGTACACCAAGGGATGAGTAGTGATCTGCATACTCA
544	CaSNP2481	ATATAAAAAGCTCGTCACACTGATATCACGCGACTAGATCGAGTAGAAGTTCAACTCAGC[A/G]CTCTCCAACTCGCCTATGCTTGTAATGGTAGCGTGAACTCTGACGGATCTAAGAAACAAC
545	CaSNP2482	TAATTCTATAAACAATTGTTGTTTTAGCTAATCATCAGAGCTTGTAAAGAGAGATTGATA[T/C]TCATAACAAAGTCTTTGTGGTAACAATAATTTTAACTACGCGATAATATCATATACTTGT
546	CaSNP2483	ATCAAAAGCACAAAAAATCCAAGATCCAGAATCCAATAGAAAATTAGTGGGAGAGACAAAC[C/G]GTACAATCACCAGTTTGTGCAACATCTATTGAAGTCTTCTAGGTTTCTTTGGATTGGAGA
547	CaSNP2484	TTTACCAAAAGCGAAGGCGTACCATGCAACTTCCAACAGTGATTAATAATGTGTCTAAAT[T/C]GATTGCAATGCTCACATTTGGGATGAGGTTTAGAGTTGGACGATCCTCTGCGAGAGCAAC
548	CaSNP2485	ATATACTGCTGTATCTACTAATTCAACAAATGGACCCTCCTACGTCACAAATTAATTAAT[C/G]GAATCAATGTTACTTCTACTAACGTCTCTAATCTAAAATACGTAAAGTTTTGCGAAGAAG
549	CaSNP2486	AAACAATCGGAATATAGCGCTTGCGGGGCAAGAGAAACTAATGTTATAGAAGAAAAGATA[A/C]GATACCTTGCAGTACCAGGAAGTAAAACATCAACTTCCCTGTCACCTATGGAGTATGCCC
550	CaSNP2487	GAAAAAACGGGCATTGACATGCCCGTCTATCTGCTTCTTCTATTGTGTTTCGCTGCATAT[A/G]TATATTTTATTGTATAATATTTGTTTCATTTTAATTCATTTGTTTCAGTTTTATTACGTA
551	CaSNP2488	TTTATACCCCATATTTTCTACTTCTACATATTAATTCGAGTTATTATTGGTTGTGCTTTT[C/G]TTTCCTACACCTGCGAGTTGCTCATTACGCCCCACTTATGCCTCAGACTTGACTACCCAG
552	CaSNP2489	ACAGAGAAATGAAAGACCCATTCTCTGACTTTTCCAAGATTCCAAACTAATAGATAACCC[A/G]ACACATCGTCTTGATACAGATAGGTCTTCTATTGTTCTTAAGAGTCATGTTGAGTATCCT
553	CaSNP2490	TATCTTATGTGTATATCTTATTTATATTCAACGAATTTTGTATCACATTATTCATTATAC[A/G]TTTGCATTGCAATTACCATATACATTTTTCCTTTAAAAACAAATTACACGATGATGTTTT
554	CaSNP2491	TGCAGCAGGAATGAGACACGTTTTTTTGTGAGGTCATGGCAAAGCTGCAAGTTGCTAAAA[T/C]TGAAGTGAGTGGTTAATACCAAAAAACAATAGAGTTGGAGCCTTGGAGGCAAAGTTAGTA
555	CaSNP2492	TTTCATAGCCTAACAATAACAATGTTTTTAGATGAGCACTCCTAATAACCATGATTACCA[T/G]CAAGGAAGCAACACAACAAGTCAACAATTATCAACATTCAAATAACAATTCTAAATTTCT
556	CaSNP2493	TGATAGGATCAAATTTACTATCCAATCCAGGCACATCAATTACCATGCTTTCCATTGGTG[T/C]ATGATTGACCTCTGATGTTGTTGCTTGAATGCATCTATCGTTTGAGCTGCAAAACATGTA
557	CaSNP2494	CAAATAGCCAAAGTCGCATAGTGGTTGAAATAAGTCAATTATATATAAAAAACTCATGCC[A/G]CACACAAATAAAAGAGGGAATGGAGGAAAATCACAGTTTACATATTGAAAGGATTACTTA
558	CaSNP2495	TACAGAGTTATTGCCCCCTACACCCCCTAGTTGCCCATCTAATAATCAGGTTGACCAAAC[T/C]GCCTACTTGATACTACATATGAGCCACACTCAACAAAGATTGATGAATGACATCAACAAT
559	CaSNP2496	AAGGTAACAAACAATTAAGGATATATTTACTTCTCAAAACATATAAAGATATTAAAACTC[A/G]TGGAACTGATTCATTATTAATGGTTAATCAAGTGATTGTTAGTTAATTAACTAGTTTTTA
560	CaSNP2497	ACAAATATATAATCTGAAAGTGACCCAATAAGAAAGGTTGCATTGCATATCGCTCAAATT[A/G]AGTGTGGATCCTTGCCACCTACAGATACCATCCATTAACTAGAGAGGAACCCAACCGGAA
561	CaSNP2498	CATAGTGTGGGATCTAGTTGCTTGTTTGTTATTACAAGCCTATATCGTGTCTGACATTAA[A/T]TATTTTTATCCACTTTAACTCCCAAAATCCTAAAATCACCAAAATTCTAAAATCATTCTG
562	CaSNP2499	GTCAAACTAAATATAGTTATATATCTCAAATAATTTTATATAAAATACATATCTTATAAA[T/C]CGGACCGCTGAGCTGAATTATGTTATATTTTAAATACCATATTATGTACTAAAATAAGAT
563	CaSNP2500	TCTTTCACTCACTGTATGTTCAACGTTCAACTCTCCATCTACAATTAAATTTTACTCTAT[A/G]TTTTAGGATATGTTTGGTTCCGTGGCTGCTAAAATTGATTTCAAATGAATTGATCTTGTT
564	CaSNP2501	TTTTCTTATAAAAATAGTTATCACTCTAGCATAGATATGTCGTCATACGAAGCTCTTTAT[T/G]GAAGAAATTGTAGGTCTCCCTTGTGTTGGTGTGAAGTTTGGGAGAAGTCTTTGTTATGAC
565	CaSNP2502	ATGAAGTCTCCTCTTAATAGACATATAGCATATATTTACTGGAATATATAATTATGAGTG[T/C]ATTTCTGATATAGTTCAAGGGTTTGACTCTTAAATTCTCACGAAAAAGATATGAGACACA
566	CaSNP2503	GCGAACAAACTCACAATGTCCAGATTAATATTATAAAACCACAAAATTCCTATAAATATA[T/C]TAAGATTAAAAATGCTTAAAATATCCATGCGTTCGGATCTGTAATATTGCCGATGCTAAA

567	CaSNP2504	CAAGAAATTGTAGGCCAACTACTTCTGAATTGAATTAAGAAACTGCACACAAGAATACTC[T/C]GATACGAATACTGGAAGATCAATCTCATCAAGATCTATAGCAGGGAATGCCGTATCTATA
568	CaSNP2505	ATCAGCATCACTGTAAGCAATCAATGTAGGAGGTGTCCCAGTAGAAAAGAACAAACCCCG[A/C]TGAGAGGCACCTTTCAAGTAGCGAATTATACGACGGACAACTGCTAAGTGAAGATGACGA
569	CaSNP2506	TAAAAATAATCATTTCAACAACTTGACTCCTTTTGCACATCAGGGTATTTGGAGTTTAGA[T/C]TACTTGAGTTTAGAACCTCAGGTCCTTGTTTACAAAACTCGAATACCGATTTAGTTTCAA
570	CaSNP2507	GTAGCCAAGTTTGTGGTGAACCAAAATAAATTATTTGTGTCACATTATTCCTGCACTCCT[T/C]AGTTTTTTTATTGCTTCTACTCCAAGTAAATCATCAAGTCTGAACCAGTTTAATCAAATA
571	CaSNP2508	CATCCCAAGGGGGAAATGGTCATTGTAACAGACAGCAAGTTAGACAATCAAAGGGTATCA[A/G]GAGGAAATAATGTTTGACCTGTATTGACATACGAGGGGAGAAGAGAGAGGAGTGCCAAAA
572	CaSNP2509	TCTGTTGTATTTTCAAGTGTAATCGAATTGCAACATTTACTTATCTAACTATTCAAAGGT[T/G]TTTTGTTCAGCGTCAAGCCCTCTGTAAAACTGATCTTGTTAGATGAAGCAGATGCCATGA
573	CaSNP2510	CTTGAGGACTAACAAATTAATGACTCTTCTATGAATACACATTGCCCTGCCATGGATTTC[C/G]TGTCATCCACACTTGTGGCCCAGTCAGCATCTAAGAAGCCTAGTATATCTAAATCAGTAG
574	CaSNP2511	ATCAGATTACCTCAAACAAGGACACAATACCTAGAAGTGGATCTTCTATCTGATGGTGAC[T/C]CTACCCAATCAGCATTAGAGTAGCCAACAACTTCGGTGTTACCCTTGTCTTCATAAAGTA
575	CaSNP2512	CTATCAACAATATCACTCATATTAAATTAATTATATGTTTATAGTCTAACCATATGTAAC[A/G]CTGCAAACTATTTATCAAGAAACAAGTTTGACTTAAAAGCTAATAACAATTCATCTCTTG
576	CaSNP2513	GTTTCCTCATTTACCTTAAAGGTTAAGGTGTCATCCGCCATATCAATCATAGTTCTCCCT[C/G]TTGCTAAGAATGGTCTCCCCAAGATCAAGGGGATATCATTGTCTTCTTCCATGTCTAGTA
577	CaSNP2514	GTGGCTAATTTAGATCTCGTGTTAAAATCTACTTTAGGATTAGTGGGGTCTTTATTAGTT[A/T]GAGTTGGCTATTCATGGTTATAATTATGCTTTTTAAGAGTGTCAAATCTATTCTTTTGAT
578	CaSNP2515	TTTTAGCTAAATTCAAAGTTGGTGGTTGATGTTATGCGGCATATGTGTAAGGATGAAATT[A/G]AATTTGGTGCTATCATTTGGGAATACAAAAGGTTACTTTTATGTACTGTTTCAACTCTCA
579	CaSNP2516	TTTGACAAATTTGGAGGCATCATTAATAACATCACTACTAACAGTCACTTAACCTTCATA[A/G]ATGACGAACTGCCCGCCGAAGAAAGAGGGCATAACAATGCGTTGCACATATCAGTAATGT
580	CaSNP2517	CTTTGATCTAGTAGTCCAATATGGGTTAACAGAATAAGATTTATCTATTCGTAGCATTCA[T/C]AGTGCAGTTAGGTTGGTTTGGTCTTCTCTGCAAAGAGCTTTGAAGTTCAAGGAATGCATT
581	CaSNP2518	AACTGCCCAACCCACCTTCTAAGAGTTATCTACATGTGAATTATTGCGACCTAGTTAGGA[A/G]AGTGTCATAGGTACTAGTAGTTGTCCGCAAGAACATTAGAAACTAGAACAGACTGTAATT
582	CaSNP2519	CTAGATTGTTGATCAATAATTAATATATATTTAGAGAAATTGGAGATGAGTTACTTTCTA[A/C]TTTGTACAATAATGTCATGATGACCGTTCATATATTTCCTAGTTGTTAATTGCGGCAACC
583	CaSNP2520	AAACTTGACATAACTATAAACTTGACATTTTATCTAACTCTAGTATGACTTGGTTGTCCA[T/C]TATTAGATATTCCTATAGAACAACTCCTTCGTTTACAACCAAATAATGGCACTCATCTGA
584	CaSNP2521	CATCTTATACCGATTTATGTACTCTTCTCCATATCCTCGCCGCTCCATAAGCTGCACATA[T/C]ATTGCTCTATTAAAAATTATCGATAGATAAATCATTATCAGCACTTAGTATCCCCAGGGT
585	CaSNP2522	CATATTAAATAAATATTTAGTTCTACTCCATTGCTCCCTCTCATGCATAGAACTGTGTTT[T/C]CAGTCACTCACAATCACTGATACAGAACAACCATTACAAAGTTAATACTTGCTAATGTGT
586	CaSNP2524	GTTGTAATCTCTGTCACCTTAGGAAATATAACATAAAAGAATATGCAGTCATAAACATG[A/G]TAAAAGAAGTGTTGCAACCTTTATCGCTCATTCCATGATCTGCTGTGAAAATATATGATG
587	CaSNP2525	GGTCTATATTTGTTTAAGTAATGCGTGCTTATAATGAACTTTTGATGTATCATTCTTCAG[T/G]GATCATAATGGAATGTTAGACCTCTTTTACTTTATGAAAACATTGTGTTTTTATTTTTTA
588	CaSNP2526	CTGATCAAATATCTGAAACTTGTGAAGATAGAGAGATCTCAATAAATTATGTCATGAATC[A/G]AATATGATGGAACCGAAAGGAAGTCAACAATGACAATGTTTGCATATAATATAGTGCTAT
589	CaSNP2527	ATTTGCACCCAAAATCTGAACATTTGTCAAGTCATCATCAGAATAACAAGACTTCTGCAG[T/C]GTGGCATTACTTAATGTTTTCAAAAATGATAATGGGGCTTCAAACACTCTAGCAACTTTC
590	CaSNP2528	GTCAGCATGAACAACTGAACTAACAGCCTCCAGTAGTTTTCATGTTCTCAAACACTGCTA[T/C]TGTGTAGTCAATTCAACTGTGTATTCCTCACCACAAGCACATTTTATCACATGACAGATT
591	CaSNP2529	ATCACACAAATTCAACACATTATACGGCAGTGTCACAATGAAGAAACAATTCTAGATCCC[A/G]GCATAATAAGAACCGATGTAAAGATTGATAATCAAAACAATGTATGGAGCTTAGGCAAAT
592	CaSNP2530	ATCCCTTGCAACGTTGGAAAACATCAATCTGTTAATATGAAGCTCTAACTTCGTGACCCT[T/C]AAAATAATTTCATGACTACTATATGTGTTTGTCTTTCATGTTTAGAATGTAAGTTGCCAT
593	CaSNP2531	ACAGAAAGTTTCAAAGTATACATAAACAGAAAGACTAGCCAATTTGTTGGTTAAATACGC[T/G]AATCAAAATTAACAGTGAAATTCACCTACAAGTTAACCTAATCCCTTTTTGACCTAAAGG
594	CaSNP2532	TAGCAAGATTTTCATGAGAGGTTATGGGAAAAACATCTAGAAGCCTAAGAAAATTGTGCT[C/G]TTCATCTACCTTCAGACATTGTTACCTAGATTTAAATCATATGCACTAGCAATGTAAAAA
595	CaSNP2533	GCCCCAACACTCCTACTATTCCTCACTACTCTCCCCGTATATATAGCCCTGCTTGGGACG[A/G]GGTTGAATGCCAAAGTAGTCGGCGAATCCTGATCAGAGAAGTCTAATGTCCTTTTCATTG
596	CaSNP2534	ATTTTGGAAGCTGTCATCTCAAGAATCTTCACAGGACCCTATGTATTATGTTCTATTGCG[A/T]CATCCACACAAGTATGTATACAGAAATACAACTGTAGAGTAAATTTCTCTCTGCGTTTGG

597	CaSNP2535	CACGGAAAAATGATTGCGTGTACTCAGCCACGGCGTGTTGCTGCTATGAGTGTTGCTGCC[T/C]GAGTTTCTCAAGAAATGGGGGTTAAACTAGGACACGAGGTTGGTTATTCCATACGTTTCG
598	CaSNP2536	TAAATACACTACTTGCTATTATTTTTGTTTTATGAATGATAGAGTTTGACTGTTCAATAT[C/G]ATTATATCTTTAAATTTGAAAGAATATTGTGAAATGATTGATATATACATATATGTAAAT
599	CaSNP2537	TTTCATGAAGCTAACTTTCTTTGTTTTCTATTTATTTTGCCCAAAGTCCTCTCTTAGTAA[T/G]CATTCCGAAAATACAAGCTTCATTTCTTGTTCACAGGTCAATAAAAAAGAAAGGAACATA
600	CaSNP2538	GAAACAAAATAGATACAAACAAGAGATTCATATCAATTGAGTTTAATTTTCATATCGAAT[T/C]GAGATATGACTCACACGTATCATAACTGATAACCATGATTCATTCATATGGTCAACAATT
601	CaSNP2539	TCATATCTTGGCATGGTTCATAGTGCACTTCATGATTTTAATGAGTTTCTCCCTCCTACT[A/G]CAAGTAATCCAATTGAACAAAAGCAGGAGTTGGATCGACACAGCACCTTCTTCATTCGCC
602	CaSNP2540	AGGAACATCAATACCATACATTGTAATATGTGCAGATGCATTAGTTGTGTCATACACATT[A/G]ACAACAATGGTTTGCTTGCTTGCAAGTTGGTGTAGAAGTTTGTCCACCAGTGATGGAACA
603	CaSNP2541	CTTCATTGCATCTGAGAATACCTTCGTCACTGCTCTTACAAAGCTTCTATTGTTCACATC[A/G]ATCTCTTTATTTGTTCATGGCATTCTTCCTGCAAAAATGTAAGTGCACTATAGTATCATT
604	CaSNP2542	ATAGTAAATAACACCTATTCTGTCTCACATAGATGGTGTGAACACAGAAAAATCATTTGG[A/G]TATTACTTACCATGGTTGTGGGGCTAAAATGATATCAACTATATTTGAAGGACTAAAGTG
605	CaSNP2543	ATTTGTCTTCTAAGTATTGAAGCAAGTAACTTGGTTTGTAGGGGCTAATGGAAGGACTGG[T/C]AGAGAATGGAGGAAGCTAGTTCCATATCTACGGTCTCGCCTTGGTAAAGAATGCAATGTG
606	CaSNP2544	AAGCTAATTAAATATGGATTGAAAACCTATAACACTATTCAATCCTCGAAAACAAAGCCA[A/C]AAACTTGTTGTGTGAAACATATGCTAATGTTAGTGCAGACTGTCTAAACAAGTAGTAAAA
607	CaSNP2545	AACCTCCACCCAAGGATAATATTTCACCAATCATTCAGGAACCTATCTTACATACTGATC[A/G]CACTCCAACTCCATCTTATAAATCGACTGATTCAACACTGCCTCAATCTCCACTACTCCA
608	CaSNP2546	AAATTTTCGTTCGTGACCCTTTACTTTGTTCTCGATTGGATTTAATGCTGCTTATTATTT[T/G]GAGTTATCGATTGGGATAATTGTGTGGGTATAACATATCAGAGCTTCGACTAGCAATCCA
609	CaSNP2547	TGTGCTTGTATATAACACGTTGATCCTGAAAAGGTCGCCTTAGAGAAAACTCATATATCT[A/G]ATGAAAACAAAAGCTTAACTGCACCCACTCTAACCTCACCATATTATATCAATTCTTCAT
610	CaSNP2548	CATAAATTTTGAAGTCTTCAAGTTGATGTTTCATCCATAGCAATCGAGTGCTGCAATTAG[T/C]TGCTGAGGTATACTCTGCTTCTGCAATAGATAGAGCAATATTGTTTTGTCTTTTGCTTGA
611	CaSNP2549	TTATTTTAGTAGAACTTAGCTTCGCAACCCAACGAAAAAGAACACCTAATTATAACACCC[A/G]CTATTTTTATTTAAATGATTAGGACTTTTGTTATTATTTAATTAATTATTTTTAATGGGA
612	CaSNP2550	CTTAAGGCTATGGGGCAGCTTTGGCTACCCAAGAAATATTATTTTCTAGAACAAGCAATT[A/G]TAGTTTTATGTTTAGTTACCTCAATTTTATATATTTAGCTACTCTAAATAAATTTATTAG
613	CaSNP2551	CACCTAATACAATAAACCGTAGAAATATTGTCTTGAGGTTTTCAAGGCAAAAACTATGGC[A/C]TCTAACTCAGAGTCATGTGTGGGACAATTCCTCTCACGTGTCTTCAATTGTCTTGAAGCA
614	CaSNP2552	TTTGGAGTAACAAACTAACTAACTAAAACAGTTACAAGTAGCTATAGCTAACTAACTAAC[T/C]GCTCAAAACAGTTTTCCTTCTTAATTTGAGTATCACTTTTAACGTACATTTAAGTTTTAA
615	CaSNP2554	TCAAGCAAACAATCGATTGGGTCCCTGAAATCATCGAACTGTGTCAGCCTAATCCCCAAC[A/G]TTAGCAATATTCCAAAATGATCCCTAAAATTGTAAAATGCTAATCAGATTCATCTATTGT
616	CaSNP2555	TTAGACCAATCTTATAACTTTCACTTGCTAATTGATAGTGATAATACCCTCCTTCCTGCC[A/C]AGCTTTCACTTATTTCAGGTTGGCTAATGTCTCAATAACTAAGCTTAAACAATTAAAAGG
617	CaSNP2556	TTATGTCGTAATTTCATTTTGGACATCTCCACACATGGTGAGCCTAAGTCCTTTACTCAG[A/G]CTTCCAAATTTCCTCATTGGGTGAAAGCTATGGAAGAAGAGCTTGCTTCAATACAACAAA
618	CaSNP2557	GTTGGAATACATGTATACACTAATTCATTAACGGAACTCCACAGCATCATTTATGGTTGA[A/G]AATCAAGATAATGAACCAAATATTTGAAGGAAGTGGAACATCTCAATATAAGTAGCAAGA
619	CaSNP2558	TAGGACCCAATTCATTTCTTATTCCCATATAGACCAAGTCAAATCTTGCATTGATGCCAT[T/C]GTTATACTTTCGTAGAGCATTGAGTAATATACCAATAATACTATCAAATACATTTTTTTC
620	CaSNP2559	AAGTCTGCTACCTGCCTGAAAGATTTTAATGTTTGAATTGATCAAATTATCATCTGATTC[A/C]TTGTCCTCAATACCAAGATACACATAATTCACAGAGAAATATATGGATACTCATTAAAGA
621	CaSNP2560	TCTTGACCGATCTCATGGGACAAGACAATGAAACGCGTGCATCCCCATTATGTTCTACTC[C/G]AATCATTACCAAGTACATGAATATTCAACACACACAAATTAAGGTTGGCATGAGACAATT
622	CaSNP2561	TATATTTTAATTTGTATCAAGATTTTTGGTATAATAAAATTACTCTAAACGTGGTTTTCT[T/C]CTACATCAGTCCATTAAATAAGTTAGATACTATGCTACACATGTGACAAGTATACATGAA
623	CaSNP2562	GAATTGAATTGATTGAATTACAAGCTAATTGACCAGTTTTCTGTACTTATTTTTTGTATG[A/C]TGAATTCTATCTTTGTAGGAATCGGCTAGAAACAAGAAGGGAAGCTTTGACCTCATGCTT
624	CaSNP2563	CCATATGGCACAGATATACATTGTATGTTTGTAACGCATTATTAAACAAGAAATGCATGT[C/G]ATACTAAAAAATGTACACAAGTATAGACAGACACTCCATGTAAATGCTGTGTCCATAAAT
625	CaSNP2564	GAATGATGAAAAAGGGTCTAACAATAACAGCCTGGAAAGGCTAAAGATGAATATTCAAAG[T/C]CTGAAGAGGCATAGTAAATAATACTTGTATGCTACTATTTAAATAGTGGATTAAGTCATC
626	CaSNP2565	GCACTGTAGTGTGGCGAAATTTAAACAAAATCACTATTTTCTGCAATTGACAACATTGAT[C/G]TGACATTTATCTGCATTCACGATGAGAGTTCACAAGTTCAATGGGTGTAGGCAAGGAAAC

627	CaSNP2566	ACAAATTGTGAGCGTATCTTCTTGTGAATAATTCTGAGTCTTTCTTATTTTGAATAATTC[T/G]GAGTGTATCTTCTTTTGAACAAATTCTAAGCAGATCTTCTTGTGAACAATATTTTGACCA
628	CaSNP2567	CATCGATTGAGCACAAGAGAAAAGCCAATTTGGTACCTAAATAGTATATACAACACCCAC[A/G]TTCAAGGGAATACACCGAAAATAACAAAACCCTATATCCTTATAACAATTGATGCTTTAG
629	CaSNP2568	TATTGCAAATCATGTCAAGGATGATAGCAATGAGAATGTAAGTTCGTGTGGTGATGTGGA[A/G]TATCAAGGTGGACAAGAGTCAGTCTCTTCAACCGACGACCTTCCTGGTGATGCCAGTATG
630	CaSNP2569	AGAAGGATTTAATCCACTAATTCAATAACTTGAATTACAAAGCAACTAACCCTCCAAGGT[A/G]GTCTTCTCAAACAACCCGACAACTCCAAACTTTTGAGGAACACACACACCTTAACTTTAC
631	CaSNP2570	TCCAAGACATCGTCATTTCACATGTCATTTGGTGAAATGATGATTACTTTGGATGATGTT[A/G]TCAATCTTCTAGGATTGTCGTGAGTTCTACAGTCCACCCGATGTATATAGAGTAATGACA
632	CaSNP2571	ATAGCAACACAAATTATGCATGAATACTAAAGATTTGCTATGTTGATGTTGCACACGTTA[A/G]TTGTAAAACTTCAAACTTATTACAGAGAACCGAGTAAAGCTTATTAAACCTTCATTCAAC
633	CaSNP2572	TCCCTGCTCGCTTGGGAATTGATATAGCGGGTAAAGTCCTGATAGCAAAAAATAAAAAAC[A/G]TGTTGACTAATTAATCATCCTTTTCTGTTAAGTGTTTATCATTATGTCTCTTTACTCATG
634	CaSNP2573	AAAATAAACACATAACTCAAACGAGGACGACGATGGAAAGAGAAGACTTTGGAATTGAGT[A/T]CCTCGAAGATAACGAAGAACATATGTCTGATGTATTGTATTGGGAGAGACATCAAATTGA
635	CaSNP2574	AGCTCCATTATATCGTACGTGAAGAAAGAATCATAATAAATCACAAGCTTATCATATTCA[T/C]CGGGGTTAAATGGAGATTTGGAGAAGTCATCAAATGTTATAAGTCGGTGAAGTTCTAAAG
636	CaSNP2575	GTTGATATTATATGCATATGTATAATCAATTGATTTATTCAATTTAGTAACTTCTCACTT[T/C]AGAATAGACAAATTCATTTGATAGTTTACACTTTCATCTGCACTCAATTCATTTTTAAAA
637	CaSNP2576	GTATATGATCATTATTTATATTCTCATCATTTACATTCTCATCATCTACATGTATATCTC[T/C]CCCATCAGTAAGAGTTTCTGTATGGGGCTTAGACGCAACATCAATGTATCTTCTGGAATT
638	CaSNP2577	AGACTCCTCAATGCTTCCCTTACGCTTAACCCTTCTCGTTTCATCTCTCTTACTCTGTTC[T/C]CTCTTCCAACCTTTCTGCCTCGGGATTCGCTCTTTCCCCACCACCACCACCACCACCACC
639	CaSNP2578	AGCTAAAAAGCAGGAGGAGACCGGCCATCAAATTATTGGTCTTTCACATCAGCCTATTAA[T/C]AAATAAATATCTTCAAAACATAAGCAAATTATTCAGATTGTACCTAATATAAAGTTAATC
640	CaSNP2579	GGGCAAGGTGAATAAGTGGAGAAGTGTGTAGGTAAAGGTGTCATGTGGTCAGTAGCTCTA[A/G]AATCCAGTATTCAAAAGAAAATTGAAACTTACCAAGAGGAAGTGTACTTGTATATACCAA
641	CaSNP2580	GTTAACTTTCTCACGAGTGATACGTTCGATGTGAATCACCCACTTGCGACGGTAGACCTG[T/G]GTAACCTTACCTTCACGGCCTTTGTAGGTCCCACGAACAACCTGAACTTCATCGTCCTTC
642	CaSNP2581	CACTTTCTCTTCTTTTGTGAAGCTTCAAAATCTGAAGGAGCTTCTCTTTTTGGACTCATG[C/G]TGAGTGCTTTGAGAAGTGAGACCATAGCTTTAATATTGTTCAACAAGAACTGGTTTCTCA
643	CaSNP2582	AGACTCTAGTTTCTTTTTACTTTCTTCTATTTAGGTGCAATGTTTTCCTCTTCTTTTAGT[A/G]TACTTAGTATTTATCTTTAAATGTAATTCTTTCAAGTACATTCCTCCGCAATTACCCACT
644	CaSNP2583	TTTGAGCAGGGAAGAAAGTGAAAGAGTTAACAAATGCACAACTGCTAAAGAAGTATGGGA[T/C]ACACTACAAACTCATCATAATGGAACACGCCATGCCAAAGAAACAAGAATAGACATTGGC
645	CaSNP2584	GGATACATTGAATTCTTCTTTAATAAATTGTATACAAAGTTTTACGGGTATTTGGATTCT[C/G]TAATGTTTTGAGAGAGATATGTTGATCATCTAAGCATTAAAGTCAATATAAGAGTGTTAT
646	CaSNP2585	CACCTTCTAGCCCTTTTCGGACTTCTGCTCCTGCTGGGGCTTCTGCTGCGACTCCTTGAA[T/C]GACTGCTCTCGTACTTCCTCACCTGCACAGATAGTATCCACAATTACATTTGTTTAATTC
647	CaSNP2586	TGCATCAAAACTTGTAATACTTTCTAAATATATAAACATCTACCAAACCATATATAATTA[T/C]GACTCTTCTTTTGGACAAACTCAAACAAGAAGTTTTACATAATCACAATTATTTAGACAT
648	CaSNP2588	AATCATTAACACCTACAATTTTCCCCCACACACTACTTTATTGGGGAACATCTTAACAAC[T/C]TAGGACAACTGTCACATTACACATTCTCCCTACTCTCTCTTTTTTTCCTTTTCTCATACC
649	CaSNP2589	GGGAGAAACTCATTAAAATCATGAAGTGCACTATGATCGGTGCCAATATATGAAGAAATA[A/G]AGCCATCGAGTATCTTCGGAGTAATGATATTCAACAAGCGGTGACAAACTCCATAGAGAC
650	CaSNP2590	TATTTTGTTTAAAAGTGGTACAAATTATGCCTTTGTTTCTTTGGATTCTTCTTCATTCTA[A/C]ATGCGGAGAGCACAATATTTTTCACAACGGCTTATCCCACATTCACTTTTTCTGCTTTTG
651	CaSNP2591	AAAGCAAAATAGAAAAAATGTCACCCCTTTGTAACTGAGCCCAGTAGTGGCTTTAGTTTG[T/C]CTCAAATAATCTATAGGCTTCAAGTTGTGTAGATAAGACTTGACTTGTAGGTCCAAGGTG
652	CaSNP2592	ATAGTTTGGTGTCGTAACAACAGACATCTGAGATGTAGTCATAAAGGACTACGAGTGCAA[T/C]AGTAGCCCAAGTGTATCTACATGTACTATCTAAATTTCTAAATAGAGAAAGGTACTTCGC
653	CaSNP2593	TATAGCTGGTGGATCCAATTGAGAACCGATGCAAAGATGAAGAAGCAAGAGTGCAAGCTA[T/C]GAGAGATTTGTTAGGCTGCATTGTCGAGCATCGCATGTCTGCGGATTCTCTTCCACCCCA
654	CaSNP2594	GAGGTAGTTAGGATGGAGAGGTTGACAGGTCATGGTTGGTGATAGATGAAATATATGTGT[T/G]TGATAATGTGTTATTGTTTGGTGAGGATGTATTGGAAGGAGGAATGATAAGGTCATTGAT
655	CaSNP2595	GCCATAAGGACCAACCAGTGTTTGCTGAGAGCATCATAAGAGAGCCTTTAATGTTATCCC[T/C]TTTAGAGTGAACCTTAGTGAATGAGTTAATAATATGATTCTCATTTTTTGGATACCACTT
656	CaSNP2596	ACCTACTATAGATATCAAATTATTTTGAATCTTTAACATTTCTCTTCTAATGGGCATGTT[T/C]TGTTGGATTTTTAATGAGTTCTATTTGATGAACTCTCGCAACTAGGAAATGATCATATAA

657	CaSNP2597	ATAAAGGAAAAATTAGAAAATATTCAACACTTCATTCATGGTTCAAATAGATTGGTTATT[A/G]CTGAAGAAGATAACACACAAGGCAGAATAAAAAAGAGATCAGCTATAAAGCAGTTGATAG
658	CaSNP2598	GGCTCCCCGACCCCCCTGCCTATCCCAATGCCTCCAGATGATAAAGATTGAAGATTTGTT[T/C]ATGACACAGGCAGAAAAGTACTGTGACTAGATATTTTATGCTTCATAAACTGAAGAGAGA
659	CaSNP2599	TTATGGTGTAATAATAAATCCTTGGGGTACCGCTTTGTCATAGTCTATAACCGGACCTTG[A/T]GAACAAAGGGCAGATGTTTATTCCCACATGCATGAGACGACATTTAGCAGAATTAGCTTT
660	CaSNP2600	ACAGATTAAGCATTTCAAAGATGAAGAGGGAAAAAAAGATTCCCCCTCTTCTATGTCTTA[T/C]TTTTGTACCTCTTTTAATGATATTTACATGTACCTCCTTCTCAACTCAATTCTTTGTCTG
661	CaSNP2601	GAATAGCTGCTGCAAAGTGGTGAGAAGTTGGTTTGTTGGTGTATTGACCTAATTGTTGAA[T/C]AGCGAAGCTGATATCTGGACGAGTGTTGGTGAGGAGATAAGTTTGCCAATTAGTCTTCTA
662	CaSNP2602	TGAGATGTCACAAGAGGAGATGCAACATGAGATGTACTAGGAATATGTGAAACATGAGAT[T/C]ACAAGAGGAGGTGCAACATGAGATGTCATAGGTGATGAAGTAGTAGTATAACATAATGTA
663	CaSNP2603	TCGTAGCACTTTAGTTGGAAGAGTAGTTAAAAACATAGAACATTAGTCGACTTTGGTTGC[T/C]GAGTGTTTGTGAATTCCCTAAATGTATCGGTCTAGGAAAGATAGGGTTGAAGCAAATCAT
664	CaSNP2604	CAAACGGAGCAACAAACGAGACTCTCGGTGTTAATTATCGAGCTCTAAATGACCTCTTCA[A/G]AATTTCCACAAGTAGAGCAAGCTTCATAGACTATGAGATTTGGGTTCAAATGGTTGAGAT
665	CaSNP2605	TGTGATCTTAATGAATTAGATTAGATATAGACATGCAAAGGACAATAAATGTATGCAAGT[T/C]TATATAGTAATTGTCATGCAAAAGGTTACGATTACAATTTGACGATTTCACACTAATTAA
666	CaSNP2606	TAAGCTTACATAACAACAACGCAAAAATGGAGTTTGTGTAGAACATCATTGAATAGTTAT[A/T]CAATCCAGTTAGCTATAACGATGTCAAGGAAGTCAAGGTCTAGGAGCCATGCATTCTCAA
667	CaSNP2607	AATTGAGCCATAATCTGACCTTGACAGTCGAAGGAGACAATTGATAGAAATGTCTCGACC[A/G]AGTGGGTGAATTAGAGAACTTAGGTCAGGGTCAACACAAGTTTTTTTCTTATCATTCCCT
668	CaSNP2608	GCTACCATCAAACACTAAGTGTAGACATTTTTTTCGGTCGGGTTAGAGCCAACCATCAAT[A/G]GTTAAACAACTTGCATTTATTGGACTTCGATTCCTTACAATAGTAGGTTCACAAAGTTTG
669	CaSNP2609	TTATAATTTGAGGTCCGATAACTTGTGGAAATTATCTTCCGGAAAAACTGAAAGTTTATA[A/G]GTTCAGAAAAAACAGTAGTAATTGTTGTAGTTAACCCATTAAGCAAAAAAGGATTAACAA
670	CaSNP2610	CTCCAACATGCTAACATCATTCTCCAACATGTTAAACATCAATCTCCAGAATGATAACAT[T/C]AATATGCAGAAAGTTCTCATCAGTCTCCATAATGATCAGATTTGTCTCCAAAATGTTCAG
671	CaSNP2611	TGGCTAGTTTGAACCTCTGCTAGTAACAAATGTTGAATCTCTTCTCATAACTCGAGGTTC[A/G]TGTCCAAAAGCATCATCACACCACCACATCATCACCCCTCTCAAACTAATACCAATTACT
672	CaSNP2612	TATTGCACACACACACACTAAATATAATATCAAGCCTGAAGGCAGTAAGGTAAATCAG[T/C]GATCCAATCATTCCTCTATATGTTTTCTGGTCAACCTTCTGACCTGATTCATCATTTTCA
673	CaSNP2614	TAATCTTAGGACTACCATTGAATTCTAGAATAGGTAGGAAACAATTGGCTGTATGTCATG[A/G]AAATCGGGTCAAAATGGGTTATCTTCTTTTCTCGAATGACTGGTTGTTTGTATTGATTCG
674	CaSNP2615	ATTTCACTTTTTGAGAGCATAGCTTTGTAAACACATTCTTATGAGATTTATTCACAAAAA[T/C]CCATAAACACGTTATGTTTGTAACCTAGCCTAGTAGTGGCTACATCCCTTTACATCTTGA
675	CaSNP2616	TTTAGGCACAATCTGTCTCATTAAAATATCATTAAAATATGCTTGGAAATTATGAAGCAA[T/C]TGCTTATACATACAACTTGGTGTTGCTTGTTTCTAGAGCTAAGGTAATAACTGGATTGGG
676	CaSNP2617	AAACATTGTTTGGAATCTGTTACGTGTTTCTCAGTGTTAAGTAGAAGTGTTACCCTCAAT[A/T]GTTGTTAGCATCATGCAATAAATCTTCATAGGAGTTTGATATTCACCAAGTAGAGATTGT
677	CaSNP2619	TGACAAAATTCATAAGGAAAAATGGGATAGAGATGAAAAAGCTTTATTAAACAAAGATAG[A/G]CTACTTATATAAACACCATCAAATGAAATGCGTAGTTATTGCTTTCACAAGTATTTGTTA
678	CaSNP2620	TACTATATTTCTTCGTGCTAAATCATCTGCTCAATCTTGTACACCTTAGCAATTGGTGTC[T/C]CCTTTCAAGAATTTTCTTTGCCTAATGGAAGTTTCTTGGTCTTTTCCTGAACATTTAATG
679	CaSNP2621	TAAATCGTGCATCATAGTTTCAACACTTTCATGCAATAACCCCCTAAGGACTGCAGAAAA[T/C]TATGCAGTTACTAATTACCATGCATTAGACAATCACCCTGTAGGCCTGTACAGGGAAGTC
680	CaSNP2622	ACGCTTGTTCAATATATGGTGAGAAATTCAACATTTTGATTTGGTGGAAGCAAAACAGTG[T/C]TCGTTACCTTATTTTGGCAACAATGGTAAGAGAAATTTTGGCCACACCAGTGTCAACTGT
681	CaSNP2623	TTCTCACACAAGAATCATTAATTGTGCTTTCTATTCTCATCGATGATCATCATAATGGTG[A/G]GTTATGTTGATTATGACTCAAACTAATCCAATAATTGTGAAATTAGTTACAATTAACGTA
682	CaSNP2624	CTTCGCTGCTCCCGTTGTACTTCTTATTGCTCCTCAAGTCGTTGAGTCCATGTAGAAACA[A/G]TCAGTCTAACACATCGACCCTCATTCAATTAACATTAGTTATAACATGACTCGTTTCGTC
683	CaSNP2625	GCTAATTGTTAATCGAGTTTGGGAAGGTGGATATTATGGGTTTTTGGAGATAATTATTTT[A/G]ATGGGAGAAGAGCTTAGTGAGCAATATGCTTAGTACTCTCAGCTGTGAAACTATAGTAGA
684	CaSNP2626	CTGAACAGCAGGTATAATCGATTATCTCACTCTGTCCACCCTTTTTTTATGCAATAATCA[T/C]GTGACTCTCTGATGGCGAATCTGTTACAGTTTTGTTGATGTTATTGTAATCTCTAATTTA
685	CaSNP2627	TTATAAAGAGAATAAATTGTTGATTGAATTATATGTATTCACATGCATGCAGGTTGTGTC[T/C]GATGAAGCAAGAGCAATGTATAATGGTGGAGCGGCAGGATTGACATATTGGAGTCCTAAT
686	CaSNP2628	AAAACTCTGAGTGTGAAAGCCAAAAAGCCTCAGGTTGACAGTTTAAGAATACTTTGTATC[T/C]TGTTGGGGCTTAATCATCTGTTAGCCTTTACTAAGAAATATGGAAAGATTCTTGATTTAT

687	CaSNP2629	TTCCAACGAACTTATTTCTGATAGCAATAAGTTAACATACTCACCCCAGTAGGGAATTGT[A/C]AACTAATAAATTACAATTACAAATGAAATTCACTCAAAGCCACATGCCCAAGTGATAAAC
688	CaSNP2630	GTGTGCTAAGTGATAACCTCTTGAATACAGGTTTGGCCTGCTATAATCTCCACTGGTTCT[A/G]GTAATTATCCTTTGAATGCAAGTTACAAAACAGCAGATGGATATGCATTCCAGGTATGCA
689	CaSNP2631	GTATGACTATTAGGGTGTCCAAGGCATAGATGCCAAAGATATTGAGAACTAGCAGATAAA[A/G]CACTACTAGAAACAGTATAATCATTGGATACAGAGATAGATTGGGAAACTCATAGGCCAT
690	CaSNP2632	AAAGAGTCAATAAGAAAGTAAACTCAATGCTGCAGAGATAAGAGTATTGCCATTTATGAA[A/T]GATTATACGAGACAAGATAGGATTAGGAATACAATCATTAGATAGAACTTGGGGTAGTTG
691	CaSNP2633	AAGCCGTGAACATCAAATTTGTATCTATGCTTATATGAACTTACTTTGTCTCTTTCCAAC[A/T]TTAATAGTTTAAGCTCCTCTTGGAAAGACTTGTTCAATTTCTGTTCCTCTGGCAGTGAAG
692	CaSNP2634	ACAAGCAGTGTTATTAAACTCTGACCAGTTGTCGACTCGGTTACGGTACTAGGTTAATGG[T/C]CAAACTAGTGGGTCGCTAGTTGAACCATACAAACTGGTCTATATTTCATTTAAACAGTTT
693	CaSNP2635	ACTGAGCACCATGTAATTCATCCAACAACTCCTCTACTACCGGAATAGGGTACTTGTCCG[A/G]AATTGTGGCCTTGTTCAGCTCCCTATAGTGCAGACACATCCTCCATGTGTCATCTTTCTT
694	CaSNP2636	TGATATTTTAACCTTGATGTCTAATCAAGGACGATGTGTAATGCGGAAATGAAGATTTAT[T/C]GGTCTCACAATGTCTTAAAGCAAAATATTTTCCTATGAGTAATTTATTGAGAGTTTCAAT
695	CaSNP2637	TTTCTAATTGCTATGTAATATATTTTAATTCATCGTAAAAAAATCATCCTCGATTACATA[A/C]GAAGAGAAAATGTTAGATATTTAGACAAACTTACCAACTAAAATTCTAGATTTGAAAATA
696	CaSNP2638	CGTTCATGGAAAAATATATGGCACATAACTCAATAGTATTAGGTAAAAAAATAAATAATA[A/C]ACACAATTCACAAGCAACCACAATTGACACAACTGACATCTTACTAACACTAAACCGACA
697	CaSNP2639	GACAAACGCATTCTTAACGCACCTCTGCTTAGTCTGTTAATACAAACTTGCTCTGATGAT[A/T]TTACGTCTGATAACTTTGGTATATAAATTGATCATTTGATAAAGTCAACAATCTAATAAA
698	CaSNP2640	CATAAGCTAAATGCAATATATTAGTATTAGTGGTATATGCACGCCTTTCTACATATACAC[A/T]TCTTTATTTTTTTCCGCACTTAATGATGATAATCAATATGGGGCTAAAGATCGTAAATTT
699	CaSNP2641	CATCAAGATAAGGTTGATCTTTCCTAAGATTTGCATTCAACCCAATGGTAATGCTAGAAG[T/G]ATATATGGTCAAACCAATGGCTCTCAAGACTTTCCCTTTTCGGTTGAAGACTTATATGCT
700	CaSNP2642	ACACCAGAAGAAATGACAGAAATTGAGTCCTTAGCAGATTCTGTTAAGGGTGATAGATAT[A/G]TGCAGGGTGTAAGAACATGGAAGGAATCTGATACTCCACCACTCTCTTCTTGGAAAGCTG
701	CaSNP2643	GTAAGAAAAAAACAATATTAGACATGATATGCAGATTCCAGATGGACCGTGGCCTATGTT[A/G]CTATTAAGCAATAAATTGGGAGAAAAATGAGTACGTAACCGATGAATATGATGATGATCA
702	CaSNP2644	TTTTTATTGTATTTGCCATGATCAGTTATTTTTCTAGTATTACCATGTTAGTTCAGCTTA[T/C]CTGCTTTGTAGTGAAATTCCATTTCAGTTGGAAATATGATACCCCATGTTTAGATTAGGT
703	CaSNP2645	GAGTATATTTTATCGATTGTCTCTCCTACATTCATTTAAAATAATTCGAACTTTCGTATG[A/C]TGATGTCTATTCTTGTCTCCTTGACATGGCTTGTACATTCATGATGAGTTTGCAATGTGT
704	CaSNP2646	TCAATAACAATCAAGGAAGCTCAATTCGTCTCTATTGCAATTATCTACAAAGCAAAGTTT[T/C]CTCTAGAACAACAAAAGGTCATCCAAGGAGAGTCTCTTATGGACCAATAATAACATGTTC
705	CaSNP2647	TTTTTTCATATATAATAGAAACTAAAAGTAGTTAACATTCCATTATAAAAGTAAATTGGC[A/T]ATCATGTCACTCTTATATTAGATTTGGATGTTGATAAATTGAAGCAATTAATATTAATAT
706	CaSNP2648	AGGGTGAGGGTAGGCCCTTCCTTTTCCAAAATTCCGAAGGTTGGGGCTAACAATTAACCT[T/C]GGCATTCAGCAATGATATATAGTGGAACTGCATATATTCTACCAATAAGGAACTCATGGA
707	CaSNP2649	TGTAACCCAGTTTGATAGTTGTTTAGTATTCCTCAAGCAGTATGTGGTTTTCAAGTTGTA[T/C]TGAGAACACTTTACTTGTATGGCAAGGTATAGTTGTCTTCAACAATCTATGGTTTTTAGG
708	CaSNP2650	TGCTGAAATTATAGAGTACCTTAGAGCAACTTATACTCTTTAGTGATGCCTTTGCATGTC[A/G]CAAACACTATCATCCTCTTGTGAGATTTAAATTCGTTCTTCTTACACATGTAAATCAACT
709	CaSNP2651	ATTTGAAGTATCGGATGAAGAACATGACATAATTCTCTTTCCTAAAAGTACATTTGTATT[A/G]GGTCGCTCTGGTACGGGAAAAACCACTGTCTTGACCATGAAGTTATTTCAAAAGGAGAAA
710	CaSNP2652	AACTTTCAATTTAGTCATAATCCAAATAGGATAATCTAATATTTAATACATCTCCCAATA[T/G]ACTTTCTAGACTCAAAGTAGCTGCAAATTACATATAAAAAACCAATTAATAGCGAAGCGA
711	CaSNP2653	CGGGGATAGTAACGTTATATTCTCTTCGGATGTAGTGCTTCAAGCAGCATGACAAGGATG[A/G]TTAACGATGATTTTGCTAGAACGATACATAGAATGTACATGATCATGGTATAAAGTTTGC
712	CaSNP2655	ATAAAAAATTCCACTTGATGCGATCATATGCCTTTGACATATCCAACTTTAAGTCATCAC[A/C]CTAAAATCTCAAAGATTGTGATTTGGAGAGTTTCCCGAAACATCAAAATTAGACGAGTGT
713	CaSNP2656	GAATGAGGCTTTGATCAAGTTTATAAATAAATTATTAGAATGAATAGAAAGAATTCAACT[A/T]TGATTTGTTAGTCAATACCAAAGTAGTTTTAGTTTAAAGCTTCTCTGTGGTGTTCTGCTC
714	CaSNP2657	TGACTGCTTCACATACACCTTTTCACCTAGTGACCCATTGAGGAAGGCTGATTTAACATC[T/C]AGTTGATGCATAGACCAGACTTTGTAGCTAGCCATTGAGATTATCATCCTTACTGTTTCA
715	CaSNP2658	GTCCAATTGCACTACGCTACAGCGTTGTAGGCTATATCTGCTATTTGACACCATTGATGT[A/G]TAATAGACCAAAATAATAAATATAAAGGATACTCCCCGGACAATTGCTCCAGCTCTTGAT
716	CaSNP2659	TTCTTTCTCACGAACTCTTCGAATGGAACCCTGATATTATCTGTTTACAGGTAACAACCA[A/G]CAACGTTTCACTTTCACCATTCCAATTAATACTAACTCTTTATATATAAATTCCAATTAA

717	CaSNP2660	TGGATTTCAAATAAACCTGCTTGTGACACTTACAACATGAGAAATGTCTGGCCTTGAGCA[T/G]ACCATAGCATACATGATGCTCCCTATGACACTTGCATATGGGATAGTCTTCATGTTTTTT
718	CaSNP2661	AACCGCAGCATACAAATACAAGATTTGTCATCCAGTTTCAATTGAGTTGCATACCTGATA[A/G]ATAGTATACAATGATGTACGCCGTATGACAGATTCCTTTAAAACATTTAGCAACATATGT
719	CaSNP2662	AAGACAAGTAACTAATTAAGTACACGTTGCCTCCTACTGGTTTTAGAGTTTGTATATGTA[A/T]TGAGTATCAATATTGACCAAGACTTGGTCTTGTGTAGTCCAACTTTGAAAAATTACTATA
720	CaSNP2663	ATATCGTGTATTATGCTAAACACATGGAAAGACAAGAAATCTTTAAGAACTAACAAACCA[A/T]GACCTAACCAGATTTGGGTACCAAAGGATAAAATTGTTTATGTTGTAGATGTTCTAAGGA
721	CaSNP2664	TTTCAAACTTAAGGTTTACAATATACAATTTGTATTTAAATACTACGTCCCATATTACAC[T/G]TATTGGTAACTATGAGTCATACCCAACATTATAATTTATTTATGCACGTGTAGTTATTTA
722	CaSNP2665	ATATTATGAGTTATTTTATCGATGTGCAAACACTCGCCCTCTAACTAAAACTTACAATAA[T/C]GATGTACAGTATGTAACAATGTGTTACAATAGACATAATACTATTTTTGTACTGATACAA
723	CaSNP2667	TCTAGATGATTCTGCAGGTATTACTTAGGATCTTTCTAGTAATAATTTCTTTGATAGACC[A/G]ATTTGGATTGTGGATTCGGGTGCCACTAATGATATTTGCGACTCTATGTACTTGTTGATC
724	CaSNP2668	TCTACATAGATATATCATTGTAACTCTATACTAAGTAACCTATATTTTATCTTAGGTACA[C/G]AGTGTTTGGTTCAAATGTGAGTTGTTAGTCATACATTGACTATGAACAAATTAAATCTTA
725	CaSNP2669	TCCTACTTGAATTATATATGATATAAATGTACTATGATGTTGTGTATTGATTATGTTTGT[C/G]TGTTATCTTCAAAATGAATGTTATATGATATATTGTTATAATTGTTGTGTTTGACATGTT
726	CaSNP2670	TCTCAACAACCTCATAATTTGGGATGAAATTGAGCTCACTTATGAAGAACTAGCTTAACG[A/G]CTGAAACAATTGAAAGCAGAGCACTCAAATAGTTTATTGAGATTATTCTACCTAAAAATA
727	CaSNP2671	AAGGCAGTTTAGATGGAAGACTGCTGAACAAATAGCGCCAGGAGGTGATGATACATGATA[C/G]ATAAAAAAGTCATAATGTGAATAATCAACAATAGGTCGACGAGGAATACTAACTCTAAGA
728	CaSNP2672	TTGCCTCTGAAGCTTATGTTCCACTCGCCCCGAAGACTTCAACTTATTCCAAGATGATGC[T/C]TCTAATACGCAACATGCTCTAAAGATGTACCAAGGTGACGTTGCCCATGGCACTAATTCT
729	CaSNP2673	ATTAATTGATCATGATATGATAAACCACCATATGTTTGAACTTCATGAGTGGAGTTTGTA[T/C]ACATATATACTCACCAAATTTTGTAAATAATTAGTCATACATCTACATTACGTGTATGAT
730	CaSNP2674	TAACAAAAGATGAAATAAAGTCAAATTGCTTTCATATAGGCTATAAGTTGTTTTCATAAA[T/C]TGTTTCCATAAGCTCTCTCAAACAGTTTTGCACGTATTTATGCCGGTAGATAAGTTTAAA
731	CaSNP2675	TCACTATGAAGAGGCTTACTGGTACCAACAAGCCCACAACAAGTGGATCACTCTTGGTAA[T/C]CGGAATTGTAAATGGTTTGATATGTCAACTTTGAAGCGCGAAAGACGCAATAGGATTGTC
732	CaSNP2676	ATCACTGCCAACTTAAAAAGCTGATTTAGCCACTTAGGGTACATAACACAAGTAAATGAA[A/G]AGTAACTAGTTGTATGGAAATTACCTGCCGCCCTATATCTGTACGGATATGTCCTTAAAG
733	CaSNP2677	GCTATACTGAAAACATAGCCACTAGTTGCTTTGGAGTCATCTGATAGGGTGTTACAATCA[A/G]CATCACTGTACCTGATAGCGCTTCAACAAGTGTACTGAATCGTAACAACTAATAATTAAA
734	CaSNP2678	GCATAATACTAGTAAGAAGAACACGCAAACAATCATAATATAACTGTATCATTACACAAA[T/C]ATTGCTCAAGAAAAAATATGACGTTAATAAGTCAAAATCACTCAAGGAAGATCAATACCTT
735	CaSNP2679	CACATCAAGTACTCCATTTGGATCCACAGTGATGTCTTTAGACAATGAGTTGAACAATTT[A/T]GCAACAACTTTGTCACTCCCAAGAGCATTGAATATGATCTCGCTTCGGTTGAGAAGTGCA
736	CaSNP2680	GATGTCTCAACTCTTTTCTGAGATTCGATTCTTTACAAAGAAATCAGAAGTAATCACACA[A/G]TATGATGTAAGGATTTAGAACTGCTTCTTCTTTGTAGAAATTGAGAACTTCAAGTATGTG
737	CaSNP2681	TAAGAAGGTGAATGTGAAGGGTTATTGAATTTTGTTGTTATGATGAAAGATTATTAAGAT[A/G]TGATGACATTGAAACTTGAGAGAAAACAAGGTGCGAGAGAATTGGGGCGGGATAGGAAGA
738	CaSNP2682	TGCAAGTGCTGTGTATGGAATTGCTGCTCCAAAACCCATCATTACATCAAATGAGTTCAC[T/C]ACCATTTGCATTTGTTCACAGTAGTTATTGTACCTTCTGTCCACCTACATATATAATAAT
739	CaSNP2683	ATGTATGCCCATTCTCAACATCTTTGATGGATAAGGATTGTTGTAAAATAAATAGATTAG[C/G]ACTACAGTATGATTTCAAACAATAACCCATTTTATCATATACGAAACTCTGCTTCATGCC
740	CaSNP2684	ACCCCCCATTAAGAGACCCAAAGGAACAAATATGCATCAATAGGCTTTTTCAGGATGTTG[T/C]GGTTCTAGATCGGTGAAAAAGGAATAAACCAATGTTTGTATCTTTTAGAGGCAGATGGAT
741	CaSNP2685	CAGAAACTATTTCTAGCAAAGGAACCCCTGCCCTGTTTAGATCAACCTGTGAAACAATAC[T/C]ATCAAATTTAACATATTTGTGTCATAGTAAAGGTTATCAAGCCTTTGGCGATAATTATTC
742	CaSNP2687	GCGACAGTGCAATGCCCGCAAGTACGACCTATCTGCTTCGATTGATGTGGTAGCCATATC[C/G]CCGATTATGAAGGTTCCCCCCACGAGACAACAATGATGTCAAGGAAGTCTTTGCCAACAA
743	CaSNP2688	ATATGTCGAAGACATGTGTGGAAGTTTATGATGGGTATAAGAAAGAATGTTTCAATTTGA[T/G]GGCTATGTTGTACGACACAATTAATGATTTTCCAGTATATGGTAATTTATCAAGATATAT
744	CaSNP2689	ATCCAGTGTTGAGCGCTCGACCTTCTGATGCCGGCAGGCATCTCAATATCGAAGAAGATG[T/C]TTTCTCAAGACATGCTTTGCAAGACCTAGAGTCTAAATTCCTCCAACAATCAGCCAGAAC
745	CaSNP2690	AAACTTGAAATTTTTTTGAAAACTAAAGATATAGTATGAGTCTTGTCGAATTGCGGCCAT[A/G]ACGGATATTGGTGGCGGAAATTGAGCATACCGCCATACAAAGTTCATGGCATGGCGGGTT
746	CaSNP2691	TGAATGAATATAGTGTTAATTGAACACGAATGAAGAAAAATAAATCACAAGAATTTCAAT[A/T]CAATTCTAAAATAACAACAGTAAAATCAAATGTAAATTAAAATTAAAAACCAGTATTTGA

747	CaSNP2692	CCATCATTAAAACACGAACCTCATTGGCAATTGCTGCTGATACATGCTTGTGAAATTTTC[A/G]TTGGCTATGGAGAACCCAAGCATCCAACTACTTATCATGCGCAAGACCTATGATGGACTA
748	CaSNP2693	TTTTGATGTCAAAGTCAAACCCAGAAAATTACAACCATATGTGAACAACATATTGAGCCA[T/C]CCATCTGTATACATGAAGTGCACAACTAAAACTGTTTTAATTACTATGAAGCACATTTA
749	CaSNP2694	TGCAACACATAACGTGCATGACATAACATTTCAACATCAACAAATCATTGTCTGGCAACA[A/C]TCAATTCACATTTACGGCTAAGCGTGCTAAGACATATTAACGTTCATCATCAAAAGATCA
750	CaSNP2695	CTTTTCTCTAAAATCCCACAACTCCAACATTAAACTTTCCAATCAGCTTTTTGTGCAGTA[T/C]TTTCTACTAGTGCAGTTTCAAGGACAGGCTCAAATGGTTTGGTGGAATTTTTTGGTTCTA
751	CaSNP2696	GTAGGAAAACGGGGGGAATAGGATGGTGATGCTTCTAACATGGCCAAAACCACTTTCACA[A/T]TTGGATCTTCACTTTATTTAATGACGTCTTGGGATAGGCATAACACTCTTTCTAATGGCA
752	CaSNP2697	TATTATTCAGTGCCATTTATCTCTCAAATTTAAATTGAACAAACAGTAAACCATGATCAA[A/G]TAATACCTTTGCTAGCTTGTCTCCACCATTCATCGATTTGCTTTCCTTTTTGAAGCTGTT
753	CaSNP2698	GTTTTTGTAACTACCATCTTTAACTAGCTAGTATCTTAATCATGGTAAAATTTAGTTTCT[T/G]AATGTTGTATTTTCTTTGGTATAGATGGTTATTGATTTCACAGCTAAATGGTGTCGACCT
754	CaSNP2699	GAGGTTATGTACGGAACACGGTGTAGGTCTTTGATAGGATGGATGCATACAATATAGTCA[T/G]TCTAGAGTTGATTTAAGATGCCATTGAAAATATTAAATTGATTCGAGATCACTTAGTGAC
755	CaSNP2700	GGATGTAGATAGGATTTGTTCCTCCTACATAACAAGAGTTATGTCTTGGGCCTATCGATA[A/G]AGTTAGACCGTGGAAATGCATGGTCATGCTAGAATGACTCAATATGTGATATTGGGTCTA
756	CaSNP2701	ATCTCTGATCCCACTAGTTGACCCGTTAAATTATTTGCCATCGCATAAACCTCTGCAGAT[T/C]ATCAACACCTTATTAACAACAAACACGCACAGAGCAAAGGGATGAGTTGACAGAAGAGAA
757	CaSNP2702	AAGGTTCGATATGGAGCTTCGAGTCGTCGCTTGTGTCCACAATGCTAAACAATCCTATGG[C/G]ATGATCAAAGTCCTTGAAGCCATAAATGCTAGCAGAGTTTCACCTGTACATGTCTCTGCA
758	CaSNP2703	CAATATTCAGCATAGGCAATATGCATTCATTTTAAATCAACAAAAGGTATGTATGCTGAA[T/G]TTTTTGAATCCTTTATAACATTATGCAATTTGAACTGATTTGTCCTCTTATAATATTCTG
759	CaSNP2704	CTATTTAATATTATAAACAACGAGCAATGTCTAACAACACGTCACTGCTATCCAAATGAC[A/G]ATAATATCTTGTGATTTGACATAACCTTTTTTCCTTTATATCTATATTGAACACAAGGCT
760	CaSNP2705	ACTTTCTTCTGCTTGGATTTCAAAGCCAAGGTTAGTTGTTTCTTCTTGGGCTCATTCTCT[A/G]CAAGTTCAATTTCATGTGATTTGAGAGAGTTGATTTATTAGTCCATCTTAAGAGTATTCA
761	CaSNP2706	GATACAATGGTGAGTGCAATGGAAAATCAAGTGAACGCGTTGGAAAGGATGGCCTGCAAA[A/G]GGACAGGTGTTAGCAGATTTGAAGGCTAACGTCGACTGTGTTACTACAGTATTACCATAC
762	CaSNP2707	TGTTCGTGGTACTTATCCCGTAAATACCAGGTTCAACTCCCACATACAATAAAAAACTCA[T/C]TAATTTGTAATAAGGATAAGAGGGGCGATAAGATCGTAAATAACAGTGTCGGTCGTCGGG
763	CaSNP2708	GATCTAATAGAGCATCTCCGTTCAATACTAACTACCCACACATTTGTATCCAAAGGACAA[T/C]TGTTCTACTTAAAATTCGCTTTAAGAGACTCCAACTTCTTTCTGAACTAAAAGGGTATCA
764	CaSNP2709	ACAATTTTACTTCAAGATGATGTAAGTGGCCTTCAAGTATTCAAGGACGGACAATGGATT[T/G]CTGTTCAGGCTCTTCCTCATGCTTTTGTCATCAACGTAGGTTACCAGTTGCAGGTACACT
765	CaSNP2710	TGCTCAGCAACAAATTCTACACCATACTGAACAAGAAAATCAAACAAGACTTATGTAAAA[A/C]TCCAACTATTGCTGATCACAGCAGTCAATAATACAAAACCCGCAGTTCATAATTCAGGCC
766	CaSNP2711	TCAGTAATATATTTAGTTTACATCCATTACCATGAAAGTAGTAGCTGATTTCTGATATCC[A/G]ACTATATATGTTGACCCTATGGTCATGGAGAAAATTGGAGAAGGACTTAGGTTCAACTTA
767	CaSNP2712	TCAGAAGGAAATGAATGTAGCTAACATCTAGCTCTATCCCTTAATGAAAAAGGAAAAAGT[C/G]TTAGTCTAATGGCGTCACTAGTCACTCCATTCATTTTCAAATTATCACAATACTCTAAAA
768	CaSNP2713	TCTTCATCAATAGACTAACCGTCATAATTGAAGTTAGAGTATGTATCTGCCAATTAGAAT[T/C]GTGTTTGCATGCACTTTGTCTTCATCAGATGATTCATCAAAGTCCTCCCAATTAGTCATA
769	CaSNP2714	CCAAACAGGCCTGTGGTTCTTATGATCTAATCCTACTAGTCCAATTCTGCGCTTTCTTTT[A/T]GACTTATATTTTGCATCGTGGATTTGCCATCTGGGTTTTTGTATTTGGGTCTGTCACTAG
770	CaSNP2715	CCTCAGTGATAAACATTTACCAAGTCGAGTAGCAAGTCGAGTAGCAAGATCTTAAAGTCT[T/C]TTATAAAAAAGATGAACATTTACCAAGTCGAGTAGCAAACTACAAGGATACAAAAGAATG
771	CaSNP2716	CATTTGTTTTCACTTTAAAAATACAATTTGAGTTCATTGTAAGCTATAAGTTCTTGCAAA[T/C]ACATATATAAATATTTATGTCATGAGATAAGTTCGTATAAGTATTTAATGCCTTAGTTTT
772	CaSNP2717	TCACTTGCTTGTTAAAATTATCAAAGGTAGACAAAAAGAGTATAACTGCGTTTAGCAGAG[A/G]CAACTGCAACAGAACATATCATGTATGTCTAGAGTCATTAGTGAAGTAGTAATATCAATC
773	CaSNP2718	CTAGAAGATTCTTGTCCTGGTTATATGCTCCAGGTATTATTGCTTCGTGTGATTACCAAC[T/C]ATAACTTGTTTTCATCATCTATAATGCCATATGGTTTCTGTTTAATATTGTGGCTTTCAT
774	CaSNP2719	GATGTGAAACAAATAAAATTGCAATCAGTATTATTTGAGATACATATGATTACATACAAT[A/G]AATCATCTAGTTTAGGTAGGATAGCGTTTTGTTTCTTAAACATCACAAATAGACTGATTT
775	CaSNP2720	CTAACTATGAAATGCATGAGCATACCCTAACACTTTCACATCAATTATTATGTAAATGCC[T/G]CTATCCAAATCAATTAGGGATGGACTTTCTCTTAGATTTTCATACCTTATTTGGTTCCTA
776	CaSNP2721	TTCAAGTCCGCCTCTAAAGTTTATGTTCTACTGACTTTGATGACTACGACTAGTTCCATA[T/G]TGTTGCCTCTGATACACAACACACTCTAAAGTTGCTCTGACTTTGATCAGGAAACAAATG

777	CaSNP2722	CATATAATAAATTAATTACCAGCATGAATTGGATCAGGAACAAAGTCATGACCAAGTGTA[T/C]GCATTTTCATCAAAGGAGTCATCCCTGCTGTATCACCAAAATCATAAGCATATATCCCTT
778	CaSNP2723	CATCAAATAAGTCAATCAAAACATATATTTTTGTGTATTTGAGAACATTAGAAATAGAAG[T/G]ACATACACTCTTATCTTCTTGTGAGATGCGGGAGATCCGCCGTTTGTCATACCATTTTCT
779	CaSNP2724	GATATACCTTAACCTAGCCCTAGTTTACACCTCAAGTGTATATTAACATTGAACGTTAGA[C/G]TGTTTGTATATATTTTGTAGGTCTTCGCCACCATGGATTTTGACCAAACACAACCACCCA
780	CaSNP2725	TATACGGATGGAATACGGTTTGAATACGGTGTAGAGTAAGTTGTACGAGATGATGATTGC[A/G]AAGGGAAAGAAGAAAAGTTTGAGGAGTGGTGGTGGTGTTGTTTTTTAATGTGTTTGAGAG
781	CaSNP2726	GGACATTATTGGTGTCTATGCATCGTATCTGTTGTCCACTTCTTTGCTTCATACCATTAT[T/G]CCTATCTAAAGATACATTCTCCCATTATCCTATAGCATAAGTGTAGAACCAATTTTCTCC
782	CaSNP2727	TTATGATCATATGATTCTGATGCCAACCACTTGGTTCCTTAAGTGAGGATGTTTCAAATT[A/T]TGATTGTTCCATGTGTACATGAATGTATGTATTTTTATTAATTGGATGGTTAGAAATTAA
783	CaSNP2728	GGAAGAAATGGTTAATGCTGGTTTGGATGCTAGTTTACTTGATGATGATGCTTTCAATTT[C/G]GAAGCAGCACAAGATAGATGCATATTAAGGCTTATAGCATCCTGCTGCAACAGTGAGTTC
784	CaSNP2729	TTTCCTAAAAGTGGAGTCATACGTGTTATGTTAGTTGGTAAGATGATATATTGTACTCAA[T/C]TAGATTGATTAGTCTTTGCATAAAATATCATATTTACACACAAATGTTACTCATTTAGCA
785	CaSNP2730	TCAACATGGAGAGGACTTGGGCGCATAAGATGTTGCTTGGTTGCAGAAGACAAGCCTTTA[A/G]CAGGAGACAAGGATTTATGAAGAAGCAAATACATTTTATGAAGAAAGAATGACAATTATG
786	CaSNP2731	ATGACAAATTCATAAGCTTTAAGATAGGTCGATAATATCCCCATAACAATGGACTATGAC[A/G]TATTCTAAATGTCGACAATGGAGACACATGACAACAAACATTGTCGAGTCAATCAACACA
787	CaSNP2732	TTCTTATCATCCTCAATTGAATCTCATACCATTCAAGAAGTTTTTCCTATTTCCTACTTT[A/G]GTCCATCATTTTCTCCTTGGCAGGAATCAGTTTCAGTTACATATTCAACTTCAAACATTC
788	CaSNP2733	ATCTGAAAGAAATAAAAATAGAAATACAAGCACATTTCAAGTAGCACACACAAGACGCGA[T/C]GTACTTTTCATCAAGATCAAGAATGACGATATTAAGCTTCTTTACTTTTATCAATTTCTA
789	CaSNP2734	CAATTCAGTTCTTATTACATCATATTTCTAGTAATGTAGAGGCACATTCTTATAGAATTC[T/C]TGCAACTACAAATAATCCAAGATTTTACAAAATCTCATGAAATAATTGGAAAACATACTT
790	CaSNP2735	CACTACATGAAGAAGCACTTGGCATTTACATTGATGATTTTAGATCATAACGTGTAATGG[T/C]GTGGTGAAAGGGAATGATGGAGGGTAAGGGGACAACCGTGTCTCTTATGAATTGGTTAGA
791	CaSNP2736	TATACCTTATTTTTTATTTGGATGTCATTTAGGAAAAACTTCTTAACTTTCAGACAGTAA[C/G]GTAGGCAAGTTGATATTGTGCAACTGCAATATAATATAATAAAGCAATAATAGAAGAGGT
792	CaSNP2737	TTGATTCAGTCACAATTGCTGTCAATGGGGTCGAATAAACTGCTTAATTTTAAAACCTTG[T/G]TTGCTAGCTACCTTAATTTAAAATATACTTCTTAATTCGTGCTTTGTTATTATTAGATGA
793	CaSNP2738	CTTGCAAATCGAATTTATCCCTTCATATAATCGGAATCAATCACTAGTCCTCTGTAGCCT[A/G]TGTCAACAGCAATGGAAGTGGCAACAGCAAGCATGGTGAGGCTGCCATTATCTAGTTAAT
794	CaSNP2739	TAGTGATGCATCATATGTTTCTACTGCCAAATCACATTACACCTTTAACTCTTCAATCAA[T/C]CGTTGAAGGTACACATCAATATTATTCCCTAGCGTTTGTGGTCCAGGTATTAATAAAGAC
795	CaSNP2740	ATTCATTTTCAATGCTTTAATTAATTTATAAATTTTATATTTTGAAGTTGAGTTATATGT[A/T]TTGTAAATGCACATGCAGGATGGGAACAGGAACAGGACCTTGGAGCTATATCCGAGGACG
796	CaSNP2741	TTACAACACAATGAGATAATAGCATCATTTGAAAAGAACATCATCCAAAAGGTGCATCGA[T/C]ATAGTAACAAATTATACACCAATTTTTGTGGTGTTGTATCCAAAAATGCAATAGATCTCG
797	CaSNP2742	AACAATAATAGCATTTGACAACAAGGGATGTGAGTTCAATCACTTTACAAATAAGATTTT[C/G]CCCCCCAAGTATCACACATCTTACTCTTCACTCATTATTCTAGCTATTCCCTCTATCCAA
798	CaSNP2743	GCACATGGATAATATTCATAAAATATAACTGAAGACAAATTCCTTTGTATCAATAAACTT[A/G]ACTGAACTATACTCTTATCATGGAATCCAAACTCACAAGTAACTGCCAAAAATGAAAACA
799	CaSNP2744	TTTTATTACAAACAATTCATTTTCAGAGTAAACTAAGGAGATCCATGAAATGTAAGATGA[T/G]TTTTTGTTTAATGTTTGTATAGAGACATGACATAATACATAGTGTTGTTGTAGACTCCAA
800	CaSNP2745	AACCTTGAAATTATACAACTTGATGTGAAAACTGCATTCCTTAATGGTGATTTGGAGAAA[T/G]AGATCTATATGGAGCAACTAGAGGTTTTCGAAGTTAAAGGTAAATAACAATTTGTGTGTA
801	CaSNP2746	CTTATTGAGAAGGTAACTTGAGACTTATCTAGCAAATGGTTTGTGCAAGACATTTTCTAC[C/G]TGATAAAATTGTGAAATTATTTATTTGTCACACATGCAGAAACCTGGTGGAATTATTGCT
802	CaSNP2747	TACATAAAACATTTGTTAATTGCATATGTCACCCAACTTATTTATTGGCTTTTCTCCGCT[C/G]ATATTATATTACACGGTTTTCCATTATAATTAGCTTTGATTCATTAACCAATATAACAAT
803	CaSNP2748	TAATCGCATTATTTTTTAACCAACTAGATCTTTCAAGTATATGTTACAAGGAATTAGAGA[A/G]TTGTCAATATACATATGAGTGCATGACTACCACCAAACTCAATGTGGTTGCATCTTATCT
804	CaSNP2749	CATATAAATTTTGCTTCATTATATCAAGAGGCAAAATATCATTCGTCCTAGTACTACTTG[T/C]TGTTGATTAATTACTAAAGATAAATAATTTTTCCCATTACAATATACATAAAAACTATGA
805	CaSNP2750	CCTTCACCATTTCACTTGTCTTTATTACAAAGTTTGAGCTATTTATTAATTTAGACAATA[T/C]CTAGCTTTTTGAACTTATACTCATTATTCATACTTTTGAATCTAACAAATGGTTGTAGAT
806	CaSNP2751	TAATATAAATATGCTTTAAACAAAATTACTCAAGTGTGGGGTTAGTGTGATTAGTGTATA[T/C]TGAAAGTAAAGGAGTAAGTTAGAGAGAATCACAACTCGACCTAATATAACCTGTATTATC

807	CaSNP2752	ATTGAATGTTTAATACCCTTTTTGAACAAAGTCCTTAATGAAGTAGTGTTTGATTTCTCC[A/G]TGTTTAGCCCTAGAATGAAGTATAAGGTTTTTGGCTAGGAAATTGACATAATTGTTATCT
808	CaSNP2753	TTGATTAGTATCGGTAAGTAATTGGTTTGAATTTGGCTCTTCTAAAGAGAGAATTTGATA[T/C]AATGAATGAATCAATGATTGATATTATAACTATCCATGATTGGTAATGCGTGTGCACATA
809	CaSNP2754	TTGTTTTATTTAGATTTGCTTAGTATATCTTTCGCAGGCTGACCTCAAAAGGCATGAACG[T/C]GAATATAATCTTGCCCAGAAAGAAACTGATGACCTTCGGAAACAGGTAAATTGGAGTGTC
810	CaSNP2755	GTATTTGTGTTTGGGATGCATCTGCAGGAAAGACTCCAACAACTAGATAATTAATTAAAT[T/C]AGCAAACCAAGGTATTACCTCACGCAACTGTAGCAGCTACTCATTAGGGAAGTCATCTTG
811	CaSNP2756	GTTGAATGTGTGGTATTTATATTATTGTGATATTGGATGTCAAATGAATTTGGTGCATAT[A/G]TTTGATCAAACGAGTTTATGTGAGGTGATAATAAAATAAGGATGCATTGTATGATATAAG
812	CaSNP2758	TCCCCATCTTGACACACTTGGCCTGGTAAATCTTTTATGCAATAACTCTTCAATTTGACC[T/C]TTGAGCTCATTCAATTTTAATGGCGACATTCGATACGACGCAATCGAAATAGGTCTGATT
813	CaSNP2759	ATTCTAAAGATTATGGTTCCCCAATGAAACAATTGACACCAACACTTTGAAGGGGATCGA[T/G]AAAAAATGGAAGATATACCTCGACCTCGAGTGAAGCTTGGAAGCCAAGGCCTAGAGGCAA
814	CaSNP2760	CCTCTTATCACTTAACCCGAACTAGTTTTAAATAAATTAGCAAAAAGAATCAAACTTAGG[A/G]TGGATAATTCAATCCTCATTCTCATGTTTGCACCTTCATAAGACCCACTCATATGTGTGT
815	CaSNP2761	CTTGATAAATAGCTCTTCATCAGTTATTGCTCCCATTTCCATTGCTCTTTCAAGTGCAGT[A/G]TGTCCACCAGCATCTTTAACTGACGGTTTTGCGCCCCTGATAAATCGGCATGTTAGTTTA
816	CaSNP2762	ATTTGAGACCAACAAATACATGGGTTATTTTGATTATAACTGAGGCCCAAGGGAGAAGCA[T/C]TTATCCTACATGTTGTTATAAATACAGAGTGCGGTATGATTTAGAAACATAGAAATCACA
817	CaSNP2763	TAATGATTAACTATGCTTAAATAGTAAAACATTTACTACGTGGGTGTCCATAACATTCCT[A/C]TTACGATATATATCAATTACCTACTTTTTTTAGATGATTGACTTGTTCCGCTTAGCAATA
818	CaSNP2764	TTCACCACATAGAAATCTTATTATAATCTTCTTGTTCTTTCTCTTCTTTCTCTCTTTGTT[A/C]ACTTTAGCAAATTCTAGACTTCATTCTCAAACAATCCCATTTTCATCTTCCTCAACAAAC
819	CaSNP2765	GGGTGATCTAGCATTGGGAAAACAAGCCCACAATTACATTTGTGATAATAATATTATACT[A/G]AGTTTGACACTTTGCAACTCTCTAATAGACATGTATGCAAAATGCGGTGCACTTCAGACT
820	CaSNP2766	GATTCATATTTGTGACATCTGAGAAACTTTTCAAAGTGCATGAATGATATATGCTGACCA[T/G]ATGGTAGTTGTGGTAGAAGATCATTTTGATGATGTTACCCTTAAATGGGTGTACCATTGT
821	CaSNP2768	TAAGTCAATTTCAAGCATGAACCGGAACACAACCTCATTGACGAGCGCCTTGGGCTTAGG[T/C]AGTCGAATCATCAACAACTTCCCTTATAGTATTGTTCCTAATCTTGGGTGGAATTTTCAC
822	CaSNP2769	AAAGTTCATCATTACTTAAACCCTCCATCCTCTTTCCACTTCTTGCTTTGTTTGCCACCG[T/G]TGATGCTGCCTCTGGATTTATTGACCCCAGCCTGCCTCAATTCCTATCAGTTGTTGCAGT
823	CaSNP2770	TCACATTTTCCTTTAATTTCTTACAACTTTAATAATAATTTGCCTTGTGATACATGTCAA[T/C]TTGCTAAACAAATAAAGTTACCTTATCCTATTAGAACTAGTCAATTTGCACGCTAATATT
824	CaSNP2771	AATAATAAACGAATTGTGACATTACTCAGCCACTCCTGTATAATTTAATACCAATGAGTT[A/G]CAAAGAAAATCCCATTTGATTGACCTAACTAAATATGCTTACAGGTCCTTCAGTAGCTCA
825	CaSNP2773	TTGACACATACAACGCACCACATATGAATGATTGTTTGCCATCTCTCCTTTGAATCAACA[T/C]TTTTCCTACTCCTTCTGCATTTACTGAACTATTATTTGCAAACCTGATCTCCCTTTTCAC
826	CaSNP2774	AAAATTAGAATTAAAAACGAAATACAATGCACTAAAAAGAAAGTTACCTGTGTCATGGAC[A/G]CATTGGCGCGAGTGAAGTAATGGTCTGAAGTGTCATCAATAGTGTCAAATTGCTTAAAGT
827	CaSNP2775	TACAGATTACCGTAGCCTGAAACACCTATTACATCAGCGAGTATCGTCGCTGGATCAACA[A/G]TGTTGGTTGGCAAATACGAGCCGGATTTGGAGAACAAGGCAGCAGACGCATTGTCTTGCT
828	CaSNP2776	CTTATCATAGTTTTGACTTAGTCATAGTGTTGACTTGAAGTGTTGACTAGATAGCCAACT[A/G]GTTTACTAGATTTATCAGACGTAGAATCATTAGGGTGAGCTTGTAATTAGATTCAACATA
829	CaSNP2777	ATACGTGTAAGACGGGTCACAGGTCTCGCAAGTGACGGGTTTACGGGTAGGGTTGTTACA[A/G]GTAAGTTTAAAACAACTAGTAACCCTCAGATCAGATTTGTATCAAACAAATCAACACAAA
830	CaSNP2778	GTTGGAGAGAAACAAAAGCGGTTTGTGATCCCAATATCATACTTGAACCAACCAATGTTT[C/G]AAGACTTATTGAGTCAATCTGAAGAAGAGTTTGGATATGATCATCCCATGGGTGGCCTCA
831	CaSNP2779	CCAAACACATCAAATCTAATATATGAGGCTTTTAGATTAGCTAATAAATCTGATATATGT[A/G]TAAACTATGACTGCAGTTATCTGGAGCCTCCCCTGAACTGATCAATAAACTCATCCCAGA
832	CaSNP2780	TAATCTGTCAATAGCAGAGAAATAAACAAAATCGTGCCGAAGACCGAATAACAGAACCAC[A/G]CCGAAAACAGAAGAGCATAATCGCGCTGGAGATAGAAGAGTAGAATCGTGTTGGAAACGG
833	CaSNP2781	TATATTACTTAACTACTAACTCATTCAAATTCCTAGATCTTGCTTTATATATTCCATTTT[A/C]TGTTGAATGATTTTCTGTTTTTGGTCTGTGTGTGTTAAGTTTTGCAAGATCTGGTTTACA
834	CaSNP2782	CAGAAAAGGTGGGGGTTTTACATTGGAAACTTGAAAATAAAGATGTCCATGACGTTCAAC[A/T]TTTATGAATATTTGTTGTGATATTATTTATTCTTATCCCTGAAATAAGGAAGCAATGCCA
835	CaSNP2783	TCAGATATCAATAACTGAAATTGAGATGTTAAAGAAATAAAAGCACTAGAAAGAGAAGTA[T/C]AAACTGAGGAATACCTTTGCTAACCCAAAATCTGCAAGTTTGACAGATCCACTTGCATGC
836	CaSNP2784	CTAAGATGTTGTTGTAAGGTTTAAGGATTGAGGCTAGTAAACCTAGTTGTTTATAAGCTT[T/C]GTTAAATATGATTTTAGTCGAGTTTACTTAGTTGATTAAGCTCATTTTCACATTGTAATT

837	CaSNP2785	TTGAGTACTGTGCTTGTTTTTCTGAATGTTATATGTCTTTTTTATGATGTTTGCTCACCG[A/T]TGATTCTGATTTTATTATTTCATTGTGTGCCTTTTATAGTATTTCAACACCGCGCCATAT
838	CaSNP2786	TGGAGGTGAACCAGGTAAGTTGAGCTTGTAATGAAGAGTAATTCCTTGGCATGTAATGAA[A/T]GAGCTGTCTGAGTCTGCGAGTAATCTGGCCTGCAATTCTTCGTCACGAGCAGTCCCAAAA
839	CaSNP2787	CAACCGAATGATAGAGGAATGACAATCCTGTAAGGACTGAGCAGTATAAATTGCAAGTCA[T/C]AGAATGATTTACTGCACATAGTAAAACAAATATTATAGAACCCAACACATCAATAAAATT
840	CaSNP2788	ATTCTTATGTTTCATAAAATGTATGTGATATTTAACAACGATGAATTTTCTATATAGGTT[A/G]AACGAGCACGTGAAGTGTATAAGATGATACAACAATATAATATAAAGGGATCTTCAGAAG
841	CaSNP2789	CTTTGGATCGTCCAAATGTTTTTTCTGCAAACGGTCAAACGCATCCCAATCTTGCAGGTA[T/C]GGGCTAAAATGCTGGATTAGAGCTTCCATTGCAGCAGGTTCACCATAAGTCAAGTAAGGC
842	CaSNP2790	CTCTGAAAGGCCGCTACAAATGCTCTCATAGTAGTCTACCACTCTCAATCTATAATGTTC[A/G]CCACCCGAGGACTTCCAAAGTTTCGAAACCTTTCAAACATATGGATGTTTCATATTTTTG
843	CaSNP2791	TCACTAGGATTCAACTCATTTTTAGTCGACCTAACCCTAATATAAGTAATAACCTAGCTT[A/C]TTGACACTAAACCTAATGGAGGCATGGCTTGGATGTCCATACAAATCAATAAGTGAAACC
844	CaSNP2792	ATCATATCAAAAATGAGATTTATGAATGGAATATCATGTTATATAGAGCAAATTATGTAA[T/C]ATATGGACAAAACCAGAAGCAGTGGCCAAAAGTAAAGTTGTGCATCGTAGATAGTGGTGA
845	CaSNP2793	TGAGTTCACTCAACACATACCATGCAAAATAGTGTTACAACTAAAATACCACTATGCTTT[A/C]TATATTATTTAGCCGCCATATAATTACAATCTACAAATAAAGGTAATATAAAAAACATTA
846	CaSNP2795	CAGTGTTGTCAATTGGAGATTGCAGAAAATAACAATTTATTCAAATTCCACTACACAACA[A/G]TGCTATAACACCGATATAGCCACTATTTGACAATATTTTATACTAAATAGCGTATGGCGA
847	CaSNP2796	TTATTGAAGATGACAAAACACATGTCAGCACTCGGATCGCAGGAACAATGTAAGTTATTG[C/G]TATGTTGGAGTAAAGCATCACTACTTGTTTGTTTTGAAAAAGAAAACATAAAGTCTACAT
848	CaSNP2798	GGTAAGAGATTCACATGATGGTTGAACATCTTCTTGGTAGTTGGTACACCTGAACCCTCT[A/T]AATTTGCCCACAATTTATGTACCTTAATTTGAAAATATATTGCATAAGTACAGGTTGTTG
849	CaSNP2799	TAATAGACTGTTTAACTTCATCCATATGAATGTGGATGATCATGCAAATTTTCTTCATTA[T/C]GGGGGTTGTTCTAGTAGTGGCTACTGGCTACTAACGAGTGAGTGATGTTCAAACGCGCAG
850	CaSNP2800	CTACTCGATAAGAATGAAATAATGATTAAATTTTGTATAAAGTATCATTTGTAATCAAAC[A/G]TAGAAAGTTGACTGGGATCCTCTGCTGATACTCTGTTCGCTGGGAGACAGAGAGAGTACA
851	CaSNP2801	TGTTAATGCTATGAATCATTTCTCTTTGCAATCTCAACCTTCACATTTTATACATCTTTC[A/T]CACCTCCATAATCAACAACAAAATGTAGTCTCAACTGGCCTTCGCTTATCATTCGATGAT
852	CaSNP2803	ACAATAACAGAGTATATATATACAAATATCGTATTATAACAAACACGACTGACATGTCAA[A/G]CACCCTAATGCAATGCTTATATGTCAATGCACATGATTCTGGAATTTCCAAACCCTCATA
853	CaSNP2804	TACATAGTACATTATGGTTCCTCAAAGGTGCTCCAATGTTTGTTTTCTCTACAGAGTGGA[A/G]TGCAACATAATTCTATTGTTTTTCCAAAAATGACTAAAGGTAGTTATTCAGAAATTATTG
854	CaSNP2805	ACAGCCATATCCGAACCCACCAACTGTCCAAGGAAAGCTGCCTGCAGAATAGCAAAAGAC[A/G]ATATATCAGCAAGACAGAATGCGCAAAGACTAAAACATGGAAAACATATAGATTGCATGA
855	CaSNP2806	AAAGTGTCATACCTGTAATCGCGTAACTTTTCCCCGCAAGATTCTTAGGTAACAAACACA[T/C]GGTAATATCAAAGTCTGTTCAACAAGGTTGATGTTGATTAATAAAATGCTATGGTGATTA
856	CaSNP2807	TGCAATCAGTGGAAGAACAGGAAAAATGGAAAATATATATGAACAAATACATATAAGAGA[A/G]AATTAGAGTGGCACAATTGAGGAGTAGATACATGCCAATGGTGGTTTTGTTCAGTAAGGA
857	CaSNP2808	CTCTCTGTAAGGAGCATGATTCAACTCTACCTGACCATTGTTTACAAACCCCTTTTCCAC[A/G]TGGGTTTGCATTGCTGGATCATCCTTCACCTCCACAAATGTTTTAACAACGTCTTTTCTA
858	CaSNP2809	CAGCTGTTACATGCATCCATTGAATATCAAGTCATCGCCATTATCATCCCTGCTCATGTG[A/G]ATGAATGCACAGTGGCATCATATTCATACATTTGGCTATGTTATACTAATCAGACATGCA
859	CaSNP2810	TTGCCGAATTCTGATGTGGGGCTGATTTAGAAGATTCAAAATCACACCTTCTAATATCAT[T/C]TTCCTTAGTCATATTTGAAAAGGAGGACTTTGATACTGGAGTTTCAGGTTTCTCATTGGA
860	CaSNP2811	ATTAAATTAAACTTGTGATCCAATCCTATTTAAAAGAGGAGGAATACTCCAAATTAAAAG[T/C]ACAATCATATAAAAGAGGCAGAAACTATGATGTCTTTCCAGAAACATTTCTTCAAGTTGC
861	CaSNP2812	AAGTCTGAATTGTTAACCAAGATGGCTGTTTATGACAATAATAATTCACAGCTTTTTGAT[A/G]TAGGCAACACAACTACATTGAGTTTTTCTTCATATGCTGGCATTTCTAGACATCTTTTTG
862	CaSNP2813	GATTAATCCCGTAAGTATTTACTAGACACTTTTTCTACTTTGGAGCATACATGCCATAAT[A/G]GACCATTGGTTCACACTAACAGACTCAACTTTCCAGAAATTAACACAATAAATGACAATG
863	CaSNP2814	CTGTATTTATATTGCTGTTTACTATGTGGCTTTATGATGGGAATGCAGCCAGGGGGATTT[A/C]GGATAAAACAAAAATGTGGAGAGTGTATGAGAGGAGGTCTAAGAGAGTTAATGACGTGGC
864	CaSNP2815	GAAATTAATAGGATATGTGGAAATAATTAGGTGGAAAATCAAAGTTACTTGAAATGCAGG[A/G]TCATGCCTGTAAGTTATAAAATAAAGTAGTTAAAATGTCATCGGATAAAACAACTTTATT
865	CaSNP2816	AAAAGATAAAAAGTATGATGTGGAGTGGAAAGATTGTATGTGTGGAAAGTGGCATGAAGA[C/G]ACCCCAAACAACCTTCACAAAAAAGAAAGAAGGGGATGCTAACGTTATATTGGCAAATCC
866	CaSNP2817	GGAAATAATGATGACATTTATGAGAGTTAGTGTTGTGTGAATTGAAAAAATTGGGAAGTC[T/C]CACATATGAGGGATATCACACACTAGTAGGGTATATAAGGTGAAGGTATGGAACTTGGGG

867	CaSNP2818	GAGAAAGCAATCTCATGAACTGAAACATTCACATACATTATAATCATGTGGGAAACAAAA[T/C]CATCAAAGTGAATATGTAATTACTAATTGGTAATTGATCCCTCCATTCTAAAATAAGTGC
868	CaSNP2819	CAACCAAAATGAGAAAAATGTCATAAGCATCCATCTTCCAGGAATTGGATTAAAAGGCTC[T/C]ATTCCAAACAACAGCAGCCTAAGGAAACTTGATTCCCTTAAAATCCTAAGCCTTCATTCC
869	CaSNP2820	TCTTTCCTTGAGATGGAGGTCTTGGATGACGAGATATTACATGGATCGCTATTTGAAGAA[T/C]CAGACTTTCTATAAAATTCAGTCACAGTCAATTATCGATAATCCGGATCAGCGAATTGTT
870	CaSNP2821	TTATTTATAACACAAGGTGGACTCATGGACTTACTTTCTTGCCCTTATGTTACAATTCTA[T/G]AATTGTTTTACAAAATATTCTCAAATAGTTATAGTTGATTTTTGTTGTAAGGGTCTCTTG
871	CaSNP2822	TTCTCGTGTTTGCACCTTCAATTGGCATCAGAGCTATGTCTCAAGGCTGAGTACTTTACA[A/G]TGTTTGAGGAAATATCCAAGGGTTCAACATGTCTAGATTCTGTAAATTAGCAGATGAAGA
872	CaSNP2823	AAACTCAGCAGGAAATCTTCCTCTTCTATAACAATAGGCAACTTTCCAATATCTTCCAAC[A/T]TTAAATCCACACAATATACAACACATAGAGTCCAAAAGATATTAGGATACTTGTCTTCTA
873	CaSNP2824	GCTAAACTGAGCAGCCTATCATGCAATGTGTGCACCTGCACTAGTTGATTAAGCTGCAAA[A/C]GTATCTAAACACAAACCTCAGTTTAAATATATTTGTTGTTTTTTCATCCATAATTTATGT
874	CaSNP2825	TCATTGCTGAGAAGCAACATGCTGTATAGTTCAAGTACACTGTCAAATGAGCAAGGGCAA[A/C]TACATCACCACCTAGATGTAGTTTAAAATAAATAAGAGTTTCCACATTTTTGTGAATTAG
875	CaSNP2826	AGACAAGACTTAAACATTGTAAAGTCTAATTCGACATAAGCTAACCTATTTCTACCTTAA[A/G]TTGTTGATAATTATTACCAAACATATCTTCTAGTCAAGTTATTGTTGTTGATGTTGTTGT
876	CaSNP2827	ATGTTAGTCAATCACAATAAAATCCCAATATTTTGTTTGCACTACTCACAATATACAATC[T/C]AGTTTCATCAATCACATAATAAAGAATCAAATAGGTGGCACATTCACGTAATATCACAAT
877	CaSNP2828	CTTCTCCAAGTAACTAAATTACCCCAAACATAGGCACAATATCCAGTGGCGAATTTTCAA[T/C]CAGTAATTGATCCTGCCCAATCAGCATCTGTAAATATAGAGACTTCTCTGTCATTAGTTT
878	CaSNP2829	GAAGAGGGTGATGATGTTGAGCTATGAGGTCAACAACTTTATATTTCTCAATCTTGTGAT[A/C]AAGGGAAATAACAACATGAGATGTACATCTTGTTCTAGTTTCTTCTATAGGCTCCCTTAT
879	CaSNP2830	AAGATTCTCATTTATCTCTGTTTCGATGGAGGATTATCATATTGATGTTTTTTGAGGTTT[T/G]TCTTGAGATGTAGTGCACCTTGATTGATGTTTGCGAGAATACATTGGAAAAGAGGAAGGA
880	CaSNP2831	TGAAGTTGGTGTGAATTTTTGAAGTGGAGATTATTTTGTCATCACATAGGTGGATATGTC[A/G]AACCTAGTAATTTTGGAGAAGTACAGCTGAATGAGTCTGATCGTGGCGGTCTGTTGTTGA
881	CaSNP2832	AACCTTCCAATTTATTCACCTAAAATAAATGATAGTAACCGTCAATTTTAAGAGCCAACA[A/G]TTATAAAGACAATGATTATAGTAACTACTAAATATTAAGAACTATAAATTGGAAAAGATA
882	CaSNP2833	TAAGCTACCTCCAACAAAGCAACTTGGGGGAAATCATCTGACTCTGATTTACAATGTTGA[T/C]TCTGTCTCGACTAAGTTGCCTCTGGCCAGTAATTCAACACTAGAAGATGATTGGATATGA
883	CaSNP2834	ATTTGTAATTTCAGTGGTGCTGCTCGTCTATTGGTTGCACTGGTACAAATCACGCATCCT[T/C]TATTCATCCTACGGTACTTCCTAAAATGAACTTCATTACCTTTTTCCTCCCTTCAAAACA
884	CaSNP2835	TATCTAGAACAATTTCTAAAGTGAAACCTACCATAAATAGAACAACGTGTAGCTGAAACA[A/C]TGGAATGTCTCTCAGAGCAAATGGATTGTGACGCTGCTCCAGAATATGATCTACGACTTT
885	CaSNP2836	ATTTCTACCTCTAATTCTGTTTCTGCCTCTGGTGGTATCCTCTGGTTCTGCCTTTGGTTC[A/C]GTTTCTGCCTCTAGTAGTATCCTCTAGTTCTGCCTCTGATTCCGTTTCTACCTTTGAAGA
886	CaSNP2837	AGATATGGAGTGCAGAAGTTCAACGTCTAAAAAAGAAAAATTAACGTATATGCACATCAC[A/G]TCTAACTAATGCATATTTCAATTCATGCATGCATACACTAAATGTCACTATGAAAAGTAT
887	CaSNP2838	ACAAAATATTATTAGTTGGAAAGTGATATTAGAGCCCTTAAGATGAATAATATGCATTCA[T/C]AAACTCTATTCGAGAATGACTAATTTGTTGGATCAATTGGCTCTTATGGAATCAACTGAG
888	CaSNP2839	AAACACATTGATATACATCATCATTTTTTGCGAGATCATGTGCTCAAAGGAAATGCTGAA[A/G]TAATATTTGTGGATATGCACAATCAACTAGCAGATATTTTCACAAAGCCACTACCTAAAG
889	CaSNP2840	TCCTATTTCCCAAACACGACCATCGGGAACCATAATTGTAGCAACATTGTTGAGTTCATT[T/C]CCAAATCGTTTTATAAATTCATCTGGAATTCTCTGCAATAATCATCACATGTTTAATTAG
890	CaSNP2841	TAGGACTCTTCCTCACATTGCTCCTCGTGGTAAGTTCTTCAATGCACCAATAAATATGAA[T/C]ACTAATAATGTTGTGATTGTCGCACATGAGCTCCTAGGAGCAACCTTGGTGGAAACAACT
891	CaSNP2842	AATCCAGCTCCATGGAATCGGTTCCCGATCTTCAGAACACTATGTACACTGACGTCGATG[C/G]ATTTCCTTGTGTTCGGCTGCTAAATCTTTCTGGAACAATCGGTTGTTCAAGTCAGTCTCA
892	CaSNP2843	ATAAAATACGGTCAACATTTGTAGAATATGTAATCAACATCTTTTCAACAATAAGATATA[A/C]TCCATTTACAAGAAGATTTAATCAAGATTTATCCACAAGAAGGTATGACTTATTTACAAG
893	CaSNP2844	GTCTCCTTGTTGGTGCACTTGTCAAACCCCATGAATGTGATTGCATTTATCATGAATGTG[T/C]TGGACTTATGATGCATCTTGTATATCTTTTTTGATCAACATTCATCTCCTTTATTGGAAT
894	CaSNP2845	TAACTAATTAGCTGACATTGTTGACTGGCTCAAGGCTGGTATGACTAGCTGGCTGATACA[T/G]ATTGGCATAAAGGGTGTTGGCTTACTAGTTGGTGGCTAGCAACCTCTTGTATAGAACCAG
895	CaSNP2846	TTGAATAACTAATTTAACTACATTAAAATCCAACGGCTCTATAGCCACAACGCATGTGCC[A/G]TTGCAACAGATAAACCAATGTCATGTATTGTGGTAGGACAGTGTTGGTAATAAACCTGTA
896	CaSNP2847	AATTCAATAAACTCATTCATATAACTAGAAATTAAATCAGAAAGTTGTGTTCTGATGTGC[A/G]CCATGCGCTCCAAGCGCACTTCAAGCGCAATACGATAGATCCAATGGTGCAGAATTGCGC

897	CaSNP2848	ATCAGAGAGAGGTTTAGTTCTTCAACTAAACAAATTCATGAGCCTTCAGAAATTGAAATT[T/C]CAGATCAAGAATCATACCTGACCTTTAATCTCTTGCTGCTTTCCTTTTGTAGATCCCACC
898	CaSNP2849	AACAAAATATCACTGTAAACATCATTTATAATAAAGTTTACATCTCCGTCTTACATTTTT[T/C]CATCTTTAATTAGGATGTTTGTGTTAGCCTAAGAAACACCTCTTGATAAAAGAAACATAT
899	CaSNP2850	AAAAAAAACCATCATTGAGTGATATATTTCTTGAGTGTAAACACTTAGTGAGTGGTGACA[A/G]CCTTATTTTTATTTTCTTATCTTGTCTTGTTATTTGCTTGATTATTGTGATACCCGTTGA
900	CaSNP2851	GATTGAAGAAGAAGCTCTCTTTTTATTTTATCATTCTCTAGCTTGATACCAAGCCAAATA[T/C]TATATCACTCTTGGTTTACTAAAAAATTTCTGCAGTATTTTTTACCTTTTCTCTTTTCAT
901	CaSNP2852	GCACTGGACCTCACTATGTAGAATTTTCTCCAAAATGCATATGTTCAACTCTCAGCTTTA[T/C]ACTATTAAACTTTATAAAAAGAATAAAAAATTATATTATTAACAAATACTCTACTCTATC
902	CaSNP2853	CACAACTAACAGTTTGCCGGTAGATACTGCACCATGGAGATGCTAGTGTTTACAGGCAAT[A/G]TTGGGACTTCAACCTCAACAATTACAACAGATTGTACTGTTTGTAAAGGTATTTATCCTA
903	CaSNP2854	CAACATCTTTTAACATTGATGGAACGCGACAAATACGTCTATCAATATAGGAAGGTAGAG[A/G]CTTCAAATGAGTTGAGTGACATATTTTGGGCCCATCCAGACGCTATCACTCTTGTAAATA
904	CaSNP2855	TCCAGTTTAAAAATAGTGTCCTTCACAGTTTATATTACGCCAAAAATGTGTAGTCTGTTC[A/C]TGGGATGCAAATCTTGAGCAATTTCTGGCATGGCATAAGCTTTAAAGAGATATGTCTTGC
905	CaSNP2856	CTTAAAAATGATAGGAAGAACAATATGTCCCTTGATGATCCTTATCGTTAATCTAGTATT[A/G]AGACAACTTTACATGCTTTACAAGATTTCCCTGTGAGTATGCTTGTTTGGGTCCATTTTG
906	CaSNP2857	CTCCATACAATCTAACTAACCCATATTGTTGAAGGCAAATAACACATTGACCCTAAAAGT[T/C]AAGTCTTCTCAACACAGTTTGTCAACCCCATATTGCATGAGGAACACTAAAGCCACAAGA
907	CaSNP2858	ATTCTCCTTGCATCATTAATGACAATTCTTCACATATTTTATTATTGGTTGATCCAAATA[T/C]GATATTGTCAACGTAGATTTGAATAAGTAAAGTGTGATCATAAGTCCTTTTAATGAATAA
908	CaSNP2859	ATCAGTTTGAACATTAGTGAAATCTCACAAGTGTGTGAGGACTGGACGTAGCATTCATTG[A/G]GTGAACTAGTATAAAAGTTTTGTGTGTCATTCTTTATCTTTTTCTCTTTCTTTTGCTCAG
909	CaSNP2860	AATGATTGCATCTCTTAATTCATCGTTGTCATCCAAGCTTCTTTGTCTTCACTGCTAATG[A/G]CTTCCTTGTAGTTGCTTGGTTCATTTCCTTTAAGATCTTCAACAACATTTAAAACATAAC
910	CaSNP2861	GACAAATAAATTTATGCACAGACTCAATTTTACATATCCCAAATTTGCTTTTGAGAATAT[A/C]GCAAACATACCACAAAAACTGGCCAGCTTATTCCATTATGTGACCTCTCTTGTTCTCCAT
911	CaSNP2862	CAGGCTACTATGATGAGTGTATCAAATAAAAGATAGTAAAATCACAAGGGTAGAGAAAAA[T/C]CTGAACAAAACATTAGGAGAACCATTGAGAAAGGTTATCACCTAATAGCGTTACTTGAAA
912	CaSNP2863	AAGGTATGCATTAGATGATATGGGTTCAGGATGTCATGTGCATCTAAGCTTGTGGCAGAA[T/C]GGCCAAAATGTATTTATGGCATCTGATGGATCATCAAAGTATGGAATATCAACTTTGGGA
913	CaSNP2864	GCTTTGATACCACTTGTTGGGGAGTCCGGAAAGCGCCAAAAGCAGCAATGAGCTAAACAT[A/C]CCAGATCCTTAAGAGATCTTAACATTTATTTCATTCATAGTAAATATAAGACTTTAACCA
914	CaSNP2865	TCATAAGGTTGCAACACATTGAAAATGTTCATTTTATTACTAATATAAGCACAATTTTGT[T/G]TATAGATTGTCAAGGATGCAATTGCGTCAGGGGTTGATGGTTATGATGATGAAGTCAAGA
915	CaSNP2866	ATTGGTTCGTTAAATGTGTGGTATTTAATATTATTGTGATATTTAATTTCATATCGTTAA[A/G]TATTGGGCATTTGATATTATTGTGATATTTGATTTAATTTCGTTAAACGTGTGACATTTG
916	CaSNP2867	GTAGCGGATTTTGTCTCTAAGTGCTTACTGTGTCAACAAGTAAAAGCTGAGCACTAAAAG[T/C]CTGCGAGGTAACTTCAACTTGTTGAAATACCTGAGTGGAAGTGGGAAGACATCGCTATGG
917	CaSNP2868	TGAGATTGGGTTGAGTTCGAAACAAATGAGTTATTTGATGATATTTCAGAAACCAAATCA[T/G]TTATAACTTGAGTTGCTATCTCAATTGTTGTATGAAAAGGATCCACAAGTAGGGGTGAGT
918	CaSNP2869	TAAAAACTAATCTCTAAATTATTTTATCTGAAAATCACTGTTTCTTCATCTTAAACAAAC[T/G]GTTATCCATGATTTATAAACCCAACGCCATATAGGTGTGGCATGGGACCAATGCCGACCT
919	CaSNP2870	TAAACAATAAAAACATGATCACAAGTTTCTAGGATCAATTGAGTTTGATGGCAATCATTG[A/G]AATATCTTAAAGGGTCTTGTTTATCAACATGAACATGATCCTCTAATAATACATAGGATC
920	CaSNP2871	AAATCAACAGTTTAACTTATCTATTTAAGTTGATAATCAAAATGACTGAAAGTATATAGA[A/G]ATAAGGGAAGAGATATCTCACACTGATATATCCTGGTTCGCCCAATTCGGGATACGTCCA
921	CaSNP2872	AAAACCAATATATGAGATTAAAATAATTCAGCCTTTGAATGATTTGAATCTATTTTAACC[A/G]AGTCTGTTACAGCCTTGTTTAGACCTTCTATCTTCTAGTTTAAATGTCTTTGAAGCTTCT
922	CaSNP2873	CCAAGTGAAAGATACATTGATAATTTAGAATTCGATCAGGAGGAAATTAAAATCCCACAA[A/T]TAATTATTTCCTTTAAAATATGAATTCGAATTCTATTGAACGATGTTTCTTTAACGAAAT
923	CaSNP2874	AGCAGATGCAGGGGGTGGAGGGTCCACAAGAAATTTATCAAGAAGTTGATCAATAGATGT[A/G]TAGTTATATTCAGGATATAGTTGTGAAACTTCAAGGTCATCTTCCCCTAGATCAAAGTTC
924	CaSNP2875	AGAAATCAAAATATCCCACAGCATGATGGTACACCAGACCAGCAAATTAAATTAATCCAC[A/G]AGAAAACAAACCTAGCTGGAACTTTTACCTTCTCCAGCTGCAATAAGAGATAGTGCATGA
925	CaSNP2876	TACAATGTGAAGAGATCAAATGACAAAAGAGTCATGGTTTGGTGATAGTTGGTCAACACT[A/G]TCAAGAATATAAAAGTACTTGGACCATAAAATAAAAGTGGTAGGATGATGTATCCTCTTA
926	CaSNP2877	CACCAAATAGGGGCCATATACACCACCATATGCACTTAACTGGTTATTCATTATTTTTTA[A/G]CCGTAAAATTGGATGCTATTGTCATTCTCTGTGTAGGTCCAAAATTCGAATCACATAGAG

927	CaSNP2878	TATTTCCCATTTTTAGCACCACTAACAATCAACACGAAGACAAGATTCCAGAAAAAGATG[C/G]ACATATATTTCCAACAAGACATGTCGAAAAGATAGGAAACATGTGTCGGTCGCGGGGCGC
928	CaSNP2879	TGCTATGATAACAAAAGGTTTGATTGAGCCCATATCCAGTGTTTTCGTGAACTTAGTTAA[T/C]GCTGATTTCACTGCTAAACAAAGAGGGCTAAGAATAACCGAGGAGAGAGTTATTCTTGAT
929	CaSNP2880	TATTGAAGTATACAACCACTATTGGGCTATGTTACAAAAGAGCAGGTTTAAGGGTTTTCT[A/G]AATTACTCTCACAAGAGGGTGTGTATAATGTTGTACTCTTAGAAAAATATTTATCAGCAC
930	CaSNP2881	TGAAAACTTGCACTATATTCAAATTTTCCACCATATTTATTGCACAAAACAAGTCATCCT[A/T]AGATTCAAATCATATGTCTAAATTTCAGTTCAGACCAGTTCAAGAAGATACTCAGTCAGA
931	CaSNP2882	AACATGAGAATTCTCCGTTTGTGAGATCGTGTTTGGAGAGATATCGAAAGTTTCCCCATT[A/G]TCCTCTTAATTTGAACTATAGTGATGATATTAAAGATATTACAATTGACCAGTTTAGGGT
932	CaSNP2883	CTTAGATTGAAGGCTTGTCCGATGTTTGCCACTTGTTAGGGTTTGACATCAACACGACAC[T/C]AATATATATAGTTACATTCAATCAATTATATTTTCATAATTATTATATTTGACATGATGT
933	CaSNP2884	AGAACCAATACATAACATCAAAGTGCATTTATATCACTTATAATTCAAGTAGGAAAGTGA[A/G]TGGAGTAATACATTTACCATTACCAAAGTTGTTTCTAGAATTATGTGTCACAAACTCTTG
934	CaSNP2885	TCATCTTCTTCAAGCTCAACTTCATGTGATCTAAGAGCGCTAATTAGTTCCTCTAGCTTA[A/G]GAGTATTCAGATCATTTGCTTCTTGATTGACATTGATTTTAGGTCTCCACTTGCTTGTCA
935	CaSNP2886	ATATAATGTCTGGAGAATTGATGTCAAGAAGGCTCTGATTCAAGACGGTTCTGATGCATA[A/C]GATTAGAATCAATAAGGCTAAGTCAAGAAGCCTAGAAGTCAAGAAGGTTCTCATATCCTA
936	CaSNP2887	AAACATGTCTTATTTCTTGGAATAGAAATGAGCTCAATAGAGAATAGAATGATCCTACAC[T/C]TAATGAAGTATGCGAAGGATCTTTTGGTAAAGTTCAACATGTTAGCATGCAATTATGCAG
937	CaSNP2888	GTGGAAAGTACAACATGAGTCTAATACCCAGTTCTTTTCACCTTCAGAATCTGAAATCAT[T/C]AATGCCTATGTAGATTCATAACCAGCATCAACAAGTGCATCATTTCCTGAATCTTGAGAG
938	CaSNP2889	CATTTATATGATTAATTTCCCAACATAGGAAGGATCTACCATTCACACCCAATAAGTTGC[A/G]ACTGTGAAATAACAAAAGAACAATACAAGCACCGACGAAAGTCATTCACGAATTTAACTT
939	CaSNP2890	TCGGTCTTCCACATGGTTATACACTACTATTGATTATTGACAATTCAAAAATAATTGATT[C/G]CCAAATGCTTTCCACACATAATGTATCTCTGACACAAGGATCGAGTCCAAGTATTGAACT
940	CaSNP2891	TAATTTGATCCTGGAGTCGATGTAAGTTACGTATGAAATTCCACTGCTAGTTTGATACTT[A/G]TTGATCTGAATGATGATGATGGATAACAATGCTGTCATGAGTATGACAATATAAATATCT
941	CaSNP2892	TAGTAATACTCCACATTGCTCACATCACAACTTGAGGATATAGACTTCGATCTTCCTCTT[T/G]TCTCACCTTGTTTTCTTGAGTGACTCCTCCCTCACTCAAAGCCTTCCACGGCTAGTGCAT
942	CaSNP2893	AATTGTTAGTGAGCAATTTCAGAGGCTGATTTGTGTAAATTGATTCTGATTTTTGCAGGT[T/C]AAAACATATTGACTTAAAGGAATAGTTGTATACTTTGATGCGTTTGGTATAAGACCACCA
943	CaSNP2894	TGTTAAGAATAAATATAACTTTGGAAATCAATCTAAAAACTCCCACTTGCATAGTTCAAT[A/G]AAAATAATTATTAACTATCCTAATCTTTGAGTTGGTATCCTCTTTATGTCTCTTTCTCCA
944	CaSNP2895	TCCATATTGGACCATGAGTAATCACACAAGTGAATTGGGCATTACACTCGGGGATCATCC[A/G]TATTGAACTAAGAACAAATACACAAGTGAATTAAACATCACATAATATCACTATTGAGTC
945	CaSNP2896	TAACTAATTCTATTATAAATTTATATTGTTATGTCTCTACCGGAGTACTCTAATAATACA[A/G]AGTGTCTGCAGCCCTCATGCAATATGAAGTTTCTCAGAGACTCATAATTTTTCTTCCTCT
946	CaSNP2897	ATTTCTGATACTAGAGCAGCAGATACTCCTCTTTAGTTGAATGTGAAATATGCTCCCTCA[A/G]ATGGTGTTCCTTTACCAGATCCCACTTTGTATCGTACTTTGGTTGGCAGCGTAGTGTATT
947	CaSNP2898	AGGTTCTGTAGCTGCTTCTTTGTGTTCCTTCAATTGGTGTAACTCAACAAGTTCACTGTC[A/C]GAAACACCTCTATGGATCTTTTGTTGTTCTATTGACTTAGTTTCCCTTTCTGCAGCAGCC
948	CaSNP2899	ATCATTTGAAACGCTAATATATTATCATAATTACTTCTAAAGTCACACATATGATTAATT[A/G]TGATGTGCTGAAGCATATAATTTTTTACACTGATAGACAATGCATGAAAATTAACTTCTC
949	CaSNP2900	TATAGCTAGCTACTGATGACTCCAGTTGTTTGCTTGAGCACTCAAAAGGTTCTCTCTGGA[A/G]ATAAAAGGGAATCAACAACAAATATAACAAATTTAAGAGGAAATCTATCACAACCACAAA
950	CaSNP2901	TTACATTGTGATTAGTAATTTTTTGACAAATGTAGCTTATGCGGCACAAATATGAACACA[A/G]TGTGATTGTAGAAACCTCGATAACTTCAATACTGCAATTGCATACCACAATAAAAACTGC
951	CaSNP2902	ACACCAAAAATTGAAGACACGTCCAGTGTCCGAAAATGTCGTATGGCACCGACACATATA[A/G]TTATATTCAATCACTTCCATTTTCTCAAATTTTATTGGTGTCTACATGTCAATATTAGTG
952	CaSNP2903	CAACAAACCCAACTCTAAAGAAAATTATAGAGTGACTATTGTAGCTTTTCCTACAACTCA[A/G]TAGTACTTCAACACAACCTATTGGTAGTCGGTCCAACAAGTAGTACATATTAATGATTTG
953	CaSNP2904	CGATTGCTCGGCCATAAGACTGATGGATAATCCAAAGTAAAACAGTCGTTGAGGGATCAA[T/C]CAATCAATCAATCCAATCCATAAATATAAAAGGAATCAAAGCAGAGATACCTTTACAGCT
954	CaSNP2905	TTTGTATGCAACATGTCTTTTTTATATATAATTTACTTATTTTGACCTCTATGCGGCCTC[T/C]GCTATTTGACCGCAACTCTATCCGCTGCTTCACATAGCGGATTTTTGGTGTTTTGCTATT
955	CaSNP2906	TATGCAAATCTTGGACTCTCTTGTTTGAAAACCCCCATTTCCTGATAATGTATCACAATT[T/G]TTTCCTTTATAATAACCATTTTTATTATGATGGTACTTCTATCCTCCTAAATCATACTAT
956	CaSNP2907	AAGCATCCAATATAGGATTGTAAGTAATTAAATTAGGTGGTTATCGCTTTAGGAATGATT[T/G]TTCTAAAATGCATTTCTTTGAAGAGGTTCATGGCTTCATCCACCATTTTAATCTTACAAA

957	CaSNP2909	ATAACCTCCATTTGAGGAATGCTATGCATTAAATCAATTTAGCTCGAATTTGAGATTATA[A/G]AATCAATGATATGTTGCATTTGACTTGAATGTGATGCGACAACACTCTGATGAGTTATGT
958	CaSNP2910	ACATGGTTCCACGCCTGTATACAATATTAAAGAACAGGTATCGGATAATTGATAGCTAGA[A/T]AGTAAACTTAAAGCATTCATCAGCCCTGTGATGGAATCGCACTATCTATATGGATGTTAC
959	CaSNP2911	TTTGCTTGTTCAGATTTTGTGCCTGAGAAAGTGAAAGCTGAGGATGCATTAAGGGAGACA[A/G]ACTCAAGAGGGAATAGATGAGCTTAGGAAATGTAAAGCAAAGATAATGGAGAATGTAAGC
960	CaSNP2912	AGTGCTAAGGACAATGTATTGAATGTTATCTCTTTTCTTTGTTGTTATTTGAGCTTTTTT[C/G]GTCAAATCATTGTAGATGCTTAAGTCTATGAATTGCAGGAGGGAATGACGCCTTTGAATC
961	CaSNP2913	GAGTTACAACAAGGTGAACTGGAATTTACTCTGACAGTTTTGAAGTTGGGTGGAAGTGCA[A/G]CCACAATGCAGTAGCAACAACTTATGATTTACTTCATCAGATGAACGGTTCTGTCTGTAT
962	CaSNP2914	ATATTAAATTAATTCGTTATTTTCTTCTCAGCCCATAATCTATCAATTCAAGGGTTTGGC[A/G]TTAGAAGGACCATATTAATGGATAATAATTATGACTTAACAAAGGAGTCGTAATACGTGT
963	CaSNP2915	ATTCTTTTTGAGATTCTTTCATTGTTGAGTAGATACTTATGTACTTTGATAATGTGAATG[A/G]CTTCTTGCTTGTTCACTTATGCAATTCATGTTCCTTAAATAAAACTCAAGTGCAATCATT
964	CaSNP2916	GTCCAAGGAGGGTCATAATCATTGCATGGAACCAAGAACCATAATTATTGTCGGTGAGAG[C/G]GTGGGAAACAAGAACAAGATCAGGATTATTAGATTTGGAGATGAAAAAATGATAGACACT
965	CaSNP2917	TGATCAATGTACTACAAGTGAGAAATACAAAGAAGAGATGAAGAAGGTTCCTTATGCATC[T/C]GTAGTTGGTAGTTTTATGTATATTATGGTATGACAAGGCCATATATTGCTTATGCAGTTG
966	CaSNP2918	TCATCATAGGTGAACGTGGCATCTTTTTGCAGCAAATTTGACAGTGGAAGAGCTATCTTG[T/C]TGAAATCCTTGATAAAGTGCCTGTAAAGACCTAGATGGCCAAGGAAAGAACAAATCTTGC
967	CaSNP2919	AGTTAGTAATAACCCATAGTTCCACAGTGACTACCTAATTAATTGCTATGACCATTCATG[A/T]TAACCCACCACCCATAACTATATAGATCAATGAGTCTTTTATAATAACAAATGTATTTAA
968	CaSNP2920	TGAGGGACCCATCATATTCCAGTTTCACATAATACACCCATTTGCAGCGTATAGGTTTGA[T/C]ATAAGGAGGACAAGATACAATGTCCCATGTGTAATTCTCCTAAAGAGCCTGAAACTCCTC
969	CaSNP2921	TTGACTTTAATAGAGTCTTGTGTGAGTGACTTAAATTTGAAGGTAGAGAAGAGAAGAGAA[A/G]AGAAGGAATGGGCATTGATAGGATGTGGATCTTCTACGAAATTATATTTATTGCCGTGGG
970	CaSNP2922	AAAATATGTTTTACATTTAAAGAAATCCACATTAGAAATGTCATTCATACATATTATCAA[T/C]CGTAGGTTGCATTTTGATGTTAAATTAGTAATGTATCATCAATGACTTTTGTAATGTCGA
971	CaSNP2924	CTGATACCTGTGAACTGCTGAAAAGCCTGCATAATTTTGAAAATATGAGAGAAGTTACAC[A/C]GCTTAAGATGCAAGCAGTCACATGCATTCAATATCTATGCAGATATATCATTTGAGTGTT
972	CaSNP2925	ACTGTGTAGGTTAGTTTTGAATGCCTGTTCCAGCTGAGTCAAATGTTGTCTCAACCATCA[A/G]TCACGCGTTTCAATAACATTTGAACTGAAAGTGATGCCACTCCAAATTTTGGGAATAAGA
973	CaSNP2926	CTAGTTACTTGTGTCGAACAAAAGAAGTTTCAGTTTTATTTGCACATAAATAGATTCTAC[A/G]TGGTCCAGGGAACTACTAGAAAAAATTGCTGTCTCCACATAAAAAATGTCTCATAGCGATA
974	CaSNP2927	CATAACCACCATCGTCATAACCAATATCATCCTCAACTTTCTCCGCAGCATTATCAGCAA[T/C]CTTATCTTCAATAAAATCAGCACCTTCCACAAGTGCAATTCCACCAAGAACCCCCGCGAC
975	CaSNP2928	CAATTCAAGGACCAAATAGAGAATCTATACAAGTAAACTTCTCTAGTGCCAATGGTGGGG[A/C]ATAATATACTCATACAGTCATACTAATTGATTACAGTTATAGCATAACTAAATAATTAGA
976	CaSNP2929	TGCTTTGGTAATGTTTCAATTTGTCAGGGGACTGATGCAATTGAAGGAATAATATTTGAT[T/C]TGTCTCAACAAGTAGATTTACATGTTCGAGCTGACACATTCAATGTGATGACTAAATTAA
977	CaSNP2930	AAACAAATTTGTATCAACCAAGAAAATAAAACATCAAAGAAATAATACTGTAAAAAGAAA[A/C]GGGAAGCTTCGTCCTATAAAACTTGTGCCAAGGAACTATTGTCACCTTCTTACCTAGTAC
978	CaSNP2931	ATATTATCATTTTATCCAATTATACATGTACATAAATCAATTAATCAAATCATAATCATA[A/G]TTAATCATGACAATACTATATTCATCTTTGCGAGAGAGTATATTATGGTGAGAGATGACA
979	CaSNP2932	TCGAATAGTTTTCTTAATGTTGTAAGTTCGTCCAAATTTGTCTGTATCTTCCAAAAACAC[A/G]TAGTTGCAGACATACACATGGAAACAAGATTGTTGGTACATCATCATTGGAATAAAAAAT
980	CaSNP2933	TGATGTAACTAAATATTAGGAAAAAATGGAACACACATTTGTAGTGGCTAACAATTCCCT[A/G]TGGAAGAAGAAGATGGTCATGAAAGAGAAAAATATACAAAAGTCAGCTACTCAAATGAAA
981	CaSNP2934	AAAAAGAGTGGAAACTAGCTCAGATGGGTTACTTCTTAGCTTGTATGTTATCCCATGCTT[A/T]TCATTTGTTTCCTGAATTCTGATAGTAGTAAAAGAATTGAGATTTGACCATATTATTTTT
982	CaSNP2935	ATTAGAAAAATATTTTATAACACAAAATATTTTAAATTAACACACATTACAATTATGGGT[A/G]AAAATAGGCTAGATCGGCCTCGTCTGACCTATCTATGATTTGTTCTGACATGTTTACTAA
983	CaSNP2937	AAAAATATGTTGAAAACAGCTTATGAACATGTCATAAGTTGGTTCCATAAACTCTCTCAA[T/C]CAATCTCACAACTGCTTATGTCAGTAGATAAGCTCAAATAAACTTAAGTACTTATTTAAA
984	CaSNP2938	GGTGGTCCTGTTGGTACTGGCAAAACCGCTTTGATGTTATCGCTTTGTCAAAATTTAAGA[A/G]ATCATTACAGTCTCGCTGCAGTAAGTACTAGCTACTAATTCATCCTATTTTCTATCTTCA
985	CaSNP2939	CCTTGCGCGCCCCAGGCGCGCTTCAAGCACAATTTGGCAGATCTAGTGGTGCAGAAATGC[A/G]CCCAAGGCGTAGGTTTCACGCTTTAAGTGCACAACATCATCTCTTTGGCGTTTAGTGCAT
986	CaSNP2940	TTCCAATATATTGTGATAATACTAGTGTCATTAATCTATCTAAGAATCCTATTCAGCATT[A/C]AAGATCCAAACATATTGAAATAAAACATCATTTTATTATGGATCATGTGAACAAAAAAGA

987	CaSNP2941	CCTCGATCTTTGATTAGTGACGAGGGCACTCACTTTCTAAACCAGAAAATGGAACACCTT[A/C]TAAGGAAATATAATGTGCGTCATAGGGTGGCAACCCCATATCACCCACAAACTAGTGGGA
988	CaSNP2942	GTTGTCAGGTTGTTAGGAGAAGACTTGGCTTGCAAGGTCAAGGTGATTAGTTCCTTCTTC[A/C]ATTTATGGTTGTCAGGCTGTTTGGGAAGACTGACTTGGAGGGTTAGGTGCTTTGTAATTC
989	CaSNP2943	ATTAAAAGCTAGAAAGCCCTTGTAAGAAATGTGCAATTTGGGTGGTGGCTTTAACAACTA[A/C]GTTACTTGATGGAATGATGCAAATTTCATCATAAAACTTTCCGCATCATTGGTCCAAGGT
990	CaSNP2944	AAGTTGATGGAAAGTAAAGGATGACAAAATGAAGTTGAATGATAAGTAAATATATGCATA[A/G]GTTTTGACTTGTACTAGGTTGCAAAATATGACTCACTTGCAGCAGTTAGATGTGCTGCAA
991	CaSNP2945	AAATAATTACCAAATATTTAGCACAACTACAACAGTCATCGACATCCACTATTCCCCAGC[T/G]TCCTAAAAGCCGGTCCCTGTACTCCCTGCCATAACCTTCCAAATAATATTTGCATTGTAT
992	CaSNP2946	CTTCTTTTACAATATAATAACATAGTATGTTAGTAATTTAATAACGAATATAAGTATGTA[T/C]AAATATTATTGCGATAGCTTCCTATTCATCAACTAAACTAAATAGTTGTTAAAATATCCA
993	CaSNP2947	TCAACCGGCTATATTCAGACTGAGACGTCTTCTTCTTATGCTAGTACAGATCGATCTCAT[T/C]GTCCGTCGATAGATAATCTTGACCAGTTAAAGGAGCAATGGGCAAGTGAGCAAGAAGAAA
994	CaSNP2948	ACACATATCCATTACTTAGAATGATAAACAAAAACCAAGATTTATCCCATTAGTAGAGTC[A/G]GCTACATGGATCAAACGACACCATAACATTCTATCATATAACATATATCTTTCCAGCTCA
995	CaSNP2949	TATCTCAAATATGAATTTGTGGTAAGGTTTTCAAAATGAAGAATGGCAAGATATGCTCGG[A/T]ACTTAACATTGGTTTCGACGGCTGCAATTTAAACATTGTAGTTTTACAGGTCGCTGTAAT
996	CaSNP2950	TTCCTTAAGCCATTTACAAACCAACTTGGCATCTGATGATGTTAAAGTATAATTTGGATT[T/C]GGTTTACCCATTTTTCCATTAGATAAGAGTTGCATCTCCAAGTCCCCATGAAAGCATAAT
997	CaSNP2951	CATGGATCCGTCCAATGACATGGTACAGCGCTGTAAAAATGCTGATCGTGTGTCCAATGA[A/C]GATAACATGGAGATGATGTTTGTGGTTGGTGACGAAGAATTCTTGCCTATCAAAGAAAGG
998	CaSNP2952	TATAAAAGAAAATGAGGATGAATTGTTTTTTCAATAATAAGCCAAATGCGAAGAAAAAGA[T/G]GGTGAATGGTTTTGTTTATGATAAGATGGTGGAGTGTTGATCTTATATTAGCATTCGTTA
999	CaSNP2953	TCATTAAATGCATGCCAAAAATATTGTTCATCGAAGGCATGCTACAGTTACTGCTCATCA[A/G]AGTCATGCTACGGTTACTATTCATCAAAGGCAGAGAAACTCCAAAAACACGTTGAATCTT
1000	CaSNP2954	AAAATTGACAAGAACAACCTTTTGTCCTGGAATAGAATAACCTTTGTACTGAAATATACG[A/T]ATACAAACTAAAATATGCTCCTTCATATTGAGAACAAACAAGAAGGAATGGTGGCAAAGA
1001	CaSNP2955	CATATCAGGTATGGGCAACCATTTATCTATGCGTTGAAGATCTAAGCTTGTTATATTCAA[T/C]GAATCTCTTCCTTCTCTAATGCGTCCTGAGAATAGTCTATCATATGGACTTATATGAGAA
1002	CaSNP2956	CCAATTCTGTGTCTATACTTTTATATCCCTCTAGGCATTTCTCTTGGTTTTTTTCCTATC[T/C]GCCTCTTTATTTTGTTGCCACCACTAGGTAATTATCTAATCACTCTTTTCATAGTGTTTT
1003	CaSNP2957	AGCTAGGTATGAAAAAATGATACAAAAATAAATATGTAAAAGGACACGCCCAGTGGGACT[T/C]GAACCCACAATCGCTTGATTAGAAGTCAAGCGCCTTATCCATTAGGCCATGGGCGCTCAT
1004	CaSNP2958	ATTTTAAATTTTAATAACTTTCCATGCCTCTAAGAGCACTAACTCAAGACTGATAGTAAG[T/C]TATAGACAAGCTAAATTCAAGATAGTCTCATCTCTGTTTTCTAAGCTACTTTTTCATGCC
1005	CaSNP2959	ATTTCCAGGGTATTCTCTCATCATAAACTGAAGGGCAGGCATGTCCAAGTCTAACTTTTC[A/G]GCCAAGTGCTGATGATATTGTGCAATTCTAAGATGATTATGACGCTTGACCATGCCATCT
1006	CaSNP2960	ATCAATTTGGAGTTTTTTTGTGACATATACTCCCATTGGTCGCTAAATACATTCATCACG[C/G]GTGTAATTATGTAAAATTCAGGCGAGTTCACCGTTAGAATTGCGTCAAACTGTTATCACG
1007	CaSNP2961	GTGAGATTAGTAATCGAAGTGATGGAATTCATCAAGTCAAAGTTCCTCTTTCAGAGACTC[T/C]GACAGGTAATTTGGATACTGATAATAGCGTTGATGCTTCAAGATCAATGGCAACCAAATG
1008	CaSNP2962	AGGTCTTTTAATGTTGTAGAGGCCAAGGTACATGTTAGTCATTTGGGGTAGTGGACTTGC[A/T]ACTTGGAGTTAAGCAAACCTACTAATCATTTTAATAGTTCACCATGACTTGACAACTTAT
1009	CaSNP2963	GCTTACCAAACAGATAGGTCACGTGTGTTACGTATTGTTCTTCGATTTCCTTTGCATCAC[T/C]ACAATTTTCTTCTTTCTTCTTCTTCTTCCACCTCTTCTTTTTTAAAACCCTAACCCTATC
1010	CaSNP2965	TATTTAGAGATTTATCATTCATAATGAGTCCATCAATGATGGCATGACAAATAACATGTT[A/G]GCTTAATTACAACACTTTGATCAATCAATTTTAGCGGATTTGATTCAAGTGATTAGTCGT
1011	CaSNP2966	TAGCATAGTCATGTAAAATCTTCAACGATCTCATCTTAATGAAAACCTCAATCGACTTCC[A/G]ATGAATCCCGTTTTGTAAATAACACGAGAGCAAAGAGTTCCATGAAACAACATCTCTCTC
1012	CaSNP2967	TAAGATAATTGTAGTTTAATATATACTAATCAGTGATGAATTTCGACCAGACATAACATA[T/C]GTTAATAAATATGCTAATTAATATAGATATTGAGGTGCATGTTGTAATTTTAGGATCTAT
1013	CaSNP2968	GTAATAAACATATTTCATGAATAATGGTGTGATGATAAATTTCAGATATTTTAACAATGT[T/G]TTTATATTAATGTCATACCCAGGGGATTAGTTTGACTAGGAGAGACATTTGTTCTGTTAT
1014	CaSNP2969	ACAGAAAACTGAATGAGGATTGAGTTGAGAATAATAGTGTACAAAACAGTCAAGTTCAAA[T/C]TATACAACTGCAAACTGTATACTGCTACTGCATAACATTACTCCCTCAATAAAAAATTGA
1015	CaSNP2970	TCGAACGTACGGAGAACCACACAATCGCTTACAGAATCTTGTGAGTATTCTTTCCTATTG[A/G]AACTACATGGAGCCAATTAGGGGACTCTATTTTGCTTCTGGTTTAAAGAAAGCATTTAAT
1016	CaSNP2971	AAGCGTCCTAGAATTACACCCTCAATATGGCAGTTAAAACCACTCTCAATATGTTACACC[A/C]TCAATATAGCGGTTAAAGTCACTCTTAATATGACGATTAAAGTCACTCGCAATATGGTAC

1017	CaSNP2972	ACAATGTACCTTGAATTCCTAGTTTTCTCTTAGACTGAGACCTCCCTGGCTTCTTTTCAC[T/C]ACTATTATTCAAAGCAACAACACCTTCAAGTTTCTCTTTCAGATTTTGAACCTCTAAATC
1018	CaSNP2973	TCTGGATAGGAGGCAGTATATTGGCTTCTCTTGGTTCCTTTCAACAAATGTGGTTCTCCA[T/G]ATCCGAGTAATTATCTCACCCAAACATACGCCCCCTTCCCCGTGTCGCACGATTGTCAAA
1019	CaSNP2974	TAATTTATTTAGTTCACTATAGATATGAAACATTTGTATGTGTGTGTAGTTAATGTTGGA[A/C]ATGGATGCTTTGGCTAAATTCATAATTCTATATGAACAACCATTTAGGTAGCTCTCATAG
1020	CaSNP2975	TTGACAGTTGGCTTTCCTATCAAGAATCTCATTCCTTAAAATTCTTGGTTGGGTTAATTA[T/C]TTCCGTTTTGTATATCGTGCCTATTGAATTTCATAGCATGTGGAAATTGATTTGTATTCC
1021	CaSNP2977	CACAAACTAGCATGTGTGCACACAAAATAATCTATGGTGATCAAATGCTGCAAAATTACA[T/C]GATCACTCGTTCTTCTTTGTCGATAAAATTCACCCAAGTTTTGTTAAACAATTTAATGGT
1022	CaSNP2978	AGAATGTCAAACCCTTTTTAAAATCATATATTAACAATAAAGATTATATTAATGACTGCA[A/G]TGTAGCAGAGTGCACCAAACCCTATATCTCCTGTCTTTTGCTTGTTTTCTCCTTCTAATC
1023	CaSNP2979	GTATTATTTGAGAAAGATGGAGGTTGATCAAAATTCTAATAAGATGAAGTTTCCGGCTAA[T/C]TATACTGCTTTATGTGGTTTTGTTTCTATATGGTTAAGTGCTACTGGCATTGACACTGGC
1024	CaSNP2980	TCTTAATTGAGCAATTAGATTTATTTTCTTGTCAAATAGAAAAACTTAATAATTTTGATA[A/T]CAAAGAATTCAAGCCAGTATCCAAACTGCTTTGTTTAATAAGGTGATTGCTCAAGTAGTT
1025	CaSNP2981	ACTACTATATACGAGAACAACAAATGATAACTTGGCAAGGGTGACAATGTTAACTTTTGG[T/G]TCAATACTTGGTTGGACAATTATTTGGTTTCTATACTTAATATTGTTGAGGACAAGTAAC
1026	CaSNP2982	AGTCATTGACTCAATCAAACGTCTATCACTATAATTATTGACGTTTGTCAGCCCCGTTAA[T/G]TAGGTGACGTGTACGGTACGTGGTTGAGTGACGTATGACTCTCTTATGTGTGGGTCTAAT
1027	CaSNP2983	GTTATGGAAATTTAAATTGAAACTACCATACAAGCCAATAGTTACACACATCAATTGTAA[T/C]ACCATATCTACTAATATTTGCAGAACTAATGGATCCTGAGTTCCTCATACATAAATTGAA
1028	CaSNP2984	AATTCAAATCAAATTCAAACAACATCTTTTGATAGTCCTTCTCTAGAAAATTCAATGCTA[T/C]CACCATCCAAAATTGTGACACATTTTAGTGAAAGTAATAATAATCATGAAATGTATTGTA
1029	CaSNP2985	AAAACTTTAGAGTTTGTTCTGTACCAAAAGGCAACACTTTGGAACGAGTCGTAGTCATCA[A/G]AAGTAGCAAAATATAAGCTTTAAAGGTAGGCTTGAAGAATGACTTTAGACTTCAAAGGCA
1030	CaSNP2986	TAAGAAAATATCAAAGCGTAGCACAAACGAAAAAATGCCATACTTAATTATATCATAATT[A/G]TAACAAGCTGAAATAGTCTTAAGATGATATACCAAAATATCCAAATATCAAATACACTGG
1031	CaSNP2987	CGCTCCCCATGCATGAAAATGGTAAGTACTCAAAATTTTACTAAAAAATCTTCCCAACAA[T/C]TATCATAATTAACAAAGATTGTTATCCGAAATACAAAAGGCATGACTTGGGACTACCGTT
1032	CaSNP2988	ACATAAGTAGATACTAGATTGTTGATTACATGTAAACCTTATGGATAGATTACAAACCAT[C/G]GTTATAGCCAATTAATATGAATGTTTAATCTCGTACATTTTTTGTTTTTCGTTGAAATAT
1033	CaSNP2989	AACACTCTACAAACACCTAACCATTATACCATTCCACTCACACATACCCTCTCCACAACT[A/G]TCTTTCATCCTACCTCACCCACGCTTGTGATCCTTTTCACAAGCAGTTGAGATAGTAGCT
1034	CaSNP2990	AATACAATGCAATACTGCCATAATACAATACATGACTGCCTGATTATTTGATGCACTTGG[T/C]CCAACCTCCTTGAGTCTCCAATTATAATGTTTATTAATTATTAATTATTAATAAGTGGTC
1035	CaSNP2991	GACCAAACCGCTACATCGGCGATGTCACCTGACAACAGAAACTGATAAAACATGAGGAAT[A/C]AGAATCACTATATAGATATTTACCACAAAGAGATTGGCAAACAAAGGATATGCTTAAACT
1036	CaSNP2992	CTATTTATAGTTAAAATTTAGGCTTCAATTTAGTCTTTGTTTAATTATGAATTGTATCTT[C/G]TTCATATTGAGTTTGTAAATTCTTCAGATCGATTTTGTTCCTATTTTGTTTAGATAGAGT
1037	CaSNP2993	AAGGTGAAACTGACATCTTTTTGCAACAAAATTGACAGCGGAAGAACTATCTTGTTGAAA[T/C]CCTTGATAAAGCGCCTGTAAAAACGTGCATGGCTAAGAAAAGAATGAATCTCACGGATGC
1038	CaSNP2994	TCCTTAAAAAACACCTCAATGGTTTGGAGAGAGTGAGTGTTTATAAACTACACTTAATCT[T/C]AATTCTCAAAACATGTGAGACTTTTGGTGCACTGGAACAGATATAAAATTGTGTAGAAAT
1039	CaSNP2995	ACTGAATGGTATTGCCCTCAGATGCACCACTGGAAGAAGGACCAAATACAGGTCTTATCG[A/T]GGAATGGAATTCCAGCACCGAACAATTGTGTGGGTCGTGGGAGACATGTGGGATAGTCAG
1040	CaSNP2996	TGCTTACGTCCAATCATCACAATATGTTAGTTTTTTCACTAATGTGTTTGGCATAATAAC[A/G]CTCTTGCTTTCACACTATATTTTCGGATCACTCTTGATCAGTTACAAAATTCACCTTTCA
1041	CaSNP2997	CATATTTTGCTATATTTTGAACGATGTAATTGACACTTATTATTCATTAATCTCTTTTTT[A/G]GAATCATTTAGTTGTTTTACTTGACACGTATTTGATTGATGTTATTGTATAGTTTGAATG
1042	CaSNP2998	GTATGCGTAAACTAAATGTTGATTATGTATTTTTCTTATTTGTTTATGCAGAAGCATATT[T/C]AGTCACAAGCTACATAGAGTTCCCAAATTATTGGGAATGCACAATCTGTTGCACGAGAGA
1043	CaSNP2999	GTGAATGTGAGAGCTTTAAAATGTGTGTTAATAATAATATGAACATAATGACTATCTACA[T/G]AATTGTTTTCTCGAGGATTGTGTTATCCTATCCAAAAATCCAAACTAGCCAATCAGTGGA
1044	CaSNP3001	ACAATGCATATGTGACTTCATGTATGCAATGCCTATCTCATTATGACTTCAAATGCAAGT[A/T]ATTAACTTAATCCTTAACATAACAATATGTAAAATAGAAGATAACATTAGCTTCAAGTCA
1045	CaSNP3002	TGGACAGCGGCACCATAAGCAACAGCTTCATCAGGGTTGATGCTCTTGCATAATTCCTTC[T/C]CATTGAAGAAGTCACGCAATAGTTCCTTCACTTTGGGAATCCTAGATGAGCCACCCACAA
1046	CaSNP3003	GTAACTACAATAAGGTGACACTATCTTATCTTTGCTTAATGATGGTGAACAAAATCCTAC[A/G]ATAACAGAGAATCAAATAATAACAATATAGGTAATGGCAATTGTTACTTGTTTTATCTGA

1047	CaSNP3004	TCATGAGAGGACCAAGCATATTGAGATAAACTGTCACTTTGTAAGAGAGAAAATCGAATC[A/G]GGCGACAGCACCACTAGCTTTGTTAATTTCAATGATCAATTAGCAGATGCATTTACTAAG
1048	CaSNP3005	CATTTTATAAAATTATTAATAGTATAGGTGAATGAATATAGTGTTATGTATTTGTGCTTC[A/G]TAGGTGATGATCGTTAAATCTAAAGAGAATTTCTTTTATCTCTTTAGAAGTTATTATCTT
1049	CaSNP3006	TAGATAAGACACATCATCGAAGGTCACTACCATCTCACCAAATGATAGATGGAAGTTGTT[A/G]GTCTCCTTGTACCACCCTATGTAAACGCTGACAATAAGCCAACGTCTATTAATTCGTAGC
1050	CaSNP3007	TTTATGAATCTCGTCTAGAAGTACAATCATTTGTCCCTAACCATTGGCAATTGGTTAATC[A/G]ACTGTCATGACTGATTAAACGTCATTTGAACTCTAAATCTCTGATAAGTAGGAAAAATAC
1051	CaSNP3008	ATCTTTCTACAATAATGTAAAGATTTGATATTATCCTTCTACAGTTCTATGCAGTTGATA[A/T]AACATTTCACTAGCAATATCATTATGTAATTGCTAGACCAAAGTTTTTGGGATGTCATGG
1052	CaSNP3009	TGATTCCCACCAGGTGTATCTTGTAATTCACCTGTCCTGATTATAGTGTAAGGAATTCCT[A/G]AGGTCTTCAATACAGATTCATCTTGCTCAGCCAATTTCTTTGCATTGTTTTTCATCATAG
1053	CaSNP3010	ACTTATCAAGGGCTTTTATATATGTGTACTAGAATTTGGATTGTCTTCCGTAACCAATTT[T/C]GCGTATTCACTAGATTAACACATCGATAATTGTTGAATTGAGATTTAACGGTCTAAATTT
1054	CaSNP3011	ATCACATGCTCAACAGAAGCATGATGGTCTCACTAGATTGATAGATATTAAGACTAGATC[A/G]ATTGGTGTTAATTAAGGGTGTTGCTCAACCCCTCCCACCGCCTCAAATCTCGGTAATCTA
1055	CaSNP3012	GTTGTTGCTGTTGGAAATATTGAGAGTAATTTTGTTGCTGCTGGAATAGTGGTTATTGAT[A/G]CAAGTACTGAGGTCGAGGCGGGAACTACTGATGAAACACATGAGGAAATGAAAATTGTTG
1056	CaSNP3013	TATATAATATCATTAGAAAGTGCCCTTACCTCGACAGTGCTTTATTACACAAGCAACATA[T/C]GATATGCCAGCATAACCGATCCGACGATTCCAACAACGCCTCTAAATTTCCTTGTACCTT
1057	CaSNP3015	TCGCCCCGTCTTTATCTATCTATTTCAAATAAATAAACAATAGTGAGTCAACGATTCCTC[A/G]TGCCATCACAAAGTAAATCATAAGCATTGTAGAAATGATTTTGGAGACATATGTTGCCCT
1058	CaSNP3016	ACTAAAGACAAGGATTTACGAAGGCGAGATGCATATTCAGTAAGTTATTCGAGTTTATTG[T/C]TGTCATGAAGTTCTTTATCTTTTCTTATGTTGCGCGAAATCTTCAATCGAGGTATGAGGT
1059	CaSNP3017	GAGGTCTGTTCATGTGAACTACGTAGAGACACTGTCAATAAAGCAAAGTTTGTAATTGTG[A/G]TCATTATCAGAGTTTATCTCGCTAAAAGAGGTAATCTTATAACTCTTATTCATGAGCATT
1060	CaSNP3018	AAGTATACCAGTATTGCTTTTTCTTCCCATCGTTGGCTTTTCTTCCTTTTTGACCGTTTC[T/G]TTTTGGAGTATTGGCTTAGTCATTACATACCCACACACCATCTCGTTTTGCTCGAAATTT
1061	CaSNP3019	ACTTTACAAGCAAGAGGGTCATTGGAATTTGAAGAAAGGGGTGAAGTTCAGATTGTCCCT[A/G]GCAACAACACTAGTCTCATTCCAGCATGGATGCTGCAACATCTCACTAACTAGGTTGACA
1062	CaSNP3020	AATCATTATCATCGTTACGGAAATCAACCTCTAGTATAATTTTTCTAACCACAAGATGAA[A/T]CGTTATACCCTGAAAAGTTGATGATGCTAATTGTAATAATATTAATAAAATACATCATTG
1063	CaSNP3021	AACAAGGGTTCTATTTTTTTACTTGGAGAGGAAGCATGAGTTTGATCATCATAAGTGTGA[A/T]TAGATTGATGATGATGATGTGTTGTTGTTGATTCATGACTAGGAAAGTTCCAATAATCTT
1064	CaSNP3022	TAAATAATAACATAATCACTATACGATGCATTTAGCCTATTAAGAAAAACCAATCTGTCC[A/G]AACTTCTCCTATCTTACCAACAAGTTAATTCAATTTACTTCTTGTTTTAAGGTTATTTGT
1065	CaSNP3023	TTTAATGAGAGGAGTCCTCAACATCATGTTTGTGATAAGTCTTGAATAAGGATTTGTCTT[T/G]ATCTTTCATGATCTATTATTGATTATTTAGGACCTCATAAGAGATATTCCACATCAAACC
1066	CaSNP3024	GCACATACGCATATGATACGCTCCCAAAGACACACAAGTGATTAACACTAGGCGTCCTTC[T/C]ATTCCATGATTCAAGAGGGGTTTGGTCTTTAACATTTGAAGTCGGATTCCATTTTGCCAA
1067	CaSNP3025	CAGTGTCTTATATTATGCTCTATGTATTTTTAGTATATATTACAATAACGCTTTTAGTTT[A/T]AAAAAAATGATCTTAAAGGTACGTCATAATATCTATATTTTAGTAAAATAAAACAATATA
1068	CaSNP3026	CTTAGGTACTCAAATAGTAAGAGTACGAGTATTAAAGTTGATATCTACGAAGATACTGAC[A/T]CATGTATGAGTATTTTTTTTAAATTGCGTATATAAAGATAAATTCTATAGTACTTTACTT
1069	CaSNP3027	TGGATACATCAAGAACGAATATTTTCAGGAACTAGTTTCTTAAGTGTTGCGTTGTAGGAA[A/C]TCAGCACAGATTGATGTTATAATTACAAAATGAGTTTACTGATATCAGTTATTGAATGGC
1070	CaSNP3028	TCCTAAAATCCTTCTTTTAGATGAACCTACTAGTGCTCTTGATGCTGAGTCAGAGTCTGC[A/C]GTCCAAAGGGCCATTGACAAGATTTCAGCAGGCAGAACAACCATTGTCATTGCTCACAGG
1071	CaSNP3029	ATTACATTGATAAGAAAAATGACTCTCCAACAATGACTAAAGTTCAACTCACTAAATATG[T/C]ACACAAGTTCAATTTTGTATATTAGTGATAGGGCCTTTAAACCAATGTCTACCATTGATT
1072	CaSNP3030	AGTTGAAAACCCAACAACGAGCCTTGGTTTAAAGGCTTCCTCTGAATTCGATAAAAGATC[T/G]AGTACCTGAAGGCACATCTTGACTGCCGGATCCTTATTCTCTGTTCATTAACAAAATAAT
1073	CaSNP3031	TGTTTTGTCATCATCAAAGAGAGAGAGATTGTTGGAACCAAGTTCATTCCAGGAACAATT[C/G]ATCTATGTGGTTTTGATGTTAACAAAGGTATTTTGTGAGAATAACTTATTACACTAATGA
1074	CaSNP3032	CACAAGGAAATGCAATAGACAAGCATCCCAATTTCAATAAATTGAATTGCAGATTAAAAG[A/T]TGATGTCTACGTAAACTCACAGCAAAATCTTTGGGTACCAATTCCACATTGTGACTAAGA
1075	CaSNP3033	ATCAAATTACAATAAGCAGGTTCATTATGTCTCATCAATTTTCAATACCAAATCAAATCA[A/C]AATTTTAAAAATTAAGCCAAATTAAATTACAATAAAAATAAAGTTAAACCAAAACAAATT
1076	CaSNP3034	CTAATATATATTATAAGTTTAATACATTAATTTTTGTTAATAAAATTTTATTCCAATTAT[T/C]TCTTTAGTTGATCTTAAGAGTGAGGTTTTGTGATCATTGATGTAATATTTGTATACTTTT

1077	CaSNP3035	TGGACAGAAAGAGTGTTGGGTGACACAAAAGGTACTTAAAGTTAAATGCATGACTTCACA[T/C]TGACACGAGCATGTGTCGAAGAACAAACGGTTAAACATATTTGAAAATTAAAATGATGTC
1078	CaSNP3036	TATTGGAACACCTCCTACATTGATTGCTTACAGTGATGCTGATTAGACCGGGTGTCCTAA[T/C]GCTCGACGCTCTGTCACCAGTTGGTGCATGTTTCTTGGTTCTTCGTTGATTTCATGGAAA
1079	CaSNP3037	AATATTCAAATTACATGTTGTCTTGCTGTTAACAATTATGTGATGCCATAACTTAAGTTG[A/G]CACTCATTACTTGGTTGGTAGTTTTCCAGTCAGTTTGGGGTTTTTCACTTTTGGATCTGA
1080	CaSNP3038	GGGCCAATCTTTGAGGTATTACCCATTCCTTACCTTGTTCCGCTAATCGCGTCCATGCGT[A/G]TAATTGACCAATCATATCCATCTAGGCATCCTCTCTTGCAAACTTATCAGCGACGTGCAC
1081	CaSNP3039	CATCTATGAACAATCCTAAATTTGCCCTCTCTAACACCATGATTGGTTTGTTCTGCAGTT[T/G]CCAATTTGACGACTCTGGTAACAGCGCGAAGCTAGTTGAATTCTGTAGGGACCCAAAGTC
1082	CaSNP3040	ATTCAATTGTCCTCGGATGTGAGAAAGTATAGTCCTAACCCTACAACTCAGTTTTGTGAA[T/G]ATTGTGCAGGGCCTAATCTAAATTCTAAAATTAATAGAAGGAAGAACAAATCTAATATTC
1083	CaSNP3041	ATTCAAATAGCGATATACTCTGTTGTCATCTTAGAAGCCACTCTAATTTAGGTATAGTCA[T/C]ACATTCTATTATAAATTTGAATCATTAGAATTAGGATCAAGTTCTCATTTTTAATTGTAT
1084	CaSNP3042	CGTTGCTGCAGAAGCTTAGTTTTGGAGCTTCTACAATTTGCGGTGAAAGAGAGAATTAGA[T/C]GCACGATTGAAGTCAGGAACTTGAAACCAAACACATGCTTAGTCAATGAGTAGCATTAGG
1085	CaSNP3043	GTCATAAACTATATCTTTAAGAATATCATCTAAGAAAGTTCTTGCAAAGAGAAAGATCAT[A/T]CAATCATTACAAATCACTTATTCTGTATCTTCCAATTTGTTCTACACTTAGTAATATTTG
1086	CaSNP3044	CTGAAATGCTTATCTTTTCTTCCCTTTGATTTTGCTATTATGTGCATGCTTTAGACATTA[T/G]GGTAAGGTGAGGACTATCCAAGCTTTCATAGTTTCCTTAGATAACTTGGGCTGATTGAAT
1087	CaSNP3045	CCAACCCCTCGACATTCGTCTTCAGAGAGAACATTATATGACTATATGTATTCGTCCCCG[A/G]AGTCAAGCCCTTAACCTATCACCCTTGCTCCATTTGCCACCCTTCTTCCTCCATTATATC
1088	CaSNP3046	TGATACATTTGATTTCTTCTCAAATGAATGACACTTTGGTTGTCACAACATATGCAAACA[A/G]ACTCTTGTTTAACACCAAGTTCAGTGATCAATCATCTCAGCCAAATTCCTTCCTTTCACA
1089	CaSNP3047	AACTATATCCTTCTGATGTAGAACTTCCACAACATACCAAATAACCAACTATAAACAAAA[T/C]TAAGTAATGACATTCGTTATTTAGTTTATGAAATATAACAAATCAAAACTCATGCATTTA
1090	CaSNP3048	TTCTGACCATTGTGTGTTTGTTAAGAAATTTGTTGATCGTGATTATCTTATTATCTTGTT[A/T]TATGTGGATGGCATGTTGATTGTTGGTCATGACGCTAAGAAGATTCAATATCTAGACAAA
1091	CaSNP3049	ATAGACAGAGGTAATCGTCAATACCAGAAATGCTGAATGAGCTTTGAACCGAAGACTTAT[T/C]GAGTGTTGCCATAATCTTGCAAACAAAATCAATAGACAAATGATGAAACTGAAGAAGCTA
1092	CaSNP3050	ATTCTTCTCTAGCCTCTTCAGGATATTATCGTTAAAACCTTTATTCGACATTTTGACACT[T/C]TATCGTTAAGGTTCATAAGTATTGTTGTACCTCTTCAAAAACCTCTTAGGGTGAGAGAAT
1093	CaSNP3052	ACACAACAAGAAAAAGCTGAAGCAAAGAATCAGGTGCTTGAAAGTACAGAAGCCACTGCC[T/G]ATGAAATTAATGTGCAGGGTACAGAAAATTGTGAAGCGGCACCCCTCTCAGCTGACCAGC
1094	CaSNP3053	AGTACATGCATCTGAATCGTAACGCGGCTTTGGTGGTCCTTGACTAATACCACGTGTTCT[A/T]TTTTCCCGTAAAGAGACCATTGACTCAGGTCTCGGTTGAATCTCCGCCGTAGGATTTTCC
1095	CaSNP3054	TTTTTAAATTTACACACTAACATACTCTATGTTCCAACAATCCCTCACTTCAATTTAAAA[A/T]AGTGTTTCTGAAATAGCGTCAAACGCTTCTATAAGATCGTGTTTAAGCAGAGGTGTATCT
1096	CaSNP3055	GTATACATTGTTGATGTGGTGGTCAAGTCTCAGAGCATCCAATGCCATCTAGTGGAGCAC[A/T]AGTACTTATGAAGGCTATGGCTAACGCACTAGTACTTATGCCACCAATTCTAGGTAAACC
1097	CaSNP3056	TTTCTCTTTCGAAAGAGTCAGTTAAAAAATTAGATACAAATTTTATGAGTAGGGATATAC[T/G]AGATTATAAGGCCAAAACAAATGAAATGCTAAACTGCGAGAAAAATTCATTATTTAACCT
1098	CaSNP3057	TGGTTATGTTAGTAGAAAAAAAGAGAGAATAAAGTTGTAGAAACTGGAAAGTAAAAAGGG[T/G]GATAACTGCCCTCTGAAAGAGAGAATGTTGGAGAGATCTTAAAAAGAAATATGTGTGAGT
1099	CaSNP3058	CAAAAATGTATATATCCATACATAACAAAAACAAATATTATGTGTAATCCTATTTCATTT[C/G]TTACTGTATCTCATTCTTCACTAATTCTTGTGTATTGTACAATGAAGATCAGAAACCTCA
1100	CaSNP3059	AAAAGGGAAATCAGGTAGAAAAAATCATTTGCACCTATACAAAAGAACTCTTGGGGTGCC[A/G]CTCTCAATTTAAGGCATATTAACTCCTTATTCTGTGTTTGTCCAAGTTAGATCTAGTAGA
1101	CaSNP3061	TAGCCATTAACAAAACTGTGTCTGAAAACAGAGAACGTTATCCGTTTGTGACTGAAAACA[A/G]AGATCATTCTCCATTTGTGGTTGCCAACAACTAGTCTGCATCTGAGTTTGACGAAGAACA
1102	CaSNP3062	CTAATGTTGTCACATCTTAGTGGGATGCATTCGAGATCAAGTCCATAGTCACATAGTTGT[A/T]GCTTGAGCCATATAATTTGTGCACAACAGTTGTCTGCTACTAGTGTATTCTGCTTCAACA
1103	CaSNP3063	CTAACTCTGATCATTTTTCATTTGTCTCTGAATCTGATCACTATTCATATGCCTCTGACT[T/C]AGATCACTGTTCATATGCCTCTGATAACTGTTCATTTGCCTCTGACTCTGATCACTGGTC
1104	CaSNP3064	GGAGTAATAAAAAAATGACAAATGATACTTTTGAGAATAAAATACAAAGTATTATGCATT[C/G]AGGGTCGGGAATTAGCAACCTAAAAGCACAATGTCATCTTTTGAAAATAAGAATCTAACA
1105	CaSNP3065	TGGTATGATCGACCTGTGTTCTTATAGTTGGATGTAAAAGTGTACTATGTGTACAATCCT[T/G]ATGGTTAAAATACTCAATTCATCTATGTTCACATCGTCAAGCCTCCATAATTCCTTAACC
1106	CaSNP3066	GTAATGGGCGGATCCAGTTTTCTGTCCAGCCCTACCAATGACTACATGATAATTAACACC[T/C]TGTGTAGTCATGATTGTATCACACATCATTGACTTGAATTTTACCCAAACTAAAATTGAT

1107	CaSNP3067	TATTTTATATTATTTAGACTAATGAAAATTATTGTTGGTTTGGGATAACGAAAACGTAAC[A/T]ATAACATAAGAATAAAAATGAGAGTATATAACAACACAACTTTGAGTTAATGGAAACAAA
1108	CaSNP3068	TAGCCTTAATTACCATTAAGTCAAAAGCCATGTTATTTCACCTTTGTGTAAAACACAGAC[A/T]ACAGATTAATGTTGGATACTTCATGGACTTTTGAATACTACTTAATTGTATTTTTGTTTT
1109	CaSNP3069	TAGGTAGAGGTACAGAGAGACCAAAGAAAACGACAGACCAAACCATCAGAGATTTAGAGG[T/C]AAATAGGCTATCTTTATACTCCATACTTGGTAGGATTTTATTGCGTTGATTGACTATGTA
1110	CaSNP3070	ATTCCTACAGATACGACACAAGTTGCTGACAACGCAACATAATTAACTCCAAGCTTAGTT[T/C]CTGTTGGGAAAAACCAGAGATAATTAGCAATAATTATCTCAATAATGCGGAATATTTTTT
1111	CaSNP3071	TGAATGAAGATTGTTTTATTGATGGTTTCACATGACGGGGTATGCTAATCAGGGACCATA[A/G]AGGCACGGTTATGGTCTCAGCTTGCAGGAAGGAAGATATCCAAAACGAGTCCAGCCTTGG
1112	CaSNP3072	TAATTATTTAAGTAATTTGATGACTAAATTGAAATTATAATAACTCAGTCAACACAAACA[T/C]AATGGTTTACTTTATTTCAATAATAAATATCTAAATTTACATTTTATTCTAGCATATTAT
1113	CaSNP3073	TGTTCGTGTCGGTGTCAATGTTTCATAGGAAAAAAGAGGACTGGATCTATCTAGGTATGG[T/C]ACTAGGAAACAGTGACCATATAATGTTTTCAATCAATGCTTTAGCAGAAAACATATAGAT
1114	CaSNP3074	GTTGAAAATTCGAGGGAATGACTAAAACTGTCAACTTTTAAAATAGGAGACCAATTTCGT[A/G]AATCAATATAAATAGAGGGACTAAAACTGGAATGAAGTCAAATCTAAAATAACTTAATAA
1115	CaSNP3075	TTTTACCCTAGTATTTGAGAATAACAGTTCGCAACGGATACATATTCCACCTTGGTAGTT[A/G]ATAAGACAATGGTATTTTGTGTATTGCATGACCAATTGATGAGTGCTTGATCGAGAAATT
1116	CaSNP3076	TTGTAAACCTCCAACATGCTAACAAGCAATTGATATTTTACTATGCCTTGTCTCTCTTCA[T/G]ATATCCGAAATAACTCCTCCAGAGTTCTATAATTAACTCCCCTATGCTCGGGCGTTCCCT
1117	CaSNP3077	CCCTTTCTTGATTTGTTCCCTCTACCTCTCCCTGCCATGCAAACATCTGTTATTTCCTAG[A/C]TGGAATTTCCTCTCTTCCTCCACCGTTATTAGTTGGGTTGGTTCCCCAATCCTTTAATCA
1118	CaSNP3078	ATGTTATGAATTGGACACTGTTCAGTTTTAGTGGCCATGAGAGCCACAATCCTTGTTGTG[A/G]TTGATAACTTTAAGTGTTCACTGTGATGGTGGGATACATTTTTCAGTCTCAGTACTGGAA
1119	CaSNP3079	AGTCCTAATATTTAAACTATAGCTTAATATATTTTTGCATGTTTCATCTCTTTAACATAT[A/G]ACATTCTTTAAGTAAATCACCAAAATATTTTCCAACTATGTAAGACGCTCTCAATTTAGT
1120	CaSNP3080	TATGTGTGTGAGGTTTCACAATAAAATAATTGAATATGAATCATAGTTTAGTGATAAATA[A/G]ACAGGTCGTTCGTACGAAATAATCTTGTAAATTAGACTATAGTGTTTAAATACAAGTTAT
1121	CaSNP3081	GCGCCGAATACTAAATGTGTATTTTGACATGTAGCGATTCAAACTCGAGTTTACCAGATT[A/C]TGAGAATCTTGACTCTTCCACTAGACCTAACCCCGTTGGTAAGATCATGGCAATATATAA
1122	CaSNP3082	ACTAAGGATGTCTACAAATAAATTATTTTACTTATAGGTACCCAAATTATGTTGGTTCCT[A/G]GTTTCTTAGTTCTTAAAGGTATCTTGTCTTTCAATATTTTCAGCATAGGACAAGTTATTC
1123	CaSNP3083	ACCGTGCAGATTTTGATCACAGTCTCGACAGATTAACATTTTGTGATCCAAGCACTGAAC[A/G]TAAGCAAGATGACATCGACATGAGTTACACACGAGGGTCCGGAGGTGTCGTCCGGACAAT
1124	CaSNP3084	TATGGTAAACAAAATTTGGGTATAGCGGATAAAAGAAATAGGGGTATCAAACAAGAAATG[A/G]TATTGACTAGATTGTAACAAATTTATGATGATAAATTGATGTTTAATTCTATTTAGTTGA
1125	CaSNP3086	CACTCCATGTCTCCTCTCAAGACAACCTTGCATATCTTCTAACTAAAAGGCTAAACAACA[A/G]CAACTTTGAAAGAATCGTATCCAAGCTGGAAATTGAGAACATCTATTCACCAGCTTGAGG
1126	CaSNP3087	CTCATTGCAATATGATATATTATCCACACTCAATTTTTCTTCACTTTTTACACAATCATC[T/C]GGATGTGGATCTTCCCCACCAACCCTCTTTACACACTCTCTATCTCACTTCATTTTGGTT
1127	CaSNP3088	GATCTTTCATTTGATATTACATATAAAAAATCAGCCACATCTAATGTAATCACCTAAGAA[A/G]CAAAATAAGTGGAAATTAATATGCTGCATAATCTTATCTACTGGTATGATCACTTGTGAG
1128	CaSNP3089	ACTTCGTCAGCTTCAAATGGATCTTTATTGCGCGAAATCGGAACTCTCTAGGTACCAAAA[T/C]TTAAGCATAGCGGCCACGACAGGTCATGGTCTGATGACTAATGAATCTGTCGCGGCCGTT
1129	CaSNP3090	AAATGATGTGTATGATAGTGTAGCAATGTGACATGGCAAGTGCTAACATGTGGCAATAAG[T/G]GAATGGATACATGTCTAATAATGATATGGTATGCTAATATGGCTCGCCTTCAACCTTGGA
1130	CaSNP3091	GAAGATGATAATGATATACATGGTATGGGCTTCTACAAAAAATCAATTGGGCAAAGAATT[T/G]ATCCTAAGTTTGATCTTGATAAATACCTTGTTGAAGAGTGTGAACCTTATTTTGTCATGT
1131	CaSNP3092	TTTTTGATTGGATCTAATTTGATGAGAAGGGTTAGGAATTAAGTCAACCGGTTTGGTCAC[A/G]CTGTAATTATAAATAAATAAATAAAAATATAATGGGAGAGTAGCAGAGACAGATATAACT
1132	CaSNP3093	GTAAACTAAAGCCACTATTGGGTTGGGTTACAAAGGGGTGATACTGTTTCTTTTTTGCTT[T/C]TTCTAAGAAGATTTGTTACAATGATTACACATATACAATGAATCAGTAAGCATCAAATAA
1133	CaSNP3094	ATATTCTATAATGGACTATCATACATTACAAGAATGACAATAGATGCTACTGCAGGAGGA[A/T]CACTGATGAACAAGAACTTAAAAAAACATATGCTCTAACTTAAGACATGTCACAAGATTT
1134	CaSNP3095	GTGGACAAACCATAAATATGTCATGGTTCAAAAGCATGAAATTTACAGAACTCTGAAGCC[A/T]CGTTAATTCTATTAGAATGAACTCATTTGGTCACCAGCAGCATAGCATTCATAGCCGAAC
1135	CaSNP3096	TAGTATTAATATCACACAATTTTAAGCAACAGACTTAGCACTACCACTTTATTTCATCTC[T/C]AATTGATCCTTTGTGTACCAATTTCGCTAAAACAAGTATTTATGTTTCTGCATACTCACC
1136	CaSNP3097	ATTCATCAATAACCCATTAATTATTTCCTTCTTTTTCTTTTGTTCTTGCTTTCTACTATT[A/T]CATTTAATTGCTAGGATCTTTTCGTGTACCGAACCTCCATGCATTTGTCAAATTCAAAAC

1137	CaSNP3098	AATAGAAAGACAATGGCGTTGATCAGTCAATGCTTGGATCTTAGTGTGTATCCACATGCT[A/G]AAACTGAAATAGATGCTAACAAGATGTTGGAAAAATTAAAAGAGTTGTATTAACGAAAGA
1138	CaSNP3099	CGATGATGACCTGAGCGAGATCTCTTTAACAAGATGAAGCTCCACCACATCAAGATACTG[T/C]GATAGCTTCTCCTGCAACACGGCGTTCTCGGAGAACGTGGAGAACGGACAGGCAGCACGG
1139	CaSNP3100	ACACTTGTGAGATTTCACTAATGTTCAAACTGATCAACCTGTCACAGAGGATGATTACAC[A/T]TTGTGTATTATTTAGCTTCACCAAGCTTACAATCAAGTTTCAACTATTTCCAAGTGTAAC
1140	CaSNP3102	AGGTGATGTGTAATTCAAGAAAATTGAATTAGTTGTTAAATCCTTATAAGTGAAGGGACT[A/G]GAGATAGCTACCAAGTTGGTGGTGAACCAGGATAAATATGCTTGTGTCACTTTACTCTGA
1141	CaSNP3103	GAATTTTTTATCCTAATTGTGAAATATCAAAACATAAATTTTGATTCCAAGGCGCAGAGG[A/G]TAATAAAGATCGATGGTGTAAGATAATTAGCGAATAATGAATAATATAATATATAATAATTT
1142	CaSNP3104	AATGAAGTTAGTGTTGCACTCTGCCTGCAAACTGATCACCCAACTTTTTCATTGTAAAAC[A/C]TAACATCATTACCGGTATTATATTCTTTTTATTACTACCATGTCTTAAGTGTTAACAAAA
1143	CaSNP3105	TCTCCCTTTTCTTCTCCCCAAATGTCATATCTGCAACCGGCTTCATTTAATGTCTCCTAT[T/C]GTATACTTTTGGTGCATAAATATTCCTCTTGGCCCACAATAAACTGCATATCTTGAATTA
1144	CaSNP3106	GATAAGTGGATGGGTTCTATGCTAGAGGAGATAGAGTCCTTAGAGAAAAACTAGACATGA[A/G]AGCTGTTTAGATTACTAGAGGGAAAAGGGCTATTAGTTGCAAGTGAGTGTATAAGAGATA
1145	CaSNP3107	GATTTGATTTGGGCGTGAACAATTTTGGGCTTGTTCTTGGACCTTAAGAAGTAAAGATAC[A/G]TATAAGTCCAATTCAATATGTTTCTCAATCTATTAGAGCCTTTGACCTACCGTAAGAGTC
1146	CaSNP3108	GCAACTAAATGAAACTTTATGTTAATATACACAAAGTAAATGATTTAGAAACCGATGCGA[A/T]CTATATATGAGTTAATGAATAAGTCTCATTAGTGATATCACAAAGTCTCATAAAGTAATT
1147	CaSNP3109	ATACAAACAAGGAAAAGATTTGTCATTCATTGGTTGTATACACTTATTAGCAGCATCTTA[A/T]AAGCAGTAGCAATGTTCCAAATTAGTGGGATACACACAGAGGTAATAATGCCATACGCAA
1148	CaSNP3110	AGAAATAAAGCATGGTATAAATCGTCCTCATGTACAATTCAACAACAACACGGTAGCTAC[A/G]CCCAATGCTTTCAAATTGAAGGATTTAACGCACAACTCACCAACTAAATTCCATGTCCAT
1149	CaSNP3111	TAGTTCATTTGAGGAGTTAATTGATAAATGTTGTTGATTCCATTTCTTTGAGTTAATAAA[A/C]TGTTTATGTTCTTATTTCCTTGATTAAGTAGTGATTTTTCGTAGTTTTACGTGGCTTCGT
1150	CaSNP3112	TGTTTAATCAAGTTATGACTTTTATAGGCCATCAACAGTTCAAATTGTTAGAGTACGAAT[A/G]TACCATGGCGACATGTTTGTAAATAATCCTTGCAAATTATATGTTCAAGGTCAAGTTGAT
1151	CaSNP3113	TTTCTAATATATGGTGAATCGGAATATGCTACCTTGTTTCTGCCTTTTTACTTATAGATG[A/G]CTGTGATGATCAAAATTTGGATGACAATGCTTCCAAAATGGTTGCTTTAGCCGTTGTTCA
1152	CaSNP3114	CACCCTTGTACTTAACCCTAATATTTTATTCAAGTGCTAAGAGATTTTCATTTTATGAGA[A/G]CATAACTTTGTAAACACACACTTGTGAGAGTTCTTCTAAAAAACCCATAAACACATCTTG
1153	CaSNP3115	TGGATGCAATGAATCTGGGCATATCAAGCCAGAATGTCCAAATCTCGAAAGAGGAAAACA[A/T]TCGAGGAAAGATTCCAGACCCAAGAAGAAGAGTCTGATGACTACATATGATGATTCTGAT
1154	CaSNP3116	CACATCCCACTTGGCCAATCATCAATCAAGTAATGGCTGTGAACCTAAAGAAACCGATGT[T/C]ACTAACATCTTAGGGATTGGTGGGGTGACTCGGAGTGGTCGAGTATACACTCTTGAACAA
1155	CaSNP3117	ATAGATCCCTTAATTCAACCCGAGACACATATATGCGACAATCGAGGTAAACGTGTGTTG[A/C]ATGGTAGCTCTCGATATTTGGAGTGCTACCTCCAAGTCACCTAGGAATTCCCCACTTGAA
1156	CaSNP3118	ACTCCTGTTAATTGAACAAATTCAAGACATAATAAGTACTTGGATTAGTTGACATAGGAA[A/T]GTAAATCGTACATTGAAGAATTAGAGAAGTTTTCAACTAACCAAATAAAGCACCGTCAGG
1157	CaSNP3119	GTTCAGCACTCCGTGCTGCTATCCAATGTTAACAACATAGTATGGAAGACTATGCTCAAG[A/G]AAAATCTAGTCCCTTTTCTGGTGTCAAAATAACGTCTGAACTGGCTTACCAATTGTAATC
1158	CaSNP3120	TAAGTTCTCAATGAAATGAGCAAAGCTAGAGTACTTAGGCTCCCACCCTATTTCCTGACG[A/T]GTTTTCGAGTTGTTTAATCTCTTACCAAGAGGATCATCAGTTCCTGCAAACCAAAATTAC
1159	CaSNP3121	GGGTTCAATTCCCACGGAAGCAAAAACTCATCATTTTCTATAGAGAGGAAAAGGTGAGAA[A/G]GTCACGATTAACATTGTCGATAACAGTAAGGGTCGGGCTGTTACATGAAGCACCCATATA
1160	CaSNP3122	GAACATTCTTGTGGATATGTTTATATGTTGGGAATGATATTGTCACAAGGATTGTATTGC[T/C]TCTCAATCACCACCTGCACCACTTCTTGAGATGATCCTTGATCTTGGGATTCAAACTATT
1161	CaSNP3123	GAATGGGGAAAGAGGTTGAGGATGTGGAAGAGGGTGAGATTTCTGATACTGCTTCTGTGG[A/T]AGAGATTAGTGAAGAGGATTTCAATAAGCAAGATGTTGTTAAGGTGAATAATAATAGTGA
1162	CaSNP3124	TAGTTCAAACTTTTGTAATTGGGTTCATGTAATTATCACGTCTTCTATGCTTCCTATTCT[A/G]GTTAATGGACATCCTACTGGATATTTTTCTTGTAAGCATGGGTTATGGCAAGGAGATCAT
1163	CaSNP3125	CATTGTTATAGTCATCTTGTTAAAAGCAACTACTCAAGTTCCAAACATTTGTATTCAAAC[T/C]CGACAGTGGTGTTAGTGTGCCTTAAACAATTTGTGGTTGTCATATTGTGTGGAGAAGACC
1164	CaSNP3126	AAGGAAGCGATCATCATGTGAGACGTTACTGAGTGCAACTCTAGCGGCCTTTCCTTTGTT[C/G]CTTCTGTCCTTGTGGGTTGTGGCAGTTGATTATGCATTTTGATTCCAGCTGTTATAGCCA
1165	CaSNP3127	GGGTTAGCCTTGGTCGATCTCATGAGGCACGGCCATGGCATGCACACGTCCCTATTATGT[A/C]CCATCCCAATCATTAGTAGGTACAAGAATATTTGGTCAAGACCACACACCTAAATTTGGG
1166	CaSNP3128	TACTTTTGTTATCATCCAACAGAATGAACTTTTTCAAGTTGAGTTGTAGTCCTTTCATCT[A/G]CTCACGTTACTTCCAAAATATGTTAATTTTACTAAGGAAAGTCTAAAGTGTTTGTTGCAC

1167	CaSNP3129	TAGCAGCCTAAGGCCCCTAGGTGTAGACTTTGTCAGTTTGAAGCGGGAACATCCTCAAAA[T/G]CCCAACGACATGCAAATCGAACAATCTATAAGGTTCAAATTCAAAGCTAACAATAACTAG
1168	CaSNP3130	AAAGTGAAAGTGAAAGTGAAAGTTGAAGTGGGCCACGTGGTGTTAATGTATTATCTGTAT[T/C]CTGTACTGTAGCATCTGCGAGGGTTTGGAATCGTTGGATCATGGGTTCCACTCTACCATC
1169	CaSNP3131	TCGAAATGGTATGTCACGTCGTTCGCAACAAGGTTCAATCTAGAATGTTGTGCTGGAAAT[A/C]GGTCAGCTCCAAAAATCAACTTGTAGACATTTTAACAAAACCTCTACAACCTGATCGTTG
1170	CaSNP3132	GAAGCAAAGAGGATCGACATCATCACTGAGATTGTTAAGGAATTTGAACTTGTTTACAAT[A/T]CTTTGACTGATACAGAGCTTGTTGTTGTTACTTCAGTGGTGAAGTTGGAGTCACAGCATT
1171	CaSNP3133	TCCTTTTGCGAGAGAAGAAAATAATTTAAATCCTAGATTTTTGTATGCGCATTCATATTT[A/T]TACAAAGTGACAACCAATGATGTAGGAGCAGGAAAAGGGGAGTTAAGCCTGTAAGAGCTG
1172	CaSNP3134	TTGGTACGTGGGAGGCATCCATAGTTGGTACACGGTATGCAACTCTTGAAGAATTGGGTA[T/C]GAGGGAGTCATACATAATCAGTGCGTTATTTGAGTTTGTGGTATGTTCTAATAATATAAT
1173	CaSNP3135	ATTACTGAAATGCAAGATAGTACTGATATGTTTTCTCTTTTACTTTGTTGATGTAGCGTC[T/C]GTGACTTTGCTGACTGTGCCAGCAGTGTATGAAAAGCATCAAGATATTATAGATATCCTT
1174	CaSNP3136	AAATCCTAACTATTGAATCGGGAACTAAACAATACCAACCTATCAGCCTTAGAAATCTCC[T/G]GATAGTTTACCCTTTTGAATGTGCCGTTTACATCACCACTAACAACTGGGTTCATATATC
1175	CaSNP3137	TATGCATATCATATTATGTAGTAGAATTAGTTTTGCTTATTGCATTTAGCTTATAACTTA[A/T]ATAAAATTGACAACTTGTGTGGTTCAAACACATGTATCACCACCTCATGCCTCTGTTCTC
1176	CaSNP3138	ATATTAAGTCTAAACTCTGTATAAACATCATTAAGTGTAGCTCATTCACAATCAATTCAA[T/C]GAGAATCTGTTTCATAGATGTCCAAGGAATTGAAAATTACTTTTATCATAGATAGGATGT
1177	CaSNP3140	AACTGAAAAATATTCACATTACATTCAATACGATTTGGATCTATTCAAATATGAGAGAGT[A/G]ATATTCTCGTTTTACTATTAAAATATATTCAATCTTACTTATTAAGTTAAAATTTTTAAT
1178	CaSNP3141	ACGGCAATGGTAGTTTGGACCAGGAAGCTGCGTCGAATAAAATGTCACTATGCGAAGAAA[T/C]TGAGAAGCCTGCAGTTAACAACAGAGGAACCAGCTTAATAACAGTTCTACTTCCCCTTTC
1179	CaSNP3142	ACTTAAAAATGAATAGCCTAAATAATGGAAAGACTAATCTTGAGAAGTACATTCCTGTTT[T/G]TATCCGAAATTCAGTAACACTCTCAACTCAATTAAGGAAATAATATTTACTAAGAAGGAG
1180	CaSNP3143	GAGCATGAATAAAAAGAGAAATAAGAAATGAGAGATAGCACACAAGAATTTATATTGGTT[A/T]AGCTACTAATAACCTAAGGTCTATGCCATGTAAATTAGACATTTCATCTATTTGATATGC
1181	CaSNP3144	CTAATTAGAGTGTGATCGCTAACAACTAATTGATTAAGAATATGTAAAGGGTGATCAATC[T/C]GAACATGTGATTAGAACAATAAGAAAATTGAATTGAACAATAAAAAGTAATTAAAATAAA
1182	CaSNP3145	TTAAGTTTGTAGTTGTGATTAACTCATATGTAATGACTAAATTGGACAGGATAGCCCAAA[T/G]GACTCTTCAATAGAAAATACCAAATATGTACAAATGAACTCCAAATTCAAAGTTTATAAA
1183	CaSNP3146	CAACAAAAGATGTTGTTTTTTCTATTCACGAAAAATGTTGCCAACGTTCTAAAGATAGTT[A/G]TTGTCACGAAAAATGTTGCCAACGTTCTAAACTGTTCACCTTGTTCCAAAAGAAGGTGAA
1184	CaSNP3147	TTGATCTTGTAGTCGCTTAACTGCGACAATATTACCATCTTTCAACCGACCTTGGTATAC[A/G]TAACTACTTCCTCCTACTCCGATCAGGTTGGAAGCTGAAAAGTTATCAGTGGCACTTTCC
1185	CaSNP3148	TTACCTGTGAAACATCAGTAGGAGAAGCAGAAAGATAAGACAAATCGACAGAGTGATCAA[T/G]AGTAGTATCATGATCGCATCCTGCCACCTGACAGCCAAAAAATTACCATTCATTTTCAGA
1186	CaSNP3149	CTTCCATTTTTAGTATAACATGTGTAGGTTATTATACTTGTTTTTGGTGTTATGATTCTT[A/G]ATGATTATTTATGGTTTTTGATACTTGATATTTGAATCTTGGTGTCTATGTTATTATTCT
1187	CaSNP3150	ATTTTTTATTTGAAAATTAAGCTTTGCAACATGATTAACTTTTAAAATTTGTGCAAAACA[T/C]CATAATATTTAGAGTCATTGGATAAAATGAATAGTAAGAATTATATAAAACAATTAAACC
1188	CaSNP3152	ACTAGTTAATAGTAGCTAAAGTAAATACTTCCTAACCCACAGAATCAAAATCCCAGTACT[A/G]ATGGCCAGTGTAGTCAATCCTACTTAGCAGAAAATAGCAGCTTGTTCAAATATGCGTTAC
1189	CaSNP3153	AATTTTGAGAATGCTTCGACATATGCTTCAAATGATTTCGACACATATACATATGAGTGG[A/G]ACCGAGTTGATGTGATTGCAAAAGCAGTTGAAGAAGATCTTCGGAATTGTCCTAAAATGT
1190	CaSNP3154	CTTTTTCAATTAGTAGACATTTTATCACGTCACTTTTTTTTGGATACTATTATAAATCAC[T/C]ACTGAGTTGTTAAAATCTAAAACTCTAATATCAAGTTAAACTCTAATGAGTTCATATTAT
1191	CaSNP3155	TTATAAAAGAAAAACCAAATATAATAAAATGATAATATTTTTTTAACCTAAAATTAATCT[T/C]AAATGTTTCAGTCTTATTTCCTAAAAAACCTAAATCGCATCTTCTTCTTCTTCATCGTTT
1192	CaSNP3157	GTATATACTTATTTGTATTGTTTGTATAACTCATACCAGACACAATGAAAACATGCCTAT[A/C]CTAATTAGTAAACATACAGAAGATGAAGCATGTGAGACAATGAACTAAGTATTCAATGCA
1193	CaSNP3158	ACCAATGTAGCAAGAACGATAAAATAGAGAACAGAGCATAAATTCATTTAGAGTTGAAAA[A/C]GATGATACAATGCCCAAGCTGCTTCAAAATGCCTTTGTAAATGGCAATATAATGGATCCT
1194	CaSNP3159	TTTAAGTAATGATTTGGATAGAAAATTTGGAGCGTTACATTATGGTATTAAAGCAGGTAT[A/G]TCCAATCAGACCGAGTGGGTCAAGTGTCTCTTAAGTGTCAATAAGATGTTAGTTGTAGTC
1195	CaSNP3160	ATTACCATAAGTTAACATTTATACAGAAAGTTCCATGCCTCACTTCTACTAGTTCAAGGA[T/G]AGGTTCTGCATTGATGAAACAGCTATTATGTCTGAATAACACCAAATTTCAAATTTAATT
1196	CaSNP3161	ATCTTACAGGTTGCTTCCACTGTCATCGACTACGAATTCTCACTTTACGGCCATCAAAGA[C/G]ACTCCGATCAGTATCGTCAGACAATTTCCCAGGTAACAGCCATCTCTTGAATCATTTCGT

1197	CaSNP3162	TTAATAATCGAATATTAATTTAAATTTTTGATTGAGTTATCTACACTTTCGAAGAGTAAA[A/G]ATTAATATGGTGATTTATCTGTTGTTATTAAGTTAATATTTTTTTGTATATTCATTCTAT
1198	CaSNP3163	TGATTGATTAGTGAAATGAATATATTGAAACACCAATGTCGAACACAACAGTAACACATT[A/G]ACACCGATAATAAAAATCATTGAATGTAACTACTTGAGGTAGACACCAAAGTCACCATCG
1199	CaSNP3164	GTTTGTATATGTGAATTTAATTTCTAAAGTTATTGTGACCGATATAAGACAATTGTTCGT[A/C]TATAAGACAATTGAATTTAATTCCTACTTCAATTAATTTGCTATATCGTTTTCATATTGA
1200	CaSNP3165	ATTAAGTGTCTCGTTTCTATAGTTGGTCAACCTGGTAGCTAACCTACAGGAGATGTCTAC[A/G]AACTTAACACAACAAAATTTTATGGCTACTTTTTTTAATATAATATGGTGTGTGGGTTTA
1201	CaSNP3166	TTGTTAGCTCTTTTGTAATTGAATGGTTGCCTTATACAATCTATTTATGTAGTACTCACA[A/G]CTCTAGAAGATGCAGCTGGAGTGACTCAGGCAGTGGCTGGAGAGGTACCCATCCCAATCG
1202	CaSNP3167	TCTATTTGTGCTTGCAAAGGCACATCTAGTGGTGCCATTTGACAATAATTAGTTTTGATT[A/G]TCAGTATGTTCAGAAGGAAAATGTCAAGAATGCTAATCGGAAAGACTCGAAAGACAAATT
1203	CaSNP3168	GGACTTGGAGATTTTGATATAACTTGCAATGATTTCAACAATGGAGTCAATAGCGATGCT[A/G]TTAGCTCTTTGACTAGTATATGGCCTTAACTATTAGATTAATGGTTGTGTCTCCCTTGTT
1204	CaSNP3169	AAATAGAATAAAAAACAATATCACTAAGAGAAAATGATAACTTCTTTTGCTAGGAAAAGT[A/G]ACATACATCACAATGTGACAAATTCATAGGAAAGATCATATAATTGTTCTTGGTTATATT
1205	CaSNP3170	GTTAATATATACTTTTTTTTTCTTTCAGGAAATGGATATAGAATCGCCAATAGATGGTAG[A/C]CGTTCATCTAAGTCCAGCCACCAACCTGATGATCGTGGAACAACATCATTAGGGACTAAG
1206	CaSNP3171	GAAACATGGTTGGTGCTTGATGGAAAACCCGCATATGTGAAACCACGATTCAAGGACGCA[T/C]ACACATATGTTGCTGCTGATAATGCAAGTAATTCCATAAACATTGCAGATGCCAACCCTT
1207	CaSNP3172	TCCCCATTCACACGAAATTTGTGAGAACATATTAACTATTCAAGCTAGTCCTATACAAAC[T/C]AGACTTAGATGGAAGAATGATGAGATGGTCTTGTTAAGATAAACTCTCAAATTAAAGTTT
1208	CaSNP3173	ATAGTCACTCGCTAAAGTAAGTTTTATAACTATTCAATAAAGAGAAATATTATTTTGGAC[A/T]CCGGTATTTGAACATAAGATTTAGGGCATTGTATGTAGATACAGTACACAAATTATTTAT
1209	CaSNP3174	TAAATGAGGCTATCATCTTGAAGGACCTTATTTTCTCGTGCCTTATTTGAAAGGCTAGGA[A/C]ATACCTCTACACATAGGTTAAAAATATAAGGAGACAATGGATCCCCTAGACGAAAACCTC
1210	CaSNP3175	CTCTGATGTATAATTCAGCATTCTTGATGGTGAAACAGGGTTATAGTTCCTAATTTTTTC[T/C]CCTTCATTCTTGATGATATACCTCAACTTCAATCTTATCTGCAAATTGTGTAATTTATTC
1211	CaSNP3176	TCCAGCGCAACACACATCTCTTCGTCAATCATCGTTCACAACATCATTCCTTCTGAGCGT[T/C]GGCTTCATTGTCGTCTCCTTGCGTTGTCGATCTCATCGTTACTACTACTTAGGTTCTTTT
1212	CaSNP3177	AAACTAATTGATAGTAACCATGGTGGAGGGACTCTTGTGTTTCGAGTTTTCGCTGAGAGC[A/G]GAAATCAAGGAAGAAGCGATCAAGGTCGGGCAACGTGGAGACCGAGCTCGTCTAATGATG
1213	CaSNP3178	CTCGGTTTACTTAATGTGCGTTTGAGGAAAATGTTTAAGTTAACTGGGCGTTGAGAATGG[A/G]GAATCTCTTAATGTCTCGGTTACTTAACTATTATTTTTGAAAATCGTTTCAGTAAATGAG
1214	CaSNP3179	GGTCTACAATACTCACATATATTGTTTATATCAACACATTTTTAGCTTTATGCCTATTTG[C/G]CTCTCAACCAAAGCTCGAAATTGTTTGAACTTCTATATCATATGGCCTTTTGTCTTCAAA
1215	CaSNP3180	GTTTTGCGTTGTTACTTTGTTTTAGTGCAGTGCTGAATTTTGGTGAGAAATCAACATGAC[A/C]TGTGTGGAGTAATTCATCCATATCTAGAGTTTTATATGTCCAATTGGAGTCAAACCAAGA
1216	CaSNP3181	TTGGTAACTTTTATGTACATAAATGATCACATTAACAAAAGAGAAGCCACTAGCAATTAG[T/G]ATATCCCACTAGCAAATAGTAAATCACCCACTATCGAATGTCGTAGATATTAAAGAATAT
1217	CaSNP3182	TCTACAAACCGCAAATTAGTCGAAATTAGACGCAACCATTACAATTTAAAGCAACTACTC[A/C]TAGGTCTAGAGGAGTCCAAGAGATGGATAGTTAAATCGAGAGGAATCCAAGAGATAGATA
1218	CaSNP3183	CTCCACTGATATGTCATCGGTCATCTGATACTTAATGAAGCATCTCACAATATACTTTTT[A/G]GCCCCAACTTCCTCGGTATCATATATCTTCTTCAAAACATCCCAATCATCTATAGCAGTA
1219	CaSNP3184	ATTAATAATATTATTTCCTATTTTAAATTTTATCAACTAGGTAATCACTTAGTAAGCATT[T/C]CATATTCAGTAGTTGTTCTATATTCGATAGCAATTTCATATTCGGTAGCCGTTCAATTAG
1220	CaSNP3185	GTTTCACCTACTTGGCACATTTGGTGGTTTTTCCCGATTTCCATTTGATTCTGGTGCTGA[T/C]GTTAACATTGTTCATCATGTTTTTGACTTGTGTGTTTTCTTCTTGTAGATTATGATATAG
1221	CaSNP3186	ATAAAAAATATTGATAAAACATATTTTTCAAGTAGTAACAATATATGAAATTTATAGTAA[A/T]AAAACAATATACCGAATTACATTGACATGATGTGACACCGTTAAAAGATAATGAGTCTAT
1222	CaSNP3187	ATGTTTTTCTTTTAGTTGTGTGATATGTGCTCATCACAAAAAAATTAGTTTGATTAATTC[A/G]GAAGCTTAGAACACATCCATTTCGATTACTTAGCTGTCAATACTTCAAAGAGGAAAGAAA
1223	CaSNP3188	ATTGATGAGAACTGGCTATGGCACTTAAGATTTGGCCATCTGAATTTTAGAAGCTTGCAG[C/G]AACTAGCAACAAAGAAGATGATGAATGGCATTCTTATGATTAATGTTCCTGAGAAAGTGT
1224	CaSNP3189	TTATATGGGTTGGAATACTCCTATAACTCCCATTGATTAATACTTCCTTTGCTTATAAAA[A/T]AATATACCAAATGTATTAGTGAGGTGAGTCAACAGGTTAATGAGGTGCGATGTAGATGAC
1225	CaSNP3190	GAACTGATCCAAAAGTCCATATACACTCTCATATTCACCCTGAAGAACATTGAGGTAGGA[T/C]GCAAAACTTTAAATGGCAAATATATAATTAATAATTATAATAATTTTTTTCCTATTTTAT
1226	CaSNP3191	CCTCTTATAAGCTTGACAAACAAGTGTTCAACTACTTTTGATAAGTGATCGTCTAACTCT[A/G]ATTTATAATGTATCCTCTTATGAAAGTCAATACCTGGAAGAAGTCTAAGCTTCGAGATAG

1227	CaSNP3192	GAATGGATATCTATAATTCAGTCTACCAGAAAGATCCTCAAACATAGTGAATACATCTGC[T/C]AATTGAAGTCCTGAAGGGATGTATGTTGTAGCTGTGAGACTACTATCAAATTTCTCCGGA
1228	CaSNP3193	GAGAATGTCGAAGAGATGACAGCCTGACTTGCAACTATTGCTGCCAAAGCAGCCACCACG[A/T]ATACCGGCCAAAAGAGGACATCTACAAAATGACAAAAATATCACTGCATTATCATTGAAA
1229	CaSNP3194	AAGAGCACAAAATTAGCATTACTCTGGTTTTCTATTGGACAAACATTCTATTATTTTCAT[T/C]AAACTATTAATACATATAGGAGTGGTGTAGTGCATATAATGAACAAAACAAGATCAACAA
1230	CaSNP3195	AGATGAAGATGATTGAGAAAAATAATACTTGGCAACTTGTAGACAAACCAAAAAATCAAA[A/C]GGTGATCGGTGTTAAGTGGGTTTATAAAACCAAACTCAACCTAGATGGTTCTGTAAATAA
1231	CaSNP3196	ATAAAACCAACTTGGTTCCAATAGAGATATGTACCATCTAATTATCACATGGTGCTACAT[A/C]AAAAACTACAAAGGTTGAAAAAGGCTCAAAGAGTGTGGAGGATTATTATAATAATTTTGA
1232	CaSNP3197	TGTCTCTTGGTACCAAAATATGATTAGGCCCTAGTTTGTTAGTCTTGAAAGGTTTCTTGT[A/C]TTTCCTTATATTTAACATGGCACAAGATAACTTAGAGTGACCTTTCGTACAAAATATAGA
1233	CaSNP3198	ATTAGGATGGTCATGCTCTTTCTTCGAGATATGCACTTGTACTTTTGATCGACCTATAGT[A/C]ACAAAACAACCTACCTGCGAAGTTGGGATGGGACTATGTGGTCATGACTGTGATAATGGT
1234	CaSNP3199	TGGAGAGGAACAAGTGCATAATCATCAAGACCATTCTAGACAAATTAAAAATGGTACTTC[T/C]ACAATACTAAAACCGTCTTAAGAAACTAACACACACAACTTTTACATACAATGGTGTGGA
1235	CaSNP3200	ATTACAATGCTACTAAAACTGAACAATGAAAGTTCTGCACCACTCAGCGGATTCCCTACC[A/G]AGCTTAGATCAACTGATCCTGAAAATTCTGCTGATAAGTCTTTACTCCTTATTGGTTCAG
1236	CaSNP3201	GGCGTCCACAAAGATGCATATGGTATGTCATGACAATGATAATAATGTTCGAAATGTACA[A/G]ATCACTAGCCACTTAGGCTACCACAAATAAAATCATAACACAAATAACCAGCCACTTAGA
1237	CaSNP3202	TATTCATGAAATAAGTGCATAATTCTCAATGGATATAGATTTTAGGTCATTTTGTTATTT[A/T]CTAGGTGTATTCTAGTCCACTTTTTCTTAATTAATCTAGTTCGTTTAATAAGTGCAAAGA
1238	CaSNP3203	AAACTAAGACACTAACATGATAAAGTGTTGAGAATCGTGAGATATTTTTCATATATTCTA[A/G]CCTCCTTAAAGGTATGTTTGGGTAGATACTTGGGATGGCTTGTACGCAAATGGATGACAA
1239	CaSNP3204	TAAAAGAGAGTGTGTTAAAATGTGTTTAAAGAGTATATTGCTAATATTCTAATAGTGAAT[A/G]TGGCATGACATGAACTTTGGACTCTAATAGTGAATGAAGGTGGTTTGGGCTTAAGATAGT
1240	CaSNP3205	AACCAGAAAGAGCAACAAACAAAGGGAACGACAATGGTGATGGAAGTAGGTAAGAGTAAA[A/T]AAGCTGAATCAATGCTCTTCAAACGTATCTGTTTATTCCCTCCAACCAATATTTAATTTT
1241	CaSNP3206	TTGTCGCCAGATGAAATTCATCTACGCTCGCTGCGCCACATCACTCACTCCCAATCATTT[C/G]TGGCTTTTGTGTAACAATAGATCTGACAACGCACGAGCAAATCCTTTAATATACTATTTT
1242	CaSNP3207	GTGACCCGTATTTTATGGTCATTACTATTAATGTTATTTATTGTTCTTTTTTATATTTGT[T/C]ATCATATTTTGGACATTAATGTTGTTCTGCAATTTTGTCTCCCAAGTAATATTCATAAGT
1243	CaSNP3208	AAGGTACCATTTACTTGCATGAACAAAAAAACAGCTTATCGTAGATCAGTGGCCTATATG[T/C]GGCTAAGTCATAGTGGAGGCGGACATAATGAATTTATTACTCTAAATACACTGGCATTGA
1244	CaSNP3209	GATGTAGACTAAGTGCAACACACCATATGATACGATGATTTCATTTAATAAAATGCCAAT[A/T]AATTATTAGTTGTAGATTTTACTTCACCAAAAATGTTTATTTGTCCCACAATTACGTCAG
1245	CaSNP3210	CGATTCTGTCCCTGAAATTGTAACATGTCAATAAAAAAGACCCCTTCGTTAAGTTCAAAC[A/G]TTAGAGAGCACGGTGTACATGTTAAACAAGGATGCGACACTTGAAGTGAAATGTCACATC
1246	CaSNP3211	AACATTTGATATTAAAATAAACAGTACATTAAGCATGCATTAGATAATCTAATTGAACCA[A/C]ATAAAAGTTTGACTAATGACAAATAAGAAAAGACTTACCTCTTGCCGTTCCCTGATTCAA
1247	CaSNP3212	TCTAAGAAAAATAAAATTATAACAAAACTCAAATTTCCAAAAGAAAACAATAACTATAGC[A/G]TCCAACAACAATCTAGGCGTCTTTCATCCAAGTTGACTCTAATGAATTACTTCCCTCATT
1248	CaSNP3213	ACTTTTTGTACTTTTACTCTTTTTCCATGGGTGAGGAAGGATAGAAAACATAAGTTCATC[A/C]TCTCTTCTGCATTGTTTATCCTCTAATGGTAGAGGTTATAATTTCATCCATGAGAAAAAG
1249	CaSNP3214	GGAGGAGAGAACTGTCAACGAGATCTAAGCTCCCCGATTGATGCGTGATGATGCGTTGCG[A/G]GATTGAGAGGAGAGTGCTATTTGTTAGATTGAGCCACCCCTAAGGGAAACATCACCCTTA
1250	CaSNP3215	TTATTCGCCAAGTTGAAGCAAGAATGATATAAATGGTGTCAAGATATGTAGAACGACCCC[T/C]ATTATCTCTCATCTCTTATTTGCAGATTAATGTTTCTTATTTTTTTCGTGCAACTAATAA
1251	CaSNP3216	CCTCTTCCCTCTCCTTTTTCTAAAACTTGCTCCTACTACTCCTAGTCCTTCATATATATG[A/T]GCCAAATTATCTACATTCAAAACTTTAACTTGTTCGGAAGATTGTGAAACTTCAGGCTTC
1252	CaSNP3217	CACCTCCCAATGCCATATGTGATTACAGTCTCAAATGCCCACTTTTTAATGTCTAATTCC[T/G]TTCTCTTATTATATACTTCACCACCTAACCCGCTAACCTACTCACTAACTAACCATGTAA
1253	CaSNP3218	TTAGTGATAGGTAAAGGGGAACAAATTGGGGAATAGGTCAGGTCGACCGACAGGGACCTA[T/C]AGACTAGCCTATGTCAGGCTCATGCTAATATTTCTTTAACAGACAAGGCTAAAGGCTTCT
1254	CaSNP3219	GTGATGGTATAGTTGCTGACAACCACCATGTGGATGAGCACCAAGAGTGTCAAAACAAAG[T/C]TGTTGCTCTTTCTCGATCCATCGACTTGGCAGAGTCTGCATTTGTTGATAAATAGGATAA
1255	CaSNP3220	ATTTGATGCAGCAGTTCCGTGTGATTGCTGCAGTGAGTAAAGCTAATATGTTATTTGGAC[T/G]TTTAGTCTATGATGCCTAAATATTACCAATGAACAGAATTTTTTGGACGAGGTTTAGTAA
1256	CaSNP3221	GCTCAATCACTTCCACCTGCAGTTTCATATCATATAATTCACCATGAACATGATGCAATG[A/G]AATCACAATTATACATAAATACTACAATGAATTTGGAACAATTTTGATGGCATTGATTAA

1257	CaSNP3222	TGTCTGACACCAACACGACACTAATTACATTTAATTAATTCATTTTCTCTAATTATTACC[A/G]GTGTTGTTCTGTCACTGTGATGTCTGGTGTCTGTGTCAGTATACTAGCTTCATTGCTTGC
1258	CaSNP3223	TCCTTACTTTACCTTACTGCTTTCAGACCTGACATCATATTTAGTTATGTGTATGTGCAA[T/G]ATTTCAAGGCACTACTAACATTACCTTGTTCTACAAGAAATCTTCTGAGTACAAACAAAG
1259	CaSNP3224	TTAAGAGTATACACAAGCATTCTATCATATCTCAAGGAGTCATGTGGATTTGAATGTTGC[T/G]ATTAATGTGAAGATTACATCGATAGATAGTATATAAGTACATTTTAAAGAGTTCTTTAAG
1260	CaSNP3225	TTGAGTTAAAATCACAAGATTATTGAGCTACCTAGGTTCAATTCAGGTGGTTGATGAGAA[C/G]ATTGTGGACAACAGATCCTAAGATTGACCATTGTATTGTTAATACTCACATCGTACCCAC
1261	CaSNP3226	AGATTACTCATGTATCTAGGTACTTCAGTCCACATAAAGTATAGATCTCATTAAGAGTTT[A/C]TATTATCTCATATTTTCATGAATTTCGGGATCATGCTTCTTTGATATATAATAACATATT
1262	CaSNP3227	TCATGATTACCTTAAGCTCGAAATATTTTTTTCTCTCACCACTTTGCATTCATAAGAATA[A/T]TTATAATCACATGCATAGCTTTGCTTGGAGTGCAAAGATTTAAGTGTGGGGGAATTTGAT
1263	CaSNP3228	ATATCAATTTATAAATTTGTTTTAAATGCCTTTTTAATTCCTCATTTTTCATTTATCCGT[T/G]ATTTTAATTTATTGTTGTATCAATACATTCTTGTATTTCTCGTGCTATGTTGTTTAGTTT
1264	CaSNP3229	AAAAAGGTGAAGTACTTGACATGAAGGGCACACTAGTCAAAGTGGTTGGAAAAGGTAAAG[A/C]AAAAGGATAAAACAATTCATGAGAAGAGGATCCACGCTAGAGGATGAACAATGAAAGGAT
1265	CaSNP3230	AAAACCTACCACGTTTCTTTGTGACAACATCACTATTTAGCTAAGACGCATATTTAGAGC[A/G]AGTTTAGGTGAGTAACTTAATTAAGCATTTATCCATTAAAAAACTATTCTAGGGAGCTTG
1266	CaSNP3231	GAACTAAATACAAGTACATAGTTAATGCAATTGATAAGAGGAGCCGACCAAGTAGGCAAG[T/C]AGTGAAGGTTTGAAGTAAATGCTATAGATCATCTTGGTATCTATTGTTCTGCAATTGACG
1267	CaSNP3232	AAACACATTTATCTTATATATTTTATTTGGCCTATGACTTGTAATGAGTACAGATCCATC[A/G]TTTTGACTAATGTCTTGCATGTTTTTTGATTAAATATAATATCATGATTATTATCACTTA
1268	CaSNP3233	GTGTTCATCATAACGCATGGAAAATTATATTAATGCAAACCATAATACGTCTTTACTTAT[A/G]AAATATGTGATTGTTGACAACATATTCTTGGAGAATCACTCAATCAATGAAGAAGACACA
1269	CaSNP3234	ACTGGCTTGGAGGGTCAAGTTCTTTGTAATTCAAGGTATTTGAATTAGTGGATTAAAATC[A/T]TCTAAATAAGGAGACTGGATGTAGCCAAGTTATTGGTGAACCATGATAAATCAATGTGTC
1270	CaSNP3235	TGGCAATGGCTCAACATCGACCTGAATCACCTTACTGATAGAAGTTTCTTTCTTTGATGC[A/G]GCCACATCTGTTTCAAGATGAACTGCTCATGAAACACAACATATCTACTTATCCCTATCT
1271	CaSNP3236	GCTTTCCCAATCAGAAGATTGATTATCTAGGCATAAGACATAAATTTGGTGATTAATTAC[A/G]ATGATAAGAGATTTTAACAAATCTATTTATAATGACAACCAGATAATCAATGATTAATTA
1272	CaSNP3237	TAATGCAAATCACTCCTAAAGTTAAAAGAAAACATACCAGTAAAAAATTAATTCAAGCTG[A/T]AATTAGATAAAAGTAGGGCTAACATCTTGCATGCCTAACCTCTTAAGAATAGAATAAGGT
1273	CaSNP3238	AAATTAAAAACTACATCGACTATGGTATTTCAATCATCTCTTTCAATAGGACAACCAATC[A/C]TCTCCTCCAATTTTTCTACCAAATGTTTGCAATATTCAACGAACTTGTAGACTTTGACTG
1274	CaSNP3239	TATGTCATAACGCAAGCCCAAATAAACTTTCCAAGTACATCTTTAACAAATAATTAATAC[A/T]AGATGTCATTTAGATATGCATGACTTGCTTGTTTTAATGTAGCGTCCACAGAAGTAGTAA
1275	CaSNP3240	ATTCTCCTTGAATCTTCTGAGTGTATCTTCCTTGCGGTTAGGGGTAAATGATCTTCACAT[A/G]AAATAAACCATGATATATGGTTTGGGAATATGATTGTTAGAGCTTGATTAATTCCAAAAT
1276	CaSNP3241	ATATATCTTATTATACATAACAGCCCAACACGTTTGACAATCCGTAAAATGTCATGTTTC[A/T]TAAGCTACAAACCTTAAGGGAGAACTTTAAAACCAAGATATTAGCTTTTTCACTCAACAT
1277	CaSNP3242	CATTTACAATGAAAGTTTTGTCATTTCTCATTGGCATGGATTTTAGACAGAGTTGCAGTG[T/C]TTCATCCATAGCTGATGTTGCATAACAGAGATGTGTCATTGTGGCATGTATATGGCTTAA
1278	CaSNP3243	CAAATTAAAGGAAAATGTATCTAGTTTGTAAATAAACAAAAACCATTTAAAAGGATTCCT[A/T]GGGAAGAAGTTATAAGCAAAATATTGAACATATGTCTATACGAATTTGAAACAACAACAT
1279	CaSNP3244	AAAATATTCATAATTTGCAAACAGAGTCTTGTTACTGCTGCTTATCTCCACGCTTGACTC[A/G]TGATATGGTGGTTTCAGAGGATATGCAATTATTATACCTACAAAGTGATGCATGAATTTC
1280	CaSNP3245	GATGAATGGCTGAAAATTATCTATTAAGGCTTGCAAATTTCTTATGATCAAGTGGTGTTC[T/C]TGAGAAAACTAATTGATTCATATTTGTCCACAACATATTTACCATTAACCAATCCGTTAT
1281	CaSNP3246	AATGTGGGAGATGCGACGTTGTGCTCGCCAACGACAAAAGGATTCTCAATCTACTCCACC[A/T]TTATACGAGACGGTACTTTTAAATGGATGTTCTGTTCACCAATATATGACTATTTGGTGT
1282	CaSNP3247	TCAGATGTTTAGGGAAATGACTAACTTCTGCACATGGATTGCGGTTTCTGTTGGGTTTGC[A/G]CAGAAGTATTCAGAACAAATTTTGATTCAACATGATCTGGATTTGGAATAAAACAAAGTA
1283	CaSNP3248	TTGTTATGGTCATTATTGTGTTAGTAGTGTAGAACTAAGTTAGTCCCTAAGTGTCTCACT[A/G]TATTTGATTGGCCTATCTGATTGATTTAGGTAGTGATGGCTAGTGGAAGGAATGACGCTC
1284	CaSNP3249	AGTACCCTTATTAGAGAAACTAATATATGGGCATTAAAACCTTCTCTAATATTTGCAACA[T/C]TATCAAAATAGATCAATGTAGCTGGAATGTACTTATCATACATAGGAATATCTTATAAAT
1285	CaSNP3250	TTGTGGGGATTTTTGAGTCGTGTAGGTCCGTGATAGACTGCACATTTTTAGTAAATCATG[T/C]TGACCTGTGATAGGTGGCACCTCGGTAAGTAGTACTGGTCTGTGATAGGCGGTACATTTA
1286	CaSNP3252	ATAGTAAAGCAAGGGAAGGATTGACTCACACAGTGACAATTTAATTTTAAACCAATTGGA[T/C]TCATATAATTGTATCTGCATAACACGAAACCATTAACTTTCTGGTAATCAATAAGCATCG

1287	CaSNP3253	TTCCTTCTCTTTCTCCTTGAGCATCTTAGAAGCTTTGCGTGCTGTTTGATTAGACGACAC[T/C]CCGTTTTGTAGCTGCTGTTTATCCAGAGAACCATAACTTCCAGAACGAATACCAATACCC
1288	CaSNP3254	AAATAACCTAGTACTAAACTTGGACTTGCTGCACCTATCCATATTGATCATAGGACAAAA[A/G]CTCCTCATTATATTAAAATACAATCCATACCTACAAACCTCTCCCCTCTGCCAATTATAT
1289	CaSNP3255	TTGGATAAACAGACATTGAACTCTCTTTATGCACTAGACAAATTGGAATATGATACATAG[T/G]TGGATATAGGATATGGGGTTGTTCTTTGACTTTATGTGTTTACCTGGAAAATATAATCAT
1290	CaSNP3256	AATAGAAATCACAGAATCAAAAGATCAAGAAAAAGAAAAAATCAAACTGCAACTTTTATA[T/C]TTGTTCCTCTCTATCGTTCTCGCATATAGAGTACATCTATTCCTCAGTCAAATAAATGAG
1291	CaSNP3257	TATTAGGAGATATATACTGAGATGCGTTTTTCATCTGCCCTAGTATCCTGTGGCAGAAAA[T/C]GGATTTGAGTTTCAGGAGTCGCTCGTTGAAGCACTATATGGGCTTAGCATATGGGATCGT
1292	CaSNP3258	GAGGAAGTATCTTGTTGAACCACAAGTGAGTAAGCCTTGCTTAAGGAGGGTAATAGATCC[A/G]TGAGTAGTATTTGAGTTATGGCATTGTTGTAGTTATCATTGAGCCTCTTTAAGAAGCAGA
1293	CaSNP3259	TTGGATGCACAAGATTGGTTAAAAATCCTAAATGGACTAGAATAAGCCTATGGAGGAAGA[A/T]AATGACCTAAATGTTAACCCAATGACAGAAACACACTTATTATATGCACAAGGTTGTTTA
1294	CaSNP3260	CACAAACAATGCTCACAAAATTCAAGATCACTAATCTTATCCCCACCTAGCATGCCTTGT[A/C]TGTCCATAATTTGCATCCCCTTTAGACTTACATGTCTCAGTCTTTGATGCCACATGCTGA
1295	CaSNP3261	CACTCGTTTAACTTTATATTACTCAGTGCTTAGTAATGGCAAAACAGAACCCAGTTTCAC[C/G]CTTTTGTATCTTAACCCAGTAATGGCTATTTAGGTTGGAACTTGAGAAGACTTGACTTAT
1296	CaSNP3262	GAAATAATTTAACAGAAACTTTTTTCTGAACCCCACTATAATAAACATCTTGAATGGGCT[A/G]GGCTTTTTATTGTTTTGGAACTCTAACTATGTGATTTTAAAGGTAACATACACGAATACT
1297	CaSNP3263	CATCATTCATTGTTATAAATGTATTCCACATACTATTTGTCGAATCACAATCTATAAGAA[T/C]GTGCATTATGGTTTTTAGAGCTTTGACTACCTGATGCAAACATCACTATCCGACATCCCA
1298	CaSNP3264	GGTCTTAGAGTTACATGATCAATATGGATTTTTTGTCAGAGGCAGATATCATTATGATGT[C/G]CATTTCATTTAGAGGGTTAGACTTTGATTTGTTATCGTTTGTCTTATATGATTTGACTTG
1299	CaSNP3265	AGCAAAAAATTTCCACCTTTCTAAAGAAGTCATAATATATATGCTTGCTTATGTGGTTCTA[T/C]TATTTAGTCTTTGAGCGACTATACATTTGTGATTCACATTTCTTTGTGGCGGATTACATA
1300	CaSNP3266	TATTCCCACATATGAATCATATTAGTGGCGCTTTTTATTCCCACCGATAATCAATTCAGT[T/G]GTGACTCTTTCACAAAAGAAATACACAAATAACATATTTTTACAAGGTAGATAGAAAAAT
1301	CaSNP3267	CAGGATGTTAGTATGTTGTAGACGGTTCTAGGAACAACAACCTGGAGAGGCTAGAGAAAA[A/G]TATTCAAAGCTTGAAGAGGTAGAGTAAAGAATACTTGTACGACTCTTTGTGGAAATAGTG
1302	CaSNP3268	TATTGAGTATTGACTTAGTCTCTATGTGCTTTGTAGTGTGATTAAAAGTTACAAAAAAGC[T/C]AGTAGGGGCTCAAGCCCATGGAGCACACTCAACATAGCTCTGCCCATGCAAGGTATGCTT
1303	CaSNP3269	GGCTAATGTCTGATGTTATTTTCTCAATACCTTTTGAGTTGGTATGTAATCTTATGTTTC[A/G]TTCTTGTGATTGTCTTCAGTGGTGGCAGTTGTGATATGGAAATGTATGATGCTTCCGAAA
1304	CaSNP3270	TAGAATTCAAAAATGATTCAAGTGTTAAAATAATGTGTCGATGAAGAATGACACTTTCTC[T/C]ATTTAAAATAAAATGACCATAGAAAATACAAAAACTTATATAAATTGTCAATTAGTTTTT
1305	CaSNP3271	GTTCAAAAGACGGAAGAGAAGAGATACTTTTTTAGTGGTTTTGTGTTTGGTTTAAGGGGA[A/G]ATATTTTAAATGATTTTTATGTTGTTTTGGTTCCCTCCTCTCCACTCACATCCAAATCCT
1306	CaSNP3272	ACCAAGAGAAAAACTATGGTGATCACCATGAGTCCTTCCTAATAGAATGTTTGGCCCGCG[A/G]GGGCTAACTAACACCAAACATAAAGGGATTGACTATTAATTTGCATTAAGTACTTTTTGC
1307	CaSNP3273	GCAAAAACTATGCTTCATATTTCACAAAGCAAGATCATTCTCCAAGTTGAGCAAGCTTCA[A/G]GTACAAAAGTGACAGACTGGACAAGACACATCAATTGTTCTTATGAGCAGCCTGCACACG
1308	CaSNP3274	ACGAATATAATTAGATAGGTGTAAAAGCATACTTTGGAAAAAGATAAGTTATGAGTAATA[A/G]CAACAAAAGAAGAAAAATAAGTCATGCTATTGGATAACTATACATTGAATAACACATTAT
1309	CaSNP3275	CACAATAATGAACCCTGTGCCATTGAATTATTTCAAACAGTTTCCTGACCATGTTTCCAC[T/C]GGGTTTGCATTTCTGAGTATGGACTTCTCAGGCACAAGGATGCCAACAAATTGCTCCTCT
1310	CaSNP3276	GAAATTGTTGGTAATGATCTTTTCTTTGCGACATCACAACCACGTGAAATTAATTCAATT[A/G]ATGATGATTTATATCTTAAAGATGATCATGATAAGGGAATTTGGATTAATCCACCGTTAC
1311	CaSNP3277	CAATGGATGAAAACTAACTACCGTAATTGTTGATACTTTGTGCAGTGCAACCTGCCATAT[A/G]TTACTATGGTGTTGGCCGACAATCTACAGGTCACTCAGTCTCACTCTCACTTTCACTTCT
1312	CaSNP3278	AGTTTCTATAGATTTTGCATCTTCAGGAATTAGGTGATGGAGAAGGCATACAAAAGAAAC[A/G]TATTGACCACGGTTGAGAGAGTCTCCAACAAACATCATTCTCTTCCCACGAAGTGTTTCA
1313	CaSNP3279	ATAATGTTTGAAAGCAAGAATCCACCTTGGAAGCTCTTTATATGATTCCTCCCAATTACC[A/G]TATATTCTCTCAATGGCCTTTTGTTTTGCAATCCATGTCTTTCTATAAGTAGTGGTATAT
1314	CaSNP3280	ATACAGATTACCATCGATATTATCAAATCGGTACAGAAATAACCCTGAAAGTCTAGATGA[A/C]ATCGAGGATTATCAGAACTTTATAGAATTCTTTACTTAAATTTATCACTGTGTTTTCTAG
1315	CaSNP3281	TCGAGTCTGGATCTAGCCCTGATAACGCAATAACGTTAAACTCTTGATGGAGACATTTGT[T/C]GTCCACGTGGGAGATTAGTCTCCGTAGTTGCATGTGACAGTTGGTATATACCAAAAAGGG
1316	CaSNP3282	TATTTAATTAAGTTGTTTATCCAAACAGTGTAAATTTAAATTCAAGACAAATGAAGAACT[A/G]GGCAGTCTGTTTGTAGATTTTGGGCAACGAATAACATGAACACTGTCATGTTTTCAACTA

1317	CaSNP3285	TGATCCTGCTGTGAAAGGAACCCTGAATGTTCTGAAATCGTGTGTAAAATCGCCATCTCT[T/G]AAACGTGTTGTTTTAACCTCTTCAATTGCTGCCGTTGCATACAATGGAAAGCCTCGAACT
1318	CaSNP3286	TCCTTTTCCTTTAAGTCTCTATTCTGATCATCTGAATCACCATCCTCTGATTATATTGTT[T/G]CCTTCAGAACATTCTTCTGGTTGGATTTCAAAGCCAAGGTTGGTTGTTTCTTCTCGGGCT
1319	CaSNP3287	CTTATATTATAAATACAAGTATTCTCTTAGTAATAATGGCTAGTTATAGTATCTTATATG[A/G]CCAAAATAATGTCAAACTACCGACCATATTTGGATGGGAACAAATAGTGAGAAGATACTT
1320	CaSNP3288	TCCCAAGATATATAGATAGATACACAGATAAACTGACAGACAGACAGACAAACAAAGAGA[T/C]CGGCAGACAGACAAACAAAGAGACCGACAGACAAACCAACAGACAAATAGACAGACAAAC
1321	CaSNP3289	GCATGACTCACCAACTCCTACAAAGGTTATGCTATATCTAAAGTCTTAAACATTTTATAT[T/C]TTGTGTAGTTTCTGAGGGAGCAAGCTGCAAATTCCGCTGCTGATATCACTACAGAAGCAG
1322	CaSNP3290	TATGAAGTTTTAGTCAGAGAACTTAGACTACATTACAGTGACCACTACACAGCCACAACT[A/G]CCATAATATGACATACATACTTGACCCCAAAGTTATGGTGACAACAAGGGACCTATTTTG
1323	CaSNP3291	ACTCTCTTGGCAACACTCATAGCTGCCACACGCCTTGGTTGGGTGCAACCTACTATTCCA[T/C]CTATAGTATAGCCATCCTCGTGTAGATACTGTTCAAAAACACAAATGTCAAATTCCATTT
1324	CaSNP3293	CATGGATTATTAAAATGAAAGTGGTGATTTTGAGATATTTATTCAGGTGCATATTTGTAT[A/G]TGTTGAAGTTATTGAGCTCTTACTATGAACATCCATGAGAATATAAACTTGATGTCTTAT
1325	CaSNP3294	TGCCCGATCATATTCCTCTTTCCTCTCTTCCACACTTCTCAGAGTGTTTCCTTTCTTTGC[A/G]GACTCATTGGATCCATCCAAACTATTTTTCTGAGGCCTAGGTCGAATGACAATTTTTTTC
1326	CaSNP3295	TATGTATTTATGCAAATAAATTTGTTTACAACATGAAGTGCCTGCGGGCATGTGGATTCT[C/G]GATTCATTGATGGAAATTTTGGGGACATGAATAGACTGAACCATGTGGCAGTGAGTATTT
1327	CaSNP3296	GTGTGAACTTTACAATTATTCATGACTTCTATTTATACGTCACAAGTCATTCAAAGTCGA[A/G]GTTAATAAAATGTGCCACTAACATATCAAAGAGCACTACTAAAGTGTGAAAGCCAATAAT
1328	CaSNP3297	ACATTCTTATATATTATTACTAACCAAATTTACTAACTTCTATCTCTCGTACCAGTTCCA[T/C]CACACATTTCAGAGCCACACATTACACAAAAAACTATTCATCGTCACCCTGATGCCAGTT
1329	CaSNP3298	TTAAAGGAAGTTAGTAATTAATAGCTATGGTCAAACTCAAACTCCCATACCCTCAAATTA[A/G]GATAAGCAATTTAAAGGATCCCACTCTTACGCATATGTATATGAAAGTTAGCGTAAATGG
1330	CaSNP3299	TTACCATATTGCAATAGTCATAATTTGCATTGCTAGTCTCCCACACAAATTATTGCATCA[A/G]TCTTATATACAATCACAATTACAATATAGAAGAGTTTGCATTTAAGATTCTTTGTAATTT
1331	CaSNP3300	AGATGGAAGGATAAAAAACATTGATATGTAGAACACAATAATCATATTCTTTGATTGAGT[A/G]CATGCTTGATTGATATATATTAAAAATAATGAATATGCCCCTCCCTTTCTACTCTAACTT
1332	CaSNP3301	GTTTAAGTTTAATATGTGTTGTTGAGCTCTAGCAGGGGTCTGTTGTGGTCAATCGACTCA[T/G]TCCATGATTTGGAAAGTGGAGTTCTCGTGTCTTCAATGAAATTTCTTAAATGTTGCTTTG
1333	CaSNP3302	GCACAAGGTGGAGTTGGAGCTTTCCAACATTTGCAATGACATTATGATTCTTTTAGATGA[A/G]CATCTTATTCCATCCACTAATGTTGCTGAATCCACAGTGTTTTATTATAAGATGTATGTT
1334	CaSNP3303	TAGGTCAATGAAGTTGGGATGTAAGTGAATTTTTGACGAGGTAGTAGGGATGGGAATAGG[T/C]TAAGCTGTCACGAGTTATGGTCTGACATGTTCTATTTAATAAAAAATTACGCTCATGCTA
1335	CaSNP3304	TTATCCCTTCATAACTTGCACTAATTTTGACTTGCATCTCTTGTAATTTATGCATACACT[A/G]AGGCAATTTTGTTTGGTCATTTGAGCCTTTCTAACTACCCTATGAAAATTTTATCCTTTG
1336	CaSNP3305	AAATAACCAAATTACATCATCTAGTACAAATAATCAACTGGATACATTGTAGTCACGATA[A/G]TTACATACTAGAGGTGATTTCATTGGTAAAGTTATTGTTGGAATCACTAGAAAAATTAGT
1337	CaSNP3306	CCACAACAATTTGGGTTGTGGTTTCAGAGTTTTTGATAACCCTGTTACCTCAATGTGATT[T/G]TAATTGGGACCGCATCAATTACATTTGACTGCAATTTTTTGCAATATGAATGATTACAAT
1338	CaSNP3307	TTAATTAAGATAGTAATATGGAAATTGGATCTACATTATAAGGTAAGCTTTTGAGAGTGG[A/G]ATACAACTACAAACTCATTTTGATATCATAGTAAGTCACCATGATCATATAAACAAACAC
1339	CaSNP3308	ACAGTCAGTAAGTGTATGAAAATGTAAGAATGATTGCCGGAACTGTGTAGACGGTTACCG[A/G]TAAAACTTGACAACATACGGTGTCCATTCAAGATTTAAGTTTCTGATAGCAAAATTGCCA
1340	CaSNP3309	GAATCATTTAATGTCTCATACAACAAGCTTGAAGGTTCTGTGCCAGAAAATGGTATGCTA[A/C]GAACTATAAATCCAAATAATCTTGTTGGCAATGCTGGCCTATGTGGTGGTGTCTTCCTTC
1341	CaSNP3310	TTGAGCAGTGTAGTCATAAGCATGATTGGGGCATTCGACTAGTAACCTCTTGAACCTCCT[T/C]TGAGTGTCACGAAGAGATTCTCCTTCTTTTTGTTTGTAATAGGAAATCTCTTGCCTTTTT
1342	CaSNP3311	TAATCCAATACTGTATGCTCATTCTAAGCATATTGAAATTGATGTTCATTATATTAGAGA[T/C]TGAGTACTACAAAATGAGGTTATTGTGGCTTATGTCCCTACATCAGATCAAATTGCAGAT
1343	CaSNP3312	GAGGAATACACACACCTTGACTCTACCTGCCAAGTCTTCTCCACGCAATCTGACAACCTC[A/T]GATTGTTAAAGGCACAATAACACCACTATTGGTTTGAATACAAAGGTTTAGAACGTAAGT
1344	CaSNP3313	TAATTATGAAACAAAACAAGACATGACAGTAATATGGAGAAAAGCATACCGACTAAGTTT[T/G]TGATGACATTAAACAGTGCCAAAGTATTTTCACATGTAATATCTTCACCAAGTAAGAGCT
1345	CaSNP3314	ATTCAAATTATGTCAAAACGCATTGGTAGAATTTTTTAGGAGACAAATTATTGGAACATA[T/G]ACTATGTTATGTTACTATCTGAATTTAAAAATTATTGAAGTCTTGGATTTGAAAACTCAC
1346	CaSNP3315	CAATACCTGAGCTTACAATCAACAATCGAAAGTGAATTCAAACTGTAAAGAGTCAATTTA[A/T]ATAGAATGATGTATATGAAAAAGATTGATTTTATGGGTAGTTTAGTTAAACTGTCTGAAA

1347	CaSNP3317	TCCCAACACTTTCCGGTTGCCATGAAGTATAAGGCTATCTAATTTGATAACAATGTCTTC[C/G]ATAAAAGACAATCTTAATATGTGGCCTAACTTCCTAACCTTAAAAAGTTAGGAAAGTGTC
1348	CaSNP3318	AACTGAAATAATACAAAACTAAACTAATAACGATAGATAGCTAGTTATGTTTAATATACC[T/C]CGTCAAACTGAAAGGGATATACAACATTTTGAGTTCGGACAAAAGACGTAAGTAAGGGAA
1349	CaSNP3319	TTAAATTTGTAAAGAATGAAGTTACATAAAAACAATTTACACGGATAGACTCAAAAAAAG[A/T]TAAAGTTGTAGAGAATAAAATCATATTTAAACTAATTATTTTTCATTTTTAAGGAGTAAA
1350	CaSNP3320	GTAAGTTACACTTGGAAATAGTTGAAACTTGATTTTAAGCTTGGTGAGGCCAAAGAATAC[A/C]CAAAGTGTAATAATCCTCTGTGAGAGGTTGATCAGTTTGAACATTAGTGAAATCTCACAA
1351	CaSNP3321	ATCCAAGCAGTGGCTATTCAACTGTAGGTCACTATTATAGATAATTGGACTTCAATAGCC[A/G]TGTCTCTACCAATAACAAATTACAACAGAATATACACAATATCCAGCAACTAGCACACAA
1352	CaSNP3322	TGTTAGAAGCAAACACTTAAAGTTCCAATCTTTTGTATCCAATGCAATAATGGTTTAGTT[T/C]CTTCTTCAATCTGGAGTAGTCGAGTTCTTAGGACAATACTTGGCTTGTAGGGTCAGGTGT
1353	CaSNP3323	AGTGTCCTAATTACTTGAAGCTAGTCAAATTAAGGATTATACATTAAGGTCCTTATGATA[T/C]GGAGTGATATTGTCAACACATCTGGTGGTTTCGATGGATACATACAACTATGAGGAATCG
1354	CaSNP3324	GAAGGGATTGGGCTAGCAAATAGTAGAATCAGTGTGACCTATTTCACATGTATAAGTGCT[T/C]TTGCATGAAGATATTTTTTATCACTCAATTCATTGTCTCAACCTAATTAAAAGAGGAGAG
1355	CaSNP3325	AAGAAGTATCCATGCTGCAATATCATTGACAGCAGCAGCTGACATAGCTATACGACCGAC[A/T]TCGGTGGTGAGGAGTTTGAGCTCAGCTAGGATTCTAGCAAGGACAGGAAATGCAGTGATT
1356	CaSNP3326	TACGGATTTTTTGGCCTAGTTTTGTATCACCCGACAAAAAGTACTAAAATCCGTATTTTT[T/G]GACCATGCGATGGCTAGTGTTTGAAATCTGACTAGGAAACCGGTACACATTTAAAAATTG
1357	CaSNP3327	GGAGATGATGGATATAAAGATGATATGAGATGATGTAGATTTAAGGAGAAAACACATAAA[T/G]CATGTAATAAGAGGCATAAAGTGTCAATTAGAGAGTTCTTTGCTTATAGAATTCAACAAA
1358	CaSNP3328	TTTTCTGTTTATTTCTATAAACTCTATATGAGAACTTATGGAAAAAGTTTGTAGCTTATA[T/C]GATAAACCCTTATGGTATATAAGCAGTTAATTAAATTGTTTATCGAAATAGGACCTACGT
1359	CaSNP3329	CTACGCTATAGCACCGCTACATAGCGGAATTTGAATAGCTTACTATTTTTTGCAGTCCGC[A/C]ATTGACAACAATAAGATATACAATAAAATTGTACTGTGTAGTTAAAATCTATGTAGCACA
1360	CaSNP3330	TTGTGTTAGGCCATTTTTGCTTGCTGGTATTCCTGTTTTCTCCATTCATCTTCACTAACA[A/T]TAAGCAAATATGATCCTGAGGGATAGATCTCTTTAATTTAATGATTTTATTAGCAGGGTT
1361	CaSNP3331	TTGGTGATATGTGATGTGTTTAGGGAACCATCAATACCATGATTGTATTACAGCACAGTG[A/T]GATAATAACATCATTTGAAAATAGCATAATTCAAAAGGTTCATCGACATAGTAACAGATT
1362	CaSNP3332	CTAAAACAAGAGAAAAGCATTGTGTAAGTACTATGCAACCAATAGTAATATTCAAAACAA[T/G]ATTAACCAGAACATTGTACACAACAACCAAATCTGTTTCCGCCTTATACTTTACAACACC
1363	CaSNP3333	ACTGTTGCACCTATTACTGTCTGGCAGCAATGATCATTATGGTCATAATATATTCATCTT[T/C]TGTTGCTGCACCTATAACTACCTATTAGTAATGGCCATTATGGCCATAAACTGCTCATTT
1364	CaSNP3334	GATGAAGGGTTATTTAGGGACACAAGACATTTTGAAGACAAACGACAGATATAAGTTGAA[T/C]TGTTGGGATGTATTTGAGAATGAGCAACATTCATCCATCGCATAACCTCCCACTCAAAAT
1365	CaSNP3335	GCATATGCATCTTTGTAGAAGTTGTCTTAGTGACATGTTTATTTTTTCATTGATATAGGT[C/G]GTTTTTTGTGTATGGAAGTTGCCTTATAGATAGATTTTATATTCGATTCATATAGTTTTA
1366	CaSNP3336	ACAAATTTAGGCCTAACTCAAGACTCAGTTTGTATAATCTGTGATAGTCAAAGTTCCATC[T/C]ATTTGAGTACAAATCAAATGTATCATATAAGAACCAAATACATTGATAATAGACGTCACT
1367	CaSNP3337	AATGACAAGATTTTCATTAGAACATGTGACCATAGTCCAACTACATGGATCAACTGCATC[A/C]CCATCCCAATTATCAAGAACACCATGCGGATCCATTAAAAAGTCTTTTATACCCATTAAA
1368	CaSNP3338	ATTAAAGGATTTGATATTTGATCAATGTGCATGAAAAAATATATGTCGATAGAGATGAGT[A/C]CTTTAAATATTTCTCAATACTTTAGATGATTAGTCTACACAGTCGTATAAAAATTACTTG
1369	CaSNP3339	TGCTGCCCACTCAGGTTGTTGAAGGGCTTCTTGAACATTTCTTGGAATATATAACCGAGT[A/G]CTGCTACAAATGCCCGATATTTTTGTGATAGTTTCCCATACGACACAAATCTTTCAATTG
1370	CaSNP3340	CTATAGTGCAATCGCTCACAGATCAATACGTTCTAGAGTGCAATCTGTCACATATCAGCA[T/C]GACACGATAATCCCCTTAAAGAAAAGATGAACCAAAAATAACATGGCATTATCCCGTGGT
1371	CaSNP3341	TTGGTTGAATTATAATAGATCGGGTCTCAAAAGAGTCTTTCCGTCCATCATCTTATGCTC[A/G]TTTCCTTAGGGACCACGTTGTTTCGATTGTTTGTTGTTTTGCTAATTTTATTGGTAATGC
1372	CaSNP3342	CCTACAACTACATCCCTTCCTAGTGAGATCCACAATATATTTCTCACTTCCATTATGCAT[C/G]AGTCACTCACTTCATAGATTTTATTCTCGGACAGCCTAACATTAAACATAATTTTAATGC
1373	CaSNP3343	ATATATTGGGAGTTGTTGACCAAGGACATTTCAAACTCACCGAGTAACGAGGTGCTTAAT[A/C]AATGTTGTCTGGAAATGGAGGAATCATTGAACTCGGTTTATAGGCAGTGTAGGGTCGCTG
1374	CaSNP3344	GCACTAACTCTGAAAATGCAACAAAGGTGAAGTTTCCCATGCCACTAATTCTAAGTCACC[T/C]GACTTTGCATATGCCTCTAACTTTGGTTGTTCAAGTATCACAACACCTGATTTCAATAAT
1375	CaSNP3346	ATCATATTGAGGTGTAGTATCATTAGATACGAGATGCGTCTAAGATGGAGTCATTTTATA[T/C]TGAGAAGACTCATACTATTGAGAATGGTTCAGATATGATGTCAAAAACTTTGCTTGTGTC
1376	CaSNP3347	ACACCCTTATAATCCTTGGAAAGAGTGTAGCTATTTTCACTCATATCCCATCACTTAGAG[T/C]TGTGTGACATATATAATGCTTAAACCTTATCGTTTCCAATAGTTAAAGTTTTTTCCTTTG

1377	CaSNP3348	TCTCAAAGAAAATACATCATAGATCTATTAGAGGAAATTGGGACGGGTGTGTGTAGACCA[A/G]CATATACTCCTATGGAATTAAATGCTAAACTTTGTGAGAAAGATAGTGTTCCTGTTGATA
1378	CaSNP3349	AGCGAATTCATTAATTAAAAGTGATTAGTCAAGCCATAACGGACAGATCTAGCGTAAAAT[A/G]TGATATGAATGTTTATAAGTAAACTAATATACTACTCATCACTCATCAAAGTGTAGCAGC
1379	CaSNP3350	TTCAAGGTTCATAAATGATATATCGGCTTATAACTTAACTTTGTCTCCTGTTTTTACTTA[A/C]AAATATCACAATTTTATTAGCAATAATAAAGTATCTTAGGATTCATAATAAGATTTGATT
1380	CaSNP3351	AATTTAGTTTTGGAAATGCAGTAGTCTTAAAATTCTTAGCGAGAGTTTTACTGTCCATCG[A/T]GGTCTTATCAGACTTGAGGGATTAGTCTCTATAGTTGTGTGCAGATGATACTCAATTTAT
1381	CaSNP3352	CAATCTCAACAAAGAACAAAGATATAAATATGTCTACATTCAACTGAGTGAACACTACAT[A/G]ATTATCAACCTTAAATTCATGTTTTTTTACTATATAAATAAATATTAATTATAATATTAT
1382	CaSNP3353	CACCACTTGGATATCCAACAAAGTACCCTTTCTTGGATCGAGGAACTAGCTTCCTTTCAT[A/T]TATATGATAGTATGCTGCATACCCAAATAGTTTTAGATGGTTATAATTAGCAGGATGTCC
1383	CaSNP3354	GAGGTAACTAATCATGTGGAATAATTATAGGAAACTTCAATGGTGCCAAGGGAGAAACCA[T/C]TGGTGATTCCAGATGAACATAGCTCAAAGGAAAGTTATCTTCACTAGATGGACCATTTGA
1384	CaSNP3355	TTTCTAAGCTCAATAAAAGAGAAACTTGTGACAACTTAAAAACAACAACAGTAATTATAC[T/C]AAATGAGACATGACCAAATGACACCAATGTTAAACTTTATGTAGAATATAAATTTAACTA
1385	CaSNP3356	GTTGTATATGTAACACAACTTTGTATAAATTCAACATCTAGTATGCAAATAAAAAATACA[T/C]TGTGGCTATGAGAAGTACTGCTCCGAGAATGTTATCTCCCTATGATCACATCATAAAAAA
1386	CaSNP3357	AAGAATATGTCACCGTCATTATTATGCGTTCTTGACGGTGAGGAGGTAGATTGTGGATAC[A/G]ATGAATAATAGTAGGACATGCAAATCCTAACATAGCAAGGAAAAAAACCTTAGATCTTGAA
1387	CaSNP3358	TGGCACAAACTCTCAAGGCAGGTGCTGAAAGGAATATGGTTGGTTGTGGGTCAGCATTTG[T/C]CATGGCAAGGCATGGATAATGCCGAAGTTAGATTGAACTGTGGCTCTTGATGATGTACTT
1388	CaSNP3359	AATTATGTTACATCTTTTTCTAAAATATGTAATAAATAGAGTAAAGATTCATTTTGGTCC[T/C]TGATAAATTTTGAGAAACTCAATTTAGTATTTGAGAAAAATAAACGCATTTTTAGTCCTT
1389	CaSNP3360	CCATATTATAGGATTCACCACAAAACACCACTAGAAAGGAACTCTACATGTCTCGTAAAT[A/G]CACCTCAGTTGGTAGTTCCAAGGACATTGATATGTAGGGGTTTGTGTTCAAACTCGGGAT
1390	CaSNP3361	ATTCCCCACCTAAATACCTCAAAGGTCTAACAATCTTGCCTTAAGGCTCAACTGCAAACC[A/G]CTGTGATTAGGCTCATTCAGTTGTACTTCTCCGAAATCACTAGGCTTGTCAGGCCTACCT
1391	CaSNP3363	CAATATGATTAAAAACTTTTGTTTCTGTTAGCACAAACAATGTTGTGACTTGTGAAGCAA[A/G]CAATTAGATAACACTGACCAATCTAGGAAGCATAGACAAACAGTCACACTCATGCCCTCC
1392	CaSNP3364	CACAATTACAACAAAAGTTATAACAATATTCAGTTCATTGAAATCGCTTTTAACAATTGT[T/C]CATGTACATTTAGTATAATATTCTTTTAAAAAATTAATCTAATTCAATAATTAATTTTTT
1393	CaSNP3365	AAACGTGATTGTTTAGTACAAATAATTTAATGATCAATACTATCATAGATCATATTATAT[A/G]ATTTATGATAAAGTTACCTGCCATAAATCATAAGCAGCATTAAGAAGAAAGAGTGGAGTT
1394	CaSNP3366	ATTGGTTGGCTTGAGTTGGAATCTCCTCCTTTTTGGCGCAATGAACATGAAGTTACCATA[T/C]GACCTAGTTGTTTGTAGTGGTAGTAGACTTGTTTTCCAACCAAGCATTATCCCCCATGCT
1395	CaSNP3367	GCCCTTATATGTTATTATTGTATTGTATTTGTACTATTTACTATTACAAATGATGGTGAT[A/T]TATTCAAGAATTCTAATTTGGGGGTGATTAGTTCTATAATTCTTCTCAATGTTTGTTTTT
1396	CaSNP3368	TTATGGTTAATTCTAGAACCCCGTAATTCTGGTATTTTTCATTTGTTTATGTTAGCCATA[A/G]CCCTGATTATAGATTGGAAGTCTAAAAATAAATTCATGACTTTTCCTCAACTATAACCGG
1397	CaSNP3369	GGTCAACTGGAACCTGCTCATCATGGTGAGGTACATGATCATCAGTTACATTCTCATCCT[T/C]TACCCGTAGATCTCCCCCATCAACAATGGTTTCTGCAGGGGGCTTAGGCGCAACATCAAT
1398	CaSNP3370	CAACTGCATTCCCTACTCTTCAAAAGTTAAACAAAAGAATAACCTAAGAAAGATGTTTGT[A/C]TCAAAAGAGTGGGTGAACACAAATGTACAAAAAGATCCTAAGGGAAAAAGAGCATATAGC
1399	CaSNP3371	AGGACATTTCATTTACGTCGTAGACTTAATAGAGCTACTAGTGTTAACGAGTTAGATTCA[T/G]ATACTGAAATTGACAACATGCAAAATATGGATGGTGTGAATAATAGAACCTTAAGACAAC
1400	CaSNP3372	CTTGTATTTAAATAATATGGTCCACTTTTACAAGATTACCCCTAAACCGTATTGCAGGGA[A/G]TTTTGCCCTGCTCCAACGATCTGTCCACTTATCGGTAAACGATGAGGCCATACCCAACAA
1401	CaSNP3373	GAGAGATTGCACTCTAGAATGTGATGATCTGTGAGCGATTTCACTCTAGAAAATTGTGCT[A/G]GTCTGTGAGCGATTGCACTCTTGTATGTCGTGCTGGTCTATGTGCGACTGCACGCTAGTA
1402	CaSNP3374	AGTAAAACAGTCTGTAAGGCCCAACACAGGGAAGTCTAGTATGTGTCTGATGCAGGAATA[T/G]AGGTGTAATGAGCAATTCGAAGGTGTAGGAAAGAAAAAGTGCAACCAGTAATAGCTCAAA
1403	CaSNP3375	AAGAGACTTAGTGAGGAGACAACAACAACCACCAACTAGAAGGGAAACACACAAAAAGCA[A/T]GAATAGTTGTATTACAATCTTCTGACAATTATACTAAATAATATTGACTGAAATATAGAT
1404	CaSNP3376	TATTTTAGATTTTTGTTGTTTATCTCAATTAAATTATTTTGTTGTTATATTTACATGTTT[T/G]GGATGGTCGACCTTTGTACGCCTTAATCAATCAATTGACTAGTGACCATGTACAATTAAT
1405	CaSNP3377	AAAATTAACAGTGAATTTTTCAAAGTTACCACCATTTCAAACATACATTTATATACAATA[T/C]CAATTCTTCAAAAGTTTACATTTCAATTTTACAACTTCGAGTTTATCAACAAAGTTTTCT
1406	CaSNP3378	ATTTTGGAGTTAGGATTATTTTGTCATCATATAGGGAGGGTCTGACAAACCTAGTGATTC[A/G]GGGAAGTACAACTGAATGAATCTGATCGTGGAGGGATACAATCGAACTATAAGGTAAGAC

1407	CaSNP3379	AGAATTAACTAATCAAGGCAAATGCATTATGACTCTGATCAACCCAATCTCAACGCAACT[T/C]TGGTGAATTTGACTAATGCAAGCTCACTTTGATAATTATGGTAATCCAGTTGATGTTTGG
1408	CaSNP3380	CTTTCAACACTGGGGGTAGGATTTTAGATGTATATAGAAGCTCATTATACCGAGAAATAT[T/C]GGAGGCTCTAATATGCACCCATAATTGGTTGAAGCCGTCTTTATACCATTTTAAAGACTT
1409	CaSNP3381	AAGAAAGTCATTGAAAATGTCAGACCGAACGTGACAAGAATAATATTATATGTTGTCGCT[A/C]TCACATAACCCATATCAGGTATGGACAACCATTTATCCATGGGTTGAACACCTAAGCCGG
1410	CaSNP3382	TACTGATTATACGCGATGATGTTTGATTGTGATCATTTACTTTGCACACTCTTCACCGTC[T/C]GATTATATCATTTGTAATTTTACTAACTTCTGCAATTTCTATCTCCACTTCACTTTCCTC
1411	CaSNP3383	ACAGATGAATAAAACTAAAAGAAAAATGGAATGAGCCGATTTTGTACATTGGTCCTCATA[T/G]CCCATAGTAATTGGGTTGGGTTTAATAATTTGCAACCCAATACTAAATAGTTGCATTTTA
1412	CaSNP3384	TGTTACCTCTATGTGAAGTAATCCTTGCAGGGCCCTATAGGTCACTATGAGCATATTCCA[T/G]AATTTCTTTTGAGCAATGTTTCCATGTAGAGAACTTGACCCTATGCATCTTTTCATACAC
1413	CaSNP3385	ATCAGCAACTAGCAAAACAGAAAAGAGAGTAATTAATTGCTACATAATTGAATATAACAG[T/C]TATGTAACTTCTAGCCGGTACTCTTATCATTATCAATGCTCAATAGGTATTTTCCAGTTT
1414	CaSNP3386	TTTTCAACAGCATCTCTTAACTGCAGTGCATAAACAATAAGTAACAAGTTCGGTATGAAA[T/G]ATTATTACTATTTATAAACAAAGGAATTGTCAATGTAACCTTCTTCAGTTCTGGACTAAT
1415	CaSNP3387	TTCAACACTCTTGCTCAAGATGGGCATATAAGTTGAATTATCTGAGCTTGGAACAAATAT[A/G]TTGAATTTGAGAAGCCTTAAAGGTTTTGTCAAGATATATGCAGGTAGATCGTTTGAGCTA
1416	CaSNP3388	ATGTCAAGAACATCCTAAGAAGTCTGCCTAGTAAGTGGGGACTGCAGATCAAAACTATCC[A/T]AGCGATCATAAATGTCTGTTATAAGAAAGGTCACTCTAAAATATCCTGTTCTAATCTAAC
1417	CaSNP3389	ACTTGAGATAAATAGGTAGACTTGCCTATAACTCTGGCAGATCCAGTGAGCTAGTGTAAC[C/G]GCTTCCGGAGAACCAGACGAAAGTTTTGCACCAACAGCTGGGTTTAGACCGGATGGAGAT
1418	CaSNP3390	GCCTCTTACCCATCTGCATTGAAGGTACCCAGGTTTGTCACATCTAATTTTTCGTATGAA[T/C]CAATTAGATCTTCATGTTACTATTTTTGTTACTTTTAAAATGTTCATTAGAGCATATTTG
1419	CaSNP3391	AGACTAAGTGAACAAAAATATAACGATCTAGTAGACATTATAAACTAATAAATGAATCAT[A/G]AGTATGATCGATCAAAGTTGACCTTCTTTACCTCATTTTCTAAACTAAATAGACCGAAGA
1420	CaSNP3392	TCTGAATCTTAAACTCATAATTCTTAATATAGAACAATACAAGTCAAATCTTTTTCCACC[A/C]GTTAGAGTCAGCTAATTGGATCAAACGACACCACACATTCCCCTATCATGAATATAGTTT
1421	CaSNP3393	ATGCTCAACCGAGAATTTGAAGAGTAATGTGGTGTGATTCAACTGCGATTCCCTTAAAAT[T/G]GAAGAGTCAAAGGAAGATATATTTGTCATGGCTTTAGAGAAATGTTTGAGCTCGAAGACT
1422	CaSNP3394	TTTCATCTTTTGTTACAGAACTAACTTATATGTAAACATTTCTATGATTAGTGCTTATGC[T/C]ATATGATTGTAATGTAGTTATTTATGCAAACAGGACCTTTATATCATCAAAGTTGGTCTA
1423	CaSNP3395	ATAGTTTTTTAAACATAAACTTCTCCACCAAATATTCGCTCGTAATGAGAATTTAATTCA[T/C]GATGCTCTACCACACGTTCTTGATGAATGAATGTCCAAAATTTCTCACTTATTCCATGTA
1424	CaSNP3396	TATATAGTTATCTGTTTTTGTGAATATGTAAATGTAAAAAATATGTAATTGTGTCAAGTT[A/C]TATAGTTCTGGTTATGTTGTGAATTTGTAATGTAAAGTTATATAGTTCTGGTTCTGCATG
1425	CaSNP3397	GAAGAAGTCTGATAGCATTAAGTTATTTGAAGTCATGAAAGCCAAAGGTCCGTCGCCGAA[T/C]GTTCACAGTTATACAATATTGATACAGGACTTGTGCAAACAAAAGATGATGAGAGAAGCT
1426	CaSNP3398	AAATGATATTTTGTTACAAAGATCTAACCTTGACAATTAACATTCGTAAACAACAAATCC[T/C]TATAATCGTCACATGTTTACCATAATAGGTGTGAATTAATGTGGGTAAAATAATTGAATT
1427	CaSNP3399	TGCAATTTGGATGATAAAGTACAAAGTACAATAATAACCATTGATAGAAAAGACAACACT[T/G]GAGATTGTAATAACTACATGCACATGGATAACAAATTATGATGTTCAAATCTAAACCATT
1428	CaSNP3400	AATACAAGAGTTTAAATTCATGCAAAATATAGAGTTGATGGCATGTGACAAATTAGTAGT[T/C]CTATGTTGTAAAATAGGAATGTTGTGGTTGCAAGTTGATTGCATGGAGCAAAAAACAAGT
1429	CaSNP3401	TCTAAGCTATCTTTATTTGTAATTTAAAAGATCAATACAAGATACCAACACACTATTAAT[T/C]TGCAATCTTTTGACGACATGAAATTAACTACTGCAAGCAAGCGGTACTCTTAACTCAATG
1430	CaSNP3402	CTCACACAAGAAACATCACTATGAAATATAGAGTTGTGTCAAACAAGAAAAATAAAACCA[C/G]GGCATCTAAAAACTATAATTAGCATATATCAATAGTATGAATCCTGTCATCTGCCAAGGT
1431	CaSNP3403	TTAATTAAGCATAAATGATATATAGTAATTTAACATAAAATAGTTGCAAATGATACTAAT[T/C]AAACATGAATCGTACATACCAATTCAACATAAATGAAACACACCCATAAAACATAAAATA
1432	CaSNP3404	ATATATATATTATGAAATATGAATATGAGCTATGAAGTACGTATATTGACTTAGACAACA[A/G]ACACGATACTGACATTGACACGTCAACTTTGATAATAATTTGAAAAAATGAATTAATTGA
1433	CaSNP3405	TTATTCCATTGTATATATATCTTATTTTCACAATGTTATTTCAAGATTCATTATATTTTT[T/C]TAATGTTTACATTCTTCCATTCTTGATATGCTATGTAGGACTACTTCAAGCAATAGGTAG
1434	CaSNP3406	CAACAAATCTCAAGAAGATGCCATTCTAAGTTGTGTTAGTATGACCAAATGTCAACATAA[A/T]GATCCTACAAAACTCATATGGGGTCCACCCGGAACCGGGAAAACAAAGACAGTTGCTTTA
1435	CaSNP3407	GATGCTCAATTAGAGGAAACTGCACATTTGTTAGAAATTGATGAGATTGTAACTAGTAAA[T/G]GTGCAAATTAAATTGGTATTGTGAAATGAGTTGGAGATCACATTTCTCTTCTATTTGTAG
1436	CaSNP3408	GATATCTTTACCAACTGTGTTGTGTCCAATGATTTGGCTTTACCGTTTTCCTCCTAAAGT[T/C]ATTGAGCTTCCGTTGATCAAAGTTAGGTCTTGGAACATAGAATTTTCTCCTGTCATATGT

1437	CaSNP3409	CTGCACATGTAGTTTGCAAGCAGAGAACAAAAATTAATGAGGGCGGTTCATAATTCTCAA[T/G]GATATAATATAGTTGAAAAAGGTAAATTAATATATTCATAGCATATAATACTTAGCAATA
1438	CaSNP3410	AATTCAGATGAATATTTGAAGGGACAAGTTGGAGATGGTCCTTGATCGGTATTTCAAACG[T/C]CCGAAAGGAAATGTGGATCCTACGTGCCTAAAAACAGATTCATACATAGTAATTGTACAA
1439	CaSNP3411	ATTAGAGACACATTACACAATGAGTACCAAACTACTAATAAGGAACAAAAAGCCATCCTT[A/G]CTATAAAAACGATACTTGTTTTCCTGCATGGTAAATATTATTGTATTCGTATTCATGATA
1440	CaSNP3412	AAGTGGAAGAGTTGAAGTGTTTGATAAGAGTGTTACCAATATGGTTTTCAGCCATTTTGT[A/T]CTCTCTAGTAGTTGTTCAACAACACACTCTCCTTGTGTTTCAAGCCCTTCAATCAAATAG
1441	CaSNP3414	CATTAGTTGGCAATGACAGCAGTAATACCAATATAGTGATGTAGGAGACTTGCACTCAAT[A/G]AAGATATCGTTCAAATTCAAGTGAGTGATTGATGTCAAATTGAGTTGGATTTAACTAAAC
1442	CaSNP3415	TAACTTATCCACTTTTACTAACACATCCTCTATTATCCCACTAGGGTGGGCAATAGATCT[A/G]TTAGCAAGTTTTAGAGAAATGTTAGTCGGTTGGAGTTTTTGTAGGCCCAACTTCCTAAAA
1443	CaSNP3417	TGGAGATGGGTATGTGTTTTGATGAAATTCTGGATTAGTGTACGTATCTTTCTGAGTATT[T/C]GAATGGATTTCTGGGTTTGTGGGGATGAAAAGGTAAGAGATGAAAGGGGATAAATGATTA
1444	CaSNP3418	ATATTAAATAAATAAATGAATAAAAAAATATCTACAAGCTTAAAAAAGATTATAATGTCAA[T/C]TAATACTTATTAAGAAATGATAAACACATAAAGTAAAAGAAAATTAAAAATAAACAAATA
1445	CaSNP3419	GTCCAATAATCCGCTTGTGTGATTACTTTTAATGTAATGTAACAATCCTCATAACTCTTT[T/C]ACATCCCAACAATGGGATTAGACGGTGAACGACTAATGTGATTTGTTCCTTATATTGAAA
1446	CaSNP3420	TTCTCTAATTTCAATTAAACTAATCACCATCTTTGTTGTTGAGTTCGCACCACCATCATC[A/C]TCTCTCGACATTCTCTGTATTCACCGCGAATCAAACTCATTCTATAAATTCATTATTTTC
1447	CaSNP3421	AAGGTAAAATCTTGATTGCTTCTAACCTTGCCACAAGAGCAAGTTTTAGTATAATCAATA[A/C]CTTTATGTTGACTATAGCCTTGAGCTACGAATCTACCTTTATTTCTTACTACTTCACCTT
1448	CaSNP3422	ATTACAAGGCCATCCTTGAATTGGGCTACCTTAATACCCATAAAAATAACAGAGTTTACT[A/G]AAGTATTTAGTCTAGAACTGATTTGAACGATGTTTCAACTGGAGTATTCCTTTCTGATCA
1449	CaSNP3423	TTTTAACACCAGTCTCCAAAATATTAACATCATTCTCAAGTGTTGAGAATACTCAAATTC[T/G]GCCAAACACTCTAAATCATTATTGTGTAAACTCAAAGCTATAAGTGTTACGTATAGTTTT
1450	CaSNP3424	AAAAGAGAAATAACATTTTATAAGATAAATATCGACTTAAAAATATTAAAATTAATCAAT[A/C]AATCAACGCAATAACTTTTCCTTAAAAATATGAGAATATTAAAAAGTTATTTGAAAGATG
1451	CaSNP3425	TGTCACGAAAAATAATACTATACGGTGCAAATATAAAATAAATTTACGTCGCAATGTTCA[T/C]AATCCCACCAGCAACACTTGCAATTACATTCGTGATTCCGACCCCAGTTTGAAACCGCGA
1452	CaSNP3426	GAATAAGAGTCACACGTTGGATGAAAAAGTAAATGTTGAATAATATATAACTGAAATGAC[T/C]CATATAATCAATGCCTTAAAGTTTTAAATGAAGATATGATATCTAACTCACTTATGTGAT
1453	CaSNP3427	ATGTTTTGTTCCAACAACAAGATCAAAACAATCGATTGCTCAGTCGACTTCATAATCACA[A/G]TCATAATCGATTTGGCATTGAGCTGAATCCATTTTGTAACTTAAACATTAGCTTCAATCG
1454	CaSNP3428	AGTTGGAATTGGTTCAATCAAGTTGAAGTTGGAACCATTAAGATTTTGTCGAAGGTTAGG[T/C]ATGTTCCAAAACTAAAGAGGAATTTAATTTCCCTAGGAATGTTGGAAGAAGTTGGTTGTT
1455	CaSNP3429	AGAGTATTTATGACTAAAAATAATAAAGATAAGAGCTAGCTTTATTGATGGATTACTTTA[A/G]TTTGTTGATTTCATGAAACTCACCTGCTCTAATGATGATATGATCCGAGGCAACATGTTA
1456	CaSNP3430	AAATGATTTTGGTTCAATGGCAACCTCTTTCTATTAGTTATTTCAAGCTCAATATTGATG[T/C]TTCGCATATTGGAACCTCGAGAATTTTCGGCATGTGGAAGTTTAATCATAAATTCTATGG
1457	CaSNP3431	TACAGGAGTAAATAAGGATGAGAGGTCTCGATATAAGACACCCTACACCCTAAACTCCTA[C/G]TAATACAATTGATCGGATCTACACATTATTCATCAAATAAACAATGAGGTGATTTAGACC
1458	CaSNP3432	AGTAACTAGTTTGATCCATGAATTCCGCATAAAGGAGACATTGTTGCTATTTATGTTATG[T/C]AATTGACAGACGCTTTATATTATTTTCCTCAGTAGATAGCTCCTGTCCATGGTAATCAAA
1459	CaSNP3433	TTGCATGTTGATATTGTGCTATCACATTGATAGTTAGAAATCCTAGTATCCTTCAAGTTT[T/C]TCTATAAAAGGACTTGTGATCTTTACTGCAAAAAGAACATACAAGAAACCTATTATTGTC
1460	CaSNP3434	CAGAAAGTGGTGTGTAAGAAGCATCATTTTTCACACTACTTTTAATTGAGTTTCCTTTAG[A/G]CCTTACCGCACTTGATTTTCCATCATTCCATGTATTAAAAGTGTTCAGTTTTTTTCTTAT
1461	CaSNP3435	AAAGATGTAGCATTATCATGTGACAGTATCATACTTTTGAATATTGCATGTTTTATGTTG[A/G]TAGCATTGAAGTCTAGGATTGATACATAAGATATACTCTTGCTATCTATGTGGAATTAAC
1462	CaSNP3436	TAGGACATGCATATCATTTCATCCTCGTGACTGTTCTATTGTGATTGATTTAGATTAACC[A/G]TTGATATTATGATCTTGAGTGTTTCTCATGTATATGTTGATTTAACTAAGTGTGCGTTAT
1463	CaSNP3437	ATGAGCAGCAACTTCAAGAGACATTAAAAATTGTATGCTGGCAATGAAGTAGACATTGTA[T/G]CCTGATCCTGACAATGAAGAGAATCAAGTCCTAAAATCAGGGAAGGATCCATTATGTTAG
1464	CaSNP3438	GTCCTAAAATATCAATACATAAACACCCACAGAGAAGTATCAAGCACATTATACATAATG[A/C]TATAGCTAGTGAGATTGTTTTGTACGAAAGCAAAGGGTTTGGCCAATTCATAGCCAAGCT
1465	CaSNP3439	AAATTTATTTTTATAAATTATTGTTCCATTCATCCCATAATCCATTACATTTTACATTTC[T/G]AAACTTGATTTGGTAAGATTATTCATTCACATTAGTTTTATGAAAATTGAATATCAAATA
1466	CaSNP3440	TATGACAATATTTATGTCAAGAATATTTTTAAAATTTTTAATATAGATCGGGGTCAACAG[A/T]CATGCGGTTCAACTAGCGACCCACTGGTTCGACCGACGACTCAGTACCTTGATCGGGTCG

1467	CaSNP3441	CTCAAATGCAAATGTAGAAAGAACCTTAACAAAATCCAAAGGTAAAGCATGCTAAACTCA[T/G]ATAAAAAACAGTAAAGTGAATATCCTCACAAGTTTAGGGAATACATAAGAAACATATATA
1468	CaSNP3443	TATTATTAAATATTAGTTTCATTAGGTTGACCTCTTCAAATTGAAGTTCTTTTAATAACT[A/G]TTTCAACCAGATGAGTTCACATGTTACTAGTGTCATGGCCTAGTATTCTGCCTTAGTGCT
1469	CaSNP3444	CCAATGTATGGTACAAAATGGGATCCGGTAAGGGAACACCATCCGAGGGAGCATATTTCA[T/C]ATTGAACTTAAGAGGAGTATCTACTGCTCTAGTATCTGAAAGACGAGCCTGGTCAAGAAT
1470	CaSNP3445	TGGCTCTGAGATAGATTTATGAAAACAATTTATAGCTTTTACAAAATTAGTTTGATTTTA[A/T]TTTATCTTTTGTTATCGAAATAACGTATACATAAGCAATTATACTAAAAGCGCTTATGCT
1471	CaSNP3446	CTTTTTGTATTTGATTAATAAAATTTACAGGATGATCTTAGTAGTTATGGTTTGGGGAGA[T/C]GGTAATGGCGATGCTGTGATGTTTTCAGTTGAATTTTTACTACATCTCCTTATCCCTCAC
1472	CaSNP3447	TGGTTAAATTATTGAAAAATAAATATCACTTGAAGAAATGAGATAGCTAGGTCACAGAAA[A/T]AAAATTCCGTGGATTGAACGGAAGGATATAAAAAGATAGTATAATAAAAGCAAACTTGTT
1473	CaSNP3448	ATTTTATTCCTTCAAGTATCAATTCATTCATAAAGGTGATTCTTGTCTTGATGTCAATTA[A/T]TATTTGTTTAAATTTGAAATTGACACTTTCACAAAATCAATTTATGTTTGACTTTATTTA
1474	CaSNP3449	CCAGCCACAAAGCTTTCTTCAGTTTGTATACATGATCTTATTTGCTTGTTTTCACATATC[T/C]TGGTGGCTGCTTCACATAAACTTCTTCTTCTAGTGATCCATTGAGGAATGCTGATTTGAC
1475	CaSNP3450	CTGTTTCTGAAATCTGAAGATACAGGAATGTGACGCATCATTACATATGGAGATTTTATA[T/C]CAATAGTTGGTCAAAATGATTCAACGTCTACTACAAAGAAATAAGCAGACCGGACAACTG
1476	CaSNP3451	AATCATATCATCAACATACAAAGAAAACATAATGCGACCATAAGTGGTAGAACTTATAAA[T/C]AGTACAAAATCATGTTCACTAAAGCAGAAACCAAGAGAAGTGATCACAATAGAGAATATC
1477	CaSNP3452	TAACATCATATAGATCATCTTGACATTTTAATTCAAAATGATTAAAATGCATAAAACAGT[A/C]AACAATTAATTCAATTATACATAATTGTACGATTAAAACACAAGATAATCATATTGTATA
1478	CaSNP3453	TCAGAGATCTATTGGTTACTGACGGAACAAACAAAAGATATCCTTTTAATTGTTACATTA[A/G]AATTATTGAACATATAAGTAACTTCTGCTTTGATTTTGGTTTTTAAATACTTGTTATTCT
1479	CaSNP3454	AGGTGGCGTAAAATTGGATCTCGTGCGCTGCAGCTGTGGAGGGTGTATGGTAAGGATCTA[T/C]ATAAGATGGCTATGATTTATGGTTCTAAAACATGAATGGTTCTGATAGCGTGTTGGAGAA
1480	CaSNP3455	CATTAGTGCAATTTTTTTTCTCACAAAGTACATTTGTTATCATCAAAACATGATAAAGGA[A/G]TTGTTCCTAGAACCAACCTTGTTTCAACATCCCTTACCTATGTTTTTTTCTGAAACAATC
1481	CaSNP3456	ATGAAATTGATTGAAGCTATACATTATGGACAATGCACTGTGAAACATGTAACGAGAACG[T/C]TGGCTTGGGGAATAATGTGAAATATGCGTCGAAAAATCTTGTTATATACTTATGATTTTG
1482	CaSNP3457	TTATTTATTTATGACAAATCGCATCTAAATAGCGACACTCAAGACCCTCTCTCTAAGACT[T/C]TAAATTGTTGCAACCAAGATCACCTCTACTTTCGTACAAGACGTTGCACAATATATATTA
1483	CaSNP3458	TTTAGAGGCATATACCTGATGTTTGCAAGGTGAAAACATTACTAAAAGTCTACAACGAAA[T/C]GTTCAAACTTATATATCAAACAAATGATATTGACCCCAATATGAATGTGTTAGAGTGTTA
1484	CaSNP3459	GCCTTCCGTTGAGGTTCATAAGTGTTGTTGTACCTCTTCGGGCACCTGTTCGGAGTGAGA[T/G]AATTATTGCTATTGTCGGGCTAATTTACATTAATTCTAGTTCGTGTGTGAATTGACACAA
1485	CaSNP3462	ACTCCCAGTCCCTGATCATTGCATTGCATTTTACATGGTATCAAATAGAACCCCTATACA[A/T]TACTAAGAACTCAAATGTACACTACACAGTGATGGCTAAGGCTCCTCAGTGTTTAAAATA
1486	CaSNP3463	TATTTTAGTGTGACCTTTCTTAGAGCAAATAGTACAAAAATCATCAGAGGGATCAAACTT[A/G]GGTTCTATTACTTTTTCTTAAAATCTTATTCCTCTTTTATTATTCCTACTAATACCGTAA
1487	CaSNP3464	CTATTCATTTCAAACTTCTCAAGCAACTCACCTATATAATTTTGCTGGTGCATAACCATT[T/C]CTGTCTTTATCTTCAGAAATTCCATGCCTAAGAAGAATGAAAGCTCACCTAGATTAGTCA
1488	CaSNP3465	TTCCTTCACGCTTAAAGTTTTTGAGTGTGTGTGGAGACGAGAGAAACTATCTATATCCTC[A/C]TAGTGGTGTAAAAGATTTATAATTTCAACGGGACAACTACGCTTATATAGTGGAAAGAAA
1489	CaSNP3466	ATAAATAAAAAAAAAGACTAGAAATATATATGATATATAACGAAATAAACACTAAATAAC[A/T]ATTTCAAGTAATATAATGTGAATTTGGCATATACAACAACAAAATACATCTTATAAATAA
1490	CaSNP3467	TTGCAAACAAAAATATAATATTTAGTGGTGTATAATTTACCAACAAAAATATCATAATTA[A/G]AATTAGTGTACGAATTTGCCCCTATGCCTTATTCAGAAGAAAACCATAAACAAATTCTTT
1491	CaSNP3468	AAAGATGTATATACAATTCGGATAGAGTTTGACTTTATTTATTACTATTTTTATTGCTCT[T/C]TCACTCACATCAACAATTATAAATGAAATATTTTTATTGATGATGGAATACCGTGTTGGC
1492	CaSNP3469	GGACAATTGCCTACAAAACAATCTATGTGGCGATAAACATGGTAGCAGCAGCCAGGGAAC[A/T]CCTCTTTCTTTCGAAACCTTCGATGAACAATGCTTCACCCTGGAATCATACCCAGAAATT
1493	CaSNP3470	ACCAACAACCTAAGATGGTTTTTGGGACAATAATCCAAAGTATGCAAATAGAAAATACAC[T/C]GGGGCTACGAGACCTATCAGACATTGATCATACCCCTAGGGTATTCTCGGCAGACATTTG
1494	CaSNP3471	ATATTTTGACTCAGACTTCATCAAGCCGCTTATTAAGCTCAACCAAGAACATGAATACCC[A/G]GTCGTTGTCCTGTCTCTTAAGAAACAAAACACTATCCTAACAAAACTTCCAATGTTCATC
1495	CaSNP3472	GTAGGTTTACTAGAACTAGAGGAAGCAGATGTAGACAAATCAGAAGAACCCAAACAATCA[T/G]AAGATGCAAGTAAATTAGAAGAATATGACAAACCATCAAAAAATGACTATGACCAAGTCA
1496	CaSNP3473	AAGATGGGTATGCAGAAACAAACTGGATGAAGAAGGTAAGGTTATAAGGAATAAAACAAG[A/G]CTAGTGGTTCAAGGGTATAACCAACAAGAATGTATTGATTATGATGATACTTTTGCTCTA

1497	CaSNP3474	AGAATTTGGATTTTCAAGGCTGAACAATTCTTTGATTATTATTCCACACCTGATAATCAA[T/C]GTCTTATTATTGTTGCGGTACATATGGAGAAGGACATTGTTCCTTGGTTCCAAATGCTAT
1498	CaSNP3475	CAATCAATCTCCATTAGTTCAAAGAAAATTATGCCTCTGAATTTTATACTAAGATTATCA[A/G]TATTTGGCAAGGAACACGTGGGATCCATTTAATTCAAAGAAGGCATTCAAATCACTAAAA
1499	CaSNP3476	TCAAATTTAACGTAATTAAATCTAGTTTTCAGAAAATCAACAATCACTCGAATATTATGT[T/C]TAGTGATAAAAGTATGACTAAAATATCACATATATGTAATGTGAATTTTAAGCTGACAAC
1500	CaSNP3477	ATGAATGATTCTCAATTAAAGAAGGTGATAACAGAGCTGCAACAAGACCCTGCATCCCGA[T/C]CGGATTTCCAATTAATCAAATGGATACTTTTCTACTGTAGCAGACTGGTATTATCGTCAA
1501	CaSNP3479	ATCCACATAAACCTCTATCTTCCTCTGCATCATGTCATGAAACAAAGTTATGATTGCTCT[T/G]TCATAGGTTGGAATAACATTCTTCAATCCAAATGACATCATTTTATAGAAGAAAGTTCCC
1502	CaSNP3480	AATGATATGGACCCTCTTTTTTGATTCTGGCTAGTGGTCTAGTGATAATGAATAATGACA[A/G]TGTTGTTTGTGGCCTTTCTTTTTCTACGGTGGAGTGTCATTATCACATTTCACACACTAT
1503	CaSNP3481	TTTTGAAATATACATACATATGCTTATATTGCTGGACAAAAGAGTCTCTCTAATATAGAT[A/T]TCTCTAGAAAAAATCCAAGGGTGGAATCATACTATTTTCAGTAAAAAGAATATTATAATT
1504	CaSNP3482	TAAAAAACGTCAATTTAGTCCATTGACTAAACAAATAAATTGACTAGCACATTTAAACGA[C/G]TAAACCATTATCCAATTATTTAATTCCTCCCTCAATTTTCATGTTTGGGTGATTGTCAAT
1505	CaSNP3483	AATCGCCAAATAAAAATATTTTTGTTATGAAAGTAATTCCACTTTGAATAAACACTTTGG[A/G]CACAAGCGTAGCAACATGCAAGGTATTTACAACAAGCAAAATGAAACCAACTCATAAATT
1506	CaSNP3484	TTGAATGAGTGTTTTTTAATGGATTGAAATACTTTTACTTTTATTTCACTGCTTTTTATT[T/G]GGTGCTGATTATGTTGAACTGAAAATAGTTTTAGTAAAACCCTTTATCCTGTGCTATATT
1507	CaSNP3485	TTAAATATATCTAAGTATAAAAATATAAATTATTTTCTTAAGTTTTTTTATGTGTTTTTT[A/G]TATAATGAAAAGGTTGATACACAATAATTGAATACTAACACGAAAGAAATAGATATAATT
1508	CaSNP3487	TTTTGTTCCTCGATGTTGCAGCTTATAGACGTCTCATTGGTAGGTTGTTGTACCTTACTA[T/C]TACACGTCTTGACATTTCCTTTGTCACTCAATAGTTGAGCCAATTCATGTGCTCACCTAC
1509	CaSNP3488	TAATTAACCTCTTTTCAAAAAAAAGAATGATATTACACACTGATTTCTCAACAACTGATA[A/T]CAGAGTGAAGGTTTAGATGGAGGAATAGAAGCAATGTATCATGTCATAAAGAAGTTTTTG
1510	CaSNP3489	TAGATTGTTGAAGGCATACAACACCTTGACCCTACAAGTCAAGTATTATCAACACAATCT[A/G]ACAATCATAGATTGCTTGAGGAACACTAAACCCACTATCGAGCTGGGTTACAAGAGTTTT
1511	CaSNP3490	AAAGTATTGAGATTGTAGTTTTTCACAATGATGGATACAATAAAACTAACATTCTCTAAG[T/C]TATGAGTGTTAAACTCTTAAAAATCAGATAAATAATTATAACTAATGTTTTCTTTCAAAG
1512	CaSNP3491	TGTGTGCATGTACACTCCCCTGACAGATCGTTGATGGAAATAATCAAAAGCCTCAATTTT[A/C]TGTTCGTTGTCGTTTCCTAAGTCAAGATCTTATATTCAATACTCCCTACAATGAACAGTG
1513	CaSNP3492	AGACAACCTTTTCGAGTCATCATCCATAGCCTGGTTTGTAGCATCATTGACATAAAGGAC[A/G]CATGCTATTCGCCTGTTATGAGTCCAAACTTCAGCCGCAATCACATTGAATTGGAGGCTT
1514	CaSNP3493	TGTGTCGTACCAAATGATCTTACTCTTCTAATCACCTCCAAAGCATATGAATCCTCCTTT[A/C]TTGAAGGTCAAGTCTTGGAACATAGACTTTTCTCCCGTTGTGTCGTGAGCATCCACTGTC
1515	CaSNP3494	TATTTTTCTTGGCTACTCGCCTCACCAAAAAGGATATAGATGTTACTCCCCAACAACCAG[A/G]AAAGTTTATCACACTATGGATGTTACCTTTGAGAACCCACCTTATTACTCCAAAGTTTGA
1516	CaSNP3495	CACACATCATCATTGCAATCTGATTAAGCAAAAGAGATCAGTATGTAGCTTTATTATCAA[T/C]TGGTCCTATAAATTGAATCAGTTAAGGATTTAATGGCAGAATTTCAGCAGCTAAGTCACA
1517	CaSNP3496	ATTACTCATTGTTGTGAATCAAGTAAGTAATAATCAACCTTTGTTTTTGTTGTGTTTGTT[T/C]GTGTTTTGTTGTCTCTGTTTATGAATGCAATGCCTGAAAATTGAAGAAAAAAAGTTTTCA
1518	CaSNP3497	ATTAAGACTTAGGAATCCTAATCTCGACGATCCAATCATATTTTATCACCTAATTAGACT[A/T]ACTATGACTTGAGAATCATAATCTCAATGGTCCAATGACACAATCTAACCTAATTTGACT
1519	CaSNP3498	ATGTAAGGAATCCAGTTTGTCCATGACCATGCGATTTGAAACGTGATCATCATCAGATGC[A/G]GTGTCTTCTGCTTCGTCGTCTGCTTGGGTAGCAGTAGAAGGTTGATCCAAATCACCTTTA
1520	CaSNP3499	TTGTCTTTAGTGTTCAAAATATAGCATATACAACCAAACTGATAAAAATATGAAATGTCA[T/G]ACTTTATTCCTTTCCATAATTCATAAGGAGTCTTATTGCGAATAGTTCTAATATAGATTG
1521	CaSNP3500	AACAAAATCATGATTATCAACAGAAAGATTTACAAGGAAAATGTGGAATGGAAAAGGGTA[A/T]CAAAATTATGATTATATTAATTAAGTAACTTTGCCTTAAATGGGAAATTAATGATGAAGA
1522	CaSNP3501	AACAAACTAGTGCAAATCAATTTGCATGGTAAAGGGGATATCCCAACAACACACTAGCCA[T/C]ATGTTAAATGAATCTAACAACATCTCCCCCGTGATGTTATGCAGGTGAACCATGTTGGTC
1523	CaSNP3502	CTGACCCTACAAGTCAGTCTTCTCAAAATAACCTAACACCTACAGACTTTTTAAGGAACA[T/C]AAACACCTTAACTCTACAAGTCAATTATTCTCAACACAACCTGAAAACCTACATATTGCT
1524	CaSNP3503	TAAAGTAGGATACAAATAGCTTCTAACTTGGCCACTGGAGAAAATGTCTCAATGTAATCA[A/G]TGTCATTTTGCTGAATGTAGCCTTGTGCGACAAGTCTTGCCTTGTTTTTGACCACTTCAC
1525	CaSNP3504	CATTACCAATCTGTGCGTATTTATCAATTAGTTTAGCGGCGATAAATGGGTCTTGCTCGT[A/G]GCCTCCGATGATGACTTGGGCGTGGGTTTTTTTAATGGAATCGACGGTTTTGCAAAGGTG
1526	CaSNP3507	ACTTGTAGTAGAAACAGTAACAAAACTAAGAAGTGCAAGAATAAAATGACACAAGTAATT[T/C]ATTATGGTTCTCCACTAACTTGACTAGATGCAGTCCCTTCACTCAAAATGATTTAATCCA

1527	CaSNP3508	GAGTCGAAAAATGAAGTTTCAAGCAATAAAATAAGCTCCGACGGCCTAATTCAGAATCCC[A/G]CTCCACGGAGAAACGTGAAATTGGCTAGTGACTCGGAGTTTGGTTTAAAGAGCCAAGGTG
1528	CaSNP3510	TTTTGATCTTGAATTTTTGGAGAATTATTATGCTGAAAATGATTTGAATGAAGATGAAAC[A/T]AGTGATGATGATAATTATGATGACGACGAACATTGGTTAGAAACAGAAATTGATTATGAA
1529	CaSNP3511	CCATATAGTGATTTCTTCAACTTATATACTTTCTTCTCTTGGTTATTTATCTCAAAACCC[A/G]GGGGCTGCTCAATGTATACTTCTTCTTCAATAGGCCCATTTAAGAATGATGACTTCACAT
1530	CaSNP3512	TGCTTGATTTTTATGATATATCATCATGTCATGACATATGCATCTTAGTGGACTGTGTGG[T/G]AATATTTTATTTTGATAATGCACCATTATGTCATGACATATGCATCTTCGTGGAAGTTGC
1531	CaSNP3513	TAATGTTTTGCTTTATTATTTCTCTCATTTTGTGAATTGGCACCACAATGTTAAAGCATT[T/C]ATTTTGGTATTGAAATTTGTTTAGAACATCAAAGTAGTGAATAGAGGATACTAGCATGTG
1532	CaSNP3514	GAAAATAAATATTGAGAAGCTTAGCGATTCTGGGGAAGTACAACTGAATGAGTCTAATCA[T/C]GGTTGTCTGCTGTTGAGCCTTAAGGCAAGATTGTTAGACCTTGGAGGTATTCGGGTGGGG
1533	CaSNP3515	TCAAGAAAATTAATTAATACAAATTCTATGACCCACCTTTCTTCCATGGGTAAGATGAAG[T/C]TTCGGGTATTCTTTTAACACAGATTTCACTTGCTGTGCTAATTCAATCCATCTCTGTGTA
1534	CaSNP3516	TTTTTTGTCAAATCAGACACAAACTAGAACTGATGTAAATCAGCCCGATAATGGCATAAC[T/C]CTCTCATTCTCAAAGAGGCACCTGAAAAGGTACAACATCCCTTATGAACCACAACGGCAT
1535	CaSNP3517	TGCCGAAATAATCTGATTACTGTGAACTTGTTTTGCTCTTTTTCTGCGATAAATGATTAC[T/C]TGTCTATTAACTTTTTCATTTAAGCTTCCTAATTAAGTGGCGTAACTGTAGGTTCTTTAT
1536	CaSNP3518	AATCAATAACTGAAAGAATGTTGATGTATCTTTCTGCGTTGCAAATCCAATGTATATGAT[T/C]TGAGCTGTTCCAAGTAATGCTTCTGATAATATTTACTGCACACTTCTTGCTCAAAGTGCT
1537	CaSNP3519	AACTTACCTATATGATGATGACAAGAATTAATTTTCATATTTCTAATAGCATTCCATATC[A/G]ATCTCTTCACATAAATTGCATCCCTCATAAGTTAATATCTAATATCCAATGCACATTAAT
1538	CaSNP3520	CAATATCAATTACTGCGTCACGCATTTAATGCGTGGACTTTGAATCATTGTTTTCAAGAA[A/T]TTAAGTTAAAACTTTGTTCTTATCTTATGCAGGCAGTGAAAGCTTGGGAGAGCTTTATTA
1539	CaSNP3521	TAGCAGTTTTGGGGTCGAGCACTCCGCACCGCTATTTGAGAGTAACAACATAGGTTAGAT[T/C]GCAAGTCTATTGACACACCTATAGATCTAAATGCCAAATTAATGGCAAATCAAAGGTCTT
1540	CaSNP3522	CGGCCTCTATCAATGTTGAATTTCAATCTAAAGACAACCTCTAATTGTTGAATACTCCCT[T/C]CGTTCCTTTTTTATCTGTCGTTGGAAGCTTATTTACACAAACCAATAAAGCTAATAACTT
1541	CaSNP3523	AGGAGAGAATCAAATGAAACTCACAAAGACGTGCTTGGTTTTTTGATGGCAAGAGATGAA[A/C]ATAGGCACATACTAAGTGATGAAGAAATCATTGATCAAGTGATTACACTTATGTATTCTG
1542	CaSNP3524	CGAAACAACCTAACAACCCTATACTTTTGAGGAACTAACCACCTTGACCCTACAAGCCAA[T/G]CCTTCTCTTAACAACCTGACAACCCTTGATTGAAGAATGAATTAAACCATTATTGTGTTG
1543	CaSNP3525	CAGGTCTGGATGCAGTAAGGTAAAGCAGGGATCCTATCATTCCTCTATAAGGTTTTTGGT[T/C]AACTTTTTCGTCTGAATCATCTTTTCCAAGTAGACAAGTAGAGTGCATTGGTGTAACCAT
1544	CaSNP3527	CAATTGATAAAGAATGTTTTATAAATAAAGATCGATTCAATTGATAAAGAATTGACACTC[A/C]TACTTTCAATATATTGGTACTCTCTCACCTTTATGAGAGTCCATAATCCAACAATCTAAA
1545	CaSNP3528	AATCCATATAGGACATTGGTGAGTTTAAAGATACGATTTGGTTTCTTTTTATCTTCAAAA[T/C]CAGGAGATTGATGAACATAAACTTCATTTAAAAATCCATTTAAAAAAGATCTTTTAACAT
1546	CaSNP3529	AAATAATTGCGCTCTCGCAAGCGCCAAATACAAGCAACGCCATGTGAATTTCAAAAACAT[A/G]TAATAGTATAAAATAATTGGGAAGAACACGTAAAATACATATTAAACATTGTATCATATC
1547	CaSNP3530	GTTGCAAGTATAATTATAGATTCACGATTAATAAAACATAAATATTGGTTATAAGTCACA[A/G]TTATTTATCTATTCATCAAAATCAAATTATTAAAAAGTTTAGAAACGTTTATCTTTCGAT
1548	CaSNP3531	TTAAGATTGTAAGCCAATCCTAAAATATATTCTCTAGTATTCTAACATACATTAAGGACA[T/G]GAGAAAGATAATTGTTAATTATGCATTGAACTGTTAATTCAACATTAGACGGGCTAACAA
1549	CaSNP3532	AGATATATGCAATGACTGGACAATTGAATGCTAAGAGTGATGTATACAGTTTTGGTGTTG[T/C]CCTTCTGGAACTTTAGACCGGAATGAAACCTATTGATCATACACTACAACGTGGACAGCA
1550	CaSNP3533	TTTAGGCCGCGACAAATTCAACACTCTAATAGAAAGTCAATGATCTGATAAAAAAGTCAA[T/C]ATTCTGATGGAAAGTCAATGATATGATAGAAAGTCAACACTCTAATAAAAAGTCAATGTT
1551	CaSNP3534	GAAAACAATATAATGAAAGGAAGAGGGAGGCAACGCCTTTGTATGTATATTTGCAAGTTG[T/G]GCAGTAGAAGGAACATACATAAGATGGACGGTGCCATTTTGAATTTTATCACGAACCAAA
1552	CaSNP3535	CCAGTAGCCAGCACTGGAGCTAAGAGCAAGCAACCAGCAGCACACGCTAAATTTCACAAA[T/C]TCCTTTGCAAGTACAAAATGAGCAGCATACCAGTGAAAAAAGAGATGAAGACAAAATTGA
1553	CaSNP3536	AACTTAGGAGATCAAATCCAGACTCTACCGTTATTATTAAGTTTGGTATATCTGATGTTC[A/G]ACCAGTTTTTTAGAGAATATATGTATGTTTGGAGGCTTGTAAGGATGCGTTGTCTAATAC
1554	CaSNP3537	GTAAAATAATAATTGTTCTTTACCTTGTTCGTTTAGCAAAGTCTAAAGGTACTTGAGATG[T/G]TTAGAGATACAGACACCATATAGACCACTCCTAAGCTGAATAATCTATTTGATGTTTCTT
1555	CaSNP3538	TCAACGAGCGGATGCGACCATTGACACTAAGACACGTCACATTACTTCCACCATCACAAT[C/G]ACTACAATTCTTCCCGATCTTTTTATCGTGGAACCAACCGTTGACACCGATTTCACCAAG
1556	CaSNP3539	GGAGGATGAATAGTCCTGACACTCTTCCTATGAGTAATAACGGGCACAATAGGAGAAATG[A/G]CAGGTGCAGAATTTGAGGCATCTGAATCCAGATCAGCAGTTGAATCAGATTTGATGTCAA

1557	CaSNP3540	GATAGTGGAAAGCCATAGTACTACCAAGAGGCTATTACAATTGTTGATAAATAAAAGTGG[T/C]TGAAGGCTATGCATGAAGAGATGAATTCCTTGCATGAGAATCACACATTTGATTTAGTAA
1558	CaSNP3541	CAGTTTCACCCCTAAAAAGTTGAGTATGTCATCATACTCTCTTTTATCAAATGAAGAACT[T/C]AAGATTTTCACCCCATCATGTAAAGATAGGTCAAGCAAGTATACTCCTTCCTCGTGGGCC
1559	CaSNP3542	GAGTCATCCAAGGATATAAAATTATCTCAAAGTGTTTACACTCAAAGTGTTTGGGCTACT[A/G]TTTACACTCAAAACACATCATGAAAGCTTGCACTACCATAGGCTTGACAAGCCTCTATAA
1560	CaSNP3543	AGATTAAGCTTTAGTGATAAAAACGAGAAAGAGACAGACAAATTGAAAGAGGAAGACATG[A/G]CACATGAGTGGGATGTATTGTTAAAGCGATTTCGAGAACTTGATGTTGTTGGTAGAATTA
1561	CaSNP3544	TTCGTTTCTGCAGCACTGAAACGGAGAATGTTCTACGTTTCTACAATGTTCTTCATTTCT[A/G]CAGCATTGAAATGGAGAGTGTTCTGCGTTTCTGCAATGTTCTTCGTTTCTGCAGTACTGA
1562	CaSNP3545	TTCACTCATATTGAATCTATTTAGAACCTTTCCTGACGTAGCTCTCTTGGGAAATTATAA[T/G]TTTTCCTTGTTCTCTAATTCTTATGATTTCCATTCCTAGGATCTTCTTGGTTGGCCCCAA
1563	CaSNP3546	GAGTCAAGAACATTATACACCAAATGCAAAGATATTTTCCTTGAGGAGGTAGAGGAATGA[C/G]TTTAGACCTTGTAGTTAGAGGATTGCCAATTCTAATTTTTTTAGGTTCTGATAAGTCCAT
1564	CaSNP3547	CAAATCCACATGTCCGTTGATATATTATGACAACCTCTTCATATTTCCACTAATAGGTTT[A/G]TTAAGACAAAATTCCAAATTAATGTTTTGATGATAATTAAATAAAAAATAATTAAGACTT
1565	CaSNP3548	ATGTCAAAATAAATCAAAAACATAGAATGTAGTAACAATGCAAGTAACCTTTGTTGTCTC[A/C]CAAGGATCTCAAATATAACAATAATCAACATTAAGAACGTGCAACAAATAAAATAAAAAA
1566	CaSNP3549	CGACATGGATCCATTGTATGCAACAGCAGCAGAGTTTTGAAACAGTAAATTTGCTACAAT[A/G]AACAATCGATGCATTCACTACTGTCGGATGAAGACCAAATCAAATGGGAGCCATTTGAAG
1567	CaSNP3550	TACTTTGATGGAACAGATTACTACTTCCGGAAGAACAAGATGCAGTTGTTTCTGAAACCT[T/C]AAGATACATGATTGTGGCGCATCATTATAAATAGAGATTTCATACCAATAGTTGATCAAA
1568	CaSNP3551	TAATCTTGTATGCCGCATTATAATTCCGCTGTGTGTATGAATCTGAATAAATCTTGCATC[A/G]TATGATTGTATGTTCGATCTTAATTGTATGCTAGACGTATTCGATTTTCTATATTGCTTT
1569	CaSNP3552	CTCCTTTTTCTCTATCTTGCAGCTCTCCCACATTTTGAATTCTACTTGTTCTGGATCATC[T/C]TTTTCTTTGCTCTCATCTTCTTCCTTTGGTTTTCCATTCACATCCACAGGTTTCGGAGAT
1570	CaSNP3553	TGTCATGCAAGAAGATATGAATTCCTTGCATGAGAATCACACATTTGAGTTGGTAAAGTT[T/G]CCTAAGAGCAGAAAAGCACTCCAGAACAAATGGATATACAAGATAAAGGCAAAAGATAAT
1571	CaSNP3554	AGGAAATTAATGCAATGCTCTAAAAATATCTATTAAGATATAACTAAATCTAACAAACGC[A/G]CTCTCAACGCGATTGTGTTGAATCAGTTAATACAAGTTCATTCTGATGATTTTTGCGTCT
1572	CaSNP3555	TAAATCCTAAGTCCGTATATTTCTCCTACGATATGGTACTATGTCAATCCAATCTCTACC[A/G]CCACCCATCTAACTTCGCAGCATCAAAATTAACTCATTAACGACTGTCATCAGGTAATAT
1573	CaSNP3556	TAGGGCATCACTAGATACATTTTTTGGAGCCTCATAGCCATCTTTGACTATGCCCACAAA[T/G]TCCAGGAACATGTGAGATGTAGAAACCTTTAAGCTTGTCCTTCCATTAATCATATCATCA
1574	CaSNP3557	GTTGAATTATACCTACATCAACTCATGTGTGGATTGTTTAGGATGGGTGAGACTACATAC[C/G]ATATGCATAAATGTGTAATAACCTCACTTATTATGGTTAACAATAAAAATGGTTCAGTGG
1575	CaSNP3558	TATATATTATGAATTTACTATTGTTTTATATTAAAGATTATATTGTTATACAGACTCATT[T/C]TTAGGTGCACAAGTGAATTAAATATTGTATGGTGTACAACGTTGTTTGTCATTAAATTAA
1576	CaSNP3559	CTTGCAAATATGAAAGGAAAACCAGCATCACAGAAAATTAGGAGTTATAAGGTTAACTTG[A/G]TGGTTGGACTGAGGAATTAAGAGGTGGAGTGATAAGGTGATACAATTAGGCCAAAGCCTA
1577	CaSNP3560	TGAGTATGCCATCGCTCTATTTAAATGTCTTCATCTACCGTAGTTCACGACATGATTCTT[C/G]TTAAAGGCGATGAGTCTTCCAACATTAATGCACGCTACATGAATCTACATCGACAAACGA
1578	CaSNP3561	TGATGAGGATTATGAAGATGATTGCGAGTCTCCCCCATAACTAGAAGGGTTAATTGAACA[A/G]AAAGCTAAGATAATCCAACCCTATCAAGAACTTGTTGAGGTGATCAATTTATGGACTAAA
1579	CaSNP3562	AATAATGTGAATCAAGAAAAGGAGGGGCCCCCACTTCAAATAAATGTAAACCCCATTCTC[A/G]TCCAATGGAATAGTTCCTCCCACTATTCACAATTCACAAGAAGCAAGCAAATAAGGATTT
1580	CaSNP3563	TCTAGAATTCATTCTTCAGGAAGATCAATATCTTTCCAAAGGATGGTTTTTGGAATAACA[A/G]TGTTTCCTTTGTTAGATCAGTGTGTAAAAATAGTGTTTCGCCCTTTTTGTTTTCAATATT
1581	CaSNP3564	ACCCTCAAATTTAAACAATTAAGTTGAGCACCCCTACACTTATTTAAGGAAAGACTCATT[T/G]ATCCCCAAGGTTGGGTCAATTCATTGTTTTTCACTTTTAGGCTTGTAATGAGCTATATAA
1582	CaSNP3565	AAAACTCAACATCTATAGTAATAGAAGAAGAAGCAATCTCTGATCCTCTTAATGTGCCAT[A/G]ATCATCACTAGAGCATTGTTATCCCTTCCTTACATTGCTCAGATTATTGTAAACTTCAAG
1583	CaSNP3566	TTTTAGGTACACAAATTTGGTTAGGTCTTGGTATGTTAGGGACAATATAATTTGGAGTGA[T/C]CTTTATAAGAGCAAGCATTACACAAGATATCAAATGGAATAGGTTTAGGTTCAATTACAA
1584	CaSNP3567	CACCCAAACCACCGTTGCTGCTGCCACCGCCGCTACCCATAAGGGCACCTTCCATGGGGT[A/G]TAAAAAACAAATACCTTTATCTCAATTGGTTAGAACGTTGTGGCTGTTAACCACAAGGTT
1585	CaSNP3568	TCTCCTCAACGAAGTAGCATAGAGAGACCTACGACGTGGCCTTTCGTTGTCACAACAACC[T/C]AGTCACAATGAATAGAGGATAAGTGACATGATAGACGAAATTGTGGCTCTGTTCAAAAGA
1586	CaSNP3569	AATCTCATAATTGCTTAACCTTGATTTGGCATTTGATTGTGTTGATGATCTAGACTTCGA[A/C]AAATGATGATATCTTATCAGGTAAGGTTTAATGTGTTGAACATTAGGTGGATCGGATTAT

1587	CaSNP3570	TACCAGGAATATCATCCAAGTCCATCCGATTGCCTTCTTGTGATTTCTTAAAATCTGCAA[T/C]AGTTTATCTTCTTGTTCATCTTCAAGTTTAGTTGAAATAATCACAAGTAACTTGCCACTT
1588	CaSNP3571	AGGAAGAGACATCATGTTTACTGAGGCAACAAAATTAGGGACAAGTTTCCCCCAATGAGT[A/G]CCATGAGAAGTAAGACCAAAAGCAGTTCATTCGTTGCACAAGCATTTCCTTCACGGTAAA
1589	CaSNP3573	GTATGTGGCGCATCATTACAAGAGTCAACCAAACTGATTCAACATCTGCTGAAAAGGATC[A/G]ATTATTTTTATCATGTGCTTTAAGCAGGGAAGAAAGTGAAAGAGTCGACGAATGCGACTC
1590	CaSNP3574	ACTGAAAATATTTAAGATCACAGAGTTGTCACAACTGATGTGTTTTAAAGTAGGGACTGA[C/G]AAGAAGTCATATGTTGATCAATCAAATTTTGTTAAAAGATTATTAATTTGGAGCTTAAGA
1591	CaSNP3575	GCATCTCTCTTCTCTTCCTCCTACTTATTCCTTTTTCCCTTTTCACACTATTTCCCATCA[T/G]ATGCCAAACAATGTAAAACTCAACTTTTTCTTTCCATATTTTATTTCCTCTGTTTTTCCT
1592	CaSNP3576	TTCAATGACAACCCTTAAATTTAATTGAATGGTTAGAATTAGCTCTTCTAACTCTCACAC[A/G]TAACAACTTCATTCAAGATATGTCTCTTAATATATATATATAATCGTTATTTAGTTGGTT
1593	CaSNP3577	ACAATCTCATCATTATTGTAAAACTCGAACTATAAGAATTTAATATAGTTTGGGTAGATT[A/G]TAAGAAATCCTATCAAGGTTGATAGGTGAACTGATTAAATCTCTTTATGGGTGGAAATGA
1594	CaSNP3578	ATCTACATCTAGTTATCCACCATTGGTAGTAGAGCAAACAATGAAGGGAAAGGGAATTAT[T/C]TGTAATGATTTTAACATGTCCGGCGCGGAATGTTTTAATGATGGACTAATCTTAAGAGAG
1595	CaSNP3579	TGAGAGTGAAGTTCTACAACCTCTTAGTATCGAGTCGTCCAATCGATGTCCTATCGAAGG[A/G]AATTTCGCCCAAGAACTTTATTATATTAGACAAACATAATAGTTGAAATGCCTCATATTA
1596	CaSNP3580	ATGCTTAATTGTTATCAGACAAATCCAATTGAACAAAAAGATTAAAGAAGAATATGAATC[A/G]AAGAGAGTCGAAGGAAATTGAATTATTACTAGAAAAGCAAAGCGATTAATGACAGGTTAA
1597	CaSNP3581	AACTAATCATCTCCCTATTAAATCCTAAATACTCTAAATTCCCTTTATACTATATGCTAA[T/C]ATGGTTCACCACATATATCATTTTCTTATCGTGTGTAAGATTGCACAAATAGCTAACTAT
1598	CaSNP3584	AAAACAAAGAGGACAAAACATGAACCTGTAAGTCCTCAACTTTCCGCACAGAATTTGACA[T/C]GCAATTAGAAGAGACTAAATATGGCTTTGAATTCTTTAACCAGGAATTAGCTTCTGATAA
1599	CaSNP3585	TCTTTCTTCTACGTTCTTCATTAATTTGTTCTTTTTTTTTTTAAGTATGTAGACAAGATA[A/G]TTAATATCAGCATAAACTCACACAAACAACTCCAAGATAGGAATTTGTACTTATCTCTAT
1600	CaSNP3586	GCGTTTTTCACCTTCATCACCCGGCCAAGGCATCCCAGAACTCTTCATCTTAGTTTTCAA[T/C]TCATCTACCTGAAATAATCAGTTCATTGGCTTCATCTGTAAGACTGTACATCATTTTACA
1601	CaSNP3587	CGACGCGTTTAATTGTCTTTTATTCTTTCAAAATTCATGTCAATCTACTAATCGAATAGT[T/G]TTCATGTGGTATTGGTTTATCTCGGTGCGCTTAATCTCAAAGCTTACTAATAGGTCAGGT
1602	CaSNP3588	TACTCTCTTTACCCAGTGGTTAATCATGATTGTCTAAACATTATGGTAGATTTATTTTTC[T/C]TCTGTATAATGCCTGTTTCTTTCGACTGTAATGTATTGTTCCTTTCTCACTTGCTATATT
1603	CaSNP3589	ACGTGTCATATGTTGAAATTGATATGATTTTGTTGTATTTTATAATGTCTTTATTTATTG[A/G]CAAGTGGCTTGAACCAAATACAAAATTGAAAATAAATGAATGGAGCTTTATGTGTGATAA
1604	CaSNP3590	CCTACTTGAAAAAAGTTGTCACTAAATATAGAATGCATGAGGCAAGACATGTTACTACTC[A/C]TTTGGAACAAAATTTCAAATTGTCCATGCAAGACTCACCTAAGATTGTAGATGATATTTC
1605	CaSNP3591	ACCATTATTTCAAGTTCGTTGTAGAAATACTATATAAAAAAAGAGATATTCAAAAGTGTA[T/C]GAAGTTGAGGGAACCACAATTTGAAGTTGTTAGAAAGTCTTCCAAGAAAAATGACCATTA
1606	CaSNP3592	GGAAGTCGATTCCCAATTTCTTGTTTCTTCTTCTTCTTCATGAGCACATGATATCCCAAG[T/G]CTGTCACTTCCATTATTATTAGGCTTTAACTGTTCAGAATGTACAGAGGGGACCACTTGT
1607	CaSNP3593	TACGACGTTCATCTTCCACCAACTTTTCATCTTCTTCCTTTCTAAAAAGAACTGTTCATC[A/T]TATTCCAATTCCTCCCTGCATTTACTCACCACTTATGGAGTATGGATAATGTCATAACAC
1608	CaSNP3594	GAGGACGCCATCATAGTATTTGTAAAGATCTAACATCGCTATAAATGTCATTGCCACCTA[T/C]GGTCTATATAGACGCTTAACCTTTAACCTTTGCTCCTACCCAATCTCTTACTGACTTGAG
1609	CaSNP3595	GTTCTTTCGGCAAAGCTTATTCAGTGCAAGACAGGGTAAGAAAGATCATGAAATGTATTT[T/C]AATCACGTGAAGACCAATGGTGACAGCTATAAGTCAAGCCAAAAATCTTGAGGTACTAGG
1610	CaSNP3596	ATGAACCTTTCATGTGGAATTACTTTGAGAACATGTGACTCACCATAAAGGTAGTATTGG[T/G]TTGGCATGGACCTGAAAAAGTTATTGGTTTGAGACGGTAGATCCTATTTTTTGGTACCAC
1611	CaSNP3597	GAATGATCAATTGTGTTTTGAAAAAGTTGTGCAAATATGCATGCTTATGACTCAAGTAAT[T/G]GCGCAAGCATCTTAATTTGTATGATGTGTTTGGTTATGCATCTATTCTTTTTATTGTTGT
1612	CaSNP3598	TCTATGCACCAGTAACCAATCATGAGACGATCATTTACATTTTTGTCAATCTACTAATCG[A/G]TAACTAAACTGAAGACGATCATTTACCTTCTCATCAAGCTACGAATCGACAACCAATCTA
1613	CaSNP3599	ATAGTCATAAAATGGATGATTTTGACTAGAGCAACCATACTTTCAAGTTTCACGAGAGAA[T/C]AAATTCATGAGAATTAATTGATTATTTTAGTGTGAATCTTCTCAACAAGTTGTACAACTA
1614	CaSNP3600	ATTCATCTTGTGTATCAAACATCAGGACAGTGGTAAACACACCAGTCAAATAAATCATAA[T/C]AGGTTCAACTCCGTCATTCATAGCATAAAAGTTAGGTTTCACATCATCATACATTTCTTC
1615	CaSNP3601	GGTGTACATAGACTTTAGTCTTGTTTTTCATATGTTCTTACTAGTTACTTTGTATAATGT[T/C]TCTCTTTAATGTTGTTGGAAATAAGATAGATTTGAACCTTATTTTTTCAATATTTGACTT
1616	CaSNP3602	AGGCCAGCACTTAGCAGAAAAAGATTTAAATGAATGGAAATGAACAGTATGTTAAACAAA[T/C]AACTATGCCCTTCATCAAGATTAAAACATGATATTATCCATGTATACTCTGCCATATTCG

1617	CaSNP3603	TTTCATTCTATAAAAGAAACATAAAGAAAATCAAAACTAAAATAATGTATTAGTGTGACT[A/G]ACATTTCTGAAAATTTAATGTAATTTGGAATCTACGAGTCTTGCAAGTAAATTAGTTAAT
1618	CaSNP3604	CATGCCGACGCACAGGTGGCATATCCTTGATATTGTATTATACATGCATTGTTTAGACAT[A/C]AAATAATAAATCTCATTTTATTTATCAGTAACACGTATTTGTTCCATAATTTTTATTTTT
1619	CaSNP3605	CAATTAATTATAGTTTCTAATTGATTAATCTTAACTTAAACTACATTAGAAATTAACTCT[A/G]CTTAGTCAATTTTCTGATTCAAATTAACTTCCTAATCGATTAATTATATCTCCTAATCGA
1620	CaSNP3606	AAAGGGACTCACAATATTAGGAGGTTAGATAATGACAAATTTAAACGGACTATAAACTCC[A/G]CAAAGAATTTGGAAAAAATAGAATTCTCTCATTTGTTTATTAAATGAGAACTTTCATAAA
1621	CaSNP3607	ACAACATTTCAAGTATAGGTAAATTAATTCTCTAGTTAGTTTCTTTTAGCACTTAACTCT[A/G]TGTAGAAACTGAACCAAAAAACTAGTTTCTTAAGTTGAATTTTTTCAACATCTTTTATAT
1622	CaSNP3609	CGCTAGCATAACAAATTTGTTATCCTAATTGACATTGTAAACTATTAGCTAAAATGAACT[A/G]TAGGGTTTATCTATATCCATTACATATAGTTGACCTAAAAATCTAAAAGAATAAAGGAAT
1623	CaSNP3610	TGTCTGGTGTTCAGTGCATTTTTATCCTCTTTTGAGTCTGAATTTGGTTCTAGTGTCTTC[A/G]TGAAAGTTTCATCTATGGATCTTAGCTTTCATATGCACTTGGCTTGACTCCAATTGTACA
1624	CaSNP3611	CATATTTTTTCTCATCGTAATGATCATGATTAATCTTGTTAGTGGTAAGGATATTGAGAC[A/C]ACTTGATTAATTCAATCTTTATACATTTTAATCTTGTTTGTGAAATGAACATACTTTGTA
1625	CaSNP3612	CGGAATACAACTTACACTTGAAAACCTATTAGTCACATTGATTATGTTCAATCTTCTCAA[T/C]CTTCTAGCTTAGTACAATTAAGTTGTGACAACCACTGTTGAACTAGGTTACAAAAGAAAG
1626	CaSNP3613	ACTAGCCCCCCTTCTTGGCATCTGTATTAATAAAAGAATCTTGCTAGATATCTAAGTAAC[A/G]AACAATTCTGTAACATAAATTATCACCGATAACAAGGATAGAGTTAGAAGGATGACTAGT
1627	CaSNP3614	AATATATGTATATCAGTGTCTTATGGTTTTTAAATTATCACTGTGGACTGGCTTGTGTCA[T/C]GGCGAGTGTGCGTGTTGGACTTTGTGCTTTATATATATATATATAAGCAAACATAGAAAA
1628	CaSNP3615	AACATAAGGCAGATCAACACGGAGTGAGTTTACTTATTTTGTCACCCCTAATTTATTTTT[A/G]GATGAAATTTATGTCCTTACCAAAGACCATGTTGTTGGATGCCAAGGTATATGTACTCTT
1629	CaSNP3616	CTAACCAAGTACCACAAATTTAGAATGGTACCATATTGTGAAGTAGAGTCTAAGCTCATG[T/C]ATAACATTGTGTTTCCTTGTGATTATTTTGCTATGATTAATAGGTTTCGTGTGCGATAAT
1630	CaSNP3617	TTCACTAAACTTTAAAGCTTTAACAAATTGGAGCGGTTTGATTTTTTTAATAATCCAAAC[T/C]AAAAGGTTTCCAAGAGGTAAAGCAAGCTTAATCGGTGGGGTCAAAGAAAATTGCAAAACG
1631	CaSNP3618	GCACAATTGAGAGCTCATTTTGACCTGGAAAGTATTCTATGGAGGTTTGCTCTGCTGCCT[A/G]TGATCTCGAATGGCGGTTTGACACACAGTCTACCCGCAGACCTTATTCAAAGGTAGTCAA
1632	CaSNP3619	GATGATAATTATATTATTCTCTTGTTATATGTGGATGACATGTTGATTGTTGGTCATGAC[A/G]CTAAGAAGATTCAATCTCTAAAGAAAGATTTGAACAGGTCTTTTGCAATAAAAGACTTGG
1633	CaSNP3620	CAACGTTTCAACCCTGAAAAACGTTTGCATTAAACTCGATACTGATTGATATTTGATCTC[C/G]TTTCCAACATGAAGAAAATAGAGAACTCACTCAACAAGATTCCCAAAGTTGATTAGCAAC
1634	CaSNP3621	CATTTTACGTTCGAGTCCAGTTGTTTGGCTCAGAGCCTACTTCAAAACATACACCATTAT[T/C]ATTCGAACTTCACATTACCAATTCTCTTTTTCCTTGAGAGCCACATGATTATGACAATTA
1635	CaSNP3622	CTATTCTTGTGGACCTACTAGAGTCTGTTTCACCTTCTCTTTTACCACTAGAGCCTATTA[T/C]GTATGTCCCACCTGAGACACCTCTGGTGGCCCCTCTCGTTGTCCCACTTGAGACTTCTCT
1636	CaSNP3623	TATATTCCTATAATAATATTGAAACACGAACATACATTTTGGTTCAAATTATTATAGCCT[A/G]TTTCTCAAAAACAGAACAATTCCTAGGTCAAATTATATGTTGATCAAGCACCAAAAAATA
1637	CaSNP3624	AATATACTCAAGATTGGATATTGAGAGTGCAACCCGAGTGGCCTAATCACAACTGATGAT[C/G]CAACCGATCTTACATAGACTCATATATCATCATAAAATTTGGATTGAATTTAACTCAACC
1638	CaSNP3625	CTTGAGTTCGACACTCGGTACTACCGTTTTATTATTACTTGCAATGATTCAGTACACTTG[T/C]TGAAACACTATTAGTGACATACTATAATATTGATATCATCTGCTCTAATTGGCTCCATAT
1639	CaSNP3626	GAAGTAAGACCGTGGAAATGCATGACCATGCTAGAGCACGCCGATATGTGATATTCAGTC[T/C]ATTAGTTTTACGCATCAACTATGAGATCGAGAAATAAAGAATTGAACATTGCAAGGATGA
1640	CaSNP3627	TGCTTATTTCGATGGGAATTAAGTCTAAATTCGAATGAGACAAAAATAAGATTTAAAAAA[T/C]ATCTTTAGAAAAATTTATGAACAAAATACCAAACGTTTACCCATACGGATTCATAGTCAA
1641	CaSNP3628	TCATGATGTGCTTTGAGTGTAAACACTAGCCCAAACACTTTGAGTGTAAACAATTTGAGA[A/G]AATTTTATATCCTGGGAGGACTCTATCACTTATGATGCATTCATTAGTTAACTTGCCCTC
1642	CaSNP3629	ATAGCTAGCATGTGAATTATTAATTAATGTAACAATGTGTGACTTATATTGGTATTACTA[T/C]TTTTTTGCGATTGAATACATTCCTAGATTATTGGTAGTTGGAAGAGCTTAATGTTGTTTA
1643	CaSNP3631	ATGACTACAAAGATTGTATCCTTGCCTCTAACTCGTGGAAGGCCACCAACAAAATCCATA[A/G]AAATATCCTGCCACACTTGGCAAGGAATAGGCAACGGTTGAAGTAGTCCAGCCGGAGATA
1644	CaSNP3632	GAATAGACACCCCAACACATAAACCCATCTATGTGGCTTCTCGCACAAACTTCAAATGTA[A/C]ACTCGATTGGAATAAGAAAGAGTTTCCATTTTGAATTACGATATTGAATTCTTATAGTTT
1645	CaSNP3633	TGGAAATAGAGGTGGACTACTTTGTGATCCTTTTGTATCATTGATTAATCATTAAAATGT[C/G]TAGTAAAGATGCTTATGTTTTTATTGGTATATGTTGGCACTTTGTGCTGCGATGTGGGCT
1646	CaSNP3634	AATTAACTATCACAAAACTTAATTTAAAACAACATAAAAATTAAAATAACAAAGAATAAT[T/G]TACAGAAACAAAATTGAGACATCATTTCAAATACTTAAAATTAGTAATAAGTTAAAAAT

1647	CaSNP3635	TAATCTCGAGAATCGAATTCGTGCACATCGGTCGAAGGAAGGGATGCAGGATGCATCATT[T/C]GTTTATTTTCTAGTTCCGGGAAAAAGCATGGCATGCCAAATAGAAACTCTTCTCATCAAC
1648	CaSNP3636	ATACAAGTAAGAAGAACACGCAAACAATCATAATAGAATCATATCATTACCTAAACATTC[C/G]TCAAGAAAATATCACATTAAACAGTCAAAATCACTCAAGGTAAATCAATAAATTTCACTA
1649	CaSNP3637	CGCAATACGTTGTGCATGTAATGGATGCTGGCGCAGTTGTGCATGGACATGTAGTCTGCT[T/C]GAATGATTATATGAATTGGTTTTGCAAAATTTCTCATCCATACATCATTCATAGAGACCT
1650	CaSNP3638	ACAGTCGGGTATAGATTGCACACTTTAGCACCATCCATTTTCGGGGCTAGTTGATTCAAC[A/G]GGTGACTTTTTACATACTCCTTAGCGAATTTCGACTTTCATGACCATTGTCCTCCTGTCT
1651	CaSNP3639	TGCACCCGATGCATATCCCCCGCAAGAATTGAGGTCTTAGAAGCCGCCAAGATAGGCAAC[A/G]CTAACTGTGTAGACACTCTTATCGTTGAACAGTGTGCAACCGATCAAAAACAAAAGAAAT
1652	CaSNP3640	ATAATAGGAAAAATTGGTGAAGTCCAGAGTAATTAACTCTAGACAGTGAAAGCATTTTTC[T/G]TGTATCAGGACGAGCAATGTAGTAATGTTATTAAAAAATAGCTAGCTAAAAGCATAAAGG
1653	CaSNP3641	GGATGAATTCTGAGTTTCCTGATGATAACGATGATGTTTTGAATCAACAACAATTGGAAG[A/C]TAGGAGGAACAGTACTAGGAAATATGTTATTGCTTGTGCAATCTTTGCTTCTCTCAACAA
1654	CaSNP3642	AAAAGAGTATTTGTTTTGTTACTTGATGCAACTTGATGGAAGCTGAAACTTATATTTTAT[A/T]TTTTTTAAATTTTATTTTATTTATAATTTGGGTGTACTCGTTTGTAACATTTCAAAACAA
1655	CaSNP3643	ACTGGTGCCTTTTGGAGCAACCGCACCCTCATCCTTGGTCTCTTCTCCTTCTCACGGCCT[T/C]ATCTCGTGGCCGCATCCTCCTCCTTGGTCTCTTCTTATTCTCATGGCCTCATCTCGCGGC
1656	CaSNP3644	AGATAATCTCGTGAGCAATTTTCTGGATTTCATGTGACTAAGCTTCCACTGATCTGTCAT[T/C]TGTCATTTGATATTTGAGGTAGCGGCTAACAACATAATTTTTTTACTCCAACTTCCTCAA
1657	CaSNP3645	TGATAAATGTTCAGAGCCTTTAAGTATATTGGTTAGAGTTAAAGTTTCTAAATGTATAAA[A/G]TAATTGTATTTGAATGGGCAAATAAAATGGTTTCTTTTACCTTAGATATTGATGAAACAA
1658	CaSNP3646	GTTTTGACCTTTTGACAATCACATATTTTGTGCCTCGACATTTGATTATCATATTTAATG[A/C]ATTTTAAATAAAAATATTTATAACACAAGAAATTAGTAAATGAAGGTTCAAACAATGATA
1659	CaSNP3647	CAAGAGAATTCCTATACGATAATAGCTTAATTCTAATCTTAAATCTACTAAGGAATTAGG[A/G]GTTATATTTGAATTAAAGGTTCTGTCACTAAGACATTAGGGAGGTTCTGTCACTAAGACA
1660	CaSNP3648	TCATATAGGGAGGGTTTGACAAACCTAGTGATTCGGGAAAGTACAACTGAATGAGCCTGA[T/C]CGTGTAGGGCTACAGATGAACGGTAAGGTAAGACTGATAGACCTTGGGGGTACCTCTAGT
1661	CaSNP3649	CATATAAAAATTAGTTATAAGTACATGCGAAACAAAAGAAGAAAGTGGACGGCGAGGAAG[A/G]TGGATGCAGATTGAAAGAGAAGGTTGATGACAACAGTTGTTAAATGAGAGTGTTTAAATT
1662	CaSNP3650	GAAGGAATGATATGGAGTATGTCAATGTACGAAGCTTCTTATAGAGGGTGGTAAAGCTTT[T/C]GGAGGGAATTTGAAGTTACTACTATCACATAGATCAAAATTAACATCGTATGAATTTTTA
1663	CaSNP3651	AATATACTAAATAAATTTGGTTAGGTTTGAGTGATTAATTTTTAGAATTGTGTGTGCATG[A/G]ATACTCACTTTTTATAAATGAAAAAAATAATACAAATATTTTGGTATTTTGGATTTATTA
1664	CaSNP3652	TCACATGACTTCATTTTATTCATACCTGTCTTAGATACCCACTTAGAAGTTGTAGAGCTA[T/C]TATATAAAAGTATAAAACCATAACACATGTGTGGTCTTTATCCCATCTTTTAATTCTATA
1665	CaSNP3653	CAAAGTCTTTCTTTGCATCTTCATCACATTTGTTCTTATTATCATCTGATTCAAGAACAC[T/C]CTGTTGAAGTTCCAGAAAATCTGATACTACTTGTTCAGGCATATCCTCTTCAATTGATCC
1666	CaSNP3654	TCAATAAAGAAAGAAAGTGAGCTATAAGGCACTTTGGTGGTTCGTGAGAGTTGTAACGAC[A/G]AAGTGGTCATGAGTTTATTAATAGAGAAAAAAGATAAAAGGAGAAACAACAAAAATATGT
1667	CaSNP3655	TTATATACAAAAAAAATTAAGAAAGTATAAGATATATCCAAAATTAATATTATCAAAAAA[A/G]TATTAAAAGAACTAATCTTGTGAATCGGTAAGGGCGCTTGAAAAAAGGTAAAATAAATTA
1668	CaSNP3656	GGAGGTATTCATGTAACACAAGAACTTTTATAGCAGCTGAAATTTTTAAAGCAAAATAAA[A/G]TAAACTGAACCTCATCTTTACTATTTGCACGTGAACATGTAATACTACTTTTCTTAAATT
1669	CaSNP3657	TATTATTTCATTATACAATCGTAATTATCATTATAATATAATAACAATATAATCAATGAC[A/T]TGTAATCCATTTGCAAATCAATCAATCTATTTATCTATTTATTTAGAAATTTATTTTTGA
1670	CaSNP3658	AAAGTTGATTTAATTACCACAGTATTTAAGAGACTGAGATTCAGCTTGAGAATGAGAAAA[A/G]GAAAGTAGCATAAGAGTGGATGATAAGCATAAAATGAAGTTGAGCTTTGAAGTAAACTGA
1671	CaSNP3659	CATAAAATATTATTAAGATAATTCTCGAGGGCCATAGCCCTAGAAAAGTTTTATGTTTCA[A/G]TATCACAAGGGCCAAAGCACTAAGTATTTTATTCATTAAATATTTGATAGGGTTGTGGCC
1672	CaSNP3660	TGGAATGACACCACCAAAGCAACATTTGCACATTCGTCACTTTCCGAATCCGAGGATGAG[A/C]TGACTTTATTGTACTCCCATGCTATATAGGGATTTTTGGATTCTTTCTTCTTGTGAGCAC
1673	CaSNP3661	ATTGACCTGCAATCATTGCTCCTGTTATACACCTTGTTCCTTCGCTCCCCATTTTTTGTT[T/C]TCTTCTCTCTCTCACTTTAATAACTTTAATAATATGGAGATATTTCTTTTTGCGAAGGTT
1674	CaSNP3662	TTAATAAAGGCAACAAGATTACAACTTTGAGCATCAAAGGCAACAAGATTACAACTTTGA[A/G]CATCAAAGGCAACACACAATTTGGTACCCGAATTTAAATATTGAAAATTGATAATGCTCG
1675	CaSNP3663	TGTTCAAATCATCTAAAAATTGAAATGTATGATTGTCAAGATAATACAACATGATTCAAT[A/G]GTAAGATTCGTAAAATTCATATTCTAAATAAAGTTATTAGAAATGTAACTATGCTAAGCC
1676	CaSNP3664	CATACCTATGGCACTTCCTAACAAGTCACGTAACAACAGAAGACCATCATCAACAACATC[T/C]TACACTTCAACTTTGACAACATTAGTCTTTATTGCACTTTGTGTCTTAGGTGTATGGATG

1677	CaSNP3665	TGAGGGTTAGGTCTTGGAACATAGACTTTTCCGACCATGTGTTTGAGCATCCATTGTCCA[A/G]GTACCATAACTGGAGTTTTAACTTTGTTGCTAAGGATATCTGCAAGATAAACTATTTTAT
1678	CaSNP3666	AGATAGTTTAATTGTCTTGAGATGGTTAAAAGAATGAGATTTATTATCTCTCATATTTTT[A/C]GAGAAGTTATCAATGTGCTCACAAGAGTGCTAATTTAGGACTTTACATTGTTAATTCTCA
1679	CaSNP3667	CCCAAACTATGTAGTATACAACAATACAACTAATTTTGCTCTATTATGATTAGATTAATT[A/G]TCGAAGTGTCACCTTTTGAAGATTTGCACCAACTAATACAAAAAAAGGGGTTTAATTGTG
1680	CaSNP3668	ATCTAGTAAACTCAGAGACGATTAACAAAAACTCTAACTTTTAAGTAGCCAGCAACAGAT[A/G]CATAAATAAATCAACAAGCAGATGGTCATTGGTCAAAATGACACCACTAACCTTCCACTG
1681	CaSNP3669	TGCATTGGTTGAATGAAGCAGGAAGATCCATGCACTTGCAACCAGTAATCAGAGGTTCAC[A/G]TGCTATGCTATGCTACTGGACAACCCGACAACCATAGCTAACAGTTCACAATATCTCAGT
1682	CaSNP3670	TGTGTATATGGTTGTTGGTGGGAAGTGTCTAAAGCAGTTTGTGGAGATAGCATGCACCAA[T/C]TGTACACAAATCAAGCAGTCTTATTGGATTATGATTTTTGGTGGAATACATTTTTTTCTG
1683	CaSNP3671	AAATCCACCAAAAGCTTCATCCTCCATTGAAGGTAAAAGTGGTGCTGACTCATCAAACTC[T/C]GGCACAGTAAAAGCATCAACAAAAGGGTATGATGGTGTTGTAGCATCAGTTCCATAAGAA
1684	CaSNP3672	ATAAATATTGAAGGTGTAAGAATATATGTGTTTAAATTTAAATATCATTATTAGATAACC[A/G]GAGGACAAGAAGTTTGGAGCAACGCATAGTAGAGTGTTAACTGACATGAATTATTATTAA
1685	CaSNP3673	CTAAATTATTTGTTTTTAACTTATATGTTGTAAGATACTATTGTTGAACAAACATCTCAA[A/G]GATAATGTTAAGAGTTAAATAGAAGAAACCGACATGGATCGTTCCTATTATAAATTGTTT
1686	CaSNP3674	TACAAACAACACAGTAATTGAAGCTATTATTTAGTTGAAATAAAGCCTAAAATAACACTA[A/C]TTAACAAACTATATGCTAAACAGAGAAATAAATACATCAATCTATTTAACTCCTTTTTTC
1687	CaSNP3675	AATCTAAATAAAAATAATGTCATTTTCTATTTATTTCCTAAACAAAGTGGTGTCAACCTG[A/G]CCCATATCAAAACTATTTTCAAGTAAGATTTTACTTAACCTATCTTACCAAGTTCTAGGT
1688	CaSNP3676	TGTCTCCAACCAAGTTACCTGGAATGAAGTCATAACAATATAGCAGTTAATAAACACATT[A/T]CCATTCAACAAGGTAAGTAACCACAAGGGACAGATTTGTTACAGCAGCTGGCTTGTAAAT
1689	CaSNP3677	TTTCCTGACCAAAATTTTCAATTATCCTATTTCAAGTTTTCAACTAGTATTAGACCACCT[A/C]TATTAGATCTAAGTATGATATGTAAAAAAATTTGCAAGAAAAATGTAACAACTATCTATC
1690	CaSNP3678	TTATTTTTTCTAGTTTAAGATATACTTCTGAATATTCTAATTGTATTGTATGTTATTATT[A/G]TTTTTAATGCATGCTTCTTTCTTTTGTAAAGGTAATGTATGATTCTAGCACATAACTATT
1691	CaSNP3679	TTATTATTTTCTCTCGTCCTTTTCTCTAAGATCCCACAACTTCAACATGGTATCTAGAGC[T/C]GATTTCGATCCTCCTTGAACGATCCCGCCTCTATTCCGCTGTCAAGTTCCCAATAGTTAG
1692	CaSNP3680	TTCTTCTCAGAGTCTAATTACATCCAAATATATTATAATTTTTAAGTCAAAATTGAGTTA[A/G]AAATTCAAATTATACCGTGATAACATTTATGATTTGAACTAACCACATGCACTTTAGAAG
1693	CaSNP3681	AAACTTAGATTACATATATCTGAATTTAATGCAGATAACTTATTTGGATGTAATTGAAAA[A/C]AACATAGGTGAAACTAGATACCATCTGATACTCTTTACTTATTTATATTACATGAAATTG
1694	CaSNP3684	ATAATTGAATCTTGAAAGCTCAAGAACATTGCATTCCATAAGACACAAAGGATTGATGTG[T/C]TTAGTCTTTCCCTAGTTAAAGGTTTGATGCCAACATAGACGAGACGTATGTGTATGAAAT
1695	CaSNP3685	GTAAAACTCTTAAGACGATGGTGTATATATTGAACCATATTCCAACCATGATTGTTCCAA[A/G]GAAGCCTTCTAAGTTAACCAAAGGTTCGAATACGAGTTTGCGTCATATATGCGTTTTGGG
1696	CaSNP3686	CCATTCAATACATTGCTCAGTCTTATATAGGAATAGTATTTCTTTTAATGTTGCGAAATC[A/T]AAAAGCTTCAAATTGATGATTGAAGCTGTTGAGAGTTATGGCCTACACTTGAAGCCTCCA
1697	CaSNP3687	TCATAACATCCGTAACCACTTCATGTACCATCATTTACGTATCATGAATCAAATTATAAT[T/C]AATCATCTCTAACAAAGCATAAACCCTGAAAATACAGGAGAATAAACATTGAAATAAAGA
1698	CaSNP3688	CAAGAATTATGTATCAATAAAATTTGAAAATTTTAGTATACATTGGAAATATTTGAAAAT[T/C]GAATGTGATTTTTGCACGAAAGGGGAAAGTGATAAATGTATAAGTGTAAGTATATGAAAT
1699	CaSNP3690	ACACGCAAAAATATCATAATCGAACCGTATCATTACACAAACACTGCTCAAGAAAAATAT[A/C]ACGTTAAAAGTCAAAGTCACTCAAGGAACATCAACATTTTTCACTATAACATCAAAGAGA
1700	CaSNP3691	TAATATTCCATTAAAGGAATGAGGGAAGGGGAAGTGGGAAAGACATTCAATTTCAAGAAG[A/G]ATGAATGAAAGGAATAAAAGCTAATTAATAATGTCATTCATTTTATGTTATAAGAATGAG
1701	CaSNP3692	TATTTTACACATTGACATTGTTTGAGAGTTTTGTATGCCTTATAAAAACAGAGCTAAGCC[A/G]TCGAAGACTGACCATGAAGCGGCGCTGATTAACCCAGATCGGTCAGTACGGCAGCTGAAT
1702	CaSNP3693	CTTAAACCTTTCGTTTAGTAACCTAGCCCAGTAGTGGTTGCATTCTTCAACAACTTGACT[A/G]TACTAAGCTAGTGGAAGTGTGATTGAATTGGTGAATTAGTGGATTAAATCCTTCAATTTT
1703	CaSNP3694	TTGGGAGTGGATCTCCGCCGGATCGGTTGATGTTGGAAATTTATCAGCATTTGGCCAACA[A/G]GGTTAGTCCTGGAAGCGACAGATCAAGGAGGCAGAGAAGGAAGGAGAAGGAAAGAATGGC
1704	CaSNP3695	TTGAAACATGAATGATGGCAGAATAATTAATGTGTTTGCATATGATGATCAGAATATTAG[T/C]AAATAGACAATCGACTCAAAGATCAAGAGTTAGGAAGTTGTAATACATGATATAATTGTA
1705	CaSNP3696	CTTGCTGGAGCAATAAGGTTAAATCTGTGAGTCCTTCAACCACAGGTTAGTTTCTTCTCT[A/G]CAATTGAACTGATTTTTTCTCTCTAGATTTTGATTCTGTTGAGGGGTTTGTAATGATTAT
1706	CaSNP3697	TCTCTCGAATATCCATCCTCAATTTCTAGAGAAAAGTCTGTTTGTCAAGAAGTCAGTTCG[A/G]TAAATGATAGGCCTGATGTAGTAAGCCAGATAAAACTTGCTTCTACATCCACCGATGACG

1707	CaSNP3698	ACTCCTGCTTGACAGTTGATCATCTCAAAGATCTTTTCCACTTCTTGGATCCACTTATCT[A/G]CTTTCTTTGAATTCTCATCACCCCTAAACTTGGGAGGATTGTAACAACGAAAATCTTCCA
1708	CaSNP3699	TATGAGATGAACAAGATAACTTGTCTCCAAGCATTTTTTATATGCCAAAATGTCCCACAA[A/G]GGCCTAATTAAGAAGGAAGACTTTTACTTATTATAATTGTCCTATTTATGTTTTGTATGA
1709	CaSNP3700	GGTCAAAACTGATATAGCATTATCAACAACTCTTTTGACCATTATTAAATGCTCTAAAAC[A/G]CCTATAAAAGGAAGGCCAGTGCTCAAGGAAATAACAGAGGTTCAAGTGCTAACTAGTCTC
1710	CaSNP3701	TTAGCTATGATTTTCTTTCGTTTCCTAATTGAACTAGTATTAATCGAACAAATGTTGCAT[A/G]TGTTCACTTATCTTGATAACTATATAAGGAATATGATATTAAAAATTTAAATAATTTTGG
1711	CaSNP3702	AGTTGGATCCTAATGGAGTAACCTTAATCACAAAGTATCATTGTAAGTAAAATTCATAAA[A/G]AATGGTGTAGGACATTCCTGGTAATTTCATTCTTCCTACAAAAGTTTTGTTAAGATTTTG
1712	CaSNP3703	TTTCGCCAATGAGTTTCCAAAAGTTTGGGAATGTATTTGCTTGATAATTGGCTATTGATC[A/G]TGGTTTGATTCTAATAGTATGTTGTTGTGTTTCTGTTGCTAAAAAAAAGAGTTAGTATTG
1713	CaSNP3704	AAACAAGCCTTCTACGCTATCTCTTATCAATTTTATTTACTCTATACATTTCGTAGTTTA[A/G]TAGCTCAAACACTCACACTACTCAATTAACACTCTTTGGAGTACTTAAGGAATCCATGCG
1714	CaSNP3705	TCAAATGTTGAATCCATAAGGCTTCTCATGTCTCGTCATTCAAGAAGAATAACAGGAGAA[T/G]GGATAATTGGAAAGAGAGTAATGACTATATCAAGGACAAAATGGGAGCATACACATTTAT
1715	CaSNP3706	AAATTATATATTTTAATTATATACCCCTTAGTCATGGACTGCTCCCCCCAGGTTAAGGTG[T/C]TAGTGAATATGGCTCAGACAGGAATCGACATCTCATGGATAGATGTTGTCTGAGTTGTGG
1716	CaSNP3707	AGTTGACCAAACAACCCATCAATGCGTGGCAAATGGTATTTATTCTTTACATTTACTTTA[T/C]TCAATTGCCTATAGTCTACATACAACCACATGGATCCATCTTCTTCTTTACAAATAATAT
1717	CaSNP3708	CTGTTAATACAAGCTCACTCTAATTATTCTACATCCGATAACCTTTGTATAAAAATTGAT[T/C]GTTTGATAAAGTCAACACTCTGATAAATTCAACATTCGAACATCATTTGCTCATCATAGT
1718	CaSNP3709	CTCAGTCCATCATCTTCAGCATAGAACCATTACTCTACAGTTTGTCTCTTCTTCTTTACA[A/G]ATTGCTGATTTATTCACAAAGATGCATTCCATTAAACGTTTCCGTTTTTTAGTTGAAAAA
1719	CaSNP3710	TAATATTAGAATATTGTATCCTTTTACATTTTTTGATCCTTCATTGCAGGCTTGACTGGA[T/C]TGTTATGTCCTTGTGTATTATTTGGGCGCAATGTAGAGAGCTTGAGGGAAAACACTCCTT
1720	CaSNP3711	TAATAATGATGTCAATTGAGTGAGCGTAACAATCGAGTTTGGAAATGGCGCTATAAATGA[T/C]AGTGGTTTTGTGGGAAGAGTTTACGTGATGGACCTATGTGTTGGGAAATCCCACCCAACA
1721	CaSNP3712	CTACATTGAATGTTTATTAATGGAAGTAAAAAACTGCACCGTTTTATTATTAAAAAGTAA[A/G]TTTTTTAAGGCATAATTTGTCTCATTAGTTAATATTTAAATATTGGGTCACTGAAGTTTG
1722	CaSNP3713	GGAAGAAGATGGAGAAATGAAGAGAAAAATATGTAGTAGCATTCAAGAAGGAGAGAAAGG[A/G]TTTATGAAGAATAGAAAGATAACTATCAAAATGACTAAAATGCCCCTTAAGTATGTATAC
1723	CaSNP3714	TGGGATTTTTTCTTAAAACGAGTCTGGTGAATAAGAGGGGACATTGCATTAGTACATTAA[A/G]GGCAACTATATGCTCTGATTGTAGGAACAATGGTGATAAAACCCCATAATAACATTGTTA
1724	CaSNP3715	ACAAAAATACATCCAAAATTAAAATAATATATCTATCATCAATTATCATAATAAGTCATC[A/T]CATTAGCACCACTCCTTGCATCTCATTAGCCAACGACAAAATCCCTTCAAAAGTAAATAG
1725	CaSNP3716	CTGAAGAAGGCAATATGTTGAGACTATTTCCATTTTCATTGAAAGATGATGCTAAGGAGT[A/G]GCATAATTCATTTCCTGCTGGCAGCATCACCACAAGGGATGACCTTGAAGATAAGTTCTT
1726	CaSNP3717	CTTGAAGAATGAAAAGAGAAAATGGCAACTCATGACCAAGTACCTATGAGGTTTGAACTC[A/C]TGCACGAAGATGGACTGCATCAGTCCAAGGACAGTTGGATGGTAATTTCGTAAATATCAG
1727	CaSNP3718	ACACGATACTTATTAAACAAATGCAACAAAGATAAACATATATTAGAGAACAAGATAGAG[T/C]AGAAGCACTTGTCAATGTGTCTTGGTTCACTATCAATCTGATAACGATAACACCTTTAAA
1728	CaSNP3719	TGCCTTGTGACACAATTGTGAATCCGCGAAATAATGTCAATGTCGTGACAACGAGGAGTG[A/G]CAAGGTAAGTAAACCAGTACCACCTAAAGCCAAAAAGAGTGGGAGTACTTCAGCTTCAAC
1729	CaSNP3720	ACTTATGGACATAAATGTTAACATTCCTCCTCTGATTGATGGAGGGCGATTGGAGTACTC[A/G]AAAGCATACTCATAAGTTTTTATTTATCTCTCACGATCTTGTACTATGGGACATAGTGGA
1730	CaSNP3721	AATTATGGCCCTTGATCATGTTTATTCTAGTTTAATAGTCAACTTTAAATTTGTTACTAT[A/G]TTACTAAAGCTTATTAATTGGAAACTCAAATTTGAATTGTTTTTTAATCAATTATATTAC
1731	CaSNP3722	TAAAAATTATAATGCTTCAATGATATGAGTAGAAGAAAGACAACATTTGAGAATTAGAAT[A/G]TATCCTTTTGATACATACAGTAACTAGCTGTCTCATTCCACTCTCAAAGGTCATAAATGT
1732	CaSNP3723	ACGTAGATTTATCCTGGTTCACCACTTACTTGGCTACATCCAGTCCCTTCATTCAGAAAG[A/G]ATTTAATCCACTAATTCAAATACCTTGATTACAAAGCACCTAACCCTCCAAGCCAAGTAT
1733	CaSNP3724	ACTCTTTGCCTCTCAACAGACCCCCAAACAGTCTCCATGGTATCAATTCTATGAACACAC[T/G]TAGTCCTTTATTCAAGCTTTTCTAGATTATGCTGACAAAAGTATGTTTCGGCATCAAAGT
1734	CaSNP3725	TATTGAATGATATCACAAAGTAAAATATATCACAATGCCATACCGATCATGCCCATCAAG[T/G]ACACAATTGGGAACAATGTCAATAAATAAAAAAGTTGCAATTATTTTAATTTGTGTATGC
1735	CaSNP3726	TAAGTCCGTCATGTCTATCCATTGTCTGTGCCATGCAGCTGCAGCCAGAACTACTCCATC[A/G]CAGATTCATAGTCTCAAATGATCCATTTGAACCCATGTTTTCAACTAGAGTTGCTGAATC
1736	CaSNP3727	TACTTCAAGACAGCTGTCAAAGATTCTTAAACTCAGCACTTCTCAAGACAGCTTTAGCAA[A/G]AGCATTCCTGCTTTAAGAGAACACTTATCCACTTAAACTGACATAAGACAGTTGGACCTT

1737	CaSNP3728	GTATTGTTTTTTAAATAAAAAAACGTGTAAAAAACTAATTCTTCTAACACATAATTCAAA[T/C]TACTTAAATTGTGACTGAAATGTTTATCATTCTTTGTCCATGTTTGGGCGCAATATTTTC
1738	CaSNP3729	ATTCGTACCATTTTCACCCTTAACTTTATACATCAAATTATTGTGCCAACTTAATTACAT[A/G]TTTAATATTTTTTTCTGTATTTTATTATATATATAGCTGAAACAATTGGTACTAATTTTT
1739	CaSNP3730	ACCTCTCTCCAAGGTAGCATCAAGTATAGACGCTGTTTTACTTTCACATCCTGATACTCT[T/C]CACTTAGGTGCATTACCCTATGCAATGAAGCAGCTGGGACTATCTGCTCCTGTTTTTTCA
1740	CaSNP3731	TTCTGCCAAACATTGGGTAGGATATCGACTTTAGAGTATATTTCTTAGCTTTGGGAACAT[C/G]TGTGCTTTACTTTCTTATACTGCTGCCAAGAATATGAATGTTTCCAACATTGAAATCCTT
1741	CaSNP3732	GAGATTCTAGATCAAGTAGGGAAACAGGGATGCCATGGAATATGCATAAGATGAAATAAA[T/C]TATGGTGCAATTAATGACACATTGAGGAAAAAGGATCTGGAAGACACCCGATGACTATTA
1742	CaSNP3733	ACGAAGGGAAAACATTATTCTCATGTCTTAATATTCTTTTAAATTACGTCTCTTAAAGAA[A/G]AAGAAAGATTTGGATTAACTGCCTCAATTTGGCCATCATTTTGTGGGTGGCAAGTAGTAG
1743	CaSNP3734	TTAAATAGAGGCGCCTAAATAGGGGTGTTGTAGGTAGACCCATAAGACTTCATGTAGATA[A/T]TTAAATCCATTCATATCATGAGATTCATAAGCTTGGCCACTAAATGTTAATATTTGAAAT
1744	CaSNP3735	CTCCGCCTTCCAGCAACAATAGCAACCAATGTTTCAGCAACAATAATATTTTAATAATTA[T/G]CAATATGTTATGTCATTCACAACAACTTCCTCAACCTCAAGCAGATCAAACTCCATACCA
1745	CaSNP3736	AATGTTTTTATAAAGTAAATAGACTCTAAGTTGTTCTTCTATTTTATACTTTGGAGATCT[T/C]TTATTATGTGAAGGAATATGGCCTAACCAAATGTATCATACCACTAGTTAAATGAGAGCA
1746	CaSNP3738	AATCAAACGACTATTAATGATGGTTCACCATGAACCATTTAAATGTTGGTCCACTCTACC[A/G]AAAATAAAAAATAAAATATGTAGAAATTTGCGACCACCGGTTATTAATCTGGGTTCACTAA
1747	CaSNP3739	CACATCAACCACAAGAGGTACTACATCAAGCGGTGGCATCACGACCTTTCAACATATGGG[A/G]ACTTGACATTATTGGACATTTTGCCTTGTATCAAGTCAACAATGAGCTGCAAGTCTATAG
1748	CaSNP3740	CTCATGCGAAAAAACATCTAATATTGATGCTCATTCTTTGACTAAGTTACATTTAACTTC[A/G]TATGATTCTTCCATAATGACTGATTTGATCGTCTAAGGAATGAACCCCTACATTGGGAAG
1749	CaSNP3741	AGAAAAAATCAAAAGACTGATGAAGAAGCATATATGGCTCATGGAGATTATTACAGTACA[A/G]TGTTAATGATGACTACTACTAGTGATGAGAAGATTAAGAATGAAGAGTGGTTTCTTGACT
1750	CaSNP3742	TGTAAGTTCTTATCTATTCAAGTTAACGAGCTCTCCTATATATGTAACTTGTAAATTAGC[T/C]TCAATAAATTTGCAAAAGTGTTTATGTGGTTGTTGGGTACAAGTTTCAATGTGTTAGAAC
1751	CaSNP3743	TTTCAAGAACCGTATCTGATAGTGGACTTCTCGTTGGATTGTGTAACTTCACTTTGTGTA[C/G]TCATAATATTTGTTCAGTAGCTTATTCAATTTTGGTTTAGTCAAACACCAACAGAGACTC
1752	CaSNP3744	AGATGTTTCGATTTTAGAAAACTATATTTTGTTTCAACCTTAAATTTGTTAATTTGACAT[A/T]AATTTTGATGCTAATTTTGGTAGGCCATGGACATAATATAATAGACATGTTTATATTAAA
1753	CaSNP3746	GCCAATTAGACCTAATTGCTAGAAGTTGAACACATATTTATAACATTGATTGGGTAGCCC[T/C]CTCGTAGATGGGCTTGTTGGTGGATATCAACTTGTCATAAAATCGTTTTGCATCTTCGTT
1754	CaSNP3747	TAGGAAAACGTATTAGAGAGGGTTTAGGGAAAAACACATTAATTGAATAATTCACCTATC[A/T]TAGTTAACCCCCATTTCTTAAATCTTACACATCATAATCATCACACAATCATATGGAAAA
1755	CaSNP3748	CAACCACGATGCATATCTTATAGTATTTGTATAGTCCTTTCAGACTAGCCATTTGTTTGA[T/G]GATGAAAAGTCGTGCTAAGTTCTTAATGAAGTTTGGAACGACTTCCAAAATTGAGCAGTA
1756	CaSNP3749	CAAGATCAACCAAGTTAATGGTATCAAACTTGGGGGAGGGGGGGATAGGTTTTATAGAAG[T/G]GGTTATAAATATTTAAAATTCGTAAGTTTAATTTGAAGAATGATAGTTTATGGATAGTAA
1757	CaSNP3750	AACATAGTTAAGCGAGAGAGAAAAAATGGTTCTTGTGAGAAAGCTGAGTTACGCGAGCCA[A/G]GATCATGCGAGAGAGAAGATGCAGTTTCACGCTAGAGAGAAGAGAACAATTTCGTGCAAG
1758	CaSNP3751	CATTCCACTCCCCTCTCATATTACTAGCATCACCATACCCTTATCGATTGATATTAAAAA[A/C]ATCAAGACCTTGCCGAGAAAGAACATCACAAATGGATTTTTTAAGAGTGATAGCCATAGT
1759	CaSNP3752	AATAATAATTATATATATAAACTAGTTTTTTCTTTAGTGCCTTGAAGTCTTGTTACCTAC[A/C]GATCAAAATTACTTCCAAATTTAAATTTGATATAAAATAATTAAATATATAAAATGACAT
1760	CaSNP3753	GATGAGTGGATATTAACAAAATTGTATTTATTTATCTATCTTGCTAACCTACCTTATTGA[T/C]AAAATTGTACATCTTTAACATTGGTCTGTTTCTGGTTACCAAATGAAATTCATTAATAGT
1761	CaSNP3754	GGGTAAGTTTAGTAGTGTTTTTTGCTTATGAATGATTGTATTGATAATAAAAGATAAAAG[T/C]TTTGACATATGATCTGACTTTGATGGAATTTTCAGGTATGAAGAGAATAATTTAAAGGTT
1762	CaSNP3755	AATCAAACTTTAGGTGAGGTTTTCATGTTAACCAAACAAGATGTACATCTAGAAGGCTAG[T/C]ACTACACAACCTCCTGCTAATCTTAAGCTTTGCTCACACCCAAGTATATAGAGTTTCACT
1763	CaSNP3756	TCACTTGCTCAAAATATATCTCACTTGTTTTCCATCGATAATATCTTTAATGATTAAGTT[T/C]TCCCATTTAGAGAGTCTTTCACCTTTCTGACATTTCTAAAATTGATTTGTGGTTGGTGAG
1764	CaSNP3757	CACAGGCTGCATTAATCCAACAATTCCAAGTCCCTCACTCTCAAGCTCCTTTTCATCACA[A/G]GTGCTTACTTCCATCCCTGACTTAGAGTCCAACTATGTCATGCTTTTTCCTACCCCTAGT
1765	CaSNP3758	TGAAAGATTATCATTTATTGAAGAGGTGAATTTTGATCAATATTCTTATTTAGGTTACCA[A/T]AATAAGAGAGGTGCTGGCTGGAAATGTGACCAGAGTTTCCCTATTGGAAAGTGTAGTACT
1766	CaSNP3759	TCCAAAAATATATATTTGGCATTTAACACAAAATACTAATACAAATTGGCTAAAAAGCCT[A/C]AAAAATTCAACATAAAAGCACACCAAAATACAATAATTACCAATTAAAACTAAGGGTCTT

1767	CaSNP3760	TCATTCTCCAAACGAATTTAAATAGAATCGAGTCTCCAGATCAATGACTTAACGTCAACA[A/C]ATTTATGACAAGCATAAAGGGTCATTAAACACGAAATAAATCTTATCAAGAACGAATAAA
1768	CaSNP3761	TGCTTCATTATACCTTCAGATTTCAGTTTCGCAAAGGGAAAAAATTAGTGAAAGCTAAAC[A/G]TATAAAAACAAATAAATGCATGTCAAAATCAGCAAGTTTAACCAAGACGCACACCTATTG
1769	CaSNP3762	AATTAGCCATGGTCTTCCCCTCCCTTCCAATTTATCTAGATCCAACCAACTGGTCACAAC[A/G]GGTAATTAATTAACTCATCATGATATCTTTATTCTTAAAATTTTGTTTTATTTTCTACTT
1770	CaSNP3763	ATAGAGAAAATGAACACTAGAAAAAAACTAGTTCTCAAAACAAGTAATCAAACACTATGT[A/G]GCATCGACACTTCAAATTGAATGCATATTGAGTGTTTAACATAGTTCGGTGTCAGATACC
1771	CaSNP3764	ATTGAATAAAAATTAATAGATATTATTTATTAAATTACAAATATAGATGATGCATATGTT[A/G]GTATGATAATTGAATGGATTCACTAATAATTTATAATAGTTAATATAATCTTAAACCGCC
1772	CaSNP3765	GGGTGCTTATTAAACTTGTCAAAGATTTGAAGTTGGAGCATCAACATAAATATGTGAGTA[T/C]TTATTTTTCACTAAATTCCACTATGTGTATAATTAACAAGGATTGATCTTGACTTGACTT
1773	CaSNP3766	ATGATCTGATATGGCTTTGAATATTTAGCATCACTTCTGATAATAAGAACCAAGATATTA[A/G]TTACTTACTTTGTAAGGATTTACCTTCTAAATTTCACTGCTGTCTTGATCAAACTATATC
1774	CaSNP3767	TTATATGTATCAATTAAAGATCGAATTTTAAACTAAATGGTATAAACTAAATGATTAATA[A/G]ACTCTAACATTTGTCATTTATCACCTGTGTCACACCATACAAATTGTTTTTTTTATCAGT
1775	CaSNP3769	CATGTATATCATGTAAAAAAGCAAGACGACAGCAGATAGGAAAGCTCCAGAAATTCTATT[T/G]ATAATTGAAAGCGTTGAAGAAAGCTGGGGCTGATAAACAGGAAGATGAGGAGATAATGGG
1776	CaSNP3770	ATTGAGCCTATAAATTACTTTTGTTGTTAGTGACTTCACATGCTCATATTTTATTTCTTT[T/C]AAATTGGTTTTCCACGTGATAGAAGTTATGACAATTCCTTATTTTGTAGGAACAAGAACA
1777	CaSNP3771	CAAAATATATACACAAATTGAAAAATGTTATTTTTTCGAGAGAAAGTCTCACTAATATAT[T/C]GCCATGAGTGTCCAATGCATATGCATATGTAAGTATGTCCATGGTCTGTGTTTCTACTTC
1778	CaSNP3773	CTCTACCTGAAAACTATCTTGAGGCTCTGATTCAAAGATCAAATGAAGAAAGCCTAAAGT[T/C]TGCCTCTGATAAGTGTTACAAGCATCTAATAAGTTTGTCTCAGATAAATGTGACAAGAAT
1779	CaSNP3775	AAACAATTCATTATGAAAATAATTTTTAAATCTAATGAATCATCCTTGTCATTTACTTTG[A/G]AGTACACTATTTAGTTTTCTCCTTTTTTTTTTAATAAATAAAAGATAAAGTGTATTCCAC
1780	CaSNP3776	AGTGTTTATTCTTCGACTAGTTTGGTTGATTCTTCATCTCGAGTATATTGGAGGCATTTG[A/G]CTTTACTTAATAATGATGCGACATAGGTGGGTAAAGAAGTATTAAGTGTTGTAAAAGAGG
1781	CaSNP3777	ACATCCATTTCCGGTCCATATTATATGACCTATATAAATGCAGTTACAAAAATAATAAGC[A/T]ACTGATAACATCAACCATTATGTAACAATTTCAAAACAAAACAGATCAGCAAATTTCAAA
1782	CaSNP3778	ATGTATATCTTTGAGATCATTAGTTCCTAAAATATTGGTGTAAAGGTTACTTCCATCGTC[T/C]ACAACAAATCAAACCAACCAATAAATTAAAAGCCATAAAATAAAGTACACAAATCTCTCT
1783	CaSNP3779	TTTAATCTTTTATAAGATTGTCATTGTTGTCTAGTAGCTTCCGTATACATGAATATCTTC[T/C]GTATATACATGAATATGATGGTATATTGTCCTATGATTTGGTTTGGATTTGGAATCAATC
1784	CaSNP3780	CCGATAAACAATATTTAACATGCTTTCGAAGGATAGTATTACTCTTCTCCCTTCTATATG[T/C]GTTTTAAAAGGAGAGCCGATAAACGTGAATGTGTTGTTTTCTGAAGCTGTGGCAGCTTCT
1785	CaSNP3781	TCCTTTCACCCCTTACCTCGATTAAAGATTTCGTCCAATGAATCCGATCTGCTAAAGAGT[T/C]GTGTCTTCGTCGATCCTTGTCTCTAATAGTCCGCAGAGAAATGATTCAATGATATAACCA
1786	CaSNP3782	TTTGGTCAACTATGTTTATTTTCTTTTTTATTGGGTTAAATATCGTATTAGTAAGACAAA[A/T]TTTATCCTGTATCTTCCATATTATATTCCTATTTTCACAAATGTTGTCAAATGATACTTT
1787	CaSNP3783	AGATGTACACGTAGTTAGACATATCTGACCGGTGCTGTGTCAGAAGGATCTTGCAGGGCG[A/C]GTGGAGATCACCCGGATTGTATAGTTTTTTGAAGCATGATCAGATTAGATGGTTGTATAG
1788	CaSNP3784	TCATTTCCTTGCATTTATGGAATAAAGGTAAAATGTGTGAGGTATAGAGTAAAATTTTCT[A/T]AGGGTTAATAATTAGTAGACTTGTGAAATTGACATTTATACATGTGAAAAACTCATGTCA
1789	CaSNP3785	TTTTATCTTTATTTTACTTAGTATGCTTTTAAATTGTATTATTTGTGTGCCGAAAAAGTA[A/C]TTCAGTTGTGTGGATTAATATATTATAAAAATCTTATATTATGTAGAATATCTAGGGTTT
1790	CaSNP3786	AATTGGTCCTTCAATAGCAATTAATGAAACCGATTTTGTTCTTTTAAGGATTGATTGGGT[T/G]TCACTCTAAGGACCATATTAATGCAGTTATAAATGAACCACCAATATTGGCCTTTGATCT
1791	CaSNP3787	TAGCACCACGTGTCATTCCCGGAATTCTCGAGGACATATTTTTTCTCGTATGCTATTTTC[A/G]GTCTTTTTTGATATAATTGTCCATGGCATAAGGCTGCCAACCTATTTTCTTCAATTAAAT
1792	CaSNP3788	TTAATTCCAAAGTAACCCCTAATTTCTTAGTGGATTTAAGATTAGAATTAAGCCTTACCG[A/T]ACATAATTTCTCTTGTAAACTATTGATTTGCAACTAATTTAATTGGTTTCATGACCTGCA
1793	CaSNP3789	CAGCACAAGTGGTGGAGATGGAGAAGGTTCAAGCTCAAAGTCGAAGAAGAGGTTTAGGAC[A/T]AAGTTCACTCATCAACAAAAAGAGAAGATGTTTGATTTTGCTGACAAATCCGGATGGAAG
1794	CaSNP3790	GAAGATTTTTCATCTTCTGATTTGTCTGCATCTGCTTCCTCTGGTTCGAGTAAACCTGCA[C/G]TATCCTCAACTTGCATCGACTTTTTAAGGTCAAGTTGTTTGCCATATAACATAATATAAA
1795	CaSNP3791	AGCGCAAGGATTCGATACATGGACTCTTGAGGTTCGAGGTGCTGGGTTGAGCTCATATGC[A/C]GACAGCTCAGAAAAAGATGAGAAGTGTCTGAAGAATTCGTCTGATATAGATTCTGCAATC
1796	CaSNP3792	AATTCTTGTTATGGCAAATAACAAGAAAAAGAAATTCATCAGAGCAGTTCTGAAGCCAAA[T/C]GACAAACAACTAAAGAATAAGAATCGCACTTCAGAGAACAACAACAAGAATGGGAACCCT

1797	CaSNP3793	TTTTGGAGTCTTTCAAACCGCACCTTTAACACTCCTTATAGTATGCTCACCATAGCTTTT[T/C]TCGATATGGCTGCTATTACATGCCTCAAACCAACAGGTTGAATTATGACCTTAAGTTAGT
1798	CaSNP3794	AATTTGAATTTCAAAGATGCTTATAATTTTCAACGGCGTTATGGAATTTCTACTATGTGG[A/G]CTAAACTTATATGGAATTAATGTATTCCTCTATCGTGTTCCTTTCTTCTTTGGAGAATAA
1799	CaSNP3795	ACAATTTTACCTTTTTAAATATATCAAGTTTTGAAGAAAGGTAAGAGATCGAACAAACCA[A/G]TAGAAGATCAACAGCTCCCTTCACATAATCTCTAATACGGATCATTACTTAAATATGTTT
1800	CaSNP3796	TGTTTNNNNNNNNNNNNNNNNNNNNNCGACTTTGTTCAAGATGCATATACCAACTTGACGA[A/T]GCCCCACTCAAACTATCTTGAAAGAAATGAATGAGAAGTTTATCGTTATGAGCATGAGAA
1801	CaSNP3798	TCGGAAAAAAATGATATACAATCTTTCTCAGCTTTATTCTCTACGTTCTCAGACACTAGA[A/G]TAACTTCTAGAAACCAAACAAACCGTATATTTCTCAATTAGCATATTAATTCAACTAAAA
1802	CaSNP3799	TAATATTTAGTCTAATATCAATTAAATTAAACTCTATAATATTTTTGAAATATCGTTGTT[A/C]TGCTGCTACTAGCCAATTGCTTTTGGTTGATGGTACAGAGTTGCATTCCTTCGTATAGTT
1803	CaSNP3800	ACCAACATTGCACACGCTGTCACGCGCGTCCGTCTTCTCTATGTAACGCCTCCACCAAAT[T/C]CCTGCGTGAGAACGCATGGACCTGTCTCCAACCCCCTTCGTCTTTTCATGATGTGGTGAT
1804	CaSNP3801	TGATTTTAAAATGTGTGCGGATGGAATTTTGAGATTTAAGGAGAGAATTTGTGTTCCCGA[T/G]GTAGAGGAGTTAAGAAAGATGATCTTAGAGGAAGGAAGTATTCACCTTGGAGCCACAAAT
1805	CaSNP3802	CTCTGAAAGAACTCGTGATTTAGGGAAAACATATGATTAAAAGAAAAGATGTAACAGCTA[A/T]GTCACAGACCTGGAACGGAGCCTCCACGAGTTTAATCAGATTCGCCAACTGCCGTAGCAC
1806	CaSNP3803	AGAAAAATATAGTAGAGCTATAAATAAAACTTGTTTACAAGTAAAATTAGAATGACACGT[A/G]AGCATATAATTGACTTCATAAAAATAGAATTATTTGAAAAAATTAACAATGAGATGATAT
1807	CaSNP3804	ATTATTATTATTATTATTATTATTATTGTTCATTTTTCTCTCTTTCAGAGGGTCAAGGAC[T/C]GAGGGATAGTCGGAACCTTGTAACAGAATGTTAAAATAAATTCAACTAATTACAAAGCTT
1808	CaSNP3805	CAGGCATATCTTGATATGACCATGCAAAGACACCCACATAATTAAATAAAAGTTTAACCA[A/G]CTTTTCTCGTTCACTTTATTTCATAGACATACCAATTTTAATTTCTTTCTTATCATGCTC
1809	CaSNP3806	ATAATTTAGATCGAAAAAAAAATGTAAAAATATAAATCATACCGAGATCTTCTTTGATAG[T/C]GTAGTGAGGTTGTGATTAGTGCAAATTGGAGCATAAATGTTGTACACATCAAGGAACAAT
1810	CaSNP3808	ATCCAATATTTCCAACAAGTTAATTCTTCAATTAGTGAATATGGTGGTGGGTATGGAATA[T/C]TTGGAGCATGTTTTGGTGCCATTGGGTCTATTGGTGTTCTTCATGTACCATATGTGGCTT
1811	CaSNP3809	TTAAAGTCCATTATACATGCATAAGAAAGCAGGATTCTAATGGCTTCTAGTCTAGCTACA[T/G]GGGCATAGGTTTCATCATAGTCAATGCCCTCCTCTTGGTTGTAACCCTTGGCCACTAGCC
1812	CaSNP3810	ACTAAAAACCTCAAATTTTTATCCATACAAGTAATGCCTCAAAATCTTAAGAGCAAAACT[T/C]GCAGCTGTTAATTCAAGATCATGGGTTGGATAATTCGCCTCATGTGACCTAAGTTGTAGA
1813	CaSNP3811	ATCATCCTTCAAATCATTTTCCTCCATTTTTGGCCAGTTTTCATCATTACCATCAATTTT[T/C]ACATTATCAAACCAATCAAAGTAATTCCATCCAACACAATCATGTCTTTGTAACCCATAA
1814	CaSNP3812	TATCCAATGTGGGACTTAATTATTTATAGAGTCAAAACTACTCAATATATGAGATTTTTC[T/C]AATGGTTTTAAATTCACACACTAACATACTATATGTTCCAACGATCCCCACTTAAATAAA
1815	CaSNP3813	TAAAGTACACCACCACTAGTCGACAGAACCATAGCTAGTTGAGGTTGATGTAAATCTTGT[T/C]TGATCAAGCCAGTTGGCATAATTGGTCGGTTTGGAGCTTGATCTGCTTGAGGTTGAGAAA
1816	CaSNP3814	CTTTCCAATTTGTTCACATAAATAGCAAGGTCCAGAATTTGTTACCTCATTTTATTCTGT[A/G]TTTGCCATCAAGCATATGTTGGCTTCTTCTCCTTCTTCTATATCTAATTTGCTGAATCAT
1817	CaSNP3815	AAAATGGTTCCTAAATACTGTTCATGAAACAACTGACAGATCAGGGTCGTACAACTGCTT[C/G]GAATTTTTGAAACATGATAATTGAGGAGCTGCGTTTACTCAAATTATTTCATTTTCTAAT
1818	CaSNP3816	TGATGATATGGAGTCTCAATAAGAAGACTATACTTTCAGTTTCTAAAATTAGCAGCCTAC[T/G]CTTTGGTGAATATGTAAGATCTTCTGGTATTTATGTCTAGGGTTAAAAACTTTCCTTGTT
1819	CaSNP3818	ATTTCAATCCCAAATCTTCCAAATTTTCTAAATTGATCTTGAATTACAGGGCAAGCAATG[C/G]AAAGTTATACGAAAAAGACTAGAATAACATATATAATATATGTAATGAATGAATAAAATA
1820	CaSNP3819	TTCAATTAATAACATACAAGTATTCTTTACTCTCTCACTTCATGCTTTGATTATTTTTCT[A/G]TAGCCTCTTCAGGATGTCATCATCAAAACCCTTCTTCAACTCTCTAGCAACTTGTCGTTG
1821	CaSNP3820	GTGGTGACACATACACCCATTGTCTGAAGGCTTTGAAGTGGAAATGTGATGTATAAATCT[T/C]TTGTGGTCCTAAAGCATTTGCCCTTTTGTTGCTCTCATACTCCCCAACAAGTGGTATCAC
1822	CaSNP3821	TGTGAAATCACCCAAGACACAAAAGATGGATCAGAGTTGGATATGTCTACTGAGATGAAT[A/G]CCTTATGGAATCGCTTTCGATCACTTGATGTTCATAAAAAGTCTACAACTCTGATGTCAT
1823	CaSNP3822	GCATATTGCGGAGCAATAGAGTTATTTCAAATTCCACTAAGCTTTGCTATAGCACTACAG[T/C]GCGCTATTTGACAACATTGCATAAAATCAACCCTTGACATAAAACTCCAGCAGCTACACC
1824	CaSNP3824	TAATTTCCTTAGTGGTACAATTGAGAAAGCATTTGTGAATTGTAAGAACCTTACACAGTT[A/G]GTTTGATGAACAATCAGATTGTTGGTTCTATACCACAATACCTTTCTGAGCTTCCTTTG
1825	CaSNP3825	AACAACACCGACGCATTATCGTAACCAGCGTTCTTTTCTTGTTTTTGATGCTTTGATCAC[T/G]GCTTATACAGATTCTGGATTCATAGAAGAAGCAATTCAGGTTCTACGTTTGATGAAAAAT
1826	CaSNP3826	GTACATATATAGTTCATGTTACCAATAAATTATTTCATGTATCACTATATATATCTTTGT[T/C]TTCCATTACTAATTAATGTGATTTTGTGTCAAAGGAATCAATACCAATAATATATAATTA

1827	CaSNP3827	TCACCAAATAAAAACTTACATGTCTTCTCCCCCTAGACTCTTGGACCAACAAACTAATGA[T/C]GACTATTGTGACCCTATAATTCAATGCAATCTAATGAAACTAACAGATGTGATGAGGAAA
1828	CaSNP3828	AGAGGTAAACAACACGAACAAGAGAGATTAAGAGTGTGCTTGATGAGATATAGATGAATG[C/G]CGGATAAGAGGTTAGAAAATTGGTTTTAATGTTTCAAGGGTGATGATAGTGATGTAAAAA
1829	CaSNP3829	TTTCCGTTGATTTTTAGTTTAGCCATAAAATAAATGTTGCTTGTTGTATATATTGAAAAT[A/T]AACATAAAAGTGTTACAACAAAAATAATATAAAATAATAGTTAAATCATGTTCACCTTAG
1830	CaSNP3830	TAGATCCTTAAGAGAGACAACTGTTCAGAGTGATAAATAATAAACATGTGTTCTATTTTA[C/G]TCCGAACGTTCTTCCATTGCAACCATAACGTTCTTGGTTTTGTGCTTGCACGCATGTTGG
1831	CaSNP3831	AAGGTTGTTATAAGGTTGTTATTGTTGATTGATGTTCCTTATTGTTATTAGAAAGACTCT[A/G]GTGTACCCATTAATTATTATAGTGGAAGTTTGTACTGGACTAGGTTCGTTGTTTTTATTC
1832	CaSNP3832	GATATGAAAACCAATATGAAAGTGCTTTTTGTTGAGCTATGCAAGTTAAACCTGATTAAG[T/C]GAATTACAATGAGGTTAACATTTGTGGCTTTCAACAAAATGGTTCACGCTCTATAAAGGA
1833	CaSNP3833	AATAAGAACAATTATCCTCTAATTATTTGCTAAGTGTGAAAATTAAGTTTGAAGGTAAAG[C/G]CTTATATTAATGAATAACAATTTTAAGGTAGTATAAATTATGAGTAAGTTAGAGATAATA
1834	CaSNP3834	GTCCTTCGGTACAGTTTTGTCATCAATGTAAGCTGGATATGTAACACCTCCTCTTGTGTG[A/G]TTCTTATATTGACCTAAAACTACATCCTCAAGCTTAAGGGGTCGCATGGACCGAAGAACC
1835	CaSNP3835	CATTGTTTATTTCGATCAACGGATCGGTAACCAACCCGAATACAGTCATTTTATCTATGT[T/C]GATCTACGCATCGGCAACCAATCAGAAGACGATCATTGTTTATGTAGATCAACGCATCCG
1836	CaSNP3836	AGAAAAAACTTCTCTCAGTTGGGTAATTCATTCATGGGGATTTGCTTAGGTCCTCCGCTT[A/C]TGTACCACTGTAGTCCCTAGTATACTGGTGAGGTGAAAATAGGTCAGAATAAAATATGTC
1837	CaSNP3837	AAGGAATCCGACTGTTTAATTAAAACAAAGCATTGTGATGGTCTCCTGCGGATGTTGATG[T/C]AATGTGATTTCTGTCAAATGCTTTGAATGTCAAAGTGGAAACGATCTTAAATAAAACATA
1838	CaSNP3838	TTCCTCATCCTCAACCTCTCACTGATTCATGATTCAAAATCATCTTCTTTGACGTTCCTC[T/C]GTGGTCCGCTACCCGGTGTATTATTCTTGTAACTCTTTTGAATCATCTCAATTGAAGGAT
1839	CaSNP3840	TTTAAATATCTTAAAGGTACTACTAACCTTGGTGTGTATTATAAGCCATCCTATGAGTTC[A/C]GGTTAGTAGGTTTCTATGATGCTAACTATGCTAGAGACAAACTATTTAGAAAAAGCACCA
1840	CaSNP3841	TAGAGTTAGGATAGGGTTTAGTGTTCAAGAGATACACACATAAACTTTATGTGAAAATTT[T/G]GGGCTTGTCATGCTTTAAAACAAGATTTAGGATGTTAGATATTAGTGATTTAGAGTTTCT
1841	CaSNP3842	AAGTCCTGCTCGATGTAACCTATATGAACCAAATGGGTTTTTAGGATGAAACTAAATGAA[A/C]AAGGTGAAGTGGTAAGAAACAAAGTAAGGCTTGTATCTCACAGGTTACATTGATTAAACT
1842	CaSNP3843	ACTGAGTAAGCTTAAACGTATCAGGGAAAACTCCATGTTCACCAAGCTTAGTAACCAACC[C/G]CATAATTTCCTCATCAGCAAGTCCTTTTCCGGGGCGCTCCCTCTTCACCAACTCCCCAAA
1843	CaSNP3844	TATGGAGAACTCACTATGTTTTTCATAAAGTTCCCTCTATAATATTGTCATCACAATTGG[T/C]TTTCTGTAAGATAATTGTTGATACATCAAACCATAAAAACAGTAAAACAATGAATAAAAG
1844	CaSNP3845	ATATACTATTTAGAAAGATAGAATGATCTTGTAAGGAGACCCTACCTAGAGTTGAAAAAG[T/C]CACAAAAGCAAGTGAGTCAGCTATGATATGTAGAAAACAAATTAGTCGAGGGAAGGTGGA
1845	CaSNP3847	TTTACTTTTGTAGACTATATATTAAATCAACTGTGTCTTGTGAAAAGAAGAGTAATGTTT[T/C]TTATTAAAAATGTATAGCAATATGTAAGATGTGTTTCTGTTTTTGAACACATTATATGTA
1846	CaSNP3848	GGCAGCCTTTTTTGACTGCATAGGTTGCCACAAAAGAATTGTATCGTTAAGGTTTCCTGG[T/C]CGCTTTGGCTGCCACAAAGGCCCACTTTTCTACCTATATAAATCACTATTCCTCTTATTT
1847	CaSNP3849	ACAATTTTTTTACACAACAAATCACAAGATGATTTTTTGTCATAAATACATCTAATGATG[A/G]TATGGTAAATCCAAAGCTGAAGTCACATGGTACATTACTTACCTATATGCATAAAACTTA
1848	CaSNP3850	AAATTTAAGAGGAGAATGCTATAAGGGTTTCTCGGTCCTTGATTTAATCTGGATTTTTCG[A/G]GCGATTTTCGTACCATAAGACAAAATATATAGAAATATGTATTTTCGGACCCGATGGTGG
1849	CaSNP3851	AAACAGCCCAAGACAACCACAAAGCACAATATTGTATGCTCCATTAATTGAGTAAATAGT[T/C]ATTTTTGGTAATTGAATATGTGATGCTCAATCATTATAGTTTATGATTGCCATGCATCAT
1850	CaSNP3852	GTTCAAGTTTGGGAGGAACCCCACTCTGATATGTGATACCGGGAATAAGGGTTTTAATTT[T/G]TATTTTACTTATTTAATTTGAATCGAATATTTGTATGAAAACCTTAGAAGTTCTAGTAAG
1851	CaSNP3853	TTGACTGCTCCTAATTGGTAAAACTTTTTTAGCTTACCCCCATTGTCCTAGGACTCATTA[A/G]ACCCATACTATTCCTATTGTCCATAATTCAATTAAATCATAAGTTCAAAGTCCAAACCAA
1852	CaSNP3854	TGACACTTACATAACACATTATGTAGTATTCTCATGGCGGGTCAATCCAGTACACGTGTA[A/T]CCTAATGTGTACTTATATTGTGATTTGATATTTCATATCTATAACATGTGAGATAAGGTT
1853	CaSNP3855	GCAACCTCAGCGACGGTGGCAGCGCCGACAACGACAATGACGACAGTGACATGGACGATG[A/G]CAACAACGAAGAAGACATCATCACATGTTCGAATTTTTAATCGTTTTATGTTTTGTTCAT
1854	CaSNP3856	TCCCCTCAACTCAACCCGAGACACATATACGCGACAATCGGGGTATTCTTGCGTTGCACG[A/G]TAGTTCCCGGCCTTAGGAAATAACCCACTAATCCACTTAGGAATTCCCCCACTAGATGTA
1855	CaSNP3857	TCCCGTTATCACATATAAGAACGAGGTTTCTCCCAAATGTGAACTTCAAGACTCATAAAC[T/C]TGTAATAATTGTGATTTCACTAATGTAGGGTCAAGCTAGTCAAACACAAACAATGAAAGA
1856	CaSNP3858	TTGAAAAATTAACAAGAATATTTCAAAACTTGCCCAAAACAACAAATGAGTATGAAGTGA[C/G]TCAAACCATTGAAACTTAACCACTTCCCAACGAAACCAATAAACTGAATCCGCAAAACTG

1857	CaSNP3859	ATGCTTAGTCAAGGTTTCGAGGACCATCTCACTCAAAATCCTGAAAAGATGGTTGTGACC[A/G]AAACATCAAAATGGAAATAGATAGATGCTCAGTTGTGTAGTGTCATTAAGTCTACACTTC
1858	CaSNP3860	CTTGATATTTCTCATCAGTAGCTTCTTCTTCAAACTTATTCATCAAATCATTCGTTACAC[T/C]ACCTTCTACGTCGTTATCAAGACCAACATCAACCTTAGTCACGAATTACAAAAATTAGAA
1859	CaSNP3861	TTTTTTGGGATGTGGATGATAGAATTTGTAGTCCTTTTGGGTGGTAGAGTACTTGATACA[A/G]ATACATTTTAAGGCTCTAGGATCAAGTTTCCTTCTACTATCAACATGAATATGGACAAAC
1860	CaSNP3862	TATTATTATCTTGTTATATGTGGATGACATGTTGATTGTTGGTCATGACACTGAGAATAT[T/C]CAATATTTAAAGAAATATTTGAAGAAGTCTTTTGTAATGAAAGACTTGGGTCCTGCAAAG
1861	CaSNP3863	TTATCTCATATTTAGGGTTTTGAGACTTTTAGGGTTTTCTATTTTCTCTCTACTTATATT[A/C]TTCCTGTAATCCTTTGGTATATATCTTTGGTTCTTCTATTTTGTATTGTTTTGTTTCTGT
1862	CaSNP3864	ATCGGGAAGTCTCTAGTGTGGTTTTAGGGCTTCCTATAAAAGTTACCCAACAAACCATTT[A/C]TGCTATTATCAAATGCCTGGATGAAATCATGGTAATTGAAAATGAATATGAATCAAGATT
1863	CaSNP3865	AGAAGCGTAACCAGGATAATGTAATTCACAAGCTGCAACCTTCCCATTATAGACAAGAGT[A/C]TGCCTTTCCAAAAGATCTTAGTTTTTTATACAATCCACAATGGCAAGGAATTGAATTTTT
1864	CaSNP3866	CACTAGGTAGGTCTTCCTAGTTATAGATTTGGTTGACTCTTTCACGGGTTTGGTTTTGCT[A/T]CCCCATGTTTCTTGTTTTCTCACTTTTCCTCTTATTTTAATGTATTTGGTGTGCTGCAGA
1865	CaSNP3867	AATTTATGTTTAAGATTCAATACAACCATAATCCAAACAACATAATCTAATTGTTTAATA[T/C]ATTTTCCAAAAGACTTTCTAGACTCAAATAGCTGCAAAATACATCATGACTCAACAAAGT
1866	CaSNP3868	TTAAATTAAAAGAAAATAAACTTAAAAAATCTAACGGAAAATTGGGTTATCCTTAAAATA[A/T]AAGTGCATTTATATCTATATTTTTTTAACAGGTTACCGTTAGTTAGTTTGTGACGGTTAA
1867	CaSNP3870	AAGCAATATCATTCTCCAAGTTTGAGCAAGCATCAAGTACGGATTTGACATATTCGATTG[A/G]ACACTTCAGCTGTAACTTTGAGCATCCTGCACACATGACAAAAATCAGAGAATCACTTTA
1868	CaSNP3871	AATGTAATTTAGCCTATTTTATATAAAAAATGTAAGGAGGTGATATAAAAATACTAATAT[A/G]TGGGTGAATATTATTGTTAATACTCGATGTTACTTCTACATCTTCGAATTGATAGATAAG
1869	CaSNP3872	AATCCTATTTAAGTCAAACATAGCAGCAAAAGTTAGTACAACAGAATCTAAATTTTAGCA[A/T]TTTCGGGAGTTTACCAGAGTCTGACTGATAGCTTTGTGAATCAGATCCTTCAATAAGGAA
1870	CaSNP3874	CTTGTAGATGAAACAAAAATGGTGCAGAAGGAGGCAAGAGTGACAAAAGCAAATACAACG[A/G]TAGAGAGATATGTTGTTGACAAATCGTTGGGCCAGAGAAGTTGAAAGTAGGAGATACAAG
1871	CaSNP3876	AAGATAACAAATGCATCTGTTCAACGGAAAAGAAACATCATCGGCGAAGATACATTAAAC[T/C]TTCTAGAATCTTCAAAAAGGAAAGAAAAAGGAAAGCTTTGAAGTTCTTTAAAAGGAAGCA
1872	CaSNP3877	TTTAGGTTTTTCTTGATGAAGTCAAACCTATCTTCAACTAGTGGTTTGGTGAATATCTCA[T/G]CCCATTGATGTTCGATTTCTACTCAACTTCTGTTGTTGACATGACAATCGAGCTTTGTCT
1873	CaSNP3878	ATTTGAATGATATGAACCAAACACATTAGGAAACAAAGGAAGTAAATATGCATGATACCC[T/C]AAAAATCTATCTCATAGATAACGATGTCCAAACTAAGATGTTATTATACGTTACTAATTT
1874	CaSNP3879	ATCATGTTTCTTGGCTCCTCATTAATCTCATGGAAAAATAAAAGACAAGCACGAGTCTCT[A/C]AATCTTCTACTGAATTAGAGTATTGTACCATGTCTACTTCTTCTTCGAAAATTATTTGGC
1875	CaSNP3880	TGACATAAACTTAAGCAAGAAAATGTTTCCAAAACTGAACTTTCTGACAGAGTGCAAAAC[T/C]GACCCACATATCATTGGTCATATACAGTTTATTGTTACGGTTTATGGGCATTAGGTTTAT
1876	CaSNP3881	TTGTTATGATGATTGATGACACTTACCCTGATATGTTTGCTACTGACTTTCCTTCATTGA[T/C]TGGTTTTGATGATACTTGTGAATTCTGTACAGCTACAAAATTGAGCTTTGTGTGTGCTGA
1877	CaSNP3882	ACCGTCTCTAATGTCATAATATTATTGGCAGGTGAAGCGTGTATACTATCCAGGCTTGCC[A/T]AGTCATCCTGAACACGAGCTTGCCATGAGGCAAATGACTGGTTTTGGGGGCGTTGTCAGT
1878	CaSNP3883	GATAATCAGATTTAGTCATTTTCATGCATTATTAAAATACAATTGCAGACCATGTGCCTG[T/C]GGTACCGGTAAGCATAAACAATTTCAATAATAAACTACACATAGATCTTGTATATCCTTT
1879	CaSNP3884	AATAGAACTATAGATGACACTAATTTAATTGGCTTTGGATGGCATGTAAGTTTCATTTTG[A/G]TAATTGTATAATTTCACATATACTTTCACACACAAAATTATTTGTGTATATGTCAATTTG
1880	CaSNP3885	GAGTTTTTGTCTTTGGCTAATGTGGAGGCAAAGATAAATGTGAACATAGGGGATGTGATG[T/C]TTGTCAATGAGTACCGAGACGTATTTCCAGCAGAAATTCCAGGATTGCCACCGGTAAGAG
1881	CaSNP3886	GATAGGTTCAGGGTTAGAGGGAGGGCTAAAAGTATTGGTTTGTGGGGATAGGGAACAAAA[A/G]GGGTTGAGGAGAAGGAATAAAAGGTGTTGGTTCAAGTTCAAATTGGGGGGATAGGAAAAG
1882	CaSNP3887	TGAAGTCGGAGTAGAGAAGCCCCCATGCGACGTTCGTTGTTCACAGCAAGAGTTACTTCA[C/G]TTTTAATGGTTGCAAGCACAAGAGGACACGTTTTGAGGTAAAAATTAGATGACAATTGAG
1883	CaSNP3888	AACACAATTTTTAACATGGAAAACTTCCCTCAAATTGAGAGAATAAAAACCACGCGACCT[A/T]GTCCAATAAAAACTTCCACTATAATAATTAATGGGTACACCAGAGTGTTCCTAATAACAA
1884	CaSNP3889	CCTAGAACCCACATGCCAAATGTGAAATCCGTTATAGAAAAGAACTGACGATTTTTCTAA[A/G]GATAATCCCCTTCAACTCGGTTAGGTGGTGGAAGATGTGTGTCTTGTCAGTGCTCTGTTC
1885	CaSNP3890	ACACTAGGGATGGGGTTCAATGGACTGTTGTGAGTCCAAGGAAGAGGAAAGGCAAGCAAC[T/C]GGTACAAGTCAGACTAGACGGTGACAAAGGGTACAATGGCAGCAAATTCAATGCATTATA
1886	CaSNP3891	CTTGACCCAAAACAGCCCCAACACCTAAATCAGAAGCATTAGTTTCCAAGATAAAAACCT[A/G]AGAAAAATCAAGTAAAGCAAACACATGAGCTTGAGTTATAGCTTGCTGAAGGTTTTGAAA

1887	CaSNP3892	AGTCATAATTTCACTCTTTCTCGCTTTGTAGGTCATAAGTTGAGCTCTAGACATCGGATT[A/G]GCGCATATGAGCAAGCATTGGAAAGATAAACATCTGAGCTACAAATTTTGTGTTGGAAGA
1888	CaSNP3893	TGAATTTACTCTGTTGTTGGAAAATGGTAAGCTATATATAGTCTTGTTCTTAAGGGAGAT[T/C]TATTGCTTATGTTCTACTCATTCGTTGCCAGACGATGAAACCATACGACATTTTCTTTTA
1889	CaSNP3896	TAAGACATAAGACAATATTTTTCAATTGTATTTATACTACCACTCTATCGCATTCGTCGT[T/C]GACATTCTCTCATTTGACTATTTTTCCCCCAACCAAGAGGCTTGACTTCTATTTAATGTC
1890	CaSNP3897	ATCCGACTATTCACAGTGACCCTGATGTGTTCAATCCTTCAAGATTTCCTGTGAGTCTAT[T/G]TCTACCCTCAAGGCCCCTTTTTCCCTCTTGTTTAATCGAACATATTATGCATCATGTGAC
1891	CaSNP3898	ATGGCAAACTATTTGTTTTTCAAATGTCAATTCTTCTCTAGAGTGTGGTGTGAGATGGCA[A/G]AGTGGCTAGACGTAATGAGTAATACTTCCAAATGTCACTATAGAACGTCTTCAAAACTTT
1892	CaSNP3900	AAGAAGATGCTTAGTGGGTTTGGATGATCAAACTTTATTTGAATACGCAACTTTAGAAGT[A/T]CCATGTTAATATTCATATGGCTTAGTTGGATGGTAATGTTATCGTTAGATTGAACATTTT
1893	CaSNP3901	ATGTTACTAAAAAAGAAAGCAACTTTATAGAGTTAATTTATTTATGTGTGACTCTAGGTT[T/C]ATAATGCTCAGTGGTTGTTCCGGCTTCTACACCTATGCAATCTATGAGCACTTGGAGGAC
1894	CaSNP3902	AAAATTATTTGGATGAGAGATAATAAATGGTTATAAGAGAGATAGAAACAAAGTTACCAG[A/G]GAATTGAAAGGGAGACAATCTCAACCCATTACACAACTCATATCATAGTAGCAACAATTT
1895	CaSNP3903	GGCCATCAATTTAGGATAGCACTGAAATACTGCCTCCAGTATTGCTAAATTAAGCTACAA[C/G]GGTGAAACAGAGGACATATGTTGGAGGAAAGTTGAAACTTGAAATACAATGCATTGGGCT
1896	CaSNP3904	CTTTGTCATAATCAAAGATGTATTCTATAACTAAAAACATCATTCCAATACAATCTAGGA[T/G]GATATGATAACTGACAGCTAGGAATTTTGCTTCCATGAAAACACTTAAGATTGAATAATC
1897	CaSNP3905	AATTGAACCGATTAATTTATAAGATCAAATAGTGTAATTGAATCAATTAATTTCACATTT[C/G]GCAAATAAAAGTGATCAAATAGTGAAACATAAATTTGACGCTAGATATCAATTAGAGTGT
1898	CaSNP3906	ATCATCTTTGGATGCATATTTATTGCAAGAAGCAATAAAGGATGAAATGGATTATCTAGA[A/G]TCTAACAGGACCTGATATTTTGTAGACTTTCCTCATTGTTGCAAATCAACAGGTTGTAAA
1899	CaSNP3907	TCATTATCATATCATTTTCAAAACCATAAAAATTAAGTCTTGCTGTGAATAACTTTAATC[A/G]CGGGAAACATGAGATCAATTTGTGGCTCAAACATTACTCCATTAGGAAAGACTTTGCCAT
1900	CaSNP3908	GAAAGGTCACATGATAGATTGTATAACCAATTTTGTGGTTACGATAATTGGTGTTCTAGA[A/G]ATTACCATTGATGTGGTGTTGATGAATTATAAGTGAAAGTAATAAAATTATCATTAATAT
1901	CaSNP3909	TAGTGTGCTTTTTGAGAATCTTCAAAATAAATATATGAAAAAAGTTATAAAGCATTTCCA[T/C]ATGACAAAATATTATAAAATACAATTTGTACAGGACAAATAATAATTTACAAAATATTAT
1902	CaSNP3910	ATGTAGACTTATCGTGGTTCACCACTAACTTGACTACATCATGTCCCCACATTCAGAACG[T/C]TTTAATCAACTAATTTAAATACCTTGAATTACAAAGAACCTGACCCTCCAAGCCAGTCTT
1903	CaSNP3911	GACAATATATAATATTTTGGCTTGACAAATGGATTCTGAACAACAATGCTCTCATGAACC[A/T]CTCTACTGATTTGATTATTGACACCACTCTTATTTTAAAAGGACGTGTTAACAAACACAG
1904	CaSNP3912	CTAAACTGAAATTAAGCTAAACTTAAAATACCATAGATGTATTTCTTTCATAAAAAAATG[A/G]ATCCCATGAGTTTTCTATATCTATATATATAGAAACTAATTATCATTGAGAAACTATCTA
1905	CaSNP3913	TTTTTCATTTTAACATTCAATCACTTTATGTTTTATCAACAATAATTTCATACATTGTCG[A/T]TAAACTCCATTTACTTTTCTGCAAATTCACTCTCACACGTATTTACTTTTCAGTCATTTT
1906	CaSNP3914	GAAGATGTTCCTCCACCAAAACAAACAAATATTGAGAAGGATACATCAAAGACAAGGAAG[A/G]ACATCCCAAAGATACTTACTTATTTACAATCAATCATTGATCAACAAATTCATCAAGATA
1907	CaSNP3916	ATGAGGTTAAGAACAATAGGAATGTGATGTCATATAAGTTAACCAAAGAAAGATCACACA[A/T]GGAAGAAGAAGAAACTTTAGCACGATGTGTGTAAGAGGAACAGAGAGAGAAGAGAGAGAA
1908	CaSNP3917	CGTCTAGTCCTCACAACTGTTCAAAACAATCTTGGTTTTACACCACCAGTTCATATCAAC[T/C]TTGATCTGTTACAAAGCAAGTCCTTTCCACCCAGAAATGAGTTCAAATCAAGTCACCTAT
1909	CaSNP3918	GTGTTGAATATAGAATGAAGAGGAAGAAAATGAGTTATGAAGAAGATGGAATAAAGAAAA[T/C]GAGTTATGAAGAAGATGAGAAGATGAGTTATGAAACAGATGAGTTGTGAAGAAGATGAAA
1910	CaSNP3919	AGGAAATTTTAAATAGGAAAATTTACAATTCAAACCCCCTTTTCTTGTAAATCGACATTG[C/G]TACTTCAATTGGCATCAGAGTCCGGTCTCTAAAGTTGAGCACCTAACAAGTGTTTGAGGA
1911	CaSNP3920	ATGAATTTGTAGTCTAGGGATAGATGCAAGTCATGAAACCAATTAAATTAGTTGCAAAGA[A/C]AATAGTTAAACAAGACAATTCTTGTACGGTAAGGCTTAAATCTAATCTTAAATCCACTAA
1912	CaSNP3921	TTTGGATGTTGCTAGTGAGGGAGCATTTGTCGATAAGACCCTAGAAGCAACAAAAGCTTT[T/C]ATTGAGAACATGTCCATTAATTCTCAAGAGTTTACAACTAGAAGCAATTCAGCAGTGTTG
1913	CaSNP3922	TTAGTTTTTCTTTAACAATGTGTAGGCTTCAGGTTGTGTAGAGAATATTTGACTTGTAGG[A/G]TAAATGTGTGGTTTGCCTCCAAAAGTCTGTAGGTTTAGGTTGTTTTGAGAAGACTGACTT
1914	CaSNP3923	GAAAGAGTTCGTCCAGCCCAATGCCCATTTGCGATGGCATTTTTTTAAAACCAACTGGAT[A/C]GAAGCATGTATCATGGTGAAATAGACCGAATTATTTTGAACTCAATTTGTGTTATTCATG
1915	CaSNP3924	TTCTTCATTTCCTTATTTATTTATTCTCACATATACTCATCATTGCTTAGGCCTCACCTC[T/C]TCTCTGTTAACTAACAGATCCCCTTCATCACTCGTGGGATCAAGCCCATTCCCACCATCA
1916	CaSNP3925	ATGTAGTGTGAGTCTTTTATCTCTCCACTGTTATGCTAAAGCAGCCGATAAAGCACATAA[T/C]TAAGACTGTTACAAAAAACTGAAAAACCAAACTGCACTGAAACTGACCGAACCACTTTTT

1917	CaSNP3926	TCTCGTACCATTGCTTTTATGGATGCTTAATTAATGATTCATTGTATATTTGATTTCAAT[A/G]TCATATACTCCTTTTATTTTGGTTGAAAATGTAACACACTCAACTATTTGCATTTAAAAT
1918	CaSNP3927	TATACATACATGATGTCAGACCTAGAAGTGGTAAGGTAAAGTAGGGATCATATCACTCCT[A/C]TATAGATTTTATTGTCAACTTTGTCGCCTGAATCGTCTTTTTCAAGTGAACAAGTAGGGT
1919	CaSNP3928	ATCCACTAACAGAATGAGTGACTCATGCTTACTGCAAGTAGATAGATACGAAACCAGGAT[T/G]TAATTACTATGACCAAAAAGAAGCACAAGATAACACGTAATTTTGCCTGACAACTTTCAG
1920	CaSNP3929	CATATTGAACTTTGGAATCTTTGGAGTTCCTATGGTTGGAGCAGATATATGTGGCTTCTC[A/T]GGCAACACAACTGAAGAACTTTGCCGGCGCTGGATCCAGGTAACAAACATAGATCCTCTA
1921	CaSNP3930	AAGTTTTGAAAATCATTATTCATCTCTTATGTGACAATATTTTATGTCATATAGACGTAA[A/G]TTACTATACTTTTATCAAACGAGAGATATCCATTATGAAGGTACTTACGAGTTGTTATTA
1922	CaSNP3931	TATTCATAAAATAAGATGAATATGGAGTATGGCTAGAGTTTGCAAACAATTCATTATACG[A/T]TGTGATTATGATAATATGTTCACTGAACATTCTGCTGCATCTATTATAATATTGCATTCA
1923	CaSNP3932	GAATTGCCTTGTTGTTGCTGCCAGTTACTAACAAGTCATCAACATATAAGCATAGTATGA[T/C]CATTGCATGCATTCCATTCAATATGAAATGCTTCACATAAAATCGATACTTCACTATGTA
1924	CaSNP3933	CTATACTTCATCATTTGGTACCATTCTACTCTGAAGTACTTGGTTAGGAGGCCTGATACT[A/C]TGCCACTACAAGAGTGGACGGACGATTCATGAATGACCAAGTCCTCATAGATCCCCTCAA
1925	CaSNP3934	AATATTCAAATGGAAATTTATCGAAGTATAGTATATACACATTCTTTTTTCTTGATCCAG[A/T]GAAAAGTACTTTCCTATAGATGTTTGTTTTACTTCACACATGTTAGGTTTGAATATAACT
1926	CaSNP3935	ATGGAAGCAACTTAATTTAATTCCCTTACTTTCCATATTATAGAAATAAATAAATTTTGA[T/G]GGTGGTACTTTGAACTAGCTAGTGCCTAGTGATATTGAAATGGGTGTCTTAAGTACCATA
1927	CaSNP3936	CTCCGTTAACACACCTCAATGAGAGCCAACTTTTTGTAGTTGTTGTTGTTTGTTGGAATT[T/G]TAATGTGTAGTTGATGGAAATAAAAATTTGAGTTTCGTTGAAGATTTAGGTTTTGGGTTT
1928	CaSNP3937	TTAGATGCATAGGTAGAGTTGGTATAAGCTATCAGAGTCAAATCTGCTTGCTCTTCGTCA[A/T]CAGAGGATTCATCAGAATCATTCCATGTATTTGGATATTTTGACTTGATATGTCCAGATT
1929	CaSNP3938	GCCACTGACTATACTAGATCAGACATTGCCTATGTCGTGCGACTATTGTGCAAGTCTATC[A/G]GTACACTTACGATGGAGCATTGACATGTGATCATAGCATTTGGGAACATGTTTTTAGCTT
1930	CaSNP3939	TAGAAGATGATGGACCATGTGTAGTAGTAAGTGGACTTGAAGGTGCATCAACAATAGGTG[A/G]ATTCGATGGTGCAACAACAGTAGGTTGATGGATCATAGGATGTGAAACATTTAAGTACCA
1931	CaSNP3940	TGTAATTCGATTGGATGATCTCGAAAAGCTCATTGATTGTTCCAATATTCAGGTAAAACT[T/G]AACCAAATATTGTTTACTCTCTCCATTTCAACATGAATAAAAAACAAAAGCATTATAAAA
1932	CaSNP3942	CACCACTATCTGCAGTAAGAGTCCTGGCGGACCTAGCCATTGTAATTGGAATTAGCTTGC[A/G]TACTTATTTCTTTCCTTTATTTTTATACATGGAGAAACTAAGATGGGGCCTTTACACATA
1933	CaSNP3943	AATTTCGAATGCAAAAAAGATGAATATAATTTGGATTAAATTTCTTAATATCCTCTTTGA[T/G]ATATATTTTGAAATAGTCAACAGATTTTAGGATATATATTTTATTTTAAAGTTGATGGTT
1934	CaSNP3944	CTGGTGCTTGTCCTAAGACTGAGCCTTATAAGAATGGAGAGAAGGAAGTTGACGGAATGA[C/G]TAGTGATATGAGAAAGGTTGAGATTGAAGAAGTGGAAAGTGCTAAGGTGAAAGGTATTAT
1935	CaSNP3945	TAGATTACACTAATATGTTTGCTTCGATGGCAAGGTTAGAAGAAATTCGCATTTTATTAT[C/G]TTTTAATGCCTATAATAGTATCGTCTTATATCAAATGGATGTCAAAAGTGCATTTTTAAA
1936	CaSNP3946	TATAGCCATTGCTTGCTTTTGAGTTATCCGATAAGGTGTTCCAATCAACATCATTGTATC[A/C]TTCAAGGACAATAGAAAATATTTGATAATATAATATGAGATTCATGGTCATTTTAAGGTA
1937	CaSNP3947	GACCAGATATTATGTTTAGTGTCTGCTTGTGTGCAAGATTTCAGGCAGATCCCAAAGAAT[T/C]GCATCTTATAGCAGTAAAAAGAATTATGAAATATTTGAAAGGAACAACCAATGTAGGCCT
1938	CaSNP3948	ATTACATATGGTATTCATAAAAAACATGAAATAAGTAAACAACCCACATTCATAGTTGAA[A/C]AATCTACAACACTAACACAACTTCTAATTCAGACAACCCACATTCATCAAGAGAGAAAAT
1939	CaSNP3949	GGGTGCATCTGCTCTTGCTTACCTATATGACAATCTTCGAGAAGCCAACATGCATCAAAC[T/C]AGAACTGTTTCAGGGTATCTGACACTTTTACAAGTAAACTGTATGATTGTTTATTGTGTT
1940	CaSNP3950	AACCTAAACTCCTTAGTCAGGTGGTCAATTCTTTATTATTTCCATTATACTTCTGTAAAA[T/C]GATGCTTTTTTTTAAATCATTGGGTGATATTGCTTTTTAGTATGTATAATCGACATTTTA
1941	CaSNP3951	TATCAAAATACTAATTAGTTTATTATAAGAGAACATTATAATTGCATATCACCCATGCCT[T/C]AGCACATAGAGATATCGAAGCACATTGACATAATTGCATAATGTATTTTGGCTAGGACAA
1942	CaSNP3952	TCTTAGTTATCCCTAACATAAGTTAGGATTGCCAATGGTTAACAAGAGACGAGTCTTCAC[A/G]TGCAGATTATTTGAGTTGTACTTACTGGACGACTATTACTTTTCTTAATATGTATAAAAA
1943	CaSNP3953	TTCATTAATATATCTAATCCATAACAATAATAATAGTATTTAAAAAGTAAAATGTGAGAG[T/C]CTCTTGACTCCACTTAATGGGACGCGGACACCATCGTGGTTACTCCTCTAAATTGGTCTT
1944	CaSNP3954	GTTGGTGAAAACGCTTATTCCTTCGCAATATGGTTCACGCAAAAACACAAACACCTTAAC[T/G]ACGCCACCATTGTTTGGATGGCAAACCGTGACCAACCAGTCAACGGAAAAAAGTCAACAC
1945	CaSNP3955	TCATCCTATTTATTATTAAAACATATACTACTAAATTGTTGCAAATTTTGTATACGTGAT[A/T]TAGCTAACAGAGTACTAGTACAAGATAACTCAACACTAATATTGATTTATCTTCTACAAA
1946	CaSNP3956	TCCCATAGTTCGGGTAGTGATGGACCCACCATCACAAGCGAATTCATCATTGCTTTGAAA[T/G]TGTACACTAGTTAGGAATCGAAATCAGATCTCTACATTAATATGTAGTTATGTACCACTA

1947	CaSNP3957	ACTCAAACCTCCAAGAAGCTACTAATCAAAGATCAAGATCATGTTTAATAGCACACAAGA[A/G]TGATATGATTCAAGATTTAGGTTATCATAATCAGTGGACAAGTGATGAATTTTCAAAGGA
1948	CaSNP3958	AGTATTGTTTAATCTTTTAGGATTTCTAGTTAATTAGAGGAGTGCTTAGGGTGATAAGTT[A/G]ATGGTTGGAGTTAGTTTCCCACATGAAATGTAGACTATTATGCTCAAATATGTTGATTGA
1949	CaSNP3959	TTGGCATATACGCATTGCATTAGGGTGCTTGGTACACGAGTCGTGTTTGATATGCTATGA[A/T]GATTGCGTGTGTATACTTAGTTGTGGTTGTGATTGTGCCCTAATTGGTAAGTGTGATTGT
1950	CaSNP3960	TAATCTAATGATCCAGGATAACAGTTCCTGCAGTTCACAAAATACTTAGTATACTTTTCT[A/G]TGTTGGGAAACTTTTATAGCCTAATAGTTGTTGTTAACTAGTATTAGAACCTAGAAGCGC
1951	CaSNP3961	AAATTCAATAACTTCAATAGATATTATATCAATTTTGTAATTGTCGAATTTTATTATTGC[A/G]TATATCAAAATACACAATATTGCTTTCTATATACAACTATCAAATTATTGATTAAAATTT
1952	CaSNP3962	CATCTCATTATAATATGAATCAAATCATCTAAGAGTAATTATTATTACTTTTGAAACACA[T/G]CAATCACCACAAAACAATCACAAGTAAATGCAATGCTATGCATGAGCTTAGACTCTACTT
1953	CaSNP3963	TGTGTCATATGAATTTGAGATTCAATTAGCATCATTATATTCTTCTAACACATCGAGAAA[A/T]ACATTATACTCAATACCGTAATTCATGTTACTTCTCAAATATTTCATAACTCTAGCAAGT
1954	CaSNP3964	TGAGAGTTGAGGGGGACAATGCTGATGATCTAGAGAAGATGGTAGGATTTGATTTGTATA[T/C]GGATGAGCTAATACAGTGTTATTGATACAGTGTTATTGCTACGATTGAACACCTAAATGA
1955	CaSNP3965	AAACCTTTTTTCTTTCTTGGATCAAACAATGCCTATGCACCTGTTAGATGATATCCCACC[A/C]AAATCGTGACTTCCCTTTTGTCTTACTGCTTTTTCATTTGCAAATCATGAACATGCTTAT
1956	CaSNP3966	CAGGTTCCCTTGGTAATTTTGGAACTGATGGTAATTCTGATTCTTTTGGTAGTTCTAGTT[T/C]TATTAAGATGTTTGATTTTTCTGCCATTTTTGATTTTGTTGATATGTGTGATTTCTCTGC
1957	CaSNP3967	ACCCCAAGCTATAAAATAATCGGTGGATATTGTAGCCATTGGACATATGATAGTTTTAAC[A/G]TTGGTTTTGCACAAATCAAACTTACGATGAGCAAAAACGAAGCAAAATAGTAAATATCAT
1958	CaSNP3969	AGTTGCTTTTTATTGGAGAACGCGATAACCAAAGCTACAAAACGCAGAATGGGGCGATTC[T/C]AAAACGAGGTGAAAGAAGACATTCTGAAACGCGAGTTAGGTTGTTGAATCAACGAAAAAG
1959	CaSNP3970	TAAATTAAATAATTAAAAAATAAAATTAAGTTTGGAGATTAGAGTTTTCTTTGCTCTCAT[T/C]TTTTCTCTATTTCTCCTTATTCTACAAAACAAAAACAAAAATTAAATATTAAACTCAATA
1960	CaSNP3971	CTCTCTCTTCTAACCCTAATTAGAACACTCTCCACTTAAATAAGTGAGACACATGAACTA[T/C]AATATTTAGGTCTTTCTCCTCATATGAAGGGAGCCCAATACTCAACAAGTGATGCTTATG
1961	CaSNP3972	GAAATTGTTGGTGTAACTTCAATTGAGTTGCCATTCAACGTTGCATTTGCTTATATGGAT[A/T]GTGAGCGTCAAGAAAACTTTTGTTGGGCATTGGAGAAGTTAAAAGAGTTGTTTGTTTGAA
1962	CaSNP3973	AACTAAGTCTGTTGTGGACAAATCTAAATGAGCATTTCCTTTTCTCTTCTTCAACCACAT[T/C]ACCACCCTCATCCTACTTCTAGCTGCACCTCTGGTTACATGAAAGGAGTACCTAAGAGAC
1963	CaSNP3974	TTCATTACTTGTTTTCAAAGTGCATTAAACACTTTGTGACAGTCATCTTTACTTCTTAAG[A/T]ATTTTACCTAAGTCCAGTTAGTGTAATCATCTACTATTACAATTCCATATTTTTTTCCAT
1964	CaSNP3975	TCATATAGGTAGGGCCTGACAAGCCTAGTGATTTTGGGGAAGTACAAGTGAATGAGCCCG[A/T]TGGTGGCGGTCTGCTGTCGAACCTTAAGGCAAGATTGTTAGACCTTAGAGGTATACGGGT
1965	CaSNP3976	CATTCCGTCTACCTAATTTGAGTATGGCTTTCTATACATAGGTCTATTCATGGGCTTTAG[T/C]TGTATTCAAAAGTATCTCTTGATGGTATTGACTATGAGGTTGAGATTAAGGGTGGGGGTT
1966	CaSNP3977	CATGTATCCTCTACCTTTGATTTTGATCATTGTTCAACTGCCTCTGCTCTAATCATTATT[C/G]ACGACCTCTAACTCTGATCAATGTTCATCTGTCTTTGACTCTTATCATTGTTCATCTTCC
1967	CaSNP3978	AATGAATTTTCCTACGAACCATGTGAAGTATTGGAAAACACAAGAAAATAGAAGTTTCAA[T/C]TGTGTTGATCGTTAAACTTTCTTTTATTTAATTAGTTCTTTTTCAAAAGCTTATCTTTAT
1968	CaSNP3979	AGCAAACACAATAACTACAGTGTGTCAGTTTCCGTATAGTTTTTTCTCAAACCAAACGGA[T/C]CCATAATGCATTTTTGGAGTATTCATATTCTACCTGTTCTAGGGTTAGAAACATGATCAC
1969	CaSNP3980	TAGATATAATTGGGTCCAAATAACATTACTCATAAATTTATTCTAACGGTACAAATTAAT[T/C]TCAACATTAAATTTTTGAACAAACAAATGGATAAAATCATAATAGACACAAATTTATTAC
1970	CaSNP3981	TCATATGTATATTGTTGAGTATCGATGAAGGATGGAAAATTAGTGTTCGTTGTGGAACAC[A/G]CACACAATCATGATTTAGCGGATACTTTAACCGGTCATTGATATTTTGGGGCGTTTAAAT
1971	CaSNP3982	ACTTGAGCGCTTTATAAAGCCATGGATGAGTTTGGTGGTTTATTTCATATATAATAGAAT[T/G]AGATAACATTTCATGGTTATATTGAATTATTATTATCACCAAATACTTAAAAGAAAAAAT
1972	CaSNP3983	TTAAATTTTAATGGATTTTTTATATAAGAATTTCTCTACCACATGAGATTTTTATAGATT[A/C]TATTTTCTTATTCTCTTTGGTGGTTCAACTTAGGTGTTTTCTATTTTACTCTTTTTTAAA
1973	CaSNP3984	GAATACAACTTAAAGGATGATAACATAAAACTTTGAGTTAGTTTTTAGGCTAAATTATAT[T/C]TGTGGTCCCTTAACTTAATTTCAGGTAACGTTTTAGTCATTTATCTTTTTTTTCCCCCGA
1974	CaSNP3985	CGACAGCTTCAAAGAGAAGAAGACGGATGATAATTGAAGACGATGATGAGAGACATCATG[A/G]TCATTAATTTATCCCAAATGTGATTATATATCAGCAGGAGGAGCCTTCTATAGTGCATGA
1975	CaSNP3986	AGAAACTAAATCAGGAATTCATCAACCATAGTTCTGTAAATCCAGTTAATATATAAATTC[A/T]AGCTCAAAAAACATAATCAAGAGCAGTGTTGTCAAAAAAACCACTACAGCAGCATTAGAG
1976	CaSNP3987	CAACTTCAATTATTTTTCCAAAGAGAAACATGCGTGCCTTGACAACTCATCATTTGTATC[A/C]ACAGAAGTAAAATCTCTTAAACTTTTTTCGATAGAATGTTGTGAGATTAGTTTAGATATG

1977	CaSNP3988	TGATATGGGCCACTTGGGGTTGTATATTTAGCGCTACCATCCCCTTCTTTATCTTAGCGA[T/C]GATAACAATATTTATCTGTTTTGAGAGCAACATCCCTTGAGCCAACCCAACCTCCATAAC
1978	CaSNP3989	TGTGGATGATGAGAAGAAAAGATTGAATGAGTTGTTGAAGGAAGCCGGTAAAGTTCGAAG[T/C]AGAAAAGCCAGGTCACTAGAGAACCTTCTTGACGGCAATGCTCACAATTTAAACAACCTT
1979	CaSNP3990	AATGGTGCATCAATAGGGTACTTATTTCCTTTTCGTCCTTGCTCACAACTCACCAATCTG[A/G]TAATTAATTTGGAGGTTGTTTTCCTTCTTTGGGCCTTTAGCTTGACTTGTGGGCTTGACC
1980	CaSNP3991	TCTTACCGACATTCCCCAACACCTCAATATGGTTGGCTGCTCTCTAATTCTATCACAACA[A/G]TAACCCTCTGACATGCCCTGTATATACAAACATAAAATACATTATTAACAACATCAATGT
1981	CaSNP3992	ACTTCAGGTTTCTGGCCGACAGATGATGATCAATAATAATGACAATTTGGCTGGGGAAAT[C/G]AATCACACTGATTATCTAAATGATTGCAAAGGTGTAGTTTATTCATATCTTTCCAAAAAA
1982	CaSNP3993	CAAAACTGGATACATGACTGGACACATCTCCTCATCAATTTGCAACCTCACACAACTCTC[T/C]AGCATTACCATCTCAGACTGGAAGGGCATTTCGGGAAATATACCTGAATGCATCACAACC
1983	CaSNP3994	TTGTCAAAAAGATTTGGTAAATTCCGCATTCCAAAACTAGTGAAAGCCACCTATGATATA[T/C]TGTCTCTAATGGTTCAAACAATGAACTACCACGACTAATTCTGCCTCTACTATAGCAAAC
1984	CaSNP3995	ATCACCGTACAAGGCCATAACTTACATGTACCAGTATTATTATTACCAGTGGCAGGCGCA[A/G]ATATTATTCTGGGTGCATCTTGGCTGGCTACACTAGGTCCTCACATAGTAGACTACGCAG
1985	CaSNP3996	ATCGCCTCTAACAACGTCTCTGTCCTCCTCAATCGCGTCTCATTCATCTCTATACGATTG[T/C]GCATATCTTCTCGTTCTTGACGTAATTCTTCACGTTCTTCGGTCCAGTTTTCATCTTCTC
1986	CaSNP3997	GAAAGTCTATGAATAGAGTTAGTGATTTACACGGCTTTGAGAATGCAAGATTTTAGAGTT[A/T]GAAGAAACTAATTGTAATTGTTATTTTTAATTTTTTCTACATATAATCTTGTGCATAGGC
1987	CaSNP3998	TCCTCCCCTCTTCCAATGTATTCTCCTAAACACAAGTCAAGCACCACCACATCTCCAAAA[T/G]AAATCTCATAAAACCTAATCAGTCAACCATCCATTTAAACAAAGATCAATGAGAATCATG
1988	CaSNP3999	TCCACCAAGTCCCAAAGTTCAGGATCTTGTGAAATAAACAAACTTCTCAGCCTGTCTTTC[T/C]AGTACTCAAAATGTTCTCCATCAAACAGAGGTGGATAGTTGATGTTTCCTTCAGTAGCTT
1989	CaSNP4000	TATCGATTGGTGTATGTTCTGAACACCACAAGACCGCTGGAGAAGGAAATGGAAACATTA[A/G]CAGAAACAAGAGAAAGGCAGAAGTGGGACAATGATGACTATATATGCATGAGCCATATTC
1990	CaSNP4001	AACATACTTCGATGAATCATCTGATAATTCTGACGATGACATTGATGTTGACGAAGATAT[A/G]GAAGCGGAAGATCAACTTATCCTACCTGATTTCGATCCACCATCTCACCTGAAGAGCATC
1991	CaSNP4002	CAATTTTGCGGATAATTATTCATCTCCATGCTCATACCCATTGTACGGGTTTTTACCCTA[T/C]TTATTATTGAGTTTTTTTTGCGGATACCCACTGAATCTGATTCTGATTGCAATCCTTACA
1992	CaSNP4003	AACATAATAGCCAGATTAAAGAGGGGTACAAATAATCTGTCTTCAAGACATGCTTCCCTT[T/C]GTTGGTGTATTCTTATCAGTTCCCTTAAGCTTTTGATATTTGTCATTGTTCCTTCTAATT
1993	CaSNP4004	AGAATGTTGAAAAAAAATATAGCTCTATACTCCAATATAAAAACAAACTGTGAAAAGACAA[A/T]CATTAAAATAAAAATATTACATAGAGAAAATATGACAATGAGATTTTTATATTAGTTTCT
1994	CaSNP4005	CCTTCTTCCTCTATTTTCAACAACTTCAACATCGTGGGCTGAGTATGCGTACATGAACTG[A/T]GTTTGCGTAAGTGAGTTTGCGTAGGAACATATATGAACTGAATTTGCGTAAGTGAACTGA
1995	CaSNP4006	AGATTGGAATTTTTCTTGTAAATTTGGATCAGAATTAGATGGTACAATATTTGGAGAAAT[A/T]GGATAAGAGTTTAGACTCACTTGAGTTGGTAATGAGGATTCTTCCGCAACCATGTTCCCA
1996	CaSNP4007	TGTAGACTGCTTCTGTAGTGGGAGCACAGCTGTGACTATAGTGAAGCAGGTACTAAGATG[C/G]AGAAGCATGTACACTCTTGTTTTCTATTCTCTTTGCAGTTTTATTAATTTCATATCTTAC
1997	CaSNP4008	AAATCGTGCTGATCTGTGATAGGTGGAACCTCGGTAATTAGTACTGTTCTGTGAGAGACG[A/G]TACATTTATGATTTACGCCATTCGAAGAAGGTGTAGAAATTCCGGAATCATGTGCACTGG
1998	CaSNP4009	AACTCAGATTAAGTACAGAGCAACGATAAAGAAGAGAAAATCATATTTGATACTAGTTC[A/T]CAATAAACTGTTGTTACATCTAGTCCTCCACTTACTAAATAGATTTTGCTTCAGCACAGT
1999	CaSNP4010	TGACAGGTGGTGGTGTAACTTTGGTCAACGTGGCACTTATGTAGTAAAGGATATGTACAA[T/C]TTTTTTATCAACATTGACGTTCAATGAGGTGTATCCATCATATCCTTATGTGTGGAATA
2000	CaSNP4011	GCCACCGTAGGAGATTGGGATACTTTGGTTGGGCTGGGGACTGGCGGGATGGATAAGGGG[T/C]CGATGCTTGTGGGAAGAGATATGGAAGGATCGTCTAGGCGAGTCGAGCCAGAGCTTCCAC
2001	CaSNP4012	CACACTCATTTTTCTTTAATATTACAAATAGGCAAGCACAAAGTTTAAATTGGTTTAATG[A/T]CTTGATAGAACAAGAGCAGAGAAAAAGGATTAGAATTATTAGCAGAGCAATTCCAATGAT
2002	CaSNP4013	ATAAGGAAATATAATATTATTTGAGGAGCAATGTGTACTTGCATAAATTTGATACCTTAA[T/G]CATCATTAGGATATTTCAATGAAATTTAAAAATAATTTTACCCTTTTATTTAATAGATTT
2003	CaSNP4014	TTTACATTTCCCTTATGTAAATAATAAACTTGAAGTAGGAAAAGAAAAAGAACGAACTCA[T/C]TGTCGAGGTGATTGGTTTCACATCAGCTGATTTGTGGTAGCTAATTCATGGTAATTAATT
2004	CaSNP4016	TAGACTGAGTAAACCCTGCCTTATTGAGAAGAAACCCAGAAACCAGATTCTTCCTTATTT[A/C]TGGAGTGTGCATGATATCCGTCAGAATTAAGGTCTTTCCAGAGGTGAATATTAGTTCCAC
2005	CaSNP4017	GCATTTATTTCTTGATAAAATAAGCTTACCCTACAATTATCCATTTGTTTAAGTCCATTT[A/G]AGCCTAATTATTCATTCTTTGTTATATAGCTCATTACAAGCCTAAAAGTGAAAAACAATG
2006	CaSNP4018	TTTCTTTAAAAGAGTTTGCCCTTAGGATTTACAAGCTTCCAGAACTTATCTTTATATCCC[T/C]CTCTGAATTGTGCTATCAATTTTTGTTACTGTAATTTTAGAGATAATGCTTATGAGATAG

2007	CaSNP4019	TATTGTAAAAAATAAATGGATAAAGATAACACATAAATAGAAACCATAAAATAATGTATG[A/G]AGTGTTTAGTCTAGCAAAAGAGACTAATTTTATATGTGTTTAAATTTTGATTCCGAGAAA
2008	CaSNP4020	TCTCCTCTCCGGTTCCAGCTTCATGTTGTACTGCTTTGTTGGAGGCTTATAGTAAGTTTA[A/T]TACGGTTTATTTTATGTATAACCATACATATGCTTTTAAGAATCATTAGTATTTGTTAGT
2009	CaSNP4021	AACATAACCTCTCCAGGGTAGGTACTAGTTAGAAGCCCCTGACCAAGTGTACTGTTAAAA[T/G]CAAAACAACGAAGGTATTAGCCTTGTAAAATTTGGGAACATATAGCAATGTCAAATGTAC
2010	CaSNP4022	AGACTATTGAGTGATTGAATAATTGAACAGCTACCAAATATGAAATGACTACCAAATATG[C/G]AATGCTACCGAATATGGAACAACTACTGAATATGAAATGCTTACTGAGTAATTGCTTAAT
2011	CaSNP4023	ATAAAGTGAATAAAGATATTTATATTAGCTTTACAATGAAAATGAGCTCTGAGTTTCGGC[T/C]GTTAGAGTTAAGTGTTTGCTCAAGAGTGTATGATTTTTATTTTCAACCAAAATTCTTGAG
2012	CaSNP4024	TTACTTTCGCAGCTTTAAGGAAAGGGCATCAGCAGGATGTGTATTTCTAGACATATCTAG[T/G]GGGAAATAGTCTTGCAACTTTTTCATTTCCAAATCAATATCCTGGTAACACTAATATAAC
2013	CaSNP4025	CTTACTGTTATGCACCTACAGTACAAATATAACTGCAAATTCTTTTCTTTTCCACATAGC[T/G]ATATGGTGTTGATCACTTCAAGTTATAAGTTTCAGCATACGAGACAAAAATACCTATATT
2014	CaSNP4026	ATCTGTAGGCCCACTTAGAGTTATAAAGAGGGTGGAAAAAGAAGTGTTTTCTCCAGCTTG[A/G]GAGCCATAGCCTTCCTAGTAAGTATACTTTTAACGATATTCTTTTCTTAAAGCGACAGTG
2015	CaSNP4027	CCAATAGTGGCTTTCGTGTTCCTCAGGCAATCTGTAGGTTTTCATGTTATGTTGAGAAGA[T/C]TGACTTGTAGGGTCGGGTGTTATGTAATTCAAGTTTTGAATTAATGGATTAAATCCTTTT
2016	CaSNP4028	GATGTTCATTCAGATGTATAATACCACCTTTCTCCACTTTCCCTTTCTTCACATGCCAGC[A/C]ATATATTACCGTTGTTGAGACAAAAAACAGAAATAACAAAAAGAGCACATTTTTTTGTCA
2017	CaSNP4029	CATTCTTGGCACATACAACACATCACATATGAAAGACTATTTTCCATCTCTCCTTTGAAT[C/G]AACACATTTCCTACTATTTATGCAGTTACTTTGCTATTATTTGCAAATATGATTTCCCTT
2018	CaSNP4030	ACTAATATAAAAAAAGACATTTTAAATTAACAAAATTCTTGTTAATAATGATGAATTAAT[A/G]ATGACAGAGGGTATTTTGCAGGAATTTATTAATTTTACGCTTCATTACATTGCTGACTTG
2019	CaSNP4031	GAGAGAGAGAATGAATGAATGATGTTAAGGCACTTGCATATTATGTTTCTGATGTCATAC[A/G]AATGGTTTTGGTTTTTTAATTTTGTTAACATAAATATGAATATAAATCATGCAATTGTTG
2020	CaSNP4032	ATTTGTTTCTATTTTTGCTTTACAAATTCAAATTGATTTCTATTTTGAAAGGATGGGATT[A/T]TTAGAAAACTAAGAGTGAGGGAAGAACTAGGAGGTTTAGCAAGTAGAAAATGTTTGAAAT
2021	CaSNP4034	CCTTCACCACCACCATAGATTCCCAAACCAAAATATGCTACTTCATCACAATCTTCTGAA[T/C]CCTCAAAATCCCCAAAACTTCAATAATCCCCTTCCAAAGTGAGTTAATCCAAATCCACTC
2022	CaSNP4035	TAATGAGCTGGCCATTCAATCTCCCAATTATATAAGCAAACAAATAAACAGAAGTTTTTT[A/C]TAGCATTACTTCATTTTGTTAGCGACTAGTTCTTCAAATACCAAAAGTATGCAAAGCAAT
2023	CaSNP4036	ACAACCAACTAAATCCACTATCTAAAACTCAACAATCATCCCACATGATCATTATAAGTT[A/T]AAATCTTTTATTATCCCTAGAAAACCCTAGTATGGCATTGTAACAATTTGTGAAATCAAA
2024	CaSNP4037	TGTGACTTGTGATAATGGTAACTGAACGGTACTTGTTTTGTTCCTTGAGTTGTACTAAAG[T/C]CATAGGGGAAAAATAGTTCCTTATTGGTTATTTGTAGTTGGTAGAAGAAAATTGATGTTT
2025	CaSNP4038	TTATTATTTTTTCCTTCTCTCTCGTTTTCTACTTCAAGCATGTCGACCGGAAGAAAAAGC[T/C]GTGGTCGCCAAAAGATCGAGATGAAAAAGATGAGCAATGAGAACAACCTGCAAGTGACAT
2026	CaSNP4039	AATGCATCTGCTCTTCTTTCTCTCCGGAGATCCATGGCCACTTTCACACGAACGTAATTC[A/G]TTCATTCATTCATTACCCTCACTTCATTTATCATCCTACATTGCTCGTGATGCATGCTTA
2027	CaSNP4040	GCGACTTCAACTGGTACTAGAGCTCTGCGTCTAAGGTTAATCACCTTACAAGTGTTAAAG[T/G]AAAATATTCACGAAGCAATGTCAAAAGTCAAGTATATTGTTGAAGGAGGATCATCAAATA
2028	CaSNP4041	ATCAAGTTAGTGGCAAAACTCACTCTTTTGTATTCGACTACATTATTTTGTATTTGAATA[T/C]GATGTTGAAATTTTGAATAAAAACTTAATGTTACAGAGGTGTATTCGACTATACACAAGT
2029	CaSNP4042	CTGGTGAGGAAGGTTTATCCCGTGCCAATCCCACAAGTTCTATATTACAATTAAAAACAA[C/G]TGGTTATTGATATTTTTCTTTATTTGCTTCAATTATTTAATATTTATTAAAATTGGTTAA
2030	CaSNP4043	AAAGTTTAGCATATATACCATGTTTTGTTCCAAGAGGTATAGTACCTTTCTATGTAGTAT[T/G]TTAGTACTAAGTACCAACAACATGCATCCATTTGTGTATTTAGTCACATCCAAGAAATAA
2031	CaSNP4044	ACATTAGTCACAATTTCCAAGGTATTGGAAACAAGAAGGAAGTTTCTCATTCATAATAGA[A/T]CAAGAGGATAATAATAATGTATTCTACCAATCATATTATAGAAGTTTACCTAACAAGTGC
2032	CaSNP4045	ATCAACCTAGTCCTGGAGATTCATTTTGTGTTGGGGAATCTATGGGCATTGAATATATGG[A/G]TCATCTCATTTATACTGTGTTCGTCATCCACTTCTTTATTTGTGATACTATAAACCTCAA
2033	CaSNP4046	AAATTAAAAGAAGATATAAATGGTCCTATAATAGTACTTGCGTTTTGTTAAAATGATAAA[C/G]TAGAATTATTTATTTAAGGTGTAAATGCATAGAATTTTTTAGATCCACCTATGTACAATG
2034	CaSNP4048	GGAGGATTGAAGCATTCTTTATCTTTCGGGTTTTGAAGCGCATTATGGCATAATGAGTGC[T/C]TTTGGAGACGTGGGGTGATTACATGTTTCCTTTGGTGGAGTTTCGATTGTTGGATTTCTT
2035	CaSNP4049	AGTGTTTACTTTTTTTATCAGAGTGTTGTCTTTCCAAAGAGTTGATTTCCATCAGAGTTA[C/G]ACGATCAATGCATTGTTATACTTATAAATGGTATACAATCAAAGAATGATCATGATTATC
2036	CaSNP4050	ACTAGTTGGTCGTCGTCCAACTGCTCCTCCAGCTCCTAAAGGACTTTACATATATGGCAG[T/G]GTGGGGAGTGGTATTTATCTACTTTTATTTTTCTTTTGTGGCTAATTTATATTGTCCATC

2037	CaSNP4051	ACAATGTCATGTGATGTATGCCTTTGTATACAGCTCAAGAAAGGCTATTATTATTAATGA[A/G]AATATTTTCTTATAGAAATGGATAACACATAACAGCTGGGTTGTAAAACTGTACCCAACC
2038	CaSNP4052	CCTCAACAACACACATATATGAATGAAATTCTTTTTCCACAAAACACTCATCAATATCTC[T/C]AAAACCGAACTCCAAAATTTCACCCATAAAAGCATTTAATTCATTTTCCTCAAATCTACA
2039	CaSNP4053	ATATAGTTTGTGTGATAGCATGAAATTAATTGATCTTGCATTATTTATGAATCATTTCCG[T/G]TTCCAAACTAGTCATGCATACAAATTAAATTCTCTACAAATATCATATATATATATATAT
2040	CaSNP4054	GTTGATGTTATCATACCGGAGAATGATGTTAATCATTTTGGAGACAATTGTTAATCATGC[A/T]AGAGAAAGTTGTTTGCTGCTGAAGATTATGTGTAACATGCTGGAGAATGTTTATAAATCT
2041	CaSNP4055	TGTCACAATCTCATCACCAACCCAAGCAACCAATTTCTCATTTGCAGGAACTGGAAGGTC[A/G]GGAGGAGGTGAAGGAGAGCTCAGAATGGATAACTTCTTCTTTACATGGCTCCGGAGCATG
2042	CaSNP4056	CTGAATTACGAAATGAGAACATGTCCATGAGTACAATGTGATATACGACTTGCATTTTGT[A/G]GGTGAATATTAATCAAATTATGAGTATCGAGATTATTAAGAATTTAGATGGATACAATTA
2043	CaSNP4057	ATGATCTATGATGGCGATGACTGATAATAGTGACGGTGATCAAAGATAGATGATCCATGG[T/C]GATGTAAATGATTATTGAACAATGTTAAAGTCTACTAATAGTGATATGTTAATTTTAAAA
2044	CaSNP4058	TCATTTGGCCACTTCTTTGTCTCTCTATCAAGCTAACAATGAGATTGAGGAAAGGTTAAT[T/C]TGTTAAATTAAGTTTTCTGTATAGTTGATAACTTGATACAGTCCAGGCAATTTTCCCATG
2045	CaSNP4060	CCCTCCATTTCGTTCCAAATAAATACTGTGTTAATGTCAAACGCTACTTGGACTTATTAC[A/G]GGCTGTTGTTTGGATTAGTTTATTTGAGTTTATCCATTGACATAACTCATATGCACTTGT
2046	CaSNP4061	AGCTGCCAAGTATTTGTTCACGCTTACGATGAACTGACTCGTTCTAAGAAAAGAAAAGCT[T/C]GTGTCAACAATCATCAACTAATTTTTAATTAAGACAATTTTTATCTCTTAGGATCTGTTT
2047	CaSNP4062	CGTTTGAGAGGTAACAACAATCCAACAGTTCTACTAAGCACGTGGCTTTCCAGGTATGTT[A/G]GGTTATATTGATTGTATGCACTGGAAATGGAAAAATTGTCTGATTGCTTGGAAGTGCCAG
2048	CaSNP4063	AAATCAGTCGATTGGCAAATCGACTTTGTTGAGTGTCTGAGTAACAGGGAGAAATTAGCC[A/C]ATTGCCAAATCGATTATGTTAGTGAAAATGAATCGACTGAGTAATCGATTGCTTGATCTC
2049	CaSNP4064	ATTTATATCTAATGACCTTAGAACCATTGGAGTACACAACAAATGTGATGTGTCCATATG[A/G]AACCATATTAGTACTCTTGTTATATAAACTTCATGATTTACTTAAATATTATTCTTCAAA
2050	CaSNP4065	CCACCTAACTTGAAAGGAAAATTTAACTAAATATAAATTACAAGTCTTCAGGCCTATGTA[A/G]GGATCGTAGTGACTTCAATTCAAAGGACTACTCACTATGGTCGAATGAGAATATCTAATG
2051	CaSNP4066	TTGTTCAAGTAAGAAGAAGTCAAAAACTCTTCAAGGAAACTCCAGCTCCCTTCACTATAT[T/C]TGTGGATCATCATTTACTGAGTTTATCTTTATTTATCTTAAACAAGTGTTGAGAAGTTTC
2052	CaSNP4067	GTGGTGGTATATTTTGGAAATCGAAGAAAGAGATGATACTGGCTACGTCTACTATGGAGT[T/C]TGAAATGATAACACTAGCTACTACTAGTGAAGAAGTGAGCTAATTGAGATGTTTGTTACT
2053	CaSNP4068	CAAACAACACAATAACTTAGTTAATTTACAATAATGATTTTGACTGTAATCAAGAGGGTA[T/C]AACAGTTGATGCTTTCTTGTACAATGATAACAATTTAATATAATTTCAAGTACAATAGAG
2054	CaSNP4069	TTGTCAACATCTTCTTAATGAGTGTTATTTGGTTCATCAACTCTTTAGTTTTGTAAACAT[A/C]ATAACTGACTTATTGTTGATGTTGACTACTTTCTTCATGTCCAACATGAAAATTGACTAC
2055	CaSNP4070	GAAATATTTATCAAAATTTGATGGTGATGGAAGTGGTAGTGGAAGAGGAGGAGAACCTAG[A/G]GAGAGAGGTGCGTTTGGCCGAGGCCGGGGTCGTGGCCGTGGTAGAGGCCGGGGTAGAGGA
2056	CaSNP4071	TGCATTATTATCATTTCTTTTTCCCTTTACAAATTATTATATCAACCTATGTAGTTAATT[T/C]CAAATAGTGGTACGTCGTGATCGACCTCAAAAAATAGAATAATGGTTTACGAGTTATCGG
2057	CaSNP4072	AAGAAAGTGTTGTTGGGAGATTCCCACACCACTAACGTTGCTGGTATTGGAGATGTGGAG[T/C]TGATATTCACCTCCGGAAAGACCTTAATTCTGAAGGATGTGATGCACACTCCAGAAATAA
2058	CaSNP4073	TTAAGTTCTCTCATCATGCTCATTTGAAATTTAAGCTACATCGACTTAGCAAATTCTTGA[A/C]AAAAAGTGTTATTAGTAGATCCAAAAATAATGTCATCAACATATATTTGAATAATCATAA
2059	CaSNP4074	TCCTGGAAAAGGCCCTGAAGTTTTTAAAGGTGAAGCTATGCATTCTATGGATTATTCTGC[T/C]TTGGACAATGACGTTGCTGCTAAGTTGATAAAAGACAAGAGTGTTACAATAGTAGGCTCC
2060	CaSNP4075	TTCCAAATCACTGTCTTCTTCACTATCATCAACCAGCAGCCGGACTCTTTTAGCAGCACG[T/C]GCATCGCCTTCTGAAAATTTATATGGCCCAATACACACAATAAAGAAGAAACCACTTGAA
2061	CaSNP4076	AAATTAGTAGGTATACCGCGAAAAATTTATACCGACATTATTTTTATATGTTGTTTATAG[T/G]TAACACATGTCTACAGTTTTTTTTTACAGTCATTATAACCTACGGTAAGGAACGTTTGTT
2062	CaSNP4077	ATTAGCAACTATTTGTAATCTTCAATCCTACATAGAATCTTCAACCTAGTTAACCACATC[T/C]CTAAGAAAAGCCCCTAATCTAAAAGTAAAGCTTAAGTTTTCAACAAAAACATTAAAAAAA
2063	CaSNP4078	TCAGGACAAAGGAAAAAAAAAGGGAGATCAGAGAGGATATCTGCTAGCTGGAAAGGCAGC[T/G]GGAAGACTGGGCAAAGGAAGGTGAGTCTAGTGATGGAGTCGCCTACCAGAAAGGTTGACA
2064	CaSNP4079	TTTTGGTGACATTTTTTGAAAATAATTACTAGAGAGAAAATGTCAAAACAATATTCTGAA[T/C]ATTAATCCTTCATTTGGAGAATTCCATGCTTCCGGTTAAAAATAAATCAAGGTTAAATAT
2065	CaSNP4080	TGGTCGATCTTTCACTATCTAATTTTCCAAATGTCATGCTTTTGTTGATTCCAAAAATCC[A/T]AAATATATTAGGTGAGCCTTATTCAGGACCCAGAGGAACATAGGTTTCAAACACACGCTA
2066	CaSNP4081	GAAGTACACTTTACACAATTGAATGATACTTCAGAATATTTTTCTGAACTTACAGATCTT[A/C]GTATTTCAGGCAAAAGACATATCAAAATCAATCTTAAATTGACTATTTAAACTATGGAAA

2067	CaSNP4082	AAACACTCAATTTTTAATAGTTTTAACATTTGAGACACCTATATCTTCATAAATTTATGC[T/C]AGACTTTTCCATCACAATTTTGTGAAAGTTTTTGTAGCCCCTTTGACCACCACAAAAACT
2068	CaSNP4084	TCTATACATTAAGTCATCTTTTCAAAATTTTCCGTTTCTAATATGCATAAATATTATATC[A/G]TACAATTGTAAATTATATGCTACACCAATTTACCTGAAATTATTCAGATCACTTGCATTA
2069	CaSNP4085	ACTCTAATATAATATTAATATTTTTTTATAAATGTTCTAATTCATCTTAACAAAATCAA[A/T]TTGTAAAGTGAAGAATGTTACTATTTATATAAACTCTTTTTAAACTTTATTTATTTCAAT
2070	CaSNP4086	GATGACCAGCTTAATTATATTGATATTTTGTCAATTATCATCTATTCCTAATGCACAAAG[A/T]CTTGCATCTGACTTTGAATATCAAGTCATAATATGACTTTTGGTTATTAAAACTATCTTA
2071	CaSNP4087	AAATAAATTCCATTTCAAGTCGAGAAATAGTGGGGGTAGTGAACCTAGTCAAGTTCCTGT[T/G]ACAAGAAGCACTTAAAGCAACAAACAAGTCGAAACAAAAACTCAAAGGACTAAGAGAATA
2072	CaSNP4088	TTGTCTCTACGTCAAGGAAGGACTAGTCCGTAAAGCTAGATGATGCACTTTGAGCCTACT[A/G]TACAACATAGAAGACTCCTATTGGTATGACTCCTTACAAGTTGATCTATGGAAAGTCTTA
2073	CaSNP4089	TTCCTAATATATATAGGCCTGTTTGGATGATTTATTTGAGTTTATCTACTGTCTAGCACT[T/C]GTGAGACTTTTTGAGAGATTTATTAAAACAATTTATGACATGTCCATAAGCTGTTTTAAT
2074	CaSNP4090	GGAAGATGGTTCTTTTGACTGCTGTCCATAATCTGTTACACACACCTCATTTCTACCATA[T/C]GCTCCAAAGATTGGCCAGGATGTTAATGTGATGTACCATATGATATGATATTTATCTGAA
2075	CaSNP4091	ACCCTCAAGTCACTATCATTACACTCTGGGAATCATAACCTAACATCTTTAATATATCAT[T/G]TCATATTATTTGGAGTTTTCTATTTTTTGGTCTCAAATAGGATTCTACTTTTGCAAAATT
2076	CaSNP4092	GTAATTCAAGACAGAAAAATGAAAGTGACCAAATCTCATATGCCACAATCAATCATCATT[A/T]GGAATTATAGAACTCAAGCAAGGAAATCTTCCATGTTGAATTTTCAAATGGAATATGTGG
2077	CaSNP4093	ATAAGTTTTAGAATTATCAAGTAGTATAATTTGTGTTATGCAACTCATGGATTAAAATGA[T/C]GTCGTTTCTAGTCAACCTTTTGATAATAATTCTTATTTATTAATTTATTTATGAGTTTTA
2078	CaSNP4094	AGATGCATAACCCTTCAAATTTAAATCTCTCATGCATTGTGAACACAACAGTATTTAATT[A/T]TAGGTCAGTGAATGGTCATTCTCATTACATTTTCATAAATTATTAGAGAGGTTATGCATG
2079	CaSNP4095	TGGGTTTCCACTCTCCACCTAAACCCACTTTCTAAATAGGTTTCCATTCTCCACCTAATA[T/C]CCACTTTCCAAATGTGTCTCCACTCGCCACATAACATCAAATTTGATTTTGAATAGATTC
2080	CaSNP4096	GTCCTTCAAGGCAAGCTTCAGCGTTACGCACAGCTATTGGAAATTTAGTGCCATTTGAAG[A/G]GATAGTATTGATTGACTTTCCAATAATAGTCTTTCCATTTCCAAGGATGAGTTTATCAAT
2081	CaSNP4098	CTTCTTAGACTCCTAAGAATTTTCTTCACATGATCAATAATAGTATAACTCTTCTCAAGT[A/T]TCTTTAATCATGAGACCATGGTCTGGAATCTGGAAAACATTGTTTCGATTTCTTCATCTT
2082	CaSNP4099	ATACTTCTCTTTTTTACTCTGTCAAAATTGATATATGTATTTGGCATATAGAGAGTAACG[A/G]CATTTATTCGATTTATAGTGCATGAAATTTTGCATAACTGATTTTTAAATAGGTAGAATC
2083	CaSNP4100	AATATCACTTTGACTATCGACAAATAATACATATTTGTCAGAATAAACTCAAACTCCTGC[A/G]ATAATTTCATCAACCAAATCAACTATTTGCATGTTTCGGTAATGACAATGAACTCTGCCT
2084	CaSNP4101	ATTTGATCATTTGGCTCTGATACAAGTCAATGCTTTGACATAAAGTCAACACTCTAATAC[A/G]AGTTAATGCTTTGATATAAAGTTAACACTTTGATATGAGTCAATGCTCTGATAGAAGGTT
2085	CaSNP4103	GGTGTAACTCAATAAGAAAACTCAGGTTCTTTAGGTGGAATGACTTGAGGTATTGATTCA[A/G]CAAACTTCCCATAGCCTAGTATCTAGTGGTCATTCTAAAATTTTGTAGAATGAACATTAT
2086	CaSNP4104	CCTTGGTTGCCAATGGAATTATATAATGCTGATTTTCTGAATCATATAGGGAATGCTTTC[A/G]GATCTATGCTCAAAATTGATAGATTAACGTCCCTACATTCCAGAGAAAAATTCACCGGAA
2087	CaSNP4105	CTCTCTTTGATCCATCAAATGCTTTAACTATCATAGAGCAAGGCTTTATATAGGATCAAT[T/C]AACATAGAGTTTTGATAGTGTTGATTGCGACATAACTTTCAACGATAGACCAATGTCAAA
2088	CaSNP4106	TGAGGATATAATTTATATTATATGTAAATAAAAATAAAAACCTACAACTAAGTGCTAAAC[A/G]TAGATGTTATCAAATCACATGTTTTGTAGACAAGCGTTCTTTTTCATTCTTGTGGAGTTA
2089	CaSNP4107	ATGACCAACTTAAAACTTTTAAAATATATAAAATTAAAATAAAGTCTGAAATAAATGTAA[A/G]GGACTAAATATGAATTTAAGTCAAGGTATTAATTTCCTCATTATATTTCCATTAAACAAC
2090	CaSNP4108	CTGCTTCCATTTGATCTGACAACTTAGGGGGTACAACGCACAGAATCACAAGTACTTCTC[A/G]GAGATTCGACAGGTTGGACCAATGATAGACTCACAAAAAAATAGAAATAAGTGAACAATA
2091	CaSNP4109	ATTTACAGTCTTGTCCATTGTAGGGTGAAACTACGACTACACTGCTATTTGTTCCTTCTG[T/C]TGATTGGCTAGTTCATGAGTAGGACTTATCAAAACGATAGTAACACTGTACAACAATGTG
2092	CaSNP4110	AGATCATCCTGATGATGCACCAAACATTAGCCTTGTTGAGGGTACCAATCCATTATAAAA[T/G]GTATCCATTTTGATTCAAATAGGTATCCCATGATGAAGACATTTCATAAGAAAATCCTTA
2093	CaSNP4111	TATCTTCTTGTCCCAAAAGCCACCTTGTAATTTCATTGTTTCATCATATGCTTTATGATT[T/C]TTGGGATAGTTCAGTAGTAAGTGAATTTTCCAAAAAGATTATGATTTCAATTGATGACAA
2094	CaSNP4112	TAGATGGTTGAATTTGAAGTAAATGCTTCACTCGGTGCGGTGATAACAAATCAATTGATT[A/C]AAACAATTGTACTCATTCTGTCAACGTTACTTACAATTTTAAATTGCATTTGCATTGACA
2095	CaSNP4113	AATTATTTGTTAATTAGTTATAATAATTGTCTTAATAGGATCATTAAATAAAGTGCATTT[A/C]TACACACCAACTATAATCAATTTTATTTATTTATGTATTGAACTCATTTGTAGAAGTTTA
2096	CaSNP4114	CAAAGCATTCTCCACCAATATTCCCATTTTTTTGCCGAACCCAACTCCCTACTCTCCAAC[A/G]GCCAACATACCACCATATTCCCTTAATTCAAAAGAAGGAAATTAATTCAGGAAATGATGA

2097	CaSNP4115	GCAGTTTTCGAGGAACTAGCATGTTCTTCGTAAGCAAAGGAATGACAATGAAACATGATG[C/G]TTTTCATTTCTTCTTCCAAGATACATCTTCTGAAAACTTCATTAGCCCCTCTCTTAAAAA
2098	CaSNP4116	GTTCGAACCCAAATAAGACATTCAACTTAACAATATCAACAATTGGATTCATACACCTTC[A/T]TAACAACAGAGGCAATTAATAATGAACAACAAATTAGAATCAACAAACTCAATTGGAAAC
2099	CaSNP4117	ATATCAGGACGACAAATTGGTGTCGGCTTTGAGTCTAATGGCCTTTATTATCTCTCAAAC[A/C]CATCAATAGTATGCCCTACCACATATTCTCCTCTTACTATACATGCTCAGTTAGGTCATC
2100	CaSNP4118	GTTGACTTTTATAGAGACATTGACTTTATCATAGGATGTATAAAATTTCAAAGTCAGACG[T/C]GTGATGAGATTATTAGAGGCAGAGGCAGTCACATGCTCGTTACGCTTGTTAGAGGTAGAG
2101	CaSNP4119	AATTTGCACATTGTATTTCATGTATCCCAACTTAGGAAGTATATACATGATCTGAGTCAT[A/G]TAGTACAATCAGACATCGTTCAAATAAATAATAATCTTTCTTTCAAAGCTTTGCTGAAAA
2102	CaSNP4120	GGTGGTGAATACACCTCATAAAATCTTTGAATTGCTTGCTTATGATGGCACCATCCACCA[A/G]ACATCCTATACGAATACTCCTCAACAAAATGGAGTTGTTGAAAGGAAACACTGTCATATT
2103	CaSNP4121	CTCTGATGAAAAATATTTCTTCATGCTCTGATGAACAATATTTCTACATGCTCTGCTAAA[T/C]AATATTTCTACATGCTCTAATGAATAGTAGTTTAAGTATGCATTTGACGTCTAAAGCCAC
2104	CaSNP4122	GTTATAAGACTTATCGAACCATAATGAAATATCTTCGCCTGCCAGAAATTAATAACAGAA[T/C]AATATTACGACAGCAATTTACTACGAATCACTGATACAGACATCAACATAGACACGACAT
2105	CaSNP4123	CAATATAACATTTTTTCACTGATATCGCTAAGTCTTCGGTATCCACGACATTCACACCAT[C/G]TTTAGCAACCAAAGCTTTTGTCCAATATGATTTACTAATGTTCTCCTCTCTAGCCACAAC
2106	CaSNP4124	GATGAAGAAAGTCGTGAATTGTTACTTTAAAGAAGTATCGTAGTTAGAGTGTTGAATAAC[T/G]TTTAAGGATTAGAACGATGGATCTTGTGTTGTATTGATCTATCAAATCAAGTTATACTTG
2107	CaSNP4125	AAAAAAATATGTTGTTTTCATACATGTTTGTTGGAGTGAGTGCAATGGAAATTAGAAGAA[T/G]AAAAAATATAGAATGGAATACATATAAAATATATATAGGTCGGCCTGTCAGGCCTAATAG
2108	CaSNP4126	ATCTGTATGCTTTTACTGTCTTTCAAAATGAGTTTCTGTTAGTTTTAATTGGACTCATTA[A/G]CTTCTATTTTTAACTATTTAATGCGGTAAAGTTGTCGAAAATACTCAGTTTTCGAAAATG
2109	CaSNP4127	TTTTTCGTTCTTACTTTTTTTAGATGTATTGTTGATTTTTTCATAATTTCTTCTTAACAC[A/G]TTGTCTTTTTCATTCTCTCTCATGCATCTATTAACTAAGTGAGTTCTTCTTAAACAGGGA
2110	CaSNP4128	ATGTGGTATGCTGCAAAGAAGAATGTGGTGGTAGTGCACGGTTGACGGTGGTTGACGGCA[A/G]TTAATGGGATAGTTGAAATGACGGTGATGGCCTCTATTTATTGTTTTCTATTTTTTTCTTT
2111	CaSNP4129	TTCATCTCTGCTGAAAGCTATTTTGTAACACTGTCGCAAAGCTGACTAATGCTAATAAGA[T/C]TGTGTTTTAATTCTTCAACATATATAACATCTTNNNNNNNNNNNNNNNNNNNNNNNNNNN
2112	CaSNP4130	TAGCCGAGAGGGAGCAATTGCCATGGCAACCCTTTGAACTGATCCCACAACACTTCGCAC[A/G]TATTGACGAGCCATCGCAGCCACATTGTCTCGCATATGATTCTCAAAGGTAAATTGAAAT
2113	CaSNP4131	TGGTGACCGTGATGGTCCATTGATTGGTTCCTTGAGTTGATTGGTCCACCTTATCCTTAC[A/G]AGAGTTTTAGAGTCGTGTAGGTTTGTGATAGACTGCACTCTAGTAAATAGTGTTGATATG
2114	CaSNP4132	ACTAGTAATTGACTTTATATAAGTAAGAGATTCAAAGAGCCCACACCATACTTCCCAACT[A/C]ATTGTGCAATACATGAGATAAAAATCCATGAGAAGAATCATTAACGTAACATATATAAAC
2115	CaSNP4133	TTAATTTTGTCTAAGTAATGCTACTAAGTTTCTTTCTCTCAGCCGCCTCTCCCAGATCTT[A/C]ATCCTAGGTTGTCTTCTACTTCCTCCGATAGAGATGAATGCTTTTTGATCAAATTTTTGT
2116	CaSNP4134	TCTTGATTATAAAATGCTTATCTTTACTCTAGATAATATTCTTAAGCATTAACAAGTCCA[T/G]ACCTACTCAATCATCCAGCTAGTAATAACTTTGCATCAACTCAAATCTACTAAATACTCT
2117	CaSNP4135	TTTCTTAATACTGCATTATTAAACCCTTCCTCTTCAAAATCAGATTGTACTCCCATTTTC[T/C]TACACACAACATCCATGGCTTCTTTGTACAATGTACTTTCAAATAATTCTAGCTCATGGA
2118	CaSNP4136	ACGTTTGGATTTGCACCCCACGTTTTGAAGTGTATAAAACCAATTTTATTCATCAGTCTA[T/C]CCGTAGATTGACCCAACCCATTCGTTTGCACACATTTTCTTGCGGAACATTCGGTTTTTG
2119	CaSNP4137	CTGTTCTTTGTTGAAATTGGAGATTCTGGACGGCCATCAGCTTAACGAGGAACTCTAATG[A/G]AGGTGCATTGTTTTGCTTTTGAGGAATATTTTATTGTTGCTGCCTAGGTTGCTGTTGTGC
2120	CaSNP4139	ATCTTTCATTTACAATATAAACAATAAAAAAAGTCTAGTTTATTTAATTTATGTCGATGT[T/C]CATTATTGATGTTCCATTCAACAAATCGTTAGTTTGGCCTCTTTTCAGTTACTTCTTATT
2121	CaSNP4140	ATGCTGTTGTGTGCTTGTTGAACTGCATGTACTCTAAATGTGATTCTTGTCATTGTAGTT[C/G]TTGAGGTGTTTTAAGTAGGGCTGGACAAACGGTAAGGTTCATAAATATCCAACCCAACTC
2122	CaSNP4141	CATTTAATCTGGACACTTAAATGGGTGTTTAGAAATGTTATTTTATGGGAAATAATCCAT[T/C]GGTGCTGAACTGAAATATCGTATCTTAACTACCCTGTCCAGCTACACAAGATTTGGAAAG
2123	CaSNP4142	ATTCTCTCAAGAGTGCCTTCACTTGGTTGGCAGATATATTGCTAGGCTTATCCCTCTCTT[T/C]TGACTCCATGAATTCAATGGTTTTACTAAAAAAATCTACGCGGACACGGTTGGCAGATAG
2124	CaSNP4143	CGTTATCACAAAAACAATGAATTCAAGTCAAAATAAAGAGAAGATAAAATTGAGGACAAA[T/C]AGTTATAAGAGAAACAATTCAAGAATAAGAGGATACAAGACATAGGTTAACGATAAGAGG
2125	CaSNP4144	TTGTTTACATGACATATCAGAGTTAAATTGGTATTTCACATTGCATCCAATAAAATATAC[A/G]AACTCATAATGAATATTGAAATAGGAAAAATAATCACATCAATAATAATCAGTTGTATAA
2126	CaSNP4145	AACCTCTCTAGACCCTCTTTATTATGATGCATCAACAGGAAACAAAAAGTTTAATATATG[A/G]ATTCCTCAAGACTACTTGTTTCACTCATCTCTTGAGTTGAGTGTTCTTTACTCTTCCTCT

2127	CaSNP4146	CTTCAACTTCTCTCTTCTCTATAATGTTTATATGTCTCTTGTTAATATTCAATGTTATAC[A/G]GTGTCAAGTAGTTCATGGTGTTTGTGTCAATAGTATTGCTTATTTATAGTAAAAAAAGT
2128	CaSNP4147	TTAAGGTCCTTGAAATCCAATCCTGGAAAGGGGTTGTGCTTCAAGAAAACTAATGACAAA[A/G]AAGTCTCTATATTTACAGATGTTAATTGTGCAAGATCAATTATTGATAGAAAATCCACCA
2129	CaSNP4148	CAGTGACATATAACACTTGTTGAGCTTGTGATACTAAATAAAAGATTCAGCTATGTACCG[T/C]ACTTTAATAAGGTCCATCTTCCCAAACCCACAATTATCAATTTGAACACCATTGTTGTTG
2130	CaSNP4149	GTCATTCTTGTTGAATATGAATTTTACTTACTATGCAAAGGTTCAAGTTGGAAGACATAT[T/C]TGTTTATGCAAAATCTTATAGAAGCGTTTGACGCTACTTCATTAAACTTTTTTTTTAAT
2131	CaSNP4150	CATGTCTCTATTTTATTTTATTTTCAAGTTGCAGCAACACACAAATTAACTTAGGACAA[T/C]AATTCAAACAAAGCAACACAATTAGAAGTTTATGTTTCACAAAATAAAATGCATAAACAG
2132	CaSNP4151	TTGCATCCAACAACTGACAAGATATATTCATCTCATACTTCTTTTTCTTTGAACAGTAAG[A/G]TATAATTTAACAGATAAAAACAACCATGTTCAAAAAGAGATTGACCAAAATTTTCAGAGG
2133	CaSNP4152	TAAAAAAATCCATGAAACATAAGAAATATGACATATCTCTTGAGCAATTGGGTAATCACC[A/T]TTATTCAGTAGAAGAGTACTGCAAACAAGAGGATACTAAAAACCAAATTTCTCGTGAAAA
2134	CaSNP4153	GATCATTAATTTATTACTTTATAAAATAAATGTATGAATACTCTTTAATTTGTAAATATA[T/C]GGTATTTCATCATCACGATTTTTTAATTTGTAAATATACGGTGTTTCATCATCATGATTG
2135	CaSNP4154	CCTCGAAGAAATTGCGAGAGATATTCAGATAGCTGAGCCAGAATGGAGGATCATTTTACT[A/G]AGATACTTCAATCCAGTTGGGGCCCATGAAAGTGGTAAACTTGGTGAAGATCCCAGGGGA
2136	CaSNP4155	TTGTACATGTATTGTTTCAATAAGGCATTAGCCAAATCCTTCCATGAACGGATTCGATTT[T/C]ACTCAAGATGCATATACCAACTTAATGATGCCCCACTCAAACTGTCTTGAAAAAAATGAA
2137	CaSNP4156	TAATTTCTCTTCCAAATGAATCTGATTCCAACTGCAATGCTAGGAAGTGAGACAATGATG[T/C]ACTTTGTAATTCTCTTGTGAAACCAATCAAACCAAATATAATCACATTTTCAATATTCAA
2138	CaSNP4157	CAAGTGTTTGTCGAGAATACTCCAGGGGTATGAGCTATGTCTGATAGGTCTCATAGCCCC[A/G]GTGTATTTTGTTGTTTAAATACTTTGGATTTTTGTTTCAAAAACTATTTTCGCTGCTTGT
2139	CaSNP4158	CACTAGACAAAGATGAGTCACACAAGATGGTTGATCAGAAAAACATTGGATGCATGATAG[T/G]TTATTTACTTTATTTAACTGCACTTAGGCCTAATATCATGTTTAGTGTGTGTGCACGCCA
2140	CaSNP4159	ACACCGTTGATCTCAAGAAAGGAGAAGTCACCAAAGGTTTGTTTTTCTTTCTTCTTTTCC[T/C]CTTTTCAATTCAATTCATTGGTATGAATGAAATAGTTATTATTTCACGTGTTTATGTTTG
2141	CaSNP4160	ACAATAAATAATAATATTTTAAAAGCAAAATACTTGTCGTAGTCTTTTAACTTAATTTCA[C/G]GTAACAATTCAGTTATTTTTTTTCCTTATCGATTTGATCTTTTATTTAATTTTAAGTAAC
2142	CaSNP4161	TGATCTTTTTGTTATTCCCTCTAAACAATCTTGGTGGAGTAAGAAGTTTGATGTAATTAG[A/G]GATAAGGTAAATTCTTTTGTAATTGGATTGTCTACAATATCTCTCCATTTTCTTTAGAAA
2143	CaSNP4162	CACGAACTTCTTTGAATTATGTGAAACAATGAAAGTGGAGGGTTGTTCTGAAGAAGGAAA[T/G]AGATTGAGATTATTTCCATTATCATTGAAAGATGATGCTACGGAGTGACTTAAGTTCTTA
2144	CaSNP4163	TTTGGTAGGCTCTAATATCATATTACCTTTCTATATTGAGTCTAACTCATTTGACAAAAT[T/C]GAATTACAAGGTGTGGATCACTCCCTTATTAAAATGTTTCCAAACTATATCTCATCTAAT
2145	CaSNP4164	TTCGTCTTCTTGTGCTTTGGCTATGACCTCCTATTATGGTTTAGTCTCTCTTATCCTTT[T/C]CCCTTTTTCATCACAGTGGTTGCAATTTTCCTCCTAAAATTTCTTTCTATTTTTTTTAAT
2146	CaSNP4165	CGAAGTTAGTTGTTTTTACTGACCATGCAACTTTGAAGTTCTTGCTGAAGAAACAAGAAA[A/C]AAAACCGAGATTGATTCGGTGGATGTTGTTACTCCAAGAATTTGATTTAGAGATTAAAGA
2147	CaSNP4167	TTTAGCTTGTTCTGTCTTATTCTGCAGCATCCATGGGCAGTGTGCAATTATAATGTTTGA[T/C]GTTACTGCCCGGCTAACATACAAGAATGTACCTACCTGGCATCGTGATCTTTGCCGGTTT
2148	CaSNP4168	ATATAACTAAAATTTTAATATAATAATTCAATGAATAAAATTTAATATATGAATTAGTTG[T/C]AATTACGTTCTCCAAAATATTATCAAATGTGTATTTATAAACTCATATATAATAATTACT
2149	CaSNP4169	ATTGGGTCAAGGTTCACTCTTGACTGATAGATGACACATGAGTTTTCTACCACACACCTC[T/C]CACCTAAACTTGACATCACCAATCCTTATGAAAATACAATTTTGCTCTTTTAACTTGAGA
2150	CaSNP4170	TTCTCGTTTACTTCACTCCCCGGAAGAAATCGACCGTTTCCTCACATACTACTCCGACAA[A/C]AGGCTCATTATTCCTAAGTATGGTACTCTTGATTCCTTTTCTGCTTTTAAGTTTCTTGAG
2151	CaSNP4171	TATAAACAAGGAACATGTCAGACAATCTATTGCACAAAGAAAGAGCATTTACAAATAGTT[A/G]ATTTCAAACCACTTTGAAAGAACCCTCCTCAACCAATAATCCTCTCAATCATTTTTTAGT
2152	CaSNP4172	AGCTGCAGAAAAGGAGAAAGATCAACATAAACCAACAATGTTCTTGGTTTCAGAAAGACA[A/G]CATACTCAACAAGACAAGACTAATGCAAACCCAATCTCAAGACAGCATACTCAACAAGAC
2153	CaSNP4173	ATAGATATGTGTAAAAAACTTATTTGATTGATTGTGTTTGTTGAATAGTTTACAAGTATT[T/G]CAATTTATTGGCAAATGTTAGTCCTAGTAGCCATAAGTCGTGGCCTACATAAATATAGTT
2154	CaSNP4174	TTTACATAAGATAAATAAATGCTTGTTAGTCAAAAAATTTGGACTGTTATCGTAATAGTG[A/C]TTGATACAAAGAAGATTAAAAATGCTTAAGGAAAGAAAGAAACTCTCTTTTATTGTAGAG
2155	CaSNP4175	GAAGTAGAGTCTAAGCTCATGCATAGCATTGCACTTTATTGTGATTGTTTTGTTGTGATT[A/T]GTATGTATTGTGTGTGGTGATAATTTCTCTTAGACAATTTGTTGTTATAGTGAAATTTAT
2156	CaSNP4176	TCATCAAATGGTAATAAAGCACATTAAGGATGTGTGTCCTTTTTGTTAATTTGTAGCCCC[C/G]ACAATAAGCAAAAGGTAGTGATTTTTTTTTTCAATTTATTTTATTTTAAATTAGTAGTA

2157	CaSNP4177	CATATCATCGTACTTAACAGTTGCATACATTTTTTTAAAACAAAACTCAATATACTCAAG[T/C]TTATAACGAGGATCAAGAAAAACATCATCAAACAAAGAAAATTTTATCTCACCACTATCC
2158	CaSNP4178	GAAAAGTGTTTTCTTTAATAGTTACATAAATAAAGAAGTGTATGTCAAACAACCTCATGG[A/G]TTTGAAGATAATCAATTTCATAATTATGTTTTTAAACTAAAGAAATATTTGTATGGTCTA
2159	CaSNP4180	TTTTTATTCTTTTGTACTTGCCAACTCTCTTTTGTCGATTTTCAACACTTCAGCAATTGC[A/G]CATAGACTCGGTGGTTGACCAGTATATTGATAAGGATTTCATTTCTCTAGGGAGTAATCC
2160	CaSNP4182	GGTCTCAAGATTAAAGGTACTTGAGAAGAGTTATACTACTACTGATCATGTGAAGAAAAT[T/C]CTTAGGAGTCTTCTAAGCAAATGGGGACCTAAGATAACAATTGTTCAATAAGTTAATGAT
2161	CaSNP4183	CCTATGCTATCTAGCTCATGTGTTCTCAGGTCGCCCCTGCTCAACTTTCTCTGCCACGTT[A/G]CACATTCCACGAAGTGAAATTGATTGATGTTAAAATGAAAGGATAAGGTAGGATGTGTTA
2162	CaSNP4184	TAACTGTCCTAATGTTCAATAGACCTTAATCATTTAAACTCGTGAGCTCCTCAACCTATA[T/C]TATACTGCAATTTTATTTAGCCAATTCTAGTAAACGTTGGACTTCTTGTTTTCTGCAACA
2163	CaSNP4185	TAAATTTTCCCCACATATATTACAATCAATGCTTTCTAATAATTGTTCTCATGATTTTTG[T/C]GTGTGTTCATTTTAACTTGTGTAGGGAAGGTATGTCGGGCAGGTGAAATGACGACCGTGA
2164	CaSNP4186	AACACCTCGGAATGTGGCATAGGGGTTTCTGATGTGGTCGGCGCTCGAAAAAGGTAGCCG[A/G]AGAGAACTGCACGTACGGGTTTTGTGGTGGCGTGTGGGGCCCACATGCAGAGAAGGTGGA
2165	CaSNP4187	TCATTATTTGTTTGTAAAATGTGACAAACACTTAGTGGGATTCATCCTTACTTCCTAAAA[A/T]TTTTACCCATGTCCACCTAAGGTAACCATCTACTATATCAAAGCCATATTTCTTTCCAAT
2166	CaSNP4188	GTAGCTCATATTTCAATGTTGCATCTAACTGAATCTAACCAAGATTCCAGTCTAGGTTCG[A/G]TTGGATATACTCTTGACGACGTCAAGATGAGGCCAAATGATTATCATCATGTCTTATCTG
2167	CaSNP4189	GTTTCTTGCACTAAATCAAGATAATCGTGATCAATTTTAATCCACTGTGGAGAATTTGTA[A/G]GGTGCTGAAACTTTACATCTTTAATTATTTCACTTGCGTGTCATGTGAAGTGGTTTGCAT
2168	CaSNP4190	ACAAAGGTTATCATCAGCACACAACAACAACCACTGCAAGTTAAGAGAAGCATTTAGGAA[T/C]TCATGAGCAAGACTATAATATGCACGACTAAAAATATTCCTCATATTCTGAGTAGTTACA
2169	CaSNP4191	ATATATGAAGCAGTGATCATATAGCAGACTAACAGCACATAAAAAACAGTTAGTTTATAT[C/G]CCAGTGATATTTGTACTGACACTTCTGATTGAAAGAGTGTTTAGTGTCCGCCATGTGTCA
2170	CaSNP4192	ACTAGATGGTAGTGTAAAACAGTTTTACACTAACAATGTATATCCTTTAAATCCTTAGTT[T/C]ATTTATATGGTTTTGTTTTGATCAATTAATTTATATGATAAAAATTGGTTTTTTATGTTC
2171	CaSNP4193	GAATTATTTATAAGAAGTTTTGATCAATATTTATCACAAGAAGATATGATTTATTTACAA[A/G]AAGATTTGAACCAGATTTATCACAAGAAGATATGATCAAGAATTGTTTACAAGAAGAAAC
2172	CaSNP4194	TTTCTCAAACACAAATTGATTAGAATAATAATAATACTTAGGTTCAAACTTTTAGTGTTT[T/C]CTTTCATTTGGTGGTGGAGGTGGAGCACACACAATAGCAACCAGAAAGACTACAAACTGC
2173	CaSNP4195	ATTAAAGTTTCAATTTGGGCTTTTGATCCAACATAAAAATTGTAATTATGATTCTTAGCT[T/G]TCCAGCGCCTGTTTGCACGTGTCAATCGGATATTCAGAGCTCAAGTTATGGCCTATAGAG
2174	CaSNP4196	AAACAGAGGCGGATGCGGGTTTTCTCCGACTAATTGATTATGTTATACACATGCTCAATG[T/C]TGTCAAATAGCGACTATAGCGTTATAGCATAATGAAATTGGAACAAATTGCTATTGTTCC
2175	CaSNP4198	CGATCATAAACTATATTTTTCTCTTTAACAATCTTTATGGTTTCCTTTAATAACAATTCA[A/C]ACTTGAATTGCTCAAGTATCTCCCAAATTATCTTATTATATAAGTTATTTTCATATGTAT
2176	CaSNP4199	AAGAAAATGCATGTGATTTCTTCTGTCATTCATATATTTTTCAAGTTCTCTACTTAAAAG[C/G]TAAGTGCAGAGAAGTCAAGGATTTTCTATTTGTAAAATATGTCATGGAGTGTTCAACAAC
2177	CaSNP4201	AGTATGAATTGACTTATCAAGTATGCAGTCTTGAAATTTGACAGTTTTAAGGTACTTATG[A/G]GAGTTACAAATTGATTATTTCTCATAGAAAACGTTTTTCATTGGTCTAGCTTTATCATAC
2178	CaSNP4202	ATGTTGAGCAAAAGAAACGGTATAAGATGCATCATAAAGCTATAGCCTTAATGATAAATT[A/C]AATCACGTCCATAGAGTTTGATAAGTGCACCAACAAAGAAACATGTAAGAGTATCTTTGA
2179	CaSNP4203	CTTTGCATTGCATCTAGCTATTAAGCCTACAGCTAGAGAGTGTTACTTCATTGTTTAACA[T/C]AATATCTTGCAAAATGAAATGATATTTTCTTGAGTATAAAAATCTTGTGGTTCTTCTCCA
2180	CaSNP4204	GTTTAAAAGTTTCATTAGTACCCTTCTATGTGGTTCAGAATTAAATAACAAGGAGAACAC[A/C]TATATCTTGGAAGAGGTTTGGTTTAGCTTATCCACCACCTTATATTCACTTTGCTTGATA
2181	CaSNP4205	TAACATCACTACCATAAATCACACATCATCATCATTCCATAAACTCATCACAAATTTATA[A/G]CATCAATATCATCAATCATACCTCATCACATAAACTTATTACAAATTTATAAAATTACAA
2182	CaSNP4207	AAAAAGTAATTATGGTAATGTTTTTTCTTAGCGCAACCACATACACAAAATATGGATTAA[T/C]CCTACAAAATGTCACGCCTAACTAGATTGTCTTTAGTGGCCAATCTCTTGTTCATAAACT
2183	CaSNP4208	CACCAAGACTTTATTCTCAAATTTACGATTTAATTTTTCCTTGAGTTTACAAATCTCTTC[A/G]TTGCTAGAACTTACCATAAAGATATCGTCCACATATAAAGTAGATAGAGAATGACTTTC
2184	CaSNP4209	GAAGAGATTAACTGTGAGTCTTTTCAAATGAAGTTTTTGGACACATACTTTTTAGTGAGT[A/G]CTAGAACAAAGTTAGGAGATGATCTTATGAAGGTCCGCCAAGGTAGTATGACTGTGGGAG
2185	CaSNP4210	TATCCATAACGAATCTCAACACTCATCAAAAGAAACAACACAAGATGCATTATAAAGGCA[A/G]AACTTTCATGATGAAGACAAAGCAACAACATTTTGCATACTTTTTCTTTAAAAAAGTTAA
2186	CaSNP4211	GCCTTCTAGACCTCCTTTTAGTGTCGTTTTCAACCAGATTGTAGATCGGGCCATTTCTCG[T/C]TTCGCTTTGGGGGTTCACTTTTTGATCAGAGCACTTTTGTTTGTGGGTCATTTGAAGCTC

2187	CaSNP4212	GGTTGCATTATCTCTGTACTTCACACTTTAGACTGGAAAGTTGAAGTACTGAGTATATAA[T/G]ATATTGGATCTTCTATGTAGTTATCAGTTGCTAATTATAGATAGAAATACCAAGGTTAGT
2188	CaSNP4214	CCCTTGGATATCAATGTCTCCTTCTTTCACTAAGTACCTTAAATATAAAAAGGAAGTAAG[A/G]AAAACAACATTGAAAGGCTTGTGCCTCATTCTGTTTGAATCTTCTAGATTAGATGGAAAT
2189	CaSNP4215	AAAAAACTACACTCCTACTTCTTGCCCCTTCTGCTCTGGGTACTTCACCATTTCCTGGGT[T/C]GTTATCGCCTTTTCCCGGTTCCTAAATTAGGGTTATGATTCCAAATATAATATTAGTACA
2190	CaSNP4216	AGTTAATATGAACATGGTACGTAAGACCTTATAACAAAATATTTATATTCGACATGTGCA[T/C]TTTTTTTCTTTCTATTCTCATACGAGTTTAATGGATGTATGTATATTGTCATTATAAATT
2191	CaSNP4217	TAACTAGATATGATAACTCTTACCATCCACTAAACGTAATTAAACACAATTTTCAATTTC[A/C]TTAATCATAATTTTCAGGTGATCAAATATACATGATATTCACTAACAATTCAAATTATGT
2192	CaSNP4218	CACTTGTACTCTTCCCGTAGCAACTTCCCTCAGCCATTACACTCATTTAGTGAAATTAAA[A/G]AGCACAAATGTATAATTATTATACTCATTTATGAAATTAAGGTACACAGGTGTATAATTG
2193	CaSNP4219	TATCTTCATCAAGAATAAACGAGGATCAATCTGAGTCAGAATTATTCCAGAATTCGAAGG[T/C]TCAAAGATATACTTCATATAAAGTTTAAAGCAATATCATTCTTCATGTGAGGCAAATGCA
2194	CaSNP4220	TCAACGAGAGAAACACTTGTTCAGGTTATAAATATTTTCTAAGTGTTCTTTTCTTAGAGT[C/G]TGAGAGTTATTATTATTTTCAGCTTTGTTAGAAGCAACTATTTAGGTTCCAATCTTTTGT
2195	CaSNP4221	CTCATGACTTTGGTACCTTATTACCCAACCATCACGTAAGGACCATGGCTCCGCTCTTTG[A/T]CTCTCCACAAAAAACCAGCTCTTCCATTTTTAGAATAAGTCAGTTTTTAATCTTTCCAAA
2196	CaSNP4222	TAAAGATAAGAACGTGGAAAGGACAACACAAACCAAGACTAAAGAGACAACATAGACTCA[T/C]AATAGGACATATAGGAAACCACAAGCGCGCAAAATGAAAATATATATGGTAGGACAAACA
2197	CaSNP4223	TTGATTTTAAGAAATATGGCCCTCCCTTATTTTGATGATAATAAAATCGTTTATGGAAAA[A/G]TTTAATGCTCTAACGTTGTACTTATGTGCGCAGGTTCGAGATCAAATATTTAGTTAATAG
2198	CaSNP4224	GACCCGATTAAGAATTGTAGCTCGCTACCATAAAATTCAGTTTCAATCGGGGAATAATAG[C/G]ACTGAGCTGTTCCACCAAGTTGTCATAACAAAGGATCAATTTGTCCTTCCAGAATCTTGT
2199	CaSNP4225	GCAGGTTGTATCAGCCTGAGGTTCTTCTTGATTATGGAGATTAATAAAATTGAGATAATT[T/C]ATTAATTCTTTCATAACTTTTAGACTTGGAGGATAATCATCAATGTTTTCCCATAGATCC
2200	CaSNP4226	GCGCAGGATTTACGCTTCGGGCGCGCCTGGACAATGCTTAAAGGCTCGAAATGCGCCTCG[T/G]GCGCAACTGGCGCTCTTCAGGCGCACAACATCTGTTGTCTGGAGTATACTGTATTGTACG
2201	CaSNP4227	TTCATCAACAATAAACACAAACCAGTGTTGTTAAATAGCAGCACTATAGTGTAGAGGAAT[A/T]TAAACAAATCACAATTTAGTACACTATTTAGTACAAATTTTTATTGCATAGCGAAATTTG
2202	CaSNP4228	TAGAGTATAATACAAAATGATCACATTCACAACCATGTAATTTCACACACAAAGATAACC[T/C]TTTTTCTTCATTCTTCTCCAATATATTCCTTAACACCAACAACAATTCATCAATTATTCA
2203	CaSNP4229	AAGTATCTTGATCTTAACATAGAACATCTTAAATTGTCCTAAGGATTATTTTTTATATTT[C/G]ATGATTTGTTCCTTTATTTCATTAAGATTAGGAGTCTAGTGATTATCATGATTAGTATTT
2204	CaSNP4230	TATTCAATTCCCAATGTATGGTCTCCTACCTGGTTATACTCCAACCTTAGTCATCAACCG[T/C]ATGAGCAATAACCTAAACGAATCAAATATCAACTTCCAAAGCCTAAATCAACCACAAAAA
2205	CaSNP4232	GCAAGTTCCTGTTTAAGATGGTTGCTTTCAGTCAAAATTATTTCACCAATAATAACAACG[C/G]ATAAGTGTATGATAGTAGCCAAAAGAAGAAAGACTTCTCTACATGGAACATTATAAAGAA
2206	CaSNP4233	CTAGACAACACTCTGATGAAGTCAACACTCTAATGAAGTCGACATTCGGATAAAGTCAAC[A/C]TTTAGATATCATTTGCTCATCCTAATAGACTTACCATTTGACTGAATAAGCACATGACTT
2207	CaSNP4234	ATGGGTATAGCTCATAGTTTAGTTAGTGATAAGTGCATTGAGAGTGGGGTTGCGGGAGGC[A/G]AAGTCCCTACAATACGATTCAAGTGTAATCTTGTAAGTTGGACCGTAGTATTTAAATACA
2208	CaSNP4235	AAATATGAGGACACGGAAGTTTCTTTTAAAATTTATAATTAAATTGCAAATAAAGGATTG[A/G]AAATTGAAGATGGATGACCTTGATTTAGTCATATTTTTTTAATGGAAGGAAAAATACTAA
2209	CaSNP4236	AATGTTGGTGAATTACGAGGCTGAAGGACCCACTATACAAGGTAAAGGTCTTCGGAAAAT[T/C]ACTCCTTTTTTCCGTTACCTATTTATCTTTTTATGTGCAAGGGTTAACAACTCTAATTGA
2210	CaSNP4237	GAAACAATGTAATTATCCTTGGTATGGCTGCCATAACTTCTTTACCACATTTACACTTAA[A/T]TGGTTCATTAAACCTTGTCAGTTTTCATGTTGCATTTGTCACATGAGTAATAGAGTCATT
2211	CaSNP4238	GTGCAACAAATTGAGTCCAAGATGGAGCCATATTTGGGTGATGATGAAAAAAGGGTGAGG[T/C]AGTTGCATATTGATGATGATTTGGAATGGGTGAGTATGGATACAATTGATGAGCACCAAG
2212	CaSNP4240	GTTTTGTTTCCTCTTAATCCACTTGTTTCTTCTGACGTACCATTCAACACAGCGAAAGAG[C/G]CATGTTGTTCCTTTCCGTTGAGTTTTTTGCTTCTTCTGGTCATTTTCTTTGGTAATATCC
2213	CaSNP4241	AAAGAAAAAAGGTAATTCAAATGGAATCAAAGAGCTTAGTAGAAGTTATTTGAGAAAAGC[A/G]ATTAGTTCATAAATAGCATACAAAAACAAAAATTACATAGATTTAAGAAGGTTTATCTAG
2214	CaSNP4242	CCTCTATTGTTTCCCTTTCTCCAGATCTGGATAGTCTGACTTGATATGTCCAGATTCATT[T/G]CATCCATAACAGATAATAATTTTCTTGTTAAAATATTCATTATTTCCTCCATTGGACTGC
2215	CaSNP4243	ATTTTATTATTTGTATTTTAATTTTATTATTTGTATTTTGACTCTTTCTTGCTCTGTAGG[T/C]CATAACTTGAGCTCTGGATATCGGATTGGCGCATATGAGCCTGCATTGGAAAGCTAAAAA
2216	CaSNP4244	ATTGGATAATGTCCCACCGTTAGACAAATGTTCTTCAGAGTTTCATGTTCAGTACCCCAC[T/C]GTTGAGTATTTTTCTCAAACATGTTGCCTTTGCAATAAACTACCGTCACATACTTGTGAC

2217	CaSNP4245	ACTTTATTGTAGCTATCACTCAAATATAGGTGTTGCCGCCATTAAATTAGTGATCTTTTA[A/G]AAATATTGGAATATTATTGTATACTATTACCTCTACTCTTATATGTAAGATTTATTTCTA
2218	CaSNP4246	TTGCTTAGAAGTATAATCCTTAATATGCTGAGCATGTCCAGGTTCCTATGGGAAATCTTT[T/C]GTTTCTTACTTTAACACCTATATGCTTTGCTAGTGAAGCATATTATTCCCACGAGATAGA
2219	CaSNP4247	AGCAGGCAGTAAGAAACGCTAAATTAGTTCCTTACAAATGCAAGTTAACTTTTTTTAAGC[A/C]AAAATGAGGCATGCAAAAGCATCAGTATAAAAACTAAGATCGTAGTAAGGTTGACACCGC
2220	CaSNP4248	GGAGAAAGACGATGATAACCCAAACCAATATAATGAGGAGATGCGTTAAAGGTTGTACGA[A/G]TATCCTTACGAAGAGCAATGAGAATGTCATGCTTAGGTGGCAGAGTCGAATCCGGAGGTG
2221	CaSNP4249	TGAAGAGGAGTTGGTGTGAAGGTATCCTCAGGGAGAGGATCGGCTTCATGAAGGGAAGTC[A/G]GATTGTGGATTGGTGAAGACTGATGAGATGGTTGGACAAGAGAGTTAGGAGAAGGAGAGT
2222	CaSNP4250	TGATATATTTATGGTTGTCAACTGTGTTATCTTCATTTGATTAGTTCAACCTTAACTAAC[A/C]ATCAAGTTCAAACTGCAAAATACATAAACATATATATATAATTTCAGTCATTCACCACAA
2223	CaSNP4251	GGAGTAAAAGAGGCCGAGGAAGAAAACATAGCAGCAAAAGTAGTAAATCTGATGTGTAAT[A/G]CAAGTGAGTGGCTGCTGGAAAACCAAGAACCCGAATTGGAGACAGCAACGAAAACAAGAA
2224	CaSNP4252	TAACAACTCCATTTTGTTGTGGAGTTCTTGCACATGAGAGATTATGAGAAATGCCAAGTT[T/C]ATCAAAGAATATTTTAAAAGAAGCATTTATGAATTCTCCTCCATGATCACTTCTTATAGA
2225	CaSNP4253	CCTAATTGATAATGACAATGTAGGCCACAAAAGAGACCCAAAAGAGCATACTTAAAAGCA[A/C]AATGAAATAAATAATCGAAGTGGTTGGGGTAAAGTGTTTAATGAACTTCAGTTAATCATA
2226	CaSNP4254	TCCTTCATTTCATATGGGTTGTTAATATTTTTGGAAATTTTGTGAAGGTGGAGATAGTCA[A/G]ATGGATGCATTGATATGGTATAGTTGGCTTGGTGGAGTTATCATTGGGACAATGATAGGT
2227	CaSNP4255	CAAAAGAGATATTAAGAATGTATTTTTAATTTTAATACATTTAAAAGTTACAGTGACGAT[A/G]TCTTACATATTTAAGAAGCAAAATGATTTTTTAATTATTATTTTCAGACTTAAATATTTA
2228	CaSNP4256	AGGGTACAAATATAAATATTGATATTTTTTTGAACGATAGGTATGGGAACAGGTAGTATT[A/G]TACTCTACCCAAACACTATTTATTATCATCCCTAGTTATTAGTAATAATTGTAATAGTAA
2229	CaSNP4257	CCTTGAGGGCTGGTCAAGTCTTTAGAAGATCATTGGTGTTCCCAATAGTTAGAGAATCTG[A/G]ATAACTTGTACTTCCATCCAATTTTAGCTTTTGGAGGATCTTACTTGTTTTAATGTTGAA
2230	CaSNP4258	TTTAATATAATATTTCATAAGAATAAAGGGGAAGATAGAGAAGCTTCATTCTACAAAATC[T/G]GAGAAGGAAGAACAAATTTAATGTGCCTTTACATAGAAACTAAGAAGCAACTATGGAGGT
2231	CaSNP4259	AATTACCATAGACTTATAGATTGATCAAAATTTCATAATTAAATTTACAAACATGCAAAA[T/C]GGTTGGAGTCTATAAAGGTTGTAATGGTGCCAGATAGATTTTGCTAGATGGAATAAAGAA
2232	CaSNP4260	TTACAAAAGCCATGATGTAGTGTTCATGGAAGACCAAACTGTTGAATACATTGATAATGT[A/G]GATAAGACTACACCTGAGAGAGACTATTATGTGTGACCAAGGATACATAAAGACTACTTC
2233	CaSNP4261	AAACAAAGTTTAGTTTACCTCAAATTTTATGCCACTGCTCCTTGCTTTTATGACATTTTG[T/C]GTGATGGTTGCACCACGTTTGACTTCATTTTCGTAAGTCTTAGTGTTGACAACTTCCTCA
2234	CaSNP4262	ATATTGATTTTGGTCATCTAGCGATGGCTTTATTTTCCGACGAGGAAAAAATTCGAGACAT[A/G]AAATTAATATTGGTCGTGTGGCGAGGCACCAACTCTTCTAGAGAAATTTGCATAATTGCA
2235	CaSNP4263	TTGAATTAAAACCTTCGTTGCTATCTATGGTACAACAAAACCAATTATTTGGTGCTCCAA[T/C]AGATGATCCATTTGTCTATTTTGAAAAATTGAATGTAAATTATGTCATACCAAATGTTTC
2236	CaSNP4264	AGATTTATTATTCTAATTATAATTTATGATATATTGAACTTCATTTTTCTTAATGTATCA[T/C]TATTCACTTTTAATTTCACAAACATGTCAAATTTTCTGTCATAGAAGGGACACAGATTCA
2237	CaSNP4265	GTTAAAATTTGGGCTTCAATTTAGTCTTTGTTTAATTATGAATTGTATTTTATTCAGATT[A/G]CGTTTGTAAATTCTTCAAATTAATTATGTTCCTTTTTTGTTTAGATTGAGATTGTAAATT
2238	CaSNP4266	TCTTTTTCTTTTTTCTCCTCAACACAAGTACTTACTTTTTGAACATTGTGATTTTTGTTG[T/C]TCATGGTTGTTTCATTTTCTTCCTCTTCTTCTTTGTTTGTTATTAATTTTATTTTATGAT
2239	CaSNP4267	TGAAACACATACACTGACACTGTAATAATTTGAGAAAAATGAGTTAATTGAATGTAATTA[A/T]TGTGTTGGTGTCAGACACCGGAATATGGCTTCAACTAGAAGTGTCTGTGCTACAAAACTA
2240	CaSNP4268	TGTAGCGGTGTCAGGTGGTTTTTACCATTAACCTTGCGGTGGTCCTTTTCTCGTACCCCC[A/C]CTCATCATCTCTTAAAAACAAAAATAACCGCACACGTGGCAATATCATAAATGCCTCTCA
2241	CaSNP4269	CTTGTTAGGAACCCAAGAATTGGACTCCTAAGAAAGAACACTTCGATTAAGAAATAGGAG[A/T]AAACTCTCTCCTCCAAGGGTTTCCCCTTAACAATAGAGAGTATTCTCTATTCAACAATTG
2242	CaSNP4270	ACAAAAGGAACATTATAATCATCACTAGCAAATGCAAGAACATTAGAATTAGTGGTGGAT[C/G]ACAATGAATTGTCAATGGATGCAGGCATATTGTGATGAAAACTAGATGCATGATGGTTGT
2243	CaSNP4271	TCTTAGAATGCATCATAAGTATTAATTTTCTCGAATTAGAGATTTAGATTGAGTTGAGCT[T/G]CGTGAGTAAGTGTTCAAAGATTTATGCAACATGCAAGGATAATTTATCACTTGATATGAT
2244	CaSNP4272	AGGGGAACATTATGTGGAGACGGATAAATTCTTGATTTGTTGGCTGTTCAACATTGGTTT[T/G]GATTGTAGAGGTGCGGGTAAATCTTTTTCTTTTAGTGATAGATGGAGTGAATGTAGCATT
2245	CaSNP4273	TTTCATATTTTTGATTAACCAGCTATTTTGTGTAGTTTTGTATTGTTTCATTGGTTTAGC[A/G]CAATGTTGAATTTTGGTGAAAAAGCAACATAACCCTTGTGCAATATTTTCATCATGTTTG
2246	CaSNP4274	CCAATGCAGAAAAGTTGCAGGTCTCTCACACCTTTGTTACTTGAAAAGATACCCGTCATC[A/G]AAAAATCAATTGTGGAGATGGTGGGGGAAGAGAGATGACATTAAGGTAGGGGAATATTTT

2247	CaSNP4275	AATTAAACTCAGAAACATCCATCATTAGATATATATATTACGTTAAGTGGAGCATCTAAA[T/C]TATCCCTCTATGTCATGGTATTAAAACATTAGACTGAGAGTGGTATCTTTATTTTCGTTC
2248	CaSNP4276	TTTTTTACGATGAATGTAAATGCTGTTTCCATTAAACCACCTTCGCAGCCTTGGTTACCA[T/G]TTTTAACATCACAATCTACCAGTTCTTGTTCAGACAGAGACACCAACTCCCCTGTTTTTA
2249	CaSNP4277	TTATTAATTATATTTTAGTGATATATTATATTATATGTGTTTGATATGGTGCGATAATTT[A/G]CTACATAGTTACTTTTGCGTACTTAGTAAATTATTTAAATTATTAATTTAAATATTTTGT
2250	CaSNP4278	TCAAGAATAACATATTCACACATTTTGACGTGCCTCGAACTTTGATTAGTGACGAGGGCA[A/C]TCACTTTCTAAACCGAAAAATGGAATACCTTATAAGGAAATACAACGCGTGTCATACGGT
2251	CaSNP4279	CAACTCTACTTGGCTTCTCTTTCACTCAGAGTCAACATGACTCCTTTTTATTTATTCATC[A/G]CACATCTACAAGCATTGTTCTACTTCTCCTTTATGTTGATGATATGGTCATTACTGGATC
2252	CaSNP4282	CTTTAGCACACATTCATGAGTTAATTTTACATCACCAAAATCAATAAGAGTATAAGAATA[A/G]AAAATATCTCTTCTTGATGTAACATGCACTATCAATCACCCAATTGGATGCACTATCAAC
2253	CaSNP4283	TACCGTTGCAGTTCCCACATGATTGCCCCAATAAGTTACAATAGTATTCAACAATAACAT[T/C]CAAGCTTGTTTTCCTTCATTCAACCAAAATTCTCATTCTTTTACAACATCATATTTATGA
2254	CaSNP4284	GACATAACCAATGTCTGCTTATGGCAAATGAGTGCCATCAGAAGTCAAAAGCGACACAGG[T/C]GGGGCATGATTCACAAATACAAAAGATTTATCATCATATGATATATGACGCGATGCTCCC
2255	CaSNP4286	ATGAAGAGCTATCATTCATTTAAAAGAAAATCCAACACATGTGAAACAACATGAAGATAT[A/C]ATTTCTCAAAAAGGATTTCAGATGATCCACAAATAGCATAAAAAGAAAAGAAAAAATAGA
2256	CaSNP4287	AATTGTTATTGTGAACTTTCAAAAACTTAAATTGTTGGCTCCATTTTCATGTTACGATAA[T/C]GATTGATACATAGTTGTAAACTATGGTTGTAATTATGCTGCAAAGCTTAATATTGTAGCA
2257	CaSNP4288	CGAGAAGAATCAAGTATATGAGTGGGTTTAGAATTTTCCATTGGAACCACTACCATTTGA[A/G]TACGTTTGCATTCTCCCAAACAATGATAATTTTCAAATAAAAATCCCAAGTGGTTCCTCC
2258	CaSNP4289	CTTCAATTCACTACCAATCAATCTGAATCCACTCCAATTGATAATGCCTTTGCTTTTGGT[T/G]CCCCAATCCTACACTCGTTACATAATTCTATCCTTGCATTTCCTAATATGGACTTCTCTG
2259	CaSNP4290	TCTGAAAATATATGTAAGAGTTGAGTTGTTAAAGAATATAAAAAATAAAATAAAAATAAT[T/G]GGAAAGAATCGTATTGTCCCGATTCACATAAAACAAAATCAATAGTTTTGTTTTGTAACA
2260	CaSNP4291	TTCAAACTTTCAGCATGATTATCATTCTTGATCTCATGTCTTTTATAGCTTTCTTTTGTT[T/C]GGATGGAGGCATGCACTTCTTCAAGAGTAATTGTTTGTTCTCTTCCAAACAAGACATCAT
2261	CaSNP4292	GTAATAAAAGCAATGATTCCTGAGCACCATGAAGTTAGCTTTTAATTTCACGATCTATTA[A/T]ATGCTCCAAAACAAATAGTTTCAGGGGTTGGTTGGTCTGCTTTAGAGCAAAAATATAAGA
2262	CaSNP4293	CTAAAAGTTGATTGAGGATTTATGTTCTTATAATCATTGCAATCAAAACCTCTAACAATT[A/G]CATGAGTTTATTGAAAAGATTTGAAGAGTTTTGGGTTTCAATTTTTGTTGGTTTGAAATT
2263	CaSNP4294	TTTTGTTTATTGATCATATTTCATCTGCTCATAGTGCAGAAAGGGCCTCCACCAAAACCA[T/C]CAGACATAGCTCTACGAGATAGTTTTCTTTTCTTACTCTTATTTTATACATGTTATTTAT
2264	CaSNP4295	TTCCGCTGTCGAGTTCCCAACAATTAGGGAATATAATTATAGTTTCTCTTTTCTAACATT[T/C]CAATATTCGGTGAGATTTTTTCGGTGAACCGTGTCCCATTTCCGGTTTACCCCTTCTTTG
2265	CaSNP4296	TCGTATTAAAATTGACGTAATCTTCTTAATAGCATATATTTTCATGAGAATCAAGTCACA[T/C]TGATCTCTCTACCTTCATTCCTTCCTTCACGCGGAAATGACCCGTATCATTACCCGTAGC
2266	CaSNP4297	GCAACTAATTTGTGGAAAAAAAATATTACAGTGAAGAAGCACGACAATGAAAGTTTATTTA[T/C]AATTGTCGCATGCAAGATTGGTCGCAGATATTTCCACGCACTAGAAATGAACCTATTGTA
2267	CaSNP4298	GGTACCCACTTTGTTTTGGGTCCTTGAGAGTTAGTTTTTTCTTTAAAGTGGTATATCATT[T/C]CTTTTTTAGAAACAGCTGTGTTGTTTGGTTTTGAAATGGGTTTAACAGTGTCAGATTTAG
2268	CaSNP4299	GTCTGAAAGCGAGAGTCCAAGGGAAGAGAGGGAGAACAAGCAGATAACTAAGAGAAGATA[C/G]GGAGAGAGGTAGATAAGAGATATGTGATGTTGAAAAGAGAGGGATAGAGCAGAGAGGAGA
2269	CaSNP4300	GTACCAAGTACTATATGCTGCTTGTTATCCAATTCAGATGAAACATGGCTTTGGCATAAA[A/C]GAATAGCTCACATCCATATAGAACATTTGAATAAACTTGTTAGCAAACAGTTAGTCTTAG
2270	CaSNP4301	AATATATGATTGAATATATGATCGGTTAGTGATATGTTGAAATCAGTTAGTTCAATAATC[T/C]CATTATAAATAGAAGCATGTATAATTCATTCCCTATCTCTTTTAATCAATCAAAATGTTC
2271	CaSNP4302	AGCATAGCTTGAGTTAAACCATCCTCCTTAGATTCTAGTTAGTATGCAGAGGTCTTAAAG[T/C]AAATTGTAAAGTAAATAAATTTTTGTTCCACAATATTGATTTGCTTATTAGTTATACAGT
2272	CaSNP4303	TGCCAACTATTTAGCACCCACACTTCAAATTTGATTGGTTGATGTTATCATGCTTCTTTT[T/C]CTGTCAGCTTTGGGAGTCTCCGGTGGCCCTGACAGTATGGCACTCTGTGTGTTAACTGCT
2273	CaSNP4304	GTCTAAAGGACCTTAATTCGTAAGGTAGGTGCAAGGCTGTTAGTAGAATCAACAGTAATA[T/C]ATGTGTGGTTATCAAAATGTGAATTAGGAGGTATATTAGAAACATGACCAATATCATCTA
2274	CaSNP4305	TTTCCATCCAAATCTCACTTATGGTTTATCTTGATTTCTTTTGACTTGATCAGATTCATC[A/C]TCTGATGATTTGTCATCTTCTTCTAATTTACTTCTATCCTTTTATTTTTCAGATTCTTTT
2275	CaSNP4306	CCGAATACCTCCAAGGTCTAACAGTCTTGCCTTAAGGCTCAACATAGACTGCCACGATCA[A/G]GCTCATTCAATTGTATATGATGACAAAATAATATTCACTTCACAAATTCTCAATATTTAT
2276	CaSNP4307	GTTTTCTTCTTAGTTGAAGTGAATATAGAGATAAAGTTATGTCATAAAGAGAAGCATAGT[A/G]ACTATGTGAAGGTTGTAATGTACACCGGAGGTTTATGTTTCTCACATATATATACATGCA

2277	CaSNP4308	TTTGAAAATCGTGTACAAACTAAATGCAAATGAACAAACTAAAATGATTCCCTTAGTAGA[T/C]GCAGTTGATGAACATGAATACATGTATGAGCATGAATGCAATGATGTCGCAAATAGTAAT
2278	CaSNP4309	AGCATGCAAAAGTACTATTTCCAAAAGCACTCTGAGTCTTAAGAGGCAGATGCATAAACA[T/C]GCTATCAGACCCAATCTTCATACCCAGACTTCACACGCAAACATTAGACCGAGACATTAG
2279	CaSNP4310	CCCCAAAACACTACTGTTCACCCAACATGCGCCGCTCCACATCTTTACACTAATTATCAA[A/G]AAGGGAATGCACAAAAATTTCCACCTTATGGTCTTCTGCCAAGTTAAACCCCACCCATAG
2280	CaSNP4311	TTTATTATTTATTATTAGCAAAGAGTTTGTATTTTGTTATGTCAAATACACCCTTAGATC[T/C]TCCATGACCAACATTTACTCTTGTTTTTTTAGACAAATGTTAGTAGAATATGTTAGTTTC
2281	CaSNP4312	TCTGTCCCGTTCGTATTGAATCTACACAATGTGTTTCACATATCGCAACTCAAAAAGTAC[A/G]TACATGATCCGAGTCATGTGGTGCAATCAAATACTGTTCAACTAAAGGATAATCTTGCTT
2282	CaSNP4313	CTTGAAGACTCTTATTATATCCATTATGTTAGCGTAAATGTTGAACGTCTTTTATTTTGG[A/G]AATTCATGCTTTGATAGTGACACCGGGTTTGAACTATATTTGCAATTATTACTAAATTTA
2283	CaSNP4314	TCCAATGGAAACTGGAAGCATTACTGACTTCCTTCGGGATAAGAATATTTTGGTCCTCGG[T/C]GCCACTGGCTTCCTTGCAAAAAGTATATATATCTCTATATGTTTAATTACTTATTAGTAT
2284	CaSNP4315	CATTCAAAGTTGGAGTAGTTCGACCGACCACCTCTTTGACCACGTCCTCTGCCACACCCA[T/G]TTTGCTGGCTTGACTGTCCTCTCTCGAAGTTGTTGCCTCGACCACCTCGACCATGACCTC
2285	CaSNP4316	AAATGGTTTAAAATTTCAAAGTATTTATGTATGAACCAAACATAACCCAATTCAAATTTG[A/G]AACACAAACACAAGTTGAATCTCTCTTTTACTCTTAACTAAGAAACAGTTCTTACATCAC
2286	CaSNP4317	CTTGGGTTTGAAGGAAATCTTAGTTTTCTTACCCTTTTGACAAACATCACATAATTTGTC[C/G]TTAATGAATTTCATCTTTGGTAATCCTACAACAAGTTCATGCTTGACTAACTTGTTTAAA
2287	CaSNP4318	CTTCAGCACTCATAAAATAATACAAAGTTTTTCACGGGTTGTAGTATATGACGAGTTTCT[T/C]GTATCTCTTTGTAGTAAAGATCACAAGTCTTTTTATAGAGAAATTTCAAGACCCCACTTT
2288	CaSNP4319	CTCTTGGTCGAGTTGAGGAAGGTAAAAGGCAAAAGACAAAACCATGGTTGGTGAAGGGAA[T/G]AAAACATAAGAAGCGATATTGAATTCGGCGGCGACGTCGAAAGCATCGGTGCCGAAGAGA
2289	CaSNP4320	TATATCCCGACGTAACTCAGCTCGCAAATAAATTTAGCCTTCAGTTAAAACTCGTAAAGT[A/T]TTTATCAATAGTTATTATTACGACATAAATAATTTGATTATTAAAATAATTAAATTTAAT
2290	CaSNP4321	TTATTTTATATTTCCAATGTTTTTATATTTCAGCTTTAATTCTACTATGATCATATGTGT[A/G]TAGATCTTTCTTGTGTTGAGATTGTAGGATTTTCAATGTTGGATCTATAACATTTTCTAT
2291	CaSNP4322	TATATATATATCTGAAGTATTGCCAACATTATTCAATTCAATAAATATATTTTACTCTAA[T/C]TCTTGAGACTCTCCATAAACTTTTAAGGTTTCTCTCTATGCACGTGCAACCTAAGAGGCA
2292	CaSNP4323	GATAAATATGGGCAAAAATGCAAAAGAGCAATAATTTTATTGAACAATGGTAAAAAATTT[C/G]TAAGCATAAAGAATCAATGCGATTTCGGTGAGGACATATTTATGAATTAATTGATGATCT
2293	CaSNP4324	AAATTAATTAATTTTTTTGCAAAGGTAGTAGTTTAAAAAGAGTGTTCTTGTACTGTAAAG[T/C]TTAATTCTAATATTAAATCCACTAAGGAATTAGGGGTTACTTTGTAATTAAATGTTATGT
2294	CaSNP4325	TCAAGTGTGGCTTTGATCCACACCAATATGGATTGAGAAAACACTTTTACTACTTGCACG[A/C]ATTGATGCTTCAAAAGTGTTACATCTAAATGAAGACCGGCTAGGTTGAAAATCCAAAAGG
2295	CaSNP4326	CTAACTCATGATTTTGGGCATCCTACCTCTGATGATTAAGTTACGTTTTTGGGCATGTTG[T/C]CTATGATGCTATTTTTCAACTATGAGTATTTCTGCCTCTTGTTTCTCCTTCAATTTTTTT
2296	CaSNP4327	TTAAACCAACTTGATTTTAACATGATGAATATTGAATGTCGTGGCAAGCAAATCCAAATG[T/C]ATCCAACTATTGCTTATTTGTCAACATGTATTGTAATGTTACCAAACTGATCTTGGGTCT
2297	CaSNP4328	CAGTTACCTTTAAGCTGCACTCATCTTTCTTTTATTTGTCTTTGTTTCACACACTGAACA[T/C]GATGAGTCATGCTAGTGATTTTTGGCAGGCAAAAACTGTAACCCAATGATGAAACACTTG
2298	CaSNP4329	TATTCCAACCTTCTCTTTTAGATTTACCATCAGACTGCATCTTATCCACAACCTGTATGC[T/C]CAATGATCAGATTAATAACAAAATTAGTCACCATCAACAATCTACGGCGTAAAAAAGCAA
2299	CaSNP4330	AAACATTGAACTAATTCCTTGCTTCATGATGTCAGCACCAGCCTTAGAATACGATCAACA[T/C]TTCCCTGCTTTGGAAAGAAAAATCGATCCTATCACAAATAGGTCAGCCAAACCTTTCATA
2300	CaSNP4331	TGATTTTATTAGATGAAAGCCTTGTATGTCCAAGAAGATGCAATGGCAATAAATGCAGTT[A/C]AAAAATGAAGAAAAAAATCACCAACTGATATAATATATTGTTGTTAGATTTGAAGTAGGTT
2301	CaSNP4332	TTTGGAAATAGCAGCAACAAGAACTTGCAACATTGGAGTCAAGGGGGACCCATTTGGTAT[A/C]CTATATCTATAAAACTTACTTCCCATCACAAAGATAAGCAAAGAGATACTCATGACACAT
2302	CaSNP4333	TGGTGTGGTGTTGAATCGTATTGGGGTGTGGGAAGACATTATGATTTCTTATAACTGCAA[A/G]CAATGAAGAAGTATTGAAGCATTTCAGCAACAAAAAAACAATGTAGCAGTGCACTCTCTA
2303	CaSNP4334	TAAAATTGAATCACTTATTGAAGAGGATTTACACAATCCTTTAAGCCAACTAACATTACA[A/T]ACTAGAGCTTCAAATGTTTGAGGTGTGAGACAACTACGGTATTCGTCAATCACTCGTCCT
2304	CaSNP4335	TGCTTTTCTTCTTTCAAATAATGCAGGGCACTGATGCTATCTGGGAACTGCATTCTACCA[A/T]ACTTTGATCCATATGCAAAATGCTATTGAGTTATCAACTTGGAAGAACTTCACTCAACGA
2305	CaSNP4336	CACGTAAATATGGATAATATCTCCCATATTTATTTTTGTATTTATTGCCTTGAGCCTATA[A/T]AAAACAGACTCCGTTGTGTAGTTCAGACACACGGTTTCACCATATGTCTCTCATTTTCTC
2306	CaSNP4337	TTCCTTGAATAATGCATCATATAAATCAGATTTAGACTTAATATCTGTATACAACTGTTG[A/G]CACACAATGCTCCAATATTCTTCACTATGACCAAGTAAAACAACAATAGCACGATATCCA

2307	CaSNP4338	TCTAATCCAAGTATCCTTTGATCATGTTGCCTCTGATTCCTGTATCCTCTACCTCTAATT[A/C]TAATCATTGTTCAACTTCCTCTACTATGATCATTGTTCATTTGCCTATGACTCTGATCAC
2308	CaSNP4339	GGTCTGTAAAACTCCTATTTGTAAGATTCGGGTATACAAGGTGATCTTACCTTGTATTTG[A/C]CAGAGGTTGATCCACTTGTTGAACTTATGACTTTTCAGTGACATGGTGACAATTTCAACT
2309	CaSNP4340	AATGATAAATCACACAAGCCAATACTAAACAAATATTTATATTACACGACTTCCATGAGT[T/G]ACAGGCACAAAAGCTAGCACCACACAAGCAGCAAGACTATTCAACCATGCCAGCTGGAGA
2310	CaSNP4341	ACCAAACTTAGATGACATGTTCAATACAAATAGACTTATCCATTTGAAATGTAAACGTAC[A/G]TACATATAGATTTAATATACATTTTAATCTAGTCATGCATATATATCTATTTTTGCAATT
2311	CaSNP4342	ACCATTATTAATTGCTCCAAAACGTCTATAAAAGAAAGGTCAATGCTCAGGGAAACAACA[T/G]AAGTTCAAGTCCTATCAAGTCTCACAAATCATTCACATTCACATACTTGAAATTCTCACT
2312	CaSNP4343	ACAATCAAAACCACAAAATAGACCTGAAACACTCAATACTAAGTTAATTTCATGATTTTT[T/G]ATGTCAAACACACACAATATCCATGGAAGTAGCAAGAGAACTAAAAAACTACAATTCAAC
2313	CaSNP4344	TCATTCTATTACAACCAATTCTTCATAGAGGCCAATTAAAAAACCTGAATCCTTGTTAAC[A/C]GTTTCATTCCGTGTTCTTCTTGATCATCATCTACTTCTAATTATTAGTTAACCTTCATTT
2314	CaSNP4346	AATTACGATGCAATTGTATCGTTTGAATGATAACCAAAACAATGATACCATGTTAGAAAC[T/C]ACAAAATTATAAAATTAAAGATGAATTTCTTATTAAATATAAACTTGAGAGCTTGACTAT
2315	CaSNP4347	ATTTTATCTGACTTGATAGAAAATTGTGTTGAAGTTTTCATGGATGATTTTACTGTGTAA[T/C]GTTCCTCATTTGATGAATGCTTGAACAGTTTAGATAGAGTTTTGCATAGATGTATTGAAA
2316	CaSNP4348	TCAAGGGTAGGTGTTTTTTCCTCCTTGTCCTTGTTATCGAGATTCACGATCCTAGTTTTT[T/C]AAATTATTTTTGTGTCATTCCTGTCTCACTTGAGTGCTGCTCTTTGCTATTATGCAGGAT
2317	CaSNP4349	AGAAATAAGATTGAGGTAGCGTTACTCTTCTTGCAAAAGACAAGCTACTAGCTTTCCAAT[A/T]AAAAAGTTTCTTTCGCATACGATTCAAAATAAAGGAGTAAGTGTCCTTATAAATTCTCTA
2318	CaSNP4350	AAATCCAAAAAGTACCATAGTACGAAAAAGTGTGTACTTGACAAGAAAGACAAGACATTA[T/C]CGCTCTTGGAAAAGGAAAAAGCCAAAAATACAAGTAATGTCCTAACTTATGGGAAAATCA
2319	CaSNP4351	TTGAGCAATTTGGAGAGATCTTAGAAGCTGTTGTTATCACAGATAAGTACACTGGGAGAT[A/C]CAAAGGTTATGGTTTTGTAAGTCTATTTTCCTTTTATTTATTTATTTATTTATTTATTTA
2320	CaSNP4352	TTAGTGGATTAAAGTCTTCTGAATAAAGGGACTGGATGTAGCCAAGTAAGTGATGAACCA[T/G]GATAAATATATATGTCAAATTAATTATGCTTTCATTGTTTATTGTTTCTAAATTCGACAA
2321	CaSNP4353	CTTCATGTGTTACTTCAAGTGTATCCCAAATTTCTTTAGCCATTTTACAATGTGACACAC[A/G]AAAGAATTCATCCATTCCAAGTGTTGATTGTAACATATTTTTCGCCTTCTTATCAAATTG
2322	CaSNP4354	ATAATTGAATGGTTGAAAAATATTAAAATAAATCTATACAACTGAAAATTCAATAATAAT[A/G]AAAAGCTTCAAATAGCTAATCCCAAAATAAAAATGATGATATAGACATAAGGTCTACTCA
2323	CaSNP4356	CAAAACAAAGTATAAAAAAATACATTTTTCCACTGTTAAAGTCATGCTATGAAGATTACG[T/C]AATGACTAACGATGAGTGTAAAACTAAAATTGTCTTACCATCCAATAAAGGACATAAAAT
2324	CaSNP4358	TTTTTTGATATTTATCATTCTTAGCTTGTAGTTCCTCATGAAGTTTCTTTAGCTAATAAA[T/G]TTGATTTTTAAGAAAACCACATTTATGAGATAGAGCGTTTGAGTCATTCAATAAGTTGTC
2325	CaSNP4359	TATAAAAATTTGCCGCGTAACTATGAACCTCAACCAGAATTTAAACCATGTCTTTTACTA[T/C]GCTCTTCATTCAATTGACATGAGAAACCTTTGGTTGATGTTATTACCAAGAAAACAGAGA
2326	CaSNP4360	AGGTCTTCTATTGAGAATGATTTAAATTTTATTAACACCAAGAGTTGGATTTTTCTTTAA[T/C]ACATTGTAAACCAGATAAATTCTTTATTTTTTTGCCGCTTGAACTTCAAACTTGTATTCA
2327	CaSNP4361	AGTTTTAGAAGCATATTGATGTTCATTACTCGGCCTTATTATACTATATTATATATCAAC[A/C]TAATTTAAACTTACGTCCATTGTACAGAACAAATAAACAGTTTGCTTTTTTCACATTTCG
2328	CaSNP4362	TATACTATTATATACTGCAATAGTGATTAGAGGAGTTTACTTTTTCCGCCTCCTTTATTT[A/C]AATAAACTTACTTACCGTCCAATAAACAGTGACTCTTGGGCGGTCGTCTCCGTAGTGAAA
2329	CaSNP4364	GTCATGATGTTTAAGACAACAAAACAAAATTCAGTCTCCTTGAATGTTCAAAGGCTTCTA[A/C]CATAGATGTAAGAAAGAACCAATAAGGATTTAGTAAACAACTTTTTTGTCTGGGAGATAG
2330	CaSNP4365	ATAAGTGTCTTTGTTTAACAAATAATTTAACAAATATTGTATTGGAGGAATATAATATGT[A/G]TTCGAGCAATAATAAGTGAAAAATATACATAAATTGTATATTTTTAAAAAAATATTCAAT
2331	CaSNP4367	AATATATGTTACTATCCGTCTAATTCTTATCAACACACTCTCAATGTACTTCTATTGAAT[T/C]TGGTTTATAGTAACTCACCATAATACATCTACATCATAAAAATATGACAACCTGGTTTGA
2332	CaSNP4369	TCGTCCTACACCGCTAGTTTTCGTTGTTTGTGATGTTGATGTTGAATGGGTGTATGAACA[T/G]AAACTTTCAAGACCTCTGCACTAGATTGTGTCGAGTCAGATATCTACCCCAGTGTGTTGA
2333	CaSNP4370	AAGAAAGTAGGATAGAAATAGCTTCTAACCAGACCATTAGAGCAAATGTCTTAGTGTAAT[A/C]AATGCCCTCTTGCTGATTGTATCCTTGTGTGACAAGTCTTGCGTTGTTTTTGACCATTTC
2334	CaSNP4371	TGAAAATCGTATTTGCGCTAGCGTAATCGACGTGTTCTTGCAAAGCTTATCGAAGTGGGG[A/T]AATATTTTTCTGTAATTCATCCACTTTGGCGTCTTATTTTTCATGTTTGTACATAAAGAG
2335	CaSNP4372	ATCGCAACCAAATCGACTTGTTTTTCATCCCATTTAGCCATCATAACCTTTTTCTTAGAC[A/G]TGCAAGATTTCATTTCGAACTTCCCTTTCTCATTATTGGGAAATTCTAACTTAATGTGAC
2336	CaSNP4373	AGATTCCTACAAATTCTAAAGGGAATAGTCAGAAGGTTCGGCCTCACAACGAGCTAGTAA[T/C]TTCACCAGAAAAGATTTGACATATACATCAACCGTGTACGAAAAGATTATCTTACAATAT

2337	CaSNP4374	TTTTAAATGCCAAAGTTTGTAGTAACCAAAATTGTAAGTAGCCACAACTAAAACTATTAC[T/C]CTGTTAGCAATCTAAACTGCATGTTATAGATATATGTTAGGATATATTAACATTTATGTT
2338	CaSNP4375	AAGTCTTGCCTTATTCCTGACCATTTCACTTTTTTCATTTAGCTTGTTTCTAAAAACCCA[T/C]TGGGTTGCAATAATGTTCTTCTTTGATTTAGGTACTAGATCCCAAACATCGTTCCTCTTA
2339	CaSNP4376	TTCCTCTCTTTTCGCTCTCTACGTCAGATCGAGTAATGATAACTGATACTCGATTTTGTC[A/G]GTTGACCTCTCTTGCCCATGATATGATAGCTTCTCGTGACTCAAAAATTTGATCAGTTGT
2340	CaSNP4377	CCTTAAATGATTTGATTAGGGACGCTAATCAGAGCATTGTCAAGGAATTCTACGCCAATC[A/C]TTTCTTCTTTCCAAACAAGAATTAGACGTATCAAACCCTAGTTATGGGAAATCATTGGAT
2341	CaSNP4378	TACCTATATAGTTTTGCATGTCGTTTTCTTGTTTCTTCATGGAAATAAGATTAGAGACAA[T/C]GGTGTATAAACGATTGACGTCATTGGAATATGTCTTCTTCTCCTTTGTCCAGACAACATA
2342	CaSNP4379	GAAGAAGATTTGTTGTTGTTGTTGGAAGACATTGATTCAAAATTGAATTGGCGTGTGCGT[C/G]TGCGTGTGTTCTAAAAGAGAAAGTAGAATGAATGTGGAAATGGACAAGAAGCACTAAGAT
2343	CaSNP4380	TTGACTGTAATTTCTTGAAAAAAAAATTCAATAGACACATGTTAGGTTATCATGTGTTAGC[C/G]TATGCAAAAAGAAAACTGGTAGTCATGCCAATTATGTTATCTATGAAATAATTTTTTTAG
2344	CaSNP4381	AAAATCTGAGTATATCCCTTGGTTACCAAATAAGACTTAAATCAATCAATCTTACCATCA[A/G]GGTCAACCTTCACCATATAAAGCCAACAACAACCTAGTAAAGACTTCCCATGAGGTAAAG
2345	CaSNP4382	TTCATTGTAGTATACTTCTACCTGTAATAATATTTTCATTTCCCTTGCTAGATTACATTA[T/C]TTCCACACTCGTGGACATGTAAATCCATATATTGTCAATAACTAAAGGGATGTTAAACAA
2346	CaSNP4383	AATTCCCACCATAAAATGTGAGAAAAAAATTAAAATGGAATAAAAGAGGGAGAATAATTT[C/G]AAAATGTATGCCTAACTCCAAGTAGTAAAGTCTACTATCCCAAAATGTCCTACCCTTACC
2347	CaSNP4384	CAAACGAACTCTAGATACCACTTTGTTGCGAAAAAGCAGAGATAATTATCTCAATAATGC[A/G]AAACATTTTTCCCTCACCTATATAGATAAACGACCGAATATAAAATACAATTCCATGGAG
2348	CaSNP4385	TTTATGTGCATATGTTTGATTACACAAGTTTATTTGATGTGATAATATAAGATGATGCAT[T/C]GTATGATATAGATTTATGTGATGATGTTGATGTTGATGTTTGATTGATTATAGTGATGTT
2349	CaSNP4386	TTATTTGGAGTTATTTTAGTGTTTTTTTCCAGCAAAAGGTAACTTGCCGCACACAGCTCA[A/G]TGGAATTCAGCATTACTGGATATAGAAGTTGGGTTCATTTCCATCACTTCCCCCTTTCTT
2350	CaSNP4387	AATGTTAATATTGGATTGGCATGGGAGAAGTGTGCAGAATTTTGTCTCTTCATGCTTAAG[T/G]GCAATGTATATTAGCTCTGTTGTATTGACGTTCTTTATGAATGTTGTTATAGTCTTGTGT
2351	CaSNP4388	ACTACTTAGTCTATGAAGAGACAAGGTTACATTCAAGAAAAAAATTATCATACCTTATTC[A/C]CGAAATTCTCCTTTCATGAGAAAGTTGTTGTCCTCTATTGTCTATGTTGATGATATTATC
2352	CaSNP4389	TTCATATGGTTGTATTATTCTTCGTTATATATGTTTTGTTCTATTCAGTAAAAGAGTATA[C/G]TAATACCTCTATAAAATGATGTCGTACTCTTACATTTTTTTCGTTTGGACTTTTTTTTC
2353	CaSNP4390	AAATGTAGTACTTAAAGCAGAAACACAGAACAACACAACCAATTTATTCCGGTTCACCAC[A/C]AACACGGTATCTACGTCCAGTCCATTCTAGCTGAAGGATTTAATTCACTAACTCACCAAA
2354	CaSNP4391	TGTATTTATATAAATTGTTGATCTACACTTAGACAAAATAAATTATAGAGAGCAAGAGTG[T/C]AGGGACGACTATGATGTGCTTATTGGAGTGCATTGTTTGTTGTTTTATCTTCAATGAAGG
2355	CaSNP4392	TGCTCGTGAAGTTTTATTTTTTCAAATTCTTTCAAGGTTGACTTTGATGTCTTATAACAT[T/G]TTCTCAAAGTTGTCGACATACTTCTGCGAGTTTTTGTGAGTTATCACTAGCAATCTCAAA
2356	CaSNP4393	ACATTTATAATAATATATACTAAATTATTTAAAGAAAACTTGCAGTGGATAAAGAAAATC[T/C]GTTAAAGAGAATACAAAATTTAATATCTAATTTAGGATATCACGTTTCTCTAAATACACA
2357	CaSNP4394	GTATAGGTAGTAAACATGTGAAAAAAATATAACTTACATTAACTATATTATTTCATAGAT[A/G]CATCCAGTTGTGCAGCCTATGACACTTCCATAGATGCCAAATGTTACATCAAAATCCTGG
2358	CaSNP4395	ATGCAATTCACGATTCATAGAAAATCATAGAGAAATGCTAGTATTGTTGGTTCATAAACA[A/G]TGATATATACTAGGAGATCAATCTTGAGAAGGCTTTAATCTTGTAAAGTTCAATCATTAA
2359	CaSNP4396	AAACTCTGTCTTTGTGTGATTAGAGCTTAAAACTATTGTATTAAGAACTAGAACATTTGT[A/G]TTGCTGGAACAATTTTCACAGTTGATGTTGAGCTGAAGAGATTTTCCTTTTTCCGTCTTT
2360	CaSNP4397	AACTTATAAATTATACTACCTTAAAATAGATAATCATTAATATGAGTCTTAGTCTTCAAA[T/C]TTAATATGGACACTTAGCAAAAACATTGTTCTTTATTTATTTGTTTTGTTATCATCAAAA
2361	CaSNP4398	GTGAGAACTGCTTTTGTCGTATGTTGTCAATCTTGGAAAGTGGTATGGTTGGAACTGAAA[T/C]TGGGCCAATGTATTCAGGTCAAGAAATATATTTTCTTATAATACAGCCCAATTCCTTTCT
2362	CaSNP4399	CTTTAGTAGATTTAAGATTATAATTAAGTCTTATCGTACAAGAATTCTCTTGTTAAACTA[T/C]TGCATTTGCAACTAATTTAATTGGTTTCATGACCTACATCTATCCCTAGACTACAACTCA
2363	CaSNP4400	GTAAATAAAGACGCGCGCGCGACGACTACGACTAAGACGTGGCGCGCTTGTAACTAGTAA[T/C]AAACACCAGTCAGTCACAAGGCAAGCATTCAATCACAGGGCCAACACTCAGTCACCTAGC
2364	CaSNP4401	ACACATACCAAATCCAACAATCACTCAAAACAGAAATAAAGAACGTTCTCCGTTTTCACA[A/T]CTTTAATCCAAGAATATTCTGGTTACAAATCAAAAAATCTATTGTAATTTAAGATCGATC
2365	CaSNP4402	AGAGTGGTTACTACATGGCAAAGTTTAACAGCGCATGGGACAAAAATAAAGTGATCAACG[A/G]AGGTTCGTGGATGATTTTCGACCACTACCTCACGGTACATCGGTGGACTCCGGAGTTCAA
2366	CaSNP4403	GAAAACAACGGGACCCATGTGAACGAAAACAGATACAGTGATGCAGTGGCTGTGATTGAA[T/C]CTGTGATTGAACCAGGTTCTGTAAAGCAAACTGATAAAGCTAGTAGAAGAAGCCTCCGTT

2367	CaSNP4404	CCCTGTAAGACTCATAGTTTTAAATTCTAATTTATGTATTTTGGTATTGAACTCTGAGAC[A/T]TTTTAGCCAAGTTATTGATATTTTGTAAGTTTAATGCCCAAAAATATCTTTTGAAGCCCC
2368	CaSNP4405	CATCAAATTATATTTCACTATTAGGGTTAAGACAAAAAAGATATTTTTGGAGATATTTTT[A/G]GAACTAACTTAACAAAATGGCATTATATTGTTATTTGATTGGACTGTGTATTAAACAGTT
2369	CaSNP4406	CGTACCTGCCGCAGTTCCCGCCGTCGTCTTCTTGTCTGGTGGACAGAGCGAGGAAGAGGC[T/C]ACTGTCAACCTCAATGCCATCAACCAAGTGAAGGGTAAGAAGCCATGGACACTTTCTTTC
2370	CaSNP4407	TAAACAAATTTGCATGTTGACTAGAATATAAATCTAGAAGCTTCTTCATATCACTCTCAC[T/C]GTCTCTCTTTCTCGTTGCCGTTGTCTTCGATTTTGCGATTCTCCACACAGGTAAACACAT
2371	CaSNP4408	GATGAGTGGGAGGTAGTGGAAACAATGCATACCTTACTTTTGAAGGAAGGAACTCTTTAT[A/G]TAGTAGAGCTATTCAGGGGAAATGACAGGGAAAGGCCGCTGAGGACATTACCAGACGCAT
2372	CaSNP4409	GATAAATATTATCATATTATGTGTATTAACACATAATAATCAACAAAATAATAGCATTTA[A/T]TTTTTTATGTATTTGGTACACATATCATGACATGATATAATATATATCATATTTAAATAA
2373	CaSNP4410	TATATGTTGATAAATTTTGTGGCTAATTTTCGTTATATGGATGCTTTCTATGTTGTGCTA[C/G]TTTGATACGTGTTATATTAAATGAGCGATATAAATAATAAATATTTTATTTTATTATTTT
2374	CaSNP4411	CACTGGGATGCATAAAGAAGGGGTCCAGCATGTCATTTTGATAACTGCGATTGAAATTTG[A/G]GGATTTCTTGATATGTTGTTGGTTGTTAGAACGATCATCAGATGAAGAAGAAGAAGAAGT
2375	CaSNP4412	AAATGAAATAAAAATTAATTATTTATAAATATTGAAGTCAATAATGTTTCGTAGAATCAA[A/T]TTCATCCATATATAATTAATCATGGGTTAAACATACTCAAAATGTATTTTTTTAATGTAA
2376	CaSNP4413	CATCTTATCTTGTATGATTGTCATATTTTACTTTGTTTATTATTGTAAATTCTGGAAGAA[T/C]TCAACATTAAAAGTATCCCTGAAAGATTGCTAAAATAATGACTTTTCTTGATCATTGCTT
2377	CaSNP4414	ACTATCATTCATTCTCATAGAATTTATTCTCGTCACATAAACTTAAAGCTCAATTGAATA[T/C]TTCGAAGCACATATACAGTATTAAATAATTAGCTTATATAAACACTATTTTAGATATACA
2378	CaSNP4415	CTTGTAGGCTTTTAGTTAGGCATGGCAACGGAGCAGGGCGGGTTTGCCCCCTCCACACCC[T/C]GCACCCGACTAATCAAAGAAAATCTGCACCCGTCCCAGTTTCATAACGGGTGTAAAATTT
2379	CaSNP4416	ATTTCCCTTATCCTTTTACTTTATTTTTGGTTTCAAAGAGATATAATGGTGAAACTCACA[C/G]ACATAGTGTGGAGAAGTGTAAATTCCAACTTGCACAGTAATATATATATATATATATATA
2380	CaSNP4417	TATCTCAAAATCCTTTCAACAACATCCATATGAGCTTCCCGTGGATCATGCATAAACTGA[A/C]TAACGACATTTACTGATATGTAATGTCTGACCGTGTATGAGACAAGTAAATTAGTTTGCC
2381	CaSNP4418	TCAACTCTCTATTCAAACAATCTCACGGAAATTTCTCTCAAGTGTTTAGCAAGAGTTATG[A/C]ATTTATCACTCAAGTCCTAGAACATGTTTCAACCTACCATAGGCTTGAAAAATTCTAGTT
2382	CaSNP4419	ATCATTGCACTGGAATTTAATACTTGCAGCTACATGTGTCACCCTTTTTTGGCTTCAAAA[T/G]CTTTAAAGGGAGTGTACAATTCATTCTAAATTGATTAGTATCTTTGTGCAGAGGGTATTC
2383	CaSNP4420	CTCTTTCTCTCTTACTTCCCTATGTATATATCTACCCCAACTCAACTACAACAATTTCAA[A/T]ACCAAAACTCATTCTATTATATTGTTATTATTATTATTATTATTATTTAGTTACAAAAAT
2384	CaSNP4421	ATTTTAACCTAACATTAGTATTGTAGCTGTTAATATCAGACAATATTTGTAGCCATCATT[A/G]TGAATAATAAGTAAATGAAATCTCCTTAATAATGCTTTGTTGCAATTGGATGCATCATGT
2385	CaSNP4422	CATCAAAGAACCAACAATTCTTGCTCATGTATAGGCTAACAGACAAGCTAGACATGGTCG[A/G]ATATTCAGATTTTGACTTTGGTGGTTGTGTTGATTCTCGCAAATCAACATTAGGAAATAT
2386	CaSNP4423	GATGAGAATCATAATTACAACCTAGACACAAGAAGAAAAGATAAGATAACTTAAAGAAAA[T/C]CTAATCTTAGACAGAAGTTCTAAAGACGTCTAACCACACGCCTTAAGAGTTCAGATGCTT
2387	CaSNP4424	TTTGACTTGGATTGATTCCTCTACACTCAATGTTTCTATATTATATACTCTAAAGACTTT[A/T]AACCTTTTTGAGTATCCTAAAACAATACACGTTTAATATTAGGAATCAAACTTTCCAAGG
2388	CaSNP4425	GTGTTATTAATGGCCACCACAAATGAAGAACGTTCTCCATTTTCTGAAATAGTCTTGTTG[A/T]TGACAACTACAAACCAATAACATTCTGGAAAAAGTGTTAAAAATGAAGAACGTTCTTCGT
2389	CaSNP4426	AGAACTGAAAAGAATCTTGTAGATCCTTTGACGAAAGATTTGACAAAGGACGTGATGTTG[A/G]AGACATCATTAAGAATGTGATACAAACTCATATCTAAATAACCATCAACCATGGAAACAA
2390	CaSNP4427	TTTTTCTTTTATTAATTAATTAAGACAAAAAGAAAGGAAGGGGTAGGGGAATGGATGTTA[A/T]TTTGTAATTTAGTTAGAATAAAATAGTGAAAAGTACCTATAAAGAGTTTTCCCTATCCAT
2391	CaSNP4428	AGAAAGGGAGAAAAAATAAAACCCTTACTTAGAACTCTAAGCTGATCTTGAACTCTAAAT[A/G]AGGATGAATGGGTATTGAGGATCTACGGGTGGCATCAAACCTCACTACTTCCGATTGCCA
2392	CaSNP4429	TTTAATTGGAATTAAACAAAAATTGTCTAGGATTCTTTTTGTAAAGAATTAATACAAAAT[A/C]ATAATTATGGTTTAAGAGAGAGGGGGTGTGAGATATAGCAAAGGAGAAGACTATGCTTTT
2393	CaSNP4430	GATCATCTATTTTTGGCAACTGCTGCCACCATCGACCACCTAGTTCCAACAAACATCGAC[T/C]ATTACCACTACTTCGACCATTGTCAATCATACTTCGACCACAATTTAGGACCACCACCAC
2394	CaSNP4431	TTTGATAATATTTTATAATAAATTCTTAAATATTTTAAGAGTGCAAAGGGAGTCTACTAA[T/C]AATGTTAAATAGTTTTATGGTGTCATTTAATCATAACCATTTAATTTGCCACATTGTGTT
2395	CaSNP4432	GATGTTACACTTGTGCCCTAATTGGACGAACTACAATTATTAATGGAGCTACATTGCCTA[C/G]AAGCGGGATGGATTAGAATAAACCTATTAATGACCTAAAATCTTAATCCGTTGAGACTTA
2396	CaSNP4433	ACGTATCGGTATCCGACACCGACACATATAGTTACATTCAATCATGATTATTTTCTCAAA[T/C]TATTACAAATGTCTACGTGTTAGTGTTGTGTCTGATGTCTGTGTCAGTACTTCATAGCTT

2397	CaSNP4434	GAAAATCGCATAATTAACTCAATGAGAGGCTAATGATTACCTCTTTAAGAATTATATTTA[T/G]ATAAGAAAATTGCATAATTTTTTGTAAAATCCAATATTAGCAAGCTTATCGCACATGTTG
2398	CaSNP4435	AAACTATGTTGAACATGTGTCTTTTACTCGATCAATGTACTCAAATAATTGACCCCCTAC[A/C]CTAAACAAAGTGGTGAGAGGTTACCTGATACTTGAGGTCCAAAATCTAGAAGTTCTATAG
2399	CaSNP4436	CCACCAAGTTGGTGGTGAATCCATGATAAATTTTGCTTGTGTCACTAATTACTTAAGTCT[A/T]ATTGCTACTACCTTTGTTTTAGTTAAAATCAACAAGCCATAACCAGTTGTTGAATAAATA
2400	CaSNP4437	ACGGTGGCCAACCATAGTATGTATTGGTGGATGATACACTCTCAAAACACTTTTAAAGGG[T/C]TTCACCATACATGATTTATGGGGAACCAATGTGTCTCAACCATGCATATACACTTTATTG
2401	CaSNP4438	ATTAATTCAATGACTGTTGAAAAGTGGTACTTAAGAATCTAGTAGGTGCTTTTTAAAACG[A/C]TAGTATGTTAAGTTGAAAAAGGCAGTCTTACTTTCTAATAGGTGCTTTTTACAATCTCAT
2402	CaSNP4439	TGAGAAGACTTGGCTTGTAGAATTAAGGTGTTTGTATTCCTCAAAAAGTTTGTAGGTGTC[A/G]GGTTGAGTTGAGAAGACTTGACGTGTAGAGCCAAGGTGTTTGTGTTCCTCAAAAAGTTTG
2403	CaSNP4440	TGGGATGAAGGTTGTGCTATCAATTGAAGTAGAAATATCTTCCCTAAGAGTATTGATGGA[A/G]GCAAAACTAAAGGGGTCAGAACGAGTACAAACTCGTTTCAATTAATTGAATCTAATATAA
2404	CaSNP4441	TATCAATAAGCTACAGTTGAATAAGTAAAATTTATTATTTATGATGACTTATTAAGCAAA[A/G]TAACTCGTGAATATAAAAGTTAATCATACAATTATTAATTCATCCAAATTAAAATTGACA
2405	CaSNP4442	AAATGAATGACCTTATAAAGTATCAATTAAATGTGATTGTGAAATTCACGACATTCAGTG[A/G]CTATCCATCTATCACCTTTCTTCAAAGATCTAACTCTTAATTTGAATGAACATTTACACA
2406	CaSNP4443	ATAGAAATTTACAAAAGGTGTCATGCATGATGATGTCAATGGAAATATCATCGAGATTAA[A/T]TCATTTTTTATCTAAAAGAAATGGTAAATTTCACCGTAATTAACTCACACAACTCTAAGG
2407	CaSNP4444	ATTAGAAAAGAATTACAAGAAAGATTCATGAATGATTCCTGATTAAACTTCTTCAATAGT[A/G]TTAGAAACGGTCGTCTTTGAGTTTCTATGTTATGGCACAAGTCTCCAAACTTTCCAATAG
2408	CaSNP4445	TTATTTGTAATGTCATTATAGAATAGACCATACCAAAAATCATACTAAATGAGATTTTAT[T/C]ACATTCACTTATAATTCATCAATACCACATCAATGGTAATTTTATCGCTTTCACTTATAA
2409	CaSNP4446	TAACTGTTCAAAGTGTGTATAGAAGAGGAATATCCTTGTCCCTTTTACTAATGAATTTAA[T/G]ATTTTTAATTATGTGGGAACTCACCTTTTTCAAAGCCATGTTGTATGGTGCTTTTAAAAT
2410	CaSNP4447	AATGATTATGTCTTTGGATTTGTATTTGTATTGATTGGTTGATGATTGTGAAGTGGGTGG[T/G]GATGTTTCTGGGTTGAAACGTTTTACTGTTCCATCAGAGAAGACTTGAAGAAATTCTGGT
2411	CaSNP4448	AATGATTTTCATCCACAGATGTTGGATCTAATTCAGGGAGTAAGCCAAAAAGAGATGTAT[T/C]CTTAAAAGCAGATATTGTCTTTAGGGAATTATTTGAATTTCCAATCATTGGAGTTGGAGG
2412	CaSNP4449	CAATATGTTCCAGAATTGTGTAACCCCACCCGATAGTGACTTTAGTGTTCATTAAGCAAT[T/C]TGTGTTTTTCAGGTTGTGTGGAGAAGATTTAACTTGTAGGGTCAAGGTATTTGTTTTCCT
2413	CaSNP4450	GTCTTTGCAATTCACATAGATACATGTGATTCATCTTCCTTTGTGGTGGGATAGAGATGT[A/G]TAACTCATGAAATTTTCTTTACCATTCACATATACATGTGATCCATTCTGGTTGTTTGGT
2414	CaSNP4451	ATTGATTTTTCATCTTGATTTTTCTATTATAATGTTGTACCTCCAACTAGAACTGACTGT[A/T]TAGAACCAATAAGGACTGGTGATTTACCTAGGGAATGGAAGTTCAACAAGAACCACCCCA
2415	CaSNP4452	GGGATAAGTTTAAAGAGATATTTCTAGAAAAGTATTTTATCCATAGTGTTCACATTTAGA[A/T]GGAAACTGAGTTTCATCATCTTTGTCAAGGAGATATGTCAGTTGTAGAGTATGTCACCAA
2416	CaSNP4453	TAGGACATAAACTGAATGGAAAGAACTACCTTCAATGGTCACAATTAGTGGCAAAGGAAA[A/C]GATCAATATCTTACTGGTGAAACAATTGCCCCAAAAACAGAAGATCAAACCTACAAAAAC
2417	CaSNP4454	CCTATCACTGAGAAGATGCGTGTCAAGAGGTTCATCAAAGGATTGAGGGATTAGTTATTT[A/T]GATATGTGGTTGGGTCAAATTGTTCTACTTTTGTTGAGGTTTTGAGTTTGACACTTTAGA
2418	CaSNP4455	GACTTACACAAAAGGCAGAGTATATCGATTTACTAGAGTCGAGCTTATATATTTGACATG[C/G]AAATTCTATTCTATCAAAGGTAAGGCATATACAAATCAAGACATCATTCAGTTATGGAAA
2419	CaSNP4456	AATATCTATAATTTATAATGTGATTTAGACTATATAGATAATCTCTTGTATGAAGCGTTC[A/G]AAGGAACTCCAAGAGACAATCAACAAATAATTACTCAATAGTTAACCATATGATTCACTA
2420	CaSNP4457	TTATAATAAATTTAATTAAATGATAAATTTATAATAAATTTTACGTGTACACAATGGTAT[T/C]CGTGGACAAATTCAATTGCTATTAAAGAATGATGAGAAGAATCTTTTGATGGTGGTTCCT
2421	CaSNP4458	ATATAGCATCAGGATTCAAAATGCAACTCTTAACCTCTTCTAGTTTCGTTTTTGAAACAA[T/C]TTCATTGAGATTTCAACTAAAGACACCACAACAACCTAATTTTATTACGTATTAGTGCAT
2422	CaSNP4459	GTATCTATGACATACCTTGACAATTGACTTTTTGGTGGGTCCATGTTCCAATTTTTTGAC[A/G]CGGCCATGGAGATATCATTCTTGGAGCAATACTTATGTGTAGGTGTCTGCCGAGAATACC
2423	CaSNP4460	ACCATGAAACGACTATTATAGAAGGAAAAAACTATAAAAAAATGCTAAACCAATCATTCA[A/G]AAGAAACTCGCCGATGTTATCTTAATATCGTTACTATTTTTTATAATATAATATATATTT
2424	CaSNP4461	TAGTATATTAGGAAACTCTTATTGGATTATGCATTTCTATCTCACTTTTGTAAAATTGGA[A/T]GTGTGGAATAAGGAAGTTCTAACATTGGTTAACAATGTGTTTTAGTAAATTAAGTGCAAG
2425	CaSNP4462	TGTTAACAAGAACATATTCATATTGCTCATCCTTTAAACATGGACATCTAATGTCTTCGA[T/C]GATAAATATTTACTTAACATATTCATTATCATTAAATTTGTATAAATATGCTCAAATGAC
2426	CaSNP4463	TGTAGGTGGAACACTCAAAAGCATGAACAATAATTTTGAATTTGGAATGAGCTGATCGTC[A/C]CCATGGAAGTAGTAGAGATGCAATTCTTCTTGGCACAATCGTCAAAACCACATTTCTTTT

2427	CaSNP4465	AATACCATCAGTATTCTTCGGAGCAATATAACCAACCATCTTCACCAAGTCCCAAACTTC[A/T]GGATCTTGTGAAATATAGAAACTTCTTAGCTTGTCCTTCCAATACTTAAAACGTTATCCA
2428	CaSNP4466	TTCCCTCAATCAAGAAACGAAAGCAATAAGCACATATGCACATTGTCAATATAATTTTGT[T/C]TCACGTTAATGCTAAGAGAAGTTCTGAAGTAGCAAATTTGTAAACACTTTATGCGAGAGC
2429	CaSNP4467	CGATTAATGTTTTTTACAGAGACAAATAGTATTAATTTCTTTGTTCTATGTATAAATTAT[A/T]AATCAACCCATGCTATACTATGCACATTGAACTAAAAAGTGTTCGAGTAAAAAAAGTGAA
2430	CaSNP4468	AACTCTAAAAATAAAATGTTATATTTATAGGAGGAAAACTTAATTAACCTTGATTATGAA[A/G]ACAAATTCATCTTTTTTTGCACTGTGTACTACAATACGCTATTTGTGTGTCTGTCGCCAA
2431	CaSNP4469	GGTGGCTCGTCAAAGGCTGAAGTTGACGGCGGTCGTTGAAGATTTGTGAAGGATGATAGA[T/G]GTAGTGATGTATGTGTGTCTATTGCTCTACATTTTCTTCTCTATTTTTTTAATTGTTTCT
2432	CaSNP4470	CTAGAGTCAATTTGATATATTGTAGTACACAGTCTACATAATATAAATGTTTTACCTTAA[A/G]ATAATGTATGAATTGACTCACCACGCTAACTGCATAAGCCAAATCTAGTCTTTTGTGTGC
2433	CaSNP4473	ACAGCAAATACGGGGAAATATTTACTTCTAAACAATAACACTTTTATGCAAAAGTAGAG[A/G]TAAATATGTCCTCTATTTACATGTACACAGCATATCAAATGCATTTTTGGTTTGGAAAAT
2434	CaSNP4474	TGAAAACAAAAATACATACATACAAATGAAAATAATATTCATTAACCTTTCATTAATTAA[T/C]CAGCACAAAACTGAATAAGATTAGGCAATTCATATAAAATCAATGTTTTGAAAAATGGAT
2435	CaSNP4475	CAAATAATAACATTACATTATTTCAAATAATATAACATTATTGTTAAAATTAGGGCTTCA[A/G]TTTAGTCATTTTTTAATTCTGAATTGTATCTCCATTCCTAATTTGGTCAACAATGGTCGT
2436	CaSNP4476	TTTATTCTTGTTCTACTAACCTAATCTAAAGAGATTGCTCTTCTCAAAGGAGGCATGACT[T/C]CTCAGCTCTCGACGTTCTCAATGGTCTCAATCTCACAAAAATGTAATTCGTGTTCTTCCA
2437	CaSNP4477	TATACAAATCCTCTCTTTTTCTTGAATATCAGCTTAAAAGAATTTCTTTTTCTTCTCCAA[C/G]AATTATGACTGATATATGAGAATAAGGAATTCATATTTGTAAACAAGAAATGGATAACAT
2438	CaSNP4478	TATTTAACATAATTCAAAATAGAGCTCCCATTTAATGAGAGCAATTTTAAAAGAAAGAGA[A/G]TGATATGTAGTATTTCGACAAAGTAGATTTAAAATTAGAATTTGGAAAGCATAAAGTTAT
2439	CaSNP4479	AATGATCAAATAGCTGAAGTAATGAATAACATGGCTACTTCTGATGCTGTAAAGACAACT[A/G]CAAAGACTCTACGAGATCTGTAGAATAGGGAAAGAGAGATACGTGTTGCTGCGTCAAGGG
2440	CaSNP4480	TACCATCCCCTTCTTTATCTCAACGGTGATAACATCTTTTATCTGTTTTTAGAGCAACAT[T/C]CCTTGAATAAACCAAACCTCATAAACATTCAATTCCAGCATACCACTTTTTGATTTGCTT
2441	CaSNP4481	TGGAGTTATTTCTCAAATCTCTTAGGTTCAATTGGAATAGAAAATTGCTGGACTTTAACA[T/C]TTAGCACCAATCAAGTTTCTAACTTAATGAGCTAGACATTCAAAGTCAAATGCCAAGGAT
2442	CaSNP4482	AGAGAGTTAAAAATAGATGTAGGCATAACATTAATGGAAGCTCCTAAATCTAACATAGCA[T/C]TTTCAAATTGATTATTGCCTATGGCACAAGGAATGAAAATAGTTCTTGGATCTTTGCATT
2443	CaSNP4483	GAATTGTAATTGTAAACTGATTTGGTATTGTAAATTGATGAGCCTAATTACTTTATTTCC[T/C]TTTTTAATATATGTTTCCTTTTTGATTTATTTTTAAATAAATTGGACAAAATTAGGGTAC
2444	CaSNP4484	GTTTGATATAGCGGAAACGGGATTAAATATTAGGGAAAACGGAGAAAAGATACATGTTTC[A/G]GTGTTTTCGTTTTCGGGACCGAGGGTTGGGAACGTGAAATTTAAAGGGAGGTTGGAGAGA
2445	CaSNP4486	ATATATTATATGATAGAGAAATGTTATTGAAACATAAAACTTGACACAAAATATGACATA[A/G]ATACATTACGATATAAAATACAGTGATGATCATGTTTTATTTGTATTTGTGTCAAATTTT
2446	CaSNP4487	TGCAAATGGACACACAATGACAAGAAAAATCTTGAGGGTTGGTTATTATTGGTTGACCAC[A/G]GAATCTGATTTCTTTGGTTACATAAAGAAATGTCATAAATGTCAAATTTATGCTAATAAA
2447	CaSNP4488	GCGCTGCCATGGCAGGCATCTCTTCACAAATTGCCTAACGAGGTTGTCGAAAAATCTGCC[A/G]CGTCATTCTGCTAAGGCGTCATTATCATGGCCACCATTTAACAACACTGATTCGGTTTTC
2448	CaSNP4489	AGGACTAAGGCCAATAGATCATGTTTGTTGCCACTACTAGATGAGATGGATATTTTTTAC[T/C]AACTTATCGCTATTGTTTATCATTATTATTTATAAAAAAAATATGGTGTCCCCTTTTGTA
2449	CaSNP4490	CATGCCCTTATTCAAAGCCGCCACACCCTGCTTACAATCAACACCTCTAAATGACAATTT[T/C]AGAAAAAGAATACTAATTTGCAAAGAAAAATAGATCAAATAAGAAATAAAAGATTAAAAG
2450	CaSNP4491	ACATATTTTTTATCATTAGACTTTGAAATTAATTGGACAAAAAAAAAAGACCTTTTGAAG[A/T]TAATATATAAGACCCAAAGAAAAAGACCGTGCAAAACATTGATATCCATTAGCCTATTCA
2451	CaSNP4492	GAAGTGTGAAAAACACAATTTTCAAATGGAGAGATTTGAGTAGTGGAATATTTATTTGAG[A/G]AAGGTCTCCCACAATTATTTGTTTCAACTAAAGAAAAGTAAGAGTCTTACAACTGAAAAT
2452	CaSNP4493	AACATTAAAATAAAATAAAAAGCACTACAAGATTGCATTGTATGGTAAAAGAGATGGGAC[A/G]AATGTTGTTCTTGTCACGTGAGTGTGTTAGTGAAATTTATGTCTTTGATAGTTGTTGGAA
2453	CaSNP4494	ATTATTCAAATTTATGTTGATGACAATATTTTTGGATCTACTAATGGCACTCTTTGTCAA[A/G]AATTTTCTAAATTGATGCAGCTTGAATTTCAAATGAGTATGATGGGAGAACTTAAGTTCT
2454	CaSNP4495	TGGGGCGCACTTCATTTTCCGTCTAGAGTTTAGTGCATGTTTCTCCTCTTTTGAGTTCGA[A/G]TTTGATTTCGGTGTCTTCGTGAAGGTTGTAGCTATGGATTATAGCTGTCATTTGAATTTG
2455	CaSNP4496	CATTAACTTTTGGAGGAAAGTAGAAATGATAGATCATTGGACACAACACTGTTGGTAATG[A/G]CACATCTTCTTCCATTAAAGATGCTTTGTTTTTTAAAGGTCTTAAACATATTTATTAAGT
2456	CaSNP4497	GAAAGACATTCTCACAAATAAAATAAAACTAAAGCTATATGCATAACTGAATTGAAAGGT[T/C]TTGTACATTGTTTTGCAGGTATGAATATGAATTTATTGAAAAGATTGTAGAAGATGTTAA

2457	CaSNP4498	TGTTTAGAAAAAGATGTGTTAAACCAAGCCGATCTACAAAATTTGATAGAAACCAACCAA[T/C]ATGTACAAATTAGAAACAAACAAAATAGAGCCGTGACCGTGACGGTGGTCGGAGAACGAA
2458	CaSNP4499	AACTTTCCATTTTTGGATGGTTTATTTACCCACAAGAATATTATATGACACAACAAATCA[A/G]TAAAGACTTCATCGGGACAATCAGTCTGATTCTTGGATGATTTATTTACCCGAAGAATTT
2459	CaSNP4500	AGGTTGTTAGATGAAGATTGATGTGGTAGCAGCGACAACGAGGTTGCTCTTTGAAGATTG[T/C]AGCGACGACAACAATGAGCTGCGACGGTGGCGAGGTTGCTTGTTGAAGGAAGGTTGCTCG
2460	CaSNP4501	TACTTTATAATATGTATCAATCAAACTTTGAGAACACATATAATTCATTAAATCAAATAT[T/C]CAATTGGGCTCAAGGTGAAAATGATTCTTCTACCACACTTCCCTCTCGACATCCTCAAGA
2461	CaSNP4502	AGTTTATGTCACTAAGAAATTAGGGCAAGAATAATAAAGAGAATTCGATAATAATTCAAT[A/T]AAGGAATTCAGTAAATAGGATCAAATTAGACCCCAAGGTTGGATTCGAAGTGAAACTCAT
2462	CaSNP4503	AATCCTAGAACATGCGTCACACTATCATATGCTTGAAACAAATTATAGTTAGCATTCACA[A/G]TGCAACAAACACATACACTTTTAGAGGACTTTCGGATTGTAATAGGGCTTAGGTAACAGT
2463	CaSNP4504	ATCAACATAACATATCACCTAACCACCCTATAATCAAGAAGACCTTCGCTACAAGGAACT[C/G]ACCACCTTAGTCAAAATATTGACTCTGATACCACTATTGTGTTGAAGTTGATTGAAATAA
2464	CaSNP4505	CACTAACTAAGAAGAACACATGGTTATTTCGTCTACAACTTTGTTCAATATATCCAATGT[T/C]ATCAAGATGAGACTAATGTTTCTTAAGAAGTGCATTCGACCTTGTGGTGATACCAGAATG
2465	CaSNP4506	ACTATGACTATCCTGTGGAATAGTTTTAATGGAATATTTTAATTAATGAGGTTATTATGC[A/C]GTTGCATCTAACATGTTAATATTCCCTTTTTGCAGGGTGATGTTGAAGCTTGTTACTTGG
2466	CaSNP4507	GTTCTCATGAACAATCTTTGAATATCCAGAATTGTGAGAAGCCACATTGACCAAGATTGA[A/T]GACTACTTTTTGTTCTTCCTTTATTCTGTTTGTAATAATACTTTGAAACGAAACGAAAGC
2467	CaSNP4508	TAGGAATTCCCCTTCCGAGTACCTCCAATGTCTAACAATCTTACCTCAAGGATTAAGAAT[A/T]GACTACCATGATCAGACTCATTCAGCTGTACTTCCCCGAAATGACTAGGCTTGTCAGACC
2468	CaSNP4509	AACAGCTATAAGCCAAGCCAAGAATCTTGAATCACTTCCCCTAGAAGAACTTATTGGAAC[A/T]TTAAGAGAACATGAAGTACTTCTACAAGAAGACAAACCAATCAAGAAGGGAAAGACAATA
2469	CaSNP4510	AAATCTAAATAAATCAATTAGATCTTAAGCTTTAGTTCATTTAATCAGATTAGACATTGG[A/C]TTGATATTTTTAAAGATGAAAGTCCAATCCGAATCGAATCACGTACTATATTTCAATCTT
2470	CaSNP4511	ACAATCTATCTTAGAAGCAATATGATCTAATGCATTAACTTCATACCTTCCTGCTTTCTT[T/C]TAAGGAGATCGATCACTTGACCATCGGCAGTGGTTCTATTATGTGTCTACAATTAGAGCA
2471	CaSNP4512	CAAGTCTCCCAACAAGCATCTAAAATAGGCCTCTCAACAAACACCCAAACAACCCTTTTA[A/G]CAAGCACCTAAACAAGCCTCTCAGCAAGCCCCTATGATGAGTGAAGTTAGAAAATATTTG
2472	CaSNP4513	TGGAGTTGTTTGGCTTATGTTAGAATCCCCGATCCGACGAATTAAACTCGCCAGTAGAGC[T/C]TGTGAATGTGTATTCATTGGGTATGAAGTAAAGAAATCGTATAGGTTTTATGACCTAAAT
2473	CaSNP4514	AATGCCGAAAGACTGTTGTTCTGGAACACCAAAAAGTCTCACATTGTTTGAAAATTGAGG[T/C]TAAAGATAATTTATAAACACCCACTTTCATCAACTCAGTGAGGTTCCTTTTAAGACCGAA
2474	CaSNP4515	TCACTTTTATACACACTCAACTACTCTATACACCATTCTCAAATTCTCAACACTTAGAAT[A/G]CTTGAAACACAAACACTTTCACATTCACCTAATCATGTATACATGCGTATAACTAATTTA
2475	CaSNP4516	TGTTCTTGCAACGCTTGCTCCCAATTGCATTCAAAGCTCTACCAAAACCTATTTGGAACA[C/G]AGTTACATTGTTTAGTCAATTCTTTTGAGAGCTTACTTCGTCTTTACTAAAATAGGATAA
2476	CaSNP4517	GAATAATAAATAAATATTAAAAAGCATTTTAAGAAAATTATACGAATTGGTGGAACTAAC[T/C]ACAATTCAATTCAATTCCATAAGATTCAATCTGGTTAAGATTGTTAATAATCCTATTGGC
2477	CaSNP4518	TAATTATTTTGTCATTTAGGATTAGCAGTAATTTGATTGTTTACAATGCAGTTGGGTACT[T/C]TTGGAGACTCTTGACAAAGTGTCACCTGGGATTGTTAATTGGAAGATTGCAAACAAGCCT
2478	CaSNP4519	ATTCAAACGTACGTAGTATCTAATTAAAATAAATTTTATCCATCAATGTTAGGATGGATT[T/C]ATATCACTACATGTAAATATGAAGGTTGTGATAGGTTGGAGGTGCTCATCCCACACACAT
2479	CaSNP4520	CTTTTTGTTTTAATGAATTTGTTTCGATCTTTTGTTAAACATAAAAAGATCATCATCAAG[T/G]CAATGCTTTGTATATATTATATATGTTCTCTTCGTCTTATTCTGATCAAGGCGTTATCAA
2480	CaSNP4521	CTTCTCTTAAAATTAATCATCTATGTAGTTAATAAAGAGCACATATATGAAATTCTGAAA[T/C]ATGATGAGCTGGTGCGGGTGTCATCAACATCTACATTAAGTGATGTAAAATACTTTATTT
2481	CaSNP4522	CTTCAAGCACAACTAGTGTATTGCTTCTTCTCATATGTGTTTCATCATGTGAGGCAATTT[T/C]AGATTGTAAAGGTGTGGGTACGTGCCTTTTACCATGTCTAAGCTACCTCTTCGATAATAA
2482	CaSNP4523	TGGTGACAAGTGTTATAGAACTAGATGTTCTCATGTACGATTCATATGGCCTGATGATCA[A/G]TTTAAGAAGCAACTGACCTTAGCACAACGGTTTGAGGATGAGCAGAGTAGAGAAGTTCAT
2483	CaSNP4524	TACCTATGGATAGAAGCATTTTGGATGTTGCTAAGGGGGGAACACTTGTCGATAGGACCC[A/C]AGAAGCGGCAAATACTTTGATTTAGAACATGTCAATTAATTCTCAACAGTTTACAACTAA
2484	CaSNP4525	GAACCAACCAAATAAAAGATCAGCTTGTATGATACAATCCTTCTTATGCAATGATTTTGG[A/G]TGATACATATCACAGATAAAGATTAACTAGGAATATGATTTGAGATCAAAATCAATAAAA
2485	CaSNP4527	TCTCCAATTATAGCACATAAACTCATATTCTTCAACTACCTCATCTACTTTTAACAACCT[A/G]TACCGTACACGATTACATACACTCACACTACTATACTCCTACATGGTCAAATCACAAGTT
2486	CaSNP4528	CTCAAAAATTTATAATTATTTTTGAATATGTCATTTGAACGTTTGATATCAAATCAATGG[A/T]TGAGATTATTTACTGCATATAACTGTTTTTTAACACGAAATCCAAATCCATGTATCGTAT

2487	CaSNP4529	GGTGAAGCTATAGCAGAAGTGTTAGGAGCATTTCCTGATTTGTTTCAAGATCCAAAAGGA[C/G]TGCCATCCAACAGGAGGCATGACCACACGCCATACTATTCATCATGGGGCAAACGGAGGA
2488	CaSNP4530	CTTCAAATTTTTTGTAGACGATGTAATGAAGTCACATGAATCATTTTGAGATTATCAACT[C/G]GATTTAACTAACGAAAATGGAAACATGCCAAGTTCCAAGTATTTATATCAAACATAGCAC
2489	CaSNP4531	CATACAATACATATGAATTATTTGATTTATCTAATGATTTGAATTGAGAAACATTTCAGG[A/C]AAATAATCAAACACTACACACTTGGACAAAACTTGGAACTCAAGCAATCAAGCAAAGATG
2490	CaSNP4532	GCTTGTCCTCAACATATCTAGGAGAAGGTTTACAACAATCTACACATAAATGTGTGAAAT[T/G]CTCATAGATCCAAGCCTAAACATATATAATAACATGTCATTAGTTTAGAATTAATAATAT
2491	CaSNP4533	AATAATTAATTAGATATTTCGGTGTATAAATGTTATATATTCTTGCTTTTATTCCTTTTG[A/G]AAAGAGATGATGATGGTTAGCTATTGTCTCGCTAGAAAGTGAGCCGCTGGCGAGGTACAG
2492	CaSNP4534	AACCCTTACCGGTGAAAATACTGAAAAATAATGGGAGCAAAATCAACCTAAGTCACATAA[A/G]AGTAAAGATAGTAAACTAGCAGCTATATCCAAAAAATAGTTACATTTAGTTTCTCCCATT
2493	CaSNP4535	TAACTATGGTGAAGGGTATTGATATTATTAATTCATTCATTTCAATAAATTGGATCTTCT[A/G]CTAGTTGTACGGTGCAGTCAGCTGTAGACTTTTTATCTCTCAGTCTAATGCTGACTGCAT
2494	CaSNP4536	TTAACACACTATTCATCCAAGGTTGTTCTCCCTCTCTACTTAATTCATAGTACTCTTTGG[T/C]CTCTTCATACCTTTGCTGAATTGGATTTGGCACCATATCTTCATTCCGATCAATGTTTAC
2495	CaSNP4537	AACTTTCCTAATAAAAAATAAACATTATGCATTGACATATGTTGCTTGAAATTATCATTT[A/G]TGTTGCAAAGTTGACTGCTAAACCGATTTTGTGGCAATGCATTGAAATTGCATATTATAT
2496	CaSNP4538	GGTGAGAGATATCTTTCACTTATAAATATATTTTTTAGGCCATAAATTATCTAATGCGAT[A/G]AATCATTTTAGTAAACTTGAATTTGAATCTTGGTTATAATAACTTCTTATTATATTTAGT
2497	CaSNP4539	GTAAAGGAATCGAGACCGTTGACACCGAATTAACGGTGGGGAGAACTCTGAAATATTAAT[A/G]TTGTTGTTATGGTTGATGGTCAAGAATGTATACATGTTGTTAAGAAATCTTTGAACTTGG
2498	CaSNP4540	GGAAAATTTTCAACAGAGTCTTCTAATCCAATACCTTAAAATGTAGCGAAATTTTTACTC[T/C]ATACTCTTCACATTTAATCTTTATTCTTACAAATGTAAGCAAATTATCTTTTTGCTCTCA
2499	CaSNP4541	GAAAGAATAACAATTTCGGTCGAATTGCATGACTTGGAGAGTAATAGATTTACCAACTTC[A/G]TATGTAATCTTGTTCATGCAATCGTTTGTGTATACTCGACCATAAGCTAAATGATGTCTG
2500	CaSNP4542	ACTAGCTCATGATCTTAGGGGGACCGCGATTTTTTATCCTCCGACATGTTGCTTCCATCA[T/C]GATTCCCTGATAAGCAAAATCTATGAGAAGGTGAGAATGTCACCTAAACAGGGTGAAGAA
2501	CaSNP4543	CCCTTACCCACAAATCCAGATTTTGAGGTTTGAGAAAGATGCAACAACATGGTCATCTCT[T/C]GGATAACAAAAACCATATCTCCCCATATATCGCAGAGCGCCATCTGCATTGATTCATCAT
2502	CaSNP4544	TTCTCACATTTACTCAACCTGTTCTTGTTTCCATCTTGCTTTTCCTTTTGGTTAGATTGT[C/G]TGTGAGAACTTATGGAAACACTGATATATTCATAACTTGTTTTCCGCTTACTTTCGTAAG
2503	CaSNP4546	AAAAATTAGAGGATACAAGAAAATCAGAAGAATATGAAAAAATTTCAAATGATGAATCTA[A/G]TCAAGTCAGAAATAGTCAAGATAAGTCAGAAGAAAGATTTGGATGGAAGTACCATTCATA
2504	CaSNP4547	AATAAAATCAAAATTTTAATCTGAACAGTTAATTTATTTTATATATAACTAATGGTCAAG[A/T]TTAATTGTTGGTGTTCGCTTAATCAACTATATATGGTCACTGAAATGATTAATCCAACCA
2505	CaSNP4548	TGGGTCTCGTGATTATGGGCCTAAGGGATCCGGGGTTAGGTTTTTCTTATAGATCTAATC[C/G]TACTTCCGGATCTGGGTTAACTCACTCGGGTCGAGCGCGAAACAGTGATTGGATCGTCGT
2506	CaSNP4549	ACCGCGGCTATTGCAAAAATTGAGAATCGTTATTATAACGGTAAGAGACGACAAATACGT[T/C]GTAAGCATATCACAATTAGATAATTTCTTTCTAAAGGAGCTGTTATTGTGGATCATGTAC
2507	CaSNP4550	GGTATGTTATAGATACAATCATCCCAAAACAAAATAAGTACAACCACCAAAAGAGAGACA[T/C]TAAATCCCTATACAACAAAAAACTAATATGATCATCCACAAATAACTATAGTAACAACAG
2508	CaSNP4551	TTTTAAAGCTGTCATCTCCTCAGCCATCGCTTCAGTCCATCTAGGATCTGCTAAAGCTTC[A/C]TGTACATTACTAGGAATAGAAATTGAGGATAATTGATATACAAATTATGCATATATATGA
2509	CaSNP4552	CAATAGATACCCATAAAAAAGTTATGCCAAGATTACATGTCATTACTTTATCTAGGTCGA[A/G]TGGTGGACTGTAGAACTCCCCCCTAATATGAATTCCTAATAGATTGACAATATCATCCAA
2510	CaSNP4553	TGACCCGCTATTTAAAATTATTACAAAAGTCTAGATTATGAAATCTACAATTTAGGCTCA[A/G]TAAAGTTCGGAGTTGGTCTTTTCTTCCAACACAAAAGTTGTAGCTCTGATTTTTAGCTTT
2511	CaSNP4554	TTGTATGTCATACAATGTGAAGAGATTAAATGGCAAAAGGGTAATGGTTTGATGATAGTT[T/G]GTCAACACTATGAAGAATACAAAAGGTAATCGGATGTCTGGTGTAAGTCATGACTTGTAC
2512	CaSNP4555	GTTGTGCCATATCTTCTGAGTTTTTGTTGATATGAAAGCTAGCTCCTCATCATCTGAGTT[C/G]AAATTCTGATCATCTGAATCATTATTCTCTAATTCTACTATTGCCTTTAAAACCATATTT
2513	CaSNP4556	GCATAACCGAGTACACAAGTACATACGTTTACAGCTTATAATATGCTCTCACTGATATTA[A/G]CCAAAAGTAATATTCCTTCCATTCTTTCTTAAAAACAAATTGGAGAAGAAAAATGACTTT
2514	CaSNP4557	AGTGGGTTGTGGCCACACAAATTCACAAATGTAAATTTATACGAGTTCGAGCAGTCGTGT[T/C]CATGTAAGAAAAATAGACATACGATTATCTGAATCTGATCAATTTTTTTATGATGAATCT
2515	CaSNP4558	ATCTCTACCTTTATTAAAATTGATATATCTTTTACTTTCATTGTTTATACTAACAACATA[C/G]TTCTGAAAAATTATCATACTCAACCATATTGAGTACAGTTCACTTGAAGGTAAATCCCTC
2516	CaSNP4559	ATAGTGGCTTTGAAGATTCAGTTTTAGATATCGAACCCATTTCGGTTCCTGAAGAACAAA[T/C]CTTTGAGCTTGTTTCACATTTGAGGCAGTAAAGTTCCCAAGAAAGTCCCAAAAAACATCG

2517	CaSNP4560	TTGTATGCCTACACATAAGGATGTAATCTTTGGTTCCATGAAGGTATCTCAACACTTTCT[A/T]TGCAACTTTCTAGTGGTCAATACATGGATTACTCTGACATCTTCCTAAGATTCCAACAAC
2518	CaSNP4561	ATGAAAAATCACCCTTTACCATGCAAGTTTCTCACAATTTTGGTGGTCGTGGTTATTCCT[T/C]TTCTTCAAATCGAGGTCGCGGCAGAGGCTTTGGTAGTGGCCATGGTTCAAAATTTTGTAC
2519	CaSNP4562	ACATTCCTAATATTGCATTTTTCCCCTTTAATCCATCGTTGACCACAATATTTAAAATGT[T/G]CGTACAACACCCAACATGCATGTGATCTTCTTTTAATGGATGTCAATTCCAATCCTCCAT
2520	CaSNP4563	TGTCCTTAGAGATATTGGTTGCATGTTCCTAATATCTGTTCATGATTTGTATATTTAAGT[A/T]TTGAGATAGTCCTATTTGATTCAGTATCATTGATTATTAATATTGCATGTTAGGTGTTTG
2521	CaSNP4564	AAACCATATTAAACACGGTTGTAACTTGGTCTATTAGTGGTTCCTGCTCTAAACAACTTG[A/G]CCGTACTAAGCTAGACGATTGAAAAGACTTGAACACTACAAGTTTGACTAGAAGATGTTC
2522	CaSNP4565	CTTTTCAGATATGGTCGTATTTAAATCAGCATCGGATTTTAGTTCCACTGATTCCATTAC[A/G]AGCTCAGAAAGTTCCAATGCTGAGTAAACTTCTTTTATGGTAAGAGTCATGTAGTCACCA
2523	CaSNP4567	AGCCGTACGACAGCGTTTAAAGACATAGGTACGCTTATCATGTTCGCTAGTTCCAAAATG[A/G]AAAGCCTTGCTTGCTTTCGTGAATCTGTATCTTCTTCCATTATCCTCAACTGTTTGATTT
2524	CaSNP4568	GTCGTTGTAGAAAGTAACTCCGCCAGAAACAGTGAGAAACTCTCTCCATCTTTTCTTTTT[A/G]AATTATTCTTCATATTTTTCTTCCCCTCTCCTGTAGCACGCGAGGATGCGATTCCACCGC
2525	CaSNP4569	AATATGTTTTAAAGAAGTAAAGAAAAATGACCAAATTCAATTCAACAACTTGTATGCTTA[T/C]GAAGTGAGATCATAAAATGAAATCAAATGAAGTAGAAGTAATTAAATATCAAAGGAAGAT
2526	CaSNP4570	ATTTATATGTATTTTTTATTTTTAAATAAGTGCATAATATCAGCTTCACTGTCTTAGTTA[T/C]GTGATTAGACATCTTAAAACTAAGAATATTTAGATGTGTTTAGGCTACTGTGTTAAAGAA
2527	CaSNP4571	TAACTGTTATCGTTGCTTTGCAGTTTGAGGGCTCTACAAGCAATGTGATCGAGAAACCAA[C/G]AGTAACCGTTTTCATAAATAACAACTTAACCGATGGCTTACAGCTCGGTGTTAAATGTAA
2528	CaSNP4572	TTTGATATATCAGATTATGCAATGAGATGTTTCAGTTGTTTGACTTGGTAATGATAATAC[T/C]TTTATGTTTTAAAAGTTTCAATATGTTAAAATATATTTTATGTAACTTTTAATTACTGAT
2529	CaSNP4573	TATGTCTCAGCCCTGGCAAATAACTGTGAGATATAAGACCCATAGATACGGAGCACATAG[T/C]TCATCCTGGGTAAATAATTGTGGAACACATGGCTTATCTCATACATATGAATCATCCTAG
2530	CaSNP4574	CTTAACCAAGATGAAAATAATCAACATTTCAACAATAGTGTATATACTTCAACAAATCTT[A/C]TGTCTCAACCTCCTTATGTGACCACTGATCAACCATTTCCATTACCTTTTTCTCCAAATT
2531	CaSNP4575	AAAGTGCATCAGCTACCACATTATTTACTTGTGTTTGATGACATATGGAAATTATTCTAA[A/G]AATTCAACTCACTTTGCATGTCTCTTATTTAGCTTACATTTATCTTTTAGATACTTTAAG
2532	CaSNP4577	CTAATTCCACTACAAAATCAAACATGCAAGTGATTTTGACAAAGTTACACTTTTTAGTTT[T/C]AACAAAATCTCGATTAAATCGTGATTTGGCCAAATTCATTGATAGGAAAATTCAAGGAAC
2533	CaSNP4578	ACTTACAGACCTTGTCTTCTTGTCCATGAATTACGAAACCTTCAGGTTTTTCTATATAAA[T/C]TTCTTCCTCCAAGTCACCATTTAAAAAGGTTGTTTTAACATCCATCTGGTGTATCACCAG
2534	CaSNP4579	ATAGAATCTTATGTTGTGTTGAGAAGACTTGACTTGTAGGGTCAAATGGGTAGTTTGCCT[T/C]TAACAATCAGCAGGTTTCAGGTTGTGTAGCGAAGACTTGACTTGTAGGATCATGGTGTGG
2535	CaSNP4580	AATGAGATAAGAAAGACAAAAATTCAATTATTTTACCAGGATGGAACACTTATAAATATA[C/G]TCCTTGAAGATGAAACATTTTAAAGAGTTTTCATCTATTTGGTGCAACTTTTAAATAATC
2536	CaSNP4581	CGTGATGAACTAACTTGTACTTTGTGATTATGTCAGATGTCTGATTTGAGTGCATGCTTT[T/C]CAGTCACGTTCTTTGATCAACTCTTGGTATAAAATCTCCATCTGTAATGATGCGCCACAT
2537	CaSNP4582	GTTATTGACAAAAAGTAAATAAGCTTTAGAAACATATTGCGTCGGTTTTATTATACAAAA[T/C]GTACACCTTCTTGATCATTAGTACTTAAAATGTTGGTATAAGGTGTCACTTCATCCCACG
2538	CaSNP4583	TCTGACATAATTATCTAATATGTTGACGTGGAGAGAAGGAGAGTTAATAGGAAAATTATG[T/C]CCAATAATTGAGGCCTGACACGCCATAGCATGTCAAGGAGTAGTTAATAGGACAAGTGTA
2539	CaSNP4585	TAAAAATTGGGGTGTTACACTGTGGTATCAGAGCCACGATTTATAGTTCTTTGGGGGGCT[A/G]TGATTTGGGTATCCTAAGCTGATCAAGAGTCGGGTTTAGGGGTATCAAGTAACTGGAGTC
2540	CaSNP4586	TGGTGTTGTATGGTTTCTTACAGTTCTCACACGATGGACGACTCTTCCTTGGCATAGGAC[A/G]AGCCTGAGGTCGTTTTGCTGTCGTCGTTGAAATTTCTGCAACAGAGACAAAATTAGATTC
2541	CaSNP4587	AGTGAGGCTTTAACACTACATCTCCCTCTAAAATCACAAGGTAATGAATATATGAGCCAT[A/C]ACTCTAATACATCCAACATTTCATCAATTCTTTGTTGATGTGTGACTAACTATTCGCTCT
2542	CaSNP4588	GTAATTTTATAATTAAAAAATTGGTAAAATGAATTTTAGTATCCTACACGTATTAATCTA[T/C]ACGACGCATTCTGCACATACTAATGAATACCATTTTGTTAGTTAATTCATGTAATACGAA
2543	CaSNP4589	CCATGGAATTTGAATCTAATTGTCATGTCATCCCTACATGCAAGTGAGACCTGGATTACC[C/G]TAAACTTCCAAAAATTATATTTATTACCCAATATGTTTTGAACTTTAACCATCCTGAGTT
2544	CaSNP4590	TAAAGATAAAATAAAATGTTATAATACTAAATAAATATTAAAAATTTTAGTGTAAAAATA[T/C]AAGTACTCTATTTCATTCATGCACAGGAGTAGATAAGGTTTTTTTTGTCATTTATTTATT
2545	CaSNP4591	AAATTCATTTGAGTACTCACACGATGTTTATGCTTTGTACTTAGTGGACGAAGAATATTT[T/C]TATTCTTTGATGTATTATTGGTTTAGTGTTCAAACATATACAATAAGTTCTTTTCAAATG
2546	CaSNP4592	AGTAGTGGACAACTCCTATCTTGGCACTTCTGATGAGGTTTGTTTGTTTGAATCACATAT[C/G]TTGTATTCACTGCATTAATCATGGTTATCTCTTCATATTAAAACTTGGCAAGTTAAAGTT

2547	CaSNP4593	TACATCCCGTCCCTACACTTAGAAGGCTTTAACTACTAATTTAAATGCTTGAATTACACA[T/G]CACATGACCCTACACATCAATCTTCTCAAACCAACCTAACACCTACATACTTTTAAGGAA
2548	CaSNP4594	TTATGTCAATGGTTAAAGTAGAGGATAACTACCATAAGAGAATAACAGACGATTTGACTT[A/C]TGTCTATTGAGAGACAGTGAATGTTGAAGTGATGGCTTTTCTAATAGTGATCACTATTAG
2549	CaSNP4595	TAAATATAACTAAGAATGTGGCCTAGCTTAACGTTGGTAAATTCACTTAGAGGAAATATC[A/T]ACAAAATAAATTTCATCACCAAAGCTTCATTGGTAGTAGCTCTGAAAAACATAAAACAAT
2550	CaSNP4596	AGCGATTGCACTCTAAAAAATCGTGATGATATGTGAGCAATTGCATGCTAGTAAATCATG[T/C]TGATCTGTGAGAGATTGCACCTTGGTAATAAGTACTGGTATGTGAGAGGTTGTACAATTA
2551	CaSNP4597	CATTATGACCACAAACAACAAAAAATCATCATTCATATGAAAAATAAGGGTAAGTCAACG[T/C]TAATATAATAATGTCTAAAAAAATGTACCACACAGTAAATCAGCAGAGAAGTTGTATGCA
2552	CaSNP4599	TGATGAGCATGGTATCGGGAATGCAGCTGGGACAAGTTCAGTCTTCCAATCCTTCAATGA[T/C]GGACCGCCAATGCTAAATCGACAGTTCACTTTTCCTGGAGACTTGGATACAAATGATGAT
2553	CaSNP4600	TTCAAAAGGCAAAACTAATTAATCCCTATCTCATCGTTACTTGTGTAGTATTTTATTTTA[A/G]TTTATCTTTTATCGCAATACACAAAGAAGTAAATGAAGAAATAATGATCATTAAAATGAT
2554	CaSNP4601	TGTGCCAAAATAAATTATGTGCTATATTATTTTATCAACTTAATTTTAATTTGTTAACAA[T/C]TCTCTCCTATTTTTCAGATAAATTAGCTACTTAATTTTATTTTGTTAATAATTTTCTCTT
2555	CaSNP4602	AAAAGTTTTGAACAAGATCTTTTTTGAAAATATGATTCTTAGCCAATTCTTTATTTTTGA[A/G]TCCAATTTGAGTAGTTATTATAGATTGATTATGTAACAGACCGACTATTATCGTTATCGA
2556	CaSNP4603	TGATTTATAGTTATATATATTCTAATTACTCATGAATATCAATGTTTATCATTGTTCTTA[A/G]TGCTTGTTCTTAATGTTTATCTATTGAATCTAAACTCTGGGAATAGATTTAGGTATAATA
2557	CaSNP4604	TCCAAATTACCTTTGGTTATGTTCTTTTGTTCCTTTTTTATCCATGCATCTTTAGGTACG[T/C]ATACTATATATATACTCTTTTAAATATTTTCCAATGTCTAGTTACTAATGCAATTTTAAA
2558	CaSNP4605	CAACAAAAACCCTCCTCAAAGGGGCGTAAATCATAAGTGTACCGCCTATCACATGCCTTA[A/G]CACTAATTACCGAGGTGCAGTCTCTACATGCTATCACGATTTACTAGAGTGTAGCCTATC
2559	CaSNP4606	TTAAGGAGATATCAAATCGAGCTAAGAAAAATTGTGCTTCTTCTAAAAGAGGAGCTCTCC[A/G]TACTGGTGGATCTATATCCATTGCTGAACATACCTTTCAGATGGTACACTTTCTATATCA
2560	CaSNP4607	GTGATGCAAGTTTGCATGCATATGGAACATATTAATATTAATGGTTATTTATGGGATTTG[T/G]TGAACAGGGTAAAGTTATAAGTGCAACTATAAACAAAAATAAGGAGAAAACCCTAGCAGC
2561	CaSNP4608	TCCAAGATGGCTATTCGGGATATAATCAAATAGTTGTGGCACTCGAGGATCAAGAGAAAA[T/C]TGCACTCACATGTCTGTATGGGGTGTTTGCTTATCAGCGAATGCCGTTTGGGCTGTGCAA
2562	CaSNP4609	GAGATTGTGGAAAAGTATAACATCTTCAATAGGGTGTTGATGATTTCTTTGGAGCTTGGA[A/C]GTTCTAATCATTGAAAGATCGAAGAAGATATCATATACAAAGATCGAAGAAGATAGCATA
2563	CaSNP4610	CTATAAAGTAGCAAATTATTCTAACATAATAATTCAAAGAAGTGCATTGTGTAACATTCC[A/G]TTTTTTCGATACATTAACTAATGAAATTTTAAACGTAAAAATACATATTCGTTTTTTATT
2564	CaSNP4611	CAGATGACCAAACCTCTGGTGCCACAACCACTATTCCCTCCTGATGTCTGCAGCAAGACA[A/C]TGATTTTCATTGATCTGGATCTCAATCTTGAAGGTTATGTTCTTGGACAGTGGTGCCTTC
2565	CaSNP4613	TCCGTTGTTGTCCAATATTCACGACGTGTTTCACGTGTCGCCACTAAGAAAATACATTTC[A/G]GATCCGCCACATGTGATCAAACCAGACACCATTCAACTAAAGGATAACTTGTCATTCGAA
2566	CaSNP4615	CCCCTGACGGGACGGGGAGAAGTAGGAGGTTGGATAATGTAGATAACCTATCTATAGAGA[T/C]CAAAGATGGTAATGACTCATTTCATTTATGTTTTGATGTGTACCGCCATAAAAGCCCTCC
2567	CaSNP4616	ACATAACAATACAAAATAAAACATTTAAGGAACTTGACAACTTTCTATCTTGTACTATAT[T/C]TGGTGTATATGGATAACATAAATAAGATAAAATAAAATTTATTATATTGTAAATTTTAAT
2568	CaSNP4617	GGAACACACAAAATAATAATGTAAGAAACTTCAAGATCGGATATCCAACTCCAAATGGCA[A/G]CCAAAATTTGTCAAAAAAATTCATCATAGTTCCCACTTCAAAGCACTACTTTCAACCATG
2569	CaSNP4618	TGGTCAAGACTTATACCAGATGGGAAAGGGCCGATAAATCCTAAGGGATTGCAATATTAC[A/G]ACAACATTATCAATGAACTAACAAACCAAGGTAAGCCAAGGTTCTTTTGTAAACCAGAAG
2570	CaSNP4619	CATTGATGATAATTGGAGTGTGGTTTGGAAAGATGAGATTCAACATTGGAATGAACGTCA[T/G]AATTACATATTGCAATGTCAGTACGTCGAAGGTGTTTTATACCACACTAACGAATATATG
2571	CaSNP4620	GGTTTTATAGTTTACAAAATTGGAACATTAAATGATGAGTAGAATTTCTTTAGAAACTAA[C/G]AAGTGATTTTGAGTGACTAAACCAATAACTCGTAAATTGTATTATTGCTTTTGGTTTATA
2572	CaSNP4621	GTTTTAGCTACAATCCTCGGGTTGATGTTTTGATGATAAAAAAAACAAACAGATAAAGAA[T/C]AACTATCCCTAACTGTATTTGTTGAGGGTGCATATGGGTTATATTAATTATTATCCATTC
2573	CaSNP4622	TCTAAGCACTTAAAGAAAAAGCAACGGAGAAAAAGAAAACCTGTTTGATGATTCCTAGAA[A/G]GAGCTAGGTTTTGCAAAGCTTCATCCAAATAAAGTTTCAACTTTTTTAGGGCATCCCTTC
2574	CaSNP4624	ATTACCTTCTTTTTGTCCTTTTCCTTTAGCATCCATTTGTTTCCAGCTCCATATATTATC[T/C]TTAATCATCACATTACTCCCCTCATTAACAAGAACCTTGTCATCAAGGTTAGGATAATTA
2575	CaSNP4625	GACTTACCTTTGTTGAGCTGAATGATAGACTTGGAGAAGTAATAAAGATGTATGTATCAG[T/C]TGAATCTTGTGGGAGAGAAGGGTTTAGAGAAAACTCGAGGAGCAAGGGTTTTATAACTAA
2576	CaSNP4626	TGAGCTGTACGAGGTACAAAGATGACAAGTACGATGATGGATCTATAGTCAACATCATTC[T/G]CAAAAGTTGAAAATGACCTAGCACTTCAGCTTACAGAAAGGTTGTAGCCCACAAAGTCGA

2577	CaSNP4627	TACTTGTATTCCTAAGAAAAATAATTGTTGGATTGCTGAGAAGACCAAATTGATGTTGAA[A/G]ACTAGTGAGACTAGTAATGAATACATGTTGAAGAGCATGTTGAGGCTACATTAAACAATA
2578	CaSNP4628	TGCGATATCATACCAACCAAAATCGGATGTGCGGTCGCAAGCCAGACAACACATGGAAGA[T/G]GTTGGCCAGTCGGCATTGGTGAATAAGGGGAATGGAATGGAACCCAATCATCCGGCGGCA
2579	CaSNP4629	ACACTTGTGGGATTCATCCTTACTTAAGAATTTTACCCATGTCCACCTAGTGTAATAAAC[A/T]ACTATAACAAAACCATATTTATTTTCACTTAGAGATGTTGTTTTCACTGGCCCAAAGAGA
2580	CaSNP4630	CAATGAGTGACATCTAGCAACCTATCACTGTTACCCAATTAATGAGAAAATCGGTGATAC[T/G]ATTTTACCAATGTTGACTGTGTAATAATTGTCGCTTCATCCAAAGATCTTAATTGAACTC
2581	CaSNP4631	CTACTTTGTACATTCTTAAAAATGCAATCGAGATGAAAGTAATATAATTGAGGATTATGC[T/C]GATAGAATAATAATGATGGAGATGATTTTAAATATGTGGAGTTTAATTTTTGGAAAATAA
2582	CaSNP4632	TCATACATAGAAGTGCAACATCTATGAAGTTAATACAAAATAAAAATTATGTGTATTTGA[A/C]TAGAAGACGAGATGATTCAAATGTTCATATAGACATATTTTGGACCCATCATGATTGTAT
2583	CaSNP4633	GATGGTAGTGAATAAGAATAGTAATTTATGTGAGAACCAAATATAGATGCTATATTGAGA[T/C]ATATCTACGATAAATTATATCAACAACCACTTTAAAATCAGATTCAATAATATTCATTGA
2584	CaSNP4634	TCACCGTCATCTTCGCAGAATAATAATAATAATAATAAGTCTATTGCTATCAACACCCCC[T/C]TAGATAATAATAATTTTGACAAGGTTTTGAAAACTGATCAAGGGTCTTCTCAGGATAGTT
2585	CaSNP4635	ATGAGGCTTGATACTATGCCACCTTGGAGTGCTACCTCTAAGTCACCTAGGAATTCCCCA[T/C]CCGAATACCTCCAAGGTCTAACAATCTTACTTTAAGGCTCAACAACAGACCGCGCATGAT
2586	CaSNP4636	ACTTAAATGAAATATCAGTGTAAGATCTATCATTCATGCATGCCATGTATTATAAGCAAG[A/C]TTTTGTTCATGTCTTTATGTTTCGTAGGATTTTAGACGTCTCAATACTCCATGAGGAACA
2587	CaSNP4637	CTATTGCCGTTACAAGTTGAGGCCTATATCTTCTTTTCAATATAAGCTTAAATGGCTGTT[C/G]TGGACTTGTTTTTGTTGAAGTAGAAGAATTTGCTTTAGTGAGATCATCGAGTTCGGATTG
2588	CaSNP4638	GCACTTGAACCTATTCTCACTTCACTCGTGTTTATCACTCCGGGATCGAAAAATCTCGCT[A/C]TGTATACCAATTGTTAGCATTCAAGCTATTATTTCAAGTGTAAGAATGATGAAACATTGA
2589	CaSNP4639	TCCACACCCGCCAATCCGGTTCACCCGACTTAACCTGCCCGAATGACTCTTTTGGGCCTC[A/G]GATCGGTCGGTTTTGGGCGGGCCTGTGATTCTTGTCCACCCCTAGGTGCAACTAACAAAA
2590	CaSNP4640	ATGTTCTATTATGTTAATTAGTTTCGCATGCCAATCTCTCTTCGTACTATACATAACACT[A/G]CTCTAAACATAAACGTAAATACTAACCAACCAAAGACCAAAATAACATTAGCAACTAATA
2591	CaSNP4641	GGGATTAGATCTTCTTGCAAAGGAAGTGGATGGTTTTTTCCAGGTGGTCATGTCTGGTCG[T/C]GATGCCTTGCTATCTAGTCTGAGGTCAGGTGCAACTGTCAATGACCGCATCTTGGGGGGT
2592	CaSNP4642	CTCTTTTGTAATAATTGAAGAAAAACAACACATTAAACTAGGAATATAAAATTACTCCCA[A/G]TAACCTTGTAATATCTACAAGTCTCTACAATAGTAATTTAAAAATCAAATGACATAATTT
2593	CaSNP4643	ACAATAAAGATTCAATAACACTTATCCCCGCCTTTCAGAGGTGCCACAATTTTGTAACTA[A/C]TCTCAATGAAGAATTTTTCTAAAATCTTCAACAACAACACAACAATAACATACATAAGAA
2594	CaSNP4645	ATATAGATGCGGCGGGTGTGAGCGGAAAATCCGAAACATGTTGTAGGCAGGAATAAATGT[T/C]TAAATGTTACACCCTCTATGAAACAGAGCGAATGCGGGTCTACATGCCTACTTTAAAGAG
2595	CaSNP4646	ATGTTAATCCAATGACACAAACGTACCTATTGGAGGCACAATATTATATAAAAACCCTAA[A/T]TGGACTCGAATAAACCTAGGGAGGACGAAAATGACCTAAATGTTAATGTAATGACACAAA
2596	CaSNP4647	AGAATGTAAACGCAAATATCAAGGAAACAATTTTAAGTTGGAGGTAGATAAAAGGGAAAT[T/G]AAGGGGATGGGAAAAATCAACGTTCCTATTTAATTTGGTCACTCCCTATTTCTTTGTGAT
2597	CaSNP4648	TTAAAGTTGTTACCATAAGACTTATTTATTCACGTGTTTGTGTGTTATATATTTGGGATA[A/G]AAAAATTATTGATTCTAGATTATAAGCATCCATGAGAATAAAACCTTATGTCATATGTGA
2598	CaSNP4649	GCTTAAAGGGCTTAGAGAGTCTAATCGAGCTACATTTATGGTGACAAACAGGTACTGATG[A/C]TTTCTCTATATGAGAACAAGATGAAAACAGATCTTGCTTAAGTAGGCCACACTGTCATTC
2599	CaSNP4650	ATTTCCACCTTTTTAACACTGTCTAAGCGCGCCCAGAGCGCAAATCCTACGCCATGGATA[T/G]AAACTCAAAGACGGCCATTTCTAACACCATTGGAGTGTAACAAGATGAAACCCTTATTTT
2600	CaSNP4651	TTGCAGTTATCAAAGAATTGATTATCGAGTACCAGTCCCATTGCGAAGTTACTGTGATGC[T/C]TACTCAGTCAATAAGTAAGTCATTTTGTTCAATTTATCTATTGATTTACAGAGCAAGGTA
2601	CaSNP4652	TTACAAGACTACAACCAAAATGTTGTATATTTGCTGAAATCAAATACATATTTAAACCTT[T/C]AATAATTGATATTTGATAATATAGGATGTCGTTCTACTATTGGTACACAAATATTATTTT
2602	CaSNP4653	AGTCATCTCATTCTCTCTGTAGAGCATTTTCTATCTCAGGAAAGGTGAAACTAGAGGAGG[T/C]TGTTCAAGCTACACTCTCTACTTTGCCAGATGAGGTTGTGGATACTGTTGGTGTGGCAGC
2603	CaSNP4655	ACATATGGATATGGTGGGAAAAAGAATGTTGTAGATTTTGAGATGTTTCCATAGCCTCTA[T/C]AGCTTGAGGTACTATAGGAGGAGAGAAATGATGATGAGTAAGATTTAAATCATAATATAG
2604	CaSNP4656	TAGCACAATGGAGACTCTTGACCGTTCAGCTAGGAAAGATTTGACCAATGTTAATTCCTT[C/G]TCAAACTCTTGCCCTAACCTTAAAATTTCAAATTTTGACTCTGACAATTCATTCGAAGGA
2605	CaSNP4657	AGTGATTATAACACTTAAAATAGATGCAAATGTAAATACAATTAATAATTTCCATTGTCA[A/G]GTTGACCAGACTTAATTCAATTAATTTGGATATACCTTGCTGGTGTAACCCCTTACATCA
2606	CaSNP4659	TATAAAGGTTTCTAAAACACAATGAAAAAGCTCATGCAGATGTCTTCAAAACATGGTCAT[A/T]AAGGAGAACAGAAGTTACAAATCTAAGAATCAACAAGCATAAAGGCATTATAGAGAACAT

2607	CaSNP4660	TAATTTATGTTATCTTAACATAGATAATCACCAACATAAATCTTTGTCTTCTAACTTAAT[T/C]TGCACACTTAGCAAATATTGTTATGGGATAATTGTTCTAATTTGTTTATTTTGTTATCAT
2608	CaSNP4661	TAAAATATCTTTTTTTGGTCTTTTAACTTTAGGACCTTTTATATATTGATAAACCCCTTA[A/G]TTTTGACCAAGTTAAAGCTACCATGAACTAATATTTTTTATCATTCAATACTATGTAGGA
2609	CaSNP4662	ACTTCAGTTCTCCCATCATACTCATTTGAAATTGCATCAACTTAGAAAATTATTGGCATA[A/G]GGTGCCGTTAGTTGAACCAGAAAATAATATCATCTACATATATCTAAATTACGATAAGGT
2610	CaSNP4663	TATCGATTCCGTAACATGCTGTGCTGGATTTTGAAAGTTGAATTACAAGAGTAAATTAAA[A/G]TAACATGTCATACAACACCTTCAATACATGCTCCGGACCAATAAATTTAAAAGAAAAATT
2611	CaSNP4664	ATTGATCTTCACATGTTAAGAATGCATCAATGTAGATTAGTGAGAATGTGTATTGAGGTT[A/G]ATTTAATCTAACTGATTATTGGTAGGGTAGGAATCAATGGTGAATGGTATCAAGTTCAGT
2612	CaSNP4665	ATCATGTGATAATTACTATCTATTCATATTCATAACTTTTCTAGTTGCGACTCTTTTTCA[T/C]AGAGCACAGTTTCTTTCTTGTCCAATCAGTTACTCATCTTCTCCAAAGCCTCAGGTAATA
2613	CaSNP4666	AGGACTTAGATTAGTGGTTAGTTAATAATAATAGTTAGATAATGAGTCAGTTAGTTGAGG[T/G]TGTTATAAGGCCTACTTGTATAAATAGGGAGGTTAGGGAGTTAGAGATAGATCATTGAAT
2614	CaSNP4667	AGAAAGAAGCTATCATGTGTCCAAATTCAAATTCAAATACTAATGAGGTAATTGTTTATG[A/T]ACCCTGTTCTTTATGTAAAGTTGTGATAGCTGAATTAGATAGCCTCTTAAATGACTCAAA
2615	CaSNP4668	AAGCAGAAAGACTTCAGAAGAAAAGCTTGATGAATACTTGGGATGACTCTGATGACTAGT[T/G]TTCACTCTGACTCTGATGATGAATCGATAGACGAAGAAATAGTTATTTTTGTTATCATAT
2616	CaSNP4669	AATCATATTTGTAACTAAATGATTATAACCACGTAAAGTCAACAAAATTACAACAAACTC[T/C]TTTATTTTTTAACCTAGCCGCATAACATCTCTTCTCTTACATTGCTTTCTTTTCATTCAT
2617	CaSNP4670	CTCCATTTTCATACTCTTTTACAATCGATTGGCAGACATCCATTTCCTATCCATATTATA[T/C]GACCTATATAAATGTAGTTACAAAAATAATAAGCTTTCAAAACATATCAACAAGTTTCAA
2618	CaSNP4671	TAAAAGTGTTGCTGTTTCACCACGAAAAAAAACCATTGATTAATAAAATTTAATATCTAC[A/G]CATTTTAAGTGTTGTCTAAATCGATAAAAACTGTTGCATGATATTAACTCAAAATATGTC
2619	CaSNP4672	TTTGATATGATAGGAAATGTCGGTGCTATATAGCTTAATAGAAGCTAATGTAACTGTGCT[A/T]TCTAAAGATGCAGTACTACAAAGTCCATTAAGAAAAGCCCTCTCCCCCATCAACATCAAT
2620	CaSNP4673	TACTTCGCTTGTTACCCGTCATAAATATGCACATCCCCAATCAAGCACTGGGTTGGCCAG[A/G]CCAAATATAATAGCATACAAAATGCATGACTATAACTAGCAATCCTATAAGTCCAAGATT
2621	CaSNP4674	GTCCAGTTCTCTTATGCAATATCCATTTGTCTTTATTTATGGGCATGAGGCAAGATTCAA[T/C]TGGTAGTTCTTCAATGTATAAAATGTAAAGATTTTTCTTTTTGGATGCAGAAAACATTAT
2622	CaSNP4675	TAGATTGCTAGGTCTTCACCTTCTTTATCCACTACCTCACAATCCATCACACTACCAAAA[T/C]CCTGTCTATCCTTCTTGGACGACACTAACCCACTTGAATGGATTTACCAAGTTGAGAGCT
2623	CaSNP4676	ACATGTACATGCATATATATATATACATACATATATAGAGTATATACATTTGGACTTTTC[A/C]AAAGCTCCATTTATGTTTGAGGTTGAGTGGAACTTTGTAATATGTGATATTTTCATTATT
2624	CaSNP4677	TTCATGAAGGTTTCGACGAGAGGAATTAAAAAATGATACCAGTTTTTTGAGACGACAAAC[A/G]TGGTGATCGGAATCGGCAAAAAATAGTCATATTGTGTTGACTTCTTCTTGTAGAGTCATA
2625	CaSNP4678	AATTGATGAAAACTGACTTTGGCATTCAAGATTTGGCCATCTCAACTTCAGAAGCTTGTA[T/G]TACCTAGGAGTCAAGAAGATGGTGAGTGGAATTCCTATGATTGATGTCCCTAAGAAGGTG
2626	CaSNP4679	GGCTATATGTTTATGATAAATAAGTCACAAGTTTCATGGGGCTCAAAGAAGTAAAGTATA[A/G]TAGCTTTATCTCATGCAAGGTTGAATATGAAGTAGCATCAATGGGAGCATGTCAAGCCAT
2627	CaSNP4680	GATTTTAATCACCATTCAATCTTTCTTACCCTTCTTACTTCTCATAGAATAAAGAACCTC[A/G]TGTGCAACAACCTATGACGATTAGGCACAAAACTACTCTGACAAAGGCTTACCAGATTCT
2628	CaSNP4681	TGAAAGTAGTTATTTTTGCTTATAATAGTGATGGGAAGGAGTATTTTTGGGGACTTGTGT[A/G]ATGTAGTGATACTGGTTTTCTATCATGATGCTATTCATTTAGCAACATTGTTTTTCTATC
2629	CaSNP4682	TGTCCAAGGATAATGTTCTTCCTAATCGAACATACAATGCCAAGTGAATGTTGTGCTCTA[T/G]TGGCATGAGTTATGATATGATTCATGATTGTCTTAACGATTGCATTTTGTTTTGAAATGA
2630	CaSNP4683	AAGGAAAAAGGTTTTATAATATTCAAGTCCATATGCAATTGTATTGTGGAAGGTATGCAA[A/G]AGAAAGAAAAGTACCAGAAATGAGTAACTGAGTATCCTCTGAAGAAAATCTGTGTTTGAT
2631	CaSNP4684	CTGGCATGTACTACATGATGTTTGACTTAGGCATCCATAATACTGAAGCCCCAATCATCT[T/G]GCAATTTTGTCATATATTTTATCTTCCTTTGTGTAACTATTTTCACATCAAAATAATCAC
2632	CaSNP4685	GCATGCGCACATACATATCGTACCGTCAATTGTTGCAAAGCAACAGGACATTCTGATAGG[T/C]ACTTTGTAGCTAGAGTCATAAGAACTGGAACTGAGTCCTCTCCAGGAATCATCATATCAA
2633	CaSNP4686	AATGTCAACCTTTGTGGCTCCTCCTAATGAACCTAGTAATGCTCTGCTTGATACACCTCC[A/G]CAACAAGTGGTAATGTCTACATCTTTCATCAAACAACATATTAATATGGGGATATATGAC
2634	CaSNP4687	ACAGAGGGAAGTCTCACCATAGTATGAATTTGGATGATCTTCTAAAAGATTTGATCACAG[T/C]TGAAACTAGTCAACTTATGCAAAACCCCTCATCTGTTATTATTAATAATAATAATAATTC
2635	CaSNP4688	GCTTGAAGCAAGCTCCTAGTGCTTAGTACAAGACGATTGACAAGTTTCTTCTAGAGCAGC[A/C]GTTTGAGAAATGTGTGAATGAGTATGGAGTTTATGTTAAGAATGCAACAACATCACCTAC
2636	CaSNP4689	CATGCTTGTTAAAGTAAAGAACCACAACTTCCAACACAATCCATTAGCAAGGAAACTCTA[T/C]GAGTTGGCAAAAATCATTGACCTTGATTTCAATTTTTTAATATATAAAATCATTTGAATC

2637	CaSNP4690	TGTTCAAAAATATGATGATGAGGACAATTAGACACTAACTTCTTGAATCTCTCCCAATAC[A/T]CATGTAATGATTCCCTATCAATCTCCCTAATGCCACATATTTCTTTTCAAATTGAAGCAA
2638	CaSNP4691	CCTTCCAATTCCAAACCACATTCAGGTATGCCATTCTTCCTCTGTTTCACGTGGTTCGGG[A/C]AATCATAATAATAGCTGAAAACTTAGCGATTGCATTTGGCTAGTACCTTTTCCTATTCAA
2639	CaSNP4692	ACTATGTCACCATTCCGATGGACAGACAATTCAAAATGGCCCTATCATCATAGATCCCCT[T/C]AACTCAACCCGAGACACATATATGCGACACTCGAGGTAAATGTGTGTTGCATGGTAGTTC
2640	CaSNP4693	ATGTTAACTTAAGTCGAAGTTATAATCGTTCAGAAGTTCCACCGATGTACATTGCCTTGT[C/G]CTCAACTCCTTAAGACTGAACAAAAATCGTAGACTTTGATGATCGTTGAACACTTCAAAT
2641	CaSNP4694	AGTTAAATACGGTTACTTGAAGTTCTTATGTGTTTGGCTCTTGAAAAGAGGATTTATATT[C/G]TTGGTATACATAATATCAATTAATTTTTATAAGCCTTTATTCTATTATACAATTCCGTAT
2642	CaSNP4695	AGAAACAATCCCCTAGAATTTCAACCAAGAGAAGCTTCTCACTTGTTGCTAATTTATCTC[T/C]TTCTTCTAAACCCGCCAACGATGGAGTCTCATTATAAAGATATTAATATTGTTGCACATA
2643	CaSNP4696	TCTAGTGATTGTAAAAGACCTACTGACCTCTGGTGTTCTATCTTTACCTTTTGACAAGTC[A/G]GGCCTTATGCTACAAAGGTGGTCACATCATTCTTCATGCTTGGCCAACAATTGTTTTTCT
2644	CaSNP4697	GTTGTCTTGCAGGACGACTCTCGTCGTCGTCTCTTTATAGCCTATCACCCTCATTTCTTT[A/G]CAGAAGGTTGTGTCTTAGCCTCTCACCGCTGTCTCTTTATGTATCGCAATGATCTGCCTC
2645	CaSNP4698	GCCATAGAACTTTAGTTCGTACCTCAATAGGGGCAACTCATTTCTCATTGGTGTATGGAT[T/G]GGAAGCGGTACTTCCTATTGATGTGGAAATTCCCTCGATCAGAGTCTTAATGGAAACAAA
2646	CaSNP4699	AGGAAGAAGTAGAAGAGAATGACAAGAGGGATTTGTATGCAAATTTGATGAGGAACATGT[A/T]GTTAAATGCTTTGGTGATGTAGAAAAAACGAAATTCAAAGAAAAAAGTTATGATGAAATA
2647	CaSNP4700	TTAAATGTAGATTGTAATGCCCAATCACAATATGGAACGGAATGGCGAGTAAAATATGAT[A/G]TGATTTACCTATAACATTATATATCTGGGAAAATGGAGTTAAGTCCCCAAATGTAAAACA
2648	CaSNP4701	TCTTATGATGATTATCTTTTGCATTTGAGAAGTGTTTTGGAAGCTCTTTGGCATGAGAAA[T/C]TGTATACAAGCATGGAAAAATGTGTTTTATATATAAATCATGTAATTTTTCTTGGTTTTG
2649	CaSNP4702	GCTTTATCATCAACTGTCCAATCTGCTTCTTTCTTCGCACCAGAGATTGACTCATTTTGA[A/T]CAACTCTTGGTATGAAATCTCCAAATGTAAAAATTCACCACATCCCTGTATCTTGAGATT
2650	CaSNP4703	CATAAGTCAGTAGCCAAATCAGGACATATGTATTCGACTGCAATAGCAGAAAAATGTAGA[A/C]TTCAGTTTTGAAACGTTTCGAAAAGGTTTCGCAATCAATCAACACTTACACACAAGTCCA
2651	CaSNP4704	CGACCTAATCAGTTAATCTAGCTATTTTAGACTATTTTACCTTCAGATATTTGTATATTT[T/C]CATATTGGTAAGTTTTAGTTTCCCACAAATTCATAAACACTCATAACTTTGTCAATTCTA
2652	CaSNP4705	AATGAAGGAAGATGTTTGCTAAAAAATACTTTGTATAATAACATCAAGATCACCAAAGTT[A/C]ATGTTCTTAATCATATCCTTTTTGATCCTCAAGGTGAGAAAATTTCTTTCAATCTTCAAC
2653	CaSNP4706	CTACTAATTTATTTTTATGCATGTTAATTTTTATTTCTATTTTATTGTGTTTATTTTATT[A/T]TAATGAAATCAAAACTTTAAGCTTATGTAATATTATTAAATATATAACATTGGAAATAAG
2654	CaSNP4707	AAATACAAATATCGGTCCATTTTAGTCAGGTGGTACGAAAATCGCTCGAAAAGTCCAGCT[A/G]AAATCTAGGACCGAGAAACCGTTATAGCATTCTTCTTCTAAATTTATAACCTTGTTTCGG
2655	CaSNP4708	CCAATGGTGTAGCCGAGTGATATGGGTTGGGACGTTGAAAGAGTATTAAGGAGGTCTAGG[A/G]TTCGATCCCTGCTGCTAACACCTTCCCCCTCCTCTTGGCAAACTAACATTTGTCTATAGA
2656	CaSNP4709	CAAATATTCCTAACATTATTATGATATAAGGTAACTAAACCAATTAATTTGAAATTTAC[A/C]TTAAAAAGCTTGCATATGATGATGAGTTGTCATTACTTCATTCAACTCACAATGGAATGC
2657	CaSNP4710	TCAATGCCAGTATTGTCTATGAACGAAACATAGATGGTATGTCTGAATAGATGGTATGTC[A/T]GAATTTCAATATTACAAAAATACTTTCCTAACTAAATGAAGATTATTCAAACAAAATTGA
2658	CaSNP4711	TGATCTTAACGGAAAGAGTAGCCTAGGTCAATGAGAGGAGGAACTTAAGGTTAGAGGTAA[T/C]TGCCAGAGAGATCTTCTTGTCATCATATAGGCATGGTCTAATAAGCTTGGTGATGTTGGA
2659	CaSNP4712	ATTATGAGTCTGAAATGATAGCACTAGCTACTACTAGGAAGAAGCAAGTTGGTTGAAAAA[T/C]GTGTTAAATGGGAAAAACCTTTGCCAATTGTCTTGATCCACTGCGATAGTACCGCGACTA
2660	CaSNP4713	TATGTGGATAATTGGTTCCATTTACAAACTATAATATTTATTGTGCTTCTAAATTTATTT[A/G]TTAAAGCATAAAGGTTACTTCAACTTTCTAACTCTGAATTGAATTCTTAAACTTAAAGCA
2661	CaSNP4714	CCACCTCAAAATCTCAAAGATACATTCATGTTTCTACCATAACCACTAGTAAGCCAATCT[T/C]AGAATCTTCAAATGTCATGCACTATGTCAAAAAAATTCCACCCCCTCAAAAGGTTACCCT
2662	CaSNP4715	AACTCGTCCTCTAAGTCAATATGTTTTAATCAACAAAAATCATGATTAATATATACAAAT[T/C]AGTCATTGAGAATTGCTCACTTATGCTCAATTTGGTCCCTATAGTCGTATTAAAAATTTC
2663	CaSNP4716	TTGTTGGGATGATGACGAGTCAATGCATAGACCTCTCAAAATTAGTGCCTTGTGGTGAAG[A/G]GGAGCGTTAGCATGGTGAGGTTCTTTCTTCGTTAGGTTGTCACCAAAATGGGGAGTTCTG
2664	CaSNP4717	TCTGAAAAAAGAAGATCATTCTGGACAACAGTCTGAAAATGAAGAATGTTCTAATTTTGA[T/C]AGCATTAATCCAAGAACATTATGGTTTACAAAGAACAAAGTCTTCTTTAATTCAATATCG
2665	CaSNP4718	AGTCTGGGGACGGTAGGAAGTACTAAGGTGTAGTTTAGTTCCTAGAGACTTTTGAAATGC[A/C]TTCCAAAACATGGAGGTAAACTTAGGGGCCCTATCTAATATGATACAAAGAGGCACGCCA
2666	CaSNP4719	ATTGATATAAAGGATAGAGTTTAATGGAGATGCACTTCGGTGTAAAATAGATTTGCACTG[A/C]CATCCAATGAAAATTGAGTATTTTGTAGTTACTTTTAAAATACATCGAAAGTGCATTTTC

2667	CaSNP4721	AGGTTATCAAAGCCAGGCGAACAGATGCTTGATGTCGTTATCAAAAGCAGACGAACACTT[T/G]CTTGGTGCATTCATCAGAGGTAGACGATCAAATGCATATTGTAGTTATTGGAATTAGACG
2668	CaSNP4722	ATGGTGAAATAAAGATGTTTGATAGTGCAAACAGGTTTGTATTGAAGGCACCATTGTCCA[T/G]AAACAGAATCTTCAAAACAACACTGTCAAATAGATCTTCAAGAATAATGGAGGTTGAAGG
2669	CaSNP4724	AGACCATAATCATCTTCACATTCCTCATCAACATGATTAATTGAGGATTCAAAATTACAA[A/G]AATGAATCTTAGCAATGCTACTTTAAAAGGAACCATTTTCTAATCAGCATTATTTCAATT
2670	CaSNP4725	CCGTGTCTTCATGAAAGTTGTAGCTATGAATCGTAGCTTTCATTTGCACTTGGTTTGACT[T/C]CAATTGGATATCTACAACTTCAGGTATGATTGAAATACTCTACATAGGTCATGTTGATTT
2671	CaSNP4726	CCAAGTACCAAAGTTTAGAATCTTGTGAAATATAGAAACTTCTCAACCTGTGTATTGAAT[T/C]GTTGCAAGTAATAATTAAAACGGTAATACCAAGTGTCGATCTCATGGATTGCGTTTTACT
2672	CaSNP4727	GCGGTTGTTATGTTGTAGTTCCGTCGATTTTTGAGTGGCCCTAATACCACTATCATTTTA[C/G]AGCGGTTTTTGTGCCTACAATGCAATGGAACTCTGATACAGTGAATCTGAGTTGTTTTAT
2673	CaSNP4728	TAAATCTACCCTACAATTATCTATTTGTTTAAGCCCCTTTGAGCTTAAGTATCTCATTCT[A/T]TGTTATATAGCTCATTACAAGCCTAAAAGTGAAAAACAATGAATTGACCCAATCTTGGGG
2674	CaSNP4729	ATTATTTTGGTGCTTAGTGTGTCAAAACTTGTATCTGTATTTTTTTAAACTGTGGGTATG[A/C]GACGACTACTATAGTATCCTACTCAAACCCTATCCATTGTCATCCCTATATGTGCTCTTA
2675	CaSNP4730	TGAGTTTTTAATACAACGAATTACGTTGTTTACGTGCCACCAGACATAAAAAATGAGGCA[T/G]AAGTATATTTAAGTCGGTTGACCGTATAAATAAGTTAACAAAATGAGGAAGTGGCAATTT
2676	CaSNP4731	GGCTATGGAGCATAAATGTTGGCAAGATGCTATAGAGGCTGAACTTATAGCACTAGAAGA[A/G]AATCAAACATGGGATGTTGTTCCTTGTCGCTTATATGTTAAACCTCTTGGCAGTAAATTG
2677	CaSNP4732	CAAAAAAAGGACATGAAAACAGTAAAATAATTAATGGGGAAACAATGATAGTAAAGAAAG[A/G]GAGTTACTAATTACTATTCCATGACGATGTGAAGCAGTGATGCAGGGAAAGGGTTGCAGG
2678	CaSNP4733	CCTCATAACATAAATACAATTGTTACAGCTATCAAAAGTGCTAATAACAACAACAATCCA[A/G]TTCTTGTGTGTACTTCCGGCACGACAATACCTTATTTAAAAGAAATAAGGTTGTGTTTAT
2679	CaSNP4734	AATCCGGTAGTCATAAATTCGATAGTTTTGATCGGAGCAACCATACTTTTATGGTCCGCG[A/G]GAGTGTTAATCCAAGAGAAAGACGATCAATTGGTCATTTCAGTGTAAACCTTTCAAACAA
2680	CaSNP4735	ACATAAAAAAAAGTGGTACATGGACCCATCAAAACAAAAGTCAAGCTGACAATATAAGGT[A/G]TAGGTACAGTCTTTCAAAATGAAATAATTACAACCATAAAAGAAAGATATGTATCCCCTA
2681	CaSNP4736	ATTGTAACTCTATGACACTAAGTTATTTTGTTTATTTGGTTTATGAAACCCTAATGGGTT[A/C]TTTCTCTTCATTCCTTCTATGTTTATCATTGTTAACATGGTATCAAAGAGCTTTGGTTGA
2682	CaSNP4737	GAAGATTTACGTTTGGTCTTAATGTCCATATATATACTCATATGCTTATTTGAATTCCAC[A/G]CTAGAGCATTATTATATTTGTTTTTAGTATGTTTTGATGGATGAATTGGTAAATTAATTC
2683	CaSNP4738	AGACCAATATCTCGGAGTGTACTGTGTTTCATAGGAAATGTATACATGATTTCCTCAATG[T/G]TTTCTATTTCCATGTGCAGGTTGCAAAAGACCCATCTGGCCATGAGATCAATGCACTTGA
2684	CaSNP4739	TTGGTGCAATAACAATGCACATTATTAGAGCTACACATACAACCTTCATGCTTGCCATTG[A/T]TTCTAAGTGAAATTTAATTGAAATGTGTTTTCTTGTTGTTTAGAATTTGAAGTTTTGTTG
2685	CaSNP4740	CTGTCGGTATAGGTAATTTTAGTTATCCCTACGGTTATTATACCTTTACCTACAATTTTT[T/C]ACTGTCACTAAAGGTAAAATTTCTTGTAGTGTGTTGAGATAATGTTTCTATCATCATTGC
2686	CaSNP4742	TTTTCAAATTAAAGTATTCATCATCCTCTGTAGAATTACACCTTCATTTAACAAATATAA[C/G]TCTTTCTTTTTCACCTTTTATGTAATTTGAATCGCCATATATCATCACACTCTTGGATAA
2687	CaSNP4743	TTTTTAGAATAATTTTGCAAGAATTTTGACAACTAACGAACCTTCAACTGATATTCCAAA[A/G]TTCATTTATGCAAAAATTTCCTCCATTGTGTAATACTTAATAACTGCAATATTTGTCATT
2688	CaSNP4744	GTCAAATCCCTTGGCCTTAGGCAAACTCACAATGAAGTCCATACTAATGTCTTCCCAAAT[T/G]GCATTCGGAATCGGTAATGGTTGGAGCAGCCCCTCCGGAGATGATGCTTCATACTTACTT
2689	CaSNP4745	GTTGTTCATTGCACCACGCCACCTTCCAACTCCTCTTGCACTTCCATTAATAATTCTCAA[C/G]GTAGTTTCAATTTTCATTTTGGCATATTAACCAAGATTATAAAGCTCAGTGGGGTTCAAG
2690	CaSNP4746	GTAGAGAATGTAATCATTGTTTCACACGGTCGTTTTGGCATTGGTTTCTTTCAAATTAAA[A/C]TTACAGTGAAAAAGAATTGTGCAAAATAGCGGATTCCATAAATAGAGAAGTCGATAATTT
2691	CaSNP4747	TGCATTACTAGATTTTTGGTGAGAAATCAACATGACTTATGTATAGTATTTCAACCATAT[T/C]TGGAGTTGTAGATGTCCAATTAGAGTCAAACCAAGTGCCAATGAAAGCTAAGATTCATAG
2692	CaSNP4748	AACTCAACTTCCATTGTGGAAGTAGCGATCATGGTTGTCTTTGCACTTTTCTAAGAAATA[A/G]CTATCCTAGCTAACATGAAAATGCATCTGGATGTTGATTTTCTTGAATCAATGCAACCAA
2693	CaSNP4749	TTTAACATCACCGTTCAGGTCAACTAATGAAGGAGAGTTAACAACTAATTCCAATGGATC[A/C]TTCAAACTAAACGTATCATCCACAGATAATCCCTTAGAGCTGGAACTGCCAGGCTCACAA
2694	CaSNP4750	ACGATCTAGCCGCGGGAACCAACCCATTATTTTCTTCTTCTTCAACTTCCATTTCATCTT[T/C]AAAAACACTCTCTTCGTCTTCTTCATCTTCATTTTTGTAGTCGTAGTCTTCATCACTTTC
2695	CaSNP4751	CAAGTGACTAACTAGTACTTACTAATTACGTTTATGCTAAGTAGGTAGTATGTATCTCCT[C/G]ACTTAAATTTCCTTTTTACTACTCATAATTTTGTTTATTGTGTTGTTCTTTTAACATTAT
2696	CaSNP4752	TTATATACTATTTATTGAACAATAGAGTTTTAATCACTAATTTGATATTCAAGTTGAATC[A/C]AACTAATGTTGTCAATAGCATAAAATTTAATCACGAGTATGGATCTAAATATGAGTATAT

2697	CaSNP4753	TATCCATTAGTAAAACTTATTAATTCTACATTATAAAACTTATGTGAGTTATGCACCATA[T/G]AATTAAAGTACTTGATTTTGATATTTAATAATTTATGGATTATTTAGAGATTTAACAAGA
2698	CaSNP4754	TTTAAACATTTATTAACATAAACTTTTGTTTTAATCTGCACACTTTGCATATGTAGTTAC[A/G]GGATATCATCAAAATATTAACCACATTATTGTAATTAAAAATACTTTGCACTAACAACCT
2699	CaSNP4755	CATCATGATCACCAATCTCATCATCATGACCACTAGCATCATTATCTCTTTCATTTCTCA[A/T]TTCATCAAATTCAAGTCTTTGTTGATCTCTTTCATTTCTCATTTCTTCAAATTCATGTCT
2700	CaSNP4756	TAAATGCTAGTGAATGATCAACTTAGTTTGGTTGTATAGGAACTTAGTGTCTTTTTGGTG[A/G]TCGTAATTATTTCATTTTGGGATGATTGTATCTATGACTTATATTATCAGTTGACTTTTG
2701	CaSNP4757	GTGGCAAAATACTCTTACTCTAATATACTCCCGATGTAAAAATCAATGATGTTAATTGTA[T/G]CTAGGGGTATAGTTGGAAGAAAAATGTTTATTATATCTTGAGTTTCTAAAGAATTGAGTT
2702	CaSNP4758	TGTTTTCAGTTTTCATTTTCATGTGTCTTGTGATAATTTCTTTCTTATTTTCTACATGTG[A/G]TAATTAGAACATCTTATTGTGAAAGGACCTAGTGAGGTTGAGAGTTCCACTAAACACCCA
2703	CaSNP4759	ATGAAAATCCAGATCATTCACTGGCTGCAGTTTTGTTAATTTAAGAAAAGTCAGATTTAT[A/G]AGGACAATTTCTCAGAATCATTTTATAAGCTTTACATCTAAATACGTAAAAGCATAATGG
2704	CaSNP4760	TAATGTTCAAATTCACATTCAAAAAGAAGTTGTACGAAGAAAAAAAGAACTTGTTACCTA[A/G]CACCTTATCTCATAAAGTAAGGATTTGTCTATTATAGTTATCGCTTATAATAGATAAAAT
2705	CaSNP4761	TTTCTAAACCGATCATCTCGATTGGAAGACCGTAGGACTGCGTGAATGTAGAATTTATCT[T/C]ATTGAAAGATTAGTTTCCTATTACTACTACCAACGGCCCAAAAAGTATATATAAATCGTA
2706	CaSNP4762	CTCACTACAAACTCATTACAAATCAAAAATATTTTGTCAAACACTTGACATACAATAACT[A/G]TAACAACTTATTCAATATTTTATTATATTTCAACAACATCAACAAGTTGACATAACAACA
2707	CaSNP4763	TTTCGTTTGGTGGGTCCATGTTTCATTTTTTTGACGTGACAATGGGGAAATAGCATTCTT[A/G]GAGCAGTGCTTTTGTGCAGGTGTCTGCCGAGAATACCCCAAGGGTATGAGCGATGTCTGA
2708	CaSNP4764	TATATGAATGGTTTTGATATTATATACATCTAGCAGGTTTTAAACAAGTCGGACGTTTGC[C/G]TATCATACCTGCCGTAATATGCTGCCCAATGAAGTGCAGTCCATCCAAATTTATCCCGAA
2709	CaSNP4765	AGGAGTGGAAATAGGCTAGGTTGAACTAGGACCGAGTCTAAGTGTGATTCGTAATATGTC[A/G]AAGGTTTTATTTTTTAGGTTTGAGTTTGACATTGATAGAAGTCTAGTATAGCGTGATAGT
2710	CaSNP4766	GTTTTGTGATCTTGTTATGGGGAAACTTGTCTGAAGTTGTGATGTTGAGTTCATTGAAAA[T/C]CAACGGTTGAAAGACATTGTTAGTGCAAATGTATCATGTGAGTCTAGAAAAGAATTAGAA
2711	CaSNP4768	AATTTAACACAATTTTTATATACTACTATAGGCTGAGTAAGTGTTGTTTTGTACTATAAT[C/G]TTTTGAACTATTCTTAATGTCTTTTAAAATGTTTGAGAAATGGTGACATTATTGTTAATT
2712	CaSNP4769	TCTTTAATTATCTATGTAAAACTATTTTACATTACATTGCATACTAATTAAATTCAAACT[A/G]TATATATTAATGGCTAAGAAAACTAATATGAAATGAGAAGTTATGATAAAATACCAATAT
2713	CaSNP4771	CTTAGCTATCTAACGCCTACTCGTACGGGTCACTCTGATATCTGGAACTCAAGTTATAGC[A/C]TATAGAGTGAGCAATGATCAATATGCTATTAATAATATTAAAACTCAATTACAATCAAAA
2714	CaSNP4772	CTATCACCTTTAAGTTGTTCATTTGGTTTATGAAACCCTAGATGGTTGTTTGTCTTTCTC[A/C]TCTCTTCATCTTGCCCCCTTATATGTTTATGAAACCCTACATGGTATCAAAGAGCTTTGG
2715	CaSNP4773	CAAAGTGAACAATTGTTAATATAGTGAGTTAGAATGTGTAGACTACTTTCTCTTTACTAG[A/G]GTACTTGTTAGTGTTTTGAACTGTTTGCAATGAAAGTAAACTAGAAAAGAAAATAATGAT
2716	CaSNP4774	ATTCATTAAATGATTGATGATGTACTAGAATCTAATTCTATGAGAAGCAACAAGATATAA[T/C]TAGCATTGACTTCTTCAACATGAAACAATAAAAAAGTTTATACGTTTCACTTTTGATATT
2717	CaSNP4775	AGGTCAAAGTGGGGGGTGCACGGTACTAACCTCTTTAAATTATTGTATCCTCCCAATTCG[A/G]ACTAACCCATATGCAAAGATCATTCCCCAAACGTTCTCCATAAGCTACTTAATCACATTC
2718	CaSNP4777	TTTGCATCAGAGAGAGACACGCGCGCACATTAGCCAAACCCACTCACTACACCAACCGAA[A/T]TTCTTTATTGAATAATTCTTCTTCATCTTCTTCGTTTTCCAACCGCTTTTGCCTCAGTCA
2719	CaSNP4778	TTTTCTGCAGGTTCTTGATCTTCATCATTGTCTTTATTTTCTTCTTCTCCCCAGTCTAGA[A/G]CATCAACTTTACACTCTTCTTCTGTTTTTTTTTCCAGTCCCAGCTCTCATCTTCTTCAAA
2720	CaSNP4779	TGCTTAACTGCATTGAAGTGTGTAAACTCACCCTTTATGGCTGGAAAATTGTATTATCAA[T/C]GTTTATTCGAACTGTAAATTATGTATGCTTATTGACAGATATTTCAAGGGAGAAAGACAT
2721	CaSNP4780	TTGTAACCGAGATTGATCTTGAGTTAATTTTTAAAAGTAAAAGTAGGAATTAATGTCATC[A/G]AAAAGGGATCACAATTCAAACCTCCATTCCTTGTGATTGATATTTCTACTTCAAGGTCGA
2722	CaSNP4781	AATTTCCCTGAAAACATATCCTACCTGCATAAGACCAAGATATGATCGACAATCTGGACC[T/C]CTCTCTATTGCACCAAAGATACAAGGTCACCTTTTGAACACATTGATCATATAGTCTAGC
2723	CaSNP4782	ATTTAGATTACAGTACACTGAAACAATTTCGACCATTTATTGAGATTGAACAGTTGTAAA[A/C]GTTAACTGCGTCACACAGTTAGTTACATTAGGGATACACTCAGCAGGCATTGGCAGGAGG
2724	CaSNP4783	GATTGATAGACTTTTGGGGTATCTCTAGTGGGGAATTCCTAAGTGGATTAATAGATTATT[T/C]CCTAAGGTCGAGAGCAACCGTGCAACACAAGAGTACATCGATCGTCGCGTATATATATGT
2725	CaSNP4784	TGAATTGAAAAGTAACAAGAAATCACCTTCTTTGAATTCCCTTCTAGTTATGTGCTTATC[A/G]TGCCACTTCTTGGTTCTTTTCTTGCAAAGTTTAGCCTTCACGTAAGCGTTTAACCTTAAC
2726	CaSNP4785	CCTAGCACATGCCAATACTATCACAAGCCTGATTGTCTCAATTCTAGCCATTGGAGCAAA[T/C]ACTTTAGAATAGTCTATACCCGTTCTTTGTAGGAATCCTTTGACAACCAATGTAGCTTTG

2727	CaSNP4786	TCCATTTATTATCAAAATTTCAGCGATGATTGTTATAGTTGGACAGAGCTCAATTTTAAT[T/G]TATTGAAATTATCATTTAATCCTTCGAGAAAACAGATTAGTTCTTCCTTTTCCCTATGCG
2728	CaSNP4787	TGTTTATAGGTAAACTTTCTATGGCAGATATGGGACAACCGAAACATATATGATAAACAT[A/G]TCAGAGTTACGATGAAGATCGATAAAAATGGTAAAAAGAAATGAGTGTGTATATATACAT
2729	CaSNP4788	AAACTCAGGACCTTAGCATTAAACATAAGGTACACAACTACTGAAAAAACAACTCTATCA[T/C]GTTAATTAAAAACGTTTATACATACATATATTCACCAATATTTTGGAAATATCATGATTA
2730	CaSNP4789	ATTTGTATCTCTTCACACATTCAACGCCAAGCCACTTTGCAAGATACATACAATCTCTTC[A/G]TAGTCAATTTTATAGGCTATTATTATCCGTTACCATGGTTTTAAAATTGTGATTTTATAT
2731	CaSNP4790	TATTTTAACAATATAACCTTTTGATTACGTCAAATTAACCAATTAGACTATGACTAATTC[A/T]TTGACTCAAAATCCATTTTTATAATAATGAACCTTGATTCCTAAGGTGATCAGACTTATT
2732	CaSNP4791	AGGAAAGTTTGATCCTAAGGCAGATGAGGGTATTTTTCTAGGTTATTCACAATCTAGTAA[A/G]GCCTATAGAATCTACAATAAAAGAACCAAAACTATAGAGGAATCTGTTCATGTAAAATTT
2733	CaSNP4792	CGATTTCATCTATAATTTTCGGGCAATTCGACATTCTCAACCAAAATATGTAATTTTTAA[C/G]CATATGAAGGCTGATGTTTGAAGTCTGATCTAGGACCTGAAACGTAATGGTGAATTTAGA
2734	CaSNP4793	TGATATGCAGCATAAAATCTCATCAAAATTGAAAACTACACATTATAAAGAAATTTGGCT[T/C]ATAATTAATCTCTATTGTTTTGCATAAAGATACCAAATCATACGAAAACATAGATAATCT
2735	CaSNP4794	ATACATGTGTTTTGTGGTGTTATTGTTGGGTAAGTAGAAATTTCATAAGTGTTCTCATGA[A/C]TCCATATCATCTAAGGGTGTCTTTAAATTTACTGTGTTGTTGATTGCTATAGTGTTTGCA
2736	CaSNP4795	TAATTGACTGATAGATTTTCATTCTTATCTCCCCATAAGCTATCCATGTTTAAACTTGAT[C/G]CAGTTAATGTAAAAAAATATATCATTGATATACTTTTGGAAAATAAAGAGAACGATAATT
2737	CaSNP4796	CTTCCTTTAGCTTATCTTTTGCACACTCAATTAAATTATTAGTACATTAAGTTATTCCCA[T/C]AAAATATCTTTTGTTATCATCAAAGCAATGTAAGGAATTGTTCTCTAAGTCAGTTTGGTT
2738	CaSNP4797	TGGATCTCACGAACATTCCTAGTTTCAACATGAACAATCCTGCTTTATGGTTGACCTTAT[T/C]CATTCACTTACTCATTACCACTGGCCTACTTCCAACGGTTAAATGAGCGGTCATAAGCTC
2739	CaSNP4798	TGGGTCTATAGGATAAATATGAACCTTGATGGATCCATCAACAAATACAAGGCAAGACTT[A/G]TGGTCAAAGGCTATATTCAACAATCCGAAATCAATTACACATAAACATTTGCACAGGTAG
2740	CaSNP4799	ACTCATTTTTAAAAACAAAGGGTAGTCAAATCATTAACACATTAATTTAGAAATAAATAA[T/C]TTTAACTAAAATAAAAATGGACAAAAAGGTAATATGGGGAATGTCCAAGAGACAAAAAGG
2741	CaSNP4800	CTCCAGTTACTTGCTCCCACTAAACCAGAGTGTTGATCAACTTAAAATGCCTAACTTACA[A/G]CCCCAAAGAAATCTAAACCGGTGCTTTGATACCACAATGTAACACCCCAATTTTTAATAG
2742	CaSNP4801	AAATGGAGTAGAGACCAAAGAGCATCATATCAAGATTACAATGTGATCAACCTCAAGCAT[T/G]AAGATGTTTATATCTTCTCATGTGCCGATAAGAATGCTTATATCTTCATCATCAGATTCA
2743	CaSNP4802	TATATCCTTTGCACCGTATTTATTTATTGCAATTCAGATTCATTTTACTCCATTTAGTTC[T/C]AATTTTAGTCTCTAATTGCTAGTTCTCTTAGTTTTGATGTTTGGTATGAGCAAGGAATCA
2744	CaSNP4803	TCTCTCTATGTTGAAAGAATCCTAAAGAAATATAAATACGTTGACTTTAAACCTTCTTGC[A/G]CACCATATGATATAAGTGTGAAACTTTTCAAGAACACTGGTGAAGGTGTTAGACAAACTG
2745	CaSNP4804	AAAAGAAGTTTTACAAAAAATAGGGATGAGAAAGAAGGTCAACCTTACGTTTAGACAGGG[A/C]AAGATATATCGGAGGTGGTGAGTAAGTTTCCAAAAGTGATTGAAATTGGTTGGAAAAAGA
2746	CaSNP4805	TAAGAGAAAATTACACTCACCTCCTTGAGGTCCTCCTAAATAACACTAACACTCCCTCTA[A/G]TTTTTAACTAAACACTAACCTCCCTCTATTTTTGTTGAAAGTGAAGCACCTCCCTTCCCT
2747	CaSNP4806	GGCATCAGTATTGTTAATCATACATCATCATCCAATAAACACATCATAAATTTATAACAT[A/C]ACTATCATCAATCATACATCATCATCACACATAAACTTATCACAAATTTATAACATCACT
2748	CaSNP4807	TGAAGTTCTATATATATATATATATATATATATAGCTTCACTTGCTACAAAATGATCTCACT[T/C]TTCACTATGATTCAGGCACTGCTAGAGGATGTGGTTGGCAGAAAATTGGTGCTTTTGTAA
2749	CaSNP4808	TCAAGGACAAGGTTGTGAGGACCATTTGCTCAAACAATCCAAGGATATTCCCACTGACAA[T/C]CAATCCGTTGGTGACTCTTTTCCCACACATGAATCATTAATGGTGCCTTCTCTTCTCGTC
2750	CaSNP4809	AAAGATGATTAATTCTTCCAACATAAGAGTATTTAGATCATTTGCTTCTTGAATGGTAGC[A/G]ATTTTAGGTCTCCATTTTCTTGCAGACTTATTAGTATCTTTTTCACAAGATCGAAGTAGT
2751	CaSNP4810	TATGTTGTGCCACATCCTCTAAATTTTTATAAGTATGAAGGCAATCTCCTATTCCTCTAA[C/G]TTCAGATTATGATCATGTGAATCATTATCCTCTGATTCTGTTGTTGCCTTTAGAACCATC
2752	CaSNP4812	AACACTGATCTCAAACCAAACATTTTTTAAGAATAATAACAATTGCTTCGTCACCACGTA[A/G]TTAATAAATTTTACATCTTTCCATAGTTCCCATGTGGTCACATAAAAAAATAACCCTCCA
2753	CaSNP4813	AAGGTGATGGTGACTAGGGTTTCAATATTCTTTTTTGTTTATTATTAGTGAGTATACATA[A/C]ATTGTGGACTTAGGGAAATTTAAAGTGGAAATTGTTGGTTAGATATTGTTGGGCCTTAAT
2754	CaSNP4814	ACACAATAATATTTTTATCCGCGACATCCAACTAAGTGTTGTAGTACCGATAGTGAATAT[A/G]TAACCAGTGGTACTTCTTCGGTGATCGACCTCACCTGCAAAGTATGCATCTACGTAGCCT
2755	CaSNP4815	TTAAAGGTGTCTTGTCTTTCCATGTTTTTTGCATTAGGCAATAAACGCAAGTAGTATCAG[A/G]TAGGTTAGCCTTAGGAGTTTCCTCTGGTTCTTCAAAACCAAAACCTCGTTTGTTGTTTCT
2756	CaSNP4816	CTCCATGAAAGAAAATGCAGGGAATTCTACCTCATAAGAGGATGTAGTATCCACTTCTTC[A/C]ATTTTTTGAGCACTTCAACTTTCCTAACTGCATTTGCATGCTCTGTCCTTAGCTTATCTC

2757	CaSNP4818	CTATATGGAGCAACCAGAGAGTTTCAAAGTCAAAGGTAAAAAGCAACTTGTGTGTAAATT[A/T]AAGAAAAGCTTGTATGGGCTCAAGCAAACACCTCAACAATGTACAAGAAATTCGATTCTT
2758	CaSNP4820	TGTCACATGTGATTCCACCACAGGAAGGGTTGTTAGTTTAACTCTTTCTGGAACTGTCGA[T/C]AACATCGATTTCCCTCTAGATACATATTTATCAGGAACATTGTCACCTTATCTTGGAAAC
2759	CaSNP4821	AGAAAAATTTATAAAGAAAAAGATAATAATATTAAACACGGTTTTATTTTATCCAATTCA[A/G]CTGTGTCTCACATCGCTATCATATCAAAATAATTTATACAAAATAATTTATGATTATTAA
2760	CaSNP4822	GGTTTTATACACCAAGTTTTATTTCCACACAATCAAACTTATTAGTACAATACAATTAAC[T/C]AAGAAATGAATAAGTCACCAACAACTTTTTAGTTCTTCCTATGAGGCAGAATAGGGATAC
2761	CaSNP4823	AGATAGTCAACCATTGTCTTCTTGTTGTGGTTGTTGGTTGTCAAAATTAAATTGTTCATC[T/C]GACGTGAACCTGTAGGATTGATGTGGTGTGTGGAAATAAATATGAGGAGATGGTTGAGTG
2762	CaSNP4824	TGATATATGATTACAATTTGGTAAATTCATGAAATCTCATTAAGGAATAATATTACTACA[T/G]ACACAAACACGACATTGACATATGAACACCGATAATAATTTCAATACATAGAAGTGATTG
2763	CaSNP4825	TGTTAAGGTTGTACATTTATGATTTACGTCTCTCGAGAAAGGTTTGAAGTTCTGAAATCA[T/C]GTGCATTGGAAAATTAACATTGCATCAAGGTGCTTTGTCACATAAGTCACATTTATTGTA
2764	CaSNP4826	CAGCAGCCAGTATATGGTAGCAGATGTGTCCAAGAACCTGCAATAATATCAACAAGTTAT[A/T]ACAAGTGAAAAATCGAACACTGATGCTAATTAACAGATAATCAAATGCAAAAAATCAACA
2765	CaSNP4827	CTACGCATTGGTAACCAATTCAAAGACAATCATTTATTTCATGTCTGTCGATCTACGCAT[T/C]GATAACCAATCTGGAGATAATCATTTTTCATCTTTGCCAATATGTTTATCACATCCTTAT
2766	CaSNP4828	AAAAAAGGACATCATATGATAAAAACAATTTTCTTTTTAGTTTTAGTTCTGAAGAGCCTA[A/G]CAAGTGGAGAAGACGGGTATTGAGGAAGCCGTCTCAAAATATAACTACAAAAAGTTTTTA
2767	CaSNP4829	ATTATTTTTACCCTTGCATTGAGGGGTTTTCCATGTTAAAATATCAATGTTCTTTACGCT[A/C]TTTTATTTTTGATCAATTTGCTGCCATCTATATATTTGTTGTTGCCCTTCACGGGTTCTT
2768	CaSNP4830	TTGCAATTGTACCGTAACACATAGGTCGATTAAGACCTGACGATTTTTTTTATAATTAAA[C/G]ATCAAACGGTTTATAATTAAAGTTTGGTATTTTAGAATTATAAATACTTCAAAATTTACT
2769	CaSNP4831	GACTTACGCTTGATGTGATTATTAAATGCAGACACAGTTATTTGCTTGTCGCACTTATTA[T/G]AGGAAGGATGTTTCTTGTAACGCTTATCAAAGGTAGATTTTTATTGTCACGTTTATCAAA
2770	CaSNP4832	GAAAAGCTTTAGAAACTTCATCTTGAAATCTCGTCAACTCAATTACAACCCTATTGTATT[A/G]ACTTGCAACAATATGTCCCATTTTTAGAAACAACGTTTGTTGGAACCAAGTTGGTTTTAT
2771	CaSNP4834	GTGGGTGGTTTGGCCCAAATGATTGTTGTTGGGGCAACTTAGGGTGTAGGGGCAATAGCT[T/C]CCCGTGATATGGTTCAAGGGTTTAAGAACAACATGTACTCTAAAATTCCTAATCCATTCA
2772	CaSNP4835	GTAAAGGTACTCCTACAACAGAACCATCACCATCTCCCTCATGCTTAACATGCCTTCTGA[T/C]CGGTAATTTCTGAGGTCCTGGCATGGAGACATTGTTCCAATCAACATCCATATCTAAGTT
2773	CaSNP4836	CCATTCTTGTCAATCTATGCATCGGTAGCCAATTCCAGGGTAATCATTTTCATTATTGTC[A/G]ATCTACACATCAGTGACCAATGCAAAAATGGTCGTCATTTTCACATTTTTCAATATACGC
2774	CaSNP4837	CATGCATGGTTGTCATGCTTGTTTGTCGCCGAATAACCTTTACCACACAATATTGTAATT[T/G]GAAAGAAAAAAGTAAATGAAAACTACAACTATTGGCAAAATCAAGTAAGCACATGTATTA
2775	CaSNP4838	GTATTTTGTACTTAAGCAACTTGTTGATTCTGATAAATTGTTTATGTATTATGTATTTTG[T/C]GATCGAACATATTACAACTTGTTGATTCTGTATTATGTATTTTGATAAATTGTCTACTTA
2776	CaSNP4839	GTATAAACTATCCATTTTGTCGAATTATGTTAACACTCAAGTTATATTCTAGACAATTAA[T/C]TCATGATGTTATAGGAATATTAATGGCAATTAATTTTTGGGAATTACTTAAATTTATTAG
2777	CaSNP4840	AATTACGAAAATGTCATTCAGCTCCTTGGATAGATGCATTCCATGTTCAAGTACGAGGTA[T/G]CAGATTTTCTGGGTACTTGGGGACACGTGCTGTCCTTTTCTCTTTCCTTTCTTTAATGTT
2778	CaSNP4841	CCTTAAATAAAACCAACCTATTTTGATGAGATGAGTCATAATACCACATAATGTCTACAC[A/G]TGGATAACTCCATCACCTTTAAATTACCTAAAGCTTCAAAAAAGGACCATCAATTTGAAT
2779	CaSNP4842	ATGGTGGATGCGCTGTCAACTGACATCACAAGTCACTTATTGTTTGGATCTTTGTCACAT[C/G]AAAACATTTGAACCATATTTTATTTGTTGGGTAAATTTATTTTCACAAAATAATTGACTA
2780	CaSNP4843	GGACATTCTCACACATAATTATTCTAGAAGATTTGGTTGAATATTTATTTTGGAATTAAT[T/C]AAACTTCAACGATCGTATTCCCAAATCCTATCTTTCAAGATTTTTTTTTTGTTAAGATCT
2781	CaSNP4844	GAATGTTATCCAAATCGCTTAGACATTCCTCACTTGAATACCTCAAGATCCACCGTGTTC[A/G]TCACCCCAAGGTCCAATAGAGTTTTGTTTCCCTGTTATGACTATGTTACTTAGCCCTCTT
2782	CaSNP4845	AGTGTTATGAACAACTATGTACTACCAATTGGAAAATATTAAAATTATAGGGTTCCTGAA[A/G]GGAAAAAGGAATACTCTGAATAATTAAATCATATTGATATTATTGTGAATGATGTGATGT
2783	CaSNP4846	TTACATTAAACATCTAATATGTTTGGATTTAATGCTTTATCAATGAAGATATTTGAACTT[C/G]TTTTTGAGCTTGTTGCTTTGATCATATTTGTTGATCTTTGTCTTCATATAAAAACATGTA
2784	CaSNP4847	AATACCTAAACCTAAATCAGTATCCTCTTCCTCTCTCTTCCAAAAACATAAACACGAACT[T/C]GCTCTGCGCTGCGCCTCTGCCTCGCCGCCTTAGTCGTTCATCAAAAAAGGTTTGGATTTC
2785	CaSNP4848	AGGTAACATTATGTCATTTTTTTCTTCAATGATACAAAAATTTATGATATTAAAAATTAT[A/T]TTTTTAGACTTTTTCATGGCGTGAATTGTTGATTGACAAAACAGTTTTGAAAAGATATAA
2786	CaSNP4849	TCACAAACAAAAGAAAGAAAGAAAAAGAAAGAGAGAAACAAAGTCCTCTTCCTTTTCTCA[T/G]ACCATCATTATCATCAAGTCAAATTACAATTTTATTTGTTCTTTTAATTATACTATCTCA

2787	CaSNP4850	ACTGACTTTTGCTTCCAACAGCTCCCCAACGACGACATGGTTCTCTACAGAGTGACATAA[T/C]ATAGATGACAAATTTACTATGAGGAATCGATTAACATAGAAAGCAGATAGCAACAACCAA
2788	CaSNP4851	ATTAACATGGTGTGCACATAAGTGTGTAAAATCAATTCTTAATAAAAACTAGGACAACCA[A/T]ACCATAATAACAGACACAGATACCCTGCTGGGAAAAAGGAACAAAAGAATAATATAATCC
2789	CaSNP4852	TTTGAAATACGTATTCCTAGATGAAGAAGATAAGAATCCAGCCATAATTAGTGCAAGCTT[A/G]AGTGAATTGCAAGAAGAAAAGCTGTTGAGAATTCTACGGAAGCATAAGGGGGCCATCGGT
2790	CaSNP4853	TTTCACTATTGTGTAGTAGGAGGTAAAAACGATAAAACAATATTTTGATGTACTTGGAAA[A/G]TAGGAGGTAAATATGAGGGAATGAACCATAAATTTCTCATTTTATGGTTCATAATCATAC
2791	CaSNP4854	ATCTTTGTGAACAAATCAGCAATCTGTAAAGAAGAAGAGACAAACGGTAGAGTAATGATT[T/C]TATGCTGAAGATGATGACGAGAGGGGTAACGAGGAGGATCAACAATCGCAGGAGGTGGTT
2792	CaSNP4855	ACAAAGGTTTACAAGGGTACCAAAGACCACAATTATATGTCATTAACATAGACATCAATA[T/G]GTGCCTTCTAATGTTCTTATTCTATGTAACTCATGTAGTCAATGAACACTCATCTCCGCA
2793	CaSNP4856	TCCAGCTACAACACCAAGCTATAGTGGATGAAACATTAACTTGAATACTTCAAAATTAAT[C/G]AGAGTAACATTCTTATCTTTTGTGATAATACTTCTACTATATGTTTAACTAAATATCCTA
2794	CaSNP4857	TCGACCAATTTGTCATCAGCGCTAAAGGCTGCAGAGTTCGTGCATGAGTGAAAACAGAAC[T/C]TAGGGAAATACAAATTTACGTGTTGATGGTAAAGGAGAGAGTGAAAACATAATTTAGCGG
2795	CaSNP4858	CCTACAATATTAATGTGAATAAGTGATTTCACATTGATTCATAATTCATGATTGATAAAT[A/T]ATTTAGCACCCATGAAAATTCAACTTCTAGGGATTTTTTTTCTTTCTGGATAAGAAGGAG
2796	CaSNP4859	ATGTGAAGGGAAAGTCCGAGAAATGTCTCGAATTATATCCAGTTCAGAAGCTGATGTCTC[A/G]TATATCACCAATTGCTGTAATAAGCACCATGTCTTGTCAATGCAAACCCACAAATGTGGA
2797	CaSNP4860	AAAGATGTTTACCCTTTGCTTTATGTATTTTAGCAATTAATGTAGATATAGAGAGTGGTA[T/C]TAGATAGATGCATTGGTGTATACGAGGGGAATTCAGTTACCCTTTCACAATATTTTTCCT
2798	CaSNP4861	TGTTGCCTTGTTAAACCATTTTTTTGTTACTACAAGTGGTTAAGGATCTAGACTTGATAA[A/C]ACCTTTGTAATTGTATGAGAAAGTGTTCAAATCTCCCTGAAACCTTCCTTGGAGAGACCC
2799	CaSNP4862	ATAAATATTACTTTTTTGTATGACTAAAAATGTAACTTGTTTATTTATTTTTTCAAATCT[C/G]GATATTAAAAGCATAGGTATAAGGTTTTTCAAACTCGTTTCTATGAGTTGTTTGTTTTGA
2800	CaSNP4863	GGGAAAGGATTGAATGGTGACTAATATTTTTAATTTTTTTGGAAAGGGTTTTAAATCCGC[A/G]AACATATAGTTGGTTGAGCTAGGGATAAAGAGTTCTATAAAAACCATATATTTGTTGGTG
2801	CaSNP4864	GAAGGAGATTCAAAGGAGTCGTTTGATGATGTATCATAGACTAGGTCAACAAAAATTGAC[A/G]CAACTTCTTAGGGAAAAGGATTTAAGGGCTTGCTTTAGGTTCATAGTGGAGGATAGGAAT
2802	CaSNP4865	ATGGTGGAAGAGTTGTATTCCGCTACTAAAAGTGAAAGAAACTGACCTAAAACATGAAAA[T/C]GCATATATATATGGAAACATTCGAATATGTATTCGGCTACAATCTGGATGAAATCGAATA
2803	CaSNP4866	AACTTCTCAGGATGATACTGTACAGAACCGACTTGTCTTGGCTTCTGGCAAAGCTAGTGC[A/C]GTTGGAGAAAGGAACATGGGAGGTTCTGGTGTACTTGAGCCTTCACCATGTGTAGCTGCT
2804	CaSNP4868	TTCATCTCTATTCCTTCTTCTTCATCTTCCTTCCTCTCCATTCTTCCACTTCCTCGCCAA[T/C]GTACTATGCGTTCTTCTCCTTCGTCGACATTTATATGGATTATTCGTGGGTGTTGTTGTT
2805	CaSNP4869	TCAGCGAAAAGCAATGTAGAAAACTTTCGAGTTCGACCTCCTACACAATCTAGTAGAAGA[A/G]CCGTTATGGAGAAAAGGAGAACGAGAAATGAAAAGATGCAACAAGAGGTACAAGAGCATC
2806	CaSNP4870	GAACATGTAAGATTTAAACATGCACATAACAGGAGCAAGATTGTATGGAGTAACAACAAC[A/G]TGTCAACAAGAATAAGGAGTAGGTGATGCTTATATATATGGTGTAAATCCATATGAGGAT
2807	CaSNP4871	GCAAGATTTCAAGTTGACCCTAGAGAATCCCATTTAACTCTGTTAAGAAAATCCTTAGAT[A/G]CCTAAGTAAAGAGGGTATATACATTCATCAAAGTAAATAGGTATATACATTCTAGTACTA
2808	CaSNP4872	AGCATTCTTCTCCTAAATTTATAACTGAGTTTTGGGTTCCGCGTCAGATTTCAAACACTA[T/G]CAATCATCTTGTTCGAAAATACGGATTTCCGTCCATTTTTTTTCGGGTGGTACGAAAATC
2809	CaSNP4873	TTCTACCACCTAAACACAATACATCCAATTCTAGAGGGAGAGATCAGGCGAGGGAGTAAG[A/G]AAGATTTGAGAGAGCGAGAGGGAGCGTAGAGAGAGAGATGCATAATTTGAATGTACATCA
2810	CaSNP4874	AATACTTTATCGTTCACATGCATTTTAAGAAATAAAACACTGTTCTCACAACACTTCCGA[A/C]GATCATCATAGTAGAGATCCAACTATTGCTATAGTGTCATCAAATCATTATAATAAGTAG
2811	CaSNP4875	GGATTTTCTTTGCGCTTGACACTCTACGTTTTCATTAAGCTTGGGGTTTGATCCGCTATC[A/G]CACACGGATCTAATATAATAATTCTGATATAATAATAATAACCGAATTGTTTCTGATATA
2812	CaSNP4876	CTGCACCTAACACAACCTTCCCTCACCAGGCTGCTCCACCTACTAACGTAACACAGTTGA[T/G]ATTTCCTTACCTAAGCACCAACACCAGACTTGAGAAGATCCACAAGGGACAAACATCCAC
2813	CaSNP4877	ACATTATCTTTTTGGAGCAAGATTGAAATTGTTCAGTGATCATAAGAGTCTACATTATTT[A/G]TTCAATCAAAATGGAGTAGAACATGAGAGAACATAGATGTATGGAAGTTTTTAAGGATTA
2814	CaSNP4878	ATATGAAACAACTACCGAATATGAAACGACTACCAAATATGAAATGACTACTGAATGATT[A/G]CCTAATTGATAGAATTTAAAATAGGAAATAATATTTAATAACATAATATATATAGGCCGA
2815	CaSNP4879	TGCTATCTGTTTTATGTAATGTGATATTAAGATTGAGCATCTTGATATATTTCAGTTGAA[T/C]AAAGTCGTTAGTGGACCAAGAAGTGCAGGAGTTATTGGTGATCGGGTGTCAAGAGAAAGT
2816	CaSNP4880	ACCATGTGTTGCAAGTGTAAAATATTTATACTACCGTTCAACAACTTTTTATAGAATGTA[A/C]AATTAGTCAACTCCGGTGTTGAATCAATCTTATCAAATTTTATATAATTTTGACATACTG

2817	CaSNP4881	CCGGTTCCGTTGTCGCAAACGACGACGTTTTTACTGTCCATGATGCGATGCGATCGCTGG[T/C]TTCGAGTTTCCGTTTGGCTTCGGCCGGAGAAGGAAGTAGTAAGCACGGGGTTGGTTGGGG
2818	CaSNP4882	GCATACTTGGACAGTGTTTGACAACTTTGGTGGGACACAAACAGACATTAAATATAAACA[A/G]CATTGATGACTGAAGGTTGGACAAGACTAAGATAGATTTATGGGATGATCAATGGAAGTC
2819	CaSNP4883	TTACTTGATTGATATCATTTTATCTAGTAATTCAGATTGAGCATTCTATTAATTATGAGA[T/C]CATCACACATTCAATAAGTATTAACGAAGTTATTTGGCCATCTGATTTTGTCTTCTGTTT
2820	CaSNP4884	TTACCACATCCATTTTCTCCACATACCAAGCAGAAGTTCAATAAATTAAAACTCATGATC[T/C]TAACAGAAGTTCAACCGGTATACCAAGCAAAAGTCAATAAATTAAACAAGAATACCAAGC
2821	CaSNP4885	ATTATGATGAATCAGAAATAGAGGATCCTAAAGAAGCCAACTTATATTTATTGGTCAGAT[A/G]CTAAAGACAATGAGGTAATAAATTCTGAACTATGTCCTTTATGTGAACAAATTGAAAAAG
2822	CaSNP4886	TCTCTTCAATTATATCCCCTAATCAACGCAAACTAAATGACAATTCTTCCTTCCATTCTT[T/C]TTATTACTAAGCTACTACTACTCTTCTCCATAATTTCCTCTGCAACAGATACCATAACCC
2823	CaSNP4887	GTTTAAAAAATCACAATTTATGCCACAAAAAATATTATTGGGTTGATTCGCAGACTAGGT[T/C]GCTCTTTTTAAGATGTTTTACAGCACTACCCAAATTGTGCAAGATAGACTATCAAGGCAG
2824	CaSNP4888	GTAGCGATCAAAATTCATTCCTATCATGAAATATAGATGATTGGCCTCTAAATGTTAATA[T/C]TTGAAAACAGGGGCATAACGACCAACAATACAGAGACAAACAACATCAACAACACAAGTT
2825	CaSNP4889	CATGATGATAATCTGATTCTTACTGGGTATGTTTTCCCATTCTAGAGGTCCAATTATTAC[A/T]AATCTGTTTTATTTTTCTGTTTCAACTTATGTTGTAATTCATATCATATCATTTTGAGCA
2826	CaSNP4890	AACCAATTATCTTCTGATCAATTTGAAAACCACACTCCTCGTCTCATTGGTATAAGAAGC[A/G]ACTTCAACCCTTTTTGTGAAATAAGCAAGAGCTACTAGGATAATCTGGTGCCCATTTGAA
2827	CaSNP4891	CATTTATTTAGTAAGAATTTGAACAATATTAAATGCTTATTGGATTTGATGTTGAACAAT[A/G]CGTAGCATATATTCATACACAAAAATGAAATTAAAGATCATCTCTTTAGACGTCTAAAGT
2828	CaSNP4892	CAGCTAAGGTTTAAACGGATACTTCAAATTAAGTCGTCCTTAACTAAAATTCATTAATCG[T/C]TTGAGTCCAACCATTTGATTAAATTGTATTTATAATTAGATAAGACATTATGTGATATAG
2829	CaSNP4893	AAACTCATGTCTCTGGATTTGCAACGTTAAGGACGTTGAAGTCATTGATTGAATCTATCT[A/G]ATTTGAATCGAAGTTAATCTGAATAAAATGAACACCGTAACTCTACGAGAAAACAAAACA
2830	CaSNP4894	TGGCAGCATCTTGGAGTGAAAATGGGAAGGCTCTCAGCTTAATGTGGTCTTCTATGACTC[A/C]TTGAGGCTTCATGGTAGAACACACAACATGGAATTCCTTCAAGTGTTTGTGGGGATCCTC
2831	CaSNP4895	TCAGTTGATATATGAAGCATGTGTAAATCAGTAAATGGTACTCAATTTTCTTCTAATATC[T/G]CTAAATATGTAACAATGTGATTTTGCTGATGGATATTTTCTGATAATCATAGTGACAGTA
2832	CaSNP4896	CATTCAAGTTCTGTGCTGGCAAGTTAATAGTAGATTGAGGATTGACGTTTACTGTTTACT[A/G]TTACTGGCAAATAGGTAAGGTTTTGGTGTTGAAACTGATTGAACTTCACTAAGTGTGAAA
2833	CaSNP4897	TTTCCTCCACATATGCAAATATTTTCCACCTCGGTCAAAAAGTTGATGTGTTTCTCTATA[T/C]ACCATCCACAATCTTATACTATATCTCCTATTTAAATATTATCTACTCAAAATTTTCTAA
2834	CaSNP4898	ACCACTAATTGATGAAATTAGTGGTTAAAACACATAACATATGTTAGAACAATCAAATTT[A/T]AAAAATATATATGAATGAAAATGAATAACCAATCAATATATTCAATGCACGAAAATTTAT
2835	CaSNP4899	TGTTATGCTTTCATTTTGCGATATCTACTTTTACTATGACTTCTTCACGTGTCTCTGTCT[T/G]TGGTTGATCCAGTATTCTTGAGTTCTTTGTAAGTATGATAAGGAAATGGACTGTGGAAAT
2836	CaSNP4901	ATATTTAAACTGTGTTAACCATCTAGATTATGTTAATTTTATATGTCAAATTTGTGTCAA[T/C]ATTATTTCTCGTTCATAACTGTTTTACTTGGTATTTAACAGTGCATGTGCAAAATAGCTT
2837	CaSNP4902	GAAAATAAATTTTCCATTTTATACATGCAATGTAAATTTTTGAAATCTAGGCTGGGTTTG[C/G]ACTTTAGATTCCTCGGCACAGTAGACGTCTAATCCTATTTCCATTTTCTCACTCCATTCC
2838	CaSNP4903	TTCTTAAGACGTCCCCTATTATGTGTGTGGTATGTGGTCCTTATTCAGAAGCAACTGGCC[A/G]TTGATTTCTTAGTTTTCCTGAGTTTTTGGACACCTCGACTCAAGTATGTACTTAAAATTG
2839	CaSNP4904	TGTATTGTAGGACCTTTGATATTATTTGGTTATAATACATATGATATTAATTATATAAAG[T/G]TGTCACACTAATGGAATCCAACACAAGTGGTTAATAGTTAATATGCAATGTATGACTGAT
2840	CaSNP4905	CTCATATTAACTGTCTTTTAAGGTAATGCAAACAACTTAAGAAAATGATTACTTATACAA[T/G]TTTTCAATGCAAAATAGTATGTTACAACTAAAATATCCTTGTACTTTATTTATTATTAAC
2841	CaSNP4906	GATAGATCATAGTTATGATTTATGAACATATAGGTAGACCATCTTGTGGTAGCAAACTCC[T/C]TTCTTGATCCAATGTATCCCTAACTGCCTTGTATAAGATTATCCATGAAGAAGAGTAGAC
2842	CaSNP4907	GTAAATTCCATCATAATTAACTTACACAATTCTGAGGTCTTGGGTTCTAATCCGGGACAA[T/G]ACGTTCAACCTAACATTATCGATATTTTTCAATTGAGCTATGACTTAAGGATATTTTTTC
2843	CaSNP4908	CGTACTTCAATTTTATCCAACCAATTCATTTGTAATATTTATAATATGTTTGATTTCAAT[T/C]AGTTTAATCCTCTTCATAAAAAAATCTTCATTTTAATTATTAATCTTGAAATAATTAAAA
2844	CaSNP4909	TTAGAAACAACAACAACAATAATAATAATAATAATAATAATAATAATAAAAGAAAGCTTA[C/G]TATGTGAAAGCGTGTCACAAAACAAAAATTAAAAGTATCCATTATATAGACAGCAATAAC
2845	CaSNP4910	TTCAGTCAAGTTGGCCTGATTTACAGCGCCTGGCTTGTTGCTGTTCTTTGAAGTGCGATT[T/C]TGATTCTTTAGTTGTTTGCCATTTGGCTTCAGAACTGCTCTGATGAATTTCTTTTTCTTG
2846	CaSNP4911	TTGTTACTCAATCTCAATTCTATCTATATATAGAGATATTCTAATAAATCTATCAATCCT[A/C]TCTCGAGGGATCCATTTAGTATTTGTGGTTTATAGTTGGATGTAGAGTTATTAATGATAG

2847	CaSNP4912	TAATAAATCAAATATCACAATAATATCAAATGCCACATTTAACGAAATTAAATCAAATAT[T/C]ACAATAATATCAAATGCCCAACATTTAACGAAATTAAATCAAATATCACAATAATATTAA
2848	CaSNP4913	TCTCCCTTAATCTCTCATCTCTGATTTCTATGAGTCCTTTTGACAATTGCATTTACCTCT[T/C]CTAATTTTCCAGCAATGCAACACAATGCATTTACAGTTATTTATAGTGCTTAAAGGTACA
2849	CaSNP4914	TGTATCCACCACTCCAAACCAGGTTCGCACAATGAAAGTAAAGGGTTGTTTGCAAGGTGC[A/G]CCTATTTTGGTTCTTATTGACAGTGGAGCCACGCATAATTTCATATCTCCTAAGGTGGTT
2850	CaSNP4915	TTTATCAATTGTGATACTTAGAGTTTTTAAACTGGACTAAAGTACTTGATTATATCAATT[T/G]TGTAGTTGTAATATGAGTGTTGTTGTTTAACTTTTAGACAATATAATTATGTTGTGATAA
2851	CaSNP4916	AAGTATTTTTCTTAGAGTGTGATGGTCATTGTTATAATCATCTTGTTAGAAGCAACTACT[T/C]AAGTTCCAAACATTTGGATTCACACTCGAATGTGGTGTTAGTGTGCCTTCAATAATCTGA
2852	CaSNP4918	AAAGCTATCATAAGATATATACAAGAACATTACAAAGCAACAAAGAAAACAAAATCCTAA[A/T]CAACTAATCTAGGGCTGCTCCTCATTATCACCAATGGGAGAGGTAGAGACTGCTGTATTA
2853	CaSNP4919	AACGAATGTTGCTAAGCATTTTTGGGCAGACGCGGTCAATACCTCATGTTACATTCAAAA[T/C]AGAGTCTGTATCACACCTATTTTGAATAAGACTCCTTATGAATTGTGGAAAGGTTAAAAG
2854	CaSNP4920	AATTCTAATCTACTTGGGTTCTTCGCCAATGTTGTTCACACAATAAGTGAAACGGGATAA[A/T]TAGATTAAGATTTTGGTTTTCAAACGGGTTATTCTAATACATTTTGGTTATAGCCTATCT
2855	CaSNP4921	GCTATCTACACGTGTCAGATGCATACAAAGGGCCAATGCCCTCGCTATGTAAACGTGTCA[T/G]ATGGACACCAAGGGTCAGGGCCATTGCAAATAGAAGATGTCGTCCTCGCTAATGATAAGA
2856	CaSNP4922	TTTCCCTATAATAAGAAATCAATGACCAATTAGTATACTTTCATATATATCCCCCAACCA[T/C]AACACATTCCTAACATTCCCATTTAAAGTCCTTTGTTATACCTGCTTGCCTATACATAGG
2857	CaSNP4923	GGAAACTCTTTATTTTTGCAATAGTAACCAACTTGTGTTCCTTTACTTTGTTAGTTAGCT[T/G]TGATTTATTATGTGTATGATTGTATAGATGCTAAAGTATATTAAAATAATGAATGACAGT
2858	CaSNP4924	TATATGGCTCCTCCAATTCTAACTTTACATATCTTTGGCAACAAAATAAGTTATATTGAC[A/G]TTATCTTATCCCAAATGTTCCAGTAATCATTTGCAAATATGAATCCGATCTCACGATGTC
2859	CaSNP4925	TGAGTTGTCCGTAAATTTGGGTGATCATGAGAAGAGTTGCAGAATCTCCATTTTGGGTGA[T/G]CATGTGGAGAGTTGAAGTATCCCCAACCATGTCATAGTGAGAGGAGGGTTTGGATGATCA
2860	CaSNP4926	GTCATTGGATTACCATTTAGGTAATTTTCGTCCTCCATAAACTTATTCTAGTTCAATTAG[A/G]GTTTTTAACCAATCTTGTGCATCGAATAGGTGTGTTTGTGGCATTGGATTAACATTTACA
2861	CaSNP4928	AAGATAAGAAACATTAAGAAAGGGGAGAAGTGAGAAAAGCCTCAGATATAGAGAGCTTTT[A/G]GTGTGATTTTCCAACTGAGGAGAACCCTCTATTTATGGAGGAAATCCGCAACTGGATCTG
2862	CaSNP4929	AAAACATATATGCCAAAAATTCTTTCTTTGCCTTATCTCTCCTTCCACATAATTACCACA[A/C]ATTTTTCTATATTTTTCGTCCTATAGGTTCACTCTCATTTAGTATAGGTGACAATTATTT
2863	CaSNP4930	TTTGACCATCATTGCACCGCATTACTCGAAATATTTTCTCTAACTTCAGCAACCATTCTT[T/C]TGCCTCAACTATATCAGAACTTCCTCAAAATGGTGGTGGATTGTTCTTTCGGAATTCAGA
2864	CaSNP4932	GCGGTTGTTAGAGATAGGTGGTGGTCGGAGGTGAGTGGTCGACAATGATGGAGATAATCG[A/G]TAGTGGAGGTGATGATTGGAGGTGGATTGTTGAAGGTGGTAGTGGTCGATGGTTGTTGGA
2865	CaSNP4934	GGCTCAAATCTTATTTAGAGGTAGAGTCAAAACTTTCGATGGAGTCCATCCATCTGATCA[A/T]CACTTGAAGAGTGGAATGTCAAGCTCAAGACACTAAACGAGCGCTTATTGGGAGGCAACC
2866	CaSNP4935	TTAACAATTTGTTTCTCTAATTGTTCTTTTAGCTTCCTAGTCTTCCCTTTAGTCAATGGT[T/C]CCGCTCCTAGTTCTCGAAGCTCATTAACCTTCATGTAGGGTTGGTCCACATCACCAAGAC
2867	CaSNP4936	ATTATAATTTTGATTGGCACACCAATTTACAAACTTCACTTTGTGCCTAGGGTCTAATAC[A/G]AGAGCAATCAACAACAACATGTTTAGATTATTGGGATTTCCCCAATACTTATCATATTTC
2868	CaSNP4937	AGCATCTTCCTGGTGCAAATAATCCATGTGCTTAACCCTGATTTGTGTAAGTGAGTGGCT[T/C]ATTCAAAGTAATCCAATGTTTCACTCTGTCACCAAACTCTCTATAGCAAAGCTCTATATA
2869	CaSNP4938	TGTTGTCTCTGTTAATTTCTGGACCCAAACAAGTGGGGAATGATATCGACGTATACTTGA[C/G]TCCTTTAATTGAAGATTTAAAAAATATGTGGGAGACAGGTGTGGAAGTTTATGATGGGTA
2870	CaSNP4939	TGAAGCCATAATCCAATCTCAGCCCTCTCGATGCAGTCACCCACTTCGTATTCGCCGTCA[T/G]ACTAAGCCTATCGCTGCCGCATCACTGTTTCCATCACCTCACTCTCAACCCTAGCTCTCG
2871	CaSNP4940	AATTGCATTTCTTCAATTAGAGGATAAGTCTAACTTTGATGAGCGAATGATGTTAAGATA[A/G]TGGACTTTTAACAGAGAGTTGACTTTATCATAGAGTTGACTTGAAGTGTTGACCATACGA
2872	CaSNP4941	CGCCTATAATAAAAGGTTGATTGATTGTACTCCAATTAATAGAATGACATTTACCTCCCC[A/G]TATGAACTGCCTGGTGCAAGAATTATTTTATCACATATGCCTTATGGAAGCCACAAATTA
2873	CaSNP4942	GGTTTTGGTTATTGTCCATGGGGTTGATGACTACAGGCGGAGTATAACCAGGGTGGGAGA[A/C]CATATATAGGAAATTGCATAGCTGCTGTACGAAAATGTGGGTTAGTCGGGGTAAAGCCCG
2874	CaSNP4943	AATACTTAAATCATTCTACCATACAATACAAACTACACAAAAATGAATCGCAACACATAA[A/C]ACATAGTGAGACAAAATATGGAATCAATTAATCAAGCATACACACAGCTAAGAATTTATA
2875	CaSNP4944	TGTACCCCAAAGGATACCTAAATTTGTGAAGATGAGAGAATGTATTTGTAAGACCCATAA[A/T]TTTAAAGTACCATTTATGTATTTTTGGTGTATTTTGGATTTTGGCTCGGAGGCTTTTAAG
2876	CaSNP4945	CTCCCAAAATCAGGATACTCGTGATTAATTTTTGCCCATTGTGGAGAATCTGCAGGGTGT[T/C]GAAACTTTCCATCTCTAATTCTTTCATCTGCATGCCATGTCAAGTGTTTTGAATCTTCTT

2877	CaSNP4946	CTAGCTTTAGACAAGGTCATATTCCCAAGCAGTCGTCTGAGGGCATAACTCAAATTCCAC[A/G]ATAATTGTAAAGGGTCACCAACCTAATTAACAAGTAGCATTAAGCAAGTATATCATGATA
2878	CaSNP4947	ATATCACGGATCACCCCTGCAGACTCAAATTCCTCGTGGAGTCTCCTTACATTGCACGGG[A/G]ACAATGTTTTTGCTGGTAGCGTTAGATGCTCATTTTATCTGTGATCTGTAGTATTGGATT
2879	CaSNP4948	CTGAGAAGGAGGCCCAATTTCTGAAGTTGTATCAAGGGAATCTCACGATAGCGGAATATG[A/C]GGCAAAGTTCGATTTGGGATTTCGACCGCCGTTTTCGTATAAGGAACTTATATGGAGTCC
2880	CaSNP4949	ACTTTTCTGACATAATAACAACAAAGAAAAATTTAGATTCATCGGATGAAAGAAAACAGT[T/C]CAGTTCATCAATTTAATCTCGAAAAGATTACATTTAAATTGATCTAAGTAATATATTAAA
2881	CaSNP4950	AGTAAATAAACTCAGTCAATACATGGCTCAACCAAGTACAGTCTCATTTACATGACATTC[A/G]TCATACACTAGTATTTGAAAGGCACCTCAACAAGAAAGTCTACAACTCATTTCTGCATAT
2882	CaSNP4951	TACTATCTTTCATTTGTAGGTAGAGTAACTCTTTCTCGATCAACTATATCTAGCATTCCT[A/G]CTTATGTGATGCAAACCATGTCAACTCCTTGTTCTATTTTTGATGAAGTTGAGAAATTAT
2883	CaSNP4952	TACATAGATAGTAAATGTCATGCTCCTTTTTCCTGTTTCATTGTGTTGCCCTAGCACGCA[A/T]CCCATGGCCTAGTCGAGGACTGTAAGATACATAATCAAGGGTCGGTCCGAAACTGGGGGG
2884	CaSNP4953	ATCTTATAGCATATTGTGATGCTGATTATGCTGGAGATAAAATCAAAAGAAAAAGCACAA[A/G]TGGAGCATGTCAGTTCTTAGGTGAAGCATTGATAAGCTGGTCTTGTAGAAAACAAAACAC
2885	CaSNP4954	TGTACATAAGGTAAAAAACTACTTTTAATTGCATTTATTGGTAATTAATTAAATTTATTG[A/G]TAATTAATCTAAGTTGAAATTTTCATTGAAGGTTTTTGTATGAGAAGTTGGTGTGCTTGC
2886	CaSNP4955	CCCCGACGCATGATCAGATAACGACTCCCAACCTACAGGTTGACCTACAACCTAAACCAA[C/G]GGATCCACAGCCCCAAAGAAATCCAAAACCAAAGCTCTGATACCACAATTGTAACACCCC
2887	CaSNP4956	TGAGAAATCAATTAGGTAATTACTAAAGATCAATCAACAACCAATACACAAATTGAATAA[T/C]ACTTTAAATAAGATCTAAACTAAACTTAATAACACAAAATACTAACGCCAAGAACTACAA
2888	CaSNP4958	TCAGTTTCCAAAGCTAACAGGATTTCTCCATGACCATATTTCCGTTGTTAGTAATCATTG[A/G]TCAGGAAACAAAGGTGAGAGGTTTTAGTGAAAGTTGTGTCTTGGCTTGCATGTATATATC
2889	CaSNP4960	TGGCTTTCTTCTTGTGAAGATTTCCATCAGCATAATCCCATAGCTGTACACATCTCCTTT[C/G]ATAGAAACTATTCCCTTAGATCCATACTCTGCATTTTAATACAATAAAATCTTCTTAAGT
2890	CaSNP4961	CCACCTAAAGTTAAACATACTCGTCCCCACATGTAACCCTTACTTCAATTGTTTTGCTTT[A/T]AATGTAACGAATGTACATTTTCCAATATTGTCATTCGATGTTGTACGTTACACTCTTTAT
2891	CaSNP4962	GGATCATTCGGGAAATGAGAAAAACTGCCTTGCATCAATCTTGGAAGGGAAGATCTTTGA[C/G]GGTCTAACAGGCTACTCGTCTTTTTATCACGTATACAAAGAGAAGAATAACTCTGATATT
2892	CaSNP4963	CTATGATCTTAATTTTGCTTCATCATCATTGCCCTTAGAAGTGTGGCCCACCATTTACAT[T/C]ATTGTTTGTCTTTTGTCAATCACCTCTTCAGATGTAGGGGCATTTCCCTTAGATCAGCCT
2893	CaSNP4964	TTGCATAAAACCATTTAAAAACTTAGAAATTAAAGAACCATTAAACGTGTTTTATTTCAT[T/C]AAGGACTAAAATTCCTTGTAAGAGATAATAACGAAGGAACTAAAAAATATGACATTTTTT
2894	CaSNP4965	TGAGATATTTGTTAAGTTATATTTATTATCTATATATGTTTGTTTGGAGGGAAAAAGAAA[A/G]GAAAACTAGAAGGAAGGAAAAGGAAAAGAAAATAGTATAAAAGGAAGGAAGGAAAAAGGA
2895	CaSNP4966	TATTTTTACCAATAATACATGTACCTTTTATTTGAGCTTTATCATTGTTACCAAACATGA[T/C]TGATCCTCCATCCTTTATCTCCAAGGAAATAAAACAGTGCTTATCCCCTGTCATATGCCT
2896	CaSNP4967	AACTCTTTATTTTGGGAATCCTAATTTTCATGTAACTAGTTGGTTAAGTATGCCTACTTA[T/C]GAAGCTGATTTTGGTTGGGGGAAGCCTGTTTATTTTGGATTAAATTCTCTGTCTCCACAC
2897	CaSNP4968	AACGAAATAAAAACACAAAACATTTTATTAACTCAAAGAACAAAAATCAACAAAATGTAT[A/C]AAATAAATCCTTAATTTAACTGATGATTGAGGATAAATAAGACTAAAACAGTGGAAAAAT
2898	CaSNP4969	CAAAAAAAATTATTATAAACAATTATTAATGTATTTTTTATAGTAAGAAACCTAATACAC[T/C]GCATCTTTTGTAATTATAATGTATCCATCATTATATTTTGTATCTTCGGTCGTTGGTCGT
2899	CaSNP4970	TACAGAGTTCTCTACCCCTATCATTTTCTCTTAACCTAAACATCTCCACTACTAACACTC[A/G]GTTATCAACAGCTATACTAAACAAACAAATGACTAAACCAATACAATTGCAATTTGTTGA
2900	CaSNP4971	TTTTTTGTTGTTTGTTTGCATCTGATTGCTTTGGGTGCTTTGGGATTGGGGTTATATGTG[T/C]CTGGCTTGATGGGTGCTGTTTGTTGGAAGCTGTTGTGTTATGAGGATTCTCTGTTTAATA
2901	CaSNP4972	CTTCCTCGCATTTCTCCTATTGGGTTGACCGTGACATTATTCACTCAAGTAACACTATAT[A/G]TGACGTCCTGTCAAATGTAAGGGTCATCTCCAAATAACAAACATAGATCAAATATGCATG
2902	CaSNP4974	TTTTTATCTCACTATCTAATTGAGTAGGAGGAGTTAAATTGTTATTAACCTATATATTGT[C/G]CACTAGCTGTGCACGACTTCAGGCACAAGTTGCGTGGTTTTAGCATTTATTTTAGCTTTA
2903	CaSNP4976	AAACCTATCTTCAGCTAATGGTATGGTGTATATGCCAACCCATTCATGTTTGGTGTCTAC[A/G]AACTGGATAGTAAGCACACCTTTTTGAACAAAGTCCTGAATGAAATGGTGTTTGGTTTCT
2904	CaSNP4977	TAGTCAATACATCATAAGAACTGCAATATTGTAGTCTTAAAACTTACAGATTTGTTTACT[A/G]AGGGTGTGTTTGGGAGTTTAGGAGATGAGGGGAGAACATTGAAAAAAAATGAAGGATGAA
2905	CaSNP4978	ATTTTATAAATTTTATTTCATGCAGCACATTTTATATAGGCAGAAGGAACAATTCAGTGA[T/C]GGAGTTGGCTATGGTTGGATTGATGGCATTAAGGACCATGCTGCAAAACATGTAAACATG
2906	CaSNP4979	GAAGAGGTCAAGAAGCAATTTGGTGTTGGTTTCCTAGAAGTGGTAAAATATCCTCAATGG[A/G]TGGCTAATATTGTCCTAGTTCCAAAGAAGGATGAAAAGGTTCGAATGTGCATTGATTATC

2907	CaSNP4980	GTTTGGATTTCTTGGGGATGTGGAGCTTTGGTTATAGATTGTAGGTCAACCTGTAGATTG[A/G]GAGTCGTTATCTGATCATGCGTCAGGGTAATCTGTCCGAGTTGTCGGGTTCGATGTAGTT
2908	CaSNP4981	GTAGAGCTTGTCAATTTCTTGGAAAATCTCTCATAAGTTGGTCATGCAAGAAACAAAGCA[A/C]CATTTCCCTATCAACTATAGAGGTTGAATATGTATTAGCTGCACAATGTTGTTCTCAGAT
2909	CaSNP4982	GGTTTTGGAAGATGATAGTTTGATTGATTCTATAAAAATTGTTAGGTGAGAAAGGGAGTG[A/T]TGGCAGAGAATTTAATTCGTCTGCGAATTATGAACATAGTGACCATCATGATCATCCATT
2910	CaSNP4983	AGTTAAAATTATTCGTAGGAGAATAATTTAATCAAATTATAGAGGATTATTATCAACAAA[T/C]ATTTTAAGATATCGCACATTATTGTTAAATTTATTTGTCAATTGGACTGCGACAAGAGTA
2911	CaSNP4984	CCTTGAATGCTTATATTGAAATATGTTGCTCCATGGAATGCAGAAATACAGGACAGCTAG[A/G]TATAGACCAGAACCATCTGAAGGTACATGTGATATGCATTGTATTATATATATGCCTTTT
2912	CaSNP4985	CTTAGAACCACCATTAGTTAACTGAATCGGGCTAACTCATTTACAAATAGTTTGAGCAGT[T/G]ATATATTTTGTTGGTCCGCAAATAAATAGGTTGATTGATCAGTTAACTATATAAAAATAA
2913	CaSNP4986	TAAAAACTGGTCTTTGGTTGTGTTGTTTGACGGAGAAGAGCCCCCATTTGACTCATGACT[T/C]GCCATAGTAAATTTGTCGCTAGTGATTGGTTCAAAGAAGTTATTGATGTTGTCGTGGTGA
2914	CaSNP4987	TATTGGCCTTATAAGAACTCTATTCACTACATGTGTGGCAGTGTTTATAGCATCAGCCCA[T/G]AAATACTTAGGAAGATTTGAGTCTTTAATCATAGTTCTAGCTAGTTCCTCTAAAGACCTA
2915	CaSNP4988	TCACACCGCATTGGTGACTGAAGTATAATTATAAATCTATTTTCATTGAGAGGACAAAGT[A/G]TGTGACTGAAGTATAACTTCCCACTCTCAAGCACTATAAATACACACATCCTTAAATAAG
2916	CaSNP4989	TTTATTTATTTATTTATTTTTCATAACCCTCAAATTTAAACAAACAAGTTGAGCACCCCT[A/G]CACTTATGTAAGGAAAGACTCCTTTATCCCCCAAGATTGAAGCAATTCATTGTTTTTCAC
2917	CaSNP4990	TAAGTGGATTGACAATGTTTTGCTTCATTTGGGCTGAATAGTGAGTAATACACTTATAAT[C/G]ACAATATGAGGCTTATGTTTTGTCCACTCACACCAATTGGTAGGTCCTCGCGGACGTCTC
2918	CaSNP4991	TTCTTGAAAGTAAAGAGGAAAAATGCAAAACCAAATGTTCATATTGTGGTAAAATTGGAC[A/C]TCTAGAATTTGCATGTTATTTTAAGAAAAGAGATGGAAAAAATCAAGAAGAAAAGGATCT
2919	CaSNP4992	TACCTACAGATTTTCACTGTCGCTAAACGTTCAATTTCTTGTAGTGAGTGAAGAAGCAAG[T/C]TGGTTGAGATGTTTGCTAGTTGAGATCCCTATATGGGAAAAACCTATGACAGATGTGTTG
2920	CaSNP4993	AACACTTCATCTTTATATCTCCCAAGTTTGAATGACACTTGTAGGTATTTTGTCGCATCA[T/C]ATCATTTATATTTAAACTCTAGACCTTTAAGGCTTTCAAGTGAGTATATATGTTTGATCC
2921	CaSNP4994	AAGCAATATCGCACTGATGAAGCAACTTCTTGGGCAATAGCATATACAGAGCGTCTACAA[A/C]ACTGAGGGTATTTATTAGGTAGATTGCCACGTGTTACCCAAAATCCACCTACGGAACCCG
2922	CaSNP4995	TTTAGAAGGCAGACTTGATGAGCTTACATCTTTAGTAAAAAGATTGGCAATAGGTAAAAC[A/C]CAAGCAGTGGTCTGTGGTATTTGCACTTCTCCAGAGCATCCTTCAAATTCATGTCCTACA
2923	CaSNP4997	TAGTTAGAATATTCTCTCCTAATTCTGATCAAGTGAACCACGTCTCATATGAGTCGTCAT[T/G]TATCGCACTCCAAAAGTAAAGACTAAGTACATGTTTCTTTGGCATTCTTGGTCATCAAAC
2924	CaSNP4998	CGACAACTCAGACGAATTACCCCGACGCATGATCAGATAACGACTCCCAACCTACAGGTT[A/G]ACCTACAATCTATAACCAAAAGCTCCACAGCCCCAAAGAAATCCAAACCAAAGCTCTGAT
2925	CaSNP4999	TAAAAGATTCACCGCAGTTAATTTAATTTATTTATCGACGAGTAATTCTAAACGGCATCA[A/C]AATATCTTTTGATCATTTAAACTCCAAATTAATAATAACTTTGGCTAAAAAGCCTCCGAG
2926	CaSNP5000	ACTTCAGATGTGGAAAAGAATGTCATATGGCACGCAAGTACAAGAACAAGACAAGCCCTG[T/C]TCAGTAGGATAACTTGACAGAAGAGCAATACATTGCTATCTATGATAACTGAAATCAACA
2927	CaSNP5001	CGGTTATTGTAGCAGTTTGTGCTAACAGAGTTTTTCACTTGAATCTTTAGTGTTGTTTAT[A/G]TTTTTCTTACAAATTCATTTACTATTTCAAATAAGACTGATGTATCAAGGATTTAATATT
2928	CaSNP5002	GATGATAATAATGAGAGCTAATTGGTATGAATTGAAAAAATAAGAAAGTCCCACATAGGA[A/G]GGATGATACACACTAATAATATTTATAAGGTAGATGTATGACAATTAGAGTGTCTCAGAA
2929	CaSNP5003	TTTGTGTTGTAGTCCCTTTGGTTTATGAGTGGTCTTAAGTACCATATTTTTAGACTGACC[A/G]TTATAGTTATATTGAAGCTGTAATTCAAGAACAATTTTCTACAGTGTAGTAGAATAATAA
2930	CaSNP5004	CGCTCGCACACACGCACATTCGCTCTTCTCGACCATTAGTTGATCGTCACTTACTCTTCA[A/G]ATGTCTTCTCTCTCGTCACTTGCTTAGTCGTCTCTAAGCTCTCAGTCTTTTCTCTCTTGT
2931	CaSNP5005	GTGTGTAGACTATAGGAAATTGAATCAAGTTACCATTAAGAATAAATATCCTTTTTCTAG[T/C]ATATATGACCTCTTAGATCAATTGAGAGGAGCTTGGGTGAGCAGTAGAAACACGTGAGGA
2932	CaSNP5006	TTCATATTTAGAAGTATGAGACTTTTATAGGAATAAAAATTCTATATGTACTTGTATTTA[C/G]AACAAAGATTGTATTCTTTCTCAAATGAAGAAATTTCTATCTCTTAAATATGGTTTGTAA
2933	CaSNP5007	TTGAAGGAAAGCTGCAGGTTACAATGGCCAAGCGATGAAAACACATCCAAAATAACAAGA[A/G]CGCCTGTAGCTTTGATGAAAAAGAAGGAAACTAGGAAATGAGTGAAATTTGCAGAAGCCA
2934	CaSNP5008	TCCTACGTCGTATTCTTGTGCAGTTAGGGTTTTAACCAATCTTGTGCATCCAATACGTTC[A/C]TTTGTGTCATTGGATGAACATTTAGGTCATTTTCGTCCTCCCTAGGCTTATTCTTGTCCA
2935	CaSNP5009	TCTTGTTTCAAAACCTTTAGAAAATGTCATATCTAATTTTGATAAATATGAAGAAGCATT[T/C]GAGACATTCTAGCTAACAATAGGAAAGAAACACATATGCTTATATGATTTATGAAGTTAG
2936	CaSNP5010	ACTTTGTAGTCCATATGTAACCATCAACATATTAAATTGATTCTATGATATATATGCTCT[A/G]GAAGTAGGTAATTATTTCTATTGGCAAATATGAGACTTTCAAGTGCATGTTCAAGTATTT

2937	CaSNP5011	GTTCCTAATCTTAAGATCCTCGTCCGAATGTATAAACAAGATGCCTTATTTTTGTGAGAC[A/G]TGTAAGATTACATTAAACCAAGAATGTTCTGGATAAACGAGATTGATTCTAAATAAACCA
2938	CaSNP5013	AAGTATTTTAAAGAATATTTTTTTTTGACAATTTTATGTGTGGATGTGTATTAAATACTT[A/G]TGGTAATAATGTTGTATGCATTTGACTAAATGATAAATGTGTGCATAGTTATATAGTTCT
2939	CaSNP5014	TCTCCGTTTTCACAGCTTTAATCCATGAACGTTCTTGTTTACAAAGAGCAAAATTGGATG[T/C]AATTTAAGATTGATCTGGGCTACAAAAGTACATCTATCTACACTAAAAAACATCAGTATT
2940	CaSNP5015	CCACAATTCCATCATTCACACATCTATCATCCCAAGACCACTTATGTCCCACACTATCTG[T/C]CCAAAACATTTCAAAAATGTTTCCTGCATTTCTCTCACCTCAGAAAACTGCATCTTCTTC
2941	CaSNP5016	CTTCAGGAGTGAACAGACCAGATTCTCCAACCATCTAAACAAACCATATAACACAATCAT[T/C]AGTTTAAAATAAAATTTCTTTCATGTAAGATAAAATGGAATAACATTTCTTTGAAGTAAG
2942	CaSNP5018	AAGATACCACAATTTTGGGCAATCGTCGTCTGAATTAGTGATAACCATTAGTAGTACATC[A/G]TCTGAACCTAAGTCATCAGCTACCATATGAGCTTCATCATCCTCTCTTTATACCTTCTTG
2943	CaSNP5019	ATTTCAAGAGGTATTATTTTATTTCTATCTTGAAAACTCCTTATATTGAGAATGAATATG[T/C]TGGATTCTTGAATGCAAAACATGGAATTGTGCTTCACCTACTTGACACATTTAGTGGTGT
2944	CaSNP5020	TTTTTTTTCTGAAACAAACACACTTATTATTACCTCTTAAACAAATATGAAACGACTACT[A/G]AGTGATTGACTAATTGAACGGATATGGAATGATTACCAAATATGGAATCGCTACCGAATA
2945	CaSNP5021	GATGAATCATCGATATTATACACTTGAATGCTTATGAGGTTGTGATAATTTGATTGCTTG[C/G]GTGTTCTCCTCGTGTATTTTATACTGTGTCATAATTTCAAACTCATTATTCGTTGTTTGT
2946	CaSNP5022	CCCTCAAATGCTCTCAATTCTCAAACCTCACCAAGGATGAAACTATATTCCTCAGTCTGG[A/G]ACATTTAAATACGCTAAGATTTTGAACAAAGAACTGCATCTCAGAATGGGCTACACATAT
2947	CaSNP5023	AATCTTTAATTTATTTCTTAAATACATTAAAATTACCAAAATCACCAAACATCAAAGTAG[A/G]AAATCCATTCACTTAATATAAAAAAATCTTGTATGATCACAAGATGTAGTAACACCATTT
2948	CaSNP5024	TTAATTTTTTAAATATTTTTAATAATATTTTTAATATTTTCTGCACTTTAAAAAATTATA[A/C]TTAAATAATCTCTAGTCAGAAAATTCCAATTTTCAGTGGGAAGTGTGGTTTAATTATTGA
2949	CaSNP5025	GTGAAATCACCAATGGGTATCTAGTGGTAACAAATGAGCAAAGTTCTACTTCTCATTATA[A/G]GATGTTTATCAAGCTATGCAAGCAAGAGGTGAATGCAATTGAAGATACCTCTGTTGATTT
2950	CaSNP5026	TCCTCCATCTGATTAGCACTCAAAGTTAAGATAACTACTATATACGAGAATAACAAATGA[A/C]AACTTGGCAAAGGCAACAAAGAGGTCTATGAAAATGGCAACGCGATTAGGGATCGATTTT
2951	CaSNP5027	CGACGTCTTTATGGAGTTTGTCACCGTTTGCTGAATAACATTACTCCGAAGACGATAAAC[A/G]ACTTTATTTCTTCATATCTTGGCATTGCTTGTAGTGCACTTTATGATTTTAATTAATTTC
2952	CaSNP5028	TACTAAAACAAAGGGTAATAACAATCAACCTTAAGTAACATAGGCAACAGTAAAGTAAAA[A/G]GTGCATAAGTAAAGTGACCCAAGCAGATTTATCTTGGTTCACCCCAAACTTGTTAGATGC
2953	CaSNP5029	AGATCCCTTTAATTTAGCACTATTGAGTAAATAGAAGTGCAAACTTTTGGTTGACACTGA[T/C]GTGACCTGGTATAAGCTATTGGAGTTCCAAATATGGCTCTATGAAGGCTATGGTGCAAGA
2954	CaSNP5030	TCAATGCTGGACAGGTTACATCAAAGATGCATGTCATTGAACTTGGTGCCCAGCCAGGTA[T/C]AAGCATCGCTAGATTTTGCATTATCAATCAAATGGTACATTCCTATATGGTATAAAAATT
2955	CaSNP5031	GATAGGCTTAATTGCAGTTTTGGTCCCTCTATTTTAGCTGAATCGCGAAAGTAGTCCCCC[T/C]CATTTTATTTCTCTCCAGTTTTGGTCCCTCAAACAGAATTTTGATCCAAAACTTGATGAA
2956	CaSNP5033	AGTTAGAGTAGAGATATGGCAAATGATCTAAATGTGAACTTATACATGTATGGTATTGTG[A/T]TGTCGGATAATCTTATGTAACTGAGTGGGATAAAAAGCTTGATTATTGACGTTGTTGTAT
2957	CaSNP5034	TAAATAAAAAAGTATGTGTACATTCTCCTTACCTCTCCCAACTTTGCATTTATCACAACA[A/T]AAATTTGCCAATGCATTTTGATGTAATGATGTTCTTAAGTTTAAGATGGTGATTATAGCT
2958	CaSNP5035	CGGAAGAGGCCCTTCATAAATGTGATACTTGAATTGCAGAAGCTGAAGAAACTAGTATTT[C/G]ACAGATTTGTTGAGTAGCCGATGTAACTTCTTAAAATTAAGAGTAGATGAAGGATTTTAG
2959	CaSNP5036	CTTAAAACCATATTTTACGAAAATCAGTTTTATAAAAACCAATTCAATCAAAATCACTTT[T/G]CACCACCTACATTGGTCTGAGTGGAACTAGACTCAGACCCTGTTAAGCAAGGTCTCGGGT
2960	CaSNP5037	CACGCTGACAATTATGACTTACAATGAAATATATACTGAATCATAACTCTAGTGAAAAAT[T/G]ATTATGATATATGAATTTAACAAGCATGGTGGTTTGCGATGACTTACCCCTTAAATAATC
2961	CaSNP5038	CAGCAGCTGATGATATTCTGGGTTGGGGCACGCCCTGCACAATGCCATCAACAATAGTAT[C/G]AATTACGTCAACAATTGGTATTATTAAGATATATCAATCATAGACACATTATTCTATTCC
2962	CaSNP5039	AGAAGTTACTTAATCCAAGAAATTGGTATTGATGAGTCTAGGCTTGTTCCTTTAAAAACA[T/G]CAGAAGAAGCTGCTAGAGCACTTGAAAAGGGTCCCCACAATGGTGGTGTTGCAGCCTATG
2963	CaSNP5040	TAAAACTTACAGGTCGGAGAGTAATACTGAGTTCAATCTCTTGTCCGCCTCTCAGAACTC[T/G]GACTACTGCTTTTTCATCAAGCTTCTTCATAGACACCAAGTGATCAAATGTTATCCGTTC
2964	CaSNP5042	TTCCATTGAGTCAGAATGTATAGGAATAAGTCCGATTCCCCAAAGCATGTATAATTTCTG[A/T]CTGAACCAACTAAATGTATTGTACATATAAAGTTTGGATAAAATAGTTCACACGCATTCA
2965	CaSNP5043	GCATCAATAATTACAAATAATCTAACAAGAAAAACGATGAATTGCTTCAGTTCTCCTCTG[T/G]TGTTCTGTCTTTTAACAAGAGAACTGTTTTTCCTTCTTTTAACAAGAGAACTACGACGAG
2966	CaSNP5044	AACTTCACTCATCACATGTTGTTTGAAGGCTTGGTCGATAGCTTGCTGGGAGGCTTGTTT[T/G]GATGATTTACGAGAGGCTTATTTAGATGATTGCTAGAAGGCTTGTTTGGATGTTTGGTGG

2967	CaSNP5045	TTCATACACAAAATGGACAGTGTCATTCATACAGAGTTAGCATAAGGTAACTTGATGTGC[A/C]AAATCATTTGATTGCTTGTGTGAACAAGTTTTAATGTGTCAATGAACTCCTTCGGATCAA
2968	CaSNP5046	TTATTTTATTCATAAAAATATTATTTTAACATTGAGATTATCAAATTGTCACAACTCATA[T/C]GTCGTAATTATCTAAGATTAATAAGATGTCTCATCTAACACCAAATGTTAAGGAAAAGTG
2969	CaSNP5047	TGCCAAAACAAGTTGTCCATGTTCCAATCCAATTTGAACCCAATATTGAGAAGGTTCCAA[A/G]TGAAGATTTAGTGGAAGAAAAGATTGAGAAAATCAAAGTCCCTCAAATAGGAGAATCTGA
2970	CaSNP5048	CTCTTCTCTTCAATTCTCTCTCAACTCATAGATTCATTCTTATTCTTTTTCTACCAAATA[A/C]GAATCACGCAGTCATTTTCTCTCTTTGGAGAAAGCAATTAAATTATTGGTTGTTATGTTT
2971	CaSNP5049	CTTTATTTTACTGTGTCTTGACCTGACATCATATTTAGTGTATGTGTATGTACAAGATTT[T/C]AAGCTGACCCTAGGAAATCACATCAAAAACAGGGAGATTGTTGGAACCAAGTTGGTTTTA
2972	CaSNP5050	TTAATCTTACATTGTGAAGTCTTCATCAAGCATGTATTGAATATATAGTATTATATCATC[A/T]AAGCTACTTTATCATGACATGTTCATATAATGAAATTTTGATAAGGGCCGTTTATTTCAG
2973	CaSNP5051	CGTCCAAATTAGTGCCACCTCACTCCTCACTGCTATCACAATTTTGGTAATACTAATTGG[A/T]AATACACGTCTATAAATTAGGTATGTTCTATTTTTTCCTAGGTGGAATGAGTTTCATGCC
2974	CaSNP5052	ATTTTTCGTGTCGTTGTGTCAGGCCCAGGATGAGCATCTTGCTGAAATCAATTACCGAAA[T/G]TCAAAGAAATGGCAAGGATAAAAGGTCAGAGAAGCAAATATAAATGAATAACAACAAATA
2975	CaSNP5053	TTGTACTGATTGCTATCATTACAAGTCTTTGATTATAGGCACACATGTTTATTTCAGGCT[T/G]TTGTGGATATGAATTAGCATGTACTTGACAATGTTATGCTTTGGTTTCACATTTCACTTG
2976	CaSNP5054	TTTATTTCCTCATTTGGCATATGATGATTAACATGTGTAGTAGGGTTATGAGTGATTATA[C/G]TAGGGTTATGAGTGATTGTAGTAGGGTTATGAGTGATTGTAGTTGATATATTATGCTCCA
2977	CaSNP5055	TATATATAAATGCACGCACACGGAATATTGAGCCATATCCCAATTGTAATAGATGAGTGC[A/T]GAATTGACTCGTATTGAATCAACCATGGCAGGATCTAAACTAGGTAGGTACAAGTCTTGT
2978	CaSNP5056	GTTGTTAACCGGAACAACCCATGTATCTTCTCCATCTTCAGTACTAAACAACATCCTTAA[A/T]GACAAAGGTCCACTCGGTGGAGAGGTAGTTGTCCAGACTGCACCGTGATCCCGATCCAGT
2979	CaSNP5057	ATTTGGATTAGAGTTAACGGTGTTTGGTGACCTGGAGAATATCCTGCTCTATTTTCAGCA[A/T]TTTCACCCAAGCAAGAGTAAAATCTTTTTGCATTAGCCTGGGTTTATTCAGATATTGCTT
2980	CaSNP5058	CACTTGAATTGACCAACTCAAGTTTACCTCAACATGATGAGTGAGTCCTTGTTTCACACC[A/T]TATGAAATGGATGCAAAGAAATTAAGCAAGAAGATGGAAAGAGGTAGTATTTGAGGAAGT
2981	CaSNP5059	TGAAATCAATGCCAAAACGACTGTGTGAATAATGGCTATATTATACCATGATTATTTCCC[A/G]CGCTTTAGGTAAGTTAACTTGATTTAGTATCGAAATAGACCTTTTTAGAGTGTGTTTTCA
2982	CaSNP5060	TAGTTTGATATGAACTCTTAAGAGGCATGTTGAGTTTGTGCACATCAGAAGCACACAAGG[A/G]CATTAGAGACATACTTCAAATTCAGTCTTCAGACCCAATTATTAGAGGGAGTCTTCAGAC
2983	CaSNP5061	GTACAATCCATTAGGTCTGATTTATGAAACGTGTGCTCTGATATTTAGGATTTGACCACA[T/G]AAACATGTACCACGATTTGTTTTCCACCAAAACATGTGCCACATTTTGTTTGACCACAAT
2984	CaSNP5062	CAAATCATATACATATATTCCTTTACCATCACAATTTCCATAGTTTTTATCCGACTTCCA[C/G]GATCGATGAATCTGCAAGTATTCCTCAACGACTTTAACAGCATTTGCAGCCTCATCATCT
2985	CaSNP5063	AACAACAAGATATACTTTGTCAAAATAATATTATGACGTGACACATAATGAACTATGATT[C/G]AATGTATGTATTAGATTTTTTACATTCTAGTGAAGCCTTTCAACTTTGTTATTTATCATA
2986	CaSNP5064	GATAGGCTTTAATTTGTTTTGTTCTATTTCTTTCTTTCTTACTTTCTTGCTGTGCTACAA[A/T]ATACAAATCATGTTATGCTCAACTGTGTCCAGATAAAGCTTTTTGTACATCAATTATCAC
2987	CaSNP5065	GAGGAAGAGTTAAAGGTATTTATTTATATATGGAAAGAAAATGTTGATGGTGATTTTGGG[A/T]GGGGTAAGAGTTGGAGATTGTTGATAAAAAGGAATAATATAAAATATTAAAATGAATGTG
2988	CaSNP5066	TTCTGCTGTTGCAAAAATGCTGTTTCAATTTGACCAATCAATTCCCGATTCTTCAAGTTC[T/G]TCTATGACACATATGAATGGAAAAGGGAAGGAGAAGGTTATTGAAGGGGGACCTTTACCA
2989	CaSNP5067	ATGAATCTTAAGTTATTGTATCAATGTTTCTAGTTTGTCATGGCATTGTCAAATCCATTG[A/C]AAGGTGAGCGTAAGGCAGGCACAGTTGGTAAACCATTGCCTGGTGTGCAGGTTAGTTGAA
2990	CaSNP5068	TTTGGCCAAAAATATATATGCTAAAATGACGTTTCACAAAAGGACGAAGAGAAACCAACA[T/G]AACAAATGGACCCGTGATGCAAAAGAGTTCTACCATCCGAGATAAAATACAGACAAGGAG
2991	CaSNP5069	TGAATGTTGTTTGCAATGACCTACTGCTTCAATACTTTAATTTGCCAACATTTGTTGATC[A/T]TGCGAATTCCTTTTTTTGTTCCAGATCGGAAATTTTCCCATGCTATTGATCTATACACAC
2992	CaSNP5070	ATTAAAAGGGAAGAAGAATCGCAAGATTAATGTTAATACTGACAACAAAAATCTCTCACT[T/G]GGTTCCTTGGAAGAATTGTCTAAGGCTCGACGTTCAGTCCTTGATCCTCCTTGGGAAGGA
2993	CaSNP5071	TCACCAACTATGACCATAATTAAATAAATTATTGATAGATAAAAATTCTGTTTAACTTGA[T/G]ATTGTTTAGGCTTTTGAGGTGATGATGACTTTGTTGAATGCATACTAAGTCATAATTAGC
2994	CaSNP5072	ACCATAATGATGGTAACGTCCCAAGAGACACCAGAGTCCACTATAGGTTCTATAAGGCAA[T/C]GCTCCACCTAGAAGGCATGGTTAGAGATCTTCGACTTTGTAACATAAAAGGTGTTCCCCG
2995	CaSNP5073	TTAATAGTAATACTAATGCATAATACTCAAGGAAGAGTGAGGGTATTAATTCAGCTTTTT[T/C]CTTTGCTAAATCTTACACCTCTTTCTTATTACTACTGGACCACCTTCCAATTCATATCAA
2996	CaSNP5074	TCCTAATAAAAAGGAATGTAAATACAACAACATTTGACAGAATATTATCAAATGTAGTCT[A/T]AGAAGCTTTAAGGATGTCTTTCCACATGGTCTACAACAATAATAAAACTGCCCAGGAGAC

2997	CaSNP5075	TGATATGAAGAGTTATGTGAATGAAGGGTTGAGAGTGTTGATCCATGAGAAGAGTATAGT[C/G]GTGGAGGATTATTTGGATAAAAGATGGGAAGAGGTTACTGGTGAGGTTGTTGATGAAGTT
2998	CaSNP5076	GAATTGAACTGGTCAAGTGGTCCATTTGCATTATTATTGTATGAGTTTGATTCTGAATCT[T/G]CCCACAACTTATAAAATATAGTAAATGAAGAGCAAGTTGTGGCTACATTATTGAATTTCT
2999	CaSNP5078	TCCCAGCCAGCATTTCATTCGCATGAAGGTAGCAATGTTTCCATTTCTGAACATGTTACT[C/G]TTAAACTTCTTGCCACATCTTCCTGTCATATATTTGACACAATTTAATCTGTTATGGTAC
3000	CaSNP5080	TTAATATCGACTTCTAGATACATCCCACAATTTTCTATGATTAAAGCACTTTTAGATTCT[T/G]ATATGGAAATTCTACATGGTTTTAGAATTATCGAATTTCAAATTAGTAATAACAACTAAT
3001	CaSNP5081	AACTCAGTTTTCATTGGCCTAGCCTATTCATACATCATATAAAAGGTTTTCTCTTCATTC[A/C]TAAACACAAAGATTATTTTACATATTCCTCATACCTACACATTCAAGATCATATCGTAAA
3002	CaSNP5082	CAAATTCCCACAAATCAAAGCACATCCATGGCATAAGGTATTGTAAATATTATTTTTGCC[T/C]GTCCATTTATACATGTTATTTTGTAGTGCTGCTCATATATATATGAATCTTCATCACCAG
3003	CaSNP5083	AAATTGTCAAAAATTAAAGATATTATTTAGCGTAACACAAATTAATTTAGATTTGTCTAA[T/C]ATAACCCAAACTCAAGTTAAATTCAAATAATCAAACTTCTAAAGATAATCTAAATGTTGA
3004	CaSNP5084	CCCATGTTTAATTATGGTCAATATCACACATGTCACTATTATGCACCTATTTGGGTTTGC[A/T]TATTCTTGGTAACAGAACAATATCTTAGTATCTACTATCACCAAGTCTTTGCTTGGCATT
3005	CaSNP5085	AGACACTTAGAATTTGGTGGAAAAATTTATAAGAAAAACTTTGAATATACCTGTAGTCGA[T/C]GTGCAACCTCAGCACACCCCTTCATGCTTAGGACTAGACATTTGGAGTGCGGACAAATGC
3006	CaSNP5086	GAGGAAGAAAATAAAACAGATTGTTTACTTTCTATTCTCTCCTTTTGCGTACGAAGTTTC[A/T]TTCTCTCTGATCACTCAATCAAGGTAATAATATTCCTAATCTCACAGTCTTTTTCACGAT
3007	CaSNP5087	AGTGTTAAAGGCCCTAATTACAGTGGTTTCATACCCAAAAAAAAGATAATTTTGATTTCC[T/C]CATTGTAAAATTTCATTGATTATGTGTTGTCTGGTTTATTGTTAATTGCAGGAGGTATTT
3008	CaSNP5088	GTCAAACTAATATACTTATATCTTTTCAGGAACATGGGTTGATGTAGAAGAATGGAGTGC[T/C]ACACTATTTACAATGCCTAAAGCATGTCCCATCATGTTATCAACAATAGTTTCACTTAAA
3009	CaSNP5089	AGATGCAACAAAGCGTTATACTCCATATATCAATTATACAAAGCTTCTATTTAAGGTATT[A/T]TAACAACTACAAACTCTTTTTCTAGCATGTAAACTCTTTTTATCGAGAAGTACATTTCAA
3010	CaSNP5090	AAGTGAGAGATAAGCTAATGAAATATCTTTCCATATGTCAAGTATTCCTCCACTCCAATT[T/C]GGTTTATTCTCTATAACCCCTTGTTGATCTTTGGATGCAAATGCATATTTTTTCTCCAAT
3011	CaSNP5091	TGAACATGTGTTGACCATATAGTCGTGCGTTGAAAATAATAGATCTTACGCCAACTCCAA[T/G]AGTTGTCCAAGCACTAACCCCTTTTCTTATGAAAGTTTTAAGGCGGTACCATGGAATTAT
3012	CaSNP5092	GCTGTCTAATTAAATCAAGGAAAATTGTCCTAGTACTTGAGCAAAATAAACTTATGTGTT[A/C]TATGAAAAGGGTGGAACCATATGAATGAAAAGGATTAAATGGATTCATTGAAGAAAGACA
3013	CaSNP5093	ATTGCAGGGTTATAAAATATGATTGAGCTGTTATAATCAATGCTAAATACATATCTTTGT[A/T]TTGATAGTGTTTACAAATGAACACTGAACACAACTGTTACGGAATGCGCGTTTGAATTTT
3014	CaSNP5094	TACACCTGATATTGGTTTTGATTGGACCACGGGTAAAGGAAACCCTTTTAGGTATTTCAC[A/T]TATGGGGCTGCATTTGCTGAGGTTGAAATTGACACCTTGACTGGAGATTTTCACACTAGG
3015	CaSNP5095	GGAGGGACTTGGGGGTTGAGGAGAAATCTGCTTCTACCACAACATTGTCTTCTTGGGTTG[A/T]GACTAAACAAAAGTTGCCGGATATGAATGTGGAGTTACCGCCATTGATTGCGAGTTTGGA
3016	CaSNP5096	GACACCAACACGACACCAGCGCATGTGGTTACATTCCATTAGTATTTCATTTTCTCAAAT[A/T]TTTATTGGTGTTTACATGTCAGTGTCATGTTCAATATCCATGTTTGTGTCAATGCATCGT
3017	CaSNP5097	AACATATCATAGGCAGCATGACTAGAGTCAAGCTCTAACTAGAGACATCATGACAAGGGG[T/C]GCATGGATTAGAAGCAGCATGATTAGAGACAACATAACCAGAGTCAGAGGATATGTGACC
3018	CaSNP5098	TTATTATTAAACAAATATCTTTTGGTCATACGAACATACGATGTAAGCCGTAAGATTCAA[A/C]ATAAAAAATGAAATAAATCTCAAACTTGTTTAACTCTTACACTAAGAAACAGAGTTTGCG
3019	CaSNP5099	TGCAGATTCTTGGTTAGATTCTCTTTCATGGTCCCCATCCATGGATGCAAGCTAGCACAG[A/G]CAAATATGAATGGTACTAATTCCAAATTGTTCAATGATTTTGTCAGGGGATTTTCTAGAT
3020	CaSNP5100	GCGAGGCCCAATATAAAAACCAAATAGGCAACAACTAACTGATAATTGCATTTCTACTAT[A/T]ATATATATTGCTGATTGTAACAGAATCAAAGTTGCAATAACCTCACTTCATTTTCTCTCT
3021	CaSNP5101	TTTTATGAGAACAATAAATTTGGATTAATGGATTCCTTATGTATGCAAAAATTTGAAGAA[C/G]AAAATTGCAGGAAGGTGCTAGAGGAAGTGACTAGAGGAAATTCATTAGAAGAGGTAACCA
3022	CaSNP5102	CTGCAAACAATGGCACAATTGTAAATGACCTACTAACAAAATTAAGTGTTCACCGTCTCC[A/C]TCCAATGTATTTAAGCTTGAATGATGATTTCCAATCTGGCCACTCTATTCTTTCAAATAG
3023	CaSNP5103	TAGTACACAGTGATGTTAAATACTAGCTATAACGGATCTATAGCATTATAGCAAAGCAAA[A/C]TTTGAACAAACCGCTATTTTCTGCTATCTACGATTGACAAAACTGATTATAACAAGAGCA
3024	CaSNP5104	TAATCCAAATCAACACACATGCAATTACATATGTTCCTCTGTTTTGCACCAATATAAATT[A/C]TTGTGTTTTGCTTAATTCATTCTCTTCTTTAATCATCTTTAATTATATTCATCCTTTTCT
3025	CaSNP5105	ACGCAAAGGATACTAACAATCTATTTTGCTGAATTCTGTTTGCACACTAAAGGATACTAA[A/T]TATTGGTGTCATAATAAGACTAGGAACAAGGATCAACATCTTCTATTAAGCAATGTTGGT
3026	CaSNP5107	ATGTCATGCAACTCAATCTCCCTCTGATAACATTACCTTCTACCTTAGTTGTATCTATTG[C/G]AGCTAAGAACCAAATTGAAACCTCTATGGCATGTCTTCAATGTCTCACTAGCCTATTTGA

3027	CaSNP5108	TCATACACAACTCAACCCCCTCTCCGAACTGATGTTTGCACATTCAAGAGTTCCATATAT[T/C]TATAGGTTCCCTTAGATGAGTAATATCACGTGGGAGGTAGACCTATGTACTTCATAATAG
3028	CaSNP5109	TTGAAGAAAATCCAAAAGAAATATAGATTTATCAGAATTTGTCACCACCTCTATTTGATA[A/G]GGGGTATAAAAGGTATATTCTACCATGAAAAAGGATAAGAAAAGTAGAATTCTCTTCCAC
3029	CaSNP5111	GGTACCTGCAAACACTATAACGCTCAAGCAAGTATTTATTCGAGGAGAGTAAGAGGGACT[A/G]GAATGAGAAAGTGTGTGTACCTTTTTAGTGATGGAGTCACTATTTTTATAGACTTGAATC
3030	CaSNP5112	AATAAGAGAACATGTTCACTCACACACTTAAAAAGCTCAAACATATATATATATAATCATTT[C/G]TTACACAAAAACTGTTTGTGAGCTACATACACTTAAGCGCATGGTTAAATAAACCATATG
3031	CaSNP5113	AGATTATTCAGAAACAATATTTAGATTTTACTCATGCAAGTTCTAGTTTGTTTGAAAATT[T/G]AAAGTATTAAAATTAAGAGTGGTAAACAAATGTACCGGGAATAAGAGTGGTCTCATTTTA
3032	CaSNP5114	AGCTTCTGCTATGGCACTCACAAAGCTTGTCCGAGTAGCTGACTCTGCCACAATTAATCA[A/G]CTATTAGCATTGCTCCTAGGGGATTCTACAAGTTCGAAGGCCCATATTATTCGGGTTTTA
3033	CaSNP5115	TGGGGTGATAGTGAACACAGACTAGCATATGTATCAGAAACCACAGTAATATTCAAGGAC[A/G]TAGTTCAAGTAAGAGATGATCAATGGTGGAAAGCACTTTCATCAGGTTACATAACACAAT
3034	CaSNP5117	GCTTCCTAGGAGTCTGATCCCATCTTGTAGAGGTCAAGGTTGACCTGTAATTTACATAAA[A/C]AACCCACGTCTCATCCTAAGTTTTCTTGGGTTAGGGGTGATTTTCACAGAGAGGCAATCT
3035	CaSNP5118	TATGTCGCCGCCCATCATTATTTTCATTAGCACGTGTTGTGCAGAGTCTAAAACTGAATG[A/C]ATGTCAGAGCAAGACAACTAGTGAACTGATGGAACCAACTTTTTATCACCGGATAAATCT
3036	CaSNP5119	GTATATATGTTCATTTTCCATGTGTGATACTATGTCTCTGTCTGATGTCGTCTGGTTTCT[A/G]CAGATGGCAAAAATGGATCTTAATGGGGATGAAGAGAAGGACAACATAGAAAAAATATTT
3037	CaSNP5120	CCCTATTATTTGTGAACAGGCAACTTTGCTTGGCTTCGAAGTTTCAATGTGATCAAATTA[A/C]TATCAATCAAATTTTACTTACCAGCTAAACTTCAAATTATCAAAATCTCTTTCATCTAAA
3038	CaSNP5121	GAAACTAAGATGCCGTGTAAATTATAGAGCATTGAGATATTCAGCTCCAATTGAAGAATT[C/G]GGAAACATATTGGTATCTAGAATGCAGCAGAATGGAAATCCTTATCTTGCCCTTCATTTA
3039	CaSNP5122	TGAAAAGGCGCGACAGGCATTTGAGCGTGTTTTGCAGGCAAGTGAATCTCCTGTGTCTAG[T/G]GGTTCAACCATTCAGCTTAATTGCTAGTACTGATGTGTGTATGCTTCATGATTTATTCTT
3040	CaSNP5123	TTCTTTCAAAAAAGTCTATCTCTTGTTAACCCTAATTAGATCACTCTCCACTTAAATAAG[T/C]GAGACACATGTACTATAATATTTGGGTCTTTCTCCACATAGGAAGGGAGCCCAATACCCA
3041	CaSNP5124	TATGTTCAAGATAGAACATTTGAACTTCCAATGCTAAACATTTTGATATATGTTATGAGA[A/G]TGGAATTCCACTTTATTTTTGTGTGGTTAATCTTGTATAACGTTCATGTCTTAGTAAAGT
3042	CaSNP5125	GGCAACTTAGCCAAAGGCATCATAGCTCTCAGTCAGAGGATACGCGTCAAGAGGATGTAA[T/C]AGAGGGTACGCATCTAGAGGGTGTTGAACAGTGATCAGAGTCAAAGGAATGTGAACAGTT
3043	CaSNP5126	GAAAATTCATAATAAATTAATTTTTGACGGGATTCATCATATGAGGTATGCAATTGTCTT[A/C]TTTGTGTTCAATAATACGTAAGTGGTTCGATAATCCTACGACCTCAATTTGCCCAATGCG
3044	CaSNP5127	AGAAGAAACTCAAAGCAACCATTTTCAAACCCTGTTAGCATTTTTTGTAACAATTAGCCT[T/G]TTCACACACACTCATAATCATTTAGAGCCAAGCTTCAGTAAATGGCCTTTCAAGCTTTGC
3045	CaSNP5128	TTTCTCTCATTTTCTAGTGATTGAATTTGCACCGGTCTACTCAGGTAAGAAAGGAACCTC[T/G]GATATTCGACTCAGTTCTTTGTTATTCGCTTAGTAGTTTTAATTTAATATATCACAACAC
3046	CaSNP5129	AACCATATGTCTTCATTCAACAACTTTGCATAATGCATAACAAGTATGCATCATTCATAT[A/C]TTGTATTTATACTCAACAACCTTCTAAACTAGTTTTCTTCAATCCTATTGATCTACACAT
3047	CaSNP5130	CTTTCATAAGACAAATCCTATGTAAGCTTCTTGTTTAATTCTCTTCTATAATTATCATTT[T/C]ATCTTTGTTGCATGTTTGAAATTAATTTTCCTATAGGTTGTTGACTGTGGAAGAATCATA
3048	CaSNP5131	TTAACAGTGTTTATTAGATAACCTAAAATCAAAATTATTAAGACGGACAACAACTATGTG[C/G]CAACCTTGTTTATTAGATAAACTATAATCAAAATTATTAAGACCGACAACAACTATGTAA
3049	CaSNP5132	ACACTAACTATTTTTACAACCTCACACCACGTGTTAGGTTTGCTCCATGGACCATCTCAA[T/C]GATTAATTTGCCCCTTTTTTGTAACACAAATTTAGGACTATAAATAACGAGGCATTGTTT
3050	CaSNP5133	NNNNNNAGATCAAATTCATGAAGATGGGATTAGTGATAATGATAATGAGATTGGTGATCA[T/G]GATGCTGGTGATTTTGATGATGAGATTGTTGATAATGATACTTGTGATCATGATGGTGAA
3051	CaSNP5134	CTCTAATATTATCAAGATAAGGCACATGTCCCCCAAGCATTGTATTCGACCTAATGGTGA[T/C]GCAAAAAGAACAACTGGTCAAGGAAGTGGATTTTCGGATGCACCCTTAGAGGTTGATGAC
3052	CaSNP5135	TAGAGACAAGACATTATGAGGTGACAAATACATTTAAGAGATGAAACAATGCTATATGAG[A/G]AGAAAATAACATTCAAGAAGGGGTTACACTTTGAGATAAGGAATAATTTGAGTTTAAATA
3053	CaSNP5136	TATGTCTTCTTTAATAAAAAGAGTTTCATATTCTTAAATCTTTCTTTGTGGCATGTCATC[T/C]TTGTAAGACTCCTAATAATATTCAAGTTGGGTGGATTCTTCTTCCTCTTGGCAGGATCAA
3054	CaSNP5137	AAACCCTAAATCATTGTAGTTCACGAAAATGAATAATCAAACGAGAATAATGAAAAATGG[A/C]GAGAAATGAGACTGAAATAAAACTGAGTGGTGTGCAACTCCTACGAGGTTATGATGGTGA
3055	CaSNP5138	TTGAATAAATATAATTAATTTCAGTCAACATTACACTCTAAAATCAAAGTATATAACTAT[T/G]GTTCACATTACTCATCAGAACCTATTCAAATTTGAGGTAGCATAATTAGAGCATACTCAG
3056	CaSNP5139	GCGTCCATCCTACTCTCTTGACAAAGATGAGTCAAGCAAAAGGGTTGATCAGAAATCTTA[T/C]AGAGGAATGATTGGGTCACTTCTTTACTTAACTGTTTCCATACCTTACATTTTATTAAGT

3057	CaSNP5140	GTGCAGAAAATGGAGAAAAAATATTGGATGTTTTAGAGTACAAGGGAAAAAGAATAATTC[C/G]ATACAGTTGTTTTTCCTTCAAAAGTTTGGAACAGGAAACCACATATAACACTGTCACTCA
3058	CaSNP5141	AAAAATAAAAAAAAGAGTAGAAGAATTCATCTTAGAGTTCAAAGACAGAAACAACTGTTA[A/G]AAATATTTTATAAGTCTTTTTTCTTAGGTTGAGAGTTATTATTATCAGCTTAGATAAAAG
3059	CaSNP5142	ATAGAAAATATAAAATCAAAGATTAGAACCTGTGACATGTTGCCGTTGTCAAAACCATCG[A/G]CGTTGTGGTCGCTGTGGCCGCCATCCCCGTTGGCGCGGTGGCCGCCGTTTGCACCAAGGT
3060	CaSNP5143	AAAGGAGGACCACAAGACTAAGGTGGAGCAGATGATGATTATGCTAAAAAATACTGATGC[A/G]TAGATATTTGATTAAAATCAAGTGGATGATTACCATGCGACGACAATGGCATATCTGGTC
3061	CaSNP5144	TGTAAAATGACAATAATAACCCTTTATATTTCATCAGATCCAAATCTTTATTCGTATATA[T/C]AACTCTTTTTACTTTTTGTGTATGTTTGAATTTGAATGATCTCAAAAAATATTTAAGTTA
3062	CaSNP5145	CACTCATTATTACCTTTTAAACAAATATGGAACAACTACTGTATGATTGACTAATTGAAT[A/G]ACTACCGAATATGGAACGACCACCAAATATGGAATCACTACCGAATATGGAATGGCTACT
3063	CaSNP5146	GAAATAAATGAATTGGGTTTGAAGAACCAACACAAAATCCTAGATTTATTTCATTTGATA[A/C]TATTTATGCATACTGTCATTAGAAAGATCATGATAGAATAACTTGTCCAATGTTAAAAAT
3064	CaSNP5147	CCGACATTCTTTGCTTTCATTATCTAGTCTGTGCCTATTGTAACATTAATAGATAAAATG[T/G]TATTACATAAACTGGTGTTGTGCCCCATTGTTTAATCAGCAATATCCACAACTTCATAAT
3065	CaSNP5148	TCAACTTTGATACATGAGAAGGCAATTCCAACTCCAGTTACTCTTTGATCTGGCGTCACA[T/C]GAGACATTTTACAACACTAGAGCTAGTCTTAGACACCACAAGGCAAACACAACTTTATGT
3066	CaSNP5149	TTTATACTATTGGTGATAATTTCTATTTTTGATTATTAGTAGTTGTGTCTCAACTGTTTA[A/T]GTAAGTTTCGCAGTGGACTTCAATTTTTGTCCTCAGTTTTATTGAAAAGACCTTTAGCCT
3067	CaSNP5150	ATACACATGATAACATTTATTCAATCATGTATTTGATGTTGGTGTAAATCTTATAGGCCA[A/G]TATTTTGCATTTGAACTTAGAACTTGTATCATGCTATTTGTTTATAAGTTTAGATATTTT
3068	CaSNP5151	GCTTCAGGATTCACAAGAGACTTTTGATACCACATCTCCACCCTCATTCATAGTCGATGT[A/G]GGACTTAACCACTCACACTTGAACTCAACACAAGTGGCATGATGCAATGCTGTTTCACTC
3069	CaSNP5152	TTATAATGAGCTTCTGACATGTGTACCATCATTCACTATAGGGATTCAAGTCCTACTACA[A/T]CTGTGGTCGAAACAAGAGGGAAATTGTGAGGCATGGGAAGGTTTTGTTTCTTTTAATGAG
3070	CaSNP5153	GATACTCGAACGCGGTGGCGGCGGAAGGCGACTGTTCGAGACGGTAATCACCTGAAATCA[C/G]CAGAGGACTGAATCGTAATCTTCTAAAAAAATGTCTGGTGAAGAGGAAGAAAACGCCGCA
3071	CaSNP5154	GACGATAACAGCAATATAACTTTAGTTTTTGACATGATTTTCACTAGAGTGTTAAAGGGA[A/G]TTCCAGTTGTAAATAGAAATAGAAGCTCAGATGAAAAGATTGTTGTGTGTAGATATCTTT
3072	CaSNP5155	ATTATCCCATAAGTTTAAAAATAAGATTAGCAGCCAAAAGGCCGGTGAAACGAGTCAATA [A/C] AATGGTGAGTTGATGTCCTAAACATCGAAGATGTACAAAACAGGGCCAGACAGATTAGGG
3073	CaTSNP6001	ATGTTCGACCGATGTTAAAGCAACAACTTCGTAATGCTAGACTCTAAATAAGTTTTATAA[C/T]TGGACTGCTGACCAGGGTCCAGGAGGAAGTTATGTACACTTCCAATAAAGACGGGTAATG
3074	CaTSNP6002	CTGATCAAGTTGGGATAAATTCGGTGTTTCTATGCAAATAAGTGTTTAGTACTGTGTGTG[G/A]AACGAGTGGTTCTGTGTTGATGTGGTTGCCTCTTTTTTTAATGTTTTAGTTTTGGAGATC
3075	CaTSNP6003	TACAATAGACAGGACGTCTACTCTTACGGATGCCTTGAAGGTGAAGGAGATTGAAGGTGA[G/C]CCGCTACTTGATGCAAATGCATTAAAAGCTTTGATCAGACTTCTACGATTGGCACAGCCC
3076	CaTSNP6004	CACTACATATGAGCGAGTAGATCAGAGTTACATTGTATCAACTCGTCATTATAGAGCACC[A/T]GAAGTTATCCTTGGTCTTGGCTGGAGTCATCCGTGTGATATATGGAGTGTGGGATGTATA
3077	CaTSNP6005	CACTACATATGAGCGAGTAGATCAGAGTTACATTGTATCAACTCGTCATTATAGAGCACC[A/T]GAAGTTATCCTTGGTCTTGGCTGGAGTCATCCGTGTGATATATGGAGTGTGGGATGTATA
3078	CaTSNP6006	CAGAGGAGGATGATGATGATTTCTGATAAGATTGTCGTTTTTCACCCTAATAGTTGCACT[C/G]AGGATCTGTTAATTTCCAACATTTGCTTATATTTCCATTTTGAAGAACTTCCATTAGCAT
3079	CaTSNP6007	GGTTGGGGCTTGGATACGTTATGCCAAGTTTGAAATGAAGAATGGTGAGGTTCCTAAGGC[A/G]AGGAATGTTTATGAAAGAGCGGTGGAGAAGCTTGCAGACGACGAAGAAGCTGAACAGCTT
3080	CaTSNP6008	AACTTATGGTTCACTATTAAAAAATCTTACGTTTATCAAGACATTTGCTTCCGGAATTCT[T/G]GTTCCCAAGGAATACATATGGCCTGTAGATGCTAGTCTTTATCTACAACAACATACCACT
3081	CaTSNP6009	TTTGGCTTCAAATTTGAAGCGGCAGAGATCTGTGCTGGACAAACGGATAATGAAGATCTC[C/T]GAGTTAGGAATATCTGTGTGAATCATTGGACAAATTTTGGTACTTCCCTTAATGCGCACC
3082	CaTSNP6010	TTCTGATCTTATAGAAACAACTTCAGAACATTGCAAGGATAGTTCAGAGGATGCTGGTAG[T/C]GGCTCGTTGTCTCTTCCAGAAGCATCTGGTACTAAAGACAAACCAATTTTAGAACCAAGC
3083	CaTSNP6011	CAGTGGAGGAGGAGCAATAGAGAGAGAGAGAGAGGAGGCAGTTTCATTTCTATTGAATTC[A/T]TTTCTTTGAGCCCTATCTACTTCTCAACAAATCCTCAAGATGCAAATCTTCGTGAAAACC
3084	CaTSNP6012	TTGTTATGATGGTTTGAAGAGGCAGAGGTTAAATGACCCTATGATTCGTGGACCTGATGG[T/C]CGATTTAAGGCTGTGAACTGGCGCGAGGCCCTTGCTCTAGTTGCAGAGGTTGCACATCAA
3085	CaTSNP6013	ATGCACCAGTTCTTTTCCCCGATGTCAAAAGTCAGAGATGAACAGAATGGTGTGTTAGAA[T/C]TGCATGATGTTGAGGACAAACAAAGGTCAAAGGACTCGTTTATAAATATGGCAGCTGGAG
3086	CaTSNP6014	ATACGTTGTCAACTCTTCATCGGTACGATGCTGGTTCGGAATAATTTTTATATTCGCGTG[C/A]CGGCAGAGCAAGGAATAAAGAGCTAGTGTTCCACCTTGACCATTGTCATTTGCTCTTAAT

3087	CaTSNP6015	GTGTTTGAAAGTTTTGTTTTTAAGAGCATGTTTAATTAAACTCCATTTCAGTTTTGTTTG[C/T]GAACTTTTTTTCCTTCATTACAATTTGTTTTAGTTGTTCAATTGATGCTGCCAACGAGAG
3088	CaTSNP6016	TGGTATTGTCCTGGCGGGAATGAGGGCGAGGTAGGTGATAGCGGAAGGGAAGAAATGAAG[G/A]ACAAGGTGCGCTGCAACGAGGATCAAAGCGAGACCCTTGCAGAGTCGCGTGAATCCAGAC
3089	CaTSNP6017	ATTGAACAATGAGTTGGAAGTTGTTGAAGGAATGAAGCTTGACCGAGGCTACATTTCTCC[A/G]TATTTCATAACTAACCAGAAGAACCAGAAATGCGAACTTGAGGATCCCCTCATTATAATC
3090	CaTSNP6018	AAAGGAAGCACTGAGTAAGGTGCTTGTACCATTTTATCCAATGGCTGGTCGTCTTCGTCG[C/T]GACGAAGATGGCCGTGTTGAAATTGACTGTAATGGTCAAGGAGTGCTCTTTATCGAGGCC
3091	CaTSNP6019	TGAAGGAAGGAGAGGGATATGAACGACCTAATGATGGAGCTGTTGTTCAGGTGAAACTTA[C/T]TGGGAAGCTGCAAGACGGAACAGTCTTCCTTAAGAAGGGTCATGATGATGATCAGCCATT
3092	CaTSNP6020	ATGTTCACCATTCTGCCACAGAATATCAACTTTAGTCTTTATCTTCCCAATTATATACAA[T/C]TCCTCAATATAAGAATTAAGGTTTCTTCCCCGACTCTTACACTCTCTTATCATACTATGC
3093	CaTSNP6021	GAACACACACATTTTAATTTCAATCCCTAACCCTAATTCCTAATTTTGATTGCAAGCACA[T/A]CGAGATCCGTGAGAGAGAGCTTAGTTGAGCCCTGAGATTGCGTTTCGTAGAGATAGAGAG
3094	CaTSNP6022	GAAGTATCCACATAGACAACATCAATATTTTAGAGATTGGTCTTCATGACCTCCGAAGCC[G/A]TCTAAGTATCATACCGCAGGATCCAACCTTATTTGAAGGGACCATTCGAGGAAATCTTGA
3095	CaTSNP6023	GAGCTCAAGAACCTTCAGAGATAGCAGCAGCAAGAGTGCAAAACAAACCAAAAGTTGAAA[C/T]GGTGATGACGCTTCCGTTGCTGCCACTGCTGCAATTGGCACAGGCAGAGACACAAAACGA
3096	CaTSNP6024	AAAGTTACATATCATGATATAGAAGCCATTGATCCTGCTTATTTCAAAAATTTGAAATGG[T/C]TGCTTGAGAATGACATCAGTGATGATCTGAATCTTACCTTTAGCATTGATGCAGATGAGG
3097	CaTSNP6025	ATTGCATTATTGGAGGATATAAAATACCACGAGACACCATAGTACTAATTAATGCTTGGG[C/G]CATTCAAAGGGATCCTGAGTCGTGGAGTGAGGCCACAACCTTCAAGCCCGAGAGGTTTAA
3098	CaTSNP6026	TGCAAGTTGATATTATTTCAGAACACCAGCACATTGTACTTTCAACTACAGGTCCAATTG[G/A]TCCAGCTGCTGGTTTGGGAACCTATGTAGAGGCACTTGTGCGAAAACATGACACTGAATT
3099	CaTSNP6027	TTCAAAACTATTGAGGAAGAACTGTGCGGTTCGTTTAACAGCAGTCATATCTCCAGTTGG[G/A]ACAAGAACGCCCATCTGAATCATTGCTTGAAGGACCTTATCAGGACTCTTCTCATAAATT
3100	CaTSNP6028	ACTCCATTTCACATCGCTCACTCTTCTTTCGGTCAACTCTTGTTCAAGCAATGCACCTTC[T/A]TCTTTGACAGCGCGAATTCGAGTGCGGAGAGTAACATTGTATTTGTGACTCGAAACGGAA
3101	CaTSNP6029	TGTCCATGTCCGTGTCGTGATTGCACTGATAGTCTCACAGCTAGTTCTGATGGGACTCCT[G/A]ACCACCAAAAAGGCTGCTTCATCAACTCCATTTCTCATTGTGCTCCCAATCCTGACGATA
3102	CaTSNP6030	ATTTGTCACACTCTTCACCTGGTGATATAACTTTCTCCCCTGTCAACAACTCTGTTCCCA[A/G]CTCTTCTCTAACAAACTTGTACAATGGATAAGACCTACACTCCTTAATCTTGTTTGGAAT
3103	CaTSNP6031	ATTTGTCACACTCTTCACCTGGTGATATAACTTTCTCCCCTGTCAACAACTCTGTTCCCA[G/A]CTCTTCTCTAACAAACTTGTACAATGGATAAGACCTACACTCCTTAATCTTGTTTGGAAT
3104	CaTSNP6032	AGATGATTATAAGCAATGGACCTCATAAAACTAAACAGCTATCTTAAGTGAATATAAAGC[T/C]TTCCCACCATCTAGCATGCTAAATATGAAAACATATACAGTTACCTCATAAAATAATAAC
3105	CaTSNP6033	TACTTTTGGAGACAAATCAAGATTGGGCAGAGGGGGTATGACTGCTAAAGTCAATGCTGC[T/C]GTTTGTGCTGCTCATGCTGGCATCCCTGTTATTATAACTAGTGGCTTTGCCACAGACAAC
3106	CaTSNP6034	GGACCTGGTCTTGGGATCATAATGTGCAGAATTGACATCAAGATTGAGAAGATATTTTGC[T/G]GTATCCTCTCGAATACGTAAGTTCCTCACAGTTCCTGTACTCCCACCGCCTGTTGTTCGC
3107	CaTSNP6035	GGGGTAAAATTCCCTTTTTTGTTCCTCCTCCACGGCTGGATGACTTGTCTGAGTCTCAGG[A/G]GCCTAATGTTAACGGCATAGAGGTTGATGAGGCAGTTGACCATAATCAGGCATCTGCTGC
3108	CaTSNP6036	GGCTTAGAGGAGTTGAAGAAGCTGTTACTACAGTTCAGGATGAACGAAATGTTTTAAGAC[A/G]TTCTGATCTGTCTGCTTTCTCAAGGTACAATGCGGCCTTTAATGGTAAGAAGTCTCCCAC
3109	CaTSNP6037	AATAGCTGGCCCTCAACTTGGAGAAGCAGCTCACCGTCTTGTCAGAAACTCCCTTCAGGT[C/T]AACAATTCATCCTATGGATTATCGGACCAACTTCCAGGCCATCATACAATGCACAGGGTC
3110	CaTSNP6038	TGGAGATGCAGGAATGAAAGATGTAATCAAGTTTCTGACTACAAAAGAAGATACATCTCG[T/C]GAAAACAAGTTGCGCTTGTTAATGATTCTGGCAGCCATATACCCTGAGAAATTTGAGGGT
3111	CaTSNP6040	AGAAGTAATCAGCCCTAATGTTTCATGCCCAAACACATGCATCAAATCCTAAGGAAGTTC[T/A]CGCCAACCATCAGACTCACTCTGCATCATCACCTCCACCAAGTTTGGCAGTGCCATTCAC
3112	CaTSNP6041	TCCTCGTCCTCGGAATCCTCTAATTGATGCCGTTGCTGCTCATGACAAAAGCAAGCTTAG[G/A]AGAGTTACTGAACGGATCATGCCTCAAATAGCACCAAAGTTAGATGAAAGAGATTCATGG
3113	CaTSNP6042	ACCATTCATAACTGCCACAGCAGATGGCCCCAAACATATTGAGACCACTCTTACAAGAGC[T/C]AAGTTCGAGGAATTATGTTCAGATCTTCTTGACAGACTCAAAACACCAGTGGAAAATTCA
3114	CaTSNP6043	TGCCATGGAAGGTGGAAAACCCACCATCATTACCAACGCCGAGGGTCAGAGAACCACTCC[C/A]TCCGTTGTTGCATACACCAAGAACGGCGACAGGCTGGTTGGTCAAATTGCCAAGCGCCAG
3115	CaTSNP6044	GTCTTCCTCAACCTCATCTTCCACTTTCTCTATATCCTCTTCATCCACACTTATTGATGG[C/A]AAGCATCCCTGCAGACAATCACCAGCTTTATCTCCACAATGTGTTAGCCTTCCAACTCTT
3116	CaTSNP6045	AAGAGATCTCTCTCAATGTATGTCTTGTGGATCACACATATAAAGAAGTCCTTCTCACAA[T/C]TGAATTCATTCTTCACTTTCTCATGCAAAATTCTACTTCTCTTAAGAATATTATCGCAGC

3117	CaTSNP6046	TTATTCAGTTACCTTGTCATTACCTCGACAACCACCATCTTTCTTGCGTGGAAGAACACC[C/A]GACAGAATAACACAAGGGCTTAATTCTGATCAGTCTTCCACCTGTTCAATTCCATGGTCT
3118	CaTSNP6047	CATCTGGAGATCACGACTACCTGATGTACACTGAACATTATGGGGATGCGCACGGACATC[G/A]TAGATGACAGGCCTCTCAAACCATGGAATTACAAAGTGTGTCCCTTCGGGGTAAACCTTG
3119	CaTSNP6048	TTGTTATGATGGTTTGAAGAGGCAGAGGTTAAATGACCCTATGATTCGTGGACCTGATGG[T/C]CGATTTAAGGCTGTGAACTGGCGCGAGGCCCTTGCTCTAGTTGCAGAGGTTGCACATCAA
3120	CaTSNP6049	GAGAATGCGTGTGATGTTCTGTGCATTGGAGGTTGAGAATATGGCGGAGATGAAGAGTAG[A/C]GCCGCCGTGAAGGCGGCTAGAGTTGGTGGTCGGAGCAGGTGCTGCATTGTGGAAGAGTGG
3121	CaTSNP6050	TCACTGGGCTGAATTTGGATGCAATAAATAAGGCAGTGGTGTTGCTTGATGAATCACAGA[G/A]GGATGAGTTTGAGGGAAGATGGAAGAAGTTGCATATTGCTGAATTGGAGCTGTTTACAAG
3122	CaTSNP6051	TGATGCTGTTACTTACACTGAGCACGCGAGGAGGAAGACTGTCACTGCTATGGATGTTGT[C/T]TACGCTCTCAAGAGACAAGGAAGAACCCTCTATGGATTCGGAGGTTAAAACAGAAACACT
3123	CaTSNP6052	GAAGAGGGTTTTCTGCACATGTGTGGAAGGTTTGATTGCAGAAAATGTTGTGGATGCAGA[G/A]GCTGCAGTTGTGGAAGGAGTGATTGCATTGATGGGACAAAGTACTGAACAATTGGTGGAA
3124	CaTSNP6053	TACAGGGTTTGGCCACCGCCACTGTGTATAGACCCTGAAGAATCTAGAAACCAACATACT[A/G]GGGATGGCATTAGGGTAAAGCTGGCAAACTCGAGCAACTAAAATAGCCCAATTTACACCT
3125	CaTSNP6054	AGGGAGAGCGAAATCTTCAAAGTGTATTCCATCAATAGCATTAATCATGATTGCAGGCTC[C/T]AGTTAGACTATATTTAATCCTTTAAATATTTAAAACCATGGTACGTATATTAACTAAGGT
3126	CaTSNP6055	TGCTGTTGCACAGGCACGTGGTTCTCTCCCTGCTGCCAGTGGCGGCGGTGGTAATGCTAC[C/T]ACAACTACTCCTGCACCGGCTGGAGGTCAACCTGGTCAAGCTTCTGCAGGGACTGGTTAT
3127	CaTSNP6056	GCAAAACATTTTCCATGGCCTCCAACTCTTCAGGTTGTAAAGTGACATCCACTTTATCTG[C/A]CTTCTGGCCTTTAAGCAAATCGACCCTTTTAGCACCTGCCTTGTCCTGGGTGCCGGAGCC
3128	CaTSNP6057	GGCGAGAAACCATTCTAGCTCAATGCAGGAAAGACTAATTTATGAAGGATGGATTCTATA[T/C]GATACTGGCTATCGCGACGAGGCTGTTACAAGGGCTGACAGATCCATTGCAATTCAGAAA
3129	CaTSNP6058	TCTTTCCTCGTCAAGGTCATAATCATTCTTGTGTCTGCTAGTATAATAAAGGGATCCTTG[A/T]TCCCTAGAGTGCCTTCTCTTAGGATGAGCATACTCTTCTACTACACTACGAGAACGAGAC
3130	CaTSNP6059	CGTCACACATTAACGCTTCTAATTGTTACCGCCACAAAAGAAAACTATGTTTATCTTTTC[A/G]ACACCGTCTTCTTCTCCTTCAACCGCATTTTTCTCACCACCTAGGGCTTCAAATTCTAAA
3131	CaTSNP6060	GACATTCCAAAAAAGACTACAATCTTGATAAATGCATGGGCAATGGGAAGAGATCCTGAA[T/C]ATTGGAGTGATGCTGAGAGGTTTATTCCAGAGAGATTTGATGGTAGTTTTATTGATTTCA
3132	CaTSNP6061	ATGGAATCTCGGGTGTGAGTAAGGAACGGTTTGAAAGCGAGTTCGATAGCTAACCACACA[C/A]ACGCTAGGGCTCCCACCACCACCACCGCTTCTTTTGCCTTTCCCATTCTTTTTCTCTCAC
3133	CaTSNP6062	ATCTGCTATTGTTGAAACTCAACCTGACACTATTTTGGAAGTTTGGCATTCTCTAGAACG[C/T]CCTGTGAGTCTTTCTGTGTTTGAAGAACTTGAAGAGAGGTACGGTGATTGGACTACTTGT
3134	CaTSNP6063	ACACCCATGGCACAAGATCTTCCACAAAAAGATGCCTCCAGAAGCTATTGATCTTGCTTC[T/C]CGGCTTTTGCAGTACTCCCCAAGTCTGCGTTGCACTGCGCTTGAAGCATGTGCACATCCC
3135	CaTSNP6064	AGCCAGTTCAGTCTTTCCCACACCGGTCGGCCCCATAAACATGAAGCTAGCGATTGGACG[A/G]TGAGGATCTGAAAGACCTGCTCTTGAACGCTGGATAGCCTCGGCTACTGCAGTAACAGCA
3136	CaTSNP6065	GAATGAAAGATGGTTTCAGTTGCAGCTTAGGAAATGAAACTGTTTAACGTGAGAAGCACC[A/T]AGGGAGAGGAATTTGGTGTGTTTAATGAAGATGGTGTCAAAGGGACACACGACATTTTGG
3137	CaTSNP6066	TGAGAGATCTCCAAGAGAGCATTGATTCACAGAAGGAAGTTGAAAAAGCGCTAGAGCAAA[C/G]TCCGTCGTTCACAGATATCGAGGATGAAGATATTGAAAAGGAACTCGAGGAGTTAGAGCT
3138	CaTSNP6067	ATGGGAGAGGAAGGGTCCACAAGTGGATTCCATCTTCCACTAGCATAGTTCATAAGCTGT[C/T]GTGAATATTCCTTAACTTGCTCCTTCTGGTAAAGTACCTTAGCAGCAGGACCTATTGCTT
3139	CaTSNP6068	GAAGTATATCGTGGCATACTTTGTGTCTTGAGGACCATATACATATAGTTTTTACAGCTT[G/C]AGGCTGGGAATTTTAGTTTTGCGCCTATGTATATGTATAAAGAAAAAGTTGCAAAAGGAG
3140	CaTSNP6069	AAGTATTTCATCCATGTATTGTGGCAAATGTGTAGGCCATTAATATTTGTCTCTCTGAAA[C/A]GCATACACATGCAGGCCACCATCCATTTGTAGAGTGAGCGTGGTTCTATATCTTACCAAT
3141	CaTSNP6070	ATCCATAACTTGTCATCCACACTAGTACCTCCTACTAGGGTAGCGATCCACAAACCGACC[A/G]GACGGACACTCATATGCCAAGTGACCACGACCTCCACAATTGTGACAAATCATCAATGGC
3142	CaTSNP6071	AAGGTTATCTAGGATCATGTTCACCCGATACTCGTCATTGATCTTCTCTTTGAACTCCTT[G/A]GCAGTTTTAGCATCAAGTTTAATTTTACACAAAATATTACACATTTGTGGTTCACGCATT
3143	CaTSNP6072	CTTATCCCCAGTGTATATATCTCTCTCAGTTGCAGATGCAAAAACAGTTTTCACCAGATC[A/G]ACTGCTTCTGTTTCAGACAGTGGAGTAACAGCATCCTGGGCTGGTAAGAGGAGTGGGCTA
3144	CaTSNP6073	TGGACTCTGAACCCCAATACCAAAAAGAAAACAAAAAACAAAAAGAAAAAGCAAACTGAA[C/T]CTGAGCCTGAACCGGTTCAGGCAAAAGTCAAAGTTGTCCGTGAGAATCCAAACAAAATAC
3145	CaTSNP6074	ACAAGGTTTGCGCACCCTTGTAGTTGCCTCTAGAAGTCTTTCTGATGCTGAACTTAGGGA[A/G]TGGCAAAGTAGGTATGGAGAAGCAAGCACCGCGTTAACCGACAGAGCTACTAAACTACGT
3146	CaTSNP6075	GAACACTCAAATCTGAAATAACCTAGTACTCTACTAATACCCCACTAAAAACCAAGACTG[A/G]TGATTCTTGTTTTCCTTACACTAAATTATTTTTCTTTATTTGTTTTTCTCCTTATTTCGC

3147	CaTSNP6076	AGGATCAGCATGCCTATGTCCATGTCCAGGTTGGTTGTACCACTCACGTTGATCCTCAGG[T/C]TCATAGTCACGCCCTCTTTCACCGTGAGGCTCCTTCTCATGTTTAGATTCAACATGATCA
3148	CaTSNP6077	ATTATCTGGCTCATCGTTACAACACTTGAGAGCTAAACTCAAAAATTTCTCGACGTGCTC[G/A]GATGGATAAGACCCCATACGTGCATCAATAATTGAAAATATCACGCCGGATTGGTATGAA
3149	CaTSNP6078	ACAAGTCCTCTTGGTGGTGGTCGTCCTGGTCGCGTGAAGCGAAGGAACCCTCAAGGCTGC[T/C]GCTAAGAAGGCTGCTGGTGGTGATGGAGATGAGGAGGATGAAGATTAGATTATGAATTTC
3150	CaTSNP6079	AAGGCCATTTTCTCTAAAGATTCGAGTTACATCAGACAAAAGGCCAACTCTGTCGTCACC[A/C]GAGAGTTCTAGCTTAATTCCCTCGGATGTTCGTCTCCGAACTGCAGCCTCCAAGCAGTGA
3151	CaTSNP6080	GAATATTGACCGTTGGATAGGGCCAACAATTAAAATGTCACTTCCAAGTTTTAGTGGTGG[T/C]ACAGAGTTTAATCCCAACCTTCTCAAGTACACTTGTCAGATTGAATCCAGGGTGCAAGCA
3152	CaTSNP6081	CGGTGAATTCTCCGACGACGAGATTGAAAGCCCCAAGAAAATGGGATATGTCAAGATTCC[G/A]GCGGCTGAGGTGGCTGCTGCTGCTCCCGCACCTCCGGCCAAGGATTCGGTATGAGAAATT
3153	CaTSNP6082	GCATAGAGTGGAATCAGAGACAGAGAGAAGAAATCCTTCACCATTTCAGCGTCTCCGTTC[T/C]ACAATGGCCACTATACCTGTTAATCCCAAACCCTTTTTGAACAATTTGACTGGGAAGCCT
3154	CaTSNP6083	CGTCACAGACCCAATCCTCTGAACAATCCTCGCAAGGCTTACTACTGAATAAACAACACA[C/G]GAAACCTTATGCCAAATTTATTATGCGGAAACTAGCCCTTTTAATGTTTAGATGGGCTGC
3155	CaTSNP6084	TTTCAGCCTATGGAAATCTATGTTGATGATGAAGCCAAGTTAACCCTTCATGGGACTTGT[G/C]CAGCACTACATCAAGTTGAAAGAGGAGGAGAAAAACCGCAAGTTAAATGATCTTCTCGAT
3156	CaTSNP6085	TAGCAGGGGATGAAGCCTGTTGGTGGTAAGGGCACTTGTATCAACATCCTCTTCCTCTCC[T/C]TGTCCGAGCTCCCACTCAAATTCCTGAACAAATCTGCCAATAGCCGTGCAAGTTATCAAC
3157	CaTSNP6086	AAAATATGAATTATCAGCACAGAACCAGAAAGATCAAGCTTCATTGGGTATAATTCAGAA[T/G]GAAGCAGATCCTGAAGAACAAGATTATGAACTAAATCCAGCTGTTAAATTAGAAGGAAAC
3158	CaTSNP6087	TTTCTGGTTGTAGGATGCATGTCATGTTTCCCCTGTATACGAATGACTATTATGGGCTTG[T/C]TTGGGGTCGATGGTTCGGGCAGTTTCCTCTTAACGCCCTTGAGCCTCCGTTTCATTCTAA
3159	CaTSNP6088	TGCGGATCCAAAACCGGTGGGTCCATGGGATACATAAGGAGCACCGTAGGGAGCAGGACC[G/A]CCGCGGCCAAAATTAGGCGGCGGCATAGGTCCAACTAGATCACTGCGACGGCCGCGAAAA
3160	CaTSNP6089	GGGATGTACTCACGTAATGGGGGTAAAAGTTGGATTAAATCCATGATCCTTACAGCATCA[C/T]TATTCCCGTTCATGTGCTTTGGAATTGGATTTATCTTAAACACTGTTGCTATATTCTATG
3161	CaTSNP6090	CCAAACATGGCTAAAGGTACTACTTTTACTACCCTTCTTTTCTTTCTCCTAATAATAACT[T/C]TCCTTTCAATTGCAATTTCAACACCAACAAATTTCATCAAATCATCATGTAGCACAACAC
3162	CaTSNP6091	GTGTGCCGACAACAACTGTTCATACAACGGATGCGCCATTATCTCCGCCTTCCACCTCCC[C/T]GTCTGCCAGTTCACCATCCCTCCTCCTCCGTCTCCGACCACCGTCTCCTCCTTCCCTCCG
3163	CaTSNP6092	GAGCTCATTAGATGTTTGAGGTCTATTCGGGAACATGCCTTCGTCGCATCGGAATATCCA[G/A]TTATAATCACCTTAGAAGACCATCTTACTCCAGATCTTCAGGCTAAAGTCGCTGAGATGG
3164	CaTSNP6093	GCAAAAGGAAAGGGAGAAACAACTATTGGAAATGCTTGACATTGCTTGTAAATGTCTTGA[T/C]CAAGATCCAAGGCAAAGACCTTCTATTGAAATGGTTGTTTCATGGCTTGATAGTGTTAAA
3165	CaTSNP6094	AGGATTTATCCTTGATCATCATGGGGCTGAGATTAATGGGCAGCTGAAGTCATTGCAAAT[T/G]GTTGCAAGTTTAACAGCCGAACGGGATGGGGATATTAGGAAAGCTGCATTAAATGCATTA
3166	CaTSNP6095	TCCTAAGCCAAAGTAATGAGACAAAATGGCAGCAGGGATAGCAGGAAAGAGAATGGAAAG[T/C]TTGGTACCAAGAACAACCTCTTGGAGATTAACCAAAAAATTCCTAAGCAAAACACATCGA
3167	CaTSNP6096	AAAAAGATTCCAACTAGATTTTGAAATTACCTCCATGACTGATTTTGGTGAAAAATTTGT[G/A]TATGTATTAGAGTTGTTGATAATGTAAAGAAAAGATGTGTATATTTATGTATTAGCTGAG
3168	CaTSNP6097	AAATGATTTTGTTCCATAAACAGATCAAATGGGGAAGCAAATTGCCAAACAACCATTATT[C/T]GTCTACTTCTAGAGTTCCATAGATATTCTTGTCTTTATTGGTTTCAATTTCAAACCCATA
3169	CaTSNP6098	GAATTTTCCGTGAGGCATGTGACCACCACCATGTGCATATCCACCTTGTGCATAGCCACC[T/C]GGTGCGTATCCACCTTGTGGATAGTGACCTTGAGAGCCGTGGGAGATATGGTGAGCACCA
3170	CaTSNP6099	TAATTTCTTCAGTCGAGCTATCTCTTTCGAAGAAATATTTTTGCTCGGATCGTTCACAAT[C/T]GACCTCACCCGTGATGCATACATGAGAGAGTTATGTGTCTCATCCAAGCTTGATTCTATT
3171	CaTSNP6100	CGTCGTTGATATCTTAATTCAGTGTTTCCCCATGGAAGTGCCGCCACTTATTAGCAGTAC[G/A]TTACAAAAATTAATTGTCATATGTTTGAGTGGAGGAGATGACCGAGACCCCTCTAAAACA
3172	CaTSNP6101	GTCTATGTCTGAAGTATTGAATAATGAAATAGGGGGATTTAACAATTTCTTGCCTAGTTA[T/C]GAGAATATCAAATTTTAAGTATCTGAATTGACTGATCAAGTAATGTTCATGGTAGTGATT
3173	CaTSNP6102	ATCTTGCACATGTTCAACTCTTCTATCCCTATCCTTTTCATGGTCTCGGGACCAATCACG[G/A]TCTCGAGACCGATCACGATGTCGAGTCCAATCACTGTTTCTATTCCTAAATCCATCTTTC
3174	CaTSNP6103	GTGTGACTGATATCTGAACATTCACTCTGAGGTTGTTGCAGAAGATGCATGGTAGTTGTT[G/T]GTTTGGAATTGGATGCTTCCATTGATGTAGAAGAAGGTTTGCATGTATCTTTTACGAGTA
3175	CaTSNP6104	TATATTTCTAGAAAGTGCATGAAATGCACCTATTGAAACCTCGTCGGTAAAAATATCTTC[G/T]GGTCGAACAAGATATTGGCCTGCAGCTGAAAGTGATATAATATCAACACCATCACTAATG
3176	CaTSNP6105	AAATAAATGTTCACCTAAGAAGTACAAAATCCACAACTATATACATAGAATGAGAAATCG[C/A]TAAGAGAGCAAGATTTTTCCAGGCTCTTACATAACACAAGGTTTAACAGAAAGTTGCATC

3177	CaTSNP6106	CCAAATAAACTAAAAATAACAAGCTTTCAATCTATCATTATTATCCATTCAAATTAGCCA[C/T]AAGATCATAACCATATCTCACAGTAAAACATAATTAATTAAAACACCTCAATAATAAATC
3178	CaTSNP6107	TGCTCTCATCGGAGTTGGTGGTTCTATTCTTGGAGTGAAGTTTTTAGATCCCCTTGCTGG[A/G]CTTCTTGTCTCGGTCATGATTTTAAAAGCTGGAGCTGAAACTGGTTACCAGAGTATCTTG
3179	CaTSNP6108	CGCAGTACTAATTGCAACGGTGGCATTCGCCGCTATATTCACTGTCCCCGGTCAATTTGT[G/A]GACGATCCAAAACACATTCCTGCAGGAAAGTCGCTGGGGGAAGCCAACATTGCTCCGAAG
3180	CaTSNP6109	CTCTCCAGACGGTGATTTCATAGCGTTTAATCACGACTTCGAAGCTAACAACAACGGTGG[C/A]GTTAAGATCATAAAATCCAACGGTTCTAAACGTTGGAGTTTATTAAAAGGAAGAACATGT
3181	CaTSNP6110	TCCTCCACCGCCACTTGAACGGTTTTGAGCTTCGTTAACAGTAATGTTACGTCCGTCCAT[G/A]TCCTGTCCGTTCATTCCTTCAATAGCATCCCTCATTGACTTCTCATTAGCAAATGTCACG
3182	CaTSNP6111	ACTTATGGCACAGGTGCTTTCATTCTGCTAAACACTGGTGAAGAAATTGTTCAATCTAAG[C/T]ATGGCCTTCTATCAACTGTAGCCTTTAAGCTTGGCCCGAAAGCTCCAACCAATTATGCAC
3183	CaTSNP6112	TAGGGTTCAACTTCCTGGACTTAGCCAAGAGCTTCTTCAGAAATTCAGGGAAAAGAACAT[A/T]GATTTTGCAGCTCATAATGCTCAAGAAGATTCGGGTTCTCTTTTGTTTAACCTGATTGGG
3184	CaTSNP6113	CTGCTGGCCACTAAATGGGAAGCCTTGCATTGGATTTGGTTCCTGCATAATACCAGTCAT[C/T]GGATTACTGGGATGAGGGGGCATAGCGGCACCCCTTGGGAAACCTCTTAATAAATGAGAC
3185	CaTSNP6115	AAACGATGACGACGAGAAGGAGAATACTGAACCACAACAGGTTGTTGAACAAGTTGAAAT[G/T]GAGTATGTTCCGGAAAAGGCAGAGCTATATGAAGGCTTGGATGAAGAATTTAAAAAAATA
3186	CaTSNP6116	TTTTGGGAAGTCAAGTAAAGTTACTATAATTACATTCCAAGATTCAGAATCCTCTGGTAG[A/G]GAATATAGCTAGAGTTTATTGTCTTGTTAACTCGGTGCGAGGAAGATGGCTGGTTTTAAG
3187	CaTSNP6117	AGCAAAATCTCTTCTTTATGAATTCTATCCAACCAGATTAAATTAAATAGAAATGATAAT[C/T]AAGAGCAAACAACCATTATAAAAAATACCAAATCATTGTCTCAACTCTCAAGTAACATAG
3188	CaTSNP6118	TGTTTCATTCTTTTTCTCTTGTGCCCTTTTCTTCCTCATGTGTGTCCTCCAATAATTCTT[T/A]ATCTCATTGTCTGTTCGTCCCGGTAGCTTTCTAGCAATTCTTGACCATCTATTTCCCCAT
3189	CaTSNP6119	ATCAACCAAACACTTAGTTATGCTAGGGAGTTGGAGAGGATTGTCTGAAAAGAAATGCAC[A/G]GGATGGTTTATGTGTTTTCCCAAATTGCTGATATTGTCGTTTGAAATTGTTTACTTCTGA
3190	CaTSNP6120	CAGAAACGACTTTGATTCATGTTCCAATCTCCTTTCTCAGTTAAAGGTGTTACTAACAGG[G/A]TTTAGAAGCCTTCCACCCTTGTTTGCAGATACTCCTAATGCAGTTCAGGAGTTAACAATA
3191	CaTSNP6121	CTCTTTTGGGCTACGGAACGGCCGTTCAGAACTGTTTGGCTGCAGCTTCCTTAGTGGAAC[T/G]TCATGGCTTGCGTTTAACCGTTGCCGATGCACGTTTCTGCAAGCCATTGGATCGTTCCTT
3192	CaTSNP6122	AAATAGTTTTACACATAAGTACTCCAACCATTTTACTCATAAATCATAAACACTAACAAT[G/T]AAACAAGCTTTTTGGAAAACACAAGACCCCTTATTCTAGCTAAAAAAACAACAATATCAT
3193	CaTSNP6123	AGGGGCAGCATAACCTTGAGTGCCCATAACTCTAGTTGAAACATGTGTGTCATCTCCTTC[A/C]GGACCATCTTTTGCCAAGCCAAAATCAGAGAGCTTTGCATTATAATCAGAGTCTAATAAG
3194	CaTSNP6124	AGCCTCTGCTGCTTTGACGGTCAAGAGGGACTTTGGTTCCCTGCAGATGTGCCGTAAGCC[G/A]GATTGCTGTACCTATTGTAAGCACAAAACAAATGCCATCCCTAAAAATGAAGAGCAAAGT
3195	CaTSNP6125	TGTCTCACCGCAAGGTCGCCCTAAAGCTTCCCGAAGCCCATCTCCCCGAAATTCAAATGG[C/T]GATGAATAAGTGATTTTTCAGAAGTATTCCAGAGACACCTTGACTAGTGCACCTGTTGCT
3196	CaTSNP6126	AAGATGTGTATTTACCAGGAAAAAGACAATTCGTCCGGGAAGGTAGCCACTGATATTATT[T/C]ACTTGTATCTGTCCACCATGTCCACTGACCATATCAATCATTTTTATGTAAGATCCTTCT
3197	CaTSNP6127	TTACCGGCAACGCTACCGGAATCAGATCCTCCTCTTCTCCGTTTCTCGCTTCGTCGTCGA[C/T]TTCTTCATCTTCTTCCGAGTCAAACGGTAAGTTTTCAGTTGAATCGTTGTTCCGGTACGA
3198	CaTSNP6128	TAATGCAATCCTGCTTGAATCTTCAAAGGAAGGATACGCAGCCTTGCATTCGTAGGAAAC[T/A]CCATCAAGTTTACTAAATTTACTGAAAACATGGGTGGGATGTAAAAATGCTCCACCACAA
3199	CaTSNP6130	TATAGTGGGTAATTAATAACCCTTCCATATAGATTGTTTGCAATCGGTACGATTTTGACC[G/A]TCCGATTAAAATTAAACGGTTCAGATTTTAAAGTCATTTTTATCATTTTTATTAAATATA
3200	CaTSNP6131	GCGCTTACGTCTTCTGCCCAAATCCACTGACTCTACAGTGGCAGCACCTGAGTCTGAGTC[T/C]GAACTGTCTTCGCTGCAGACCGACTCTCAACCCACTCAAATTGTCCAATCTCCTACGTGG
3201	CaTSNP6132	GAGAACAGAAACATATGCATCTGAAAGGCCAGTGTATTTTCCCACCATAGCAATTCTAAC[A/G]GTCTCATGACATTTGTCATAAATCTTTGTCCTAACGGTCCACTCCTTCAAATTAGGCTCT
3202	CaTSNP6133	TTTTTTGTAGAAAGAAGTTGAACTTTTCGCGTCCAAAGGTATCAAGTTGCCTTTCACATA[C/T]AATCTTCTCGAGCTTCCTGTTCTGGATGAAGGGCTCCACATGGAAGCCATGTCAGCTCGC
3203	CaTSNP6134	GCCGCTTGGGCTTCCTTTTCCGTTTACTCTTCCTGCCTCGTTTCTTTCCCTTAGTAGCG[T/C]CAACTTTAACAGATGAATCGCCTCCATTTGCCACGTCATCACCTTCACCAGTTTCTTTAT
3204	CaTSNP6135	AAGGAATACAGCTGGGATATTGGAAATCATCACAGGCGCCAAGAAAGCAGTGGAACAAGA[A/T]CTATGAGTGCTGGTTTGAGCGTTATAAGTCGGTTGGCTGAAGAATTTGAGCAAAGGAGTC
3205	CaTSNP6136	CATTTGTTTCTGCCATACTTCTTCCATTGATTTCATTTTAGTTCATATTCAGACCATCT[G/A]TTATCGTACTGTTGAAGACGTTGATGAAGAATGTCATTTTCCTCATCTTTCTCTCTCAGG
3206	CaTSNP6137	CACGGCCAGATTTTAGGTTTATATTCGATGCTATGCATGCCGTTGCTGGTGCTTATGCAA[C/A]GCCCATCTTTGTTGATAAACTTGGTGCTGGTGTGGATTCAATTTCAAATGGAATACCTTT

3207	CaTSNP6138	TCTTTTTACTTTATTAATTGCTGAAGTAGTATTCTTATTACTACTATAAACATAGTGCTG[T/A]TTAGTTCGCTTCTGTCCTGATCGCAGAGATGTAAAAACAAACAATCCAAGTAACTAAACA
3208	CaTSNP6139	TTTGACTATGATTTTAATGATGATGATGATGATATTGAATATGATAATGGGAGAAAGAGT[A/G]ATGAATATGATCATGAATTTTATAATCATGGAGTTGAAGGGTGTGAAGAAGTAGATGTAC
3209	CaTSNP6140	AGAGAATGCTGCCTCAGATGCAGCATCCATTATACTTCTGGGCAACAAAATTTCACAGGT[A/T]ATTGATGCCATAGACCTAGCACAGACAACAATGGCAAAAGTTTACCAAAATTTGTCTTGG
3210	CaTSNP6141	AGATGTCATGTTAGACAGCTCTTTCCAACCTAAGTTACTAGAGGTGACGTACTGTCCTGA[C/T]TGTACGCGAGCTTGTAAGTATGACATGGATATCGTTGTTGGAGAAGGAGGTGTTGCGAAA
3211	CaTSNP6142	AATCTCAATTTCACACTACATACAACTATTTCATCATCATATTTATACAACACACTCAAT[T/C]ATTCCCCTTCCATTTCCTTCGCAATTTTTTCTCTCTACACTTCCAACAAGCTAGTATACT
3212	CaTSNP6143	CCTCCCTCTCCCCTCATCAGCACCACCTCCAACAACCTCCTCCCGCCGTCTCCCTCCACC[T/C]TGCTGGACCCCAGAAGAAACTGCTGCCCTAATCGACGCTTACCGTGACAAATGGTATTCT
3213	CaTSNP6144	TGCATCTGTCTCATTACACATAAATGCTTCTGGAGACATGTAACTCAAAGTACCCACCTG[C/T]GAATCCCGTTGAATATTAGTTGTATCACTCATTATTGCTTTGGCGATACCAAAATCAATC
3214	CaTSNP6145	AAAACCTATGGGACCAACTATGGTCCCATCTCACTCGGTTAACCAAACACCATCACGTCC[T/A]GCACAACAAATGAGGGAACTTGTACACAAATATGGTCAAAACAAAACAAATGTATCTTCA
3215	CaTSNP6146	GGATCCTCCTACATCATGCAGCGCAGGTCCAGTTGCTGAGGACATGTTTCACTGGCAAGC[G/A]ACAATCATGGGACCTGCTGATAGCCCTTATGCAGGTGGTGTGTTCTTAGTTTCGATTCAT
3216	CaTSNP6147	AAGGGATACACAAGCATCACTTGTTACACTTGGTCGAAGTGCTCGGTAGATCTGATGTGA[A/T]CGACAGGCAAGGTGTCTTGATGTGCACTCCATAGCCCACCTGAGTGCTTCTTCTCCCCAA
3217	CaTSNP6148	GTTAATGGCGAAGATCCTACTCTGTTAGGAGCATCTGTATTTGTTGATGATTATTGGTGT[G/T]ATAATGTCTTCCAATCTTATTTAATGCAATTTATATTTTTAGTACCGATCAGTTTCTGTT
3218	CaTSNP6149	ACCTCCGCCTGATGTGTATATGGATCAGAGAACAAGATTGAGGCACCAGAGTCTCCTACA[G/A]GACTATGAAGACTTGCACAAGGAAACAGAAGCCATGAGAATGAAATTGCAGGCTGTGAAG
3219	CaTSNP6150	ATTGAACTCTGAATCAAGAAGAAGAAATCCATTTCCTTCAGCATTTCCTTCTTTATCACC[A/G]AGACACGAGATCATAATTTCACCAGAAGCAAGGCAATGAGATGTGTGAGGATAAGCTAAC
3220	CaTSNP6151	CTAAACTCAATCAAATTTGAAAACCTCTGTTATGGCGACAAAAGAAGGCAAAGCAATTGG[C/T]ATAGACCTCGGCACAACCTACAGTTGCGTCGGCGTATGGCAAAACGACCGCGTCGAAATA
3221	CaTSNP6152	TCACAGGTCTCTGAGACATCCTAATATTGTGAGGTTTAAGGAGCTCATTTTAACACCTAC[A/G]CATCTGGCAATTGTAATGGAATATGCATCTGGAGGAGAAATGTTTGAACGAATCAGCAAA
3222	CaTSNP6153	CAAATCACAACCGCCACCGCCACCGCCGCCGCGACCACGACCCCCACCACCTCCACCGCC[A/G]CCGCCGCCACCACTTCTAGAATTGTGAGAAAGCTCTACCCTCCAACCATTCTTGCCATCT
3223	CaTSNP6154	TGTTCCAGTAAAAAAGAGTTGCTCGTCAGGACTGAAGCCAATATTTGTTTGTCCATAGTG[A/G]TTTGGCAGTTCATCAAACACCTTTAGTGCTTCCTTTGTTTTGCGAAGATCCCAAACCTTC
3224	CaTSNP6156	AGATCCAGGGCTGCCGTCACTTACCTTTGCAACTATTGCTGCTCGCTCAGAGTTCTTTTC[A/G]GCAGTGACAGGCTGTTCTAATAACTCCATTCGTTTCAATGACGGAACATCTCCTTTACAA
3225	CaTSNP6157	TACTTCGGAACAGTTGAAGATACAGGCTGATAAGGCAAGGCATGATTTGAGTGAGGTGGC[G/C]AAGGAAATCACTGAAGAAGGAAAAGAGTATTTGTCTGCTGCTACTGAGAATTCACCCGAC
3226	CaTSNP6158	AGGCAGAAAGAAGAATGAAACAATAGATTTGAGGAATCTTCTGCTTCTGTGCTCACAATC[T/C]GTGTATGCCAACGACAACTTAAATGCCAATGAACTACTTAAGCAGATTAGGCAACACTCT
3227	CaTSNP6159	CATGTTTGTGTTGTTCTGTTTGTTGCTAATACAAGTTTCCGTTGCTGAAATCATTTTCGA[G/A]GAACGCTTCGAAGATGGGTGGCAAAGTCGCTGGGTAAAATCAGACTGGAAAAGGAGCGAA
3228	CaTSNP6161	GAAAAACATGAACCTGAGCCAGTTATACCAAAGTATGAAAGAAGCATATGATGTAATGCA[A/G]ACACCATTGGTTCCCCAAATGCCACCTACCTTGGGGCTGAGAGAGCTGAAAGAGTCTACC
3229	CaTSNP6162	ACCGAATTGGCCACATCCGCGCACTTTCATGGTGACATTTTCACTTCCATTTGGTCCATA[G/A]CTGAACTCTTTGACAGCTCCTCCTGAATTGAACATCTTGATTAAACCAATAGGAGCAAAT
3230	CaTSNP6163	AATACAGAGACCTGAAGCACCTCGAGCTCCCTTTGAACAGCCAAAAAATCTGCATCGCTT[A/G]GGATTGCTTGTTCGGTTCTGTGAAGTTATCCCTGTTTCCTGAAGCCCAGATGTGGTGCCA
3231	CaTSNP6164	CAGATAGGTTCAAGGCTGTTTCTGGTATGGGGCATCTTGTGGAGTTTTCCTGAGACTCGA[A/T]CTCATTTACTTGTTAGCTCCCTACTAATCAGCTGGTCTATCACTGAGATAATTCGCTATT
3232	CaTSNP6165	TCTGGTTCAAATGTCTTAGATCAAGACCGTTCTGATAAACTTCTGGGGCAATCCATGACA[G/T]CTCAACAATTTCTCTCAAATTCTGCTCCTCCCGGTGCCTTTCCAGGGTCCATGAGTATAC
3233	CaTSNP6166	AACAAGAAGCTAATACGGGAATACCCAAATCTCTTCAATTATACCAAAGATATTTTCCAA[T/A]TTCCTGGCATTAGTAGTACAGTGAACATGGAACATATAAAGCTGCATTACTATGGAAGTC
3234	CaTSNP6167	AAAGGATCAAATTGCTGCATATGTTGAGTACTTGATTCAGTTTACATCCGAACAATTTGC[C/T]GATGATATTGCTGAGATGATCCGCAACCGTTACCCGTCAAAGGAAATTCTTCTCTTTGAT
3235	CaTSNP6168	CCCAGACCAGCAAAGGTTGATTTTTGCTGGAAAGCAGCTTGAAGATGGCCGAACCTTGGC[C/T]GATTACAACATTCAGAAGGAATCCACCCTTCACCTTGTCCTTCGTTTGAGGGGAGGTATG
3236	CaTSNP6170	AAAGATCATTTTTTGGGTTAATAGCCCAAGCGACCCATTTGTTTGTTGATGTGATTCCTG[C/T]GTGTCTGAATGCTATGTCTAGTTTTCCTGATGTTTGGTCGTAGGTCCAGTGAAGGTACGA

3237	CaTSNP6171	GTGGCTTTGATGCAAGGTGAGCTCAGACATCATAGGTTCATCCCACCAATATTTTTTATC[C/T]TCACTACTACTACAATTTTTAGCATTATTTGTTGATGTCACTGCCACTGGACATTCACAA
3238	CaTSNP6172	ACCAGGATAACCTGAAAAGATAACAACACTATCAGCAGATACCCTCACCAACTCTGGCAG[G/T]GTTTTATTAAGGTATTTTGGAGATAAGTAATCCAATGCATCCGACACAATGACAAGAGAA
3239	CaTSNP6173	CACCACTACGAGCCTCTCCTTGTAATGCTAATTGAGTTGCAACTACTGCTGCTGCAAATA[T/C]TCCCACATCCAATATCCCACCTACAATTGCTGTCTTCACCCTTATTTTTTTGGGAGCATA
3240	CaTSNP6174	CCCATCCTTTTGCCTTTCCCTCCTGCGAGCAGGACAACGGAAACGCTTCTCTCTTTCACA[G/A]GAGACTCAGACTTAGATTCTTGGGAGGCAAGTGCAGAGCAGAATGTGGTGAGCCTCGAAT
3241	CaTSNP6175	GTCCCCGTAGTTTTCTGTCCATGTAGTTAATATGACGACCATGCCGATTGCCAGAAAACA[T/C]GCAGCAGCATCAAAGGGGGCAACTGGTCCTAGAGCCAATGTATCAACAAGGACATTTCCA
3242	CaTSNP6176	TACGCGACTAACACCGTCGACAAGAACCGCTTTTGCTTTTAACGAAACAACACAACGTTC[G/A]TCGTCGTTGTAGAATCGGAGACGAAGCACGGCACGGCGGGATGAAAGTTCGGAAGCGGCG
3243	CaTSNP6177	GTTGCTAATATCAAGCTTGTTAAGACTTTTGCAACTGAGAATTGATTTTGGAATATCACC[A/G]AATAAGCTATTTCCAGACAAAATCAATTCCTGAAGGTTCATAAGTTGTCCAAATTCTTGC
3244	CaTSNP6178	TACTCTAGTAGGTACTCCATTGATGTTAACACTCTTGACTTTCTTATCAGACTTATTATC[A/G]CTAGCATTAACAATAACCCCAGCTCGTCTATCGGTTTTCTTAGCCATCAAAGGAGTAGTG
3245	CaTSNP6179	TCTTCCTGGTATTAAAAAGGCACCTAAGAAGCGCATTGCTGCTATTTTAGAACCAGCTGA[C/T]GAAGGAACGGAACAAGGTAATGGTGGCGACGCCTTGGACGAGAGTGAAGAAGAGAACACC
3246	CaTSNP6180	TCTGGGACGATTCCTCCTCCGGCGCCGGAAAACTCGCCTCCTTCAACACCTCCTTCCTCA[C/T]CAACGTCTTTCGTGTCAACAACGGAACACCCGGCGAAGGTCTCGCTTTCGTCATTGCACC
3247	CaTSNP6181	TTGCACAGCAGGTTTTGCAGGAGAAGTCCTCAAATGGAAGGGTTTAGAGTTATCTTTCAA[A/G]GCATTCACAATGGATGCAAAAGACTTTTTTTGGAGTATCCTCCTGTGTATTAGAAGCAAC
3248	CaTSNP6182	AGGAGAATTTGATGTTTTCTCAAACACTCAAAGGACCAATCATCCAACCATAATAAAGGT[T/G]ATATGGGAGAACAAGGAAATGGTTGAAGATGGATTGCCACATTTGATATACATATCTAGA
3249	CaTSNP6183	TCTTATGGATGGACTAAAATCTCGTGCTCATGTTATTGTTATGGGTGCCACTAATCGTCC[G/A]AATAGCATTGACCCAGCATTGAGAAGGTTTGGTAGATTTGATAGGGAAATTGATATCGGT
3250	CaTSNP6184	CCCTCAACACTCTGGACGAGTTTTCTAAAGATTTGCTATTTTCATCTACACTTTCAAAAT[T/C]AGGATGAAGCCTCAGATCAATTTTCAGGGCAGCATTTATATCATCTGGCGGAGTTTGTTC
3251	CaTSNP6185	CAACAAACATACCCTGGTTTCTATGCCCACACCCAATAGCCACGTTTCGGATCACAGTTC[T/G]CCCGAATGTAACCGTTTCAAGGGCAAGTGTCCCTTTAGTATAGGACCCGTCCCCATAAGA
3252	CaTSNP6186	CGGCGTTCCAACAAAAGTATTTCTAGACCGTTGTCGATCACCTGAATCAAAAAGACAAGC[C/G]GAAACACCGAAATCGCCGAGCTTAACAGCACCGCGAGAATCAATAAGAACATTACCAGCC
3253	CaTSNP6187	GTCTGATGTCAACCGTAAACCTGCGGGCCAGACCGACTTGTTGAAAATCGAAACACTACA[T/C]CATGCCGATAAAGATAAAACAGAAGGGTATATACTTGAACTGGTTATCTGGCTACATCAC
3254	CaTSNP6188	GACGATGTTAACGTCGTCGTATTTCATAGCAGATAACGCGGAGAAAACGGCGTCAATTTG[T/A]GCGTCGAAAAGGTTCAAGTATCTTAACCCGTTACCCGGATCCACAACACCCGGGTTTTCT
3255	CaTSNP6189	AGCCTCCGTCAAGGCCGTCGACGTCCTCACTGCCATGGCCGTTCCTGTTGAACTCTCCGA[C/T]CGTGACTCTCTTGTTAAATCCGCTAGCACTTCTCTTAACAGCAAGGTTGTAAGCCAGTAC
3256	CaTSNP6190	CTCCATCTCCATCCAACAACCTTCTTCAAACTCTTCTTCTTCTTCTTCCTCCAATTCTTC[A/T]TCTTCTGCCATTGACGCTTCTCTCGCCATCGCCACCAGATCTGAACCTCCACTCCTCAAT
3257	CaTSNP6191	CCGTACATGATCACTACAAGCCCTTTCCAAATCCTGTAAGAAGCTACGAGCCCAATACCC[G/A]ACATCATGAGTACTAACATATCTATAATGCTTTTCATGTCTTAGCTGTTTTTCCGAATCA
3258	CaTSNP6192	GCAGAATAAATTAAAAGAAAGCAACAAAGCTGGCCTAATAGGCTTAAATTCTTCTGCACA[A/G]GTATCCAAGCACAGCCATGAAAATTCTATCATCGGGGAAAATCTTAATAAACAGAAGGAA
3259	CaTSNP6193	TGCAAGCGACACTGCCAATATGGCATTTGCCCCAAGCTTTTGTTTGCACCAACCCCACTC[G/A]TTAACAGTTCCATCAAGCTGCTGAACCATAAGGTTGTCAATTTCTGTCTGCTGAGTTGGA
3260	CaTSNP6194	TGCAAGCGACACTGCCAATATGGCATTTGCCCCAAGCTTTTGTTTGCACCAACCCCACTC[G/A]TTAACAGTTCCATCAAGCTGCTGAACCATAAGGTTGTCAATTTCTGTCTGCTGAGTTGGA
3261	CaTSNP6195	CTTGTAAAGAGTGGGATTCTTATACTCGATCATTCTGTTCCTGCCTTCGAGCATGCTAAT[C/G]GCAGAATACTTTTTCGGGGGCGGCGTAGGGATCTTATTTGCAACAGCCTTTGATGGGTCA
3262	CaTSNP6197	ACAATTTCTTTTCTTGAATGGCCTCTTCAACTGTGGTGTATCCAATTATCTGTGACTCAA[T/C]GACTTCTCTGGTAATAGCTGATTCTGATGGACCATAAATTTGAGGTTCTAGTTTACTTCT
3263	CaTSNP6198	ATTGATTCAATGTTTTGAGTGGAAACTAATTGGTGAAGAAAAAATTGATATGACTGAAGA[A/C]AGAGGAATCACTATGCCTAAAAAGACTTCATTGAAAGCCATGTGTAAAGTGATTAATCCA
3264	CaTSNP6199	CTCAATAAGACCATCCGCATCTCTATCAAATTGTTCCATGTACTCCATTGCAGCACGGAC[T/C]GCAGGCCACACATCGACTCCAAATTGCAAATCACCTGTTGCAGCAAAATCTCGATACACC
3265	CaTSNP6200	AATGCTCTGCATTGGTTGCTCAGGCTCTTCAAATTGGTTACCCATTATCTTCTCATCCAT[G/T]AGGTCCATGGGAAGATTCTTCAGAAACCATTTATGTTCCCAAATTTCAGGCATGGTGATT
3266	CaTSNP6201	CGACGACATCATAAACAATTTCATAGACCCTCCAATAAAACCATCAGTAGATCCAAGACA[C/T]GTGTTATCTCAAAACTTCGCACCAGTTCTTAACGAACTCCCTCCAACAGAATGCCAAGTC

3267	CaTSNP6202	CAGCAGAAGGGGGTGTAGCTGAGGTGGGAGCTGTTGTTGAGGCAACATTCGGCTCAGGGG[A/C]CACTGAAGTGGAGCTGCTTCCAAGTGTTCTTCCCACTCCCTGAAATGAAGCTTGGCTCTT
3268	CaTSNP6203	ACCGGATTGGGCATCACTAAATCTCGGCGTTCTTGTTTGCATTGAATGTTCTGGTGTTCA[T/C]CGTAACCTTGGTGTGCACATATCAAAGGTACGCTCTCTTACATTGGACGTTAAGGTGTGG
3269	CaTSNP6204	CACGTTAAAATACCGTGGTAGCATGTGGGGATACAAAAGATTTTTCAAGAGAACAGCACT[A/G]GAGACATCAGATTACCTTAAAGATGATTGCCTTAGTGTTAATTGTAGCGTTGGTGTTGTG
3270	CaTSNP6205	CCAGGTCTGCTGCAAAAACCCAGCTGAATTGTCTTTTCCTGATTGGGCCGTTTCCTTAGT[G/C]GACTGGCCCATTTCTGACCCCTTTTCTCTTGCTCGACCAGTTGTGTCTTGGGCTTTTTCT
3271	CaTSNP6206	AGATTTACTTCACTTCAATATTGTTGTTGTTATTCTGATGATTTTTGAGGATTCTCTGCG[T/C]CAATTTGCTTTACAAGTTAGTTCATTTTGACTTCCATTTTTTAGATAAACTTTTAGAGCA
3272	CaTSNP6207	GCTTGAAGAGTTCCGAGCATCAAAAGAGCATTTCAGAGGATTGAGTTTCCCTACTATTTC[T/C]TCCGTTGGCCCAAATGCCGCTATTATTCATTATTCTCCACAGGCAGACACATGTGCCGAG
3273	CaTSNP6208	CAAGGACCTAAAGAAGCTCCTTTGGAAGCTGCTTCTGCTTGTACTGGTGTTTATGCTAAA[G/T]GTGCTTACCCTGGTTATGCCGGAGATCTCTTGGTGGACCCCACAACGGGTGCTAGCTATA
3274	CaTSNP6209	ATGGCCGAATCAGCTTGTTCAAATGCTTGTACCCTAAGATGTCTTGTTAGGTGAGCATGA[A/G]CTAAGTATATGACAACAAACTATCTAATGGTCAACATTAGTGATAGAGTTGGGCTTTAAT
3275	CaTSNP6210	ACAAAAGTATTTTCCCATGCATACTCCACAGTTTATGCAGTACTGTTGAACATCTTGCTC[C/T]GTGTCACATGAGGAACATATAATCTTTTTAATTTCATGACGCGGTATATCATGAGGATCA
3276	CaTSNP6211	AGTTATTACTCGCGTCAACTTTTCTATCATCGTCCGTATTGTGAACAGTTTTGTCTTCGG[T/C]GGTTGAAGGGTCTTTCTGAAGAAGGCAGTATAACGAGTTAACCCTTGACATGACGTAGGG
3277	CaTSNP6212	AGAATCTAAGTTGAAATCAAATTTGGGTCGTTTGTTTTGAAGAGATGGAACATCATCTTG[T/C]TGTGTCTCCAATTTAACTTTCTTAAAGCAGTGATTATTATCAGAATCAGATCTCATTAGT
3278	CaTSNP6213	CTCTCTTCTTTTAGTAGCAACAGCTCTATGCTATATTGTGGTTGTGTTTGGAGTATTACC[C/A]TTTTCCTCAAATGCACAACTTGATCCATCCTTTTACAGCAAAACATGTCCAAATGTTAGT
3279	CaTSNP6214	CCTTCAGTCTTCATCGGTGGCTGTGAAACGATCAGCGGCGGCGAAACTGAGATTACTTGC[T/C]AAGAATCGCGCCGATAATAGAGTTTTGATCGGAGAATCCGGCGCGGTTCCTTTTCTCGTT
3280	CaTSNP6215	TGAACTCAACTCAACAAAAGGAAACAGTTACATCTGTGAAGTTGCTGTGGTTGCCACACC[A/G]CTGGATGAGATAAATATTCAATTTATTCCCCCAATTTCAATTCCTGAGAGAAAATTACAG
3281	CaTSNP6216	ATCTACCAGGCTTCAGCTTACGTGGAGGTAAGGGACATCCAAGTAATTCTGCCATCTCGT[C/T]AGGCTTCAGCTGCAATCCATTGACAGTCAACTGCTCCGGCTGAAGCTGATTAGCCGAGCA
3282	CaTSNP6217	TTTGCTTGGCTACGATATTGGCGTTATGAGTGGAGCAGCCATTTACATACAAAGAGATCT[G/C]AAAGTATCGGACGTACAAATCGAGATTCTGTTAGGAATCATTAACATCTACTCTCTCATT
3283	CaTSNP6218	TCCTTTGCATTGTGCTGCTGAAGCAGGACATTTGCAAGTTGCTTTGTTATTGATTTCTCA[C/T]GGTGCTTCTCATAAGAGTCTCAAGACATTTCAACATGCTTCCCCTCTTCAATTGGGTTCT
3284	CaTSNP6219	TTTGGCACTAAAGTTATTTACCATTTCTACAGAAGAATCACCTGATCTTTGAGCATCCCT[C/T]GCTTGATTTATATAGTAACACAAACGTTTATGATATTCAGCAAATATTTTTGCAGCTTCG
3285	CaTSNP6220	ACACAATTGCAACAACTTCAGCAATGAAGTTGCTCAATTTTTGGTTGGTGCTACCATTCC[G/T]GATCATATTCTTCAGCTCCCTAATGAAGTAATGAGCAGCCCAATGGGGTCTCTTTTATTG
3286	CaTSNP6221	GGTCCATCGCCGGACGGTGATGGTGATCATACGGTGCCGGCAAAGGCGCCCGAACAGTTG[T/C]CGGCGGCGGAGGATAGTAATTAGGGTAGTTAGGATACGGAGTAGTGGCGGCATTGTAAAC
3287	CaTSNP6222	TCGTCCATTTTTTGTGGACCCAGCTGTCTTTTTGGTAGCCCAACGACGGAACACCAGACT[C/T]AATCCCTCACCCGAAACATCACCAGTGCTTCTGTACACAGGAACATTTGTCACGAGTTCC
3288	CaTSNP6223	GGCCAACCCGGGAAAAAAGTCCGACACATTGGGTTTCCCCAAAAGCCCTACCATCTCCGC[A/C]ACCACCTCCCTGAACTCCGCCCCTAATCTTTCCCTCTCTTCCCCTTCCACCCCCGCACCC
3289	CaTSNP6224	AAGAGAGGGGATTATTATGGTCAATGGAGTGCTATTGGAAGGACAGCTGTCACAACTACA[T/C]TGGCTGGATGCACTGCTGCTCTCACAACTCTTTTTAGTAAACGGTTATTGGCTGGACATT
3290	CaTSNP6225	GCACTCCTTGTTTTCTACCATAGCATTGCTGGTGGCTACTCCTGGCCTATCTGAAACATT[T/C]GGAGTGGAAATTTGGCAAGGTTCCAATATCCTTCCATTTTCCGCGAACGGATGAGAGGAG
3291	CaTSNP6226	TTCCTTTATTATAGTAGAACGCTCATGAGGCTTCCCATGTTCCAGTACATGCTGAACGAC[G/A]TAGTTTCCATACTGATCCTGAGCTAACATGCTAACAGTTCCTAAAATCTCATCCATAACC
3292	CaTSNP6227	GTGAAGGGGAATGATGAAGTTCGTCGTCGTCGTCGTCTTCATCGTCGTCATCATCATCAT[G/C]GTGGTCTAATTCATGGTCTGGTTCGGAATCGGAGTGGAAGTGAAGGTGGGAACCGGAATC
3293	CaTSNP6228	GAGATCGAGGAACCACGAAATGCCGGAGAAGGGAACTATGATGGTAGACCTTGTAGCAGA[T/G]GTTTTCTCAGAAATCTTTAAGTAACGACCACGAGGGTTTTCTTTCAGATCGAAGTAAAAC
3294	CaTSNP6229	GAGATCGAGGAACCACGAAATGCCGGAGAAGGGAACTATGATGGTAGACCTTGTAGCAGA[T/G]GTTTTCTCAGAAATCTTTAAGTAACGACCACGAGGGTTTTCTTTCAGATCGAAGTAAAAC
3295	CaTSNP6230	GGCAGAGAAAACCCATGACAGTTTCATAATCCACAACATATTTTTTTAATAAAATTTTTG[G/A]CCACCGCAAAAAGTAAAACCCCCTCCTACTCCAAAAATCGAACCGGTGAGGATTTTTGAT
3296	CaTSNP6231	TAACATTTGGGATGAGATTAGTAATGCCGGACAGGAAAACACCGATAACGGTGAACCCCG[A/G]ATTTATATCATCAGTTGGAATGACCATTTCTTCATCCTCAAAGTTGAAGACGACGCTTAC

3297	CaTSNP6232	TCCAAAGGTAGTATCATCCAAGCTATCCTTTATCTCTGTAGAAGTATTGACATCATTACA[C/T]GCAGATACACTAGCATCTTTCCTAGTAGTGCAGCGGACTAGGCATTTTATGGTAGTAGGT
3298	CaTSNP6233	AAGCCATTGGTGAGAGGGTAGAGTTTTGAGATTTTGTTCAATTGGTTATAGATTTGAAGG[A/T]AATGGTGGTGAATAGTAACGCGGTTGAGTTGACTCGGTCTACGAACCCGTTTCTTGAACC
3299	CaTSNP6234	TTGTCAAAACGGTTCGGGATCTGATGTGAACGACGTTCACGTGAGTGACACTGTTGCGGA[G/T]GTTGGTGATGAATTCGCAAGTGTTCAAAACGGTGTGTCGGATAAAGAATCAAACGAGATT
3300	CaTSNP6235	CATCCTTTGGTTGTGATGCTGATGCACCTTCAGCTGTAGAACCGGTTGCTTTAGATGATG[C/T]TTCTTTTCCATCCTTACCATCTTTGGCATCTTTAGCAGGAGCAGGTGGTGGAGGAATCAT
3301	CaTSNP6236	ATCTTGCAAAATCGCATACAAACCCTAAATAACAAGTTCAACTAAAACAACAATTCAATC[C/G]ATAATAATTCTACGAATTAAAAGAAAAAGTTTTCCACAGCATAAAACATGAACCGAAAGC
3302	CaTSNP6237	TGTGGAGCCATAAGTCAAAGATGGAGCTTCCAACAATTCTATCTACCAAAGATGCCTTAG[G/C]TTTCGGTCATTTAGCTAATGTACCATGTCAGCTTTAACTTTCTGTAACTTCTGTATAGGC
3303	CaTSNP6238	CGCAAAGAAAGCACTTATTGGTACCACACACGTTGCATATATTTCAAATGTCATTTTGAC[C/A]GGAGACACAACCTTAAGGACACGAACAAGGAAAAAAGCAACAGCACCAGAAAATCCCATA
3304	CaTSNP6239	AAGACCAGAAACTCTCTCGAGGAAATCTTTTAGGTGGGGACGGCAGCGTACATAGACACT[A/G]TGCTCCTTTTGATTAAAATCCACGGTGAAAGTAAAGTCTACATCCTCACAAGGCTCTAGG
3305	CaTSNP6240	TCGAGTACGGGTCGTAGCAGTATCCAAAACCAAACCTATTTCCATGATTCGCCAACTCTA[T/C]GACGCAGGTCACCGTTGCTTCGGCGAAAATTACGTCCAAGAAATCATCGATAAAGCTCCT
3306	CaTSNP6241	TTGGGGTTTTTTGCTTGGATTTTCCACTCTTTGGGTTTTCTTCCTTAGACTTGGCAGAGG[G/A]CTTTGGCGTGATAGTGCTGTCGTCCTTTGGTTTGCTTGTCTTGGAACTACCAATGTTCTT
3307	CaTSNP6242	GAGCGTATCTCTGTACGCTCTGGCGATTAGAGAGAGTGTGGTGTCGTTCACGCGCGGACA[T/A]AGTGTGAGATCGAGATTTTCAACGGAAGAGTAACGCGCGAGGATTGATGGTATGTATTCG
3308	CaTSNP6243	CAATTTCCGCCACATCGATCATACAACTCTCTCTGAGAATTCAAACCCATCATGGAATAT[G/A]TGGCATTCGAAGCACACAATTGAAATGCAGCAAAGTCTATCAATGACTTAAACTTGAAAG
3309	CaTSNP6244	GGCTGGTCGTATGGCATTTTAGGTCAATCTCATCGATGTACACTCTGTCAAATTCCTTCA[A/G]CGATATGGTGGGCTGAAAACGGTATTTTATTAAGCTTCTGACAGCCAGTGTTCAACATTC
3310	CaTSNP6245	CACCATGCTAGAGTCATGCCACATGGTTTATTGCTCTAGCATAACTTCAGCTGGTGTGGC[T/C]ACCGTGGTTTCTAGTTGTCCCAACATAAAAAAGGTTCTTGTTGAGAAGTGGAAGGTTAGC
3311	CaTSNP6246	GTGAGGAAGCTAAGGTCTCTCTTACGTTCTGCTGGATCAGCTCTAACAGTGGTATTAACA[T/G]AGTAACCATCTTCAAGTAGTCTTTTGATAATCCATGAACCAAGAAAACCTGTCCCTCCAG
3312	CaTSNP6247	TAACCAACCATGGTTCATACTCTGAAACCTTCTTGGAAACAATCATCAATCAGGGTGACA[T/C]TTTGATGATAATTTGATCATCAAATAAAGTACTTGGTCTTAAGAGTTTTGAAGATTTTGG
3313	CaTSNP6248	ATCAACACAGGAACCAGGCCAAGCAGGTGGAGGCACTGGATCAGTTAACAACTTGGGGCT[G/C]GAGATGTTATCAAGTATGTTTGGTGGACTTGGAGCTGGTAGCCTAGCTAATCCAAACAGA
3314	CaTSNP6249	AGATCTTGAAAAGCAGTATCACGAAGACAAATCACTCATAAAAGATACACTTAAGTCAGG[C/A]AAGATTACCGTAGTGACTACATCAGTATTTGAGGACTTCAAGTCTGTCGTATTAGAAGAA
3315	CaTSNP6251	ACAACAGGCACAATGGGAGCACCCAAACCATGGAGGAGTTAATCCATCTTTCTTACTACA[G/T]CAGCAACTTCCAAGTTTGAACATGGGTGGCAATTACCGTGAGGAAGCACCGGAAATGGGG
3316	CaTSNP6252	CAACCTGAATTCTGTTGAGAAGCGTGAAGTTTCCATTGTAATTCTGGAAATAGTTCGAAA[C/T]CTTGATGATGCATCACTGGTCAAGGCATGCCAGCAAAGCATTGCCCGGACTAGATTGTTT
3317	CaTSNP6253	TAAACCAAAAAGAGGAACAAGAATTGTGAGTGTGAGAGATGCCTTGGAGCAGAATGTCAG[G/C]GTCAGTATCTTCCCCTACGGTGGATGTGGTTATTGACATGGGCAATCCATTCCTAAACCT
3318	CaTSNP6254	GTTGTAACATTCTCAATGTGTTAGGTGGAAGAAAAAGATTCCAAGTTTGAGATAGTTTTA[T/A]TATATTTTTCATATTGGAGTGTCTTCCTGTGTAATTTTGTTCATTTGTGTACAATGTTAA
3319	CaTSNP6256	CAGGGTTCCATGGATAAGGCAAAAGGGTACTCGGAATATATTGCAATTGAGGAGGGAAAC[C/T]GTCAACTCTTTTATCCATTGATCCATTTGAGCCAGTGTGGTCTTTCCTCTCTGACAAAGT
3320	CaTSNP6257	CTCTTCCCTAAATCACGAACTCATTGATCTCATCAACCGCGATTACGCTGATTTCGTTAA[C/T]CTTAGCACTAAACTTGTTGACGTGGACGCCGCTGTGGTTCGTATGCGGGCTCCACTGGTT
3321	CaTSNP6258	AATCTCAGAAGCTGGGGGGAAATGGCCTTCTCATATGGAAGCTTCAGCTTTTAGCACACC[A/G]CCTCGTAACATGGGGACAGAGGATTTGGGTATACTGTTGAAGGGCCATAGGTTCCGTAGT
3322	CaTSNP6259	GCACATCTCTTTCACATCTTTGAAAGCCTTGGTCATGTATGTCTTTAGCTCATTGTTCAC[C/T]TTCTCCTCATCCCACATGAACCCTTGGATGTTCTGAACCCACTCAAAGTAGCTAACTGTA
3323	CaTSNP6260	ATTAACTGATTTTGATCTATCATTTATTACATCTTGTAAACCTCAGATTGTGAAGCAGTC[G/A]TTACCTAGTAATAGAAGAAGATCTCGAAGTCAGCCACCGCCAATATTTGTCGCAGAGCCA
3324	CaTSNP6261	GGAAGGGAAGAAAGAGGAAACTGAGTTGGGACGAGTTTTGTCTGACTCGTCTGGAATCGG[A/T]GAGTTATGGGAGTTTGAGAATCAGATCCTGAGTTATGACTCGTTCGAGTTAGGAACCGGA
3325	CaTSNP6262	GGGTTCTCCGGCTTGTGTCTGACGCTCAGACCTTCTATTTCCAACCCACTGTTGCCTTGT[T/C]TGGTTCCAGAGAAGAAGACCATGATTAACAAATTCTGGAGGATCAGTCTGAATGCCTTCA
3326	CaTSNP6263	CGCCGATAAAGAATTTGTCAGGAATCACATCCTCCTCTCTCTACATCAACTTCCTCCTCT[C/A]TTGAGGGTTCAACTTGGTGAGTGCCTCCGAACGATTCTAAATTCTGATTATCCAGACCAA

3327	CaTSNP6264	CCATGGTAAATCAAATGAAGGATCTTTCTTTTGCCATCTTAACAAATCACTAAGTCTTTC[T/C]AGCAAACTCATTGGACATATTGCAGTAATCTTCACAATCACAAAGCTCACCGAAGATGGA
3328	CaTSNP6265	CCTCAGCCTTGAATAATCTCCATAGATAAATTTCTAGGACAAAGGGAAAAAAGAAAATGA[C/T]GATGGCTTCTAAACTTTCAAAAGTTAGACAAAACTAACATTGAAAGTTTGTTGCCTAATG
3329	CaTSNP6266	CGAAGCAATCTATCATGACAATAATTGTGCTCTGAATTATGGCTGAGTAATAAGGTTACA[C/A]TTAAAACTGTTTCTTTTTCTTTTCCAAGTAAAAATAGTGTCTCTGAAAGTTCAACACTCC
3330	CaTSNP6267	CGATTCTTTTTCTGGTGCAGGATGTGTATAATGATCCAACTCCCTCTTTGTGCCCCCATC[A/G]TTACTGTCATTGCTTCTACAAGGGTAATCGAGATCATCTACAACTTCCTCTTTGGTTTCA
3331	CaTSNP6268	ATTCCGGAAGGAGCCCTTCTTTTATGGACATGATAACTATGATCAACTGGTCAAGATAGC[C/T]AGGATACTTGGGACAGATGAATTAAATGCATATTTGAATAAGTATCGTATTGAGTTGGAC
3332	CaTSNP6269	ATTCCATTATAGTAAATCCACAAAAACAAGCCAATTCTGCTGCAGAGATTCCACTCACAT[A/C]TTGAACAAAGTTAGCCAATGGAGCGAGCTAACAATTTATTATCGCTATGCATTTTATGCG
3333	CaTSNP6270	AGGTGCTATTCTTGATTGGAGTGATTACTACTTTTTGCACTATTCTCCCTTCTCTCTTAA[G/A]GACTACAACAAATGGCCTTCTTCTCCTCCATCTTGCAGAGAAGTGTTTGATGAGTATGGA
3334	CaTSNP6271	GCTCCTTAACCTGCACAATGTTGCCTCGAAGAATGGTGACGTGCAATTGACAGACTTTGT[G/T]GAAAGTGAGTTTTTGGGTGAACAGGTGGAAGCCATCAAAAAAATCTCAGAGTTTGTCGCT
3335	CaTSNP6272	GAATTTATGTGGCAATATGCACTCAAAGTTCTTGGCTTTCTTGAGAATATCTGAAGAGGC[C/T]GTGGAGGTAAAACATGAACTGATTGATCTGCAAAAGCATATTTCAGCTCAAGGTATTCTT
3336	CaTSNP6273	TGGTAGAAATGTTTGATGAGTTCCTTGTATTGAATCAAGACCAAGAGTGAGTGAAACACC[A/G]TTACTTGAAAACCTTGGTGTTGTGAATTGTTCTGAGTCAAACCTACCAATATCACCAATT
3337	CaTSNP6274	ACTCACCACCACACTATGAATCCCTGAATTGCTGCCGCCATTATTATTATTAGTAACACC[C/T]GAACCCGGCGACGGTGAAGGCGACCCACCACCAGGCATAGCACCACCCTCCCGAAAAGAA
3338	CaTSNP6275	AACTTTTGAAAATTATAGCTACATGGAGGAAGATGATCAAGAGAATGGAAGTCCAGCCGC[T/C]GTTCCAGTTCCGTTGCCTGATATGGTGTTGCCACCATCATTTGACAGCGACAACCCGGCC
3339	CaTSNP6276	GCGTGTGGTCCGATTGAGTGAGGTGATGCCGAGAATATCAGATGTTGATGTACAGACCTC[G/A]CGTCAATGTATTTCTGCATTCTTGTCTATGCACACAAATTATGAATTACTTCCTGAGTCA
3340	CaTSNP6277	GGTAAGCCCTTACTACACATCATAAGGTTGCTTACCAGTGAACTTGACCTCAATTGGGTT[A/T]ACATTCTTCCCAATAGACCTGAAATTCTGACCAACCCCTTCACGACCAGGATTAACCTTC
3341	CaTSNP6278	CCTCGTTGATGATAACTGTCCAAGTTTAATGAAAGTTGGACCGAGCTGTAATACACACTC[T/C]CTTAGCCATGAAGCCGTTTTCCGCATTCTGCTTTTCTGCTTTTCTTCTGTAAAGCCACCT
3342	CaTSNP6279	CTATTCCCTGCCCAGCACGGGAAGTTTTGAACAACCTATGAACTTCAGGATGCTCAATGC[C/T]AGTTCCATTCTCATTATTTTCAATGTATCGGTGGTCATCACTACAGTCAGAGGACGAGAT
3343	CaTSNP6280	ATCCAGCGTCACATTACGGCGGCATCTCCGGGACAGCTTTTCCGATAGGTCTGGCTCCGG[T/C]AGCATTGCCGGTGGCGAGTGGTGAAAGATTGATCACCAAGCCAATTAGGCCAACGCCAAT
3344	CaTSNP6281	GGAAACTGGAATTCCAATTTATGAAGCTGAGCCACTTCTTCTATTTTTCATTATTTTCAC[A/G]TTGCTAGGAGCCATTGGTGCTCTAGGTGATCGTGGTAAATTTGTTGATGATGAGCCTAAT
3345	CaTSNP6282	ATCTGCTCGTCATTGGATTGTTAGGAACACGAGGAGTGTGAACCGTACCGGGTATCTAAC[C/T]GGTTACAAACTAGTTCCTGGTGCAAATTGTTTACCTTTAGCTGGATCAGAGGCTAAGTTT
3346	CaTSNP6283	GAAAGCTGAGGCTGTGGATAGGATATGCAAGCCGATTATGACAAAACCAAGGCCAGCCAA[G/A]CCAGCTGCTCCACAAGCACCACCAACCCCAGCTTCTCCCAGTGCTGAGCAACAGCAGCAA
3347	CaTSNP6284	TTCCATGGACTCCGAGGATGATATGCACGATGCTAACGACGTGGAGTCCCTTGAAGACGA[T/C]GACGATTTCTACAGTGGTGACACGGAGGATGCTTCCATGGATTATTACAGCGATTACGAT
3348	CaTSNP6285	ATGAATGGATGAAGTTGATTATTGATTATTAGATTATAAAGTTAAAACCAAGTGATTCAT[G/A]ATCACATGCAATGCCACTAACTGCAAAAAGAAACTCTAAGTTTATAATATTTCTTTTTAG
3349	CaTSNP6286	GAAGATTCCGCAGAATTGCGTAACTTATTCGGAGTTTATTGAAATGTGTGCTGAAAATTG[T/C]TCCGATCAAGAACAAGCAAAGAAGATTGCGAAGATACTTGATGATTCAGCAACTGTAATT
3350	CaTSNP6287	TGAAATTATCTATTTATTTAAGGAAAATGAAAAGAGAGTGAAAGAAAGGCGACAGAGATG[G/T]CCGCGGCGGGATTTGAACGTTATTACATCGGAGGAGTAGGTGATGAGTGAAGAGGTGGAA
3351	CaTSNP6288	ACAATACGGACACCTGTTGCTACCAGCACTCGAAGATCCAGCAGTCACAGTGGATCAACT[T/C]CATTGGCATTGATGACCTCTACACCAGACCAAAGTGGTAGCTACGTTTATGTCCCAGGAC
3352	CaTSNP6289	ATGTCTGCTTTGGAGCCGCCAATAAAAGAAAAGCTTTCAAAATATCAAGTATAATTCAAT[T/C]GAGTGAGCATTGTATCTGCATTAGCCTTGGTAATTTTGTGTTGTCGAGCAATATTGGAGT
3353	CaTSNP6290	AAGGCGGGCTTTACTCGTGGTTTAAAGAGGATCTACCAAGTGTTTCAGAGGAGGAGTGAA[C/T]GAATGAATCTATTGCTAATATTCTGCATGCAGAGAAGCCTGGACCTAGTTTTGACCATAA
3354	CaTSNP6291	ACAAAGATTCCTTGGAACCCTTGCTTGATTTCCTCCGTGTTCACACATATAAAGGCCATG[C/G]ACTGATGTTAAATGATAGATTACAAAGCATTTCCAAACTTCAGTCTGCTTTGGTCAAGGC
3355	CaTSNP6292	TCCATCTTTCCGGAGCAAGGAATTCAACAGGATTGCTGATTTGAATCCAACAAACCTTGA[C/T]GACCTGTTAGCATCTACTGATCCTTCTTTACTATCTCAACTACACGGACTCTCTATGCAA
3356	CaTSNP6293	CGCTTAGCAGCCGAAGAATTTCTGTTATCAACCTGAGACATGACGGTAAGCAGTGACTGC[G/A]AGGGAAAACCTAAGACCCGGAGATCCTTCTTCTATTTCTCGGTCGGGGCGGTAATGGGAT

3357	CaTSNP6294	GCACATTTCACGGAAAACGGATCAAAGAGAATGAATTCGATGCGTCGAATACAAGTAATC[T/A]ACAAAATCACATAGAGGCTAGGGTTTCTATCTTTCAGTTATTCGCTATTGCGCTTTTTAG
3358	CaTSNP6295	TGATTTCAAAGAGGTCACTGAAACTCCTAAAACATCTAAGAAGCCTATTGAAAGAAGTGT[A/T]TCTAACTCAGTGCCTCGATTCATGAATTCAACGGTTGCTAGTCGGCATAGGCAAATTGCT
3359	CaTSNP6296	AGAAGAGGAAGATACGTGCCCAATATGTTTAGAAGATTATGATGCTGAGAATCCAAAACT[G/C]GACACAAAATGTGACCATCATTTTCACCTTGCTTGCATTCTGGAATGGATGGAAAGAAGT
3360	CaTSNP6297	ATGCAATTGTTCATCACGTAATCGGTTAATCTCTGAGACTGTTGAATCCAATGATTGCAT[G/A]TCTACAATATATCTTGTATGCAAATGACTGACAACTGCTTTTGCTTTCTCCAATGCTTCA
3361	CaTSNP6298	GTTGAAGGCATTGAACATAGATGGAATCAACATGGTCTTGGAGGGGATAATGTTGTTGAT[G/A]GTTGTAGGAGTTTGCATCATGGACCTGTTAGTTTGTTGCATTGGAGTGGTAAAGGGAAAC
3362	CaTSNP6299	AATGCATTGAAATAAATCTCAATTATACAGTATTCCTACTGTTAAGTCAACACAGCAAAG[G/A]GGACACCAACGTCTCCACTTAAACTACCACTGAATGGTACAAAGGAGGAAAAATTCACTC
3363	CaTSNP6300	GATGTTGTTTTGTTTGGATCGAGGGTCAATATCGACGAATACGACGTCGTCAGCGACGGA[G/T]GAAGAAGAAAGGGGAGAGGTAGCAGAAGTTTCTTGGATGAAGCGTTGGAAAGCCCACTCG
3364	CaTSNP6301	CATCACATCCCAAATACCATCACATCCTATGATGAGGAATTCGTCATCCTCTGTTAGAAC[C/T]ACTTGGTGGAACTCAGGCTCTGCTATCAAAGGTGAGGGAGTACCTCTAGGTAACTTCATG
3365	CaTSNP6302	ACAATGTGATTAAGAATAAAAGGAAATCAAATGAAACAAATACATATTTCTTTTAAGACT[C/G]AAGAACTTACTACCTTCCTAATCACATCACATGCTAAGAACTTTAAAGGCAGTAGCCTCA
3366	CaTSNP6303	ATTGAGAAATCAAAGCAAATGTGGTCTGACATGCTTCAATGGAGGAAGGAATTTGGTGCT[A/G]ACACCATTGTTGAGGATTTTGAATTCAAGGAACTAGAGGAAGTGTTGCAATATTATCCTC
3367	CaTSNP6304	TGAGTCGCTGAATCGGAAAGTCGAGGAGTCTGATAAATTAGCAGACATGAAAGTGGATGC[T/C]GCCAAGGCACAGGTAGAAGCCGTGAAGGCTAGCGAAAACGAGGTTTTGCAAAAGTTGGAA
3368	CaTSNP6305	ACCAGAGGTTGACCAAGTTGGTTCGGACGAGCCCATTGAGTCACCTGAAGTAGACCCTGT[T/C]GGCTCTTTTATTCTAGAAGGTTTGTAATCAGCTGGGCACTACTGCTGTTCTTATCAGACA
3369	CaTSNP6306	ATTGCCAACTGATGCTGCTCTTTTTGAAGATCCTTCCTTCAAGGTATATGCTGAGAAATA[T/C]GCTGAAGATCAGGAAGCATTCTTTAAAGATTATGCTGAAGCACATGCCAAACTCAGCAAC
3370	CaTSNP6307	TGGACTCGCACTCAACTCAACTAACCATCAAAATCTAAACCTCGCCGGAACACGCCGCCC[G/T]ACTTCATTCTCGCCAGAACACCACCGTCTGAAACAGTCAATAATTATGCTGTTTTAAATT
3371	CaTSNP6308	GATGCAGAAATACATGGTGAGGCTCGTGAAAATAACAAAGTATAAAAAGTGTGTTCAAAT[T/A]ATCTCTATCTACTCATTCATGCGTGTAGGTGTATCCAAACACTTGGCCAAGACAAATAAG
3372	CaTSNP6309	AACAATACGCTTGCTGGATATCCAGTAATGAGTATATGGAAAAAGAGTGCACTCATCCCC[A/T]TCTACCTTGTGAGAATCGTGTTGATAGAAGAATAGAGCAAAGGAAATTAGGAGTACTAAG
3373	CaTSNP6310	CGTAATACTTGATGAAGCTGACAGCATGACATCCGGAGCACAACAAGCTTTGAGAAGGAC[A/G]ATGGAGATATATTCTAATTCTACTCGTTTTGCGCTTGCTTGCAATACATCTTCTAAGATT
3374	CaTSNP6311	CAGTGCAAGCATAGGTGCACAGAGGGTGAAAAACTCCTGCATTGCTTTTCGCAACTTCTG[C/T]GAGGAACAAAACATAGATGCTTGCCTCAGATGTTTGCAGCAAGTGAAGCAAGAGTACTTC
3375	CaTSNP6312	GTTGGAAGTATAAGAACTGTGGCTTCTTTCTGTGCTGAAGATAAGGTTATGGAACTATAT[G/A]GGAAGAAATGTGAAGGTCCCATGAAAACAGGGATAAGGCAAGGGGTGATAAGTGGAGCAG
3376	CaTSNP6313	TTCAAAGAAGCAGGAAAGGAGATACCTAATATATGATCTGATGGCAGTCAACTTCATTTC[A/G]GTGATAGAGCGACCTTTCTGTGAACGGTGGAAGATGCTTGAGAAGGAAGTGATTGAACCC
3377	CaTSNP6314	CGAGAATTTTAAGGTTGATAGTCCTAATGTTAAGTACACCGAAACTGAGATTCAATCTGT[T/C]TACAATTATGAAACTACGGAACTTGTTCATGAAAACAGAAATGGCACTTATCAGTGGATT
3378	CaTSNP6315	ACGGATTGTTCCAGAGGTTGAACATGAGGCCCATGTGCAACATGTTCCCAGCAAGAAAGC[G/C]GCTTCTGAAATACCCACTCCTCAGTTTGTTTATGTGGATACATATGAAAGGGACTATTCT
3379	CaTSNP6316	GGCTGGCGTTGCGATGGGAGGTTGAGATTGGGAGGAAACTCCTCCGGTGGTGTTTGTGTT[G/A]GATTGGGAAGTGGAAACGGGAAGTGTCGGTGGAGAAGACATGGTGGCGCGTGAATCTCCG
3380	CaTSNP6317	CTTACAATCTTACACGTTAATCGAACATTCACTCAATTTCTTTTTCATTTAACTTCGCAA[T/C]TACTCTGCTTCTCCTCTGTTTTCTTCATACTAATATTGTTCTCCTTCTTATTCACCTCTG
3381	CaTSNP6318	TAACAACCTCTTCTTCCTCGATTTCGTGCGATAACTGGAGCAAGTATCAGATGTACTTTC[G/A]TCTTTTTCACGGGTCCTTGCTGTGCTGACTTCTTCCTCATCATAGTTCGCTAGTTCATCC
3382	CaTSNP6319	ATCCACGTAATTATAAGGAAATTCAAGGTGCTTTTCCCATTCGTTCCATTTAGGCTCGCT[C/T]TGCACCTCGCTTGCAAACTCCGGCGACACCACGTGTTCTGAACAGCTCGAGTCCGTGTGC
3383	CaTSNP6320	CTGATGAGTGAGTTCCTCCACTCTGTCCCTTAGTCTAGAAGACCTCGTGTCTGCCAACTT[G/C]AGAGATTTCTCTGCTGCATCTCTGGCTGCCATGGCAGCAGCAGCTGTCTCTTCTTTGAAT
3384	CaTSNP6321	ATCTTTAGCTCCAGAACCAACAAGTACAACAATCATTAGGAGGAGTCCCCCAGCACCACT[T/A]GCTGATGATACTACTCCAGCAGATTCTGATGAGTCACCAGCACCAGCACCAAGTCCCAAT
3385	CaTSNP6323	CGAGCCACTTTTCTTCAATCTGTAATCAAAGTATTTCTTGAATATGTCGTTCACATTAGG[A/T]GTACGAGGAAGTTTAACAAGCTTGCCTAGATGAGTAATAAACTCAAAATCATCAACTAAT
3386	CaTSNP6324	TCCTTCTATGGTTGATATGGTGACTCGTTCGCAGACAATGGTGTTTGGCGCTCCGCTAAG[A/G]CGTATTCTCCAACCTGCTAAATCATTTCCTGAGAGGCACAGAAGATAATAACAGCACTGG

3387	CaTSNP6325	TATGGTCAAATCCTTGTAGAATGCCACAGATTATACTGTGAGCAACGCCTCTCTTTGATA[C/A]GAAGCATAGTTCAGCGGCGAATATCTGAATTTTCCAAGAAAGAGTCCTTGCCATCATTGA
3388	CaTSNP6326	CTCTAGATGGTTTCCTCTTGGGAAATTTCCTTTTAAAGCGTGCTTTCTTAGGCTTTGAAG[C/T]CTCGTCGACAACAGCGACAGAATCAGTTGCCCTTTTCCTGGATGGAATGACTGTTAAATG
3389	CaTSNP6327	AGGTTGAACACCTTGGGTTTCAAATCCCCAGAAATGACCAGTGCGAGCAAACCCAGACTG[G/A]ACCTCGTCAGCAATACAAAGACCTCCAGCTTTTTTAACAATATCATAGGCAGCAGGCAAG
3390	CaTSNP6328	TTTCAACAACCGGGGTATGGTGGTCATGATAATCATTATATGGAGCCTCATGGTGGGTAT[A/G]TCAATCATGGTAATTATCCCATGCAGCCACCTCAAGTACCGTTTTACATGCACCCTCATC
3391	CaTSNP6329	CAATTCAGAAGCCTTCCAACTCCGACCATAGACAACTGGTTTTTCATCATCAGGGGTCCT[G/A]TCTACCTCAAAGAACTCTTGAAGAGGGTTATGCCCTTGACTGCCAGCAGCAGCAGTAGTT
3392	CaTSNP6330	GAGTGTTTCCTTTTATCAACCTACAACTGAGTCTACTCTACCTTAGCGTGAAATGAAGCA[T/C]GCGCAGAAGCGAAGTAATGATGATGGAGGAAAGCGTCGATTATAAGGGAAGAAGTGGTTG
3393	CaTSNP6331	CACCTCCAAGTCATCTGTCCACTCCAGCAATGCAATCTCAGATGGGCTGAAAAGACTACA[C/T]GCTTGATCAGTTATATCCAACAAGGATGCTTCCTGTTTACATAAGAACCAGAGAGATGAT
3394	CaTSNP6332	AGGACTCCTTCAAGCTCAACGAGATTCCTCTTGTGGTAAGAAGAAGGAAGATGAAGAGAA[T/A]GTGATTAGAGGTATATTGGATGGAAGTGGAGAGTATACTCTTGTATATGAGGATAATGAA
3395	CaTSNP6333	ACTATCAGCTACTCCAAGATCTTCATGCTCATCCAATGGGTTTCTGGCAGTACTGTACAG[A/G]TATGTAAAAATTTTGGGCAGACCACTTCTTGCTATTCCACCTCCCTCATCTGATATCTTT
3396	CaTSNP6334	CTCACAACAAGCTCAGACAAAAACTCTCTCTTCTTCTTCAACACAGCAACGGGAACTCTA[G/A]CAATAACAATCGAGAGAATAGTGAGAAGCGCGTCGATTAGGTGGTTAGGCGGATTAGTTT
3397	CaTSNP6335	CAACAGAATCTCTCTGCAATAGAGTAAGCTGTGCAGAAAGTGTAGTGGCTTCAGTCTGCA[G/A]AGTCTGAACTTTATGTTCTAGTTCCGATATATAACGCATCTTGCGTTCTTTCGACCGCGC
3398	CaTSNP6336	TTAACATCACACAAACCTTTCAAATCGCCACACAAACTAGGATTACCAATAAAACTATCC[C/A]TATACATATCCTTATTCAGAAGAGGGGGAATTTCTCCAGATAGCTTATTGTAAGACAAAT
3399	CaTSNP6337	TGGAAATGCAGCTTCATTAGTAGACGGTCCTGCATTTTCTAGTCTACCAAACCTGCAAGA[G/A]CTTTACCTGAGAAAAATGCGTCTCACTGAAATTCCGTCAGATATATTGGGGTTGCATCAA
3400	CaTSNP6338	TTCATTTGCTTGCTGCCTCATATCCTGTCTGATTTTCACATAATTATTTTGCAGTAATTT[G/A]GCTGCAGCTTCCGATAGACGAGGGTGGCATTCAGTTCGACAATATTGTAAGTACCTCTTC
3401	CaTSNP6339	GCTTTCCCTGCTCAAGTTTTCCGCAGAAATCTTCTGGTCAGTGGTACCATATCGCAACCA[A/T]AGGCATGATATTCTTGCCAATGCTGCCTGTCCAGCAACTCGCACACGGTAAACAACGCTT
3402	CaTSNP6340	AGACCTTAAAATCTCCGATTTTGGTCTCTCCGCTTTGCCAGAACAGCTTCGGCAGGACGG[G/A]CTTTTACATACTCAATGTGGGACCCCCGCTTACGTGGCACCCGAGGTTGTTAGAAAAAGA
3403	CaTSNP6341	TATTCGGGTGGGACATCAGTTTGGACACAGTTCAGACTCCCTCATCAGATTATGTCACCA[G/A]CTGCAGGTGCTACCTCTTCTGTGTCACAGCTGGATTTCCTGGGAGAACTTAGTCGCCAAA
3404	CaTSNP6342	TATGTCATCATAAACACAAATTGAACCACCATAAAAAATTGTCAATTGAGTAGGTGTACC[A/G]TTTGATTTGGAACAATTCCTATTCCTTAAATCAGTTGAACCAATTAAGGAACCTATTGAT
3405	CaTSNP6343	GGAGTTTTGGAAAGAGAAGGGTTTGAGTTCTATGATTGAAATTGTGACTATGGATTACAT[T/C]AACAAAGCATTTGAAAGATTGGAGAAGAACGATGTGAGGTATAGGTTCGTTGTGGATGTC
3406	CaTSNP6344	GTCCGTTCTTCCAGGCAATCTCGCAGCAATTTGAGACCATTTGTTACCAAGGAAAGTGTG[G/A]AGGCTAATGATGAGTTCATCTTCTTGGGGAGTGAAATTACCTCTCTTGAGATCAGGTCTA
3407	CaTSNP6345	TCCTGTGGAACAAAGGCTTCTTTGGGTGGACTGTGTGGAGCTCATCTGGGTCACAATACT[T/G]TCAACATATTCAAATGAGAATTCACAATCCCGAAAATCTGAGGAAGTGTCAGAAACAATG
3408	CaTSNP6346	AGGACTTTTTTATTGCTTTGTATCTATGCTCTGTTGCTTTTTATTTCTTTTCAGTTTGTT[G/C]AAACATTTCTCATCCCACATTAATACAACTATCTATGTCAATTCAGATTTTGAACAACTA
3409	CaTSNP6347	AGAGATTTCTAAGGAAGAATATGCTGCTTTCTACAAAAGTCTTACAAATGATTGGGAAGA[T/G]CATTTGGCTGTAAAGCACTTCTCTGTTGAAGGTCAATTGGAGTTTAAGGCCATTCTTTTT
3410	CaTSNP6348	TGAAGTGTACACAAAAACCTACAACAGTGAAGAGTACAACAACAATTATGTGAATGGTTA[T/C]GAGAGGAAAGGAGAAGGAATGAGTGATACAAGGTTTATGGAAAATGGAAAGTACTATTAC
3411	CaTSNP6349	TAGCATGTATTGATCACAGAGGAACACCTGAAAACCCTGCAAGAACTTGCACTTTGGAGC[T/A]GAAAGAAGGGGCAATTTGCTGGGGAGCTGCGTATTGTGTTCGAGGAGGCCCTGAAAAGGA
3412	CaTSNP6350	TTTCTCTTCAACAACAAGGGCTTTGCAAACAGGACATTCTTGAGAATGTGAATGATGATG[C/T]AGCCATCTATAAAGACAAGGCCAACAGAATAAATGACCACAAAGTGTGATCACAGGATCC
3413	CaTSNP6351	TGGTGACTATATTAGACATTTTCTTCCAGTTTTAAAAGATATACCCAAGGAGTACATCTA[C/T]GAACCATGGACTGCTCCTCTAAGCATCCAAACCAAAGCTAATTGCATAATTGGAAAAGAC
3414	CaTSNP6352	TAGATTGACCGGCGTTGATGATCCTGAGTCCGTGCAGCGATCCGTTGGAGGCCTAAGTAC[G/C]ATAGGACAAAGACTTGAAAAGCTAGCAGAAGTACTTGGATTGCCATTTGAATTTCGAGCC
3415	CaTSNP6353	ACTTCGTCTCGCTGGTCATAAGTATTTGGATGTAAGGACGCCTGAAGAATTTAATGCTGG[G/A]CACGCTCCTGGTGCAATTAACATACCTTATATGTACAGAGTCGGATCAGGGATGACAAAG
3416	CaTSNP6354	ATTGATTGCGGTGTATTTGGCTTTTCAGCATTTCTCAAGAACAAGCTTGCAGAAATCATT[T/G]GACCAAGGTTCAATTGTTGCCACCTTAGGGGTCATCTGTATCACTGTTTCATCTTTGTTG

3417	CaTSNP6355	GCAGAGGAAGGCGGAGTCGGGACGGCAGTAGAGTGTGGCGGTGGCGGATTTGCAAGAGTC[A/G]CATAATTTGGAAGCCATTGGAGAGAAACTTTCTTTTTTTGTTTGAAATTGAATTGGAAGA
3418	CaTSNP6356	TTTTATCAGCATTCATAGGTCCTGATCAGGATGCAACAGAGACCTTATTGGCATCTGATC[C/T]GGACATTGCTCCATGGATACAAAAATACCAACGGAGCCGCGACACAGTCTCTCGAACAGA
3419	CaTSNP6357	CTTAGTTCTAACTTCCCTATTTCCAACTTCACGACCCTTTGTATGGCTTCTGACTCTGTC[C/G]CTGAAAAGATGAAAGATCGTTTAGTCGTAGAAGACGAATACATCTTCAAAGAAGAGATCC
3420	CaTSNP6358	GAAACTAGTGAATCCGATTTGTCAAGCAATGAAAGTGATGATATTCACAGGAAGAAAAGC[T/C]ACAGAAGGCACCATAAGCATCACAGGCGATCACATAATGCAGAGGTGAGGTCATCTGATT
3421	CaTSNP6359	GTTTTCGATTGTTGATTTTGTAGAAGCATCTTGTTGTTCCTTCAATCGAATATCACCGTA[G/A]CATTTGGAGCAGAGATTCATGGTGGCGGGACTACCGAGGAATCCACAGTTGTTAGCACAG
3422	CaTSNP6360	TTCGCCCTAACACCGTTTACCGTTCCCACTCTCCTACTATCGTTCGCTCCTTCCACGCAG[A/C]TCCTCCTCCTTCTTTCCCTTACTTAACTCTTTCCAATTCACACTCTCAGGTCTCCGTTGT
3423	CaTSNP6361	ATTTTTAGCAGGTTGATTTTTGGAAGGAACAGCTTTTGGTGCAGGTTTCTCATCTTCGGA[G/T]GATTCCTCCTCAGAAGATTCCTCTGAAGAGCTAGAAACTGGCTTGGCTTTCTTCAAGTTC
3424	CaTSNP6362	TAACGGTTTTGATTGAGGTTGAAAGAAGAAGGTAGCCAACAGAGGAAGCTAACAGCTGAG[G/A]CTCAATGGCTTATATACTGATATTGCTGTGATTTCTGCCTTTGCATTGTAAACTTTTCAT
3425	CaTSNP6363	CGCATCGGCGCGTGGCGTGTTCGAGTTTCTGGTGAAGAGTGTTGCGGGGTCCACAGCAGA[G/A]GCGTTGTGTGCGTTGAAGAAAGGAGATGTGGTTGAACTCAGTCAGGTTATGGGGAATGGT
3426	CaTSNP6364	TTCAATTGCGTCACTTATATTTGAAAGGGACATGAGCGTGTGGGTCATAGGCTGGGACTC[A/G]AGGCTTCTTGCTTGTGTTTATTCTGGTGTGATATGCTCTGGAATGGCATATTATGTACAA
3427	CaTSNP6365	AAGGTATCAAGCGACTGGCCGTGGGTTTGTAGTCCGCCATATCAAATTTTCAGAAAATTA[C/T]AGACTCTACTCTCGCAGTCATTTTGTTAAAGGGTTGGAAGTTGTGCTTCTGTTGATAGTA
3428	CaTSNP6366	ATTATAGATCACGGTCGCACGCAGCTGAACTAACGGCTGGCTACTATAATACCCGACACA[T/A]CGATGGATATCTTCCAATAGCCAAAATGTTTGCAAAGCATGGGGTTGTCTTCAATTTCAC
3429	CaTSNP6367	AGAGAAATCTTGGGTACCATTCAAAATCTGAAATTTTTCTCTCTATCTTTCTGGGCACTA[T/C]ACAATCTAATCTACCCATTTTTTCCCTTCAAAATCCGACATTTTCCCGAGAAAATGTCCC
3430	CaTSNP6368	AATAAAACTAAAGCTATCTATATCTATATATGTATATGTATATGTTATGTGGGTTATTGA[G/A]CACTTAAGCAATAGAATCATGACCATGTCCCATCATCGCATGATGAACAACCCTCTTGTG
3431	CaTSNP6369	GACCGGGCCTGTGTCTGGACCCGGACCCGGTCCGAGAAACAGTGCTGCATCTGCTAGAGA[T/C]GAGATTTCGGCAGATGAAAGCGCGCGCAGGAGGACCGCGACTTTTTGGACAGCGTGTCCT
3432	CaTSNP6370	AAAGGGAGACACAATAAAGGTACAAACAAGTAACTTATTGTGTTTTACATCATAACATGC[C/T]TTGGTTCAGTACTAACATCAGTAATAAAACACGTCACACAATAAGAGATATTGTCATAGT
3433	CaTSNP6371	TAGTCCATTGTTTGAGTTTAGGAGCTGTGCATTGATTTGTGTTGCTACTTGTGTTGTTTG[A/G]AGGAGACGGATCAGTGTGGTGAATCCTCCTGCTTTTTTAAGGATTCTGATTATGTCTGTT
3434	CaTSNP6372	GCAAAAATACAGTACGGAATTAGCTACTTCAGTAATGAAAAGAGCTGAAGCTATTTGCAC[T/A]AACTTCGACGCTACCAATATAAAAATTGAGAAAGTAGTTGGGACTGGAGATGCTAAGAAT
3435	CaTSNP6373	AACAACTCAAGAAAAAGAAGTGATTTTCTTACATGGTTTATATTATGCAGTAATTTACAT[C/T]AATGTGGATAAAGAGTATGGCTTCTTTTCTATCTGAAGTATTTGACTTGAGTATTGTCAA
3436	CaTSNP6374	GGGAAAAAAAGAGCAATGGAGGAGGAAGTAAAGTTGGTGATAATTTGAATTTGAAGCAGAA[A/G]TTATTGAATTTAGGGAGAGAAGTGTTTATGCAGAGATCGCATTGGAAGAAATTGGGAGAA
3437	CaTSNP6375	CCAACTAGTAAATCATGGTGTTAATTCTTCTTTGGTGGAGAAAGTAAAGTTAGAGATTCA[A/G]GATCTTTTCAACCTTCCAATGTCAGAAAAGAAAAAGTTTTGGCAAAGTAGCCAACATATG
3438	CaTSNP6376	ACTCATCGTAGTTTCGATATTTAATTACCCTAATTTCATTTCATTTTAATTTTATTTCAC[A/T]ATTTTTGGATTGAATTAGGGAATATATCAGAATGTTGGAAGCAATGGAAAGTTCAGTGAA
3439	CaTSNP6377	ACCAACTCGCTTGTTGCAGTTGCTACAACGTTTCGGACCATCCTTTGGCTTGACATCACC[A/G]CTGCCCCCTGAAACTGAGCCAAATAAAGGTTGCTGGGAGATAGTTTTAGGCTCCACTAAA
3440	CaTSNP6378	TGGTTTTCATGTTTTGTTACAAATTTTATGTTATTCGTCAGTTTTATTTTGCTGAAGACA[G/A]TTGTTGAAATTTTGTTTTAAATTAATTATGACAAGCCAAATCAAAATAAAATTGCAAAAC
3441	CaTSNP6379	AAACGCCGGCGATCGGAGGAGGGTGTGAATATTCCGGCGGCGATTAATCTTGATCTCCGG[T/C]TGACGCCGATTTTCCTGCAGAAAGCGTCGGAGAATCGTCGGCCTGGATCGCCGTCGATGA
3442	CaTSNP6380	AGGTCGTCTAAGTGGACGTATTGCCCTTGCTTTATGATTGCATAATGGGCCTCATTTCCT[G/C]TGATAAGTGAAAGAGCAGTGATTAGACTTGGTGGCATTGTGGGCATAAGAAAGGGGCCAA
3443	CaTSNP6381	GAAGAATCAATTATACAATACTTAAAGTGCTCAAAACTTGGACATTCATTGCACTTACTT[T/A]ACAAAATTAATTAACAATAAGAAATTCACATAGTTTTAATTCACTTTCCAGGAACAAAGT
3444	CaTSNP6382	TGCAAGCTCCTCCTGTGCAAATTCTAAAGCTTCTTCTACTTTTCCATTCCTGATTAGTTC[T/G]ATCAACCGTTGCTGTTGAAGATGGAAAAATAATTGGGGGTTCGTGTCTAGTATCTCAGGA
3445	CaTSNP6383	ATTTCGTCTTTTCTGTTGAGGGATAGATGGAGAGAAGAAGAGGAAGAAGAAGAAGAAGAG[A/G]TTGCATGAGAAATGGTTTGTTTGAGTTGGTGAAAGGAACATGGATCGGGAACTTCGATTT
3446	CaTSNP6384	TGGCTCTTATCCTGCTGTGCCAGGTCTACAACATCCAATGGCATATCCTGCTGCTGGAAT[C/G]ATTAGTGCAAGGCCTATGAATAGTCCTCCTGGTTCTGTTTCACCTGGAAATAGCAACTCC

3447	CaTSNP6385	ATACCCTACCGGCGCACGCGGCGAACGAATTTGGCGTCTGATCCGATAAGCCATAGTGTA[A/C]GGCAGCGATTCAGCGGAGGTATGAGGAGATCTGGACGGAGAACAACTTGTACTGGACGAC
3448	CaTSNP6386	TTCATTCAAAGCTACACCAATTATATATTCAACTGTTTACAGAAAAGACTTCTTTCATTC[A/T]TAGCTTCTGTTCAATTCAATTCAATCTTTGAAGATGGCCAATAGAAATGTTGCTGCTAAT
3449	CaTSNP6387	TGTTGATAGGGATGAGTTTGGTTTGCTTTTGTTTAGTGGTGGTGAGACCAGGAAAGACGC[T/C]GGACCGCGTAGTGAGGCGCAGAGTTATTGGGCTGTTGCTGATTTCAAAGGATGGTTTGGT
3450	CaTSNP6388	AAAGAAGCATAGTGATTTCCATACAAAAAGACCATCATACTCATTGATTAAGAATGAAAG[A/G]ACTTCATCATTCAATTATAAAAGCACTCTTAATGTTCCATTTTATGAATTGCCAGGGGCT
3451	CaTSNP6389	TTTGTGATCATAGAATTTTAGGGTTTGTTGAAATTGGTATCAGTTTCTATTTAAAGAGCA[A/T]GTATTACTCTGTTCATGTTAAAACCGTGATCAAATTTGTTGTTCCTGTTTTAATTTTGCT
3452	CaTSNP6390	AAATCTTGCAGTTTTCACAATGTCTGTCCACTCAGGCAGCTCCATCTTGCCAGATCGCTT[A/G]AGGTGAGCGGAGTAAGCCTTAACGAATTCATGAGGAGAAACATCCTTCACTGTCCTCGAA
3453	CaTSNP6391	TGGTGCTACCAAGGTTTCAAAACTTAAAAAGACAACTACGAAACAAGGTGGAACACCAAT[C/T]GACGTTCGAACTGAGATACGAAGACAATCAACATGGGAACTCGAAGTCTTCAAAGAACGC
3454	CaTSNP6392	AACAAGGTTTAACAATAACAATTGTACCAAAATATCTCATTTTGTAGACCATGCATGAGA[T/C]AACATAGCTATCAAAAAAACTTGGAAAGCAAAACAACCTTAAAGCACAAACACGACAACT
3455	CaTSNP6393	TTTATCATACTCGACATGTTCAATCTTCGAACGATAAATGTCGATATTGAAAGAGAACAA[G/A]CATATATTCAAACAGGTGCTACGCTGGGTGAAGTTTATTATAGAATTTATGAAAAGAGTA
3456	CaTSNP6394	TCCCTTTGATCGACACGGCATGCTGAAAACAATATCTTCAGCTATTCCGTATGGATTTCC[A/G]CTGGTATACACACCGGTAGAAAACCAATCACCCTTGGGCGTAGGAGTGATTAAAGATCGT
3457	CaTSNP6395	CTGGCTTATATGCTGTTCCTGTTAATTCTTTTGATAGACATGTTACTGGACTTCCATACA[A/C]CACTCTGATTCCGTTGACTTACCGCACACCCACCAGGCCAAGTTCTGAAGCTGCTGCTGG
3458	CaTSNP6396	GTTGTTCAGTGCCATCGTTGGACCACTTGGTCCAAGCTTGCTGGTTGGCTCCACCTTCTA[T/C]GTCCTCCCCCTCTACTGCAGAAACAGGAACATCAGCTACAAGTTGAGCAACTGCACCAGC
3459	CaTSNP6397	GAAGGCTGAGGTTCCACCAAAAAGAAAGAGGTCAGATAAAGATGATTCAGATGATGACGA[C/T]GGTGGAGAAGATGATGAAAGGCCCTCTAAGAAATAGAGTTGGGTCCATTGGTTTGCTTGA
3460	CaTSNP6398	GAGTGTACCGAGTTGACTCACCGCACGCGCCACACAAGTTGAACACTGATCATTGTTCAA[A/G]TCACCACGGCACTGAAAAAGACCGTATACGGTTTCCGACGCGGCGGTTCCGGGAACGGTG
3461	CaTSNP6399	TTGGAAGGTCCTGAATATGTAAGAAAGGTATCAGAAAATTGTATTGCTCATATGAAAGCT[G/C]AAGGGACATATGGTGATGCTGAAGAAAAAGCCATTCAAGAATTTAGAGAAGCTTACAAGG
3462	CaTSNP6400	CGTCGTAGAGGTTATGTCGACGGAGGGTCATTTGCCGCAATTGAGTTCACCGGATGTTGT[T/C]ATTCCGGTGATACTCAAACATATTAGACATGATATTGCGGCTTGATGTTAATGTGTGGTG
3463	CaTSNP6401	CTTCTCTTCCTCCTCAAGTTTACTCTGAATATTTACCCTCTTCTCAACATACTGTTCTCG[A/G]GTGACGAGACCAACAGTTTCTTTCTTAAAAGCAGTCTCAAGAATCTCGGAAGTGCTGGAG
3464	CaTSNP6402	ATATTTCTTCTGGGTAACAGTACATTTGAATCAGCTACAAACTGTCCATTTTGTTGTCTT[C/T]GGTGCTCATTTCCGAATCTTGACCCAGATAAACCCATAGATGATTCCATCAACCTCTTCA
3465	CaTSNP6403	ATTCAATCTTGAAAAAGGGGGCATCTATTTGGCTCTGTCTTGAATTACATCAAATCCCAT[T/C]TCAGTTGGATCGGTTGCTTGAAGTTCATATGATATTCCATCCAAAGCTCTTCTCAACCCA
3466	CaTSNP6404	AACTAAGATGAAAACCACCATCTTCATTTTTTTCTCATTCAACCCTAATTTCATTCCATT[C/T]CATTATCCGATGCTATTTACGAATCGATCCTGCTAATTCCAATTCTCTTCACCGAACCAT
3467	CaTSNP6405	TTTTCACATAAAATTCCAACATGGTGAACATTAACAACCCCATAATCACTTTCTTCATTG[G/A]ATGAGAACGATTCATTATGAATTACACCCCCTCCAACAGCAGTATGAGCTTTTATCGTCA
3468	CaTSNP6406	TAAGCCAGATGATGCTACCCTTGTTTGGGATAGGTTACCTATGACTATTGCTTTTACGTC[A/G]ATCATTGCAATATTCATCATTGAGAGGATTGATGAGAGGAAAGGAATGATCTCTATTGTA
3469	CaTSNP6407	GAACTTATCAGTGATCGAAGTCTTCCAGAATCTACTATCTTCCAGGAACCTAGGCCTGGC[G/C]AGGTCGAGCAAATACAACTTAATCACGACGGTCACGAGTCTTATGACACTATTATACATC
3470	CaTSNP6408	ATGTTAATTATGGTTTATATATGTACAAAAAACTCGAAGACATGCGAGTTTATTTAAATC[G/A]AGGTCTTCTGTAGCTAAGAAGGGTGTATGTTTAATAAAGTATTATGTAGTTTGAGTACCA
3471	CaTSNP6409	TGTCTGCAACTGAGAGCTCAAGTCCTCTATTTTCGCTTCCCCAAATATGACGTAAGTCTC[A/G]GAATTTGGACTCTTGAAAACATCAGGTTTTGAGATAAAGAAAAGAATATTTTTGGTTCTC
3472	CaTSNP6410	GGGTGCTGGTCAATTTTCCGGTGAAGTTTATGCGTTAGACACTGAAACATTATCATGGAC[G/A]AGGTTGGACGATAAAGTAGAATCAGGTGGCCATCCAGGGCCCCGTGGGTGGTGCGCATTC
3473	CaTSNP6411	TCCCACCGAGTCAACTCGGAGTATCTTACTCCAATCAGGCACGTTCACCGGCGCTGACGT[G/A]GCCATATTATGCTCCGCCCTCTGTGACGCACGATGGACGATCCTCGTTGAATTATCGAAC
3474	CaTSNP6412	AATTGGGTCTCCCTTTGGTTCCTTCTTCTGAGTGATCATGGGTATGCCCTGTCGTTGTTC[T/C]CATACTTCCCACATCACTGAAATCATCATCACTAGCTTCTTTCGCCATAAGTCTTAGATC
3475	CaTSNP6413	TTTCCCTGTTAAGATTCTTTCTCTTCCCTTTTCTCCTTCTTATCTCTTCCTTCTCAATTC[C/T]ACCACAACCACTCCTTCTTATCATCAATCAGGTAGGAGATCTTTCATCTGAAAACGTAGA
3476	CaTSNP6414	TTTACAGGTGTTGATGGATCAGAGGAAGAGGAAGAGGATGATATCGAATCGAGAATCGGC[T/C]AGACGGTCTCGGATGAGGAAACAGAAGCACTTGGATGATCTTGTGTCTCAAGTGGCTAAG

3477	CaTSNP6415	ATGATTAGGAATAGATACATTCTCCTTCTCAGTTGGTGGAGGTGGGAGTTTTGAGGAACC[A/G]GGTTCTTCCCAAGGACGGAGTTCTCTTGCAAAACCAACACCTTGAACCCTTGCTGCTTCA
3478	CaTSNP6416	CGTGAAGATCTTCCGAGTTCCGACCACCGTCTACGGTCGATTGTCCAGCCTCAGCCCTCA[T/C]CGTCAAAGATCTCATCTCGCGGCTTCTTTCAAATCGTATCTCGCTGGAAAGCAATGGCAT
3479	CaTSNP6417	TTCCTTTACATCACTGTGTTGACCATTATTGGTTATAGTAGACAAACTGATACCACTATT[G/A]CAGGTAACACTGAATGTGATGGTGTTGGAGTTTTGGGTATTGCTTGGGCTTTTGGTGGTA
3480	CaTSNP6418	AAAAACACTATCCATTTGTTTCTGAACATTCTTGGGCCTCTTCCTGGGCCTACGATTGGG[C/A]CGAGAACCAGTCATGACGAAGATGTCTTCTTCGATCTCTTCCTTCGAAAGTGCGATCCAG
3481	CaTSNP6419	CGCGCTTGCAATCTCATACAATTGTTTATCCGGGAAATAACAAATTGTTCCAAAGCTTAT[G/A]TAGATCACCGACTTATCTCGCTTTGAATTGAGCCAAGCCAAACACTTTTCCACACTCACC
3482	CaTSNP6420	TAGCAATGGAAGATGGGCAATGTTTGGTTTTGCTGTTGGGTTGTTAACCGAGTTTGCAAC[C/T]GGCTCAGACTTTGTCGATCAAATGAAGATCCTTCTCTCGAATTTTGGGATACTAGATTTG
3483	CaTSNP6421	CGGAAAGGGACACTATATATCGCGAATCTTGGAGACTCTCGCGCTGTGATCGGTTCAATA[G/T]GTGGATCTAACAAGATCATTGCTGAACAGTTGACAAGAGAGCACAATGCCAGCAAAGAAG
3484	CaTSNP6422	CATACCATATAGATTTAAGATTGGGGAGAAAAATACCTTGGATATTTCAGATGAATTGGA[G/A]GTTCTTGTTATAAGTTCTCAAAAAGGCAAAGGGATGCTATTTCCAAAGGGAGGTTGGGAA
3485	CaTSNP6423	AACTGTCAACAGGCTAACATTGTGTTCAGCACAGAGAGCTTTCACCAGTTTTACATAATC[C/A]GGTTGGTCGCAATCTTCAGCTAGAACACAGAGCTGGGCAGCATGCTTCTCAATCACTTTT
3486	CaTSNP6424	TGCTTCAACTAGCTGTGACTTTGGAGGGGTTGCTACAATTGTTACCACTAACCCTAGTTC[C/T]GGTACTTGCATCTATCCACCATCTTCAGGAGGAGCAGGCACATCTGGCTCTGGCACTACA
3487	CaTSNP6425	CCTCTTAATTGCAATATACATTGGGATATTGACTCCTTTCAAACTGGCCAAAGCAAATGC[A/G]ACATTTGCATTATAGAAAAATGAAACCGGGAGTAGTTGCTTGGCCGTTGCCATATCCACT
3488	CaTSNP6426	CCGCTCCAAAAATAGCTCCATCTCCACAATTTGCAGCTCCTTCCACTTATCCTCTAAATC[G/A]ACCCTCTTAGAATTCTCAATCAACTCCAATCCCTTTTTCGCCACATTCTCATTCAAACCA
3489	CaTSNP6427	AATCTAACCTCTACAACCTCCCATTCAAATTCCCCTTCTTCCTCTGTTACTCTTCCATCA[C/A]AGAAAAATCACCTCCTTTTTCCACAGTACACATAGATCAAAGCTGCTGCTGCAACAACAG
3490	CaTSNP6429	TAAAGAAGCAAAGTAATATCAAACCTTGGAAATAGTTAGTGCCGGGCTCCACTGTTCCTT[A/G]AGAATGTCGAGACATATACTGCCATTACTGTTGATGTTTGGATGGAATACCTTAGTCCTA
3491	CaTSNP6430	TTTAGGCATTCATTAAGAAATGCTTAGATTCACTTTGATGTATCATATTAGGAAAACCTG[G/A]TTGTGAGCAAACAGTATCAGGCCTTGATGGGTGTTTGATATTATTGCCAAAGCTTGTTAT
3492	CaTSNP6431	CCTGTCTGTGTGCTTCTGCTAATTCGTCCTGCAACTCAGCTAGATCCTTTTTCATGGTGC[C/T]GATCGACTTGTATTCACGAGCTAGAGGATTAAGAACTTCAACCACCTTTTCATGGAGGAG
3493	CaTSNP6432	CATGCTTCTGATCTTGGTTCATCAGAAATATTCACCATTTTGAGGTATTTTCTTTCTCCG[C/G]CAAATGAGGCTTATAACACTATGGTATCTGTAAAGAAGGAGTGGGAAAACCAAGCAGTGT
3494	CaTSNP6433	ACCAACCACCGTCTCCGCCACCGCATACTCAAGACCAGCCTCATAATCCTTCACCAAATC[A/C]TCCGCCACATACCGCGCCTTAATCCACTCATTAGCACCACCATCTTTCCACATAACCAAA
3495	CaTSNP6434	TGATGCACCTGATTATTTAATTTCAAATCTTTACTCTAATGTGCACAAAGAATTAAAAGG[G/A]TTGCTTTGGGATGATGGGTGTGCACAAGATAGTTTGAATTTCAATTCAATTGATGATGTT
3496	CaTSNP6435	AAATGGCTATCTTTTGCTGGAATTCTTTTCCTCTGAAGTGAATGGAGCAGTGCTCACCAC[T/C]ATTGTGTCCACAATAACAGCTGCATATGTTGCATTCATAATTTACCTTATTTCAAGTGCC
3497	CaTSNP6436	CCAAATGGAAGAAATGAACTTCCTGAGGATCTATTCACTGTAAAATATTCATCCTTCCAT[T/G]CTCCAGTCCCAAGCTGGCAAATGGGTGCACCACCTGGCATGGGCATCTCAGTTCAATATA
3498	CaTSNP6437	TTCTGCTGCTCCGGCGGTATCATCGACGGCCTCACGCGCTACGTTGAGCCCGATTTTTGT[T/A]AGCGTGTGTTCGCACTGTGCTAATGCTTGTGGATGTGAGATCACGCGCGTTAGGTATTCT
3499	CaTSNP6438	TAGCAAGACAGCAGTGTTTGGAGCGAACATCAGGTTTTACGATTCGTTCCGGAATGGCGA[C/T]CTTGCAACCATGCAATCCATGTGGTCAAAAAGAGACGAGGTATGCTGTGTACATCCCGGT
3500	CaTSNP6439	AGGAACAGAATGAACATCTTGCTTCAAATTCTTGCAATTCCCAAAAGATCCAGGTTGAGA[A/G]ACACCTTCAGGAGCTTCTCTTGAACCCGGTTCAATATTATTATTCCCATTCCCACCAATA
3501	CaTSNP6440	GTCAATTCTTGTCTCATAAAAATCTTCCATTGAAGATCTCTTTCCAGGGGAGCTAGCATA[A/C]CCATAGCTGAATTTTCCATTCTGACTGAGCCCCCCACCACTAACAGGTGAATCATCAGCA
3502	CaTSNP6441	TTTGTTGCCGGAAAGGGAACTCCGATGAGGCCTGGTCCTATCGATGCATACTTGTTTAGC[C/T]TGGTAGATGAAGACGACAAAAGCATTCAGCCAGGTAACTTTGAACGTCATTGGGGATTGT
3503	CaTSNP6442	ATAGTAGTACAATCCACATAATGGGTGATAAAATCACAAAGACAACTCAACGTCAAAGGA[G/C]CAAGGCAACAAATATGTCTGCAAAAACAAAACTCGGCTCAGGATAGTAGTATCCCTTGAT
3504	CaTSNP6443	ACCATTAGTATTAAGTGTATGTCGTAGATGAAAGGTTGGCTCAGATGCATTAGCAAGTAT[C/T]TGAGACAGCATTTGTTGATTGCTTTGAGAAATAACCTTCTCAGATCCATTTTCTTCTGCT
3505	CaTSNP6444	AGTGACCATCTGAACAGAATTGAGCAACTCACCTATATATACAGCATCTTGTTCGTCTAA[C/T]GAACCATGCTCACGGCTAATCCCTATCCAAGATTCTTCATCTTCGCCATCAAATGAAGAA
3506	CaTSNP6445	GAACATAGTCCACTCCTTCCTACATCTCTCCCTTACCCCTTGCCATGGTGCTTTCCCAGT[T/C]TCTGTTACACCCTGATTCCCTAGTGTGGGGACACCTTGGTGGACAATCCATTGAGCATCA

3507	CaTSNP6446	GGTGATTCTATGTAGAACATTGAATGAAAAACATTACCATGTCCTATAGACTTATTATCA[A/T]ACGTGGACCCGGTGGCTGGAAAGAATTCAAGTAGACTGTTTGGCAATTTTGCATCGACTT
3508	CaTSNP6447	GGATGGATGCCACATCATTTTCAGCAGCAGACCTAAAGAAATGCCTTGAGGGTCTAGCAC[G/A]CCATTTATTTGGTGATGTAGAAATGCGCTGGGTCGATACCTATTTCCCATTTACTAATCC
3509	CaTSNP6448	GTAGTTGAATTGAATTGATCACTTTTGCATGGTGGTAAACCGTTAGAATCATCACCGTTA[T/C]GGTCGCAAAGGTTGTTGCCGGCGACTGAACTTGCATTGATTGCAAAGAAAGCTTCAGTGG
3510	CaTSNP6449	TACTAAGATTGAGTGTGAGTTATGTGGTGCTCAGTTAGATTTTGCATTGCCCTCAGCTTC[T/C]TCTGTTGAAGCTGATGCTTCCAGTGAAGAACTTTCTAAACAGCTTGATAGCAAGCACAAA
3511	CaTSNP6450	ATAAATCTGTCAACATCCCTGCTCTTCCTGCAGCAGCATCTGGTCAAAGTTGTCTCTACA[G/A]TCATGACACGCGGATTTTCCTTGTTAATTTATTCATTCTCTTTATGGCACTCATGTAATT
3512	CaTSNP6451	TCTCGTTAACCTTGCATTCACCGAGGTTCCTGGCACATGGCGATGGATGCTTGGTGTTTC[A/G]GGTGTTCCTGCACTTATTCAGTTCATTCTCATGCTCTTCCTCCCTGAATCCCCAAGATGG
3513	CaTSNP6452	ATGTTTGGTCCTATTTCTTCCAAGCTTTCAACGCCTTTCGTACTGAGGACTCCTTTGATG[G/T]CTGCCATGCATGAGGATGATTCACCTGACTTGATAGAAGTATCAAGAATCCTCAATGACA
3514	CaTSNP6453	AGAAGAGGGGTTTATGTCACTGGTTTCGAGACTGTGGCCAATTTCACCGCAAATTGCAGC[T/A]GTTGGTTCATGCTGTCTTGTTGGTATTATTTGTAATGGAACCCTTTATATAGCAAATCTT
3515	CaTSNP6454	CAGAGGATGAGAGCACAGAAACCAGCTGCATGGTGAAGACAATCATATGACCCTTGAAGA[A/T]GACCCTTTGTTCACATGAAGTCTCGTATATAGAAGTCTTATTGTGGGTGAAAGCATAGGC
3516	CaTSNP6455	CATGTTGTTGTTCATTTGAAGATGGATTATACAATTGGTGATGAAGTTGAACTGCAATCA[A/G]ACCAAGACCAACTTCGGATCCTCCTACAGAAGAACCCTTCAAAACTGTCGGTGAACTCTT
3517	CaTSNP6456	TGCAATGAATGTTAGCCGCAGGGTTATTGTTATTCTTCTTCTTCTTCTTCTTCTTCTTCT[T/G]CTCTCTCATTTCATCACTTCTTCTTTCTCTTTTAACATATAATTTCAATGGCTGTGGCTA
3518	CaTSNP6457	CGCTAGCGTGGATCGATGCTCCTTCGCTTCTGATGCTCATGACCATGACTCTTTAACCTC[C/T]GAAATCTCACTGCATTTGGCAGGGAACGTTGATTTTGCTCCGAGTGAAAGCTGGAGCGGT
3519	CaTSNP6458	CTCCAAAAGCTACATGTATTTTGACTTCACTCAACGACAAGTCAAAACATGGACGAGTTT[C/T]CTTCATTTGTTTCTCGAACACGGAAAAACTTGTAATCTTACCTTTGAAATGTATAGGAAA
3520	CaTSNP6459	TTTTCCTTCTTAACAACAATAATTAGATCTCTCTCTTTGATTTCACTTCAGGGTCAAATT[C/T]AGTGAGGAATGCCACCAAAATGGGGAAGAATGCAAATTGGTTTTCCAATGTGAAGAAAGC
3521	CaTSNP6460	CCATACAGTAGTTGTTACAGATGATGGAAATTCCCTAGCATTTGGTTGGAACAAACACGG[C/T]CAGCTGGGTTCCGGTTCTGCAAAAACTGAAATCGAATCATCACCTGTTCGCTGTCTTGTG
3522	CaTSNP6461	CCTCAGCCAGCAGTGAACAATGAAATTATGCTTCCACATGAGTGGACATACTGATATGAA[T/A]TGATCCTTTGATTTGTTTGAAGGGAAGTAGTTTATAGTGAGAAGCAATAGGTTTTTTTGT
3523	CaTSNP6462	TTAAGAATGCAATCGAAGATGCAGGTTTCGAAGCTGAGATCTTACATGAACCCGGTTCAC[T/A]TGGACCGAAACCGGTCGGGGAAAGTGCGGTAGTTGGACAGTTTACAATTGGTGGCATGAC
3524	CaTSNP6464	CCCAGTTTGCCAAAGTGGATTTGTTGAAGAAATGAACAGTGCTGCTGGTATCAGCGAGAT[A/G]CAAGGTATCGGCGAGATGCAAAATATACCAGAAAATGATTTTGGTTCGGATCGTGCTGCC
3525	CaTSNP6465	CGGGAACATTTATAAACTCAAACCCGGTCCAGGGTCTCAAAGGCTTCAACAACTTCTAGC[C/A]GGACTAACGTCGAAGGCTAAATCGGGTGAGGATCAGTGTACATAAATCATGTTGGGAATT
3526	CaTSNP6466	TGCCAAATATGAGCCTTCTTTGTACCAGGCATATGAAGCTTATGGAATTCTCTCATCTTA[G/A]ATGCTATCAAAGCAGATATTTCGAGGTCACGGAGATCGGAAGCCGAGAGAGTTCTGGCAT
3527	CaTSNP6467	GACTCTAACCCTAACCTCAACCCTATCCTATCACCCTAAAGCCCCAACTCTCAACTTCAA[C/A]CCTCTTTCTATACGTACCAAAATCCCTACTCGAAACCCTATCCGAATTCGAGCGGTGCTG
3528	CaTSNP6468	AGATGATTCAACTATTTCAGGGATATTATCTAGCAACCGATTCATCTCATCTGGAAACTC[C/T]AACTTTGCTCTGTTAGTCTTCCCTCCAATAACCACTGTTGTCCCATCTTGTAAGGCCCAT
3529	CaTSNP6469	GCGATATTCGGCGATTTTAAGGAACATAGGCATGGCAAGGTCGAAATGTGGACGAGGAGG[G/A]TTACACCAACCACCATTGTCGTTAGGGAGAGCATAGTTAGGAGGACAAAAGTTAGTTGCG
3530	CaTSNP6470	TGCGAATCACTTTGACCATGAAGAAGCAGAGGAGGAGGAAGAACCCGAACAGTTATTCCT[G/A]AGATTACGCAAAGGAAAAGCTTGTGTAAGGCCTGAAGATGAAGAAACTAAACCTCGGTTT
3531	CaTSNP6471	GAAGTGTGTAAAGATTGCGCACATTCCTGTCAGCATCAGGTGATAAATATTTTCCATTGT[A/T]GCGATCAAGATCAAGTTGCAATGGAAACTCATAACGGTCATTAATCTTTACCATAGTATC
3532	CaTSNP6472	TTGGGTTATCAATCAAGAGTTTCTTGACAAAAACAAAATTGATTTTGTAGCTCACGATTC[G/T]CTTCCATATGCTGATACCAGTGGTGCTGCAAATGATGTTTATGAATTTGTTAAAGCTGTT
3533	CaTSNP6473	GAAGAATATCTGGTGAATGGGATGGATTTGGAGCAGACTTCAGTAATCAAGGGAGCACAA[C/T]TGAACTGCCAGAGTCGGTGGTTCCTGAAGCGTACCGAGAGTGGGAGGTCAAAGTATTTGA
3534	CaTSNP6474	AGGAGCTGTAAGAGAAGTGAAGGAAGAAACTGGGATTGATACAGAGTTTATGGAAGTCAT[T/A]GCATTCAGGCATGTACACAATGTGGCATTTGAGAAATCAGATTTGTTCTTCATCTGCATG
3535	CaTSNP6475	GGACTTGTAGCGACTATGGAGCATGTGAGTGAAGGTTAATGAGGTGGAGCGATTGCGTAG[G/A]AGAGGGCCTACGGTAAGGTGGAGGACTTGATCTAATGAAATAAGGGAGCTTAGTCCGTTT
3536	CaTSNP6476	TCAGTGGTGCAATATTAAAGCACTTCCTGGTGCCAATCGGTTGATCAAACATTTGAAGAG[A/C]AATGGTGTTCCAATGGCCTTGGCATCAAACTCTCCAAGGGAAGGCATTGATGCCAAAATA

3537	CaTSNP6477	GGGAACAAGTGCCAGAACATGCCTTCCATTTTTGCTTGCGGCCACTGTGTTAGGCTACAT[A/G]GTTGGTTCTGGGTTGCCGTCGAATGTGAAGAAGGTTTTCCATCCTATAATTTGCTGTGCA
3538	CaTSNP6478	CGCCGGTGCCGCACGAGCTGCAACTACTGGTTTCGGTGGTTTCGCTGGTTGCCGCCGACG[T/C]CGTTTACGGAGGCAGAGTAACAGAATTAGAAGTAGTAACGCTATGAAAAGAACGGAACCA
3539	CaTSNP6479	GTTTAGTGCACTTTCCAGTTGTTGAGGTGGCAACTGCAATAATACATGAAAAGAACGTGG[C/T]TGTGTCTGATATACACATTTTAAGAAACCAACCCAAAGTTTTGGCATTCGCCACACCTGT
3540	CaTSNP6480	GCTATGATCTCACCTGCAAATTCCAATGTTGAAACTTGTATTCTCAATGAGTTCTATTAT[T/C]TACAAACTGGTGATACTGCAAAACTTCCTCTGCTTTGCCATTACCCTGTTAAGGCACAAT
3541	CaTSNP6481	AAGATATTAACGCCGTTCGAGCCATGGAGATTAAAATGTACCCGGAAGTGTCTTCCGCTC[G/C]AAGGTCCAATACTTATTCTAGCTTCACAGTTTTGGTTCATCTCAAAGCTACAGCTGCAGT
3542	CaTSNP6482	ATTGTCCACCTCATCCACCTTACGCAGCTTCCTCATAGTTCCATGTGATGAGATATCAGC[A/C]GCCTCCTCATCGCCTATGTTATGAGGTCTAGATGGAACAGTATCTCCATACTCGTCATTT
3543	CaTSNP6483	TTTCAATTCATTTCTTGCATTGACATTGACACAAGTGGTTATGGACCAAACGAGTTGTCG[G/A]GCTACATTTGGCTGGGAATCATCTGAATCGTGCAAGTAAAGCCTTCCAAGAATGTCCCTT
3544	CaTSNP6484	CCAGGTGTTACTTTCTTGGCTTTCTACCTTGAATTCCTTGAAATTACCAGTCATAAACAA[C/T]GTATCGCCATTGACCTCATTCAATTTTTCCCAAACCAGATCAGGCTGATTTATGTAGCCT
3545	CaTSNP6485	AAATGAGGAGGAGAGGGTAAAGGGAAAGACAGTTGAAGTTGGAAGAGCACATTTTGAGAC[G/A]GAGACATCAAGGTTTACAATTTTGGATGCACCAGGTCACAAGAGTTATGTCCCTAACATG
3546	CaTSNP6486	CAAAACTTGGAGAGCTGAAATATTAGTTAGTGACATGGGAATAGGACCCATCAAGCTGTT[A/G]TTATTAAGCCGGAGGAAACGCAATTTGGACAACTTGCCCAATGAATCCGGGATAGGGCCA
3547	CaTSNP6487	CATTAAGAGAATGCACCAAAAGCCTAGACATGATTCCTGTATTGTCAGAAACCCTTGAGT[A/G]CACGAATATCCAATACTGAATAATTCAACGAAATTAAATTTAGAGAGAAAAAAGAGTAAA
3548	CaTSNP6488	ATCCAACCTCTTTGGAAATTCAATATCTCTTTGCAACTGCGATTTCAAATCCGTACCGCA[C/A]GAAAAGGCCCCATTCACCGAATAATCCAAGCTTCCCCCATTTCCAAATCCCCAACCCCCA
3549	CaTSNP6489	GGTCATCAGATGTCTTTAATTGATCAAAAGCGTCAGCTGACGATGACCGCAAGAATGTCG[C/T]ATCCTCGTCATTGTATGGAAGATTAACATTGCACTCTTGAAGCTGCTTAAAAAGAAGCCC
3550	CaTSNP6490	GAAACATCTTTGGCAGTTTAAAAGATGCCTAGTTATACAGCCATAGCAAGACCTATGCGA[A/G]CAAGGTGCAATCCGGGCATCAGCTTCACACGCGTAACATATGCAGCACAAGCTATCATCA
3551	CaTSNP6491	AATGGAGAAGGAGGCTATGGAGTGTTACGAAGAGGCTTTTGTAAAGGGAAAGAAGATGAG[C/T]GATATTGCTTATAATTCAGTGCTTGATGCTCTTTCCAAGAATGGAAAATTTGATGAGGCG
3552	CaTSNP6492	TTTGTTCATTGCAATTGCAGAATGCATTGAATCATGAACACGCTTAAACTCTCTCTCGGA[T/G]TTGCCGGAATCATTAGGTGCTGAATTTCCTTCTCCTGCCGATTTATTTTCCTTTGAATTA
3553	CaTSNP6493	TCTCTTCGAAGTGATTCCTCAAACGATTCATCACCTAAAGAGACTATAGAATTCCCATCA[G/A]CAGCATCTTCCTCCATCGATCGAAAAGAGACATACTCCGGTTCAAAAGGTTGCCTATTCT
3554	CaTSNP6494	AACATATACTACAATGTGTGGTATACTTGCCGTGCTTGGTTGTTCCGATCCTTCTCGAGC[T/C]AAGAGGGTTCGCGTTCTTGAGCTTTCTCGCAGATTGAAGCACCGTGGCCCTGACTGGAGT
3555	CaTSNP6495	ATTTCTATTACCACCCCCCCTCCCCATAATTCCTTCTCCGCTTCTCAGATTCTGAAAAAC[T/A]ACACTCATGGCTGCCAACAAATCAATCTCCCTCGAAGAAATCAAGAACGAAACCGTTGAT
3556	CaTSNP6496	ATCTTAGGACTGCAATGCGCATGAAGATATTCTAGGCCACGTGCAGTTCCCAGAGCCACT[T/C]GTTTTCTTGTAGGCCAATCCAAAACAGATTCCCCAGGTTTGAGTTCTCGTAGACGATAGG
3557	CaTSNP6497	GGCGTGGTACATGAACCCAGCCTTCTCAGTTCTGATAGGAGGAATACTCCCATTTGGAGC[G/T]GTTTTCATTGAGCTTTTCTTCATCCTCACCTCAATCTGGCTGAATCAGTTTTACTACATC
3558	CaTSNP6498	AGAAAAGAACTTAAGTCGGTGCTCTGGGTAATCTTCAAAGTTTTTTGTAATCATCTCTAG[A/T]GTGCACTGAAAAACAGCTTCAAATATGCGAGGAACGTCTTCAATCATTGCAGCTTTATAT
3559	CaTSNP6499	CCTGTACTTCCCCTTAACATTGAGGCACCTAATAATGAAGATGCTTTGAAGCTTGGACTT[A/G]CTGTGCTTGCAGCTGTAAAGGAGTTTGCAGGTTTAGACACATCAGCGTTGAACAAATTTG
3560	CaTSNP6500	AACTAGGGTAACAGCCTTAAGAATAGTTGAATTCCCCTTCTCACTCTGTGAATCTTCAAC[A/G]GGAACAGAATCCTTATCTTCATTATCATCACCACCGCGATCTTTTCCCTCATCGTCATTC
3561	CaTSNP6501	CACGCTCAGGGCGAGGTCGTTCCAGTAAAGTTTTCCATGGCATTGTGGAACCAATATTTA[A/G]GCTTGCTGGGTTTAGATTGGAGGTAGTCAAAACAACATCTGCTGGTCATGCCAGAAGTCT
3562	CaTSNP6502	TGGATTTTCTCGGGTAGCGGCGTGGTGCTCTGAAGAAAACAAAGCTAGATGTGGAATATC[C/A]TTATATCACACTTTTTACACGTGATCATGGCCATGGTAGCGATAATTTTAGTTTTATGAT
3563	CaTSNP6503	GATCATCCAAGAGAGCTAGCTAGCTAGCTAAAATGTTATTGAATCATCAGTCTTATTATG[G/C]ACTGATCCAATCCCAGTGTATGTGTATTTGAACTTTGAATGGGATTGGGTATTGATTTGT
3564	CaTSNP6504	GCCCCAGAATACGGGCTTTTTACCCAAACCTCAACTTCAGACAGGCTCCTAATTCCAACT[G/C]CATCCGCAAGATTTCTGGACAATGGTCTTCAAATGATTGAGTTCCTTGGAAGAGTAGTTG
3565	CaTSNP6505	TGCTATATTCTGGACAGACTATGTTGCAACAAGGTGGTATAGGGCTCCTGAGTTGTGTGG[A/T]TCCTTTTTCTCCAAGTATACACCGGCTATAGACATATGGAGCATTGGCTGCATTTTCGCA
3566	CaTSNP6506	TTTGCTAGCTCGATGCCATTTTCCTTCAGAATCAATATAGGTTAAAGCAGTCCTCTTGTC[A/T]ACAGGATTGAATGGAAGAAAATGAACCTCCCTAATACCGGCTCTTGCCTCTTTTGGATCA

3567	CaTSNP6508	GTCCTCAACGTTACTGTTGATGTTTCGAACAAACAAAGTCCTGGATGGATGCTCACCGTA[T/C]GGATGCTCCCCGGCAACTGTTCCCACACCATTTGCAATTGCATAAGATCCAATACCATTT
3568	CaTSNP6509	TCTAACAAAGGACCAGTTTAGAATAGTAACAGGGCCAGTAAGCATTCCTTTCATAGGCCG[T/C]TTGGTCAAACTTTGAGCTGTTGAAGACCAGAAAACAGTCATTGGCTTGGGACGGCTCACA
3569	CaTSNP6510	ATTTAGATAATGACCCACCCTGCCCTCTAATTGCAGCATACTTGTTGCTGATGGCTTCTC[T/G]GCTCGAGCCATATCTGATTGTCTCTTCGTCCAAAGTTTTCGATAGACCTCTGTTCGCTTC
3570	CaTSNP6511	AAGAGTTGGGTACTTCAGTTTGTTTAGCTTCCACCAACCCAATACGTCAAACTCTGGTAC[A/T]CGCGGCAACAGTGACTCTTCCAGGTACTGGTCCAATTCAGATTTTGTCTGTTGGCTACTA
3571	CaTSNP6512	ATCGATGATGATGAAGTTTCTTTGCAGCTTTGGCGGTAGAATATTGCCGCGACCAAGTGA[T/C]GGAAAACTAAGGTATGTTGGAGGCCAAACGCATATTCTCCGTATAAGAACGGACTTATCT
3572	CaTSNP6513	AATGAAAGGCTGTTCTAGAAGAGCAAGCTCCCCTAGTCTCTTCGAGATCCTTCCCTCGAC[A/G]ATTTTTTCTCTTATGTTCTCGGGTTTCGAAGCAAGGTCTTCTCTTTGCATTTCGAGTTCT
3573	CaTSNP6514	GGAGATCGAATTCGTGAAGAAATTGCAGCCGAAATTTTCGGAGTTTCGGCGCGCGTATTC[G/T]TCGCCGGATTTCAGCAAGAAGGTTCTGGAAAAGTGGAGGCCTAGGGCGAAGATTCGAATT
3574	CaTSNP6515	GTATGACCAAGTAGCTGTCTACGGTTTCAGCGATAAAGTGGGCCTTCTCTCTTTCCCTCA[T/A]AGAGAGGACTCATATGGGATGGCCAAACCATACAGCAGCAAAACTGGTGCAATCATCGAT
3575	CaTSNP6516	CAATGTCAGGTGCAAAAGGGATGGTTTATATGGAGTACAGTACTGGGCGTAAAGGCCAAG[C/T]ACAGATCAAAAGCAAGCCGAAAGATCCAGCGAAGAGACACTTCTTATTCAAAAGACGATA
3576	CaTSNP6517	TAGTGGAGAGTTACCTAATGGAAAAGTCAGGAATACTAGTTTCAATAGACGAATGACTGC[T/A]CCATCATCTGAAAGTACATCAATTCAAACGGGCAGATCAGCTCCCTCTGTTACCCATGGG
3577	CaTSNP6518	CTTCAACACCTTCTTCTCTTCATTAAAAGCACGTGCATCTTCATAAGCTTGCCTTTTCTC[C/A]GCAGCATGGACGACTTGAAGGAAATCAATAACCAGTTTTCCATCCTTAGATGCATAACCA
3578	CaTSNP6519	GATTGGAACTGCTGGCCTATACTTAGCAACTAACTTGGCTGTACTCCCACCGCGCGTCAG[T/C]ACGACGATGAGTTTTGCTCTAGCCTTGTAGGCTGTGCGTACAGCAGAGGATGCAAGGCTT
3579	CaTSNP6520	CCAAACATCTGGGTTATTGGCTGTGGCAAATAAAAGAAACATCTGATATAACGTAGCACT[A/G]TAGGAAGTGAACACTGTTTTCCCCTGTATTGTATCTTCAAAAATCACATAAGCCACCCAA
3580	CaTSNP6521	ATTCAGTGAAGACTAGCTTAGACATCAGTCCTGAAGCAACAGTTGAAGAGGAAGATGACA[C/T]GGAAGTTGAGGTTGAAAGTGAAACAAAAGAAGAAACATCTACTTCAAATCCTGAAGCTGA
3581	CaTSNP6522	GACTGAGAGTGGTGGTACTAAGGATGACCTCAAGCTTCCATCTGATGAAAGTCTGCTTTC[A/G]CAGATCAAGGATGGATTTGGTGAGGGTAAAGATCTGATTGTGAGTGTTATGTCTGCCATG
3582	CaTSNP6523	GTTAGGTTCTTCATCAGGACCTGCTGCATTAAGTTTATCATCGGAAGGACTTGTTGAGTC[G/A]GGCTCAGCAAAGCCAAGGAGGCTTTCATTATCAGAGACTAGACAGATTCCTTCTGGTGAT
3583	CaTSNP6524	TATCGGATCTGGTTCACCACTGAATCGACCAGCAAGAATAATCGAGATTGTTGTTGGCAC[G/A]TAGGTATAAGACCCTCCAATAACTGCAGGTAGTCGACTTCCGAAGAGTGTTTGCAGCAAC
3584	CaTSNP6525	TGTGGCTTGAACTTGTTTGGCTTATTGATTGGTGGGCTGGAGTTAAGATCCAAATATTCA[C/T]GGATCGCGAAACTTTTCGTTTAATGGGTAAAGAACATGCACTTGTCATATCCAATCACAG
3585	CaTSNP6526	TCCTCAGCTGGTTCAGATGTTCATAGGAGATGGAGCAAAACTTGTTCGTGATGCATTTCA[A/G]CTTGCAAAAGAGAAGTCGCCATGCATTATATTTATAGATGAAATTGATGCAATCGGTACA
3586	CaTSNP6527	GATGAACATTTTGCGTACATTCACAGTCAGCCCATGAACTGCCTGGTTCCAGTGACCTTC[A/G]ATATTTCTCTCAAACGCTTCAAATATTATTGGTAATACCACAGTCCTGTTTTGAGCAATT
3587	CaTSNP6528	AAAGACCGGGACTGGCAAGACGTTAGCCTTTGGAATTCCAATTCTCAAGGGTCTCACCGA[G/A]GATCAACAGACCAATCTCAGGCGGTCTGGTAGGCTTCCTAAAGTACTGGTCCTTGCACCT
3588	CaTSNP6529	AACTTCTCTCCTGACCTCCTCATCGGCTACATTATGATCTGTTGACAAGCGCACAGCCTC[C/A]ACCGGACAATTCTTGCTCTCAGAATACTTCCTGCCAAGAACAACTCTGGAGTCTCCGAGG
3589	CaTSNP6530	TGATGATGAGCTGTCAGCTGGGCAACTGCGCAACTTGGAAAAGATTTTTGGTGGAGATGT[T/G]AGAGTTTGCGATCGAACTGCTCTCATCCTTGATATATTTAATCAGCGAGCAGCAACACAC
3590	CaTSNP6531	CAGAACCCTGGTCCAATATCCTCTGTAGCTTAGCAGTCCAAGGTCAGAAAGGGGTCTTTC[T/A]GGTGTGCCAACTTTTCCCTCCTTCTTTGAAAGTTCATAGGAGAAAGCAATCAGAAATTTT
3591	CaTSNP6532	AGCATTGGCTGATTCTCTTGACCATGCTGTGGGTGATGATCCATACTTCAATAAGTTGAT[T/C]CGCATTCTGGCAACCAGATGCATGAGTCAGGCAGTTTATTTCTGTAGTGGGGACCTTAGC
3592	CaTSNP6533	TAGTTCAATTTGAGGGAGGAGGATGGTGCAACAATATCACAACTTGCCTTGGTCGCAAGA[T/C]GAATCGTTTAGGTTCATCAAAGCAAATGGCTAAGCAAATTGCCTTTTCAGGAATTTTAAA
3593	CaTSNP6534	TTTGCAATCAGAAACTTTGCACCAGCAATATGAATTGGTCAAAGAAAGGACTAAGAACGG[A/C]AATACACTCTATGGTTCCCACGTGATGCAATATGGTGACATAGGGCTCAGCGAGAATAGT
3594	CaTSNP6535	GTTGGTCTTCAATCCACTTGACACAAAATAGAGTGGGAGCAATAGACCAGACACAAGATC[T/C]TCTATTTTCTCTACAAGAGCACCTGCAAATGGTCCGTCTTTTGGAACCAAAATTCCGAAA
3595	CaTSNP6536	GAATCATATCCACCAATGAGATCAAATGCCAAGTTTAGTCTTGATCCTTGGCAGGAAACA[G/C]CTATGTACAAAGGCCACACACCTGGAGGATCCCCAAGAACGAGACTTTTTTGAGTTGAAT
3596	CaTSNP6537	AGTCCTTTTAATGGTTGAAATTCCAGTGAAGGTGAGGGAAGATGACCGAGCATGTCCATT[A/G]CGCGAGTTTGATGGGGACGACGACATCACATTCCCAGATTCACTGCTCCACTGCCGGGCA

3597	CaTSNP6539	CTCCACTGAAGTTGCTTATACGAAGTGTGAAATGTTCCCAATCACCTATATGCTCACCAA[T/C]CTTGTTAAAAGAAATGCTTTTAATTCCAAATTTTAGAGTAGAGGGTCCATTGAATGGACA
3598	CaTSNP6540	AAATGTCGAAGACGAAATGGTTCAGTTGTTGCAACTTGCTGTAGATTGTGCTGCCACATA[C/T]CCCGATAACCGCCCTTCGATATCTCAAGTGAGACATCACATAGAAGAGTTGCATAGATCT
3599	CaTSNP6541	TATCACCAACAAGCTTCCAAAACCTATCAATCGAATCCTCCCTTCCCAGAACCCTCGCCA[T/A]TGCATTATAAGTACATCCATCATGCTTAAAAACTTCACTCTCCTCCACCCACCTGAAAAA
3600	CaTSNP6542	TATTATTTGAGCATTTTTGGAATTTGATCTGACATCCCAATCCTCAACTGTCTTAGGAAC[G/A]CAAACATCAAATAATATTTGGCCAAGAACCCCATTAGCGTTGATGCTTGGGCGGCTTGGA
3601	CaTSNP6543	ATCTGGACCGCCGATTAAGGACCCCGGGGCATTGTTGAGATATGCTCTTCCTATTGACAA[T/C]AAGGCGATTAGAGAAGTTCAAAAACCGCTTGAAGATATTACAGATAGTCTCAAGATTGCT
3602	CaTSNP6544	CCTTCTCCTACCCAAGTAACTTTCTCCCACAAGAGTTGCATGATAAGGACGTAAAGAAGC[A/G]ATTAGTTCTTTCTGTCTTGGCAGTTGAGGTAGGGATGACGACTTCACCTGGTTTCTGTTG
3603	CaTSNP6545	GGGAATATTTCCAAGAGCAGCTTCCTTGATTGGCTGGTGATCTGTTCTTGGATCGGTGAG[G/A]ATAAGCAACCTAGGTTCACTGAAGGAAGTTTGTAGCTGATTGGTGAAGGTTCCAGGAGTG
3604	CaTSNP6546	CAAAGCAGGAATTTTATTTACCAGTATCCGAGCTTTCTCAGCTCGCTTAAGGTTTAAGTG[T/C]GCCCCTCTGCTTGCATTATACCTGTTCTCATCCCGATTATAGTCCTCAAGCCAGCTCTCT
3605	CaTSNP6547	GATGCTTCATCGAACTCCATGTCGCTGAAAACAAAGATCCTCTTAATCATCTGATCCTCT[G/C]TGAGATTCCCATTGACAGCAACTTCCAATATGCGATCAAACACCTTCTGGAAATCAGTGT
3606	CaTSNP6548	CACCGTCTCACCTTCCGAATCTCCGGTTGAGTCTCCGATCGATTCTCCTCCCGCTCCCGT[C/T]GCTCCTACTATTTCCCCCTCCGCTTCCGAACCTGCTACCGTCGGCGATGGTCCTGCTCTG
3607	CaTSNP6549	TCTAGATGGGTATTTGATGGAGAGAAGGAATAGATATGGAAAAAAGATGGAAGTACTTGA[A/G]CTTGGAATGCAAGAAATAAGAACGTATTACAGGCTATTTTTATTATCTTCAGTTTTACTA
3608	CaTSNP6550	CTAAAATGATCACATTATGGTACAGCGGCTCCTCTCTTCAGTTGGTTGTGAGTTTACTTG[C/T]TGGAACCTGTGTGACTCGGATGGGTTGTATGTGAATTCCGATGTATCCAGCCCGTACTTC
3609	CaTSNP6551	CCGAGGAGAACGCCTTCCGAAACCATTGTTGATGGATGCGAAAAGAGTGAGTTGAGAAAG[A/G]AGAGGCGAAAGAATGTTAATGTGAGAGTGGGGAGTAGGTTTAAATAAGTCACCAGTGAAC
3610	CaTSNP6552	CGGAGAGAATATGGATCAGAATTTTCTTCAGTTTGATCCAGCCCCTAGGCAAATTGAGCC[C/T]GATGCTACACGCAAGACTCCAGATTATTTTTTATAATAACTTCACGTATCTGCCCGTTTG
3611	CaTSNP6553	TACAATAGGTGTTCCAGCCGGAGGTTGGACTGCTATTAGATTCAGGGCTGATAATCCAGG[C/T]GTGTGGTTTATGCATTGCCATTTGGAAATTCATACAACATGGGGACTAAAGATGGCATTT
3612	CaTSNP6554	TGGATGGAAAAGTCAAGATGAAAGACTTAGCTGTGGGTATTCAAGCTTTAGGGGGAAGAG[G/A]GTGACCATGGAAGACTTCTATGATGTTAAGACATCAACCATTGATGGTCGTTCTGTGTGC
3613	CaTSNP6555	GCGAACAAGCACCGCTGTTGTTTTTGGGTAGTCTTCGATTGCTTTAGCAAAAGATTCAGT[G/A]AGTTGATGTTCATGAGCCGTGTTATCGATTATGGGGACCACTAGTTCATCATAATAACCA
3614	CaTSNP6556	TCCAACAAATTGTCCATCAACTCTTGGACCCTCACCTGGTTTCCTTGCCTCTTGAATTAT[T/G]GACAGTATTTCCTCAATACTCTGACATGAATCATCCACATTATTCACATCATTTTGTAAC
3615	CaTSNP6557	AGCTATGGCAGCCTTAAGCTGCCATTTCCATCCCATCAACTACTTGAATTCTCATAAGTT[C/T]AACAGAAGAATCAAGCATTCCATTTCCTGCTCAGCTCAACCTATACAAAGCAATATAAAG
3616	CaTSNP6558	GCTTCGTATAGAGCCCGATGACCCTGAGGATGTGAAAGCCACCAAACGGAAGAAAATACA[C/T]GCCTTTAAGTCCAAGATGCGGATGGAACAACTTGAAGTCACACAAAATAAGCGACAAAAT
3617	CaTSNP6559	TGGCATGGTAGATACAGTGTCAGGTCCAATAAGAGGAGCAACATATAAGGTGTCAGGATA[C/G]GCAGGATTCTTGACGCTAGTTGAGGCCCAAAGGAGCCTTTGCTTCTTGGCGCCTTTTTTG
3618	CaTSNP6560	CAAAACCCACCGTTGTCAACCTCAATTCCATTATTCTCTTCGTCGGCGTCACGATTCTCG[G/C]CAATCGTAGGTCGGAACTTCGCGAATCACAGAGCTGTGAACCTCCGTTCGCGTGATTTGT
3619	CaTSNP6561	ATTCTACCGTGATTGTAACATCTACGGAACCGTTGATTTCATCTTTGGTAACGCCGTTAC[A/G]GTCCTTCAAAACTGCAACATCTTTGTTAGAAAACCAATGGGGAACCAACAGAACACTGTA
3620	CaTSNP6562	CATTTTCCCCATTGAAGAGAAGCTTCAAGCGACCTTGAGTAGTGAACTGTGCCATAACCC[T/C]TTGCTTATTGTATCCTTCAAAGTGGGAATTTAATCTTAAAGATTGTAACTTTCTTCTGAA
3621	CaTSNP6563	ATTTTCGGCCTGTAATTTTGCCACTAAGGTGAGTCCAAAAACATTGAGAAAGATGTAAGC[G/T]GGATTGAGTAGCTGTCCCATAGCTGCTCCATTGACTAAAATGGAACTTATAAGAATGAAC
3622	CaTSNP6564	CTTTGATCTTTCAAGCTCTGCCAACACTTTGACAAATTTCATGGGCTCAACTTTCTTGCT[A/C]TCTCTTGTTGGTGGTTTCATCTCAGATGCTTACTTGAACAGATTAACCACATGTTTGCTT
3623	CaTSNP6565	TAAAGGTTATGAAGGTGTTGTGACTCGTTGGGGCGTCACTCGTCTTCCACGAAAGACACA[T/C]AGAGGTCTCAGGAAGGTTGCTTGTATAGGTGCCTGGCACCCTGCTCGTGTTTCTTTCACG
3624	CaTSNP6566	GAACCCCCCAAGTGCAGACAGTGTAATTGTTTTGCTTAAGCAAGCATTAAATGCTGATGA[C/T]CGTGCCCTTCTGCTGGATTGCTTGTATACGCAAGATGAGAAGGTTATTAAGAAGTCAGTC
3625	CaTSNP6567	GTCATCAAGGTATTTAGCTGGGATAACAGGTTTTATCACATGCTCTTTTAAATCCACGGC[A/G]ATTTGTTCGTTCGTGACAGTTTCATCATGCTGAGTTGAGATGAGGACTGTGTGCACACGG
3626	CaTSNP6568	AAATGGAAGCAAATGTGGTTGATGGAAATAGTACCGAAGCAGGACATGTAATTGTAACTA[T/C]CATTGGGGGTAAAAATGGCCAGCCAAAGCAGACAATAAGCTACATGGCTGAGCGTGCTGT

3627	CaTSNP6569	ATGAAAAAGGCAATGCCAAGGATGAAAATGTCTCGTGGTGCCTAATTCTTGATGATTTGC[A/G]CGTAGAACTGAAGGCCATCCCCTCCCAACGTACGATGTAGTTGGGTCCTTGCCTTTTTGT
3628	CaTSNP6570	AGAGAAGGAAATTTGCAATCCCTTTTTGGCGGGTCTTCATGTACAGTGTCTTCGTCTAAT[G/T]CAGCACCCGATCCACTGCTGTCATCATTTATATCGCCTTTAGGTAATGAATCTGTTAGTC
3629	CaTSNP6571	AAAAACTACACCAGAAACATACAAGGATAACTCACAAGGCAAACATCAACCCTCACCCGA[A/G]CTGTGTAGCGTGCCAGCCAGACCAGCTATCGATCTTAATACCAATCCATACTACCAGCAG
3630	CaTSNP6572	ATTGCCTGTTGTCAGAAATCAGCATTTATTGCTTTGCACCCTGCTAATTTGTAATGCCGC[T/A]GCCATGGAGGCACTTCCTATTTTTCTTGATAGTCTTGTTACTGCCTGGGGTGCTATCCTG
3631	CaTSNP6573	GTGGAGTTTGTGGATGCATTGGTTGGTGCAATAATGAAACAAGCGCAGCCGGGATCAGAC[G/A]TTGGTTCTCGGCTGCACTTGTCAGTTGTCATGAGCTATGGCAAGGTCTCGGAGAATGATG
3632	CaTSNP6574	CTGCAACCTCGCCTTGATGTCCTACTATTTGAAATAGTTTTCCCCCTTATGTGCTTTAAT[A/G]ATAATGATCAAAAGCTTTGGGAGGAAGATCCGCATGAATATGTAAGGAAAGGCTATGACA
3633	CaTSNP6575	GCCAATTATCTGCTGGTTTGGAATCGCTTTCCCTGACTCGTACTCCTGGATCACTTGAGG[C/T]TTCTCATTGATAATTTGAGCAAGTTGCGCCTGAGTAAGTTTCTTGTCCATCCGGGCTTGC
3634	CaTSNP6576	CATGCTGGCTTCAACTTATGCATTTCATTTTGCCACTACAAATTTAGTGGAGAAATACGC[T/A]CAAATGAAGAAGTCTCATGATGAAGAATTAGTTGCAGATGTCCATGCTCTCTCGGCAGGT
3635	CaTSNP6577	GTGCACCTTCACGTGTCTCCCATGATGGCTTTTTTTCATTAGCAATGTCCTTCTTGATCA[G/A]TCCTTTTATTGGCTTCAGGATGAACAAGTATTGTAGAGCAAATATGAGAGGAATCCATAA
3636	CaTSNP6578	ATTCTCATCACAAATAGAAACCAAACAGACAATAACAATGATGCTCAGAATTCGAAGCCG[C/T]GACGGCCTAGAACGAGTGAAAGTCGAAAATCCCAACGGAACAAAAGTCTCCGAACTCAAA
3637	CaTSNP6579	TGCATTCTCATTCTCACTTGTCTGTTTTTCTGCTTTCTGAATTTTGGGTGTTCCGTCGAA[G/A]CTTTTGTCAAGGCTGCCGGTTCTCTTGTAGAGTTGATCATAGTGTGGTCTGCAATACAGC
3638	CaTSNP6580	TTTCTTCTTCTTTTTTCCACCACTACTGCCATCACATTTATTATCACCATGTTCTTTATC[T/C]GAATCTGAATCTTCACCATCACCTACAACATCATCTATACAACCTTGATCACCACTACTA
3639	CaTSNP6581	TTTTGTACAAAGAGATTGGCGAATCTAAAATCTAAACTTACAGAGCAGTGTGCCTCTAAA[C/G]GCTTGTCTGTTTTCATCAAAATATAGCCCATATGTGGTTAAGGGGTTCTAGTACTTAAAT
3640	CaTSNP6582	CAGTTGCTATAAACAGTTTCAGAACTTTTTCAGTCAGCTTTCCAGCTTTCAATATTCTCA[G/A]GAAGAGGTCGAGGAGGATATGATCAGGGATGGGAGAGAGATCGGAGATGTCGGAGAGATT
3641	CaTSNP6583	TCCGGGGATTATTGTTGGATCTGTTTGTCTCCTGTGATTCAAGTGTCCTCACAACATCCA[G/T]TCCATCAATGACATGTCCAAACACAACATGAGCCTTGTCTAACCATGAAGTCTCTACAGT
3642	CaTSNP6584	AACCCCTGGGAAATCTTTCAACGACCAACGAACCTTTCGAGCACAAGCTTCACAATGCAT[G/A]AACACTTTCAGCACAATCTCCGCCGCTGGTGGCGGAGGACCACCACCTTCCTCTTGTTTT
3643	CaTSNP6585	TCAAAATCAACCAACTCACAGAAATGTTATGGAAAATGACCTGTTCAGGCAAATATATAA[T/C]GAAATGGACAGAGGCATGGAAGACAACATCAAGATTGTGGAAATGGATGTATGTGAATCA
3644	CaTSNP6586	ACAAAGGACATATTCCCGAAGTGATTTTAATAAAGAAGAGTTATGAAGAGAAACGTCAGA[G/A]GAAACGTGGGAGGTCTCGTCCATGGAAGCTTAAATCACTTCCCATGGAGGTTGATGATAG
3645	CaTSNP6587	GACATGACAGAAATTCGAATAGTCGCGATAATTCAGCGGATGATTTGAATGATATTGAGA[G/A]AGAAGAAATTGATCGTGCAATAGCGCTATCTCTTTCAGAGGAGGATCCCAAAGGGAAAAA
3646	CaTSNP6588	AAGAAGAAGAAACTTGAGAATCTATTTCAGACCAAGTTTATTTAACTTGATTGAATTTGA[A/G]TTTCAAGTCTTGCAATGCAACTAACTCAGGGTATGGAAGTTCTTTTCTGTTGTAATACAA
3647	CaTSNP6589	GGATTAACATATCTCCATGCTCATAATATAGTACATGGGGATATCAAACCTGATAATCTG[C/T]TGGTTACTCGTGATGGTACAGTAAAGATAGGGGATTTCAGTGTCAGCCAGGCTTTTGAGG
3648	CaTSNP6590	ATTTTCGCAGTCGCAGCACCCTTCCAACCACAACAGTTTCTAGGGTTTTGCGATTTCATC[A/T]CACAATGGTTCTTCAAAACGACATTGATCTGTTGAATCCACCAGCCGAACTCGAGAAGAG
3649	CaTSNP6591	CCTAATTGGGTTCATGGCTGGAGCTGCTATAATTGTATCTCTACAACAACTGAAGAGTCT[C/T]CTTGGAATCACACATTTCACTAAACAAATGGGACTTATTCCTGTTATGAGTTCTGTTTTT
3650	CaTSNP6592	GACTCAGGAGAAGGTTGCATACTATGCGATATCGGAATGTTGTATTGTGAATGCTGTGAG[G/A]GATGGAATGAATTTGATGCCTTATGAGTATACTGTTTGTAGACAAGGAAGTGTTGAATTG
3651	CaTSNP6593	TCTCAAGGAGCTGATACAGTGCTTTAGAAACTCTCTATCTGCCTCAAGAACTTGTAGTCT[T/C]TCATAAACATGGTCCACCTCCTCTTCTAAACCAACCTTTTTCTTATCCAAATTGGCCTTT
3652	CaTSNP6594	AACAACTGCCAGCACTCTAGTGGCTTAGAGACTATATCGGCTCATACTGGTGGCTTTCAG[G/T]TTATACAGGGTTGCTGCACCAGCAATCTGTGGGGTCATGTTGAGGTTCATCTGGAGGCCG
3653	CaTSNP6595	ATTCTATCGACTATAGTTCCCTTTTTGATCTCGAATATGATGGGCCAAATTTGTTGTACG[A/G]TCTTCCTGCCCTTGAGAACACCATAGAACCAGCAAATTTTCATTATGTAGAATCCTCTGA
3654	CaTSNP6596	CGTAAAGGACAAGCTAGATATCACAATTGGAGTTGCAGTTGGATCATCAACACAGATATC[A/T]ATGTTTGTGATCCCTTTCTGCGTGGTTGTTGGATGGTGCATGGGAAAAGAAATGGACTTG
3655	CaTSNP6597	AGAAGCGGCGTTATCAGATAATGAAATGAGAGTAATGGTGGAAGGGTATTTATGTCATTA[C/T]GACGAAGTATTTAGGGTGAAAGGCGTGACTGCAAAAGCAGACGTGTTTCATGTTATAAAT
3656	CaTSNP6599	TACCAATGTTTCAACTTTTTCTCTGTTTCTTATTTGCCAGTCCAAAAACATGTAAAACTA[C/T]AGATTCATTCACATCAAGGGTGAACTAAAGGGTATCTGGTAGGGCCTCTGTAGTCTCTAC

3657	CaTSNP6600	TACGACAACCGGCATCAGCTGGCTGACTGCAGGACCCAACTGCTCAAAGTAAGGGACTGC[T/C]TCTGTTGCTGGTCTGTTGTTGTAAGCAATTACGGCATTAGCCTTGGGTAGTTTCTCAGAA
3658	CaTSNP6601	AAAACTTGTTCAAACATACAGTGTATTTCCTAACCAAATGGAGACAAGTGATGTGGTGGT[T/C]CAACCATACAATTCACTTTTGACACTCAAGCGACTGACTTTGAATGCTGACTGTGTTGTG
3659	CaTSNP6602	CAACTTCTTCGTCTGCGAGGAGTGCAGACAACATTTTTACAAGATGTGTCAAAGTGTTTC[G/T]AGTCCTTTCAACAAAGCCCGCGATTATGCTCTCTGGTTGTGGAATTCCCACAACAAGGTA
3660	CaTSNP6603	AATTCAGATAAGAACAACGGAAATGGATAGGTTAGCTGTTGGTGGAACGGCGTCTCATTC[G/A]TTATATAAGGCTGGTCTTACTGACCCTGAAGAGGCAAAGCGTTTGAAGACCATCATGCTG
3661	CaTSNP6604	TTTTAAACAGATTGCCTTGAGCTGTGGGCAGTGATGTTGCTCGGCCAGGGCAAGTGTTGT[G/A]GCTACGGTCTCCGTATTGATTTCTTCAGACAACTTTGATTCACATAACATTTTTAGCCTG
3662	CaTSNP6605	AAAAGGATATTGGCAGTTTGACATGGGAGATGTACTTATTGATGGAAAACCTACCGGATA[T/C]TGTGCTAATGATTGTTCAGCAATTGCAGACTCAGGGACGTCTTTGTTGGCAGGTCCAACG
3663	CaTSNP6606	TAAAATTGCACTAAGATTTGACAAAGTGTTTTGGCCAGACGTAGAACTTATGGGTGTCGT[A/C]GCTCCTACCTCTTACTCCTGTGGCTACTTTCTCAATCTTCATAAAGCAACAGGCCATCCA
3664	CaTSNP6607	CAAACTTGATCAAAATCATGATGGCAAAATTTGTGGTGATGAAGTTGACAATTTAATCGA[T/C]TATCTCCTTGCTCGTGGCTTTGGAAAGAAATAATAATATAATATATAGGTGATCATATTT
3665	CaTSNP6608	GAACAAGATATTCAAGTAATAATAAAGATTTCATCTTTAAAATTTGGAATCTGTTTAAGT[G/T]TGTGAGAATTTGGTTGTTTGTGTCCTGTGTTTGTAGTAGTTCAGTTGTATGGTTGTTACT
3666	CaTSNP6609	CGTGGTTAATGGAGTGCATTTGTTGTTGCAATTCGTTCAAACAGGCGAAATTGAAGTTCA[T/G]AAAACATCAGTTGATGAAGCTTCGGATGCTCTAGAAACTTCCTTTGATGGTGAAGTTCCT
3667	CaTSNP6610	AAAGACTGCCCTGGCCAATGAGCGTGAACAGGACATATATTTTCAAGGGATTCCCGAGGC[G/A]GTTCCAGAGAATGAGCAGAGAGGTGGTGGATGTGCATGCTGATATTGAAATACTGAATCA
3668	CaTSNP6611	GAGTATGCTTGACATATTAATGGTGGCTTTGCTCTTCTCTAGCAATGGGGCTGCACTTGG[T/C]GTTGGTGTCATTGCCAAGCTAGGAAACTCACATGTGCGATGGAACAAAGTGTGTAATGTC
3669	CaTSNP6612	TCAGGCATTCCGTGAAGCACCGGCATTCCGCAACGGCAGAGAATGTCCGCCTCGCGAAAC[A/G]TCCGTCATCCACATTGCTATGACGCTTGACGCGACCTACCTCCGCGGCTCCGTCGCCGGA
3670	CaTSNP6613	TGAAGAAATTGGAATCAAGTTGAGCTCTGGTTCCTAGTCATTTTGAAAGAAACCCTTTAG[A/G]TCGGGTCAGAAATGGCTCCTGGAGGCAGTATTTTCAAAGAAGTTCTTGAATCTCATCTCT
3671	CaTSNP6614	GTATGCATACCTTTGAGAGCTTTGGGAAGGCGCCCAGAGATAACAAATAACACAGAGTAA[C/T]GCATATGCTAGAATGTCCCAGAATGCAGTGATGATCCAAGCACTCTGCCAGCGTTCATTA
3672	CaTSNP6615	CAGTCTTAAAATCATCCACTATGGACCCATCTTTGAACTTCAATGCAAATGTCGAAAGAC[A/C]GTCTTTCCCTTCAGTTGCACAAGCAAAGGTAACCCCTTTCTTCTCCATATTTGTGAGTTT
3673	CaTSNP6616	GTCTGTTCCAGTAGCTTTCCAATACCCAGATCCAGCTACTCTATTGGGTCTAGTCCCATT[T/C]GGATACTTCCTATCTCTTGGACTGAAAAAGTACCATTCTTTCTCACCAAATATCGCCTTA
3674	CaTSNP6617	TAACATGGGATGCAATGGAGGTCTCATGGACCATGCTTTTGAATTTATAATGAAAAATGG[T/A]GGCATTGATACTGAAGAGGATTACCCTTATCGTGCTCGCGATAATACATGTGATTCAAAC
3675	CaTSNP6618	GTACCCTATTCCACTTGCAAGTGTATGAGCTGGAGTTGTCAATCCACCAGAGAGATAATA[A/C]AGAAGATAAGAAGCTGCAGCAGATGCTATTAATTGATCAATCCAATATAGAATTGAACGG
3676	CaTSNP6620	TGGTGTAATGATTGATTCCAACAAAATCTAATGAACCCTTAACAAGAGCAGCCTCAGATG[G/A]AGAGAATTTTGGCAGCCTGTTTCCTACTCTGCTTCTCATTGAACTTGGATAATCTCCAAA
3677	CaTSNP6621	ATTCAAATCCACCTCTGCTAACGGATCATCGGAAAAATCACTGTCGCTTCCGGAAACATT[G/T]CCGTCGGAATCAGAATCGTCGTCACTGTCGTCACTGTGATCATCATCCTCCTCTTCTTCT
3678	CaTSNP6622	GGCGTAAGATCCCGACGAAAAGGCTTGGTTCGGATACATTGTAGGCAAGATTCGCAATTC[A/G]AAATATGAAGCTTGTTTCTTGCTAGCTCCCTTTATTACAATGGAATCAGCTTGACATCTC
3679	CaTSNP6623	ACCTGATGTGTCCACTGTTTGTGTTGGATTAGCAGCAAGTATGGGTGCTTTTCTGCTAAG[T/C]GCAGGGACCAAAGGAAAGAGATACAGCTTGCCAAATTCAAGGATAATGATTCACCAACCG
3680	CaTSNP6624	CGGAGATATTCGGTTTATGCAGCTGATCAGTCGAAATTCTATGGTTGGTAGCATCTCTTG[C/A]AGATGGAAAGCTTGAAATAGATCCACCTTCAAGTCCATTCAGAGATACAACATCATCAGG
3681	CaTSNP6625	CACGGTTTTGGCGAAGAACCAGTAACAAGAAGAACACGTGGTTTGTTAGGTCTAATAAAA[C/T]TAGGGTAATCAGGGTTATTACGAAGCCATTTAGATCGTTGTTCATCCCAATCGGAGATTT
3682	CaTSNP6626	CTTTTTCTGCGACTGAACCAGCATCTTCAGGACTATCCTTTTTATCACATGGAATGTCAG[C/A]AGGTGTGACGTCAAAAGAAATGACAGCTACAGAGTCTTGGATGGAAGTCTCGTGTGCTTC
3683	CaTSNP6627	CCAGCCACAGTATGCTGTGTCTGAGCTGAACGAGAAAAACTTCTGGAGGATGTTTGATGC[G/C]GGGCTATACGAGCATTCTAGGAGGAAGTATGGAGCTGTTGGAACCTTCATGAGTGTGTAC
3684	CaTSNP6628	ATATATATAGGTCACAACTGGTTGTTCCAATAGTAAATTAAACCCAATATCATTATAGGA[G/T]GTACATTATTACAATCCAGTGGATCTTTTGGCCATTCCAAATGACATATTCATCCCAAAT
3685	CaTSNP6629	CATCCAAATCTCACATTTGAGAGAAGAATCAACCGTCCGAACACTTAGACGAAGGTTATC[G/A]TTAAACACAGGGTTCCTTCCTCCACCATTGATAATCTTAGTAGAGACAGAATTTTCAGGA
3686	CaTSNP6630	CAGCTGCTAGAAGGTTGCTTCCTGGTTGACACACATTCACAATGCACCTTTGTTCTGTTG[A/T]GTCAAGTGTGGGGTCATCACCAATGTCAGCAAGGCATTCATCATTGGGGCTGTAAATCCC

3687	CaTSNP6631	TAGAAGTTGTTCTTCTGTGTTTGATTACTTTGAAAACTAAGCATAAGACCCATCTAAGAA[G/C]TGCTATGATTAGAGCAGCTTCGTATATGGCTTGGCTTATTGTGAAACCAGAGATGGATTC
3688	CaTSNP6632	AGGGCATGTTGCAGGAAAGCCCGGACAAGCTACCCTGACTGGTCTTCCACCGATAAGTGC[C/T]GTTCGCTTGGAACAGATGAGTAAAGCGGTTCGGTGGCTTGTGATTGAACTGCAGCATGGT
3689	CaTSNP6633	ATCTTCTTTTCCTGTCTCGGGATCATAATAAACAGCACGACGGACACACAAGCTGGCTGT[C/T]TTGCCATCAAAAAAATCTCCATACCTCGTGGAAGCAATTGAATATAGTCGAACATTATGG
3690	CaTSNP6634	CTGTTTATTTGAACAAGGAGAAGCTAAAAAAAATGAATTGAAAATTTGGGTTTTGAGAAA[G/C]TACTATGACATGTTATTGTTTGACACAACAGAAATAATAACCGAATTGAAAAGCGAAAAA
3691	CaTSNP6635	GGTTAAGTGTTGGGATGAATGTCTGCTTATTGTGCTGCCTGATTGATTTTGCCTCTACTA[A/T]GTAATGCACTTTCTGAATTGTTCCTCTGGTGACAATTTGAAGGAGTTTTTTTAGCTAAC
3692	CaTSNP6636	TTTTCCTTGGGATTGTGACTCTTCCATGGCGGTGATTCGGTTGAGTTTTTTCCGTAGGGC[T/A]CGGAGGCGTTTGTTGATTAGACTCATGACGGGACCGTCGGTGGTTGTTGAAGCCGCCATT
3693	CaTSNP6637	TGGAGCTCCAAATAGATTTTTACCATCTATGTAACCAAGTTGTTCTAGTGAATCTACTAG[A/G]GAAGCCATGTAATCTGTCACAAGCTTTAGACGAGGATTGAGATAGCGAAGAGAGTAGGTA
3694	CaTSNP6638	TAATGTTCTTCTCTGCATCCACGTGCCCTTTTTGGAATTCTTCCACCGTCCTGACATCAA[G/C]ATAGACATGGGTTGTCTGGATAAGAGACTTGGCTGTAACCACATCAACTGTGACAACCTC
3695	CaTSNP6639	TCCGATATTCATCACTATCATCTCCTTTACTTAAGCTTTGAATAAGAATTACCACTGCAA[C/T]CACAAAAACAAAGATGTACACCATTCTAATGTCAAAAGATCTATGGATAAATTGCATCAG
3696	CaTSNP6640	TGTTGATAGGGCTCAACCTCAACAAGGATCAGGTAAGGATGATGGTGATCGCTACAGAGA[A/G]CGTGGTCGTGATCGTGACAGAGATCATCGTGGTGGTCGGGGATCTAGTGATGGAGGATGC
3697	CaTSNP6641	CATCAGTTCAAATTTCACCGGCTCATCACATTCCAATTGAATAGCCACTTTTAATAACTT[A/C]AGCGAAAAACCACAAGAGCCACTTCCCATATCCGCTGGCAATAGACCGACAATGGTCTCC
3698	CaTSNP6642	GAAATCCTGAACCCCTTTGCCTGCAAAAACTTTATCAGAAAGCTCAGATAAAATAGCATT[G/A]ACAGTAAGCAAAACAACGCTAGAACCAAAGATAACACTAAGAACAACACCAGTTGTACCC
3699	CaTSNP6643	GGTTTTGAGCATGAGTAGATTGGGTGGAGCTGGTGCAGTGGCGCCACTGGTAACTGACAT[A/C]CCATTATCGTCAGTCGAGGAAGAAGGAAGTGATAGTGGAAGAAACCAACCAGCTTGGGAG
3700	CaTSNP6644	AAAGAGTCTTCTTTCATTCTAAGTTATCAGCTCGCTGAGGACTTGGGCAGAGCTTTCTCC[A/G]AACGAGCTATACTGAAAACGTTTATGGAGGCTGAGTCAAATTTACCAGCTGGTTCATCAA
3701	CaTSNP6645	GGTGTAACCTGTGAGAGCTTCTACAAGAGAGATTTTCTGTGTGACGACAAGATCATTGCC[A/G]TCCCTCTTGAAGACGCTGTGAGGCTTTTCGTCGATAATGAAAACAAGATCTGCTGGTATA
3702	CaTSNP6646	CCTGATACGGACATGGCGGGCAGTTCGGTCTTCTCTTCGGGTAGTGGATTTGGCTTCAAC[G/A]ACAAAAGATAAAGATGAATTGGAATTGGGATTTGGCGTTGGAATTGTTAAAGAAGAAGAG
3703	CaTSNP6647	TCCATTTTGTCGGAATCCGGTAGACTTCACCAAATCAGCAGGGCTAGACTTTGCTCGGAC[G/A]CCATTGTCCTCTTTCATCGGTTCTATCTTTTCTTTAACCTGTGAAGAAGACCTCTTCCTT
3704	CaTSNP6648	CATCAACTCCTCACAGTTGTCCATGATGAAGACACGACGGACATACAGCTTGATGTTGTT[A/G]GGCTTCTTCTTGGTGTCAAAGAGGTCAAAAGGTGCCCTCTTGGGTACAAAGAGGACAGCC
3705	CaTSNP6649	CACCATGACCCGACGTTGCTCCCATTGTAGCCACAATGGGCACAACTCACGAACATGTCC[G/C]AACCGCGGTGTTAAGCTATTCGGAGTCCGATTAACCGACGGGTCGATCCGGAAAAGTGCT
3706	CaTSNP6650	ACAGTTTATTGCAGTGAAAGAGCTGCTGAAAACAGCTATCAATCGTGCAACATCGCGTAA[T/C]GACCTCTTGACCATTAGAGATGCTCTTGAAGTTTCTTCTGATATGTATAAGAAAGACAGT
3707	CaTSNP6651	TGTTAAAAGAGCTCTTAGTTACCCTCTGAAATTAATAGCAAAGAATGCTGGTGTAAATGG[C/A]AGTGTTGTGAGTGAGAAGGTTCTGTCCAGCGACAATACAAGATATGGTTATAATGCTGCC
3708	CaTSNP6652	CGTTTCGTTTCTCTTCACAGTGAGGCTTACTCTGTCCTAAATCACTCAGCTTAATCCAAC[G/T]TCACAAAGATGAATCGTGAAAAGTTGATGAAGATGGCTGGTTCAGTTCGAACTGGTGGAA
3709	CaTSNP6653	CCATAATTCTGCAAGCTCAGCATACAACTTTCTGATCTGTGGACCTGCATTTTCATCCAA[T/G]CCATCAACAAAAATGTCATAGAGATCACAAATCTTGTCCTCCAATGCAGCATCCATACTA
3710	CaTSNP6654	GATGCATTATCCTCAGTATCATTACCCTGGTTATTATCCACCTGTGCCTATGCCCCATCA[A/G]CATCAACATCAACATCATCCACACATGGACCCAGATTGGGTTTCCCGTTACTACCCTCCT
3711	CaTSNP6655	TCAAAGTTGGACTCGCACTAATTCCACTGCCCGTTAAAACAAAAACTTGCCGGAAAACGC[A/G]GCCGGAGTTTTTTGCCGACAGCCAGAAACATTGAACAAGTTCAGAATTGGGAAGACAACT
3712	CaTSNP6656	ACGTGCATAGAGCACAGAATTTAACATATTCTCAAACAACAAGAAATATCCCATCCATTC[C/T]GAAATGATTATATCAACTTTAGGAACTGGAAGTTCAAGTTCTTCAATCTTCCCTTTCAAA
3713	CaTSNP6657	GGGTAGTCCCCATGTCGTTGTTGACAGTGTTGATTCTTACTTTCCAGCTGACAACTATGG[T/C]GGATGTGTGGCTCCAATTGAAAGAGTTCAATCAGAGGAGGATGATGGGAGTGATGATGGG
3714	CaTSNP6658	TGATCCAACATACCCTATTGCGTGCGCGATTTGGGCCTTGACCCAACAAGTCACTACACA[C/T]TGCTTCAGCTTCAGCATAGGTCCTTATAGGTGCAATGTAGCCTAGATGATCACCGTATGA
3715	CaTSNP6659	TCTTCACAGGCCACTAGCAGATGCATGTTCTGCAGCTCTTTTTCAAACAACAATCTTGCC[C/T]GGACTGATATAAAATATGGCACCCATATCACATTTATGTTATCTCCTCCCACGGTGACGT
3716	CaTSNP6660	CTTGGCCGAAGAAGACGAAGATTACGAGGATCTTTACAACGATGTTAATGTCGGTGAGGG[T/C]TTCCTTCAATCGTTACACAAAAATGACGATTCAGTAGTTAAAAACGACGACGCTGAAGCT

3717	CaTSNP6661	AAGTCTTTTGATTTTGCCTCACCATCTTCAGGTTCTCCTGGATTTTCGTCTTCTCCTTTC[G/A]TTAATTCTTCTATTACAATGCAAACTCCAAACCCTGAATCTTTGGTTATTAGTAATAAAG
3718	CaTSNP6662	AAGGGTTCATGTATTCAAATATAGGTTTAAAAATGCGTTGGATTTTGGGAAGAATGGTTT[G/C]CATTCGATGAGAGTGAATCCGGGATTGTCGTCGTTTGCGGAGGATCCGGATGGTGCGGGT
3719	CaTSNP6663	AGCTGGTAGTGGCAAGTCTACATATTGCTCAAGTTTGTACCAACACTGTGTAACTGTACG[G/A]CGATCAATACATGTTGTGAACCTTGATCCTGCTGCTGAAAATTTTGACTATCCCAGTTGC
3720	CaTSNP6664	ACGCAATCTCTCTCTCTCGATCTCTCGATCTCTCTCCCGACCGTGTCAATCTATCTCTCC[T/C]GATCTCTCTTCTCACTGTCTCTTCCGCGACCATATCTCTTGCCACCGTCTCTCGCGATCG
3721	CaTSNP6665	AATTAGGTTCCGTTCAATCGGTTTGCATTATGGAAGGCTCTGATGTCAGTAAAACGGCAT[C/T]GTATTCCACAGGAGTCACTATCCAGTTTAAAAATGAGGAGGAGTGCGAGGCATTCCATTC
3722	CaTSNP6666	AAATAATTCATCCCTAGCTAGGTCTCTACAAGATTTGATGCAAGAGGCAAATCAAGAGGT[A/G]CCTGCTTGGCTCTCGCGGTTTGCTGCACGATCTTCCTTTGGTGGAGGGAAGAACCGTCGA
3723	CaTSNP6667	CTACATAGATGCTAGTGCTGTACAGGCTTTGAAAGACTTGTATCAGGAGTATAAGTTACG[A/G]GACATTCAGATTGCAATATCAAATCCAAACCCAGAAATTCTGCTTACGTTGTCTAAATCG
3724	CaTSNP6668	AAACCTCTCCCATCCTTCACCACCTTAACACTACTTTGCATCACAACACTCTCTCTCCTT[C/G]CATCTCTCACTTCTTCTCGTTTCATCACTCCATCACACACTTCTCTTCTTGATGTCTCTT
3725	CaTSNP6669	GAAACTGCTGCAACTCTCCGATTCGCTGATGTCAGCACTGGGACTGTAACTACCGCATCG[C/G]TCAGAAACTTCTTCGGAGGAAACACTCCCCATTGTTATTGAACAGAAATTTCAGACGGAT
3726	CaTSNP6670	CTTCTGTCATAATCATAATCATAACATTGAATTCCAAATTCACACTCTCGACCCGCCTTT[A/G]TCAAGTGTGTAGCGGGGACGAAATTGCCCACCACAAAACGACCCTCAATCTTGTGCGGAA
3727	CaTSNP6671	AGTCCTTGAGCTTGATGACGATATTATGATGACATGTGATGATGTTGAGCGAGGTTTCAA[C/T]GTGTGGCGTCAACATCCCGACCGAATTGTTGGATTCTATCCTCGGCTTATTGCTGGTAGC
3728	CaTSNP6672	TCCAATTGCACCACTCTCAAGCCTTAACATATTCTCAACATACATGACAATCTCAGGTTC[C/A]GACATTTTGCAGGATTTCGGCATTTTCTGGACGACTATTGGAGATAAACGCAAGGGAGCA
3729	CaTSNP6674	AGACCACTACCAAAGATGATAGTTCTGAGCCTCATAATGATAATAAGCTAGCTTCTCCAG[T/C]CAGTGTTCCACCTGGTCAAAACTACATCTCTTCTCCAACAGCCATGTGGCCCGGATCACA
3730	CaTSNP6675	CTCTTCTTTGGAGGTGTTGTCTGTGCTTGTGTTGTTGGATCTACTACCCTTCTTACTAAC[G/A]TCACTATGACTGCTCAAACCAAAGAAGAGCTTCTTGCTACCCATCTCGAACAACAAAAGA
3731	CaTSNP6676	AGACATTGATATTTCATTTTTCTTTTCTACTAGTACTGCATGATTAGAATTAGAAGGAGA[A/T]GCACAATTTTCTGAAACAGAATCAGATAATCTTTCCTCAGAGTGAGATTTGGAGCACACA
3732	CaTSNP6677	TGAGCCAGAGCTTGAAAGAGAAACAGGGGAGAGAAGAAGAGGAAGGGATATAGTTATAGC[C/G]ATAGATCATGGTCCCAATAGCAAACATGCTTTTGATTGGGCTCTTATTCATCTATGCAGA
3733	CaTSNP6678	AACAGTTGAAGAATAATTGTCGCGAGATGGGAATCCTAAGGAACCTTGTGCAGAGGTTGA[A/G]GTTCTATTGATTGGGAAGAAAGAATCTTCGTATGATGCTAATGGGAATTCAGCTATCTTA
3734	CaTSNP6679	CCACCGGTTCTAGACTGAGCCCTTTCCCGGTCTTTCTCTCTCTGGTTACCACGAGTCATG[G/A]TTGTTGATGGATTCTTCTGAACGGAACGAGATGAGAGTCGACGAAAAACCCTGATTAAAG
3735	CaTSNP6680	CATGTTGTAACCCCTAACAGAGTAGGGACCCCCAAGAGTAAAAGCATCATAACTTGGGAG[G/A]TCGCCTACGCAGCCACCGTAGTGGCCATGGAGGACAAGCACTGGCGGCGGTGGTTTGCCA
3736	CaTSNP6681	AAGCTCTTTACTACAGAACTGATTATACTCCAAAGTATCACCTTTATCTTTCTTGACCTC[G/C]ACCACCAAAAACGACGGCGTCACGGCGTAAATATCCGCCGCAATAGCCAATTTCCCTTTC
3737	CaTSNP6682	TCACTGCCACCGGCTCGGTGTCGCACACCGCGACATCAAGCCGGACAACATTCTCTTCGA[C/T]TCCGGTGATAATCTAAAATTAGCCGATTTCGGTTCAGCCGAATGGTTCGGCGACGGCAGA
3738	CaTSNP6684	TTGCAAAATGGTGGCTTTATTGGGAAGACCAAGAAAACCTATGCTTAAAGACAGTGCGAC[G/A]ACGGGAACAATATTCGGTTACAGAAAAGGGAGGGTGAGTTTAGCAATTCAAGAAGACACG
3739	CaTSNP6685	GCCACCATTGAGCCAAAATGAAAATGATAACTTTACGTCTAACTTTGTCATGGGACGGGC[G/A]TTGAGCTCTCCATACAGTGCAAGTCCGAGCAGGTACAATAATCCCATGGGGTATCCTGGT
3740	CaTSNP6686	TTACTATAAAGGTGAAGAAGACAAAACAAAAGCAAAATCAAAAACAAAAGCTACTTCACC[G/C]GCTGAACAAGAACACCGTAGAAAAGTTCTCCGTGAACTTAATTCTCTAATATCTGGTAAT
3741	CaTSNP6687	TCTTCGAAGGAGTCAAAAGAAGATGGCGAAGCAAGAGAAAAGAGGAATACAAGGGAACGG[C/A]CAAAGAAGAAGAAATGGTTTAACCTCCACTTAAGAGTTGATAAGAGAAAGGCTTGCTAAG
3742	CaTSNP6688	TCCAGCAACTTCAATAACCCACCTTTGGAGATTGTCGGTGACTTTGTGCTTGAAACCAGC[T/C]GGGGGATTAACTTGCCACTCGACGAGTTCTTTCTGGAGTCTATTGCAGGCAATCTTACTC
3743	CaTSNP6689	GGTGAGAAATTCTCAGAAATTGTTGGAAGTCCATATTATATGGCTCCCGAGGTGCTCAAG[A/C]GGAGCTATGGACCTGAAATAGATATATGGAGTGCAGGAGTGATACTCTATATCTTATTGT
3744	CaTSNP6691	GTAAGAAGAAAGAGCTCCTCATTCCTTGTCGGGCTTCAAAACGACTGGCTGGATCTGAAC[G/A]TGGGCTGATGAACTCCATATTCTGTGAAAAAGCTTCCAAATATAAAAGCCAAAGGTCTAA
3745	CaTSNP6692	TCTTATCCACCAAAGCTTTGAAAACCAGTCCTCAAAATCAAGACATGCATACAGCTTGCA[C/T]GTGGTAGATACGGATGGCTGCAAATGGGCACCAGAAAATTGGATCAGACGGAAAGTGGAG
3746	CaTSNP6693	ACAAGTATGTCAGAAATAACCTTCGATCTGAACACAGTTACAGATGAAAATCCTAACAAC[C/T]TGTTCAACCGTCATGGAGCTGGTGTTGCTCCGTACGATGGAATCAACGGTTCAGATCAGA

3747	CaTSNP6694	AAACCTAATTTACACAGTTGCAACCAAGTACCCTGGAAATGCCTTGTCTCATCGTCCAAC[T/G]TTGCTGCAATACGTTGTTTCGTCTAAGGTGAAAACAACTGCGCAGTTAGATGCATCACTA
3748	CaTSNP6695	TGATGATATAAGAACATGCTGTTATATGCTTTTTTGATTGTCTATTGTAGTCTGATTTCA[G/A]TTCACCCAACTCATCTTGTTATCATTATTCATGCAATTTAAGTTCCTTGGTTAAAAAAA
3749	CaTSNP6696	CTTCAACTTTCATCTCACAATTCACGTTCGTAGTTTCGATCAGATTCGTCTTGCGTTTTC[A/G]AACAGGAAATTGAATTCTGCTGGAGATCCGAATGATCGGTTTGTAATTCTTCCGATCGGC
3750	CaTSNP6697	AGTGTGTGTTCCGTTACATCAATTAATGAAAATGGTACTGAGAACCAGTTTACAAATTTG[A/T]GCCTGGACGCGTCTGAGGCTGAAAGTTTGAAGAGCGAGTTCAAAATTGGCAGTGGTGATG
3751	CaTSNP6698	ACCAAAATGAGCATTCATGTCTAGCACGTTTCTTAGCATGTTGAAAGGTGGTGGAGGATC[T/C]TCATCGCCGGGTCTCTTTGGATGATCCGAAAATATCAAAGGTGACAGAAGAGACCAATAA
3752	CaTSNP6699	CCCACACTTTCACATCTTCTTTTTTCATACCAGGCATGTCAAACCTCATCTTGTATTCAA[C/T]TTCTCCTTCTTTAATCTCCCATGGTGTTCTTCCCCTCCTTCGATATCCACCTGCTCCTTC
3753	CaTSNP6700	CAATGCCAGGCGGAGCCACCGTCTGCTCCGGTCACCGGTTACACTGTTTTCCTCTTCTCC[A/G]CCATTCAACTCGTCCACAACTCACCAATCCAACTCAGTCTCGACTCGTTTCGATTCAAAC
3754	CaTSNP6701	GAGATCTGCTCTTATGACAACGGCTTATACGGCTTACAAAAACGGTCTAAAATTGCAGGA[C/T]AGTGCTACTGGAAAATATTCGACGCCGTTTGATCATGGATCTGGACACGTGGACCCTGTT
3755	CaTSNP6702	GGAATTGTCAATGTTGGGGGAAGAGGAGGAGTCGCTGTTCGCGGATCTCGGAGAGTTGCC[A/G]GAGTGCTCGGCGGTGTTCTGGAGAGGGAGGAGGAGTATTGCCGGCGAAGTGAGTGTGGGA
3756	CaTSNP6703	CAGGATTATTTTCTGACCTCCCTTGAGTGTCAGCAACTCCTGGTCCATTCTGAGGCATTG[C/A]TTGATCAAAACCAGGTCTGAACCCTGTCCCGGGAGGCAGATCTGTGGCAGTTAATCCCTG
3757	CaTSNP6704	GGTTGTGTTTGAAGAATTGGGTGGAGACCCTAATGGGATCTCTTTGGTCAGAAGGGATAT[T/A]GATAGTGTATGTGCTGATATTTATGAGTGGCAGCCAAATCTTATAAGTTATCAGATGCAA
3758	CaTSNP6705	AGAAGGAGTGATTGTTTGTGGATTTGGTCTCTGCGTCTCTCTCTCGAGACCTAAGAAGAA[T/G]GAGGTGGAGAGAGAGAGAGAGAGAGAGAGAGAGTGGGGCAGTTTTATTTTTGTGATTGGT
3759	CaTSNP6706	TTCTACTTTCTTTTTCACCGGACAATTTGTATGAGTACATTTGTAGTAACTTCGCGGATA[T/C]TCGCTACCCTTCACTTGCTTTTGACCGTATTTTCTCCAGTTATACCCGTCTTCAGATGGA
3760	CaTSNP6707	TATCGAAAGCCTAACAACTTTTATCTTGGAGAGCTTATTTGGGGCACCACCAGTGACCTT[G/A]GCGACGCGAAGGAGCGAAAGTTCTGCTTTCAGATCCTTCAATTGATTCAGAAGGTCTGCT
3761	CaTSNP6708	AGTCACCTCTTTCTCCTCTTCAACATTCCTTGTATTTGCTTCTTGTTGAGAAACCTCAAG[A/G]CGAGTCTTAATTTGTTCTGAGATAACACCAAATGCAAGAAAACCAACCCATGCAAGACCA
3762	CaTSNP6709	TCCAATATCAGACTGATGCTGAGGTGGTTAGATCTTCCGAAGTGGAGAATAATAGACCAC[A/G]TGGATTTATTAATGGCAATATTCCTTACAATAGGATGAATACAGTAGGTGAAAAGGAGCA
3763	CaTSNP6710	CAAAGAAATCAAACAATATGTGTTAACAAATAAAAAAGCTACTGCAACATTAGCTTTTCT[T/A]AACACAAGATTAGGAATTACACCATCTCCTATAGTAGCAGCATTTTCATCAAGGGGGCCT
3764	CaTSNP6711	GATGTCCAACATATTCCAGATATTGACGCAAGGGATGCCTTTGTAAATCTGCAACAGAAT[G/A]ATTATGAAAATCATTGGAATCCGCACTTGCCTTAGGATGACCAGAGTTTCCAGATATAAT
3765	CaTSNP6712	CCAAAAAGCAACAAAATTATATCATCACATAGAACAGTAGCATAATTGATATCAATTTGT[C/T]TCAAACCAAAACAAAAACATAGACAAAAACTGTAAGTTCAATTTCCAGCAGATTTCCTAC
3766	CaTSNP6713	TTCCTGGGGGTGACTATGAGTACATCGATGTGAATTATGGTGGAAATAGATACATTGTTG[A/G]AACATCCCTTATGTCAGAATTCGAAATAGCTCGTCCCACAAATCAATACACCTCTTTACT
3767	CaTSNP6714	AATTGGATCACGCTCCTGTCTCACACCAGAAATTTCATCACGTGTTCGGTATGTGCTGCC[G/A]GGATCAGACATGGAGTGACCATGGTATCTGTAAGTGTCCATTTCGAGAATAAGGGGCCCA
3768	CaTSNP6715	CAGCCTGACTCCCTTTTTCTATCATCTGGGGCTAAAGATGCTGGAAATCTGGCCAGTCGC[A/C]CAACGTCATTGAGTGATGAGCATGTTTGTGGGGGCCACGTGAGTGAAGGAAACAATCAGG
3769	CaTSNP6716	GATCCAGAAGGTGCTTGTAATGAGATGATGTCCAGAAATTGGCAGCCATATCTTCTTCAG[A/C]GATCAAAGAAACAATCCAAGATGAAGCAACAAATGGTGGAAAAACAGGTGAGAACGAAAC
3770	CaTSNP6717	ACATCGTTCTGGTCGTACTGGACGCGCAGGAAAGCTAGGTTCTGCCATTCTGATGTACAC[A/C]GGTAGCCAGAGAAGAACAGTTAGATCCCTTGAGCGTGATGTAGGATGCAAATTTGAATTT
3771	CaTSNP6718	CCGTAAAACTGAAAGGCGTTCACGAGGGTTTTGAATCGCTTCACGAAGGAACTGATCAAC[C/T]TGATTGACGGCATCATCGGAAACCTTATCGCCACTGGTGGAAGAAGAAGACGAAGGAGGA
3772	CaTSNP6719	AAAGCTGGTTCTTCATCTTCCACGCCTCCCATGAAAAGCCCATATATAATATTTTTCCAG[A/C]AATGAGCAATAAAACACAGTGGACAGTAAGAATACAACAACAAAATGCTTCCAAAAATAG
3773	CaTSNP6720	AAACTTGTGTCTCCAGCACAGAAGGATCATTGTCAGGAGTGGCAGGAGATCCAATTTCAC[G/A]ATCAGAGCCTTTAATTTCATTTTCATCAACATATCTGAAAACATCATTCAGAACAAAGTA
3774	CaTSNP6721	TTCCTCTGCTCGTCAGAAGTGCCACTGGTAGATAGCTCGTGTATATAGGTTTTCAACCAA[T/C]TTGTCCATGCTTCTTTACGCTCTTTACCAATATCCAACAACACTGACTTTAGTGGCACTA
3775	CaTSNP6722	GTGGAAGTATGATACAAACTCAGAAGGATATGATTTGCAGAGCACCTCTACGGGAGTTGA[A/C]ACCTTCGTCTCACTAATTTTGTCATATTTTTGTTTTTTCGTTCCAGCCTTTAGTCCCTGC
3776	CaTSNP6723	TGCTCCACAAAGTTCTGATTGGAATGGATATAAGGCACCTGTCTACTTATCTGAGAGGAT[T/C]GTGCATCCATCTTCTACATATGTCATGAACAAGCCAACAATTGAAACAAATGTCTACATG

3777	CaTSNP6724	GCCACCACCATAGCTGCTTGGGGCACCATAGCTTCCACCCCTGTTATTATCATTACGTCC[A/G]TATGTACTACCACCATAACCACCACCGCCAAAGCCACCACCCCCATAGCCACCTCCACCT
3778	CaTSNP6725	AGCAAGTTAAACGAGGTTACAATAGAATAGTGAAAAAAACAAATTTGCTTCCTAATGAAT[C/A]CGCATAAACACAGAAAAGAAAGGCCAAATAAAATTGCTTATGATTATCCCAACTCTTCTT
3779	CaTSNP6726	GCAGCCGGTGATGCGTCCACCCGTAACTCAAGGATACTACGCTGTGCAACGAATGCCATC[C/T]GATTCTTACCGTGAAACGCAGGTTTACGGCGGCGCACCTCCTTCTAAAGCGCCGTTTTCG
3780	CaTSNP6727	GGTTGTCGCTAGTAAACCAGATTGGTGGCCTTCTGATTGGCTTCAAGATCTTCAACAGCA[C/A]ACTGCTTTTGCATAGGATGTTTGATGTTATTCAAAAGATAAGTATCTTTGAATAATTTGG
3781	CaTSNP6728	AATCTTTTTTTTTTTCGTCGTAATCATGATTATAAAAATATAAAATGGCAGTCATCAAAA[G/T]ATATCTTCACGTCCAAACAAAATCCATTTGCATCAACACTACCGCCTCAAGCTCTCCACA
3782	CaTSNP6729	TCCCACAAACAGGATCTACCCTGAACCCTATTTCCCCCAAATCGATCACTTCGATCGTAT[A/C]CCCGATTCTCTTCTCCTTCTCGTTTTCAACAAGATCGCTGATGTTAAAGCCCTAGGTCGA
3783	CaTSNP6730	TCTTTCCTTCACCGACTTGTATTGTTCTTTCACCGTTTCCCTTTTTAGATTGTGTGTCTC[G/A]CTGTCTTCCATCCAAGCAACACTATACAAATCCCCAAGGCAAGTGATGAACTCAGGAGGT
3784	CaTSNP6731	ATCTGTAGCTCAGATCAGCAAACTCAGGGTAGGACAGGTCTATGCAGCATCAGTAATGTA[T/C]GGCTACTTTCTTAAGCGTGTTGTCCAACGGTTTCAGCTGGAGAAGACAATGAAAATTCTA
3785	CaTSNP6732	AAGCATAATATGCCTAATTTCTACAATATCAGGATGACGTAGCAAGCGCAAGAGCTTGAT[C/T]TCTCTTAGTATCCGTGTGGCATCTGATACATGCTCAAAAACATCATTAATTTTCTTGATT
3786	CaTSNP6733	GTTTGGATTAATCCTCCTGATCAACCCCAACACAGCATCTCTTGGACAATTCGCCGTCAC[C/T]GTTTCATCTGAAAGGTTCTTCAATCTGTATAAACAGTTAACAACTGTTACTTCTTCTCTA
3787	CaTSNP6734	GGAGAAACATATGGGAGCCTCTTTTGAAGAACGTCCTCAGCCTTGGGTTACAAATATCAC[T/C]GTTGATGACATCCACTCTGGAGATTTCCTTGCCATATCAAAAATTCGAGGTCGCTGGGGT
3788	CaTSNP6735	TCTTAGCTCCTTGGTGTGATGAGAAGGAGGTGGAATTAGATGTCAAAGAAAGGACAAAGA[G/A]TGAGATGGGAGCAGCGAAGGCTCTATGTAGTCCCCTTGATCAGCCAGAGATCCTAGAAGG
3789	CaTSNP6736	TAGATTGAGTGGGAAGCTTGGTGGATTGATCTTGATATGAGAGTGGTTCATCTTGTTTAT[G/A]GATGATGCAGATTTGGTTAGAAGGAAAGAAGCAGAAGCAGAAGCAGAAGCAGTAGTCATG
3790	CaTSNP6737	TTGGCAGCCAAAATTATCAATTTGGTGGGTTCCAAAAGTAAAATTAACTAATTGTATACT[G/C]GTCCCATCACATTTTCACTCATTCTTTGTGTCGGCATATCAAGACTGTTTTGCATGGTGG
3791	CaTSNP6738	AAAGTGGTCTGGAGTGGAGGGTGACTACAATGTCATGGCTATTGACCTTCTTGGACCAAG[C/T]CTTGAAGACTTGTTTAACTATTGCAGTAGAAAATTCTCTCTGAAAACAGTGTTGATGCTT
3792	CaTSNP6739	GGGAGAGAAAGCAATGGTCTGGCAGAACTCATGGGCCTATAGTACTCGAACTATTGGTGT[G/T]ATGGTGATGGTTCATGGTGATGACAAGGGATTGGTGCTGCCTCCTAAAGTAGCATCAGTT
3793	CaTSNP6740	TGTCATTGAGTCCATCATTGTCATGTAACGCTTAATTTTTGCCTGGCTTTCCTTCTCACG[A/G]CCTTTTGGCATTAATTCATTGCTAAATCCTGGTAGCATAGAAAAAACCTGGCCAATGGGA
3794	CaTSNP6741	AGGATACGACAATGTTCCTCCAGAAATCAACAAGTTGAGATGTAGGGTCAATTATCACGC[A/T]CTGAAATTTCTTCCTGATATAGAGCAGATGGCTGATTTACTAGCATCAAGAATGAGAAAC
3795	CaTSNP6742	AAACCACTCTGGTAAATTGGATATCTTGGTAAACAATGCTGGAATTGTAGGAGCACAAGT[G/A]AATGGTGAGGCTTTGGCTGCACTTGGTGTTGTGGTAGATCCTAGTCAAATTGACTGGAAA
3796	CaTSNP6743	AAGGATTAGCAGTGGAAGTTTGGAAGGAATCACAGCTGAAAAGTTCAATGAAAATAGTGA[A/G]TTGTGGAAGAAGAGAGTTGCATACTACAAGACCCTTGATTATCAGCTAGCAGAGCGTGGG
3797	CaTSNP6744	AACAAGATGACACAACCTTTTGTGGATACCCAAATCCATATTAGGCGGAACAACAACATC[G/C]GAAAAGACATGTTTGGAAACAGCTGAGCTAAAAAAGGTAACAGCTTCAGTACCTAATACA
3798	CaTSNP6745	AACGAGGGTTCAGATCTTCGATTAACGAAACTGGGTACCAATGATCCCTCCATGAAAACT[C/T]CGAAGACGAACTCTCATCACCAAATTCATCATCAATTTCGTTCCTTTCAGCTTCTGGGTA
3799	CaTSNP6747	CTTGATAGTTGACCTCAAATTACCTTATCCTAGTGGCTTTGCAACAGCAGTTCTCATCAA[T/C]GGATTCCATACACAGGATAACAAAATGGCCAAGAAGCAAGTGCACGGGTTCATGAAGTAT
3800	CaTSNP6748	AGGAGTCTCTTTCTCTTGCTTCTGAAAGCATATAGTGATTCCAGTTTGATCATATATATT[A/G]TGGGTGTATACATATATGAAGATGCAAACGTTTCTTTTTAATGATCAATAATACATTGCA
3801	CaTSNP6749	CATCTGGAACCACACCTTCTCCACCATCTCCTACCCCACCTACCAACGGTGGTTCACCAC[T/C]TCCGTCGTCTCCAACTCAGCCTGATGCGACTCCTCCATCAGAGGGCTCAGCCACCACCCT
3802	CaTSNP6750	TGATCCTTTACTTATCCTTGAGAGATACCTATGGCGAGGGAAGCATACATTTGCCATTGC[T/G]CAACATAAGCATCACCGTAGTATATACGAGGAGGCTGATGCAAACAAGAGGCGAAAGCGA
3803	CaTSNP6752	AACTTTTATATTTGAACGTGACATGAGTGTTTGGTCTTTAGGCTTCGATTCTAGGCTTCT[G/A]GCTTGTGTTTACTCTGGTATAGTGTGTTCTGGAATGGCATACTATATACAAGGAGTTGTG
3804	CaTSNP6753	ATCTGATGGTGCTGTTTTACCTGCATCTTTGCCATTGCCTCCCCCACCTCCCATGCCTCC[A/G]AAGCCTGCTGCTGCAGTGCCTACATCATTACCACCACCTCCATTGCCGCCTCCACCTCCT
3805	CaTSNP6754	AAAGATTCCCAGCAATGCCGCAGGCACTGGTATAACAAAGTAGAAGTACAAAGTTGCTCT[C/T]GGATAGAGGAATATGTCAAGCAGCATAATTGCATTCACAGCTCCACTTGCCCCCAATGCT
3806	CaTSNP6755	AAAGGTGAACGAGACAAAAGCTATGGTGAAATTTCAGCTTAAGAAGGTGCTCTGCATGGG[A/T]GTTGCCGTGGGCAACATTAGCATGGATGAAAAGCAAATTTTTCAGAATGTTCAACTTAGT

3807	CaTSNP6756	GAGAACTATTCTTAGTGACCATATAGAACAACGGTTGTTTAAGCGGCTAAAGCACGAAAG[G/A]CAAGAGAGAGCAAGGTTTCATGGGAAAAGCTATGATGAGGTGCCTGGAGCTGATTCTCTT
3808	CaTSNP6757	GCGGCGGTGGAAGCTGCAGGAAGAAGAACGACGCCGTGGAGAGAGGCGTTTTACGGCGCT[G/T]GTATGAGGCCGGTGCAGTTGAGTCAGTTCGCGGATTTTCAGGCAGAGTGCTTGCTGGCGA
3809	CaTSNP6758	TGTCTCTGTTGGTGCTAACATCTCTGGTGGACATGTCAATCCTGCTGTTACATTTGGTGC[A/C]TTCATTGGTGGCCACATTACACTTATTAGAGGCCTTTTGTATTGGATTGCTCAGTTGCTT
3810	CaTSNP6759	CTCAAGGACATGCCTGCAGTGGCTTAAAGAAGAGAAGAAAATCACAACTTTGAGCTGAAT[T/C]TGTTGGTCTTTTCAGATGCATTGCTCCGTCTGATATATTATCATGCTATTCATATAGTTT
3811	CaTSNP6760	AAACTTGGAGCGCGTGATGTTGCTATTGCCTCATCGGTCTCCAAAATTTTTGCATCCACA[A/C]TGACATATCCCCATGAGGTTGTACGATCCAGATTGCAAGAACAAGGGCATCACTCAGAGA
3812	CaTSNP6761	TGTTGTTTGCTATGTTTTGCTTTCACTTATTTCCTCTTACAGGCTATTTAGCAAGTATGA[T/C]GCACCAGCACCACTCAACAAACCCAACAAGGACATTGACAATGTTGCTGCTTTCAATGGT
3813	CaTSNP6762	ATTTCCATCGAATTTCGTGGATGGAAGATCCGTGTTAATCCAATTATCCTTCAAAAAACC[T/C]GCACTCCACAGAACACTCCCTGCTTTAGGCTGTGGCGATTTTCTCGGTTTCCAAACACAT
3814	CaTSNP6763	AGTCTTAAACCCAAGACCAATAGATTTCCAGAAGCGGTTTCCACCTTTTCCAGGCCTCTT[T/A]CCCTTCCCAGATTTCTTAGAGCATAGAAACACTTTAGGTTGCTTCAGAAAAGCCTTCTCA
3815	CaTSNP6764	AAGGATTGTGATTGGATTGTATGGTGACGATGTCCCCCAAACTGTAGAGAACTTCCGTGC[T/C]CTTTGCACCGGAGAGAAGGGCTTTGGATACAAGGCCTCCTCCTTCCATCGTGTCATCAAC
3816	CaTSNP6765	CCCATTCCATCTACCAAGTAGATCCCGGCGTTTGCATACCCGCCGCTGGTCAACCATCTC[C/T]TCCGCCTGTTTTAGACTTTCATCCGTCAAAGGAGAGCATAGACGCAGCTATTAGTGATGC
3817	CaTSNP6767	AGATGAAGAATTATCAAACTTCGATTTGGAAGGGGCATTTTGGGGATTTGGCTTGATCTT[A/T]ACCGTAGCGCTTTTGGTCTCTGTTGTAGCCATATATAATAACACGACAATTCGATTGATT
3818	CaTSNP6768	TCTGATAAGGAAACATAAAAAACATATGGTTGTCACATAGCCAACCTCTTGCAGCTTCTT[A/G]CGTCGCCCTTTGGATTCCACAGGAAAGCGTTGCAGCATCAAGAATAGTCTTCCACCATAG
3819	CaTSNP6769	AAATGCCGGTACTCAAGCTCCAATATTGGTATATCCTAATGACATAGATGGTGTACCAAA[G/T]ATATCTTTACCAACTTTTGCTATGGCATCCTATAAGTTAAAGAGATCTATTTGGATGCAA
3820	CaTSNP6770	AATCAACAAGTTGACTAACCCTTCTCTGGTTTTAGATTCTAAATCCATCGAAGACACTAA[G/C]GCCGTTGTTTACGAGCTCCGTGTGTTAGCGAAAACAGATTCTGAGAGCCGAGCCTGTATA
3821	CaTSNP6771	ACCCCTTGTCATTTGGCTTAATGGAGGTCCAGGCTGTTCATCAGTGGCATATGGAGCATC[C/T]GAAGAGATTGGCCCATTTAGATTGAACAAAACAGCTTCTGGGCTATACATTAACAAGTTT
3822	CaTSNP6772	TTTTAGAATTTTCTGGATGGATTTTCTCACTTTTCCGGCGGCCGACGATCGATTCCGCTG[C/T]GTTTCCCGGTAAGTTGAGATCAGTGATGCGACTCCAACTTGTTTCAAACGAGTGATTGTT
3823	CaTSNP6773	GGTCCCCTTTTCCCCTGTGCAAAGAGCTCGGAAGTTTTCTGCAGTTTTTGGAACTGTCTT[G/C]CCATAAAGACCTATTACAACACGACCTGCAGCCTTTCCATCAATCTCAACATCAAAGTAA
3824	CaTSNP6774	AGCGCCTCCTGCTCCTAGAAATGTTGCCCCCCCACCACATGCTCCCAATGGGTCTGCACC[G/A]TCTGCTGTTAAAAGCCGTATATGCAACAAGTTTAATACTGCAGAAGGCTGCAAATTTGGC
3825	CaTSNP6775	GGAAAATAAATTGCAGTACAAAGAATACTGAGCATTATAATGAGAAAGGTTTTCTATACA[T/C]ATCGAAAGGTTCGCACGCTGTTAACTGTGGTTCCTGTCTCAGCCCACCAGCGCTAAGACT
3826	CaTSNP6776	GCTATGTCATCCTCTGTAGGTTCTCTTGCTAGCTCCTTCTGTAAATCTTCTTTCACTTGA[T/A]GGAGTGCCTTCATTGGTTGCATCAACTTGAACAAAAATGCATGCTCTGAAGAATGAAGAA
3827	CaTSNP6777	GTGGGATCCGAACTCAGACATCAAATCTTGGTCTTCCGGCAGAGTCTTCAGGGTTGGAGA[T/C]CAAATCTGGTTGGCGTACTCTGCAGCAGAGGGATTAGTTGCGGAGCTTGAAAGCAGAGAA
3828	CaTSNP6778	AGGCTTATCATGCTCTTCATAAGAAATCTCTTGAGGAAGATGTTGCTCATCACACAACCG[C/G]TGACTTCCGTAAGCTCCTATTACCTCTGGTTAGTTCATATCGATATGAGGGAGATGAAGT
3829	CaTSNP6779	TGAATTGAGACCGTAAACGGAGCTCGACGAAGCCCAAACGATGGCGGGTTGAGGGTTTGC[C/T]GACTTACAAGCTTCTAGAAGATTCACGAAACCAGCGACGTTGCTGTGAACGTAGGAGCCA
3830	CaTSNP6780	GCGCAATTCTAACAATCCATGGGAGCCTGCTGATCTGGCAGATGCCCTTTGCAAATTAAC[G/A]CCAAAGGCTTCTCCGAAATCATCGTGAAGAACCAAAACTCCACCCGATGCTTTTGCAAGA
3831	CaTSNP6781	TAATAAATGGTCTAGGATAACTCCCTTTGGAGAGCCTCCGACCCCAAGGGCTGCACATGT[A/G]GCCACTGCTGTGGGAACGATGGTAGTTATTCAGGGCGGCATTGGTCCTGCTGGTTTGTCA
3832	CaTSNP6782	AGGCTTGTACGCTGGATTGTCTCCAACTCTTGTTGAGATTATACCTTATGCAGGACTACA[G/A]TTTGGAACCTATGATACATTTAAGCGTTGGGCCTCGGCGTGGAACCATAGTAGAAATTTT
3833	CaTSNP6783	AAGTCTTCTTCCATCTATAGAGGTGGATTCATGTTTCCATGCAGGTCCAATAAGCCATGA[C/T]GCAGCAATTCCACCAATCAAACCTCCTAAGTGTCCCCAGTTGTCAATGCCATTTGATGAT
3834	CaTSNP6784	AGCTGCACTGAATGTGATTGAGTAGAATGAACCTTTATTTAATTTCACTTTTTGTTTAAT[G/A]TAAGCTTCATTACCAAGCCTAACAGCATATGCACCTTCTGGTACAATTAAAAGCATGTCA
3835	CaTSNP6785	AGCCGTTAATGCATTTTTTAATGTAATATTTGCTAGGGAAAGAGAAGTGTCTGTAGGTGG[G/A]ACCACCACTGATAACACGGGACCACTACTAGCTGAAAAGAACCATGATAAGTAATGAAGA
3836	CaTSNP6786	GTTTTTGAGAACAACGGCTTTTTATCCGCCGGAAGTGATTGTAACGAAACGGAGGGAGG[T/C]AGATCGGCGAAAGCGTCATCGACGGGAATAAACATCGTGATTCCGGCGCCGCCTTCACCG

3837	CaTSNP6787	GGCAATGACTTCTGTGTCACCAATTCTGACTTTTGCTGAACCATTTGCCTGAGGAATGAC[T/G]CCAGTTTGAACATAGATAGGTCGATAACTCAATCTTTTTCTACCATCACAGCGAAGATCT
3838	CaTSNP6788	GGAATATACAACAGTAGCTGCTAAAAGCTTAAACCCATCATCAGCCTCCAGAATCCAGAT[C/T]CATATTATTGGAGGTACATTTTTGTGGCAAGATGGGTTTTGTAACAGATAAATCAAGGAG
3839	CaTSNP6789	CATGTTTGAATTAAAAGCAAAATCACAGGAACCATCTTGATAACATAATTATGGTGTCAC[G/C]GTGATTTCAAACATACACTTAGATCTAAGATATTCAACTGATTCAGCGGAATTGAGCACC
3840	CaTSNP6790	GGCAGACGAAGATCAAGTTAAAAATCTTGTCCAATCAACCGTCAAAACTTATGGACAGGT[G/A]GATATTATGTTCAGCAATGCTGGGATATTAAGCCACACTAAACAGACCATAATGGAACTC
3841	CaTSNP6791	AAAGCCTCCTATAAAGATGCCATTTCGAAAAGTGGAGAATTGTAACCAAGTTATAAAAAT[C/T]GGAAAGGACCTTAACTTTTCCTTAGTGAATGTTGCTGGTAACGATATTGTACAAGGGAAT
3842	CaTSNP6792	AATATGGGGATGCAAGGATTGTGGCAAAGTGAAAGCAGGAGGAGCATACACTTTGAACAC[C/T]GCAAGTGCCGTGACTGTACGGAGCACCATCAGGAGGTTGAGGGAACAAACTGAAAGTTAA
3843	CaTSNP6794	TTGTTGAGGTTATATGGTGCTGCACTCATGACCCTTTCCTTGCGCTTCTCCAAAAATCGA[G/A]CCAGTGATGCTTTGCGAGCCTGTGGTATAGCTGATGGAAACATGGTGGTTGCATTGACTA
3844	CaTSNP6795	AACCTTAGCCGTAACTTCCTTCTTCGGCTTCGCGGCTTCAGATAGTTTATACGAAGCCTT[T/G]ATCTTCACCAGCTTCCCTTTCGCGGCTTGATTCTTAAGCTGCAAACTCAGCATCTTCTTG
3845	CaTSNP6797	AGCATGAGGTCCCCAACCATCAATGTCATATGGAGGTGGAAAGAATTGTAAGTAGCAAAA[C/G]GAAGATATTACCGTCCCTATAAGACCTCCAGCAAACACATCTTGCCAATGGTGCCAATAA
3846	CaTSNP6798	TTTGACATGTGAGACCAAACTCGCATTTGTTTTCCGACCTGCAAGTTTCTCCGATCTTGG[A/C]GCTGGACGAATAGGCGAAGAGACAAATAGCAATTAACACGACGAATTGCGTTTGCAAATT
3847	CaTSNP6799	TCTTCTCTTTAAAGAGGTGTCCTTCTGAGGTTGCTGCATGACAGGCATTGAACCAGGAGC[A/G]ACAGGATCATTCATGTAATTTTGAAAATAAAGATCTTCACGCAATTTGGATGTTAGTTCC
3848	CaTSNP6800	GAGTTACTCTGAATGTTACCCTGGGTATCAAGAATATAACCGTGAGGTAGTGGACAGCGA[C/T]GATGAAGCCGACTTATCCAAAATGGATATGGGAGGAAGGGCAAAGGGCCGTCTTCATAGA
3849	CaTSNP6801	GGTAAAATAGTTTAGGATCAAGAGACTAGTACCTAGTTAGAGGCTAGAGCAGAACATTCA[C/T]AGGTTCTGGTAAAGAAACAGTTGTGACAGTAAAAAGATGAAAATACTGAAACAAACAAAG
3850	CaTSNP6802	CACTCTTAGTGCTGTTAACACCTCAGACCCAAAAGATTACATAAAAAGTGTTGTCAAAAA[T/C]ACAATGGATAATGTGATCAAAGCATTTAACATGAGCGACAGACTCACAGTTGAAAATAGC
3851	CaTSNP6803	TAGACAAGAGCTTGGAGATATTAGGTGTCTGGCACTAGAAAAGGTGGCTGAATCACTTGA[A/G]TTACCGTGCAAGTACTACTCCCTTGGATGTCCAGAAATCTTTCCATACTACAGCAAGCTT
3852	CaTSNP6804	AACACCATCTTTGCCACCACTGGCACAGAGAGAACCATCAGGTGAAACAGCAACAGTGTT[G/A]ACATAGCCAGAATGGCCAGCGAGTGTGTTTCTCAGCTTGCAATTAGTCAGATTCCAAACC
3853	CaTSNP6805	TATAGCAGCTACTCCTTCACATCCTGAACTTAGAGCAATCTTTGCTGGCTGTGAAATATC[G/A]GTAATGTTGGGTGTCATCTTGGCCCAAACAGGAACTGTAGCTTTTGCATTTATCCATCCA
3854	CaTSNP6806	AATACCCCGACTTAGAATAGACGGCGGTGTTGGGGTTGCACTTGCGAACCGATAAACAGC[A/T]ATAAAAGCTCCACTGGACTCTACTAGGGTAACAAAGGATGCCATCATCATGGCAAAGGCT
3855	CaTSNP6807	ATATGGAAGTAAGATGGAAGGAGGTGGTGTACATGGAGGAGGCGCTCATGGCGGCCAATC[A/G]CATGGAGGAGGAGCAGTCATACCTATTTATGCAGCCCGGTGCTGCAAATAAAAATCACCAG
3856	CaTSNP6808	TGCCGCTGCTAATGCAATCATCGATGCTCTCTGTACTCATCTCTCTCAACTTTCGTCCGC[T/C]GCGGATTATAGCTCTGTATTCGCCGCTATTCATCGCCGGAGACTCCATTGGATCCCTGTT
3857	CaTSNP6809	TTTCAGTCTTGACTGGGCTAAGGCCAAAAGGACCCTTAAAAATTTGAGGATCAAAACTAG[C/T]CCAACCAACCAAGAGTACAAGATCACGGGATTAAGTGAACAACCTTGCAAAGACCAGCTG
3858	CaTSNP6810	AGAGTCAAAAAAGCTTACTGCTTACCATGAGAGCGGCCATGCAATTGTTGCCCTTAACAC[C/T]GACGGTGCACATCCAATTCACAAGGCAACAATCATGCCACGTGGATCTGCTTTAGGAATG
3859	CaTSNP6811	TGATGAAATCCTTGAAGAAGAAGTGAGAAAGGTTGAAGCAGCACGTTTCATAGCCATAAT[T/C]AAACAAATTGTGACAATAGCAATTAGATGAAAGAAGAAGAAGAAGAAGAAAATGTGAGAA
3860	CaTSNP6812	AACTATTGAGGAAGGAGAGCAAGGACGATTGCCTGTGTTGATTTTGAGCTTGAAGGAAAG[C/T]CATAAGCAGAAAAGAAGGCCTGCAGTTGTTTTCCTTCACAGTACAAACAAGTATAAAGAA
3861	CaTSNP6813	TTGGATGGTTAGTACCCAACTTCATTCCTGTATCGATATCCTCTTTGGTTGCAACATGGG[C/T]ATACAGAGCATAAAATGCTTCATTTATCATAGGCATAAGGATTCTGTTCACTATGAAGCC
3862	CaTSNP6814	GTCACCGATGATGATGTATTTGAAGAGGTACGCGTAAGACATTGTTTTTGCTCACGAGAG[A/G]GAACGATGAGGATGAAGAAGAGGAGAAAAATAAAATGGAAGGAAAAAGGAAAGAAGGAAA
3863	CaTSNP6815	AAAAGTCAGGTATATTGGGCTATCTGAAGCTAGCCCAGATACTATAAGGAGAGCGCATGC[C/T]GTTCATCCAATCACAGCTGTACAAATAGAGTGGTCTCTCTGGACTCGTGACATTGAGGAA
3864	CaTSNP6816	TACATACCCGCACTTTCAGATATTATTCTCTCATAGAATTGGTATGGAGAAATATTCTCA[G/A]TTTAATTATAGTAAAATGGATATTCCATAGATAAATTACCTCCTATCAGATTACCCTGGA
3865	CaTSNP6817	ACACAATAGGAACGTCCTTAGTTGGCTTAAGGCAATTTTCACTGAAACTCAGGAGTATAC[A/G]GGCTTTCTCGGTCCAATACATATATGGCATGGAATGGATGACAAAGTGGTTCCCCCTTCA
3866	CaTSNP6818	TACAACTGAGTGAGTTCCTCCCGCAGCAACCTGCCGAACAACAAGCTTGGGAGCACAACG[T/C]TGAGGGATCATTATATCTCTCCTCAAAGGTCTACCAGTGTCGTCCTTGCGCTCAGGTTCT

3867	CaTSNP6819	GAAGTGTGTTTCTAAATAGATTTGTAATTCGTGACACTGTTTGTCTGTATTAATTCATCC[A/C]AAGATTGGAAACAACTTGTAATATATTTGCGGTTATCAACCAAGATGGGAAACAACCAGT
3868	CaTSNP6820	GCTTCCAAGAAGTCTAATCGAATTGGGGGAATTTGAACCAATGTCTCCGTTAATCCTCAT[G/A]CGTTCCAACGAACCATTCTCATTCTGATTCTGATGATGATACATCATCTCCGATTTCTGA
3869	CaTSNP6821	CACAGTTCTCAGATCTTCAGCACCAAGTCTGTACTTTTCCATACTCTCGTACAAGAGAGC[G/A]CGCTGAACGAGGACAGATACATTGTTTTCATTGTTTCCAAGAATCTTGGTACAATCCGCA
3870	CaTSNP6822	ATTGTTGAATAGTGCTGTGCTTAGTGCTGCTGTGTTTACACCATATCCTTGACTGTATAA[G/A]TTTCCATACCCACAAGCACCACCCATTGTTCCAGAGGCATCACTGCCACCATAGAAAGTA
3871	CaTSNP6823	AGACAAAATGACTGCCAAGGAAAGTACAACATGGCTTGCCATCATCAATCAAGAGGAAGC[G/C]TTAGCACGAGTGCTTTACCCCAATTTCATCCCTCCATTCGCCTCAGTTGGAGGAAGTGGA
3872	CaTSNP6824	TGCGGTAGTTGAGAATCCGGGCTTGTGGAATTGGACATTCACCTTGAAAGATAAAGATGG[C/T]GAGGTGCTTGGTCAAATAGATCGTGATTGGAGGGGTTTTGGCTTTGAGATTCTAACTGAT
3873	CaTSNP6825	TTGTTCAGTCCTATATATTAATTATGGAAGTATTACTGTCATGACTGATCATCATTTGAA[T/C]GAATTTGCTTTTGGAATAGCAAATAGCAAGGTACCATTTTTATGGATAATGAGACCAGAT
3874	CaTSNP6826	GATAAGGCAAGATGCGGTGAACAAAGCTAAAGCATTTGATGAGAAGCATCAGTTGACTGC[T/C]AATGCATCTGCAAAGGTCAGTTCTTTTGATAGGAGAGTTGGACTGACGGAGAAATTGACA
3875	CaTSNP6827	TGTTCCCCGCGGAACCACCGCGACCTCTCTTCGACATTGTTGAAGCTCGATCACACACAA[G/C]AAAAGAAAACCCTAGCCGCTCGACTTCAAGAGATGGCGAAGTCAAAGAATCACACAGCTC
3876	CaTSNP6828	CCCGCAAGACACTGAAGACTCTCAAACTCACAACCGTCAAATCCTTCCCCATTAGGCGAC[A/G]CCCCTTCTGCAATATTCCCATCTGCTCCAGGACGAAGGCAATAGACCCGATCATCACACT
3877	CaTSNP6829	ATTACTAATAATAATAATAATCACAATTACAGTAAAAAATGAAAATCATTGGAGAGCCAT[C/T]GGTTTTTCCTTTATAAAGTTAACATCACCATTCGTTGGAGTTAGCAAATGGTGCTTGGAA
3878	CaTSNP6830	CTTCTCAGAGCTTTTCAAATCCATTGAAGAATATGAGAAGACTTTGGAAATAAAAAGAAC[C/A]GCATGATCCATGTTGCGATCTTGTTTGTGTTGCTATCTAATGTAATAAGTCATTTGCTAA
3879	CaTSNP6831	AAACACCAAACAGGAATCGTGTCAGTCATTGAAAAAGCTATTCTTGAGCCAGAACCAATC[G/A]TACCATAAAATGCATTGTCGAAACAACTTAATACACAAACGTTCTGACCAGAGCAATGTT
3880	CaTSNP6832	TTATGATAAAACAACAGGAAGAAGCAGGGGATTTGGTTTTGTTACTATGTCTTCGGCTGA[A/G]GAAGTTGAAGCGGCTGCTCAGCAGTTCAATGGCTATGAAGTGGATGGAAGGGCATTGAGA
3881	CaTSNP6833	TTCAGTTTCAAAACAACTCGAACTCCATTTCGAACCGGAGCTTCGGAATCAGGCTTGGAG[A/G]CGAAGCAATTTAAATGGAGGATTAGGATTGAGAGTTTGAAGTTATGTTTGGCTTTAACGT
3882	CaTSNP6834	TTCACTAGACTTGTCTCTCGCCAAGAGTCTCTGAATCTGTGTTTCAGGATCAACCCACAC[A/G]ACAATAATGGGCTTCGTGAACCTGTCCATCTTCGCCTCAAATAACAAAGGGATATCGAGA
3883	CaTSNP6835	TGTTGTTGTGTCTGTTGCCCCTGCAATTAATGTCAAGCAAGTGGCTTTAATGACAGTGTC[A/G]AGATGATAACCAGCAAGATCAACACCATCAAGAACAGACAACAACACATCAATGAAATCC
3884	CaTSNP6837	AGACACTTATCGTCATAGATAACAAAGCCAGCGAAATTCTGGCTTGAGCTAAGCCAGCCG[T/C]GGCAAAACCAAATAGAACAACTACCTAAAATGTTTCTGGCTTAGGTAAAACATGAAAATG
3885	CaTSNP6838	AGCCAAATTGTTGGCTGCAACAACAGCTTATGTGAGCAGCAGAGTCAATGTTCTTCATCT[G/A]GTGCCACTTGTCCATATCAAGTTAACTATCTATCAGATGATACTTCAACTACTGGTTTCT
3886	CaTSNP6839	GGATTTGAGCATCTGTCTGACACTGATTAGTTCTTCCCAACTAATCGAAGCGATTTGAGT[G/A]GAAGCTTATCTTCAGCTCCTCGGCGATAAACCTCAACTCATCTCATTTGAGGTAAAACAC
3887	CaTSNP6840	ATTCTTGAAGCCTAACAAAGCTGTGATTGTCCTGCAAGGCCGATATGCGGGAAAAAAGGC[C/T]GTGATTGTTCGTACATTTGATGATGGCACTCGTGAGAAACCGTATGGACACTGTCTTGTT
3888	CaTSNP6841	ACATCCTCAACATTATTAGCCTCTGACATTGCTTACTCTCTTTTTCGTATTGGAAGAAAT[T/G]AAGTATGAAAAATTCGCGATCTTTAGATTGAGTGAAATGAGAAGATGAAACTGATTGCTA
3889	CaTSNP6842	TGTGAAACTTTATGTGTTAAGCCAGCAAAAGATGGATGCTCAACCGGGGTTGCAAGATTA[C/T]GTTGTTCCAATGACCTTGCAATATATATCAATGCTCTGGAGGAATGTTTCCTAAGGATTC
3890	CaTSNP6843	TGCCTCTTCTATCATCAGTTCTGCAGGGGTGACATCTGTGAGAGCTAATCTAGCAGTCTT[G/A]AATTTCTCTTTGAGGAAGAAAGAAGCTTGTTTTTTGAATTCATGGAACGAAGGGGTGCCC
3891	CaTSNP6844	CTCCAATATTTTCTCAATATTATCCACCATGATAGTGCGTATCTCGCCAACTTCACCCCT[C/A]ACACGATTGAGGGTATCAACACTGGGATTACTAGAAAAAAACTCCATTTGCTGATGTAAA
3892	CaTSNP6845	TCTTCCTGAACCACAAATCAATCTATTGTATCCAGGAATTGTGTTGTTTGTTGTTGGAGT[C/T]ATTGGCAACTTCTATCATCATTACCTTTTGTCTAAATTGAGAGGAAAAGGTGAAAAGGAA
3893	CaTSNP6846	GTTTTTGGTCCTGGTTATCTGGCTCTCAATTTGTGGTGGATTTGATTGTTCAAATTAGCA[T/A]TAATTGAAACTTCACAAATCAGGTTTTTATTATAGGAGTTTTATAACTACAGATTGACAG
3894	CaTSNP6847	AGAAGATTCTAGATTAAGGAGAGACCAAAAAAGAAGAGAAGATAGAAAGTCCTCAGGGGG[T/C]CTGACCCGAAACCCGAAATTACGACCCGATTCGAATTCATTCGATGCGGAGTAGGCGTCT
3895	CaTSNP6848	TGCTACTGGTTTGACTATTTACCTTTCTGGAGGTCAAGGACAGGTGGTTGGAGGTGGTGT[G/C]GTTGGCTCGCTTGTGGCTGCCGGACCTGTTATGTTAATGGCGGCTACGTTTTCGAATGCA
3896	CaTSNP6849	GATGCGGAGGAGGAGGAGGATCGGAAGTGGAAGAAAGAGTTGGAGGAAAGAGATGGTAGA[T/G]AAATGGAGAGCGGATCGGAAACTCTACAACCATGAACGGTTAAGGAAGGAGCGAGAAAAG

3897	CaTSNP6851	TAACAACACAGGCACACAAAACATGACAGGTCTTATAAACAACACTGGTTACACCAAAGG[C/A]AATGGTAACGGATCCATCGTCTTTGGTGGATTTGACTCTTCCAATAGACATTATAATTAC
3898	CaTSNP6852	TAAATGGGGACCACAATTGGAGATTCTTTCACACAAATCAACAGGAGCATTTCTGAGTCA[C/T]TGTGGTTGGAATTCTGTTTTGGAGAGTCTTAGTCAAGGTGTGCCAATTATTGGATGGCCA
3899	CaTSNP6854	CCTCCTCAATTTACCCGGGTCGGGTCAAACCGTTTACGGCGAACTCTACTCTGTTTCTCC[A/T]CTCGGACTTTTAAGGATGGATGAACTTGAGGGAACCTCACGCGCTCACTACGAGCGTCTG
3900	CaTSNP6855	TCGTCTCGCTCCAAAAGCTAAGGTTCTTCGAGATGGTAGGTGGAATGAGCAAGATGCTGC[G/T]GTCCTGGTTCCCGGTGACATCATCAGCATCAAACTTGGAGATATTGTTCCAGCAGATGCT
3901	CaTSNP6856	CCGTCCATCATTTCGATATTTAAGCATTTGTTCGAACAGAGAAGAAGGAACAAGCCTTCC[G/A]ACATCTCCTCCACCGGTACTAGGTGGTATTGGTGTTGGTGTTGATGAAGATTTACATTGG
3902	CaTSNP6857	TCCAGGATCTGTTATTTGCTTTTTTTCTGCCCTAAATTGATTAAGTCTGTCCCTCCACAA[G/A]GCCGCGTCTTGATAGCGCTCTTCTACAACAGCTTTCAACATGTTGTGCAAAAGATTGAAC
3903	CaTSNP6858	ATCATTTGGTTCACCCTTTATTAAGTGTGTAACTGTCACTAATCTATCAAATTACACACC[C/T]GACTTTCATCTCAAACTGTAATTGATATGCTGTTTATCCCCCATATGGTGGTTTGACACT
3904	CaTSNP6859	AGTAGTAGGAGCATCTACAGTTGAACCTTCGGAATTTGATAAGATGACGTGCAAGTTCTC[G/A]AAGGATGCCAGATGTAATCCAGACTTTAGTTGACTTGTCAGCTCATTGAAATTTGATTGT
3905	CaTSNP6860	TGCGGCAAACATCGCCTTGTATGCAGTTGACTCCCTTCTCTTTAAGAATATGCGACCAAG[C/G]AGATGATTGGCGGAGGTCGAAGGCTCGCACCTCTTGAGCGCCTCTTCGTATGAGTTCCAA
3906	CaTSNP6861	GCGACGTGCCGACTGGGGTTACTCGAAGCCGGTTGTATTACTGGACGTGGCGTGGAACAC[G/T]GCCTTTGTGGTGGTCGCGGCTGTTATGTTAGGCTGCACGGTCGAAGAGAATCCGAACACG
3907	CaTSNP6862	TGCTTCCGACAACCCTTTTCTTCTCGGCATGACGGGTCGTAAAGTTCTTCAAACACTTCC[T/C]AAAACACTTGATTCTTCTGTTGAATGGGAACCTGTTAGAGATGGTGTTCGTGTGAAGTTG
3908	CaTSNP6863	AGTTAAGCTTGAAGAAGGTCTCAATCCTTTTAAGTCTGATAGATTCGATGCTGAGTTCTA[C/T]GTTCAGTCCAGTTGCTCTCTAAACGACAAGGAAATTAAGCAGTTATGTACATATTTAGTA
3909	CaTSNP6864	TTCTAAGGTTCGAACTCGGAGCTTTAGATCTCCTTGCTCCAGTCTTTGGATAACTGTAGC[A/G]AGCTTGTCAACCCTGTCAGCCTGCCTAAATAAGTTGTAAAATGCTTGAGATTGTCTGTCC
3910	CaTSNP6865	AGCTGAGCTTCGTCATGTTATGACAAACCTAGGCGAGAAGCTGACCGACGAAGAAGTGGA[T/C]GAGATGATTCGTGAGGCTGATGTTGATGGTGATGGTCAGATCAACTATGAGGAGTTTGTC
3911	CaTSNP6866	GTCCAAGAAACTATCAAAATCCATCTCCCGGACATTTCCACCACCACTCTTTCCACGGCG[A/G]ACATGTTCATCGTATACTCTCTCAATAAAAATCTCAGTCAGGGTTCCATCTGCATATTCG
3912	CaTSNP6867	TTCCCTCTTGAAACCACCATAGTTAACTGGGATCTCCTCAATTGGAATGTACTTGATCAA[C/T]GTTTCGGTAACCTTAGCAGGACGAGCAACCACAAATTTGCTCTTTGTTCTTTGTGTTAAG
3913	CaTSNP6868	TTCTCTGGTAGCTCCAAGGTTCCAATTCAAAGGAGCAAACAAAAGGGGCATTCCAGTTTC[G/A]AGGGATCCAGCAGCATTGGTAGCGAAGTACTCCGATCCACTGGTCTACACAGGACCTATA
3914	CaTSNP6869	CTCCTCCGGTGGGTGTTTCTATTTACAACATTCCAAATTCTCCTTCAACTACGGGAAGTG[G/A]TGGAAGTATCCGTGGCCCAACTTATGGAACATCAACTTCTACAAGATTACAATATTCTAT
3915	CaTSNP6870	ATCACTGATCCCTGACAACACCCCTGCAGTAATTGCCTTGGTTCTGAGAGGGTGTTGCTG[A/G]AGCTGTTTGATGTAATTGTTGAGTCCATTCTTTGCTACTGAACCCATCTTGCTTTCACCA
3916	CaTSNP6871	ACCGAAAAATTTATCGACAGGTTTTGAACATGATACAAAGGATGATATTATTCCTTCAAA[T/C]GAGTGGCTCGGAGAAAGTGGTGAGCCGGTGTATCAAGTTGTTCCTGTTGATACGTCATTC
3917	CaTSNP6872	TCAAAAATATCTGATTTGCTAAAGTAACATAACAATACATGTGTTCCTCAAACAAAAGCA[G/A]GTTACTCAGCATCTTTCTTCTTCTTTGGCATTCTGTAACCACCAACAACACAAGCATGGT
3918	CaTSNP6873	CCGTTATCAATGCTTTTATTATCGATCTCTTCTGCACTTCCGCCATGGAAATAGCTTCCG[C/T]CATAGGAATTCCTGTTTACTACTTCTTCACTTCAGGAGCTGCTGTTCTCTCTCTCTATTC
3919	CaTSNP6874	TAGATAGAAGAGTTTGAAAAGCCGCTGCTTCTTTTCCCGAGGGAGCATGTTGAATATCTC[C/T]GTAACATCAACCAGATGCTTTCCTTGTCTGTACAATCTAACATTGTAGTAGGCGTAAGGA
3920	CaTSNP6875	TAGAGGTAGAGGAAAGGGTAAAAAGCTAAATGTTACTAATCAAGAGGATGTTGCAAGTGG[C/T]GAAGATGAAAAGGTTCCTGTACAGAAGAGAAGAGGGAGGCCACAGAAACCGTTAAAGGAA
3921	CaTSNP6876	CTGAACTCGCCGGACGTTTAACCGAAGCCATATCTCCTCTTATTATTTCGATTTTATTTG[A/G]TTTTACAGCAGAATTAAACAAAAGGAAATCAAGCGGAAATAGGAACTGTAGATCTAATTA
3922	CaTSNP6878	CCATGTCTCCACATGCAGAGCCGTTCTCCTCCCCTCTTTCGGTGGACCCCACAAACTCGA[G/A]CTCCACCACAACGTCGAAGTCCCTCCTCTCAAACCCCACGAAGTCCTCGTCCGCTCACGC
3923	CaTSNP6879	TTTTTCCACACATGTTGTTCCATCAGGGCTGTTTTGGGCCAATTGATTTGGGCTGCAGCC[T/A]ATTTGACCAATCCCAAATAGTACCATTTTCCTTGCTCCATAATTATAAAGAGTTTTAAGT
3924	CaTSNP6880	AATCTGGTGATATAAAGCAGCAAGTGCAGCTCCAATGAAAGGTCCAACCCAGAATATCCA[C/T]TGGTCATCCCAAGCATGCTCTCTGTTGTATATTATGGCAGCACCAAGACTCCTTGCTGGG
3925	CaTSNP6881	CTCTCTTCTCCTCCTTCTCTTCTTCTTCACCGTTTCTGCTCACAGTGGCCACAACGACGA[C/A]GATGGTGACTCCGACACCGACTCTGATGTCCAAAGTCCTCACGACCTCCGTTCAAAATCC
3926	CaTSNP6882	TTTCCAATTTACGTATTCACCTAATAGAACAAAAGTTCTTCAATTCAGCATAAGATCTTT[A/G]TCAAATAGTTGCTTCCATAACAACATGGTTCTTATTTTTCCAAATAGGCACCAACACAGA

3927	CaTSNP6883	ACCCAAAGGACCTGATGTAGTTTGCACAGATTGAGTATCAGAAGTAGCACATGGAATCTC[A/T]CTAGATATCAGCTGTCCATTTGCGAGACGGAGATTTTGTTGAGAATCATGTCCAGAAGAT
3928	CaTSNP6884	GGTGGTGGTGGTGGTGGCGGTGTTAGGTGATGGACGGGATTGGGGGCGTCCACCTTCGAG[G/A]CCGAATTCTTCGTCGTCGAGGAGAAGCTCGTCGGATTGGAAAGCTTGTGAGAGAGAGATG
3929	CaTSNP6885	ACGACCTCGGATGGAAACATTTCCAGTGAATGGACACTTCTTGTCAATGTAGGTTCCTTC[C/G]GTTGCTTCTCTGGGAGTCTTAAACCCAAGACCAATAGATTTCCAGAAGCGGTTTCCACCT
3930	CaTSNP6886	CCCTCCTAGTCTTTCTGCATCAGTATCTCCAACGAGAACATCTAGACCCATGTCTCCAAG[G/A]CGACATTCCATTTCCTTTTCAACCTCAAGAGGCATGCATGATGATAGGTCTTCCGTGTTT
3931	CaTSNP6887	AATAGATTTGACCGGAGCACCAAAACAGGTTATAAGCAAATGTGAGCGCTCATAATGCTC[A/T]TGATATAGTGGAGAGCCTTCAGATCTTCTGTTCGAGAGTCACATCAAGTATACCAATAGA
3932	CaTSNP6888	TTCCAACTTGAATGAAAGACTTCAAAAATGGTGCTGCTACCCCCAGACTTGAGCTGTCAT[C/T]CAAATTTATCTTTTGAGAAAGTTCTCCATTTGTAATTGAGTTCCATGAACTTCTTGACGA
3933	CaTSNP6889	CTCTAGCCCCATTCGTCGCAGCCTTCAGCCGTATCAGTTAGCACAGGGGAGCTGTCATTC[G/A]AGTAATGTCATGTCATCCGGGAATGGAACCCGGAACAACGAAATGACATATCCTAGTGGC
3934	CaTSNP6890	CCGGGTTTGCAAATCAATGACGCGCTAAGAATGCAGATGGAGGTTCAGAAACGTCTGCAC[A/G]AACAACTTGAGGTTCAGAAGCAGTTACAAATGAGAATTGAAGCTCAGGGTAAATACTTGC
3935	CaTSNP6891	TTATTCCACCATGGTTTGGGTACTTTCTGTGAGCCAACAAAGGCCACCTTCTATCTCTTA[T/C]GAACCCTTATCGCTTGCATCGCCTTCTTCTTCGTTATTTCTCGCCTTGAATGCGCTTGGA
3936	CaTSNP6892	TCATGTGGCCGAGGAACTTTTAATCGAAGCTAAAAACAAAACAGTGATAAGGGATCCTGA[G/T]GTTTATTTAAAGCTTGTGCTAATGTATGTTGAAGAAGGTTTATTGGAGAAGACTTTGGAA
3937	CaTSNP6893	AATCGATTGATACTGAAACGTTCCTTGACTTCCTTAAAACAAACATCCCTGGCATAGAGA[G/A]TCAGATTGACCTAGTACTGTGGACAGAAGGTACTGGCATCCCATCTGATGCTTTTGAGCC
3938	CaTSNP6894	GAGAATTGCCAAGGCAAAATCAGAGGCAGCAGAGTATCAAAAACTCCTTGCATCCAGATT[G/A]AAGGAGCAGAGGGAACGCCGAAGTGAGAGCTTGGCAAAGAAGCGGTCTAGACTTTCCAGT
3939	CaTSNP6895	TCTTCAATTCCTTCATAAATAATGACTCTGGAGGCTCTTCTCCATTGTTTTGGTTCTTCT[G/T]GGTATCTTGAGAACTACAACAGACCCTATTAGGATTGAAGGACTTGGAAGTAGTGAAAGT
3940	CaTSNP6896	TGAATGTGAGATGACTAGAAATCTTCCGGAGGGCTTAAGAAGAGACATCAAATACCATCT[A/C]TGTTTAGATTTGGTTAGACAGGTGCCATTGTTTCAACATATGGACGAGCTGGTTCTAGAG
3941	CaTSNP6897	TCCATTTCCCATTCACCGAAATGGCATCGGCAGCCGCAAGACCCCTTGTCACTGTCCAAA[T/C]CCTAGAAGGCGATATGGCCACCGATTCACCAACAACTCTCCCCATCCCTGACGTCATGCG
3942	CaTSNP6898	ATCAGTGGAAGAAGGTGGCGGCGGTGATGAAAGAGGGCGGTAGATTGAGATCTGAGAGAG[C/G]GAGTGGAGACGGTGAGATCTGGCGGTGATTTTAGAGTCGCGGATCCGAGCGAGGGCGCCT
3943	CaTSNP6899	ACAGAAAAGTAGAAAACCTTTATTCTCTTCAACAACTACAATGGCTTCAATCTATAGCTG[T/C]GTAGAATGCGGCACCAACCTCAATCTCAACTCAACCCACGCTTACCCACCCGATTTCTAC
3944	CaTSNP6900	TTGACCTCTAAGACGACCAGTCCTCTTGGCAGCAATGAGACCAACCTTTTGTCCAGGAGG[T/A]GCATCACGTCTGACAGTACTTGCATGACCAATATGCTGGTGATTACCTCCTCCATGAGGA
3945	CaTSNP6901	CAACCGTCTTGTTTCTCAGGTGATTTCATCCTTGACTGCTTCTTTGAGGTTTGATGGGGC[C/T]CTCAATGTTGATGTCACTGAATTCCAGACCAACTTGGTCCCATATCCAAGAATCCATTTC
3946	CaTSNP6902	AACCCTTTCTCGCTGGCCGTGCCCTTCGCCTCAACAACCTCAAGCTTTTTTCTAGAACCA[A/C]CAGGTCTTATACTTGCAAGGCCATCTATTACCCTGAGGTTACTGTCAAGGATGAAGGTCA
3947	CaTSNP6903	CAATAGGTCTTTATTGTGTGGTGTGGGGTAAAGCCAAAGACAATTCAAACACTACGCCAG[T/C]ATCACCTACAACAATGAAGCAAATAGAGACACAACACCTTCCAATTACTGCATCTGGTCA
3948	CaTSNP6904	CAAACTCCTTTGTTTCCCATGATGTCTATTAAGGCTTCCAACATTGTTTTTAGAAGCAAC[A/G]TGCCATTTCCATTCCCTATAAGCAACTGAACCAATAACTCTACCCCATTTGTTTTTCATG
3949	CaTSNP6905	TCTTCCCCAAGAATATACATTGTTTCTTTCAGTAACAGCAATTGTGTGTCTCCACCCACA[T/C]GAGATCTGAACAATTTTCTGATCATGGGGAAACTTCACTTGCGCAGGAGAGTTGCGATCA
3950	CaTSNP6906	AAATCATCATTGGGTTTCTGATATCAAAATTTCCTCTTGTCCAAGCAAATATCGCCAAAA[C/T]GAGATCAGCATAAATCATCGAGTCTCATTTGAATTATCGGCCCAAAAGGACACATTGTCA
3951	CaTSNP6907	GCCGGTGTTGACGTTATTGTACCCTTTGTATGCATCGGTGGTAGCGATAGAGAGTCCATC[G/T]AAGTTGGATGATGAGCAGTGGCTAGCTTATTGGATAATCTATTCCTTCCTCACCCTTGGG
3952	CaTSNP6908	AAGAACAAGAGTTAACTAATTAACTGAGAGCAGTGAATCCACCACCCTTCATCATTTGCA[T/C]AAACTCCTTATAATCAACCAATCCATCTCCATCAACATCAACCTTCCCAATCATCTTCTT
3953	CaTSNP6909	TCTGTATTTCCCATGCGGGATCATTAGAGGAGCTCTTTCTAACTTGGGAATTCCTTGTGC[A/C]GTTTCTGCAGACATATCAAGCCTCCCAGCATGTTCATTTGTGGTCCGTATAAAAGCCTGA
3954	CaTSNP6910	ACCTGATCAGCAATTGGTTAAGATTGTTCACGAAGAACTTGTAAATCTAATGGGTGGAGA[T/G]GTCTCTGAATTGGCTTTTGCCAAAACTGGGCCCACTGTCATTTTGTTGGCTGGACTCCAG
3955	CaTSNP6911	GCAACTGCTACACCTGTAGTACCCTCTGGGATAAGGGGAACCTTTGATGGGTTTCTGTCC[A/G]TACTTTCTCCCATGCCCATGAATCAGAGGGTGGGGTATTGCTCTCTCCTTTTAGCCTTGT
3956	CaTSNP6912	TGGTTCGCAATGCTATTTTGCTGATTGTGCATTGCTTGTCAAAAAGCCCCCAAATATTGG[C/T]TGGCTGGAGCTACACTCAAGCAAATTTTTTATTGAATTAAAACATTTTTATCACTATTTG

3957	CaTSNP6913	TAGAACGCAAGCTTCTGATAGTGAAGGCTCTTGGGGACTTAACTTTACTTCACAAGGGAA[T/C]CAAGTTAATATTTCTAGTGGAGTTGTTACTACTTCCATTAACAATGCTTTGAGACAGCAA
3958	CaTSNP6914	AGGTTTTGCTTTGTGACTCAGGTGACATGGATGGAATTTGCAGGGAAAATGGTATGGAGG[C/A]AGTTAGGAGGGTAATGGAAGGGAGTGGCATGCAATAGATTTGGTTGACTATGCTTTATTT
3959	CaTSNP6915	TGCGTCCTCTTGCCAGCATGAAGTAAGAATTTCGGCCAATTCCTCAGGAAGATGGTCCGC[A/G]CTCGGCCTAACATTCTTAAAGGCTGCAGCATATGCTGCTTGAAGGTTTGACATGCCTTCA
3960	CaTSNP6916	TTTCAACCATTTTAGCATGGGAGAACAGTAAGAAAGCTGCTACAGAAGCTGAATTGAGAA[A/G]GCTTGAGGAAAAACTGGAGAAGAAAAAGGGAGAATATGCAGAGAAAATGAAAAACAAAAT
3961	CaTSNP6917	TTTTCCTGAGACTCATTCAGTTTCTGTTAATCTTGGTCAGATTACTCTTTCCAACAATCA[A/G]CTTTCTGGAGTTTTACCTCCTTCTATAGGTAACTTCACCAGCATGCAGAAGTTTCTTCTT
3962	CaTSNP6918	GGGTAAAAGATCTTCAATGGAGGATTTCTTTGAGACCAAAATATCAGAGGTTGATGGTCA[A/G]ATGGTTGCCTTTTTTGGTGTTTTTGATGGCCATGGAGGTTCGCGGACTGCAGAATACCTG
3963	CaTSNP6919	AGGTCCAGGCCTGGAAATTGCATTTATAGGTAACTGACCACCAAAATTGGCATTACGCCC[A/G]TTTCGTGCAAGATCAGGGCCACCACCAGGATGCTGCATGACAGTAGGATCACCTAGAGAA
3964	CaTSNP6920	ACTAGTCCCACCAAACTTATGTGAAGCCTACGAACTTTGTGAGACTAATTTGAATTTCAA[C/T]AAACGGAACATATGTTTGAGATGTGCTCCTCGTCGATCCCACTGATTATCTTTGGGATAA
3965	CaTSNP6921	TGAACATGATCGACTTATAACCAAAGTACAAGGTGATGGAGTCATCGTTGCAACCCCTAC[G/C]GGAAGTACTGCCTATTCTACAGCTGCCGGAGGTTCCATGGTCCATCCAAACGTCCCCTGC
3966	CaTSNP6922	CATAGGTCAGTTCACCCTTCATTGTATAGTCACAACCATAAGTGTAGTGAATTATGTATG[C/T]CTTTCCAAGTTCTTTGTCCCATGGAGGCTGAATCATGAAGTCCTTGTATAATATGTTAAT
3967	CaTSNP6923	CCGGCCGGCAAGGTTATTGTCACAATACTGGTCAGTCATCTGGAGAAACTCCGCCGCACA[G/T]CGCAAAACGGCGACGTTATGGACCGTTATCTCGAAGTTAACGCCGTAACAGAACTTAGCT
3968	CaTSNP6924	AGGCATTTTCCTTGCAGCCTTCCGAGTAGCTGATCTTGGAACATCTTTAAGAATTTTGCA[C/A]GCCATTCGCTGCGAAACATAATGGACAGCAATTCTTTCTAAAATCCTTCTAGTGGTCACA
3969	CaTSNP6925	TGGATTTCTTGTTGAAGATGCGACGCTCTGCAAACAAGAAAACGGGTCAATCCAATTCCA[A/T]TCCTTCCGTCAATTCCTCCGCAGCGGATCTCTTCCGCTCCGCTTCTAGTAAAGCAAGCTC
3970	CaTSNP6926	TCATATTGCTGCTAAGCTTGAATACTTGCAATCATGCTGCAGTGTCAAAGATAGGATAGC[A/G]TTGAGCATGATTCGAGATGCTGAGAGTAAAGGCCTCATTACACCTGGAAAGACTGTTCTT
3971	CaTSNP6927	CTTTCGTTGCTGCGGGTTATTCACGGTTCGACCCTTCTGGTTATGATAGCTTCCGGTCGG[C/T]GTCAACAAATAAGCCGAAGCTTCTTCAGTGCTCTCGGCTTCCCTCGCCTCAGATATGTTC
3972	CaTSNP6928	GATTTGCTCCAAGCGGTGGAGGCTCACCAAATTTCAATATGTAAGCGACTTCATGAACAG[A/G]ACGTAATTGCAGAATAACTTGAGCATCCAAGTAAGCGTCGCAAGAGAAGCACCATACTGA
3973	CaTSNP6929	GAAGAGGAAAAGCGAGAGTAGCAGTGGAAGTGAGAAGAAAGGTTATGGGGGTGTGGATAG[T/C]AATAGTGATGGAAACACTGAAACAGAGAGAAGTAAATTAGTGTTTTTCGATAGAAGGAGT
3974	CaTSNP6930	ACCTTCTAGGCCAAGCATCTTTGGGATGCTTGGACCACACATGTCAATGTCAAGAAGACC[C/T]ACTTGAAAATCCCTTGCAGCTAAAGCAAAAGCCAGCTGGGCAGAAAATGTGCTCTTGCCA
3975	CaTSNP6931	AGCCTCGTCTTCGCGGATGCATTGGTTCTCTACAAGCACGTGGCTTGATCAATGGCTTCA[G/A]AGATTATGCACTGACTAGCGCTCTCAGGGACCGCAGGTTTCCACCGATACAGGCTAAAGA
3976	CaTSNP6932	ACCCAATTTTTCTTCTCCCTCTTTCTTGTTCTCCATCGCGGCGTTGCTCCTTTCGATGTT[C/T]GCAAATCTCATATTATTTATTACCTAATTCAAACATAGTTCAATCTCTCTATCTCGTTCT
3977	CaTSNP6933	AGAGCATTCATGCTTTCTTGACTGCCATCTACTGCTACTACTACAAGGTTGAGATTTCCG[G/A]ATTCTGACATCTCTGATAGATTGGATTTTGGATGATTCACGACTCTCTGTCTTTCTATCT
3978	CaTSNP6934	TTGGGGTGTTGCTGTACTTGTTGGTGTACCAAGCAAAGATGATGCATTCAAAACTCATCC[T/A]GCGAATTTCTTGCATGAGAGGACTCTTAAGGGTACCTTCTATGGCAACTTCAAACCTCGC
3979	CaTSNP6935	CATAAAGAATCCTATCATTGCGGCTCGACCATTAAGTAGTTCAGCTTCAGGGAGATGGAA[T/C]CTTTTTATCCATGCCCACCAAGGTACAATGGAGGTGTCAAACACTACAGGACTGTTGTTG
3980	CaTSNP6936	GTTGGCAAGAGAACCGGAAGAGGTCAATGCAGTGAAGAAGTTACCGTCCTTCGAAATCAT[T/C]TGTCAAGATATCAACAGAATTCTAACACCCACAGTAGAGCACGGTGAAGTTTTGGGGTCC
3981	CaTSNP6937	CTTGAGGGCTTTGAGCTCACTATGGTCTAATCGACCCCGCGAACCTGCTTAGTCTTGCAT[G/C]AAGATTACAATAATGTAAACTTTGTAATATGAAAGTGATGGGACGAGTATGGTCATTTTC
3982	CaTSNP6938	TACTTTGTCAAAGTTTTCAAGTTCAACTGCCATATCCAGTGCTGATCTTTTTGGTGACTC[T/C]GGAGATTCCTCCATTGATCTTGCTGCTAGCGATCTCATCAATCGATTATCTTTTCAGGCA
3983	CaTSNP6939	CTGACTGAGATTCTTTTTTCTCACTCCACAATATTGAACTCTAGCAAATATATATATGTG[C/T]CATGGTTAATCAAAGTCCTCCAAATTATATTCATTTGATTATTTCTTATATATATGTCAA
3984	CaTSNP6940	TTTTGTAGAGATTGTCTTCCAAAACGACGAAGACATCCTTCAGAGCTATCATCTTGATGG[C/T]TACTCTTTCTTTGTCGTTGGTATGGATGGAGGACAATGGAGCCCTGCTAGCAGGAACCAG
3985	CaTSNP6941	AGCTAGGACACATTCAATTGTATTATTGAAGAAGCACACATCATCATAATAACAAGAAAC[A/G]AAGAGAAACTTATTTTCACAATCTGATGTACTTCTTCTGCTCATACTTGTTTACATTTAA
3986	CaTSNP6942	AATTGACTGGAGCTATGGAGAGCCAACAGAAATGTTAACACAAGGTGTAGACAATGACAA[C/T]GACAACACAGGCGAAAGCAGCGGCGCGTACATCATTCGAATACCTTTCGGTCCGCGTGAC

3987	CaTSNP6943	AAAGTGGAGCAAGCATAGAAAGCTAATCAACTCTGCTTTCCATTTAGAAAAATTGAAGAT[T/A]ATGACACCAAGTTTCTTCACAAGTTGCAATGATTTGATAAGCGAGTGGGAAGAAATATTG
3988	CaTSNP6944	ATGAGGCGGCTGATACCGGAGTTAACCACACCGGCGTCGGCGATAGAGGCGAAAAATCAA[C/G]AGAAGGAGGAATGAGTTCTGGCAAAAAGGCAGGGATAGCGGTTGGAGTAATCGCAGGAGT
3989	CaTSNP6945	AAAAGCTGCACTTGAGTTGTTGTTACCTTATCAAAGATCAGATTTTGAAGGAATTTTTCG[G/T]GCAATGGATGGTCTCCCAGTAACGATTCGATTGTTAGATCCTCCACTTCATGAATTTCTT
3990	CaTSNP6946	CATCATTACGGCGGCTCCGATTAGGTATCTGAGTGGACTTGGAGGCTCCACTTCTATTAA[G/A]CTTCTAATAGGCATGTCGGTGGTGATTATTGTGATGACGATCGCTTCCAATGTTTAGTTG
3991	CaTSNP6947	ATGGGTCGCCAATGTTGGAGATTCACGAGCAGTACTGTCAAGGGGAGGGGAAGCTTTACA[G/A]ATGACTACTGATCATGAACCTAATACGGAACGGGGCAGCATTGAGAATAAAGGCGGCTTT
3992	CaTSNP6948	TGTTTCTTCCTTCCTTCTAATTACAGTGAAATATTGTCTTCAATCGAGACGGCGCAATAG[G/A]TACTCAACCTGAACCTCCTTAGTAAGAGGTATTACCCATTTCTCATACAATTGAGAAGCC
3993	CaTSNP6949	ATTCAACAACTTAGCAATCCCCTCATCCTCAAAAGACTCAACCTCCATAACATGACACCA[G/A]TGACAAGTGCTGTACCCGATTGATAAGAAGATAGGTGCATCTCGGCGGCGTGCTTCAGCG
3994	CaTSNP6950	CTCATTGAAAAGGTAATCTCTGATTCTAAGAAGTTCATTTGCTTCATTTCGAATGTCATC[C/A]GCCCGACCACGAGCAGCTCCTGCTGGAGATTGGAGTGCAATTCTTGCAAGAGGCATTGCA
3995	CaTSNP6951	TCGAATTTTTTTGCACAAGACTCAAGTAAGCCTCTCGAGTATATCCTCGCCTCATTCTCT[G/C]AAGCATTGTGCTACTTCCTGTTTGTGCAGGTAAATGTATCAATTTGCATATATTATGTCT
3996	CaTSNP6952	AAGCTAATCTTGATGCTCAAGTTGTAAATTTGTTTCTTAATTCCTTCAAAAGGAATTGTT[G/A]TATGTTGTAACAAATATGCTGCTACCTGTATGAAATACAAATTTGAGCTCTATCTCAATG
3997	CaTSNP6953	ACGCAGTGCTTGAACAGCCTCATTGTGCATGTTAAGACAAAAGGCAATCCGAGAATTAAA[T/G]GCCAACTGAGGCTCATTTGTAGAGTAGATATCTCCAGTTTCCTTAGACACCATCCATTTA
3998	CaTSNP6954	TTCCCATCCTGACGGTAACGGTAACCCTGCCCTAATTCCAATCATCTTGACGGAAGGATA[C/T]GAAACGAGTTGAAGAGGACCAACGACAAGGAAGAACATACGCATAACATCTTTGTAACAT
3999	CaTSNP6955	GTGGTGCTGGATGTTACAACTGCGGTGATACTGGTCACATCGCTAGGGAATGCAACCGGA[G/A]TAATAACTCGGGCGGTGGTGGAGGTGGAGGCGGCGCTTGTTATACTTGCGGCGCGTTCGG
4000	CaTSNP6956	TATTAACTTTCAGTTGGGTACTCCATTCAAACCATTTGACCAGCTTCTTGGAGTTTTTCC[G/T]GCTGCAAGCTCTCATGCTCTGCCTGAGCCATATAGGAAACTTATGACTGATCCAGACTCA
4001	CaTSNP6958	TAATCTTCTAGCCGAAGATTCTGTTTCTTCGTTTAAATTAAATTCAGATGCCATGGTTGA[T/C]GATCCCTCATCCGAGAAACCTCATGTTTGCAACCAAAATGGTGAACTTGAATACTATGGA
4002	CaTSNP6959	TGATATCTTTCTGACTAGAGATTGTTTAGCATATTCCAAGATAAAGCCAGCTGTGAATCA[A/G]ATTGAAACTCATCCGTACTTCCAGCGTGACTCTCTGGTCAAATTTTGTCAGAAGCACGGG
4003	CaTSNP6960	TTCCAACGGTTCGGTTCGGTTCAACTTGCCTGAAAATTGAAGCTCTGTGAAAACAATGAA[C/T]CGTGCAATTCGAATCGGAGTTCGCCGCTGCCGAGTCACGAACTCAACTCGGATCGCGTTG
4004	CaTSNP6961	GAAATAATATAGTAGATATAGTCCAAACATCAACTCCGGCGTTGATGTGAAGGAAAATAT[G/A]GACATAATCAACTTCCAAAAGCGAAGCTTTCCGAAAATATCCTGATAAGACAACCCCAGA
4005	CaTSNP6962	TTGATAATAATAGTCTTCTATCTCATCATAGATATCTTCATGAACCACATGCTCTTCAAC[A/G]GTTAAAGGCTTGAACCATTGGGCCCGAAGTAGCAAGCAAACACTTTCCTCAAACATGTAA
4006	CaTSNP6963	GGATGGTAAGAGGTTTTGCTGACTTAATGGATGGAGGTGGTAGAAGCCAGGCTATGGCAA[T/C]TCAGCCTCAATCTAATCCCTACCTCAATTCTCCTCTCCCTGTTCAAGCACCAGCAGCTAG
4007	CaTSNP6964	GACAAAAGATAACAAAGCCATTTGTGATGATTTTGGTCTTCTCTTTGTTGATAAGCATAG[C/G]TGGAGGCTCTTTGCTTGGATGGTGGCTTCACAAATATCACTCAACAAATAGAAACCTTTG
4008	CaTSNP6965	TTTTTTTGTAGAAGGAGATGGTTCTCCTTCCGGAGGAAAATCGGAGAGAAAAGCCAGCAT[T/C]CGGCGAATACTTCCGGATCGCGCCGGCTTGAAGCTATTTGATCTCTTCAAGTTCTTGGAG
4009	CaTSNP6967	TGGCCATATAAGCAAGACATATCAATATTAATTCCTAAGCTTTGGCCATATATTATTCTC[A/G]TAATAATTGTACTACTACGTGTAACGTAGTATATTCTCTTCTCTCAATGTAACCCTCAGT
4010	CaTSNP6968	GAAACTAGTGAATCCGATTTGTCAAGCAATGAAAGTGATGATATTCACAGGAAGAAAAGC[C/T]ACAGAAGGCACCATAAGCATCACAGGCGATCACATAATGCAGAGGTGAGGTCATCTGATT
4011	CaTSNP6970	CACCAACATCAACACCAGGATCAAGATTATCATCACTATCGCCATCTGCACCACCGCCAA[C/G]AACAGGAAGCTTACACTCACTTGTACCACCGCCAAGAACAGGAACACAGCCACCACCAGA
4012	CaTSNP6971	GGAGGGAAATGTAAGAATCGAACATATAGACATAATTTTATGCGTTTTTTCTTAAAAAAC[T/A]CTGCATAGAAGTTTGTTGAGTGCTTGTGTTTAACTGTTATGTTATTCTATCTAATTAGTT
4013	CaTSNP6972	AGTTTCCGTGGTTCGCTTAAATCAGTGAGATTGTGGACGAAATTTTTAGGGTTTGGAATC[G/A]GTGAAAGAGATCTATTTTGCGACAGTGAAGTTTTTGTACTTTCTTGCTTGTTTCTGATAC
4014	CaTSNP6973	GGGGGTCCACCACCAGGAGGTCCTCCATATTCTTTCTAGTTCTAGTACCAGGAAGGATTA[T/G]TATTATAAGTTTGTTGCCTGCTACTAACCTGGATCATCATTAAAGTTGAAGCTTCCCTAC
4015	CaTSNP6974	TGCTTTTGCAGCTTTGCTTAAACCTTGAAACCATGGCTTCTCGTTATCATCGGTTGGGAG[A/G]TAGAATTTCGCTGGAATGAGTTCGATTAATTTGTCGAAAAATAGGGTATGGTTATGGATT
4016	CaTSNP6975	AATCTATATCATATATAAAAACATAATCATCCCAGAGTGTCTTTCTTAAGTTGTTGTATG[A/T]TGATTGATGAGCATAGGAACAAGAGAAGTTGATCATCTTCTGAAGCTGGCGGGGTTTGAA

4017	CaTSNP6976	ATCAGTATCCTGAAGAGTTAGGAATGTGGTAGCTACACCTGTCTTTCCTGCACGGCCAGT[T/A]CGCCCAATACGGTGAGTGTACATTTCAATATTCCCAGGCATATCAAAGTTGATAACGTGA
4018	CaTSNP6977	GATGACGAGGAAGAGGAACAGGAACACGCATACGAATAAAATAATTACTCAGTTGATTGT[T/C]CCTGCTTATTGCATTATGCTATATATGATGCGTAAGTGTGTTCAGTAGTACTCCTCTATC
4019	CaTSNP6978	AACAGTGCTTTGTAGTAGTCATTGACCTTTCTTCCTACATTTTTATTTCACGGTTAAATG[T/C]TGATTAGTGGTAAGTCCCTACTATTGGTGGTTGTGGTTGAATGGTGTCTTCTTAGTTTGT
4020	CaTSNP6979	TGCTGATGCAGATGATGATTCACAGCCCAAAAAGAAGGTCCGTTCAGGGATTCCTGCTTC[A/G]CAATGGATTTCAGTTTACAATGCTAGACTACCGATGAAACAAAGGTATCATTACAATGTG
4021	CaTSNP6980	CCCGGCTGTCGGAAATTCCGTTACGGACTTCGTAGCTTGCGCTTTGCACGAAAACGGCGT[T/C]GCATTACGAACGACGAAACGGTTAAACGAGAGAGTTGGAAACGAGGAGATTGCATACCCA
4022	CaTSNP6981	AAAGACGTGAATCTCAACCATTATGGTGTCGATGGGCCAAAGAGAGATGAATTGTTAGAT[C/T]TTGGAGAACAAGGGAGGGTGGAGCTTGATGAGGAAGATCCAGAGGAAAATCCAGAAGAAG
4023	CaTSNP6982	GAATATCCTGATAGGGACAGGGAGCGGTCACGCTCCCGGGACCGTGAAAGGGAAAGGGAC[C/T]GGGAACGTGATAGGGACAGATATTATGACCGAGATAGGAACAAGGACCGAGATGCTGAAA
4024	CaTSNP6983	TCCTGCTGTAAATAGATTCAAGAGTAGTGCCTTGATGTTTGTGAGTGTTAATCTCTCTCC[A/G]TCAGAATTTTGTGTACTTTGAGCAATAAGAGTGTCTAAGAAATCAGGTTTATCCCGATTC
4025	CaTSNP6984	TGAAGTTGAGTTGAAGGATGATGACAAGGAAGATACATATGCACCGGATGAAGCTGAGAC[G/A]TCAAACGCTTTGTTCATGTCTGGTCACTTTGCCTTTCCACCCAGTCAGCATGAAAACTTG
4026	CaTSNP6985	TCATATATGGCATCTTATTATTATTATAAGAGAATAAAATGATTAGTATGATGTCATGAT[T/C]AATGTTTTAGTGTTTTATGATTTTTTGCCAAGAAAAAAGATCAGTACGTACTAGCTTGTT
4027	CaTSNP6986	TATGTTGCACACATCAAATTCACGGTCTTGTTAATGCCAAATGGATCGGATCGCATCACA[A/T]CTCATTCACTCCAGGAATTGCAGCCTACTAAAACTGTAGATGATCCCGAAATTAAGGCCT
4028	CaTSNP6988	CTTCAATTTTTTGTCTACCTTTCTCTAATTCTTCAAGCACTTGTTCTACTTTCATCCCAC[T/C]TTTCTCGTTGAGTTGAAATAATAGCCATTTGAGCTCTTCTTTTGTTAGCACAATTTTCAT
4029	CaTSNP6989	ATTAAATTGAAGAAAGAACACAAGGAATCCTACAGATGTTTAGCTACCTTCAGGAAGCCA[A/G]CCGTATCTGCGTTCAGAAACTACTAACATTGAAGTCATCAACGCAGAAGCAGTTGCCGTA
4030	CaTSNP6990	TATAGCTTTGGCAACCAACTTATCCAAGTTGTCTACTCGGTACGCATCTAAGCGATCCAC[G/A]TAAAGTAGAACATCTATGGTCTTGTCCAGAAGGAAACTTTTTATTATTCCAAGCGCCATA
4031	CaTSNP6991	CTTCTGTGCTGCAAAAGGGATTTACCCAGATATAGAACTGATTCCAATCGGGTACTCGAA[T/C]GAAGCTCTTGATAGGGTCATAAATAAGGACGTAAAATATCGGTTTGTAATAGATATTGAA
4032	CaTSNP6992	TAATTTGTTAGGTGGCCATGCCATTTATTTAGGACCTGATGATATACAGATGGGTAAGCG[G/A]GAGGAGACTCGTGATGTTGCTCGTGTGTTGTCTCGATATAATGATATCATTATGGCTCGT
4033	CaTSNP6993	TGGACAAGCCAACTATGCTGCTGCAAAAGCAGGAGTAATTGGCCTGACAAAAACTGTTGC[T/A]AAGGAATATTCTAGCAGAGGCATCACTGTTAATGCCGTTGCTCCAGGATTTATTGCATCT
4034	CaTSNP6994	CTCAAGAAGCTTGAATGGAACAACCTTATCATCAATTTCAATATCGAATTGGTATTCTAC[T/G]GAATGTCCAACCTTAGCCTCTCTCAGATCAAACCCTGTAATCTTGGTATCATCTTCAGCC
4035	CaTSNP6995	TGTTCCCGTTACAAGTACCCTTCTAGCTCCCAAATCATAAAGTCTCCTGAGAACTTTGCG[G/A]TACTCAGAAATGATATATCGAACATAATCTGGGAGAGAATATTGGCGAGATCTTGCTGAA
4036	CaTSNP6996	TTCTGTTGTATAATTTGCACAAGTAACGATATTGAAGAGAAAGCCACGTGGGATTTGATA[C/T]CCACCTTCTCCAGCTGGTCCGCGAAGATTCCTCAGTATAATATGGCAATAGAAGTTTGAA
4037	CaTSNP6997	GCTGCCGCTGCTAAGGCGAAACCTGCAGCCAAGGCAAAGGCTGTGAAATCGCCTGCTAAG[T/G]CTTCTAGAACCTCGACGAGAACGTCTCCTGGGAGGAGAGCTGCTGCTCCGAAACCGGTGG
4038	CaTSNP6998	ATCTGATGTCTTCTTTTCATTAGCTTCGATTACATCAGGTTTGGCACCACTCTTCAACCC[G/A]TTCTCAGGTTTCCCATTGGCAGCATTAATTGGAATATCTTCACCATCAAAATTATCAGAA
4039	CaTSNP6999	TATTTCATTCTTTGCATGCATATACTGCATGCTATGCAATTTTGCAGGAAATAGAACCTA[C/T]GGAGGTGTATTTCTTGAATAATGATATCAGGAAGTGTCATAATCTGATTAAACTTATACC
4040	CaTSNP7000	CACAATGGGAATTAAGCCTCAGGCCTTTAGCAAAGTCAGAGATCCAGAAGTGAAGGCATT[C/T]ATTGAGAAATGCATAGCACAGCCAAGGGCAAGACCTTCAGCCACTGATCTTCTTAAGGAT
4041	CaTSNP7001	GGACAAACTTGTCATTGTTGACTTTTTCTCTCCTGGTTGTGGTGGCTGCAGAGCCCTTCA[T/C]CCTAAGATATGTCAAATGGCAGAGATGAATCCTGATGTTGAGTTCCTTCAAGTGAACTAT
4042	CaTSNP7002	AGCCATAATCAGCCTTCAGCAAAATGAATATGAAGTTGCCCAAGCTAGCGGTGGAAGTGG[T/C]CGTGAGCAAGCCATTCACGATGAATTGTATCCCGGCGAGGGAATGCATGATGACAGACCC
4043	CaTSNP7003	TAGAACATGTATCTGATGCGGATGATATTGGTAATTGTCATGTGGGGGAGGGGCGCTTAT[G/A]TAGAGATTTTGAATGGCAACAAACCACTCTAAAAATGAAGAGTTCCAAAAAGTGTTCAAC
4044	CaTSNP7004	ATCTTGCTTGCAGCATGCCACTTAGGGTTGATATTTTCTTAGCAAATAAATAAATTGGAT[C/T]TGGCTGAGTTGAGTTTTTGAAGTGGTGTCCGGGATGATATCTTTGATCCTCTCTGTTATT
4045	CaTSNP7005	TCTTAACAAGCCTCCTATTAAGACTATTCAGAGTCCAGATGGAGATGTAATAGATTGTGT[A/G]CCTGTTTCAAAGCAACCAGCTTTTGATCATCCTTTCCTCAAAGATCACAAAATTCAGATG
4046	CaTSNP7006	CTCATTTAGCAATAGTTATGGAGTATGCAGCTGGAGGGGAACTCTTTGAGAGGATATGTA[A/G]TGCTGGCCGGTTCAGCGAAGATGAGGCTAGATATTTCTTTCAGCAGCTGATATCCGGTGT

4047	CaTSNP7007	TCTCCTTCTCCCTAGGACCAGGGGATGAAGTGGCCTGGAATTTTCCCGAAGAAAGTGATA[C/T]CAATGGTCCATTTTTTGAAGATAGCGTATCTTCTTCAAAATCATCAAAGATATCTCTCGC
4048	CaTSNP7008	TTTCCTATTAAATCCTCCTACACATGTGAGTCAAGGTGATGAGTTAAGTGTTAATTTTCT[A/G]ATGAGTCGCTCGAAGGAAAACCATCGTTTGATGGAGGTTGAACTTGGGTGTGAGATTCAA
4049	CaTSNP7009	TACTGACGAAGGCCGAGACATTTCTGTCACCATATCAGGATCCAAAGAGCGCAGATATTC[A/G]AGCAAATTGTTCCGAATTCCCCTGTCGTGTTCTAATTGCATCATTTTCTGCTTCTGGGCA
4050	CaTSNP7010	AGATGCTTGCAAAAACGATACTGAACTACGGGCTGTCCTTGGAGATAGTATCGGCAACCC[G/T]GAACTCATGAGGAACCGCGTGGAAGATAGAATTCGAAAGAAGGGTAAAGACTTTCGTAGG
4051	CaTSNP7011	AAAATCTTGTCTTTTTCCAACGGTGTTCTCAAACGCCACCACCACCTTAACAACCACCAC[C/A]ACAACCACCAAGTAACTGTTACTTACAAAGAATGTCTCAAAAACCACGCAGCCAACTTAG
4052	CaTSNP7012	TTGTGGAGAGGGTGGAGGAAATGGTTAAGGGAGCGAAGGTTGTTGTGAAGAGAATGGAGA[A/G]TGGTGACGGTGCAAAGTTGGAAGGGCATGAAGGTAATTTGCTTGGTCTTGTTGTGGTTTA
4053	CaTSNP7013	TCTATTTGCAAAGCCTTGCATTAGTATGGCCCCTGTGTCTACATTCTGAGCTATTGCCAC[G/T]GCCAAACCTTTATCATCCCATTTTATACCGTCCAACAACGAGTCTACCTTAGGGTCAGAT
4054	CaTSNP7014	ATGATCCAGCATTCTACGAGTCCTTTGTCGACATCTACACTGCCCCAAGCTTGCAAAAGA[C/T]TTGGTACAGTGTTTTGGGAAACCATGACTACAGAGGTGATGTTGAAGCACAATTAAGCCC
4055	CaTSNP7015	CTCCGCCCCAACAGCAGTGTTAGTCAGTGGGCTTACAACATGGTAATACGAAGCATTAAG[G/C]GTGTCACCTTTATCATTGATAGTTAGAGATGCAATAAGGTCAGCATGAGTAACATTCAAC
4056	CaTSNP7016	GAGTCAACAATTCAACATGTTCCTTCTTAGAAGGCAAACTCAGAATCAGCAGGAGAATGG[T/C]TCCTCACATTATGATGCTAAGGTGGCTGAACTAAGAGCTGCTATTGGGCCCTTATCTGGG
4057	CaTSNP7017	GGATATAGTAATTGCAGTGGACCCATGTATAGCATCAATCAAACCATCATTACAATTAGA[C/T]AATGAACAATGATCCACCCAAACATGACTTCCACCAAAGATGGAAACACCATCACCGTCC
4058	CaTSNP7018	ATACACAAGTGATGATTATTTCATGAGATGGAATTACCCAACCTACAATTTTACAATGGC[A/C]TCTTTTTGGACACCCCATTTGGTAAGATCCAAAGAATCAGATATAAAGGGTCCAGGTTCA
4059	CaTSNP7019	AACTAGTCCAGGCTTTCCCATAGCTTCCGCTTTCTTAATCACCTCGTCATCATAACTGTA[T/C]GCCTCAGGGTCCACCACCATCACCTGTTGTGTGACGGGATCAATATCAAACACACGAGTA
4060	CaTSNP7020	CACAACAATAACATGAGGAAGATCAAAGTTGATTAATCTTTGAATCCTAGGAAAAGCCTT[G/A]GCAAGGATGGAAATGGTAGTACCATTCCCTCCACCAACATCCACTAAGGAATTAACACCA
4061	CaTSNP7021	TTATTTGATTTCATGGTATTAATTAATTAACATCATTAGAAACTGAAGTGTGAAGAAGAA[G/C]AATATGGCCGAAGAAAAAGAATCAACGTCAATTCCTCTGAGTCAAGCTGACAATGCCTCT
4062	CaTSNP7022	ATGAACTATGTTACCACCAGCACTGTCACCACCAATGAAAACTCTGTTGAAATCACCATG[G/A]TTGATGAGCCATGGCTCTTCATTGATGGGTTTTTGATCTAGAGAAGAATTAGAAGCAACC
4063	CaTSNP7024	TTAGCTGCTCTCAGTCTTGGTGTTCTCTCATCAGAATTTATGGCTTTTGATTTGTTAGTA[C/T]TACGACGATCACGAACAACACCAGCAGATAAGTCGTAGAGCTTTTCTTCTGAACAAGTGG
4064	CaTSNP7025	TCCCAATAGAAATTGTATAACAGGTACCTGGAAAGACAATCCTGTACTGAACATAAGAAC[C/T]AGAACAAACTCAAAGTATTGATCAATGGACCACAACGATTCAACAGCCCCTTCAGCATAA
4065	CaTSNP7026	ACTGGTTTCTTGGACTTTATATATTCTGCCTCTAATGCTCTCAATTAGAGGTTGATGGAT[G/A]TTTGGATTGTTGTTGGTGAACTTATGAGTAGCAAAGAGTTTTGCTTCTCCGAATTGCGTT
4066	CaTSNP7027	TTGCTGCCAAGATGGATTCATTTCAACCTGATTGACTGCAGGGGTAATGGTAGCTATTTC[C/T]AAGAGTTTGGTGAGTTTTTTGGTACCATAATTGCATATGCCAATAGACTTTGCTAAGCCT
4067	CaTSNP7029	AGCAGGCTGGTCAAATTTTCTGTGCAAAGGGAAGAAATTTCAGGATCCTCTTGATCTGGC[A/G]CTTCTTTTTGTTGGTGGAGAGTTACAATCTTCAGATTTAAGCTTGAACAAGCATGCAAAC
4068	CaTSNP7030	TTGAGCCAAAACAGAGGAAATTTGAGCAGCAAGTGCAAGAATATCAGCACAAGAGACTAT[A/G]TTAGGACAAGCATTTTCCACCGCAGTTTTAATTCGATTCACAACATCCAAACCTCTTATT
4069	CaTSNP7031	AGATTGGGATTCTCTATTACAAAATGATCAGCTAAAGCTGGAGCTAGAGAGTGGACACAT[A/G]TTAAGAGGTTGGCATGAAGGAGCAATCAAATTTGCCCCAACATACAAGTACTTTCCAAAT
4070	CaTSNP7032	GCAGCCAAGGCTCCACATGTCTAATGAGTAATCATAGTCTTGCAAATCAACTAGAAGCTC[A/G]GGCCCCTTAAAGTATCTTGAAGCCACACGAACATTGTATTCCTTTCCAGGATGATAAAAT
4071	CaTSNP7033	AACCCCATTCAACGGTTACTCCGTCAAGTTCAGCCCATTCTACGAAAACCGCATTGCCGT[C/T]GCCACCGCCCAAAACTTCGGCATCCTCGGTAACGGCCGTCTCCACGTCCTCGAACTCTCC
4072	CaTSNP7034	AATCAGGGTTCTACCCTCTGATTCTATAATGGCTGTCAAAAAGAATATTGAAGACGTACA[A/G]GGCAAAGATAATTATCCATGCGGACAGCAATTGCTGATTCACAATGGCAAGGTTTTGAAA
4073	CaTSNP7035	TTCTGCTGCATATAATGATCCAAGCATCATAAGCCAGAAGCTCCCCATTGTCAAGCGCAC[G/A]ACCCAAAAGCTTGTAATTTCTTCTTAAATTATAAGGCTGTAAATTCCCTTCAGGGGTTCT
4074	CaTSNP7036	TTTTGAGGCGTTCAAAATTATTGTGACCGATCCTGATACGGTTTTGAGCACTCTGATGCG[T/C]GAAATTAAGGAAGTTGGGCCTGATGGGCAAGAGGTGACTAAGGTGGTACCTGCTGTGTCT
4075	CaTSNP7037	TCTTGGAGTCTAATAGTATCCCTATTGTTGAGCTAACAGTTAGAAATGGAAGCTCATTGA[C/G]CTTAAGATTACCTGATGCTCATGTTACATCTTACAAACCAAAAGTGTTTTGGAAAGATGA
4076	CaTSNP7038	AATAACACTAAGAGCAAAGATCTTGAGGAACTGTTTTTTGGAGTGGATTTGTTGGAACGG[G/A]ACGATATGGAGGATGTTAATGGCGACGTAACTGTAAGCGGCGCATGATAGCATGTGGAGC

4077	CaTSNP7039	TTCCGACCTACGCTATTCCAACGGAAAGCGCCTCACGCGACTTTCAATCCAGGACGTAAT[A/T]GCAACGGCGGTGAGATCTAAACCGTTCCCGGTCGATCACCGGAAAGGAATCTACCTTGTT
4078	CaTSNP7041	GATATCCTCTGTTGGGTGCCACCTCACCATTTTCACATCCTGAGTATGTCCTTGCAGCAC[C/T]GACACACACTCAAACTCATTGCCTGGCTGCACTTCCCATATCCAAACAGACTTATCTCTA
4079	CaTSNP7042	TACAAAATACATAATAAGCGAATGAACAGGAAAAAAATACATGTAAGAAAATATTACATAG[A/G]CGATTGAAACAATTTACCTGAGTCCTCATCACAAAAGATACAATAAATACAGTATGCAAA
4080	CaTSNP7043	AACTGGAGCACCTAAACCTTTAGATAGGCAAACTGAAACCGAATCAGCTGCTTTGACAAG[C/T]CTATCCACAGGAACACCAAGTGCAATTGATGCGTTAAAAATACGGGCTCCATCGATGTGA
4081	CaTSNP7044	TACAAGTTCATAACTCATCTTTCTACGGTATCAGATCCCTGCTTCACAATAATAGACAAA[G/C]TGGACGAGGGAAACCCAGTCTCTGCCTCATTCCATTAATCTTCAAATGTTTGGCTAGAAA
4082	CaTSNP7045	TTTCTTTAAGTGGGTACTCACTGATAACATCACTGTTGAGAACAAAAAATGGCTCGCCGG[A/T]GTCATCAATAAGCTTATCCCTAGCAAGAGCCAAGGGGCCTGCAGTTCCTAATGGTTCAGT
4083	CaTSNP7046	TTGGAAGTGGAGGGATCATAAAATTCACTATGTTGTGCAAGGAGAAGGCCCTCCTCTTGT[T/C]CTCATTCATGGTTTTGGTGCATCTGCATTCCACTGGAGATACAATATACCAGAATTGGCT
4084	CaTSNP7047	AAAAATGAGGCTGATGTCATTGGTTGATCTCAGCTCTGATGGATCTGGTCAAATTCCATA[T/C]GAACTTATTAGGGACACACTTCAGATCAATGATGATGAAGTTGAACTATGGGTGGTAAAA
4085	CaTSNP7048	TGCATGCCAAAGAAACTCCTTTTGCAAAGTTGATTGCTTCCAATCCATGGGCCGGTAAAG[C/T]GTTGCTAAAGCTCGGAAACTTCAAGCAGTTGCATGCATCATTGGCGAAGAAGCCTCATGC
4086	CaTSNP7049	AAGGCTTAGTGAAGATAAAGTGAAGCAATGTGTTGATGTTAGATTGAAGGGAGAGTACCC[A/T]TCCAAGTCAGTTGCAAAGATGGCTGCTGTTGCTGCACTATGTGTTCAATATGAAGCTGAG
4087	CaTSNP7051	ATAATCAAAGCTTCCATTTCTTCTGTGCCATTAGCAATTGGTACAAGAATCTTGGGTGGA[C/T]TGTCAAATGTCCATTGCACTGGGTTTAGCTCCAAAAAAGTATATTCATCACCATGATTAG
4088	CaTSNP7052	GTTAGATTCACGGTCCCATGACTCACGTGATCGCGACCGAAGAATAAAGGCTTCGGGAAC[G/A]GAGACACGTGCTGTAGCATAAAGGAAAGTAACAACAGTGTAGTGTGTTGGATTCTCAAGC
4089	CaTSNP7053	TAAACAAAAATGCAAAACTAGATTAACTGTCCATCTGTTGAATGTATACTTGTTGGTCGA[C/T]GACGTCACAGAAACAAAAGTTGTACAAATGCACGCCTAGTAGAAATCAAAATCAGCTTTC
4090	CaTSNP7054	TTAACAACTTGAGTGAAATATCAACCTGATTTTGTCCAGCCAAAACGATAGCAACAGCAG[A/T]AATGGATGGATCTTGGATGATGTCATGAAGACCGTTATCTCCCCATTTAGAGTGAACTCC
4091	CaTSNP7055	GTCAGTGAAGCAGGTCAATCCTCGACAGTATGTTGCTCAAATCCCTCAGATTTCCAACTT[G/A]GAAGTCTGGTTCCAGAGGCCGAAAGACATTGTAAGAAAATTGTTATCCGGAGATCTTGAT
4092	CaTSNP7056	CCTTTATTTCATCAGCTCCTTTGGTTCCAAGCCTCTGTTCCACATTACATAAGAGTCTAC[T/C]AATAAGATCCTTTGCTTCTGGTGATAGTTTAGCTTCTTCTGGAAATTTCAAATAAGCTCT
4093	CaTSNP7057	TGCTGTCCATATAACAATTGGTGATGGAGGGAACAAAGAAGGCTTAGCACATAAGTATAT[T/A]ACTCCACAGCCAAAGTGGTCAGACTTCCGTGAAGCAAGTTTCGGTCACGGTGAGCTAAAG
4094	CaTSNP7058	TGGATGCACCAATTCCACAAGAATTACTGGATTATGTTGATATTTTTAGTCCTAATGAAA[G/C]TGAACTTGGTCGCCTTACGGGAATGCCGACCGAAAGTTTTGAAGAGATTACACAAGCTGC
4095	CaTSNP7059	GCTCATTAGCTTGTTCTTCTTTGCTACTAAGTACATATGGTTTGCAAGAATAAATGTAAG[A/T]GGAAATGTGCTGATCGCTCCTGTGAGGCTCATGAAGTCTCCAAGGAACGGAAGGAGAGCC
4096	CaTSNP7060	TACTATTAGGTAATTGATTAATTATTATTATCTTTATTATTATGTTGCTTTACTTCTCCC[A/T]GTTTCATTTCAGTTTTTTGGTTCATATTAGCTGTGTATTGGTGTCTGAGATTGACGTTTG
4097	CaTSNP7061	AAGGATACCGGGTGTTTGCAAGCATCGTCGCTCAATTAGAAGATTGAGAATAGCAGAGGA[A/G]CTAATAATGCAAGCGTCTACTAGGCTATGAAGTTGTCTTGTTGAGTTTAATTTAATATGA
4098	CaTSNP7062	CACAACATCATCCATGTCATCATATCTTGCAGCCTCAAGCAAGGCTTCGATGTTCTCCGG[C/G]GTCGTTTCAGGCGGATTGATGTTGTCTTCCACCGCACCCATTTTCAGTTGTTACCTGACC
4099	CaTSNP7063	AGTTTCAATAGAACCATCCTTAGAGACACGGATATCAGGCCTTCCAAACAGAGCCTGAGT[T/C]TGCTTTTCAATCAATGATATAGCTTCCTTGGAGCGTACGGTAGCAAGTCTCTGAAGTGTC
4100	CaTSNP7064	ACAATTCTCAGTACCAATGTCCTCCTCTACTCTCTCACCATCACTCTCTTCGCCTTTCCT[T/C]CACGATTCTTCACCTTCTCGCCATGGAATTCACCTCTTTCCCTTTTCTCACGCTACTTCC
4101	CaTSNP7065	CTTCTCCTTAAGAAATTTTCCCAAGGCAAAAGCTGCACTAACAGCTGCAAGAACTGCTGC[A/T]AATGCTATTTATGTTCCACCGGCTCAACAAGGTGCGATAGATTTGCAGAGTGGGATTCTC
4102	CaTSNP7066	TCGTGCTCTTGGTGATCAATTAATTGTTGGTGTTGTTAGTGATGCTGAAATTATTGCTAA[C/T]AAGGGTCCTCCTGTTACCCCTTTACATGAAAGGTTGATTATGGTGAATGCTGTCAAATGG
4103	CaTSNP7067	TCTCCCTCTACCGTATGATGGTAAAGGTTCAATCACAGGCCACTCAGTTATATCCTGAGA[A/T]GCAAGGAGAATAGCATCTTTATCCAAATAGAGAGTGAAATGACTAGTAAGGCTGAAACTA
4104	CaTSNP7068	TGCTTTGTTAGAAGCTGCTGTTGTTCTTGGTGCGATTCTAGGTGGTGGTTGTGATGAAGA[T/C]GTTGAGAAATTGAGAAAATTTGCTAGATACATAGGTTTGTTGTTTCAGGTTGTTGACGAT
4105	CaTSNP7069	CCTTAAAAAGAGCATAGCGGTTGCATGGAAATGGCAAAAATCTCGTCCTAATGGTTATGG[C/T]GTTTCGTCTACAATCTGAAGTTCCTAGAGCTATAGTAAGGCATCCATAGAAAGCTGCCAT
4106	CaTSNP7070	ACCAATAGCTTTCATTCCAACATCTGTAATTCCTTTACAGTTCTGCAAATTGAGGATCCT[C/T]AAGCATCTAAAACCAGTGGCAACGACACCAAGATCGGAATCAGTGACACCGGGAAAGAAG

4107	CaTSNP7071	AGTAACCATAGTTCACAAAGGATCAAGATTGCTTGAATATATTGGGACAAAAGCTTCAAG[G/A]AAGACATTAAAATGGCTTAAATCAAGAAAAGTTGATGTGAAATTAGAACAATCAGTTGAC
4108	CaTSNP7072	TCTCAGTGACATTTTCATTACCCAAGTCCACCACATGATCAACATCGAGTGATTTCAAGA[G/T]CTGCACCTTTTCAGCTCCCCTGGCAACAGCAATAACAATGGCCCCACATGCTTTTCCAAT
4109	CaTSNP7073	GTTGCAAACTTGTCCTATGAAGCAAGGTCTAAAGATCTCAGGGAGTTTTTTGATTCTGGA[T/A]CTGGTAGAGTTGTTTCTGCAGAGGTTATATTTAATGAGAATCCTAGAAGGCCCTCTGGTT
4110	CaTSNP7074	CTTGTATTGTATTGCTGACTACAGCAAGAAGCTGCAAGTATTCATCAGCACCATCAACTA[G/A]GCACTTATCTGCTTCACCCAGCTTCTTGGATATTCTTGCCTTCTGTTCATCTGATATGTC
4111	CaTSNP7075	AGGATGGGCAGCCTTCTGATTATATATACGAGTATATTAACTATGAGGCTTTTATTATTC[G/T]CTGATTTTTTTGGGGTTCTTCATTTTAAGAAAAGAAATAGTGGAGGAAACTGAAGAAAGA
4112	CaTSNP7076	GTTGATGGCAGCCGCAGTCCGCAACAAGCAGGACCTATTTAGTCTTCTGGGGGTTGACTA[C/T]ATCATAGCTCCATTGAAGGTGTTGCAGTCTCTCAAAGAATCTGTTGCTTCTCCTGATGAG
4113	CaTSNP7077	GCATCCTTTTGTAATCAACAGGATTAAGGGAAACAGCTACAACTTTGATGAGGACTTGAT[T/C]ATCTTTGAGTTGTGGTATAGACCAATTAGGATGCAATTTAAGAATATCTGTTGGTTTACC
4114	CaTSNP7078	CATGAGCATCAGAATCCACGTCGACTTTGTGATCTTTTCCTTTACCATAAGAAGTAATGC[A/G]GAGCTGAGAACAGTTTTCAATATCTGTGCCTGCAATCCATTGAAAAATCCCAGGAGGCCT
4115	CaTSNP7079	TGTTGACAATATCTTTATGTGAACTTGATCCAACTTCAGAATCGTTTATGCACAATATAT[C/T]GTTAAAAATGGCAATCATAACCGATTCCAAATCAGACCTTCCTACAAAATTATCTCCTGA
4116	CaTSNP7080	TAGCCTTTTGCTTCAAATTATAGCTGGTCAGCCTGCATGATCACAATGGATTTAATGACA[G/A]TATAAGTGATTTAGAAGACATTGGTTAAACCCGGCTGCATAGGGAGAACCATCAGCTCCA
4117	CaTSNP7081	GGCACAAGCAATGAAAGAGCAAGCTCAACTCAGGGAGGAGATGGCTTACCAGTACAAGCT[T/C]GGAAACTTTGAGGCTGCTGCTGCAATCCAGAGAAGGTTGGACCCTGATGCTGCAGTGTAG
4118	CaTSNP7082	AGGGAAATCATGCCCTGCCAATATATCTAATATACCAGCCGTGGTAAGACCTCCTAGTAG[C/T]AGCACCCATATTTGAGTGATTACCTCATTCTCGGATATTGCTCCAGAAGGAATTGGTCTA
4119	CaTSNP7083	TAAATGGTGTGAGTTAGGGGCTTGCTTTGTAGGAGGCTGTTGCAGAACGACTCCTGCTAC[C/A]ATTAGAGGAATATACAAGATACTCTACGGCAATGAATCTGCTACTCTTGCCACAGAGTGA
4120	CaTSNP7084	GAGAGTAGGATGCCTTAGCTTGCTTTGCGAAGATGAGAAACAAGCCGTGGCTGTAAAAAG[C/T]CAGTTGCAGTTGATTGCCAGACCCATGTACAGTAATCCACCTCTTCACGGAGCACTTGTT
4121	CaTSNP7085	TACTCGTTATAACTTCCATGGTGGAGTTAATATCAAATATGTAGCAGTTGGGAATGAGCC[C/A]TTTTTGAAATCTTACAACAATTCATTCTTGAACATCACACTGCCTGCACTTCAGAACATT
4122	CaTSNP7086	CTAAAAATAATCTTTAACTGACATCAGACTCCCAGACCAAAGTAGCGAAGAATGTGATCA[A/C]GATCATCCAATCAAGCCAGCGAGCATACTTTATCTTGTCTGATACTACTGCAGCAAAGGC
4123	CaTSNP7087	GTCTTTAAGAACGGTTACACGGGCGATTTTACCGAAAGTTGAAAAGAGAGTGTGTAGGTC[A/G]GAGTTTGTTAGCGAGTAATCTATATTTGAAACGTATAAGGTTGATTTTGATGGTGCTAAT
4124	CaTSNP7088	ATGGATCACCTGAGTTTGATCCTGCAGTGCAAGCTCTTGTTTATCAAGGCGGAAGTTATG[G/A]TGGATCCAGACCTGAATTCCGGAGGGAAACCTTGGATGAAGTAGAGATACGAGAGTTGCT
4125	CaTSNP7089	TATTGTTGTTCATTTTCCAACCATCATTGAAATCACTTGGTTTTGAAGTGAAATTAGTGA[A/C]GGTGTTGTTGATATCAGAAATTCCATTGTTTTTGACATCAAAGTTTCTCCTTTGATCAGG
4126	CaTSNP7090	TGGTATGGAAAAGAATATCTTTGGAAATCATCCAGTATCGTTGTCAGGGACAGATTCACG[C/T]AGTCCTCCACCTGCATTTTCTTCTGATCCATTGATTAGTAGGGAAAATCTTGGAAATTCA
4127	CaTSNP7091	ATCATCACAAGCTGAAAAACAAACTGCTAGCCCACCTCTAGTGCCCTTGTAGCCAGCTCT[G/A]GAACTTCTATGATCTTCCCATCCATAATAACCTTTTCGGTTAATCCTGCACATAAATGGT
4128	CaTSNP7092	TAGGGAGAATACTGAAGGCGAGTATGCTGGACTTGAACATGAGGTCGTTCCTGGCGTCGT[T/C]GAAAGCCTTAAGGTGATCACGAAGTTCTGTTCTGAGCGTATTGCCAAATATGCATTTGAG
4129	CaTSNP7093	CTTTGTACTTGGGATTGTTGCACTTTCGGCAACATTATTGCCATCTATTAAGCGCTTTCT[A/G]CCGAAGCATTGGAATGACGACCTCATAGTCTGGCGTTTTCCATATTTCCGCTCTTTTGAG
4130	CaTSNP7094	GGGTCTATTCGTTGAGGTTGCAAAAGAGAGAGCCTTGCCTATTGGACCCGAGGAAGGCGG[T/C]TTCGGAATGTCATACGATCTTCTATACGGACGAGCTGGATTCTTATGGGCCGCATTGTTT
4131	CaTSNP7095	ACATTCCATTATTCTCTCTGCCTCTTGAGGTAAATTTGGTTTGAAATCTACTGTTAGTTG[A/G]ACTGCAATTAAACTTCCATTATCTGATGTTGTTGCTAATACTGCTCGTGAGTCACCAACA
4132	CaTSNP7096	AACAACCAATTCCTTCTGTTTCCACCATCAACAATACAGAATCATCTGCAATTCCCTCCG[C/T]ACCAAAGCCAATTCTCAAAAGTGCCCTCAAACGACCCAACCCTACTCAATCCGATACCCA
4133	CaTSNP7097	GGCAAGGCGGCCAAGACCTGCCTGGAACGAAGATCACCCCGTAGAATACATCCAGTGTGA[T/C]ATCACCGATTCAAACGACGCCGAATCAAAACTCTCCGTTCTCACCGACGTCACACACATC
4134	CaTSNP7098	GGAAGGATACAATTGGTGCCAGGAGAAGAATGTGGTGACTGTTTTTAGTGCTCCAAATTA[T/C]TGCTATCGATGTGGGAATATGGCTGCGATTCTGGAAATTGGGGAGAATATGGATCAGAAT
4135	CaTSNP7099	CTTAGCATATAGCGATAGAGCAACTGGGGAGTTGTATATGCCAGCACATCCACAAGAACA[C/T]TCCTTTTTACGCCTATCATGTGGAGCACTATCGGTTCCAAGGAAAAATCGTTTACTTCCA
4136	CaTSNP7100	CGGCAGTGCATTGGTTGGGATATTCTACTTTATTGGGTTCGGATTTTTCTGTCTCGAGTC[A/G]CTGCTGAGCGTCTGGGTTTTTCAGCAAGTATTGATGTACTTCCGTGGAAGTGGCAAGGCT

4137	CaTSNP7101	TGTAGACTTTCCGACATTTGGCAAACCAACAATGCCAATCTTGAGGTGAGAAGAGAATCG[A/G]CCAAGGATGGGTCGTTCAGCTGGAGCTTCCTTGGATTTGGCAGATTTTGGAGGCATTTTT
4138	CaTSNP7102	TAAACACGATGACGTGACTCACTCGGAGTCAAAGGAGGAATTACCAAAGGAGAACATTGT[G/T]GCTCCTGTTGAAAATTCCGACTCACTCCGTAACATCGATCTGAATGCCAATATGAACGAA
4139	CaTSNP7103	AAAGACAAAGTTAAGGTTGCCATCTCCAACTTCTTTAACGGATAAGTCGGAGAAATTGTT[G/A]CCAAGAATTGATGAGAGGGAAGGAACGGATTGAATGTATTCGATGAGTGTGTTCTCATTA
4140	CaTSNP7104	GCCAATGATAATATCAGCCTTATTTTGTATTTCTTTGCTTCACTTACCACAAAGTCCAAG[G/C]CCTTGAAGACATCTTCATCATAAACTGAAGGGGATTTCTGTAGGGCCTTCCATTGGCCAT
4141	CaTSNP7105	CTTCTGCTGACATGTTGATGTACTGATTGATTGATTGATGAATTTTGGTTTGAAATTTAG[A/G]GAATGGCTTTTGGTTGCTTCTTGGTGTTAGTTGACTGTGTAACAACCCCTGTTTGGCTTG
4142	CaTSNP7106	CAGATGAACCACCATGGTCTTCTCCCTCTCGACGACAATTATAAGCAGCACCTTCAAATT[G/T]CTCTTTCAAATCAGGAGCAACAATGCCAATCATCTCACTTAGTGCTCGTCGTTCATTCTT
4143	CaTSNP7107	AGTCTATGTCATGATGTCTGGCTTGGATCTGGAAAGGCTTGTTTTAGCAGCTGGTCCACT[C/T]GGCATTATGCAGGCATGTCTCGATGTTGTCCTTCCTTATGTTCGACAAAGAGAGCAGTTT
4144	CaTSNP7108	AGGAATCCAAGAACTATTGGTCCAGATGCTATGGCAGTGGATGCCATGAAGAAGATGGAA[G/T]CACCTCCATCACCAGTCCAGTTTTTACCTGTGATAGATGATGAAAATGTCTTGATTGGAA
4145	CaTSNP7109	GTTACCAGAGCTTTCTTAGCACCATGGAAATTGAAGGACGTGCTTGGAAACCCGCTCTCT[G/A]GAAGCTTCTGGAGAGACAACTCACTTGCGAAATTCGCAATCTCGCACCGAATTATGCGTG
4146	CaTSNP7110	TTTCAGTAATTCTATTTGCTCATCTATTTTCCCACATTTCTTGTAAAGATCTATTAGTAC[A/G]TTATCAAGAGACTCTTGAGATTGTTTGGAGCAAAGACCTCTAAACGAACAGATGGCTTCA
4147	CaTSNP7111	GGTAACGGTGTGGATAGATAGGTTATTGTTTTTGTGAGAGTTAGTGATGGGACGAAAAGT[T/G]GTTGTTGAGCGTTTGAGTGGGATGAGGTTTGGATGGAAAAGGAGAAGACGTGTTGAGGTT
4148	CaTSNP7112	CCCTCAGTGGAGCCCTCAAAGTCTTTCAAAGGCTGTTCCGAATTTGGAAGAGGCTGGTCT[G/C]GATCTGCTCTCTCAAATGTTGCAATATGAACCTTCAAAGCGGATTTCTGCAAAGAAAGCT
4149	CaTSNP7113	TTTCGTCGGCGTCAAAGGAACTACAAACGGTGGTGGTAATCGGGGATTAAGACCAGATGT[A/G]TCGAGTTTGTTGAGCAAGGTTTTGTTAGGTTTTAGAATCTCTGCTTTTGTTTTCTGTTTG
4150	CaTSNP7114	AAAATGGTTGCAATTTTTCGCAATCAGATGGTAAGTGTCTCCATGATACTTTGCAGAGAG[A/G]CGCTCCATAAAAGAACGGAATTCTGATAGAGACATATCAGTGCTGCCCAACAAGACTGAT
4151	CaTSNP7115	ATTTGATTCTTCAATTGATGAAGTGAAAAGGCAGTACCGCAAGTTATCACTGATGGTTCA[C/T]CCTGACAAATGTAAACATCCACAAGCAAAGGAGGCTTTTGGAGCACTAGCAAAAGCCCAA
4152	CaTSNP7116	ATAAAGAAGAACCATCTTAAAGTTGGAATCAAAGGCCAGCCACCAATTATTGATGGGGAG[C/T]TATATCGATCTATCAAGCCTGATGAATGTTATTGGAGCATAGAGGATCAGAGTACACTTT
4153	CaTSNP7117	AAATAATATATAGCAAAATAAGAATGATCGATCTGCAGTCCGAAAACATATCTAGAACAG[T/C]AAGGAGAGGAAAGCACATCCAAATACTAAGCCAAGCACCATCTTCTGCGACCACAACTTG
4154	CaTSNP7118	TTTCCATGAATCAAACCGTTTGGTGACTGCAATTCTTGGGCACAGTAAAGGAGGTGGAGT[G/A]GTGCTTCTTTATGCTTCAAAATATCATGACGTTAAAGCAGTCGTCAATCTCTCTGGACGC
4155	CaTSNP7119	ATTAAGTGCTCTCAGCTCCTTCTTGAAATCAATATTAGGTCCTTTAGGATAGAGTTGACG[C/T]ACTCCTTGTTCTTCCAAGCTTGGTAGAACATCACTGGTAGTGTAACTGCCTCCAAAACAA
4156	CaTSNP7120	CACACAGCGATCAAGACTCAAGAGGCGCAGCAACTTTGCTCGTCTCTTTTTACAATTCGC[A/C]AACATGGCAAGAGGGTTGAAGAAACATTTGAAGAGGCTCAATGCTCCAAAACATTGGATG
4157	CaTSNP7121	AGAAACACAAGATAAAACATAAAAACAAAAGGATGCTTCTTAGATACACCCTCCATACTT[G/T]GAAGCAAATTATGATGACAAAAGGTGAAGTTAATTTGTTGAGTTGACAAACTTAATTAGA
4158	CaTSNP7122	GTACTACTATTAGCAAGAAGCATAGACTCAACAATTACAATTACAATGTTAGTAAGAAAC[T/C]CTCCATTTCCATGGCCATGCTATCTGCTCCCCAGGCATATGATTCAGAGTTAGAGAACAA
4159	CaTSNP7123	ACCAGACACACACGCAATGTACGGCCACAATACTTTCGATTCCGAATTTGCCCTTTTAGA[G/C]TCTATTCGCCGCCACTTATTAGGCGAGTCCGAAACAATATTCGGAGCTCCAACAACTTCT
4160	CaTSNP7124	CACACCCAACAGTTCCAATTTGACTATCACCATTAGCAAATGGTGTTTCCTTCTCAGTAT[C/T]ATTTTGGATGGGTTGAGTATAATCAATCTGGACCCTATTGGCTTCTTTTGCCTGATTTAG
4161	CaTSNP7125	GAGCAATTTGGACACCTTCTGGTTGTAGGAACTTGCAGGGGTCTAACAGAAGTACCAGAT[A/G]CCGAAATCGGATCAATGTTCAAACATACACCACTTGTGGAACACCGAGCGCAGGCCAAGT
4162	CaTSNP7126	TTTTTATTTTTTGTTGGGCAATTGTTGCATTTCTGTCATGCATACAGTTGTGGTGGCTTA[T/C]TTGCCCTTGAAATCTTGAAACAAATGCGTAAATAATTTTAAGAATTCTACAAATAGATGT
4163	CaTSNP7127	AGAGGATGAAGGTGGTGAGGAGAGGGAGGAAGGAGGAGAGGAGGGAGGTGAAGGCGGAGA[T/G]GAGTTTAACGGGACGACCGAAGAAGCCGTGGAAGATGCTGTGGGTGATGGTGGCGAGGGC
4164	CaTSNP7128	ACAATCCATAGCTGCAACTGCAACTTGCTCATACAAAGTCCATTCTTCAGAGCCGAGAGT[G/T]GATCGTTGTTTGGGATCGTTCAAGATGGACAAACCGTGCTTGAGGACTTTATCAGAACGG
4165	CaTSNP7129	CAAGCTCCAAAGCCAGCCTACACCACTCCCCGGTCAGCCTCCCAAGTATTCCGGTGCATT[C/T]GATGCTGTCAAGCAGACAATAGCAGCCGAAGGTCCAAGAGGTTTATATAAAGGTATGGGA
4166	CaTSNP7131	TTGAGACATAATAAAATCTAAGCTTATCTCATGCATTGTTACAAATCTGAGTAGAACCAG[A/T]CATACATATCAATTCAAACAACAAACTGTCTTACTTTCCACCATTTAGAGACCATGATTT

4167	CaTSNP7132	CTTGGATGAGCCAGATGAACTTTCTAGGTGCATTTTAAATGTGTCAATGCTGTGTGACTT[G/A]AAATCCGTTTGTTCCTGTTACCTTGTAATAATCCCTCGAAAACTGCAAATAGAACTTGTC
4168	CaTSNP7133	AGGAAGGTAATTTCATTGCCATGGACTTGTAAATCATGCTTTGCTAACAAACTCCACATC[A/G]AATAAGAGTACCGAATCAGGAGGAATGACACATGAACCTCCTTTACAACCAGCACCTCTT
4169	CaTSNP7134	AGATTCTTCACAGAAATAATTACCAACAATCATCAATCTGTCCAAATAAGCACGCCTCCT[G/A]TTCTTCGCCCTAACAGATCTCTTCCTCAATTCCTCTGAGGTCGGAGTCAGCACGCTCCGC
4170	CaTSNP7135	ACTCACAAATAGGGAAATAATAAAATCAAATCAACATCATTATCAACAACAAGAACAAGT[G/A]TTGAACTCTGAAATATACAATGTTCAAATGGATTCTACTCTGCCTCTTCCAACAACAATG
4171	CaTSNP7136	TGACCCTCAAGTTCAGGACGCAGCAAATCATGCTATTAAGACCATCCAGCAGAGGTCCAA[C/T]TCACTAGTGCCCTATGAGCTCCATGAGGTTGTTGATGCAAAGGCTGAGGTTATCGACGAT
4172	CaTSNP7137	TGTCGCTTCCGAGACTTAGAGGCGAGGCAACATGATTACTTCATGATTTATCAAGTTTAT[G/C]GATGCATTATATTTTGCATGTATTCTGTTGCTAATTATGCAATAAAAATTGCAGTGGTGG
4173	CaTSNP7138	TACATAGGAAAATTTATAGTTGAGGCAAGTGCAAAAGCAGGACACCCCACTTTTGCTCTG[G/A]TTAGAGAGAGCACAGTTTCTCATCCTGAAAAGTCAAAGCTAATTCAAAGCTTTAAGACCT
4174	CaTSNP7139	TCTTGGCTGCGCGAGGGCTAAGTGAAGGCTAAAAGAGGAAGCCGCGGTGGCCGGTGGTGG[C/T]GCCTAATAGGCTACCGCCGGCGGAGCAGTGGTTGTGTGGTGAGGAGATTGGAAAAGAGAT
4175	CaTSNP7140	ATCATCACAGTTGTAGTATAGTTAACAATGCTTTGCAATACAGATATTATGTGTTGAACT[G/A]CCAAACTAAAATTCCTACATATGCTAGAACAAATTCTTCAAACTTTGGTGTGCCCCAATG
4176	CaTSNP7141	TCTTTATCTTAATTATAATATTATGGGTTATTGCTTGTGGGGGTTTCAAATGTTGATTGA[C/G]CTATAAGAAACTAAGTCTCTATTTGGGCACATTAAGGGATTTACAAAATGATATATTCGA
4177	CaTSNP7142	CTTACAATGGTTTCTTCAACTTCAACCATGTTGTTGTATCCTAAACTAGGAACATGTTCA[C/T]TGTCACTGTCCAATTGTACAACTTCACATTCAAAAATCAAACATTTTCATCTCTATTCAT
4178	CaTSNP7143	TGGAGGTGTGAAAGATGCGTAGAGGGGTAGATGAAGAAAATGGGAGCGTGGGTGTGGCAA[C/T]ACCAGTGTTACCTTCTTCGAGTTCAAGCAGTAGCGCTACCACGACGGCACGAATCGGACG
4179	CaTSNP7144	CCACTTAATTGGTGTACTAAATTTCCTAACCTTCTTACTCTCAATCCCAATCCTAGGTGG[C/A]GGAATATGGCTAAGCAGCAGAGCAAACAACACAGACTGTCTCAAATTCCTCCAATGGCCA
4180	CaTSNP7145	CATTGATGACAATACGAAGAGGAAGATTGTATTTGTAGATAACAAGAAGCTGAAATCAAC[A/T]TTGCTTGAAGAGATTGATGAGAGTCAGATCCCAGAGATATATGGAGGCCAACTTCAATTA
4181	CaTSNP7146	AGCCAAGCTATGGTATCTCTCTTGTCCTGCTGTGTCCCAAATCTCAAATTTCACTGTGGC[A/G]TCATTTACTGCCAATGTCTGGGAAAAAAACGCTGCCCCTATTGTTGATTCCTGAAATTCA
4182	CaTSNP7147	ACTTACTTATCTTTTGGATATGAATTACTTTGGTCCAATCTTATCTGATGGCTCAACCTT[T/C]GATTTTCAAATCAACAATTCAAATATTGGAATAGACCCTTTTGTAAAACCTCAACCGGTG
4183	CaTSNP7148	GGGAAAGTTGTGCGTTGGTTGATGGAAGCTTGTTGGATTGAATTGAATGAATGAGAGTCG[A/T]ATATCGTGCTTTCCGTGAGGACGAGGATTTCCACTTCCACTTCTCGTGAAAATATTTGTC
4184	CaTSNP7149	AGTAGCAGCTTCAAGGTTTCTGTCCACAATTAGAGACTTTCTAATGATTTCTGCGTCGCG[T/G]CCAGCAGGGTCATGCATCCAAAGTAGAACTGCAGTCTCTAACTTTCCAGTGAGCTCCGAA
4185	CaTSNP7150	AAACAAGCCTTTTTGTTGGAGGATCAACTTGTAATATGTATTATCGAAAGTTGTTGAAGA[A/C]GGGTCCAAAGTGGTGCCTGCATTTTTTGCCTTATTTTTTAACGGACAAATTGATTTTAGT
4186	CaTSNP7151	CTCTTCCTTATCTAAGTCTTTTATTGAACCTTTTCCTCTGATGAATACGCGACAACCGGT[A/G]GTAGCCTCGACTCGCTTGAGTGAATTACCTCTTGGTCCAAGAAGCCTCCCAACAAAGTTG
4187	CaTSNP7152	GAGAAAGGAGCTGGTGCTGTTAGAGGTTGGAGTCTCACTATCCGAACTACCACCACAAGC[C/A]ACCACGACGATGCTACTTTTTCTCTTCAGCCTCGCATCCTGACCCAGCAACCGTAAACCC
4188	CaTSNP7153	CCCTAACAACAAAAAAGAAGCCGAAGCCAAATTCAAACAAATCTCAGAAGCCTATGATGT[G/A]CTGAGTGATCCTCAGAAGAAAGCTATTTATGATCAATATGGGGAAGATGGTCTGAAGGGT
4189	CaTSNP7154	GAAGCAAGAATGCCATAGAACCAGTCAAAGATGAACATATTTTCTGAATAGAAAGAGAGA[T/G]ATTCAAAACCCAAAACTTGAGAAGAAAATGGGTTTGATTGAATCTGAAAAGGGGATCAGA
4190	CaTSNP7155	TTTTATGTATTCTTCCATCCATGATTTCCACGCTTGAGTAAGAGCTTCTCTGTCAGGTTC[G/A]TCTACTACCCCAACCTTTACGGAGCCACAAAGCTCTGGCACCGATTTCCAGTGCGCCAAA
4191	CaTSNP7156	GGAGCAATTATCAGCAGAAGACGAAGAGGAGATTTTGGCAGAGTTTGAGAACTTGGAAAC[C/G]CAGCTTGCAGTTGAAGATCTTCCTGAAGTTCCTACCACAGTTCCTGAAGAGATTGATGAA
4192	CaTSNP7157	GTATATCATAAAACCATAAAACCAATATTTTCTGTAACTGCATATTGGCATCCTTAATAC[T/C]CTGACTACAAATTCCTTCAAATAAAAGGGAAAAAACAAGTACCCCGAATGCCCACAAAAC
4193	CaTSNP7158	TGCCAACATTGAGAAGAAAAGAGGCAAAAAGGATCAGGATAAGAACCCATCAATCACTGT[T/C]CTCCAAGGCCCTAAGCCAGGCAAACCCACTGATCTTTCTAGGATGCGGCAAATATTGTTA
4194	CaTSNP7159	GGGACTCTTTAGCATCTACAAAGAATCCTACAACATCATAATTTCATGGAGAAAGATCTT[C/T]AGCCAAATTACCTTAACTCTAATCCTCCCTCTCACCTTCATCTTTCTTATCCACATAGAA
4195	CaTSNP7160	AATAGCTTCTTCATGTCGAAAAGGATGTCCATATTTCATGGAAGGAATCCTAGTTCCATG[C/T]CCACTATAATGAAAATAAAGCACATCCCCTGCTTCAGCTCGATCAATCATGCTACCTAAT
4196	CaTSNP7161	TCAATTCACAATTCAACCTACATCTTCAGATTTCTTCTCCTTTCTCTCCATCCACCGTTT[G/C]TCCCCCCTAAAATAATAAACAAAAAAAGATTCTTATTAATTTTATTATTATTATTTTTC

4197	CaTSNP7162	GAAGGCAACGCCAACTTTAGCAGCAGCCTATTCTATATCGCGAGCTGTTAAGATACCTGT[T/C]ATGTGTTCATCTGGGCTAAGTGCTGTCACAGCACCTATGGCTATCACTGCTGGAGCTGCT
4198	CaTSNP7163	TGACCCCACAGATAACGAGGCTGATGGACAGCAGCAGTCAGAAGAATCACAGATGCAGCC[G/A]ATATCGGCAAATGGAATCTCTCATGCTGGTATTGATACTCAGATTGTTCAGTATGCAGCA
4199	CaTSNP7164	AGAACCTTCTGGTTATCTCCGCAACAGCAACACTCTTCTTGAATTGGTCTAAATGGTGGA[G/A]GTATGATGCATTAATGCTGTCACCCTTGTCGCCAAATATTATTGAAGCGGATGAATCTGG
4200	CaTSNP7165	GATGTTAACCCATCTAGCCCAAGTTGTCAAACCTTTATAGAATGCAATGAAACGGTTCAT[A/G]TCTTTGTACAATTTGTTACCCTCTTCAATATAATCCCACGGTTGTGCATTCCCGGTGTGG
4201	CaTSNP7166	AAGATTTTAAGAATCCATCTGGCACTTCTCTTTTGGTACCATCGGTTCAAGAGTTAGCTA[A/C]AGAGAATATTTCAGAAGTTCCACCAAGATATATTCAATCTCAACATGAAGGCTTGGTACT
4202	CaTSNP7167	GTGGGTGGGTAATTCTGCAAAACTATGTCCAGGTATGTGTGCATATCCCTTCGCTGTACC[T/C]GAATATATCCCTAATGTTAAACCGTTTAAGTCACCTAACGGTGACGTTGGAGTAGACGGA
4203	CaTSNP7169	CATAAATACATGAACTCTCAAAATGGACAAATCATATCAATGATATGTGCATGAATTCTT[C/G]TTGAACTTGATAGGTTTTGATCCAGAAGAGTTGATAACCAAAGGGCAAGTGATTTCCTTA
4204	CaTSNP7170	AAATTGAGCAATAAGAACTGCTTCCTCTTTATCATCAGCAATTGAAACAATTTCTCCAGT[A/G]GGCCCTGCAATGAAGGAGTTTCCATAAAATACTATCTCACTTTTTCCATGCTCAGTCTCA
4205	CaTSNP7171	GTACTTTAGAAAACTGAGAATGCTTCTCCTATTGAATGCAAAGAAAATGTATCACATAAT[G/T]TCATTACTGTCACATTAGTTAATGCTCTCATATTGCAAAAACCACAATAAGTAATCAAGC
4206	CaTSNP7172	AGGGAGCGGCTGATGATTATGAGACATGTGTCAAACATTTGGTCAATACCAATGTAATAG[T/C]TTTATCATCATCTAGTGGATTTCCAAAATTTGATCTCATGTTGCTTGGCATGGGCCCTGA
4207	CaTSNP7173	GATGATGTATACAAGAGTGCTGAAGTAGTCAAAACAGTCATTCAAGAGCTTGGAGGGAAG[A/G]TTACCAGGGAACCAGGGCCAATTCCTGGCCTCAACACAAAGATCACTTCTTTCTTAGATC
4208	CaTSNP7174	CTTCCTCCAACTTTGACTTCCAAATAGCAGAAAAGGTGTAAACTGGATTTTCAAATGCAG[T/C]ACAGTTCTAGTTGAATCCATTAGGTACACCTTTACTTTCTGAAAAATCATGTTGAGAGCT
4209	CaTSNP7175	TGCTCTTCCAAATGTATGAGCACCTGAAAGTGCAACTAGATCATTTGTGTTGAGACCTTG[A/T]GCAGCAAAGGAAGATTTTAGTTGAGTAAGGTTGAAAGAAGGACCTGGAAGATTTTGATTT
4210	CaTSNP7176	GGGGAGTGCTAAAGCATCGCCCTCCTTAATTGCAATAACTTTCTCTTCTGATTCTGGGAG[T/C]ACAATTCCAGCAACTCCACTTCCTTGTAGAACATAAGCAACTTTAGAAGAATCAGAGTAA
4211	CaTSNP7177	CGCCTCGCCCATTGGGCAAGTGATGGGGTGGTCTTTGTTGCTTCCAATTGGTAGTGCCAT[C/G]CTCAAGAACTTGTCCATCATGTCCAATGTTAAAAATGGTGATTGTGGCATTTCGAGTTCG
4212	CaTSNP7178	AACTGATATCTTATCTTCCTCAGATGGTGTTCTTATATGTTTCCATGATGTTACCCTTGA[T/C]GAGACTACTGACATTGCAAATTACAAAGAGTTTGCAAATCGTAAAAGAACATATGAAGTC
4213	CaTSNP7179	CCTTTGCAGCATAGGACTCTGCCTACAATCCATTACTAGGTTCCCCATGCTTGAGACGCT[A/G]CTTGAGAAAGAGCCCAGTTGCTGTCTTGTTCAATATGCCAAGAAAAATATCCAAACAATC
4214	CaTSNP7180	AGAAGTAAAAGGAAAATTGCAGGTTGGAGCCCATTATTTGAGGAAGGAAATGTGCAGTT[G/A]GATGCAAAACATGAATGCAAAGATGCAACTCTTTTTCAGGCTCCTGAGGATTGTGCGCTT
4215	CaTSNP7181	ATGCTCTTGGTGCAACTGTTATAGGAACTGTATCCAATGAAGAGAAAGCAGCTCAAGCCA[A/T]GGAGGATGGCTGCCATCATGTGATATTATATAAAGAAGAGGATTTTGTGACCCGCGTGAA
4216	CaTSNP7182	AAAGTGACAGTGATTCGGATGATTACTTCGATGATGATGACGATATGTCCTACAAAGAGG[T/A]CAATGATGATAGCGAGGAGGAAGTGAAGGAAGATGAAGAAGAAGAAGAGGATGAGGGAAG
4217	CaTSNP7183	AGGGAGGTGAATTCGCGCAGAGATTAATGGGTTTTGTGAAATGACAATGTCTTTTGATGA[G/A]ACAGAAGTTTGTGGATCTTGGAGAGAAGGTGGTACGAAAGAAGAGCCTAAGAAACGTTCG
4218	CaTSNP7184	ACGGTTGGCATATTCCTTTGCAAGCAAATACCTATTTGGCCTATCCTTGTCATCACTGAA[C/T]ACTTTCCACAATCCGGGATAAGACCGAAAGACTGCACCAAATGGCATAGGTCGCATATAA
4219	CaTSNP7185	ACCCAAAAGGCTCGTTCAATTCAAACCCCCAATTATTCCTTTATCAAAACCCACCGAACT[T/C]GATGACGATGAAGAAGATGACAAAGAAGAAGAACGAAACAGAAGAAGAAAGGCTGAATCT
4220	CaTSNP7186	TGAAGTTGTTGAGGCCAAAGCTGGAGCTGGTTCTGCAACCCTATCAATGGCATACGCTGC[C/T]GTTAAATTTGCAGATGCATGTCTTCGTGCCTTGAAAGGAGAGTCTGGCATCATTGAATGT
4221	CaTSNP7187	CTCCCCAGACGACATACTGCTTAAACAGAAGCTTGACATTCTAGAATCAAGCCACCCAAA[T/C]CTAAAGATATTCTACACTGTAGATAATCCAACAAAAAATTGGAAAGGAGGTGCAGGTTAC
4222	CaTSNP7188	TTCATATTCCTGCCTGAATTGACTTGATTGCTCAGTTGACTGGAAGTATTCATCATCAAA[C/T]CGCCCATCTGAATCAAATTTAATTTCAGCACGTGAGGCAGAAGCTGCCCAGCCACCAGTT
4223	CaTSNP7189	ATATACCATTTCTCTTGCACGAATGCTCATTTCGGTAGCTTTTCCACCGGCAGATCCGAG[T/C]GGTTGATGGATCATAACTCTGGAATTTGGCATACAATACCTCTTCCCTTTTGTACCAGCA
4224	CaTSNP7191	ATTTGTCAAGGTTTCCATGGATGGAGCACCTTACCTTCGTAAGGTTGACTTGACAATGTA[C/T]AATACCTACAAAGATTTATCCGATGCCTTAGCCAAAATGTTCAGCTCCTTCACCACTGGT
4225	CaTSNP7192	GAAGTTCCAAATTCCAGTTAGATTTCTCTTCGCCATTTCTATTGAGATAGGAAGAAGTTC[G/A]TCTAAGATAGTCATCGAGTTTGGAATATCGACAACCTTCTCGTATCGAGTGATCTTTGTG
4226	CaTSNP7193	AATTGCAACCTCATCCCAACGAATTTCCTCGGGCTTATAGGCTGATCTCCACTTGAGTGT[A/G]TCTTCCAACATCTTCTTCGACTTGTCGACATTCCAATTTCGAGCTTCAAGATATCTCTTC

4227	CaTSNP7194	TGTGCAAGCTGGGCTGATGAAGATGATGGGAGATGGAAGAAACCTTGTACTTCGGAGATT[A/C]CCGAGTGGTTTTGTTGTTTCTAAGTTTCCCTTGGCAGACTAAGTAGCTGTTTGTCTAAGT
4228	CaTSNP7195	TCTCATTTTCCGTTTCCCTTTCTCCGCTCCACCAACCATGGCTTCTCATCTCAACCTTCC[A/G]ACAACGACATTCTGCATCCTCCTCACCGTCATAATCTCTCTATGTTTTGCCACCGAATCA
4229	CaTSNP7196	GGGTCAAGCATTGGGTTGTTATCAAGTAGATCAGTCAAATGTTGCGATCAAAGAGCAGTT[C/T]GGAAGATTTGTTGATGTTCTTGAACCTGGATGCCATTGCTTGCCCTGGTGTCTTGGTTAT
4230	CaTSNP7197	CCACAGCATTGAAGAGTTCGACGAAGCACTAGAATCAGCAAAAGATAAACTGGTTGTGGT[A/T]GAATACGCGAGCAGAGACAGTTCAGAGAGCATAGAGATATATCCTTTCTTAGTTGAACTA
4231	CaTSNP7198	GTTAACGTTAACGCTCTTCTTCTTGGAATCGCCACCTCCGAATCCCACCGGAGACCAGAA[C/T]TCAGTTAGAGGCGCAGGTAGATTGAGGTCAAATCCACGGTGGCTGACGGAGGAGGAAGCG
4232	CaTSNP7199	CAATAATCATGCACATGAACAAGTTTTCATGTCTCCTTATAATGGAGTTAATAACTCTTA[C/T]GAGTTTAAAATGCAGGGTCAAAATATGCATAGAAATCCTCTTCATGAGAATTCATACTTA
4233	CaTSNP7200	CATGTGGTTCTTGAATACTTGGCCAATATTAGGTGGCATCTGCCCACCTGGTATCGCGGC[C/T]TCTGTTCGTAATGCTACTGTGTGCATTACTTTCGCAGCTTTTATTGGAAACTTTCCGTGA
4234	CaTSNP7201	TGGAGGAGCACCTAATTTGGGTAATTATAGCTTAGACCCTTTATCATGTGATAGAAAAGT[G/C]TTGAAAGCTGTTTTGACTCAACTATCAAAAATGGAAAGAAATAACCCACTTAATAATAAT
4235	CaTSNP7202	CATCAAGTAGTAGTTCATGTTTAACCCACCATCTGCCCCGAATCCGAACCCGAACCCGCA[C/T]GCAATCACCTCTATTAGCAAAAACATTGTTATCAAATTAGCCATTACCATTTTCAATGAC
4236	CaTSNP7203	TAGGCAAGAAGATTCTGGTGTAACATTCTGTGAATCAACAGATGCTGATGATGTTTGGTA[C/T]AAGAGAATGGAAGCCTGTGTTACTCCCAATCGTAAAGTCTACGGTGATCTTAAGCCTTTT
4237	CaTSNP7204	GCCAATGGCTGTTCCTTCCCAGCTGCATCTTACGGTCCTAGGCATGGAGAACGTCGATTC[T/C]GGTCCCTCTTCAAAGCCTCAGCATGTTGTGCTAAATCATGTCTTCATAGAGAAAAACATG
4238	CaTSNP7206	ATAAGTTCCTAACATTTTCTTTGTCTTGTCCACTCTAACCCCAGTTTTCCAAACTACATT[A/G]GTGTATTTGAGGCCAGATACAATGTTGTTGGAGACAGTGAAGGAGAATTTAAGCCTGTAT
4239	CaTSNP7207	TGCTAGATACTTTGAATTTGATCCTGATGAAATCGATAAAGAGGATTATTCTTTTCGATA[T/C]GATACCACTTTTCATCGTGGATCAACATATACTCTAAAGGATCTTGATCTTACAAAGCCT
4240	CaTSNP7208	TTGGGCTGAACCAGGTGAAGCCATGGCCACAATTCTTGTACTGCAGAAACCGCGCTTGGT[A/G]TGACGAATGGAAATGGTGAATTGGGGGTTGAAGAGAAAAGTGAGGTGATTTGAGGAAATG
4241	CaTSNP7209	TAAGAATTTTATATCCCTGGGTGGTCCACATGCCGGCACCGCTTCTATTCCATTATGCGG[C/G]TCTGAGTTGGTATGCACCCTTATTGACAGTGTAATACAATTAGGAATCTACAGCAGCCTA
4242	CaTSNP7210	GAGATTACCAAGGGCTCAGCCATCTCAAAACCCAGCAGGGATGGTTCTTGTTGATAGACC[T/C]GCCGAAGGATCTAAGGCTGCATCGTGTTGTTCATAAGTGTTTGTTTCATCTGTATTTACC
4243	CaTSNP7211	CAAATTCTCAAATTGGTATGTTTGTAAATAGTCAGAGATTGTCTCATTGCAACAATACAC[A/T]GACCAACCTTTGCATTCCCACCCTTTGTCACATACTTTCTTTCCCTTCACTGTTTTCACC
4244	CaTSNP7212	GAATCATATGGGGAATTCTTGGTTGGTGGGGAACAACAGGAAATGCATTTGGAATAACTA[A/G]CAGTGTTCCAGGTTTTAGTCCTGGTCTAACCTTACCACAAGCACTTTCCAACACAAGAAC
4245	CaTSNP7213	AGATACATTATCTTTGTTAGCATAGACAGCAATATAACTTAATGTGTTTCATCATTTCTC[G/A]CGTCATTCCAATCTCATTTGCACATAGTCGTCTTGAGCCTCTTTGCAGCTTTGACAAAAC
4246	CaTSNP7214	GGTGGTGTTACTAGTATAGTCACCACTATACGAAACTTCATCCCCTTCAGTTTCTCCATC[C/G]GTCAATAGCCCTTGGGAGAGAAAATTGTTGATGCCGCGCTTAGTACGTTGACGCCTCGGT
4247	CaTSNP7215	TGGTGCTCCATCCTTGCCTTGTCTTTTTGGAATAACTGCCTTGTTTTTGAGGCTAGGTTT[G/A]ATGGTTGTTGGGGGCATCATATTGGCATCTTTCATGACATATGCTTCCGAACATCTTGCT
4248	CaTSNP7216	TCTTCAGGCTGCTTATTTCTTCATCCACTACCTTTTCGCAAGCCAGACTGGACATGTAGG[T/G]GCTTTGTACTCGGCATTCCTTGCCATGCACAGGGCAGCCGGTGTTCCTGGTGCCCTCGCA
4249	CaTSNP7217	AAACTCTGTAGATGACTTGACTCCTGAATCTCTAGGTTGGGCAGGATTGGTATATGAGCA[C/T]GTCCTTGGTGAAGAAAAATATACATTTGTTGAAAATGTTAAAAATCCATTGTCTTGCACA
4250	CaTSNP7218	TTGCATTGTATCGGGGGGCGGCACAGCTAAATAACGCGGAAGACGGGCTTGGAACCACGG[A/G]TGTTGGCGGATCTCAGGTATGGTCATCCTCTTCATTGGGTCAACCACAAGCATCCTTGGT
4251	CaTSNP7219	CTTAAAATGGCAACCACTGCAGTGGAACCAGCTGTCTCAGGAGCAAGAGGTTCAATATTA[G/A]ACTCAGAGCCACCACTTTTTGTTTCACTGTTACCTGCACCATTTCCTCCAACCTCATCAT
4252	CaTSNP7220	TGCATATAACAAAACTTCCACTCTCTTTCCAGATGTTAAAGATCCGTTGAGAGCTTCTCC[T/A]GCTGATTTTGTTATCATCACAACTGGAATTGAAACATCTGCCCCAGAGCTATTGGAGCAA
4253	CaTSNP7221	TTTAATACTATCAAGATCTAGAGTTCTCTTTTTCAAATCAATTCTCATAAGTCCACAGGA[A/G]AATGAAGGAAGAAGTGAGCATGTTAAAGCCCACAAACAGCAAGCAAGCAACAAATACTTG
4254	CaTSNP7223	ACAAGCCTTTGGATTAAACCGAGCTTTGATCAAAGGATGCGAGAAATTCATGTACTGCGC[G/A]TATCTATGGAATGACCCAAAACATGTTTCTACAGCTCCATTCACCTTGCCGTCCATATCA
4255	CaTSNP7224	ACTCAGATTGTGAAGTTGGGTTGGAGAGTGCAAGAATGAGTGGTTTCTCATTCAAGGATG[C/A]CATGGCTTCAACTACATCCTTGGTAAATGTCTTTCCTACTCCTGATGATCCAATCAACAC
4256	CaTSNP7225	TCCTGCCATGTCATGACTATTATTCCTTGCAGTCGGTACATCATGATTGTGTTTACCCTC[A/G]TATGTGGTTATTACGGCTTTCGGATCATGAGACGCCCGCTCCACGTGTTTTCTAACTGGG

4257	CaTSNP7226	TTAAGATTGTTATTTTGTATGTATCATCAACATTAGGTGGTAGCTGTTGAACTGGTGGAC[A/G]CGGACCAGTGTTAGGTCGGTCATGCTTGAAGGTTAGGTTTTGTTAATTCGATTTAGTCAC
4258	CaTSNP7227	GGTAGAGAATACAAGCATGGTTCGTGATATTCCAGAATCCCTTACTCGCCCATCAAAGCT[C/T]ACTAGATTGAATGATGGAAGGGGCGCCTCTCTTTCTGCTGGGACTTCAAACATGCCTGTC
4259	CaTSNP7228	TTTCTTTGGAAAGCTGGACTTGACTACAGGCATGGAACTGGGCATGGTGTAGGAGCTGCG[C/T]TGAATGTTCATGAAGGCCCTCAAAGTATCAGTTACCGTTATGGAAATTTGACCCCTCTAG
4260	CaTSNP7229	GATCTTCCTCCTAGTTTTCGGACCGGTGTGACCGACAAACTCGCCAACTAAATCGGTTCG[C/T]TGTACTTCTGTCACTTTATCAGTAGGTAGAATGCCCACCATATGAAGTAATCTTCCAAGG
4261	CaTSNP7230	ATCACCAATTTTAATACTAATACTAATCTCAATTCTAATTCTAATTCTAATTCTAATTCT[A/C]ATTCCGATTCATCTTTTCACTATGGAGTCGCGAATTAAGACTAATCCCCAATCTCGCAAA
4262	CaTSNP7231	CCAGGCCCGATCGATCAGGACTAACCTTACAGCAAGTGGACCTAGGCCTAATCCACAAGG[G/A]TCCTACCATTATGGTCTCATAAACACAACAAAGACCATCATTCTGTCTAGTTCTCCTGGT
4263	CaTSNP7232	AAAGTTTTTCTGTGACATGAGTGATGATTTCTCTAGCTCCTCATACCCTTCAAGTCCTTC[T/C]TCAATCTCTTCAAGATCAAACATGGAACCTGATCCTTTTCTAACATAATCTTTCCCCATC
4264	CaTSNP7233	GAACTCTGTTTTGCTATTTTTACCTGCAATGCCACCCAATACAAGTAATGGTGAAGTTAC[C/T]AGCGTCCATATTAGGACTATCACCAAAATTGTACCAAAAGGAAGTGCCGCAGTTGCACTA
4265	CaTSNP7234	TCTATGTCTTGGTTTGTTTGCTTCTATTATTGCACCTTTCGGAGGATTCTTTGCAAGTGG[G/C]TTCAAAAGGGCTTTTAAGATAAAGGATTTTGGTGATAGTATTCCAGGACATGGTGGAATT
4266	CaTSNP7235	GATTTCCGAAGGGAAAATGGAAACTGCATCGGAGATCCTCGCTCGTTTGGTTCAGAATCC[G/A]AACCCGATTTCAAATTCGGATCGTAGATTAACAGATTGCATGGTTTCAGCGCTGAAATCG
4267	CaTSNP7236	ACGCCTCCCCAAACCCATGAGGAAGTTGTCGGGCTTGATGTCCCTATGTAAAAATGATTT[G/C]GAATGAATATATTCAACTCGATTTATCATCTGATCAGCAAGCATGAGTACAGTTTTGAGG
4268	CaTSNP7237	TCCGATTGTGCCACGTAGGACGAGTATTGTTACGAAACCGCAGAGGATAGTGATCTTTAT[A/G]TTGTTGAAGGTTTTCTGGATCTGACGTCCACGTGGCAGATTACGGTTTCGAGACGACGTC
4269	CaTSNP7238	TGGGGCAATTGGTGCTGAATGGTTAAAAGCATTCAAAGGCTATATTGAGAATCCAGAATC[G/C]ATGTTGTTGTAAAAGTAAATAGAAGACCTAGACGCACTCAATTCCTTCTTCCGAGTGATA
4270	CaTSNP7239	GCGTTCTTTCCAATTTTAAGACCTTGAGGCAAGATGAGTCAACAAGGAAGGATTATGTTA[A/G]TCAGCTTAAGATCGATATATCTTCATACTATGGCTACAATAAGTTTCTAATTGGTGCCTT
4271	CaTSNP7240	CCCTGACCACTAGTGTCTGTCAACCTGTAAATTATAGTTTCCTTTTACAGTTGTCTGGCA[A/T]TCATTGATGTCTGCTGTGTTTCAAAATTTTGCTTCATCTGGAAACTGAAGAGATATAGGG
4272	CaTSNP7241	ATGATGAGACATCTTGCATCTGTTGGAGAAACCTATGCTCAAACCAGCTTCGATGAAGCT[G/T]CTTGGAACGTTTGGCCTATTAATCCGTCTCAAGCTTCTGCGAGTGGTCCGAGTCCGACCG
4273	CaTSNP7242	CATGGCAGTTGCAACCCCCACGCCAAAACCAACGAGGTCAATCAACCTGAAAGGTCCCAT[A/T]GGCATTCCAAATTTGTATATTGCCTTATCAATTTGATAAACATCTGCACCATGTTCAACA
4274	CaTSNP7243	AGATATGTCCCATACTTTTGCAGACTTGTCAGCAGATACAGTCAGCACTTGTTTTCCATC[A/G]GGACTCCAGCTAACAGCATAAATACTGCCAGTATGGCCACCTTCAGAAGACAACTCACCG
4275	CaTSNP7244	TTAGTAATCTTTTGGAAAATCGGGCTTCTGAAATATCCTAGGATGATTTTTTAGCTGCTA[A/C]ATTAGTGTTGTAAGATGTTGAAAAGTTATATTTCATTTGTAATTTGTATTCAACTTCGTA
4276	CaTSNP7245	GGAACAGAACGTTGCTTTAGATTTAGCTCGGCGAGAGGCAGAGATGGCAATTCATGCACG[C/T]AATGATTTCCTTGCAGTCATGAATCATGAAATGCGGACACCAATGCATGCAATTATAGCA
4277	CaTSNP7246	CAAAACTTTCCTGTTTTCTGTCATGGTTGTGTTTAACCAACAGTAAAAATCTCCTTGGCG[G/A]TCATGAAAATGGATTTTGAAATCCCCAGCCAACTCTGTGAGACCAGGCTCGCCAGGCAAA
4278	CaTSNP7247	TCCTTCGCTTCTAAGGAAATACATTAAGGTGGCTCTTCATTTCTGGGGTACTGGTGGAGG[C/T]GCCCTTCCGGTTGTTTCATGTCTATTTATGAGAGAGTTATGCATCTGTATTGGATCTGGC
4279	CaTSNP7248	GAGCTTTTTACTTAATAATGGGAAGGTGATTGAAGGGGATGCTTATGTGTCTGCAGCACC[A/G]GTGGATATTCTGAAACTTCTTCTGCCTGAGAACTGGAAAGGGGTTCCTTATTTCCAAAGA
4280	CaTSNP7249	ATCCGGTTCAGGCTCCCTTTCCCTCCCTCTCGTCAACCTCCGATCCAACTACTCCTTCCG[T/A]ATATTCCGTTGGACTCGATCCGAAATCAACCCTAAACGTAAAGACCATGACAACAACCCC
4281	CaTSNP7250	GTTAACCATTTACATTGGAGACTCGGTGGGTGATTTACTTTGTCTACTTGAAGCAGATGT[T/C]GGAATTGTGGTTGGTTCAAGTTCAAGTCTTAGGACAGTTGGGATGCAATTTGGTATTTCA
4282	CaTSNP7251	TGTTTGTGCATCAAACTCTATACTGTTCGCTCTTTCCCAACCCAAAACTGATCTAGCAAA[T/C]TCAATTACAGAAATTTGCATTCCCAAGCAAATCCCCAGGTAAGGAACATTGTTCTCTCTG
4283	CaTSNP7252	GAAGGCTCGGTTGCAGGCCCATTTTGCTCAAATTCGTGCACCTGCTGGAATGGCACCGCT[A/G]CCTGCAGGAATTCCTGGATACCATTCTGGGGCACCAAGACTTGCTCCTCAGCAGTTGTAT
4284	CaTSNP7253	ATGAACAACTCCTCTGTCCATTGTGTACTCACAACAGCCAATACCTTCCACATCTTCGGG[C/T]AATCTCATGGCAGCAAGATCACCATAAGTGCAGTCTCTTCTAAATGATAAAATAGGTGGG
4285	CaTSNP7254	CCTGTTCCAGCTGCTGCAGAGTTGGAAGAGAAGAATGCCTTGGCTTTGGCCATCGTTCCC[A/G]TTGCTGATCAACAACCTGCTGCTGTTCCAAATCAAGCAAATGGAACCACGGGGTGGGAAC
4286	CaTSNP7255	GTGTTTCAGAGGTATCTTTCGATGATCAGGTCTGGCCAGATTGACCAGGTAAGCACAAGA[A/G]AAGACATTTGCAGCTCTTGCAATTCCAATGAACATAATGGAGAAGAATATAAATCCAACA

4287	CaTSNP7256	GGTGGAACAACTAGGTATTGATCAAGCCATTCCTTCTCGTATATTGGAGTTAATGAAAGT[T/C]GACGGCTTGACAAGGCATAATGTAGCTAGCCATCTTCAGAAATATAGAATGCACAAGAGA
4288	CaTSNP7257	CGCCAAGGTCTGCAACGGTGCGTCTTAAGCGCCGGACTTCAGCATCCAAAGTCTCGTTCA[G/A]AGCATCTTTCAGTTGGGACTGTTGTTCCAAGGATTGAAGCCTCAATTTGTACTCGTTATT
4289	CaTSNP7258	AAGAAAGCATGGAAACCATATGGAACTCTTTGAGGCAATTCTACAACTGCAACAGGATCT[G/A]CTGACATTGTTTTTGCATCTATGACGTGCACGAATGATTTCCCGATATTCTCATCATGTA
4290	CaTSNP7259	TTCTATGTATGTTTGTGGAGTCACTGCCTATGACTACAGTCATCTCGGTCACGCACGTGC[C/T]GCCGTTTCTTTTGATATCCTCTACAGGTACCTTAAACATTTGGGCTATGAGGTAACATAT
4291	CaTSNP7260	TGCCTTCCTTCAGACATTCTCTAAAATCATTGAAAACCCAGAAAGCCTAACATTATAGTC[G/A]TTCTTCACTTTGAGATAAATTTAGTTATACCAAACATTGTTTCTTGAGAAGCTGTTTGGC
4292	CaTSNP7261	ATCAATGTCCAGGATGCAATATGAACAACCTCTGTGTCTCTTTTTAAAAGCAGGCATTGC[T/C]GGTGGACCTTTTTTCACCTGTGGGCCGCCATCAATCTTACCACCAGCAGTCAAAGTATTG
4293	CaTSNP7262	CGACCTCGATTCGTCTTCGCTGTCGTCGTCACTATTTGCGGAGGAGGAGGAATCCATCAC[C/T]GGTTTTATCGAGCATGAACGCAAGTTTACCCCTGGATTCGATTACCTTTCGAAATTCGAA
4294	CaTSNP7263	TGGCTTCAGGCCAGCTTCTCTGAAACGAGTTACCAATCTAACCGCGCCTCGGTAGTCACC[T/C]TCAACCATCATCTTCCAAGCAAGGTACCCAGTTGGCCCTCCTTTTTCAGGATCATCCTCA
4295	CaTSNP7265	TGCCTCTCCTTCCAACACTGCAGGCCTTGAAAGAGCATGCCCCTCAGGTCCAGTTATTCC[A/G]AGCTCTATCATCTTCCTTTGCCCTTCATACACAGCATCTCCATTTTCTGCGTGAACCATG
4296	CaTSNP7266	GAAACTGAGGGCAGAACCAAAGGCCAATGACCAGTATCTGCCTCCATTTGTTATCGTTGA[C/T]GAGAGCGGGAAATCTGTTGGTCCGATAGTTGATGGGGATGCTGTGGTTACATTCAACTTC
4297	CaTSNP7267	ATCGATAAGAGCAACAACATCAGTGGTGATTATGTCCACATATTTATTTATGAAAATCCC[G/A]AGACCTCTTATTAGGTAGAAGTCCAATCCTATAGCTTCCTCAATGCTAGGACGTTGCACC
4298	CaTSNP7268	AAATACAGAGCATTATTTGAAGATGCTGTGAATATTAGTTATCATTGATGATGAATGCAA[C/T]AATTATCATCAAAATCACACATCCAAGACATATTATTGCATCTATTTCACTTGCGCAAGT
4299	CaTSNP7269	CTAGTAACATAATTACAAAAGGATTATATTCCCATTAGAATTAGAATAGATTAGATGTCT[C/T]GTATAGCTGGCTCATCACTTCCTCGGAGGGATCCTCCATGATCACTTCCAAATGTCCGCC
4300	CaTSNP7270	CGATTCTCAGCTTTACGATCTGTTCAATCAGGTTGGCCAAGTCGTTTCGGTTAGGGTTTG[T/C]AGGGATTTGGCTTCGCGTCGTTCACTTGGTTATGGTTATGTTAATTTCAGTAACCCTCAG
4301	CaTSNP7271	TGGTACAATTGGGCATATAGCACCTGAGTACTTGTCAACTGGAAAGTCTTCAGAAAAGAC[T/C]GACGTTTTTGGATATGGCGTGATGCTTCTTGAACTAATAACTGGACAAAGGGCTTTTGAT
4302	CaTSNP7272	GCTCGTTCCGCCACCTGGTAATGAGAGCTAGTAAGGCAGCAAGCCAATCGTCGAAACAGC[A/G]GCACCATAATCTTCTGAAATTCAGCCATGCTAGTCATTTCCAAAACCTCTTCCAACTCAC
4303	CaTSNP7273	AGCATTTGTGAGGGCCAACGGCCGGGTTTTCTGCAACAGACCCCGCCTTCACAATCCCCC[G/A]CGTTAACTTCTACACAATTCTATTTGAGCAATTAAAATTTTGTGAAGTATCCAGCTTCTG
4304	CaTSNP7274	TGATGGCTTTGACATGTTCCCAACATTATTAGTAACTGATGGTTCTTGCATGATAGATCG[G/A]AGGATGGGCATACATGGGCATCCTTTGGAGATTCAGGCACTGTTTTATTCTGCCTTACTT
4305	CaTSNP7275	CATTGCTTCTATAGTCACCCGTCCGAATGTCTCTCCAAGTCCCTGAGAGTTTATGACATA[G/A]ACATCTGCAGCAGAGTATAGTGAGGCAACCTGTGTTGTGGATGGAGTCCACAAAACTGTT
4306	CaTSNP7276	GCAGTGGCGAATTGCTAGCACCCATGAAGATTTCTGGAAGAGTTTGAATTTTGAAGATCG[G/A]AACATATCTGTAGAGCAATTTGAGGACATGTGTAGGCGTTATCCAAATGCTACAGCAATG
4307	CaTSNP7277	TTACTCTTGATGATGGGATCAATAGCTTTTGTAGAAATACGCCTGGACCTTGTGATCCTA[A/G]GGTTACTACTTTGCTTGATATTGCTGCTGATTTTGGGTATCCGATTCAGTTGGCTAGTTC
4308	CaTSNP7278	AAGTGTAACTTTAATACCATAGTCATTGCAAGCAGCTACAACATCCTCCTGAGCTTTGTA[T/C]AGTTCCCAAGCAGCGGTGAAGCGGCCAGCATCTTTACCAGAATCAGAATATCCAACCATA
4309	CaTSNP7279	GGCCATCTACCGAAGAAATTGCAGAGACTGTTCAATCTAGCGTGTTGCCTGAGATGTTCC[G/A]AAGTACATATGAGGCTATTACAAAAGGCAACCCTATGTGGAATCAACTTCAAGTTCCAGC
4310	CaTSNP7280	CTTCATTATTCTTCTCTGCACTATAAGAATCCTGACCACTAACTACTACTTCTGTTGATA[C/T]AGTCTCGCTTAGAATAGTCTCGGTGACTGTAACAGTGGTCTCCACAGTAGAAGCATGGGT
4311	CaTSNP7281	ACTCGTTAAATTTTCGGCTTCTTTTTCATGCAATTTCTTTCTGATACGCATGTAATCATC[G/A]ACCATATCTGTCCACCCACATGACCTCAAAACTGACTTTGCTTCTTTCCCAAGAATGTCC
4312	CaTSNP7282	TTATGTCCTTCTACTTTGTCCATTACTTGTTCTCATCAGTGAAGATCGATTGAGATTCTC[A/G]CTCTTATCTCTCATTACCCAAGTAAAAGATCAATCAATATAACTTCTGCTGAGTTATTTT
4313	CaTSNP7283	GTTATTTGCATCAGCAGAGAGATCATTATTGCTCCCATCATAAAGAATTTTCTTTGCCCT[C/T]GCAGCACTATCAACAGGAATCCATAAGTTCCACAAGCCTTCTTTCTTTGCTAACTCCTTC
4314	CaTSNP7284	ACAGAAGTGGGATAAAATTGGTGAAGTTGTTGATGGACCAGAGGAATCAAATCGCCCACT[T/C]TTCGATGGTGTTCAGTATGATTATGTGTTTGATGTGGATATTGGAGATGGTATGCCTATC
4315	CaTSNP7285	AAGCTCAACCCACTCAGCCAACAATGTTAACAAGCTATCATCTCCATCTTCCTCCTTCAA[G/C]GACAGAATTTTGTCGGTAACTTGTTTACATTTCCCAGTTATCGGCTTCACCTCCTTCGGC
4316	CaTSNP7286	TGTTTTTGAGCTCATGGAATCGGATCTTCATCAAGTCATTAAAGCAAATGATGACTTAAC[T/C]AAAGAGCATTATCAGTTTTTTCTTTATCAATTGCTTCGAGCACTCAAGTATATTCACACT

4317	CaTSNP7288	TGGAACCGGGGAATTCAAACCCTCGTCGGTGCTCACATCCTCGGTTACTTGTTCGTCCTG[T/C]GAATGCTTGTTATCAAGCTTTAGTACATCAGAACTAGGTATGGTGCTAGTCTGTCCTACA
4318	CaTSNP7289	AGCATTTAACTGCTTTCCCAGAAGGAATATGGTTTCCTGACACTCGGCTAGCTTTTCGGC[A/G]GCTGCTGCCAAATCTCTCTCCTGCGTAGACTTTTGTTCATTATCAGCCGTTGATCTCTCA
4319	CaTSNP7290	TTGCTTTTGGTTTCTTTGCCCGGAATGGCAAGTATGTAGAGTGTCTACTTTCTGTAAGTA[G/A]GAAACTGGACGCGGAGGGCCTAGTCACCGGAGTCTTCTGTTTCTTGCAGTTAGCTAGCCC
4320	CaTSNP7291	ATCCATCATGTATTTGAAATATTGAGGTGCGAATTCTTCAAACGATTCCAATTCTGTCTT[T/C]GTCACTTGTTTAATAATAAATCTTTCATCTAATGACTTGGCAAAATATACATTACTTTTC
4321	CaTSNP7292	GGGATGGACGGAGATGCCAAGCTCTAAGAACTGATGTTTCTCCACTAGCAGGAATGTGGA[T/C]GGTTAAAGTTGTTTCATCTTTCTTATTCTTCTCCGTGATTACACCTTCCCGCCAGCTTTC
4322	CaTSNP7293	ATACATAATCCATTGTTCATGCTCATGGCTATAAGCAAAGCAATGATAGTGCTGGCCATA[G/A]TAGCAAACCACCGAAACCAAGTTATGGGTGCTTTTGGGATCTAAACCACGATAAAGGACA
4323	CaTSNP7294	GATATATGGTATTATAGTATATGCCATGATGGGATTGGATTGGACAACATCAAAATTCTT[G/A]TGGTATCTTTTCTTCATGTACTTCACATTCTTGTACTACACTTTCTATGGTATGATGACC
4324	CaTSNP7295	GATGATCCTCTGTTGAGCGTGTCACTGATGGCGTGAATCAAAACCCAAAGTATAGAAGGC[A/G]TGTCGCTTGCCCAATTCGGAAACACTATTTTACCATCATGAAGAGTTGAACGTTCAAACT
4325	CaTSNP7296	CTATGCGGACAATAAAGCTGTTGAGATAAGGTTGTATAGAGTTATTTATGAATTAATTGA[T/C]GACGTACGAAAAGCAATGGAAGGGCTACTGGATTCTGTTGAGGAACAAGTAACAATTGGC
4326	CaTSNP7297	AAGTGACTGAAGAACCAAGTCAAGAGGAATCCGACTCTATATTTACCGATGAAATAATCA[C/T]TGAAATAAGGCATTTGGTTACGGGCATTTCATCTGAGAAGCGTCGAAAAACAAAATCCAA
4327	CaTSNP7298	GATCTCTTTAGTGAACCGTCTTCAAGACATTTTCTCCCGCGTCGGCAGCCAAACCGCTAT[T/C]AACTTACCACAAGTGGCCGTTGTCGGCAGTCAGAGTAGCGGCAAGTCCAGTGTCCTCGAA
4328	CaTSNP7299	ATTTTTCTGTGACCTCAACGGTATAATAGATTACACTGATGGTTATGGAAGACATCTGCA[T/C]CTGTAACAGAATATCTACTTTGTAGCTTATTTTTGAAATTTAGAATAGGAAACAGGAAGA
4329	CaTSNP7300	TTTGCCTGCTGTGTGTCTTCTCACTAACAAGTTCATCATTCCACAGATTAGTAACATTGC[A/T]AGTATATGGTTTATCTCTCTCTTCCTTTCCATCTTCGCAACTGGAATCCTCGAGATGAGA
4330	CaTSNP7301	AAGCAACCACACACCCGATCAGGGCGCACAACGATAAGAACATTCATTGTATCATACTTA[G/A]CATATTCTTTCCATCTGTAATTTTAGCATCTTAGTTTTTTATTTAGATTTTGATTCCTCC
4331	CaTSNP7302	CCAAACCTTCCATTCGAAAACCCCAGTTCCTCGTCCCTTTCGGATTCATTCCTATAACCA[C/T]TTACAGGCCAAATCCTCCCTCCACCTTGACCATACAACCACCTATCACTAACATAACTAG
4332	CaTSNP7303	CGTAGCTTCTTGAGCCCAAGCACCACCAACATTCGGCGAACCATCATGAAGAATAACATC[A/G]AAAGCACGATAACCGTTTTCATTCATCAGTTTCTTAATTCGCGACTTACATTCAGGTCTG
4333	CaTSNP7304	GGTGGAATTAGGGGAACTGGAACCCCTCAGATTGGAGGAGGGGCTAAAGCCAAAGAGGCT[C/T]TGTGGCAGCGGTTAGGGAATTGTATGGATCAATTGCATTCCATTACTGTGGCAGTGTGGC
4334	CaTSNP7305	TTATGTTAGCAGCCCAGAGAGCGAAAAGAGTTCAACATCTCTTTCGAAAGTATCTCAGGC[T/C]GTTGAAGAAACTGCAGGGGAGCAGGCCTCAAGCAATCAAAATGCTTCAGTTACTTCTGTG
4335	CaTSNP7307	ATGAGGTGGTGGAAACTTAAGGACTTGTTCACCATCAGTGCGTAAGATTTCTTTAAGGGC[G/T]GGTAGGGGACTAATTTTGCTGAGTATATCTGTAGGAAGTTTTATTCCACCTTCAAAAAGT
4336	CaTSNP7308	AAATCCTGTCATGGCTCTGGTTGCCTTTTTAGCCTCTGCGGTTGGACCAAGAGTAGCAGC[T/A]TCTGCTGCTCATGCAGCATTATTAGTATTGTCAGACGATAATACTGGCTCGCAGACGGAA
4337	CaTSNP7310	AGATCATATTTGTCAACATTTCCAATGAACTCATCATTCATCATCATATCCATTGGATTA[A/G]CAGCATCTATATTATCAGCTAGAACATCAGCAGCAGAAGAAGTTCTCAAAACTGGAGAAG
4338	CaTSNP7311	GCCATGATGGATTGCTCTTGGCATAATTAGTTTGCACCTGGAGATGATGGTGTGAAATTT[C/A]TCACGGTGACCAATTTTGAAGCACATGGTCACAATTTTGGCATCAAAAAGTGCAGCAACA
4339	CaTSNP7312	ACCGGCATCGTACATTCCTTTTGATAGATCCAAATTTGCTTGCTTTCCATCCTTAAGTGC[T/C]GAATCCCCTCTCCAACTTATTTTGTTGTTCTCTAACTTACCAGATTTTTGTACATCGAAG
4340	CaTSNP7313	GGAGGTGGAGAATAATTCTCAGGTGCAGGCAGTGCTTTGATTTCGTTCATATCCGGTACC[A/G]GCTCCTCCTCTTGTATTTCCACCTTGGGAGCTTCCTCTTTCCTCTCTGGACTTTTTGGCC
4341	CaTSNP7314	TCTCAGATCTATAAACTTCTTTCCTCTTTTTCTTAACATTCACAAACCCAAATTCTTAAC[A/G]AACTACACTTTCTTCTTTTGCTATGGCTAATCAACCTGAATCTTCCGATGCTAAGGGAAC
4342	CaTSNP7315	GAGTAGTGGTAGTAGTAGGTTGCAGCAGGAATCTGAGTCTTTGGTTAAGAGTGTTGCTCC[C/A]ACTAAGAGAATTGTTTATACTACAACTAGGCCTAGTGATATAAGAACTCCTTATGAAAAA
4343	CaTSNP7316	TCGTGCTTTTATTGTTGGTGACCCAAAGGGTAAACGTGTTGTGTTTGTTAATCTTGATGC[T/C]TGCATGGCTTCTCAGATTGTTACTATCAAAGTTCTTGAGAGGCTTAAAGCAAGATATGGT
4344	CaTSNP7317	AGATAGTTGTTTGGACAGATACTCTGACGATGGTTTGACAGAAGACTGGATAATGCCGAT[T/G]ACAGACGAAATAAGTGATGACAAAACCCCTCAAGGAGATTCCTCAGTTGATTGCTTGAGT
4345	CaTSNP7318	ATCCGAAGGCCAGAGGACATGGGAAGACGTGTGGTCTACTTTCCCTCACCACTCGTCTAC[C/T]GCTACAGATGAATAGATGATTGAATTTAACCAAGTCACCAATCTTGGTTGGACCTTTCAT
4346	CaTSNP7319	GTAACACTGTCTAAAAGCATAGATAGAAGCCAACTTAAAGGCAAGTTCACTGGAATCAAC[A/G]GCATGAGCAGCACCATCTGTCAAGACAACTCGGAGATTTTCAACTGGATGCCCAATTAAT

4347	CaTSNP7320	CACCACATGGATGAGAATGAAAACCAGAAATGATGCAAAGTGCTCTGACGTCATTACAGC[A/G]TTAAATCCAACAACAGGCACCAATGCGGCCAATAGTGTGCCCAATACAACAAGAGGTGCA
4348	CaTSNP7321	TCGGTGGTATTTGCTGGTTTTTCTTCGGAATCTCTTTCACAGAGAATCATTGATTCTAAG[G/C]CAAAAGTTGTAATTACTTGTAATGCTGTTAAAAGGGGATCTAAGGTTATCTATCTGAAAG
4349	CaTSNP7322	TATGTCCAAAAAAGTCGGTTGTTACAGTCCTTCTTCCAACGTTCTTTGGCGCACTGGTGG[A/G]TTCCTCACCAACAGTTACAGCCCTAAGAAGTTTCGTTCTCGCAACAAGAGGCTTTGTTTC
4350	CaTSNP7323	TGTGGTTGATGCCAGTGGAAGCATGGCATTGAACAGGATGCAGAATGCAAAAGGTGCAGC[A/G]CTTAAGCTTCTGGCAGAAAGTTACACAAGCAGGGATCAGGTATCTATAATTCCATTCCGT
4351	CaTSNP7324	CTCTTAGCAAATGCAGGGAGATATGCACAGCTTTGGGGACAGCAAAATAACACTACTGAT[A/G]CAATTGACACAGCTATCAAATTGGGAGCATAAAAGTAATAGAAAGCATCTTCCTTTCTCT
4352	CaTSNP7325	CACTGCAAGCATCTTGTTGACTCTATCAGCAATTTCTTCAGTTGGTGGTATTCTTGGAGA[G/A]TGAATGTCATAAACTCCAGGTCCAATTCCAGCTCCATACTTAACTCCATCACGGAACACC
4353	CaTSNP7326	TCGATGTGTTGTCAGACGCGGTTACAATGATGCCGTTGAGGAGCCTCTTCAGTTTGAGTC[C/G]CAATTGATACAAAATCTCAAGAGATTCATTCAACAAGAGAATTTTATGATCGATGTTAAT
4354	CaTSNP7327	CAAACCAAAGCTAGGAAAATTGTCAAATCCACATGAATCCCCAATACCACTGAAGAACCA[C/T]TTAAAGCAACTCCAGCAGAAACTTATGCTTAAATATTTTCCAAGTTGCCGAACTTGGTTC
4355	CaTSNP7328	TCTTCTATGTGATGATGAAAAGCAAGGAAAGTGAGTGGTGAATAAGGCTTTATTACTAGT[G/C]AGAGGAAGGGACCCCATTTTCGTATTGCTATCATCAACCGAATTTCATTTCACTCTTCTT
4356	CaTSNP7329	TCATTTGCAAGACCCTGTGATTCTACTACCTGTAAACATTGTTAAGTTCCCCATACATGA[T/C]CAGAGGCTTCCTCCAAGAATTTATTGTCTCCATCAACAAACAAACCTATGGAAGTGCTCA
4357	CaTSNP7330	GAAACAGAAATAACTTAAGCTACCTCACAAACCTAGCCACCCAATTAGGAAAATTGGTTT[C/A]AAAGTTCCTTCTATAGATCCTAAAGTTCTTCAGAGAAGATAGGTGTATCCCAAAACCAAC
4358	CaTSNP7331	ATCATGTAAGGTTCAGAGTCATCAAAGCTTCTTCTACCCCGGATAGGTTGATACAGAGAA[C/T]TAGACTCATATGCATTGGAGGCTGGCATCTCAGGAAGAAGTTCAGTGTCAAATGAATGCA
4359	CaTSNP7332	TTTTATATAGAATTTTTTTGCAAATTCAATCAACAATTTCATCGATGAATCAACATATAT[A/G]ATGATCGGGGTAGTTTTCAGCGCTACAAATCGATTGCATGGGTTTAGAGAATGCCGGTGC
4360	CaTSNP7333	TGGCAGGCTTTGACTCCTTATATGCAATCTTCTGTTGGGGACTGCGGTTCAAATAAATCC[A/G]TTGGGAGGCTGCTAGGACCTGCTCTGGGTGATCACAACCAAGGAAACTTTTCCATCAATG
4361	CaTSNP7334	AATAGAGTGCTACACGCAGTTCATTGATGTTGGAACAATGGTTTCGCCAACTGTCCATGC[G/A]CGTCGCAGCTTGTGTTATCTCATCAACGACATGCCTCAGGAAGCAATGAACGATGCGATG
4362	CaTSNP7335	TATTTTCTTAATGGCTGATTCAACCTCTTCAGGAAGAATGTGGTGCTCCATCCCAGAACG[C/G]CTTCACCTGCATGGGGCCATGTTGGCCTTGCAGTTTGGCTATGCTGGTTTCCATGTTGTC
4363	CaTSNP7336	TGGAACAGTGTGACTAACACCATCTCCAGAGTCCAGAACAATACCAGTTGTACGGCCACT[G/A]GCATAAAGGGAAAGCACGGCTTGAATGGCAACATACATAGCAGGAGTATTGAAAGTCTCA
4364	CaTSNP7337	TATTTTGTTCACAACATCCAAACCTCTTAAGGAATTGTTATTTGGAAATGCTTGTTGTTC[A/G]CTCACGATCGTACCACTATTATTTAGCAAAACTGACGCATCACAACCTTGAACAAAACAA
4365	CaTSNP7338	CATAAAGATAACCATAAGGCACCAACAGATGATTTTCATCATCCAACCCCCATGGTGCAA[G/A]CCATCACGAGGATCCTTCTGAGTTTTGACACCAATCATTAGAGTAGCCAGAATAGTGAAA
4366	CaTSNP7339	CAACCTTTGCATCTGGTAATTCTTTTGATAAAAGCTCCTTGAATGATTCTATTGTTCCTT[C/G]TGACTGAACAATGCTTGCAAGAGGCCCTTTGCTATCCAGATCTCCATTTTTCATCGGCAC
4367	CaTSNP7340	TGATGATGAGGATAATTGGTCTGTCGCATCCTCTGCTGTGTCGATGCGCACTGCAAGATC[G/A]AAGGTAACTCAGGGTTCGGCCCCTACATTTAGAAGCTCAGAACGTGCAGCAAAACGGAGG
4368	CaTSNP7341	AACTGCTCAGAAGATAGCGGCATTGGCTCGTCAGCGAGCACTGGACAAGAAACGGCCGTC[A/G]CCGTTTTCAGCTGCTGCTCTCGAGGCTGGATACCGATATTATGGAGGAAAACTTGATGAC
4369	CaTSNP7342	TCTTCGTCGGAATTCGTCTCGAACCGCCACATAGGCCGGTTCAACTAGCCGGGTGAACAC[C/G]GTACCGGAATCAAAAATGGTGCCGGCTCCGGTGGTCGGATTGAAAGCCAATGCAGCCGGA
4370	CaTSNP7343	CTACAGAATGGACTATATGGACTTTGTTCAAAATCACCGAGAGAGCGGTGCAGATATCAC[G/A]CTTTCTTGCCTGCCAATGGATGACAGCCGTGCCTCAGATTTTGGTCTAATGAAGATTGAC
4371	CaTSNP7344	ACCTTATGGTACTTTTGTTGGTGTTTATGATGGTCATGGTGGACCTGAGACTTCTAGATT[T/C]GTTTGTGATCACCTCTTCCAACATTTAAAACGATTTGCGACTGAGCAGAAGTCTATGTCT
4372	CaTSNP7345	CGTGATACCCTAAACCTTGATTTTATCAAGGAAATGTTGTAATTGTTGTGGAGTAACATT[G/T]CATGATTCTCATCTGCAATGCTGTTGCGCCTGGTACGGGTCGTGTAGCCAATGATGCGTT
4373	CaTSNP7347	TTGATTCTTCTGGTGTTGAACAATCGAACCAGAATTGTTAGGGTTTTGTATCGAAAATTC[G/A]GAAATTGAAGGAGATGGTGAAGAGAGATGGAGCGGAGAGTGGCCTGGAGATTGAAGCATT
4374	CaTSNP7348	ACAACAGCAACTGCGGTTGAATGGGGAATAGCACAACTCATTGACAACCCTGAGATTCAA[G/A]CAAAGCTTTATCAAGAAATTAATGCAATAGTTGGAGACAAAAAAGTGGAAGAAAAAGATG
4375	CaTSNP7349	TCGAATTGCTTTTGTGGAGTTCGCTGTGGCTGAGAGTGCAATTGCAGCACTGAGCTGTAG[C/T]GGTGTGATTTTGGGTTCAATGCCTATAAGGGTAAGTCCGTCAAAGACACCAGTCCGAGCT
4376	CaTSNP7350	TCTTTGAACCCTAATCTCCACCCAAAACCCATTTTCCGGGAATATTTTCTCCCGGAAAAA[T/G]AAGAAAAAGAAGCTCAAAATGCAGAATCACAGGCTCAAGCAGCAGCAGCAGCAACAACAA

4377	CaTSNP7351	GGAGAATCTCGACTCCACCATGCAACTTCTATTTATGGCTTGCCGTGGTGACGTTAAGGG[T/A]GTTGAAGATTTGCTTAACGAAGGCATCGATGTTAATAGCATTGATCTCGATGGAAGAACC
4378	CaTSNP7352	TATTAAAGCAAGGATCTTACAACTTTTCTCTTGTACGAACCAATTTCCTTTCCGAAGCAA[T/C]CTTAGGAAAGGCTCGTAAGTATCTTCGTCCGCAAGGGTGTTGTCATGAAACAGTCTTGCT
4379	CaTSNP7353	GTCAATTGCTGCTGCAGTGTTGTTGCGCCATCGCCTGTCGGTGGCACCAGGACACCGCGT[G/C]GAGCAGAAGATGTCGTTGACGCTTTTCATGAAGTACGGACTTATGGTGAATGTGCATTCA
4380	CaTSNP7354	GCAGTGACAGCGCAGTAGACTTGAACCACAAATGTAGCTGTAGCAGTTATAGGCAAATCG[G/C]ACGAACCTGATATGCGTGATGATTGAGCTAAAAGCATTACTATTTTTTTCAAAGCTCAAA
4381	CaTSNP7355	GTACATAGCCGAGCTGGAAAGAAATGTAAATGCTTTACAGGTAGAAGGGTCTGAAGTGTC[G/A]GCTGAGCTTGAGTTTCTTAACCAGCAGAATCTTATCCTGAGTATGGAGAATAAAGCACTC
4382	CaTSNP7356	CTATACTGCTGAGCCACTAGAAGGTATACATAAAGATTCAGAACAAAAGCTTGTAATTGG[C/T]GGAGAAGTTTGTATGTGGGGTGAGACAGCTGATACATCTGATGTTCAGCAAACAATTTGG
4383	CaTSNP7357	TGGTTCAAATTGCAATTAATGCACTTAATTTGTTACTGAAAACAGCCAAGCCAAAGCACA[G/T]CACAGAAAGAGAAAGCTCTGGATCTAAAGATGACTATGGAGAAAGATAGTAACAAGAACC
4384	CaTSNP7358	AGTAGAAAAGAAAATCTTTGTCTTTGCAGAAAGCTCATGATACCCTTCAACATGTTGCAG[C/T]CGAAGGAAGTCTTTGTGCTGCGCTTCTTCTGAATCAATGGCTCGATCTAGAATGCTGTTG
4385	CaTSNP7359	AAACGATGAATTGATGCTGGATGGAAATCCAAGACTGAATCTTGCATCATTTGTGACAAC[A/T]TGGATGGAACCTGAATGTGATAAACTCATCATGGCTTCCGTTAACAAGAATTATGTTGAC
4386	CaTSNP7360	ATTAATTGATACATCGTTCTTCTTTTATGTTGATAAAAGATTGCTACATAATTAATCTTC[C/T]TTGAAATGCCTTTCAATTGATCCGAGAAGAGTGGAATTAATATAATCTTGGGGACCGTGA
4387	CaTSNP7361	AGACCACGGCACTTCAAACTTTCTCACTCTCTTACTCATTCATTTCTCACCTAACTTTCT[C/A]TTCATCTTCATCTCCATCTCCTCCGATCACCACCGTCAGAATCATGGGATTTCAGAAAAT
4388	CaTSNP7362	GTGGGAAACTTGGTAAGCCAGAGATCTTTCCTGCCTCAACAGATGCACGCTATTTCCGCA[A/G]TCTTGGATTGCCAGCAATTGGGTTCTCACCTATGGCAAACACTCCTGTTCTACTCCATGA
4389	CaTSNP7363	TAAAGTTGCAAAAGGTCCCAGTTTTTCTTTGGCAACCCCATCGGCACCGCTGGGATTCCT[T/C]AAGAAATCACTGAAGACCAATCGAGCAAAACTTTTCTGCACAAGCTTGTTATAAGGAGAG
4390	CaTSNP7364	AAAGATGAGCTTCACTTGGGATGCAATGAGTGTGAAACCACCTGGAAATGTTAATCTAGT[G/A]AGTCAGTCCTCCATATACTCTCTCACTTTTGATGAGTTACAGAACACCATTGGTGGAATT
4391	CaTSNP7365	TAGGATGGGCTCTTCAATGCGCTGCTCTCTAGTGGCTTGCAGTTTCTCAATGACCCCTTC[T/G]AGATAACCAATGGCAGCTTCTTTCTCAGAATACTGTCGAGAAACCATCACAGCAAAATGT
4392	CaTSNP7366	AGAGGGACAATTGAAGGGAATTCTTGGATACACAGATGAGGATGTTGTCTCTAATGACTT[C/T]GTTGGTGATTCAAGGTCGAGTATCTTTGATGCCAAGGCCGGGATTGGGCTTAGCAAGTCC
4393	CaTSNP7367	ACCACAAATTATTGGAACCAGAATTAACCAACTAGCAAAAACTAAGGGTTATGACATTAA[C/T]TATGTGTTGGATGGTGAAAAAGGGAAAAATATCAAACTTGCAGCTAAGGTGTTTGATAAA
4394	CaTSNP7368	TGCTGTGTCAAGATTAGAAGCAGCATTGGCATCAACAGACATTAGAACCCCAAGAATACC[G/A]GTCATTTCCAATGTCAATGCTCAACCGCATACAGACCCTGACACAATAAAGAAGATATTG
4395	CaTSNP7369	ACGATTTAAATCCAGATCCCAGAGTTTTTCGCAAGCTCGTGACAGATCAGCAAGTTCATC[A/C]TGCGAAGGCTCTATTCTAGCACCAACTTCAACTTCATTGGAGTATTCTTGCTCATCAGAA
4396	CaTSNP7370	AACTGAGGAAGGTAAACCCCTTGTGTTGGATGTAGTGAGGCGAGTTGAACACCAACTATT[C/G]ACCGACGGGTCACGCAACAAGGAATATATTCCAATTGTTGGGCTAGCTGATTTTAACAAA
4397	CaTSNP7371	ACAGGATAGAAGAAAACTGAAGAATGTTATGGATCGTCAATTGGGAGATAAATATCCAAT[C/T]AAAGCTGCACTTCCTGTAGCTAAGCTTGCTTCAAAATGTCTAGCACCTGAGCCCAAAATG
4398	CaTSNP7372	GAAAGAAGATGTGCCATATTGAGGTGGCGGCAGAGGAATCCTGTGTATATCGTTCTTTCT[C/A]CGAGCGCTCAATATATTAAGTTCTAGTGTTGCCTTGTACTGGATAGGTATTTCCATCAAG
4399	CaTSNP7373	ATCTAGCCAAGTTGCAGTCAGTCTTGTTCGCCGTGGATTAAGAAGGCACAGCGACCCACA[T/G]CAAAGTGCCAGAGAGCTAGTAAAGGAGGCACTGCGCCTAAATACATCAGATAACCTCACT
4400	CaTSNP7374	ATTTGCAGTTCATGTATCCCATGAACATCCAACTTCCATAATTGGTTCAAGGCCTGGACC[T/C]CTGTTGGCTGGCTGTGGATTTTTCAGAGCCGTAATTAGTGGAAAAAGAGCCCTTGCTGCA
4401	CaTSNP7375	AAATAATGGAAACTCTTCCTTTTGTCAGAGTAATGTGTCAGTGGAAAATGGAACAAGCTC[C/T]GATGTCATCGAGACTGAGGCAAAACATTCAGCTGATAATAAGAGAAGCGAAGCGGTGATT
4402	CaTSNP7376	TTCATGTCCTTTTCTATGGACGCAGACAGCTTTCTTTATTGCTGCCAAGCTTGTGGATGA[G/T]GTTATTGTGTAGCCCACTGTGATACCAGCAAGCTTTCCATACAGAATCAATCCACAAATC
4403	CaTSNP7377	CCCATAGTTTAAAACTCCAGCAGAAGGATTCAATTCAATGTTCCCAAAACGCTTAAGTTC[A/G]CCCTTAGAAAAGCTTCCATCTCGAGTGCATTTCATAATATACATATAATCAGTAGGTTGA
4404	CaTSNP7378	TGATGGCACCGGGGTTCGGGATTACATTCATGTTGTTGATTTAGCAGATGGGCACATTGC[C/T]GCATTGCTTAAACTAGAAGAAGCTGATATAGGTTGTGACGTTTGTAACTTGGGGACAGGA
4405	CaTSNP7379	GATGAAGTTTATGATAAGTTGGCATTTGATATGGAACACATTTCGATTGCTTCTTTGCCT[G/A]GAATGTTCGAGCGGACGGTGACAATGAACTCGTTGGGGAAGACATTCTCCTTGACTGGAT
4406	CaTSNP7380	GAATAACACCCTCCCCTCTACTGCTAGAGCTTTATTAAGACCCTCTGACAGAAAGTCTAC[G/A]GTAACTGTTCTGTATGGCGATATCAAATTTACAGCAACCGACGATGATGCCAAAGTGGCG

4407	CaTSNP7381	AGTAAGAAAGAAGAAGAGTGTGGTTGTACGAACTTCACAAAAGATTCAAACCCTTGACAC[T/A]GTGCGAAATACTCCCCACCTTCTGAATCTACAAATCGACGATGGGTCAAGGAACTCCCGG
4408	CaTSNP7382	ACCAAGAATTTGGGCCCCAACACCAGTAAACCTGGGCCCATATTGGGCCAATTCGAAAGG[C/T]GTCAATTGTTTGCGAAACGCAGGATTCTCAACTGAATACATAAAGCAAAAGCTCTGTCTC
4409	CaTSNP7383	GCGTGGAACGTACCTTCCACTACCGGCTTCATTGTAATAAACATTGATACGCTCGAGCTG[G/A]AGATCGGAATCTCCGCTGTATTTTCCAGTGTGATCGATGCCGTGCTCGTCGCATATAACT
4410	CaTSNP7384	CGCGTGAACCGCGTGATTTTCGTAGTTCTCGATTCGGTCGTCGTACGGCGTCGTTTTAAG[C/T]GCGTGGATTTCGTTCGGTTTGCAAACGTCGTCGTATCGGAGGGTCGGTTTTCCCGCGAGG
4411	CaTSNP7385	GATATAAACTACCTCTAAATGTTTGCTTAATTGCCAAATGCTCTAAAGGGCAACCTTTCT[T/G]GGAGGAACAACCACATTCTTGTTTCCAGGAAATGCATTTCGTGATATGTCTACCAGTGAC
4412	CaTSNP7386	CCTGTTCTATTGGTTCATTGAAGCAGAACATCAAGATCCTACTTCAAAACCTCTTCTTTT[G/A]TGGTTCAATGGTGGTCCTGGTTGTTCATCTATTGCTTTTGGCGAGGCTGAGGAAATTGGC
4413	CaTSNP7387	AATTGACCCTCCATTGGACATCACGGTCCCATCAAAACACACACCCTGGCCAGATTCTCC[G/T]CCTGAACTCCCATCTAATGAAAAAGTTTTCGCTGGCACAGAAGAGCAACCGAACAAGTAC
4414	CaTSNP7388	GCGACGGAACATGGAATCACCCTCCGAGGCTAAGTCAGATTGTGGATAGCAGCATCTTTT[A/G]TTCTGTTGAGTTAATCTAAAAATTAGATCAAATTTCTCAACAGCCCATAACAGCACAATT
4415	CaTSNP7389	TGATTGTGATGATTTCAAGGCCAAACTTCTTTGTCACCAGGACAAGCCAGCAATTATAAA[T/C]GTGAATATAGGTACAACTGTGAAAGGAGCTGTGGATGATCTTGATCTGGTCATACAGAAG
4416	CaTSNP7390	AACCGAGAAGAATTTGCGTTTGCAGCTGACAACTGATGGGGAGAAATTCCAACAATTCCA[T/C]GAAGCATTGACAAAGAGCAAGGATGCATTTGAAACATTTAAACAAGAGATTGAGAAGACG
4417	CaTSNP7391	ACCACCAAAACCTTCTAATTCTCAAGGGAGAGCACTTAATTGGGTTGCTGATGCTGAACA[T/C]TCTCCAAAGTGTAGATCAGGGACATGTGAATTGTATACCTCAAATAGAAGGCCTGTCACA
4418	CaTSNP7392	ATTTAAGCTCAATGCACAGCAGTCAGTGGTCAAGCTACAGAGAGGGGATCCCAAGTATCG[T/C]GAGGCATGGCAGCAAATTTGCAATATTAGTAGGACTGAGTTTAACAAGGTCTATGAACGC
4419	CaTSNP7393	ATGTGTTTTACTTGATTCACATCATGCAGATCTTGAGAAGCAAGAAATAGAAGAGACCCA[C/T]CAAGGAGAAGATGAAGAAAACAAAGAAAGTTTCGAACTTTCTTCATATTCTGAAACGTTG
4420	CaTSNP7394	AGTGACTGTTCCTTGCTCCAATTGCACATTGGGAAGGGAGGCAGCCTTCTCTCTCATCCT[C/T]TGAATAAAACGCCCATTGTGAAAGCTTCTGCCAGCGATATCTGAGTGAAACTTTTCCAAT
4421	CaTSNP7395	GATAAGAAACCTTGACAAAGGATCAAAGGTTAATTGCCTGTCATAGAAATAGGATTTAAT[C/T]GAACCGAATAATCGCGATGAGACAGCGAAAACAGGTATGTGCTGCAAATCAGGATCATAG
4422	CaTSNP7396	CAATGGCCCTGGAGATCCATCCGCTGTTCCTTATGCTGTTGAAACAGTGAAGAATATTAT[T/C]GGAAAGGTGCCTGTTTTTGGAATTTGCATGGGACATCAATTGCTAGGCCAGGCTTTAGGA
4423	CaTSNP7397	GACATTCCAGAACAAATTCAATCAGCCGAAGGCTTCCGGTGAAGTAGACAGATCCTCGGT[A/G]ACAAAACAGGTTTCTTCAAGGGGGACTGCATCAAAAGTTCCGACATCTACAGCTTTGCGG
4424	CaTSNP7398	ACAAGCAAGATAAGCTTTATGTCAGCCCCAAGTTCAACCTTTTTTGCTCGGGATACCTTG[C/T]GTTTTAACATAGGTTCATATCAAACTGTTAATCGTCGAAGGGGATCAAGGTTGATAGTCA
4425	CaTSNP7399	GCTTACTGTAGGCCTTTGTCTAGCCAAAACCTTTTCAAAATCTGTTCTCGTAATAGGGGG[T/A]GGAAGGATCTTAGAAGCAAGTCCTTTTGTAGCAAGATCCTGCATTGTAGTTTGAACTGCC
4426	CaTSNP7400	ATACATTGATCTCACTTGACTTTGCTGTAGATATGGATGTTTCTAAAGAGTCTTTGCTCA[G/A]ATAAATCTGGCAGCCAGAAGTGTTGTCCACTGAAATTGTAGGAGCCGAACCCTGGCATTG
4427	CaTSNP7401	AGATTGGACGGCAAAAAGAGAAGATGCACAGCCACTATGTTTTGAGGTGACTGAACGAAC[T/C]GATAGAGGGTTTGCAGGTTGGTGGGGACGAGAGAAAGACGGGTCTATGCCTAACTTTTTA
4428	CaTSNP7402	CCTGTTGAAGTTACCTTTGAAATACCTATAACCCCAAGAGTTATTAGCCACTGAAAGGCT[A/C]GTGCAGTTCTTTTGGTTCATCCCTATATCCAAATCTCTATAATTCACATAGTTTTCCCTT
4429	CaTSNP7403	GACCGAGGAGTATTGCGATGGAAACCTCGCTGGAAGAACGGAAGGTTTTCTCTCTCAGGT[G/C]GCCCTCACAAATCTTTCCAGCGCCGTCGTCGTTTTAAAATCCTGCAGTCAGCTTCTCCCC
4430	CaTSNP7404	CATCACAAGCATCCTATGGCTAACTAAATCATTCTCACAACTCGAACCTTTTCTCCAAAC[C/T]CTCCACGCCAAACACGGCCCTATCATCACTCTCCGTATCGGTTCACGTCCTTCCGTTTTC
4431	CaTSNP7405	ATTTTCGATAGATATGGCTGAATCACAACCGGTCGCCCAGTGATGAGAACAACTACACAT[T/C]GAATAGAGCCACATACATTGGTGATGGTGCTTGGACCTGGCTCAGGTATTGTTAGATTCA
4432	CaTSNP7406	CAGCTGCTAACTATGTGAAAGCCGTCGACGAAGAAAGTTTCAATCCTGTTGAGCTTAACA[A/G]TTTTAACGGGACAGTCAAATCTCCACCTAATGGAAAACACATTATAGAATGGCATAATGC
4433	CaTSNP7407	CTAAAGAGATGTCTCCCGCATCCCGATCCAAGTCTAAAGACAAAAAGGCTAACAAGGAAG[C/T]TCAAAAGGCTTCTGCAAAATCCACAGGATCTGGTAATGCAGCAGCGGGTATTCCAGCCAG
4434	CaTSNP7408	GAGAGATGCTTTGCCTGATTTGCAGGATATGGTTTCGGATGAGTTTGTGATCACTGAGGC[G/A]AAGGGTGTGCTTCGTGGACTTGGTGAGGCTGCGAAAGGGACGTTTACTGAATTTGAGAAC
4435	CaTSNP7409	TTGGTTTTTAGTGATAGAATCGGCGGCCATTTGAACATTCTCATTATCTGATTTTACTAC[A/G]GTTTCTAATCCACTACTTGCTTTAAGAATGTCCTCAAGTTCAATTGGAAGTTTTGCATCA
4436	CaTSNP7410	CAGGTTCATTCCTAGTAGATCTGCTTCCAAATTTGCTCTCTTTGATATCAACACACCTAC[T/C]GAGGGACGCGAGGATAGTTCCAGCGCATACACCACTCTCCTCCGTACCGCTTTGTTTGGA

4437	CaTSNP7411	AGCTGTAACGATCTTTCTCGGATGCCAAACATATCATTCACTATCGGAGACAAACCCTTC[G/T]TCCTCACACCAGAACAGTATGTTCTAAGAACTGGAGAAGGCATTACAGAGGTCTGCCTTA
4438	CaTSNP7412	TCCAAGCTGGAATCACCCTCGATATCCCTTCTGCCCCCCTTCTGAGTTGAAAGAAAGACA[G/T]ATTAAGAGACAGACTACAAGAAACACCTTTTTTATAAAAACCTGAGATGAATTTAGGTAT
4439	CaTSNP7413	GCCCAAAACAACTTTAGTTTCGGAACTCAATATACGTTGAGCATCCTCCAATGTAGTAAT[A/G]TTGTGTATACCAGGTCCAGTCTTCTTCTTAATCCACGTCACTATAGCTTCTTTTGTTCTA
4440	CaTSNP7414	CATCGCTTCCAAGGTTACCTCTATTGCGTGGAAAGAAAATGAGCTGAACATGGTGGATAC[T/C]CCTGGACATGCTGATTTTGGCGGTGAGGTTGAACGTGTAGTTGGTATGGTTGAAGGAGCA
4441	CaTSNP7415	AACCTTATGTTCTTCGCTGTCAGAATGCATCATCTAATGTTCTTCCGATATATCCCATAG[T/G]TAATAACTCTAAGGATAAGACCAAAAGGTCTCCAAAATCTAGTTGTTGCAAGAACCATAA
4442	CaTSNP7416	AACCAACTATATTTTGTGGTGTTCCTAGAGTTTTTGACCGTATTTGTGCTGGTATCAAAA[G/T]CAAAATTTCATCTGCTGGATCACTGCAAAGTGCATTGTTTCAATATGCATACAACTATAA
4443	CaTSNP7417	ACACATGGATCATCAAAATGAACTGGCACAGAAAAGATGGGGTGTTAGTGTTGCTGCATC[A/C]AATCCTTCCAATATGTTAACTATGTCACAGTCCATGCAAGCAATCTCTCCATTTGTTAAA
4444	CaTSNP7418	TTCTCCTATCCATAAATTTGAGGCTCATAAAGCTGCTGTTCTTTGTGTTCAGTGGTCTCC[T/A]GACAAATCATCTGTATTTGGAAGTTCAGCAGAAGATGGTCTGTTGAACATTTGGGATTAT
4445	CaTSNP7419	AATTCAACCATTTCTGCAACGGTTTCTGTATTTTCCGTTGCCTTGGTTTTTTCCGTTGCA[G/A]AGAAATGGCATCTTCAGTACTCTCCGTAGCTTCATACACTATCTCTCCCTCTGCTTCACT
4446	CaTSNP7420	TATCAGTGAGTTGTCCATGTCTGACTTTGAATTAACAGTTGTAGAAGAATCTGGGGTCAC[G/A]TTAGGTTGATCTTCTTGCCTTGTGGGCATCAACTCCACAATATCACTTTTCTTGTTAGTC
4447	CaTSNP7421	ATTAGTGAATAGGAATCCAAAGTGGTCAAAGAAGTTGAATAATGATGAAGAAGTGGATAA[T/C]AACAATTTTGAAGAGGAAAATGTTCAAGTTGTGGAAGCTAGTCAAATGGTGAAATCACTT
4448	CaTSNP7422	ACCCTACATACTCCAACATTTTAATCAGAAGGGCTATTAGAGTCTTTCAATTTGCTGATA[A/G]ACACAGAGGATCTTACAGCAATGCATTGAAGCCCTTTGTGTGTCCCTTCTATTGTTCTTA
4449	CaTSNP7423	GTCACTCATACTATGGCACGCTCATCAAAACGACGCCGCTGCGGTTCGGAAGCTTCTTCA[T/G]GAAGATCCTTCGCTTGTCAATGCTACAGATTACGATAACCGCACTCCGCTTCACGTCGCT
4450	CaTSNP7424	CGAATACTTGGACAAGATTACAGGTGAGCTTGTTCAGGGCATTGTTGAAGCTGGTAAGAG[A/G]GCAGGGCATGCAATATGTGGTGGACATATAAATGGGATGTTTGGGTTTTTCTTCACAGAA
4451	CaTSNP7425	ACCTGAAATTATTAAATATCAGTGTCCTAATCTGAGGTTAAATTCGTCTGCAAAATCAAG[A/G]ATGACTCCTAAAGAGTGGTTCAATTCTAATCGGTTTGTCAATGTTGAATTTTCAGCTCTA
4452	CaTSNP7426	TTTATTTCCTCATTTGGCATATGATGATTAACATGTGTAGTAGGGTTATGAGTGATTATA[C/G]TAGGGTTATGAGTGATTGTAGTAGGGTTATGAGTGATTGTAGTTGATATATTATGCTCCA
4453	CaTSNP7427	TTCAAGGCCATTTGCCACTTGGTTTAGAAAATCCAAAGTAAACATGTGTAAGCAAACGTT[A/G]CTCCAACAAAATCGAAGACGGCCAGTTGCTTGATTGACTGTGGAAGCTAGTGATGGGTCC
4454	CaTSNP7428	GAAATACAGAAGCACATACCCCAGTGACTCCAAATCATCCCGCCGGCTTTGCTCAATGCC[A/G]AGATGAGTATTGCAGCTTGCATACCGTGCAGTCCCTGTTAAGTTTTTGTTCTCCCTGTAG
4455	CaTSNP7429	CGTGGGGGCACGTGCAGGTGGAATCCCAGACATAATCCCTGAAGACCAAGATGGCAAAAT[T/C]GGCTATCTCTACACTCCGCGCGATCTCGAAGACTGCTTGAGCAAACTAATTCCTCTACTG
4456	CaTSNP7430	ACCGACCAGTTCTTCTCCCCTTGTCAAGATGCTATGTCATCAAGGGCCAGTCTCGGCCCT[C/G]GCATTTCACTCAAATGGCCACTTAATGGCCACTGCTGGGAAAGATAAAAAAATTAAGCTT
4457	CaTSNP7431	TGGTAATCAAACACAGGATCTTCACAGTAGTCTTGTATTGGATCATCCAAGGGGCTATTC[C/T]CGTCAGCTTCATAAGTGCATTGTTGCTCATTCACAGGGACAAGCAGTTTTTGACGGAAAC
4458	CaTSNP7432	GTTACCTAAAGCTATTTCTCGCATAGATTCCAATTTTAAGCATGTTAAGGGATATTGTCT[C/T]TCTGCTCAACATGTTTTGGAGATTTCTGAGGGGAAGATAGTTGAAGTTCCTTTGGCACAG
4459	CaTSNP7433	AAGTGATGGTCATGATGATCATTTTCACCATCATGGTCACAGTCACGGACATGATCACCT[C/T]GGTGAAGATCACATTCATGAAGCGGGTTATATGGATGCATATGGGCTTGGTCATGGAGCT
4460	CaTSNP7434	TACCGGCCTTAATTTCTTGTTCACAACCGCAAAAGCAGCCTGGATTGGAGTCATTTCCTC[A/G]TATGGAATTGTTCCGGTCAACATTTCCCATAACATAAGCCCAAAACTGTAGACGTCAACC
4461	CaTSNP7435	CCTTCAAGGAAAGCCCCAACAAGATGACCCTTACTTACCCAATATGCTCCAAATGTGCTG[C/G]TATCCGAGAGATCTCCATAATAGACAACTTCCCCAATATTATCACCATAAAATTGCCAAG
4462	CaTSNP7436	AACTTGGCAATGAGATGGATAAGTCTGTTGAAGAATCACGAGTTGTTGAGAAAGCTGAAG[T/C]TGTACAAGAGATGGGTAGTGGAAATGAGGTAATTACTGAGAAGGCTGGTGTTGATAAAAC
4463	CaTSNP7437	GTTGGTTGGTCATACTCAAATACATCTTCCGATTATAACTCCGGCGATGCCTCCACAGCC[G/A]ATGATATGGTTTTGTTCTTGCTGAAATGGTATGAGAAGTTTCCTTCATACAAATCAAGGG
4464	CaTSNP7438	CTACTTAAAGAACCCTAAAGCTAATGAAGAGTCCTTTGCTAATGGATGGTTTCATTCTGG[C/T]GATCTTGCTGTAAAGCATCCAGATGGATACATAGAAATTAAAGACAGATCAAAGGACATC
4465	CaTSNP7439	AACAGAATCTCAAACTATGCGAGTAAATTATATTTCTTAGAATTCTGATACCATAAATGG[T/G]TACCCTTTTGTTTCGGGTCTTGAATATTGCGGCGTGATTTACGATATTTTATTGAGCAAC
4466	CaTSNP7440	GTACGTGGCTAGATTTACCGAGCATTCGGACTTCATGTGCAAGCACAGGAATGAAGCAAT[A/G]CTTGATGGCAGCGACGTTAATGGGAGGCGGGTGAAGCAAAGGCTTTAGTGTGAAGTGAAC

4467	CaTSNP7441	GGTGGTCGAGGAGAGTAGGGAGACGGAGACGGATGAGACGGAGATGGTGACGTGGCCTTT[A/G]TGTGTGAAACCGAATTTTTCGAAGAGGATCATGGGGCGGGTGTCGGAGTTTATTGTGAGG
4468	CaTSNP7442	ATGGTGACCTAGTGAAGATTGCAGTTGAGGTGGATTTCCGGATGAATGTGGTGGCTGCAG[T/C]TGAGGTAGATGAGGTGCAGGCTGAGAGTACTGAGGATGAGGAGTCTGCTGATATTGTTGA
4469	CaTSNP7443	TCAATCTAGAATCATGATTGACCTCAATCTCAGTGTGGCTCAGTTTTCAATGTTTGGTTC[G/A]ATATTGACAATTGGTGCTATGATTGGTGCCATTGTGAGTGGTACAATAGCAGATTATGTT
4470	CaTSNP7444	CACTCTATCTCCAAATTCGGTGAAACAAAGATCCGCATAATCTCGAAAATCATCGATTAT[C/G]TGAGAACTTAAGAAACCACCGTACTCATCTTCTAAAGCTTGAGGAAGATCCCAATGAAAA
4471	CaTSNP7445	TATGCTGGAGGCAAGGAAGCGTGGGGACTATGCATTCCGTGACAAGGATTTTAAGACAGC[C/T]GTCGATAACTATTCCCAGTTCATTGATGTGGGAACGATGGTCTCGCCTACAGTTTTTGCA
4472	CaTSNP7446	GTTTAGTTGAAAACCAAGACAATCATGAAGCTCGGGAGGAACATTACAAGGGCGATGATG[T/C]GTCTAGTGCTGTGGCTCATGAAACACATGCAACTAGCACTGAAACTGAAACAATTAATTT
4473	CaTSNP7447	TTTGGCTGAGAACGGTTCGCTCGGACCGGAGCCATTCATTTTCGGTTCGTATGCGGTTCG[A/T]GTGGTAAAGAATGAATTGCAACCGGTTCTTCAATTCTCTGTTTTCGATTCTACACTTGTT
4474	CaTSNP7448	TGTCGATGTATGAATAATTGATTGATAGAATTGATTGTAAGAAAGGGATCGTGGAAACTC[C/T]AAATGATTGAGAATTTGCAGAGTATTTGCTTTTGTAAGGATATCAAATAAGTATATTATC
4475	CaTSNP7449	TTGTTCAAAGGCTAGAGGAAACAATTCTGCAAAGTCTAGGCAATAGCCAATATGCACCTG[A/G]TTCGTAGACTTCATGAAATTGACTAAGATTATATGATCATTTAGACTTCTTTCCTTGTTT
4476	CaTSNP7450	CTCTTCTTCCTTTGACTTTGCGGGGGCCACTGGTGATGGAGAGCCTTCTACTCCCATCTC[A/C]GCTCTCAGGTCAGTGATGCTCTGCTCCAACTCATTTATACGATTTCCCATATCATCAATC
4477	CaTSNP7451	TAGAAAGATAGAAAAAGAGAAAAACTCAAAATCGCATCGATGTCAGATAGAGAAGAGAAC[A/G]ATTCTGACGCCCCTGAGGAATTCACAGCTGAACAGGGTGTAAAACTAGACGAAGAGATTC
4478	CaTSNP7452	GCTGAGCTGGTTGTCAGTGAAATCAAAGGTAGATGATGATGAAAGTGCCACGTGTACGGC[C/T]AAGATCGTACACACAAGAAAGAATGCAGAGAGGCTGCTTCTCGTTGTTCCCATGTTTGAG
4479	CaTSNP7453	ATTACCTCACAAATATGTTTTGGTTTTGCAAGTGATAAACTGAAACTAAATGGTAATGAG[T/C]TTATGACGAGATGCATATCTTAGTCCTCTCCAAAAGCATATATATTACACAATCACGCAA
4480	CaTSNP7454	AGAGATTCAACAAGGAACCACCATACAAAGAAGCCGATCCACCGGTGCTGGCCGCAACTA[T/C]CGTACCGGAAAAGTATCTCCGGCGATCGACCCACCATCTCCTAAACTTTCCGCTTGTGGA
4481	CaTSNP7455	CACCAAAAGCCTCATTCTAATGGCACCAAGAAAAGATGGATCAACCAAAAAACCAATGAA[T/C]AGAGGAGCATGGACTCCAGTGGAAGATCAAAAGCTAGCTCAATGCATTCAAACTCATGGT
4482	CaTSNP7456	AACAACTAAGTACATCAGCTATGGTGCGCTTCAGAGGAACACTGTCCCCTGTTCTCGTCG[C/T]GGTGCTTCTTACTACAATTGCCGACCCGGTGCTCAGGCCAACCCTTACACTCGTGGATGC
4483	CaTSNP7457	CGGCACATACGTTATTGGAGGATCCGCTTTTGGATGGAATTTCATCACTTTTCCAGCCGC[A/G]CATCCTGTCTATTATGGTAGGACAAAGGAACAATTTCGATCAGAAAAAGTGACCGAGTAA
4484	CaTSNP7458	ACATTGGCTCCATTTGGCGGCTTTGCATTTATAGCAGCTTGGGGTAGCTTGTTTTTCTGA[C/T]GACTTCACTGTTTTGGTTTGTGATGCTACAATATCTATAGAATGTTTGTTAATAAGCAGT
4485	CaTSNP7459	AAGAGCGATATCGTTGGTTCTCACCCTCTTCAATCTGCTCAGGAATCTGTAAGTAGAGTT[G/A]ATGAGGTGATGAAGAAGCAATGTATGGTCAATTTGTATGGAACTGCTTTTCCATTGAAGA
4486	CaTSNP7460	CATACCTTTGTGAACGGCACCGTTAACCCTAATGTCAACATAGTCACCGATATGATAAGT[C/T]CTGAGGTAGGTGGTTAGGGCAATGGTTCCCTTCTTACGGAATGGGCGAGAGAATGAATCT
4487	CaTSNP7461	CTGGCAAGCAATTCTTTTGGCTAGTTATTATCAATTAATCCTTGCAGACAAAGAATATTG[G/A]AAGATGATTCTTTCCTTAACAATAACAATATGTTACATTTAACGTTACATGTACCCTTTT
4488	CaTSNP7462	CCATTTCTCTCTGCTACAGTTAAAGGATTAGGGTTTTTCTGCAAATAACGATGTAGAGTG[C/A]AGTGTGAGGGTTTTTTAATACGGTACTAGAAGCAGTGGTTCTGGATGGGAATGGAAGGTT
4489	CaTSNP7463	GATCTATATGATGTTGTTGATTGTAAATCCATTGCTGCCCACATTAAAAAGGAGTTTGAT[G/A]CAAGATATGGTTGTGGATGGCAATGTGTGGTAGGTTCAAGTTTTGGGTGTTTTTTCACTC
4490	CaTSNP7464	TTCATAACCTTGGTACTATAGTGTTCTTATTGAATTTCCATATGGAAACAAGGTCATGTA[G/T]CTGTGAGATTAATGATGGTGCTGATGAAGATGAAGACATGGAACTCATTATATAATAATT
4491	CaTSNP7465	TTGTAATAGTCCCAACCAATATTCTGAGGGTTGTACAAATTGTATGTTGCTCTTACGTTG[T/C]TCGCACTTTGACCACTCACTAACATCGTTCCCATTAGAAACAACAACACCAACAAACTCA
4492	CaTSNP7466	CTGTCGCTCCGATACTCCGGACGGCGGCGATATTGAGCTTCGCGACGCTTTCGATCTGTA[C/T]GACCACGACAAAAACGGCCTCATCTCGACTGAGGAACTTCACTTGGCGCTTAACCGCCTC
4493	CaTSNP7467	ATTCACAAAATAAGAAATAACTCTGTGCATAAAATTCCCACCAGCCATGTTTTAACCTTA[T/G]CTTCAAACTTGAGGGGATAATTAAGATTGGAATTGGGGATTGTGATGTGAGAGAAAATTG
4494	CaTSNP7468	AGTATAATAAGATCTTCCAGAACGTGGTGGACACGGGATTCTGTTAGCAGAGAGTGCACC[G/A]TAAGATATGTAGTACTTCACTCTACTCCAGAATAAGGATCTTCTACTCAAATCACCACCG
4495	CaTSNP7469	AAAGAGCCGAAAGGCACATTTCACAGCACCATCAAGCGTCCGCCGCGTCTTGATGAGCGC[A/G]CCTCTCTCCGGCGATCTCCGATCCAAATACAACGTCCGATCCTTACCGGTCCGAAAGGAC
4496	CaTSNP7470	AAATTTGAAAATAAATGCTTAAGCTGCAGTCTTCTTCAGCTTAGCAAGTTCTTGCCTAAC[G/A]ACAGCAGCCCTCTTAATCTTTGCCAACATAATCTTGAACCTATCAAAATCATTGAGGGCT

4497	CaTSNP7471	ATTACTATGATAATCAAGGTGATTATGATGATGATGGTGTTGTTGTGAATCATCATGGTA[A/G]TCAAGATGAAGAAGAGGAAGATGATGAGTATAATACTAAGTTACCACCACATGAATTTAT
4498	CaTSNP7472	AGCAACACCAGATGCAAGTCCAATTGCGAACGTCTCAACATAACCGGGCTTAGTATCAAG[C/T]TTCATGTCAAGTATAGTGAGTCTATTGTTCACTGAATCCATTTTTCTTGAAGATTCATTC
4499	CaTSNP7473	TCAAAGGTAAACCGCTCCTCCTCGCCGCCATTATGAAACTCACTCTCTCTTTGCTCACAG[C/G]ACAACGAAGAAATGAAAAGCGGTGAAGTTAGATATTATGCCACCAAACTAAACCTTGTCT
4500	CaTSNP7474	TCAACTTTTAGTACAATTTAGATACACTCAGTCTAACAACATTTACAAGTCTTGAAGTAA[A/T]TATTACCATTCATCTGCTACATTTGTCGCTCCAAGAACAATTGCAATTGTTGCAACAACA
4501	CaTSNP7475	AGTGATCGTTGGCGGTAGTGCAAGAAGTTTCACCGCTAACAACAGAATTTATCGCTTACG[G/C]GTTAGGTCTTCTATGGTTGATTCCTCTTCCTCCTCCGATTTCATTAAGCGCGTGGAGCAA
4502	CaTSNP7476	CCAGGAGGAGCAAGACATTAAGGTTTCTAGTACAAAGGAAGATAGTGCATGAGACATTGA[T/G]ATTATATTCGAATTAGACATGTCTTAATAAAATTGTACCATGGCGTTATGCTATGCATTT
4503	CaTSNP7477	CATCCATCAGGGTATTCACATGCTGTCAGAACCTTTGTTTACAAACCTGTCAATGTTCCA[C/A]CTTATTGCATCTGATATGTACCTGCTTTTGTTCTGAATCTATTAGTGTTTGTGCTTGGTT
4504	CaTSNP7478	TCAAAGTGGCCTTTAACTCTTCCTCAGATACAAACCTTTCATCCTTCCCTGGTTCTAATA[A/G]TACATAAGTATCTGTTCTCTCATTTGCTCTTCTCCTCCTTAATGCAAAAACCTTTGTTTT
4505	CaTSNP7479	CAACGATGAAACAGACTCAGTTCAAGCACCAACAGATGAACAACCAGTTGAACTAAGGAT[A/C]CGGAGAAGATCGCGAAGACAAGCCAGACGACAGAGAGAGAATGGAGCTGTTTCAAATGGT
4506	CaTSNP7480	TGCATGTAAAAGGGTCTGAACCAAACCGACCGCAACAGGGTTCGAACCGGGTTTAATGAA[G/C]CGGGTACGGGAAAGTAATTCGATGAGTACGGACATGAAGAAAACAAGAATCAAGGCCAAT
4507	CaTSNP7481	AGCAGCTCCCCAAAGAGGAAGTGCAGCAGCTACTGCAAGCATGAGGAGAAGGAAGACAGC[T/C]GGTGGCGGGGCCTCTGGAGGAGCTGCAGGGACTATGCTTCAGTTTTACACTGATGATGCC
4508	CaTSNP7482	AAGCTATCCATTAAATAATGCGATAGAGTTGAGAATGACCAATCTTGATAATCGATTCCC[A/G]AGTGCAAAGGTGTTGTCTTAGCTTCAAAACAGTGATTGAAATGGAATTTCTCCTTGATAT
4509	CaTSNP7484	TGCACATTCCAGCGCACTATAGTTCCCGCGGCGCACTCAAACTAAAACGTCCACGTCACA[T/C]TCCGGCGCCGGCGCAACCGATTTAACATTAACGGCGGCGTAACGCGTAGAGAAATTGCGA
4510	CaTSNP7485	TTTATCATTTTTGTATACCCTTTTCTCTTAAGTTTTCATCTTTGTTATTTGGATCAACAT[C/T]GTTAGTTTTTTAGTTCTATTGGGCGGGTTTAATTTGAGTTAATAATTTTCTAGAGAAAAT
4511	CaTSNP7486	TCATAAATTCATATAATACGAAACTGCTTTCCAATACTACACAGTTCAAGGTGACAGCAA[G/C]GACAGTAAAGTTTTTCACATTTGTTGTAGATCAGTTCAAGACTATTTATTTACTACTAAT
4512	CaTSNP7487	TGAGGGAGATACCGATCCCGCCCTTGGACACCCAATTGAATATATATGCCTTGACTTGGC[C/T]GAGCCAGCTGTCTGCAAATATTGTGGCCTACGTTATCTTCAAGATCACCACCATTAGGAT
4513	CaTSNP7490	TAAAAATGCTCATAGCAAAATCTGGCAATAGACGGAAAAGATAAACATCGGGAATGTTTC[A/G]AGACAGCCTAGATACTTTCCTCAACCTAAACACATTACATGAATAGATTGGGCTTTTTGG
4514	CaTSNP7491	TGATGGAAACAACAACAACGAGTTGACTTGCATTACATTTGGTTTTTCTATCGGGTTTGG[A/G]TCCTCCGTGGCTGATTACTAATTGAAATTGGTCCTTCAAATCTATGTTTAGATCCATATA
4515	CaTSNP7492	TCCGCAAATTCCTTAAATTTGTTCTGACTTCTGAAGCAACCACATGTAAATCTAGATCCT[C/G]AACCAATATCTCAACATTCATTGATAAACTTGGAAGGCTTTCAGTCTAACCATACTTCAT
4516	CaTSNP7493	AAACCTCATGTTTTCTTAAGATCACAATGCAAAGTGAAAACAGGCATGACAATAACCACG[G/A]ACTTCAGGTAAAGTACTAAATTAAGAACCAATTGCAAATTTCGCTACAAGGAAAAACACA
4517	CaTSNP7494	ACTGGTCGAGAAACGCAACACTGATGAATCTCTGTAGCCTACTTCACACAGTTGAGGAAT[A/G]TAAACAACAAGCTTTCCTGTTTCTTCATTGAATTCATAGTTAGTTGCATCACGAGGAAAG
4518	CaTSNP7495	AAAGAGACCAAAGATATCGGCGTGAGAGATGGCGAGAGAGAAAGTTCTGAGCGCGTACCG[G/C]GCGCTGTTGAAAGCCACTCGAAAATCATTCGCCGGCGACACTTTGATGCTGAAAGGATCT
4519	CaTSNP7496	AATTCAAAACTCAAACTAGTCAATACAATATCGAACACAACTTTAGCCGCACCAATTCAA[G/A]CATCTTTAAGCCTCCAATTCAAACATTTTTTCAGTATAACATAATAATGAAGAAATAAGA
4520	CaTSNP7497	CTGTAGAACCCCTCTTGCTCTTTCTGAAGATATTGTTTCCAAGTCTTTCCACAGCAGACA[T/C]GGGAAAGCTTATCTCTTCAGTAAGGTTGTGAATGTTTCTGTTGGAGAAAAAGAGGACAGA
4521	CaTSNP7498	AAGGATTGGAAGTAGGACTCGAATGTGTGATTTGAGGTTACATTTGATGAAGGAGATTTC[G/C]GGTATGCCAACTTCTCTAAATAGGGAAACCCCACACACTGCAGCATCTCCTGAGGCAATG
4522	CaTSNP7499	TTCTTCTAAGGAACAATTTGATTTCTTCTACTTTGTTCAACAGTGGCCAGGAGCATACTG[T/C]GACACAAAGCAGAGTTGCTGTTATCCGAAAACCGGAAAACCTTCAGCAGATTTCAGCATT
4523	CaTSNP7500	CTCCCTCGACTGCAGCTGAAGGACCGAGAGAGAACATGATCGGCAAGAGCAAGAGGGGTA[C/T]AACAGAATATGCATCGGTAAGAACTTCCTTCCAAATCAAGAAAGAAAACCCTACCCATCA
4524	CaTSNP7501	CACAGGAGCTTGAGGACTTATGTCCCTAAAGGCAAGTACCTTATCGTCTTCATAAACGAC[G/A]GTAGAAGGAATCTCCTTGTTGATGATCTTGTCAAATATGGTGGGAGAATCAGAATCAGAG
4525	CaTSNP7502	TAAATTACCATGACAAACATGAAGTAAAAAAGCTTCAGTTAGGCAACAGCTGCTAAGTAA[A/G]GACAGTCTGCATAATATTAAAAGTTACAAGTGTCTTTCAAACCAACATATATTTGGATTC
4526	CaTSNP7503	TGAAACATGTTGTAATTTTTCTATCCCTTGCTCTGTAATTTCTCTTGCCGTGGATGCAAT[C/T]TCACATGCTCAAATAAAATCTGGTACTTGACGAGCATTTCAAATTTAATTGAGTGGATAT

4527	CaTSNP7504	AGCAACTAGAAATAACACTCAAATTTCTTGATATATCTATCACTTGCATAGAAAGGAAGG[C/T]GCCTAGTTCAGTCACTGGAAGCAATATCGCCTACTTACTAAGCATTACACATTGGCATTA
4528	CaTSNP7505	TTCATAGATTATATATATTTGGTTGAATATTTCTCCTCATCACTCTTAAATTCACTAGCC[A/C]TTTAATTACCTTTAGGCATTATTCCATTAATTATTGATCAAACCTTACATTAATTAATTA
4529	CaTSNP7507	GCACAGGAGGAGATGCAACTGGTGACTTGGCGGAAGGAGAAACAGCAGGAGCCTGAGAAG[A/T]TGAAGGAGTTGCAACGGGAGCATTGGTCGGAGACGAAGCAGGAGACGATGCCGGTGATTG
4530	CaTSNP7508	GAAATAATTGAAAAGCCTGTTGGGTTATCTTTGGCTGAGAAAGCTATCGGTAACAACTCC[C/A]GATGCAATGATTGCCATGCTAAAGGTGCAGTGCTTTGCGCCACTTGTGCTGGTTCTGGTC
4531	CaTSNP7509	TGGATTGAAGGTGGAATTAGGGTTTAGCAAATACAGCGAGAAATTGAATGGTCGAATTGC[A/G]ATTCTTGGATTGATAGCACTTTTGCTTGTGGAGCTTGCAACTGGGAAAAGCGTTATCAAT
4532	CaTSNP7510	TCTCATTAAAAAGAGTTCTTCAGCTGCTTTTGGCAATTTTTTGATGGTGGGAAAGAAGTC[C/T]TATCATACCTTTTCTAATCTTATTTGGATGCAGATCATCAATCAGAACTTTTATTAGGGA
4533	CaTSNP7511	AACTCTTCTCCTCGTCTGTACTCTCAGTGCTGCACCTCAGATTGAGACAAGATGTTCGTC[T/G]ACCTATGTTTGAAGATCGCTTCGATAATATTGCACCAACATTATCAGCGTAACTGTGGCT
4534	CaTSNP7512	CTTTCAATTATCGAATGCCACAGGGATGAGATTCGAGAGCTACCTGCCAAGGCTGAAGTG[A/T]TAGCTAGATCGGATAAGACTGGAATTGAGATGTTTAGATATGGAGATCACATTATGGGAA
4535	CaTSNP7513	CCTTTAGAGCGTAAAATACGGAGAGGTGGGAGCCACAACGGTGATCAGCGGAGCACCGAA[A/C]GTCCGGTCGGTGTTGAACTTCAACTTTATCTCTCTATCTATTTCCTCTCTCTCTCTCTCT
4536	CaTSNP7514	TGACAATTATGGCAGTCCCAGAAGAGGAGGTATTGATAGATCACCCAGTCCAGGTTATCG[C/G]AGACGACCAAGTCCTGACTATGGACGACCACGCAGCCCTGTGTATGATAGGTATAGTGGA
4537	CaTSNP7515	ATATGCTTAGTTACAGAGTTTGATCACTAATAGTGATTTGGACGTGAATGAATATGGTTC[A/G]CTGTATATTTGATGTAACTAGTTGATGATTTAGTACTTGTGTGTGATGGCAATGTTCAAT
4538	CaTSNP7516	AGGTTCAAAAAGAAGGTTATCATCAACAACATCAAGTAGAGGACTTGGAGGAGTTCTCAA[A/G]GAACAAAGGGCTAGGCTATACATAATAAGAAGGTGTGTGGTCATGCTTCTATGTTGGCAT
4539	CaTSNP7517	CTGGCAACCTTATTATTAGCTTTATCCGCTTCTTATCTTTGGATCGTTGAGGCGGAAACG[A/G]ATCAACTTACTCAACTCAAGCTTAATTCTCATATCCTTCAGGAGTCTATTGCTAAACAGA
4540	CaTSNP7518	TTTGCAAGAGTCCTCACAATGTTCTTAACGTGCTTCATACTGGACAATACGGCGTGTACG[T/C]TTCTTGTAAAGGATACGATAACCACAGTCACGGCACCGAATAACATCTCCAGACTTTATG
4541	CaTSNP7519	TTCCTCTGCCTCCTTAAGTAGCTTAACAAAAAGTGGGTGATTCAGGTAATTCAATGGTAC[C/T]GTAAATCTTTGTTGATCTTCTCCATGACCCACCTTTATTGCAATACACCCTTTTGTGTGC
4542	CaTSNP7520	TTTTTCAATAAGTGTATTTGCAAGTTCAAGCTTGTCTTCCTCAACAAGGGATGACCCAAC[T/C]GAATAACCCTGAGCCTTGTAAAAGGTAAAAATCATCCCTCCACCAAGCAAGAGAATGTCG
4543	CaTSNP7521	AGGGTTAGCGGTACGAAGAGGGGACACAATTCCATTGTTGTTTGTGATGCTGATCCTAGC[A/T]GCTTGTTCCAAGTGAGAATCTGAGTCAACCTCTGAACAATCTTTCCTTGGACTCTTTGCA
4544	CaTSNP7522	GATGGATAAAGAGGAAGATGAGTTTGTAGTTGTACTCATGGTTGTTGTTGTTGTGGGACT[C/T]CCTAGGTCTAGTTGAACCACGTCGTTTGAGTAACGCCGTAGACCTTGCATATTATTCTAC
4545	CaTSNP7523	ATCAAACGTCAAACACTCTGCAACTGTTTTATTTTCCAAACACTAAGAAACATACATTTA[G/A]CATAGAATTAAAATGAGACGCAGCGATGTTTTTAAGCAAGGACACCAGCTTCTTCCAGCA
4546	CaTSNP7524	AGCTTCCTCCTTGTTCCTCTCTGCAGTCTGCCCTGTTGTGTTAGCAGCTGCATGAGCTCT[G/A]TCTGCAGCTGCAGAGGCTGATTCCTTTGTAGATTGAACCCAATCTTCTGCCTTAGCCTGA
4547	CaTSNP7525	CTACTTACGTTCCTACTCACTGCACCCTTCCACCACCTGATCAAGTGATAAAGACTGAAC[T/C]TGGAAATATCTTACCGAGGCACATTTATTGCAATGTTCTGGAACAGGTAAAACCTAACGA
4548	CaTSNP7527	TTTGTTGATCGTGATCAGTACTTTGATGAATATAGGCGTCCTCTTTATTCACAATACTAA[A/C]CACATGCCATTTGTGTATGTGTTTCTTCCCACGCTTATCTTTTGTGATCAACTCTTGACT
4549	CaTSNP7528	ACGGAACATAAATTATAATACCAAAATATTCTCCCGTTACTTCAGTTTGACGAATAGCTT[T/G]GGTGCAACACATTTGTCAACACAATACCAATAATCAAAATACTGTCCAGTGCAGTGTTTC
4550	CaTSNP7529	GGACAAGGATTCTAAGTTCAGGTTAATTTTGGTTGAGAGCAGGATCCACCGTCTTGCGCG[C/T]TACTATAAGAAGACAAAGAAGCTTCCACCTGTTTGGAAATATGAATCAACAACTGCCAGC
4551	CaTSNP7530	GTTGTTCATGGTGTTTATTTGGTAACAGATTTGTATGATATTGAGAGTAAATGATTATGC[G/A]GAGCATGTATCAGAGGTTAGATCAGTATTGTTTACATCACAAACTCAGGTTTCTCCATAC
4552	CaTSNP7531	TCTAGTTTTCACATCCACATATATTAATAACAACCAAAGTTTAAGCAACTACAAACTTAA[C/G]ATAAATGATAGAACAATCACTAAACAAACACGTATGTTTCTTCAATCAGATTAAACGGTA
4553	CaTSNP7532	CGCCTATAGTCGACCTCCACGACTTCCTCCTGTTATAGGACCCTTGGTTGCCCTTTCGTT[A/G]TTGGATACATGGTGGAACTGGAGCTCCGATGATGATGGAAAGTGATGTGAGATATCATAC
4554	CaTSNP7533	ATTGGAACTTATGACCATGTGTGGGCAACCTTGGACCACGCCAACCAGGCTGTTTCCAAT[C/A]ATTATTCCCTTCTTTGATTTTAGCATAATCTCCACCCTTCTCCTCCTCTTTGCCAGAGAA
4555	CaTSNP7534	GTTTGATGCATCATTTGCTCCTCATAAATTGATTGTTATTGCTCGTGATGCACTTATTGA[G/T]GATGCACGTAAAGCAAGGTCACAAAGATTAAGGATCCACACTTAGTTTGTTTAATTTATT
4556	CaTSNP7535	ATCATCAACATCCACAAATATAACTTTGTGTTCACTTGACGTGAAATGAAAATTATCCTT[A/C]TTTGCCGTATCCTTCCTCATAGAAATATCCATCAACACCTGAAATGGGTTCTCTTGTAAT

4557	CaTSNP7536	GATTACTTGTACTTTTGTAAAAGAAATCTCGTTTTGTGTTGTCTTAAGTTAATTTTCATG[T/G]TCATGTGTGTTACGGACAGAAAGCATAGCCACGTTATTTAGTACTTTAGCAATATATTTG
4558	CaTSNP7537	TGATAAACCTGCTGCAACGGATGCAGCTGCGGCTCCAGCCGGTTCGACTAAGGGTGTATA[T/C]GTACCTCCCGGCCAGAGAGCTGGTGCAGAGAGGACTTCTGGGTCAGACATGAGGCGGAGA
4559	CaTSNP7538	AAAAGAATATGGCTTTGTTGTGATAATTCTCGTTCTCTATTGCTTTCTCAATTTCTACAT[G/T]ACCTTTCATGTTGGCAAAGCTCGCAAGAAGTACAACGTCTTTTATCCAACCCTTTATGCT
4560	CaTSNP7539	TTCACAACATACCCTAAGGAAGTTTGTCCGCAACAAGAGCTCTCAGATTATGCCATCCGT[C/T]AACAACTTTTTCTCTGATCCAAAATAGACCAAATGCCAAACTCTCTGAATCCCTAGAAAA
4561	CaTSNP7540	TTTATCGGAATTCCGATCAATAACAGATAAGAGCGTTGATTTCCTCATACTTTCCGATCG[T/C]CTTCACGAGGAAGAATGGCAAAGAGTTACTTCAAACAAGAGCATGATCTTGAGAAAAGAA
4562	CaTSNP7541	GCAACATGAAGAAAATGAATAAGGTTTTGTGATGAACCTTCTATGTTTGAATATGCCTTG[A/G]TCACTTTGTTGTACTAACTGTTTAGAAAGTGTTGCAAAAAGTGATGGTGGCTGCTTATAG
4563	CaTSNP7542	AGGTTGCAAACATGTTTCTCAACATGCTATTAAGAGGTGCAAGCACTTTGAGATTGGTGG[T/C]GATAAGAAGGGAAAAGGAACGTCTCTATTTTAGTTGCAGAATTAGAATTCTGCATTTTCA
4564	CaTSNP7543	GTAACTAAGGCTTAAGGGGAGCTGCTCCCCCACAAGAAAGCCTACTATTCATAAAATAAT[A/T]ATTCAGTTTGCACGGCTTTTGAACACATCAAGACCCATTTCAGTGGGATCAGTTGCCTGC
4565	CaTSNP7544	GATAAAGTTGATGGTTGTTTCCATGGTTTGGGAGTGGAGAGAGGTGGAGTGAAGAGAGAG[G/A]GAGAGAGAAGGAAAATATCGAAGATGGGTATATGCTTTGGTTGCTTCGGTGGAGGTGACA
4566	CaTSNP7546	TATAATAATTACTCAAGAAAAAGAAAAGCATCAGATAAACTGAAAACCAGGTTAAATACT[A/C]ATGTAATGTAACCTTCAAGAATTATCTTTAGCTGGTGATTCACTCTCAGTTGTTTCCTTG
4567	CaTSNP7547	ATGGGCCTTCAATGTGAAATTGCTTCGAGCTGCAGGATTCTTTGCAGGATCAATAGTACT[T/G]ATGAGAAACTATGGTGACCTTATGGCAATCTAAAAGTGGAAGACTGTGTTGTCTGTAATG
4568	CaTSNP7548	TGGAATTGGAGGAACATGCACAAGTACATGGAAAAAATCTTGCAATAACTTGAAGTGTTC[T/A]CCTTGGAAATCACACTAGTTATTTGTGTTTTTTTAAAAGTATTTCATTTTCTGTACCTAT
4569	CaTSNP7549	GGGTGGTGTGGTTATAAATCCAGGTTCTGCAACTGGTGCCTATAGCAGCATCACATATGA[T/C]GTAAACCCAAGTTTTGTCCTCATGGACATCGATGGTCTGCGTGTTGTTGTCTATGTATAT
4570	CaTSNP7550	GTGAAAGATAGACCCATTTTCAACTATTCTTTCAGATTTGAGAAAGAAATTATTTTTGTG[G/A]TGAAATGAGAGGAGATCTCTTCTTCTATTCGTGTTGTGGTTGGTTTTGGTGTTTGCATAA
4571	CaTSNP7551	GCCACAAGAGCTTTTTTTCGTCACTGGTATGCGTAACTCCAAGGGGTCATCTTTTGATTC[A/C]CGCAACTTCGCTTTTTGAACATTAATTAACCTCCGCAAGACCAGCTTAGCTTTATCAAGA
4572	CaTSNP7552	TGTTATTGATCTTGCAAACGAAGTCAGGCGTATTATTTCAGAAGAACAATGAATTCTGTG[C/T]TATCAGCACCAGACATTCTGAGACTTTTTGAGCAAGCCCTTTTATTAGAATTCATTGGAA
4573	CaTSNP7553	AGATTCGGGTCAATTGAAAGCGGGTTTTTAAGGGTAACGTAAGCTTTCTTGTAATCAGGT[T/C]TAGCAAACACGATGCCACCACGTTTCTTCTTCTTCCCGTCCATGTTCAATGTTCGAACTT
4574	CaTSNP7554	TCTAAATTATTTATACAAATATCAATAAAACTCCATACACAATGCTATTTCTGCCATTGT[A/G]TCGTGGCACTTGAGCTGCTTGGCCTCTGGCACAAGCACAACCTTCGGTAACTTAATCTTC
4575	CaTSNP7555	TACCGTCGACAAATTTAAGTTCCATTTGCGATTGCTTTTAAAATTGTCAGTACATGCTTG[G/A]GAATCATTTGTATCTGATTATCTGACCGATTGGGGATTCTCTTACTAGAGTAGCCCCCAA
4576	CaTSNP7556	TGGCATATGTAGAGAGGATGGTGTTATTTTGGATTTTGCAGGTCCTAATTTTGTTTGTGT[C/T]GACAATTTTGCATTTGGTGCTGCCACTCGCTATCTTCAAATACCCAAAGAAAAGTGTTGC
4577	CaTSNP7557	AACTGCTGTTGGACTGATTGCAGGTGGAATTGCGGCAGCCGTGTCTCGTAAAAAGTGACA[G/T]AATTCTGTCTATTAAACATATGATAACAGCTGATTATGTAAGAGGCAAATTTGTATGATA
4578	CaTSNP7558	AAGGCTTTGGTGAAGGTGGTGGTGGTTTAGGCTTGGGTGATGGTGGACATGAACCATTAG[C/T]CTCAGTCAATGTTGAGTAAGCAAGGAGAGAAAGCACTAAAATAATGGCAGAGAATTTGTT
4579	CaTSNP7559	TGGGCTTTCCTGCGCTGGGCTGGATTCCGGGCCACACTGGGTGGGGACACTTGTTGTATA[G/A]TATTATTGCTGCTGCTGAAACCTTCAATACTGGAGTTTGGGAGATGAGGAAAGTGGTGAA
4580	CaTSNP7560	CTTTTGTGGAGAAGGATGTTGCCAACCATATTGATGCAGCTGCTTAAGATTCGGTTGTCC[T/C]TGATTGTAACATGTGTTAAAAAATGACCAAGGCTTTGATACTAAAATGAAGTTTGAGAAA
4581	CaTSNP7561	ACGCATCAAGAAAAGGTGGACTCTTGAATCTCTTGGTTCATTAGAACCTAGACAAAGTGC[T/C]CTCGAAGAAGATTTTGAGGAGGAAGAGGAGAATGAGGATAATGAACAACCTGCTGATGAG
4582	CaTSNP7562	TATTGTTGTTCTTGTTGGGAACAAAACTGACCTTGTGGATAAGAGGCAAGTCTCCACAGA[A/G]GAAGGGGAAGCGAAGTCTCGGGAGCTAAACGTTATGTTTATTGAAGCTAGTGCCAAAGCC
4583	CaTSNP7563	TGCTTTGGAAGGAACTGTTAGTGGTGGTTCAACTATATCTCAGTACTTGATTGAAACTCT[A/T]CCTGGTTGGCAAGTTGATGATTTTCTCGATTCTTCTTCTGTTCCATTTGCTTTCTCTAAG
4584	CaTSNP7564	TGTTCGGCAAAAATCAAAGTAATCCAGGTATAGAACACTAAAAAACTACAAAGAAAGTGA[T/C]GAGGTAGGTACCTTCAAGGAACCAAAGATTACAAAAGATACACATCAATTTCCAATATAC
4585	CaTSNP7565	TTTTCATATATGGTCTGGTCTGTCTTTTTCTTTCTTCTCTGTATTCTTGTAGTTGGTTAC[T/C]TGCGGTTTCAGAACTATCAATTGTAGGTTTCCCCTGAGGCATCTGTATACGGAATCCTGC
4586	CaTSNP7566	CAGCCGCTCTGCACTCACAACTGTTGCGGGATAGTACTTGCCGACGATGCGGAGAATACG[A/G]ATTCCGTTTCTGTCGGAGCCTTGGAGAGTAATGAATTGTAATAGTTCGAGGTCGGAGAAA

4587	CaTSNP7567	GCATAGTCTAGATTTCCAGATATAGCCAAATCTAAGGCAATACGTTCGCCGTGAAATGAT[A/T]AACTAACGAACTCATTTGTATGTTCCCATGCCGACACCAGTCTCACCGCCAAGATAAGGT
4588	CaTSNP7568	ATATCAAATGAAATGCTAGTTAAAACAAACATTAACAACACCACAAAATTACACAATCAC[A/G]AGACACCCATACATATATAAATACACTATGTCATCTTTAATATACCACACAAGACTAATC
4589	CaTSNP7569	CATGGGTCAGGGTTTAGGGCTTCGTCCTGGAGTTATTGATGGGGCCATCAGTATATCTGC[C/T]GTTCAGTGGTTATGTAATGCTGATAGATCGTCTCACAACCCTCGGTTAAGATTGAAGGCT
4590	CaTSNP7570	TTCTCTTCTGTAACTATGAAAGTTGATACTTTCTCAAAAACATAGAAAATTGGACCTGAA[G/A]TTAAGGCTGGTCCAAACTTTGAAGATGCCTCAGATACTGCTTTTGATCCAGGAAAATCAA
4591	CaTSNP7571	GGGAGATCCAAGAGAGAAAATTTGTGATGCTGTTGGTGATTTGAAGCTTGATGCTTTGGT[C/T]ATGGGTAGTAGAGGTTTGGGTGCTATACAGAGGGTATTGTTGGGGAGTGTGTCTAGTTAT
4592	CaTSNP7572	TGATGGTGTCTGGGAACTATTTATTGCATTTTTGACAGGCGTGTTAAAAGACGAGTTTGA[G/T]GGAGTTTGGGCAGAAGTGATTCCTTCCATCTTTAGCTTGGTGCGGAGGTGGTTATGTATA
4593	CaTSNP7573	ATATTTTACCTGCTACAACCTGTTCTTTGGCTTTCAATACCTTTACAAATTCAAGAAGAG[T/C]GTTCTCTTTGTTGTTGTGCTGTTGAACGGCGAAACGAGCGAGGCTTTCGATCTCAGCAAC
4594	CaTSNP7574	AGTTTCTCGCCGGTTGGCTCTCACTGTGCTTTTTGGTTCTGCTGTTGTTGCCTCTAAGGT[T/C]TCACCAGCTGATGCAGCCTATGGTGAATCTGCCAATGTGTTTGGAAAACCAAAGACAAAC
4595	CaTSNP7575	AACAGTGGAATATAAGAACATCAGCTTCACTGTCTGGGATGTCGGGGGTCAGGACAAGAT[T/C]CGTCCTCTATGGAGACATTACTTCCAAAATACACAAGGACTTATTTTTGTGGTTGATAGC
4596	CaTSNP7576	TCGGTTTACAGCTCCTGGGATATAAAAGATCACATTTTCGAATAATCGGAATTTTTCAAG[C/T]AAAGGTTCTAGATAAAACCCAGTTGCAGACATCTGCAATGCTATTAGATAGGGTATACAG
4597	CaTSNP7577	CCGCCGCTTGATCTAACTCTGACACCATTTTCCGCCACCGGCGTCGGTGCTGTTTCTTTG[C/G]CTTTTCCGGTATCCCGTCCTGTTTTATTTTTCGACGCGTTTGCACGCGCTGATACTGCGC
4598	CaTSNP7578	CTTTTTGATGGAACCAGCTGATTGAGTGGCAACGTGGCGGTTGAGATCAACGAATCGGAG[T/A]CCGGCAACGGCGGATCGAGGATGAGAATGAGAGAGATTAGAAGAAGAAAGGGTTTTGAGG
4599	CaTSNP7579	AAGGTAAAGGGTCCACCAGAAAAGCCATGAATCTTTCTTGTTCATCCGAGCCAATGACTC[T/C]TGGTCAAGGCATTCAAACTTCCAAACACTCTCACCAAGATCATTGATTTCATTCCACCAT
4600	CaTSNP7580	TGTAAAAAGAAAAGAAAGAAAAGAATAATAAAAAGAAGAATTTGGTAATTGAAGAGTCGA[T/G]TCGAGATGATGGCAGGTCAATTGGCTGCAGCAATGACAACCATTTCTTCTATTCCTTCTC
4601	CaTSNP7581	CAATATCACATTTAGCACACAAAGCAGCTTCATCAGCACAACAAATCACTGTTGCTGCTG[C/A]TTTCTCACAAACATCACATTGAATTTTCATAACTTATGTGAGAAAATTTTAGATTAAGAA
4602	CaTSNP7582	GTACCCACCCTGCGAGGTTTGGCGTCTTCTGCATTGGTAAGTGAAGATTCTGCTTCATCT[T/G]GAACTTTATCAACGGGTTTCAATGATTTTGGCTTTTTCTTCTCCTTGGCAACTTCAGAAG
4603	CaTSNP7583	TATTTAATTATTTTATATTGCGAGGACCAAACCGGGCACGGATTTACCGACCCGATCGGA[G/A]ACGCTACTCTCCCAAACACAAGGGAAACGACCGGAATTATGCGCCGCCGGGACAATTTCA
4604	CaTSNP7584	CTTCTCATAGAACCCGTGTGGAATCCTACCAATATACAAAACCGAAGAAGTATTCTCAAG[A/C]GGTTTCTGTTCCGGAATTTTGCGACCAGGACCACCTTCCAACGGCAAAAAATCAGCACCC
4605	CaTSNP7585	CGATTTAACGCCGCTCTCAATTGGGAACAAGCACACGAACCTCTCCACGCAGACATCGAC[A/G]CGAAGAAAGTATGCGGCATAGGTCCTGGAATGTCATTCGCAAACGCAGTGCGGCGGCGCG
4606	CaTSNP7586	TGAATTCGAGGTTTGGATGGAAGAACCGTGTAATGCAAAGTTCGAAAACGAGGTTCACTA[C/T]GATCCAAACATTAAAGGTACCCTTGGTTATGGTCGGATCGGGGAATTATCTGGTATGTCT
4607	CaTSNP7587	TGGCTCGATTTGGCTTGGATGATATGAAGGCTTCATGAAGCATGGATTCATGATTAGAAG[C/T]CTATTGATGAATGGCTCTTGAATTTTCACTCTCTTATTTGGTTGAGTCTTAAAGCTCAAT
4608	CaTSNP7588	AATGTAATCATCTTTCATGTATCCCTTTTTCACACATGACAATTTGCTGGCGGAAGCGTC[A/G]TCATTGGTAGCTTGTACCGCCGCAGTGTTACTGTTATCCGCCATGGTTGAATAACGAAGG
4609	CaTSNP7589	CGTGAATCGGTTACCATCAACGGTTATTGACTGCGAAGAAGAGGAAACAGGAGTTTCGTC[T/A]CCAAACAGCACAGTTTCGAGTGTTAGTGGAAAACGAAGCGAGAGAGATGACGTGGACAGA
4610	CaTSNP7590	GTCGCTACAAGGAATGACAGCTCTAGTTACGGGTGGAACCCGAGGCATTGGTCATGCAAT[A/T]GTGGAGGAGTTAGCGGAATTTGGGGCAAGTGTGCATATATGTGCACGAAATCAAGATGAT
4611	CaTSNP7591	TTGAATAGCAAATGTCGTCATCTTTCTTAGGTGGTTAGAGACGCTTCGTTTTCACGTAAT[C/T]TGTGTGGCTTTCAAAGATTTCCTCATACACAAATTATTGGTCGCATGCCTCTTCGTTACT
4612	CaTSNP7592	TTTTTGTAGTCCAGGCCGGGATAATCGCCTCATATTAACTACATTTGCACTGGAACTTAG[T/C]TTACAGAGATGTGATGATAAAGGGGGAAGGTTTTTGCATTGAGTGGTTCCGTGGATGTGA
4613	CaTSNP7593	GGGTTTATGGATTTGGGTTTTGTGTTTTCTAAGGAAGATAAAGATTCAAAATTGGTTTCT[C/T]TGATACCTGGATTGCAAAGGCTTGGAAAAGAAGAAGATGACGAAGAACAGAAGAAGATTG
4614	CaTSNP7595	AATAAGTGTTATGATCTGAACTTCAGGTACTTTGACTCCCTTCAAAATTGACTTTTCAAT[C/T]GCAGTTACTTGGTCTACGAGTTGATCAAGTTGCAGAGACACATTATTGATGGCACGATTT
4615	CaTSNP7596	GACCATTCTTGCGCCCGACGATGGTGCCTTTTCGCAACTAAAACCCGGATACTTCAATTC[G/T]CTCGGCGAACGCCAACAGAAACAACTGATACAGTTTCATGTTCTTCCTGTTTATGTATCA
4616	CaTSNP7597	TTCGGAGGAAGGGTCGTCACCTAGAGGTTCTGTTTCGGAAAATGGAAATGTCAATGGTGC[T/C]GACTTCGCACAAGTAACAAGAGGATTTGCTGAACGGCCCGAGGCTCAAGACAAATCGGCA

4617	CaTSNP7598	AAGGTTAAAGATGACACTGCAAAATCAGAGGCCAAAACACTTTCCAATGCCATTAAGAAT[G/A]TTCATAAGAAACCAATTGTTGAGGATGATGAAGTAATAAGGATACTGGCAACAAGAAGCA
4618	CaTSNP7599	GGATTCGGTGGCTGGGTTAGTTGTCGCTAATGGTCTTCAAAATTTCTTGAGCCTTTACAA[T/C]ACCTTGATTATTGTCAGGCTTGTTCTTACCTGGTTTCCTAACGCTCCTCCTGCTATTGTT
4619	CaTSNP7600	TTCCACTAATCCGCAGCAGCTGCACTTTGCGGTGGAGACCGCTTCCACCTCCGTGGCCTC[G/T]ACTTCTTTCACAATCTTTCCAGCCATGTGTAAAGCCTATTTAATTGTTCCTAATAAAAAT
4620	CaTSNP7601	ATTCAACTGATAAAAACTCAGACATCAGAAACCAAATAAGTTGGAATAAAACTAGAAAAC[A/G]CTCAAGTCTATTGTAAACTCATTCAATAACGATTTAAGCAGCAATTGCTTGTTGGGCTGC
4621	CaTSNP7602	TAAGACCTTCAGAGCTGGAAGTTCGATTGATGAAGCAGATATAGTCAAGGAAACAAAGCA[A/G]TTCACATATAAAGATGGTGATCAGTTTGTCTTCATGGACTTGAGTACATATGAGGAGACT
4622	CaTSNP7603	CCACTGATAGACTGACTCTATACCCGGTACCCTGTTGACACAAGCTGCAAGCTTCTCTGA[G/A]ACAATGCTTTCAGCCAACTTCTTGCCTGCTTCTTTGTTGGGAACAGTGACATATACAACG
4623	CaTSNP7604	AGGAGAGATTTAGAACACTAACAAGTTCTTATTACCGAGGTGCGCAAGGGATCATTATGG[C/T]TTACGATGTCACTCGGCGAGATACATTTACAAATCTCTCTGAAGTATGGGCTAAGGAAAT
4624	CaTSNP7605	AGGTACTTGCTACAATATGTTAGTAACACCTGGCTCGTGTAATTGCTCTGGTGTTGCCAT[A/G]CTTCTGAAATGGGTGAGTGTACATCCTTGAATGAAATGTTATAAATTATTATGATTTTAA
4625	CaTSNP7606	CTTAGATTGCTCATACTTTAACTTTTCCTCTGATTTTTTATGCTCGTTACCCTGAAATTG[A/G]TTCAACGTGAAGAATCAACGACTAACCTAGATGGAGAAATGCTTAGTTTTTTGGTGGTGG
4626	CaTSNP7607	TGAATTAGCAACTGAATCATAGTGAGTTGTATCTCCTCGTACCACAGCTCCTATGCATAC[G/A]ATTGCATGATATTTGCCTGATTTGCCAAGCCTTGTTGCAACTGCGCCAATTTCAAAACAA
4627	CaTSNP7608	TCTCTTGGTGTTGATGCACAAAGGTGATGCCTATGAATTTGTAGTTGGAGGACAAAAGGG[A/T]TGGAGTGTTCCAAGTGACCCTAATGGCAACCCTTATAATCAATGGGCAGAAAAGAGCAGA
4628	CaTSNP7609	CTCTCCTAATTCTCTCTTAGCACAAGCACAATGATCCAACCATATCCCCAACACTTGCTT[C/A]GAAATCGGCTCAATATCGTTCCTGATGATCTCGTCCATCCGCTCTCCTCCATCATTTTCG
4629	CaTSNP7610	GTTAATCCATCCGAGATTCGCTCGTAGTGTTTGCAATTATCTATTACTTGATCTTCTGAT[C/T]GTGACACACACCTACATGTTATATATATAATGCGTAAATAGAAGTCAATGTTTGGCTCCT
4630	CaTSNP7611	TGCATCAACTGCAACATCGTCTGCTAAGGATTTTTCATCACTAGGAGAAGAAAGTTGAGT[A/C]AGAAAGAGGCGTTTCCATGAAGTAAGATCATAAGCAACAAGAGAACCATATCGGTTTCTC
4631	CaTSNP7612	GTCAAGAATTGAAGATGTAAGGGAGGAAATATTAAGGAAAAGAAGAGCTGGTAAATTGCC[T/A]GGTGACACAACTTCAGTCTTAAAGAATTGGTGGCAACAACATGCAAAGTGGCCTTACCCA
4632	CaTSNP7613	CTCTTCCGCATAAACCTTCTCAACTTTCCGATCCACATCGTGCAGAAACACGTGCGTCAC[A/G]CCGGAACCTTTTCTATTTCTCGCCATGACCGTCATCGAAAATATCGCCGCCATACGACCC
4633	CaTSNP7614	TATGACCAGTTGCTGCTGCCTGCTTGATATTGTCTGAAAACTGGAGCTGGCAATGTTGCT[A/G]TTTGGTTCAAATTATTGGTGCAATTACTGTTGCACCCTTGAGAGTAACCAAAAGCTGCAA
4634	CaTSNP7615	AGCTGAGTGCAATGATGTATACGGATTAGATGATGAGCTTTTGGAAATGGTTCCAAAGCC[T/A]GTTCTTGCTGTGCTTTTTCTTTATCCCCTTACCACCAAGAGTGAAGAAGAGAGGTTGCGA
4635	CaTSNP7616	AATCGCGAGCTTTATACGAACTATCCGCCATCGTTCTCAACCTTCTCCGTTCTCCTCCAA[T/C]TCCCTTCAATTTTTCCGATCATCCATTGGACCAACCGTTACGGCGTTCGCCTCCGCCTCA
4636	CaTSNP7617	TTCAGTTTGGCAATAGTGTTAGTGAAGATGGTGGAAACAAGACAAAGAGAACTTGGAAAC[C/T]TAATGTCCAGGATAAGAGGCTCTTTAGTTATATCCTAGATCGTCACATTCGTGTTAAAGT
4637	CaTSNP7618	TTGTGGCGGAGATTTTGGGGGAAGAGGAGAATCGGAGAGGTTTGGGGTGGAGGCGGTTGA[A/G]TTTGAGGGGGAGGGTAAAATTGGAAGAGAGGGTTTTGGTTTTGGGGGATAAGAGTGATGT
4638	CaTSNP7619	TGGATGACGACTCATATTTGTTGAGAGAAGCAGCTCTAAAACCCTAGATTTCTAACTGTC[A/G]ATAGAAACAGAAACAGAAACACCACTCTAAGTCACAGCACAGGTGTAACTCGTTTCGTAT
4639	CaTSNP7620	AACCAAGCATTCATTTTATAACATGAAATTAAGTGAAGCATATCACTCCCATGAAACAGG[A/T]ATTCAACATCTAAACCATTATCAGCTGCTCAACGATAGCTACTTAAAGGGTCAGCTGTTT
4640	CaTSNP7621	AAGGGCATTAATTCAACACAAAAACTAGAGGAACTCTTACAATCTTTCTTCTTTAGTCCC[G/A]CCAACTGTTTCTGCCATCAAGCTTCCACATGATTGAACCGACACCAAAAGGTATTACCTT
4641	CaTSNP7622	CAGATAAATGCCAACGAATGCTGGATAGTTGAGTCTCATGCTCAGGCAGTGACTCCTTCT[G/C]CTGTTCATCAATAACTACAACTGTAATGTCAGCTCCATACTTTTTAGCTAAGGCAGTGGT
4642	CaTSNP7623	TGCTGCTTTCTACAACCGTTTCCCTTACAAATCTTTCCAATTTGTCACGTGAAGATTGCA[G/A]TACATAGATTGAGAGGATGAAAGACAGCAAGAGCCCTGAACCAAAGAGAACAAAAAATCC
4643	CaTSNP7624	CCAGTGGTTAAGGTTTTCAAAAGATTTCATGACATTTACATCATAAACAAGGACACAGCA[G/A]TCTGCACCTCGGTAGAAAGCAACGCCAAGACTTTGAAACCTCTCCTGACCAGCAGTATCC
4644	CaTSNP7625	GCAGATTGGTCGAATGTGGGATCAGTTGAATGAATTGTACGTCAAGCATACGGGCCAACC[A/G]CTTGATGTTATTCAAAAATATATAGACAGGGATTATTTCATGACAGCTCAAGAGGCGAAG
4645	CaTSNP7626	GACCTTAAACGTGCTTAATGCGGCTAAGGAAGTTGGGGTGAAGCGCGTGGTTGTCACTTC[T/C]TCTATCTCTGCGATTACTCCTAGCCCTAATTGGCCTTCTGATGTTGTCAAGAGAGAGGAT
4646	CaTSNP7627	CACATTCAATTCTTGATTCAATATTAGTCAGAAACTACTTATCACACATAAAATTAATAG[A/G]CACATTGAAAACATTAAGCACAAGTCAGCATCATGATCATCATTCATCATACAAGTAATT

4647	CaTSNP7628	GTTGAGAGGGTGGTACGAGAAGGATGATGTTGTGGCTGAATGGAAGAAAGTGAAAAATGA[C/A]ATGTGCCTACATGTTCATTGCTTTGTCAGTGGTCCTAATCCATTCCTGGATCTGGCTGCT
4648	CaTSNP7629	ATCAAACTACTCGGAACTGTCGCACTTATATGACAAATTTAAAAGTAAAGGTTTGGAGGT[G/T]CTAGCTTTCCCTTGCAATCAATTTGGCATGCAGGAGCCTGGATCAAATGAAGAAATTAAG
4649	CaTSNP7630	AAAATGGATGGTGTCACTGTGAAGGTTGAGCCAGAGCAGTTTATGGTTCTAGAATCTTCT[T/C]CTATAGCTGCTGATTGTGTTGATGGCGTTAGTTCCAATATGGGTGATGTGTCAATGATGA
4650	CaTSNP7631	TTTCTTGAAAGATAACAACATTTGAACCTTGTCACTATCAAGTCCTATATCTGGACGTTG[T/A]AGAACATAGGTTTCATGTCCTTGTTCTAAACTTGCCTTAACTATTCTCCTCCCAATGTAA
4651	CaTSNP7632	TCCAAAGTCTTCAACATTATCTGAATCAGTGAGAAGATGGAAAACAGTGTGGCATATCAG[T/A]CTGATCTTAACTTGAAGGCTACTGAGCTTAGATTGGGACTTCCAGGGACAGAGGAAACAC
4652	CaTSNP7633	AATCTCTCTGTCAAGGAGATGCTGGTGCAGATTTTCAGATTCCTCATCTGTCTTGTTCAC[G/A]GACTCTTGAATTCTTTGGAGAAGGGATGCAGGGGAATTCAACTTTAACATTTCTTCTTTC
4653	CaTSNP7634	ATAAGGTGTTAAAACTGCATGGGAAGACCACACCATTCAAATCATGAAAGATAATTGAAT[C/A]ATGAGCTCATGAAACAGATTGTAGCGCTCTATCAAAGCTTTCTTCAGCTGCTGATACCCT
4654	CaTSNP7635	ATCAAATTAATATAATAACTAAATGAAGCATTCCTAAAGAAGAAATCACATAGCTGTTTA[T/A]GATGATGATACACTAGTGTTTCATATTCAACCAGAGGCCGGTTCAGAAGAAGTTGCATCT
4655	CaTSNP7636	TAAGAAACCTAAGCTTCCTGAAGAAACTCTTAGAGATTTCCTCTCTCATCATCCTCAAAA[T/C]ACTTCTTATATGAGTTTTGGTCAAGCTGCTGTTCTTGCTTATGTTAGACCTGATAACCCT
4656	CaTSNP7637	AGGATCAGCACCACCATTTGCAGTGAAGTTGTATAATCTGTTACTGAATAATTGACATAA[A/C]GTAGTACCAATAGTATGTCCCCCAACTAGAGTGACAAGATCTTGAGTGTTGAGACCCTTA
4657	CaTSNP7638	AGGTTTTGAACACATTCAAGCCAGCCAGTGGATTAACAAAACACACCAACAAACAACACA[A/T]TTATTATATCTAAGAGAAACTGCACACATCAACCCTTCAAAATGCTAAATTACATCATCC
4658	CaTSNP7639	GTTGGCTTCTGGTTAAGCAGCGCGACACAAAGTTTGGGCTTGAGTTCATGGGAGATGAAA[A/G]TAGATTGGAGCTAAGCAAATTCTCTCAAATAAATAAACCTGATGATGAGGATGATGATAA
4659	CaTSNP7640	GGAAGCCCATCAAATCCGTAGCTTTCAAGCAGCAATAGCAGTGCAGTCGAACACTGTGCA[C/T]GAGCTTAAAGAAAAAGCCCCTGTACCTGCTGCAATCCAGGAAGAAAAACCTACTGGAAAG
4660	CaTSNP7641	GTTATCTTCGATTTTCTTCATCGCAGACTCAGTAGTTAGAGGAAATTTAAGAATTTGGTA[A/G]TGGTCCAACTTGTTTCTTGGTGTCGCACTAATGCGAGGATACTTAGGGTTCCTTTCCTTT
4661	CaTSNP7642	TCATGCTCTGTTGGCTTCGTAAATTTGCAATTTTCATGGCTCACCAGTCGTTCAGATGGC[G/C]GACCGGCTTTCTTTAGCAGCTGAATGGTAGCTGTCAGATGCAGTTTCTGCTTTTCCTGGT
4662	CaTSNP7643	GTCAATTAATGTTGGTGATGCATCTGCTGCCAAAACCGGTGGCAGAGTTGGTGGTCAGGC[C/A]TTCAGGTCTTCCGCTCCTCCCCGCAACTCCGCACCAAGAATCAACAATAATAATTCCAGG
4663	CaTSNP7644	CATCACTTACACTGCTAAGGATGGAAAAACATCACAACTTGAGCATGTATTTATTCGAGG[C/T]AGCAAAGTCAGGTTTATGGTCATACCGGACATGCTGAAGAACGCTCCTATGTTTAAGCGT
4664	CaTSNP7645	ATCAACCAACTACAAACTCTATTCAGGCTTTTTCTAGAGCAATTCATCCTCCTCCACATG[C/T]TGATTTCCTCTATGAGCAGGCTTTGATGAGAAGAGAAAGCAAAGGAACAGGAGTATTTAT
4665	CaTSNP7646	CAGACCATAATGTATTGTCATATGCAATTATTCCACCCTTCTTCACCAATTTTAATAAAA[T/G]CTCATGATACTTTGTGAAGTTTTCCTTGTCAGCATCCACAAATGCATAATCAAATGATTC
4666	CaTSNP7647	AATTAAGTTATCACCAAATTTACATTAAAATCGGTCTTTTCTTCCACGTTGATTCGAAAC[A/G]AATTACGAACACTTCATTCCTTTGATGATTTGTTGATAAGAGACTTGTGGATATGAGGAA
4667	CaTSNP7648	CACCAACGGTGATGGTCTTGCCTTGTTTGATATTAATATTAGAAGGTGCATCATCATCAT[G/C]ATTATCACGAGGTTGATTGGAAGAAAGTGATTTCTTACTGATAATGCGGTATATCTCTCC
4668	CaTSNP7649	GGGTTTATCTTCTCTTCTCCGTTATACTCCTCTGTGAATTATTGATTTAGTTTGGAGGCA[C/G]AATGAGATTGGAAAAGTGTTGGTTTTGTTCTTCTACTGTATACCCTGGACACGGAATCCA
4669	CaTSNP7650	TTTGTGGCTTTGGCATAAGGTTAGAACTCAACCACATACAACCATTGTTGGCATCAATGC[T/A]AGTGGTCGTAGAAATTGGGTTGATGCTATGATGAAGGACAATGATAAGAAAAACATCCTA
4670	CaTSNP7651	CCCTATCTATGGCTACACAACTCCATCAATTTTACTCCACCATGTTTCAATCCAATAACT[G/C]ATTCAAAGCAACACTTGAACCTCAAAAGCAGCAACTCCAAGCTATTTATTGTTTCTTATA
4671	CaTSNP7652	CAATATAATTTACATCATTATATAAATCATAGGTAAGCTGAATACCTAGTTTATGTTTTG[T/C]CCATCACTTTATGTTGTAAGAAATCCTTGAATGGAGATTGTCATGAATAACCAAGGTACT
4672	CaTSNP7653	TGAGTCGGTTAGAACTCCGCCACACCCGGCTGATGGAATCTGGCGCAGAAGCGAACCGTC[T/C]ACGTTTAGTATCACTTCCTCATCGGCGTGTACTCTGACTGCTGCCACGTCAGCATCGGCT
4673	CaTSNP7655	TACTTACCTTAATAACCTTTAATTATCATAAAAACCACCATGTTTGAAACTCAATGGCAC[G/T]TCAAAAGGAGTAAAATCAAAAGGCCTCTGCCCAATATAATTACCAGGAGGATCATAATTA
4674	CaTSNP7656	AAGAAGCCCAAAACCAAACACTGATCTATATTGGCATGTCATACAAAAATAAATATATAA[T/C]CCTTTCAGCAAAAGTAAAATAACAATGTTGGCAGCCAGAAAGTTTGTGACTTAGTTTATC
4675	CaTSNP7657	ACTAATCAACAAACAAATTGATGCTGAACCATATTTCAATAGGAATATTTTATCTGATTT[A/G]GAACCGAAAATTCCACTACTAAAAAGATGGCTAGCATTGAATGCATTGTTGTTTAGAGCT
4676	CaTSNP7658	TATCAATAGCATTCTTGACATTTTGCCTCACAGGTTTCCATTTTTACTGGTAGATAGAGT[C/G]ATTGAATACAACCCTGGAGTTTCTGCAGTCGGTATCAAGAATGTCACAATCAATGACAAT

4677	CaTSNP7659	AGCATTGGTCTTCTGAGTCTTGTTGAATACAAGCTTCTCAAAAGTTTCCTTTGCACTATT[G/T]GTAGGCCAATCATCCGAGAAATACTTAACTGCCACACGTTTCCCATCAGAATCCAGAAGA
4678	CaTSNP7660	AACTGTCAGCAGGCTAACATTGTGTTCAGCACAAAGAGCTTTGACAAGTTTAACATAGTC[T/C]GGCTGGTCACAATCTTCTGCAAGAACACAGAGTTGTCCTGCATGTTTCTCAATTACTTTT
4679	CaTSNP7661	TGTTCAATCCGGTGGCTGCTTTCTTCACTTTCCTGTGATGTCTTCGTAGCTTCTTCCACC[G/T]GCCGAGAATCGCACGGCTCCATCGTGTTCTTCCTTTAGCTGTAGAGGCGAGGACTCGATC
4680	CaTSNP7662	TGATGATGTTGATGATGATGATGGTGATTTGGATGATGAGCTTGTTCCATGGGATATTGG[G/A]AACAAGTTAGGGAGACAGAGGATGAGGAAATTGGGGAAAAGGGAATTTACAAAGATGAAT
4681	CaTSNP7663	ATTTGTACTTTTTCCTGAGGATGTTAAGTTGGAGGAGCCTAAACTCAATGAATTTGATGA[G/C]AGTATGAAAATGTTGCTTAGGAAAGGACACACGTATGACACTTATATGAAAGTGTCTCCT
4682	CaTSNP7664	ATTTGGATTGCTCCAAACATAAGCTTGAACCGGCAAGTTTTGAGAATTGGGCTCCAAAAA[G/C]GAAACCTGAACGTCAGTTCTAATATATTCAACATCTTCAAATTCATCTAAAATATGGAGT
4683	CaTSNP7665	CATTGTTCACAACAAGTATTTTAAGGAAAATCCAGACTATTACAACTCTGCATACAATAC[T/C]AGATATGGAATTTACTCTGAGAAATGTGGACTTAACAATGTGATGATGTCATGGGGACAT
4684	CaTSNP7666	AACATAAACATTCCACAGGTTATATAATAATCGCAAATTAAAATAACAAACTTTAATTCA[A/T]AGATCTCAATAGAAACGGTCCAAACAAAATTTGAAAAACCGGATATAAACCTTAAGTTGT
4685	CaTSNP7667	AATATTTGGGACTGAAAGGAGCCTTTCGTACAGGTATGTACCCAGTTCCACCTCATACTC[G/A]TGTATGGCTTGCATTCCAATCCCAGATAAGTAATCAATTGCAGCTCCTAATCCAATTGCT
4686	CaTSNP7668	GTGTCGTCGTATTATTTCTGATAGCGAGATTATGAAGGAAGATGATAACAACTGGCCAGA[G/A]CCAGACCGGGTAGGGCGCCAGGAGCTTGAGATTGTGATGGGGAATGAGCATATCTCCTTC
4687	CaTSNP7669	TTTTGGATAGGAGGATTAGGATAACTGAGTTGGTTTTGAGGTGTGTCATCCTTTGTTTAG[C/G]TCTTCTTGCAGTTTTTCTTGTGGTCACTGATTCACAAGTTAGAGTTTTTTTCACCATTGA
4688	CaTSNP7670	ACAAATCAAACATTCTGCTGCTGCAAAGACAAGGAGGAGAGTTGTAAGAGCAGAGAGTGT[C/G]ATAGAGAAACTTGGCATCAAGATTGAGAGAAACCCTCCTCACTCCAAACTCACTCAACTT
4689	CaTSNP7671	CGTGTAAATTGGTTAAATCCATTGTCCGTGCATGTTTCCAACACCTTCCAGTGCCAAATG[T/C]TGTTGCAGAAGGAAATTGAGTTCCTTATTTTCATCCTCCAAAATCTTGCCATTTCTCTTG
4690	CaTSNP7672	AAAATCTCCTCCCTGACACATAAACTCTGGAATAATTCGATGAAAACCAGAACCTTTGAA[G/A]TGTAAGGGTTTCCCAAAATTTCCAATCCCCTTTTCACCAGTGCATAGAGCCCTAAAGTTT
4691	CaTSNP7673	GCAGCCTTGGAAGAAAGAAGAAAAGCCGGCGTTATTATCATCGTGACAATGATGCTGGAC[T/G]TGGTGGCAATGATAATTGGGAGGAGGAGGTGGAGGAGGATAGCCATCGTTGAAATAGCCT
4692	CaTSNP7674	CAGACCTACCATAGGTTCCACCGCCGTTGATAACGGTGCTGTTGAAGCTCCTCCCTCTGA[C/T]GATCCTCCTTCTTCTTCCCCAACCACAGAGGATGCACCTGACACCGGCAAGATCGAGACA
4693	CaTSNP7675	TTGGGACAAAGTTGGCTTGCTTTCTATAGTTTTGCAGTTGTTGACATTCAATGAATTCTT[G/A]AAGAAATGTGATGCTTAATATGCTTTAGATTCTTATTCCATGTTCGATGTTTATTTTGAT
4694	CaTSNP7676	TTCCGGTGGTGGAGGATCTCCTTTTTGTATCTCTGAAACATCAACCTCAAATGGCCAAAT[G/A]TATTTGTCTGTGTTATCTGCTCTGGCCAAGCTCCAGTCGTATATTCTTCTTTCTTCTGGC
4695	CaTSNP7677	CTTTCGTTCCCCATTGGATTACAAAGAGACACACATAAAGAGAATAATTGCATTACCTGG[T/C]GAGTGGTTTGTTAATCGTCAAAGCCATGACGTACTAAAGATTCCGGAAGGACACTGTTGG
4696	CaTSNP7678	TACTTCCTTTGAGATCTTCTTCTCTGCTTTGGGTTTCTTCTCAGCAGGGGCTTTCTCTGC[T/A]ACTGGTTTCTTCTCTGCTGGCTTTTTCTCTGCCTTTGCGGGAGCCATTGATGGGGATGGT
4697	CaTSNP7679	TCCTTGTGCAAGCTGGCATTGTAAAGAAGGAGAATATCAAGCTTCATGGTTTCTAAGACT[C/G]TAATCAACAGTGCAATTTAGCTACATGTTAAATATTTAGCATGCAGACTATAGTTCAATA
4698	CaTSNP7680	ACTATACTCTTCTCTTCCAAATAACAACTCAATTCATTCACAAACAAAATGGCTAGTATT[C/A]AGGTTGTTGCAATTTCTGGTTCTCTCCGAAAAGCTTCTAACAGCACTGGTCTCATCCGTT
4699	CaTSNP7681	CGAGGTCGACAAGATTCCTTCCGCTGGAAATCACGGGGGTCGTCTCAAAGCAAAGGGGAT[A/C]ATCTATTTGTCAAATATAAGAATGGTTTTTGTTGCTAAAATTCCTGTTGATGGCCTTATT
4700	CaTSNP7682	CGCCGCTAACAAAGACAAAGACAAGAAAAAACCCACTTCCCGTTCGTCTCGTGCCGGAAT[T/C]CAGTTTCCTGTGGGTAGGATTCACAGACAGCTGAAACAAAGAGTCCAGGCCAATGGACGT
4701	CaTSNP7683	TTGTGCAAGGAATTGAGACGGATTAAAGTGGCCTGTCTGAAAGGGGAACAAAAGGTGCGG[C/A]GGCCATGGTGGGCACCGGCATGCCTATCTACAGTGTTCTTAAAGGTTGCAAAATCCAACA
4702	CaTSNP7686	CCTCCGCACCCGATCCGAACACCTCACTCGTTCTCTTTCCAACTCAGCAACGCGCCTCCT[A/C]TTCTCCACCTCTACTCACCCCCACAACGATAACACTGTTAGGGAACAAATACAGTCGCCG
4703	CaTSNP7687	TCTTTTGTTTCCTCTTCTTTGTCTTTCACTGTCACAACTTCTTCTACCTTCTCAGCTTCA[T/A]CCAATGGCTTGGTTTCTTCAGCTGAAGATTCAACAACAGTTTCTTTTTCTTTGGTTTCTG
4704	CaTSNP7688	AGCATTGGGAATGTCTAGCCTTGAAGCTGCGAAAATCTTTTTGTGATTATTGATGAGACA[G/T]ATTCCAACGTTCCTGCGGTAACCTTCGGGAGGAGCTTCCATCGGAGCAGGTGAAGAACGC
4705	CaTSNP7689	ACACTGTAGTATGGCTACTGTGATTGGTCTTTGTTTGAGAGTTAAGCTAAAGCACTACTT[C/T]CCTCCTCATTACAAAGTGGACATTAAAGTGTCTCCAGGATCTCATGCCTCTGAAGAATCA
4706	CaTSNP7690	AATTTTCTATGATCTACTATCCATATAACAGATTTGCATTAATCAAATTATTGACTCTGA[T/A]ATGCTTTGTAAAGAGGGTCGCCAGGATTTTGACCTTTGAACAGCAGCAAGATTTGTGGAC

4707	CaTSNP7691	TAGCGTGAAAGCGCAGTTTCCCACCGTAAAGAAGAACAATTCCATGGCGGAGTCCACCGC[G/A]CCTAAGTGGGCCCAGAAAACCATCTCTCTACCTCCTTACAGACGCGGTTGCCACCTCGTT
4708	CaTSNP7692	ATAAGCGGCGTGGAAAAGAATGAGAGCACCAAAAACACCAATGGTTAAAGCTACAGCCAT[T/G]ATTGGTACAGAGTTTGAAGATGGTGAGTGAAGTGAGTAGATTACTTGAAACTTGCAAGTC
4709	CaTSNP7693	ATTTGGATGTGAAATTGATGAAGAAACATCTACTAAGCTGGAAGGGTTGCCTGGAGTTCT[G/C]TTTGTTCTCCCAGATTCATATGTTGATCCTGAACACAAGGACTACGGTGCTGAGTTATTT
4710	CaTSNP7694	CTAGTCCCGAGGAACGCGCAAATGCCACACAAGTGCAGGGTTCATACTTGCCCATTGTGA[C/G]GGAGAAGCCATCTGTTGAGCTTGTTAAGGTTACAGATGAAATGAAGGCATTCAAAGCGTA
4711	CaTSNP7695	AAAAGAATGGATTATAAAAAACAGGAATAAGTGGATCTAAAACGCAATGTCAGTGAAGAA[T/G]TGAAGTCTGGAAACCCTATGGAAGCAAGGAAGATGAGGAAGACGACTTGTCTAAGCTGTT
4712	CaTSNP7696	AGTATCCATGTGACAAACAACCATAGCTTCAACCTTATCACCATTATCACCAACAAGTGA[G/T]ACTCTATATATCCTATTCTCACTTTCTTGACTATGGCAATAGAAAACAGCATAAGGGTAT
4713	CaTSNP7697	TTCGATGTCAGTGACATCATAAACAATAATAATTCCATGTGCTCCTCTATAATAACTGCT[A/C]GTTATAGTCCTGAATCGCTCCTGTCCAGCCGTATCCCAAATCTGCAGCTTGACGGTTTTC
4714	CaTSNP7698	AGCTATTGCATAAACTGCCAGAAGACTAGCTGGACCCTCCAAAACTGCAGTTAGTCCTTC[A/G]ACAGTGACGACCGCTGCATCCCTTCCTGCATACCTTGAATCCCCTTTGCTATACTCCTTC
4715	CaTSNP7699	TGAGCGTTGATCAACATGCTTAATCCATCTATTGGAAATAGCATGTCCCTTATGATTCAT[C/A]GCTGAAACAGAGTTATCACAACCGCTAGCCGAAAAGTGCGCCATATGAATTCCCTTCCCC
4716	CaTSNP7700	TTTGCCTGTGTTAAAAAATGACTGATTGAACTTTAGTTAATGCACATCTTTCGAATCATA[C/T]CATTGTTGTAACTGTAAAAAGTGTCAGATAAATCATAAGACGCTCTTATAAGTTATGATA
4717	CaTSNP7701	CAACAGTCTTCATTAGTTTCTTTTTAGTCAAAATAAATCATCTAACAAACTTTAGTCCAC[A/G]TTCAGGACCTTTGGAACTTTAATGTAAGGCTCCTCATAGCTTGGAACAGAAGCGATGATG
4718	CaTSNP7702	ATCTGTAGAGGCCATCCATGAGAATTTGCTCTATCTGAAGGGAAGGGAAGCGGAGATGAG[A/G]ACCGTGAGTGAAAGAACAAATGGAAGAGTGGCATGGTTTAGTATTATGTCATTAGGCATC
4719	CaTSNP7703	TTTCTTCAATGGATCCTTACTTGACCCTCTTCCTTCCGAAGCACTACGAAACAACAACAA[G/A]TCACTCAATTTCCACCTCCTCGAACCCTTCGAATTGTTCGATTTCGTTTCCTCTTTGATG
4720	CaTSNP7704	TCGAAGAAGCGATGAAGTTTTTCTGTTTTTACTTGCAGAAGAGAATTTATCAGGGAAAGA[G/A]TTTTTGAATTGCATTTAGACTTGTTTTCCATAATTTATATATTTGTCAATTTCTTTACAA
4721	CaTSNP7705	CGGACTTCGCTCTCTCTCCATATTCGGTCCTTTATCTTCAGAATTTAACGGCTTTGATCA[T/C]GGTTTCACAATTATTGACAATGGCGGCCGAGTCTATGCTATGAGACACGGAAGACGAGTG
4722	CaTSNP7706	TAGAGGTTCTCTTCAGTTTGTTCAATACACAAATGGGAATTATACACATGTAACTGGAAA[G/T]ATAACAGGGTTGTCCCCAGGTCTTCATGGATTTCATATTCATGCTCTCGGTGACACCACC
4723	CaTSNP7707	GTCCTTAACAGCTGGACCCAAAGTCACGGTTTCTTCCTTTGGCTCCCTAACCTTCCTCCT[A/C]GACGACATGGTTGATTGAAATTGCGAAAAAGATATATGAGATTCGAAGAAGTTAGGGTAA
4724	CaTSNP7708	TGTCAACCGATATATTGATGAAGGTGTTGCAGAGCTTGTCCCTGGGGTTCTATTTATTGA[T/C]GAGGTGCATATGCTTGATATGGAATGTTTTTCCTATCTTAATCGTGCTTTAGAGAGCTCC
4725	CaTSNP7709	TTATATAGATACAGATCCAAAGTCCAACAAGAGTCCGTGAGTGATGGACTAATATCTCCA[C/A]TTCCCTATATAGTTAAAAATCTCTATCATATATATTCTCTCACAGCACAGCACAGCACAC
4726	CaTSNP7710	GATGTGATATATACTGTGTTAAAGGCACTGTTTGATTGTTCGTGCATTTGAAAAACAAAA[T/C]GTGGTTGATCTCTCTGCCTGAGCAGCATGTATGCTGTGGTTGAGAATAACAATACCCTGT
4727	CaTSNP7711	TTTTTGTTTTTGAAGGATGGTGCTCCATTGGAGAAGCTAGTTGGTGCCAATCCAGAAGAG[A/G]TTAAGAAAAGGATAGATGGATTTGTTCAGTCCACTCATGCTTCAATTGCTTAGTACAAGC
4728	CaTSNP7712	TGCTCGTGCCTCAACTTTGTCGTAATAGATGCTTAGTTTTTTGTTTGGGTTGCGTGCTGT[G/C]AAATTGAGGACCATGTTGTAGTGTAGTGTGTTGTTTGTGTAATCGAATTGTGTTAGTTTG
4729	CaTSNP7713	AATTATGGGATGAATCATACGGCAAGGACCACACCATGGAGCCCAGAATTCGACCAACAC[A/G]GGAACATCAGATTCGAGGACAAGGGATTGCCAATTTGCATCAATAATAGAAACCACTTCA
4730	CaTSNP7714	AGCGATATCGGCACCGGTAACGTTGTTGGGTGAAATGGCGAATCAGGGTGCTAAGAAACG[C/A]AAGGATGAAAACGCTCGTCACATGGCTAGGCTCCGCCGCATCATCATCGCTTGCAACGTT
4731	CaTSNP7715	TCAATGATTCAACATCCCTTGAACCTTGATATTTAGCAACTTCTTCACCGTCATAGAAAA[T/C]CTTAAAGGTAGGATATGAGTGAATATCAACTTTAGAACAAACAGCTTTGTCGGTGCCGCA
4732	CaTSNP7716	TTCCCTTAAGTTCATGACATAAGATAACGCTGTCCAGTGAATATAGTTAACACTCAGCAC[G/A]AATAATCCCATAGACAAACAGAGTACATAGCATAGATAAATCTACATTGAGGATTTTTGG
4733	CaTSNP7717	GAGGTTGATCCGGAGGAGTTCATCATGATGATGAACAGGACAGGCTTCCGTCACTAGTTA[C/T]TATATCTACATGGAGTAGTTACCTTTTGTTTGGGTTGACTATATGAAAATCAGAAGAAGC
4734	CaTSNP7718	TGATGAACTTACTACTCAAAATGATAGCACGGTAGCAACCATGTTGGTGGGAAATAAGTG[T/C]GATTTGGAGAATATCAGAGAAGTGAGCACAGAGGAGGGAAAAACTCTTGCAGAAGAAGAA
4735	CaTSNP7719	AATTTGTGTTTCATAGAAGACTGGAATGCCAATCCCAACAGCAACACTCAAGACACCAAC[A/G]GTTATAGCCACTACCGTGTTTTGATCAAGTTCAATAGCTCTAATACGTGGATAAAATGTG
4736	CaTSNP7720	AGCGGAGGAACGAGAAGAGAGTGAAGGAGACAATGACGATGATGTAGACACTGATTACGA[C/T]GAGGATGAAGATGAGGATGATGTTGAAGGTGATGAAGACGGTGATAATCAGGTTGTACCT

4737	CaTSNP7721	GGTTACAATTTTGTGAAAGCAAGGAAATTGGGGTCAAGAAACACAAATACAGAAAACACT[A/G]CACTAAGACATGCCTTGCCTGGCAGTAAATATAATCCAAACACATGAATTGAAATCAAAC
4738	CaTSNP7723	AACCGATAATGCTGTGAAGAATCATTGGAATTCGACGCTTAAGCGTAAGGCGAGTGGTCT[C/T]GGTGGTGATTGTTCTGTTGTGGTTGCCGGAAATGTAACTGCTAGTAATGAGGGGGGAAGT
4739	CaTSNP7724	CCTAACATGTACCAAAAACACGACCATCTATTATTCTGAGTTAAGATAAATTACAACCAA[A/C]CCTATGAAGACAACATTTCGAGAAGGATTGAAAACCATCCATGAAGGCATGAAGGAAGCA
4740	CaTSNP7725	ATGGATATTGGTATTCTGCTATTGCCTTTGCCGCTGAACCAACAGCTTTGCCTGCCATAA[A/C]CCCTTTTGTCTTCTCTGGACAGATGTTCTAAAAGCTTCTCGCTACCTTCCTTCCGACGAC
4741	CaTSNP7726	TCTGGATCATCCTTACTAATTCTAGCAAATAAACAAGATATTAAAGGCGCCCTTTCGCCC[A/G]CAGAAATAGCTAAGGTGTTGAACTTGGAAGCTATGGATAAATCTCGGCATTGGATGATAG
4742	CaTSNP7727	CAGAATTGCGTTCGAAGGCAATGGAAGAGAACCAGAAGCATCGACATTAGCAGGATAGCC[A/G]ATCAAGCTGTGTTGCAAATGTGGAGGAACCTCGGGTGGATCATGTGCCTCATCTTCATTC
4743	CaTSNP7728	AAGTATGAATCAACAACTGCCAGCACCCTGGTTGCTTAGAGGATGCGCTGATTTTCATGT[G/T]ATGCTACTTTGTAGTACCATCTGTAGTTTTTTTGGTTGTCTAGATAGTTTATGTGGTACC
4744	CaTSNP7729	CTGACCTTGGAATATCAATGACAAACTCATCATCACCTCCTGCACCACCACCTGCATTGA[C/A]TGAGGATAGTAAGAATGAGGCTAGTTCAATTCTACAAGGAAGATCCCTTTGGTTGGTCTT
4745	CaTSNP7730	ACCAATGGGCATTTGAATGATTATATGTTACAATATTAGATACTCAGTGAAGGGTCTGTC[G/A]TGTGGGTTTATGTTATGCTTATTTAGCGCATCAAAGGACCCAATATAGATTTATCTATGC
4746	CaTSNP7731	GGTGAGTATGAGGTGGGTTGAGGCCATCTGGGGCATAGTCAATCCTTGACAGAGAGACAC[T/C]AAGAGTGTTGAGTCCTGGGATTTTCTGAACATTGGCTCCAGTCACAACTGAGCCAAATGT
4747	CaTSNP7732	CATCACTTCTGTAAACACACCCAATCTAAAGTTTCCCAATAACAACGGAAACTTCTTTCT[C/G]TTACCCTCTCCCTCCTCCAATCATCACCGTCCCCTTAAACCCATTTCAGCCATCATCAAC
4748	CaTSNP7733	CTCAAAACCCCACCGCTACCATCTCCGGCCGCCACGTCATCAGATCGGACCTCCTCCATC[C/T]GTTGTGGTCAATTtCATCTTtAAGTTTTGTTTCCAGGGTCAATTTGAATTTCGAGTATGA
4749	CaTSNP7734	GGAACTAAATGCCACCTCACCCTCATTAATAAGTCCCACGTAGATTTTGCTAGCTAAACC[C/T]GAAGCCGTTGAGAGTGTAGCCAATTCTGCGTGGCTTCCAACGGCTTCAACAAGTTTCTTT
4750	CaTSNP7735	ACACCCTTCTTTCCTCAGAATGAACCCAAGAAGGAAATCCATCAGAATCCAAAGAAGCAA[T/C]TGGTGAGTTAGAGTTATCTGACAACAACATACGGCCACGTCCAAATGTCTGCATTGTAGA
4751	CaTSNP7737	AAaGGAAAAACTCATGGCAAGATGGTACATTGGGAACCCAATGTCCCATTGCACCAGGAA[C/G]CAACTACACGTACAAATTCCAAGTTAAGGATCAAATTGGTAGTTACTTTTACTACCCAAC
4752	CaTSNP7738	CGGCCTGGATCGCCGTCGATGACGTCGGAAGAATCGGTGACGACGACGGTGTGTTTGGAG[G/A]CTGGGATCGGAGATCCATGGACTCACGGCGGTGATCGGAAAGTTCTCAACCTTTTCATAT
4753	CaTSNP7739	ATCAACACAGGAACCAGGCCAAGCAGGTGGAGGCACTGGATCAGTTAACAACTTGGGGCT[G/C]GAGATGTTATCAAGTATGTTTGGTGGACTTGGAGCTGGTAGCCTAGCTAATCCAAACAGA
4754	CaTSNP7740	GTTTCAGATTTCACCTCCGAAAACCGACATTGAACAGAGTATTGTGGTGAGTGATTACGC[C/T]CAAATGGATAGGATTTTGAAGGAAGAGAGGAGTTATATTCTAGGTATGATTAAGAAGATT
4755	CaTSNP7741	GTCCAAACATCAGCTTGAAATCTTTCTGGAGGAAGAACTGTTGGTGAACGTAACACATCA[C/T]GAGCTTGTGCCAGAACATCAAGTGCTCACCGACAATGAGAAGAAAGCCTTGCTCCAGAAA
4756	CaTSNP7742	CTGTAACACACAGAATAAAACCTCCTATTGTGCCTTCATTACAAATGTCAACATTGGAAG[C/T]GGATCCATCCATGAGCATTTCTGATAAAGAGGAAACAGGTCAGAAAGGCCAAGTTTATCA
4757	CaTSNP7743	TCCAGGAATTGGAACATATGAAGCAAGTGCAAGTTTATGGGCCTCTTCAGCTTGGGCTCT[T/C]AGAAAATCATCAAACTCAGCAATCCCAGCCTCAAGGCATGGAATAGTAATGGTGAAAGTA
4758	CaTSNP7744	GTTTGCTGGAGTAGATCCACTACAAGCTGGAAATGTGGTTCAACTTGTGCTGGTTTTGGG[C/T]CTCACAGTTGGATGGATCTCAACATATATCTTCAGAGTTTCAAACAAGGAAATGACATAT
4759	CaTSNP7745	TTCTGCTCTACTTTTAAGAGATATATATGCGGTGTTTATCACATAACATTTATAGCATCC[G/A]AATAACAGGCAAGCACAAGTCTGTCAATAAGCAAGTTCCTTGGACCCTTTACTTTTAATG
4760	CaTSNP7746	TCGGTTACTCGAAAGGCTTTTTCGATTAAGGAAGCGTGTCTGGATTCTAAAGCTCGAAAC[T/A]TTCGGTCTTTGGAAGTTAAGGCTACAGAAGATAGTAGTAAAGGCACAAAAGCAAGGAGCA
4761	CaTSNP7747	GCAGCACTTTTGAGGGAGTAACTGATCTCACAGATTGGAATAGAAGTGGAATACGGATTG[T/C]TAACAGTGACCTTAGCCAAATACTCGACACACTCGCGGGTCACGCGCTTGAAATCAACGT
4762	CaTSNP7748	CTTGAACCTTCCATTACTCAAAATATGACCATGATCTCCAAGATTGACAaCAAAAGCTCC[C/A]TCAACAGGCTGTACAGTAATCCAAGTTTTGCCATTATCTTTTGTAGCTTGAAGCCCACCC
4763	CaTSNP7749	TTCTTCCAAGAAGCCAAAGCGCTTCGAGATCAAGAAGTGGAATGCTGTTTCTCTCTGGGC[A/T]TGGGATATCGTTGTCGATAATTGTGCTATTTGCAGGAATCATATCATGGATCTGTGTATT
4764	CaTSNP7750	TCAGAAACTCGTTCTCAATATTTCCGTTGGTGAAAGTGGAGATCGTCTCACCAGAGCCGC[C/T]AAGGTTCTTGAACAGCTTAGTGGTCAAACCCCTGTTTTCTCCAAAGCTAGGTACACTGTC
4765	CaTSNP7751	ATTTGTCACACTCTTCACCTGGTGATATAACTTTCTCCCCTGTCAACAACTCTGTTCCCA[A/G]CTCTTCTCTAACAAACTTGTACAATGGATAAGACCTACACTCCTTAATCTTGTTTGGAAT
4766	CaTSNP7752	TCTGCTAGGGTTTGTTGCGATGGCGGAAGAAGCACAGTATACCCTAAAGCGCAAATACGA[C/T]GACCAACCCACCGCCACCGACATAGAACTCGAAGTCGCCAACGCCAAATTGCGAGCTCAA

4767	CaTSNP7753	TTCAATCCTATATAAATTAAAGCGGTCCTCATAAGTGATCAAATTCACCGCTAAACCAAG[G/C]TGGCCAAACCTCCCTGAACGACCAACCCTGTGCAAATAAGTTTCCGAGTTCTTGGGAAAA
4768	CaTSNP7754	ATGGAGGCTCAGGGAAGTGGTCAACTCTTGACTACTCTTTACCAATCCTCAGGTCTCGCC[G/A]TGAACCAACAAGGGTTCATTAAGGTTGCCCTTATTGGTGAAGGCGAGATCGCTTTTGGCT
4769	CaTSNP7755	CTTTTTGCTTTGTTAAAATGATTCCTTCAGTATTCACTTGCAAGAGATCCATGCACGATA[A/G]CTGATATTCTTCAAGAACCTTTCGTAGTTCATTCAAATTACTACGTGAAATCACAATTTT
4770	CaTSNP7756	GTATTGTTATAGAAGACGCACTAGCTGGAGTGCAAGCTGCCAAAGCTGCACAAATGAGAT[G/C]CATAGCAGTAAGGACCACATTATCAGATGAAGCTTTGGAGTCTGCTGGCCCAACTTTTAT
4771	CaTSNP7757	CCGATCTTCTTCATCGGATTCACTGTCGCTGCTGTCGCTATACTTTTTtATTCTTTCTTC[C/G]GATTCGCTATGCCTCCTACTCTTTCTCCGCTTAGGATCCTCTTTTTCTTCCGAATCACTA
4772	CaTSNP7758	GCTGATGCATCGACGGCTCCTGTTGCTCCTCCGCCTCCCACTCCTTCTCCTTAGCCATAT[G/A]CTTCACTCGGTTCGGTACCTTTTtGTAAGTCTATGTTGTTATGTTCTGTTTTATTGGACG
4773	CaTSNP7759	AGATGCGTCAAAGTTACCATCAACAAACCCTCCATTACCATGACCATTAACAGTACCACT[G/A]TTATTTATAGTGCGGCCATGGTAATTGTTGTTGGTGCGGTGTCTGTTTCGGTCACCAGAA
4774	CaTSNP7760	ACTCTGATGAAACTTCATTTACAACTAGCAAAGCCTGCAGCGGCTGGTTTTTCAACTAGC[G/A]TAGTATTGCTACTCAAATTGTTGATTGGTTTCAGATTCTTCAGAGACGAAGCACTTTACC
4775	CaTSNP7761	AAGGCCGGGTCAGCCTGGTCAAGCACCTCGCCCTGCTGTAGCTGGAGCTGCTCCTGTCCG[T/A]GTCTGAAAGAAAATATCAAGATTTTTAGTCGTGTTGGATCAGTTTTATGTGCCAAACCCG
4776	CaTSNP7762	ACTATGCAATTTGTCAACAATGTCATTATTCATGGTCTTCACATAAGAAACATCAAGGCT[C/A]GATCTGGTGGTATGATTCGAGACTCTTATAATCATGTTGGACTAAGAACAAGAAGTGACG
4777	CaTSNP7763	ACTCTCCCACTTCTCTGCTTCTTTtACGTTGATAATTAAATGTTCGGGTGCATACAAGTT[C/T]GAGAAACTGATAGCCTCAAGCATATCACGTGCATACACAATAAAACTGTGGCTTAATGCT
4778	CaTSNP7764	GTCTAATGTCGTTCGTGCTGTTCTGGACATTTGCATATGACATTGCTCATATATTTTGAC[T/C]GCCGGAATGTGCAAATGCTTCTAGAGAAGACAACTTTTTCTTCACCATTGGGGCTATTGA
4779	CaTSNP7765	TTCTCAAAGGCAGAGGGTTCTGGATCTACCGGCAGCAGCTCCTTAAGAGCCCTAGCTTTA[A/G]TAGGAGCTGGTGTTTCAGGTCTTTtGGGTTTTGCAACAACAGCATCAGCTGACGAAGCTG
4780	CaTSNP7766	GCACCGTTTTCCGGCAACACGGCACCGTGCTTCATTCATTCTCAATCGATTAGTTTTGAT[C/A]ATTTTGGATTGACAATCTTAGGAACATAGAACTAAGAAAGATTGGATATGGCAACTAGAC
4781	CaTSNP7767	CCCAATTGATGAACTCATTGAGAAGGCTGATGGATTTGCTGGAGTCTTCCCTGAACACAA[G/A]TACGAAATTGTGAAGAGGTTGCAAGATAGAAAaCACATTTGTGGTATGACTGGAGATGGT
4782	CaTSNP7768	ATCAAATACTCTTGGACTTATCTTTCTTGAGAGTATTTGTGCACGAACATAATCCTGTCG[G/A]TCTAGGCACAATCGAACTTGTTCAAGAATGAATGCAATTTTCTCAGTTTTTGCCATTGCA
4783	CaTSNP7769	TCACCCAAAATTCATTTCTGACACCAAATCAGCAATATCTGATGCATATAATCATACCGA[T/C]TCTGAATATCTGAAATCAGAAAAGAATCATCACAAAGATGCAGGATCTACTGCATCTACT
4784	CaTSNP7770	CCATGGTAAATCAAATGAAGGATCTTTCTTTTGCCATCTTAACAAATCACTAAGTCTTTC[C/T]AGCAAACTCATTGGACATATTGCAGTAATCTTCACAATCACAAAGCTCACCGAAGATGGA
4785	CaTSNP7771	CCTTATACCAAGCAGGCCTGTTTTGAAGGTCAATAGGAACCAATTTGATCTTCTCTTGTA[A/G]CCCCTTGTAGTTCCTAGTGATCCATACACGTTGTGCAAAAGGGCAACTATAACTGGTATA
4786	CaTSNP7772	ATCCACGTAATTATAAGGAAATTCAAGGTGCTTTTCCCATTCGTTCCATTTAGGCTCGCT[T/C]TGCACCTCGCTTGCAAACTCCGGCGACACCACGTGTTCTGAACAGCTCGAGTCCGTGTGC
4787	CaTSNP7773	TTGCATTTGCAATTGTGGTTGTTAGTAACTATTATTACTCTAGATTTTCCCATTTCGTCC[A/G]TAAACTGTTTAGGAATGTGACTTACTGTGGTGATTCCCCCTTCCTATTGGCTATTCCATT
4788	CaTSNP7774	GTTTTCGATTGTTGATTTTGTAGAAGCATCTTGTTGTTCCTTCAATCGAATATCACCGTA[A/G]CATTTGGAGCAGAGATTCATGGTGGCGGGACTACCGAGGAATCCACAGTTGTTAGCACAG
4789	CaTSNP7775	AAAGGAAGCACTGAGTAAGGTGCTTGTACCATTTTATCCAATGGCTGGTCGTCTTCGTCG[C/T]GACGAAGATGGCCGTGTTGAAATTGACTGTAATGGTCAAGGAGTGCTCTTTATCGAGGCC
4790	CaTSNP7776	AAATAGTTTTACACATAAGTACTCCAACCATTTTACTCATAAATCATAAACACTAACAAT[G/T]AAACAAGCTTTTTGGAAAACACAAGACCCCTTATTCTAGCTAAAAAAaCAACAATATCAT
4791	CaTSNP7778	ATTCACAACTTCACCCACCATAGAAGCTTTCTCCAATGACTTAACCAAAACACCCAACTC[C/T]TCCTTTCTAATAGGACCAAACATCTCAACTTTGGAAGGACTGAGAAGTTTCAAAGTGCAC
4792	CaTSNP7779	GCAGATTACAATATTCAGAAAGAATCTACCCTGCACCTTGTTCTTCGCCTCCGTGGTGGC[T/C]TCTAAGTGTGTCCTTTTAGCATATTTCTCTGTCTTGAATTTGTGTTACTCGTATTACTCT
4793	CaTSNP7780	TGGACTCAATCTGGTATCAACTGTTCAATTTATATCACATACGAGTAAATGAAGGTGATT[C/T]AATCCACAATCTAGTAATACAGAACAATTTGATTGCAAGCCTTTATTTATCACAAGGGGC
4794	CaTSNP7781	AGGCTTTATTTAAGCACAATAGACATGTTTCTTACATCAACTTTGTATGGTTGTTAAATG[C/T]TGTGACAATTTCAATTTTGTTTGGATTGTGTTAAAAGTTTTtGATTAGTTGAATGGATAT
4795	CaTSNP7782	AGAGAAAgAAAAAaaGAATCACTTTTtGTTATTCAATTAACATCCCACTCACAATCCGAA[G/T]TTTCCGGTTCAGGAATCTTGTTCAAGTCAACCCTCTCCACAAAATCACCAGAGCCACCAC
4796	CaTSNP7783	TACGATTTACTGGAAAAGGGTGAGATTAGTGTTATCGCAGAGGAAGAGTAAAAATGACAT[C/T]GATATGTGCCTATGCTCTCCATATATAGGCTCTTTTGTTTATCGCTATATTCAATTGGCT

4797	CaTSNP7784	AACTTCAAAAGCCTCTGCAACATCTTCACAATATAAATAACTCCTCACATTCGAACCATC[C/T]CCGTGAATCGGAAGAACTTTTCCTTGCATTGCCAAGAGGATGAACTTTGGAATTAACTTT
4798	CaTSNP7785	AATCATACCATAAAGCATCTCTGCTGCCGATTCGATTAACTCGTTTTGTTCCTCATGGGA[T/G]GACTCAACATCCAAAATCAAATCAAGAGCATAATCATAGTACGGCACTTGACTGCTTAAT
4799	CaTSNP7786	TAGAGTATAATCTCCATCTTTAATAACTGGAACAGCTCCAAAGGGCTGTAATTTGAGGTA[C/G]TCAGGATCCTTATGTTCTCCCTTAAAGACATCAACAGGGACAACCTCAAATTCAATTTCC
4800	CaTSNP7787	GAAATGGGAGGCTCAAGTTGAACAAGACCTCGATAAGATGCTCGAAAAAGCTAAGGCTGC[A/G]AACGAAAGACGTTACATAGATGGAGATGACGATTAGTGCATGTCTTCAAGGATTGCTACA
4801	CaTSNP7788	CTCCAACTTCTTCAATAGCATACGAATGAATAAAAGGAAGAAGAGGAGAGAAATACAGGT[T/C]TGGACAAATGGTTGTCAAATTTTTTAGACGACATAGCAAATCACAATATCGAACAGGCCT
4802	CaTSNP7789	TTATCGCTTCCTATGCCGACTATCGCCGCCGTCAACGGCCACGCATCCGCCGCCGGATTC[A/T]TGCTTGCGATTTTCCATGATTATGTTTTGATGAGGAGTGATCGAGGTGTGTTGTATATGC
4803	CaTSNP7790	TCAATTGGCTTATGAGAGAAAGAAGCAGCTCAGTAAATTGAGGGCAAAGGCAGAGAAGAT[T/C]GTTGACGAGAAGCTTGCTCCCCAACTTGAAGTCCTTGCTCCTGTTAAGTACTGAGTAGTA
4804	CaTSNP7791	GCACTAATGAAGAATCCAAGAGTGATGCAGAAAGTACAAGACGAAATTCGTAAAGCGTAC[G/A]AAGGGAAGGGTTTTATAGAAGAAGAAGATGCTAAAAAGCTTCCATATCTAAATGCAGTTA
4805	CaTSNP7792	CATAGTATCTTCAATTATCATCTTCCACACACCAGTTCAGCCCGTCAATGCTCTTGGAGC[T/C]GCAATTGCTGTCTTCGGGACCTTCTTATACTCACAGTCAAAACAATAGAGTTGTGGAACA
4806	CaTSNP7793	CTCCATTGCCAGTAAGACCTCGGCTACATAAAATTTTACTGCCTGCTCAGGAAAGTGCTT[T/C]CCTGGTTGTTTCTGCCTAAGCGTATGAAGGTCTCCACCAGGACAGAACTCCATCACCAAG
4807	CaTSNP7794	GAGTAAAGCTCGTGCAGTTCTCACAATGGGTCGAAAGAAGAATCCTCAAAACCCTGAACT[G/A]TGGCTAGCAGCTGTTAGAGCAGAGTTGAAGCATGGATATAAGAAAGAAGCTGATATTTTA
4808	CaTSNP7795	AGTATGTTCATACATCATCAGCAAAGTCATCATCTAACATTCTTACCTTTAATTCCACTG[C/A]TAAATGGAAGGTTCACCTTAAAGCTTCTGAACAAACTAACAAGCTGATGGTGGTTGAATT
4809	CaTSNP7796	ATCATCAATGGACATATGATTTACTACTGAGTTGCCTCACTAATCATCATCTCCATGGCG[T/G]CGTCTCCTCTTTTCTGATTTTTTGACATCCCTACCAATCCTTGAATCTTTAAATCCATCA
4810	CaTSNP7797	GGTGGGAGGGAAAGTTGTGGAAGTTGTCTTCGTAAGGGTTTTGGAAGAGGAGGAGATCGC[A/G]GTTGAACATGAGGAAGTAGCGGCCGCCATTTTTTtGtGAGTGAAGAGTTGAGAAAGTGAAA
4811	CaTSNP7798	TTCCCATGATTGTGTCTGCCCTCTTGTGAATTTTCTCCAACTTTGGTCTCATCCCAGTTA[T/G]TACATGTAAAAATTTCATGGAAGGAAACAAaTCATCCACATCAAaTCcACcTGACAaTtC
4812	CaTSNP7799	ATCGGTGAAGAGGGGAGTGTAAAGTGTTCCCTTTGTGATGAAATTATCAAGCTTTGGTTT[C/G]CACATGCAATGTTGAAGAATAGGCATGCACATACTTTGACATGGGGCTTTGTATTTTGCT
4813	CaTSNP7800	CTCTCTATCTCTACGAAACGCAATCTCAGGGCTCAACTAAGCTCTCTCTCACGGATCTCG[A/T]TGTGCTTGCAATCAAAATTAGGAATTAGGGTTAGGGATTGAAATTAAAATGTGTGTGTTC
4814	CaTSNP7801	CTCCCACTAAGATCATAACATGTATCCAAAAGAGAAAGCGCCGAAGCAGGTGGATACTTC[G/A]ACATGCCTTTTTTGAAGGCATCTCGAAGTACAGAGTAAGCGGTTGGCGGCAACCGTGTAA
4815	CaTSNP7802	GCTCTTGTACTTGACTGCTTCGCCAGATGTCATCATCATTCTTCTTCTAACAGTGTCGAT[C/T]GGGTAAGATGCTAGACCTGCACCATTGGTGATGAGCCATCCAAGTCCGAAGCTAGCAAAG
4816	CaTSNP7803	ATCCATAACTTGTCATCCACACTAGTACCTCCTACTAGGGTAGCGATCCACAAACCGACC[A/G]GACGGACACTCATATGCCAAGTGACCACGACCTCCACAATTGTGACAAATCATCAATGGC
4817	CaTSNP7804	CATCACATCCCAAATACCATCACATCCTATGATGAGGAATTCGTCATCCTCTGTTAGAAC[T/C]ACTTGGTGGAACTCAGGCTCTGCTATCAAAGGTGAGGGAGTACCTCTAGGTAACTTCATG
4818	CaTSNP7805	GTTGATGTGCGATTTGTTAGGATCATTTTGTGTGTATATCTGCGCTTAGATCTTTCTTTT[A/G]TAATAGAATGATTTAATGCGTTTGAAACAATGTAATTTTGATTCACTGTCTCCATTTAGT
4819	CaTSNP7806	ATCTTTGCCACTTGTCTTGTTTTGATATTGTAACAAAAGTGTACATGTGTCGCTAATCCT[G/C]TTGGACCCGAAATAGATTTTGGAACAAACATTACCATAAAGATAATTTGGTCGTTGATCC
4820	CaTSNP7807	AAGCCGATAGACAATACAGCTTCTGGAGATATACCATCAACTTCTTTGTCATTGGCATTG[G/A]CTGGCTGGTCAGGGTAGTTATCAAACGCTCCGCCTTTACGTTTCACTGGTAAACCTGCTC
4821	CaTSNP7808	CATGAGGATTGGACCAGCCACTAATAAAAATCCTGCCGCCACCACTCAGCAGCCGAAAGC[C/T]TCATATCAGAACTCTCAAGGTGCACAAAGCGAGAATGATCCAAAATAATACAACTATTTT
4822	CaTSNP7809	ACAGCACGCCCGTACAGGATCAGAAGAAGCTTCCAGAACATAAAGGAGATATAATTATTA[A/G]CAACAATGAAATTGAAATCGTAGTTCCATCAAATATTATTGATGAGAAGAAACAAGAAGA
4823	CaTSNP7810	CCAACAACAACACAAAAATCAACCAAAGAAACTAGGATCATATCCATTGCTAGAAGTAGC[T/C]AAGATATAATAAGCAACAATATGACCTATAGAACCCCAAGCCAACACATCAACAAGGTTA
4824	CaTSNP7811	TCTGTTTAGCAATAGACTCCTGAAGGATATGAGAATTAAGCTTGAGTTGAGTAAGTTGAT[T/C]CGTTTCCGCCTCAACGATCCAAAGATAAGAAGCGGATAAAGCTAATAATAAGGTTGCCAG
4825	CaTSNP7812	GAAAAGTTTATTAATTCCATTCAGAGAAATCAAGGTTGAATGTACTATACCTAAAGACGA[C/T]GGCACGTTGCAATCTTATGTTGGATTCAGAGTTCAACATGATAATGCTAGAGGTCCTATG
4826	CaTSNP7813	TGTAGCAATCCTTGAAGACATGCACTAATCGTCATCTCCATCTATGTAACGTCTTTCGTT[C/T]GCAGCCTTAGCTTTTTCGAGCATCTTATCGAGGTCTTGTTCAACTTGAGCCTCCCATTTC

4827	CaTSNP7814	GTCCATCTCAAGAAGAATGTCTTCTATGTTACATTTGTCCTCGTACAGACTAAAGAGACC[G/A]TGGCTATGAATGAGGTCGTATGTCCTCGGATATGTGGAGAAACCTTCACACCAATCATGA
4828	CaTSNP7815	TTCCGATGGCAAGATCCGTTACGAAGATTTCATCGCTAGAATGGTCGCTAAGTGAAGCTA[G/C]ATCATGCAAATCGAATCGGTTTAAATGCTTTTTCTTCTTCTTTCTCTAGTGTTTCGTTAT
4829	CaTSNP7816	AAAGAAACTTAGGCTTACCAAACAACAATCTTTACTTCTCGAAGAAAGTTTCAAACTACA[C/T]AGCACACTCAATCCTAAACAAAAACAAGCTTTAGCGAGGCAATTGAATCTACGGCCTCGA
4830	CaTSNP7817	GGTTATGGGTTTAACGGCGTTGGATGCGGATGCAACGAGAATTGAATACTATGCTACTGT[A/G]GCAGAGCCTATGTGTGAACTCAATTACGCCAAGTCTGGGCTTGGTTATTGTGATGTTGTT
4831	CaTSNP7818	GGATTTCATATTCTATTGGGGACATAGGATATTGCACACAAAATGGTTATACAAGCATGT[C/A]CACAGTGTACATCATGAGTATGCTACACCATTTGGACTTACATCGGAATATGCCCACCCT
4832	CaTSNP7819	TTCCCGCGTAACCAATGAAGGACTGCGGTTTTTGAAGCCTTTAAAGAATCTATGCACTCT[A/T]TCCTTGGAGTCTTGCAAGGTAACTGCAGCTGAAATTAAGAAGCTCCATTCAGCTGATCTT
4833	CaTSNP7820	ATCTGATAATATCACCGGTGTTAAAAGATACCGAGACGATGATGTTAAACCATTTTCTGC[C/A]GCTGAAATTAAGAATGAAACAGGAGGCCAGCAGCCTAATGCTCCTTTGGCTCATCAGATG
4834	CaTSNP7821	ATCAACCAAACACTTAGTTATGCTAGGGAGTTGGAGAGGATTGTCTGAAAAGAAATGCAC[A/G]GGATGGTTTATGTGTTTTCCCAAATTGCTGATATTGTCGTTTGAAATTGTTTACTTCTGA
4835	CaTSNP7822	AAGGCCGGGTCAGCCTGGTCAAGCACCTCGCCCTGCTGTAGCTGGAGCTGCTCCTGTCCG[T/A]GTCTGAAAGAAAATATCAAGATTTTTAGTCGTGTTGGATCAGTTTTATGTGCCAAACCCG
4836	CaTSNP7823	TACATGTGTGGATGGATTTGATAACACTACTACCGAAGCTGGTGCAAAGATGAAAGAGTT[A/G]TTGACATCAAGCATGCACATGGGTAGCAATGCTCTTGCCATTATTTCAGATTTTGCTGAC
4837	CaTSNP7824	GGAGGGTGAAGATGAAACTAAAGGTGTATACATTGCCAAACTTGAAGAGCTTGAAAAAGC[A/T]AGGTGACCCCATTGAAGAGCGTTACAAAGAATTCACGGAGAGGGGTACAATAATTGAACA
4838	CaTSNP7825	GCTAAAACCCTAGAATTTCACATCACAGTTCACACCACCACGAAATCGCTTCTTCATCAC[A/C]AACTGTGGAACAGAAAATTGAAGCTAGCTAGTAGCAATGGACTTCGACGAGTACGAGTAC
4839	CaTSNP7826	CTAACCGTCAACCTGGAAGAGCCTTTTTGAATGCCAGTTCTAATGCTTAGGCTTGTCTGT[T/C]ATGTTGAAATATTAGAGTTTGTATCACATTGGTTTTCCAAGAGTGAATTTGAATTTGTTA
4840	CaTSNP7827	TTTGCGGCATATGGTTTTGTCTTGATTGTATTTGCGAGCCAAAGCCATCAAAGAAGGTTC[G/A]ATGATACCACCACGAAGCCTGAGAACAAGATGAAGTGTTGACTCCTTCTGGATGTTGTAA
4841	CaTSNP7828	TGTGGAGCCATAAGTCAAAGATGGAGCTTCCAACAATTCTATCTACCAAAGATGCCTTAG[C/G]TTTCGGTCATTTAGCTAATGTACCATGTCAGCTTTAACTTTCTGTAACTTCTGTATAGGC
4842	CaTSNP7829	ACTCTCTCCTTTGCCCAAACAAACTAATTCCCCTCTAACTTCATGCTTTTCCTGCAAACC[C/G]ATTTTCGACCCTCGTGTTCTGTTTCATCACCGTCCAAATGTTTCTGTCTATGTTGCCTTT
4843	CaTSNP7830	AAGCTATCCATTAAATAATGCGATAGAGTTGAGAATGACCAATCTTGATAATCGATTCCC[G/A]AGTGCAAAGGTGTTGTCTTAGCTTCAAAACAGTGATTGAAATGGAATTTCTCCTTGATAT
4844	CaTSNP7831	AAAGGAAGCACTGAGTAAGGTGCTTGTACCATTTTATCCAATGGCTGGTCGTCTTCGTCG[T/C]GACGAAGATGGCCGTGTTGAAATTGACTGTAATGGTCAAGGAGTGCTCTTTATCGAGGCC
4845	CaTSNP7832	TAGTTCATCTGCTGCAGTTGGTAGCACAGGGAGTTCCATTTTTGGGTTCAGCTCTTCAGC[G/A]ATGACAACTGTAACTAGCCAGTCTCAAAGTTCTTTTGGTTCTGGCAGTGGTTCTTTGTTT
4846	CaTSNP7833	CTTGACTATTGGCAATGGCAATTGTGTTTACAAAAGTATATGTTTGATTGGTTTTCTGAC[G/A]ATATTCTTGATTTCAACTTCGAAATTTTGTTGATTCTGGATATCGATCAATCTCCCTTAA
4847	CaTSNP7834	GTGGCTTTGATGCAAGGTGAGCTCAGACATCATAGGTTCATCCCACCAATATTTTTTATC[C/T]TCACTACTACTACAATTTTTAGCATTATTTGTTGATGTCACTGCCACTGGACATTCACAA
4848	CaTSNP7835	TTTTACATGGATATGCTAGCAGAATGCAGAAAGAACTCTGGATCCTCATTGAGTATGTAA[C/T]TGCCCTTTGTCATGTTTTGGATGGTTACAACTGCAAATGTGAGATCGGAAGAGCGTCGTG
4849	CaTSNP7837	GCAGATTACAATATTCAGAAAGAATCTACCCTGCACCTTGTTCTTCGCCTCCGTGGTGGC[T/C]TCTAAGTGTGTCCTTTTAGCATATTTCTCTGTCTTGAATTTGTGTTACTCGTATTACTCT
4850	CaTSNP7838	GTCTTATCATCAAAGGGGTTGCCATCAAACCAAAGGAATAAGAAATATATATTTATGTAA[C/T]GTAATGTGGGAAGAAAGCTTACTTTCATTATTATAATAAATAGAACAAATAAATCACCAC
4851	CaTSNP7839	TCTCCGTTCCTTTTTCCGTTACTGTCACCGTTAAATAACGGTTCTCAGCGCTGAAGGCTT[C/A]TATCAATTTTTCAATCGGCAGCGCCGCTTAATTAGGCTGAGATGTGAAGCCGCCGCTGGT
4852	CaTSNP7840	ACACCGAATGATCCATTGGCAGATTAATCTCTCTAGGTGCAGCTGATTTTTGTGGAGTGA[C/T]AAAACCATCAAGTCCCAATAATTTCTTAAGTCTCTCATAACACTCCTCAAGATTGTCATT
4853	CaTSNP7841	ACCCCATGCAACAACTTTGCGATCTTCCATGCTCCAAACTTGAACTAAATCATCTTCTCC[A/T]CCCGTCAAAATATATTTTCCATCCATGCTCCAAGCACAACATAATAAAGCACCATAATAA
4854	CaTSNP7842	TTGCACTTCTTAGTATTGATGCTGTTTCTGATAAACTACTTTCCAACTCATCCTTGAGCT[G/T]CTCTACTTCACTGGAGGATCCAATTTGTCTACCAGTAATGACTTCAAGTAGAATAACACC
4855	CaTSNP7843	AGCTAGATTGGTCAAGAAACTTGCCAAGATTAAGGAAGAACAAGGGCTTATAGCCGAGGC[C/T]GCTGATTTGATGCAAGAAATTGCGGTGGAAACTTTTGGCGCAATGGCAAAAACTGAGAAA
4856	CaTSNP7844	ATCATCATTCTCAGACTCTAGATCACCCTCTTCTCCAGATTCTGCATTATCATCATCAGA[T/C]TCATAGTCTGATGATTGATCATCATCAATATCATAAGGAGCAAGGATGCTGCTGTCAAGC

4857	CaTSNP7845	ATTAAGCAGTCAAGTGCCGTACTATGATTATGCTCTTGATTTGATTTTGGATGTTGAGTC[A/C]TCCCATGGTGACATGTTCACAGAGGAACAAAACGAGTTAATCGAATCGGCAGCAGAGATG
4858	CaTSNP7846	ACACCCATGGCACAAGATCTTCCACAAAAAGATGCCTCCAGAAGCTATTGATCTTGCTTC[T/C]CGGCTTTTGCAGTACTCCCCAAGTCTGCGTTGCACTGCGCTTGAAGCATGTGCACATCCC
4859	CaTSNP7847	TGAACTCAGTGGTGTGATTCAAGGAACTACATATACACTGAGAGTTGCACTTGCATCTGC[A/T]ACATTAGCTGAATTGCAGATTCGAGTCAATGACCCCAATGCAAGGCGGCCACTATTTACC
4860	CaTSNP7848	AAGACTCATAATTTAATTCAAAAACAGTACCATCAAAATCAAAACAACCACAAAATAAAG[G/T]TCGACCATCGCCGATCAAGAACCCAGTTTTGCAAGTTTGGCTGCATAAAAACGGCAAAAT
4861	CaTSNP7849	GAAGTTCCAAATTCCAGTTAGATTTCTCTTCGCCATTTCTATTGAGATAGGAAGAAGTTC[G/A]TCTAAGATAGTCATCGAGTTTGGAATATCGACAACCTTCTCGTATCGAGTGATCTTTGTG
4862	CaTSNP7850	TCCAATAACACCTAGATTTGTACACGTGAGCTCAGCGGGAGTTACCAGACCGGAAAGGCC[C/T]GGACTTGATCTCAGCAAACAGCCTCCTGCAGTTCGATTGAACAAGGAACTGGATTATATC
4863	CaTSNP7851	AATAAGAAGAATAGAGAGAGAGAGAGAGAAAATAAAGATTTACGTAATCGGAAGAATGGG[C/T]TGTTCTTCTGTGTTTATGAAAAGAGTGTTCCACAGAGTGTTCAATTCCTCAGCGAACACC
4864	CaTSNP7852	TTCTGGAATAAATGATTGCAGTCAGGTCTGGGCAGGTCCTTTATCAGGAAGCCGTTTGGC[C/G]GTTGCTCTGTGGAATCGATGCTCAAAAGTTGCGACTATAACAGCTTCATGGGAAGCACTT
4865	CaTSNP7853	CCAGTTCTCCTTAACAATTTTCGACTTTTCGAGGATGAATTTTCCTTCCTTGTACTTCTG[A/G]GATGCCTCGTAAGCTCTTTCGTATTTCCAACCCAGCACTTCCCGGCAATCACCGCAGTAA
4866	CaTSNP7854	TGGTGTAGACACAAACTTCACTGTCACAGCAAAAGCAGCAGATGATGCTGAATTTGGTGA[C/T]TTGTATCTCTTCAAATGGACTACACTTCTCATTCCACCAACAACTCTTATAATCTTAAAC
4867	CaTSNP7855	TGGCTTGGGTTAGTGGTAAAGACATTGAAACAGAAGCAGAAGCCATTAATAATTATTATT[T/G]GTTTGATTTGGTTTGCTTTTGTTTCAATGTCTTTACCACTAACCCAAGCCACCCACAAAA
4868	CaTSNP7856	AATGCTAATATCAAAATCCACTTTAGGCTGAATGATGAAGTTTCCTTCTCTTATAGGCTC[G/A]CGTATCAGTAATGCAAATCTGCCTTCACATATTCTGACCCGAATACAATCACTATCTGTC
4869	CaTSNP7857	CTTCTCGAGGAGGACTTCCACGATCTTATCAATTGCTTCACGGTTGTTCTTTATATGTGT[C/T]AATGCAATTTCATATGCTTCATCTGATAACCTCTTGACAGCGGTATCAATGTCTTCTGCA
4870	CaTSNP7858	GAGGAACTTGACAAAGAGCTGGAGACCTATCATGCTGAGGCTATGAATATCTCATAATAT[T/C]CGAATCAGCAAGTTTTTAGTCTAAATTAACTATTGGCTTCCTTGTACAGAAGCTGTTTTT
4871	CaTSNP7859	TGTTATTTTGACTTTCACTTGTGTTTGATGCAGCTGAATTTGTGAAAAGATTAGGTTGCC[T/C]ATTAATCCCTCCTATTGGCTGAAATATGGAGTCTTGTATCAACATATCTTGTGATGGAGT
4872	CaTSNP7860	ACAAGCTCTTGAACTTCAAAGTAGGAGGTTAATGAGTTTGCAACTTCTTGACATCAAGAA[A/G]CAGCAACACCAACGCGCACTTTCCGCCGGAAGTCCAATTCCTTCTCCGACTCACTCTCCT
4873	CaTSNP7861	TGGCTTTATTGAGTTTACTAGTCGTGCTGGTGCTGAAAGAGTGCTTCAAACATTTAATGG[C/T]ACTATTATGCCAAATGGTGGGCAGAACTTCAGATTGAACTGGGCAACATTTAGTTCTGGT
4874	CaTSNP7862	TCTTGTAAACGGAACCGCTGTTGGTTCCGGTTTAGCTTCGATCGTTCTGTTCGAAGCTAA[C/T]GTGCTAGCCCTTTTGTCTGAAGTTATGTCAGCAATATTTGCTGAAGTTATGCAAGGAAAA
4875	CaTSNP7863	ACAATCTCAAACAACCTACAGGGATAGGGCAGCTGAGAGAAGAAGTTTGTATGGCTCGTC[G/A]TCTTCGGTTGGCAATGATTTGGCTGACCTTGAAATTGGGGATTCAACTCGAGATTTTTCA
4876	CaTSNP7864	AAAATGGAAAGCTCTAAACAGGAAGCGAGCAAACGATGTTTATTTGTTTACAGAGTGTCC[A/C]AATTGTTATGGTCGAGGAAAGCTTGTCTGTCCTGTTTGTTTAGGAACTGGTTTGCCAAAC
4877	CaTSNP7865	AATCATTGCTGCTTGGTGTTATAATCTTCCAAGAATTAGTAATGAAGTAGACATTGTTCT[C/T]GATCAACTCTATTCTGTTATTTCAAAACCTTATTGGGATTCAGCACGTAAATTGGTTGAT
4878	CaTSNP7866	AACCACCACCACCACCACCGCCACTGCATCAGATCCAGATCCTCTTCAAGACCTCTGTGT[C/T]GCAGACCTTTCCTCCGCGGTTAAGGTGAATGGATTCACATGCAAATTGGCATCCAATATA
4879	CaTSNP7867	TTGGTCAGCTGCTTGGTACTGGGAAGCCTCCAATGTGATCCAAGTATATTATATCAGATG[T/G]TCTATTTTGCGGTTTGATCTGTCTGGCTATGACTACAATATTAGTAGTTGCATATGACTA
4880	CaTSNP7868	GCGCTTACGTCTTCTGCCCAAATCCACTGACTCTACAGTGGCAGCACCTGAGTCTGAGTC[T/C]GAACTGTCTTCGCTGCAGACCGACTCTCAACCCACTCAAATTGTCCAATCTCCTACGTGG
4881	CaTSNP7869	CAAGAAATTCCTTGTTCACAATGTTAGTGATTTGGAACTTCTCATGATGCACAACAGGAC[C/A]TACTGTGCTGAGATTGCTCACAATATATCCACAAGTAAGAGAAAGGAAATAGTTGAGAGA
4882	CaTSNP7870	TGCAACTCCGGTACAGCGAGCATGTCGTTTGCCGGGAGGAACAGGCCACTGATCGCGCTG[C/T]AATTCATCTAACAGCGAATTAAGAGTGTATTCACCCTCAGATGCAGGCAACAAAAAGCGC
4883	CaTSNP7871	ACGTTGATTTCAAGCGCGTGACCCGCGAGTGTGTCGAGTATTTGGCTAAGGTCACTGTTA[A/G]CAATCCGTATTCCACTTCTATTCCAATCTGTGAGATCAGTTACTCCCTCAAAAGTGCTGC
4884	CaTSNP7872	CTCCAACTTCTTCAATAGCATACGAATGAATAAAAGGAAGAAGAGGAGAGAAATACAGGT[T/C]TGGACAAATGGTTGTCAAATTTTTTAGACGACATAGCAAATCACAATATCGAACAGGCCT
4885	CaTSNP7873	CACATATTGGAATACCCTTCTAAACTTGATCCAATTTCCATCAAATAATTTATTTCTCTT[G/A]TAACATCATCATAATCATTAATCATGGCTCTTCTGGGTTATTTTGTGCTTTTCCTAGCCC
4886	CaTSNP7874	TTTAAAATTCCTGAAAAGGCAATTTGCTTAGCCATTTGCTTTGATGAACCTAAACGATTC[A/G]TCTTGCGACCAAGGCAAGTTGTGATATTGTTGCACCATCCTCCTCCCTCAAATTGAACTA

4887	CaTSNP7875	TGGAATAAACTACAGGATGCAATTCCTCCAGAAATTGGGGAGCTTAAAAGTTTGACGCAT[C/T]TGTACCTAAGCTTCAATAGTTTCAAGGGTGAAATCCCCAAGGAGCTAGCAAATCTTCCTG
4888	CaTSNP7876	TGTATGACGTGTAGAAAGCGCGTGGGGCTCACGGGGTTTAAGTGTAAGTGCGGGTTGATG[C/T]TGTGTGGGACCCACCGTTACCCGGAGCAGCACGCGTGCGAGTTTGATTTTAAGGGGTTGG
4889	CaTSNP7877	GTCTGATACTAGTTGGTTGTATACGCTGTTGGCAATGATCGGAGAACAATTTGATCACGG[A/T]GATGAAATCTGTGGAGCAGTTGTGAATGTTAGGAATAGGCAAGAGAAAATTTCTATTTGG
4890	CaTSNP7878	TGCCATGGAAGGTGGAAAACCCACCATCATTACCAACGCCGAGGGTCAGAGAACCACTCC[C/A]TCCGTTGTTGCATACACCAAGAACGGCGACAGGCTGGTTGGTCAAATTGCCAAGCGCCAG
4891	CaTSNP7879	CAAACAACGGTTCCTTCCAGGCAGGATTAATTAATTTGTGAAGTAAATAGTACTAGATTA[A/G]AGAGAAAGAAGATGGGGGCAATGATGTCGAGGTTTTGGTTCATGTTATTCCCTGCAAAAG
4892	CaTSNP7880	TTTCTGAACTTCCCTAAGCTGAACTGAGTTTTCAAAGCAGTGAAAGAAACCAAAATGAGG[C/G]AATCTCTCATACCAAGTATCTTGCATTTCGTGAGTGTCCCAGTGTTCCCCGCTACTGCTC
4893	CaTSNP7881	GGGCACTGAAAGTGATCCAGAGTTCAAAGGGTACCACAATGGCAATTCTGATCCTCGTGG[C/G]TTTGGACACATTGGCATAACTGTTGATGACACCTACAAAGCATGTGAAAGATTTCAGAAT
4894	CaTSNP7882	CTTCCCCGTAAATGCAACAACCGGTTCATCAACATCATTCTCATCCTTCTCATCATCTTC[G/A]TTATCATCATCAATTACATCAAAAGCCGATGCACTGAACACACTACCTCCTCCACCTCCT
4895	CaTSNP7883	ACTGTCATCGGACCAGCAAACACTCTCTCGCCGGAATGCTGCGCTGGCAATTCGTTTTCC[A/G]GTTTGGAGAAGAACCATTGCATCTTCCATTAGTCCTAAATGGCAGCATGCTTCACCCAGA
4896	CaTSNP7884	TCAAGGGAAAGAATTTACTGTTTTCCAACTAGTCATCTATTGTGTCTTTGGCGTGCTGGC[T/C]GGTATAGTTGGTGGATTGCTCGGACTCGGAGGAGGTTTCGTTATGGGTCCACTTTTTCTG
4897	CaTSNP7885	CCTTCCTCAAACTACTACCCATGTTCCCTCCACCTCTGATGTAGGTAATTCCATAAGTGC[T/C]CTCGTTGAAACACAAAAATCCAACGTTAAACAAGCTGTTTCCAACCTCACTGGAACACTC
4898	CaTSNP7886	TTTGTATGGTTCTTCCTCATGAAGAGAACCAGCAGTAATGTTCTGATCAGACAGAACACC[G/A]TTTCCCAATAATCTCAACTTTTGGCTAAATAAGTCAGACAGTGAGCTTGAGAATAGACTA
4899	CaTSNP7887	GCCAGAACTTCTCCAAATGGACCAACAAGAGTGGAGTGACCCCAAGCCACATAACCAGAT[G/C]CAGCATCCCGAGCAGGTGAACAGGTTGCCACATATAACTGATTATCTGTAGCCCTGGCCC
4900	CaTSNP7888	AGAGGAATCTTCAGGAACAACAAATAATGTTGATGGAAAATCTAGGGCTAAAAGTGATAG[A/G]TCCAAGACTCTCATTTCGGAGAGAAGGAGGAGAGGTAGAATGAAGGATAAGCTTTATGCA
4901	CaTSNP7889	TATGATTCTACTGCATATGTGACTTTTAAGGAAGCTTATGCCCTGGACACTGCATTATTG[C/T]TGAATGGATCAATGATTTTAGACCAATACATTTCCATTTCGCGCTGGGGAGCTTATACTG
4902	CaTSNP7890	TTCTTATTCCTCTCCACCACCACCACTAGTACCAGTCTACTCACCACCACCGTCACCGGT[C/T]TACTCACCACCACCATCACCAATCTACTCACCACCACCAGTTTACTCACCACCTCCACTA
4903	CaTSNP7891	TACATAGAGCATTCCCTTGTCACTCTCACCAGCAAATGCTATAGACAACATTTCAAGCTG[A/C]TCAGCATACATTCCGATGTAAAGGAAGACCTGGTCAGTCAACTTCCCTGAGAGTGATAAC
4904	CaTSNP7892	TTACTTATCCTATTCAGAGAACCACTTCCATTGCTGCCGCTAACTTTACCATTTCGCCTA[C/G]TGTACTACCTTCTGTTAAATCCATTTCACAGATAATTGAATCGCTGATTAATGGTGTTGA
4905	CaTSNP7893	TATATCATCAGTTTCAAGGATACCAACAATGTCATCATCCTTCACAATAAGATGCTTTTC[G/A]CCATTGAAATCCACCTCAGTACCTGCATACTTCGAATACACAACTTGTGCACCCGGCTTC
4906	CaTSNP7894	TAAGTTGGCTGTTGGAGCTGATTTTCATTGTTTCAAATATAAAATTGAACCTAAATGGGA[G/A]GACCCCATTTGTTCTAATGGTGGAAAATGGACTATGACTTTTCAGAAGGGAAAGTCTGAT
4907	CaTSNP7895	AATCAAGACTGTTGATGCTATGCGTGCTGCTTATGGAGATGATTACTATATTTGCAGATT[C/T]CAGGAACCTGGCAAGATGGAAGCTCAAATGGCTGAGGTTGGCACTGCATATGTGTTGAAA
4908	CaTSNP7896	TTGCCTAGGAACTAAAAGTTGACTCCCTTTGGATGCAGGTTGAAGTTCAGCAATATCCTG[A/G]CCAGAATTTGAGTTTCTGATAATTGACACTTTTGTCCTTGCAGAATTCATTCTTTTCAAA
4909	CaTSNP7897	TATCTTGAGTGGAAATCTGCCTCCTGGCTTTGATTCGAGTACTTGCCGCAGTGTTTATGT[C/T]GGCAACATTCATCCTCAGGTTACAGAACCCCTTCTTCAAGAGCTTTTTTCAAGTGCTGGC
4910	CaTSNP7898	AGCGTCGAATGTGTTCTCCAAAAATATTTTATTAACCGATATTGGATCTCCCAAGTCGGC[A/G]TATATAAATTGAAGCCTTCCTGGTTCTGGAAATAAGTCTTGAAGAACCCTGATGGCACCC
4911	CaTSNP7899	CAGCAGAGGATGTCCCTAGACTGAGAAAGGAAAGAGAGGATGGACTGTTCAAACCGGATC[C/A]GCGATTCGAAGAGGCAAAGAAGTTTATTAGGAGTGGAGTGTTTGGAAGCTATGACTACAA
4912	CaTSNP7900	CACATGACTGATCCAAGATCTGATGGACTTGGTGTGTCTACATGCATCCAGAGCAGCCTT[A/G]TAGATGCTGGTGTGTCCCCTGAGGAGGTCAACTACATAAATGCACACGCAACTTCCACTC
4913	CaTSNP7901	CGTAGCAAAAACATACAGATCCATGCAGGTCCAGCTGTGTGCCTCCCCTGTCTTGAGATA[G/A]AGAATGGCCCCACCCAAGGCGGAAGCATCTGCGTCTCCAGCAGTGAACTCAAGTTTGGCT
4914	CaTSNP7902	CTTCTGACCAACAGTTCTATAATCAAAAGGGCAATCATGTTTATCAGAGTAGCGGTGCAT[T/C]GAACAAAAGAGGTTGCCACATTTGCAGCTGAAACCAGTTAATCCAACACGTTTCCGGCAA
4915	CaTSNP7903	GGAGTTTTTGGCGGAGAGGTTTGTTGGGAGTGATGGAGAAAATCTGTTGAAACACGTGTT[C/G]TGGTCCAATGGACGTGAGACTGATGAGCCTACAGCTGATAATAAAATGTGTCCCGCTAAG
4916	CaTSNP7904	GCAACAAGGTTACCCAACACAAGAAGGGCACACTTCCAACTATGGATCGCAAGGAGATTC[G/A]TCCCAAACTTCCCAACCCTCTACTGTTGCCCAGCAAGGTTATGCTACCAACCAACAAGGA

4917	CaTSNP7905	TAAAAAGTGTACATGGGTTAATCATATTGACACTGTTTCTTCTCTTGCTGTTTCAAAAGA[C/T]GAGTCTTTAATTTACTCTGCTTCATGGGACAGAACATTCAAAATTTGGAGAGTTTCTGAT
4918	CaTSNP7906	GATATATGAGTACATGGAGAACGGAACTCTCAAGAGTCATCTGTATGGTTCGGAATTCCC[G/A]AGCTTAAGTTGGAAGGAGAGACTCGAGATATGCATTGGATCTGCTAGAGGACTTCATTAC
4919	CaTSNP7907	GAAGGGGAGTGTCTAAGTTTAACTTTTGTATTCCGAGTTTTGTGCCGTTTGCTTCTATCC[C/T]ATTCTTCGTGGATTTGACCTGATGTGTGGCTTTTGATGGACGGCAGTGTGAAGAAGATCA
4920	CaTSNP7908	TGGGAAGCGTTTGGCATCAATTGGATTGGATAATACTGAGGTCAATCGACAGGCTTACAG[A/G]CAACTTTTGCTGACCACACCTGGTCTTGGTGAATACATCTCCGGTGCCATTCTTTTTGAG
4921	CaTSNP7909	GAACCACGACTATGTTCTGTTTCACTATCCAGGAACTAGGATCCAATGTAGCATCTACTC[G/A]GAATCCTGCATATTCTGAGTAGGAGCCTCTGAGCCCTTAGTGAGTGATAGCTTGGTTGGA
4922	CaTSNP7910	CTTCCCAATAGCCTGTAGACCTTCATTACCAATATTAGGACACGAGTCTATCGATAACTC[A/G]ATCAGTTTCAGGCAGTTTTTTGCTACTGCAACTAAAGCCTTGTCAGAAATTGTGGGCAAC
4923	CaTSNP7911	TGTTTTAGCCCTCATAATGATTGGTGTGAAGGACCAGAATGATAGACGTGATTCATGGCA[C/T]CATGGTGGATGGACTGTAAAGACAGTGATATGGCTTTTGCTAATTGTCCTTGCTTTCTTC
4924	CaTSNP7912	CACCAAAGTAAATCCATCAGATAGTCCTGCTAATGTTAACTGAGGAACCAACCACAAACC[T/C]GACATCGATGAAATAGCGCCTTTTCTCGGGTTCGATTCCGATAGGATTATTAATAGCCAT
4925	CaTSNP7913	TTCTGGGTTTGAGATATCACGAAGACTATTTGGTGCCATTCCACATAGCTTGCAACCTGA[C/T]GAAGAGCTGCGCAAGGAAATGGGGTTTGCTAATACCGATTCGAAATAAGGTTTAAAAAGA
4926	CaTSNP7914	GATGAAGCTGGGGAAAATTTAGCAGACACACTTGATAATTTTGTTGAAGCCACTGATACC[C/T]CTGGAAGTGAAGGATCTTTGCTTAATGAAGTGGAAGAGAATTCAAAAGAAGCTGAGAGAG
4927	CaTSNP7915	TCTTTACTGTTGGCCTTGCATTTACAAATGGCTTCATTTAAAAACCAGTGTCTATTCGGA[A/G]TACGAAGAACAGCAGAAGCCGCAATGTCCGGTCTGCAAATCAGAACTCTCTCAATCCTCC
4928	CaTSNP7916	AGCACCATCAACAGGCTTCCGGATTTACCGACCTCATAGGCTCATGCCGGAGACAGCTCT[C/T]GGACATCGAAGTCTTCCTCCCATGGGCTTCCTCCAAGTTGATGATGTTGCTTTAATTGAT
4929	CaTSNP7917	CGACTCGGCGTGGCTTAGGGCAAATGAGAGGCTCCTTGTGATCAGTAATGGTAACTGAGC[C/T]TCTCATCTCTTCACAAGCTGCAAAGGCGTTCTGCTGAAGACTGTAACAGTTCATCTTCAA
4930	CaTSNP7918	ATTGAAAGAACGCAATTACCTTGGATTGTCGGATTGCTCATCGGTGGACAGTTCTACTGT[A/C]CCGAGCTTGTCCGATGAGAAAAAAGAGAATCTAAATTTGAAAGCTACGGAGTTGAGGCTA
4931	CaTSNP7919	ACAAGTACTGTCCTTATTTATTGGTAGTAGTATATATACGCACACATAAAGTATATATGA[T/G]GTTCTAAAAACTAAGGATGATGAGGGTGGGGCTTCTCTAAGTCCTCAAGGCTGGAAGGGC
4932	CaTSNP7920	GCTAAGCAAACCACCAGATTCAACAACTTCAATAGTGCCATTGAAGAAGGTGCTTTGATA[C/T]GGATGCTCGTCTATCTCATACACAATAGTGCCCACAAGATCGAAACTTCCAGGCTGCAGG
4933	CaTSNP7921	CTGCTGCGGCTGTCTCTTCAACCTAATCTTCAAACTAATCCTCACCGTCATAATAATCCT[C/T]GGCATCGCAGTCTTCCTATTCTGGCTCATAGTCCGTCCCAACGTAATCAAAGTCCACGTC
4934	CaTSNP7922	ACTGAAAGATAGAGGAGTTCTTGCCAAGGCAACACATGACACAATTATTCGTTTCACTCC[G/A]CCACTTTGCATAAGTGACGATGAGATCCGGCAAGGTTCTAAGGCACTGGCTGATGTGCTT
4935	CaTSNP7923	TATTGAGAAAATTAAGAAATGTGTTGGTGATCCTGAAGCGGATGTGGAGAATGAAGTTCT[T/A]AAGATTGAACAAACAGCACAGATTGGAGGAGGATCTCGCGGCGATGTCACCATTTTGCCA
4936	CaTSNP7924	GGGGTCGACACAAGTGCAATATCAGTTGAGTGGGCCATGGCTGAGTTGATAAAGAACCCA[A/C]GAGTGTTGCAAAAGGCACAAGAGGAGCTAGACAAGGTCATTGGTTTTGAAAGGGTTATGA
4937	CaTSNP7925	GATGATTCAGGCTTTTCATAAGGAAGGAAACTTGAAGGCTTCTGCTAATGTCAAGCAGCT[T/C]GATCGCGTCGGTGCTAGACTTATTAGTAGCGATCACCTTTGATTTCGTTGTAGCTTGTGA
4938	CaTSNP7926	AGCTAACACTGCTGCTGAAGTGGCCTTGAGGCTTGTAAGGCCAGGAAAGAAGAACAAGGA[T/C]GTAACTGATGCAATTCAGAAGGTTGCTGCTGCTTATGATTGTAAAATTGTCGAGGGTGTG
4939	CaTSNP7927	TGGTCACATGCCCCATGGGAAATTCAAGCATGGCAAGTTCAAGCATGGAAAATTCGGCAA[G/A]CATGGGAAATTTAAGCACGGAAAGTTTGGCAAGCATGGTATGTTCAGGAAGTGGAAGTGA
4940	CaTSNP7928	TTTCTCTTTGTTAACGGTCTCAATTTCAGATTCAAGAGATTCGATCTTCTTTGACATTTC[G/A]AGACTTTCTTCCTTAGCCGCAAGAAGACAACGCTCCGAATCCTCAAGATCTTCTCTTTGC
4941	CaTSNP7929	GGTTAGCATAATCGCGATCTCCTCAGCACGCGCACCAAGACGCGTGAGAACTAATTCTAG[C/G]TCTTCACGCGAAACGACGCCGTCGTTGTCACGGTCAATTAACCGGAAAGCCTGAGAAAGC
4942	CaTSNP7930	ACGCTGCTGGATGGAATATCTTTTGAAGCTTCAACTCCTGCCGGGTTTGGATTAACATTG[A/G]CAGGGACTTCTTGTTGTTCTTCAACTATTCTGTCACCTGCTTGATCTAGAATACCCTCTT
4943	CaTSNP7931	CACCTTTTCAAGAGTTTCTTCAATTGCTTCATCACTCAATTTCTCACTTCCTCCTTTCTT[G/A]AGAATGTTATCACAAAAAGTGGCAAGAAGTTCAGCACTAGAGTTTCCAGCAACACCTTTG
4944	CaTSNP7932	CATCTTCTTCTGACGTATCATATCAACCACAAGGCCACATGCTTCTCTTGCCTCAGCTTG[T/C]CCCACAAATCCAGATGCAAAAGGCAATGCTTTTCCACTGACCTCGAGACCTAGGCCTTTT
4945	CaTSNP7933	TGCAATGTCCACACCATGGTGCATAAAACTCAACCAAGACATCTTTGGTTTCATCCAAGA[C/T]AACTTCGTTGAAGTTTTCAGGTGTTAGCACCACTACACTAGATGGCGCAGTAGCAATTTT
4946	CaTSNP7934	CTCTTGCATTTCCTTCACACCTTTATTAAGACCCTCCTCACGCTGCTTCAAAACCCCTTG[C/T]ACACCAAACAAACCCTCCGTTTCAACCTCGGTAGTCTTCCTCAACGACGCAACATCATTA

4947	CaTSNP7935	AGCTCCGCATGCGCTTCCGGGGATAACAGCAATTCCGTGCCTATTAGCTAGCCAGCGAAC[G/C]ACATCAAAATCATTGTAACCGTGCCCGGTTGGGATCTTCACCCAAAGGTATATAGCTCCT
4948	CaTSNP7936	TTTGGGTTTAGTTGGAGATGCTAAACGAGTTTTGGAGATTCTAAATAAAGAGATTAAGGA[C/T]GATCCTTTTTGTTTGGGGAGCAATCATCCATGGGTGGAAGCTATTTGTAATAAAGCAAAG
4949	CaTSNP7937	CGTGAACATGGAGGTTCAAGCGGAGTTTATGGAAAGCGCTATGGCTGGATCGACCTCTCT[A/G]TCTACACCGGAAGGAGAGGTTAACAGTTTGATGCAACAGGTTGCCGATGATTACGGACTT
4950	CaTSNP7938	TGCTGCCGTCGATCATGGAGGCAATTTTGAAGAGGAAGTTATCGGGGAACCACGAAGATA[C/G]CGATGACGAGTTGATGGATGAACTCGCAATGCAACCTATCGATGATGTGGATGATAGAGA
4951	CaTSNP7939	AGAGCAAAATTTTCTTGGTGGAGATGAGACATGTGATTTCTTCTCAGATGAACAACCACC[G/C]ACTCTTCAGTGGTATTGTTCGGACGATTGGAATTAAGAGAAAAGAGTTTTAAACTTGGGG
4952	CaTSNP7940	TTCATTTAATTCCTTGGAATTCGACCTTTCTCTCCTCTCTCTTAACCATGCCCGGTATGC[C/T]GATTCCATTATATCCGCTGATTCCTGCACCTCTTCTGCTGCAGAGAAAAAAACGTCTTTT
4953	CaTSNP7941	CAAATCTGCTAGCTTTCTTCTCTCAGCAAGATCTTTCATCTTAGATATCAAGGAAGTCTT[G/A]GACCAGTTCTCATGCATAAATAACAATGATTGTGCTGAGCACTTCTGACCGCTGCATGCA
4954	CaTSNP7942	TATGCAACTTAAGCTTGTTCCAGGAAACTCAGCTGGCACTGTCACTGCCTATTATTTATC[C/T]TCAAAAGGAGCAACTTGGGATGAGATAGACTATGAATTTCTAGGAAATGTAAGTGGTGAA
4955	CaTSNP7943	CACCTGGTGGACTGGCTGTTCTAAATCCAAAATTAACGATTGTGAGAAAGCTGTCGTCAA[A/C]TGCAGCTAACACTACATCCAATGGGAACGGACCTTCAGAAACAGCAGATGATGACTTGCC
4956	CaTSNP7944	AAAACTCGTTTAAAAGCTGTGCCGTCATATTCTTTCAATCGTGGACAACTGAATAGAGTG[C/T]CTTCTGGGGATCCCAACCAATTTCCTCATCAGATGCCTGATGGATATCAAACCAATGAGG
4957	CaTSNP7945	CTGCAACTGAGAATATGAGATCCGAATTACTGAGCCAGAAAGTGAGGTGGCATGCTTGCA[C/G]ACAAGGGCTTCAGTAGCCTGCTCCTCAGTATCAAACTGAACAAGACCTTGTTTCTTCCCA
4958	CaTSNP7946	GTATAGGCAAGCTATTACTGCTGCTGGCACTGGGTGTATGGCGGCTTTGGATGCAGAACA[C/T]TTCCTGCAAGGGATTGGTTTACAACAGGATAAGAGTGATTGACTAGATACTAGAGGGTGT
4959	CaTSNP7947	CAGGGTCTAGTTATGGAAGTGGATTTGGTAATGGATACAATGGAGGAGAGATTGATGAGC[C/A]CGAGAAGCAAGTGGTTACTGAATATCCTAGTGGATATGGATTGGAAACTTTGGATATATG
4960	CaTSNP7948	AAGACCGCCGTTACCGGAGAACACAAAGTCAAACGTGTTATTACCAAACGGAATCTGGTG[T/A]CCTTCGCCGGATTTCACCAACGGCTTCAACGCCTTCTTCGAAATTCCAACAGCGTCCACA
4961	CaTSNP7949	CAATGCTGATGAGCTTCTTGAAAGTTTCTTGGAAAGTTTCCCCGAAGAACCTGCACTAGT[T/C]CAACTACAGTTGCTGACAGCTACTGTCAAGCTTTTCCTTAAGAAGCCAACCGAGGGCCCA
4962	CaTSNP7950	GCTTGATCTTTGCAAACTTGGTTCGCTTACTTTTAAAGCTCCTGACAACGTTAAGTATCC[G/A]TCCATGAATCTTGCCTATGCTGCTGGCCGTGCTGGAGGCACCATGACAGGAGTTCTTAGT
4963	CaTSNP7951	TGAAGGATGAGTATGATGAAGATTTGTTTGACTACTATGCGACCTCTCAGCTTGTTTTAG[C/T]ATCTTTGGATGGTACTATAAAGGATTTTGGACCACCAGCTGTTTATACCTCTATGGACCC
4964	CaTSNP7952	CTTTCCAAAAGTGTCGTGGTCATAAACTTCAACAAGTAGCATTTCATGTAATCCATCCTC[A/G]ACTACAAAGTCAAATGTTTGATTCCAAACAGGATTCAAGCTGTTGTTCACAACCCTGGTT
4965	CaTSNP7953	TGTGATGGTAAGAGCTTTCTTGTTTACATATTGGGGTTTAAAGACATCTCAAAGCTTCTT[T/C]ATTGGATTGCTTCGAAGTATCCTCCATGCACCTATGTCATTCTTCGATACAACTCCTTCT
4966	CaTSNP7954	TGAATATGGAATTATTGACGGGGTAATCGATAGCGATATGATTATTCCTCTCATGCCAGT[A/G]CCAGAACAGGTGGAAGCAACACTAAACACCGATGATAAGGATTTCCTAACCCCAAATATC
4967	CaTSNP7955	TTCTCTTTCAAATATTCAATGGCTCTCATCGTTAATCTTCCTATTGGAACTCCACCTCAA[C/G]TTCAACAAATGGTGTTGGACACAGGAAGCCAACTCTCGTGGATTCAGTGTCACAAAAAAG
4968	CaTSNP7956	TTCTAGACTTCGAATGAGTTCAGGAATGTCATCAGAGTGAAAATACTCATGAATTATAGT[C/T]ACAACTTCCTTTTTGTACTTCCTCACTTTCTCATCTTCAACTTGGTAATCCCCATCTTCA
4969	CaTSNP7957	TCCTAACTATGAAAGCAGTGTTGAGCGAGAAGATATCAAAGAACCATTTAGTGAGGTATC[A/G]ATGTTTAAGAAGAATTACGACCATCTTCCAAAGGATACATACTTTGGTATGTACAAAGAT
4970	CaTSNP7958	TAATGGGAAAGTTGTGCCCGCAGCTTCAACAGGTTGAGTTGACTGGACTTAAGGGAGTGA[A/C]CGATGCTGGGTTGCTTCCACTGCTTGAGAGCTCTGAGGCTGGTCTGATTAAAGTTAATCT
4971	CaTSNP7959	CGTTAAAGGTAGCTTCCTTCTTGAGTTTCTCGATATCGGCCTTCTTGTAGATTGTGCGGG[C/T]AACGGCAGGTCGGTTACGGCGTTGGTCACGTGTGTAATACTTAATGTCGTATACGGTTTC
4972	CaTSNP7960	AGGAGGATTACTCATTGAATTTCCTTTAGCTCGTCGCAAATACACTACCTGCATATGATA[T/C]GTGTCGTCAGCATTTTTTACCACATAAATTTCAAATATTGGTGTTCCGGTAGATGCTGTG
4973	CaTSNP7961	AGCAAGATCAGCTTTCTCTTTCGCCAACTCTTGTGCTCTTTCAATTCCGCCACAACTCTT[T/C]ACCAACGCAATGGCTTCATCGAGTGAACCGATCTCACTAAACTCAGATTCGATGATATCC
4974	CaTSNP7962	CGGTTGGAATTTTGCTAAAGAGACCGGTTTCGATGTGGTGATGATTAACCCTGGTACAGC[A/T]TTGGGCCCTTTGATTCCACCAAGAATGAATTCAAGCATGGCTGTACTTGCTGGTGTTCTC
4975	CaTSNP7963	GCTAAACATTTTACTTCTTCTACTTGTTGGATGCTCACAAGATTCCATTGATTTACTATG[C/A]TTCCATCTGGATTTAATTCACCCTCCATTATTAGAGCTGCTTTATCCAAGAACCTGAATT
4976	CaTSNP7964	TTGATGATCATCTTGATGACATAAGCAAGGATTGGCTAAACCCAGGGCTCTTCAACTGAT[C/T]GTGGCCCTTATTGTATCCATTTTAATCGAGTTTACTTAGCAAGTGGATGATGTTCATGCA

4977	CaTSNP7965	CAGATCATCAAATTTTTCAGGATCTGGTTTTTCACCAGTTTCATTGACAGGGGTGGTACT[C/G]TCGCTATTGCAAACATCCAGTTTAGGCTTTTTATCCTCCTTTTCCTTGCCTCCCTCGGGT
4978	CaTSNP7966	CCTTCAGTATGCTGATCAGATAATGGGACTATTTGTCAGGGTCTTTGCTTGTAGAAATGC[A/C]ACTGCCCATGAGGAGGCCATGCTAGCTATCGGAGCCCTTGCCTATGCACTAGGCCCTGAT
4979	CaTSNP7967	ACCAGTGTTCGACAGTGTTAAAACTATTCTGGGGATGCTTGAGGTCTCAACAGAGTTTGC[A/T]ATGAACATTACTTTCAATCGGGAGAGAATACAGAAGGCTTTACCCGCTGGTTATCTCGAT
4980	CaTSNP7968	AACAATTGAGCAACTGCTTCCTATTTTCCTCTCTCTTTTGAAAGATGAGTTTCCTGATGT[T/C]AGGCTAAATATTATCAGCAAGCTTGATCAAGTGAACCAGGTTATTGGTATTGACCTGTTA
4981	CaTSNP7969	AGTTCGAGTTGCCGTGACACAGCTTCTTTGGAGACCCAGGGGTTTGTTGGTGGATCAGTT[C/T]GATTCCAAGGAAGATCTCATCAATGCAGTTTTTACTTCTTCCTTTATTCCAGGATATCTT
4982	CaTSNP7970	GACATTGCGAACCCTTGAGCGTGAAAATGTTGCAACCAACGATGCTTTGGAATCCGAGGT[A/T]GGAGCTGGATCCTCCAAGAAGAATCGGACAACTTCCGCTAATGCCAGTGCTTTGAAGCTT
4983	CaTSNP7971	CCGATGAGAAGGAGATTGAGAGGATGGTGTTGCAGAAGAAGAAGACAGACCTGTTGAATA[G/A]GTATGTTAGTGAGGTTCTCATGGAGGAGCAGACGGAAGCCAAGGACATGCTTAACATTCA
4984	CaTSNP7972	TTATCCCCTCCAAAGAATTTATTCCCCTTTATCTCCTCTTCTATTTTCTCTATTGCTTCT[C/T]TGGCTTGCTTTAGAGCTATTTCCTGGTCTTCCCCACTGGTACACATAGCAACCCATGCTA
4985	CaTSNP7973	ACAAGGTGCTGTAGAGGATGGAGCACAGCCTGAAGAGGAAGAGGAGGTCGATGAAACTGG[T/C]GTGGAAGCCCACGACATTGATCTTGTAATGACACAGGCAGGAGTGTCGAGAAGCAAGGCT
4986	CaTSNP7974	CAGACCTAAGGTCGACGAGATCTCACTCCTCACCGTTAATTCCTCCTCCTTCGTTGCGGA[C/T]TCGTATGGCTATATGGTTGCATTTGCTGGTCGGAAATATGCAGCAAGGTCCCCTCCGGCT
4987	CaTSNP7975	CCATCCAGATGTTAAAAGAAGGCTTCTTTTTGTTTCCCCAGAAGTAGTAGTATATGAGGC[C/T]TCTATCTTCGATGGAGCTTTTCCCCATACCGAGCCTTTTCCATTTGTCCTACGAAACTCT
4988	CaTSNP7976	GTGATTGGAGCATTAGGCTTTTGGCCGTTATCTGTTTACTTTCCTGTAGAGATGTACATC[G/A]TACAGAAGAGGATCCCAAAATGGAGTAGGAAATGGATTGTTCTGGAAATGATGAGTTCTT
4989	CaTSNP7977	CAACCTTCCAATTCTTTCTCATCGACGCCTCTCCCTTTCTCCTCTTTCTAATGATGCGGC[C/G]ATGGGGCTGGTCTTGAGTGCTGCTACGGGTAGAGGATGGACAACAGGTTCAGGGATGGAA
4990	CaTSNP7978	GTTCCAGGTTAACTCTAACAAGCATCCACGCGACTCTGGCTCAGGGCCACTAATTGTCCC[G/A]GTTCCAAGGAGATCGCCTGGCCTCAGGTTGCAACCATTGATTGTGTGGTGAGCAAGTTGT
4991	CaTSNP7979	CATCGAGTTCCTTGGAAAGTTTGCCTCAACAGTGAGGCTTGATTGGAAGTTGTGGCTTGC[T/A]TCTATTTGTATTGGCCTTGTCAGCTGGCCTCTTGCTATAGCTGGAAAATTCATTCCAGTT
4992	CaTSNP7980	CTGTCCAGACCAGTGGAGTTAAAAGATTGATGATGAAGAGGAAATAAATTCCTGTAAGAC[G/T]ACTCAATTATCTTTGTAAAAAAATGGGTTGTTTGGTTGGTTTGTGGTTCTCATCTTGGAC
4993	CaTSNP7981	TACATCCTGAAATCCGTCCGTACACGTTTCTTCATCAGTAAGCGCCGCGCTCATCCACGT[C/T]TGTACGTTACTCATCTGAAAGAGGAACGAGTCGGTTCCAGAGCCGGCGGCTCCTAACTGA
4994	CaTSNP7982	TATGTTGATATATTACTATCTTTCTGTATGTTCTCTGGAATTTTTTGGGGCTTAAGGTCT[C/G]GGAAAAGTTGAATGTTTCTTATATGTTAATGCTTCTTATGTATCTGGGATGAAAAAAACA
4995	CaTSNP7983	AAAGTAACCGTACATCACTGATGCTGCATAGACCTGCCCTACCCTCAGTTTGCTGATTTG[A/T]GCAACAGATGTTAAATCTCCTGCTCTGTTACCCAGAATGAGAGCCAAGTGATTTTGGATC
4996	CaTSNP7984	GCATATTGGCAAGGTAAATCTTCGTGCCGGAACAAACAGAATAGCTCTGCTCAGTGTTGC[C/T]ATTGGACTTCCTAATGTGGGAGAACATTTTGAGACATGGAACACAGGAATCCTAGGTCCG
4997	CaTSNP7985	ACACACCGTGAAAGATATTCACATGGATAATTTCACTATCTCTGTTGGTGGCCATGATCT[C/A]ATTGTTGATGGTTCTGTCACACTCTCTTTTGGAAGACATTATGGGCTTGTTGGTAGAAAC
4998	CaTSNP7986	CCCTGTTGATTTTCCTTATAGAACAGCTGATGGAAGATATAATGACCCTTTTAATGATGG[C/T]GCTGGAAGCCAAGGAACTTTCTTCGGTAGAAATGTTCTTCCCGTCGATCAAAAGAAAAAG
4999	CaTSNP7987	CGTCAAAGTACTTTTCTTCTGGGTAAATATTCTTGAGGTTGTTCGGAAGGGTGATAACCT[T/C]GATTTCTCTGTTGGGGTCGCTTCTGCGTCTTTTCCTTTAGATAATTTCTTTGTTTCTCCA
5000	CaTSNP7988	CTCAGAGTCCTGAGACTTAGCAATATCAACTAGGATTTTAGCAGCGGGATGAACGATATC[A/G]AGAAGTTTCATAATGGTGGCGCCGTCATTAGAAATGGTGACGGAGCCTTTATCGTCGTGG
5001	CaTSNP7989	AATCTTTGCTCTGGCCGTGTTTTCACGATCCAGAAGAAATGCAGGAACAGCTCCTTCCTG[A/G]ACATTATCATCAGCCTTACGCCTAGATGTCGACTCTTCATGCATGGCCAAAGTCTTCTTC
5002	CaTSNP7990	AACACTTGCTCCTGACTTCATCAAAGGAGGTAGAGCCATGAGATCAGTCTTTGATCTCCT[G/C]GACCGTCGTACTGAGATCGAACCAGATGATCAAGATGCTACTCCTGTACCTGACCGTCTT
5003	CaTSNP7991	AAACAACTCATATCAATTTCTACTGCCGTCCGCAAGAAACTAAGCCCAAAATTGTTGAAG[A/G]TTACAATGAGACTGATATGGATATGAGAGATCATTCCAATGATTTTGGCAATACATTTTT
5004	CaTSNP7992	AGAAGAAGAAATGTAACACAGTCACATGTTATGCAACAAGAAACTGGTGGAGGTGCTTGT[G/A]TTGAATTAGGAGAGTATGGTCTTGAAGGTGAGATTGAAAGGTTGAGAAGAGACAGGACTT
5005	CaTSNP7993	CTTCTCTTCCTCCTCAAGTTTACTCTGAATATTTACCCTCTTCTCAACATACTGTTCTCG[G/A]GTGACGAGACCAACAGTTTCTTTCTTAAAAGCAGTCTCAAGAATCTCGGAAGTGCTGGAG
5006	CaTSNP7994	TGTCACGATCTTACAAAACTTAACATCAGTGGATGTTCAGCATTTAGTGATAATGCTCTG[A/G]CTTACCTTGCCGGTTTCTGTAGAAAACTCAAAGTTCTTAATCTCTGTGGATGTGTTAGAG

5007	CaTSNP7995	CTTCTTGGATTCTGAAGGGAGAGTCACAAATTCCGAAGCTTTGAGAAAGAGAATATTTTA[C/T]GGAGGCCTTGACCACAAATTACGAAATGAGGTTTGGGGTTTGCTTTTGGGATACTATCCA
5008	CaTSNP7996	TTTTGCATTACATCTCTAAGAGAATTTAAATCAGGCATGCCCCGCTGTTCAGTATTAGCA[T/C]CAAGAATCTGACTCAAAGTTTGGGGGTTGGAAAGCATACTTTGCATCATTTGTGAGATAG
5009	CaTSNP7997	AGCTAATCGGTATAAACTGTCCCTTATGCACAATTTGGTTCTAATATCCAACTGATCCAA[T/C]GCCTGTTGGAGGTGGCAAAAGCTGTTTGCTTCAAGCGAGTTTTGGTTCAGAGCAGAGCTT
5010	CaTSNP7998	GGGCTAGGAAAAGCACAAAATAACCCAGAAGAGCCATGATTAATGATTATGATGATGTTA[T/C]AAGAGAAATAAATTATTTGATGGAAATTGGATCAAGTTTAGAAGGGTATTCCAATATGTG
5011	CaTSNP7999	GCATAACACATCCTTCAGGAACAACTACCACTGCACGTTTATTGCCAAATATTGTCCACA[G/A]ATAAAGCACAGTGAATGCTGTTAATGCTCCACATCCAGTACCAAATATCATTGCAGCAAG
5012	CaTSNP8000	GCATCACTTAGTTTTGGTTTTATTATCTCTTGCTTTGACTAAGGTTGTTGATCAGATAGG[T/C]GAGGGCTTTTTAGTTTTATTTTTAGAATTTTCTGGCAGCAACAAATAAGAAGGGTAGATT
5013	CaTSNP8001	TCAGCTAGTTTTCTTCACCGACAGCCCTACAACCTCAGTCTCCGTCGTAACTTCCAGCGG[C/A]CTCGACGCCGCCGTCATCGCCTCGCTCCCCTTGTTCTTCTACGATCCGAATACTCATCCG
5014	CaTSNP8002	CTCCAAAACAACACTAGGATATTTCCAAGTAATCGCGGACCCAGTCTCCACCTGGGTCCA[T/C]GATATCTTCGACCTCGCACCAGCACACACACCTCTCTTAGTCACAAAATTATATATCCCA
5015	CaTSNP8003	TGGCAATTCATATCGGTTCTTTCTCCGTGATATATATGAGGATGTGATTCAGAATTTGGT[G/A]GATTTGTCAGCTGCGGATAATATTTTCATTTCACAACCCTGCCGAGACAACACATTGTAC
5016	CaTSNP8004	TGTTTACTTGACGGCCAGATCGATGGGTCCGGATGCCAATATCAACTCGAATTACAACTT[G/A]ACTTGGATATTCTCAATTGATTCTGGGTTTTCCTATCTTGTGAGACTTCACTTTTGTGAG
5017	CaTSNP8005	GCTGTTTCTCACAGCCCCGTCCAGGTGCTATTTCAGTGAGTCGACCTGGTCGGTCTAAAA[C/T]ACTAACTCCGTCAAATCTTGACGATCTATTTTGTGCTGAAATTTCTTCGTCTCCTCGGTA
5018	CaTSNP8006	CTCTCATCTGCTGCTGTCCCAACAACAAGAAGCCTTTTAAACTGGAAAAATACATTAGAT[G/A]ATCAAGTTACATGGTGGTCCAGAATTAAGAAATGAAGAAGAAGAGTTAATGGCAGAGAAA
5019	CaTSNP8007	GTTCAACCCGTGCTGTTTCTTGGCCTGCAAAGCCAGCACCACCTTCTGACATTTCACACT[C/A]CGCAAATAGGAAAACTGGAGGATCCTCTGAGAATGTTGCAAAGAGCTCTCCTATTCAACC
5020	CaTSNP8008	ATTCTGTACATGAGTATCTCGTGATGACCTATGGGCCAATTGACCAAAATCATCATCTGA[C/T]TCATCATCTTCATGGTTTACATTTACAAGTGGCAGAACTGGAGTTTCTACTTCTCTTGCA
5021	CaTSNP8009	GCAAATTTGTTGATATGCTCCCTACTAGCTCTCCCATTGCTACAACAAATATTTGGAATG[C/T]TAGACTGCCATTGACAAATGAAGCTATTGTGATTCCTACTCAGGTTAATTATGTTGGGAA
5022	CaTSNP8010	GCTCAACTATTTCACATATAAGGCTGTGAGGACTGTTCTTCAGCAACTGTATGAGATGAA[C/T]CCACCTAAATATACATGGTTTTATAATTTTGTTGCAACAAACAAACCAGCAGATGGAAAA
5023	CaTSNP8011	AGAGGCAATGGCTGAAAGCGGTGCCATAGAGGCTCTCTTGGAGCTCCTAAGATCTCATCA[G/A]TGTGAGGAACACTGCTGCAAGACTCTTAGAAGTATTGCTGAACAATGTAAAGATTAGAGA
5024	CaTSNP8012	TAAAGAATCAAAATTGTTGCTGTCAACAAACTGTGGAAGAATGTGGAACTGTAAAAGTTC[G/A]ATTTTTTTGTTTTCGTTTAGCGAGTTGAGGAACCCGGGTTTGAGATTCGAGAAAGCCGAG
5025	CaTSNP8013	GCAATCTTAGCTACGGACGGGGCTTAAACCCTTACTTTATCGGCAACTCCAATAGGTTTG[G/C]AAGTCCTGTTGGATATGAAAGTGGTAACGGAGGAAACAGTTCTTTCTTCAGTTCCGTGAC
5026	CaTSNP8014	TCTACCAAATAAATATTCTTTCTTGGATTGGAAGCCAGAACCAGATGTTTTATCTAAAGA[A/C]AGAGATAGAAGTTGACCACCGTTGAATATCTTAGCACGTTGATCACCCCATGTTAGATCA
5027	CaTSNP8015	AACCTTGGGTGTTTAATTATGTACTTAATTATTGTTGCGGATGTACTCTCTGGAAATCAA[G/C]AAGAAGGAGAAGTACATTTGGGTATTTTGCAACAATGGTTTGGAATTCATTGGTGGAATT
5028	CaTSNP8016	GAAATCAATAGCCTTCTTCAATCCATTGTTAGCATCGTGAGAAAACTCTTCCTCGTTGCG[T/A]ATAGAAGCATTAGGTCCACCGGTCTTCGTTTTAGCATCATAAGTGCCCGCATCGTGCCAC
5029	CaTSNP8017	AATTCTGAAAGTAATTTCCTTCCCTATCAAGAGCTTCCTCAGAAACTCTCTGCTTTCCCA[C/A]GCAAATGGCTCATCCACACCACCTCTACGTGCCAATCTAGGAGCAATCAGAGACGATAAA
5030	CaTSNP8018	TAGTGATTGGGTTATGGAGAGTATGAATAACGGCACTGACAGTTACGGTGTCACCACCGG[G/T]TTCGGCGCCACGTCACACCGGAGAACCAAACAGGGTGGTGCTTTGCAAAAAGAGCTCATA
5031	CaTSNP8019	ATTCCCGGAACAGATACTGCCGCAACAACAGCTACCACCGCCGCAACTACCTCAGGCACA[T/G]ATGTATGAACAGCAATATCTCCAGATACCATCGCAGTCACAGGTGCAGCAACTTTTTCAA
5032	CaTSNP8020	GTGGTTGGTTTCAAAAGGAGAGTTAATTGGCTTGAACCTGTCAAATTGACTTTCATCGCG[G/A]GTAAAAAAGTTTGCTGCTTCCATTACCATTCCTTGACTCGAAAAGAAATTGTCCTGTTGT
5033	CaTSNP8021	GGTTGTCTCATCAGGGGCAGTCCACAAAGACATAGAGTCAGTCCCAACAGTTTCTGGAGG[G/A]GATATAAAATTTGACGTTAATTCTGGTTATAACCCTCCCAGTGAAGTGAACCATCAAATG
5034	CaTSNP8022	GGTATTCTTCCCGCGAGGTCTAACCAGCACCAACCTAGCACCGATGCTGTCCCCAACGAC[A/G]GCGAGGTTTCAATTAGGATAATCGGTTCGACAGAGCAGGATAATCACAGGGAAGAACAGA
5035	CaTSNP8023	AAGTGGGAGAGGGAGAGTGAAACGTTCGTTCCAGATAGGGGAAGTGTTGCCGGAGTCGTC[A/G]GATTTAGTGGCAAGTCGACGGTCAGGATCGAGCCAGAAGACGACGTAGGGTTTGAGGTCG
5036	CaTSNP8024	AGAAATATAGAGCTTGGTTCCAGTTTCTATCGAAGGCGCACGACCACCTTGAGCAGAGTA[A/C]GCTGCCGCAGCCACGGGTTGATCTGCATACAAATCATGCTGCCACGTCGTCTCCGGAGCC

5037	CaTSNP8025	AAGACAAACAACATCCAATCTGGGGCATCTGAATAAACCGTCCATTCAAGCATTGATCCA[C/T]GGGTTGAACCGACATTATTATTCCATAGCCATCAATTACAGGAAGAATGAACTTGAGGAG
5038	CaTSNP8026	CGTACAGCTCCGCTAGCTTTTCTCCTGCATCCTCTTCCTCATTATTATTATTGTCATCTA[C/T]ACCTTCATCAGTAGCTACATTCTGTAGAGCAGCAACTTCTTGTCGAACTTTAACAAGTTC
5039	CaTSNP8027	GAGAAGATTCTCTTGCAAGCCCATAGCATCAAAACTATCATAAACCTCATCAGAAGTTCC[A/G]AAGAAGTCCTCTCCATCAGCAGCAATTAAGTCATTCATTTTGTCATCATATTTTTTAGCA
5040	CaTSNP8028	TTACATTTATTCGCCTAAGAAACCAGATACTGCAGCTGTCAGTGATCCAGGTCCCTCTAC[C/T]AATGCTGTGGCAGAAGTACCTATCATTGTCCCTCAGAAAGAAACAGCACAACCTAAACCA
5041	CaTSNP8029	AATCTCTGCTAGTTTGGTGGAAGTTGGTGGTGTCAGCATTATAAGGCGTATATTCTGTTC[T/C]CTATCTATCTCTGTAATAGTACATGTCAAAATGGTATGTGCAATTGACAGTTGATTTCAG
5042	CaTSNP8030	GAATCTACGGCTGCGTCCAGGTGGAGCCAGCTGAGCATTATTATACGGAGGCTGTGTATC[G/A]TAAATTATAAACTTGGTTCCGAGAAAATTAGACCTAAGCTTTCCGATGTAAGTGCTACTA
5043	CaTSNP8031	GAGAGCGGGGAATCCTTCTAGAAGGTTCGGTGATAGTAAATCGAAGACTTCGCCAGTTTT[G/A]TCAATTGGGCTTATAGTTGTGGGAGGTTTGTTTCTCATTGGTTACTTGTATAGGGGCTCA
5044	CaTSNP8032	TAGGGTCTGGTGTTGGCGTTCGAGAGCACACACTGTTTGAATCAATTCCATTCTTTCTCA[A/T]TCGGATTGCATCACTCTCCGATCTTCAAACCCTAACAACAATGGTCAGAGGATTGCTCAA
5045	CaTSNP8033	CATCATATTTCCTCTGAGTGTGACAGCAATCCCAAGTGGTTGACCTTCCCTGATCTTGAA[G/A]GTTGCAACGGAATTCCTCGCCCGAGTCTTAACAGGTCTCTGCCCAGTGATCAGTGCTAGA
5046	CaTSNP8034	GAATCGGCTTCCGAAGAAGCCATTCCCGAGTCGGGGACGAGACTTGACTAGTGAACCGGA[T/G]AACGTGGACGACGTTGCCTCTGACTAAGATCTCGCTACCATGAGCTGCAGGAACCAGTTG
5047	CaTSNP8035	GAAGAATGGTTGCTGTAGAGATCTTTGCCAAGCATTGGGTACTGCATGCTGCTTGAAGAG[T/C]GATGAAATGCTTTTGCTTGCTGAGGTTTATGAGCATAAGATTTATCGATATCTTGACATT
5048	CaTSNP8036	GAGGTTTGAAATCACACTCTCATTCAAAACCACTGCACTTTCTTCAACTTGTTCAACATT[T/C]GAATTTCCACCATTCTCATTAACAACATTCTTGTTACCATCATCGACAATCTCGCCATTC
5049	CaTSNP8037	AAATAATTAATTCACATTACAATGTAGCTCAAAACTTCAGAAAGGTAAGGATCCTGCTGT[G/A]ACCCTCTCTTCCTCTCGTGACAGTTAACAAAACCAGCATGACCATAAACAATAACACAAA
5050	CaTSNP8038	AATAGCTCTAGGCCACTCAGCTTCTGGAATGGCAAGTTTTTCACGCATTTCCACTGGAGC[C/A]GTGTGCACACTGAGCCCAATGACGACAATGCTGCTCCTTTCCTTTGTATACCTATCAGCT
5051	CaTSNP8039	CATTACAATTCCAAGGTTCATTTGAGCAACTCCAAGAAGAATTGACATTTTCATTTTCAA[C/T]GAGTTCAGGAATGGTAATTCACTGCGGCTACCATGCCATACAGGATCCACTCCAAATGGG
5052	CaTSNP8040	AAGTGCAGGCTCTCCTACTTCGTGGATTTCGTAATTGTTTGTGTTGGAGGGACACCACCC[T/A]CGATATTTCAAGTTTTGCTTTTCCATTTCCTATATTCAATTCTGTTCTGTTCTGTTCTGT
5053	CaTSNP8041	CCTGTTGGCATGATACCAGGGAATGAAGGAAGAAGGATCGGAAGAAGGAGGAGGAGGAGA[T/G]ATAGCAGTTGTATTATGAGGTTTGACGATAGTATCTGGAATCCTTTTGTTCAAATCTCTA
5054	CaTSNP8042	AACAAGGCATGGCTACTGGAAAGCAACAGGAAAGGATCGAAATATCATATTCAATTCTCG[T/G]TCTGTTGGAGTAAAAAAGACACTAGTTTTCTATGTAGGCAGAGCTCCTAATGGTGAGCGA
5055	CaTSNP8043	AACACCATCTTTGCCACCACTGGCACAGAGAGAACCATCAGGTGAAACAGCAACAGTGTT[G/A]ACATAGCCAGAATGGCCAGCGAGTGTGTTTCTCAGCTTGCAATTAGTCAGATTCCAAACC
5056	CaTSNP8044	TGTAACAATTCCCACTCCAACAGATGACCACAAAATAACCAGCCACAAAGGCATCGACTT[C/T]GGATATAAGCCATGTCCAAATTTGATGCAAATGAGAAGTTCTACAATACAAATGGCGAGG
5057	CaTSNP8045	TCGAAAAGCTGAGGAAGTAGTTGATCCAAAGCTACCTGAGATTCCATCTTCAAAGGCTCT[T/C]AAACGCGCTCTATTAATTGCTCTTCGTTGCGTTGATCCTGACGCAACAAAGAGGCCTAAA
5058	CaTSNP8046	TATAGATGCAAGGTTGGAAGAGCTTTTGATCGATGGTATTCTTTATGCCTTCCAAGAGCA[G/A]ACCAGTGACGATGCTAATGTGATGCTTAATGGGTTTGGTGCCGTTGTAAACTCGCTTGGG
5059	CaTSNP8047	AGAGGAAGAGCCAAGCGGCGATGAGAACAACGAAGACGATGAGTGAGATTGGATGCCATA[A/G]GAGACTTAGGAAAATCACGATGAGAAAGACGATTGCGTAGTTCATCTGGAAGTAACCGAT
5060	CaTSNP8048	TCATGGTGGTAACTTTCAAGGAACACCTATTGGAGTTTCAATGGATAACACACGTTTAGC[T/C]CTTGCATCAATTGGTAAACTCATGTTTGCTCAATTCTCTGAACTTGTTAATGATTTTTAC
5061	CaTSNP8049	CAAATGCTTCACTCAAAGATGCAGCCAGAATCCAATATTCAGTCACCAGACTATAATTTA[G/A]CCCCCAGAAGAAGACTTCCCAGAAGTTTTAACTAAATGTTGGGTCAGCAATGCATTGTAC
5062	CaTSNP8050	ACAATACGGACACCTGTTGCTACCAGCACTCGAAGATCCAGCAGTCACAGTGGATCAACT[T/C]CATTGGCATTGATGACCTCTACACCAGACCAAAGTGGTAGCTACGTTTATGTCCCAGGAC
5063	CaTSNP8051	TAAATTGTTGGCAGATCAGTCCTTTGTATCTTCTGTCCTTGCATCGCTTCCTGGGGTTGA[T/C]CCAAATGACTCATCCGTTAAAGATTTGCTGGCTTCCATGCAAAGTCAGTCTGACCAGAAG
5064	CaTSNP8052	CGATAAAAGAAACGACACCGCAATTGGTCGCGCTCTTTCTCAGCTTCTTTCTGCGTTAAA[C/T]GATATTCCTGTTAGTTCCAGAAAATACGAAGTTGTTCGATCGTTAACGGAGAAGATTATC
5065	CaTSNP8053	ACTTGCTTCCAAGCCTGTTTCCACGGTCACTATTCCGTTGGAGGATTCTGTTGATTTGAA[G/C]AATGTTTCGGATAACTGTTGTAAAGTTGAAGTTTCGTTTTCGAGTGAAGGTTCTTCACCA
5066	CaTSNP8054	ACACGGATCAGGGACCAATCCACCAATATTTGCTGAGAAACAGATTTGTCAGGAAGAATC[G/T]TCTGGCGGAGTAGAGACTTAGTCTTGAAGGGTATCACATTGTGATAACTGAATCTAGCAT

5067	CaTSNP8055	CTGTGCATCAACCATGTTTAGTTACATTAATCTTTATGGACATTACCCTCCTGGACTCTT[T/C]GCCAGCGAGTGTCGTGAAGGGAAGTCAGGTCTAGCATGCCCGGCAATGCCGCCATCTGTG
5068	CaTSNP8056	TCACGTCGAGGAGAGAAAGCGAGTGAAGAAGATAAGAGGAAACGGTTATTGTGGAATCGA[C/G]TGGAGATTGAAGAGGGAGAGTGAAAGTGGCGATGATGACGATGAGAGAAGGAAGTTCCAG
5069	CaTSNP8057	TTTCTCAACACCATACTCAGTGTTTTCTTTGCGACATATCCATCGCATACCCTTGGCAAT[C/T]GTTGCAACTGCGTCCGCAAAATAACTTGACTCTCGAATTATAGCGTGTCCGCCTGGTCTT
5070	CaTSNP8058	CACTGTCAACCTTGTCTCGTCATCCACGGACAACACTTTGAAGCTTTGGGATTTGTCTAC[A/G]TGCACGTCTCGAGTTATAGACTCGCCAATACAATCGTTCACAGGTCACATGAATGTTAAG
5071	CaTSNP8059	TACAATTGTTGGTGATGCATATATTCATCCATCTGCCAAAGTTCATCCATCTGCAAAGAT[T/C]GGTCCTAATGTTTCTATCTCGGCAAATGTTCGTGTTGGTGCTGGTGTCAGGTTAATCAGT
5072	CaTSNP8060	TATTTTCCATCACCTTCTTCTTCCTCTTCCTCAATGCTCTCATCCAATTTGGAAAGTGGA[T/C]TGCGACCATGCCCGCTTCCAAATCCAGGCTGAAAAGGTGATCTGGCAAGAGGACCATCCA
5073	CaTSNP8061	AGGGCAGACTGATATTCCTTCAATTGCCGCAGTCGTCAGCTCTAGACAGTGGCCTCTAAT[A/T]TCAAAATATAGGGCATGTGTTCGCACACAGTCTGCAAAAGTTGAAATGATAGATAATTTG
5074	CaTSNP8062	ACTCTTAACAGTCCAATAAGCCATTTTGTCCAAAAACGTTGTCGGTGCGTGATGTTTATT[C/T]AGATCAATCGTAACGTCCGCTTTGTATGTTTCCCATGGCTTGAAGCAATTCCACTTCCAT
5075	CaTSNP8063	TAAGGGCAGCGTTCAAACATATTTGGGTCGTCCGTGGAAAATGTATTCTAGGGCTGTATA[T/C]ATGTTGTCGTATATGGGTGATCACGTGGACCCACATGGATGGTTGGAATGGAATGGAGAC
5076	CaTSNP8064	TCTTGCCCCTAATAAGAAACTGAGGGTGTATTGTATGGTTGCACATACATTGCAGCCACA[T/C]CTTGTTGCTATTGGAACCAATATTGGTGTTGTTATCTGCGAGTTTGATGCTAGATCTCTA
5077	CaTSNP8065	AACCGATATGTTCCGTACTAATTACACTGCTCGAGTTTCCTTCCTAAGAACCCATGATGC[T/C]GAAAAGGCATTGAACCATTCTCAAAACAAAAACCCTTTTGAATCTTCAGAAGTTACTTTT
5078	CaTSNP8066	CAGTGTTACAAAATATGGAAAGAATTCTTTTATGTGGTTTGGACCGATACCGAGGGTGAA[C/T]ATCACAGATCCTGAGCAAATTAAAGATGTACTTAACAAAATCTACGACTTCCGAAAGCCG
5079	CaTSNP8067	TTACGCAAGAGTAAGCACCAAGGGTTATATCGGAATGTACCAAACAAGTTCCAAACCCGT[G/T]GATGTTCAGAAATTCAACAGTTTCCAACAAACGTCGTCGTTTCTTTTGCTCCGGTTAGAA
5080	CaTSNP8068	ACCTACTGATGACATAGAAATGGGCAGGAAAGCAGTATGGGAGATGAATGCTGGGATGGA[C/T]GATTCAGATAATCAACTAAACAATGACCGCGTGTCTATAAAAGATGGTGAGTTGTCTCCT
5081	CaTSNP8069	CGTCTCCCAACACCTCTTCTCCGCCAAATACGTCCCTTTCAACGCCGATCCATCTTCCAC[C/T]GAATCCTACTCCCTTGACGAAATCGTCTACCGATCTCAATCCGGCGACCTCCTCGACGTC
5082	CaTSNP8070	CCTCCACTCTACGGTCTAGAGTATTCTCACAAATATCGTGGCAACGGTTATTCATAGTTT[A/G]GTATGCATAGATTAGATTTCAGTTTTTGTTGTTTGTAGTTGTCTCCTGACTAATGCAGTA
5083	CaTSNP8071	CTCTGCAGCTGCAGGAGCTTCAGAAATGGCAGATAACACAACAAAGGTACCAAATAAGAT[C/A]GTAATCAAAGCAGTGAAGTTGACAAGTAGAGCCTCAGAACCATATTTTGCCAATCCTGAG
5084	CaTSNP8072	ATTTACCTTCAATGCAAGGCTGTCTCCCTTTCCAGAACCACTTCTTTGCCATGCTCCCAA[T/G]AGATAAAACAAACAACATAGAGCAATCACAGCGAAAATCGACAAAGGCCTTCGTGTTTTG
5085	CaTSNP8073	GAATGGAAGGAAGAATTGACACTTTATGTTAAGGATATTAACACACCAATAAACCTGACA[A/G]TTTGCGACAAAGACACTTTCACTGTGGACGACAAAATGGGCGATGCTGACATAGACATTA
5086	CaTSNP8074	GAGGTTTTTCCTCATAGACCCTCTGCCATTCTTGTTATATCTGGTCACTGGGACACCTCT[G/C]TTCCAACCGTCAACGTTGTACAACACAATCAAACCATCTATGACTTCTCTGGCTTCCCCA
5087	CaTSNP8075	CACTCTGTGCAGCACTTGCTTGATTTGGTCCCCTTCTTTTATGTTGAGTATCTTGATGGT[T/G]CCAGCTTCACCAGTTCCTTCAGTCAACTGAACACTGTCAATAATTGGCACAATTTTTGGG
5088	CaTSNP8076	AATCATTGCTGCTTGGTGTTATAATCTTCCAAGAATTAGTAATGAAGTAGACATTGTTCT[C/T]GATCAACTCTATTCTGTTATTTCAAAACCTTATTGGGATTCAGCACGTAAATTGGTTGAT
5089	CaTSNP8077	TGGAGGATCAGCAGTCTTTGTGGCTCGGAATAGGTTTGCTGTGCTTGAAAAAAGCAGCAA[C/T]CAAGTTCTTTTAAAAAACCTGAAGAATGAGCTTGTTAAAAAGAGCGTGCTCCCGATTGCC
5090	CaTSNP8078	GGGATTGACCCATTTTGATGAGATTCTGCAAGAAGCTGATGGTATTATCCTTTCCCGTGG[G/T]AATTTGGGCATTGATCTTCCACCAGAGAAGGTTTTTCTGTTTCAAAAATCTGCCCTATAC
5091	CaTSNP8079	GGTTGTTTTCTTGAATGAGCGACCTCAACCAAGGCCTGGAAAAGGTGTCACCAACACTTG[T/C]GAAGTCTGTGAGCGTAGCCTTCTCGATTCATTTCGATTCTGTTCTCTTGGTTGCAAGATT
5092	CaTSNP8080	TGTATCATCAAGTATATGAAAAATGTCATCTTGAAGTTGAACACCGGAGTCAAAAACCGA[G/A]TTAAGACACGATTCGGCCGAGTCAACTCGGGTAAGCTGACACTCTGACGAATTAACATCA
5093	CaTSNP8081	TTCAGCCGACGATCCCTCTGCCTTCTTAAGCTTCTGGAGAATGGAGGAAACAGCAGAACG[C/G]AGAGAAACGGAGTCATCACGGAGTCGGATGACATCACGAGTAGCACGAGGGACGCGGAGG
5094	CaTSNP8082	CACATGCTAGCTTCATTGAAAGAACAAGAGTGGAAAATGGAGATTGAAGAAATTTGTGAA[A/G]CACAAGCACTTCCATTGCTTTCATTGAATCATGTGTCTCTCTTGTGCAGCTCAGTGTTGG
5095	CaTSNP8083	CCATCCGCGCCACCTACGCTCGTAGTTTACTGGTGGACCGGACAAAACCCAAAATCCTCC[A/C]GGACGAAGAATTCGGTGTATTTCAAGGAGATAAATTCCACCAAATTCAGTCCATGGGATA
5096	CaTSNP8084	ATTTAAGCCGAGTGGTGCTAATGGATCCTATGGGTTTGAATATCAGCCAGTAGAAGCTTC[C/T]ATTGCAATTGCTGGATGTGATACTACCCAGCGAAATGTATTCTTGGATGCAGAAAGAGGA

5097	CaTSNP8085	ATTGAACAACTTGATTGAAGATGCAATTATAGAGGCTGAGTTAAGTGGAGCAAAGGTGAT[A/T]AGTCTAGGACTTTTAAATCAGAAACAACATTGGGAACTTTATATAGAAAGGTTCCCACAA
5098	CaTSNP8086	GCTGGCTGAAAATTGGAGTGATATTTCAGATGCTGTCGTTTCTGATGCTAAGCATGCATT[A/G]TCTAAAAATACCGAGGACAAAACTGGCAAGGAGGTTGTGACTAATGTGTTCCGTGCTGCC
5099	CaTSNP8087	TTGCTTTGGAAAGGACTCTATAGAGGAACAAGATGAGAGATTGTTGAGTATAATACTGAG[T/C]TCTGTTTTCCCGTCAGTTCAAAGACAAGAGATTCGTCGCATGGTTAAAAACATGGCGGAG
5100	CaTSNP8088	TACAGACAAATCTATAAGCTTCAGCCAAGCCACGAACTGAACGAGCCTGATCATTTTTCT[C/G]ATCTTCGGCGTGTCGTCTAGTTAAATCAGAAACATCATCATAAGCTGTAACACGTTCTTT
5101	CaTSNP8089	CTGGTTCCAAAAATTTCAGGTCAATGTCGATAGGCCAGACCTTGAGGCCAAGTAGTCTGA[C/T]GGGAGTAATCTGACCAGGTATGATGCATTCAGGACCAGCTGCTTCCACAATAGCCTCATC
5102	CaTSNP8090	GTCACCAGTGCACACACCTTCCCCGATGAGGGCGCCTCCGGTGAAGCCCTTGAAGCAGCC[C/A]AAGCCAAAGGCGAGGGACCCAAATAAGAGGGAAATGAGCCTTGAAGAAAAACACAAGTTG
5103	CaTSNP8091	CCCTGGTCGTCAAAAGATCATTGTTAGCAGGAAGTGGGGATTCACCAAGTTTAACCGTCC[T/C]GATTATCTGAGGTACAAGTCAGAGAATAGGATTATGCCTGATGGTGTGAATGCAAAGCTT
5104	CaTSNP8092	GGCTCTCGCACTGAGTCCAGTTGTCAGAGCTCTTGTTGATCCGGATGGTGCACTGAGGGA[G/C]ATAAGGAATTTGGATAGTATTAGCTTTTCAGACTGGTTTATGTCCAAAGGTGGCACGCGG
5105	CaTSNP8093	AGAATCAGCTCTAAATGAACAGGCTGGCAAACTATTGCTCGAAAATTATGAGGAGTATGC[C/T]AGACATGCCAGGCTTTACACTGGAATACACGCAAAAGCAAAGCCCAAATTCAAAAGCGGG
5106	CaTSNP8094	CAATGGATGGGAAGGCATGATCTTCATTGGAGTAAATGCAGCTATAAGTGTCAGAGTTTC[A/C]AACGAACTTGGACTTCGACATCCAAGAGCTGCCAAATACTCTGTCTACGTGACAGTCTTT
5107	CaTSNP8095	AGGCTTGATCACATGTTCTTTCAAATCAGCTGCAATTTCATCATTGGTAACAGTTTCATC[G/A]TGCTGTGTTGAAATTAACACAGTGTGGACACGAATAGGAACCATGGCACCTTTGTCATTG
5108	CaTSNP8096	ACCAGGGCAACCTCCTTAAGTGCATTGGAAGAGGACTTGCAAGAAAATTCCTCAATTGCA[C/A]GCAACAACAACAATGGCATACCCTCTTCCTGAGCAACAGCAGCAGCAAGGTAACTCTTCT
5109	CaTSNP8097	GTAATAAGCTAGCTCATACATGGCTCGTGGAAAGATCCAGATCAAGAGGATTGAGAACAC[G/A]ACAAATCGACAGGTCACTTATTCAAAACGAAGAAATGGTCTTTTCAAGAAGGCTAATGAA
5110	CaTSNP8098	ATTACAAGTGGCCTATACTATAAAAGCTCCCAAAATTCCATCCTCTCTTTTCTGGTTCAG[A/G]ACGGTAAAACATGACAACAATGCAACACTAGAGAATCATTTCAAACGATCACAACGGGCT
5111	CaTSNP8099	AGTCCACCTACAGATGTCTTCCGTGCTGTAGCTGAAAACGCTGTCCAGCTGTCTGGGAGT[C/G]GAAGTGCTGCTGCTGATCTTTTGGATGAGTTATGTTGCAATGAGAGTCTTGCCAGTGAGT
5112	CaTSNP8100	TATGATTATGATAAAGTGGAACTTGCAAACATGAATAGGCTCTTCTTTCGTCCTGATCAA[G/A]CTGGTATGACAAAGACAGATGCAGCTGTACAAACACTTTCAGATATAAACCCTGATGTTG
5113	CaTSNP8101	ATCAATCTTTAACCTATTCACTGTGGTTCCTGTGTTTGTGACTGCATATATCTGCCATTA[C/T]AATGTTCACAGCATAGATAATGAACTTGAGGACTCGTCACAGATGCAAGGTGTTGTGCGT
5114	CaTSNP8102	GTATTTGATAGACAGTGTTGGATGGTTCTTGTAAGCCTCTGGAACAAGAATCATCATGGA[T/C]TCCTCAGGGCTTCGACCACTTCTTATTAACAATTCTGCAGCACTGTCAAGATTTGCCGAA
5115	CaTSNP8103	AGGTAAAATCGCAGAGCTCGATGGAATTAGAGTTCGCGCTTTCTTTAAGTGGTGGTCCAT[C/T]ACCGGAATTAAATCCTCCGGTGATGATGTTGTTTTCGAGGTTGGAATGGTCACCGCGAAG
5116	CaTSNP8104	TAAGATGATACATAAAGGAAAGAATACAAAGAGTAGGATTATATCGAAAGGTATTTCTGC[A/T]GGACATTCGAGGAACTGTTATAGAGGGCTTGTTCAGGTTCAGTCTAAAGCGGAGAATGCT
5117	CaTSNP8105	GATTGATGATATTGGAGGCGTTGAAAATTTTGGATCCAAATTTCTCAAAAGAAGAAAAGA[C/T]CAGAATCTTCCTGTATTTATTCCTCCAGAGCAAACGGCAGATGCTTTACTTGAGTCAGGT
5118	CaTSNP8106	CGTTGTGTTTGTTGGTGATGGTGGCAAAAAGTCTGTTATCACTGGCAACAAAAACTTTAT[T/C]GATGGTGTTAACACCTACAGAACCGCCTCTGTTGCTATTCAAGGAGATCACTTTACAGCC
5119	CaTSNP8107	TCAGCCAGGTTATGGGGTGCCCCCAACCTCCCAAGCTGCTTACGGCAATCAACCTCAATC[C/T]GGTTATGGAGCTGGATATGGAGCCCCTCAAGCTCAAAAGCCAAGTGGCACTCCACCTCCA
5120	CaTSNP8108	CACAGTTGGTGGAGATGAGAATATTGTGTTCAATATGTCGTTAAACCATCCACCATTTGT[T/C]CTAACTGGACCATGACCATCTGGTGAAGTGTTCATGATAAAATATTGAGGAATTGAGATC
5121	CaTSNP8109	TATTGTAAAGTCCCATTGTTTGATAGAACCAACGTTTTCCCAGTCACCTTCATGCACTTT[A/G]GCTCCATGTACTGAATCTGATGCAATATTAGGAATATGATGAAGTTGCTTTCTAAAAATA
5122	CaTSNP8110	CAACTTGAAAGGTACCAAATATTGTTGCACTATTAAAAACAAATGAAGTCATATATAAGA[C/T]GGAAAACACTACAATTAAGCAGCACCCTTTCCCTTCTTCCTCTGTAACTTCTTCATGTAA
5123	CaTSNP8111	AAAAATTCCTTGCAGAGGAGACATCATAGATCTGTCCTTTAACAGAAAGCAAGAGAGGTT[G/T]ACTAGGATCAGAGCCATTATACAACCGCAATTCCGGTTCAGTAATTTCACCAACCTGAAC
5124	CaTSNP8112	TTCACCGTCTTGCTCATGCCAAATGGATCGGATCGCGTAACGTCTCATCCACTCCAAGAA[T/C]TGCAGCCCACAAAAACAATTGAAGATCCAGAGATCAATGCCTGGCTAGCCTTGGGAACAA
5125	CaTSNP8113	TGATCTTCCTACTCAACCTGAAAATTATCTTCATCGCATAGGGCGAAGTGGTCGTTTTGG[A/T]AGAAAAGGTGTTGCAATAAACTTTGTCACATTGGATGATGCAAGAATGCTTTCTGATATT
5126	CaTSNP8114	TCTCTTCTTCCCTCTCCGGTTCCCACTCCGATCCCCTCTGTCACTGGCTCTACGACACTT[T/A]CCTCTCCTCCGACCCTCACCTCCGCCTCGTTGTTCTCTCCTTCATCCCTCTCCTCTCCGG

5127	CaTSNP8115	TGAGTGTTGGTCCGGTGGTGAAACCGGCACTGCTACCGCCACTATAATGATTCTCGGAGA[C/T]ACTTGTACTCGAGGTTGCAGATTTTGCAATGTTAAAACATCAAGGACTCCTCCACCGCCT
5128	CaTSNP8117	ACATAACATAGGCTCTTAAATCAGTTCCTGTAAGCGGGGCACCCATAACTTCAAGAATCT[G/C]AGCAGATGTGTTAAGTCTTGTCATGGCCATTCTATACACTTTATCAGGGTTGATTGTGAA
5129	CaTSNP8118	ACAGAACGTTTTAAAGCCAAAGGCTGCTAGTAAAATGCTTGTGGGTGAATTGAAGGAAGA[A/G]CTAGAAGCACAAGATTTACCTACTGATGGAACCAGAAATGTTCTTTACCAGCGTGTTCAA
5130	CaTSNP8120	TCAACAACCAACATTTTCTGGCATAGATTCTTCCTACAGTTTACCAAACAACACTGCACT[A/T]CTAACTCTGCAGGATTTTGGAAATGGAAAAGTACTCCTAAGGCTAGCTCATCTTTATGAG
5131	CaTSNP8121	ACACCAGCAGCTGCCAGAATCAATTGGCCATGACAAATGCAAGCAATTGCTTTTCCAGAA[C/T]TGACAAACTTGGTCACCAGCTCCACAACACTAGGAATGTGAGCAAGATACTCCGGCGCCC
5132	CaTSNP8122	TCCACAGTTTGGTCCTGGTTCAATTCAGCAGCATGGGTTTGCAAGGAATGTGGATTGGAC[G/T]GTTGGTGATTCTGAGAATGTGGAGGGAAATCCTGTAGTAACTCTGGAACTCAAGGATGAT
5133	CaTSNP8123	CTCTGTGGAACAGAACACTCTAACTATTAAAGGTAAAAGTGCAAAAGAAAGTGATGAGGA[C/G]GAAGAAAGTGTTCGTAGATTCTCTAGTAGAATTGATTTGCCTGAGAAGCTTTACAAGATT
5134	CaTSNP8124	CCAGATCACAGCTCTAGCTTCCCTCGGTTACCGCTGTATAGCACCTGACCTCCGCGGATT[T/C]GGCGACACCGATGTACCGGCATCGCCGACATCCTACACGAGTCTCCATGTCGTCGGTGAC
5135	CaTSNP8125	ATCGGCGACGACGCCGTTTTCTTTCAAGATCCCGGCGAGCATGTGACCGAACTCATCGTC[A/G]CCGAGTTTGCCTACAAACGCAGCTTTGCCACCGAGTCTGGAAACGGCGATGGCTACGTTA
5136	CaTSNP8126	AGTGGACAATTGGATCCTAACAAAGTTCTTCATGGATTTCAATGAACTATAATTGGTGCA[A/G]TCAAGTCAAAAGTCAATTGAGATTTATTTAAGTCTCATAATTGTGTGTGCACTTACATTG
5137	CaTSNP8127	TAGCGTTTCTGGTCCACCACAATTCATGCCTGCTGGTAGCACTCCTCGTCCAACTCAATC[G/A]ACGCCCGCTGGTACAGCCCCCCGTCTGCAACAGGGTTTTGCAGGTCCCAACCTCTCAAAT
5138	CaTSNP8128	TATTCACACCGGCGTTCAGAGAAGCGAGAATTTCCCCGGAATCTCTTCTCTCCGATCTCT[G/T]TACGACGCGATTCCAGCGAACGTGAAAGAGAATTTAGAAGCAGTTTATTTTATTCACCCT
5139	CaTSNP8129	CTCCAATGTTTGAGAAAAACAAGCATCCTATTGACCTCAAATCCTAATAGCTTAGTAGGA[T/G]TGGGAGAAGAAAGTTGTTTGTGTTGGCGATGTGGTGTTAAGAAATGCAAAGGTGTAGGTT
5140	CaTSNP8130	TCAAGCTGTGATATTCTGTAACACCAAGCGAAAGGTGGACTGGTTAACTGAAAAAATGCG[C/T]AACAATAATTTTACTGTCTCATCAATGCACGGTGACATGCCTCAAAGAGAAAGAGACGCA
5141	CaTSNP8131	AAGTGGACTATTCAAGCATGCAATCACAGTTGGTAAGAGGGTAAGAACAGAGACAAACAT[C/T]GCGGCCGGAGCAGTTTCTGTCAGCTCAGCAGCTGTTGAATTGGCCTTAATGAAGTTACCT
5142	CaTSNP8132	AATAAGTGCAAAAGTGTAGCCATCAGGATCCTTCACAAAGGCAGTAACAGTTGTTCCTCC[G/C]TCAACTGGACCAGCCTCTTTAGTGATGTTTCCACCTTTGTACCTAATGTGCTCAACCAAT
5143	CaTSNP8133	CATCCTTGTTTCTCTTCTTGACAGATTTCTTTGCAAGTTTTATTGCCTTGATCTTCTGAG[C/T]AGAATCCCTGAGACCTTCTCCTGGCACAACTTCATGTGGTGGGCGGGAGACAGACCTAGA
5144	CaTSNP8134	GTGTCTGAAGGCTCAGAAGCAGCCGTCTCCGCCGGGGTCTCCCGGTAACAACGACGTGAC[G/A]ACTGACGGTTCTGATCGGGCATGTTCTGATGCGTCCATTCTGTTGACTAAGTTGGCGTTG
5145	CaTSNP8135	TGCCGAGATACAATAATCAGGATGCCGAAGCAAAGCTAATGTTGTCTCTGTCAATACGTA[G/A]CTCAAACCTTCTGAAGATGATGTGTCTGCAACTTCGGCAATCCTATTCAGATCCCTTTGA
5146	CaTSNP8136	CCTTTGCCAGTTGTAATAGACAAGTGCATTCAGTCTGCACCAATTGACACAAGGAGATCA[C/T]TATATAAGAATATAGTGCTGTCAGGAGGATCAACAATGTTCAAGGATTTTCACAGGAGAT
5147	CaTSNP8137	GTTGTTTTTGGCTGTCAAAAGCAAGATAAATTAGGAATGAGGGATCATATAGCATAAACC[G/A]TTCCTAATATTACATAGGATTGTTGCCTTTGGTACAATTTTAACTAGAAGCTATCCAAAA
5148	CaTSNP8138	TGTTGCTTGGTTTATAGGGGACTTGCTATTTAGAGCAATATTATAGCAAACTCTGATAAC[T/C]GCCAGCAAAGGCTCGCCATGTACTCTGAATTTTGAGGAAGCCACGGCAGTAAGAAGTACC
5149	CaTSNP8139	CAACTAAGTACAGGCCAATTGATGAGATTGAATATTGGAAAATGGAAAGAGATCCTGTGA[G/A]TAGATTCAAAAGATGGGTAGAAAGGAATGGTTGGTGGAGTGATAAGGATGAATTGGAGCT
5150	CaTSNP8140	ATCAGGAACAACAATATCAGATTTCTTGTCTAACGCTTCAGTGTAACCTGTTTGGGGATT[G/A]TAAAGGGACCAAATCTTCTTTCGGAATTCTAAGTCAGCGAGGAAGATTCTAACCCAAACA
5151	CaTSNP8141	CCACAACCTTTCCGGTGAACCTTGAAATATGTCATGAACTCCCTGAAAGACAAATCTCTC[A/G]TCCATTCCTTGAACAGATAGAAGACCTTTCATCCTATAAATATCTTCACTACGTTCCATC
5152	CaTSNP8142	CATAGAGACACCTAAGAGAGCAAATTTTCTTTTGGAACTTTTATACTGTTTGCTCTAAGG[A/T]TCGTCACTAGCTTGACTCTGCAGGAGGTTTTTCCTTTGGATGTTTGGAAGCATAATGATC
5153	CaTSNP8143	TTCACATGCTGCCGATTTGTTCAAGAGTATCCGTCATGCTTATGAAGTACTATCCAATGA[G/A]ACGACGAGAGTTCAGTATGATCAGGAACTTCAATTCATTCACAAGCCTTACCGAGAAAAA
5154	CaTSNP8144	ATTCCTGCACCAGAATTATCTATGTTGCAGACTGATGAGCCAGATCCTGATGACCAAGAA[C/T]TTCAGAGGATAAATGGTGCCATGACTGCTATTTGTAACCGATTGCAGAAAGCCTTGGAGA
5155	CaTSNP8145	TTGAACTCCCTTTCTACCGTGCAAAATGTCATGAAGAGAGCCCATAGTTGCAAACTCATA[C/T]GCAAGTACACGAAGATTTCCTTCAACACAGTATCCATGCAGCTCAACAAAATTTTCGTTC
5156	CaTSNP8146	TTAATAATGAAAGACCTGCTGAGAGGAATTCCACTTACAAGGAAAGATTTAGTAGCTTGC[T/A]GAATTTGGTACTTATCATGTTTGAGCAAGACACGGTTTTAATACCGAAAGAAACATCCTG

5157	CaTSNP8147	AGGAAATCAGGATAAACCAGCATGCTTATCTTCCTTACAGCACATATATCAACCTTTTGA[A/G]TGACAACCTCATCTTGAGAGAGGCTTTTGGAGCAATGCAGTCAAGTAAAGTTCCCCATTC
5158	CaTSNP8148	GTTGGTCCACCACTTCCTTGTGTCTACATAAAGCTTGTTTCTTGGGAAGAAGGAGGGTAT[C/T]TGTCGTCAGACAAACCAATGCCACGAGGAGAGATTGTAGTTGGCGGATTCAGTGTGACTG
5159	CaTSNP8149	TGGAGATAATCTTCCATCTTTGCCTGAGGGGAAAGTGCCGTCTCTCTTGATGCCTCCTTC[G/A]CCCGTCATGTGTGGAAGTGATTGGCCCCTTCTCAGGGTTACGCGAGGCATGTTTGATGGT
5160	CaTSNP8150	TCTATTTCAATATCTTGAACTTTAGGCTTCTTTGCGTTTGCATTACCGACGACCTTTACT[G/A]TTGCTGTATATCCACAGTGGTTAACGACAACAACGGTCAAGGAAGAAGTGTCATTAACGG
5161	CaTSNP8151	GTTGGCATCTTAACCCTTCTCGTTACCGGATCCATTCTCCTCCTCTTCACCGGTTTAACC[G/C]TATCTGCCACCGTTATCTCTATCATCTTCTTCTCGCCGTTGATCATCGTTTCCAGCCCAA
5162	CaTSNP8152	AGTAGAAACTGACAGTGCCAATGCTGAGAATGTGATGATGGGTGTTTCTGATGAGGAGTT[A/G]TACAACTTACCAACGGCAGATTACATTAATAGGCGGGCAGGATTCTTGAACAGGCCATTT
5163	CaTSNP8153	CTCCTTAACCTTTTGTTTGTTAGCATCGTTGAATTCCCAAGGAAGGTTGGGGTTGTTGTC[T/G]GGAGAGTCAAGGTGATAGTTCAGAGCGGTTGAGAAAGCCCTCGATGGCTGACGGAAAATC
5164	CaTSNP8154	CAACAGATACTGTGAGGAAGAGTTGCCATAGATTTTGGTATTTTTATAATCAAACTCAGA[C/G]AGACTATGATTACAAGAAATTGATTTGAACTGCATATATTCTTTTGGCCCTGTGTATAAA
5165	CaTSNP8155	GAGTCTAGAGACGGCGATCGCGACATTAGCGGGAGCGCCGCCGGGAGCTTTGAGGAAGCC[A/G]GGAGCTTCGGCGAGGGAGACGCCGGATACGGTGGGGACGAAGTCGATTAGCATCTCGCCG
5166	CaTSNP8156	TGCTTGTGGCTGGACGCCGCTCCACATTGCAGCTAAAGAAAGGAGGAGGGATGCCGTGAA[A/G]TTCTTGGTAGAGAATGGAGCATTCTTGCCACCTGATATCAATGATAGCAGATTTAATCCT
5167	CaTSNP8157	TGAATTTACTTTCGATCAAAGGCTTGAAGAATCGCGAGATATTGTTGCCAAATACCCCGA[C/T]CGAATCCCCGTGATTGTGGAAAGGTATTCGAAATGCGATCTACCTGATTTGGAGAAGAAA
5168	CaTSNP8158	CATGGAATCTTCTGACACTCCTGCCTTTCCATTCTTATCATATGCTTCACGCTTCTCAGG[G/A]TCGCTTAAGACCTGGTAGGCCTCGCCAAGCATCTGAAAATTTTGTGCAGCCTTAGGATCC
5169	CaTSNP8159	ACTGGTATCTGAATTTTTATTGCATTGATGCTTTTCTTGATCGTCATTATTGCACACTGA[T/C]GTCGAAGGCATGTTCTCTGGAGGATCAGAAATTGGAAGGGGTGAATCAATAGGACTGGTA
5170	CaTSNP8160	ATTGGTTAATAAGAAGAGAAAGAAAGGTGTTAAAGGGAAGAAAGGAGGGGATTCTAGTGA[A/G]TTGGGAAGTGTGAAATCATTGAAGGATTCTGCTAAGCGGAAGAAGAAATTATCAGTTGAG
5171	CaTSNP8161	ATAACCAGCAGCAATACCAAGTATCAAGTAGAAAAACAGCATACCTGTGATGAGTGTTCC[G/A]CGAGAGGCCGGTGACATGAATCCGAGTGCAGCAAACAATATTGTTACAACAGCCATTCCT
5172	CaTSNP8162	ACGCCTGCGTAGGCAGCTGAACACAGTAGCATGGATGGCTGCTACACTCTTGTCCACATT[A/T]GTATTGTTTATTTTTGGAACTAAATGTTTGTCAGCCCGCTTACAGAGGTAATCCTGGATT
5173	CaTSNP8163	GTCTTCTTTCAGTGAATCTTCATTAGTCAAATCCTTTGATTGGATAGGCAATTGAATAGG[C/T]TCTTGATCATCACCCAAAATTCGTGCAGTGAATTTTCTCGCCTCGAAATCAAGGTCTGGC
5174	CaTSNP8165	TCATCCCTCCGACGTTAAATCAATCCTCCTCGACAAAAACGGCGTCGTTTCATCTCTAAA[C/T]CCTAACACCGTAACCGTCGATACAACGAGTTCACATCCAGATCTCGCTAAAGAAATCGCC
5175	CaTSNP8166	TACTGAGGGTTGGCACTACTAAAGGTGATTTTTTGACACCCTCTTTTAAAATCGCATCCT[G/C]ATTGAACAAATTAGGCCTCTCTGCAGCTGGTGCAGCTAAACAATTCTGATTACCAGGGAT
5176	CaTSNP8167	AGAGGTTATTGGATTCTACAAAGAAAATACTAGCATAATAGTGGAGACATTTGATTCTCT[T/C]GGGTTTAAAGTCTACGGAGGGAAATGCGCGCCGTACGTCTGGGTCCACTTCCCTGGCCAA
5177	CaTSNP8168	AACTGAGCCCAATCGTTGTGAGCGTGCATTTGTTGGCAACACAATTGGTCAAGCAAATGG[T/C]GTGTATGACAAACCATTAGATCTCCGGAAATGTGACTTCTCAAATGATAAATCCAACCTG
5178	CaTSNP8169	CCTACACTCAAAAGAATTCTTCTTCAGATTTGCAATAGCTATCAACCCACAGTGTTCGCA[T/C]ACATGGACCCTATATGCATCACTTTGATCAAACAATCTTTCCTTTAGGAAATGAGCAGCT
5179	CaTSNP8171	CGATGCAGAAACAGAGTTGAAGAAGAACATGAAGAGCAAAAATGGGTTAGCGTTGAAAGT[A/G]AAGTTGGATACGAAAGTGAAGGCTAAAATGGGAAAATTGAAGACACCAAATGTTGGAATC
5180	CaTSNP8172	GCAAATGCTATGAGGCGTCTCAAGATTGGTCCCATAAATATTGTAAAATTTAACACCCGG[A/C]GGAAGTTTGGCAGAAGAAAGAATCTCCCATGTTTTGTTTGCCCATTTCAAAATTTCTGAG
5181	CaTSNP8173	ATGTAGCATTAGCAGTGCATTCCTTTGTTTCTGAGGACTTCACTTTAAGAACAAATCTTC[C/T]AAGTTTCTCAACAGAAATGCCTTGAGGAGCAGCAAGAGAGGGAATTTTACCACAAGTTGA
5182	CaTSNP8174	AAGGACTATTATATTGTTGCATCAACAAGATTTTCAAAAAGGGTTCTCACAACAACTGCA[C/T]TGTTACACTATTCAAATTCTCACTCCCCTGCTTCTGGTCCTTTGCCTAATCCTCCTTCTT
5183	CaTSNP8175	ACCAATCTTGAATCATTCTACGAAGGGAGTGATTAGGTACGATGTTGTCGAAGTTAGTTG[G/T]TAATAATAACTCTGTTTTTGTGACGGGACACGTGTTGTTACCTGATTCGATCCATTTTTC
5184	CaTSNP8176	GACTTTCGGAAACTCGGTGGCTGAGTCACCGAGTTTGATCAACGATTTATAACTCGTGAC[A/G]TTTGAATTTTGAAGAATGTCGGTGGAATCGAGAAATACGGCGGGGAGATTGAGGTGTTTG
5185	CaTSNP8177	GCTTGATTGGGGTGAAGAAGGCCAAAGGACAGAAGCTGAGGTTTCTTCACTGAGGGCAGC[C/G]TTCATACAGGAAGTTGCTGTTTGGCATAAACTTGATCATCCCAATGTCACTAAGTTTATA
5186	CaTSNP8178	ATGAACAACCAAGTTTCCATCGTTAACTGAGATTTTAGGTGTCACCGTCATCTTTCACCA[C/G]TTGTTGATCACGTATAAGGAGTGTAATTAATTAATTGAACAAAGAGGAGAAGTGCATTCC

5187	CaTSNP8179	AAACGTTGACGCCGTTTATGTTCCGCTTCCAACTAGCTTGCACGTGCGGTGGGCGGTTAT[G/A]GCTGCTCGGAAAGGTAAACACGTGCTGTTGGAGAAGCCTGTTGCTCTTAATGCTGTTGAG
5188	CaTSNP8180	ACTCGTGACATCAAGAGCAGAAATACCTGAACTACTTAAGCTGGACGATGTAATTGATCT[A/G]GTGATTCCAAGAGGCAGCAACAAACTTGTTTCTCATATCAAGAGTTCGACGAAAATCCCT
5189	CaTSNP8181	GAAGACATGCTACGTGCAATTTCCTCAGAGATTTGATGGCATTGATTTGCACGATCGATA[C/T]GCCAATCGCAATATTGTTTTCTTCGATATCAATATGAAAGGTCACGATGGTATTCAGGGC
5190	CaTSNP8182	ACAATTAATTAGAAAATCTTATGAGGACATTTATCATGAGGATCTTGTCAAACGCTTGGA[G/A]TCTGAGATCAAAGGAGATTTTGAGAAAGCTGTGTACCGTTGGATATTGGAGCCTGCTGAT
5191	CaTSNP8183	GCTGGTTTCTGGTGGTAGTGATAATCACCTGGTTCTTGTTGATCTGAGGCCATCTGGTAT[C/T]GATGGTGCTCGGGTGGAGAAAATTCTTGACATGGCTTTTATAACCCTCAACAAAAATTCA
5192	CaTSNP8184	TCGGAGGACATCTGCACACGACGGTCCAGAAACAGCCCGCGTCACCGCAAAACCGTGTGG[A/G]CCGTCGCTCTGTAACACCCTAACAACACTAGGGCCACCAAACAGGTTGTGGAACCCACCA
5193	CaTSNP8185	TTAATTGAAGGCAGGCAGCATGAGATCATCCAGTTGTTGCCTCGGTATTAACCGTACAAC[C/T]ACAAAACCTTCTCTCGTCCCGACAAAGCCACCTCAAGTATCCTTGGTGGAGAAAAACAAA
5194	CaTSNP8186	AGAGAAAAATTTAGTTCCATTTGGTCCCTCGGGTAGCTCAGCAGATTTAACATCAAGTGG[G/A]ACATAAGTCTTCTTCTTCCTCTTCCCAGTAGTTGCGGCAGCTTTGACTACTGCTAGATGT
5195	CaTSNP8187	TTCTTTGTCTTCTTCTGAGCGAAAATTGTAAAACTCAAATTTTTGTTGGAAAATTGAGAA[C/G]AAGTCTTCAAGTTAGGGGTGAAAGTGCGTTGCAATGGAAGAAGAGAAGAGGTTGAGGAGA
5196	CaTSNP8188	GGATGAGGTTGGTGAAAGCGACGAGGAACGGGACAAGATGCTTCTTCAGTTAGAGCAGGA[A/G]TGCTTGGATGTGTACAAGAGAAAGGTTGAGCAGGCTGCAAAGTCGAGGGCACAACTACTT
5197	CaTSNP8189	AAAACATCCATCATGATATATGAAGTTAAAGGGCAAAACCAAGTTTAGTTACTTCTTTTT[C/G]GGAACCTCAAGCCTAAGACGGAAATCCTCTTCCATAAGAGGAAGACCGTCCCGCAACTTG
5198	CaTSNP8190	CCACAACAAAACCACAATACACCAAAGGGAATTCTCACATCATGCCCTGAAATCAGCTGC[G/A]TCAAACTCCAGTCAATCGAGACCCGGTAAAACACCACTTAAGTGACATCATCAGCAAGGT
5199	CaTSNP8191	CTTTTCGATTTCTTGGTACTGGTTTGCCCAAAAGGCTTGGAGCTGTTGCTGAAGCTGCTG[C/T]TGTTGTTGCTGATGAATGTGCTGATAAGCAAGTTGATGCTGTCCCAGCTGAGCTCCAGCA
5200	CaTSNP8192	TATAGATATCAATGTTCAACCTATTGAGGATGATGCTTTTCATCAACCCCAAAATCATAA[C/T]GCTCCCCCTAAACCTGTGAATCAAATGGTTGATAAGGCACCATGGCAGGAACTGGGAAAC
5201	CaTSNP8193	AATCAAAAATGGAAAGAAGATTGCAGCTTTTGTTCCAAATGATGGTTGCTTGAACTACAT[C/T]GAAGAAAATGATGAAGTGCTTATTGCTGGTTTTGGCCGAAAAGGGCATGCCGTCGGAGAT
5202	CaTSNP8194	TTTCTGTCCTGGCTTTTAATTTATGTAATTCCCAGATTTGAAAAATCAAATCTTCTATAT[A/G]ATTTATTTGTCAAAATGTTTCAGCAGTTTCTTTATAATTTACCTTATACTTTAGGCTTTT
5203	CaTSNP8196	AAAAGCTCGCGCACTTTTATCATCCTCTCCCTAAGTTTTTCCAATTTGTCAGCAGCATCT[G/T]GACGATTTTGTTCTTGCCCAAACACGGATTTAATTGCAGAGGTAGCAGAAGCTATCTTTT
5204	CaTSNP8197	TGGTTTTGTCAAAGGTTATGCGCTCATTGAATATGAGCGTTCTGAGGAAGCTCGGAATGC[A/T]ATAGAGAATTTGAATGGATCTGAGCTTCTTACACAAACTATTTATGTTGATTGGGCTTTC
5205	CaTSNP8198	CATCCTTGTTTCTCTTCTTGACAGATTTCTTTGCAAGTTTTATTGCCTTGATCTTCTGAG[C/T]AGAATCCCTGAGACCTTCTCCTGGCACAACTTCATGTGGTGGGCGGGAGACAGACCTAGA
5206	CaTSNP8199	TAATATAATTTTCATCTTCGACTTTCCTGAAGCACTTGAACTGACTAGATTGCTCTCCAC[C/T]TCCTCTGCCTCCTCCTGAGTAAGCAATGGAACTTGTTCAGGTACAACATCACTCTTTGTA
5207	CaTSNP8200	ATGTTCAGATTCTTCTGGTTGCAAATCTGCTTCTGAGACTAATGCTCAGTATTACCAGCA[G/A]GAAGCTGCCAAACTGCGGGTGCAAATCAGTAATTTGCAGAATCACAACAGGCAAATGATG
5208	CaTSNP8201	CATGGCTTTCTTTACTTGAGCGGCAGCATATGCATACACCTCTAGCTCAGAGCTAGTAGC[G/A]GCACCATGCATATAACGAGGATGCATGAACAACTGAGCTGTTCCCCACAAAACGCTCTTC
5209	CaTSNP8202	TCGGGAACCCCGAGGAACCCCTTTAGCAAATTCCACGATAAGACGACTTCCATCGACGTC[G/A]CGACCATCCAAATTGTATCTTGCATCATCAGCATCACGGGGATCGCTAAATTCAACAAAG
5210	CaTSNP8203	TTGCTCAAACCATGAAAGCTTTCAGAAATCATGGTGTGAAGGTTTCAGGTCAAAACTCAT[C/T]GGAGTTCGGTTCTCCGGGAGGTTTTGAACAAATCAAGAAGAATATATCAGGTGACAATGT
5211	CaTSNP8204	CTCCTCACGTGCAACGGCTTCATGTAGCAATGGATTAAAAGGCTTGCCCACTGTTGCTAC[A/C]ACAGAAACATGATGGCTCCTCAAAGTCTCTACAAATTGCTTGTAAATACTTTGATAGCTC
5212	CaTSNP8205	TCGTTATGGGTCTTCTGCATACACATCTAGAACAGGCTCTGTTGATAACAAGTTTAGAGC[T/C]GCAGGTAATGGTTATGTTAACGATTCCAGGAAAAATGTCGATGGTTTCAGTGAGCTAAAT
5213	CaTSNP8206	ACTTCTTCAGATGTTGATTTTGCTGAATTTCCATTCACAGCCTTCTTTTCCACCTCAACC[C/T]TACCAGACCTCCCACCAGCAGGTGCAAGTTTTCCTTTACTTACTCTTGATGTCAGTGGCT
5214	CaTSNP8207	CGCTCTCATCTCCAACCTCTTCCACAAAGCCCAGCCTATGGATATGTGGGTTTATCTTTC[T/C]GAAAATGAGAAATTCAACGACTTTGGTAGTGAAAATTCTCTTGTATGGCACGAAACCAAT
5215	CaTSNP8208	ATCTTCTGGTCCTGCCAATAGCACAGGAACAGCTAATACAGCGGGCCCATCACCGAGTTC[G/A]GCACCCTCAACTCCCTCAACTCATACGCCTGGTGATGTGATATCAATGCCTTCTATACCT
5216	CaTSNP8209	AACACATCTTGGTATAGTTATGGAGTATGCTGCTGGTGGAGAGTTATTCGACCGAATTTG[T/C]TCTGCTGGAAGATTCAGTGAAGATGAGGCTCGGTATTTCTTCCAGCAGCTCATCTCAGGA

5217	CaTSNP8210	GGCGGATGACACCGCCGGCAGGAGAATTCCGTTTGCGTTTCTGGAGGAAATCCATCAGAG[G/A]TTTGTTAGGACTTATGGACGCGCTGTTCTTTCTGCTCAGGCTTATGGAATGAACGACGAG
5218	CaTSNP8211	TAATGTTAGAAGCATATGCCTCTTCTGTAACTTCTTGTTCCTTGTCGTTTTCTACATCTG[G/A]GCCATCATCAGAAACAGAAGCCAGAGGCTGAACTTCCTCCACTGGCAGTGCCTTGCTTAA
5219	CaTSNP8212	ATCTTTCTGTTTAATGTGTTATCTGGACTAAATAAATAAGAAATCAAACTATCATCTGGA[C/G]TAAAACTTATTGAAGTTGGTGATACATATCCAGGCAACGGGTATTGCGCAATCTCTTCAA
5220	CaTSNP8213	AATATCCAAAATCCTGCCATATTGAGAGAATAAGCAGTACAGAGATCTCTTCAACTCTTC[T/C]TTCTTAACCTTCTCATTGAGATTCTTAACGTAAATGGTTTGATTTGGAGGTATGTCCCCT
5221	CaTSNP8214	TTGCTATTGATCCCAGCGTTCAAAACGAGAATGAACTTGAAGGAAGAGAAACACAGATTC[A/G]CAAATCTTTTGATGCACAAGATCAAAGTGGAGGTGGCGGCTTCATTAGTGTGGAGGGGTT
5222	CaTSNP8215	CAGCATGCCATTGCAAATTATCCCTGCTAAACAACATGAACCAGAAGATGCTATCTGTGG[T/C]TTGTTCAGCCACTGCTTCTTTACCACAGACAGAAAATCATCTTCTGTTGCAGAAAAAGCC
5223	CaTSNP8216	TGGTTCAGAGTACTCAAAAATTGTGTTAACATCTTGAATTTTCTTCATGTTGGTGCCACT[G/A]GCTGCAAGATCAAGTACTGTGACAAGGTGACCAATTGATTCTAGGAGTGGCTTCACCTTG
5224	CaTSNP8217	GCTCGGCCGTGGTGGATTTGGGGTTGTTTATAAGGGAGAGTTGGATGACGGGACTAAGAT[T/C]GCTGTAAAAAGAATGGAAGCAGGTGTGATTAGCACTAAAGCCTTAGACGAATTCCAGGCC
5225	CaTSNP8218	GGAAGGATTTCTGTGCAAGGGATTGATGGGGTTTTGTTTCCACATCAAGAAGGTGAGGTT[C/G]TTCCTGATAACACTTTGACCCGTACCCGGATGGGTCAACCCGCTAAGGTTCTTGCTAAGC
5226	CaTSNP8219	AATTGTTGTTTGGAGTGGGAATGATGATCAATGCCACGATGCTAGATGGGGTTATGGGGC[A/T]ACTGGAGTGATATCTGTTGCAAGCAACCTGATTCCTGGTTTAATGCGAGATCTCATGTTC
5227	CaTSNP8220	CAGGACCTCACCACGGTCTTCATAAATGTAGTCCAATCCTTTGTAATAGGGTGGGTGCCC[A/G]ACAGAAAGATAATTGATGCTATTTGAGATATTTCTCCTTGTCAACCCTCACAATCTGACC
5228	CaTSNP8221	AAAAGGAACAGTCCGTCGAAGTACTGATGGCCATATTATTCATGCCAACATTGACACGGA[C/T]GTAATTATTGCTGAAGAACCTCCTTATGGTTCAACACAAAAGCCTGAAGACATGTATAGG
5229	CaTSNP8222	AGGAATGTTGATGTTGGCATCAAGGAACTTCTCAGACAAAAGACCGCCCATCACTGTCCC[A/G]TACGTTATAAGTTTGACTCCTGTAAGCTGACAAAGCTCTGCCATTCTCTGTTGTGGACGC
5230	CaTSNP8223	TCGTGTGGTCATAGTGGAGGAGTGCATGCGAACAGGTGGAATTGGTGCTAGTCTGACAGC[C/T]GCCATTACTGAGAATTTCCACGACTATTTGGATGCTCCTGTCGTATGTTTATCCTCTCAG
5231	CaTSNP8224	CACATCTGTCGCACGAATTGGGTTGAAATCAATCAGTTCTGCGAACCCAGTCTTAGCCAT[T/G]ACAGAGATTGCCTGATCCATCCCACCAGATTGCGTCCCAATATGTCGTTCACAATCACAT
5232	CaTSNP8226	TTTCCCTCGTCTTAAAATCATTCACACTTCCCGGAAATTTATGGGCCAAATCTATATCCC[C/T]GTCCTTAACTGGTTTCTGCTTGCTGTTTCTCTGGTGCTTGTCTGCTCAATATCCAGCATT
5233	CaTSNP8227	TCAGAGTATCCAAATCTTCCAGTATGTTGCTCTGTTTGTTTGTTACAAGAAGCAGATACA[A/G]AGCTTCTATTGGCTGGTAAACATAGCGCACGTTCTCAGTCTCAACATATGTGTGTTGTTT
5234	CaTSNP8228	ATGATCTTTGCAATTACATCCGCGCGCATTTTATCAGCATGTTTAGAGACATCATCAGCA[C/T]CAATCATTATCATGTCTGCACCTGCTTCTAAACACCTCTCCGCTCTTCTAATCAAGAGAT
5235	CaTSNP8229	GAACTGGCAGAGCAACAACTATCTCAATGGTCAAAGCTTGTCTTTCAAGGTTACAACCAG[C/T]GATGGCCGTACTATTGTTGCCAACAATGTTGCTCCAGCTGGATGGTCTTTTGGTCAAACT
5236	CaTSNP8230	CTTTCCTTCACCTAAACGGATGTGGATCGGAGCTAGGTTAATCTCTGGTGCATCTGGAAT[T/C]ATCTTACCCATAATTAAGGCTGAAGGAATTCGCCAAATGTTTTTCCCTGCCACTGTTCCA
5237	CaTSNP8231	TGGTATGTGGACACGCTGAGCTCCTGATATAATCTCCTCACCGCGAATAAATACATCAAA[G/C]GAGTTACTGTATTCTGGATTGTCATAGCAAGGCATTGTATAAAATGGTCTTATAGCCAAA
5238	CaTSNP8232	AATCAAGACTGTTGATGCTATGCGTGCTGCTTATGGAGATGATTACTATATTTGCAGATT[C/T]CAGGAACCTGGCAAGATGGAAGCTCAAATGGCTGAGGTTGGCACTGCATATGTGTTGAAA
5239	CaTSNP8233	AATAATGGTGTTGAGGCGATGAGCAATAATGAGCATTGTACAGGATTTAAATTCCTCTCT[G/T]ATTGTTTTCTGTATGAGTGCATCTGTTCTGACATCAACAGCTGCAGTTGCCTCGTCAAGT
5240	CaTSNP8234	GGACGAGAAGACTGACGTATTCGCGTTCGGGGTTTTGCTATTGGAGCTCATAACCAGTCG[C/T]CGTGCGGTTGATTCGGATAGTAGGCAAAGTCTTGTGATCTGGGCCAAGCCACTGCTTGAC
5241	CaTSNP8235	AACAAACCATGGCTCTCGGTAATTCGTCCGATTCGTTACATGCTTCTTGAACAGCGAATC[G/A]TTTTCGTATTAATCGGAATCGTCATAGCAACGCTATTCTTCACACTCGTACCTAATTCAT
5242	CaTSNP8236	AGTTTTTTTATCAAGCACCACTGTCCTTGGAATACCCATAACAGATGCCCAACCTTTAGT[G/T]ACATCAGCATATTCACTATCAGACTCTCCAATCCAACTCCACAACACTCTTCTCTCTTTA
5243	CaTSNP8237	AGAAGAACGAAAGTCATGGCGAAAGAATCATCCTCATGGTTTCGTTGCCAAGCCTGAGAC[T/A]CTTCCTGATGGCACTGTTAATTTGATGGTTTGGCATTGCACTATTCCTGGCAAGGCTGGG
5244	CaTSNP8238	TCTTATAAGCTCCTTTTCTTCTTTATCTTCTTCTTCCGTTGTTCACCATGCCACGTCTTC[G/C]TCGTCTTCTTCTGCTGACGTGGAACCCGTTCACATAACCGAAAACTGCGTTCGAAGAATG
5245	CaTSNP8239	GTATTCCAGAATATATTCTTGAGAAATTACTTTGCCAGGCAATTTCTTGGGTTTCCTCAA[G/C]GGTACAAACTTAGCACCTATTGCCAAGGCAATGGGAGGACCAAAAATGAAACCTCGAGCT
5246	CaTSNP8241	CAAAGAGGATTACGAGGATGTTTGGAGAGAGATTCAGATAATGCATCATTTATCTGAACA[C/T]CCACACGTTGTTAGAATCGAAGGAACTTATGAGGATTCAACTGCTGTTCATCTTGTTATG

5247	CaTSNP8242	TCACGACAGTCTTGTCATTTCGCAACTTCAAATTGTTAAGGCACTGAATCAAGCAAGATT[G/A]GAGGCGAATATTAGAGTGTATGGTGACGAAAAGCACGAAAAAAGATGGCCAAGTCCTTAC
5248	CaTSNP8243	AGCTTCGTGTGCTGAAATGGCCATATATCTCATGTTGGGTCTTCTTCGAAAGCAAAATGA[G/A]TTGCAGATTTCCATACAACAAAGGAAGCTTGGAGATCCTATTACTGATACTTTATTTGGG
5249	CaTSNP8244	AAAGTTACACCGTGCCATGAACAGTTCTCCGAGAATCTCTAAGAACTCTCCAAAATCAGA[T/C]TCAAAAGGTAGTAGACCGAAGAGGCCTAGAAGCTCTTCGAGTTTTGAAATAACCGAGGGC
5250	CaTSNP8245	TGATTCATCTAATCCAGCTACCAGAAGACCAACCCCATAAGGACGCTTCCATGATCGCTG[G/A]GTGCAAACCTGAGCCTTATCGGCGAGCTGAACAACGAGACGACCAACAGGAAGCGGCGAT
5251	CaTSNP8246	GGATGAGTTTGTTTGGGGAGGCGCATGGATGTACTTTGCAACTGGAAATAACTCGTACCT[T/A]AAGCTTGCTACTACTCCCGGCCTTGCTAAGCATGCTGGTGCTTTCTGGGGTGGCCCTGAT
5252	CaTSNP8247	GGAACCTCCCTTCTTGCTCTTTTTTCCAAACCCTAGAAACCTAACTAGAAAACCACCGCC[G/A]CCACCACCATTTTTGGCTTTACCATTCTTTTTCTTATTATCACCCCCCAAATCATTACCA
5253	CaTSNP8248	CGTGTTGAGTACCTTTTCAATGGATCCGGGTCGGGCCAACATAGAATGAAGCTCGAAAAC[A/G]GAGTTAACAGCAACGGCTTCACCGGGTCGGATCTCAAGCATATTCGGGTCAAGATCCGCT
5254	CaTSNP8249	GCTGCAAGGGAGGACATTTTGATCTGTCGTTCTTCAAAATTTGATTACTCTTTATTTTCA[C/A]AGTACGCGTGAATTAGAAGGTTGATCGAAGAAGATCGCCCCTGATTTGGAAAGCTTTATA
5255	CaTSNP8250	TGGCTTCCAAAAGGAAAATAAAAAAACTGTGCAGCTCTGGTTACAAAGTCATAGTTGCTT[T/A]GTTGTGTTTCTATGCATGAAATTGGAGTTAAAGATAATTCTAGATGGAATCCCTTTGGAA
5256	CaTSNP8251	GGTTGCAAGGAGAAGATCTCAACGAGTTACGGCAAGATCGAACGGTTTTAAACTGATCAA[C/G]ATTAAGCTTCTTATTGATGATACCAACTTGGAAGTAGATATTATTAGAAGGAGGCAAATC
5257	CaTSNP8252	TCTTTCCATTCATCCACACAACATCACAGATAAAACTATTACTAATAGATTCACTCCACA[C/T]CCTCTTTTCTCTTACAATACAACTACGCATCAATGTCTTAAATTCCCAACTTCTCACTTA
5258	CaTSNP8253	ACAAGGGACTTTTGCTTCAAAGTAGGTTGTCCTTGATAAAGAGAAACAGGTGGCAACAGT[G/C]GACAGAGAAGTTGATGATGGTATTGAGACTGTGTGTTTGAACTTACCAGCAGTAATAACC
5259	CaTSNP8254	CACGCGCCAAGCTATTACCCATAGCTCACGAAGTCACCCACCTCTTCTGGCTCACCTTCA[A/C]TCATGACAGAGACGAAGAAGTCAACGTCACTGTCAACAAATCCATGCTTCAAAATGTTCT
5260	CaTSNP8255	GGCTAGAACAATCTCAGCAGCACCTTTCCAATGTATATGAACATCAGAGTCAGCCGTCTG[T/A]ATTGCAACTCCGCCTCTTTTTTtCTCGGAATTGAATGGAAAGACATGAATAATTGATGAT
5261	CaTSNP8256	CTTCATATGAAAAGTCAGAAGATTTGAAGCAAGATGTGAAGTTATTGCTTGCTCCTGTTA[A/G]ACTTGGAACTCAAGCATTTTCATGGGCTGCTGGAAAATTGGAATCAAATGGCATAGGATT
5262	CaTSNP8257	CGGTGCTGAAGTCCGTTGGTGTTCCTGCAACATCTTCTCCACACAGGATCACGCTGCCGC[T/C]GCCATTGCACGCGACAGTGCTGCTGTATTCGCTTGGAAAGGAGAGACTCTCCAGGAATAC
5263	CaTSNP8258	TCCAGTGGAGCTTGCTGCTGTAGGAGTTTCTATAGCTCTCTTCAATCAAGCATCAAGGAT[C/T]GCAATATTCCCGCTTGTCAGTATCACAACCTCTTTTGTGGCCGAGGAAGATACCCTCAGC
5264	CaTSNP8259	ACCTGTGTTGCCTGGAGGAACAACGACATCAATTGGAGCAACCAAACCAACACGAGCAGG[A/G]GCGCCCACCTTGTACTTAGCAACCTCTTCGCTAACTTCCTTCAAGTCACCTTTGGTGAAA
5265	CaTSNP8260	TTCTGGAATGTTGTCGAACCAAGGTTTTGGTGACTTTGATAGACTTCAGCATAGAAGTCC[G/T]AGTCCCATGGCTTCTTCAAACCTCATGTCAAATGTCTCCGGGACAGGAATGGCCGCATGG
5266	CaTSNP8262	CGACTCCGATTACGAAGAAGAAGAAGAAGATGAACAAGAGCAAGAGCAAGACCAATTTCA[T/C]GACGCTGAAACTTCCACCCAATCCCATTCCACACACCTCGACGAAGTAGACGCTAGACTC
5267	CaTSNP8263	TATGTGGAATCAACCATACGGAAAAACCGATTTGGAGAGCGGGTCAAGGACAAGGCCTCT[G/C]TACCCTATGATGCTTGAAAGCCCCGAACTACGGTGGTCCTTCATCAGAAAAGTCTACGTC
5268	CaTSNP8264	CCGTGACAAAAGGCATAAAAGGATGAAGCAATCGCAAGCCATTGTCCAGACAAAACCACA[A/G]AGTATCTTGAGCAAACTGTCTTTCGGAAGCATACTTGGGATCATTGCCAGCAAAGTACGG
5269	CaTSNP8265	ACGGCCGCGTGAAGAAGGCAACAAGAACAAGGTTTTGGAAAGACTTGATTCCGATGCGCA[G/A]AAGTTAACAAACCTTCAAATAACCATACAAGATTTGATGAATAAAGTGGAAACTACAGAG
5270	CaTSNP8266	TGGTCTATTAGGAAAATATCCAGCATAATTGTATTGACCAAAGTTCACTGCTGCATGATG[G/A]CCTGATGTTATCCAAACAATTGTTGTCAAAATTTCAATAAGGTCTTCATTTGTTTTCAAA
5271	CaTSNP8267	ATCGTATGTAAATGTAAACAATATACTAAACAGAGTAATATATCACCGCACCAAGGAGCA[T/G]AGAAGAACAGAGGACTTAACGGCGTTGACTGAACTGAACAACGTTGATCAATAATCAAAC
5272	CaTSNP8268	AAGGGAGAACCGGAATTGTGATGGAATTTCCGGTTGGTGATGAGGAATCACCGTCATCGC[C/T]GGTGAGGTTGCCGAAGAGACTCCGGCGACGGCTTCTTGATACGGAGTGTAAGTCACCGAG
5273	CaTSNP8269	AGATTCAACAGCCAAGTAACGTTGAGTTGTGAGGTTAACAGAAGTTACAATCTGATGATT[C/A]TCGTTCCTTAATTGACCAACCTGAACAGCAAGATCATCCAAGTGTTTCTGTTTTCTCATT
5274	CaTSNP8270	AAGAGTGGCTTCACAAGACATGAAATTAGGAGATTTGATGATCCCAAGAGGAACATGTCT[C/A]ACAATCCCAGTTATAATGATTCAAAGGAATGAAAAATACTGGGGTGAAGATGCAAATGAG
5275	CaTSNP8271	TCGGCAATCAATTGCTGATGACTATGAATACGTCATGTATGGCAAGCTCTACAGAGTAAC[C/A]GAGGGTTCTGGTCGTGAAAAAGCGGAGTTATATATATCATTTGGTGGGCTTCTGATGTTG
5276	CaTSNP8272	CAGTGCGTATTTGAAAGATTATGCTGATTATAGAAGATCGCTTTACGGTCAAATTACTCA[T/C]AAAGCTCTTCTTGTTGATGCTGTTGGTACTCTTGTTATTCCTTCTCAACCCATGGCTCAG

5277	CaTSNP8273	ACAATTTCCAACCACCACCGGAGTTTTCTTTATCTTCTTTGAAATATCTAACAAGTCAAC[A/T]ATTACTTGAGGAGATGTCCGCTTGGTACGTACAATTTCCAGAAGTGGCATAACATGTGCA
5278	CaTSNP8274	GAGGTCTTATGACCCTAATCTCATCCAAGACCCCACTATTTACAGGTTGAATGAAGCTGT[T/G]ATGCATTTTGGGGAGAGCATCAAAGATATCATCAATGAAGAGTTTGGCGATGGGATCATG
5279	CaTSNP8275	CTTCCAAAAGTAGTGTCAGATATAGACATTTCCTTGCATCTGAGGTCATTCTCCGAAATA[A/G]TTGTTGCCCATTTTATTCTTCCTTACCGAGAGTCAAGAAAGCAAGTAGGAAGCTAATTTG
5280	CaTSNP8276	TGCAAGCTTTGGGTTTTTCTTTGCAACTTCAACATTTTTTGACCACCCAACTTCCTCTCC[T/G]ATGCTATTCTCTCTCTCCACTTTATGCCTTTGTTTCATCAACTCAATGGTCATCGGCGTA
5281	CaTSNP8277	GTGGTGGAACGACGTCGTTTGGGTCGAGTTTAGGATATGGAGCGTAATCAGGGGTGTGAG[T/A]GTGAGAGTGAGAGTCGTCAGAGAGAGAGACGGAAACAGTTTGTTCATCGGGTTGAACATG
5282	CaTSNP8278	AGCTCAAATGATCCTATGTCAGGTCGTAGTTGCAGCTGCTATTGGGGCCAAGTTTGGAGT[T/A]GATGGAAATCCTGGTGATTTACCAAAGTGGTATGCTGTAGTAGTTGTGCTCTTCATTTGC
5283	CaTSNP8279	TGATGTCGATAAAATGCTTTTTATGGACTGGAGAGATTCACACTTGGACAATGACAAGGA[A/G]CTCAAGGAGAGAAACAGTAAAATACCTACTTTTCTGTATGCAATGCCGTTTTCATCCACC
5284	CaTSNP8280	GGCTGTTCTTGATTGTTTGAGGAACCGACATGAATGGGCTAGGTTGGTTGACCGCGGTCC[A/G]AAGACAAATACTAATAGGAAAAGGCGTAAGCTGGATGCTGAAGAATCTGATGATAATGGG
5285	CaTSNP8281	GAAAAGAGCTTTAACTGCATTGATTCGACCTTCTTCGCATCAACAGAGGAGGAGATCGGC[A/G]TTCGAGCCAAGACCTAGTCGTCTCTCCGTCATGTCCAAGGCTGAAGATCTTTGAATTTTG
5286	CaTSNP8282	AGAAAAGAAAAACAATGATGAACATGGAAATAAGTATTATTGCTGAGCTTTGAATGGCTG[A/G]TATTTTTTGAGATCATGAGCAAGGCCTTGGATGGAACCAACAGCTGAGATGATAGAAACG
5287	CaTSNP8283	ATTTTTGGGTGGAAGAATCGATTGGGATAAACAAAATTAGTATTAATGTTGTTGTGTCTG[G/T]TGGTGGCAAAGGTGTGAGTGAGGGTAGGTGGCAGAGCATGGCGTCGTGTGTATAAGAGAA
5288	CaTSNP8284	GCCTAGGTCCACTGCTCCTCTGCCACCATGTGCATGATGGCTACATATTACAGCATCATA[A/T]GCTCCAGCATCTAAGGCAGCCTTTCTAACCGCATTTAGCTCAGCTTCAGTGTCAGTTGAA
5289	CaTSNP8285	TCATCTCTCTTTCAGATAATCAGGACAGCAACGAGTGGAGGCATGGCATAATGGAGCTTT[G/T]AGTTGGACTGTGTCATCTTCTCTTTCTTAGCCCTGTAGAAAATATGACTATACTTTTCTA
5290	CaTSNP8286	ATCATCATCATCTTCGTTATTGCTTCCGTCGGGGATTTTTCTTTTCTTGGGACCATGCTC[A/G]AGCCTATGACTATCCAATTCTTTGTCCATAGTTTCTTGTAAATTATGGTCATTGTTTACT
5291	CaTSNP8287	ACATTTTCAAATAGGGGTGGTAGATTACCAGTTCCAAGTTGGTTAGCCATGAAAGATGCC[C/A]CAGCTGGATTTCCACCATATCCACTCAGGCCATAGTTATTATACGTTGAGTTGGTACCAT
5292	CaTSNP8288	CGTTAAGCCTCCTGGATCCTGCGACACCTTATTCCTCGGCGGCGCTGGAGTCAGGGGTCT[C/T]CAAATCCATGACAAATTCGTTAAATTCACCGCTATTGCCATTTATTTGCAGTATACATCT
5293	CaTSNP8289	TGATATTCTTCACTTCAGAGGAATAAACCTGGAGGAGTGAGTAGCACCAATACCAGGCCT[A/T]CCATGCTTAACTGGCTTGTAGGAGATTGAGAATTCGGCCAGATAGTGTCCAATCATTTCA
5294	CaTSNP8290	TCCTTCATCCACAACATGGTTCACTTATGCTCAACAGGGTCTTGCAGCTTTAGCTATTAC[G/T]TTAGCACTTAACTTTAGCCCAATTCTGCATAGTGGCAATGCATTGGCTTCTGAATTTGAT
5295	CaTSNP8291	CCTCACACGAACATCACCAACCCTCCTTCCTCCAATATCAGCTTGGAAATGGAATACTCA[T/C]TCGATTCAACTGTATTGGAAAATGCCCCTTCAAAGCGGTTAAAGACAGAATCATATGCAT
5296	CaTSNP8292	AGTTAGGAAGGTGGAGAAGTTTGAGAAAAACATCACAAAAAGAGGAAGTGTGCCAGAAAC[C/A]ACTGTTAAGAAAGGAAAAGACTATCCTGTTGGCCCCGTGCTGCTTGGTTTCTTCGTCTTT
5297	CaTSNP8293	CTACTGGGCAGCCTACGGCTCATTCAGTCTTGTAGAAGTATTCACGGACAAGCTTATTTC[C/T]TGGGTTCCCATGTACTATCACTTGAAGTTTGCATTTCTTGTTTGGCTTCAACTTCCACCT
5298	CaTSNP8294	ATGCTCAGAAGTCTCAGTGAAATTAATGGTCTCTTCAACATTATGATTAGGCTCTTCAAC[G/A]CACTGATTGTGCAGAAGACAGGATATGAAATCAATTAGAGCAACACAAAAAGCTTCTGGT
5299	CaTSNP8295	AGGAGTGCCCACAACAGTGTGGACACCACCTTGGAGAATACGCTGATCCTCACGAACGCT[G/A]GTTCCACCAACACAAGCATGAACCTTAACACCAAGGTAATCACCAAGAGCACGCATAACC
5300	CaTSNP8296	GAGAGAGTTGTTGGGGCGGTGAATCTAGGACCGGGGACAGCAGTTAGGTCAACGAGTATA[A/C]TGGTGGTGGGAGGCACAGGCACTTTGGGAAGGCAGATAGTGAGGAGGGCACTGGACGAAG
5301	CaTSNP8297	AGCAAAGAAAGTGGATACATTGACCAAAGCTATGGAAGTTGAGGCAAAGAAAATGAGAAG[A/G]GAAGTAGCTGTCATGGAGAAGGAGGTAGCTGCAATGCGTGTGGAAAAAGACCAAGAGACA
5302	CaTSNP8298	TTTGAGATTTCCAGAAGATCCCCAGTTAACGCTTGAGGCTAAGGATCTAATCTACAGATT[A/G]CTGTGTGATGTCGATCATAGGTTAGGTACTCGAGGGGCACAAGAAATCAAGGCTCATCCA
5303	CaTSNP8299	CATGGAATTGGGATAGTCTCCATGCAACAAGGGTTCCATAATCCAACCCAAATAAAAATC[A/G]CGGATCCGTTGACATGCTGCCTTGTCTTTCTCTGTATTTGTCTGAGGAACTAACCCAAAT
5304	CaTSNP8300	GAACCTTTCGGTTTCTTCGCCAAGATCTGGTAGATTTTATGATGCTAGATTTGAAGATCA[T/C]CAACCTCATTTTCTTGAAGCTTGTTTTCTTTGTAAGAAACCTTTGGGAAACAATAGAGAC
5305	CaTSNP8301	ATCAAACTTTTAAAAGGCTATGATTCGTTGGAGAATAAGCATCGTTGGATAGTTGACGTA[A/C]GAGACCTAGTTGATGCAATACTTTTGGCTTATGAGAAGCACGACGACGCGGAAGCAAGAT
5306	CaTSNP8302	GGCTTCTCATTAAGGACAAGAGCTTGCAGAGAGAGAAGGACTTGAAGAACAGTGGAAGCT[T/C]CAGGATTCCACACTTCGGTCGACGTACCAGTCCATGTGTTTAAGAGACTCAAACAGACCT

5307	CaTSNP8303	TCTTGTGCATTTGTTTGGGAACCCTAGGTTTGAGTTAATTCGTCATGATGTTGTTGAACC[G/T]ATTTTGTTGGAGGTGGATCAGATCTATCACCTTGCTTGTCCTGCATCACCTGTGCATTAC
5308	CaTSNP8304	AAATTTGGATTTCATAATGCCGGCAGGACCACCCTTGAAACCATACAATGTGCTTCCACT[T/A]GCACGCTGTTGCAGGTAATCGAAAATTCCAGAAATGACATTGTGACCTCCGGGAGCCTGA
5309	CaTSNP8305	GGCTTCATTCATATCATTCTCACTAGACATTGTAACAAATCCAAAACCACGTGATCGACC[G/A]GTCTCTCTGTCATAGACTACCCGAACACTCTCAACCTTACCATGTTCGCTGAAAATTTGT
5310	CaTSNP8306	GGACCAATATCTGTCAAATCAAGGCATACTTTATGCTTTCAATGATCCAAACCATACAAC[G/A]ATGGAATCCTCCCCTCCTTGCATACAAATACCATCTGATGGAGCTGATGTTTGGGAAATT
5311	CaTSNP8307	AGGTGTTCCCTTTGTGCCACCAATGCCTCTTTATTTTGCAGGGCCAGACCCTCAGTTGCA[T/C]AGTAAGATAGTCATACAGATTGACTACTATTTTAGCAATGAAAATTTAATTAAAGATACA
5312	CaTSNP8308	ATTCAAGACAAAGCTTTGATAGAGGTTGAGCAACTTCAACATCTGATAAATTAGTTGTCA[T/G]GTGCTTTGATATGTATGGATCAAGCAATGTTAGTAGGTCATTAGACTTTGTTAATTCAAT
5313	CaTSNP8309	GTCGAAGTATGCAAACAAGGAGTGGTACACATAGGAAACATTGTACTCCACATTAATCTG[C/T]TCATTAATAGCAGCTTCAGATTGATCTTCAAAGTTCTGACGAGCCAAAGAAACATTATGA
5314	CaTSNP8310	GTGGAGTTAGCGTGTTGGGAAATGACATATACGCGATGGACTCGAAGGCGAAGAAGATGA[G/C]TGGGTTCGGTTCGGGATTGAAGAAGGAAAAGATAAGAGATGGGTTTGAAGAAAGGGTGGT
5315	CaTSNP8311	CTACGCTATTCTTTTTCAACTTTCTCCCTATCATCTCCAACGTTTTCTTTTCATGCTTGA[T/C]AGGACTTCCAGCTAAGACAATAATCCTTTGCTGTTGTTTTTTATTTTGACGATGCTTAAG
5316	CaTSNP8312	ACCAATTACATTTTCATGATCTAAATGCCTAAGAAGCTTAATCTCACGCAGCGTACGCTT[A/C]GCATCCATGTGATTATCAAAAGCATTCGCTATCTTCTTAACAGCAACCAACTCATTCGTC
5317	CaTSNP8313	TTGAGACTGCGTTTTAGTAGCTTCTGCATGTTGCTGCAATGTCAATGATTGACTGGATGG[C/T]TGCATCATCTGCAATTGATGTTGAAGCTGGGAGAAGGCCTGCTGAGATGAATGAAAGCTT
5318	CaTSNP8314	ACACGGTGCCTCCCAATGTCTGGTTTGGTTCTTTTCCTACCAATGATTTTACTGTCTCTG[T/C]TGATGGAGGGTTTGTGAAGGCCCCACCTAGAGATGGTGAGAGTCACTACTCTCTTGTAGG
5319	CaTSNP8315	ATTATGAATTGGAAAGAAACTAAAGCCAAAATCAAAAGAAGTAAGCGATAACATGTGTTA[A/G]TGAATCTTGAATTTGGAAGCCTAATGATGAGATAACGGGCTTCTGAGTATAAAGTAAGTG
5320	CaTSNP8316	TGCACAGCAAATTATAGGATGGAAAACCTTCTTCACATTCGACGGCAACCCAGAACCAAC[T/C]ATGTAGCCTAACACAGTGGCCGCAAGCAAAAATGGAAGGCATGTTCTGGCACTTGTTCCC
5321	CaTSNP8317	AAGTTGTAATTCAGATCATCCACTTCACAATTGGACAACAGGAGCTGGAAGAGATGTTGT[C/T]CCATTGAATGTCACTCGCCATTATTATTTCATAAGTGGCAAAGGTTTTTGCTATGGTGGT
5322	CaTSNP8318	TTTAAGTTGAAAAAACATAAGATTTTTGAGCTGTTCATGTTTGGGTTCGGAGGGATCGAG[G/A]TAGGAGAGGACTCTGTAACCGCCGGTGAGTTCTGTTGACGGAGTGTAAGTTCCGGTGAAG
5323	CaTSNP8319	CGTCTTTGGCCTCTGTCACCTGTCCCGGTCCATCATTGTTCCAACTACCACTAGCTCCAC[T/C]ACTTGCTGAACCCCAGCTCTGCCATTGCTTATTACTTCCACCTCCAGGATTACTTGAGCG
5324	CaTSNP8320	TCTCACAACCTTTGAAATCACACCCAACCACACCATTCGTAAAATGATTCAGAATTGGTG[T/C]GTTGAAAACAGTTCTTACGGGATTGAAAGAATCCCAACTCCTCGTATACCAATTTCGAGC
5325	CaTSNP8321	GGGTTGGGTGGCTGGTCCTGCTGTTCTCTTTGCATTTTCTTTCATTACTTATTTCACTTC[C/A]ACTCTTCTTGCTGATTGTTACCGTTCACCTGATCCTGTTCATGGCAAACGAAACTATACT
5326	CaTSNP8322	AGGCCTCCTGAAAAGCATGATGTGTGGCTCGGGACGATGAATTGCATAGTGAACCCAACC[T/A]CGGCTTTGCTGAACTCCTATTGCACGCCACTCATTTTCAGAGAGGAGCCGATTCTTGGGA
5327	CaTSNP8323	GACCTTTATGGAGAATCCTTATCTGTCAAAGTTGGATCAGCCTGGGCATACTCACCTGTA[A/G]GTAGTAGTGAACGACCTCCAGCTGCTAAGGAAAATGGAAAAGAGAATACACTAAGCGGAA
5328	CaTSNP8324	TTACAGCTTTTAGAAACTCATGATTTTGTAATGCTTCTCTCATCTTTCTTATTTCCTTCT[C/T]GCCGTCCCCCGAACCGCCAGCTAAACTCTCTACTAGTCCCTACCGAACCGCCAGCTAAAC
5329	CaTSNP8325	GCAGGATCTTCCTCAACACCAGGATTATGAATAACCATTTCAACGACATCATATTGAGCT[T/C]TAAAACCATAACCAATTGGATCCCAACCACTACTTGTATACAATGTTTGTATAGGAATAA
5330	CaTSNP8326	AGTTTCTGGCGTGTTTGTTCATACCGTTGGAAAAGAGTACGGACAGAGGACTGCGGAGGC[A/G]GCTGCGGAGAGGATGAGAGCGGTAGCGGAGATGGTTAGAGTGATGAAACCTGGTGGCGTA
5331	CaTSNP8327	CAAGCCAATTATCTTCACCATGGCAAGGTTGGACCGAGTGAAGAACATTACAGGACTTGT[A/T]GAATGGTACGGTAAGAACGCTCGCCTAAGGGAGTTAGTAAACCTTGTTGTTGTTGCCGGA
5332	CaTSNP8328	CCTGTGTCTGTACTGCAATGATCGGTGTCATCCTTTCAGCAGCTTGGAAGCAGTCAGGAA[G/A]CATATGGCAGCTAAAAATCACTGTAAAGTGCATTATGGTGATGACGATGATGAGGAGGAA
5333	CaTSNP8329	CAAGTTTGGAGCTATCCATTAGCTTTCTCGGAGTTCCGTCAGGCTTAGTACTATCCCAAA[C/T]GAGATCGCCTTCGAATCCAACTACTTCCTTCATCGACTCGGCCAATTCCTTAATAGTAAC
5334	CaTSNP8330	CGTACCTGAACTCGTTGTTATTGCCGGAGTTGCTGCCATCGTTTTCGGACCCAAGAAGTT[G/A]CCAGAAGTCGGTCGCAGCATCGGTAAAACCGTCAAAAGCTTCCAACAGGCAGCAAAGGAG
5335	CaTSNP8331	AATGGCTTCTTCTATGGCTGCTATAGGTGTTGTCAAAGTGCCACATTCTTCTTCTTCTTC[T/A]TCAAATGTAGCAAACAAAGCCATTCAACAAAGCAACCTCTCATTCTCATCATCACACCTC
5336	CaTSNP8332	TCTAGAAAGAACTGCACTATCAATCGGTTTATCAGAGGCCTCTCCACCTTCTCTCATCCA[C/T]GGGTGTTCAAGGACTTGTGCAGAAGTAATCCTCTTCTTTGGATCCTGATTAAGCATCTTC

5337	CaTSNP8333	GTCTAGTCGTGAGGTAGGACCTGACAATGTTTACAATACTGCACGGTTGATTCGAAGTAA[G/C]AATGATTCTGTGCCTAATGTTAATATGACATGGGTGTTTCCGATTGCGGGTGGATATAAG
5338	CaTSNP8334	GCTTCTTAAAGACTTGCGTCCTACCGAGCCTGAAGTCCTTGCCATCACCGACACACCTGC[G/A]TCTACCACATCAACTGCTGCTGGGTCAGGACAAGGATTGCAGAGTTCTTCATCAGCGATG
5339	CaTSNP8335	ATAACATGTTGCATTGTGAGAGAGAATAATTTATGCATGGCAGCCGGTTAGCACACATCC[A/G]TATTTGTTGAGCTGTAAATCTAAGGTTGTGTGTAGTTTAAGTGCATGTTTGGATTTGCAG
5340	CaTSNP8336	TAACATACTAGTTGATCCTAATGGTGAAATCAAACTAGCAGACTTTGGAATGGCTAAACA[T/C]ATAACTTCTGCTGCTTCAATGCTTTCATTCAAAGGAAGTCCATACTGGATGGCACCTGAG
5341	CaTSNP8337	GGGGATACTCCCAAGAATTCATAATGAGAACCTTCAAAACTGAGTAAATCACCAAAGTCT[T/A]CACCCAACAGCTTATTAGGTTTTTCCTCTGAATTGGTTTGTTTCTTTTTTTCACTACTTT
5342	CaTSNP8338	TGACCCTTTTGTTGTGTCTTCTTTATTATATTCTCCTTCATTTTCGTAATCTTCTTCGTC[A/G]TCGTCGTCGTCATGCTCATCCACAGGCAAAAGGGGCATTGATTTTGGATTGAAACATGAA
5343	CaTSNP8339	CATCCTCATTCCTTCTATCATATCTTTAAATGGCAGGATATCAACAGGAAATTTGGCAAC[C/T]GTATCCGATAAAGCAGCGTCAAGCATATCAAACGGACGCCCCTTGGAAAAGTTCTTCCAAT
5344	CaTSNP8340	TACATTTTTGCGACGCCTCTCGTTATGTCCAGCTAAACGCCTTCTACAACTTCTTTTTAA[C/T]TCATCAAATTCTGATACCTCATGAAACCTGCTACATTGTTGGCAAAACCTTTGTTGTAGG
5345	CaTSNP8341	CTCACCACCCTGAGAACCTCCATCTGATGAACCGCCACTAGAACCACCTGACATATGTTG[T/C]CCAATCTTGGACACAGCTTTGTTTGCAGCATCAAGCTTTGACTTAATTTCATCAGTATTG
5346	CaTSNP8342	GTGGACAAGTTTTATTATCTCTCGGGCAGATATGACATTGAGCTGACTGTTGGTGATGCT[A/G]TCATGGAGAACTCTTTCTTGCGGCCACTTGGTTTTGTAGAATTAGATCTTCCAGAAGCAC
5347	CaTSNP8343	AAATGAACCACCCCATTGAAAACTCCTAGACAAATCATTGCCTGTCCCAAGTGGTATGAT[A/C]CCTACTGGAGGAACTGGGTCTCGGCCTAGCTTGCTAAGTTCTGTAAGACATCCTAACACC
5348	CaTSNP8344	TCTTCAGGTGCTAGATACTCGTGGACATTTGTTGGTTGCCTTGGGTTACGGGTTATGGCC[G/A]TCAATGGTTCTTATCTCAGAAATTGTCCAGACCTTCATCTTGGCAGATTTCTGTTACTAT
5349	CaTSNP8345	GAGTCCGACTTGGTGATGGTTCTGCTTCAGCCCTGCTAGATCTTGACAACTCACACACTG[C/T]GTCTGATCATGGAACAGCAAACAGTTTAGATGGTAACAATAATGATTCTGGAGGAGGGCT
5350	CaTSNP8346	AACAAGGAGCTCAGTGGAAATGATATATTTGGCCCACCTCCTGAAATTGTGCCCCGTTCC[G/A]TAGCAGCTGTACGAACTTTGGAATCAAAGGAAAGTAAGGACATGGGAGAACCTCTTCCTA
5351	CaTSNP8347	TCTCAGGATGTTGGACTCCATGCTAGGCATATCAGTACTGTGTCCACGGTTGACAGCGAA[T/G]TAAATGATGAATCTGGGAATCCTATATGGAAAAATAGAGTGGAAAGTTGGAAGGAAAAGG
5352	CaTSNP8348	GATATTTGAAGTATAAACTTTTGGGGTGCCACTTGATTGTGGTATAGCAACAAGAGCTTG[G/T]GCTCCGATCATAGCTGGGTCGAGATTGTTGTTAGGATTGATGGCCCAAGCAACCCATCTA
5353	CaTSNP8349	ATAGTTTAATCTCAAAAATCTCTCTCTCTTTGTTTCTTCTTTAGCATCAAAACTTCCAAC[T/C]ACACACTCCTTCTCTTCTTGTGCAACTGTTTCATTATTACAATCAATATTCCATTGAAAA
5354	CaTSNP8352	GGCACGCAAAATCCGCTTTGAGTTTCACAAAGGCGCAGTAGATCATACATACATGAGGAT[T/C]GATGGGGAACCTTGGAAGCAACCTCTCCCAACTGATGATGATACTGTTTTGGTAGAGATT
5355	CaTSNP8353	GAACTTGAAATGCAGTAGTGTGTTTCTTGATGTCAGCTTCCGAGCTGTATTGGGAGACTT[T/C]GGGTTCGTTCTAATGGGAGCAGAGTCGAAACAGTTTGAATTAGCAGTGTGTAATGGCGCT
5356	CaTSNP8354	AAATTTTGTTAATTTAATAAATGCCAACCCTAATTCACCCATTGTCTCTCCCATATCTTG[T/C]TGTGCTTTAACAAGTGATTCAGCCTGCTGAGATGCACCATTGATTTGCAGTTCAAGCTCA
5357	CaTSNP8355	ATACACCATATTTGGTATTTTCTGTGGCACGGTTCAAGGAAGCAGAGATACATAGACCAA[C/T]GATTTTTCTTGTATCAGCTGGATCAATTATTCCATCATCCCAAAGCCTTGCTGTTGAGTA
5358	CaTSNP8356	AAGGTGAACCACAAACACAGCAAAACCAATTGGAAGTGGTGCCAAAACGGGAACATGAGA[G/A]TCTCTAGCATTTCTCTTAGGATCAGTGGCAGAGAAAACGGTGTAGACAAGAAAAAAGGTT
5359	CaTSNP8357	ATTCCCTAAGAAGCCTATAACCACAGACAACAAGAGATTTGTGATTCTCACTCTTGTGCT[C/G]CTAGTATTCTCCTATCTTGCTGCTATAGCAATTCCAGATATTTGGTACTTCTTTCAGTTT
5360	CaTSNP8358	CTTGCATGGGTTCAAACAGATAGAAAGTCAGGTGGTCTTGGTGATTTGAACTATCCTTTG[G/A]TTTCTGATGTCACCAAATCTATATCAAAATCTTACGGTGTTCTCATTCCTGATCAGGGGA
5361	CaTSNP8359	TGTCTTCCTTCGGAAACTCGGCTGCTAACTTTTGTTGATCACTTAATCTCTGATTGCTAC[T/C]ATCTGGCCTTGAAGTTTTCTTGGATGTTTTTAAATCCTCCGGGTCATCACCATTGCTCTT
5362	CaTSNP8360	AAGTCCGCGTATTCGTTTCAACAAGGATGGAACTCTTTTAGCAGTCTCATCAAACGATAA[T/C]GGAATCAAAATTTTAGCCAATGCAGATGGTATTCGTTTATTGCGTACATTAGAGAATTCT
5363	CaTSNP8361	GGAACTGATGATGCCAAAAGAAGGGGTGATGCTTTTGCTCGTGCATTTTCTGCCCACTTG[T/G]CAAGGTTAATGGAGGAGCCTTCTGCATACGGGAAGTTAGGTTTGGCCAATCTATTGGAAA
5364	CaTSNP8362	ACTGAAAGTAAGAGAGCAAGATGTTGCAACCCCATTGGCCAAGCCAGCTTCAGGAAACTC[T/C]CCGAATTCATTGAAACACTGTGCTGAAGACATCAAGGAACCTTTATCTCCTGAGTCTGTG
5365	CaTSNP8363	AGGTTGTGGAACAGCGTCAGTCCATGGTTGATCAATTGCTGCGGGCCATTGACCGTCGGA[T/G]GGAATAGCAGATACATTGAAGTCAGCAATGGCGTAATCTGGTGCAGCAACTTCATCTTCC
5366	CaTSNP8364	ACAATAATCCCTACTCAGATAAAGTCGACTTCAGCTCTCCATTACTTTTCAGCTCCATAA[C/T]AATATCGCAACCGCCTATCAGCTCACTTTTGTAGTAAAGCTGAGGAAAGGTAGGCCAGTT

5367	CaTSNP8365	AAACTCTGCTATAGCCAACTGCTCCCGCGATCCTAAAGTTGTAGCAAGGTATTCCAAATA[A/G]ACTGATAATGCTGATTCAACAGCACCTCCACCAGCCACCACGGTATTTGACTCAAGCGTT
5368	CaTSNP8366	AGTCTTATTCTCTCCGTTGGATCCATGCTGCTGGTGATGGTGCTGATGATGATGCGGCAC[G/A]TGAAGCGGTTGTTGATACTGGTGGTGGTGGGGGTAAGGCACATAAGGCGGCGGCGCGTAA
5369	CaTSNP8367	TGGTTGAAATTTCTCATGTAAAAATTCTAATCCCCTAGCTGCATCAACTGCAATTCTAAC[A/G]CGCTGCATCCAGTTAAGAGTTGGCCCGGGTTGTGCCCCTTGAACTCCCTTTCTACCGTGC
5370	CaTSNP8368	CGATGCGGCGGTTGGAGAAGGTGATGTGGCAGGGCTGATAGTTTTAGCACCAACGTTGAT[T/C]GAAAGCTTTTGACCAGCGGAGCAGTGTCCCGAGAAACCACATATGAAATAATGAGTACCA
5371	CaTSNP8369	ACCATGAGGTCCAGGAGGATCCATGGTCATGTCCTTGAAGTATGGAGGCTCGTGAATATA[T/C]GTTGAGTTGGGGTCCCATGAGTAGAGCTTCTCAGCAGGAACTTGTAGTTGGTTCCACATC
5372	CaTSNP8370	CAATTCCACCACATACAAATCATGTGATTATAATGATGCACAAGACAAGGACACATTCCA[G/A]TGGTCATCACTTGATCCATCCAACGCTGAGACACATACCGTGGCAGTTCCTTTACTAAAG
5373	CaTSNP8371	CCTTGGATCAGGTTCTGTATAACCTGCTTCCTTTGCTTCAGCAACTACATCGCTAAAAGC[T/C]CGGCCATCTTTGAAGTTATTAAAGATGTAACTCAAAGTCCCACTGAATATTCCTTCAATT
5374	CaTSNP8372	TGTAATCTAGAATTCGGCCCGGTAAGTAAAACTTGACCAACAATTGTTCCAGCCAGAATT[T/C]GAACTCGTCTTTTATTGGAACTATATAATGGATAATTTGAAGGAAAGGAAGGTGGGTTTG
5375	CaTSNP8373	GTTTACCTCTGGGGCAAATGTTGTGGTTGTAGGAATAAACATCACAACCCAAGTGAAATT[G/C]ACAGATGACGATCTCCTTGAACTGAAGGAATCTAAAGGAAAGCATGCATCTCTTTTAAGT
5376	CaTSNP8374	GGCGAAGCTCCCTTTGTATGGTTTCAAAAAGGGTGACAATGTTGAGGGAAAGGACTTGTC[T/C]CTCATTGCATGTAAGGAAGGCCCTGTGGCTTTTTTGGACTCAATTATTCTTGGACAGTGG
5377	CaTSNP8375	AGTTTGGTGTGTGTGCTGATTTATTGGGTTTGATTAATGTTCAACTTGGTAAGCCACCAA[G/A]GACACCATGTTGTTCACTCATTGATGGTCTTGCTAATCTTGAAGCTGCTGTGTGCCTTTG
5378	CaTSNP8376	TAGTTGATTCTTCATCAAGTGGGACAACCTCGCCGTTTGAATTGAGAACTTTGTCACAGG[T/A]AGCAAGAACTATTTCAGAAGCACCTTTACAGTGAGCTCTTAAACCTCCACTAGGGAGCTC
5379	CaTSNP8377	TAATAATAATGGAGGATGGTTAAGGGCTTCAAATGATCAAGGAAACAATCTTGAGTCTCC[T/C]ACTTCTACTTTATCTTTTAATGAGAATGTTCCACCAACTATGACTAGTGATGGAGTTGGG
5380	CaTSNP8378	CCTGAATTAGAATGCATATTCACCCAGTCCACCAGGTTACCCTTAAATTCTTCATCGCCA[G/T]TGTTCACCTCGAGAGGTTTGCACCCGGTAACCAACTCCAAAAGCAAAACTCCAAATCCAT
5381	CaTSNP8379	AAGGAGGACGTTTGTGGTTGCCACTGTTCCTCCTCGTTAATTGACCGCTCATTATTTTTT[G/C]CAAACAGAGAGCAAGAGAGAGAGAGGTTAGTTAGGTTAGGGTTCTTCCGTTTCGGTTTAT
5382	CaTSNP8380	GGTTGTCTTGTAATCTTTTGTAGATTGAATTTAATAGCGAAATCTCATCTCCAATGCTGT[C/G]TAGGCTAGAAATGAATAGCAACACATAGTTATTCTTGAAGACTTCAATACCCGATTTAAC
5383	CaTSNP8381	TTCATAAAAGTTGTCAAATCCATCATCTTTAGCATTATCCCAGCCAGCCCAACCAGCATC[G/A]TTATTATGGGGAGCTGATCCTTTGGCTGGTTCTTCCTTCCTAGAATCTTTGTGGTCCCAA
5384	CaTSNP8382	AACAGTAGAAGCAGCAGCTGCTGCTCAGCGTGCAATGCACATGAGGTGGTTTGCACGCAG[G/A]TTAATTGCAGCTATTTACATGCAACCTCAGGTATATGAAGCTAAGTTTAAAGGGGAGGTT
5385	CaTSNP8383	CTAGAATTTCTGTTGCTGTTGCACAGGAAGAAGTGGTTGTAGCTGCTGAAGAGAATGCTG[A/G]AGAAGTAGAAGTAGAAGAAGAGAAGGAACAAGGAGGGGAAATTGTAGCCAGAAGAAGACA
5386	CaTSNP8384	AGATATGTTGGAAAAGGCGGCTATAGAGTCACCTCTGTCTGAGATTCTGGAAATCAAGAT[T/C]CCTGCTCTGAACATAGAAGAAAATCCGATTCAAGAAAACAAACCGCTTCCTGATTTACTA
5387	CaTSNP8385	TACGCGACTAACACCGTCGACAAGAACCGCTTTTGCTTTTAACGAAACAACACAACGTTC[G/A]TCGTCGTTGTAGAATCGGAGACGAAGCACGGCACGGCGGGATGAAAGTTCGGAAGCGGCG
5388	CaTSNP8386	TACAACAATTGATGAAAATAAAGATGGACATCTTACTCATGGTGAAATGAGAGCACTGGT[C/T]GTTGGAATTCAATTTGAGGAGATAGACCTGGATCATGACGACGCTGTGAAAAGAATAATG
5389	CaTSNP8387	GCAGTGGCTTGTCAAGATTCCATCTGAACAGGAACAGTTAAGGGCACGCCAGATAAGTGC[A/T]CAGCAAATAAATAAGGTGGAAGAATTGTGGAAGACAAATCCTGATGCTTCTTTTGAGGAC
5390	CaTSNP8389	AGTATGGCATTTGTTTCTTCTGGGGTAAGAGAGGCTTTAGTAGCCTTAGCTTTACTGACA[G/A]CAGGTACCAAGGGCATCTCATCTATGTCAGGTCTATCCTCATGGTTTTCTATCCAAGTTA
5391	CaTSNP8390	AAGCACAAAAGGCCTGTTCAATATTGTAATTCACTAGGTGTAAACAATTGATTACCCTTT[A/G]TTCTACCTGTATTCCCCTCCCCTTCTTTCTTTTAATCATTTCTTTGTTATTAAAGAAGTT
5392	CaTSNP8391	TTCTGCCTTGAGGAGAGCAGGAGGCACAGCTTTTAGTAATATGGCTGTTGGTTATAATAA[T/C]GTAGATGTTAATGCTGCAAACAAGCATGGTATTGCTGTTGGGAATACTCCTGGAGTGCTC
5393	CaTSNP8392	GTCATAAGCTGAAGACCACTTGTCCTGAAGGATGTCGAGACATATGTTGCCAAACTGGTC[A/G]ACATTTGGATGAAAGCACATAGTCTCAAACTTCACTTGGGGTGGCTTAAAAGGATAGTCC
5394	CaTSNP8393	TCAGGAGTATGAATGTTACCCAAATTGCCAAAAGAAGCCATCATTGGTGAAAATCCAAGA[T/C]ATTCATTTTGCAAATGTAAAGGGAACAACAATATCACCAATTGCAGTGGATTTGAGATGC
5395	CaTSNP8394	GTTCATTGAGGAAGTCATGGACCTGGTGGAGCTGAACTCATTGAGGAACTCCTTGGTTGG[T/A]TTGCCTGGTGTGAGTGGTCTCTCCACCGAGCAACGCAAGAGGCTGACTATTGCAGTCGAA
5396	CaTSNP8395	CATAATCAGCCAATGTTTAAGCAATGCAAACGATGGTTACAGTGGCGTATTATCAACTAC[G/A]CGGTCAAAGGTGAACGATAAAAGAATGGTTGCTGTTAGAGCTGGTGGTAAAGGTCTTACG

5397	CaTSNP8396	CTTCTTCAGATGATCTATATCTTCAGGTTTCTGTGGTTGGGATCCCACAATCAAATACTC[C/A]GTTATAACTGTATAATTCATACCCAGATCGTGATGATACTCATAAGGGTTCCTCATCATC
5398	CaTSNP8397	GACTCTTCTCCAAGCATATTGAATACATATAAGCAGTGTAGGAAAAAATAAGTAGAGTAA[C/T]CTCAAAACCACAAATGCAGTGGCCAAAGCCAAATAAGTTTCCTGACGCAATAAATTTTCT
5399	CaTSNP8398	TCATCAATTCCAGGAAATGCAAGTTTACTTGGATGGGCTATGAGCTCCTTGACCCTGAAG[A/G]CCAAACCTTCTGATCATGCTCCAGTTGCTTCTGTTAGTTCTAGTGTCCTTACACCAACAT
5400	CaTSNP8399	AACTAAATATGGTATGAGTACTGAAGTTGGACCTGTCACTCACAATTACTATGATAATGG[A/G]AGAAGTATGAGCTCAGAGACTAGGTTGCTTATTGAGAAAGAAGTGAAGAACTTGCTTCAG
5401	CaTSNP8400	AGGCATGTACTCATACACCAAAAGACTTGAATCTTCGCTAGTTATACTGCAATAAAGCTT[C/T]ACCACGTTCACGTGTCTTATAGAACTCAAAGCCTGAACCTCAGCGTCGAATTCATTCGAC
5402	CaTSNP8401	AAAATCAGGAAAATTCGATGAAGAAAGTTTGAATTTCCAAAAGAGAATAGTAATGTCATC[T/A]GGCATAGGTGATGAAACATACATTCCAAAGTCATTAATTTCATCAACAGAAAACACAGCA
5403	CaTSNP8402	TGACAATGATAAGGTTCAATGTCCTGAAAAAACTTTCGGTCAATGTTCAAAATGCACAAA[T/C]CAATTTCATGATATTAAGAAACATACCTGCAATGAAGTAACAGCTACCAAAAATCCGATC
5404	CaTSNP8403	CGGAGGTGACAATGAGTCTGGTGGTGGTATCGAAGTCTGCGCTTTTGATAATCGGGGCGT[C/G]GGTCGGAGCTCCGTGCCCATTCAAAAATCCGATTATTCAACCATGATCATGGCAAAGGAT
5405	CaTSNP8404	ACCTGCCCAGTCTCTAATGGGTGCATATCCAAATGCACCTAATCAATGGACTAACTATGG[A/C]GCACAGCCTCAAACTTGGCCGGCGGCCACACAAGCACAAGCACAACAATGGCCTGCTGGT
5406	CaTSNP8405	TGGATAAGTTTGTTTCAGAGGTAGGTCGCTTCCCTGTCGCTGGTTCAGAGGACGATGCAC[G/A]TAAATTCATTTCTATTGCCAGTAATATTAATGAAAACTTAGGTGATGGAAGGTTGGAAGA
5407	CaTSNP8406	TATTGTCAATGTTATGCGGGCTCTTTCTCTTGGACAGCTAATGATTTGTTCCTTCCTCCT[T/C]TAGCACTCGAACAAAGCTAGAGCGGTTAATGGCCATGCAAATTTCAGCAATGGTTTCTCC
5408	CaTSNP8407	TGCAATGAATTCTTTGGTACGGGCGGCAGCAATTATCACAAATGCACCTTATCTTCTGAA[C/T]GTCGATTGTGATCACTACATTAACAATAGTAAGGCACTTAGAGAAGCCATGTGTTTTATG
5409	CaTSNP8408	CAGCGGAAGCAACCAAGTAGTGGATATTGGGAAGATGTGAAAGGTCGGCGAGTGTGAATT[C/G]ATCACCGGCCAAGAAACGAGTCTCCCCAAGCCTCTTATCGTACACATCAAGCACTTTCCC
5410	CaTSNP8409	TTCCATGAGAAGACGGAGGAAGAGAGAAGGAGAAGGAGTATGAAACGCAGCGTTTGGTTG[C/T]TCATATTGTGATTGTGAATGTGAACGTGATTACATAGCAAAAGAAAGCAGAAATGAAAGT
5411	CaTSNP8410	GGTGAGAGGCGAAGAAAAGAGAGGGGTCATGACCGACGACACCGGGACAGATATAGAGAC[C/A]GATATAGAAAGCATCGCCGTGATTACATGGATGATTACGATCATGATGAACTCTAATGGT
5412	CaTSNP8411	GGTTCTAGTGACCGGAAAATCGCCTGCGAGACACTTGCCGACGATCACAAAACGGATCCG[T/C]CAGAGCGTCATCCCGATTCTCCGCCGGAGTCATTTTGGCTCTCCAGAGATGAAGAAACCG
5413	CaTSNP8412	TAACAAAATGATTATTGAGGAGACAAAGCGCAGTATTCACGATGCACTGTGTGTGGCTAG[A/G]AATCTTATTCGCAATAATTCAATTGTCTATGGTGGTGGTTCAGCCGAGATATCTTGCTCC
5414	CaTSNP8413	ACCGGCATATGCTGCAGCAGCCACAGCAACCCCTGCTATTAAAGGTGTAGTCATAATTTC[G/A]AAACCTTGAACAACGAAAGTGACGAGTTCGAAATAAGATTGAAGAATAGAATGAAAGGAA
5415	CaTSNP8414	TTTGTATTCAGGTATCCCTGAACAAGCTGAAGCTCCAGCAGCGTCTCGAGTGCCTGCAAG[T/C]GCACAACCTGCAAATCCTGCAGCTGCAGCTCCTCAAGCAGCACAACCTGCTCCTGTTACC
5416	CaTSNP8415	GAGAGTGAATTGGCCTGGTTATCATGTTATCACAAGTGCAACAGAGGCTAGTAAGTTTAC[A/G]GTAAAACAACTCATCCAAGGTAATGTTTGGTTGAAGAACACAGGAGTGGCTTTTATTGAA
5417	CaTSNP8416	GCGACAGAACCTCCACTTGCGAACACCATTTCACTATCTTCCCGTTAACATTCTTTTCAA[C/G]TTCCTCTTTACAACTCAACTCATCATCCTCTTTTTTTCGTCTACTTTTTTCTCTCTAATT
5418	CaTSNP8417	TAGATGGTTTAGAAAGATTGCGGAGCTGCATGATGTTCCAGATATAACAAGTGCAATATC[T/C]GATGACGATTTCCCTGAAATAATGCCAATGCGTAAAGGAGAAAATCGATTTGTTCTTAGC
5419	CaTSNP8418	ACACCATCAGGAATGATTTCCACAGGAAATTCCTTCACAGATACATGAAGTAAGTCCTTA[A/T]GTCAGAAGCCTGAGTTGTTTGGAGTCTGAAGAAACTTGCAGTCTGTATCAAAATGTATTA
5420	CaTSNP8419	AATTTTGGAGCAAGCTGTGCTAGAAATGAACGATGATTTGACTAAAATGCGTCAAGCTAC[A/G]GCACAAGTATTGGCATCACAAAAACGGATGGAAAATAAATACAAGGCTGCACAACAAGCA
5421	CaTSNP8420	AACTCAATTGCAGTGTCAGGGTGTGTGTGGATGTGATAGGTTCAAAAGTGGTTCAATTGG[T/C]GATGTTATTAAAACAATAGTGAAAAAGGATGGATACAAAGGGCTCATGCGAGGGTGGGTT
5422	CaTSNP8421	CTTAATCTTTCCCTCTTCAACCAACTTTTTAAGCTCTCCCATAGTGTCCTCAATAGGTAC[C/T]GATGTGTCAACACGGTGCTGATAATAGAGATCAATGTATTCGACACCAAGACGTTGAAGA
5423	CaTSNP8422	GGTAATGAGCTATGATTCACATACCAATAATGTAATGGCAGTTGGATTCCAGTGTGATGG[G/A]AACTGGATGTATTCTGGTTCGGAGGATGGCACGGTTAAGATCTGGGATTTGAGGGCACCT
5424	CaTSNP8423	AGTTCGGGATGAATCTATTTTACATTCGTCTAGCATGTTATGTAATGGTTACCCTGAGAA[A/G]TTGATTAGTGGCAGCTCTAATGGTTTGCTTAATAATGAAAGAAACAGGCAAAGCCTTGGA
5425	CaTSNP8424	TCCTAAGTGGAAGGGATGTGACTCTTCGGTGTTTGTCCATGAATCTGTAAGGCTTATGCA[C/T]GAGGCAGGTTATGATATTGGAAATTTAGATGCTACATTGATACTTCAGAGGCCGAAACTA
5426	CaTSNP8425	AATTATCAAATCTCTAACTCGTTCCAATAGAATCGATTCTCCGATTCTTCAAAAACTTTA[C/T]GCCGAAATCGAAACTGATAAAATTGAAGCTGACGGTCACTTATTGAATGATATAATTCTC

5427	CaTSNP8426	TGGTACAAGTGGAACTGTGTTTAATCCTGGTGATGGTGCTGTTGCTGTTGTTGGTAATGG[C/T]GGTGATGATGTTGGTGGTAATGGTGGTGATGGTGATGTTGTTGGGGGTTGAATTGGTGAT
5428	CaTSNP8427	CTAGAAAACAGAATATAAATGTTCCTTATAACCGACTTAACATCGAAGCGGCCGAAATCT[G/A]CTTCTGCTCTTCCAGGCTCCTGCCACCTAGTAATATAGAAGCCTTTTGGGAGAAGATGAT
5429	CaTSNP8428	ACAAAACAATAAATTTGTAGTGGGGACCAATGCCTCTAAAAACGACAAAAAAAGAGAGTA[C/T]ATAGTTGGTTTTCAAAAGGACTATTGGTGGTGGCCGGTTAGTGAATAAAAAAATGGTATG
5430	CaTSNP8429	AGATTGAGTCTTCGAGATCATAGCATCTAAGAGATGGTCAGTGTTTGTTCCAGAGAAGAT[A/G]CCATTGTTTGATATTGCTTCGTTAACCGCATAAATATTATCATGACTTACAACCGGAAAG
5431	CaTSNP8430	CTCAATCGGAATCGACATAATCCACGATCCAATCGTACTTTTCCTCGACGAGCCAACTTC[A/C]GGACTCGATTCAACAAGCGCGTTCCTGGTTGTAAAAGTTCTTCAAAGAGTCGCACAAAGC
5432	CaTSNP8431	CTTCAAGAGGAATTAGTTAAAAAAAGTGCTGAATTTAAGCAATCAAGTGCCGTTCTTGAG[G/A]TCAAAGAGTCAGAGCTTGTTGATGCTAAGCTAGAAATCCAGCATTTGAGATCTGAAAAGG
5433	CaTSNP8433	GCTCATTTTAGTGGCCTTGTTGCTGCTCAGGTGGTTAACAATCCGTTTGAGTTCTGTGAC[G/A]TAGTGACGACAACAACTCACAAGAGTTTGAGGGGCCCTAGGGCAGGTATGATCTTCTACA
5434	CaTSNP8434	AAACTGAAGGTAATCCAACATGGACAGGTACTTAGCATTTCTAAGTTTCAAGTCCTGTGC[A/G]TCGTTTGGGGTCTTCTCGGCTTGATTCTCAATGTAATGCTGCTGCATTTTTGCCGACTCA
5435	CaTSNP8435	TGGAATGTGGACTGACATAAGTGTTAGACTTTTTTCTCTTCCTGACCTTAATCTCATCAC[G/A]AAGGAACATTTAGGAGGAGAGATAATACCTCGATCTGTTCTTCTTTGTGAGTTTGAAGGG
5436	CaTSNP8436	CTCTTCATCTCCTTGTTTCTGTAAAATCAAACTCAAGAACTTCCCTCTCCATTCCGCTGT[C/T]GTTCCTTTCATTCCTCCTGACAACAACTTCCCCGACTCACAGATTCAACCTATCGCCGCT
5437	CaTSNP8437	TGGATGTTTTGCTGTGACCTCTCTCAACTCTTTCATGGCTTCATGTTCTCTTGGGAACAC[G/A]CTTGTCTCTAGAATATACTGGTAAAGAGCATCACTTTGCAGAAGGCTTTTGTGACCAACC
5438	CaTSNP8438	AATGTCTTCTGGATTTGCCACTGATGACCAGTATAATGTGTATGACAAAGGCTTGTTCAC[T/C]GCACAGCCAACACTTTCCACCTTGTACAGGCCGAAGAAGGATCTTGATAACGAGGCTTAT
5439	CaTSNP8439	CAGTTCTTTGTTAACCTTTTCCAGTTCTCTGCCGAGAGGCTCGGTTGGGATCCTAAACCA[G/A]GTGAAAGTCACGATGATGCATTGCTGAGAGGAGAAATTTTGACTTCCCTTGCCGAGTTTG
5440	CaTSNP8440	CTTGTTCCCATGCTTTTAGGTTACGCTTCTATGCTCCTTTATCGTCCGAGATGTGGTATC[G/A]TCACTTTCTTTTTGGGATTCGGTTACCTCATTGGCTGCCATGTTTATTATATGAGTGGTG
5441	CaTSNP8441	CATTCACACCTCTTCCAAAATTCAGCGAATTGAGACATTAAAGTTTCAAAATTGATGATA[A/G]ATAAGAGCTTGATCATATAGCTACAGATTTGGACTACTCTAGATTGGTGTTGCAGCAACA
5442	CaTSNP8442	TATTAGATAGAAGTATAGCGGCCCCACCCATTCGGAAAATGCAATTAGATAGAAGCATAG[G/A]ACGGTCATTTCCAAAATACCAATTAAGGGTTAAATTCTCTGTGCTCACAACCACAGCATA
5443	CaTSNP8443	GGAACCATCAAGAAGCTAACCGCTGTTCATGGTGGCAAAACAAGCTATGTGCTACACAAA[T/C]TAGATGCAATTGATGAAGCAAACTTTGGATATAACTACAGCTTAGTGGGAGGAACAGATG
5444	CaTSNP8444	TTTCACTCTTGGATTCTTTTCATCTGAACACTTGGTAGGAATTGGGTAGTCTGGAAAATA[C/G]TTGGCAAGAATTTGAACTAACTCCCCACGGTGCAAGGAACTTTCAGCACATAAGTATCGA
5445	CaTSNP8445	GTGGGCTAAACAAGACCTTCTCCGCGAGCAAAACAAAGAACTTGCATCTTTCAGAAGGGA[A/G]CGTGACCATTCAGAAGCTGAAAGAGCTCAGCATATACAACAAATACATGATCTGCAAGAA
5446	CaTSNP8446	ATTATGTTCAATAATTCTTTGTTTCAGATCTTCTGCTGCTTTTTCACTCAAAACTTTCC[G/A]GAGACCATCTCATTGTCAAACTCATTCTTATATGTATCCCAAAGATTTGTCCATTGGATG
5447	CaTSNP8447	CTTATTTTTAAAGTCTTTGGTTTCAAGACACAGACAACGCATTGCTCATATAGACAAAGA[C/T]GTTGATGGATAAAAAGTTGTATAAATGATATAATCACCATTGAGCAAATTGCTACTGTGG
5448	CaTSNP8448	GAATGCTGGCCCTAATATTCCTGGTCCACCTCCAAACAATGCAGGTGGGTACCCTCCTAA[C/T]AATGCTGGAGGATATACTCCTGCCCCTCCACCCAACCAGGGTGGGTATGCCCCTCCACCC
5449	CaTSNP8449	GAACGAGTTTGCCGGAGAGGAAGAGATCAGGTGTATGGCGAACTGCAGACATATATTCCA[T/C]CGGACGTGTGTGGACCGTTGGATTGATCATGATCAGAAGCTGTGCCCACTTTGTAGGACC
5450	CaTSNP8450	GCCACAAGAAGAACTAGCTCAAATTTCCGTCACATCGAGTTATTCGACCTAATTAACAAC[G/A]AGTTCAAGGAGTTAACTCGTTTCATCTCACCTCAATCTCACCACCTCAACCCGTTTATCT
5451	CaTSNP8451	CCTTGTACCTCCTGGGTATGTAGCATGGGATGAAACTTCCTTGCTCTTGAATACTGAATC[C/T]ATCATATCCAGCACTAATTGTCAGGATGAACTCAACAATCTTCAGGGAAATGAAGCTGAT
5452	CaTSNP8452	CATCCTCATTCCTTCTATCATATCTTTAAATGGCAGGATATCAACAGGAAATTTGGCAAC[C/T]GTATCCGATAAAGCAGCGTCAAGCATATCAAACGGACGCCCTTGGAAAAGTTCTTCCAAT
5453	CaTSNP8453	CCGTCTCCTTTTATTATGGCCAGCCAAGCGCCTTCGACAGCTTCTCTTTCCTTCATCGAA[C/T]TCTTGAAGTATGTGAAACCTACTACACTGTTGACAAAACCTCTGCATTGCATTTCCCACA
5454	CaTSNP8454	AGTGTAGGTTTGAGGAAGAGGAGTGCAATGATGAGAATTAGTGATTTTTCTGATCTCAGA[G/A]CGCATCCTGTAGAAAACATGTTTCCAACTGCCTCTGTCGCGTGAAGCCAGCAGTTTGATT
5455	CaTSNP8455	CCCCATCTTTTCCTGGATTGCATGTAGATCAACCCTCATAAACGGATTGGTTTCCAGCTC[G/T]TCTTCAATGGTTGAGGGAATTGTTGGTTGGCCAACTTGCCGCTGATTTTGAGCCCAGGTT
5456	CaTSNP8456	CCAGTTGTGCTGAGGTGCATATCTTACTATCTCATAGATTGTCCATCCATGCCAGTAATC[A/G]CTATCAATAGTAAGAAGCTTTGTAAGAGCAAAAGTACCAGCAGCAAGAACAATACTAGCA

5457	CaTSNP8457	CAAATTCCTCACACCCATAATCACATGAACACCACGCAAAGCAAGGACACGAGTAGTCTC[A/G]GCCCCAATGCCGCTGGATGCTCCAGTGACAATAGCAGTGAGACCAGTGGCATCGATTCCA
5458	CaTSNP8458	GGGCGCAGCTTGTGGAACCATAGAGTTCTGGGGAGTAGCTTGAATCCCAAAATTATTCTG[G/A]CCAAATATGCCTGGAGTTCCACCGAAGCTACTTGCACCAGCAGGTTGTGTCTGGCCGAAG
5459	CaTSNP8459	GCTATCGACGGCTCCCGCAAAATCATCCCTGGTAGAAAGCATGCAGATAAGATGGTGCCG[G/A]AAGGACTGATAAATATACCCATTTCAACACCATTGAAGACCAAGAAACAACTTGATAAAA
5460	CaTSNP8460	GATGATACAATATGATGTAGATACATGAAATGAATTAATAATGTTAGGGTCCTCCGAAGG[T/A]AGGTTAAGTTAGAGAGATAGAGAGAGAGAGTTGGGTTTGTGTTTGAAACCTGAAGCTCTG
5461	CaTSNP8461	TGGACTGTATTTCACTGTATTTTCAGTTGTTTAGACGATGTACAAGTTTTAATTTGGTTC[T/C]GATGTGTGTTTGCTTGAGTCAACGTTCAAGGGTTTGTTGGACTGTTAATTTTGTGGCCCT
5462	CaTSNP8462	TCAACAACTTCAGTTCCAGCATTTTGAATCCTAACTGTTAGCTCATCAATTTCTTCATCA[G/T]TGAAACTCACTGAAGGTCTTGTCTTTGACAAGATAGGAAGAATGGTAATCCCGGCATGGC
5463	CaTSNP8463	ATACACCTATGTGCTCAATCGCCTCCAGGGCATCATTGAACCAGCTTTTTCGAAGCCTCG[T/A]ACTCCTTCAGCCCCATGTTTTTGCCTTCAAGTTTCCACTACACACCAGCAAAATTCAAGC
5464	CaTSNP8464	GGAAGACTTGGTTTTGAATATCTTTGCTTCATCGTCTCCTTTGTATTCCTGACTCTGTAG[C/T]GCTGACAATTTCTTTTCATGCTCAATCTTCACACCTTCTCTAGACTTAACTTCGTCATAG
5465	CaTSNP8465	AGGGCTAGCTAGCAAGATAATGGAAGTGTTGTCTCTAGAACTAGGGCTAGAAGGAGGAAG[A/G]TTAGAAAAGGAAGTTGGTGGAATGGAAGAGCTTTTACTTCAAATGAAAATAAACTATTAC
5466	CaTSNP8466	AGAATATTCCTCAGAACACCCGGCAGAACGATACTCTGTACCACTGTACTCCTCATATCC[G/A]CTGATTTCAATCCTGCTATGGAGCCATTGTGTTCCTGAAGGAAAGTGTATCATGGAGATG
5467	CaTSNP8467	TCAATCGTATTAAGTACTTTACCCTTTCTTCTAATTTCTCAAAATTAATCACAACACATA[T/C]ATGGCTTTCTTTCTTTTCTTTTTCCTCTTCTTTCTTGCTTCATCAGCCACCGCTTGTGAT
5468	CaTSNP8468	TCACAGATCAAAACCAGGCCTGACTGAACGTTTTGAATTGTTTGTCAACAAACGTGAGCT[A/T]TGCAATGCTTATACTGAATTGAATGACCCTGTAGTACAACGGGAGAGATTTGCTAATCAA
5469	CaTSNP8469	GCTTCTAGTGATAACTTCATCAAATATCTCACCTTCTCCGATATGGGAAGCACCAGTTCC[A/G]AACTCTGATTGTGATGCAAGGTTGAATGGCAGAGCAGTTTGCTCTTCATAAGCTGCAGCA
5470	CaTSNP8470	ACAGTACACTACATCCCACTCTCCAAATATTAAGGGGCATTTCACAGGCTCTTTCACACA[A/G]TACTTCTGCAATTCCTGAACCACTTCAGCTACCTCTTTATGTTCTTCCTTTTGCAGTAAA
5471	CaTSNP8471	CATTGCCCCCTGGATATTTTGAGTGTATCATTCATTCAATCTGGGAATCAAGATGTTCAG[T/C]TGGTTGAGTGACTGCACTAAGCTGGGGAGCAGAATGTAACTTATATATGCCCTAGATTAG
5472	CaTSNP8472	CAAACCAACGAATGAATCTGTTGGCTCAGTTGCAAAATCAAGTACACAGCGATCCACAGG[A/G]GTAGTAGCGATGTCAGAATAATTGATTGCATCCACTGTGCGAAAAGCTGCAAAAAGTGGG
5473	CaTSNP8473	GGTTGTTAATGTTGCTTTGGTAGGAATGTTGGCACCTTATGCACGAATTGGAAAACCGTC[A/G]ATATCTAGGGGATTCCTTGGTAGTATGCAGAAAGCTTATGCAGCTTTGCCTAGCAGTGTA
5474	CaTSNP8474	TTCAACAGGTGAAACCGCTTTCGCTGGTGCCCTTTGAACTTCTTCAATAACTTCCTTTGA[T/C]TCAAACAAATTTTTGGCTTCTAAACGTTCCTTATTTTTGACTTTCTTGAAGTCCAAAACT
5475	CaTSNP8475	AATGGTCTGGAAAGCCGTAATGGTGTTGAGACAGCAGCAGAATCCGGATGATCTTCAATA[C/T]TCTGAATTTCAGTTTTCTGAATAGCTTTTTCTGCCACACCCTTGTCACTTGTTTCTGTTT
5476	CaTSNP8476	TCTGCACTAACTTTTCAGCCTCTAATCGAAGATTTCGGTTTGATTTCTCCAACATATCAC[A/G]ATGTTTTTCAGAATTCTCAAGTTTTTCAACTCCTTTCTCAACTTCATTTTTTAGTTGTTC
5477	CaTSNP8477	AAAGTGGGGTGCTTGGAACAGAATCATCAAAACTGAATCCACCGGTCTTCTGGAAGAGAT[C/T]ACCAGCCTGTGGTGAAGCGGTTTTAATGGGGTTGAGGCCCAGGTCCCCAGAACTAAAGAA
5478	CaTSNP8479	ATAGTCATCTGTATTTATATATCTTGGAGAAGCACCAAGGAGAATAAGCCTCTTGAAAAG[T/C]TGAGGTCTTTTAATGGAAGCTAAGCAACCAATCATACCAGACATAGAATGACCAACAAAG
5479	CaTSNP8480	TGACAAGTCATCTGGTGCTTCTGGAGCATCTCAATCTCCCCTGCTACAAGAAAAGAGCGC[G/A]AGTGTTAGAAAGGTTCATTCCAGCAGCACTAAGAGGGACTACATTTTTCGTCAGAAGTCG
5480	CaTSNP8481	CTCTTTATCCTTCTTCTTTTTCTTCTTCTTCTCTTTCTTCTCATCTTCGACCTCTGCATC[C/A]GCTTCTTCTACTTTCTTTGTCTTCACTTTTTCTTCATTTTCAACTTTTGCCTTCTTGGCA
5481	CaTSNP8482	AACAAGGATTCCGCCGATGCCGCCATCGCCGCTGGCCACGACCCTTCCTTTGGAGTCACC[A/G]TTAACTCCAAAAAGGTTCAAGTGTTGTGGGCAACGGATCCTCTGGCTATGTGGAGGGAAG
5482	CaTSNP8483	AGGACGTTATTTGTCACGTCTTCAACGTCAAGGTTACTATTTTCTTGACCTTTCAGCTCG[A/T]GGTCTTGGTGATCCTGAAACTACTCTCACCAAAGTTCACCCTGTTTGTCCTGCTCATTTA
5483	CaTSNP8484	ACTAGGGAAAGACGATGAAGTTGGACTCGCGTGGTAGGATGGTACTGGACTTGGAAACGA[T/C]GACGATTGTGGACTTGGCTGCATCGAAGAACAAGCACTCATATTTGGTGGAGTTCCTCCA
5484	CaTSNP8485	GACCCTCGAAGGGTCCGTGCCATATTCAATTTGGTTAGGGTTTGAGGTAAAAATGGTTGA[T/C]TCAATTGCTGCAACTTCACTACTTAGCCACCGTCCTGTTTGTGGTGGAAATCTGATTAGA
5485	CaTSNP8486	CAGCCGAAGTGGAAATCACAAGCTCAACAACAAATCTACTCATCCAAGCTACGCCAAGCC[C/T]TAACCGGAATCAACCCGCCACGCCGAGGTAAAGCCGTCCGCGAAGCCGCCGATAGAGCAC
5486	CaTSNP8487	TTTGGTGCCTCAGATATAATTGCTGTCGATATTCAGGATGAAAAGCTACAGAAAGCAAAG[G/A]CACTTGGTGCTACTCACACGATAAATTCGGCAAAGGAAGATCCCATTGAAAAGATACTTG

5487	CaTSNP8488	ATCTGATTTGAGAAGAGGACGATCGTCGCGAACGGTGTTGATGTTTCCGTTTGATTTCGG[T/C]GGTTTTGAATTGAGGTCTTTCAGTTCTGACTCCATCCCCTTCATCATAGTTCATAGATTG
5488	CaTSNP8489	GTGTTTGTCTTCCGTGTTTGAGGAGTATCAGCGGTTTGCTGGTGTTAAATCCAGGGCAAC[A/G]GATTTAACATTTATAGAGATGTTTGACATTATAAGTCTTAGGCAGTGCCTCTACAGCATC
5489	CaTSNP8490	CTCTTCCCATTGGATCTTTGCCAAATAAGATGCAGAAGACTTGGAGATTGACAAGTCTGC[G/A]TGCATGTAGACAATGGAAGCAACAAATAGAAAGAAAAACCCGGAAATCAACATCAATGGC
5490	CaTSNP8491	TCTCGAAAGTCATGATTGATGAGCATTCAGCAACGCTCTATAAACCATATCAATGAATTG[G/A]TTGTCCTGAACAAGCACCAGAAGAGCATCTCGGACCTTTTCTCTAGAAATAACTGGAACA
5491	CaTSNP8492	ATTGCAAGGCCAAGGACATTTGTTTTAGGAAAGATTCCAAATTCTGAAATATACAGAAAT[A/G]TTGAGCATTATCCAAATGCAAACCGTGTTTCTGGAATACTAATTCTAAAGATTGATGCAC
5492	CaTSNP8493	CATCACACTTAAGAAGCTCAGCAGTCGTATTAAGATGGAACCTAGACTTCTCATCCTTAA[G/C]GACGGAAGAGTCGATCTCCTCTCCTTTTGCAAATTCAACATTTCCAAGGTGCAAAACTGC
5493	CaTSNP8494	TGCTTTAGCTGAAGGAGATAAGATCACCTTGGAAACTGCAAAACTTTTGAGAGAGGATTA[T/C]CTTGCTCAAAATGCTTTTACGCCATATGACAAGTTCTGTCCCTTCTACAAATCTGTCTGG
5494	CaTSNP8495	ATCAAAGCCATCTCCGTACCGTCCACACTTAGTTGCTACAATATATTCACTTCTCGGAAC[A/G]TTCAAAGCTTTTAATGCCTTACCGAGAACTTTTTCCGACAGTGTTCCTCCATAGTACGGA
5495	CaTSNP8496	ACACTACACCTATTCCGACGTACAACTCACCTCTCGTAGAGTAGCCTCCGGTCTCCACAA[T/A]TTGGGAGTCCAACAAGGTGACGTCATCATGATCCTCCTCCCCAATTCCCCTGAATTTGTC
5496	CaTSNP8497	AATGGGTAAGCAGTTTTTGAACTGTGAAGCAAATACCCTTTGTGTTGGTGAAGGTTCATC[G/A]ATGGCTGTTAAAGCAATGAAACAGTTGGGTTTTTCAACTGTTAGTGGTGTTTACAGACAC
5497	CaTSNP8498	CTTTCTTTGGTTTGATCCTACAAAAGAGTTCCACAGATACTCCATTCTATGGAATATGTA[T/C]CAGATTGTGTTTTTTGTGGACGATGTTCCCATAAGAGTATTCAAGAACAGCAAGGATTTG
5498	CaTSNP8499	TTTCAATCTTCATTTGATTATAGCGAGCTTTATAAGAGACAGAACATAGTGCCGCTTCAG[C/T]CTCGAGCCATAGATTTTTATACAATAGAGTCTGAGGCTCTGTTGCTCCATCATCATCATC
5499	CaTSNP8500	ACAACCGGAGCAGCGATTAACGGCGCGTTTGGCTTCTGACGACGTCGTTTGAGGTTGAAC[T/C]TGCGTATCATCGTCGCTAGGGTGAGATCTCTTTGGGGAGCGTGATGACGTGGCGGATCTG
5500	CaTSNP8501	CAGTGTGGATTTAGTGCTCTTGCAGTTAGAGTTTTAAAGCAATATGATGTTCCAATAAGC[A/G]CTAGAAACATTTTGGAGGATCCTGAACTGAAAAATGCTGTAAAAGCATTCAGTCACTGGC
5501	CaTSNP8502	GGTATCTTTTGCAGGGTTTCGAAATTCGTTGCCATTTTCTGGGCTAGTTAGACAGCTTGA[A/G]CAAGAGATGGAAACTGTGATTAAGGTGCTGCAGCCTGGACCTTTGGGAATCATAGAGCAT
5502	CaTSNP8503	TAAAACAAGAACTAGCAGAAGCCGAAACTCGTAACTCTGACCTTAGCAAGGAGCTCCAAT[T/C]AATACGAGGTCAGCTTGCTACTGAGCAATCAAGGTGTTTCAAACTTGAGGTGGAGGTTGC
5503	CaTSNP8504	TCTGCAACAGAGGCGGTAGCATTTAAAACTGCCTCACTGTCGCCTGTTTTTGGAGACCCC[A/G]CATTAGGGGAAGGAAGACCCAAGATACGCTCATCCATTTGCATAGACCCTGCATTAGGAG
5504	CaTSNP8505	AGCTTCTCAAAGTTCAGGGGCACAACTAACACAAAATGCTGACACTGCCAAGCATGATGC[T/C]GAAGCTGTTCAAAACCCCAATGAAAGATGTGAAGGTGATGAATCTGAAGAGTCGGAAGCT
5505	CaTSNP8506	CAGACTTGTTGAAGCTGTAACCAAACCAAAGGCTAAACGGTAAAACTGGAAGTCATGACA[A/G]TATCTAGCAACTCTCCTGCATCTCCAAGAGATTCAGGCACTATTTAGAATTCACTTCCAT
5506	CaTSNP8507	ATGAAGTTACCAGCACTGGACATTCTCCGAGTACGATGACCACTCATGATAACCAGAAAC[C/A]TATTGGAATCAGAGGAACTGCCGAAGAAGTGGCTAGCTCCTCCCCTGGAAAAACTAAGAA
5507	CaTSNP8508	TGCAGCAGAAACAGCAACAGCACCACCACCAGAAGGAACTGAAGACAACTTTTCTCTTCC[A/G]CTAGCAATAAGCTCGGCAAAATCTTTTCCCTTAACTTCAGTTAGGAGAAGCTCAATCTTC
5508	CaTSNP8509	ATTCAAGAGGAGGAGGAAAGTTTCGGCCGAACCTTAGTTAAGGGGATTGCGAAATTTAAG[A/G]CAGCTGTTCAACATGTTCAGGGAAACATACTGAGTGGGGAGGTAGCATTTGAGTTGTGGG
5509	CaTSNP8510	TCCACGTCTGCGAATTGTACGTTACGGGGAAGTCCTTGAATTAAAATAGTTCTTCCGTCG[A/T]ATGGAGTTACAATGTCCCATTGAGAACGATCAATCCTATCCAACTTGTACAAACGACCCT
5510	CaTSNP8511	TCTAAGGTTCTGCGCTGAGCTACTTCATAAAAACGTGGTAGCACACGATAACGCTACGGT[G/A]CGCCACGGAGTAGAAGATTGGGAAAATGCACCTAGGCTTTTTCCACTTGGAAATTTCGAT
5511	CaTSNP8512	GAAAATGGAAAGACATGGATAAAAAATGGACTTCTGAAGATGAGCACGAGGAAAGCGTTA[A/C]TAGAATAATTAAGGAAACTGTGAGTAGAAGTTGTAATGTGACACATTCCCGACATAAGGA
5512	CaTSNP8513	TGCTGAGGTTTCCACAGGTGATGTAAATGTTGAGACAGTTAAGGATTTGAAGATAGACTC[G/C]ATAGAAACTGATGAGAAGGGTGTTGTTGCTCCTGAATCAATAACTGATGAGAAGGGTATT
5513	CaTSNP8514	ATGCTACATGCATCAACTTCTTAGTGGATTGGATCATTGTCATAGTCACGGTGTCCTCCA[C/T]CGCGACATAAAGGGTTCAAATCTTCTCATTGACAATAGTGGTGTTTTAAAGATTGCCGAT
5514	CaTSNP8515	GCAAAGATTGAAACTTTGAGAGTTGAGGTTGAGATTGAAGGGTTTATGGGAAATGGGTCT[T/A]TGAGGGAAGTTGGTGGTGGTGAGTGAAATGGAAGAATGGGTGAATGAGAGGTTGTAGATT
5515	CaTSNP8516	CCCTCCGACAGGTGACACCGCCGGTGTAGGCTGATTTGTGTTACCCGAAACCGGCGCAAC[C/T]GGTGAAGGATTGTCGTGGTAATTCTTTAACGCCTCCACGGTTAAAGGAATGTCATAAACC
5516	CaTSNP8517	ACCAGACATATCCCTTTTCGTGGCGAGGACGGTTTTCAACAGTGACCTGAGAGGGATTCT[T/A]GGATTGGTTAGTGTACCGTGTTGTATTATGCAGACGGCGAGGGATATGTCGGTTCCGGCG

5517	CaTSNP8518	GATGATCGGTCGAGTGGATTTCAACAAAAGTATGGATTTCTGGCAACAAGATAAATGGAA[T/C]GGATATTTCCCTGTCAAATGGCACATTATAAAAGATGTTCCAAATCCTCAATTACGGCAT
5518	CaTSNP8519	CAGTCAAAGTACCAGCGATCTTTGCATCTTGTCCCAAGAGTCACATGTTTGAACCTGGTG[T/C]CTTTAGTTTATTGGATAGGTTTGGAACTTTGGTTTATAAAGGAACCATTTTTGCTGTTGT
5519	CaTSNP8520	GGTGATTTCTTAGTGGCTGAAAATTCACCATTGCAGTTCGACAGTAGTGAGAGAGCTTCA[A/T]GTCCTAAACATAATATGCCTAACTTAAATTATAATGTTAATACACCGAGTATGGATGGTG
5520	CaTSNP8521	ATAAAAACATGCACAGCATGGCCAGTGGACTGAACAATGATACTGGGGAGTTTACCTCCA[C/T]GGAGAAAGGATTCGGATGAACCAACATCAACACTAGTTATATACCAAAGATAATCACTTG
5521	CaTSNP8522	TCCCGTTCCACCGCCAGCGGCTTACTCCGTTCCGCAATATCAGGCGCAACAGTTATTTGA[G/A]AGAGATGCACAGATAATAACGCCGGAGGCTCTTGAGAATGTGAAAGCAGCGATTGCGAGT
5522	CaTSNP8523	GAGACACCATTTTGGGTCACTGTCACATCTCATCTCGTAGCAAGATCCACAGCTCAAACC[G/A]TTGTTAAAAAGTGCAGTGCTGAGTGCAGCAGTGTTGGTTCCATAGCCTTGGCTGTACAAA
5523	CaTSNP8524	TTTCTCTCTCTACATTTCATTTCCATCATCTAAAATGTCCACCAGCACCGAAGCCGAACA[C/T]CACGAAGAGGAGGAAGTTCCCGCCGGTGACGACGAAGACACCGGAGCTCAAGTCGCTCCG
5524	CaTSNP8525	CCTTAGGGTCAGGGCTAAAGCTGCTGTTTCGCTATCCAAATGTGTCTCCAAGATGCAGGG[A/G]ATTGAGGAAATGGAAGAGTTTGGGATGGAAAAGTTGATTGAAGTTGCAGCTGATTTACTG
5525	CaTSNP8527	TGCTGATCAGGGTGGGGAGCTCTGTTCTTTGAGGTATGACTTGACAGTTCCATTTGCTAG[A/G]TATATGGCTATGAATGGTCTGACATCTTTAAAAAGGTACCAAATAGCTAAGGTCTACAGA
5526	CaTSNP8528	TACAATGTCCAACTCACTAAAGGGTAGACCTGTTCAGGGTCGCATATTTGATGGTAAAGA[G/A]GCACCACAGTTTGTTGCTCTTTTCCAACCTATGGTGACCCTCAAGGGTGGCTTGAGCTCT
5527	CaTSNP8529	TTCTCAATATCCAGATGCTGAACTTAGAACTGCTAAAAATGGACAATTTGTGAAGGTTTC[T/C]GGGGTGGTTACATGCGGTAATGTTCCTCTAGAGTCGTCGTTTCAAAAAGTTCCAAGATGT
5528	CaTSNP8530	AGCAGCATCAAAGGGGGCAACTGGTCCTAGAGCCAATGTATCAACAAGGACATTTCCAAA[C/T]AGTCCAGAAAAAAATGGCGACAAGACCATTTCCAAGAAATATCGCCTTCGAGAATGTTAAT
5529	CaTSNP8531	AGATGAAACGAACACGCCTCTTGAAACGGCGCCGTAAGATCCAATTGCAATTGGATCGAG[A/G]TAATAAGGGGAGGCAATCCCGTCGCCGCCGCCGATGGAAATAGAAATTACATCAACACCG
5530	CaTSNP8532	TTCTTGAATGCCACTGTTTTCCAATCGAAGATCGGTGACATTGCAGAAAAATCCTTGACC[A/T]CCCTCACAGAGACCTCCAAGATCAACTCCATCAGGTATAGGTTTATCAAATCTTACACCA
5531	CaTSNP8533	AAGAATTTGATGAAGCCTAAAAAGCGCAAAGAAATCATCATTCCCGTAAAACACCCTTGA[G/A]TCCTTCACATCTTCAACAAATGAAACTGCTGAAACATGCTTTGTGAGAGGTTTCACAGTT
5532	CaTSNP8534	CGCCGATCCCAAGCCGGAAGCCATCCGCTTCGCTACTATTCCTAACGTCATCCCATCGGA[G/T]CGCGAGAAAGCAGCCGATTTTCTCGGTTTTTATGAAGCCGTCATGACGAAGTTGGAAGAG
5533	CaTSNP8535	CATCTTATCTGTTGTCCACGGTCTCCTTGCTACCCTTTCTCCTAAGATGCATTCGAAACC[G/C]TCAACTATGTCAGAGAATGCAACAGTTGGAGCAACAAATGTTGGGAGTGAGGATTGTGCT
5534	CaTSNP8536	AGCTACATGTTCTTTAGCTGAAGCTATTAACTCTTTCATTTCACTTCTTTTTGTTGTGTA[T/C]GTAACATCATAAGCATCAATTTCAGGGTGTTTTTCTTCTATGTATGCAGCGTAGTAACGG
5535	CaTSNP8537	GAGACAGAAAGTGGATTTTTCCCAGCGAGATTTGCAAATTGAAGCATCGTAACCAAGAGC[C/T]AATAATCCATCAGTAACAATCTTTCTGCAATTATTATCTTTGCGTTTACACAATGTTTTG
5536	CaTSNP8538	TCATCATGGGCATCTTGTTCTTTTCTTGGGGAACAAGAATACTGCTCCTCTTATGTCTGT[C/T]CAAGCTTTAATATTGCCTCCCACCCATTTGAAGATAGTGCTTTCTCTAGTACCGGATACT
5537	CaTSNP8539	CTTGGCTCGATACTCTTCATTGGTTAGATCAGCGAACCGGGTCAGACCCAACCTATAGGT[T/C]GAATTCAGAACATTATGTTCATCGATGAACTTAAGGTTGTCTTTGAAGATGTCAAACCTC
5538	CaTSNP8540	CGTATATTTCCCTTCAACAGATATTCTGCGTCTATTTAGAGTACTGGTGCTAGTCTCCAA[C/T]TACATAATCCATGTTAATTGGACAAAATATTGACACTACTCAACTTGCTTGCTTTCAGTG
5539	CaTSNP8541	AACCCCTCTCTTAAACTCTTTATCATCGTTTTTCCTTTCGTGATCAGAACAGTTTCGTGT[T/C]GAACGAAATTGTTCGAGTTTTCGATGAAGCCTTTCGCGTAATTCTTCGTAAGTAACACTT
5540	CaTSNP8542	CCACTCCTTTTGATAGAATCCTCCAGGTGCTATAAGTGAAGGTTTGAGCTTGGTGTTCTT[G/A]TATAGTGTGTGTAAATTTTGTTTAAGCTTTATCAAGTCCTTCCCATATTGTGCAGCACCA
5541	CaTSNP8543	AAAGTGCCAATCATGATCATAGATGTGTCGATGAAAAGCTTTTAGTATTGGAACCTTGAC[A/G]TCTGTATCTATGCTAGTATCATCCTCAACGGTATCGAGAGCACGAAGAACCAAATAGAAT
5542	CaTSNP8545	GTCCGAGATGCAGGCTCTAAGGGCAAACAGCATTTCGACGCCTCAACCGAATCCGCAAGC[A/G]TTGCCTGGTGCGAGTGTTGCCACTGGACCTTCACTTCCTCAACACCTGGCTGTGCATCCC
5543	CaTSNP8546	AGCCTTCCATTCACAATCTGGTGGTGTACCACAACATAATCTTCTATCATCTATGTGGTC[A/C]ACATCAAGTCCAATAAACCAAGCTCCTAATGACACATCCTCATTTGCATACTTGTGAAGA
5544	CaTSNP8548	AAATCCAGGATCAATAACATAGACTATACCATCAATTGTCAAGGAAGTTTCTGCTATGTT[C/T]GTTGATACCACAATCTTCCTTCCGGCGGGGCCTCCCTCTTTTACTGGAGGTGGAGCTGGC
5545	CaTSNP8549	TATGATCAGGAGTTGAATCCTTGGGGCAACTGAAACTATCTGGGACAGTTTTGGAGATAA[T/C]CTTAATTGTGGTTATATTGAATCCAAGGTAACACAAGTAGTTCAAGTAGTCAACGGTGGT
5546	CaTSNP8550	GGCAGAGAGAGCTTGGAGTCATGCCATGGAGAAAAGGCAGCTTCCGAATGGACCAAATGC[T/A]TCTCAGCGTATCTATCTGATTGGTAGGTTGCGGAAGGCAGTAAAATGGGCTACTTTGTTT

5547	CaTSNP8551	GAACGAAATTCAACTAAGATAAGCAGGGATCTTGATGCGAATGAATAGCATACTCATAAC[A/G]TAAGAAAGAACAATCGACGAAACACCAGCAGAAGCATAAGAAACCTTAACAGATTTATCA
5548	CaTSNP8552	TACAGAAATGCACTTCTCAGTTGCAGAAGCGCAATGAGTTAAATGTTTTGAATGTGTTAT[T/C]CTATGTCCTTCTATGTGCATAAATAAAATGTCGTCATGGTGTTAGCTTGTGTATATGGTC
5549	CaTSNP8553	ACCACGAGAAATACGCGTACGGGGCTCCACCACAAGAAAACATGAAGCAACCTTTCCTTC[T/C]GTAGAGAAATGCTCCTCCAGGTCTTTCTCAGTGACTCTTGAAGATAGGCCAGTCACATAG
5550	CaTSNP8554	GTTACAAGGTAAATGCTGAGTATGGTGGAATTAAAAACACTACTCAACCTCCTCGCCAGA[C/T]AAATTCTGCCACACAGGATCAATCTCAACAACCGGGGTCCTCTAACATGCTTGCTCTTCC
5551	CaTSNP8555	GACCCATTTCTCCATCTTGTGAAAAACAATGGCTGCTTCTTCTTCATCAACCTCTGTGTT[G/C]CTTCTGTTTCTTCTCTTTGGCTTTGCTGCTTCAGCTAAAGATATCTTGCTTGGAGGAAAG
5552	CaTSNP8556	AAGAAAATGGATCCAAACTTTGAGACACCAAACAAGCCTCCACCTACTTTACTCAATGCT[T/C]TAACTTGGGCTGCTCATATGGGTTTAAGTAGTAATCTTAGGTACCAAACTCTAAATGGTG
5553	CaTSNP8557	TCATCCTACCTGAAAGGAACTTAAAGGACTTGGTTGAAGTACCATCATCGGTGCTGGCCA[G/A]TTTGGAGATACTACCTGCAAAACGAATGGAGGATGTGTTAGAGCATGCTTTTGACAGTGG
5554	CaTSNP8558	GTCGGTTACTTCGCCTGATAAAATTAAAGGCAGACACGACAGTGCCATCGGTAACTTCGG[T/C]ATACCTCAATACGGAGGTAGCATGGCCGGTACCGTCGTTTATCCAAAAGATAACAATAAG
5555	CaTSNP8559	TGGTGATGAGATAAGAAAGATATGGCTGAAATCTTGCAGGCAGAGCTTTGTGAAAATGCT[C/A]GCAGATAAGCAGCGCCGTGAGACAGAGGAGATTAAAGCAAAGGCTCAAATTTCTAATGCA
5556	CaTSNP8560	TGCCTTGAGTAGTCCAATAGAATTCCATCAAATTCAACCATCATTGATTTGGATCTCTCG[T/C]CGTCACTCAATAAATCACGTAAATGATTCGCTTTAATGTCCTCAACGTGCGCCTTCAACT
5557	CaTSNP8561	ACAGGAATCCTATATCAGAGAGGCTATTGGTTCTCCGCTGCTTCAATATTCCTTGAAGCC[T/C]GTGCATGCAGTTATCATTGGTGAAGAGACGTTCCATGGGATTTCCAGCATGTCCTTTGCT
5558	CaTSNP8562	GATCATCCCGTTTTCCGCGAACTCGTCGAAAGGTCACGAGAAACAGAACAACAAAACGAC[A/G]CCGTCAATGTCGTCGCATGTGAGGTTGTACTTTTCGAGCATTTGCTTTGGATGCTTGAAA
5559	CaTSNP8563	TCACCCTGCCCTAATCCCCTTTCAATTCCCTGAACCCGGTCCACCTCCTCCTCCGGCCTA[C/T]GGCTTCCACATGCTCGAACGGCGAACCATCATCCTCGCCGACGGCAGCGTCCGATCGTAT
5560	CaTSNP8564	GAGAAGTGTAGATCCTTCGAAACAGCATCCTGGTTCTTTACATCAATTCCAGCAACCGCT[A/G]AACTTTGCAAACCGGACCGCTGCTTTAATGCAGGCGCAGATGTTGCAACATTCACAACAA
5561	CaTSNP8565	CAGGCAAGGTCTGTTTTACTGACGTACCTTTTCTGTGCTTCAACTTATCCACTTCTTCTT[C/G]TTGTTGTTGTTCCACGTAGACGCCGTTGCCGTTGAAATTTTCTTTCTCCGCCATTTTCAG
5562	CaTSNP8566	GGTGAATACAGAAACATAAAGAGGCCCTTTCCGCTCTATGGTCCACGAAATTATACAATA[C/T]GATAGCCCAGTGCAAATTACTCCCGCATAAAGAGAAGAGGTAAGTCTCATTGCATCATGG
5563	CaTSNP8567	GGGAAGAAGACGATCCAAGATTTTCCAACACTGTATGACAAGTGTCAGTGGTAACAGGAG[G/C]CTTCTTCTCTATCGCGAGAATCTCAAAAACTCTCAGTGCCTCATACGCATCTTCCAAACT
5564	CaTSNP8568	CCTCCGGTGGTTGTTCAAACTTTTCTTCATCCTTATTCTGAACAACTTCAGACCCCTCAG[T/C]TCCTTTGTCATCAACTTTCTCTTTTGATGCTTGTAACTCTCCATCAGCAGTGTTGGATTT
5565	CaTSNP8569	CACCATCATGGCGAGACGACCGGAGATGATTTCGAAATCGTGGCTTTTCTTAGAGCTTTC[G/A]ATATATTCGGAAAGTGTGGCGAAAAAGGATGACACGTCATCGTTAACTTGCATGTCTTCT
5566	CaTSNP8570	TCCAGTAAATCTTCCAATACCAGCACCAAGCTCAATAACTGATTTTCCTTCATATGCTGG[T/C]AGAAGGGAAAGTACCTCGGGTCTTTCTTCTTTGTCTAGATCGGAAGCTTTGGAGTCGAGC
5567	CaTSNP8571	GGTTAGCCGTTGTGTGCGGAAGTCACAGGAAATGTTGTGATTCGCAGCCGGCTCCTCATA[T/C]CTTGCTTTTGTGAAGTGAAAGACATACAACTTAATGTAAGGATTGGTTCGCCTGCTACTG
5568	CaTSNP8572	TATACTTCACCTCCTTACATTCCACACCCAGTTTATCACTCACCTCCACCACCAGTTCAT[A/T]CTTCTCCCCCATACATTCCACATCCAGTTTACCACTCTCCACCACCAACTCCAACCAAGA
5569	CaTSNP8573	GATCCTTGGAGAAGAGTACAGACAGAACCTGAGGTGGAACGGCGATGACGACGTAGATGA[A/C]GAAGACGGTAATGGAAGAGTGATTCCTCCGCATGAATTTCTTGCTAGAACGAGAATTGCT
5570	CaTSNP8574	TCCGACCCTGTTTTCCTTGGCCTCTACTAAACATCTTTTGTAAGGTTTAAACCCTGTTCG[G/A]CGAGTCTTAAGCTTACCTTGTCCAAGACTTATGGTTAGCAGTCCCTCCTCCTCATTGTTA
5571	CaTSNP8575	TCCGGATCAATTCAAAATATACTATGTGTTAAATCAGCCTCCGGAAGAATGGAATGGTGG[T/C]GTCGGATTTGTATCAAAGGAGATGATTGAAACTCATTGCCCTCCTCCAGCACAAGATGTT
5572	CaTSNP8576	TATCTCCAAGGAGGAATCAGAGATCCAAAGGCTTCAAGGTGAAGCATGCTCTACAAATAA[T/C]TGTTCTTGTTGGTGTTTGTATTTGGTTGGTTTATCAAATCGGACACTCTCGGCAAAAGAA
5573	CaTSNP8577	TGCAAGCAGATAATTGGGCATTTCCCCATCCATTACAAATCTCAAAGTTGGAGGCACCTT[T/A]AGCAAAAGCTTCCCTGCTCCCAACACAAAACCGGCATCAGTATTTCCGATTCCTGTTGCA
5574	CaTSNP8578	CGTCTACGGTGACATCACCGACTCTTCCTCTCTCCTCTCCGCTTTCTCCGACTGCTCCGT[T/C]GTCTTCCACGTCGCCGCTCTCGTTGAACCCTGGCTCCCCGATCCCTCTAAATTCATCACC
5575	CaTSNP8579	ACAACTACAAGTGTTGACACATGAAAGTCTTGGTTGTTTTGTGACACATTGTGGTTGGAA[C/T]TCAACATTGGAAGCTTTGAGTTTAGGAGTTGTAGTGATTGCTATGCCACTTTGGACTGAT
5576	CaTSNP8580	AGAAACTACCATTGCACAATTTATCAAGCATTTTGGTAAATATGGTGAGATTACCGATTC[C/T]GTCATCATGAAGGATAGGAAAACTGGGCAGCCTCGTGGATTCGGGTTTATTACTTATGCA

5577	CaTSNP8581	GAAAAGTAGAGTAGCTGCTGGTGGTACTTCAAGTAGAGCATTTTGTCAGGTCGAGGATTG[T/C]CGTGCGGATCTCAACAATGCTAAAGATTATCACAGACGTCATAAAGTTTGTGAGATTCAC
5578	CaTSNP8582	TTTTGTAGTTGAGCAACCAACACAACCAGTACAAGGAATCCAAGCAACATCATTACTAGT[A/G]TCCATAGCCATAAGCAAAGTTTGAGGTGGATTACCAATTTTAGCCTTCAAAATATATGTT
5579	CaTSNP8583	TTATCTAAATGGGTTGGGCATTCAGTGGTCCAATGTGCGCAATGTCATGGACATGAGATC[A/T]GTCTATGGAGGGTTTGCAGCAGCTTTGAAGGATTTGAATATTTGGGTGATGAATGTGGTT
5580	CaTSNP8584	AATATCCAGTCCAGTGGGAATCTGCCACACAAAGAGCAAAACATTCACCGTATTCAACGC[A/G]ATGTGGAGATTTCTGGCTGTTTCACTCCCTTTCTGCATTGGTGGTACTAGGGCAGCTGCC
5581	CaTSNP8585	TATCTAACAAAAATAAAAATGTAGTTCTCCTTTGATGTTTATAAAGGTTTTAGTACCAAA[G/C]TAACATTAATATTCCCAAACTGAAGTTCAGAATTGTATCACATTGATTTCCATTTGCAAA
5582	CaTSNP8586	TCGCCGTGGGGCGCGTGGCCTCGACCCCTCGGTAATCGAGACTTTTCCAACATTCGAATA[C/T]TCTGTGGTTAAGATTCACAAAATTGGTAAAGGTGCACTAGAATGCGCCGTGTGTTTAAAC
5583	CaTSNP8587	CCGATACGATTGGATGTGTAACGCGCTGGAGGAGTTTATGCGAGTTATGACAGACGACGG[T/G]AAGCTTGAGAAGCAAGACGAAAATGTCGATCCAGTTGATGTCTCAGAATGTCAAAGTCTT
5584	CaTSNP8588	TCCACCATATCAGAGCGTTTGAACGATAAAATCCAAATGCCCATCGCAACAAAACCAGCA[C/T]TATCATGTGTCATTAGAACAACTCTGAAAATATCAGAGTATATAACTGAGGACACTTGCA
5585	CaTSNP8589	ACAAGCTTTCCAGAAGCAACATTCTTCTTCAAATTGTGAAGCAATAGCTTCTTGAAATTC[G/A]GAGGAAGCTGTTTATATTTCTCTTCGATGAATTTTGCAATAGCGTATTGGCTTGAACCTG
5586	CaTSNP8590	GTATCCATCAAAGGCGGAAGTTGAAGGCCAGACATCGGCGACAGCGGCGGAGTCCTTGTG[C/A]GTCCGCGGAAGAAGCCTCTTGTAAGATTGAATGTAGAGAAACAGAACGAGATCATGAACA
5587	CaTSNP8591	AGGGGAATTCAAGGAAGTTATTTTCCAGGCCACACGAGTAGCTTACAGTGCTACTGGCTT[A/T]GTCACTCTGTTAGCTGGCCAACCCAAAGGAGGAGTTTCTGTTGAAGCCCGGTCAGTGTCA
5588	CaTSNP8592	CCTGGGTGCGCTGTACGTATCTGTTGGTGGCATGCTACTGTTTGTCTTTACGTTTGCTCT[C/T]GGAGCTGGCCCAGTACCAGGTCTCCTTCTAACAGAAATCTTTCCTGGTCGAATCAGAGCC
5589	CaTSNP8593	GCTGGATTTGTTGGTGAGTATTTTGTTGCTGTAGAAGCTGAGATTGCAAAAGTGGGCTAG[A/G]CGTCGAAAGAAGTTGTTGCTGCTGCTGCAGTTTATGTAGAAACTGCATGTGCAGTTGTTG
5590	CaTSNP8594	GTCCTTGGAAAAAGGGTACTTGAAATGGTGCATGAGGTCTTTGAAATTGCAATGGATTTG[A/G]ATTTGGATTTGGATTTGAATTTGAGTTTTGTGTGAGATCTAATGTAACAGTAGGGAATGG
5591	CaTSNP8595	TGCATTCCATTCTAGCCATCCACGTGGATGTATGTGATCTCCCATGTATGATAACATGTA[C/T]ACAGTTTTAGAGTATAGTTTCCATGGACGGCCCAGATACGTTTGGAAGCTACCCTTGGAT
5592	CaTSNP8597	CATTGGAAGGTCCATTAATTCTAGAGGTGAGGCTTGACCATCCAACTGATCTTTTTCTGA[T/A]ACCAAGAAACCGTGAAGAACTCAAGTTTGAGGAAGAAGAAATTTGTGACTTGGAAGAATT
5593	CaTSNP8598	CCCATAATGGAATGACCCTTGTGGGTTAGGCCTAGCAGCATTAGCTGTCAAATTCCACCT[G/A]TAAGTTATAGCTTGTTTCATAGACCAGTCGTATTTGTCCACAGGGGGAGCAGGCAAGGGT
5594	CaTSNP8599	TTCCTTAAATTGACTCCTAGCTTTTACCGTAATCTCAATCCGACTTTTTGAATGCTTTTC[G/A]ACTTGAGCTTCCACCCAAAATAAAGGCTTAACCTGTGTGCTGAGCCTATATGTCATCAAA
5595	CaTSNP8600	TCTGAATTGGTTATATCTGGCTACATTCCTCTCTCTATCTCTATAAATTTCAAGAGCAGC[T/C]AAGTCCACATGATCAGATCTTTCTGTGCCATCTACGTTATGTGGTACTAAGTCTCTGAGC
5596	CaTSNP8601	CTTGATTGAGTTGATTGAGGATTATGGTTCTGATGCATTTCCTTTCACAAGAAAGAGACA[C/T]GAGGAATTGAAAGCCATAGATAAAAGAAAACGTGAAGAAGTAAATTTGGAAGAATTATTG
5597	CaTSNP8602	CTCGAGGGATTTTTCATGGTTGAGAATGTTAGGAAGGCTATAAGGATTGATGAACATGAC[T/C]CCGACAGCCTTACCACTTCTATGGTGGATGATGTGTTCTATGTTTTGCAGAGTTGCTTGC
5598	CaTSNP8603	AAGGTGAAAGAAGAAAACATGGGGCCAGTTTCTTCTGTGGCAGAAAACCCTGTAGTAATG[A/C]AGTCATCACATTATATGGAAGAGAATGATGCCGTGTTTAAAGCATCTGTTCCATCGCATG
5599	CaTSNP8604	GCAACTCTCATCCTCTTCCTCATTGAATAGCTCTATGAGGTCATCTTCCAGTTGCGTTAG[C/T]CAACCACCTCTGGCCATTGTCACAGAGGTCTCAACTTCAGAAAGTCGTGTTCAATCAGCT
5600	CaTSNP8605	TGAAGAGCAACAAACCCAGAGTTTGATTGATCAACGGATCAAAGAACATCTTGGGCAAGC[T/C]GCGGCTTTTCAGCAGGTTGGTGTGGCTTTTAATCATTTAATGGTTCAAAGGCCAAACCTT
5601	CaTSNP8606	TTCCCTGGCAGATGGTTTTCCTCAGGCTTGTTGAAAGGGTAATTCATAGTCTTTCCAGGT[G/A]TTTTCTTCAATGTTTTTCTCTTATGCCTCCTGAAGTGGGCCAGCTTCAATACAGTATCTT
5602	CaTSNP8607	AAATGTTTGGTAATTCAAGATGAAGAAAAGCTTGGATCCTTTCATAGACTTTATACTATG[T/A]CTCCTGTTGCCAAATTCTTGGTTCCTAATTCTGATGGTGTTTCATTAGGACCCTTGTTGT
5603	CaTSNP8608	GAGACAATTTTAAATGGTGAGGGCTTTTGACCTTTTGGAACCTCGCTCGTGTCATTGCCA[A/G]ACCATGCTGCAAGAATACCAACACCTGGTGCTGCAATATCAGGCTTGAGAATATTCTTTG
5604	CaTSNP8609	TGATTGTAATAGACGGAGCTTCAAGTCTCGAAGCTCTTTTCCGTGAAAATTCCACTAAGA[A/T]TGGTCAACCACCTGCTTCAAAGGAATCAATTGAAGCTATGAAAAGGGTTGAGATTGAAGA
5605	CaTSNP8610	AATAATAAGGTCTTGAAGATCGAAGGATTTGGCTTTGGAAGGAAGGTTACCGGTACGGAG[G/A]AGAGAGAGGAGGAGAGAGAAAAGTTCAGGGTCTCGGTCAACGAAAGGTGTGTAAGGTGAA
5606	CaTSNP8611	CCAGAGTTTGATCAATGCAGATGGTGTCATTGAGTCTTGTTTCACTCCTGGTCGATATGG[A/C]ATGGAGATTAGTTCTGCTGCTTACAAAAGCTTTTGGCGTATTGACAAAGATAGTCTCCCT

5607	CaTSNP8612	CATCTCATTTTTGCCATATTATTGGCGTGCTATGCAATGTGCAAGACGGTGGTTTGATGA[C/T]GGCGATGTGAACCATTTGGCTAACATGGGAAAGTATGTTTCGGCGATGATCGCAGCAGGG
5608	CaTSNP8613	AGAAACCCTCACCTTGCCAAGCATTGGTATAAATCCACAATTCATCACTTTCACGCATGT[G/C]ACGATGGAATCTGATAAGTATATATGCGTTCGAGAAACGGCTCCTCAGAATAGTGTGGTT
5609	CaTSNP8614	CAATGGAGGAAAGTGGACTCTCACCATCAGTAGAAAGCCCACTCTTGATGCCATGTGGCT[A/T]GAAACTTTGATGGCTTTAATTGGGGAGCAATTTGGAGACTCCGAGGACATTTGTGGTGTT
5610	CaTSNP8615	CCAGATCTAGGTGAAGAGACATAAAGGTTTGTGAAATTTGTCATAGAATTTGTAACAAAA[T/C]TATTGGCACGATAGTTAGCAAGATCCATGTTTGTTAAGGAAGCTAAACCGTGGTCTTGTT
5611	CaTSNP8616	CCCGTTGTTCCGGAAGAAAACGGAAGAGCCACCGCGTCTTCCGGATCGATCTCCACTTCC[G/A]GCAATTCCTCTTCATTTCCTTCGGAAATCACCGAGAAATGGAGACAATTCACCGGCGGTT
5612	CaTSNP8617	AGAGGAATGGAAATGGTGGGTCAAATGAGGGTGGATCCCGGAACAATCAGAGCTCCAAAG[C/G]TGACAGTTTTAGTTCTCATAATGATGCAAATGGGTCACATGTTGCTGGAAATGTTGGTTC
5613	CaTSNP8618	CGAAGAAACTCGCCACAACGGCGTCGTTTGAAACGCCACTTCCTGCGGTTAGGGTTCTTC[A/C]ACGATTCTACCACGAAAGGGTGGTTGACCACTACGATAATCCCCGGAATGTTGGATCCTT
5614	CaTSNP8619	GCGGGAAATGCAAACCTTGATTGAAGTTGGGGCCTGAGAGTTATAGTGGATGTAGAAAAT[A/G]GAGTAGTAGTAGCCATGGAAACCGCCATGTGCGAGATTCAGATAATGTTTTGTAAGGGAA
5615	CaTSNP8620	GCTTTGTCGGATTCATGCAGAGTATAGTGCAGCAAAAGTACCTCATGGAAACGTGAAATC[A/G]TCTAATGTGCTACTTGACAAGAATGGCAATGCTTGCATATCTGACTTTGGCTTGTCACTG
5616	CaTSNP8621	CGAGGAACTAAACAAAGATGTAGTACAAAAGGAAATTGATATTTTGGCAAACTCGTCGAC[A/G]AGTATTAAGAGCATGATAGAGCAATTGAAACTGGAGGTTGCCAGGGCTGGAGGGAAACCA
5617	CaTSNP8622	AGCTGATAGAATCAAGGCTGCAGCATTGAGTGCTGCCAAAGGTCTCAGTCGTGCTCAGGC[C/T]GAAAGGGCAGCTACTGCTGCTGCCCGAAATGTCAATGCTTATGGTCAGAAGGAAGAAGGT
5618	CaTSNP8623	CTTGATTTTTCTGGACATGAGAAACATTGGAGTACTTGCTCTGATAGTTGTTACATCGTA[G/C]GTTTCAACATGGTAAATGACTTCCTCAAGTGCATCTATACCTTCCTGAACTGTGAAATGA
5619	CaTSNP8624	GCTGCCACTGCGGTATAAGTCCCCATAGGATCCTTCATAACCACCAGCCGAGGCATTAAA[T/C]GCAGAGGATGGATTCACAACTGCATTGTTGTTTCTTCCATACCCTGCTGCTCCACCTAAA
5620	CaTSNP8625	AGTTGTAGTTGTGGTAGCGTAGATCGCTTTACCTGGAATGGCGTTTAGGTCATTATCAGA[T/G]GAAGATGAAACCGAAGGGTTTTGAGGTTGAGGGTCAAAATTGGAAAGCATTGTTTTAAGC
5621	CaTSNP8626	ATCATCATCTGGTTCAACAAATCGATTTTCAGATGAAGCATATGAGGATGATGCCGATGA[C/T]TGGTTGAAAGAAGAGGATACTTCTGAAATGGTTGGTCCCAGTGGAACTTCCATTCATACT
5622	CaTSNP8627	TTCCATTTTTATTACTGCAACTAACTTTTGTCCTCCTAATTTTGCTCTTCCAAATGATAA[C/T]GGTGGATGGTGTAACCCTCCTCGTCCTCACTTTGATCTTGCCATGCCCATGTTTCTTAAA
5623	CaTSNP8628	CTGCAAGAGATGCAGCTGTGAATGAGACAGATTTGCCACCAGCAAAAACTTTGTAAAGCT[T/G]ATCAACATCCTCTAATGTGGTTGTTTCATCAAAAGCAACAGTGATAGTGTTTCCATCTAC
5624	CaTSNP8629	TATATAATGTTTTGCAACTTCGTCTTTCTTGGAATGACTGTGAAGTGCTTCTGCACGAAC[G/A]CGTAGCTCATCATTGCAACGCCACATAGAAAGTTCAAACATAAGATCTTCAATCTGTGAA
5625	CaTSNP8630	CTCTCTGAGGACCAACAGAGTTATGCACATTCAACCTGTCAAGAACAGAGTTGAAATCTA[T/C]AGTCTGTCCACTCTCCTCCATCCTTTGGATCACACTCGCCACATGGTCACCCCTGAATCC
5626	CaTSNP8631	TTGGTTGTGAAGGTTTCTCCAGAGTCAGCCAGTGAATTTGGAGAATTGGTAGCTGATCCA[C/G]TCACCAATGAACTCTTGCATTACACAGAAAAACCTGAAACTTTTGTTAGTGATCTTATAA
5627	CaTSNP8632	GTAATATGAGAAGAATTTGTTCACTGTGTCACAATATGATTTGTCTGCAGACCAGGGAAT[T/C]GATCGAACAAGAACAGTAAAATGACTTGGACTTGGAGGAGATCCAACAATATGTAGAAGC
5628	CaTSNP8633	TACAGCAAAACTTAAAATTGCTCAGGGGGTTGTTGGAGCTGTTAAAGATAAGGGTTCCGT[C/G]TTGAAGTTCGTTCCATACACCATGCAAGCTGTCAGGCAAGGGTTTCAAGATATTGGTGCC
5629	CaTSNP8634	CCTAGTCTATTGGTTACCTTGGTATTAGAAATTTTCTGATGATTTATGCTTGAATGTAAT[C/G]TTAAGCTAGCTTTTCTAATAGTTTGCCATAAGGAAAGTAAGCATCATTTACTGAAATTGG
5630	CaTSNP8635	GATGTTAATTAGAAAACCCTTCGGACCAAGTGCATTAATGACCTCCCGGTTGATGATGTG[A/G]TGGGTTTCCTCGGTAAGAGGACATGCAACAACTAGTATATCGCAGTTGGATGCTAACTCA
5631	CaTSNP8636	CCAGCAGGGAATATTTATTTCAAGCACAGGAGAGCAGACCCATTCAATGAGTGGAAATGG[C/T]GCAATGGCATTTGATGTAGAATACGCACGATGGTTAGAAGAGCATAATAGGCAAACCAAT
5632	CaTSNP8637	CAAAATCTCCACTCCATGGCATCCAACCCTGTGGTGTGATAAGGGCTTCCAAGAAAGAAT[G/T]AATGAAAATTGTTCTAGAATACTCCTTCCATGGCCTTCCTAGATAGTTCTTGTGTACTTT
5633	CaTSNP8638	AATTATTCTCACCAAACCAAAAGCACTAAATGAATTGTTGAATTTAGGGAGTGGAGGAAT[G/T]ATTGGAATTGAGGTGGCATTGTTTCCACCCACGATGTCTTGCCAATAAAACCTAAAATGG
5634	CaTSNP8639	AGTACGAAACGTTTCCTGTAAAAGATAATCAGTAACATCAGGAGCAAGATCTCCGACGAA[A/G]ATAGAGTGATCGGGACCGGCATCAGGACGGCGTTCACCGATACCGAATGAAGCCCAATTT
5635	CaTSNP8640	GCTGGTTCACTTAACTGTCTCACCAATTCAAGTGTATCCTGGTGAAGGTGCGACTCTTTA[T/A]ATATAATTGAAAAATCTATTTTAAGTTCATCTTCTACGGCTGCAGAGGTAGCTTCCAACA
5636	CaTSNP8641	TTTGCCTTATGACCCAAAATTGGTGAATTTAGCCAACAAACCAGAATGGTTCCTTGAAAT[A/C]AGTCCTGAAGGTAAAGTTCCTGTAATAAAGTTTGATGGGAAATGGGTTCCGGATTCAGAT

5637	CaTSNP8642	AGATGTTGATAATTGGCCTCTTGATGCAGCTATATGTAAAGCAGTGTTTCCTTCATTATC[A/T]GTTGAGTTGATTATGTCAAAGGATGATGCTAGATATTTAACTACCTCAACTTTCCCTCTT
5638	CaTSNP8643	GTCACCCAACTGCATACTTGCCTGAGCCCTTCTATAGAGGGCTTTCACATTAGTGCTCTC[A/G]ATGTCCAACACCTTAGTACACAATTTCTCTGCATCTTTGTACTCTTTCAACTTCAAATTG
5639	CaTSNP8644	CGGTGGTTGAGCAGTTGTCAACCATGAAGGAAACACCACGAGCATGCATGAATGGATTCA[A/G]AGAATCAACTGACTCAATCACACTCTCATTAATGATTTCGGAGTAAGAGTGGCCTTTCTT
5640	CaTSNP8645	GATAGAGGAGTACTACTGAACACACTTACGCATCATATATAGCATAATGCAATAAGCAGG[A/G]ACAATCAACTGAGTAATTATTTTATTCGTATGCGTGTTCCTGTTCCTCTTCCTCGTCATC
5641	CaTSNP8646	TCCCGGAGAAAAAGGCTTGATACCTCGTTCGCTAGAACAAATATTTCAAACACGGCAGTC[A/T]CAGCAACCGCAAGGATGGAAATATGAAATGCAGGTGTCAATGTTGGAAATATACAACGAA
5642	CaTSNP8647	CCGGCGGAGAGAAGGATATTGAGGATGGGTGCATGGCCGTGCTTTGCAGCGTGCATGAGT[A/G]GAGTGAGACCATCGCCGTCGAAATGAGTAACGTCGGCGCCGGATTCAATCAGAGCCGTTA
5643	CaTSNP8648	CGCCGACGAAGGTCGCCGGAGAAGAGAAGACAACATGGTGGAGATCCGTAAAAGTAAGCG[C/T]GAAGAGAGTCTCTTGAAGAAGCGACGTGAAGGTCTTCAAGCTCAACCGCAGTTCCCTACT
5644	CaTSNP8649	GCTGGGTAAAAAGCATACATATTCTCTTAAAGAAGAAGAAAATACCAAGAAAATAAGGGA[A/G]CTTGGCCCATTAACATCAGATGCTTTTGAGGCGTACAAACGGAGGAATATTAAAGATTTG
5645	CaTSNP8650	TAATCAAGGTGTTGATGAGAGGGATCAAATTAATGAAATGGTAGAAAAAGATCAAGGGGT[A/G]ACTGATTATTTTGTCTATGGTGCCAATTTGACATTTCTTGACTTAGAAGATATCAATTTG
5646	CaTSNP8651	TGTGAAAAGTTGCGTGGAGAGGCTGCTGTGCTCCCTGGCTTAAGATCAGAACTAGAAGCA[T/C]TGAGGAGGAGGCACTCTGCTGCTTTGGAGTTGATGGGGGAACGTGATGAAGAGCTGGAGG
5647	CaTSNP8652	TAGAGAGTCTGATCCTCCATTGGAAGAAGGATACATTTACAAACCGATAAAATCTTCACT[C/G]TGTCCCATTGCCATGCCTAGTGATTATGGAATGTCTGGTTTGGTCGGTACCGGAGACAAT
5648	CaTSNP8653	CTCCACATTCCTTGGAACACCATGATAATCCATTTCCACAAGCACTTTCTCCGACTCCGA[A/G]TGAAGCTTAAAAGGATCCTTTAGGCGACATATTTTGCTAATACAATCAAGCATAGCATTG
5649	CaTSNP8654	GTGGAGACCATCATAAATCAGCATTACCCTTTCAGAATAGTTACCTTCCTGACCGTACAG[G/A]TCACATCGTGTTGTCTGGATATCATACGCTGCAATTTCACGTCCATAATAATCTGCTAGT
5650	CaTSNP8655	CCTTCCTACACTTTATTCGCAGGGTCCGAATGGATCAGATCCTCAACATCTACTATCCAT[C/T]GAAATAGACGAAAAGAGAAACCCAATCCTAGGTGTCTAGTTTAACTAGATGATAGGATAT
5651	CaTSNP8656	AATCAAATGCAAATTGACATCAATAACCTCACAACGGCTGGTACAGAGACTTGTGAAACT[G/C]AATCAGAGCCAGCTGAAGTAAGCCAACATGAAAACTCTGCTCATGCCCATTCTCTTGATG
5652	CaTSNP8657	ATGTAGACGTCCTACATGCACAGATTATATTATCTCTCTACATGCCGATGATATGTCAAG[A/G]GGAAGCAACAACTATGTTGGGAAACTGAGATCAAATTTCCTCGGAACGAAGTTCACAGTC
5653	CaTSNP8658	GTTCGCCTGATGGTTCAGCTATCCCACACCGAATAATAGCATCGCCAGCTCTTAGTCTTA[G/T]ACCACGATGTTGTTGTGGACTTAGCCGCGGGATAACCTGAAAGCTTAGAACCACCATCGA
5654	CaTSNP8659	CGTACAATCCCTCACCGTTCTCTTCAATGCCTCACTGCAAAAACCAATCAAACATTTGAA[T/A]TTCCCATAGATCAAAACCACCCATGTCAAATTCTCACTGAAAACACCATCACAGGTAAAG
5655	CaTSNP8660	CACTGCCTGGGAAGCACTGAGAGAGGACCTTGCAGCTGAGAAGGTTGCAATGACTCTCGA[G/A]GAGGTGAGAGAACGGATGAGAAACGTGCTGTCACTCACAGATGTCACGCGCTTTCCAATT
5656	CaTSNP8661	GAGGAGCTCTGAAACCCAAGGAAACACCTTTGGACTCTGTTCTGGCAGCTTTAATGACAA[G/A]CCTTGTTGGGTATGAGTAGTTTCTAGTATTGAGTTGGAAGCGAAGAGGGTGAAAACACTG
5657	CaTSNP8662	ATACTTAACCAAACCCGCAAAACGGTTCCTCGCTATTCCGTCCAACGTGTCACCCGGTTT[T/G]ACTTTGTAACGCGGAATTTGGTTCGACGTCCCTGTTCCGTTACCGCATTTACACGGAAAC
5658	CaTSNP8663	TAAGCAAACCAGTCAATCGGAAGGTTATGGTTTTATCGAGTTTAATTCTCGTGCTACTGC[T/C]GAGAGAGTGCTTCAGACATATAACGGAGCCATTATGCCTAATGGTGGCCAGAGCTACAGA
5659	CaTSNP8664	AAATTGAGCAGCCATCATTCCAAATCTTGTTGGGAAACCAAACAACAAACCATCAGCCTC[A/G]GGTAGTTCATTGGGTGTAATAATTGGCACATCACTCTTTGGAGGTGCTCCCATCTTCCCA
5660	CaTSNP8665	GAAAAAAGCTGAATCTAAAGGTAAAGGTAAACCGAAAGAAGCTTCTACATCTGAAGGTGA[G/T]AAAGCGGAAGCAGAAACCTTTGAGGATGTTCCAGAAGCTTCTAACAAGACTGTTGTCATC
5661	CaTSNP8667	GCCCAGATTCTCATCCATCACTTCCATGATATTCTCTGCCAGTTTCTTTAACTCAGACCG[A/G]TATTCTAACATGGTTTCCCTGAAACATGGTGTATTTTCCGGCCATTCGTTATTATCCAGG
5662	CaTSNP8668	GTCTTCCAAAGCTCCTAATAAAGAACGTGGTTCCATATCACTTGGATCAGTGTGGTGGCC[A/G]AGTTTGCTATCGACACCCCAAGAAAATGTATAAACCCTGCCTTCTCTTGACAATACAGCT
5663	CaTSNP8669	TATTTTTCTGGACCCTGACCGGCCGAGCCCTGCCATGGAAAAGCTTAAACGTCAATTAGC[T/C]GAGGCGGAGGCCGCACTAGAAGCACGAAAGAAACCACCAGAGGAGACCGGTCCACGAGTA
5664	CaTSNP8670	GTTTAGACCGGCGGCGATTTTGCGGACGGTGAGGTGGACGTCGGAGTAGGTGAGGGTTTC[G/T]CCGGTGTCGCCGTTGATTAGGCATGGACGGTTGTGGAATTGAGAGAGGTTTTGGAAGCAG
5665	CaTSNP8671	TGGTCACATGCCCCATGGGAAATTCAAGCATGGCAAGTTCAAGCATGGAAAATTCGGCAA[G/A]CATGGGAAATTTAAGCACGGAAAGTTTGGCAAGCATGGTATGTTCAGGAAGTGGAAGTGA
5666	CaTSNP8672	TAGCCTCGTCATCTAAGTCAACTATGGTTTCAATCATCTGTGATCTGTAATCTTGAGCCT[T/G]CTCTAGGAGATCTGCAGGTATATCTTCATAAGAAAACTTGGCACCAAGCTCTTCTCCACT

5667	CaTSNP8673	CCCGAGAGAATAAGCCGGGTGCAGGAACTGTTTCCCTACGACGAGCGCAGAGGATCCGCT[T/A]CCTCCCTTGTATTTCTCTGTTGTTTTCCACCACGTGGCAGCGGATGGAAGAGGCGCACCC
5668	CaTSNP8674	CTTGGGCCTGTTTCCTCTCTGTGTATTTGATGCCTGGAATTTGGGTTGCCAATGAGATGA[G/C]ATGCGTTCTCCAGTACTTCGGCTTTCCTTTAAAACAGCATTGACATCTGGTTGATTGTGC
5669	CaTSNP8675	TATTGCCAAATTCTTCTATGGCTGCCCTGAAAATGCTTTTCAGGTTGCTTTGGCTGGAAC[T/C]TCGTATTCACTTTTACAAAGAATAGGTGCTATCGTGAGGAATGGAGCAAAGCTATTTGCA
5670	CaTSNP8676	TGTTCCATTAGGAAGGGTTGGACATATTTTCTCTAGATACGCCAACGCGTGAGATGTCTC[A/G]AGAGCTGGAATGATGCCTTCAAGTCGTGAAACTCTCTTAAAAGCTTCCAATGCTTCATTG
5671	CaTSNP8677	TAAGATATATTGGCAACAAAACAAAATGTCTATGGCCGATGAACCACTGTATCCAATAGC[C/T]GTGCTTATAGACGAGCTGAAAAACGATGACATCCAACTGCGGTTGAACTCAATTCGTAGG
5672	CaTSNP8680	GCCTTTAGACCCTTTGAATGATTGTCCAAGGAATGCATACTACATGGATGGAGTGTTTGC[A/T]TCTGCTGATGGAACACCCTTTATACAACCAAACATGATTTGCATTTTTGAGAGTTATGCT
5673	CaTSNP8681	CCGTCCTTGTGGAACGGTGTAGTGATCGGAATCAATGTAGCGGACGGCTCGATTTGATTC[A/G]ACTATCCAACCAGGTAGTGATGAAGTTTCTCCGTCTAGGTTTGTTGGAAGTAGTACACCC
5674	CaTSNP8682	TGTTGGTGGCGTTGCAAGAGCACGTGCTTTTGCAAAGAAATTATCTGATGCACCTTTAGC[T/C]ATTGTAGACAAAAGGCGCCACGGACACAATGTAGCTGAGGTGATGAATCTGATCGGAGAT
5675	CaTSNP8683	GAGAGTCAACCTCTCCGGCGGCGGAGGGCCTCGCCGTGGTAAAGCTATCCGGGAAGTCGC[T/C]GACAGAGTGCTCGCCGTCGCCGCCAAAGGGAGGACAAGGTGGAGCAGGGCCATCCTAACC
5676	CaTSNP8684	TACTGCTCTGGGAATTTATGCAGAAAGCTTTGAGAAGCCATTCCTTGAATGTACATCTGA[G/A]TTTTATGCCACTGAAGGTGTAAAATACATGCAGCAATCAGATGTTCCAGACTACTTGAAG
5677	CaTSNP8685	CCATTCTTGCATAACAGGTTGAGGAAGTGCAGAGGAGCCACACATCATTAGACGCAAGTT[C/A]CTTGCAGCGGATGCTGAAGTGGCTTGTATCTCAGGATCCATGGCATGATAACCTTGTATT
5678	CaTSNP8686	CTTTTTCCGAGCCTCTACCTCACCGACACGTGTCAGTCTTTACGGATCGTCACCGTCTGT[G/A]TCGGCTTCGTCGGTTCGGTTTTCACTAGACCGTTCAATTTCACCGAACCGGTCTATTTCT
5679	CaTSNP8687	CTTTTATTTCTGAAAATTTTATGTCGCTTTGCATAAACATAAGAGAGGGCGAGGAAAGCC[G/A]CGGCAGAAGTTGAGACCGAAACCCATGGAAAGGGAGGGTCCTTAAAGCACACGAGAGACG
5680	CaTSNP8688	TGCGGAGGAGTTCGATGTTGACACGTGGAAGTGGGGTCCAGTGGTGGAGGAGTTTTTGGA[T/C]GAGTCCACGTGTCCGAGGACTTGTGTGGATGGTTGTGACGTGGAGGGGGAGAATGTACATG
5681	CaTSNP8689	AGCTGCTGGACTAAAAGAGGGATTCAAGCTCATGGATACAAGCAACAAAGGCAAGATTAA[C/T]ATTGATGAGCTGCGAATAGGATTGCATAAACTAGGCCATCAAATTCCTGATGCAGATGTC
5682	CaTSNP8690	CCCACCCGACTTGGCAATCGAATCAACACCTTGATCCGGGTCTCGTCTATAGACAGGAAC[A/G]TCATTCGACTTACAAAACTCTTCAATTCTGTCATCCAATGCAACAACCAAATAATTAGGA
5683	CaTSNP8691	ATGCACAACCTGTTGTTGAAGCTGGTGGTATTGAGGAATGTGAGTCCACTTGTTTAGGAA[G/A]TTGTTCTTGTACTGCATATGCTTATAACAGTAGCGGTTGTTTCGTTTGGAGGGGTGAACT
5684	CaTSNP8692	TCCATAATCTTTCTTAGTAGTAAAGGCAGTCTTGTTTTCCAGAAGATTTTGCCAAGCTTT[A/G]CCACTCAATAAATAGCGAATGAAAAACTTGATGACGTCAAGAGGAAGATAGAAGATAATA
5685	CaTSNP8693	TAAATTTAAGCACATTAGCGAAGAAAGCTCTTTGATATTTTTCACACCAGCATCAGTCAA[T/C]CCACCACTGCATATTTCCAAGGACCTCAGGTTCTTGAATTTTTTCAAATAATTTGTCCCA
5686	CaTSNP8695	CCATTCTATCATTTTCATTGTTTCCAATACCCCTGGTGGGATGTTTTAAGCCCCTATCAT[G/A]TCATAAGATAAACCACAGGAATCAGTATCATTTGTGAATCTTCTAAAGCCGAACGGGGAA
5687	CaTSNP8696	CTTCCCCACAACTGCCGACGCCAAAGAATTACTCTTCGGCGGAACTTTCTTAGTCTCAAG[T/C]TCATCCTTTACCGGTCCGCCTTTCGACATACTCTCAACCCAAACCCTAACTTTCTCCAAA
5688	CaTSNP8697	TCGTGAGGTTGTGGAGAGAAGAGTTATTACAGTCACGGGAACTAGGCCAGATGATGAGAC[G/A]ATCGACCACTTGATAGAAACTGGAAACAGTGAGCAAATCTTCCAGAAGGCAATTCTTGAA
5689	CaTSNP8698	TATCATTGCGAGAGCAGCCTCGATTGGCAGGGAATCAGCAGCTAATGCACGGTCATCAGC[G/A]ACAAGGGGATTCAAACCATCAGCAGTAGAAAGTTCATGAATGCTAGCCAAAAGGGGGCGC
5690	CaTSNP8699	GGGGCTCAAGCCTTGGATTTGAGATTGCCATTTGGTGAAATTGAGGTTCTTCGGGAAAAC[G/A]TTGACTTGATCAAGAGACAGATAGATCCAAAAGTTGTGGAAGATGTTGAAATTTTATCTG
5691	CaTSNP8700	CTCTTTCTTCATTTTCTGGAGATCAGCTTTGTAGTTCCGTAACTTCTCAGCACGTGCCCG[T/C]GCTTCATCCAAAGGACTAAGTTTAGAAGTCTTCCCTCTCCTTATCGGCCCGGCGGTCTTT
5692	CaTSNP8701	AGAGCGTGTGAAACGCAGGAATCAAATTACTGCCGAGTTGTTGAGTGAGGAAAATGGAGG[T/C]ACCTCAAATAACTTATCGGCTGCTGAAGTGAAATATGAGGATAATGAGAATTTTGTTGAT
5693	CaTSNP8702	AACTGGAGACCCTGATTCATTCAAAAAGTATCTGTTAGAATATGAAAATACAATCTGTGG[A/G]CGCCATCCAATTAGTGTTTTTCTCCATATGCTGAGGAACTGTTCAATAAAGATAAAAATC
5694	CaTSNP8703	ACTCCCTGTCAAAGTTGAAACGCTTCCATTTGTATTATTAGCAATAGCTATTTTTATGGT[A/G]CTATCGGAATCAGATGGATCTCGAGGATGAATCACAAGTGAACTTGGAGCCTGGAATCCA
5695	CaTSNP8704	TGCGTTTGGAAGTGTTGGTGGTGTTGGTGGTGGTGGCATATTCGTTCCTATGCTTAGTCT[C/T]GTTATTGGGTTTGATCCAAAATCATCGACGGCTATCTCCAAATGTATGATTTTTGGTGCA
5696	CaTSNP8705	TGGAAAGTTAATGGAGGCATTAGAACTTTTTCACAAGATGCAACTAGAGGAAAGGGTTGA[G/A]CCGAGTGAGTTTACAATGGTGAGTTTGTTAAATGCTTGTGCTCATTTAGGAGCACTTCAA

5697	CaTSNP8706	CTCAATGCACTTCTCTGCATCCTTTAAACCTTCAGGCATTGCCCCCAGTTTGGTGTAACA[C/T]GCAGCTCTGTTACTATAAGTCTTAGGCTCTTTCGGGTTTCTTCTTAGGGACTCAGTGTAA
5698	CaTSNP8707	TAGCCGCACTAATGACATTTCAGGAGCTGGTCTAGATGACTTTGAGTCATGGAGTCTTGA[C/T]GCTGATGATTTGAGGCTGGAACTGCAGGCTAGGAAACAACGAGAGGCAGCTCTTGAGGCT
5699	CaTSNP8708	TCTTGACACGCCGTTATGCAATGCACATACAACAGGAACAGATATACTATGGGCAGGTCT[T/G]CCTATGGTTACTCTACCTCTTGAGAAAATGGCTACTAGGGTTGCTGGGTCACTCTGTCTT
5700	CaTSNP8709	TGAAGATTATTTTGCTGATCGGGTGAAGCAGTTGACCTATACTTTTCCTGAAGATGCTAC[A/G]ACTAGTACTGGAGCTCCTTTCTGGTCTGCCCCAAAAAGATTTCCCCGTCCACTGCAGTTC
5701	CaTSNP8710	CAAGAAAGCTACCTTGGCTCGTCCAGTTCTTGGAGGATCTAGTACCTTGCCATATCCTCG[G/A]AGAGGCAGAACAGGCAGAAAACCAGCTAAGAAAGATCCTAAGAGTGAGAGTCGGAGCGAC
5702	CaTSNP8711	TGATCCAGAATAAGTGAACCCATGAGAAAATGAGCCAAGTTTGTTGCTTTGCGAATGTAT[A/G]ACTTCTGAAATTTCTGGGCTCACAAGGACAGCTCCAATAGGCATATATGCAGAAGAAAGT
5703	CaTSNP8712	TGTGAAAAGTGCTGTTTCATATGCTTTAAATAGACAATTGAGTTTAGAGGATCTCACTAA[G/A]CCAGTGGAGGAGGAGAATATTAAGATTACGATGGATGACTTTTTGAACGCACTCCATGAA
5704	CaTSNP8713	CAAAATCGATATTCTGAACCAGATAATCGTGACTGGCGTGGCCGATCTGCGCAACTTCCT[G/C]CTAACGCAGATGAGAGGTCTTGGGATAATCTCAAGGAGAATAGAGAATTTAGCAATTCTA
5705	CaTSNP8714	ACAAACTTACCCTTAATAACATAACCCTTGAATTGTTCATCGCTATGTCTCTCTGAATAT[A/T]GGTTATGGCACACACAACACAAGAGTTGATGTGGTTGAATTCAATGAAGTTAAGGTTGTT
5706	CaTSNP8715	GTTATGGTGGTCCAGGCACTGGCGGAGGTTATGATTCAGGTGCTGGTGCTGGTTTTGGTG[C/G]GACAGGAGGACTCTATTCTAGTAGGGGAGGTTATGGGGGCAGTAGTCGATACCATCCTTA
5707	CaTSNP8716	TCTCGCGCGTTTCAACGACGGGTCGTAATAGGTGTTAAAATTATCAAGACCTACAACGCC[A/G]TCGCCTCGTTTTTTAAGAGCGAGTGAACAGTGACTACCGACGAAACCGGCTGCACCGGTT
5708	CaTSNP8717	CCTTCCTACACTTTATTCGCAGGGTCCGAATGGATCAGATCCTCAACATCTACTATCCAT[C/T]GAAATAGACGAAAAGAGAAACCCAATCCTAGGTGTCTAGTTTAACTAGATGATAGGATAT
5709	CaTSNP8718	AACAGAAAGAGACGACGATTTAGGCAGTCAGGAAGAAGCAGACGATGATGATGGGCGAAG[T/C]GACCGTGAAGGCGATAGCAGTGATGAATCAAATATCGGTGATGATCTCTATAAAGATGAA
5710	CaTSNP8719	GAACCACGACTATGTTCTGTTTCACTATCCAGGAACTAGGATCCAATGTAGCATCTACTC[G/A]GAATCCTGCATATTCTGAGTAGGAGCCTCTGAGCCCTTAGTGAGTGATAGCTTGGTTGGA
5711	CaTSNP8720	CTCTTCTGATAGCTCTCAAAGCAGCAAGAGGAAAAGGGTCACCTTGTTACCTAACAATGA[A/T]CATGGACCTGCCATTCGAATTCGACTACCATTGAGGAAACATAGAGAACCTGAGGAATCC
5712	CaTSNP8721	GGCAAGTCCAAAGGGAAGCTGGGTTATGGATGATGACTCCAAAAGCATGGCCAGTGTACA[A/G]TCAGATCGGTTCCGTAGGCACTCTATTGCCGGATCAACAATTAGAGATGACGAGAGCCTT
5713	CaTSNP8722	AGAACTCCATAATATCGTTTCTCCTTCGATTCTTCTTCTTCACAGAAAAATGGAGTTTGC[T/G]GTTGCAAAAGCTTTGAAACCGAGTTTAAGGAGGGAGTTTGTTTTTCAACAAACGCTAGGC
5714	CaTSNP8723	TCTTATCGCGAGTCACTGTAAGTTGCTTCCTTGCAATAGTCGATGTCGGCTTGAAAGCTA[C/T]TCTCATATTAATTATTTCTCCATTGGAAATTCCTCCCTGTATCCCACCAGAGCGGTTTGT
5715	CaTSNP8724	TCCACCAAATGGCGGTGATGGTAACCTAGAGAGGGACTTAGACAGAGGAAGAATTAGGGG[A/C]AGGTTGTCATCTGGAAGGCTGCAAGCCCCTCACGAGGGAAGGATCCAGGATAGATTAAGA
5716	CaTSNP8725	AACACCATTAATCCTAGCAGCCTCAATCATGTTGAAGCTAATCATTGTGTTGTTATACAT[T/A]ATAACCGAGTGGTTGGATTGGATAAAACCCATTCCACCCATATCAGCAGCAAGATTGAAA
5717	CaTSNP8726	AGGAAAAGATGTACCTGATGAGCTTTTCATGATTTTGCAAGAGATGCAGAAGAATTTGGT[G/A]TATTTTCGAAGTATGGAACAGAAGAGAGAAGCTTTGAAACTTGTTGATCTTGAGAATGTT
5718	CaTSNP8727	TGAGATTGGTGTCATCCCTCAGGTTGCTAGTGCAACAGCAACATTTGTAATGATGTTTTC[C/G]TCATCATTGTCTGTGGTTGAATTCTATCTACTCAAGAGATTCCCTATTCCCTACGCAATA
5719	CaTSNP8728	TTTCTCAGACTGTCTGTCTCTTGAGCTTGTCTTCAATGACAGTTCTGCAGAACAGTTTGA[T/C]GGGAAATGCAATGAAAGGAATAACCTTAGGCCAATTTCAAGTGACAGCAACAGACCTTCT
5720	CaTSNP8729	AGCACATGGTGGAGCCTGAAATGGTTCGTGTGCTTGGAAGATCGGCTTTTCATGATACCA[C/A]TTTAACATCTTCTGGTGATCTCTGTTATCTTGGTGGTGAGTGGAGGAGAGATGTTGCATT
5721	CaTSNP8730	TGATAATCATTTCAATGATAAGGAGTTGATAGTTAGATATCAAACTGATATTGATAGCAA[C/T]AAGATTTTTTATTCTGATTTGAATGGATTTCAGATGAGTCGAAGAGAAACTTACGATAAG
5722	CaTSNP8731	ATCATAGAGAGCTTCGTTATCCAAAACCATGCATTCATCAGCATTCTCAACAAGTTGATG[A/G]ACAGAGAGAGTAGCGTTGTAAGGTTCAACAACTGTGTCAGAAACCTTTGGAGATGGGAAC
5723	CaTSNP8732	CTTCCTCCTCCACCACCTGCTCCTCCCAAAGCTCCTCACCCACTCACCGCTCTCACTTTC[G/A]CCATTTCCGACCTATTTGACATAGAAGGACATGTGTCGTCGTTTGGACATCCAGAATGGG
5724	CaTSNP8733	AACATCTCAACCACCTTACATTCAAACACCACCATCAACCTCTTCTCCATCATCTTCACA[G/A]TCACCCCTTCTTCCAACTCCTCCAAAATCCTCTCAACCTCCAATAACAGCACAAACACCC
5725	CaTSNP8734	ACAACTTCAGAGGAGCTGGTGTTGCCACAAAGCCAGTGGATGAATTGGAATTGCAGACAA[A/C]TATTGATGTTGAAGAGTCCAATCCACATCATCATGTCATGGATTTGTGTTCTACAAATGA
5726	CaTSNP8735	CACCAATTTCTACACCAGGGTGTACGATGACGATGAAGAATGGTTTCGTTCAATATTTGC[G/C]AATTCTAACAAACAAGAAGCTATTCAAAACCAGTACGAATTTTTCGTTCAAAGAATGGGT

5727	CaTSNP8736	CGTTCCATTACTTGAGTCCAACAACATAGTCCAGAGAGACAGAGGAAGCTGGAAGAAGCG[G/T]ATCACCAGGTTATCTCTGTCTTTATGGTTCTTTAGGCGTAAAACAATTAGCGCTAATATG
5728	CaTSNP8737	ACGAGATTGAAGTTCCTTCAGTTCAGTTGCCAAAATTGCTTTTTCTCCATAAATTTCGCC[T/A]GCATGCATGTCATTTGCTTCTTTGGCGTGCACCAACATGGCTTTATACTGTTCTACCCTA
5729	CaTSNP8738	CAAAAGGGGTGTTTCAGTATCCTTCTTAGGTGACAACAAAAATGGTACTTACTCCATTTC[A/T]AGAAGGAGATTTGAGATCAATGCAAGAACTGCTGGAGCTTCAAAGACAATTGAAGTTGAA
5730	CaTSNP8739	TTAATGCCAAAGTTTGACATTAATGCATTTTTGAGTCATGTGAATAAATATAAAGTCACT[A/G]TTGCTCCTGTTGTGCCACCAATTGTTTTGGCTATTGCTAAGTCACCTGACCTTCATAAAT
5731	CaTSNP8740	GCAAAAAGAAGTCTTCCAGATATGGAGAATGCAATGGAGGTCACAACATGTGCCATCTCA[G/T]TGTCCCTGCGTGGCTGATAATATACTTGAAGTTGGTGCCCAGTCCTAATGTCAAACAATC
5732	CaTSNP8741	CACAAACCTACTTTCATTGAAAACACTTATTTACAGGAGTGCATTGCTCCTTAAGTTTCA[T/C]TCATCATTGATGTCGAGATTGAGAATCCGACGAAGGGTCTCAGTAGCAGAATCAGCATTC
5733	CaTSNP8742	ATATTCCTCAACTTTATTAACAGGAAAGCCATTTGCAATACCCATTTTGTCCAAAGACAC[G/A]TCTGTTGTTTCCACAAAATCTCGGATGAGTGAGCGACTTTTGAGAAACTGTTGGTTGGCA
5734	CaTSNP8743	CCGACATGCTGGGTCCTTTGGATGAGAATGAACCTGATGGCGAGGACATTGATGAAGAGG[A/C]TGAAGAAGATGACGACAATGATGAATTGGAATCAAAACTGAAAGGCCTTGAGATTTAAGA
5735	CaTSNP8744	AGGGATGGGAATATCACGTTCTCAAAGGAGCTTCCAAGCTACTCTCAAGGAAGGGAAGCC[G/A]AGCTCCTTATCTTATATATGAGGAAGATGAGCGTTTGTTGCAGGCCAGTAATAGCAGCGC
5736	CaTSNP8745	CTGGTCGAAGCAAAGAAGGCCGGGGACGACTTGGTGGATGCTTATGCCAAATATGTCAAA[C/G]AGTACTACGTCGAAAGCTTTGGCGTCAATGTACAAACTTATGACCAGAAATATTTGAAAA
5737	CaTSNP8746	ACCATTCCCCATAACCTGACTGAGTTCAACCACATCTCCTTTCTTCAACGCACACAACGC[C/T]TCTGCTGTGGACCCCGCAACACTCTTCACCAGAAACTCGAACACGCCACGCGCCGATGCG
5738	CaTSNP8747	AGAAAGGAGATGCTGCGCAGAGAAAGCATCCCACACCGAACACCAACAACTGTAACTTGA[G/A]TGATGTGAACAATGGCATGGGTGGCCTCGAAAATGAGACCGAGCATACTGAACTAATCGA
5739	CaTSNP8748	ATGAGAGGTGGAATTGTGGGGCTTTCTCCTGATCAAGGAATGAATGGTAATCTTGTGCAA[A/G]GTGGAGGGATCGGCATGGTTGGTTTGGCGCCCGGGGCTGTTCAGATTGGTACTGGATCGC
5740	CaTSNP8749	CGGTCAGATCGATGAAGAAGCGGTTGGATTGACCATATGGAGGTTACCAGGAGGTGGATT[T/C]GCCGTTGGGAGATTCATCGGCGGTCGAAGAGCCGGCGAGAGTCACTTGCCGGTGGTGTTT
5741	CaTSNP8750	TAGTGATACCTCACCTGGTGTAGCACGCGATGCTTTCAACACCTTCTTCAGCGAGACCGG[G/A]TCCGGAAAGCACGTGCCAAGAGCCTTATTTGTTGATCTAGAACCTACGGTTATTGATGAA
5742	CaTSNP8751	CATGTCCATTTGTTCAACTATCTTTCTTATTTGGGGCTCAAAACCCATATCCAGCATCCG[G/A]TCTGCCTCATCAAGAGCCAAATATCTTATCATCTGCAATGACACCCTTGCCCTCTCAAGC
5743	CaTSNP8752	AGACCAGTTGTATGCAATTGAACCCGTAATACCACTAAGCCACAAACACCCAACAGTACG[T/G]AGCTTGTGTTCGACGACCCATTTTCTGAGAGACTCAATTTGGGTTTTAGCATCCGCCATA
5744	CaTSNP8753	TGCCATTGCCACCCCTTTTGCCTCCTTGCTTTGTCTAAGGTCAACAAGAGCCTTGTATCT[G/T]CCAATAGTTGCCATGCTTCCAAGATGCTTGTAAACAAATGGATCCCCAAGTTCTATTTCT
5745	CaTSNP8754	GTGCCTTCCAGTACTTTGGACTCAAGGAAGACCAAGTACCTCTAATTATTGTTCAGAATA[C/A]TGATGGGAAGAAATTTTTCAAACCCAATTTGGAAGCTGATGATGTTCCAACTTGGTTGAA
5746	CaTSNP8755	AGGAGGAGGAGGAGGAGGAAGAGGAACAAGTTTCACGACGCGTGAAAAGAGAACGCCTCA[C/G]TGAAAACAACGCCGCCTCTGATCATCAGGACACCACCAGTCGTAGAATCATCCGCTCCGA
5747	CaTSNP8756	AGGTTTACGGAATTCTCTATGTATCTGTCTCCTTAAAACATGGAAAAAGTTGGCAGGAGT[C/T]GTGACATTCACAATCTGCAAATTACACTCCTGAATCTGTTTCCGAAGAGTTGGATCCATC
5748	CaTSNP8757	ATAATAATTAACAGCTTCATTTGCAGCAATGGTTTTAACATTTGGCCATTTAACTCTATG[A/T]TCAGTGTTAGCACCTAGTCCTTTGTTATTGTATTCATAGTAAGTGCATGTCTCTTTGTTA
5749	CaTSNP8758	AACAAGACTATCCCTAACAAGAGCAGAAACCTGAGGCCTATCGGTAAGAAGCTGATCAGC[A/G]TTAAACTGAGCAACAACAGCTTTAAGAACTTCATTACCAATAGAAGGAAGAACCTTCTCA
5750	CaTSNP8759	TATTTCATCATGGTTCTTCTCATACATGTCACCATTTGACATATAAAAAGCAACCACAAC[A/T]CCAGCAGTGTAGTCAGCAGGTAACTTAATGGAAGCACTGAAATAGCCATGAAGGTAAAGA
5751	CaTSNP8760	CATCGCTTCCACCGCCGCCTTCGACGCTGTGTACGCGCCGTAACCTGGCTTAAACGCCGC[T/C]GCTAATGAAGTCGTCAGAAGTATGATTCTCCCTCCTCCGCCGCGCTTTAACCGGTTCGCT
5752	CaTSNP8761	AAATGCTACATGAATATCAGATAAGTAAAGAGTTCCTGCAACAGGCCCTGTTGTTGTTGA[A/G]AGGTAACAAGCGAATGTCTTCTTCAGCTTCTCATTTGGATAGGTAGTGAATATTTGTTTG
5753	CaTSNP8763	TATACCTAATCATCGGACATTCCCTCATGTCTATCTTCTCCAAGCTTACCAACTTACCAA[T/G]AGCTTCAGGGAAGTTTTCAAGGTTAACGCATTGAGAAACGTCGACATACTTCAATCGAGT
5754	CaTSNP8764	TTATGGTTATGGAAGTCCATTATCCGTACCAGCGTTTGAGCCACCTCGAAAGCTGAATTA[T/C]GTTTTAGAAATTCCTCCTGTAAGTGATGAGAATAATTCAAAGACAGATGATGAACTTGGT
5755	CaTSNP8765	AACCGTTAAGGTTCGTCCGTTTCACCGTGGTAATGGGGGAGTTTTACGTTGCTCTCATAA[T/C]GATACGCCGTCGTTTCACGACGATCAAGGTCCTCCTCAAGAAGCTGTGTTGAAAGCCATT
5756	CaTSNP8766	GTCAGTGGGGAAGTGGTTGACTAGACGACAAGCACACTTAGTTCGTGCCCAACAGGGTAT[A/C]CCTGTATCTGAATACGGGGTTTTGGTTGATATGATCCGCGTGCCTGATTGGGCATTTGAA

5757	CaTSNP8767	AACCATTCCAATTCTTTGGTAGCAAGAGGTCTAGTGAGCTCGTGCTTTTTCAGTGTCTCT[T/C]GGACAGTAGCTTGGACCTGGCTATGATATGCAGTGGCTGATTCCAAGAAATTCCCATAGG
5758	CaTSNP8768	AGAATCGAGTTTGAATCAAAGAGAGACTAGTGGTTGGATTTCGCGGTTGTTGAATGTTTG[T/C]ACTGAAGATGCTAAAGCTGTTTTCACTGCTGTTACTGTTAGTTTATTGTTTAAATCGTTT
5759	CaTSNP8769	CTCCCTTTGTTCTATGTACCGCCTCTCTGCTGGTGTAAGATGATCATCATAATGAGCAGG[C/T]TTGTCTTCTCTGCTCAATTTGCTCCCATCGTTTACTTCCTCATCATCATTAGGATCTTTA
5760	CaTSNP8770	TATGCTTCCCGACAAATTCGGTAACACAGCATTACACGTGGCAACCAGGAAAAAGCGAGT[T/A]GAGATAGTGAATGAGTTGTTACTTCTGCCAGACACCAACGTAAATGCATTAACGCGAGAC
5761	CaTSNP8771	TTCTTCACATTGTAGTCATAGTAACCAAGGCAAAACCAGAAATGACAGCATTCCTGTGGG[A/C]TTAGGTCTTGGCCTTGGACGGAACACCAGATCTGTTTCACCCTCATCATGATATTTACAT
5762	CaTSNP8772	ACGCATGAAGTAAAGTGCACTGTCAATAGAGACAGCTTGTGACCTAAAGTTAAGGGACAA[C/T]GGCATCTTTATTATACTTCCAGGCATGTTTCCTGGACGACTCCAAGAGTTTTTCACCGAG
5763	CaTSNP8773	GTCGCTGTCTCGATTAATGAAGAGTGCCATTGGGGAGGGAATGACACGAAGGGATCACGC[C/T]GATGTGTCCAACCAGCTCTATGCAAATTATGCCATTGGAAAGGATGTCCAGGCAATGAAA
5764	CaTSNP8774	TAAAATATCAGCTTCTTTCTTATATCCATGCTTCAACTCTGCTCTAACAGCTGCTAGCCA[C/T]AGTTCAGGGTTTTGAGGATTCTTCTTTCGACCCATTGTGAGAACTGCACGAGCTTTACTC
5765	CaTSNP8775	GATGGAAAAACTTAATAATATATTCTTTACGTTGATGGCTCGGGGACCAGAAGTGGATCT[C/T]TCCGAAGTCACCGGGAAAGAACAAATGGATGCACAACCGATTAAAAGAGGAAGAGGAAGA
5766	CaTSNP8776	GACATTGAGAAGATCTATGCTTTGTAAGATAGTGATTGTGCGTTACCGCACAATACAACA[T/C]TGGATGGTGGATGTGTATAGTATGGTCCGAAATAAGTTCCGATCCATTGTGGGCAACTTC
5767	CaTSNP8777	GTCAATTACATCAACAAAGCCAAAGGTGAATAGTAGCGACGATGAAGGGACTTCATCTGC[C/A]GCGGCTCCTATTATGCCTTCATCGGCGGTTGACTCTGCCCGCCCTCAGATCTGACCGTCT
5768	CaTSNP8778	ACCTTCTACCACCTCTCCTCCACCACCGCCGCCGTCGACTAAGAGCTGGGCCGACCAAGC[T/C]GAAGAAGAAACCAATCCATCCTCAACCTCCGCCGCAGAGCCCTCGACTCCGTCGCTCGAC
5769	CaTSNP8779	CTATTATGTTAACCATCATAATTGTGATTGGCGGTATCATAAGGGATGACCATTTAACAA[C/T]ATAAAGATCAGCAAACTCGTCGTCCACGTCATCACCACCGGATTTTGAAGTCAATGTAAA
5770	CaTSNP8780	AATCACCATGCCTGAAATTTGGGAACATAAATGGTTTCTGAAGAATCTTCCCATGGACCT[C/A]ATGGATGAGAAGATAATGGGTAACCAATTTGAAGAGCCTGAGCAACCAATGCAGAGCATT
5771	CaTSNP8781	GCTTCCTGGTTCCTCTCCGCCATCGTGTATTAGCCGCCCAATCTCTTTCAAAGAGACTAC[A/G]TCCTCTGTAATAACTTTGGCGAAAATGCGGCCAAGAAACTCTGGTGCTTTCGGTGCATCC
5772	CaTSNP8782	ATCAGGCACAGAAACACAAGCTTCCCATTCTGCAGTATGACTTGTCTTCACAAAATAGCC[A/G]TGCTCTGCAGCAAGTCCAAGCTTTTCACAAGAAGAAAACCATTCAGTAAGAGTCTTTCTC
5773	CaTSNP8783	CTCAAAGTCAATCGAATTCAAAGATATTGTCAAGATTGGACGCACTCACACTCAAGATGC[T/A]ACACCTTTAACTCTGGGACAGGAGTTTAGTGGGTATACTACACAAGTGAAGTATGGTATT
5774	CaTSNP8784	CAGAGCGTTTATGACTAGAGTCAAATGCAGTAGGACTTAGAACAGACATAAAACCAGCAG[C/G]AGATGTTATAGGATCAGATAAGATCTTAGTCTTATTATCATCTTGAGAAACCTTGAAAGA
5775	CaTSNP8785	CTTATCATTCAACAACCTTATTGGAATCCTTCCGAAGCAACTGGCCAACCTTCCCAATCT[G/C]CTGACCTTCAATTTATCTCACAACAACCTTCGAGGTGAACTGCCTGCTGGTGGTTTTTTC
5776	CaTSNP8786	AAAACCAACAATTTCATATCCAGATGCCTTATTCTTATCAGTTTCAGCAATAACACCCAT[G/A]CCTTCCTCCCCAGTACCAATGATCTCAACACCATTTCCTTCATATTCATGTACCAAGACC
5777	CaTSNP8787	TGTCTTCTTCCTCCTCCTCCAACTCGTTATTGCCACCATTTATATCACTTCCACCATCGC[C/T]AATGCCACCCACAAACACCAACAACCCCATCAACAGGATCTTCACCAAGCACAAAACTTT
5778	CaTSNP8788	TTTGTTGGAGGAGAGAGTAGGAGGAGGAGGATAAGGAGCAGTGTTAACCTGATGGTCGAC[A/G]TGGTGAACAGCGGTGGTAGGAAAATGAGGATCGTGGATTTCATGAGAAACGTGGTGGACG
5779	CaTSNP8789	CGAAATGCTGGAAAAGCAGGGTGCTAAAATTGACAAGGTGCTCAATTTTGCAATTGATGA[T/C]GCAATCCTTGAGGAGAGGATTACTGGACGCTGGATACACCCATCCAGTGGCAGAACCTAC
5780	CaTSNP8790	AAGGTTGAAAGAGTGAACAAAAATATCATCGAAACCCTAATTCCCAAAATCACATAATCA[A/C]TCGAATCGAATTCAATGGAGGGAATGTTATCTTCCAATGATCAACAATCATTGGTCTCTT
5781	CaTSNP8791	AAATATATGAGTTGTTCTTGGATTTGGCCCGTGTGAAATCACACGTGTATAATCTTCTGA[A/T]AGCTCCATTTCACTTGCAGAAAGACACCCTTTGAATACTCCATGAGTATTATTAAAGGTT
5782	CaTSNP8792	TTTCAAGATAAACCACCTCTGGTGTGGATCGCAGTTCCTTGAGCGTGTGCTGGAAATCAG[T/A]TATATCTGCTAAAGCATCATTTCCACCATAAGCCATCCACAAAGGCAATGATTTGGGTAT
5783	CaTSNP8793	CACAAAGCTCACCAGCTACATTTTCTTCATTCTCATTATCCTTGTCTGCAAGAATCTTGA[C/T]CTGCACACCAGGAAATGGTTTGCCAACAGTGCCTGCTTTGCGCTGACCTTTCAACGGATT
5784	CaTSNP8794	GGAGTTCGAACAGAATGAAACAAATAGACTTGTACAAAAGCTTCTAAAAAACACTATTTC[T/A]GAAGGTTTTACAAAAGTGGAACTAAGACATAGACTAACGGAGATGACGTTTCAATGGTAT
5785	CaTSNP8795	ATTCAAATTCAGTGACAAGTGTGATATATATAGTTTTGGGGTTATGCTTGGTGTGTTGGT[A/G]ATAGGGAAGCTTCCTTCTGATGAATTTTTTCAGAATACAGATGAGATGAGTTTGGTTAAG
5786	CaTSNP8796	TTCATCTGGCCAAATGGTATCACAACCATTTTCTGAACCAACCAGTCAATTTGCTCAGCA[T/C]GGCTTCTCAGCTGCAACCAGTCAACCTGCACAAAACCTTCCCAGTCCGTATGCACAGCAA

5787	CaTSNP8797	GGTCCTCTCAGTAGCCGTGAGAGTAGAGTTCAACAAATGAACATATTGCTTCAGCTGCTC[A/G]TTGCTCTTCTTCTGTCCATATCGAATTTCTAATTTTCTGGACTGGTAGTCTGTACAATTT
5788	CaTSNP8798	CACTTATACTTGTCACTGCTTCTGTTGATAAGATTGTACTAAAGAAGCCAATTAGTGTCA[A/G]CATTGATCTCACAATAGTTGGTTCTGTTATATGGGTTGGGCGCTCCTCAATTGAAATTCA
5789	CaTSNP8799	TACCGGAGGCTCCCAAACTCCTACCAGCGTCCTTCCTGAAATCTCTGGTGACTGGTCCGA[T/C]GTCACCGTCGACGTTCAGTGGGAGCTTTCTCAGGCTTTCCGGTTAATTGACCGTGACAAC
5790	CaTSNP8800	TAATAAGGCTGCACAGTCTTTAACAGATGCCATGATTCCAACCACTCAGAAGAAAACTGC[T/C]TTGAATCGGCTGGTTAAGGATAATGTCGCACTAATAGTTGTTTTAGAAGCAAAAGTTAAT
5791	CaTSNP8801	TACGGGCATAAACAATCCTACATTCTCTTTCACACTTATTACTCATCTCATATGGCTTCG[C/A]ATCACATCTCAGCTACAAGCCGTGCAGGGGCAACTAATGATGCTATATACAAGGAATTGT
5792	CaTSNP8802	AATCAAACGATCATTGTTCGGTATTTCTGAGTTTGTTTTTGAAATAGGCCAAGGTGAGAC[C/T]GAGACATTCTTGCTATCGTCAGACGCGTCTTGACACAAATGAGAAGGACAACTAGAATGA
5793	CaTSNP8803	GGCTCTTTATTTTATTTATGCTTCGAGTCTTGAAGAGGATGAAGAGTCAGGATTGTCTCT[C/T]TGAAGCATCAACTTGAATCATGGATTGTAGAAAGTCCTCCAGTTATTTTCTCTTATATGA
5794	CaTSNP8804	CAATGAGCAAGGTTCAAACATGATTGGAGATGGAATTCAACTGTTTGCTTCACAATATCT[G/T]TATTACTCACGTGCTGTCTCTGCTGCTGATCTATACAAACTCAACATCATATTCAGTGCT
5795	CaTSNP8805	TCTGAGACCCGTTGCAAGATCCGTTGTAGGTTTGTACCCGAAATCTTTGTAAGCCAATGT[T/C]ACATTCGCATGCGTGTATGGAACGTCACCGTTTCGCGGCATCTTAATGATGTGTTTCTTC
5796	CaTSNP8806	AGGAAGTGCACTTGGTGCTGAAATGATCGGAACTTTTGTACTTGTGTACACAGTGTTCTC[T/C]GCCACAGACCCAAAGAGAAACGCACGTGATTCGCATGTGCCTGTTTTGGCTCCTTTGCCA
5797	CaTSNP8807	TTCAAAGACAAAGAAAAAAGGAAGAGATCACAAAGAAGTGATAGTGAACGCAATAAGAGA[A/G]GCAGCTGAAAAGTACACTTCAGTTTACGTGTTTTCTTTTGAGAACATGCGGAATCAGAAA
5798	CaTSNP8808	AAGGAAGATCCCATACGTAACAGACGCCCAGTTAGGGTTCTTCGCATTGCAATCGAAACA[C/T]GACTTGTTTTCGGACTTGGACTGGAGCTTTTTGAAAACGGTGGTCTTGTCAGTGAAGCTA
5799	CaTSNP8809	AATGTAATGCAGTATTTAAAGTAGCTTGACTATGTTGAATGAAGATTTGAGCTGGCTGTG[G/T]ATACACAGTTATGAGACTTGAATTGACATCAAATTCTTGCTAGCTTACTGAGAAGCTGAT
5800	CaTSNP8810	CTGACGATTGAGGGTAGTGGGGTCATGTCGTGACCAAAAGAGATCCAAGAGATTTGTGTA[A/T]GGACAAATTTTCGGGTCAAATTGAACCCGAACCACTTCCACGTGGTTGGTGGATCCGGTG
5801	CaTSNP8811	GAAAAAGGTTAGAAGCTTGGAGCAGATGAATGGAAATCTCCTACTTGAACTTGAAAAGTG[T/C]GTGATCCATCATCCATCATTTATTTTCCACGATGAAGGAGAGGAGGAATCATTGGCGAAC
5802	CaTSNP8812	AGCTTTCGTTTTTGGCCATGGCGGTGATGGAATCGGAGCTAGGGTTAGGGTTTGGAAATG[G/T]AGTGAGAAAGGGAAGAAAATGGTAGAGATAATGTGAAGTGAGAAGAAAGTGATTGCAATT
5803	CaTSNP8813	GACCACCATCTTTGATAGTTCAAAACCCCATCGAACGTATAAGTATCTGAGGGCTTGGGA[A/T]CGAGATGCCAGTACTGGAATTACATTCAGTCTTGTCCCATTTTGACAATATATAGGATGG
5804	CaTSNP8814	TTATGGGGAACAATTCCAAGATCATTGAATGTCAAAGCATTTTCTGAAACTACGGTATCT[G/T]TTGCAAAAGCATGGATCTGATCCAGGTTGAACATCTCAGGCTTGGGTAGAGGAAAGGGGA
5805	CaTSNP8815	GGCAGGTCTTATAAAGGCTTGGTTCAGAGAACTTCCAACCGGGATACTGGACCCTCTTTC[G/A]CCAGAGCAAGTAATGCAGTCCCAGTCAGAAGAAGAATGTGCTCAGCTTGTGAGGCTTCTT
5806	CaTSNP8816	CTACAACTTCAATGGCTTTGAATAATTCACTCTGAATATTATTATTGAAAGAGGTGTCAT[T/C]TATGATTAGGGTTCCGGTTTCTGATTCTAGATTAGGATTGGGAGGATCCTCTCTGTAATT
5807	CaTSNP8817	GAAGGAAAATAATATTTTTGACATGAAATTGGACTCACCAAAGTCTAGTGAGGACATGGC[A/G]TCTCCTAGAATGAAGAATATGGAGAAAGAAAAGGTTGTGATGATGGAGTATGATGATGAG
5808	CaTSNP8818	ACCGAATTCCAGATCGGATTCATTGGGCATCCGTTGAATGCGCAACCCCACTAATCCGAG[C/T]TCTTGCATAACAGGATGCACGCAAGATGGAATGAGGCCCAAATCTTCCCCGCAGGCCAGC
5809	CaTSNP8819	GATAATTGGAAACAGCAGTGGAAAAAGTGGGTCCAAATTTGGGGGTTCCAAGAAAGGGTT[G/T]GGTGGTAAGGCTGGAAATGATCATGATGGTGAAGTTAAAGAAATGTGGGATACAATTGCG
5810	CaTSNP8820	GCTTTTCGAATTCAGGAAGGCCAGCAGCCTTCTTTTCTTTCCTTATTCTTCTATCTTCAT[T/C]GCGTTCCTTCCGTAGCCAAACTTCTACCGTTCTCATTAGTGACAGTTTGCTAGTTTCTCT
5811	CaTSNP8821	CGCCGTGTCGAAAGTGCCGAGCCAAACGCGGCTCTTCTTTCCCGGATCTCTAATCTCGGC[A/G]GCGTATCTTCCCCATGGCCTCTTCCTGACTCCTCGAAAATGAACCTCTTTAACGACGCCG
5812	CaTSNP8822	ATCTGCTAATCTCAAGGAGAAACATGCCAAAGAACTGTCTGCATTAGGCTATAGCAGTGG[C/T]AACGGAAATGAAAAGAGCAATTTGGACAATTTGGTGAAGGCCATAGCTGGAGTATCTGTT
5813	CaTSNP8823	AAACTCTACAAAACCTGGCCACTTCAAAGACTCGGATTCAAAAAGAGCAAAGCCAGCATC[T/C]GGTTTACCCCTCCTAATGTATTCAATCCTTGTGCTTCTGCATTTCAAGGAACTGTAACTG
5814	CaTSNP8824	TCGAACAGAAGCAGGAGGTGAAGGTGTACCGTCGGACTTTTTCTCAGAAGGTAATGTTTC[A/G]GTCTTTGCAGTCGGGCTCTCCAGTTGAAGAACCGGTCTCTCTATAGACTCAAATTGAGTT
5815	CaTSNP8825	TTCTGGTATGAGTCCTCGAGCATCCATCTTCTTCTTCTCCTTTGCCACCATTAAAACTGT[C/T]GACGACCACTGCGGGTTATGGCTCCCCGGCAACCTATTCCAAATGTTTTTCAACAACAAT
5816	CaTSNP8826	GCTTTTCACATGTAGCCACCATTTCATATCTGGTAGCTTATCAGAATTTTTATTATCTGG[T/C]GCTGGAACTGAATCAGCCTGAATTTGATCATTAATTGAATTGCTAAGTTTCCCATCAGCT

5817	CaTSNP8827	CGGAAGCCTAGATGGTCCTATCAAATATTAGAAAAGTATTGTGGTTGTAAACGATGTATT[G/A]TTATTGTTTATATCTTGGCTTCTATTGAGTTGCAAATAGGAACGGGAATGGATGTAGCTT
5818	CaTSNP8828	AAGATTGAAAGTTTTAGTTGTTGTGGAAATGTGTGGCAATGAAGAAGCGGGCGTGCTTAC[G/A]AGGAAAGAATTCATGGTGAATTAGGTTAGGATAATTCGATGATGTAGTTCCGATTCCACT
5819	CaTSNP8829	GCTCATTTTTCCACGGAACATTGCAGATGCTGTTAAGTATCTGCCATGTCGTGGATCTGC[C/A]GCACACATCATGTTTCTTGCGTCCCACATTTGTTGTGTGAGTTCTGGAATTGTTAGGGAA
5820	CaTSNP8830	GAGATTTATAAAGTCAAACATGTGTTTCACTAATTTTGTCAATCAAGATAAATCCATTCG[T/C]ACACAAAGCGATCATAGTTTAACCATTGAGGCTTTGGATGAAGAAGGTAGCCCTATTCTA
5821	CaTSNP8831	AGAAGAAACACCGTTTACATCGATAACTCAGCTTGTTCATCATGGAATGATTTTTGTGCC[G/A]ATTGGGTACACATTTGGGGATGGAATGTATGAGATGGAGAAGGTGAAAGGTGGATCGCCC
5822	CaTSNP8832	GAAAACAACAGAAAGGCTTGTGGCCTTGTATCAAAATGAACGCAGACAGGCCTTTTTGCC[T/C]TCTCCAAGCTTAGTTCATGAAACTATTCATTGGGTCATCATGATGTACTTTCTTCATCCT
5823	CaTSNP8833	CAACACAATTGCAGAAGGGAAGTATCGAACAGCTGATCTTGGTGGCAGTTCAAAGACAAC[G/A]GAATTCACAAAAGCAATTATTGATCAACTTTGATATTTTTGCTTTGAAGTCGACAAGAAA
5824	CaTSNP8834	AGATTGTGCAATTGGATTCGGCAAAAACGCCATAGGTGGAAGAGATGGTAAAATTTACGT[G/A]GTAAACGACGACAGCGACAACGACGCAGTGAACCCGAAGCCGGGTACACTCCGTTACGCC
5825	CaTSNP8835	AATGATGTTAGGGTTCACGTTCATGAAGAAAGGGAATGAGGGAGGAATATTGGTACGGAA[C/G]ACGGTACTTTTGTATTTCTCCATTCGTGTTTTGAAGAAATTATCTGGCTTCTGGAACCAG
5826	CaTSNP8836	CCCTCCTGCAGAAAGGCGGTACAATTGCTTGATTCCTCCTCCCCCAGGATACAAGGTTCC[T/A]ATTAAGTGGCCACAGAGCAGGGAGGAGGTATGGAAAGTAAATATACCACATACTCACCTT
5827	CaTSNP8837	AGAAAGGTAATCGTGATATACGACGAGCTCCAAGCTTTAGTGGTCCATTAATGCTTCCTA[A/C]CCGAGCTTCGGCAAATAGTTTATCAGCTCCAATAAAATCCTCTGGAGGATTTAGAGATTC
5828	CaTSNP8838	GTCCTCTAAATATAACAACTTCTCTCTCTCTGATTGTTGAAGTTTAGAGATTGGGATACC[A/G]GTCCACTTGCTCACAATTTCAGCAATATCATTCCCAGTAACTTCCTCTCTCAACATAGAC
5829	CaTSNP8839	ACAACAGCAACAATCAGGTGATTTGAAAATGAGAGCAGCCACACCTCCATGTTCCAAAGA[C/T]AATCCTTCATCAGATGTAAATCCTTCTGTAGCCAAGGATTGTTGAGAAACTACCCGTCTT
5830	CaTSNP8840	GCTTGTTATAGAAATGGATTAGAGTTGCATGATAAAGAGGAAATAGCGGAGGAGATTGCA[A/C]GGTTGAGGAGTGCTGTTAGTGTGCTTACGGAGGCAAAGAAAAACTCGAAGGGTGCTGCGG
5831	CaTSNP8841	GGCTACGACAGAAAGAATGGAATGCATTCTCTTTGTCCAGCAGTGTGGTCGAAACACAGA[A/C]GGTGGCTGTTGATGTCCATGATTTGCTTGAATCCCGTAGCTTGTTCATTTGTGGATCTTC
5832	CaTSNP8842	TTTGAGATGCACTGCTTTGGAAGCTTGCATTCATCCCTTCTTCGATGAACTGAGGGACCC[A/G]AACACTCGCCTTCCCAATGGTCGCCCACTGCCTCCGCTGTTTAATTTTAAACCTCAGGAA
5833	CaTSNP8843	ACAAGCAGAATCCCAGACTGTCCCTGAATCTAGCATCATTCAAAAGGCTAGTAATGCATT[G/A]GAAGTTTTAAAAGAAGTTCTTGATGCTGTTGATGCTCAATATCCTCAGGGAGCAAGTGAT
5834	CaTSNP8844	AGCTTGGTGTGGACCTTGCAGGTTCATTTCTCCTATAGTTGGGGAGCTCAGTAACAAGTA[T/C]CCTCATGTCACAACTTATAAGATTGACATTGATCAGGAAGCAATTCGAGACACGTTGAGC
5835	CaTSNP8845	TGCATAGCCGTCCGTTGATGGGACAAAGAATGAAGATTTCTTGATTGATGCCATTTTTGT[G/T]GCCACATATAATGGAACCTGACACTGTACATCAATCCCACTCTTCTTGTATTCAACATAG
5836	CaTSNP8846	CATCATCTGTTCCAAGTCATTCAGTTAGTGAAATGATACAGAACAATTCTGCTGATTCGG[T/C]CTTGTCTGAAGCATTTGCCTCTACTGTGAAGACAACTTCAACAACCATTGCAAAGGCTCC
5837	CaTSNP8847	TCACAAATTATGGTTACAAGGGAGAAAAGTTTTGGCACTTTTGATTCAGAATAGAGTTTC[T/A]GAGGTTTTTGCTGTTGATATTCATCCTGGTGCTAAGATTGGAAGAGGGATTTTGTTGGAT
5838	CaTSNP8848	TCCCCAAAACTGTGTTGATCAGAGTACATATGCTGACTACTATTTCCGCATCACAAACAG[C/T]GAGCACAAGACTGAGCTCAAAGAAAAATTCAAGCGAATGTGTGACAAGTCAATGATAAAG
5839	CaTSNP8849	ATACTCTCTATCCATAGACGGTCACAGCTATGTGGATGGTTTAGTAGGCGGAGGTCCAGA[C/T]GATTCTCAACAGTCAGTCCCAAATGTTGGTAAGCCCAGACGTGGGAGGAAAAGCAAATCT
5840	CaTSNP8850	AATTCTGATGGCCCTTTTACTTCTGCGGCATGCACTCTCTGTAACAGATGGTGATAATTC[T/C]GATGATGATCCGTCCAATTTTTTCTCTCTTTTCTTGCTTCGAGCTGCTGGATTTCTTTTA
5841	CaTSNP8851	CCATTGTTTCTTAAACCGAAACCACCAGGTTGTTGGGAATTCATCAACTTCTCTGTTCAG[A/G]TCGCCCTCGACGTGTCAATGGTTTATTTCTGGGGTTGGAAATCCTTAGCATACCTCATAC
5842	CaTSNP8852	AAAAGCTGAGAAGGTCAAAGGACGAGACCCCGATCGATGGCGCAGAGACCCCCTCGGTAA[C/T]CTTATGTTTCGTAAACTCGTAGGTTGCCCTGGTTGTCTCTGTCATGACTACGATCACATT
5843	CaTSNP8853	TTTTTGGTGGTTGTGGGGTCCCCATTTTGGAAATGATTTGTTTTGTGAATGCTTTGCTTC[G/T]GTTGAAATCGTTCCATAAGGGAGTTATTGGAGGTGGTGAAGTTGGGAAAACTGAGGCTGA
5844	CaTSNP8854	CAGAGGCACATCTTTTAGCTCATACTTGATTGTCCTTATTGATCTCTAGTTCGCCTTCAC[T/C]GGTTTTGTTGGGATGTGGGCTCTGGACCTTTTTATCATCACCTTTTGCATCTCCATTTGA
5845	CaTSNP8855	AACTCCACCGCTGCTGCAGTATTTTGGAACACTTTTAACGAGGGGAAAGCTGAATGCCTT[T/C]GAATCATTAGAATTGTCTCGGTTGGTTGTTAACCAGAACAAGAAAAATCTGTTGGAAAAT
5846	CaTSNP8856	ACATGAAAGTACAGATGGCTTATTGATTGAGAAGGATGAATGTCAGAGTCAGATAGTTGG[G/A]ACAAGCTCAGAGACTGTTGGAGATGATGATTTGTCTGTCCCACATTAGCTGAAGCCATTC

5847	CaTSNP8857	GGCGCAATCGATTTTCAACCATGAACCTGGCAAGGTGGATAGGGCAAAGGTGGCAGAAGC[C/T]ACCTCTGATATTCTAAGTGCAATTGGTGTTGATGAGAGTAAGGGCTTTGGCAAGTACGTT
5848	CaTSNP8858	ATAACTTCCAACAGAAGCTGCAATTAAAATGGGGCGTCTTCTATATCTTTCATCTCTCAT[T/A]ATGTCAAAAGAATCTTTAGAGCATTTATCATAATAAATATCACGTGCTTCACGTGCAAGC
5849	CaTSNP8859	TCTGGAACCTGGAACCATGGATTCCGTCAGATCCGGTCCGTTTGGTCAGATCTTCCGTCC[T/C]GATAACTTCGTTTTCGGTCAATCCGGAGCCGGAAATAATTGGGCGAAAGGTCATTACACC
5850	CaTSNP8860	AATGGCGTTGAACCTAACACTACCAACCGTATCACATTTCATCCTCAACGAAGCTCCTAC[C/T]ATTTGGAACTTCTTCCTCACTTGCTTCACACCACCTTACCTTTACCTTCTCCTCAACCTC
5851	CaTSNP8861	GCGGAAAACAAGGCAAAGTCTTCTGTGGCCGATCCCAATGACACTGACAACAACAACGAC[A/G]ATGATAAAACAAAGACCGATGCTGAGGAAATTAAGCAAAGTAACGTGGAAGGTGAAAATG
5852	CaTSNP8862	TCTAGAAGGTTCTGAAATCGAGGTGGTTGTTGTTGCGGCAGTTACGGTGGTTGTTGCGGC[A/G]GTTACGGTGGTTGTTGCGGCGGTTTGAATGTTGATGCATGGTGATGGTGTGATGGTTTCG
5853	CaTSNP8863	TCCTATCGAGAGCCAGTTCATTTCCAAGCTGGCCGATCAGCTGAATGCTGAGATTGTTCT[T/C]GGTACTGTTCAAAATGCGAAGGAAGCCTGTCATTGGATTGGATACACTTACTTGTATGTT
5854	CaTSNP8864	TCAAGTCACCGTTGACCGCGTTGCTACCGGTACCTTCTTTTATCAGTGTGTTTATCTCCT[C/T]CTTCACCCCACCGATCAGATAGTGGAAACCAATGCTCACTGCTGCAGGCATTTCGGTTAT
5855	CaTSNP8865	GAGTCCTAGCGCCTTACCGTGGACTTGGTATTGGAACAAAGCTGCTGAATCATGTTCTTG[A/G]TCTCTGCTCCAAGCAAAACATTTCTGAAGTTTACTTGCATGTGCAGACAAACAATGAAGA
5856	CaTSNP8866	TCCTACAAGCATGATTACCACGTAGGCATCGGTGTGATCTCGAAGCTCCTTCAACCATCT[C/T]TCCACGTTTTCAAATGTCACGTGCCTTGTAGTATCGTACACAAGCAGTGCACCAACGGCT
5857	CaTSNP8867	AAATACCCATTATGTTTCTCCAGCTGAATGGGTCTTGGAGCAAAGTATAACCAAATGCCA[A/G]CACAAGGCATGTCTTCAGGTGTCCAAGAACCTGATAGGTGACTGGAGATGTCTTTCCAAT
5858	CaTSNP8868	AGCACCTGGTGGATTAATTGAAGGTGTAGCGGATTATGTGAGATTGAAAGCTAATTATGC[T/G]CCTAGTCATTGGAAAAAAAGCAGGGGAAGGTGATAAATGGGACCAAGGTTATGAAGTTAA
5859	CaTSNP8869	ATTGCATGCTTGGATTGATTATGATTCAAATTCGAGGCGTTTGGAAGTTAGGTTGAGTCG[A/T]TATGGTCATTCGAAGCCGGTCGATCCATTGCTCTGGCAAACAATTAATTTCTTTAATTTG
5860	CaTSNP8870	TTTGATGGTTGGACTTTCTTCCTTTCCTGGAACATATTCTCATGAACAGTGGCATATCCA[A/T]GTCCGCTTTTTTCCACAGGCCTTGGCCAATGTGAAGGAGATTTATCTTCACGCGGTCCAG
5861	CaTSNP8871	TCTGGCCCACAAGTTCCGATGTACGGTGCACCTCCCTCTTCCATCTACCCTAAGATCGGT[C/T]CTCACCCTCATGCCTCCGCCGCTGCCCCTCCTCCGTTACACTATCATCACAATTCCACTC
5862	CaTSNP8873	TGCCACCTTTGCTGCCATAGGAGGAGTCTACATAGGTGTGGAACAGTTGGTGCAGAACTA[C/T]AGGAACAAGAGGGATCTTGTAAATGGCGCCGTTGGTGGATTTGTTGCTGGTGCAACTATT
5863	CaTSNP8875	GAAATTTTCCCTAGGAGAGGAGAGCACAATAGTTAGTTCTAAAATCGCTGTGTCTCCTCG[C/G]GTTGAAGGTTCTTCTCCCCCGCTAGTTTTGGATGGACAAGACATGACTTTAGTTTCAGTT
5864	CaTSNP8876	GATAATTCACACTTGTTGCTTCCCCTGCGAAAAACAAGTTATCTACTGGGACCCGTAGCA[T/C]CTCATACAGTTCGTGCGGTTTCCCAACTTGATCATAGCTATATGAACCCAGTGAATCTAT
5865	CaTSNP8877	ATACATGTTTCCACTATCTACTCCTGATGGGCCGAGATTACCAGAGTTATTGCCAGCAAA[C/T]CCTTGAAAACCTTCGGATACTGCCAGGTTGTTGAGTGTAGAACTACTTTTGTTACCAGAC
5866	CaTSNP8878	TGTCGATGGCCGTGCTAAAGACACCATTGTGCTTTCAACAGGTGAAAATGTTGAACCGGC[G/A]GAACTTGAAGAAGCTGCCATGAAGAGTAGCTTAATTCAACAAATTGTTGTTGTTGGTCAG
5867	CaTSNP8879	GATTTTGGAGTACCATCCGAAGATGTTAAAGGAACATCTAGAAGGAGCAGAGCCAACAGG[A/G]TTCATGTATCCAAGTGCTGTGGACCACTTCAAGAAGCAATTTGCATACCTTGAGGAGCAT
5868	CaTSNP8880	TCACATCGAGGTCAAGATTAATTATCAGGATTAAGCTTCAGTTCCTGCTTCCTTTATTTG[G/T]ATTGATGTCTTGGCTAAACTTGCTTCCAGTTCATTCAACTCATCCAAGTCCAATTCATCA
5869	CaTSNP8881	CCACCAGCGAGGTGATTCGAGTGGCGAATCGAAGTAAGGGTCGAGGTTGAATACATAGTG[A/G]AGAGAGGGTTCCTTGAAGGCACTGAACCATGATTTGCATACAAGCATCGCACCGCGCCAT
5870	CaTSNP8882	AGGAGGATTAAGTTTATCAGTTTCCTTAGCATACTTTTGTTCAATCGAATTTCCATTGCC[A/G]CTGTTGTAGCAGTCAATTATTGATGTCGTTTGGAAGCAATTAGACCATTCTCTATACCGA
5871	CaTSNP8883	ACCTAACATGTCCTTAGATCCATAAGCCCTATTATCAGCACCACCGTGAAATGAAATCAG[C/T]GGAGCACGCAGGTTATCACTGGTACCGAAGGGGCTAGCCTCTGGATCCCCTGTGCTAAAG
5872	CaTSNP8884	ACTCGATCGATGACCTCTTCTTTGGTGAGATGATCGTCGTGTGAAGACATGAAGCGAAGG[C/G]AACCATTCCATGGTTGGAATTTTGGAGTAGCTTGGAGTGGGACGCGAACATGGCGGAGTA
5873	CaTSNP8885	TGAGAGATCGCGATGCTTTAACCTGGAGGCCAAAATTACAGAACTCCAAAAGTTGTTGGA[G/A]TCAATGCAGTCTGTAGAAGATCAGGTAAATGCTCTTCGGGAAAAGAAGTCTGCTTTAGAC
5874	CaTSNP8886	AGCTTATGAAGAAGTTATGCAAATTGGTTCTGCTTTAAGAGCATCTGGTGCTGAATCTGG[A/C]TCTAAAATTGGAGTTTATGGATCTAATTGCCCACAATGGATTGTAGCAATGGAGGCTTGT
5875	CaTSNP8887	GGTTGTAAGGCGCAACGGAAGACAATGAAGACCTTTGGGCAGTGTTTTTGCTGTCAACTG[C/T]GTTAAGAACAAAGTCTGCTTTTTGAGCACATGTAGCTGTTCTTCTGTTGAATGGATCATA
5876	CaTSNP8888	ATCATCTCTTTCTCCAACTAACTGCGCAAGCTGTTCGCGAATTGTAAGTTTCTGAGAGCT[G/T]CCATCACTTTCATATCCATTAATCGATGAACTTGTAGATGTAAGTTGCTCCAAAAGATCC

5877	CaTSNP8889	CCTGAGGATGCGACCAAGGGGGACAGCAATGTCACCTCTGAAGACAAGGAGGAAAGTTCC[G/T]TAGACAAGTCTTCTGAAGATACAAAGACAGAAGATGTGGGTAAGAAAACAGAAGACGAAG
5878	CaTSNP8890	AGGAAGGAGACTGTTGCAACTCCAACCATGAACCGTTCAAAACCTTAGGGGCTTTCTGCT[G/A]GAACATGATTCACAATATCAAAGTTGTTCACAGGTTTGGGCCTCCACTTAGTCCTACCTC
5879	CaTSNP8891	TGGAGATTTCCAGAAGGGTACTCCAGAAGAGCGCAATGTTCGGTTTGGTGTTAGGGAACA[T/C]GCGATGGGAGCGATCTGTAATGGTATTGCTCTTCATAGCCCTGGGTTCATTCCGTATTGT
5880	CaTSNP8892	GAGGAGGAGATCGTAATTCCGGTGGATCGAACCTCCAAGTGAATTTTCCACCGGAAGAAC[C/A]GCGCGATCGGCGATCCAAAGCTCAACCGCTTGAAACGCAACTTCAAATTGGTCACACGGA
5881	CaTSNP8893	TGTTATTGAGTGGTACAAGACATATTTGAAACAGTCGTCTCCTAAATGTCGAAGACTACT[C/T]GTTCGGGTATGGGGTTGCAACACAGATATGAAGGATGCCGAAGCACAGCCAGAATCTGTG
5882	CaTSNP8894	CAATGTGGTTGGAGAGTTAGAATCTCAAATTGATAGCTTTGAAGCTGAGCTTGAAGGCCT[C/T]ACCGTCAAGAAAGGCAAGAACAGGCCTCCTAGATTGACACATCTAGAGACATCCATTACT
5883	CaTSNP8895	GGATTTTCCAGATGAGTATAAGAAAACCCATTTTAGCCTTTGGACTCTTCTTGAGTGGTT[G/A]AGTTTGATTTTGATCATTGGTGCATTAGTAACAACTTTGAGTGTTCCTAATTTGAGGGAG
5884	CaTSNP8896	ATCTGCTCGTCATTGGATTGTTAGGAACACGAGGAGTGTGAACCGTACCGGGTATCTAAC[C/T]GGTTACAAACTAGTTCCTGGTGCAAATTGTTTACCTTTAGCTGGATCAGAGGCTAAGTTT
5885	CaTSNP8897	CCTGGATCGAATCGTTTGCATAGGAACAACCTTCATCCATGCAATAGCAACATAGCTAAA[T/C]AAAGAACAAGCAGTCATGTGACACATGGTGAGAAAAATAGGGTATTTAAACCCATAATTG
5886	CaTSNP8898	TAACAAAGAGGACTATGAGAAGTTCTGGGATAACTTTGGCAAACACTTGAAATTGGGTTG[T/C]ATTGAAGACCGTGAGAATCACAAACGTATTGCTCCATTGCTTCGGTTTTTCTCATCCCAA
5887	CaTSNP8899	AGTAGGCGACTAGAGGTATGGAGGTGAAAGGGTAGATAGTTGTGTTTGCATAGGCAAATC[G/T]CTCCAGCCACTTCAGCTTCCCTTCCTTGTAGCCATACCATAAAGGGCAATGATGACTGAA
5888	CaTSNP8900	CTACACAAGCTGCTGATCGCATACATCAAGTATTCAGAATGCAGTCCTTTCAAAGGAAGC[G/A]GTTAACTCAGTATGAGGATGACGAGTTTGGTTTGTCAGATCAGCGGGCTCTTTCCCTTCT
5889	CaTSNP8901	TGCCTCGAGGTCTCCAAGGTACAAAAATTTCCATCCCCTAAGACTTGCTCGAACAGCAAG[G/A]TCCATATCCTCAACTGTTGTTCTGTCTTTCCACCCTCCTGCTTCGTCGATTGCAGCAATT
5890	CaTSNP8902	GAGAATTGTATCTAACGGATTTGAGTCCGATGAGTTTGAATCACACGGTTCAACCTGGCA[G/A]CTGAGATGCCACCGGAAGGCTCTGCCGGGATGGTCGTTGACGGCGAAATTGAGGAGACCG
5891	CaTSNP8903	AGAAGAAGGAGCTGAACTGGGCCCTTCTGTTTTATTCTTTGGATGTAGGAACCGTCAAGT[A/G]GACTATATCTATGAAGACGAATTGAACCAATTCGTTAATGGTGGCGCACTTTCTGAGCTC
5892	CaTSNP8905	TTGCACATGAAACCAGTGTCTTCTTCCCCCTGGTGTGAAAGTAAATGTGGTTCTCACTAT[G/T]TGTCAAATGCTGAAAAATCCCACTATTAGAGTTCCATCGGACTTTATGGCAGGGAATTAC
5893	CaTSNP8906	TGCTAGGATTAATGGTGTTAAGAGGTTTTTCTATGCCTCTAGTGCTTGTATCTATCCTGA[G/A]TTTAAACAGTTGGAAACAAATGTGAGCTTGAAGGAGGCTGATGCCTGGCCTGCTGAGCCA
5894	CaTSNP8907	CAACAGGAATGTCTTTTTGTGAGTGCCTCTCAGGACTCATCCCAGACAACTTATCAACTG[G/C]TTCTTTATCTCTAGTCCAACTTGTGCTTGTGTTCAACCCTGGAGAAGTGTTACCCTTACT
5895	CaTSNP8908	AGTTCATCAGAGTCCAGGTAGCCAAGTGAGCTTCAATTTTGTGGTCTTCACAGATCCTTC[G/A]AGATGCAAAACACCACTCAACCCTGTGACATTCCCATCTTGGTCCTTCTCTACTTCCTTT
5896	CaTSNP8909	TACGACTGCCGACGAGGATGCAAAGAAGAAAGCTCGTCTTGCTAGGTTTGCATCTGGTTC[G/A]AAGACTGACCCTGCAGAGGAAGATAAAAGGAAAGCGAGAGCACTTAGGTTTTCAAATTCA
5897	CaTSNP8910	AAGATCAAGCTTCTGAGGGAAAGTGTCCATGTGCCTCAAGTCACTTACTCTAATCCCATT[A/T]CGGTTAACCTTGCTGGAGTAGCACGGGCCAATCCCTCTCTTGGTGGTGCCAATGAAAGAT
5898	CaTSNP8911	TAGGCAAATGAGTTTCCCAAGAATTTTGGCAGCAAGCAGATCAAAATCCAGCAGACGAGT[T/C]CGTTGTTCTTGTTTCCCGAATTACAGGAAACCAAGTATGCTCCAGTGGTTGTTGAACTCA
5899	CaTSNP8912	TGAGAAGGGTTATGAAAAAGATCCTGCTGTTGTGGCTAAAGAAGCAATTCAGGAAGCTTC[G/A]CGGAATGGTTCAGATGTGGTTCTAGTTGATACAGCTGGTCGTATGCAGGACAATGAGCCA
5900	CaTSNP8913	ACACGAAGCTAGCAAAAAGAAGAAAGAGCAAGCCAAATTTCTTAAAGAGATTGTGCTACG[A/C]GAAAAGAAAATAACTGACAAAAGCAACAAACTTGACAAATATCAACCTGAGCTTCTCAAG
5901	CaTSNP8914	TTTAGTCCTACCACCATTAATAGTGGACGTGGAGCTGAATTTGAGGGTGCTGTTATTGCT[T/C]TGTTCCATTTGTTGATAACTAGAACAGACAAGGTCCGTGCTCTTCGCGAAGCATTTTACC
5902	CaTSNP8915	TCTTCGCGCCGGATTTGATCCCACAAGGTGTATCTTTAATGGAAATGGAAAGATCTTGGA[T/G]GATCTGGTTTTGGCTGCCCAAGCAGGCGTGTTTGTCAACATTGACAGTGAGTTTGACTTG
5903	CaTSNP8916	ACACAAAATCATAACTTCTAAGGCTGAAGCATGGATTCAAAAGAAAACTATGTCATGGCC[A/T]TGGAAAGTAAATGATAGGGAAGGATCCGAGTCAAAGAACGTTCGTGTTTCTTGGCCTTGG
5904	CaTSNP8917	TGAGTTGAAGATAAGTGTGTTTAAGACTAGATTCGAATTAGGGCCATCAAGCGCAAAGAT[G/A]GATTTtCCTTTTGACTGAAGAAACCATATCACCATAAACTGATACAGTGATTGTCCTAAG
5905	CaTSNP8918	ATAATGTCCGCGAAAAGCGGGACCGATGTTGATAATATCGAGTGAAACCGGTTTGAATGA[A/G]TGGGTCCCACTTATGAACTGGTAAGCTCGGTAATCGTGAGTGTGAATGTGAGAGGTTTGA
5906	CaTSNP8919	AGCTCATTGAGTGCCTTTGTCATCCTTTTCACTCGCCTGCGGAGTGTACTGGCTGGTTGA[T/C]GATGGTGAGTAACCAGGTTGACTTGGTGAGTAACCCGTACTTGGACTAAATTGCGGTGAA

5907	CaTSNP8920	ATTTATGACAGTGAAGATGTTAAAAGTGCTACTACATCTACAGCAGCATTTGAGCAAGTT[C/G]AAAGTTTAGGAAATTCAGGAAATGATTTTAAAGGTACAAAAGTTATGGAAGAAGAACCAC
5908	CaTSNP8922	CCCCGATTCAATTGTATTCAACTCTTTTCAGCTCTATGGAACTCCTAGCTACTGGATGCA[A/G]TTATTTTTCTCAGAGTCAAATGGAGCAACACTTCTGAATTCATCACTTCAAGCAACCGCT
5909	CaTSNP8923	TGTTGCATCCAAAATCCACTTGGACCATTATTGCGTCATGGTTGAAACATGACCTAAATC[A/G]TGGCTTATTGCGTCATGGTTGAAACATGACCTAAATCATGGCTCACTCTTTGTCCTTTCT
5910	CaTSNP8924	TGGAATTCTTATTATGGAAGTGATTACAGGCAGGAATCCAGTCGATTATTGCCGTCCTCC[A/C]GAAGAGGTGAATTTAGTTGAGTGGCTCAAGAAGATGGTTGGTAACAGAAATCCAGAGGGA
5911	CaTSNP8925	TCAAGCTATGTATTACCAGAGAGAAAATCCAAGCTTTCATCTACAAAGTGTGGACGACGT[G/A]ATGAAAATTGTGGGAGAGGCAAGAAACCCACCTCCAGTATCCAGACCTGTCAGAGGCTTT
5912	CaTSNP8926	AACAATTGCAACCAATGCCAAATATGATAAGCCTTCAACAGCACCTAATAACCCAAAAGG[C/A]CCATTAGGAAGACCAGAACCTGTTTTAGTTTTGGTGTATAAAGACCAACCTACAATTCCA
5913	CaTSNP8927	CGCATCAACCTTTCTAGAAATCCTAATGGACCGTCTCCAAAACACCAACGCCGCCGCCGT[A/C]GCTCTCAAATGCTTAATCGTCGTTCACCACATCATCAACCACGGAAGTTTCATCCTCCAA
5914	CaTSNP8928	GACATGTAGTGGGGTGGGGCCCATTGGAGTAAGGTACTCTTTCATTCCTCCCCTTATAAT[A/G]TCACACTTGAGTTCTAAGAAACCACTCATTTCTATTGCACCGCAGAATGAGATTTCGCCA
5915	CaTSNP8929	CTGATTAGTCAAACTAGCAGCAATACCAGCCTTACAAATTTCCATCAAATCTTCTCTATT[G/A]TAAGCAATTCCACCGCCGGTACCACCGAGCGTAAACGCCGGTCGAACAATCAAAGGAAAC
5916	CaTSNP8930	TTCAGCAAAATCCCCTTCATCATTATCCACCTCTTCCTCCTCCTCATCATCATCCTCATC[A/G]TCGTCCTCATCCTCATCAAACTCAACTACATCCTCTTCATCATCACTATCCTCAATCTCA
5917	CaTSNP8931	TGCCAGTTCTTATCATTCATATTCACAAAGTCCTCCATATGATAATCAATATGAGGATCG[C/G]CGCTATGGAAAGCAAGCAGCGGTTCTGACGAGAAAGCCTGGTTCAGATAAAGTCCGTTAT
5918	CaTSNP8932	TATTAGCTTAACTGTTGGAGTCACCTGTGCCAACACCGACGGAAAAATTGTCCTTGAGGC[G/T]TTGATTTTGACATCTGCGGTGGTTTCATCTCTTACTGGCTATGCCTTTTGGGCTTCCAAG
5919	CaTSNP8933	TTGCCCTGAGTTTGAAGAGCATTATTTTGTGAACTAGAAGCTGTGGAAGACTGTGATTTG[G/A]CATTAGGAATTCCAGTATTCTTGTCCTTGGACCCTGAAGAAGAAGAAGAAACTGGTGGAG
5920	CaTSNP8934	GCGGCGAGAGTGACCTTCAATAACAGACACTATGACTTGCCTCCTTGGTCCATAAGCATC[C/T]TACCTGATTGTAAAACTGATGTATTCAACACTGCAAGAGTGAGGTTTCAAAATTCAAAAA
5921	CaTSNP8935	TGGGAATTCCTTCATGAGCATCGATATTAGTGTTCCTTTACCGACTCCAGAAGGACCACT[C/G]ATAACAACAGGTCTCTCAACATTGCCTATCACACCCTTACTCCATGCAACAACCTCAGTC
5922	CaTSNP8936	ACGGTGGTGCGGCGATTCACCGGCTTGGATCTCTTCAACGAAACCGCCTTCGCAGTGAGG[A/G]CATACGATGATGGTGTGATCTGAAAGGTTAATGAAGCGGGTGCAGCTGTAGCACCAGTAC
5923	CaTSNP8937	TGTTCCCATAGCAGCCTTCATTTTTTCAGGGTCTTTGGAAACAAGGAAAGAATCAGCACC[A/G]AGTTTGTCAATGGCCTCACTTTCCTTGCTTGGAGAGGTACTAATGACAGTAACCTTCAGC
5924	CaTSNP8938	AAATGGTGAAAATGAAGCTGAGAGTGCTTACAATGGTGGAGCTTTACATCACATGGAAGC[T/G]TTGGAAGAGGTTCTTCCTATTAGGAGAGGCATCTCAAACTTTTATAATGGCAAGTCCAAG
5925	CaTSNP8939	TGGGGCCAGCTCAGGCCTCAACCGTCTACTTATGCTGTTGATATCATTCCCCTGACGAAC[C/T]GCATGTAGCAGGAGGAAGAAATTCATAGCCTCTATACCCATCTGAAATCTCCCATTTCTT
5926	CaTSNP8940	TAGCTCTGGCTCAAGAGGTGGATTTGGTAGAAGTATTCTCTCTTCAGCTAAGAGCAACTC[T/C]TCCAGACGTTTCAACGATACATTTGCATTGACGACCTGAGTTATAATATTCGGAAGCATG
5927	CaTSNP8941	GGTTGCAAACCCACGTCAATTCAAGATCCCAGATTGGTTTTTGAATAGGAAGAAGGATTA[T/C]AAGGATGGAAAGTTCTCTCAAGTTGTTTCCAATTCACTTGACATGAAGTTGAGGGATGAT
5928	CaTSNP8942	ATACCTTGACAGCTATATCAGTACAATTGGAGTGGACTTTAAAATTCGCACTGTTGAGCA[A/G]GACGGGAAGACCATTAAACTTCAAATTTGGGACACTGCTGGTCAAGAACGTTTCCGGACT
5929	CaTSNP8943	GAATCGAAAGTTCGAGAAGACTATTCGTTATGCTTCAAGGAAAGCGTATGCAGAAACGAG[G/A]CCTAGAATTAAAGGAAGGTTCGCAAAACGCACTGATATGAATGTGAATGTGAATCTGATC
5930	CaTSNP8944	TAAGTTGGGGGATCTAGTCCTGGGAGCCATGTGGGTCGCTGTTCCGCTGCTGCACGAATT[T/C]GCCTATCAGGTGCAGTGGCTCCTACTCCTGCAGCAGTTGGCCAAAGCCAGGAGTGTCCTC
5931	CaTSNP8945	GAGTGCATCGGAAACGTCTTTGTTGTTACTGGATTGAAAGATTAAAGTTGCAATGTTAGT[A/G]GCGGTTTGAATGACTTGTGTTTGTGCTGCTTGGATCTCAACCTCAAGAGCTTTGTACATG
5932	CaTSNP8946	CATGACTGCTACTCTGTTTCTTACGGTGGGACTATGGATTTTTGGAGGGGTTCTTAATAT[C/T]GATGCTGTAACTTCTGCCATTCTTGGATTAGCTGTACTCCTCATCACAGGGGTTGTAACA
5933	CaTSNP8947	TGTTTACACGGTCGTAATAGTAAACTGTACCTTCCCGGAGCCGTTAAACGCCGACAACTC[C/A]GGCGGGAAGTTGATTCTTTACGCTTCAACTTCCGGCGGCGGTGATACAAGCTTAAACACC
5934	CaTSNP8948	CGGCATTCTTGATGTGGGCTATCGTTCAACGGAGGAGGCTTGGGCAGCAGGTGGTAGCGG[T/C]GTTCTGTTGAGAACTAATAATGGTGGCAAGTCATGGATCCGTGACAAAGCTGCTGACAAC
5935	CaTSNP8949	GGACTTAGACGCGTTGATCACAGTAACAACAGACGAAGACGTGGAGAACATGATGGACGA[A/G]TACGACCGCGTAGCACAGAATGACAACCCACGATCGGCTCGGCTTCGTCTCTTCCTCTTC
5936	CaTSNP8950	TACACCCTCACCAATTCCCATAAAGGCGCGCATAATAAGCAAATAAGGGAGTCCAATTCT[T/G]GCAGCAATAGGTGTTAAAACTGTTGCCATTGACCACCATACAACTCCAAAACCTAGCACT

5937	CaTSNP8951	AAACTCAGATAATGGTGTTCTTGTGAGAAACAGTGGAATATGCCAGGAAAATACTCTTGG[A/C]CAGTACTTTGGACTTGGTTCCGAAACACGAGGAACAGCCACTGATATTCCGGATCCTTTC
5938	CaTSNP8952	ACAGCTTGAAAAAGACTATGGCACATTGAAAGCTAGCTTTGATAAACTCAAAGATGACTA[T/C]GAACATCTTGTTCAAGAGAATGACAAGTTAAAAGAAGAGGTGAATTCTCTAAAGAATAAA
5939	CaTSNP8953	TCTTATCAAGGCTTTTGCAGTGAAAACCAATGATATGATGCTGGTAATATACCTATCGTC[C/T]CTCATTAGAAGTGTAATTGCACTTCACAACTTGATTAACAACAAGATGCTCAACAAAGAA
5940	CaTSNP8954	TAGAGCACGCGTGACGCCTAACTGACCATTCAAGTAACCATCATCAACAAATCCGCCAAG[T/C]GACTCAATTCTCTTCCTTTCTTTGATGCATAAGGGCCTGTGATCTTTGGACATTTCTATA
5941	CaTSNP8955	TGAGAGGGCCGTGTGGAATGATACTGCATTCCTTGCTTCAATATATGTGATGGACTACTC[G/A]TTGCTGGTTGGCGTGGACGAAGAAAAGCATGAGCTAGTTTTAGGCATCATTGATTTTATG
5942	CaTSNP8956	ATCAGCAATTTTTCTCTGCAAATCCATTGGCAATGTCGGGTATTGCTCACATAGGTCCCC[G/A]TCAATAACATCCTTAACAGGAAAATAGGCAGATCTATAAGCCATGTGATCTCTTCCACAC
5943	CaTSNP8957	GAGGAGGGTGCTCGACGAGGTCCCTCTATGATGCCTGGTGGTTCGTTCGAGGCTTACAAA[C/T]ACATAGAAGATATTCTTCTCAAGGTGGCAGCTCAAGTCCCTGATAGCGGCCCTTGTGTGA
5944	CaTSNP8958	ACCTGCCCAGTCTCTAATGGGTGCATATCCAAATGCACCTAATCAATGGACTAACTATGG[A/C]GCACAGCCTCAAACTTGGCCGGCGGCCACACAAGCACAAGCACAACAATGGCCTGCTGGT
5945	CaTSNP8959	GGTGCTTGGGCCGCCCGGAAATTTCTTGTTATCGTCCCAGCCTCTTACCCATGGCTGCCC[A/G]GGTGTTGCTCTTGTTTCAGGAGCAGAATGGCTGTTAAGATTCTTCATAACTCCTTCTCGA
5946	CaTSNP8960	TTTCCCACTCGTTTCGTTGTCGCTTCTTCAACCTTTGCTGCACTTGATTAAGATGAACTC[A/G]GTGAAAATGCAGATTGTTGGGTTCATTTTATCACTTATGTCATCCGGGTTTGGATTCTAT
5947	CaTSNP8961	AACCTTTGAAACCTTTGAAACCTTAGAAACAACAGGAGGTGCTGATGCTGCTGTTGATGA[C/T]GCAGCTGATGATGCTGCTGACCTTACTCTCAGATCCGGATTCTGCACTGCCTCTGGCCGA
5948	CaTSNP8962	TCATGGTGTTAATTTGGCTATTGAGAGTGTGGTGGATGAGAGTGAAAGAAAATCTTCTGG[C/T]GGTCTTCATGGTGCTTCTTGTTCTGCTTGTGAGATGGCAGTTGTTTGGATGCAGAACCAG
5949	CaTSNP8963	GCTATTATTCTGCAGCAGACACACCATAGCAGAAACCAAATCACCACAAACCAAAAACAC[A/G]TACATAATTCACACGGACAAATCCACCATGCCACAAACTTTCACTGACCACCTTAACTGG
5950	CaTSNP8964	CACAGAAGAAGGATACAAGAACATGGAAAATCAGAACAACAACAACAATAACAAGTATTA[C/T]AGCTACAACAACAATGCTGCTGAAAAGGAAAAATACTACTTCAAAAGTAATGCTGTTAAT
5951	CaTSNP8965	CAAACTGAAGGGAATCGATTCTCCACCGTGCCACGTGGCAACAGTCCCTCTAATTTCAGA[G/C]TCAGACACACACCCTCAATCCCTCGCTGCTAGCTTCGATTTCTCCAAAACCCAAATCAAC
5952	CaTSNP8966	GGATCTGCTGCAGTCACACACCTTGGTGTGGTAGGAAGAGGAATTAAGCGAGCATCAAAT[G/A]CTAGTGTGGAAGAAGCAAGCATAGCAAAGAAACCGGCATTAGAAACAACTGAAGGCAAAG
5953	CaTSNP8967	CACGAGTGTTCTATTTGTGGTATGAGATTCTCTTTGGGTCAAGCATTAGGAGGACACATG[A/C]GAAAACATAGAGCTGCAACAAATGAAGGCTTATCTTCTATGAAACAAATGATTGCGAAAA
5954	CaTSNP8968	CCTTTGTGTAGCTCTTCCCTCTTCAGGTGTTAAGGTGAATGGATTTGCCTGCAAAGCTGA[G/A]ACAAATGTAACAGAAGCTGATTTCTTTTTTGGTGGTTTATCCAAACCTGGAATTATCAAC
5955	CaTSNP8969	TGATGCTATTGGCGTTTCATTTCCCATAAAAAATGGGGTACCTTGTTTGGTTCCAAGAGA[A/C]GGAAAGATACTTGACGAGGAAGATGCATCAAAACCAGATAATGATACCAATTCATATACA
5956	CaTSNP8970	TTCCTTTCCTTTTTGGAGAGCTGTCGTTCTGCCTCCTTTGGTGCCACAGGAACTTCAGCA[T/G]GCATCTTAACTTCAGGTTCGGGTTCAACAGAGTCCTCTGGTTCTGGTTCATGGTCTTGTT
5957	CaTSNP8971	ACAACTTATGAATGCTCAAAAGGGTGGTGGTGGAAATGGTGGAGCTAATGGTAAGAAAGG[C/T]GGTGCTGTCGGTGGTGGTGGTGGAGCTGTTCCTGTTCAAGTTCATGGCTTGGGTGGTGGA
5958	CaTSNP8972	AACTCCTTCTTCGGTAGTTTTCCTCTGCTTCGGGAGCAGGGGTTCTTTCGATGAGGATCA[G/A]GTTATGGAAATCGCGCATGCTATTGAGAATAGTGGCGCCCACTTTTTGTGGTCTTTGCGT
5959	CaTSNP8973	AAGTAGCCAGATTTCAGCCTCCCTCACAGCAAATGGCAAATATATTGTTGCAGCAAGTGA[G/A]GATTCCCATGTATACATCTGGAAAAATGAAGCCGATTGCAGGCCTAACAGGAGTAAAGGT
5960	CaTSNP8974	TATCATGGGTGAGGTGGCAGTGTTGCACTCTGAGCCTCTGCACTTTGAATGCAATGACAT[C/G]AAACATGTGACTGAAGAAGATGTTTATCCACCAAAATCAAATCCAGAATGCTTGCCTGGA
5961	CaTSNP8975	TGCTGACAATTGCAAAGTTCTTGTCAATATTGAACAGCAGAGTCCTGATATTGCTCAGGG[T/A]GTACATGGCCATCTCACCAAAAGACCTGAGGAAATTGGTGCTGGTGACCAGGGTCACATG
5962	CaTSNP8976	GCCGTCAATTCTCTTGATCACAATTTTAGTAATCCCTGGAAGAATCTCGATTGCGTGAGC[G/A]CGCACACCACCGCCGTTGACGTTCGTCTTATAGCAAGACGCGACAAATCGAAAAAACTGT
5963	CaTSNP8978	TCTATTGTTTCCTAACCAAGTACCTTGCGAAGCTCATTGGTCAACTTATTGCGACTTGAT[C/G]CTTCCAACTTGCTTAAAAGGGCGGCAAGACCTTGAGGTAGAGCTTGTAAATCTAGATCTG
5964	CaTSNP8979	CATCCCGGCGTGAACCATCACCGCTTCCTTATTCCGACCCGACGAAAACAACGGCACACC[A/G]CCTATGCTTTCCAACGCCACCATCTTACTCCAACTTGGATCCGACCCGCCGGCCATTTGA
5965	CaTSNP8980	TTGGTTACGAGATAGATCCAGATCTGCGAGACGGTAAATACGAGAAATTGAATCTGGAAT[A/G]GGCCCGCTTATTTTGTTACCGCTTAATAAGGCCCGGCTCAGCATTGGAAGCCTGCCAAAG
5966	CaTSNP8981	TCCCATTGATTTAGGAGGAAGATTTGACTTAAATTTTGCACGAACAATACCACTGTTACC[G/A]TGAGGCCTTGTAACCTTGCCCCATAGGCAGCGGTAATGGGTTCCATTCTTCTTCACTTTG

5967	CaTSNP8982	TTCAACTACGAATAAAGTCAAGCTCCGTTTGGAGCAAAGTGAAATATCTAGCTTCGTACC[T/C]ATATTTATTAAATAAAAGTCAGGATGCATCATCTTTGAGCTACAATATTTGTCATCGAGA
5968	CaTSNP8983	ATTTGCATGCTTCAATCAACTTTACAAAATGGATCCTGAAATATTTAATACATGGTGTAA[C/T]ATACTTAAACGTGTACCCAACAGCGCACTTTGGCTCCTGAAATTTCCTGCAGCAGGCGAA
5969	CaTSNP8984	AGCAAAAGCTTGTACAGGGCGGGTTTCACCAATCTTGCAGACATATATCATATTGTCTGT[T/C]GAACTTGTGGCAAAAGACGTATTGCTGCGCCAGTCAACATCAAGTGTTGGACCTGAATGA
5970	CaTSNP8985	TACAACCTTCAATCCTTTTTGGCAGAAATGCTTGCACAAGCAAATGGAACCTGTTTCACT[T/C]ATCCCTCCATGGCAATTAATTCCAGCTGAAGGAAAAGTTGAGAAATGTTCAATAGAGGAT
5971	CaTSNP8986	GCTCAAAGCTCTCTTCAAGCTTTTCGTTTATTGGCACAGTGGAAGATGATTCTCCAACTG[T/C]AGAATCAATTCGAGCAGCAGAACCGTTTCTCTGTACTTGCGCCGCAACTTGGAGTGATTC
5972	CaTSNP8987	AACTCCGCGAGGTTCTAATTTCAACCACGCTGATTTCTACTCCATGATGGGTTACGCGCC[G/T]AGACATTCCAATTTCGGCGCGGCAGATTTGTATTCCATTCAGTCCAATTCACGTGGACCG
5973	CaTSNP8988	GGAAAAACTACTTAGGTTCGAGGTGTTTAAGGATAATCTAAAGCACATTGATGAGAGAAA[C/T]AAGATTGTTAGCAACTATTGGCTTGGTTTGAATGAGTTTGCTGATTTGAGTCACCAAGAG
5974	CaTSNP8989	TCTCCCATCATTATCAACAGCTAATGTTCGCAGTGGATGAGGAGCAGGGAGAACATCGCC[G/A]CCACCCCTTCTTTCCATCAAATACGATGCTCTTGGATACGGTTGATTGCTGCTAGAACCC
5975	CaTSNP8990	AACTGATTCTCAGCTACAAACAAATCTTGACTATGTGTGTAGTAGAGAAGGCATAGATTG[T/C]AAAGAAATTCAACAAGGAGGTGCTTGTTTTGAACCTAATACAATAGCATCTCATGCTGCC
5976	CaTSNP8991	ATTGATATTCACCAGCTGGTGAAGAACAAGTTTTCTCCCATTTAGATTTGCAAATTGAAG[C/A]ATCGTAACCTAAAGATGAAAGACCTTCAGCAACGATCTTTATCAAATCGTCTTTTCTTTT
5977	CaTSNP8992	GTAATTGAATGCACCATAGAAACCAGACTCTTGAAACTTGTCTGCAAAGACCATGAGTTC[T/C]TCCTCAGTTATCCATGATGGCAAAACAGAAGGAGTTGGCAAGAAATCAACAATCTCCATG
5978	CaTSNP8993	TCCTACTACATCACAACCTGCCGCTTCTGATATAAATGAACCTATATTGTCAAGTCATAA[T/C]GATTCTACTACACCACAATCTACCCCTGCAGATACAAGGCCAGAAATAAAGAATGTATTA
5979	CaTSNP8994	AACAGCCGTTGGATCAGGAACTTCATGACTACCCTTTTCATATTCAGCCGTCCATTTCAC[A/C]TCACACCCTTCTCCAAATGGTGTTACAGAGATATGTCCCTTGAATACTTTGTAGTACTCA
5980	CaTSNP8995	AGGATACGCCCCATGTTGCGGCGGAGGATATGCCGGAGTACCGTATGGCACCGAACTAGA[A/G]CCCGCCGCCGCTCCTGGAGGAGGATAAGCCATGACTGGTTCTTGTTTCGCCGCCGTTTTT
5981	CaTSNP8996	AATCCTCGCCATCGACAATGCCGCCATGTCTTCTCTCCTCGACAAACACCTCTCCGTTTC[C/A]ACTCTCAAAAACGTTCTTTCCCTCCACGTCTTAGTTGACTACTTTGGCGCCAAAAAACTC
5982	CaTSNP8997	ACCCGCCTCAACTGGGTACCAACTATCACCCAATCGATAAATGCCTTGCCCTTCCAATAG[C/T]AGTAAACCGTGCTGGTTATAATGGACCTCCTTAACATTAAGAAATTCTCCAGGCTGAAAA
5983	CaTSNP8998	TAGTAGGGAGGAGATGCACACAGTTGTTGTTGATATTCTGAAGGAAGTAGATTTCAATAC[C/T]GCAACTTTGTCCGATATTCTCCGACAACTTGGTACCCATTTTGGCCTTGATTTAATGCAT
5984	CaTSNP8999	CAACATGACTGGGCCTTACCAAATTTTGAGCAACGGGCTGAAGATGTGTTGAAGAAAGTC[A/G]TCAGCTGAACAATGTTATGCGGAATAAAAAGTTTAAATGCTTGCCTTAAGCCAAAACACT
5985	CaTSNP9000	TTGAAGAACGGACAGAGTCACATGAATGATCTCTTTGGGGGTAATTTTCTGTTTATGCAG[C/G]CAAATCCTATTCCTTTTGGATGCAATCACAAACCTCATTGATTATGGCATTCACATATTA
5986	CaTSNP9001	TGCTGATTCCGTATTGGATACGGATCGTGCCCTTTTAGACTCTATGCGTGCTAAAATTGA[C/T]GATGAACTTAAGAAGCTTGACGAAAAGATTGCTGATGCTGAGGAAAACTTAGGTGAGAGT
5987	CaTSNP9002	GAACTTCGAGGGTCGAGTTCATCCATTAACAAGGGCTAATTATCTAGCTTCTCCTCCTCT[C/T]GTTGTTGCCTATGCCCTTGCTGGCACAGTTGACATTGACTTTGAGACTGAGCCCATTGGG
5988	CaTSNP9003	GTAAAGCCGAATAAAACCAGCAGCATCAGCTTGATCATATATATCACTATCCTCAAATGA[C/T]GAAATATCTTGCCTATACAGGCTAAAGGGACTCTTCCTGCCTGTTACAGTAACAGAACCT
5989	CaTSNP9004	GGCTCAACAGTGAAAATTGGGCTGCAGTGGTGCCATTTGAGATGTAGACTGTTGCCAATA[A/G]AGTGACATTTGTGTTTATGCCATGCTTCTTCAGTCAAATGTTGGTTTGTTTGTTTTCTAG
5990	CaTSNP9005	GAGAGAATCTGATTTTAGTGGTTCTACTTTCAATGGTGCATACCTTGAGAAAGCTGTTGC[G/A]TACAAGGCAAATTTTACAGGTGCTGATTTGAGTGATACATTGATGGATCGCATGGTTCTT
5991	CaTSNP9006	CTACATTGATCTAAAGGAGTTCAGCGAATTACACAAAGACGGCGGCGACGTCACGGAGCT[C/T]CGAGAGGCGTTCGAGATGTACGATCTGGACAAGAACGGGTTGATTTCGGCGAAGGAGCTG
5992	CaTSNP9007	CCACCTATCTCAGATAGCAGATGCGGTGATTGTAGCTAGAAGTCTTGGGGCAACACTTGT[G/C]ATTCCTGACATCCGAGGAAGCCAACCTGGTGACAAGAGGAACTTTGAGGACATTTATGAT
5993	CaTSNP9008	TTGCTTGGATTTTCCAGTTTTCGGGGTTTCTTGCTTAGGCTTCTGCTTTGGTGTGTCAAC[G/A]TCACCGTCTTTTGATTTCCTTATCTTGGAAGCATCTGTCTTCTTTGAATTGTTTTTGTGT
5994	CaTSNP9009	TGACAACTCAGATAAAAGATTCAAATTATCCCCAGTCACAGGGATATAGCCCCCAGCACC[T/G]CTCTGTTCGCTAGAAGTAGACCCGGTATAGAGTGACATAATATCAGGTGCATTTGAAACA
5995	CaTSNP9010	ATTGTAAGGGGCATTTGTGATTATGCTAATGGTATACATATACAAGCAAAACTAAATTCC[A/C]TTGCCATCGAGTGGAGGGTAGGGCTCATTCACCTAAACAAGGGCAATATTCCCCACTGGT
5996	CaTSNP9011	TCCTCTGTACAACATCAAAGCATACCTTCCTGTCGTCGAGTCCTTTGGATTCTCTAGCCA[G/A]TTAAGGGCTGCAACATCTGGGCAAGCTTTTCCTCAGTGTGTGTTTGATCACTGGGATATG

5997	CaTSNP9012	TCACCAGCCAATCAAGGAAGCTGCTCTTGGAAATATTCCTACAATTGCTTTCTGTGACAC[C/T]GATTCTCCTATGAATTATGTTGATATTGGAATTCCTGCTAACAACAAGGGTAAGCATAGC
5998	CaTSNP9013	CTACCGGAAGGCCTTATGGTGGAAACTTTATTCAATCACAGCCACGCATGTCACCAACAC[C/G]TGTCACATACATTCCTGACGATGATGATGATGATGATTATAATTACTACACGATGGATAA
5999	CaTSNP9014	TACCACCACCAGCGCTGCTCAAGGTGGCGCTATTGGTGCTATCATGGGTACTCTCACCGC[C/T]GACGCACCCTCCGCTTTTCCAATACCTCCACCTAACGCTGCTCTTAACCCTCAAGCCATG
6000	CaTSNP9015	TACGGAAAGATCTATGAGGATGATCCACCACATGCAATAACCGTGAACTTGACTTTTTAA[C/G]AAGAGCAGCATCAGTCTCAAATGCAGCAATATCAAACACGATTACCCTTGCTCTAGTTTC
6001	CaTSNP9016	ACGTTCCAAAATCTTGCGTTGAAACTCTAAAGAAGATTCATCAAAATCACCTTTGAGTCT[G/A]GAATGTTCAACGAATTTGGTGAATTTAACACTGAGATTAGAAGGGGGTTTGTAACAAGAG
6002	CaTSNP9017	ATCCATAAGCTTGTAAATCTTATCCACCCACTGGTTATCGCCACGTTTGAGTGCAGGGTT[A/C]GCCATTAAAGCTTCCAATGCCAATAGGGCCGAACCGGAAACAATCGGAATGTCATCACCG
6003	CaTSNP9018	CCATGCCCGCTTCGGTAATAAATGGGCCACAATAGCCCGATTACTTTCCGGTCGAACTGA[T/C]AACGCAATTAAAAACCACTGGAACTCGACGCTGAAGCGGAAGTGTTCATCCATCTTCTCA
6004	CaTSNP9019	ATTCTTGATAACAGCATGGAACTGACCGTGTCTAGATACCCATGAATTAACATTTGCAGG[G/A]TCGCTCTCTAAAATGTTGCTGCAGATTGTATCTCTGTCAACATTCAATATCTCAATCTTT
6005	CaTSNP9020	ACCAACTGCTTTCAAGCTTTTTACAGGTTCGGCGTGGATGGCACTCTCAAGATCTTTCAT[C/T]GACTACTGCATATGGGGATGGGACAACTTACCACGAACCGTTCTCATGTACTATTCAAAT
6006	CaTSNP9021	GGGTAAGTACATGAAGAAAGCCAAACCAAAAGGTGAATTAACTTTGGTTGATTCCACCAC[T/C]ACTATCACTACCACCGCAACAACCACCACTTATTGTGGTGTTCGAACTCGTGCCAAAACC
6007	CaTSNP9022	TGGTCACCAGTCTTATCCTCATAAAATTCTCACTGGTAGAAGGAAAGAGATGCATACACT[A/G]AGGCAGACGGATGGATTATGTGGGTTTACAAAACGGTCCGAGAGTGAATTTGATTGTTTT
6008	CaTSNP9023	GACAGTTGAAGGAACTTCTGAACTACAAATCCCTTCCGGCACCCAACCTGGAGATATCCT[C/T]GTCCTTGCAAGGAAAGGTGTACCAAAATTAAACAAGCCATCAATACGTGGTGACCACCTA
6009	CaTSNP9024	GCTGTTGTTACGGTGAACAACTTTCAACAATATCGTCACATCGCCGCACCTGGCTGGTCA[T/C]TAGGATGGACATGGGCGAAAAAGGAGGTAATCTGGGGCATGGTGGGATCGCAGACCACTG
6010	CaTSNP9025	TTGAAAGGATCCTTGTCTGCGACAGGTACGGTTGGTGCACACCCTTATTTCTTGGAGTTG[A/C]TGTGCTTGTGCTTTGGCGCGTGCAGGAAGCCGATTTCGTTTGGTTGATGGGGCCGTCGCC
6011	CaTSNP9026	TTTCATTGAAGGAAGAGGAGAAGCTGCAAAGTCTGATAAAAATGTCGATGGTATTGACAC[G/A]AGATGCAAGATTGTCGATTTCGGAAATGCGTGTTGGGCTGATAAGAAGTTTGCAGAAGAA
6012	CaTSNP9027	GTTGCAGAGTTTGCCACCGGAGAAAATGGAACAGGTTGTGCAGATTATAAGGAAGAGGAA[T/C]GGGCATTTGGAACAGGATGGAGATGAGATAGAGCTAGATATTGAAGCTGTTGATACCGAG
6013	CaTSNP9028	CCTAAGACTCGGTGATGATCAAGGGAGTAGTCTGTTTACAAACTCCAACTGGTTTGCATT[T/C]CAAGAAGACAGAATTGGTGATGCAAATGGTGGAACAACATCATCTGACATGATGGATGAG
6014	CaTSNP9029	ATTTTGCCAATTTTGTGAAGCTTCATAACGTGTTCGAAAATTCCAGATGTGTGACTTTGA[C/T]TTCTCACGAGCTTCAAGGATTTCCTCGACTTGAGAAGTGGGTTTGGTCTTGAGCATTAAG
6015	CaTSNP9030	TACCACCCTTTCGGTTGCAGCTGAACCACCACTCCGACATAACTCGAGCAATGCTGCAAC[C/T]GCGTTCTCTTTCCCCCTCGGTGTTCCGCAGCGCATCATTCCTATCAATCCTGCTACTGCT
6016	CaTSNP9031	ATCTCACTAGAGCTGATAATTGAGCTGCCATTGCTTCTCTCTACACCTTCAGATTCCGCC[G/A]GTTTTGGGATTCCATTCACCTCACCAGAGATGTCAGAAGATTGAGCTACTGACTGATTCT
6017	CaTSNP9032	AATTCTGAAGTGACCATGTTTATGTTTGTGTTTGGAATCAGAATATGGATGGCAGAATGA[G/A]TAGAGTTTCCATCCGATTTTGGACCGTGTCTTGGAGTTAGTGTTAGTATATATAATATTT
6018	CaTSNP9033	TGTAATGAAGAGTGGGAAGTATACTTTGGGTTATAAAACTGTTCTCAAATCTCTTAGAAG[C/T]TCCAAAGGAAAATTGATCATTATTGCCAACAACTGTCCACCATTGAGGAAATCTGAAATT
6019	CaTSNP9034	TTTTGGTACCCGCTTGATAATTTTCTTCTTTTTTATTACTTTCACGGTAGCATCGTTGGT[T/A]GTTGTATTAACTGCTGGATCTGGCTTGTCCAGAGGTTTATCTTCAAAACTGTTAATTTCC
6020	CaTSNP9035	TTCATAATCACAACCATACTTTTTTCCAAAAGCTATTCTACAAATAAGCCTATTTGTGAA[G/A]GACATCAATATTTCACTCAAGTTCACACCTTTTTCATCATCACAATATTGTGACAACTTT
6021	CaTSNP9036	GGATATCCTTTCCAACGGTGGAGTTGTCCAAGAGATGGCACATATTGCTAATGGACGAGA[C/T]ACTGGAAATTGTGTCTCCCTACTTCGAGTGAATAGTGCAAATTCAAGCCAAAGCAACATG
6022	CaTSNP9037	GATTCCTGCTCCAGCCTGTAGATGGGCAATCCATTCTCTGCGTTTGTTCACATCCTTGTA[C/T]GAGTACATTGTGTCAAAGCGAGTATTAGTGGGGAAAACGATGGTCCTCAGAGCAAGGGCA
6023	CaTSNP9038	GATTGGAAAGCAGGGGGGCACTATAAAAACTTTTCAAGATGCCACTGGTTGCAATATACG[C/G]ATCCTTGGTTCAGAACACCTGCCAATTTTTGCTCTACGGGATGATAGTATTGTTGAGATA
6024	CaTSNP9039	AAGCATTGTATATGAAGATGAAAAGGTCCTAGCCTTTCGGGACATCAGTCCACAGGCTCC[G/A]GTGCATGTTCTGGTCATTCCAAAATTCAGAGATGGATTAACAGAACTTGGGAAGGCTGAT
6025	CaTSNP9040	GCCACAAGAAGAACTAGCTCAAATTTCCGTCACATCGAGTTATTCGACCTAATTAACAAC[G/A]AGTTCAAGGAGTTAACTCGTTTCATCTCACCTCAATCTCACCACCTCAACCCGTTTATCT
6026	CaTSNP9041	AAAAGAAACGGGCTTTGAGGTGTAAAGAGAAGCGACGGTGAAAACGACGGCAGCGGTGGA[G/A]AGGAGAGAGAAAGCGAAGAGGAAAATAAGGTAGAAGAATTGAATGATTAAAAGAAGAGTC

6027	CaTSNP9042	AGGTCAGATGCATTCTAATGGGGTTCAGAACCAGCACTTGGTTGTGAACAATGATGGGTA[C/T]GTGATGAATGGTGTTATAAATGGAGAGAACGGGGGCGAAAGTTTCAAGCGTGAAATGAGG
6028	CaTSNP9043	GCAAAGCAGCCTCCATGGACAAGCCACCTGAATTTCTACCTTGATCATTTGCTGAATCCG[T/C]TGAGTTAAAAGGGCTGGGAGAAGCGGAAGATGTACCAGACACTTGAGAAACTTTACCTTT
6029	CaTSNP9044	ATCGGTTTCAAGGATACCAATGATGTCGTCATCCTTCAATATAAGATGTTTTGTACCATT[G/A]AACTCCACCTCAGTTCCTGCATACTTTGAATACACAACTTGTGCACCTGCCTTCACACTA
6030	CaTSNP9045	ATATGGTTTCTAATCCATGTGTTATTGGTGGTGAGTCGAAACTGGGAATTGATGGTGATG[C/T]AGTTGTCTTGAACAGTTCTGATAAGTCTGGTTCTGTCCGGGACCAGAGAGCCATAGTTCT
6031	CaTSNP9046	GGGCAAGGATTTACATAATATGGAGATGCACTGTTTTACTATTGTGTTGGAACGATTAAG[C/T]GTGTGTTTGGTTATGCGTTAGAGTTATGGTTCTGCGCGATTTCAAAGAAGCTACAAAACT
6032	CaTSNP9047	CAACAGAAACTTCCAAGGTTTCAATTGGTGACAATGATTCCAAGGAAGACATGGATGTGG[C/A]AGAGCCTGAAGTTAACGAAGACAAAACTTCTATGGATGATTCTGAACTAGCTGAGAGTGG
6033	CaTSNP9048	GTCCCCTGAATCGAGATTGGCGGTATACAAAGTGTGCTTAAAAGACATTGGGTGTTCTGG[T/G]TCGTCCATCCTTGCGGGGTTCGACGATGCGATTGCCGATGGAGTTAATGTGCTTTCGCTG
6034	CaTSNP9049	GGACCAGAGCCATATTGAATCGGGTAGTTTGAGCTCCAATGATTCTGGACCGTATGGTAC[A/G]TTTGTTGGTGTCTATGATGGACATGGAGGGCCTGAGACATCAAGGTTTATCAATGATCAC
6035	CaTSNP9050	TACCCCACACATCATTCACAGAGACATGAAATCAAGCAATGTATTACTTGATGAAAATTT[G/A]GAAGCTAGGGTCTCTGATTTTGGAATGGCTAGGCTTATGAGTGTTATGGATACACATTTG
6036	CaTSNP9051	AGTGATGACAGATGGGAAGAGCAAAAATTTAGTGGCATATCATGAAGTTGGGCATGCCAT[A/T]TGTGGAACTTTGACTCCTGGCCATGATCCTGTACAAAAGGTAACCCTTGTTCCACGTGGG
6037	CaTSNP9052	GATCTTGGATCCATCAACACGATACCTCACACGCTTTCCAACAATCTCAGCAGGCAATAC[G/A]ACATCCTCAAGCATTGCTTCATGAACAGCAGTGAGAGTACGGCTTCGGGGACGCTGAGCA
6038	CaTSNP9053	ATTTGCTTGGGCATCGTTTCCATTACCATTCGTCTGGGCACCATCTTCCTGTTTCAAATT[T/A]CCTTTGTGAAGACCACACTCCTTTGCTTTGGCATCCTCCCACCACCATCTTCCCTCTCTT
6039	CaTSNP9054	CACAAAATACCCTCTGTATTTTGTGTGATATGCATTCCTCGCAGACCCTGCTAACAAGCA[T/C]GCCTCAGCTCCCAAAAAACTAATCCATGAGAGAACGAAGAAGAGGACGGAGAAAGTGGTG
6040	CaTSNP9055	TCTCAGCCTCTTTCATCACTTCTGACTCACAAGTAATTTGCACTAACAAGTACCATTAAA[T/C]TGTCTTCACAAGCTTTCTAGTCGGAGTTGGAGTCATAGAAGTCACTTGGGCATGGAGTTA
6041	CaTSNP9056	GGCCACGAACATGTGACACGTGCCGCTCGGCCGCATGCGCGGTCTTTTGCCGCGCAGACT[C/T]GGCTTACTTATGTGCCGCGTGTGACGCGCGCGTCCACGCGGCCAATCGTGTAGCCTCGAG
6042	CaTSNP9057	CAAAAGTCCGGCTGCATACAACGGGAAAGTCACATAGGTAGCCTTTCTCTCAGCAATCCA[A/G]AGTCTCTTCTCGCTTTCGTTAGTCTTTCTTGATGACCAAGTTAGTTGAAGGAGACGGAAT
6043	CaTSNP9058	GGATGCTTTTTTTAATTTTCTACTAGCATCTTTCTGTCGCTTTGCTGGAGACTGTTCATC[G/A]GCTATATCTGACCCTAACTTTCTTTTGTCACGTACATTTCTCTTTAGATTATTTTCACTA
6044	CaTSNP9059	TCCGAAAACCTACTCAGAGGTATATTGAAGAATCTTCAAATGCGAGATCAAAAGAAAAAG[T/C]ACCAACTACTGGTGCAAAATGTAAACGACGTAGTTTGTCATCATGTAATGAACTTCATGT
6045	CaTSNP9060	TATCACCTTTGTAAACAATGCCAGCGCCACCTTTTCCTATAATGTTATCCTCTTTCAAAC[A/T]ATCAAGAACATCATCAACGGTGAAATCCAACCGTTGAAACGCAGTTAATTTCCATGCACG
6046	CaTSNP9061	GCGGGTCCCTGATTCCAATTGGACTGTGGCACTGGTCTGCTTTGTTTTCTCCTGGTTCAC[G/A]TACGTATTCGCGTTTCTCCTGTTGCTAACCGGTGCTGCACTGAACGATCAGCGCGGACAA
6047	CaTSNP9062	TGGTGTTGTTTACAAAGCTAAGGATAAGAAGACTGGAGAGATTGTAGCACTGAAGAAGGT[T/G]AAGATGAACATAGAGAGGGAGGGTTTTCCAATGTCAGCATTGAGAGAAATGAATATTCTC
6048	CaTSNP9063	CCAATCAACTACTGGTCTCTGTTGAGATGGTATAGAAGATGCCACTCCATCAGAGCCACT[G/A]TTATCACTTCTTCCAGATGTACTCAATTGACTTTCCTTACAAGCTATATAAGCAATTGCA
6049	CaTSNP9064	AATCTATCACATCTAGTTATGTTCTCAAGAGGATATATTCTCAATGTTCGACTGGTATTT[A/G]GATCTAGCATGCGAATTCCATCCAAACCAACCTGGGAAAGAACATCCATTGTGCTCTGAC
6050	CaTSNP9065	TATCTTCATCTACTATTATTACCTTGACTGGTAAAATGGAACCGAGCAAGTCTCTTGCAA[T/G]TTCTTGGATAGTTTTCTCCGGTGCTTTACAAGCATGCAATGGGCTCAACTGTGGGTATGG
6051	CaTSNP9066	AGCGAGTGAAGTATCTGTAGGATTAGCAAGGAAGTTGTGAAGGATGTGAATAGCTTCTTT[A/G]GTAGCGCTTTTCTCCCGCTTGATGGTACAAGTGATGAGAAAGGCAGAATGAGAGTGAGTG
6052	CaTSNP9067	AGTTCATCTCTCAGTAAAGGATGTTAATGATTGGAATAGTGATTTGCTAAAGGAAAACTT[A/G]GTAATAGACCATCAAAGAGGAAATATTGAGAAGATTTTCATAAACAATTATGTCAGGACA
6053	CaTSNP9068	TCTCTTGCGTGGTTCAACGAAACCTGTCGCAAAGGAGCTGCTGAATCGTGTTGAATTGGC[C/A]ATCACAAAGCTGCAATGTGTAACCAATGGTGAAGATGTTAAGGAGGGTGACAATCTTATG
6054	CaTSNP9069	TGACATTAGGCCTTATATCAAAGTTCCTAATAAATTTATCATTTCCTGCATCTGTTACCT[G/T]TGGATTTTGAACAAGTTCTTTGATTGTTTCAGGCATAGGTTGGTCATTCATCATATTCTT
6055	CaTSNP9070	CGTCCATGTCCAATGGCAGTAGCACAGCGTTGATGGTAGTGACACGTTACAGTAACAATA[T/C]TGTATTGCTTCTTCTTCGTGTATGTCATGTTCAAACTTGTGTGTAATGTAGCATTTCTTT
6056	CaTSNP9071	TTTCAGGAGCTCAGCGTGTTCACGTGCCAGAATTTTTGGAACAGCGTGCTGAGGCTTGCG[A/G]CATTGAGGTCAAGACAATATCTGCTTACATTGATTCTTTCAGATATGGTGCACCACCACA

6057	CaTSNP9072	AGTTTTTACGGTATTTGCAGAATCGTATTTTTCTTTTCAAATCAACTTCTGCAAAATCTT[G/C]AGTAGTGGTTGCAGAATTTTGCAATATTGTTATGTGTTCTGATTCCTAATTCAAAATCAA
6058	CaTSNP9073	TTATGGATATGAGGTACTGATTGAAAACCTCATGGACCCTGCACATGTTCCATATGCACA[C/T]TATGGAATAATGAACACTCCAAAACCAAAAGAAAAAGCTGATAGAGAAGGGGGAAGACCA
6059	CaTSNP9074	CAAGGGTGATGTTGTCATATTGCCTGCTTTTGGAGCTGCAGTGGACGAGATGTTGACTTT[G/A]AGTGAGAAAAGTGTTCAAATCGTTGATACTACTTGTCCTTGGGTTTCAAAGGTATGGAAT
6060	CaTSNP9075	TCTTTTCTGCATTCTCATGAGTAACGGGCCAATTCGTTGGCGAATTGTGTACATTGTCCT[C/T]CCGTATTCCGCAGCTGGCCAAATTTCGGTCCCAAAAACAGCACTAGTGGCAAGAACTTTA
6061	CaTSNP9076	TGAAGAAGTTGGTTGTCAAATTTCACAAGACTTGTCACCATGTGGTCTCATCCTTGGTAT[C/T]AAACAACCAAAGTTAGAGATGATATTACCGGATAGAGCTTATGCCTTCTTTTCACATACT
6062	CaTSNP9077	TCCACTTCCTCTCACTTCCATTTCCACTCTTGGAGCAATTAATCCAAACTACAGCTATAT[C/T]GAATCCTCCACTCCCGCCCTCGAAGCAACTCAAAAGGTTCCTCAAGATCCTAGTGCAGTT
6063	CaTSNP9078	ACGCGCTCAAGGTGCGTCTGCGGGTGTGGTGGTGAATAGGGTGACGAGTGGAATAATTTC[T/A]ATGACGTTTTTGTCGTTATCTGATAAGATCAGTATTGGAGGGGCTTTCTTCCTCTTTGGT
6064	CaTSNP9079	ATCCCCGAGGCGAAGGATGCTTGTGGCTTTTACTTGTACCATCTGTGGCCAGCGAACTAC[A/G]CGCGCTATTAACCCTCATGCTTACACTGATGGCACTGTTTTCGTTCAGTGCTGTGGGTGC
6065	CaTSNP9080	TATCAAACTTGTGTAATATATGTAATTGAAATAACATAAGACTGCCATGATCGTATACAG[T/C]CCGATACGTCTAAACTCATTAGCTTCTTCACAGGTCCTCTGGGCCATTAATTTCCTTGTT
6066	CaTSNP9081	GTCTTGTAGGTAATCGAAAATTCCAGAAATCACATTGTGACCTCCAGGAGCTTGACCACC[A/G]GACAAAACTACACCAATCTTCAGCTTTTGATTAGGTTGCACCGTGTCCGAACCACTCGGA
6067	CaTSNP9082	TTCAATACGCTCCTGGATCACAGGTTGCACCAGCTTAAGCCATTCCATATCACCTTGTCC[G/A]CCAGCACATTGGCCAAGGCTGATAGGACCATCCTTCAATCCATCAAGCTCATAGAGTACT
6068	CaTSNP9083	TTGTATTGTTCAAAGAAACAATATTTGAAAATAATCTACTCTTGCTATTAGAAGCAAGTT[G/A]AAGTTCACCCATACTCAACCTGAAGCTAACATCTTCAGAGCTTAACCTCGACATCAACAC
6069	CaTSNP9084	TATCCCAATTATTGCACCCAAGTCCAATGTTAATTCTTATTTCAATAAGAGACACACTCA[G/A]AGGAATCAGAAAACTTCTTACTCTGTTACTGTTCAAGGTGTGGTTGATTCCAAAGGAATT
6070	CaTSNP9085	TTCGGTTTTTGGAAATATTCTTTCCTGCAAGATAGCAATGGATGCTTCTGGTCTGTCTAA[G/A]GGCTATGGTTTTGTGCAATTTGAGAATGAAGAATCTGCCGAAAGTGCAATTGACAAGTTA
6071	CaTSNP9086	AGAGCGAGCTTACTATTGCGTGACTAAGGGAGTACCTTGTTCCTCTATGCCAAGGCACAG[C/T]AAACCTAAAAGAGTAGATCTATATTATTCGAGCTATTGTGTTCGTGCAGTTAAATCACGA
6072	CaTSNP9087	ATGTTTTTGTGATCATCATGCCTAATTTCTTGTTCACTTTTCCCTTCATATGGCTCTTGC[C/T]ATCCATATTTTTTATTTGAAGGAAATGGAAAAGATTGATATGTTATGTGTGGAATTTGTT
6073	CaTSNP9088	GAGGTATTGCAGAACTTTTTTGATCTAGAGAAGTATCCTCTGCTTTCAGAACTCACTCAC[C/T]GTTTCCAGGGTTTCAGAGAAAGATTGTTGGCAGATCCCAAATTCTTGCATAGATTAGCCA
6074	CaTSNP9089	GTACACGCTAGCAACTCCACCACTAGCTCCATCACGAAATGTTGCATGATAAATTGCTCG[C/T]CGAGCCAGTTCAGAAGCTTCCTCAATTGACATGTCATATTTGTACCCGCTGTCCAAGACA
6075	CaTSNP9090	CATAATGTTGTCTGCATATGGTGATACGAAGCTGGTGATGAAGGGAATTTCCCATGGAGC[A/G]TGTGATTATTTACTGAAACCTGTGAGAATCGAGGAGCTAAAGAACATTTGGCAGCATGTA
6076	CaTSNP9091	CTCAATCGGACTATGACGGTGGAGGAAATAAAAGGAGGAATGGAGGTGATGATAGGGAGC[A/C]GTTTGTGATTGATAAGGATGATACTGTTTTTCGTTATTTGTGCCCCGGCCGAAAGATTGG
6077	CaTSNP9092	ATCGAATAAATGTGACTGCAAGTAGTTAGGTACATTGTGACCAGCATGGCCATCAAATAT[C/T]GCAAACAAGCCCAATTCGTTGTTATCAATTTGCTTAAATCGAGCAACGAGATAATCTTCC
6078	CaTSNP9093	CAGAGGTAGGCAATGCAAAAACTGATCAGCAGATGTGTACTCCCACGCTTCTGTCAAGGA[C/T]TGGACCTGAGAAATGATGCTTCTGTGTGTATGAACAACTCCCTTAGGTTTACCTGTTGTT
6079	CaTSNP9094	GGCAAGGTCAGCATCAACCTGGCTGAGATTAGTGAAAGGGGTTCTGGAAGGTTGGTGAGT[A/G]GTGGTTTCAGATCCAGTAGGTTGTTCGTGTTGTTGTTGTTCTTGTTCTTCTTTTTCTTCA
6080	CaTSNP9095	AGATGAGGACATGAGGAGAGCGATGAGCAAGTCATTTCTGGAGTCAAATGGAACAGTGCT[A/C]TCAACTGATTGGAAAGAAGTGGGATCAAAGAAGGTGGAAGGCAGTCCTCCAGAAGGCATG
6081	CaTSNP9096	AATTGGTGAAGTTGTTGGTGCACGTTTAATTTCCCATGCTGGTAGCCTCACAAATTTAGC[C/T]AAGTGCCCCTCTTCAACTCTTCAGATTCTTGGTGCAGAAAAGGCTTTGTTCAGGGCATTA
6082	CaTSNP9097	ACTCTTCTTTTCCTGGAAAGTTTTGCTGAAAATTTTAATCTTTGATGTACTCTCTCTTAC[A/G]GCATACTCTCCATCCGTAGACCAAGCAAATTCCAGTGCTGAACCAAATGACCTATTTCTC
6083	CaTSNP9098	TTACAATGAAACCGACGCCGATCAGTACAATCTCGTTTTCGCTAATTGTCACCCTCAACA[A/G]CTCAAAGTCACCATGGATGTTGAATCTGCTATGTATAATCTCGACGGAAAGAATAACGTT
6084	CaTSNP9099	CCTTTTAATTTGGCTCTTCATGAAGTTCCTCCACTTGTCGGTCACCTTACCGGCAAAAAT[A/G]TCATCATCCGAAAGCCCTGCCTGAGCCAACTCATCTTGTGGTAGATACACTCTTCCTCTC
6085	CaTSNP9100	AAGTTCTTGGCAAGAAGTCTGTTCGTCTTGACGAACATGGTTTGATACAAGGCGATGTTC[A/G]TGAAACACCTCAAGAGATTAACCTTCCTGACAACGTTGTGCTGGATAAAGTTCCAAAGGC
6086	CaTSNP9101	AGCAGAGCAGCAAGGTTCCGCAAATTGTTTTCTGCCACATGACAGTGTACATTCTGAGTT[G/T]GTAGTATTTTTCAAGCTTCATCGGTACAGGGGCCGGATATAAACGACATTCTCTCTTGGA

6087	CaTSNP9102	TTGTACAGCAACAATATTCTTCATTTCATTAGTGATCTCTTTGGTAGCCATGGTTTCGTC[A/G]CCACCATTTACAATGTGCTCTGACAATAGCTTCAATGCATCTCTCTGACGCATCAATTGT
6088	CaTSNP9103	AGCATTCCTGGACATGACAACACCCCAGCGACTGCCAGCAGTAGTCATGGAGGTAACATG[G/A]AATCCTTCTTTCCATTTCTTGTTTATCCATTTGAAAGGAAATGATTCGCTCACTTTGTAG
6089	CaTSNP9104	TTGAACGATCACATAACCTTAATTCTTTGAGAGTCCACAAAGATTGGAACTAGTTCCAGG[C/A]ACATGGATACTCTAACTACTTCTCCTTTGCTCACTTCTAAGTTCAAACCTCTGTTTTCTC
6090	CaTSNP9105	AAACCCTTTATTCATCTTCCGCATTCTCTCTTTCTTCTACACCTTCTAATCCTTCCCGTT[C/A]TCTTCTCACTTCCGCTTTCCCTTTCGCATCTTTCAAAACCTCACCTTTCTTCAAATGTCG
6091	CaTSNP9106	AGTCGAGGTTCGGACGAGACGAAACGATCATGTCATTTTTTGTGAAACACTCTTTGGCCA[G/T]TTCCCAATTGTTAAGTACTATAGCATGTTTGGAACCAAGCTTGATTGTGAATATTGGTCC
6092	CaTSNP9107	GTGGGTTGTTGAGGTAACAATATCACAGTAATCAAATGGACTAGCAACTTCCTTAGCCGC[G/C]ACAAGACCACTAATATGAGCCATGTCACACATCAAAACAGCTCCACATTTATCAGCAATC
6093	CaTSNP9108	GAGTAGGTCGTCGGTTACGGTTGCCGGAGTTGGTTCTCCGGCGAATAGGAGTGATGATAC[A/G]CTGCTGCAGCAGCATGATGGAATTCAAAGTGCCATTCTTCATTGCAAGAGATCGCTTAAC
6094	CaTSNP9109	CTGAAAAGCCAAAAACCACCAAAGGACACAATTTAAGAAGCAGCAGCAGCTAATGGCGGA[A/T]GAGGTGATGGAGCAACTCCATTGAAGAAACCAAAAGATTTTTCAGATGAATCTTCTTCAA
6095	CaTSNP9110	CGAGCAGTTTCCAAAACCGATTCCAAGAGTTGATTAACACCTAGAACATCAGGGACGTTA[A/G]TTGAAAGTGATGTTCTGCGATCTGACTGGCTTCCACTTGGAGTCTCTTCATCCATCATAT
6096	CaTSNP9111	AGGCACATTATTCTTACCTCTCAGCCGTGAAGATATATTATCAATGGCAGAATTTAGTTG[C/G]CCTATCTTTTCGTTCAAGGTCTGCCTGGTCCCCTCTAGCCCTTCGTCGTAATAGATTGGT
6097	CaTSNP9112	AACCTGTGATGAAGTGAATGAGAGGTTGCGTGCAATAATGGCTTTGGATGATGATGATGA[T/A]GAAGAAGAAGAAGAGGATGGTGGTACTTTGAGAACACCGATCGCAGCCATTGATGCCATG
6098	CaTSNP9113	ATTTCCTAATGCCCACACAGCCTGTTCACGGACATCATCGCTAGGAGAAGCAAGGAGCTG[G/T]ACAAAAATTGGGACAGCACCGTGATCAATTACTACCTTAGTGTTTTCAGATGTTCCAGAA
6099	CaTSNP9114	CAAGTGCACTGAACTGTCATGTCACTTATCCTTTTTAATATTCGGCTAAATCATAGAATC[G/A]AGGTATGAGTAATTCCTGCGCCGGCCATATTCAAGAAGGCTTCCATAAGTACGATCCCAA
6100	CaTSNP9115	GACACCACTGAATTGGGAAATGAGATCCGGGATTGCACTAGGAGCAGCCCGTGGCATTGA[C/G]TACCTTCATTCACAAGGGCCTAATGTCTCCCATGGAAATATAAAGTCTTCTAATATTCTC
6101	CaTSNP9116	ATTCATAGATTTCAGCTGCTGCTCCAACTTCTCTTTCTCTGCCTTGAGCCTCTGTTTCTC[G/A]TCACGAAGTTCATTCTTCTCAGCCTTTAACTCTTTAATCTTTTCTTGAAGACCCGTATTA
6102	CaTSNP9118	TGCTAATTTGAGTGAAAATCTGCGCGGTGTTAAGGAAAAAAACGATGGATCTTCGAATGC[C/G]TTTGGGAATGTAGTGTTTTCTGATGGATATGCTATCCGTGGTTCTAGTTCAGATTCTAGA
6103	CaTSNP9119	GTCAGAAAAAACTACCTCATTGTCCGGCTTTGTATCAGATATGACCACCTCACTGCTCTG[C/G]TGTGAGTTAGAATTGACTACCTCACTGCTGGGCTGTGCTTCAGACATGACCACCTCATCA
6104	CaTSNP9120	GACAATACAGGCTTCAGATTTTCATGACAATGCAAATATTACAGATTTGTCCTTTAGGTC[G/A]GTAGACAAAGAACAAAGGCATAGTAATGTTGATGCAGAAAGCCTTGAGTATATTTGCTCC
6105	CaTSNP9121	AACAGAAGTGCCAGTTGAAGTTGGGACTACACAAGAAACAGAGGAAGACAAACCAAAGGT[A/C]GAGAATCCAGCACCCGAAAAAGTTGAGGAAGTGAAAGAGGAAGCAACAACTGAGGAAGCC
6106	CaTSNP9122	AACGTTCATGGAAGCAGCACACTTATCGGTATCGGGATCGCTGATGAGGAGAACTTGGAG[A/G]GAGTTGCGGAGAAGGATTCTTTTGTAATTCCTCTTGTCGGTGCGTGCCTTCACAATCTCT
6107	CaTSNP9123	ACCATCAAGTGGAGGTTTAGAAGGATGTTTACTATAATCAAAAACATAAACCTCAGCACT[T/A]ATAGTTTTAGTGGCAATAATAAAACTATTCTGAGGCATATAACGAGCCCTATTAACCTCA
6108	CaTSNP9124	GTTAAGTATAAGGACAGGGAAGGTGATTTGGTTACTATTACTACTACTTCTGAATTGAGG[C/T]TAGCTGAGGGTTGTCATGTGCTTGGCTCGATTCGGTTATATATTACGCAGGTGGATTTGG
6109	CaTSNP9125	TCCTGCAAAACTGTGGAACTTTGAGTCTAGCTCTGCTCGAATATCAGCAGTAAGTGGCAC[A/G]ACAGAACCATCATCATTGCATAGAATGGTTGTACATTTAGAGATTATACTTTCTGGAGCA
6110	CaTSNP9126	TTCCATAATATCTTGTGTCACAAACTTCATCCTCTTTTCGAGCGATTTATCCGATGCCAC[G/A]ATCTTGCTAGCAACCTCATTAGCCTTCTCATCAATATCAAAATTATCAAAGTGTGTTGAT
6111	CaTSNP9127	TGCCGGAGATTCATCATTAGGGCTAGAAAGCATTGATATGATACTCAACACTATACTCTC[C/T]ACTGTATGAACAGGTGTCCAGCGCTCACTTGCAAGCTCATAACCATTTGGATCATCACCA
6112	CaTSNP9128	TGTGGGAAGTGATGAAATTTCAGAGGCTTCTCAGTTGCTATCTTGTTTGGCTGACTTTGT[T/G]TACTGCACGGTGTGTGCATGCATGCAGACTCAGCATAAGGTTGAAATGGACAAGCGTGAC
6113	CaTSNP9129	AGAAAGTATATATGGAGGAAAGTTTGCAGATGAAAATGTCAAACTAAAACATGATGGACC[T/C]GGTATCCTTTCAATGGCTAATAGTGGTCCTAACACCAACGGATCTCAGTTTTTTATTATA
6114	CaTSNP9130	TCTGTAATATGTAAGCTAGCTTGTGGAAGGGTTCTTCTTTCTGCATCAAGCTTGTTCAAC[G/A]CTGGGTTAGGATTTGGGTTGGTTTGGCTTTCTTGGGCAGTAAGTAAGCTAAAGGCCACGA
6115	CaTSNP9131	ACACATGGCTCCCATCATTAAACCCTCCACGTTCATGTTACCACCTCCTAAGTATACATT[A/G]CCACTCTCTTTATGTTCACTGGCTCCATCTATGACATTCTGGTAAATCTCTTTTGCCATT
6116	CaTSNP9132	CTTCTGTTGGTAGGTTTACCTCTCTTCCTTCTCTGTTCTGACATCTTTTCCCTTTTCACA[C/G]CTCCACCTCCCAACCCATCTCACAACCACCATCGTCACCACCACCACCACCATCAACCAC

6117	CaTSNP9133	CTTTGCCTCTGAAGCCTTGAGAACTCTTTGTTTGGCTGTCAAAGATATAGATGAAACACA[T/A]GGGGAAACCGTCATACCTGAAACTGGATATACTCTGATCGCCATTGTTGGAATCAAGGAT
6118	CaTSNP9134	CCAAACGGAATGCCTAAACAAAAATCATGAATTTTTGCAGTCCTTTTCTGCTGATCAACA[C/T]CTTCCTGGATTGTCTCTCCTGACTCAGTGACATTCTCTTCTATTGTTGAAGACAACTCCT
6119	CaTSNP9135	TGGCCCACAAAATAGCCAAAGGATGGCTTCATTTTTGCTGTATTTAACTAATGTTGAGGA[C/T]GGTGGCGAGACTATGTTTCCATTTGAGAATGGCTCGAACATGGATGGTAGCTATGGCTAC
6120	CaTSNP9136	ATGTATATACTCCTTAACTCCTGGTGTGTTATATGTAAGGTCCAAGTCTCCCACAATAAA[T/C]TTCACCGGTACTTTGATCTGTTCTCCGGTCCATGGCGCCATCAGTTCCCAGGTTAGGTCG
6121	CaTSNP9137	CACAGTTAATTTCTTCTCCTTCATGTCCATAGTGATAGCATCAATCCCTGAAAGTGTTGA[G/A]ACTGCCTTCAAGGCCTTCTGCTTCGCCTTATCATCTGCCAAATCTAACTTCAGCACAAAC
6122	CaTSNP9138	TCTTTATGAACTTCAGTCTACACCACTAGCTGAGACAAAGCGTATTACTGTTCTTAAACC[G/T]TCGAAGATGGTTGACAATGAAAAGTTCAGTAGAAAAGGAAACAATAGCGACAAACATATT
6123	CaTSNP9139	GAGGGAGAAGGATGATGGGCGAAAGAGACGCAGTGATTCAAGTGATAGGGAGAAATATCG[T/C]GATAAAGATCGTGATAGGGAACGTGATAGGTACAGAGGTAGAGACTCTGATCGTGAAAGA
6124	CaTSNP9140	AAGATTGAAACTCTTGAGGAATTTGAAAAAGTTTCTACCAGTGAGGAACGCCAGTCCTTT[G/A]TTGAGAAGCTTGATGAGGTGCAAGATTGGTTATATACAGATGGTGAAGATGCCAACGCTA
6125	CaTSNP9141	TTTCGATTTATCTGGTTTGTTCGAGGACAAAATTCAACAGAAAGAGATGCGGTTTACATC[T/C]AACAAGCCGGCCTCAACTATAATCTCGAAGTTGGAAGAAATTTGCAAGTGTCTTTGCTTG
6126	CaTSNP9142	CATGTTTAACCGCTCCTCCAAAAACGCAGCAACAACGCCATTGGAATCAACCTTTCCCCT[A/G]AGGCGGCACTGTCTTAGGATGTAGTCATAACCAAAGCTAGCAGTAACATCTCTTGACAAG
6127	CaTSNP9143	GAAAGAGAGCGGTGGAACGTAGATGTTTTGTTGAGAATGCTGGTAGAATTGCTTCCTCTC[A/G]TGCACCAAAAAGCAATTGAGACATGCCCATTCTTGTCCACCACAGAAACTACCCCAGCTG
6128	CaTSNP9144	GCTTGGTTCTAAAATTGGTTTGTCTTTAGTACCAGATGCTTCTGGAAGAGACAACGAGCC[A/G]CTACCAGCATCCTCTGAACTATCCTTGCAATGTTCTGAAGTTGTTTCTATAAGATCAGAA
6129	CaTSNP9145	AAGTGAGACACAAATCAAAATATATTACTCCGCGTCTTCCTCATCATCACCATCTTCAGC[T/A]GCCCCAGCATCGTCTCCAATGCCTTCAAGTAGTTGATCGACATCCTCCCCCAACTCCAGC
6130	CaTSNP9146	ACAAGAAGCTTTAGCAACTCTTGTTGTGAACAGACTTAGAGGATCACTGAAGATTGCAGC[A/G]CTTAAAGCCCCTGGATTTGGAGAAAGAAAAAGCCAATACCTTGATGATATTGCCATCTTG
6131	CaTSNP9147	ATTATCATCACTCCAAACACCAGCAACCATGGATGGTAAATGTTCCAACTTTTTCTCTAC[A/G]ACGACAGAGTGAAGAGAGGAAGGAATCTGCTGAGGTTGAAGGCCCTCACGCCTCTTTTTC
6132	CaTSNP9148	CCCGGAGATCAAAGAGTCGCAGTCCAAGAAGGCCTTTGAAAGGAAGAGGCAGTAGCAACA[C/G]TAGCAGCAGCAGTTCACCACCCCCAGCTCGCAAACCATAGATAACTCCTGTATATCTACT
6133	CaTSNP9149	CTTCAATGTGCTGACTGATTTTGATGGCCAGCAACACCTTTTTAATGACTTGAGCTGTTT[G/C]TCACAGCCTGGCCCTGGTTCTTGTCGGTCTAAGACACTAACTCCATCAAATCTTGAAGAG
6134	CaTSNP9150	ATTTGGCGAAAAAGAGACAAAAAGCAAAAGGAGCAACCCCGGGAATTTTCCACGCCTCGA[T/C]AAACCCTACTGCTTTCTCTTTCTCCTCCTTTAACAGAGGTTCTGTGACATCGTCTACGCT
6135	CaTSNP9151	GAGATCCACACAGGAAGCAATAGTTTTGCCACCATGGGTTGCTCTAGCTGTTAGACCAAG[G/A]CCTGGTGTTTGGGAATATCTGAGAGTGAATGTGCATGCTCTTGTTGTTGAAGAATTGCAA
6136	CaTSNP9152	CTATCCTGTCTGCTCCAGGCTTACATCACTAAACTTGAGTTATGCTACAATCCAAAGCAC[G/A]GATCTTATCAAGCTTGTTGGTCAATGTGAAAGTTTGCAGCGGCTATGGGTGCTGGATTAC
6137	CaTSNP9153	GAAGAAATTGGAAGAAGATGAAGCACCGCGAATCTACCACAGCGTCGGATTAATAGTCGG[T/C]GTCACAGGTATCGTCGGCAACAGCCTCGCCGAAATTCTACCTCTCCCCGACACACCAGGC
6138	CaTSNP9154	TATTGGAGTATGTCACAGGGATATTAAACCTCAAAATTTACTGGTGAATCCTCACACCCA[T/C]CAACTGAAAATATGTGACTTTGGAAGTGCTAAAGTCTTGGTAAAAGGTGAACCAAACATA
6139	CaTSNP9155	TTGGGTCTACTCAACATAGATAATGTAGTTGATGTATTTCAACTTGCATTACTGTGTGAT[T/G]CTCCACGGCTCAGTCTCATCTGTCACCGTAAAATAATCAAACACTTCAAAACTGTTTCGG
6140	CaTSNP9156	TTACATACTTTTCCATAAACTTGTATTCCCAATCCTGTAATGCATCAAGCTCAAATGGGC[T/C]GAGACCAGAAATATCGCCGGTTAAATCTTTCTCTTCAAAAGACATCTTTGCTAAAGCTCT
6141	CaTSNP9157	CGCTAGGGTAAGCAGAAACCCCGATTTGATCAGGGGAATCGGAAAATACTCAAAATCTGC[C/T]ATGTACCACAAGAGAGGTCTCTGGGCCATCAAGGCCAAACACGGCGGCGCTTTCCCTAAA
6142	CaTSNP9158	CGACGGTTTTATTAACCTCTCGGAGTTCGCCGCCTTCTGCCGCTCCGGTTCGACAGACGG[T/C]GGCGCCTCAGAGCTACGTGATGCCTTCGATCTGTACGATAAGGACAAGAACGGCCTCATC
6143	CaTSNP9159	TCTCTTCATTATGATTCCAAACATGGCTTATGCAAACCAAAAGCCTATATATATAACTTG[T/A]TGGGAGTTTCCTTGTGATGCTATATGCAAGATGTCTTGTTGTTAAAGTGCTTCCTTCAAG
6144	CaTSNP9160	AGAAAAGTTAATAGAATTTGGCAACAACACAGAAAGGCTTTCAGGTATGTTTCCAGTTAA[T/G]AGATTATTTGAAAGATCAAGAAGGTTAAGTGATTCCAACAAAGAAAGTGAACTAGGAATA

Table S2: Genetic positions (cM) of 6698 markers distributed on eight LGs of the chickpea interspecific linkage map

S. No.	Linkage groups (LGs)	Marker Name	Genetic Positions (cM)	Scaffolds	Size (bp)
1	CaLG1	CESSR139	0	scaffold00763	110066
2	CaLG1	PIP160	6.328	-	-
3	CaLG1	PIP146	7.275	-	-
4	CaLG1	NCPGR6	12.72	scaffold00692	143771
6	CaLG1	CaGMS433	14.7	-	-
7	CaLG1	CaGMS453	14.7	-	-
5	CaLG1	CaGMS462	14.7	-	-
8	CaLG1	TS57	14.8	-	-
9	CaLG1	CaGMS1146	15.99	-	-
10	CaLG1	CaGMS321	16.09	-	-
11	CaLG1	H3H021	16.17	-	-
12	CaLG1	CESSR43	16.2	-	-
13	CaLG1	PIP152	16.95	-	-
14	CaLG1	CaTMS1095	18.6	-	-
15	CaLG1	TA203	18.84	-	-
16	CaLG1	CaTMS652	19.2	-	-
17	CaLG1	CaTMS746	19.21	-	-
18	CaLG1	CaTMS1110	19.23	-	-
20	CaLG1	CaTMS870	19.24	-	-
19	CaLG1	CaTMS928	19.24	-	-
21	CaLG1	GAA40	21.63	-	-
22	CaLG1	CaSNP4447	21.82	scaffold02944	32324
23	CaLG1	CaGMS51	24.05	-	-
24	CaLG1	CESSR42	24.1	scaffold00334	229764

25	CaLG1	CESSR45	24.14	-	-
26	CaLG1	CaGMS1318	25.49	-	-
27	CaLG1	CaGMS1234	25.49	-	-
28	CaLG1	CaGMS1313	25.49	-	-
29	CaLG1	CaTMS977	25.54	-	-
30	CaLG1	CaGMS1297	26.4	-	-
31	CaLG1	CaSNP710	28.18	-	-
32	CaLG1	CaGMS331	28.24	-	-
33	CaLG1	CESSR159	28.52	-	-
34	CaLG1	NCPGR33	28.65	scaffold03001	29165
35	CaLG1	CaTSNP6056	30.08	-	-
36	CaLG1	CaSNP3970	31.4	scaffold02261	44496
37	CaLG1	TR43	32.07	-	-
38	CaLG1	NCPGR165	33.04	-	-
39	CaLG1	CaTSNP7283	33.14	-	-
40	CaLG1	CaTSNP7669	34.31	-	-
41	CaLG1	CaSNP915	34.87	scaffold00497	144034
42	CaLG1	CaTSNP6814	35.29	scaffold05768	10170
43	CaLG1	CaTSNP6924	35.29	-	-
44	CaLG1	CaTSNP7255	35.4	-	-
45	CaLG1	CaTSNP8887	35.41	scaffold00076	413143
46	CaLG1	CaSNP215	35.5	scaffold00066	415502
47	CaLG1	CESSR433	35.51	-	-
48	CaLG1	CaTSNP9043	35.64	-	-
49	CaLG1	CaTSNP7543	35.65	-	-
50	CaLG1	CaTSNP8323	35.7	-	-
51	CaLG1	CaTSNP7793	35.78	-	-
52	CaLG1	CaSNP883	35.78	-	-
53	CaLG1	CaTSNP7659	35.84	-	-

54	CaLG1	CaTSNP8246	35.85	-	-
55	CaLG1	CaSNP555	35.86	-	-
56	CaLG1	CaSNP53	35.91	-	-
57	CaLG1	CaSNP249	35.93	scaffold01580	67777
58	CaLG1	CaSNP164	35.99	9096683	34354
59	CaLG1	CaSNP218	36.02	scaffold00082	406676
60	CaLG1	CaTSNP6202	36.11	-	-
61	CaLG1	CaTSNP8594	36.12	-	-
62	CaLG1	CaTSNP8304	36.12	-	-
63	CaLG1	CaSNP166	36.12	scaffold00132	303872
64	CaLG1	CaTSNP7576	36.15	9213002	55988
65	CaLG1	CaSNP743	36.17	-	-
66	CaLG1	CaTSNP8652	36.27	-	-
67	CaLG1	CaSNP2327	36.42	scaffold00981	100022
68	CaLG1	CaTSNP7173	36.47	-	-
69	CaLG1	CaTSNP6170	36.5	-	-
70	CaLG1	CaTSNP9053	36.52	-	-
71	CaLG1	CaTSNP6214	36.54	-	-
72	CaLG1	CaTSNP6685	36.55	-	-
73	CaLG1	CaTSNP7234	36.56	-	-
74	CaLG1	CaSNP173	36.57	scaffold00243	236162
75	CaLG1	CaTSNP6206	36.59	-	-
76	CaLG1	CaTSNP8945	36.59	-	-
77	CaLG1	CaTSNP7175	36.64	-	-
78	CaLG1	CaTSNP7911	36.68	-	-
79	CaLG1	CaTSNP7978	36.69	-	-
80	CaLG1	CaSNP5053	36.71	9133848	45669
81	CaLG1	CaSNP549	36.74	scaffold02356	39114
82	CaLG1	CaTSNP7030	36.74	-	-

83	CaLG1	CEST86	36.81	-	-
84	CaLG1	CaSNP2731	36.82	-	-
85	CaLG1	CaSNP2004	36.83	scaffold00406	173097
86	CaLG1	CaTSNP7135	36.91	-	-
87	CaLG1	CaSNP4130	36.93	scaffold00347	181673
88	CaLG1	CaSNP2262	37.06	-	-
89	CaLG1	CaTSNP7163	37.07	-	-
90	CaLG1	CaSNP5105	37.07	scaffold00025	636138
91	CaLG1	CaSNP3513	37.13	scaffold06342	8729
92	CaLG1	CaSNP4572	37.16	scaffold03416	25492
93	CaLG1	CaTSNP7101	37.28	-	-
94	CaLG1	CaTSNP6633	37.37	-	-
95	CaLG1	PIP220	37.43	-	-
96	CaLG1	CaSNP2790	37.44	scaffold01188	77233
97	CaLG1	CaSNP714	37.5	-	-
98	CaLG1	CaSNP2419	37.61	scaffold01141	79431
99	CaLG1	CaTSNP7764	37.65	-	-
100	CaLG1	CaTSNP7069	37.74	-	-
101	CaLG1	CaTSNP8859	37.93	scaffold00824	119875
102	CaLG1	CaSNP421	37.93	scaffold00800	115869
103	CaLG1	CaTSNP7634	37.97	-	-
104	CaLG1	CaTSNP6393	38.09	scaffold00713	145455
106	CaLG1	CaSNP2214	38.17	-	-
105	CaLG1	CaSNP4302	38.17	-	-
107	CaLG1	CaSNP3527	38.55	scaffold00304	190190
108	CaLG1	CaTSNP7211	38.55	-	-
109	CaLG1	CaSNP3521	38.72	scaffold01115	100803
110	CaLG1	CaTSNP6209	38.81	scaffold02082	44897
111	CaLG1	CaTSNP8683	38.81	-	-

112	CaLG1	CaTSNP7154	38.85	-	-
113	CaLG1	CaTSNP7244	38.92	-	-
114	CaLG1	CaTSNP8916	38.92	-	-
115	CaLG1	CaSNP4039	38.94	scaffold01262	80297
116	CaLG1	CaTSNP8001	39.33	-	-
117	CaLG1	CaSNP2191	39.42	scaffold00548	143078
118	CaLG1	CaSNP2358	39.51	-	-
119	CaLG1	CaTSNP8648	39.51	-	-
120	CaLG1	CaSNP55	39.53	-	-
122	CaLG1	CaTSNP6408	39.55	-	-
121	CaLG1	CaTSNP8075	39.55	-	-
123	CaLG1	CaSNP3802	39.55	scaffold02444	39306
124	CaLG1	CaTSNP7655	39.59	-	-
125	CaLG1	CaTSNP8443	39.59	-	-
126	CaLG1	CaSNP3257	39.61	scaffold01724	60528
127	CaLG1	CaSNP2078	39.65	scaffold00046	466868
128	CaLG1	CaSNP3961	39.65	-	-
129	CaLG1	CaSNP2627	39.67	scaffold00831	109251
130	CaLG1	CaSNP372	39.67	-	-
131	CaLG1	CaSNP440	39.67	-	-
132	CaLG1	CaSNP843	39.7	-	-
133	CaLG1	CaSNP4081	39.71	scaffold02598	36056
134	CaLG1	CaSNP3732	39.78	-	-
135	CaLG1	CaTMS873	39.8	-	-
136	CaLG1	CaSNP472	39.87	9210538	55153
137	CaLG1	CaSNP3120	39.93	-	-
138	CaLG1	CaTSNP7759	39.94	scaffold00720	138634
139	CaLG1	CaTSNP8737	40.04	-	-
140	CaLG1	CaSNP3246	40.08	scaffold00876	100638

141	CaLG1	CaTSNP8971	40.14	scaffold01036	86467
142	CaLG1	CaSNP327	40.19	-	-
143	CaLG1	CaTSNP8513	40.19	-	-
144	CaLG1	CaSNP2093	40.26	-	-
145	CaLG1	CaSNP131	40.28	scaffold00013	708368
146	CaLG1	CaTSNP8663	40.34	-	-
147	CaLG1	CaSNP81	40.36	scaffold01307	83897
148	CaLG1	CaTSNP8932	40.37	-	-
149	CaLG1	CaTSNP7645	40.41	-	-
150	CaLG1	CaSNP3090	40.42	scaffold01918	59264
151	CaLG1	CaSNP1972	40.43	9202483	40420
152	CaLG1	CaSNP689	40.52	-	-
153	CaLG1	CaSNP396	40.6	-	-
154	CaLG1	CaTSNP8480	40.82	-	-
155	CaLG1	CaTSNP8659	40.93	scaffold05689	10359
156	CaLG1	CaTSNP6245	41.21	-	-
157	CaLG1	CaTSNP8657	41.52	-	-
158	CaLG1	CaTSNP9022	41.56	-	-
159	CaLG1	CaTSNP8203	41.57	-	-
160	CaLG1	CaTSNP6611	41.58	-	-
161	CaLG1	CaTSNP6311	41.62	scaffold00453	170763
162	CaLG1	CaTSNP7505	41.67	-	-
163	CaLG1	CaTSNP6847	41.74	-	-
164	CaLG1	CaTSNP7490	41.78	-	-
165	CaLG1	CaTSNP8564	42.47	-	-
166	CaLG1	CaTSNP8940	42.7	-	-
167	CaLG1	CaTSNP8684	42.72	-	-
168	CaLG1	CaTSNP6729	42.72	-	-
169	CaLG1	CaTSNP8759	42.77	-	-

170	CaLG1	CaTSNP8233	42.8	-	-
172	CaLG1	CaTSNP6190	43.25	-	-
171	CaLG1	CaTSNP6569	43.25	-	-
173	CaLG1	CaTSNP8512	43.88	-	-
174	CaLG1	CaTSNP9030	44.17	-	-
175	CaLG1	CaTSNP7877	44.2	-	-
176	CaLG1	CEST104	44.21	-	-
177	CaLG1	CaTSNP8422	44.23	-	-
178	CaLG1	CaTMS673	44.38	-	-
179	CaLG1	CaSNP3397	44.39	-	-
180	CaLG1	CaSNP2891	44.43	scaffold00043	491956
181	CaLG1	CaSNP2834	44.46	scaffold00026	610816
182	CaLG1	CaTSNP6063	44.55	-	-
183	CaLG1	CaTSNP7894	44.55	-	-
185	CaLG1	CaTSNP7596	44.61	-	-
184	CaLG1	CaTSNP7846	44.61	-	-
186	CaLG1	CaTSNP6878	45.21	scaffold01404	68698
187	CaLG1	CaTSNP7916	45.21	-	-
188	CaLG1	CaTSNP7081	45.35	-	-
189	CaLG1	CaTSNP7636	45.37	-	-
190	CaLG1	CaTSNP7675	45.37	-	-
191	CaLG1	CaTSNP7201	45.4	9137411	44100
192	CaLG1	CaTSNP6986	45.46	-	-
193	CaLG1	CaTSNP7393	45.46	-	-
194	CaLG1	CaTSNP8133	45.52	-	-
195	CaLG1	CaTSNP6267	45.53	scaffold00303	197652
196	CaLG1	CaTSNP8198	45.54	-	-
197	CaLG1	CaTSNP8324	45.54	-	-
198	CaLG1	CaTSNP6243	45.56	-	-

199	CaLG1	CaTSNP9057	45.56	-	-
200	CaLG1	CaTSNP8448	45.57	-	-
201	CaLG1	CaTSNP6465	45.58	-	-
202	CaLG1	CaTSNP7713	45.63	-	-
203	CaLG1	CaTSNP6035	45.8	-	-
204	CaLG1	CaTSNP8629	45.8	-	-
205	CaLG1	CaTSNP8973	45.81	-	-
206	CaLG1	CaTSNP8386	45.84	-	-
207	CaLG1	CaTSNP7033	45.88	-	-
208	CaLG1	CaTSNP7577	46	-	-
209	CaLG1	CaTSNP7330	46.13	-	-
210	CaLG1	CaTSNP8733	46.14	-	-
212	CaLG1	CaTSNP6093	46.14	-	-
211	CaLG1	CaTSNP6555	46.14	scaffold01351	69045
213	CaLG1	CaTSNP8541	46.15	-	-
214	CaLG1	CaTSNP8841	46.19	-	-
215	CaLG1	CaTSNP9092	46.29	-	-
216	CaLG1	CaTSNP6974	46.34	scaffold03254	25939
217	CaLG1	CaSNP2479	46.62	scaffold01487	84264
218	CaLG1	CaSNP4328	46.76	scaffold01090	91485
219	CaLG1	CaTSNP8058	46.78	-	-
220	CaLG1	CaSNP4839	46.82	scaffold06277	9389
221	CaLG1	CaTSNP7521	46.84	-	-
222	CaLG1	CaTMS955	46.88	-	-
224	CaLG1	CaTSNP6783	46.9	9207696	40749
223	CaLG1	CaTSNP8860	46.9	-	-
225	CaLG1	CaSNP2115	46.98	scaffold00217	247421
226	CaLG1	CaSNP3130	47.07	scaffold02027	47022
227	CaLG1	CaSNP3302	47.07	-	-

228	CaLG1	CaSNP2285	47.07	scaffold00205	241351
229	CaLG1	CaSNP2804	47.08	scaffold01208	87731
230	CaLG1	CaTSNP6616	47.09	-	-
231	CaLG1	CaTSNP7295	47.09	9208649	33868
232	CaLG1	CaTSNP7567	47.12	-	-
233	CaLG1	CaSNP1932	47.25	9202602	37024
235	CaLG1	CaTSNP8326	47.25	-	-
234	CaLG1	CaTSNP8870	47.25	-	-
236	CaLG1	CaSNP2970	47.45	9215808	20494
237	CaLG1	CaSNP2445	47.56	scaffold01450	74240
238	CaLG1	CaTSNP6375	47.65	scaffold01119	92110
239	CaLG1	CaTSNP8066	47.65	-	-
240	CaLG1	CaTSNP9126	47.66	-	-
241	CaLG1	CaTSNP7474	47.67	-	-
242	CaLG1	CaSNP2414	47.72	scaffold00433	158128
243	CaLG1	CaTSNP7513	47.81	-	-
247	CaLG1	CaTSNP6801	47.85	scaffold02450	39527
245	CaLG1	CaTSNP7058	47.85	-	-
248	CaLG1	CaTSNP7614	47.85	-	-
246	CaLG1	CaTSNP7784	47.85	-	-
244	CaLG1	CaTSNP7961	47.85	-	-
249	CaLG1	CaTSNP9146	47.85	-	-
250	CaLG1	CaTSNP6651	47.86	-	-
251	CaLG1	CaTSNP7166	47.86	scaffold03226	28959
252	CaLG1	CaTSNP8863	47.87	-	-
253	CaLG1	CaSNP3587	47.88	-	-
254	CaLG1	CaSNP2079	47.95	scaffold00054	437934
255	CaLG1	CaTSNP7296	48.01	scaffold01313	90114
256	CaLG1	CaSNP5149	48.02	-	-

257	CaLG1	CaSNP2325	48.04	scaffold00546	145604
258	CaLG1	CaSNP2104	48.09	9174820	48193
259	CaLG1	CaSNP3611	48.12	9202516	2550
260	CaLG1	CaSNP5022	48.14	-	-
261	CaLG1	CaTSNP8160	48.2	-	-
262	CaLG1	CaSNP2286	48.22	scaffold01002	87513
266	CaLG1	CaSNP2236	48.23	9208845	25833
263	CaLG1	CaSNP2815	48.23	-	-
264	CaLG1	CaSNP3896	48.23	scaffold02237	42110
265	CaLG1	CaSNP4567	48.23	scaffold02943	29871
267	CaLG1	CaSNP3574	48.24	scaffold00350	200794
268	CaLG1	CaSNP1992	48.24	scaffold01063	97092
269	CaLG1	CaTSNP9148	48.33	-	-
270	CaLG1	CaSNP2580	48.4	scaffold00769	127520
271	CaLG1	CaSNP4371	48.48	-	-
272	CaLG1	CaTSNP7412	48.51	-	-
273	CaLG1	CaSNP7	48.51	-	-
274	CaLG1	CaSNP2525	48.56	-	-
275	CaLG1	CaSNP3216	48.7	9212167	54426
276	CaLG1	CaTSNP6382	48.8	-	-
277	CaLG1	CaTSNP7789	48.8	-	-
278	CaLG1	CaTSNP8340	48.81	-	-
279	CaLG1	CaSNP2565	48.84	scaffold01500	63463
280	CaLG1	CaTMS618	48.88	-	-
281	CaLG1	CaTSNP6235	48.89	-	-
282	CaLG1	CaTSNP8302	48.89	-	-
283	CaLG1	CaTSNP6336	48.9	scaffold01931	49443
284	CaLG1	CaTSNP7434	48.9	-	-
285	CaLG1	CaTSNP7833	48.91	-	-

286	CaLG1	CaSNP3441	48.92	-	-
287	CaLG1	CaSNP2397	48.92	scaffold00319	192175
288	CaLG1	CaTSNP8603	48.93	-	-
289	CaLG1	CaTSNP8821	48.94	-	-
290	CaLG1	CaSNP4889	48.96	scaffold01403	74309
291	CaLG1	CaTMS544	48.96	-	-
292	CaLG1	CaTSNP8851	49.12	-	-
293	CaLG1	CaSNP3329	49.3	scaffold00782	124586
294	CaLG1	CaSNP124	49.54	scaffold00223	223726
295	CaLG1	CaSNP4831	49.66	scaffold03195	28384
296	CaLG1	CaSNP3174	49.66	scaffold00591	143690
297	CaLG1	CaSNP2009	49.66	scaffold00668	131427
298	CaLG1	CaSNP4099	49.68	scaffold00910	108946
299	CaLG1	CaSNP3652	49.69	scaffold02324	46401
300	CaLG1	CaSNP2728	49.69	scaffold01612	63302
301	CaLG1	CaSNP4107	49.69	scaffold01005	87807
302	CaLG1	CaSNP2117	49.7	scaffold02086	45929
304	CaLG1	CaSNP2226	49.7	scaffold00786	113799
305	CaLG1	CaSNP2446	49.7	scaffold05457	11053
306	CaLG1	CaSNP2558	49.7	scaffold00938	103824
308	CaLG1	CaSNP2746	49.7	scaffold01399	77720
307	CaLG1	CaSNP3212	49.7	scaffold00485	164063
309	CaLG1	CaSNP3428	49.7	9203845	7026
303	CaLG1	CaSNP3810	49.7	scaffold01787	63493
310	CaLG1	CaSNP4027	49.7	scaffold01318	118135
311	CaLG1	CaSNP4449	49.7	scaffold03402	41966
312	CaLG1	CaSNP2601	49.7	scaffold00832	123037
313	CaLG1	CaSNP2839	49.7	scaffold01467	81987
314	CaLG1	CaSNP3650	49.7	scaffold02852	61459

315	CaLG1	CaSNP3990	49.7	-	-
316	CaLG1	CaSNP3557	49.71	9207810	30879
317	CaLG1	CaSNP3095	49.71	scaffold00268	236171
318	CaLG1	CaSNP3394	49.71	scaffold02498	41638
321	CaLG1	CaSNP2021	49.71	9203344	14029
319	CaLG1	CaSNP2462	49.71	scaffold00657	131174
320	CaLG1	CaSNP2608	49.71	scaffold00099	339370
322	CaLG1	CaSNP4262	49.71	scaffold02436	47207
323	CaLG1	CaSNP4499	49.71	scaffold00683	145162
324	CaLG1	CaSNP3502	49.71	scaffold01371	72166
325	CaLG1	CaSNP2708	49.72	scaffold04679	15421
326	CaLG1	CaSNP3876	49.72	scaffold08799	5308
328	CaLG1	CaSNP4496	49.72	scaffold04846	13471
327	CaLG1	CaSNP4740	49.72	-	-
329	CaLG1	CaSNP2999	49.72	-	-
331	CaLG1	CaSNP2681	49.72	scaffold00748	124336
330	CaLG1	CaSNP3766	49.72	-	-
334	CaLG1	CaSNP4162	49.72	scaffold03367	30852
332	CaLG1	CaSNP4759	49.72	scaffold05882	9886
333	CaLG1	CaSNP5059	49.72	scaffold15706	2250
335	CaLG1	CaSNP4848	49.72	-	-
336	CaLG1	CaSNP4778	49.72	scaffold02619	34796
337	CaLG1	CaSNP3637	49.73	scaffold02050	45502
338	CaLG1	CaSNP2844	49.73	scaffold01130	86028
340	CaLG1	CaSNP2520	49.73	scaffold01530	70908
339	CaLG1	CaSNP2545	49.73	scaffold01644	65645
341	CaLG1	CaSNP3510	49.73	-	-
342	CaLG1	CaSNP4325	49.73	9214046	5898
344	CaLG1	CaSNP3191	49.74	scaffold00297	225323

343	CaLG1	CaSNP4377	49.74	9214667	11411
346	CaLG1	CaSNP2048	49.74	scaffold00730	149623
347	CaLG1	CaSNP2754	49.74	scaffold02205	42072
348	CaLG1	CaSNP3334	49.74	9214706	36730
345	CaLG1	CaSNP4372	49.74	scaffold02791	32876
349	CaLG1	CaSNP4810	49.74	scaffold03202	34429
350	CaLG1	CaSNP4558	49.74	scaffold02693	55084
351	CaLG1	CaSNP5122	49.74	-	-
361	CaLG1	CaSNP2813	49.74	scaffold01529	63915
365	CaLG1	CaSNP2820	49.74	scaffold01419	66644
359	CaLG1	CaSNP3017	49.74	scaffold02475	47569
358	CaLG1	CaSNP3073	49.74	scaffold00521	155034
357	CaLG1	CaSNP3198	49.74	-	-
360	CaLG1	CaSNP3348	49.74	-	-
352	CaLG1	CaSNP3447	49.74	scaffold02448	58165
353	CaLG1	CaSNP3463	49.74	9205477	42466
364	CaLG1	CaSNP3830	49.74	scaffold00274	232240
363	CaLG1	CaSNP3848	49.74	scaffold02211	59693
354	CaLG1	CaSNP3976	49.74	scaffold01197	89279
362	CaLG1	CaSNP4152	49.74	9212470	3364
355	CaLG1	CaSNP4932	49.74	scaffold00980	113528
356	CaLG1	CaSNP4943	49.74	scaffold03137	36893
366	CaLG1	CaSNP2185	49.74	scaffold01624	66790
367	CaLG1	CaSNP2920	49.74	9181938	19858
368	CaLG1	CaSNP4469	49.75	scaffold05013	15290
369	CaLG1	CaSNP2704	49.75	scaffold06118	9249
370	CaLG1	CaSNP4616	49.75	scaffold02559	36356
371	CaLG1	CaSNP2423	49.75	scaffold01452	91271
373	CaLG1	CaSNP2847	49.75	scaffold00774	114496

372	CaLG1	CaSNP3877	49.75	scaffold05125	12302
375	CaLG1	CaSNP3920	49.75	9212089	12739
374	CaLG1	CaSNP3995	49.75	scaffold06514	8331
382	CaLG1	CaSNP2546	49.75	scaffold09552	5632
379	CaLG1	CaSNP2776	49.75	scaffold00625	153601
384	CaLG1	CaSNP2833	49.75	scaffold02827	40221
376	CaLG1	CaSNP2948	49.75	scaffold01797	54507
380	CaLG1	CaSNP3044	49.75	-	-
387	CaLG1	CaSNP3099	49.75	-	-
385	CaLG1	CaSNP3181	49.75	scaffold00111	375405
388	CaLG1	CaSNP3368	49.75	scaffold01767	53538
377	CaLG1	CaSNP3480	49.75	scaffold02271	45289
383	CaLG1	CaSNP3712	49.75	scaffold02394	41383
381	CaLG1	CaSNP4256	49.75	-	-
378	CaLG1	CaSNP4466	49.75	scaffold03236	26289
386	CaLG1	CaSNP5003	49.75	scaffold04971	13035
389	CaLG1	CaSNP2807	49.75	scaffold00237	230927
391	CaLG1	CaSNP3300	49.75	scaffold00839	111126
390	CaLG1	CaSNP5061	49.75	scaffold02163	57816
395	CaLG1	CaSNP2864	49.75	scaffold01259	80990
392	CaLG1	CaSNP2878	49.75	9204557	28452
396	CaLG1	CaSNP3501	49.75	scaffold02340	51193
394	CaLG1	CaSNP3857	49.75	scaffold01284	78385
393	CaLG1	CaSNP4289	49.75	-	-
398	CaLG1	CaSNP2503	49.75	scaffold00502	150052
403	CaLG1	CaSNP3662	49.75	-	-
400	CaLG1	CaSNP3909	49.75	-	-
397	CaLG1	CaSNP4074	49.75	-	-
401	CaLG1	CaSNP4412	49.75	scaffold02930	33770

399	CaLG1	CaSNP4737	49.75	9200971	15834
402	CaLG1	CaSNP5071	49.75	-	-
404	CaLG1	CaSNP2518	49.75	scaffold00681	124284
406	CaLG1	CaSNP2856	49.76	scaffold01031	92578
405	CaLG1	CaSNP3886	49.76	scaffold02605	34898
407	CaLG1	CaSNP3215	49.76	-	-
409	CaLG1	CaSNP3361	49.76	scaffold01268	96139
408	CaLG1	CaSNP4593	49.76	scaffold02578	40073
410	CaLG1	CaSNP3967	49.76	-	-
411	CaLG1	CaSNP2759	49.76	scaffold01636	58357
412	CaLG1	CaSNP3704	49.76	-	-
413	CaLG1	CaSNP2290	49.76	scaffold06506	8342
414	CaLG1	CaSNP3555	49.77	-	-
415	CaLG1	CaSNP4556	49.77	scaffold02127	50952
416	CaLG1	CaSNP5064	49.77	scaffold01728	73803
417	CaLG1	CaSNP2989	49.77	scaffold05422	11628
418	CaLG1	CaSNP4691	49.78	scaffold02664	58975
419	CaLG1	CaSNP4699	49.78	scaffold00273	242905
420	CaLG1	CaSNP4722	49.78	scaffold03405	31414
426	CaLG1	CaSNP3744	49.79	-	-
424	CaLG1	CaSNP4327	49.79	scaffold02376	39270
427	CaLG1	CaSNP4350	49.79	scaffold01308	90298
425	CaLG1	CaSNP4479	49.79	scaffold03473	30410
422	CaLG1	CaSNP4564	49.79	-	-
423	CaLG1	CaSNP4805	49.79	scaffold01562	74069
421	CaLG1	CaSNP4887	49.79	scaffold03242	26123
428	CaLG1	CaSNP4704	49.79	scaffold04344	16274
429	CaLG1	CaSNP4610	49.79	scaffold02004	63567
430	CaLG1	CaSNP3395	49.79	scaffold01081	108540

431	CaLG1	CaSNP2213	49.79	9215374	34533
432	CaLG1	CaSNP2421	49.79	scaffold00565	140709
433	CaLG1	CaSNP2233	49.8	scaffold00700	124226
435	CaLG1	CaSNP3399	49.8	scaffold01087	88333
434	CaLG1	CaSNP5117	49.8	scaffold01832	64728
436	CaLG1	CaSNP4742	49.82	scaffold02601	34949
437	CaLG1	CaSNP3315	49.82	scaffold00534	171320
438	CaLG1	CaSNP2551	49.83	scaffold00695	128496
439	CaLG1	CaSNP4906	49.83	-	-
440	CaLG1	CaSNP2146	49.84	scaffold02778	51712
441	CaLG1	CaSNP2085	49.86	scaffold01096	89397
442	CaLG1	CaSNP2741	49.89	scaffold02666	38511
443	CaLG1	CaSNP4260	49.91	scaffold02268	45771
444	CaLG1	CaSNP2803	49.93	scaffold01150	82662
445	CaLG1	CaSNP4876	50.02	scaffold00977	111647
446	CaLG1	CaSNP2832	50.11	scaffold01391	86866
447	CaLG1	CaSNP4824	50.14	-	-
448	CaLG1	CaSNP3152	50.14	scaffold01941	49505
449	CaLG1	CaSNP3779	50.14	scaffold04140	17680
450	CaLG1	CaSNP3844	50.14	scaffold01658	66330
451	CaLG1	CaSNP820	50.36	-	-
452	CaLG1	CaSNP3940	50.39	-	-
453	CaLG1	CaTMS731	50.4	-	-
454	CaLG1	CaSNP3275	50.45	-	-
455	CaLG1	CaSNP3987	50.45	scaffold01000	100955
456	CaLG1	CaSNP2241	50.5	scaffold00341	198169
457	CaLG1	CaGMS31	50.8	scaffold04999	20786
458	CaLG1	CaSNP4040	50.86	-	-
459	CaLG1	CaTMS557	51	-	-

460	CaLG1	CaSNP3828	51.01	scaffold03842	20932
461	CaLG1	CaTSNP7418	51.07	-	-
462	CaLG1	CaSNP911	51.1	scaffold00484	177884
463	CaLG1	CaSNP2330	51.12	scaffold00254	241771
464	CaLG1	CaSNP4970	51.15	scaffold01803	56189
465	CaLG1	CaTSNP8159	51.16	scaffold01415	68337
466	CaLG1	CaTSNP6544	51.17	-	-
467	CaLG1	CaTSNP6410	51.22	scaffold05282	11837
468	CaLG1	CaSNP3430	51.26	-	-
469	CaLG1	CaTSNP6749	51.32	scaffold00793	137549
470	CaLG1	CaTSNP6512	51.34	-	-
471	CaLG1	CaSNP3787	51.42	scaffold02701	34192
472	CaLG1	CaTSNP6846	51.42	-	-
473	CaLG1	CaSNP2162	51.44	scaffold00438	162849
475	CaLG1	CaTSNP6965	51.49	-	-
474	CaLG1	CaTSNP8327	51.49	scaffold01250	102959
476	CaLG1	CaTSNP7008	51.49	9205518	17583
477	CaLG1	CaTSNP6646	51.51	scaffold03276	25732
478	CaLG1	CaTSNP6800	51.51	-	-
479	CaLG1	CaTSNP7520	51.51	-	-
480	CaLG1	CaTSNP6258	51.53	-	-
481	CaLG1	CaTSNP7893	51.53	-	-
482	CaLG1	CaTSNP6395	51.55	scaffold01062	88526
483	CaLG1	CaTSNP7630	51.55	-	-
487	CaLG1	CaSNP2037	51.58	scaffold00233	217940
486	CaLG1	CaSNP2910	51.58	-	-
485	CaLG1	CaSNP3484	51.58	-	-
484	CaLG1	CaSNP3925	51.58	scaffold02507	36613
488	CaLG1	CaTSNP6014	51.67	scaffold03312	34240

489	CaLG1	CaTSNP8692	51.67	-	-
490	CaLG1	CaTSNP6831	51.69	-	-
491	CaLG1	CaTSNP6835	51.75	-	-
492	CaLG1	CaTSNP6173	51.76	scaffold04761	14840
493	CaLG1	CaTSNP6124	51.76	scaffold04867	24575
494	CaLG1	CaTSNP6073	51.78	-	-
495	CaLG1	CaTSNP6141	51.78	-	-
496	CaLG1	CaTSNP8095	51.78	-	-
497	CaLG1	CaTSNP8551	51.78	-	-
498	CaLG1	CaSNP3985	51.79	scaffold03262	42015
499	CaLG1	CaTSNP8995	51.81	scaffold01572	61527
501	CaLG1	CaTSNP7232	51.86	-	-
500	CaLG1	CaTSNP7854	51.86	-	-
502	CaLG1	CaTSNP7953	51.92	-	-
503	CaLG1	CaTSNP6786	51.92	-	-
504	CaLG1	CaTSNP8130	51.94	-	-
505	CaLG1	CaSNP612	52	-	-
506	CaLG1	CaTSNP8554	52.07	-	-
507	CaLG1	CaSNP390	52.2	9185398	33040
508	CaLG1	ESNP18	52.21	-	-
509	CaLG1	CaTSNP6117	52.24	-	-
510	CaLG1	CESSR20	52.24	-	-
511	CaLG1	CaTSNP7923	52.27	-	-
512	CaLG1	CaSNP253	52.32	scaffold01649	76123
513	CaLG1	CaSNP387	52.35	-	-
514	CaLG1	CaSNP2980	52.37	-	-
515	CaLG1	CaSNP242	52.38	scaffold00899	99654
516	CaLG1	ESNP64	52.38	-	-
517	CaLG1	CaTSNP7366	52.39	scaffold02109	50514

518	CaLG1	PIP54	52.49	-	-
519	CaLG1	CaTSNP8831	52.53	-	-
520	CaLG1	CaTSNP6467	52.55	-	-
521	CaLG1	CaTSNP7947	52.63	-	-
522	CaLG1	CaSNP632	52.71	-	-
523	CaLG1	CaSNP209	52.74	scaffold00439	167076
524	CaLG1	ESNP32	52.74	-	-
525	CaLG1	ESNP15	52.76	-	-
526	CaLG1	ESNP33	52.76	9211845	14933
527	CaLG1	CaSNP5139	52.97	scaffold02467	55637
528	CaLG1	CaSNP71	52.98	-	-
529	CaLG1	CaTSNP6994	52.98	-	-
531	CaLG1	CaTSNP6426	52.99	-	-
530	CaLG1	CaTSNP7711	52.99	-	-
532	CaLG1	CaTSNP6172	52.99	-	-
533	CaLG1	CaSNP3847	53.11	scaffold01279	73912
534	CaLG1	CaSNP14	53.54	scaffold00183	263231
535	CaLG1	CaSNP2132	53.87	scaffold00167	277535
536	CaLG1	CaSNP2426	53.87	-	-
537	CaLG1	CaSNP702	53.97	-	-
538	CaLG1	CaSNP815	54.02	9203123	65001
539	CaLG1	ESNP14	54.06	-	-
540	CaLG1	CaTSNP6902	54.19	-	-
541	CaLG1	CaSNP810	54.22	scaffold01584	65821
542	CaLG1	CaSNP784	54.22	scaffold02790	39485
543	CaLG1	CaSNP578	54.42	scaffold00145	278516
544	CaLG1	CaSNP203	54.48	scaffold00841	113703
545	CaLG1	CaSNP3270	54.56	scaffold01993	52377
546	CaLG1	CaTSNP6726	54.72	-	-

547	CaLG1	CaTSNP9027	54.74	-	-
548	CaLG1	CaTSNP6991	55.68	-	-
549	CaLG1	CaSNP3457	55.78	scaffold01146	95646
550	CaLG1	CaTMS776	56.06	-	-
551	CaLG1	CaTSNP7143	56.34	-	-
552	CaLG1	CaTSNP7298	56.34	-	-
553	CaLG1	CaSNP4530	56.4	-	-
554	CaLG1	CEST171	56.71	scaffold00125	322820
555	CaLG1	CaSNP4132	57.03	scaffold01247	84283
556	CaLG1	CaSNP2270	57.06	9182023	34282
557	CaLG1	CaGMS14	57.19	-	-
558	CaLG1	CaTSNP6106	58.04	-	-
559	CaLG1	CaTSNP8646	58.65	scaffold00397	173164
560	CaLG1	CaTSNP7216	58.74	-	-
561	CaLG1	CaTSNP8046	58.75	-	-
562	CaLG1	CaSNP2024	59.11	scaffold00867	110545
563	CaLG1	CaSNP3849	59.11	-	-
564	CaLG1	CaGMS1195	59.13	-	-
565	CaLG1	CaSNP3851	59.18	scaffold01154	80610
566	CaLG1	CaSNP298	59.28	scaffold00432	160480
567	CaLG1	CaSNP2919	59.41	-	-
568	CaLG1	CaTSNP7404	59.43	-	-
569	CaLG1	CaSNP3592	59.48	scaffold01029	89268
570	CaLG1	CaTSNP8900	59.49	-	-
571	CaLG1	CaTSNP8052	59.56	-	-
572	CaLG1	CaSNP2779	59.57	scaffold00927	107791
573	CaLG1	CaTSNP7091	59.58	-	-
574	CaLG1	CaTSNP8226	59.58	-	-
575	CaLG1	CaTMS893	59.72	-	-

576	CaLG1	CaSNP359	59.89	scaffold00906	98813
577	CaLG1	CaSNP795	59.89	-	-
578	CaLG1	CaSNP1	59.9	scaffold00010	831732
579	CaLG1	CaSNP2647	60.05	scaffold00494	163765
580	CaLG1	CaTSNP7658	60.07	-	-
582	CaLG1	CaSNP446	60.1	9215489	38993
581	CaLG1	CaSNP601	60.1	scaffold00948	101274
583	CaLG1	CaTSNP7637	60.1	-	-
584	CaLG1	CaTSNP8701	60.1	-	-
585	CaLG1	CaTSNP7271	60.17	-	-
586	CaLG1	CaSNP2284	60.24	-	-
587	CaLG1	CaSNP491	60.33	-	-
588	CaLG1	CaTSNP6608	60.33	-	-
589	CaLG1	CaTSNP7515	60.33	-	-
590	CaLG1	CaTSNP8892	60.39	-	-
591	CaLG1	CaTSNP7888	60.46	-	-
592	CaLG1	CaTSNP6927	60.55	-	-
594	CaLG1	CaTSNP7608	60.56	-	-
593	CaLG1	CaTSNP8538	60.56	-	-
595	CaLG1	CaTMS629	60.56	-	-
596	CaLG1	CaSNP536	60.7	9195264	79373
597	CaLG1	CaTSNP7876	60.99	-	-
598	CaLG1	CaSNP796	61.09	scaffold00044	508485
599	CaLG1	CaSNP646	61.23	scaffold00288	202971
600	CaLG1	CaTSNP8286	61.54	-	-
601	CaLG1	CaTSNP7969	61.62	-	-
603	CaLG1	CaTSNP6614	61.64	-	-
602	CaLG1	CaTSNP8807	61.64	-	-
604	CaLG1	CaGMS506	61.65	-	-

605	CaLG1	CaTSNP6597	62.09	-	-
606	CaLG1	CaTSNP7534	62.56	-	-
608	CaLG1	CaTSNP6898	63.07	-	-
607	CaLG1	CaTSNP7115	63.07	-	-
609	CaLG1	CaTSNP8546	63.1	-	-
610	CaLG1	CaTSNP8835	63.11	-	-
612	CaLG1	CaTSNP6703	63.21	-	-
611	CaLG1	CaTSNP8163	63.21	-	-
613	CaLG1	CaTSNP6032	63.23	-	-
614	CaLG1	CaTSNP8268	63.23	-	-
615	CaLG1	CaTSNP6406	63.35	-	-
616	CaLG1	CaTSNP9036	63.51	scaffold00743	127637
617	CaLG1	CaTSNP8369	64.05	-	-
618	CaLG1	CaTSNP8209	64.18	-	-
619	CaLG1	CaTSNP6089	64.18	-	-
620	CaLG1	CaTSNP8842	64.23	-	-
621	CaLG1	CaTSNP8291	64.37	scaffold00642	159021
623	CaLG1	CaTSNP7648	64.76	-	-
622	CaLG1	CaTSNP7878	64.76	-	-
624	CaLG1	CaSNP5042	64.81	scaffold01015	86559
625	CaLG1	CaTSNP6042	64.82	-	-
626	CaLG1	CaTSNP7697	64.85	-	-
627	CaLG1	CaTSNP6043	65.1	-	-
628	CaLG1	CaSNP3072	65.77	scaffold01925	49412
629	CaLG1	CaGMS490	65.87	-	-
630	CaLG1	CaSNP2105	66.23	scaffold00473	177468
632	CaLG1	CaTSNP6171	66.25	-	-
631	CaLG1	CaTSNP7834	66.25	-	-
633	CaLG1	CaSNP4075	66.31	scaffold04744	14000

634	CaLG1	CaSNP3439	66.91	scaffold01744	58555
635	CaLG1	CaTSNP8801	67.26	-	-
636	CaLG1	CaTSNP9058	67.32	-	-
637	CaLG1	CaSNP4196	67.93	scaffold01423	66824
638	CaLG1	CaSNP376	68.47	scaffold00882	110007
639	CaLG1	CaTSNP8352	68.72	-	-
640	CaLG1	CaTSNP8669	68.72	-	-
641	CaLG1	CaTMS687	68.73	-	-
642	CaLG1	CaTSNP8115	68.86	-	-
643	CaLG1	CaSNP4386	68.89	-	-
644	CaLG1	CaSNP3495	69	scaffold01725	61763
645	CaLG1	CaSNP459	69.22	scaffold00101	339983
646	CaLG1	CaSNP490	69.37	scaffold02365	39388
647	CaLG1	CaTMS651	69.44	-	-
648	CaLG1	NCPGR72	69.65	-	-
649	CaLG1	CaTMS864	69.7	-	-
650	CaLG1	CaTSNP8427	69.87	-	-
651	CaLG1	CaTMS868	69.91	-	-
652	CaLG1	CaTSNP6520	69.96	-	-
653	CaLG1	CaTSNP6765	70.09	-	-
654	CaLG1	CaTSNP6348	70.11	-	-
655	CaLG1	CaTSNP6989	70.43	-	-
656	CaLG1	CaSNP405	70.99	-	-
657	CaLG1	CaSNP290	71.52	scaffold02111	55998
658	CaLG1	CaSNP692	71.57	-	-
659	CaLG1	CaSNP93	71.6	-	-
660	CaLG1	CaSNP2127	71.61	scaffold00108	326474
661	CaLG1	CaSNP136	71.62	scaffold00058	425158
662	CaLG1	CaSNP867	71.64	scaffold00176	247024

663	CaLG1	ESNP57	71.64	-	-
664	CaLG1	CaSNP841	71.65	-	-
665	CaLG1	CaTSNP6913	71.7	scaffold01737	55074
667	CaLG1	CaSNP411	71.73	-	-
666	CaLG1	CaSNP888	71.73	scaffold00206	237280
668	CaLG1	CaSNP3037	71.73	-	-
669	CaLG1	CaSNP572	71.73	-	-
670	CaLG1	CaTSNP6054	71.75	9183294	53093
671	CaLG1	CaSNP814	71.75	-	-
672	CaLG1	CaSNP2141	71.77	-	-
673	CaLG1	CaSNP521	71.79	-	-
674	CaLG1	CaSNP2182	71.84	9216120	19923
675	CaLG1	CaTSNP6670	72.06	-	-
676	CaLG1	CaTSNP8866	72.13	-	-
677	CaLG1	CaTSNP8496	72.17	-	-
678	CaLG1	CaTSNP7165	72.18	-	-
679	CaLG1	CaSNP44	72.18	9213651	48209
680	CaLG1	NCPGR65	72.2	-	-
681	CaLG1	CaSNP370	72.24	scaffold00042	458825
682	CaLG1	CaSNP460	72.33	scaffold00461	171341
683	CaLG1	CaTSNP6040	72.34	-	-
684	CaLG1	CaSNP688	72.42	-	-
685	CaLG1	CaSNP65	72.48	-	-
686	CaLG1	CaTSNP8105	72.48	-	-
687	CaLG1	CaTSNP7299	72.65	-	-
688	CaLG1	CaSNP357	72.72	scaffold02681	33820
689	CaLG1	CaTSNP6085	72.74	-	-
690	CaLG1	CaTSNP6065	72.74	-	-
692	CaLG1	CaSNP60	72.76	scaffold04880	13708

691	CaLG1	CaSNP706	72.76	scaffold02029	48661
693	CaLG1	CaSNP116	72.79	scaffold06094	13111
694	CaLG1	CaTSNP7034	72.8	-	-
695	CaLG1	CaTSNP8742	72.8	-	-
696	CaLG1	CaTSNP8929	72.83	-	-
697	CaLG1	CaSNP346	72.85	scaffold00574	158764
698	CaLG1	CaSNP416	72.85	-	-
699	CaLG1	CaTSNP8444	73	-	-
700	CaLG1	CaSNP644	73.01	scaffold00127	309055
701	CaLG1	CaSNP263	73.17	scaffold08854	6444
702	CaLG1	STMS13	73.79	-	-
703	CaLG1	CaTSNP6975	73.97	-	-
704	CaLG1	CaTSNP9023	74.11	-	-
705	CaLG1	CaTSNP6357	74.31	-	-
706	CaLG1	H3A03	74.32	-	-
707	CaLG1	GA11	74.39	-	-
708	CaLG1	CaTSNP8394	74.7	-	-
709	CaLG1	CaTSNP6297	74.71	-	-
710	CaLG1	CaTSNP8655	74.74	-	-
711	CaLG1	CaTSNP8717	74.74	-	-
712	CaLG1	CaTSNP8688	74.83	-	-
713	CaLG1	CaTSNP8651	74.9	-	-
714	CaLG1	CaTSNP7331	74.91	-	-
715	CaLG1	CaTSNP6784	74.95	-	-
716	CaLG1	CaTSNP7169	74.96	-	-
717	CaLG1	CaTSNP7624	74.98	-	-
718	CaLG1	CaTSNP7578	75.12	-	-
719	CaLG1	CaTSNP7275	75.12	scaffold01143	79342
720	CaLG1	CaSNP2653	75.71	scaffold00269	207605

721	CaLG1	CaTSNP6332	76.47	-	-
722	CaLG1	CaTSNP7937	77.06	-	-
724	CaLG1	CaSNP2064	77.07	9192909	46130
723	CaLG1	CaSNP5050	77.07	-	-
725	CaLG1	CaTSNP6704	77.08	-	-
726	CaLG1	CaTSNP8047	77.08	-	-
727	CaLG1	CaTSNP6840	77.11	-	-
728	CaLG1	CaTSNP8171	77.12	-	-
729	CaLG1	CaTSNP8069	77.35	-	-
731	CaLG1	CaTSNP8113	77.35	-	-
730	CaLG1	CaTSNP8295	77.35	-	-
732	CaLG1	CaTSNP7236	77.41	-	-
733	CaLG1	CaTSNP8839	77.85	-	-
734	CaLG1	CaTSNP8588	77.95	-	-
735	CaLG1	CaTSNP8125	78.08	-	-
736	CaLG1	CaTSNP7715	78.26	-	-
737	CaLG1	CaTSNP6371	78.3	-	-
738	CaLG1	CaTSNP8012	78.43	-	-
739	CaLG1	CaTSNP8573	78.46	-	-
740	CaLG1	CaTSNP7054	78.53	-	-
741	CaLG1	CaTSNP6469	78.57	-	-
742	CaLG1	CaSNP2343	78.63	scaffold00028	567099
743	CaLG1	CaTSNP6908	78.63	-	-
744	CaLG1	CaTSNP8029	78.67	scaffold03447	23536
745	CaLG1	CaTSNP7214	78.68	-	-
746	CaLG1	CaTSNP7388	78.68	-	-
747	CaLG1	CaTSNP7413	78.69	-	-
748	CaLG1	CaTSNP6417	78.71	scaffold12870	2989
749	CaLG1	CaTSNP7896	78.71	-	-

751	CaLG1	CaTSNP7867	78.73	-	-
752	CaLG1	CaTSNP8639	78.73	-	-
750	CaLG1	CaTSNP8749	78.73	9206673	29807
753	CaLG1	CaTSNP9110	78.73	-	-
754	CaLG1	CaTSNP6875	78.73	-	-
755	CaLG1	CaTSNP8258	78.75	-	-
756	CaLG1	CaTSNP8980	78.76	-	-
757	CaLG1	CaSNP2364	78.78	scaffold01072	83605
758	CaLG1	CaTSNP9028	78.78	-	-
759	CaLG1	CaTSNP6183	78.81	-	-
760	CaLG1	CaSNP5094	78.82	scaffold01701	55366
761	CaLG1	CaSNP2145	78.84	scaffold00105	327204
762	CaLG1	CaTSNP7727	78.88	-	-
763	CaLG1	CaSNP4929	78.88	9211483	26729
764	CaLG1	CaSNP2427	78.92	scaffold00483	157850
765	CaLG1	CaTSNP7119	78.97	-	-
766	CaLG1	CaTSNP7906	78.99	-	-
767	CaLG1	CaTSNP8162	78.99	-	-
768	CaLG1	CaTSNP8189	79.02	-	-
769	CaLG1	CaSNP3291	79.03	-	-
770	CaLG1	CaTSNP6253	79.07	-	-
771	CaLG1	CaTSNP7805	79.17	-	-
772	CaLG1	CaTSNP8571	79.17	-	-
773	CaLG1	CaTSNP8176	79.36	-	-
774	CaLG1	CaGMS232	83.67	-	-
775	CaLG1	CaTMS884	85.13	-	-
776	CaLG1	TA113	90.04	-	-
777	CaLG1	NCPGR109	90.32	-	-
778	CaLG1	NCPGR136	98.8	scaffold04070	18154

779	CaLG2	PIP140	0	-	-
780	CaLG2	CESSR47	1.494	scaffold00079	413662
781	CaLG2	TS52	4.093	-	-
782	CaLG2	CESSR172	12.22	scaffold00201	233654
783	CaLG2	CaTMS709	14.77	-	-
784	CaLG2	CaGMS1278	15.26	-	-
785	CaLG2	PIP107	15.59	-	-
786	CaLG2	CEST102	20.13	-	-
787	CaLG2	GAA60	22.71	-	-
788	CaLG2	CaGMS1287	23.97	-	-
789	CaLG2	CaTMS1053	26.24	-	-
790	CaLG2	CaTMS590	28.97	-	-
791	CaLG2	CESSR72	29.52	scaffold02021	47550
792	CaLG2	CaTMS541	30.86	-	-
793	CaLG2	NCPGR188	34.35	-	-
794	CaLG2	TR19	36.33	-	-
795	CaLG2	CaTMS716	36.69	-	-
796	CaLG2	CaGMS485	37.77	-	-
797	CaLG2	CaSNP3540	38.7	scaffold01906	62199
798	CaLG2	PIP65	39.1	-	-
799	CaLG2	CaGMS4	44.07	-	-
800	CaLG2	CaTMS704	44.7	-	-
801	CaLG2	CaTSNP6162	46.55	scaffold01827	54392
802	CaLG2	CaTSNP6329	46.59	-	-
803	CaLG2	CaTSNP7375	46.61	scaffold00190	230696
804	CaLG2	CaTSNP6648	46.68	-	-
805	CaLG2	CaTSNP7316	46.76	scaffold00020	631783
806	CaLG2	CaSNP4617	46.83	-	-
807	CaLG2	CaTSNP8331	46.86	-	-

808	CaLG2	CaGMS41	46.9	-	-
809	CaLG2	CaTSNP7964	46.94	scaffold00358	193401
810	CaLG2	CaTSNP7683	47.08	-	-
811	CaLG2	CaTSNP8898	47.68	-	-
812	CaLG2	CaSNP2540	47.7	-	-
813	CaLG2	CaTSNP7347	47.89	-	-
814	CaLG2	CaTSNP7000	47.9	-	-
815	CaLG2	CaTMS712	48.28	-	-
816	CaLG2	CaSNP2451	48.47	-	-
817	CaLG2	CaTSNP6629	48.55	-	-
818	CaLG2	CaSNP2781	48.62	-	-
819	CaLG2	CaTSNP8013	48.73	-	-
820	CaLG2	CaSNP2334	48.76	-	-
821	CaLG2	CaTSNP7687	49.03	-	-
822	CaLG2	CaTSNP6791	49.92	-	-
823	CaLG2	CaTMS750	50.36	-	-
824	CaLG2	CaTSNP7156	50.39	-	-
825	CaLG2	CaTSNP8786	50.4	-	-
826	CaLG2	CaTSNP7885	50.42	-	-
827	CaLG2	CaTSNP6227	50.5	-	-
828	CaLG2	GA6	50.54	-	-
829	CaLG2	GA16	50.83	-	-
830	CaLG2	CaTSNP8865	51.02	-	-
831	CaLG2	CaTMS529	51.15	-	-
832	CaLG2	CaSNP2025	51.21	-	-
833	CaLG2	CaSNP5112	51.3	-	-
834	CaLG2	CaTSNP8188	51.34	scaffold00569	131431
835	CaLG2	CaTSNP7371	51.4	scaffold09348	4841
836	CaLG2	CaTSNP8477	51.41	-	-

837	CaLG2	CaTSNP8454	51.42	-	-
838	CaLG2	CaTSNP6664	51.43	-	-
839	CaLG2	CaTSNP6546	51.78	-	-
840	CaLG2	CaTSNP6517	51.81	9211514	67152
841	CaLG2	CaSNP419	52.19	-	-
842	CaLG2	CaSNP873	52.31	-	-
843	CaLG2	CaSNP781	52.37	-	-
844	CaLG2	NCPGR144	52.51	-	-
845	CaLG2	CaTSNP7439	52.59	-	-
846	CaLG2	CaSNP470	52.63	scaffold01561	72078
847	CaLG2	NCPGR194	52.7	-	-
848	CaLG2	ESNP61	53.48	9160172	17105
849	CaLG2	ESNP39	53.56	-	-
850	CaLG2	CaSNP254	53.73	-	-
851	CaLG2	CaTMS548	54.06	-	-
852	CaLG2	CaTSNP7795	54.14	-	-
853	CaLG2	CaTSNP8609	54.22	-	-
854	CaLG2	CaSNP344	54.24	-	-
855	CaLG2	CaTSNP7098	54.35	-	-
856	CaLG2	CaTSNP6624	54.51	-	-
857	CaLG2	CaTSNP6636	54.51	-	-
858	CaLG2	CaTSNP7963	54.51	-	-
859	CaLG2	CaTSNP8952	54.51	-	-
860	CaLG2	CaTSNP6862	54.54	-	-
861	CaLG2	CaTSNP7499	54.65	scaffold03386	26714
862	CaLG2	CaTSNP7814	54.65	-	-
863	CaLG2	CaTSNP8825	54.65	scaffold06838	7702
864	CaLG2	CaTSNP8850	54.67	-	-
865	CaLG2	CaTSNP6662	54.85	9091072	33977

866	CaLG2	CaSNP146	55.16	scaffold02466	41024
867	CaLG2	CaTSNP9034	55.21	scaffold00380	185313
868	CaLG2	CaSNP4419	55.21	-	-
869	CaLG2	CaSNP3891	55.22	scaffold05309	11579
870	CaLG2	CaSNP2687	55.36	-	-
871	CaLG2	CaSNP2698	55.39	scaffold00344	195976
872	CaLG2	CaSNP3067	55.39	9208404	23522
873	CaLG2	CaSNP5109	55.39	scaffold00877	163450
874	CaLG2	CaSNP4788	55.42	scaffold02162	46365
875	CaLG2	CaSNP2954	55.43	scaffold01555	73618
876	CaLG2	CaSNP4333	55.55	scaffold04333	27019
877	CaLG2	CaSNP3367	55.57	scaffold00742	128830
878	CaLG2	CaSNP4042	55.57	-	-
879	CaLG2	CaSNP4380	55.61	scaffold01431	76712
880	CaLG2	CaSNP4491	55.61	scaffold01826	52310
881	CaLG2	CaSNP5096	55.63	-	-
882	CaLG2	CaSNP4456	55.65	scaffold02698	33610
883	CaLG2	CaSNP2495	55.66	scaffold00295	212852
884	CaLG2	CaSNP3047	55.66	scaffold01688	83777
885	CaLG2	CaSNP4712	55.72	9207296	8740
886	CaLG2	CaTSNP7826	55.98	-	-
887	CaLG2	CaSNP466	56.05	-	-
888	CaLG2	CaTSNP6415	56.24	scaffold02429	50037
889	CaLG2	CaTSNP7998	56.35	scaffold00601	143281
890	CaLG2	CaTSNP8857	56.35	-	-
891	CaLG2	CaTSNP7609	56.42	scaffold02301	65565
892	CaLG2	CaTSNP6789	56.46	scaffold00990	91157
893	CaLG2	CaTSNP6362	56.56	scaffold01506	64261
894	CaLG2	CaTSNP6482	56.57	-	-

895	CaLG2	CaTSNP6759	56.57	-	-
896	CaLG2	CaTSNP6547	56.57	scaffold00083	405373
897	CaLG2	CaTSNP7873	56.57	-	-
898	CaLG2	CaSNP595	56.64	-	-
899	CaLG2	CaSNP16	56.67	-	-
900	CaLG2	CaSNP177	56.67	-	-
901	CaLG2	CaSNP4951	56.68	-	-
902	CaLG2	CaSNP4274	56.8	-	-
903	CaLG2	CaSNP2120	56.83	scaffold00084	372744
904	CaLG2	CaSNP2235	56.83	-	-
905	CaLG2	CaSNP4592	56.84	-	-
906	CaLG2	CaSNP2868	56.86	scaffold00929	105004
907	CaLG2	CaSNP3964	56.91	scaffold02603	50891
908	CaLG2	CaSNP2136	57.07	9204064	37946
909	CaLG2	CaTSNP6742	57.08	scaffold01390	77364
910	CaLG2	CaTSNP8507	57.08	-	-
911	CaLG2	CaSNP2764	57.08	-	-
912	CaLG2	CaSNP3420	57.08	-	-
913	CaLG2	CaSNP2211	57.08	-	-
914	CaLG2	CaSNP2655	57.08	scaffold01524	76861
915	CaLG2	CaSNP2685	57.08	scaffold00772	118821
916	CaLG2	CaSNP4275	57.08	scaffold02631	37277
917	CaLG2	CaSNP4428	57.08	scaffold01814	76422
918	CaLG2	CaSNP4738	57.08	scaffold00864	103529
919	CaLG2	CaSNP5085	57.08	scaffold02465	37348
920	CaLG2	CaSNP2849	57.09	scaffold01898	50287
921	CaLG2	CaSNP4010	57.09	9214878	18604
922	CaLG2	CaSNP5002	57.09	scaffold01753	75008
923	CaLG2	CaTSNP6285	57.27	-	-

924	CaLG2	CaTSNP8353	57.28	scaffold01520	89834
925	CaLG2	CaTSNP7315	57.3	scaffold00281	209277
926	CaLG2	CaTSNP8091	57.3	9168576	41973
927	CaLG2	CaTSNP7778	57.3	scaffold11668	3468
928	CaLG2	CaTSNP8455	57.32	-	-
929	CaLG2	CaTSNP6369	57.34	scaffold00937	124829
930	CaLG2	CaTSNP6356	57.36	scaffold00900	110653
931	CaLG2	CaTSNP6839	57.36	scaffold03558	27965
932	CaLG2	CaTSNP7339	57.36	scaffold04460	15562
933	CaLG2	CaTSNP6127	57.44	scaffold00653	133710
934	CaLG2	CaTSNP8405	57.44	scaffold00902	114212
935	CaLG2	CaTSNP8534	57.44	-	-
936	CaLG2	CaTSNP7791	57.45	scaffold01414	77535
937	CaLG2	CaTSNP8354	57.46	-	-
938	CaLG2	CaTSNP6933	57.51	scaffold00829	106051
939	CaLG2	CaTSNP8131	57.51	scaffold01421	87927
940	CaLG2	CaTSNP6494	57.55	scaffold01370	90389
941	CaLG2	CaTSNP8420	57.55	scaffold01775	58796
942	CaLG2	CaTSNP7626	57.57	-	-
943	CaLG2	CaTSNP9132	57.57	scaffold01845	55752
944	CaLG2	CaSNP765	57.94	-	-
945	CaLG2	CaSNP3500	58.02	-	-
946	CaLG2	CaSNP2386	58.04	-	-
947	CaLG2	CaSNP3022	58.04	-	-
948	CaLG2	CaSNP3027	58.04	-	-
949	CaLG2	CaSNP3049	58.04	scaffold02651	57307
950	CaLG2	CaSNP3651	58.04	scaffold01054	108530
951	CaLG2	CaSNP2782	58.05	-	-
952	CaLG2	CaSNP3205	58.06	scaffold00942	123512

953	CaLG2	CaSNP4126	58.06	-	-
954	CaLG2	CaSNP2579	58.08	-	-
955	CaLG2	CaSNP2865	58.11	scaffold01109	94013
956	CaLG2	CaSNP2029	58.13	scaffold01410	67080
957	CaLG2	CaSNP4268	58.19	scaffold01010	110578
958	CaLG2	CaGMS22	58.21	-	-
959	CaLG2	CaSNP2903	58.23	-	-
960	CaLG2	CaSNP3889	58.24	scaffold05217	11937
961	CaLG2	CaSNP2819	58.25	scaffold02364	40302
962	CaLG2	CaSNP3511	58.25	scaffold06459	8466
963	CaLG2	CaSNP3669	58.27	scaffold01396	74826
964	CaLG2	CaTSNP6942	58.28	scaffold11575	3503
965	CaLG2	CaSNP3127	58.28	scaffold01080	90871
966	CaLG2	CaSNP3158	58.28	scaffold01762	55575
967	CaLG2	CaSNP3778	58.28	-	-
968	CaLG2	CaSNP4908	58.28	-	-
969	CaLG2	CaSNP1965	58.32	-	-
970	CaLG2	CaSNP4729	58.33	9202205	6408
971	CaLG2	CaSNP4621	58.4	scaffold02685	44519
972	CaLG2	CaTSNP7265	58.41	9214224	27810
973	CaLG2	CaSNP4321	58.47	scaffold01501	71369
974	CaLG2	CaSNP3639	58.48	scaffold02147	46180
975	CaLG2	CaSNP2796	58.49	-	-
976	CaLG2	CaSNP832	58.49	scaffold00389	190458
977	CaLG2	CaSNP2812	58.49	-	-
978	CaLG2	CaSNP2975	58.49	scaffold01488	70489
979	CaLG2	CaSNP4337	58.52	scaffold03513	27552
980	CaLG2	CaSNP1962	58.52	-	-
981	CaLG2	CaTSNP8210	58.53	-	-

982	CaLG2	CaSNP3782	58.55	-	-
983	CaLG2	CaSNP2593	58.56	scaffold01273	73147
984	CaLG2	CaSNP2861	58.56	scaffold00926	99393
985	CaLG2	CaSNP2154	58.57	scaffold00810	123788
986	CaLG2	CaSNP4361	58.58	scaffold00732	131847
987	CaLG2	CaSNP4313	58.58	scaffold02772	35352
988	CaLG2	CaSNP4956	58.58	scaffold01990	58388
989	CaLG2	CaSNP3214	58.59	scaffold02051	50820
990	CaLG2	CaSNP4020	58.6	scaffold00651	136422
991	CaLG2	CaSNP2373	58.6	scaffold08318	5809
992	CaLG2	CaSNP4976	58.6	scaffold00613	166579
993	CaLG2	CaTSNP6518	58.6	scaffold00381	188287
994	CaLG2	CaSNP2985	58.6	9207929	10725
995	CaLG2	CaSNP3837	58.6	scaffold03113	37295
996	CaLG2	CaSNP4497	58.6	scaffold01936	70050
997	CaLG2	CaSNP2224	58.6	scaffold00386	187167
998	CaLG2	CaSNP2452	58.61	scaffold00974	89938
999	CaLG2	CaSNP3410	58.61	scaffold02087	47463
1000	CaLG2	CaSNP4163	58.61	-	-
1001	CaLG2	CaSNP2854	58.61	scaffold01610	91977
1002	CaLG2	CaSNP2886	58.61	9203293	8077
1003	CaLG2	CaSNP3128	58.61	-	-
1004	CaLG2	CaSNP3388	58.61	scaffold01272	81011
1005	CaLG2	CaSNP3450	58.61	9208727	25395
1006	CaLG2	CaSNP3489	58.61	scaffold01495	79032
1007	CaLG2	CaSNP3733	58.61	scaffold00722	148194
1008	CaLG2	CaSNP3734	58.61	scaffold03016	30887
1009	CaLG2	CaSNP3749	58.61	scaffold01211	83533
1010	CaLG2	CaSNP3820	58.61	scaffold02026	55900

1011	CaLG2	CaSNP3829	58.61	scaffold03457	28279
1012	CaLG2	CaSNP3845	58.61	scaffold02309	47920
1013	CaLG2	CaSNP3966	58.61	scaffold02036	57564
1014	CaLG2	CaSNP3998	58.61	scaffold01954	59074
1015	CaLG2	CaSNP4233	58.61	scaffold07383	6868
1016	CaLG2	CaSNP4949	58.61	scaffold02985	35827
1017	CaLG2	CaSNP5025	58.61	scaffold01602	88678
1018	CaLG2	CaSNP5140	58.61	scaffold01793	72217
1019	CaLG2	CaSNP4524	58.61	-	-
1020	CaLG2	CaSNP4448	58.61	-	-
1021	CaLG2	CaSNP4463	58.61	scaffold00833	122404
1022	CaLG2	CaSNP3220	58.61	9210230	29149
1023	CaLG2	CaSNP4195	58.61	9150399	17674
1024	CaLG2	CaSNP4088	58.62	scaffold03133	35271
1025	CaLG2	CaSNP2696	58.62	scaffold01009	100768
1026	CaLG2	CaSNP3343	58.62	scaffold01686	68622
1027	CaLG2	CaSNP3554	58.62	scaffold02661	46960
1028	CaLG2	CaSNP4309	58.62	scaffold02149	48373
1029	CaLG2	CaSNP4678	58.62	scaffold01988	47640
1030	CaLG2	CaSNP5108	58.62	scaffold02633	57316
1031	CaLG2	CaSNP3914	58.62	scaffold02514	37864
1032	CaLG2	CaSNP3082	58.62	scaffold00996	96114
1033	CaLG2	CaSNP3242	58.62	scaffold02334	39477
1034	CaLG2	CaSNP3327	58.63	scaffold02599	51206
1035	CaLG2	CaSNP4611	58.63	scaffold03671	30091
1036	CaLG2	CaSNP3175	58.64	scaffold01145	79602
1037	CaLG2	CaSNP3681	58.64	-	-
1038	CaLG2	CaSNP4490	58.64	-	-
1039	CaLG2	CaSNP2069	58.64	scaffold01665	71903

1040	CaLG2	CaSNP2243	58.64	scaffold05174	16461
1041	CaLG2	CaSNP2302	58.64	scaffold01092	94150
1042	CaLG2	CaSNP2578	58.64	scaffold00418	198057
1043	CaLG2	CaSNP2649	58.64	scaffold00638	132413
1044	CaLG2	CaSNP4119	58.64	scaffold03315	34169
1045	CaLG2	CaSNP4158	58.64	scaffold01182	97920
1046	CaLG2	CaSNP4240	58.64	scaffold03450	34372
1047	CaLG2	CaSNP4775	58.65	scaffold02724	43366
1048	CaLG2	CaSNP2349	58.65	scaffold01930	57039
1049	CaLG2	CaSNP2377	58.65	scaffold04317	16519
1050	CaLG2	CaSNP2415	58.65	scaffold00887	99818
1051	CaLG2	CaSNP2420	58.65	scaffold00972	112265
1052	CaLG2	CaSNP2581	58.65	-	-
1053	CaLG2	CaSNP2836	58.65	scaffold00689	123392
1054	CaLG2	CaSNP2866	58.65	scaffold02060	45309
1055	CaLG2	CaSNP2885	58.65	scaffold04005	18919
1056	CaLG2	CaSNP2939	58.65	scaffold02463	52796
1057	CaLG2	CaSNP3006	58.65	scaffold01712	63646
1058	CaLG2	CaSNP3094	58.65	-	-
1059	CaLG2	CaSNP3169	58.65	scaffold03069	42156
1060	CaLG2	CaSNP3354	58.65	scaffold00821	135193
1061	CaLG2	CaSNP3422	58.65	-	-
1062	CaLG2	CaSNP3686	58.65	scaffold01811	64843
1063	CaLG2	CaSNP3748	58.65	scaffold02214	48587
1064	CaLG2	CaSNP3816	58.65	scaffold02452	57499
1065	CaLG2	CaSNP3866	58.65	scaffold02276	54842
1066	CaLG2	CaSNP3913	58.65	scaffold02174	70968
1067	CaLG2	CaSNP3974	58.65	scaffold02125	48811
1068	CaLG2	CaSNP4145	58.65	scaffold01699	58892

1069	CaLG2	CaSNP4436	58.65	scaffold03894	26378
1070	CaLG2	CaSNP4650	58.65	scaffold01784	62708
1071	CaLG2	CaSNP4675	58.65	scaffold06573	8198
1072	CaLG2	CaSNP4716	58.65	scaffold01934	62098
1073	CaLG2	CaSNP4853	58.65	scaffold02953	35824
1074	CaLG2	CaSNP4875	58.65	scaffold04121	24989
1075	CaLG2	CaSNP3235	58.66	scaffold04042	22455
1076	CaLG2	CaSNP3322	58.66	scaffold01810	61662
1077	CaLG2	CaSNP3922	58.66	scaffold00250	240966
1078	CaLG2	CaSNP4884	58.66	-	-
1079	CaLG2	CaSNP4049	58.67	-	-
1080	CaLG2	CaSNP4369	58.67	scaffold01170	111810
1081	CaLG2	CaSNP5044	58.67	scaffold00464	164109
1082	CaLG2	CaSNP5101	58.67	scaffold05386	16812
1083	CaLG2	CaSNP2872	58.68	scaffold00327	218716
1084	CaLG2	CaSNP4726	58.69	9214491	11648
1085	CaLG2	CaSNP3735	58.69	scaffold00724	138407
1086	CaLG2	CaSNP4687	58.69	scaffold02168	54588
1087	CaLG2	CaSNP3840	58.7	scaffold01960	83839
1088	CaLG2	CaSNP3145	58.71	scaffold00845	121098
1089	CaLG2	CaTSNP8654	58.76	9213328	38531
1090	CaLG2	CaTSNP8565	58.77	scaffold00679	122739
1091	CaLG2	CaTSNP7304	58.82	-	-
1092	CaLG2	CaTSNP6734	58.83	-	-
1093	CaLG2	CaTSNP8370	58.85	-	-
1094	CaLG2	CaTSNP6427	58.88	-	-
1095	CaLG2	CaTSNP8888	58.89	scaffold00378	177803
1096	CaLG2	CaTSNP8705	58.91	9210152	46523
1097	CaLG2	CaSNP4183	58.93	-	-

1098	CaLG2	CaSNP4403	58.97	scaffold07524	6665
1099	CaLG2	CaTSNP7574	59.01	-	-
1100	CaLG2	CaTSNP6830	59.01	9204284	10242
1101	CaLG2	CaTSNP7230	59.01	scaffold00147	270449
1102	CaLG2	CaTSNP8972	59.01	-	-
1103	CaLG2	CaSNP5120	59.03	-	-
1104	CaLG2	CaSNP3680	59.21	scaffold02741	33923
1105	CaLG2	CaSNP2307	59.21	scaffold00797	118648
1106	CaLG2	CaSNP2570	59.21	scaffold01303	74281
1107	CaLG2	CaSNP2605	59.21	scaffold08384	5717
1108	CaLG2	CaSNP4406	59.21	-	-
1109	CaLG2	CaSNP4477	59.21	scaffold02539	37686
1110	CaLG2	CaSNP3520	59.22	-	-
1111	CaLG2	CaSNP2770	59.35	-	-
1112	CaLG2	CaTSNP7557	59.42	-	-
1113	CaLG2	CaTSNP8021	60.63	scaffold02009	52200
1114	CaLG2	CaTSNP6904	60.75	scaffold00967	91303
1115	CaLG2	CaTSNP7407	60.9	scaffold00065	424690
1116	CaLG2	CaTSNP6865	61.8	9205238	38231
1117	CaLG2	CaTSNP6809	61.84	-	-
1118	CaLG2	CaTSNP7204	61.84	-	-
1119	CaLG2	CaTSNP8020	61.84	9188703	40817
1120	CaLG2	CaTSNP7991	61.89	-	-
1121	CaLG2	CaTSNP8812	61.89	-	-
1122	CaLG2	CaSNP2733	62.25	-	-
1123	CaLG2	CaSNP2491	62.62	-	-
1124	CaLG2	CaSNP3627	62.62	scaffold01625	68619
1125	CaLG2	CaSNP2014	62.63	9211308	26366
1126	CaLG2	CaSNP2658	62.65	scaffold01071	92185

1127	CaLG2	CaSNP3785	62.78	9212018	33346
1128	CaLG2	CaSNP4591	63.12	scaffold03807	25440
1129	CaLG2	CaSNP2005	63.2	-	-
1130	CaLG2	CaTSNP6852	63.28	-	-
1131	CaLG2	CaTSNP7988	63.32	-	-
1132	CaLG2	CaTSNP6471	63.32	scaffold00523	155590
1133	CaLG2	CaTSNP6996	63.33	-	-
1134	CaLG2	CaTSNP8649	63.35	-	-
1135	CaLG2	CaSNP2475	63.36	scaffold01245	76093
1136	CaLG2	CaTSNP7385	63.38	-	-
1137	CaLG2	CaTSNP7403	63.51	-	-
1138	CaLG2	CaTSNP6429	63.59	9136476	31082
1139	CaLG2	CaSNP3406	63.7	scaffold02902	32344
1140	CaLG2	CaSNP3545	63.77	scaffold01963	58479
1141	CaLG2	CaSNP3617	63.77	-	-
1142	CaLG2	CaSNP4122	63.78	9216043	25058
1143	CaLG2	CaSNP2180	63.8	-	-
1144	CaLG2	CaSNP4661	63.8	scaffold01716	86754
1145	CaLG2	CaSNP4749	63.8	scaffold00715	130584
1146	CaLG2	CaSNP5093	63.8	-	-
1147	CaLG2	CaTSNP7324	63.88	scaffold00248	220230
1148	CaLG2	CaSNP94	63.91	scaffold02326	39542
1149	CaLG2	CaSNP429	63.93	scaffold00870	107691
1150	CaLG2	CaTSNP8452	63.97	-	-
1151	CaLG2	CaTSNP7538	63.98	9202122	32194
1152	CaLG2	CaSNP464	63.99	scaffold01078	103319
1153	CaLG2	CaTSNP8339	64	-	-
1154	CaLG2	CaTSNP7146	64.01	-	-
1155	CaLG2	CaSNP4803	64.03	scaffold03086	34574

1156	CaLG2	CaTSNP6761	64.06	9174575	22591
1157	CaLG2	CaSNP3825	64.12	scaffold03714	30792
1158	CaLG2	CaSNP108	64.18	scaffold01267	75927
1159	CaLG2	CaSNP4869	64.18	scaffold01526	77602
1160	CaLG2	CaSNP760	64.2	9200809	30890
1161	CaLG2	CaSNP4512	64.21	scaffold03521	24646
1162	CaLG2	CaSNP4860	64.21	scaffold00872	104613
1163	CaLG2	CaSNP5086	64.22	scaffold01241	76570
1164	CaLG2	CaSNP5016	64.22	scaffold03135	34668
1165	CaLG2	CaSNP2289	64.24	scaffold00151	287069
1166	CaLG2	CaTSNP6103	64.26	-	-
1167	CaLG2	CaTSNP6982	64.26	-	-
1168	CaLG2	CaTSNP6269	64.29	scaffold01118	84319
1169	CaLG2	CaTSNP9080	64.29	-	-
1170	CaLG2	CaTSNP7045	64.3	-	-
1171	CaLG2	CaTSNP8088	64.32	-	-
1172	CaLG2	CaTSNP7361	64.33	-	-
1173	CaLG2	CaTSNP8175	64.35	9215035	5864
1174	CaLG2	CaSNP2679	64.38	-	-
1175	CaLG2	CaSNP2810	64.39	-	-
1176	CaLG2	CaTSNP7757	64.42	-	-
1177	CaLG2	CaTSNP6394	64.46	-	-
1178	CaLG2	CaSNP3462	64.56	-	-
1179	CaLG2	CaTSNP9099	64.56	-	-
1180	CaLG2	CaSNP3278	64.66	-	-
1181	CaLG2	CaTSNP6833	64.66	-	-
1182	CaLG2	CaGMS1125	64.66	-	-
1183	CaLG2	CaTSNP6092	64.68	-	-
1184	CaLG2	CaTSNP7497	64.68	-	-

1185	CaLG2	CaTSNP7458	64.71	9211629	23453
1186	CaLG2	CaTSNP8855	64.74	scaffold00696	116706
1187	CaLG2	CaSNP2308	64.75	-	-
1188	CaLG2	CaSNP3243	64.76	-	-
1189	CaLG2	CaSNP3986	64.78	-	-
1190	CaLG2	CaSNP2012	64.82	-	-
1191	CaLG2	CaSNP496	64.83	scaffold03143	52730
1192	CaLG2	CaSNP2169	64.83	-	-
1193	CaLG2	CaSNP2905	64.86	-	-
1194	CaLG2	CaSNP4649	64.91	-	-
1195	CaLG2	CaTSNP8516	64.92	-	-
1196	CaLG2	CaSNP3658	64.93	scaffold00917	122398
1197	CaLG2	CaTSNP6188	64.94	scaffold01491	65451
1198	CaLG2	CaSNP2510	64.97	scaffold04771	13869
1199	CaLG2	CaSNP2673	65.08	-	-
1200	CaLG2	CaTSNP8015	65.08	scaffold00503	155389
1201	CaLG2	CaTSNP6316	65.1	scaffold02048	45584
1202	CaLG2	CaTSNP7601	65.11	scaffold00801	107945
1203	CaLG2	CaSNP3756	65.11	-	-
1204	CaLG2	CaTSNP6271	65.12	-	-
1205	CaLG2	CaTSNP6714	65.37	scaffold03206	26978
1206	CaLG2	CaTSNP8782	65.38	-	-
1207	CaLG2	CaSNP3589	65.64	9209697	17729
1208	CaLG2	CaSNP2340	65.79	scaffold01207	77775
1209	CaLG2	CaSNP2902	65.82	scaffold01992	55458
1210	CaLG2	CaSNP666	65.9	scaffold00849	99257
1211	CaLG2	CaSNP695	66.03	scaffold01736	69974
1212	CaLG2	CaSNP2577	66.1	scaffold00915	97777
1213	CaLG2	CaTMS871	66.37	-	-

1214	CaLG2	CaSNP804	66.88	scaffold00856	110050
1215	CaLG2	CaGMS26	67.09	-	-
1216	CaLG2	CaSNP889	67.29	scaffold00573	133805
1217	CaLG2	CaSNP3736	67.73	scaffold05121	12312
1218	CaLG2	CaSNP3842	67.74	scaffold02590	50502
1219	CaLG2	CaTSNP7905	67.74	scaffold03283	25663
1220	CaLG2	CaTSNP6687	67.82	scaffold02061	45326
1221	CaLG2	H2B061	67.83	-	-
1222	CaLG2	TA59	68.17	-	-
1223	CaLG2	TA27	68.42	-	-
1224	CaLG2	NCPGR193	68.48	scaffold04071	27490
1225	CaLG2	CaSNP3020	68.55	scaffold00958	98449
1226	CaLG2	TA96	68.59	-	-
1227	CaLG2	CaSNP87	68.77	scaffold00119	316585
1228	CaLG2	CaSNP895	68.77	9213592	24260
1229	CaLG2	CaSNP4535	68.81	scaffold00203	232623
1230	CaLG2	CaSNP648	68.84	scaffold01263	93576
1231	CaLG2	CaSNP3973	69.01	scaffold02843	38950
1232	CaLG2	CaSNP237	69.09	scaffold00264	211873
1233	CaLG2	CaSNP347	69.13	scaffold00072	419544
1234	CaLG2	CaSNP2901	69.27	scaffold01293	74743
1235	CaLG2	CaSNP3944	69.27	scaffold00843	113304
1236	CaLG2	CaTSNP7690	69.28	-	-
1237	CaLG2	CaTSNP8994	69.28	-	-
1238	CaLG2	CaSNP3661	69.32	-	-
1239	CaLG2	TS82	69.56	-	-
1240	CaLG2	TA37	69.86	-	-
1241	CaLG2	TAA60	70.15	-	-
1242	CaLG2	CaSNP4537	70.8	scaffold02987	29314

1243	CaLG2	TR58	70.81	-	-
1244	CaLG2	Foc4	70.93	-	-
1245	CaLG2	CaSNP584	70.93	-	-
1246	CaLG2	CaTSNP7704	70.94	scaffold00129	306331
1247	CaLG2	Foc5	71.02	-	-
1248	CaLG2	CaSNP2982	71.12	scaffold01839	55246
1249	CaLG2	CaTSNP9045	71.13	-	-
1250	CaLG2	CaTSNP7077	71.16	-	-
1251	CaLG2	CaTSNP8524	71.16	-	-
1252	CaLG2	CaSNP110	71.16	-	-
1253	CaLG2	ESNP52	71.16	-	-
1254	CaLG2	CaSNP2791	71.19	-	-
1255	CaLG2	CaTSNP9102	71.2	-	-
1256	CaLG2	CaTSNP8179	71.2	-	-
1257	CaLG2	ESNP3	71.25	-	-
1258	CaLG2	CaTSNP8579	71.28	scaffold01084	98083
1259	CaLG2	ESNP49	71.31	-	-
1260	CaLG2	CaSNP323	72.05	-	-
1261	CaLG2	CaSNP4277	72.07	scaffold02547	44016
1262	CaLG2	CaSNP4171	72.1	-	-
1263	CaLG2	CaTSNP6978	72.78	scaffold00155	267491
1264	CaLG2	CaTSNP8111	72.78	-	-
1265	CaLG2	CaTSNP7325	72.81	-	-
1266	CaLG2	CaSNP3363	72.86	scaffold00932	112565
1267	CaLG2	CaSNP2378	72.89	scaffold01095	83860
1268	CaLG2	CaTSNP7501	72.91	-	-
1269	CaLG2	CaTSNP8931	72.91	-	-
1270	CaLG2	CaSNP884	72.93	-	-
1271	CaLG2	CaSNP2228	72.93	scaffold00158	297206

1272	CaLG2	CaTSNP7706	72.94	scaffold00858	111867
1273	CaLG2	CaSNP434	73.03	-	-
1274	CaLG2	CaSNP2193	73.08	-	-
1275	CaLG2	CaTSNP7765	73.19	scaffold01409	81178
1276	CaLG2	CaSNP244	73.81	-	-
1277	CaLG2	CaTSNP7010	74.08	-	-
1278	CaLG2	CaTSNP6519	74.1	-	-
1279	CaLG2	CaSNP615	74.15	scaffold00468	157100
1280	CaLG2	CaSNP103	74.15	-	-
1281	CaLG2	CaSNP4589	74.19	scaffold02749	34179
1282	CaLG2	CESSR73	74.38	scaffold03638	32912
1283	CaLG2	CaTSNP8041	74.46	-	-
1284	CaLG2	CaSNP5040	74.46	scaffold00415	173483
1285	CaLG2	CaTSNP8126	74.48	-	-
1286	CaLG2	CaTSNP8474	74.52	-	-
1287	CaLG2	CaTSNP7936	74.54	-	-
1288	CaLG2	CaTSNP9145	74.54	-	-
1289	CaLG2	CaSNP650	74.67	-	-
1290	CaLG2	CaTMS759	74.67	-	-
1291	CaLG2	ESNP66	74.74	-	-
1292	CaLG2	CaSNP352	74.83	-	-
1293	CaLG2	CaSNP204	74.87	-	-
1294	CaLG2	CaSNP2711	74.95	9210014	28141
1295	CaLG2	CaSNP223	75.07	-	-
1296	CaLG2	CaSNP2780	75.27	scaffold00532	145998
1297	CaLG2	CaSNP2664	75.36	-	-
1298	CaLG2	CaSNP142	75.43	-	-
1299	CaLG2	CaSNP455	75.5	scaffold00986	94331
1300	CaLG2	CaSNP202	75.52	-	-

1301	CaLG2	CaSNP2063	75.75	-	-
1302	CaLG2	CaTSNP8580	75.78	-	-
1303	CaLG2	CaTSNP6866	76.06	-	-
1304	CaLG2	CaTSNP8065	76.2	scaffold00061	425424
1305	CaLG2	CaSNP3412	76.44	scaffold04430	25049
1306	CaLG2	CaTSNP8411	76.53	scaffold06530	8317
1307	CaLG2	CaTSNP8152	76.58	scaffold00944	115853
1308	CaLG2	CaTSNP6811	76.69	scaffold07890	6255
1309	CaLG2	CaTSNP8642	76.7	scaffold01198	84721
1310	CaLG2	CaTSNP8087	76.74	scaffold06004	9594
1311	CaLG2	CaTSNP6543	76.75	scaffold02774	32429
1312	CaLG2	CaTSNP7917	76.9	scaffold00431	180307
1313	CaLG2	CaTSNP6657	76.92	-	-
1314	CaLG2	CaTSNP8434	77.16	scaffold00518	158206
1315	CaLG2	CaSNP2515	77.19	scaffold00441	164704
1316	CaLG2	CaSNP2869	77.22	-	-
1317	CaLG2	CaSNP5127	77.22	scaffold01807	53090
1318	CaLG2	CaTSNP6818	77.32	-	-
1319	CaLG2	CaTSNP6700	77.32	9199862	42200
1320	CaLG2	CaSNP2707	77.43	-	-
1321	CaLG2	CaTSNP6147	77.45	9213384	43739
1322	CaLG2	CaSNP2313	77.53	-	-
1323	CaLG2	CaSNP2821	77.53	scaffold01358	78833
1324	CaLG2	CaSNP4420	77.53	scaffold03176	36117
1325	CaLG2	CaTSNP9106	77.7	-	-
1326	CaLG2	CaTSNP8007	77.7	-	-
1327	CaLG2	CaTSNP8849	77.71	scaffold00301	198945
1328	CaLG2	CaTSNP8730	77.72	-	-
1329	CaLG2	CaTSNP7047	77.72	-	-

1330	CaLG2	CaTSNP7009	77.73	-	-
1331	CaLG2	CaTSNP8533	77.76	-	-
1332	CaLG2	CaTSNP7986	77.77	scaffold00150	296668
1333	CaLG2	CaTSNP8560	77.77	-	-
1334	CaLG2	CaTSNP8378	77.83	-	-
1335	CaLG2	CaSNP4859	77.93	scaffold01998	74546
1336	CaLG2	CaSNP2706	77.95	scaffold01552	65628
1337	CaLG2	CaTSNP7478	77.97	-	-
1338	CaLG2	CaSNP4296	77.98	scaffold02982	41850
1339	CaLG2	CaSNP2275	78.03	-	-
1340	CaLG2	CaSNP4904	78.09	scaffold01576	63829
1341	CaLG2	CaSNP4031	78.19	scaffold00214	255375
1342	CaLG2	CaTSNP7196	78.2	-	-
1343	CaLG2	CaTSNP9020	78.2	-	-
1344	CaLG2	CaSNP2225	78.22	-	-
1345	CaLG2	CaSNP1970	78.27	-	-
1346	CaLG2	CaTSNP7743	78.53	scaffold00370	187853
1347	CaLG2	CaTSNP8687	78.53	-	-
1348	CaLG2	CaSNP5036	78.63	-	-
1349	CaLG2	CaTSNP8838	78.7	-	-
1350	CaLG2	CaTMS566	78.8	-	-
1351	CaLG2	CaSNP3228	78.95	scaffold00403	178425
1352	CaLG2	CaSNP2098	78.97	-	-
1353	CaLG2	CaTSNP8317	78.99	-	-
1354	CaLG2	CaGMS43	79.02	-	-
1355	CaLG2	CaSNP3523	79.03	scaffold01046	98500
1356	CaLG2	CaTSNP6388	79.05	-	-
1357	CaLG2	CaTSNP6381	79.14	-	-
1358	CaLG2	CaTSNP9091	79.21	-	-

1359	CaLG2	CaSNP1989	79.26	-	-
1360	CaLG2	CaTSNP8377	79.37	scaffold00326	196988
1361	CaLG2	CaSNP2407	79.45	-	-
1362	CaLG2	CaTMS788	80	-	-
1363	CaLG2	TA110	82.76	-	-
1364	CaLG2	NCPGR13	82.98	scaffold03059	28813
1365	CaLG2	NCPGR117	85.04	-	-
1366	CaLG2	CaSNP2722	85.55	scaffold01578	61692
1367	CaLG2	CaGMS1160	86.26	-	-
1368	CaLG2	CaTMS798	86.76	-	-
1369	CaLG2	NCPGR28	94.34	scaffold02262	63594
1370	CaLG2	CaTMS690	96.37	-	-
1371	CaLG2	NCPGR110	98.74	scaffold05493	10876
1372	CaLG2	CaTMS648	98.78	-	-
1373	CaLG2	CaTMS516	99.9	-	-
1374	CaLG2	CESSR131	104.5	-	-
1375	CaLG2	PIP81	108.7	-	-
1376	CaLG2	NCPGR40	124.5	-	-
1377	CaLG2	CaGMS218	136.6	-	-
1378	CaLG3	CaGMS1158	0	-	-
1379	CaLG3	CaTMS930	0	-	-
1380	CaLG3	CaTMS1051	3.409	-	-
1381	CaLG3	CaTMS671	4.102	-	-
1382	CaLG3	CaTMS664	4.182	-	-
1383	CaLG3	CEST103	4.399	-	-
1384	CaLG3	CaGMS12	9.067	-	-
1385	CaLG3	TA76	9.285	-	-
1386	CaLG3	GAA45	11.25	-	-
1387	CaLG3	CaTMS580	13.17	-	-

1388	CaLG3	CESSR105	13.55	scaffold00006	858964
1389	CaLG3	CaTMS722	14.07	-	-
1390	CaLG3	CaTMS823	14.07	-	-
1391	CaLG3	CaTMS982	14.07	-	-
1392	CaLG3	CaTMS615	14.07	-	-
1393	CaLG3	CaTMS781	16.11	-	-
1394	CaLG3	PIP69	18.18	-	-
1395	CaLG3	NCPGR272	18.78	-	-
1396	CaLG3	CaTSNP8442	18.81	-	-
1397	CaLG3	CaTSNP8602	18.95	-	-
1398	CaLG3	CaTSNP8584	19.15	scaffold04520	15226
1399	CaLG3	CaTSNP7519	19.15	-	-
1400	CaLG3	CaTMS666	19.17	-	-
1401	CaLG3	CaTSNP7859	19.17	-	-
1402	CaLG3	CaTSNP8574	19.34	-	-
1403	CaLG3	CaTMS859	19.39	-	-
1404	CaLG3	CaTSNP6044	19.41	-	-
1405	CaLG3	CESSR136	19.46	scaffold00287	225553
1406	CaLG3	CaSNP3391	19.53	scaffold00194	230652
1407	CaLG3	CaSNP3004	19.6	scaffold00711	131265
1408	CaLG3	CaTMS811	20.03	-	-
1409	CaLG3	CaSNP410	20.19	-	-
1410	CaLG3	CaSNP198	20.42	scaffold00018	644708
1411	CaLG3	NCPGR62	20.67	scaffold02806	39229
1412	CaLG3	CaTMS654	21.02	-	-
1413	CaLG3	CaTMS914	21.28	-	-
1414	CaLG3	CaSNP409	21.32	-	-
1415	CaLG3	CaGMS39	21.4	-	-
1416	CaLG3	CaTSNP6635	21.49	-	-

1417	CaLG3	ESNP60	22.34	-	-
1418	CaLG3	STMS5	22.38	-	-
1419	CaLG3	CaTSNP7207	22.65	9199797	57082
1420	CaLG3	CaTSNP6601	22.77	-	-
1421	CaLG3	CaTSNP8002	22.79	-	-
1422	CaLG3	CaTSNP8104	22.79	-	-
1423	CaLG3	CaSNP304	23.17	-	-
1424	CaLG3	NCPGR54	23.36	9073711	15843
1425	CaLG3	CaSNP2042	23.4	-	-
1426	CaLG3	TR26	23.58	-	-
1427	CaLG3	CaGMS1164	23.65	-	-
1428	CaLG3	CaTSNP6995	24.31	-	-
1429	CaLG3	CaSNP657	24.83	scaffold00184	269194
1430	CaLG3	CaSNP137	24.91	9202610	60555
1431	CaLG3	CaGMS310	25.07	-	-
1432	CaLG3	CaTMS1116	25.34	-	-
1433	CaLG3	CaTMS1097	25.47	-	-
1434	CaLG3	CaSNP766	25.5	scaffold00034	513174
1435	CaLG3	CaSNP791	25.5	-	-
1436	CaLG3	CaSNP70	25.54	-	-
1437	CaLG3	CaSNP903	25.54	-	-
1438	CaLG3	CaSNP468	25.9	scaffold00332	184134
1439	CaLG3	GA105	26.06	-	-
1440	CaLG3	CaTMS1070	26.17	-	-
1441	CaLG3	CaTSNP7546	26.3	-	-
1442	CaLG3	CaTSNP7672	26.69	-	-
1443	CaLG3	CaTMS1079	27.01	-	-
1444	CaLG3	CaTSNP6815	27.19	-	-
1445	CaLG3	CaTSNP8330	27.19	-	-

1446	CaLG3	CaTSNP8906	27.19	-	-
1447	CaLG3	CaSNP324	27.21	-	-
1448	CaLG3	CaTSNP6958	27.21	-	-
1449	CaLG3	CaTSNP8359	27.21	-	-
1450	CaLG3	CaSNP3400	27.28	-	-
1451	CaLG3	CaSNP211	27.62	-	-
1452	CaLG3	CaSNP3052	27.68	-	-
1453	CaLG3	CaTMS691	27.7	-	-
1454	CaLG3	CaTSNP6392	27.91	-	-
1455	CaLG3	CaTMS743	27.94	-	-
1456	CaLG3	CaSNP749	27.96	-	-
1457	CaLG3	CaTSNP6037	28.07	-	-
1458	CaLG3	CaSNP230	28.27	-	-
1459	CaLG3	CaSNP144	28.32	-	-
1460	CaLG3	CaSNP423	28.32	-	-
1461	CaLG3	CaTSNP7493	28.38	-	-
1462	CaLG3	CaTSNP6909	28.63	-	-
1463	CaLG3	CaTSNP7443	28.65	-	-
1464	CaLG3	CaTSNP8191	28.65	-	-
1465	CaLG3	CaTSNP8777	28.65	-	-
1466	CaLG3	CaTMS783	28.72	-	-
1467	CaLG3	CaSNP3076	29.21	-	-
1468	CaLG3	CaTSNP6184	29.37	-	-
1469	CaLG3	CaTSNP7228	29.4	-	-
1470	CaLG3	CaTSNP7433	29.4	-	-
1471	CaLG3	CaSNP3358	29.55	-	-
1472	CaLG3	CaSNP623	29.56	-	-
1473	CaLG3	CaTSNP7180	29.67	-	-
1474	CaLG3	CaSNP1926	29.7	-	-

1475	CaLG3	CaSNP3518	29.7	scaffold01947	58125
1476	CaLG3	CaTSNP8050	29.72	-	-
1477	CaLG3	CaTSNP6288	29.76	-	-
1478	CaLG3	CaTSNP7456	29.76	-	-
1479	CaLG3	CaTSNP7301	29.89	scaffold00333	192794
1480	CaLG3	CaTSNP7095	29.96	-	-
1481	CaLG3	CaSNP890	29.99	scaffold00133	278845
1482	CaLG3	NCPGR43	30.24	-	-
1483	CaLG3	CaTSNP9113	30.34	-	-
1484	CaLG3	CaSNP273	30.69	-	-
1485	CaLG3	CaSNP690	30.74	-	-
1486	CaLG3	CaTSNP7411	30.99	-	-
1487	CaLG3	CaSNP2522	31.19	scaffold00576	135713
1488	CaLG3	CaTSNP8566	31.29	scaffold02581	35945
1489	CaLG3	CaSNP2166	31.31	-	-
1490	CaLG3	CaSNP365	31.37	scaffold00142	271827
1491	CaLG3	CaTSNP7504	31.6	-	-
1492	CaLG3	CaTSNP6717	31.61	-	-
1493	CaLG3	CaTSNP7640	31.66	-	-
1494	CaLG3	CaSNP413	31.67	-	-
1495	CaLG3	CaTSNP7811	31.68	-	-
1496	CaLG3	CaTSNP7866	31.68	-	-
1497	CaLG3	CaTSNP8064	31.72	-	-
1498	CaLG3	CaSNP119	31.81	-	-
1499	CaLG3	CaSNP232	31.87	-	-
1500	CaLG3	CaSNP583	31.93	9201208	30379
1501	CaLG3	ESNP94	31.93	scaffold00033	520839
1502	CaLG3	CaTSNP8581	31.96	-	-
1503	CaLG3	CaTSNP9154	32	-	-

1504	CaLG3	CaTMS890	32	-	-
1505	CaLG3	CaSNP212	32.03	scaffold00017	653561
1506	CaLG3	CaSNP479	32.03	-	-
1507	CaLG3	CaSNP816	32.03	9139990	28153
1508	CaLG3	ESNP78	32.03	-	-
1509	CaLG3	CaTSNP8345	32.05	-	-
1510	CaLG3	CaTMS915	32.29	-	-
1511	CaLG3	CaSNP422	32.32	-	-
1512	CaLG3	CaSNP819	32.35	-	-
1513	CaLG3	CaSNP774	32.39	-	-
1514	CaLG3	ESNP25	32.39	9211922	9185
1515	CaLG3	NCPGR12	32.43	-	-
1516	CaLG3	CaTMS1007	32.53	-	-
1517	CaLG3	CaSNP502	33.17	-	-
1518	CaLG3	CaSNP830	33.22	9180910	9543
1519	CaLG3	CaSNP250	33.25	-	-
1520	CaLG3	CaSNP494	33.25	-	-
1521	CaLG3	CaTSNP7550	33.35	-	-
1522	CaLG3	CaTMS774	33.61	-	-
1523	CaLG3	CaTMS698	33.73	-	-
1524	CaLG3	CaTMS843	33.78	-	-
1525	CaLG3	CaTMS579	33.87	-	-
1526	CaLG3	CaGMS24	34.11	-	-
1527	CaLG3	CaTMS1094	34.13	-	-
1528	CaLG3	CaTMS581	34.13	-	-
1529	CaLG3	CaTMS921	34.21	-	-
1530	CaLG3	CaTMS624	34.31	-	-
1531	CaLG3	CaTSNP8591	34.44	-	-
1532	CaLG3	CaTSNP7951	34.46	-	-

1533	CaLG3	CaSNP4902	34.48	scaffold02850	31276
1534	CaLG3	CaTSNP6658	34.49	-	-
1535	CaLG3	CaTSNP6876	34.51	-	-
1536	CaLG3	CaTSNP7883	34.51	-	-
1537	CaLG3	CaTMS1024	34.61	-	-
1538	CaLG3	CaSNP3289	34.67	-	-
1539	CaLG3	CaTSNP6623	34.67	-	-
1540	CaLG3	CaTSNP7800	34.88	9213471	13799
1541	CaLG3	CaGMS34	35.02	-	-
1542	CaLG3	CaTSNP7245	35.06	-	-
1543	CaLG3	CaTSNP6760	35.11	-	-
1544	CaLG3	CaTSNP7761	35.11	-	-
1545	CaLG3	CaTSNP6021	35.12	-	-
1546	CaLG3	CaSNP5063	35.21	-	-
1547	CaLG3	CaSNP3867	35.26	-	-
1548	CaLG3	CaTSNP6834	35.28	-	-
1549	CaLG3	CaTSNP8822	35.29	-	-
1550	CaLG3	CaTSNP7822	35.34	-	-
1551	CaLG3	CaTSNP6148	35.41	9210073	25691
1552	CaLG3	CaTSNP9136	35.44	scaffold01070	89369
1553	CaLG3	CaTSNP6973	35.54	-	-
1554	CaLG3	CaTMS522	35.54	-	-
1555	CaLG3	CaTSNP8289	35.56	-	-
1556	CaLG3	CaTSNP6165	35.57	-	-
1557	CaLG3	CaTSNP8800	35.57	-	-
1558	CaLG3	CaTSNP6384	35.6	-	-
1559	CaLG3	CaTSNP6578	35.6	-	-
1560	CaLG3	CaTSNP6580	35.6	-	-
1561	CaLG3	CaTSNP8267	35.78	-	-

1562	CaLG3	CaSNP3576	35.81	-	-
1563	CaLG3	CaSNP1999	35.82	-	-
1564	CaLG3	CaTSNP8703	35.93	-	-
1565	CaLG3	CaTSNP8981	35.95	-	-
1566	CaLG3	CaTSNP8217	35.95	-	-
1567	CaLG3	CaTSNP6514	35.98	-	-
1568	CaLG3	CaSNP2281	35.99	-	-
1569	CaLG3	CaSNP1980	35.99	-	-
1570	CaLG3	CaTSNP6274	36	-	-
1571	CaLG3	CaTSNP7921	36	scaffold09014	6185
1572	CaLG3	CaTSNP6273	36	-	-
1573	CaLG3	CaTSNP7079	36.03	-	-
1574	CaLG3	CaTSNP8358	36.03	-	-
1575	CaLG3	CaTSNP8515	36.03	-	-
1576	CaLG3	CaTSNP7827	36.66	-	-
1577	CaLG3	CaTSNP8308	36.68	-	-
1578	CaLG3	CaTSNP8488	36.68	-	-
1579	CaLG3	CaTSNP6193	36.74	-	-
1580	CaLG3	CaTSNP6194	36.74	-	-
1581	CaLG3	CaTSNP6655	36.74	-	-
1582	CaLG3	CaTSNP6910	36.74	-	-
1583	CaLG3	CaTSNP6929	36.74	-	-
1584	CaLG3	CaTSNP7041	36.74	-	-
1585	CaLG3	CaTSNP8067	36.74	-	-
1586	CaLG3	CaTSNP8238	36.74	-	-
1587	CaLG3	CaTSNP8727	36.74	-	-
1588	CaLG3	CaTSNP8815	36.75	-	-
1589	CaLG3	CaTSNP6504	36.76	-	-
1590	CaLG3	CaTSNP6567	36.76	-	-

1591	CaLG3	CaTSNP7568	36.76	-	-
1592	CaLG3	CaTSNP7639	36.76	-	-
1593	CaLG3	CaTSNP7652	36.76	-	-
1594	CaLG3	CaTSNP6413	36.79	-	-
1595	CaLG3	GA31	36.84	-	-
1596	CaLG3	CaTSNP8089	36.94	-	-
1597	CaLG3	CaTSNP7990	37.33	scaffold00528	151863
1598	CaLG3	TAA169	37.49	-	-
1599	CaLG3	CaTSNP7314	37.55	-	-
1600	CaLG3	CaTSNP8497	37.57	-	-
1601	CaLG3	CaTSNP7841	37.6	-	-
1602	CaLG3	CaTSNP8463	37.62	-	-
1603	CaLG3	CaTSNP6654	37.66	-	-
1604	CaLG3	CaTSNP8721	37.66	9213186	19396
1605	CaLG3	CaTSNP8018	37.69	-	-
1606	CaLG3	CaTSNP8468	38.01	9206248	3341
1607	CaLG3	CaTSNP7536	38.02	scaffold00040	472599
1608	CaLG3	CaSNP2347	38.12	scaffold02808	38750
1609	CaLG3	CaTSNP8552	38.29	-	-
1610	CaLG3	CaTSNP7678	38.49	-	-
1611	CaLG3	CaTSNP8909	38.49	-	-
1612	CaLG3	CaTSNP9004	38.49	-	-
1613	CaLG3	CaTSNP9119	38.49	-	-
1614	CaLG3	CaTSNP9138	38.49	-	-
1615	CaLG3	CaTSNP6442	38.54	-	-
1616	CaLG3	CaTSNP6999	38.54	-	-
1617	CaLG3	CaTSNP8202	38.54	-	-
1618	CaLG3	CaTSNP8967	38.54	-	-
1619	CaLG3	CaTSNP7441	38.57	-	-

1620	CaLG3	CaTSNP8215	38.57	-	-
1621	CaLG3	CaSNP2932	38.68	-	-
1622	CaLG3	CaSNP2949	38.93	-	-
1623	CaLG3	CaSNP2088	39.05	-	-
1624	CaLG3	CaSNP3549	39.16	scaffold00123	295072
1625	CaLG3	CaSNP4923	39.2	-	-
1626	CaLG3	CaTSNP8404	39.25	-	-
1627	CaLG3	CaTSNP8958	39.44	-	-
1628	CaLG3	CaTSNP6154	39.56	-	-
1629	CaLG3	CaSNP4984	39.73	scaffold00031	561848
1630	CaLG3	CaTSNP6022	39.79	-	-
1631	CaLG3	CaTMS969	39.96	-	-
1632	CaLG3	CaSNP2983	40.19	scaffold00210	252444
1633	CaLG3	CaTSNP6034	40.2	-	-
1634	CaLG3	CaSNP2443	40.22	-	-
1635	CaLG3	CaTSNP6744	40.26	-	-
1636	CaLG3	CaTSNP6294	40.27	-	-
1637	CaLG3	CaTSNP6997	40.28	-	-
1638	CaLG3	CaTSNP6788	40.3	-	-
1639	CaLG3	CaSNP2121	40.33	scaffold00316	206248
1640	CaLG3	CaSNP4484	40.33	scaffold00308	225104
1641	CaLG3	CaTSNP8510	40.66	-	-
1642	CaLG3	CaTSNP8537	40.84	-	-
1643	CaLG3	CaTSNP9072	40.84	-	-
1644	CaLG3	CaTSNP9129	41.17	-	-
1645	CaLG3	CaTSNP7024	41.22	-	-
1646	CaLG3	CaTSNP7083	41.22	9184401	70758
1647	CaLG3	CaTSNP6914	41.27	scaffold00296	204015
1648	CaLG3	CaTSNP7440	41.92	-	-

1649	CaLG3	CaTSNP6061	42.04	-	-
1650	CaLG3	CaTSNP8760	42.06	scaffold00085	373145
1651	CaLG3	CaTSNP9130	42.08	9202071	65125
1652	CaLG3	CaTSNP6613	42.09	-	-
1653	CaLG3	CaTSNP7336	42.17	-	-
1654	CaLG3	CaGMS13	42.24	-	-
1655	CaLG3	CaSNP2875	42.33	scaffold03705	27868
1656	CaLG3	CaSNP378	42.47	-	-
1657	CaLG3	CaSNP500	42.72	-	-
1658	CaLG3	CaTMS724	42.84	-	-
1659	CaLG3	CaSNP602	42.98	-	-
1660	CaLG3	CaSNP512	43.47	-	-
1661	CaLG3	CaSNP538	43.47	-	-
1662	CaLG3	CaTSNP7415	43.54	-	-
1663	CaLG3	CaTSNP6574	43.55	9215750	39716
1664	CaLG3	CaTSNP6774	43.59	scaffold00164	255174
1665	CaLG3	CaTSNP7902	43.59	-	-
1666	CaLG3	CaTSNP7333	43.63	-	-
1667	CaLG3	CaTSNP8660	43.63	9169222	20586
1668	CaLG3	CaTSNP6647	43.64	-	-
1669	CaLG3	CaTSNP8873	43.64	-	-
1670	CaLG3	CaTSNP8590	43.7	-	-
1671	CaLG3	NCPGR274	43.81	-	-
1672	CaLG3	TS19	44.21	-	-
1673	CaLG3	CaSNP135	44.4	scaffold00039	484273
1674	CaLG3	CaSNP335	44.4	-	-
1675	CaLG3	CaSNP408	45.44	scaffold00365	182807
1676	CaLG3	CaTMS999	45.92	-	-
1677	CaLG3	CaSNP170	46.01	scaffold00645	151929

1678	CaLG3	CaSNP210	46.01	-	-
1679	CaLG3	CaTSNP8910	46.28	-	-
1680	CaLG3	CaGMS1214	46.31	-	-
1681	CaLG3	ESNP96	46.44	scaffold01372	75864
1682	CaLG3	CaTMS616	46.54	-	-
1683	CaLG3	CaTSNP6177	46.81	scaffold00703	122244
1684	CaLG3	CaTSNP9122	46.85	9199729	27279
1685	CaLG3	CaTSNP7840	46.85	-	-
1686	CaLG3	CaTSNP8689	46.85	-	-
1687	CaLG3	CaTSNP7929	46.99	-	-
1688	CaLG3	CaTSNP8528	47.27	-	-
1689	CaLG3	CaSNP301	47.6	-	-
1690	CaLG3	CaTSNP9134	47.66	-	-
1691	CaLG3	STMS14	47.69	-	-
1692	CaLG3	CaTSNP8272	47.74	-	-
1693	CaLG3	CaSNP2738	47.8	-	-
1694	CaLG3	CaTSNP8799	47.81	-	-
1695	CaLG3	CaTMS1089	47.87	-	-
1696	CaLG3	CaSNP469	48	scaffold00109	323515
1697	CaLG3	CaSNP3244	48.12	9213630	73727
1698	CaLG3	TR31	48.16	-	-
1699	CaLG3	TR56	48.22	-	-
1700	CaLG3	TA64	48.39	-	-
1701	CaLG3	CaTMS949	48.45	-	-
1702	CaLG3	CaTMS1082	48.45	-	-
1703	CaLG3	CaTMS792	48.45	-	-
1704	CaLG3	CaGMS1161	48.46	-	-
1705	CaLG3	CaTMS515	48.46	-	-
1706	CaLG3	CaTMS650	48.46	-	-

1707	CaLG3	CaTMS1036	48.46	-	-
1708	CaLG3	CaTMS634	48.46	-	-
1709	CaLG3	CaSNP295	48.55	scaffold00220	234936
1710	CaLG3	CaTSNP8076	48.7	-	-
1711	CaLG3	CaTSNP7048	48.72	9202970	15429
1712	CaLG3	TA34	48.73	-	-
1713	CaLG3	CaSNP4389	48.75	9210744	28666
1714	CaLG3	CaTSNP7566	48.79	9214105	20200
1715	CaLG3	CaTSNP7865	48.79	-	-
1716	CaLG3	CaTSNP8279	48.79	-	-
1717	CaLG3	TA135	48.85	-	-
1718	CaLG3	CaSNP2496	48.92	-	-
1719	CaLG3	CaTMS963	49.01	-	-
1720	CaLG3	CaTMS1054	49.01	-	-
1721	CaLG3	TR2	49.03	-	-
1722	CaLG3	CaGMS1322	49.04	-	-
1723	CaLG3	CaGMS21	49.04	-	-
1724	CaLG3	CaTMS1098	49.04	-	-
1725	CaLG3	CaTMS512	49.04	-	-
1726	CaLG3	CaTMS753	49.04	-	-
1727	CaLG3	CaTMS794	49.04	-	-
1728	CaLG3	CaTMS885	49.04	-	-
1729	CaLG3	CaTSNP8102	49.04	-	-
1730	CaLG3	CaGMS1325	49.05	-	-
1731	CaLG3	CaTMS525	49.06	-	-
1732	CaLG3	CaTMS964	49.07	-	-
1733	CaLG3	CaGMS1276	49.07	-	-
1734	CaLG3	CaTMS1013	49.07	-	-
1735	CaLG3	CaTMS1020	49.07	-	-

1736	CaLG3	CaTMS1061	49.07	-	-
1737	CaLG3	CaTMS545	49.07	-	-
1738	CaLG3	CaTMS701	49.07	-	-
1739	CaLG3	CaTMS807	49.07	-	-
1740	CaLG3	CaTMS936	49.07	-	-
1741	CaLG3	CaGMS1143	49.07	-	-
1742	CaLG3	CaGMS1264	49.07	-	-
1743	CaLG3	CaTMS768	49.08	-	-
1744	CaLG3	CaGMS1312	49.09	-	-
1745	CaLG3	CaGMS1227	49.1	-	-
1746	CaLG3	CaTMS601	49.13	-	-
1747	CaLG3	CaTSNP9159	49.47	-	-
1748	CaLG3	CaSNP121	49.51	-	-
1749	CaLG3	CaTSNP8922	49.54	-	-
1750	CaLG3	CaTSNP7302	49.6	-	-
1751	CaLG3	CaTSNP6961	49.62	scaffold03225	37605
1752	CaLG3	CaSNP519	49.64	scaffold00541	138086
1753	CaLG3	CaTSNP8645	49.65	scaffold00424	166226
1754	CaLG3	STMS10	49.67	-	-
1755	CaLG3	CaTSNP6675	49.67	-	-
1756	CaLG3	CaTSNP6977	49.67	-	-
1757	CaLG3	CaTSNP7924	49.67	-	-
1758	CaLG3	CaTSNP8406	49.8	-	-
1759	CaLG3	CaSNP3	49.82	-	-
1760	CaLG3	STMS4	49.83	-	-
1761	CaLG3	CaSNP461	49.86	scaffold00144	293813
1762	CaLG3	CaTSNP7651	49.93	-	-
1763	CaLG3	CaSNP13	50.17	-	-
1764	CaLG3	CaSNP674	50.17	-	-

1765	CaLG3	CaTSNP7508	50.19	-	-
1766	CaLG3	CaSNP233	50.2	-	-
1767	CaLG3	CaTSNP6990	50.32	-	-
1768	CaLG3	CaTSNP8417	50.33	-	-
1769	CaLG3	CaTSNP7160	50.34	scaffold00182	267587
1770	CaLG3	CaSNP3818	50.4	-	-
1771	CaLG3	NCPGR220	50.52	-	-
1772	CaLG3	CaTSNP6577	50.69	-	-
1773	CaLG3	CaTSNP8575	50.8	-	-
1774	CaLG3	ESNP17	50.9	9194419	24521
1775	CaLG3	CaSNP4514	51.53	scaffold03186	28268
1776	CaLG3	CaSNP2122	51.6	-	-
1777	CaLG3	CaSNP3053	51.6	scaffold02175	43398
1778	CaLG3	CaSNP3294	51.6	scaffold01214	76100
1779	CaLG3	CaSNP3383	52.52	-	-
1780	CaLG3	CaTMS866	52.69	-	-
1781	CaLG3	CaSNP3999	52.73	scaffold01834	59623
1782	CaLG3	CaTSNP8597	52.74	-	-
1783	CaLG3	H1B04	52.78	-	-
1784	CaLG3	CaSNP5047	53.08	-	-
1785	CaLG3	CaTSNP7812	53.22	-	-
1786	CaLG3	CaTSNP6826	53.23	-	-
1787	CaLG3	CaTSNP8633	53.23	-	-
1788	CaLG3	CaTSNP9103	53.23	-	-
1789	CaLG3	CaTSNP8817	53.23	-	-
1790	CaLG3	CaTSNP8750	53.33	-	-
1791	CaLG3	CaTSNP6259	53.47	-	-
1792	CaLG3	CaSNP4881	53.54	scaffold03050	31451
1793	CaLG3	CaSNP2090	53.62	-	-

1794	CaLG3	CaTSNP7037	53.71	-	-
1795	CaLG3	CaTSNP8205	53.71	-	-
1796	CaLG3	CaTSNP6562	53.71	-	-
1797	CaLG3	CaSNP2333	53.85	-	-
1798	CaLG3	CaSNP2232	53.86	-	-
1799	CaLG3	CaTSNP8788	53.88	-	-
1800	CaLG3	CaSNP3411	53.9	scaffold00408	164156
1801	CaLG3	CaTSNP8894	53.97	-	-
1802	CaLG3	CaTSNP6560	54.01	-	-
1803	CaLG3	CaTSNP7085	54.03	-	-
1804	CaLG3	CaTSNP7321	54.03	-	-
1805	CaLG3	NCPGR180	54.24	-	-
1806	CaLG3	CaSNP4812	54.45	-	-
1807	CaLG3	CaTSNP8587	54.68	-	-
1808	CaLG3	CaSNP4182	55	scaffold01220	110021
1809	CaLG3	CaTSNP8120	55.61	-	-
1810	CaLG3	CaSNP2130	56.12	-	-
1811	CaLG3	CaSNP2556	56.12	scaffold00825	116211
1812	CaLG3	NCPGR225	56.5	-	-
1813	CaLG3	H3H04	56.75	-	-
1814	CaLG3	CaTMS741	57.17	-	-
1815	CaLG3	CaTSNP6621	57.7	-	-
1816	CaLG3	CaTSNP8068	57.7	-	-
1817	CaLG3	CaTSNP9143	57.7	-	-
1818	CaLG3	CaTSNP7189	57.87	-	-
1819	CaLG3	CaTSNP7673	57.87	-	-
1820	CaLG3	CaTSNP7891	57.87	-	-
1821	CaLG3	CaTSNP8761	57.87	-	-
1822	CaLG3	CaTSNP8464	58.7	-	-

1823	CaLG3	CaTSNP8096	58.73	-	-
1824	CaLG3	CaTSNP6104	59.12	scaffold02636	36823
1825	CaLG3	CaTSNP7588	59.14	scaffold00471	150645
1826	CaLG3	CaSNP3699	59.33	-	-
1827	CaLG3	CaSNP3575	59.34	scaffold00011	779820
1828	CaLG3	CaTSNP8884	59.46	-	-
1829	CaLG3	CaTSNP6143	59.58	-	-
1830	CaLG3	CaTSNP9049	59.62	scaffold05465	10996
1831	CaLG3	CaTSNP7473	59.75	-	-
1832	CaLG3	CaTSNP6884	59.79	-	-
1833	CaLG3	CaTSNP8798	59.79	scaffold00357	184185
1834	CaLG3	CaSNP1957	59.92	9197961	38262
1835	CaLG3	CaSSR5	60.01	-	-
1836	CaLG3	CaTSNP9077	60.15	-	-
1837	CaLG3	CaTMS932	60.15	-	-
1838	CaLG3	CaTMS543	60.16	-	-
1839	CaLG3	CaTSNP7586	60.6	scaffold00465	162026
1840	CaLG3	CaTSNP8489	60.6	-	-
1841	CaLG3	CaTSNP8449	60.76	-	-
1842	CaLG3	CaTSNP6731	60.88	scaffold00788	107512
1843	CaLG3	CaTSNP6523	60.89	scaffold01367	87936
1844	CaLG3	CaTSNP7721	60.95	scaffold03385	25361
1845	CaLG3	CaTSNP8333	61.01	-	-
1846	CaLG3	CaTSNP6113	61.16	-	-
1847	CaLG3	CaTSNP8045	61.32	scaffold04793	14300
1848	CaLG3	CaSNP3417	61.38	-	-
1849	CaLG3	CaTSNP7742	61.43	scaffold02575	45176
1850	CaLG3	CaTSNP7747	61.43	-	-
1851	CaLG3	CaTSNP8827	61.43	-	-

1852	CaLG3	CaTSNP8033	61.44	-	-
1853	CaLG3	CaTSNP7871	61.49	-	-
1854	CaLG3	CaSNP4641	61.64	-	-
1855	CaLG3	CaSNP2894	61.74	-	-
1856	CaLG3	CaSNP2543	61.75	scaffold00407	169941
1857	CaLG3	CaSNP2633	61.75	-	-
1858	CaLG3	CaSNP2798	61.75	scaffold01674	68135
1859	CaLG3	CaSNP2882	61.75	-	-
1860	CaLG3	CaSNP2207	61.98	scaffold00230	232557
1861	CaLG3	CaTSNP7913	62.05	-	-
1862	CaLG3	CaTSNP7334	62.21	-	-
1863	CaLG3	CaTSNP7773	62.23	-	-
1864	CaLG3	CaTSNP6900	62.32	scaffold05271	11926
1865	CaLG3	CaSNP2846	62.38	-	-
1866	CaLG3	CaSNP2256	62.41	scaffold00320	191303
1867	CaLG3	CaSNP3290	62.43	-	-
1868	CaLG3	CaTSNP6743	62.52	scaffold00271	209559
1869	CaLG3	CaTSNP7457	62.52	scaffold00905	112137
1870	CaLG3	CaTSNP6984	62.56	-	-
1871	CaLG3	CaTSNP8950	62.68	scaffold00499	151422
1872	CaLG3	CaSNP4030	62.76	scaffold00628	136417
1873	CaLG3	CaSNP4156	62.76	scaffold01864	55711
1874	CaLG3	H4F09	62.77	-	-
1875	CaLG3	CaTSNP8698	63.06	-	-
1876	CaLG3	CaSNP2785	63.42	-	-
1877	CaLG3	CaTSNP7106	63.63	-	-
1878	CaLG3	CaTSNP8219	63.63	-	-
1879	CaLG3	CaTSNP8034	63.66	scaffold00090	347567
1880	CaLG3	CaTSNP7266	64.35	-	-

1881	CaLG3	CaSNP368	64.42	scaffold03702	30386
1882	CaLG3	CaSNP467	64.44	-	-
1883	CaLG3	CaSNP271	64.46	9209923	38133
1884	CaLG3	CaSNP4235	64.6	scaffold00324	224821
1885	CaLG3	CaTSNP8260	64.6	-	-
1886	CaLG3	CaTMS1108	64.7	-	-
1887	CaLG3	CaTSNP7066	64.72	-	-
1888	CaLG3	CaSNP205	64.79	-	-
1889	CaLG3	CaSNP542	64.79	-	-
1890	CaLG3	CaTSNP8022	65.04	-	-
1891	CaLG3	CaSNP871	65.21	scaffold00804	111933
1892	CaLG3	ESNP13	65.21	scaffold00644	134581
1893	CaLG3	CaSNP35	65.22	-	-
1894	CaLG3	CaSNP516	65.23	scaffold00960	102451
1895	CaLG3	CaTSNP6074	65.25	scaffold01647	66741
1896	CaLG3	CaTSNP6157	65.26	scaffold00562	148988
1897	CaLG3	CaTSNP7602	65.26	-	-
1898	CaLG3	CaTSNP8911	65.26	-	-
1899	CaLG3	ESNP34	65.27	scaffold02239	53179
1900	CaLG3	CaTSNP7623	65.33	-	-
1901	CaLG3	CaTSNP8199	65.33	-	-
1902	CaLG3	CaTSNP8519	65.33	-	-
1903	CaLG3	CaTSNP8004	65.51	9200021	54790
1904	CaLG3	CaSNP1973	65.54	scaffold00414	163409
1905	CaLG3	CaTMS832	65.75	-	-
1906	CaLG3	CaTSNP7012	65.76	-	-
1907	CaLG3	CaTSNP9141	65.84	scaffold00159	267128
1908	CaLG3	CaSNP4664	66.19	scaffold00185	240475
1909	CaLG3	CaSNP2862	66.23	scaffold01053	95448

1910	CaLG3	CaSNP2239	66.23	scaffold01856	50948
1911	CaLG3	CaTSNP8329	66.3	-	-
1912	CaLG3	CaTSNP6327	66.34	-	-
1913	CaLG3	CaTSNP7494	66.34	-	-
1914	CaLG3	CaTMS519	66.35	-	-
1915	CaLG3	CaGMS7	66.48	-	-
1916	CaLG3	CaGMS1199	66.59	-	-
1917	CaLG3	CaSNP2896	66.62	scaffold02089	55452
1918	CaLG3	CaSNP3225	66.62	scaffold00702	133683
1919	CaLG3	CaSNP3404	66.62	scaffold01698	58352
1920	CaLG3	CaSNP4080	66.62	scaffold02302	43468
1921	CaLG3	CaSNP2269	66.62	scaffold00947	92620
1922	CaLG3	CaSNP2676	66.66	scaffold00440	162222
1923	CaLG3	CaSNP4127	66.97	scaffold02753	36203
1924	CaLG3	CaTSNP7332	67.1	scaffold01719	62112
1925	CaLG3	CaSNP3473	67.16	scaffold00706	133238
1926	CaLG3	CaTSNP7770	67.2	-	-
1927	CaLG3	CaTSNP8796	67.2	-	-
1928	CaLG3	CaTSNP6559	67.21	-	-
1929	CaLG3	CaTSNP8957	67.21	-	-
1930	CaLG3	CaTSNP6219	67.22	-	-
1931	CaLG3	CaSNP4680	67.24	scaffold04368	25689
1932	CaLG3	CaSNP876	67.24	-	-
1933	CaLG3	CaSNP4561	67.27	-	-
1934	CaLG3	CaSNP3254	67.29	-	-
1935	CaLG3	CaTSNP9024	67.3	scaffold00180	296104
1936	CaLG3	CaTSNP6724	67.32	9207770	26395
1937	CaLG3	CaTSNP6771	67.33	-	-
1938	CaLG3	CaTSNP7218	67.33	-	-

1939	CaLG3	CaTSNP7591	67.33	-	-
1940	CaLG3	CaTSNP6055	67.33	scaffold03157	27774
1941	CaLG3	CaTSNP6064	67.33	-	-
1942	CaLG3	CaTSNP6264	67.33	-	-
1943	CaLG3	CaTSNP6451	67.33	-	-
1944	CaLG3	CaTSNP6493	67.33	scaffold00168	295856
1945	CaLG3	CaTSNP6541	67.33	-	-
1946	CaLG3	CaTSNP7011	67.33	scaffold01139	96918
1947	CaLG3	CaTSNP7217	67.33	scaffold03221	27748
1948	CaLG3	CaTSNP7367	67.33	-	-
1949	CaLG3	CaTSNP7762	67.33	-	-
1950	CaLG3	CaTSNP8634	67.33	-	-
1951	CaLG3	CaTSNP9087	67.33	-	-
1952	CaLG3	CaTSNP7580	67.33	-	-
1953	CaLG3	CaTSNP7018	67.33	scaffold00936	93906
1954	CaLG3	CaSNP2978	67.34	-	-
1955	CaLG3	CaSNP3981	67.35	scaffold00580	139784
1956	CaLG3	CaSNP4762	67.36	-	-
1957	CaLG3	CaSNP5119	67.36	scaffold01283	87860
1958	CaLG3	CaSNP3392	67.36	-	-
1959	CaLG3	CaSNP2876	67.37	scaffold01137	92569
1960	CaLG3	CaSNP4431	67.37	scaffold03611	24969
1961	CaLG3	CaSNP4588	67.37	-	-
1962	CaLG3	CaSNP2966	67.38	-	-
1963	CaLG3	CaSNP3763	67.38	scaffold02834	32903
1964	CaLG3	CaSNP1954	67.38	scaffold00392	166732
1965	CaLG3	CaSNP2229	67.38	scaffold00747	112596
1966	CaLG3	CaSNP2588	67.38	scaffold00587	170478
1967	CaLG3	CaSNP2617	67.38	-	-

1968	CaLG3	CaSNP2637	67.38	-	-
1969	CaLG3	CaSNP3179	67.38	scaffold01098	95314
1970	CaLG3	CaSNP3414	67.38	scaffold01596	77775
1971	CaLG3	CaSNP3573	67.38	-	-
1972	CaLG3	CaSNP4094	67.38	-	-
1973	CaLG3	CaSNP4229	67.38	-	-
1974	CaLG3	CaSNP4267	67.38	-	-
1975	CaLG3	CaSNP4269	67.38	-	-
1976	CaLG3	CaSNP4304	67.38	scaffold02677	38537
1977	CaLG3	CaSNP4349	67.38	scaffold01579	69395
1978	CaLG3	CaSNP4717	67.38	scaffold02041	55399
1979	CaLG3	CaSNP5011	67.38	scaffold01627	63035
1980	CaLG3	CaSNP5098	67.38	-	-
1981	CaLG3	CaSNP3605	67.38	scaffold00988	94437
1982	CaLG3	CaSNP2296	67.38	9188145	18015
1983	CaLG3	CaSNP2392	67.38	scaffold00402	196332
1984	CaLG3	CaSNP2403	67.38	scaffold00328	208376
1985	CaLG3	CaSNP2736	67.38	-	-
1986	CaLG3	CaSNP2974	67.38	scaffold00355	198414
1987	CaLG3	CaSNP2991	67.38	scaffold00208	261591
1988	CaLG3	CaSNP3019	67.38	scaffold01727	72781
1989	CaLG3	CaSNP3337	67.38	-	-
1990	CaLG3	CaSNP3882	67.38	scaffold01621	70582
1991	CaLG3	CaSNP4247	67.38	scaffold00823	120138
1992	CaLG3	CaSNP4311	67.38	scaffold00417	190469
1993	CaLG3	CaSNP4427	67.38	scaffold03279	35905
1994	CaLG3	CaSNP4573	67.38	scaffold01720	58024
1995	CaLG3	CaSNP2459	67.38	-	-
1996	CaLG3	CaSNP2538	67.38	scaffold01212	92225

1997	CaLG3	CaSNP2789	67.38	9215602	41823
1998	CaLG3	CaSNP3323	67.38	scaffold01950	63254
1999	CaLG3	CaSNP3616	67.38	scaffold02037	50928
2000	CaLG3	CaSNP3620	67.38	scaffold01622	68823
2001	CaLG3	CaSNP3653	67.38	scaffold02508	36441
2002	CaLG3	CaSNP4012	67.38	-	-
2003	CaLG3	CaSNP4203	67.38	scaffold05156	12186
2004	CaLG3	CaSNP4287	67.38	scaffold02967	37698
2005	CaLG3	CaSNP5004	67.38	scaffold03461	25761
2006	CaLG3	CaSNP5118	67.38	scaffold01760	72544
2007	CaLG3	CaSNP5136	67.38	scaffold01568	66251
2008	CaLG3	CaSNP2018	67.39	9209817	17125
2009	CaLG3	CaSNP2300	67.39	scaffold04251	20255
2010	CaLG3	CaSNP3162	67.39	scaffold01825	52002
2011	CaLG3	CaSNP4467	67.39	scaffold00535	166574
2012	CaLG3	CaSNP4509	67.39	9189986	4183
2013	CaLG3	CaSNP5043	67.44	scaffold00559	155926
2014	CaLG3	CaSNP3285	67.45	scaffold00459	150187
2015	CaLG3	CaSNP571	67.66	scaffold00069	438970
2016	CaLG3	CaTSNP6775	67.84	scaffold08816	5293
2017	CaLG3	CaTSNP7338	67.84	-	-
2018	CaLG3	CaTSNP8802	67.85	scaffold02738	40540
2019	CaLG3	CaTSNP8774	67.86	-	-
2020	CaLG3	CaTSNP7794	67.88	-	-
2021	CaLG3	CaSNP3001	67.91	scaffold03438	24483
2022	CaLG3	CaTSNP6095	68	scaffold07053	8292
2023	CaLG3	CaTSNP6220	68	-	-
2024	CaLG3	CaTSNP6720	68	-	-
2025	CaLG3	CaTSNP6822	68	-	-

2026	CaLG3	CaTSNP7548	68	-	-
2027	CaLG3	CaTSNP8026	68	-	-
2028	CaLG3	CaTSNP8271	68	9204776	9601
2029	CaLG3	CaTSNP9131	68	scaffold01945	60252
2030	CaLG3	CaTSNP6716	68	-	-
2031	CaLG3	CaTSNP7518	68	-	-
2032	CaLG3	CaTSNP6079	68.01	-	-
2033	CaLG3	CaTSNP6116	68.01	-	-
2034	CaLG3	CaTSNP6203	68.01	-	-
2035	CaLG3	CaTSNP6461	68.01	-	-
2036	CaLG3	CaTSNP7082	68.01	-	-
2037	CaLG3	CaTSNP7223	68.01	-	-
2038	CaLG3	CaTSNP7356	68.01	9191524	38406
2039	CaLG3	CaTSNP7435	68.01	-	-
2040	CaLG3	CaTSNP7718	68.01	scaffold04961	13034
2041	CaLG3	CaTSNP7830	68.01	-	-
2042	CaLG3	CaTSNP8473	68.01	-	-
2043	CaLG3	CaTSNP8756	68.01	scaffold01809	52910
2044	CaLG3	CaTSNP8869	68.01	scaffold02319	40451
2045	CaLG3	CaTSNP9144	68.01	-	-
2046	CaLG3	CaTSNP8415	68.01	-	-
2047	CaLG3	CaTSNP7319	68.01	-	-
2048	CaLG3	CaTSNP7482	68.01	scaffold02157	49364
2049	CaLG3	CaTSNP9107	68.02	-	-
2050	CaLG3	CaTSNP7088	68.02	-	-
2051	CaLG3	CaTSNP7113	68.02	-	-
2052	CaLG3	CaTSNP8000	68.02	-	-
2053	CaLG3	CaTSNP8781	68.02	-	-
2054	CaLG3	CaTSNP7641	68.02	-	-

2055	CaLG3	CaTSNP8621	68.02	-	-
2056	CaLG3	CaTSNP6010	68.03	-	-
2057	CaLG3	CaTSNP7267	68.03	-	-
2058	CaLG3	CaTSNP7327	68.03	-	-
2059	CaLG3	CaTSNP6689	68.03	-	-
2060	CaLG3	CaSNP2148	68.15	-	-
2061	CaLG3	CaSNP3038	68.19	scaffold00761	107525
2062	CaLG3	CaSNP4359	68.22	scaffold02058	48786
2063	CaLG3	CaSNP5091	68.23	scaffold01702	76474
2064	CaLG3	CaTSNP8112	68.23	-	-
2065	CaLG3	CaSNP4474	68.23	scaffold01790	71457
2066	CaLG3	CaSNP3598	68.23	-	-
2067	CaLG3	CaSNP4442	68.25	scaffold01890	51418
2068	CaLG3	CaSNP3071	68.26	scaffold01626	76999
2069	CaLG3	CaSNP3204	68.28	9202146	12225
2070	CaLG3	CaSNP4549	68.29	scaffold12330	7077
2071	CaLG3	CaSNP4899	68.3	-	-
2072	CaLG3	CaSNP4014	68.31	scaffold01148	90614
2073	CaLG3	CaSNP5018	68.32	scaffold00342	186535
2074	CaLG3	CaSNP5010	68.32	9211988	38892
2075	CaLG3	CaSNP4320	68.32	scaffold07233	7747
2076	CaLG3	CaSNP3713	68.33	-	-
2077	CaLG3	CaSNP4051	68.33	scaffold00278	213177
2078	CaLG3	CaSNP2566	68.33	scaffold01304	82359
2079	CaLG3	CaSNP2642	68.33	scaffold00396	184840
2080	CaLG3	CaSNP2835	68.33	scaffold02871	31218
2081	CaLG3	CaSNP3023	68.33	scaffold01763	53544
2082	CaLG3	CaSNP3045	68.33	scaffold01314	81488
2083	CaLG3	CaSNP3138	68.33	scaffold01187	87098

2084	CaLG3	CaSNP3272	68.33	-	-
2085	CaLG3	CaSNP3556	68.33	-	-
2086	CaLG3	CaSNP3916	68.33	scaffold00577	142858
2087	CaLG3	CaSNP4520	68.33	scaffold03235	33243
2088	CaLG3	CaSNP4643	68.33	scaffold02615	35222
2089	CaLG3	CaSNP4657	68.33	scaffold00385	205075
2090	CaLG3	CaSNP5013	68.33	scaffold02241	45220
2091	CaLG3	CaSNP2215	68.33	scaffold01616	82301
2092	CaLG3	CaSNP2517	68.33	scaffold01397	74414
2093	CaLG3	CaSNP2524	68.33	-	-
2094	CaLG3	CaSNP2536	68.33	scaffold03647	26194
2095	CaLG3	CaSNP2603	68.33	-	-
2096	CaLG3	CaSNP2606	68.33	scaffold00811	128661
2097	CaLG3	CaSNP2811	68.33	scaffold00785	118462
2098	CaLG3	CaSNP2826	68.33	scaffold01781	65658
2099	CaLG3	CaSNP3040	68.33	scaffold02118	46694
2100	CaLG3	CaSNP3050	68.33	-	-
2101	CaLG3	CaSNP3107	68.33	scaffold02227	54675
2102	CaLG3	CaSNP3282	68.33	scaffold02038	47715
2103	CaLG3	CaSNP3317	68.33	-	-
2104	CaLG3	CaSNP3429	68.33	scaffold02212	46357
2105	CaLG3	CaSNP3543	68.33	scaffold01138	94134
2106	CaLG3	CaSNP3626	68.33	scaffold02583	47976
2107	CaLG3	CaSNP3665	68.33	scaffold06397	8616
2108	CaLG3	CaSNP3762	68.33	scaffold01981	59252
2109	CaLG3	CaSNP3767	68.33	-	-
2110	CaLG3	CaSNP3798	68.33	scaffold02524	41998
2111	CaLG3	CaSNP3858	68.33	scaffold02818	37544
2112	CaLG3	CaSNP3868	68.33	scaffold01368	90494

2113	CaLG3	CaSNP3983	68.33	scaffold01292	75041
2114	CaLG3	CaSNP4048	68.33	scaffold02259	51149
2115	CaLG3	CaSNP4115	68.33	scaffold03039	36614
2116	CaLG3	CaSNP4188	68.33	scaffold04992	13673
2117	CaLG3	CaSNP4367	68.33	-	-
2118	CaLG3	CaSNP4396	68.33	scaffold03110	28750
2119	CaLG3	CaSNP4508	68.33	scaffold02948	38779
2120	CaLG3	CaSNP4587	68.33	-	-
2121	CaLG3	CaSNP4636	68.33	scaffold02092	53198
2122	CaLG3	CaSNP4672	68.33	scaffold01563	70813
2123	CaLG3	CaSNP4703	68.33	scaffold02377	60142
2124	CaLG3	CaSNP4939	68.33	scaffold02232	61521
2125	CaLG3	CaSNP5088	68.33	scaffold01471	86227
2126	CaLG3	CaSNP5143	68.33	scaffold00478	188563
2127	CaLG3	CaSNP5150	68.33	scaffold02540	47381
2128	CaLG3	CaSNP4882	68.33	scaffold02065	61890
2129	CaLG3	CaSNP4365	68.34	scaffold04587	14830
2130	CaLG3	CaSNP2816	68.34	scaffold01252	87628
2131	CaLG3	CaSNP4437	68.34	scaffold02520	47563
2132	CaLG3	CaSNP4789	68.34	scaffold01838	54605
2133	CaLG3	CaSNP2958	68.34	9194899	46200
2134	CaLG3	CaSNP4604	68.34	scaffold00817	119188
2135	CaLG3	CaSNP4013	68.34	scaffold03377	32344
2136	CaLG3	CaSNP2258	68.34	9205993	44057
2137	CaLG3	CaSNP2304	68.34	scaffold01254	89895
2138	CaLG3	CaSNP2652	68.34	scaffold01181	99489
2139	CaLG3	CaSNP2703	68.34	scaffold00052	546235
2140	CaLG3	CaSNP3433	68.34	scaffold02971	37675
2141	CaLG3	CaSNP3588	68.34	-	-

2142	CaLG3	CaSNP3677	68.34	scaffold02584	41567
2143	CaLG3	CaSNP3738	68.34	scaffold00908	111229
2144	CaLG3	CaSNP3832	68.34	scaffold01065	111368
2145	CaLG3	CaSNP3834	68.34	scaffold02928	30120
2146	CaLG3	CaSNP3994	68.34	scaffold02493	41255
2147	CaLG3	CaSNP4044	68.34	-	-
2148	CaLG3	CaSNP4082	68.34	scaffold01708	56323
2149	CaLG3	CaSNP4310	68.34	scaffold03866	46854
2150	CaLG3	CaSNP4452	68.34	-	-
2151	CaLG3	CaSNP4640	68.34	-	-
2152	CaLG3	CaSNP4681	68.34	-	-
2153	CaLG3	CaSNP4709	68.34	scaffold03471	26029
2154	CaLG3	CaSNP4794	68.34	scaffold03114	27759
2155	CaLG3	CaSNP4823	68.34	9196841	14546
2156	CaLG3	CaSNP4602	68.35	scaffold01297	87366
2157	CaLG3	CaSNP2662	68.35	scaffold01406	71448
2158	CaLG3	CaSNP2161	68.35	-	-
2159	CaLG3	CaSNP3116	68.35	scaffold04878	13372
2160	CaLG3	CaSNP3757	68.35	scaffold02403	46346
2161	CaLG3	CaSNP4627	68.35	scaffold01426	72450
2162	CaLG3	CaSNP4702	68.35	scaffold00993	109138
2163	CaLG3	CaSNP3266	68.35	scaffold00600	126749
2164	CaLG3	CaSNP4173	68.35	-	-
2165	CaLG3	CaSNP4414	68.35	-	-
2166	CaLG3	CaSNP4856	68.35	scaffold01902	52515
2167	CaLG3	CaSNP4936	68.35	-	-
2168	CaLG3	CaSNP2000	68.35	scaffold00561	152604
2169	CaLG3	CaSNP2409	68.35	scaffold02496	38309
2170	CaLG3	CaSNP2744	68.35	scaffold01508	72358

2171	CaLG3	CaSNP2884	68.35	scaffold02607	41163
2172	CaLG3	CaSNP3061	68.35	scaffold02075	61734
2173	CaLG3	CaSNP3134	68.35	scaffold01888	50993
2174	CaLG3	CaSNP3160	68.35	scaffold00973	104337
2175	CaLG3	CaSNP3261	68.35	scaffold00604	144390
2176	CaLG3	CaSNP3313	68.35	scaffold02459	42456
2177	CaLG3	CaSNP4246	68.35	scaffold01996	69148
2178	CaLG3	CaSNP4571	68.35	-	-
2179	CaLG3	CaSNP4864	68.35	scaffold02644	44542
2180	CaLG3	CaSNP2831	68.35	9212832	19766
2181	CaLG3	CaSNP3276	68.35	scaffold00868	120190
2182	CaLG3	CaSNP4261	68.36	9214521	4605
2183	CaLG3	CaSNP2795	68.36	scaffold02690	37936
2184	CaLG3	CaSNP4628	68.36	scaffold01567	82267
2185	CaLG3	CaSNP3062	68.37	scaffold06879	7610
2186	CaLG3	CaSNP4629	68.37	9190030	17588
2187	CaLG3	CaSNP3835	68.37	scaffold01607	71017
2188	CaLG3	CaSNP3326	68.37	scaffold08031	6758
2189	CaLG3	CaSNP4223	68.37	scaffold00904	129328
2190	CaLG3	CaSNP4746	68.37	scaffold06701	7939
2191	CaLG3	CaSNP3146	68.38	scaffold01800	54096
2192	CaLG3	CaSNP4053	68.38	scaffold03665	28486
2193	CaLG3	CaSNP4506	68.38	-	-
2194	CaLG3	CaSNP4542	68.38	-	-
2195	CaLG3	CaSNP4903	68.38	scaffold03041	28579
2196	CaLG3	CaSNP3850	68.38	scaffold02474	44979
2197	CaLG3	CaSNP2351	68.38	-	-
2198	CaLG3	CaSNP1996	68.38	scaffold00068	421811
2199	CaLG3	CaSNP2478	68.38	scaffold01681	64929

2200	CaLG3	CaSNP2544	68.38	scaffold00511	149155
2201	CaLG3	CaSNP2880	68.38	scaffold01718	58631
2202	CaLG3	CaSNP3115	68.38	-	-
2203	CaLG3	CaSNP3595	68.38	scaffold00950	109305
2204	CaLG3	CaSNP3864	68.38	scaffold00680	157436
2205	CaLG3	CaSNP4045	68.38	scaffold03577	22439
2206	CaLG3	CaSNP4271	68.38	-	-
2207	CaLG3	CaSNP4400	68.38	scaffold01059	108487
2208	CaLG3	CaSNP4440	68.38	-	-
2209	CaLG3	CaSNP4500	68.38	scaffold02922	30095
2210	CaLG3	CaSNP4719	68.38	scaffold00543	147574
2211	CaLG3	CaSNP4986	68.38	scaffold03178	27049
2212	CaLG3	CaSNP5129	68.38	scaffold03282	25974
2213	CaLG3	CaSNP3172	68.38	scaffold00705	150444
2214	CaLG3	CaSNP2143	68.39	scaffold01522	62755
2215	CaLG3	CaSNP2632	68.39	scaffold00718	125359
2216	CaLG3	CaSNP2693	68.39	scaffold01175	99141
2217	CaLG3	CaSNP3002	68.39	scaffold00472	174696
2218	CaLG3	CaSNP3633	68.39	scaffold00624	131794
2219	CaLG3	CaSNP3708	68.39	-	-
2220	CaLG3	CaSNP3838	68.39	scaffold01068	91062
2221	CaLG3	CaSNP3988	68.39	scaffold01326	85466
2222	CaLG3	CaSNP4105	68.39	scaffold01348	76990
2223	CaLG3	CaSNP4539	68.39	scaffold01938	50113
2224	CaLG3	CaSNP4673	68.39	scaffold01843	65843
2225	CaLG3	CaSNP4713	68.39	scaffold02067	52135
2226	CaLG3	CaSNP4748	68.39	scaffold02656	51814
2227	CaLG3	CaSNP3690	68.39	scaffold01553	77219
2228	CaLG3	CaSNP3696	68.39	scaffold00854	128287

2229	CaLG3	CaSNP4517	68.39	scaffold00987	99368
2230	CaLG3	CaSNP3423	68.39	9211874	32279
2231	CaLG3	CaSNP4940	68.39	scaffold03170	27442
2232	CaLG3	CaSNP3926	68.39	scaffold01743	66455
2233	CaLG3	CaSNP3113	68.39	scaffold01300	77100
2234	CaLG3	CaSNP4204	68.39	scaffold00096	360744
2235	CaLG3	CaSNP1942	68.4	scaffold00667	126397
2236	CaLG3	CaSNP2336	68.4	scaffold00305	196279
2237	CaLG3	CaSNP2890	68.4	scaffold01209	116345
2238	CaLG3	CaSNP3048	68.4	scaffold01027	98779
2239	CaLG3	CaSNP3110	68.4	scaffold01754	64417
2240	CaLG3	CaSNP3123	68.4	-	-
2241	CaLG3	CaSNP3144	68.4	scaffold00216	256264
2242	CaLG3	CaSNP4417	68.4	scaffold03999	24350
2243	CaLG3	CaSNP4518	68.4	scaffold02192	52715
2244	CaLG3	CaSNP5134	68.4	scaffold02146	62271
2245	CaLG3	CaSNP4745	68.4	-	-
2246	CaLG3	CaSNP5005	68.41	-	-
2247	CaLG3	CaSNP5125	68.42	scaffold05654	10439
2248	CaLG3	CaSNP4866	68.46	scaffold02855	31529
2249	CaLG3	CaSNP4443	68.5	scaffold00745	140269
2250	CaLG3	CaSNP4925	68.57	scaffold00802	120240
2251	CaLG3	CaGMS5	68.65	scaffold03219	32147
2252	CaLG3	CaTMS815	68.72	-	-
2253	CaLG3	CaSNP3248	68.9	scaffold02132	50504
2254	CaLG3	CaSNP4585	68.96	9206097	19594
2255	CaLG3	CaSNP3525	68.98	scaffold02756	32902
2256	CaLG3	CaSNP713	69.88	-	-
2257	CaLG3	CaSNP4551	70.62	-	-

2258	CaLG3	CaSNP68	70.71	-	-
2259	CaLG3	CaSNP3353	70.87	-	-
2260	CaLG3	CaSNP88	71.16	-	-
2261	CaLG3	CaSNP686	71.3	scaffold00650	141255
2262	CaLG3	CaSNP515	71.73	scaffold01735	62495
2263	CaLG3	CaSNP596	71.76	scaffold02101	54737
2264	CaLG3	CaSNP3466	71.98	-	-
2265	CaLG3	ESNP19	72.33	-	-
2266	CaLG3	CaSNP886	72.34	-	-
2267	CaLG3	CaTMS721	72.37	-	-
2268	CaLG3	CaSNP535	72.6	-	-
2269	CaLG3	CaSNP148	72.62	scaffold00931	99148
2270	CaLG3	CaSNP513	72.88	-	-
2271	CaLG3	CaSNP106	72.98	-	-
2272	CaLG3	CaSNP118	73.12	9210244	22708
2273	CaLG3	NCPGR171	73.16	-	-
2274	CaLG3	CaSNP909	73.18	-	-
2275	CaLG3	CaSNP480	73.22	-	-
2276	CaLG3	NCPGR240	74.06	-	-
2277	CaLG3	NCPGR266	74.97	scaffold12596	3095
2278	CaLG3	NCPGR128	75.47	-	-
2279	CaLG3	NCPGR157	75.58	-	-
2280	CaLG3	NCPGR192	76	-	-
2281	CaLG3	NCPGR141	76.37	-	-
2282	CaLG3	NCPGR100	78.24	-	-
2283	CaLG3	NCPGR55	79.22	9175359	19979
2284	CaLG3	CESSR61	80.4	scaffold00036	498806
2285	CaLG3	NCPGR103	82.3	9216038	9390
2286	CaLG3	CaTMS562	84.2	-	-

2287	CaLG3	GA13	86.48	-	-
2288	CaLG3	NCPGR213	91.03	-	-
2289	CaLG3	CESSR103	114.9	-	-
2290	CaLG4	CaTMS720	0	-	-
2291	CaLG4	PIP12	6.868	-	-
2292	CaLG4	PIP75	25.69	-	-
2293	CaLG4	CEST164	26.46	-	-
2294	CaLG4	PIP72	32.75	scaffold01816	72127
2295	CaLG4	CESSR22	41.06	-	-
2296	CaLG4	CaTMS582	41.39	-	-
2297	CaLG4	CaTMS1119	41.99	-	-
2298	CaLG4	CaTMS606	41.99	-	-
2299	CaLG4	PIP35	42.22	-	-
2300	CaLG4	CESSR434	46.03	-	-
2301	CaLG4	H4F03	46.06	-	-
2302	CaLG4	PIP96	48.04	-	-
2303	CaLG4	GA129	50.59	-	-
2304	CaLG4	TA46	52.58	-	-
2305	CaLG4	Foc0	52.75	-	-
2306	CaLG4	CaTMS856	53.05	-	-
2307	CaLG4	H3C041	53.11	-	-
2308	CaLG4	CEST51	54.07	-	-
2309	CaLG4	CaSNP236	55.54	9205467	15971
2310	CaLG4	CaSNP495	55.59	scaffold00425	174469
2311	CaLG4	ESNP77	55.59	scaffold08729	5382
2312	CaLG4	CaSNP1930	56.27	-	-
2313	CaLG4	CaSNP2670	56.4	-	-
2314	CaLG4	CaSNP3804	56.45	scaffold01978	51741
2315	CaLG4	CaSNP2961	56.47	scaffold00280	225184

2316	CaLG4	CaSNP603	56.5	scaffold00051	443471
2317	CaLG4	CaSNP50	56.58	scaffold00195	237463
2318	CaLG4	CaSNP5076	56.62	-	-
2319	CaLG4	CaSNP4911	56.95	9211642	8941
2320	CaLG4	CaSNP2683	57.05	scaffold00207	254513
2321	CaLG4	CaSNP3571	57.05	scaffold07324	6937
2322	CaLG4	CaSNP1947	57.26	-	-
2323	CaLG4	CaGMS1263	57.32	-	-
2324	CaLG4	CaTSNP8408	57.42	-	-
2325	CaLG4	CaTSNP7909	57.55	-	-
2326	CaLG4	CaTSNP6860	57.59	9202511	12465
2327	CaLG4	CaTSNP8099	57.62	-	-
2328	CaLG4	CaTSNP6940	57.64	-	-
2329	CaLG4	CaTSNP8719	57.94	-	-
2330	CaLG4	CaTSNP6953	58.01	-	-
2331	CaLG4	CaSNP3794	58.04	-	-
2332	CaLG4	CaTSNP7197	58.04	-	-
2333	CaLG4	CaGMS1150	58.07	-	-
2334	CaLG4	CaTSNP6521	58.24	-	-
2335	CaLG4	CaSNP733	58.9	scaffold00529	147815
2336	CaLG4	ESNP26	59.07	scaffold00074	424271
2337	CaLG4	CaSNP4159	59.31	-	-
2338	CaLG4	CaSNP2493	59.39	-	-
2339	CaLG4	CaTSNP6015	59.42	9185396	7788
2340	CaLG4	CaTSNP7790	59.42	9152325	2419
2341	CaLG4	CaTSNP7193	59.81	-	-
2342	CaLG4	CaTSNP7285	59.81	scaffold00366	185859
2343	CaLG4	CaTSNP6622	59.83	-	-
2344	CaLG4	CaTSNP6530	59.83	9199637	36422

2345	CaLG4	CaTSNP8690	59.83	-	-
2346	CaLG4	CaTSNP8934	59.83	-	-
2347	CaLG4	CESSR60	59.84	-	-
2348	CaLG4	CaTSNP7563	59.9	-	-
2349	CaLG4	CESSR28	60.39	scaffold01033	95867
2350	CaLG4	CaSNP2448	60.53	-	-
2351	CaLG4	CaTSNP6376	61.79	-	-
2352	CaLG4	NCPGR254	62.09	scaffold01085	109866
2353	CaLG4	CaTSNP8318	62.47	scaffold00694	123450
2354	CaLG4	CaTSNP6607	63.62	9208255	12256
2355	CaLG4	CaTSNP7270	63.64	-	-
2356	CaLG4	CaTSNP8763	63.64	-	-
2357	CaLG4	CaTSNP6128	63.65	-	-
2358	CaLG4	CaTSNP7703	63.7	9204159	41162
2359	CaLG4	CaTSNP8539	66.11	-	-
2360	CaLG4	CaSNP2387	66.34	-	-
2361	CaLG4	CaSNP2196	66.5	scaffold02093	47419
2362	CaLG4	CaSNP3078	66.5	scaffold00799	133840
2363	CaLG4	CaTSNP7925	66.59	scaffold00374	182946
2364	CaLG4	CaTSNP8963	66.6	-	-
2365	CaLG4	CaTMS952	67.15	-	-
2366	CaLG4	CaSNP3694	67.24	scaffold00265	244004
2367	CaLG4	CaTSNP6819	67.3	-	-
2368	CaLG4	CaTSNP8071	67.3	-	-
2369	CaLG4	CaSNP2729	67.37	scaffold01226	78858
2370	CaLG4	CaTSNP8517	67.55	9190032	37941
2371	CaLG4	CaSNP3274	67.57	-	-
2372	CaLG4	CaTSNP7276	68.59	scaffold00641	129371
2373	CaLG4	CEST21	68.69	-	-

2374	CaLG4	CEST132	69.05	scaffold04338	26260
2375	CaLG4	CaTSNP6737	69.62	scaffold00794	122446
2376	CaLG4	CaSNP3826	69.64	-	-
2377	CaLG4	PIP217	69.86	-	-
2378	CaLG4	NCPGR127	69.87	scaffold00113	339829
2379	CaLG4	CaTSNP8031	70	scaffold00122	330031
2380	CaLG4	CaTSNP8632	70	-	-
2381	CaLG4	CaTSNP8787	70.28	scaffold00907	112649
2382	CaLG4	CaSNP314	70.3	-	-
2383	CaLG4	CaGMS20	70.32	-	-
2384	CaLG4	CaTSNP8107	70.32	scaffold00311	190055
2385	CaLG4	NCPGR129	70.37	scaffold01551	82746
2386	CaLG4	CaSNP2173	70.39	-	-
2387	CaLG4	CaSNP2494	70.46	-	-
2388	CaLG4	CaSNP4990	70.46	scaffold03076	28156
2389	CaLG4	CaTSNP6930	70.49	scaffold04267	16828
2390	CaLG4	CaTSNP6097	70.6	-	-
2391	CaLG4	CaTSNP8628	70.6	-	-
2392	CaLG4	CaSNP2504	70.61	-	-
2393	CaLG4	CaSNP2921	70.61	-	-
2394	CaLG4	CaTSNP8938	70.65	scaffold00998	89134
2395	CaLG4	CaSNP906	70.67	scaffold01706	74990
2396	CaLG4	CaTSNP7522	70.73	-	-
2397	CaLG4	CaSNP3642	70.82	-	-
2398	CaLG4	CaTSNP7855	71.17	scaffold00238	238748
2399	CaLG4	CaSNP4339	72.49	scaffold00369	197715
2400	CaLG4	CaGMS33	73.01	-	-
2401	CaLG4	CaSNP5058	73.52	scaffold00954	92440
2402	CaLG4	CaSNP428	73.83	-	-

2403	CaLG4	ESNP50	74.26	scaffold00275	210672
2404	CaLG4	CaSNP576	74.4	scaffold01478	64737
2405	CaLG4	CaSNP899	74.48	9205492	53517
2406	CaLG4	CaSNP316	74.57	-	-
2407	CaLG4	CaTSNP6600	74.66	-	-
2408	CaLG4	CaTSNP7600	74.72	-	-
2409	CaLG4	CaSNP2550	74.78	-	-
2410	CaLG4	CaSNP4050	74.96	scaffold01230	105763
2411	CaLG4	CaSNP415	75.22	scaffold00098	347727
2412	CaLG4	CaTSNP8943	75.49	-	-
2413	CaLG4	CaSNP616	75.56	-	-
2414	CaLG4	CaSNP315	75.65	scaffold00224	229184
2415	CaLG4	CaTSNP6440	75.71	-	-
2416	CaLG4	CaSNP66	75.74	-	-
2417	CaLG4	CaSNP663	75.74	-	-
2418	CaLG4	CaSNP2557	76.07	scaffold01342	69931
2419	CaLG4	CaSNP3199	76.07	scaffold06881	9377
2420	CaLG4	CaSNP2249	76.1	scaffold00733	111986
2421	CaLG4	CaTSNP6340	76.16	-	-
2422	CaLG4	CaSNP2498	76.24	scaffold00721	113098
2423	CaLG4	NCPGR150	76.28	scaffold00739	127512
2424	CaLG4	CaTSNP9071	76.32	-	-
2425	CaLG4	CaSNP201	76.49	scaffold01953	49045
2426	CaLG4	CaTSNP6497	76.52	-	-
2427	CaLG4	CaTSNP6948	76.52	-	-
2428	CaLG4	CaTSNP9008	76.65	-	-
2429	CaLG4	CaSNP5113	76.66	-	-
2430	CaLG4	CaTSNP8555	76.67	-	-
2431	CaLG4	CaSNP2203	76.7	scaffold00506	168284

2432	CaLG4	CaSNP2814	76.76	scaffold01330	83347
2433	CaLG4	CaTSNP7419	76.77	-	-
2434	CaLG4	CaTSNP6922	76.88	-	-
2435	CaLG4	CaTSNP6091	77.07	scaffold00530	142588
2436	CaLG4	CaTSNP7582	77.23	-	-
2437	CaLG4	CaTSNP8702	77.23	-	-
2438	CaLG4	CaTSNP8431	77.38	-	-
2439	CaLG4	CaTSNP7086	77.62	-	-
2440	CaLG4	CaTSNP9124	77.76	-	-
2441	CaLG4	CaTSNP7958	78.24	-	-
2442	CaLG4	CaSNP2095	78.25	-	-
2443	CaLG4	CaTSNP8325	78.29	-	-
2444	CaLG4	NCPGR190	78.53	-	-
2445	CaLG4	CaTMS992	78.68	-	-
2446	CaLG4	CaSNP868	78.87	-	-
2447	CaLG4	CaTMS686	79.13	-	-
2448	CaLG4	NCPGR69	79.13	-	-
2449	CaLG4	CaTSNP7647	79.16	-	-
2450	CaLG4	CaTSNP7294	79.17	-	-
2451	CaLG4	CaTSNP8208	79.21	-	-
2452	CaLG4	CaTSNP6441	80.33	-	-
2453	CaLG4	CaTSNP8974	80.33	-	-
2454	CaLG4	PIP77	80.38	-	-
2455	CaLG4	CaTSNP8470	80.4	-	-
2456	CaLG4	CaTSNP6506	80.42	-	-
2457	CaLG4	CaTSNP7572	80.42	-	-
2458	CaLG4	CaTSNP7754	80.49	-	-
2459	CaLG4	CaTSNP8328	80.49	-	-
2460	CaLG4	CaTSNP8665	80.49	-	-

2461	CaLG4	CaTMS830	80.6	-	-
2462	CaLG4	CaSNP2943	80.94	scaffold00345	194488
2463	CaLG4	TS54	81.08	-	-
2464	CaLG4	CaGMS48	81.26	-	-
2465	CaLG4	CaTSNP8338	81.34	-	-
2466	CaLG4	CaSNP3168	81.36	scaffold01086	95294
2467	CaLG4	CaSNP4665	81.36	-	-
2468	CaLG4	CaSNP4058	81.55	-	-
2469	CaLG4	CaTMS572	81.65	-	-
2470	CaLG4	CaTSNP8384	81.75	-	-
2471	CaLG4	CaTSNP6153	81.8	-	-
2472	CaLG4	CaTMS983	81.89	-	-
2473	CaLG4	CESSR191	82.56	-	-
2474	CaLG4	CaTMS584	82.62	-	-
2475	CaLG4	CaSNP2773	82.66	-	-
2476	CaLG4	CaSNP3754	82.82	scaffold02244	43923
2477	CaLG4	CaTMS714	82.97	-	-
2478	CaLG4	CaTMS631	83.01	-	-
2479	CaLG4	CaGMS1248	83.06	-	-
2480	CaLG4	CaTSNP8063	83.1	-	-
2481	CaLG4	CESSR66	83.24	scaffold00007	873403
2482	CaLG4	CaTMS976	83.32	-	-
2483	CaLG4	CaTMS550	83.6	-	-
2484	CaLG4	CaGMS1154	83.64	-	-
2485	CaLG4	NCPGR224	84.35	-	-
2486	CaLG4	NCPGR76	84.37	-	-
2487	CaLG4	GA2	84.45	-	-
2488	CaLG4	CaTMS1090	84.68	-	-
2489	CaLG4	TR20	85.17	-	-

2490	CaLG4	CaGMS47	85.23	-	-
2491	CaLG4	CaTMS985	85.42	-	-
2492	CaLG4	TA13	85.49	-	-
2493	CaLG4	TS72	85.64	-	-
2494	CaLG4	TA72	85.87	-	-
2495	CaLG4	STMS26	85.93	-	-
2496	CaLG4	CaTMS608	86.08	-	-
2497	CaLG4	TA146	86.16	-	-
2498	CaLG4	CaSNP3226	86.17	-	-
2499	CaLG4	CaTMS744	86.21	-	-
2500	CaLG4	CaSNP2040	86.43	-	-
2501	CaLG4	CaTMS594	86.44	-	-
2502	CaLG4	CESSR164	86.48	scaffold00778	112266
2503	CaLG4	CaSNP2278	86.54	-	-
2504	CaLG4	CESSR114	86.72	-	-
2505	CaLG4	CaSNP1956	86.78	-	-
2506	CaLG4	CaSNP308	86.78	scaffold00211	263412
2507	CaLG4	CaSNP3492	86.78	scaffold02102	44564
2508	CaLG4	CaSNP817	86.85	scaffold02115	59728
2509	CaLG4	CaSNP4694	87.06	-	-
2510	CaLG4	CaSNP3390	87.27	-	-
2511	CaLG4	CaTSNP7617	87.31	9179993	4651
2512	CaLG4	CaTSNP8321	87.4	9214561	17163
2513	CaLG4	CaSNP2068	87.41	scaffold00429	167086
2514	CaLG4	PIP148	87.46	-	-
2515	CaLG4	CaSNP577	87.5	-	-
2516	CaLG4	CaSNP3189	87.51	scaffold01360	73073
2517	CaLG4	CaSNP3338	87.55	-	-
2518	CaLG4	CaSNP3405	87.55	scaffold02712	39364

2519	CaLG4	CaSNP4085	87.55	-	-
2520	CaLG4	CaTSNP6075	87.71	scaffold01984	47468
2521	CaLG4	CaTSNP6618	87.71	scaffold00807	116175
2522	CaLG4	CaTMS707	87.76	-	-
2523	CaLG4	ESNP89	87.79	scaffold03163	37760
2524	CaLG4	CaTMS761	88.14	-	-
2525	CaLG4	CaSNP910	88.26	-	-
2526	CaLG4	CaSNP732	88.38	scaffold01020	91087
2527	CaLG4	CaSNP5114	88.47	scaffold06360	8710
2528	CaLG4	NCPGR214	88.51	-	-
2529	CaLG4	CaSNP574	88.54	9180975	30923
2530	CaLG4	CaTSNP6810	88.79	9204771	35765
2531	CaLG4	CaTSNP6921	88.96	scaffold00965	103316
2532	CaLG4	CaTSNP6352	89	scaffold00170	281199
2533	CaLG4	CaSNP3799	89.09	9182165	4533
2534	CaLG4	CaSNP4186	89.13	scaffold02744	33111
2535	CaLG4	CaSNP2555	89.38	scaffold00266	249735
2536	CaLG4	CaSNP3717	89.38	scaffold01634	64790
2537	CaLG4	CaSNP2697	89.4	scaffold03818	20106
2538	CaLG4	CaSNP827	89.42	-	-
2539	CaLG4	CaSNP2006	89.42	-	-
2540	CaLG4	TA130	89.56	-	-
2541	CaLG4	CaSNP3786	89.6	-	-
2542	CaLG4	CaSNP2134	89.69	-	-
2543	CaLG4	CaSNP2046	90	-	-
2544	CaLG4	CaSNP2170	90	9215854	15800
2545	CaLG4	CaSNP2668	90	scaffold01540	83431
2546	CaLG4	CaSNP2615	90.07	scaffold01180	84153
2547	CaLG4	CaSNP701	90.24	scaffold00704	143920

2548	CaLG4	CaSNP833	90.24	-	-
2549	CaLG4	CaTSNP7357	90.28	scaffold01751	70441
2550	CaLG4	CaSNP892	90.31	scaffold01133	92240
2551	CaLG4	CaSNP2469	90.31	scaffold01527	62597
2552	CaLG4	CaSNP2575	90.31	-	-
2553	CaLG4	CaTSNP6431	90.35	-	-
2554	CaLG4	CaSNP4826	90.36	scaffold00608	153420
2555	CaLG4	CaSNP855	90.56	-	-
2556	CaLG4	CaSNP180	90.72	9208964	52762
2557	CaLG4	CaTMS732	90.81	-	-
2558	CaLG4	CaTSNP7663	90.81	scaffold10626	4016
2559	CaLG4	CaTSNP7552	90.82	scaffold00285	204121
2560	CaLG4	CaSNP436	90.86	scaffold00235	226064
2561	CaLG4	CaTSNP6794	90.87	-	-
2562	CaLG4	CaTSNP8109	90.87	scaffold00866	132126
2563	CaLG4	CaSNP801	90.87	9215966	29621
2564	CaLG4	CaSNP2152	90.89	-	-
2565	CaLG4	CaSNP4043	90.93	-	-
2566	CaLG4	CaSNP343	90.94	scaffold00736	115521
2567	CaLG4	CaSNP579	90.98	scaffold02647	37608
2568	CaLG4	CaSNP528	91.03	scaffold01225	91713
2569	CaLG4	CaSNP541	91.14	scaffold00517	154350
2570	CaLG4	CaSNP664	91.14	scaffold00693	130134
2571	CaLG4	CaSNP4808	91.22	scaffold01195	88410
2572	CaLG4	CaSNP318	91.23	scaffold02511	45975
2573	CaLG4	CaTSNP7944	91.25	scaffold01043	88212
2574	CaLG4	CaTSNP6109	91.34	-	-
2575	CaLG4	CaTSNP6191	91.34	9209686	9945
2576	CaLG4	CaTSNP6234	91.44	scaffold00283	243659

2577	CaLG4	CaTSNP6892	91.44	-	-
2578	CaLG4	CaTSNP6768	91.48	scaffold01186	90610
2579	CaLG4	CaTSNP8982	91.5	scaffold00420	189553
2580	CaLG4	CaSNP3033	91.5	scaffold00593	157341
2581	CaLG4	CaTMS791	91.51	-	-
2582	CaLG4	CaSNP375	91.56	scaffold02398	39690
2583	CaLG4	CaSNP748	91.56	scaffold00935	93327
2584	CaLG4	CaSNP537	91.61	scaffold00676	142610
2585	CaLG4	STMS11	91.64	-	-
2586	CaLG4	CaSNP4093	91.85	scaffold02801	32710
2587	CaLG4	GAA47	91.88	-	-
2588	CaLG4	GA24	91.88	-	-
2589	CaLG4	CaSNP4297	91.97	scaffold02873	31010
2590	CaLG4	CaSNP2586	91.98	scaffold02281	50021
2591	CaLG4	NCPGR199	92.01	scaffold01942	57412
2592	CaLG4	CaTMS1058	92.09	-	-
2593	CaLG4	CaTMS726	92.13	-	-
2594	CaLG4	CaGMS37	92.27	-	-
2595	CaLG4	CaSNP4019	92.28	scaffold01302	86487
2596	CaLG4	CaSNP4682	92.4	-	-
2597	CaLG4	CaSNP2771	92.41	scaffold00623	133452
2598	CaLG4	CaTSNP6045	92.49	9080947	6822
2599	CaLG4	CaTSNP7031	92.58	scaffold03955	19888
2600	CaLG4	CaSNP2138	92.63	-	-
2601	CaLG4	CaSNP3904	92.76	scaffold01749	62291
2602	CaLG4	CaSNP3306	92.77	scaffold00394	219262
2603	CaLG4	CaSNP2107	92.78	scaffold00690	143657
2604	CaLG4	CaSNP2747	92.8	scaffold01448	68776
2605	CaLG4	CaSNP3408	92.8	scaffold01525	73377

2606	CaLG4	CaSNP4624	92.8	-	-
2607	CaLG4	CaSNP4659	92.8	9206026	19656
2608	CaLG4	CaSNP3923	92.84	scaffold01545	75348
2609	CaLG4	CaSNP4245	92.84	scaffold02277	45702
2610	CaLG4	CaSNP2195	92.85	9213581	11046
2611	CaLG4	CaSNP4947	92.88	scaffold03038	30003
2612	CaLG4	PIP213	92.93	-	-
2613	CaLG4	CaSNP3655	93.04	scaffold01696	59410
2614	CaLG4	CaTSNP7362	93.12	-	-
2615	CaLG4	CaTSNP7880	93.17	-	-
2616	CaLG4	CaTSNP8991	93.2	-	-
2617	CaLG4	CaTMS840	93.26	-	-
2618	CaLG4	CaSNP3507	93.67	scaffold01519	82040
2619	CaLG4	CESSR46	93.8	scaffold00024	630645
2620	CaLG4	CaSNP2960	93.87	scaffold01764	57226
2621	CaLG4	CaSNP3693	93.89	-	-
2622	CaLG4	CaSNP2705	93.89	scaffold01021	106682
2623	CaLG4	CaSNP3440	93.9	9211781	22526
2624	CaLG4	CaSNP4821	93.94	-	-
2625	CaLG4	CaSNP2192	94.01	scaffold00757	126776
2626	CaLG4	CaSNP2059	94.02	9211584	33331
2627	CaLG4	CaSNP2614	94.02	9101375	10303
2628	CaLG4	CaSNP3800	94.02	scaffold00923	106972
2629	CaLG4	CaSNP3854	94.02	scaffold02343	40208
2630	CaLG4	CaSNP3880	94.02	scaffold01131	97914
2631	CaLG4	CaSNP4103	94.02	scaffold02510	42807
2632	CaLG4	CaSNP4292	94.02	-	-
2633	CaLG4	CaSNP4696	94.02	scaffold01944	62674
2634	CaLG4	CaSNP3579	94.03	scaffold00815	115743

2635	CaLG4	CaSNP5081	94.03	scaffold01216	105697
2636	CaLG4	CaSNP5092	94.03	scaffold04239	17748
2637	CaLG4	CaSNP2199	94.03	scaffold00847	102005
2638	CaLG4	CaSNP2332	94.03	scaffold01134	95549
2639	CaLG4	CaSNP2997	94.03	-	-
2640	CaLG4	CaSNP3663	94.03	-	-
2641	CaLG4	CaSNP3978	94.03	scaffold01261	88198
2642	CaLG4	CaSNP4625	94.03	scaffold03507	29545
2643	CaLG4	CaSNP4793	94.03	scaffold03048	28474
2644	CaLG4	CaSNP4800	94.03	scaffold01496	80188
2645	CaLG4	CaSNP4958	94.03	scaffold01975	55611
2646	CaLG4	CaSNP1997	94.04	scaffold00413	182349
2647	CaLG4	CaSNP2125	94.04	scaffold02894	31557
2648	CaLG4	CaSNP2500	94.04	scaffold02610	34786
2649	CaLG4	CaSNP2675	94.04	scaffold00871	104720
2650	CaLG4	CaSNP2713	94.04	-	-
2651	CaLG4	CaSNP3043	94.04	scaffold01952	65546
2652	CaLG4	CaSNP3177	94.04	scaffold01901	60661
2653	CaLG4	CaSNP3184	94.04	scaffold01411	68825
2654	CaLG4	CaSNP3347	94.04	scaffold01710	55469
2655	CaLG4	CaSNP3380	94.04	scaffold01559	61267
2656	CaLG4	CaSNP3449	94.04	scaffold01921	50268
2657	CaLG4	CaSNP4645	94.04	-	-
2658	CaLG4	CaSNP2984	94.04	-	-
2659	CaLG4	CaSNP2432	94.04	scaffold01673	78263
2660	CaLG4	CaSNP2690	94.04	-	-
2661	CaLG4	CaSNP4554	94.04	9216055	30083
2662	CaLG4	CaSNP5027	94.05	9100433	21507
2663	CaLG4	CaSNP2376	94.06	scaffold00770	117120

2664	CaLG4	CaSNP3434	94.06	scaffold05720	10272
2665	CaLG4	CaSNP3859	94.06	scaffold00575	164196
2666	CaLG4	CaSNP2007	94.06	scaffold01274	87650
2667	CaLG4	CaSNP2294	94.07	scaffold00857	100083
2668	CaLG4	CaSNP4221	94.07	scaffold02718	33506
2669	CaLG4	CaSNP2109	94.08	scaffold01023	99831
2670	CaLG4	CaSNP3531	94.08	9213705	21426
2671	CaLG4	CaSNP3568	94.08	scaffold02419	48087
2672	CaLG4	CaSNP3951	94.08	9214756	21794
2673	CaLG4	CaSNP4555	94.08	scaffold02074	56319
2674	CaLG4	CaSNP2784	94.08	-	-
2675	CaLG4	CaSNP2140	94.09	9209198	48722
2676	CaLG4	NCPGR226	94.1	scaffold00708	144296
2677	CaLG4	CaSNP2574	94.1	scaffold00777	142455
2678	CaLG4	CaSNP2231	94.1	-	-
2679	CaLG4	CaSNP4481	94.1	-	-
2680	CaLG4	CaSNP3233	94.14	scaffold03082	44070
2681	CaLG4	CaSNP2623	94.15	scaffold00768	131025
2682	CaLG4	CaSNP1964	94.16	scaffold01276	84746
2683	CaLG4	CaSNP4647	94.16	scaffold06416	8736
2684	CaLG4	CaSNP4422	94.16	scaffold01239	80377
2685	CaLG4	CaSNP3927	94.16	scaffold02868	40639
2686	CaLG4	CaSNP4553	94.18	scaffold13301	2854
2687	CaLG4	CaGMS25	94.18	-	-
2688	CaLG4	CaTMS680	94.18	-	-
2689	CaLG4	CaSNP4214	94.18	9211325	5679
2690	CaLG4	CaSNP2045	94.21	scaffold00526	170225
2691	CaLG4	CaSNP2043	94.26	9210497	39308
2692	CaLG4	CaSNP4660	94.26	scaffold04084	24096

2693	CaLG4	CaSNP3256	94.26	-	-
2694	CaLG4	CaSNP3231	94.27	-	-
2695	CaLG4	CaSNP4528	94.33	scaffold02230	45030
2696	CaLG4	CaSNP4425	94.33	scaffold00318	212843
2697	CaLG4	CaSNP4560	94.33	scaffold02717	36901
2698	CaLG4	CaSNP2108	94.34	9214963	42307
2699	CaLG4	CaSNP4263	94.34	scaffold03344	35422
2700	CaLG4	CaSNP3936	94.35	scaffold00538	161728
2701	CaLG4	CaSNP2382	94.35	scaffold08405	5694
2702	CaLG4	CaSNP2883	94.35	scaffold02941	39690
2703	CaLG4	CaSNP3673	94.35	-	-
2704	CaLG4	CaSNP2237	94.35	scaffold00941	123999
2705	CaLG4	CaSNP2344	94.35	-	-
2706	CaLG4	CaSNP3357	94.35	scaffold03008	41690
2707	CaLG4	CaSNP3659	94.35	-	-
2708	CaLG4	CaSNP4212	94.36	scaffold02368	43161
2709	CaLG4	CaSNP2401	94.36	scaffold00242	253303
2710	CaLG4	CaSNP4238	94.36	scaffold00971	106621
2711	CaLG4	CaSNP4686	94.36	scaffold00255	227536
2712	CaLG4	CaSNP5135	94.36	scaffold03643	22996
2713	CaLG4	CaSNP2119	94.36	scaffold00160	293868
2714	CaLG4	CaSNP3079	94.36	scaffold00448	178538
2715	CaLG4	CaSNP3209	94.36	scaffold01880	66839
2716	CaLG4	CaSNP5138	94.37	-	-
2717	CaLG4	CaSNP3092	94.37	scaffold01633	80841
2718	CaLG4	CaSNP3097	94.37	9194117	15464
2719	CaLG4	CaSNP5023	94.37	scaffold02674	43740
2720	CaLG4	CaSNP3937	94.37	9213209	27614
2721	CaLG4	CaSNP2200	94.37	scaffold00852	105325

2722	CaLG4	CaSNP2749	94.37	scaffold00729	128256
2723	CaLG4	CaSNP2752	94.37	scaffold01191	87948
2724	CaLG4	CaSNP3129	94.37	scaffold00557	159521
2725	CaLG4	CaSNP3476	94.37	scaffold02479	45841
2726	CaLG4	CaSNP3599	94.37	-	-
2727	CaLG4	CaSNP3635	94.37	scaffold01288	81050
2728	CaLG4	CaSNP4108	94.37	scaffold02775	32419
2729	CaLG4	CaSNP4136	94.37	9085008	4080
2730	CaLG4	CaSNP4141	94.37	scaffold04918	13236
2731	CaLG4	CaSNP4150	94.37	-	-
2732	CaLG4	CaSNP4294	94.37	scaffold01319	81886
2733	CaLG4	CaSNP4697	94.37	scaffold04659	14602
2734	CaLG4	CaSNP4714	94.37	scaffold02234	48511
2735	CaLG4	CaSNP4971	94.37	scaffold01218	75636
2736	CaLG4	CaSNP5009	94.37	-	-
2737	CaLG4	CaSNP3311	94.37	scaffold01972	55787
2738	CaLG4	CaSNP3425	94.37	scaffold02199	46096
2739	CaLG4	CaSNP4895	94.37	scaffold00371	197156
2740	CaLG4	CaSNP3301	94.38	scaffold01185	112101
2741	CaLG4	CaSNP2147	94.38	scaffold02485	36788
2742	CaLG4	CaSNP2422	94.38	scaffold01041	95383
2743	CaLG4	CaSNP2482	94.38	scaffold02222	41883
2744	CaLG4	CaSNP2602	94.38	scaffold00844	132314
2745	CaLG4	CaSNP2702	94.38	scaffold00975	121365
2746	CaLG4	CaSNP2873	94.38	9215131	17921
2747	CaLG4	CaSNP3026	94.38	scaffold02809	33239
2748	CaLG4	CaSNP3232	94.38	scaffold00775	142429
2749	CaLG4	CaSNP3324	94.38	scaffold02139	49776
2750	CaLG4	CaSNP3328	94.38	9215534	11632

2751	CaLG4	CaSNP3360	94.38	-	-
2752	CaLG4	CaSNP3385	94.38	scaffold02177	43886
2753	CaLG4	CaSNP3703	94.38	scaffold02595	43769
2754	CaLG4	CaSNP4356	94.38	9211556	22437
2755	CaLG4	CaSNP4478	94.38	9202976	36066
2756	CaLG4	CaSNP4510	94.38	-	-
2757	CaLG4	CaSNP4529	94.38	9208387	29553
2758	CaLG4	CaSNP4983	94.38	scaffold03319	25691
2759	CaLG4	CaSNP2863	94.39	scaffold01132	106057
2760	CaLG4	CaSNP1952	94.39	scaffold00399	174849
2761	CaLG4	CaSNP2374	94.4	scaffold01215	81702
2762	CaLG4	CaSNP3739	94.4	-	-
2763	CaLG4	CaSNP4199	94.4	scaffold01038	93629
2764	CaLG4	CaSNP4865	94.4	scaffold03410	24384
2765	CaLG4	CaSNP3407	94.4	scaffold01605	77358
2766	CaLG4	CaSNP4761	94.41	-	-
2767	CaLG4	CaSNP3183	94.41	-	-
2768	CaLG4	CaSNP2599	94.41	scaffold00999	109563
2769	CaLG4	CaSNP2981	94.41	scaffold00723	125597
2770	CaLG4	CaSNP4679	94.41	-	-
2771	CaLG4	CaSNP5072	94.41	scaffold01067	86400
2772	CaLG4	CaSNP4118	94.42	scaffold00751	128163
2773	CaLG4	CaSNP3398	94.42	scaffold02538	48727
2774	CaLG4	CaSNP3862	94.42	scaffold01740	55295
2775	CaLG4	CaSNP4851	94.42	-	-
2776	CaLG4	CaSNP2103	94.42	scaffold01648	92128
2777	CaLG4	CaSNP2739	94.42	-	-
2778	CaLG4	CaSNP3124	94.42	scaffold02317	44700
2779	CaLG4	CaSNP3666	94.42	-	-

2780	CaLG4	CaSNP4232	94.42	scaffold00738	137390
2781	CaLG4	CaSNP4863	94.42	scaffold03122	34097
2782	CaLG4	CaSNP3905	94.44	scaffold00376	195666
2783	CaLG4	CaSNP3499	94.44	scaffold02052	60579
2784	CaLG4	CaSNP4330	94.44	scaffold02696	36919
2785	CaLG4	CaSNP2956	94.44	scaffold01958	49866
2786	CaLG4	CaSNP2268	94.44	scaffold00423	177960
2787	CaLG4	CaSNP3721	94.44	scaffold01123	102483
2788	CaLG4	CaSNP4715	94.44	9176412	28258
2789	CaLG4	CaSNP4919	94.46	scaffold03102	28652
2790	CaLG4	CaSNP3751	94.48	scaffold04629	14586
2791	CaLG4	CaSNP2952	94.49	-	-
2792	CaLG4	CaSNP4563	94.51	scaffold03153	32881
2793	CaLG4	CaSNP2454	94.51	scaffold01386	77749
2794	CaLG4	CaSNP3649	94.52	9205346	33281
2795	CaLG4	CaSNP2489	94.53	-	-
2796	CaLG4	CaSNP4218	94.55	scaffold04113	18954
2797	CaLG4	CaSNP2634	94.55	scaffold00309	228419
2798	CaLG4	CaSNP2197	94.57	9190370	64303
2799	CaLG4	CaSNP2080	94.6	scaffold00553	139498
2800	CaLG4	CaSNP2287	94.61	-	-
2801	CaLG4	CaSNP4354	94.61	-	-
2802	CaLG4	CaSNP5007	94.61	-	-
2803	CaLG4	CaSNP2440	94.65	scaffold00284	207809
2804	CaLG4	CaSNP4352	94.69	scaffold01004	109458
2805	CaLG4	CaSNP3559	94.7	scaffold09729	4569
2806	CaLG4	CaSNP2641	94.78	scaffold00035	529391
2807	CaLG4	CaSNP4631	94.79	9166279	6293
2808	CaLG4	NCPGR75	95.39	-	-

2809	CaLG4	NCPGR80	95.5	scaffold00003	1127306
2810	CaLG4	CaTSNP8696	95.84	-	-
2811	CaLG4	CaTSNP7774	96.13	-	-
2812	CaLG4	CaTSNP8383	96.13	-	-
2813	CaLG4	CaTSNP8527	96.13	-	-
2814	CaLG4	CaTSNP7125	96.14	-	-
2815	CaLG4	CaTSNP6897	96.22	-	-
2816	CaLG4	CaTSNP8751	96.22	-	-
2817	CaLG4	CaTSNP6231	96.25	-	-
2818	CaLG4	CaTSNP7989	96.35	-	-
2819	CaLG4	CaTSNP7693	96.36	-	-
2820	CaLG4	CaTSNP6738	96.36	scaffold00754	113481
2821	CaLG4	CaTSNP8399	96.36	-	-
2822	CaLG4	CaTSNP6328	96.36	-	-
2823	CaLG4	CaTSNP6551	96.36	-	-
2824	CaLG4	CaTSNP6599	96.36	9101335	3732
2825	CaLG4	CaTSNP7751	96.38	9195470	13599
2826	CaLG4	CaTSNP6156	96.45	-	-
2827	CaLG4	CaTSNP6858	96.45	-	-
2828	CaLG4	CaTSNP9120	96.45	9202424	12422
2829	CaLG4	CaTSNP7524	96.5	-	-
2830	CaLG4	CaTSNP8048	96.55	-	-
2831	CaLG4	CaTSNP6359	96.55	-	-
2832	CaLG4	CaTSNP6030	96.58	-	-
2833	CaLG4	CaTSNP6031	96.58	-	-
2834	CaLG4	CaTSNP8078	96.58	scaffold00048	532239
2835	CaLG4	CaTSNP8620	96.58	-	-
2836	CaLG4	CaTSNP7862	96.59	-	-
2837	CaLG4	CaTSNP6527	96.6	-	-

2838	CaLG4	CaTSNP9133	96.66	-	-
2839	CaLG4	CaTSNP6421	96.66	-	-
2840	CaLG4	CaTSNP7491	96.74	9183474	4143
2841	CaLG4	CaTSNP7555	96.8	-	-
2842	CaLG4	CaTSNP9059	96.8	-	-
2843	CaLG4	CaTSNP6832	96.98	scaffold03776	20418
2844	CaLG4	CaTSNP8880	96.99	-	-
2845	CaLG4	CaTSNP6221	97	-	-
2846	CaLG4	CaTSNP8451	97.15	scaffold03191	26847
2847	CaLG4	CaTSNP6101	97.18	-	-
2848	CaLG4	CaTMS1074	97.54	-	-
2849	CaLG4	CaTSNP6918	97.64	9204332	22640
2850	CaLG4	CaTSNP6979	97.64	-	-
2851	CaLG4	CaTSNP8288	97.64	-	-
2852	CaLG4	CaTSNP6764	97.66	-	-
2853	CaLG4	CaTMS699	97.68	-	-
2854	CaLG4	CaTSNP8856	97.79	-	-
2855	CaLG4	CaTSNP6013	97.8	9200885	13994
2856	CaLG4	CaTSNP7509	97.8	-	-
2857	CaLG4	CaTSNP8367	97.8	9121324	22756
2858	CaLG4	CaTSNP8824	97.8	-	-
2859	CaLG4	CaTSNP6537	97.82	-	-
2860	CaLG4	CaTSNP8755	97.82	-	-
2861	CaLG4	CaTSNP8284	97.84	scaffold06039	9480
2862	CaLG4	CaTSNP6721	97.85	-	-
2863	CaLG4	CaTSNP8460	97.87	-	-
2864	CaLG4	CaTSNP7247	97.87	9214935	20147
2865	CaLG4	CaTSNP6365	97.88	-	-
2866	CaLG4	CaTMS773	97.9	-	-

2867	CaLG4	CaTSNP8037	97.93	-	-
2868	CaLG4	CaTSNP6399	97.95	-	-
2869	CaLG4	CaTSNP8145	97.95	-	-
2870	CaLG4	CaTSNP8891	97.96	scaffold00487	163871
2871	CaLG4	CaTSNP6893	97.97	-	-
2872	CaLG4	CaSNP2925	98.06	-	-
2873	CaLG4	CaTSNP6036	98.12	-	-
2874	CaLG4	CaTSNP8141	98.39	-	-
2875	CaLG4	CaTSNP7638	98.42	-	-
2876	CaLG4	CaSNP4438	98.52	scaffold04594	17806
2877	CaLG4	CaSNP2178	98.63	-	-
2878	CaLG4	CaTMS749	98.7	-	-
2879	CaLG4	CaSNP5045	99	-	-
2880	CaLG4	CaSNP2129	99.32	scaffold00419	188799
2881	CaLG4	CaSNP2472	99.42	-	-
2882	CaLG4	NCPGR91	99.45	scaffold00030	572599
2883	CaLG4	CaTSNP6136	99.62	-	-
2884	CaLG4	CaTSNP9037	99.62	-	-
2885	CaLG4	CaTSNP8438	99.62	-	-
2886	CaLG4	CaTSNP8895	99.66	-	-
2887	CaLG4	CaTSNP7320	99.68	-	-
2888	CaLG4	CaTSNP6134	99.69	-	-
2889	CaLG4	CaTSNP6135	99.69	-	-
2890	CaLG4	CaTSNP9079	99.69	-	-
2891	CaLG4	CaTSNP6872	99.75	-	-
2892	CaLG4	CaTSNP7070	99.75	-	-
2893	CaLG4	CaTSNP6185	99.76	-	-
2894	CaLG4	CaSNP672	100	scaffold00239	257882
2895	CaLG4	CaSNP882	100	scaffold00131	310065

2896	CaLG4	CaSNP529	100	scaffold00634	122739
2897	CaLG4	CaSNP4461	100.1	-	-
2898	CaLG4	CaTSNP6849	100.9	-	-
2899	CaLG4	CaTSNP6289	100.9	-	-
2900	CaLG4	CaTSNP6453	100.9	-	-
2901	CaLG4	CaTSNP7002	101	-	-
2902	CaLG4	CaTSNP7448	101.1	-	-
2903	CaLG4	CaTSNP8391	101.1	-	-
2904	CaLG4	CaTSNP9066	101.2	-	-
2905	CaLG4	CaTSNP7141	101.4	-	-
2906	CaLG4	CaTSNP6807	101.5	-	-
2907	CaLG4	CaTSNP8227	101.5	-	-
2908	CaLG4	CaSNP2458	102.6	-	-
2909	CaLG4	CaSNP2845	102.6	-	-
2910	CaLG4	CaSNP2930	102.8	-	-
2911	CaLG4	CaSNP3753	102.9	-	-
2912	CaLG4	CaTMS738	103	-	-
2913	CaLG4	CaTSNP8414	103.3	-	-
2914	CaLG4	CaTSNP6261	103.3	scaffold00384	187713
2915	CaLG4	CaTSNP8677	103.4	-	-
2916	CaLG4	CaTSNP6058	103.4	-	-
2917	CaLG4	CaTSNP8319	103.4	-	-
2918	CaLG4	CaSNP2600	103.4	-	-
2919	CaLG4	CaTSNP7260	103.4	-	-
2920	CaLG4	CaTSNP7635	103.5	scaffold00686	120430
2921	CaLG4	CaSNP2184	103.5	-	-
2922	CaLG4	CaSNP2527	103.6	-	-
2923	CaLG4	CaSNP3771	103.6	-	-
2924	CaLG4	CaSNP3083	104	-	-

2925	CaLG4	CaTSNP6002	104.1	-	-
2926	CaLG4	CaSNP3029	104.2	scaffold02210	54732
2927	CaLG4	CaTSNP6439	104.2	scaffold00428	166793
2928	CaLG4	CaTSNP6634	104.2	scaffold00616	130510
2929	CaLG4	CaTSNP6694	104.2	scaffold01050	105447
2930	CaLG4	CaTSNP9054	104.2	-	-
2931	CaLG4	CaSNP4054	104.2	-	-
2932	CaLG4	CaTSNP7259	104.3	scaffold01973	48717
2933	CaLG4	CaSNP2384	104.3	-	-
2934	CaLG4	CaSNP2436	104.3	-	-
2935	CaLG4	CaSNP4258	104.3	-	-
2936	CaLG4	CaSNP2714	104.3	-	-
2937	CaLG4	CaTSNP7707	104.4	-	-
2938	CaLG4	CaSNP3953	104.4	-	-
2939	CaLG4	CaSNP757	104.7	9182752	38072
2940	CaLG4	CaSNP684	104.7	-	-
2941	CaLG4	CaSNP730	104.7	-	-
2942	CaLG4	CaSNP2879	104.7	-	-
2943	CaLG4	CaSNP806	104.8	-	-
2944	CaLG4	CaSNP26	104.8	scaffold00012	765023
2945	CaLG4	CaSNP618	104.8	scaffold00019	627903
2946	CaLG4	CaSNP3210	104.8	-	-
2947	CaLG4	CaSNP565	104.9	-	-
2948	CaLG4	CaSNP609	104.9	-	-
2949	CaLG4	CaSNP299	104.9	-	-
2950	CaLG4	CaSNP259	105	-	-
2951	CaLG4	CaSNP3824	105	scaffold04570	14930
2952	CaLG4	CaSNP168	105	-	-
2953	CaLG4	CaSNP3692	105	scaffold01003	93954

2954	CaLG4	CaSNP4985	105	-	-
2955	CaLG4	CaSNP4344	105.1	-	-
2956	CaLG4	CaSNP406	105.1	-	-
2957	CaLG4	CaSNP703	105.1	-	-
2958	CaLG4	CaSNP4816	105.1	-	-
2959	CaLG4	CaSNP384	105.2	-	-
2960	CaLG4	CaSNP262	105.2	-	-
2961	CaLG4	CaSNP699	105.2	scaffold00037	498102
2962	CaLG4	CaSNP4609	105.2	-	-
2963	CaLG4	CaSNP4534	105.3	-	-
2964	CaLG4	CaSNP2380	105.3	scaffold00115	325533
2965	CaLG4	CaSNP3103	105.4	-	-
2966	CaLG4	CaSNP4038	105.4	-	-
2967	CaLG4	CaSNP2799	105.5	scaffold02299	54340
2968	CaLG4	CaSNP286	105.6	scaffold00781	121170
2969	CaLG4	CaSNP3827	105.7	scaffold02250	41314
2970	CaLG4	CaSNP2889	105.8	9196384	21012
2971	CaLG4	CaSNP2994	105.9	-	-
2972	CaLG4	CaSNP2186	106	-	-
2973	CaLG4	CaSNP4752	106	-	-
2974	CaLG4	CaSNP3386	106.1	-	-
2975	CaLG4	CaSNP4079	106.1	-	-
2976	CaLG4	CaSNP1931	106.1	-	-
2977	CaLG4	CaSNP2598	106.1	-	-
2978	CaLG4	CaSNP2709	106.2	-	-
2979	CaLG4	CaTMS790	106.3	-	-
2980	CaLG4	CaTSNP6728	106.3	-	-
2981	CaLG4	CaGMS1310	106.3	-	-
2982	CaLG4	CaSNP2534	106.4	-	-

2983	CaLG4	CaSNP2065	106.4	-	-
2984	CaLG4	CaSNP3341	106.4	scaffold01533	62880
2985	CaLG4	CaSNP3607	106.4	-	-
2986	CaLG4	CaSNP185	106.4	-	-
2987	CaLG4	CaSNP2240	106.5	-	-
2988	CaLG4	CaTSNP7354	106.5	scaffold00493	174924
2989	CaLG4	CaGMS1303	106.6	-	-
2990	CaLG4	CaSNP530	106.6	-	-
2991	CaLG4	CaSNP2176	106.6	-	-
2992	CaLG4	CaTSNP8381	106.6	-	-
2993	CaLG4	CaTSNP8303	106.7	-	-
2994	CaLG4	CaTSNP6062	106.7	-	-
2995	CaLG4	CaTSNP8457	106.9	-	-
2996	CaLG4	CaTSNP7003	107.1	-	-
2997	CaLG4	CaTSNP7381	107.1	-	-
2998	CaLG4	CaTSNP7142	107.1	-	-
2999	CaLG4	CaTSNP8221	107.1	-	-
3000	CaLG4	CaTSNP7235	107.1	-	-
3001	CaLG4	CaTSNP8250	107.1	-	-
3002	CaLG4	CaTSNP6970	107.2	-	-
3003	CaLG4	CaSNP875	107.2	9199317	35847
3004	CaLG4	CaTSNP6023	107.3	-	-
3005	CaLG4	CaTSNP8814	107.3	-	-
3006	CaLG4	CaTSNP8656	107.3	-	-
3007	CaLG4	CaSNP3018	107.3	scaffold01786	58430
3008	CaLG4	CaTSNP6610	107.4	-	-
3009	CaLG4	CaTSNP7200	107.4	-	-
3010	CaLG4	CaTSNP7882	107.4	-	-
3011	CaLG4	CaTSNP8572	107.4	-	-

3012	CaLG4	CaTSNP6919	107.4	-	-
3013	CaLG4	CaTSNP9156	107.5	-	-
3014	CaLG4	CaTSNP6131	107.5	-	-
3015	CaLG4	CaTSNP6466	107.5	scaffold00707	113119
3016	CaLG4	CaTSNP8920	107.5	-	-
3017	CaLG4	CaTSNP6187	107.5	scaffold07836	6313
3018	CaLG4	CaTSNP6420	107.5	-	-
3019	CaLG4	CaTSNP8042	107.5	-	-
3020	CaLG4	CaTSNP8214	107.5	-	-
3021	CaLG4	CaTSNP8941	107.5	-	-
3022	CaLG4	CaTSNP7227	107.5	scaffold00663	125126
3023	CaLG4	CaTSNP7701	107.6	-	-
3024	CaLG4	CaTSNP7945	107.6	scaffold00312	196739
3025	CaLG4	CaTSNP6639	107.6	-	-
3026	CaLG4	CaTSNP7252	107.6	-	-
3027	CaLG4	CaTSNP7653	107.6	-	-
3028	CaLG4	CaTSNP8030	107.6	-	-
3029	CaLG4	CaTSNP7868	107.6	-	-
3030	CaLG4	CaTSNP9108	107.6	-	-
3031	CaLG4	CaTSNP7781	107.6	-	-
3032	CaLG4	CaTSNP6676	107.6	scaffold00126	310627
3033	CaLG4	CaTSNP8791	107.6	-	-
3034	CaLG4	CaSNP46	107.6	-	-
3035	CaLG4	CaSNP76	107.7	-	-
3036	CaLG4	CaTSNP6557	107.8	scaffold00544	177745
3037	CaLG4	CaTSNP6710	107.8	-	-
3038	CaLG4	CaTSNP8731	107.8	-	-
3039	CaLG4	CaTSNP6137	107.8	scaffold00277	198135
3040	CaLG4	CaTSNP6358	107.8	-	-

3041	CaLG4	CaTSNP8567	107.8	-	-
3042	CaLG4	CaTSNP8264	107.8	-	-
3043	CaLG4	CaTSNP7907	107.8	-	-
3044	CaLG4	CaTSNP6069	107.8	-	-
3045	CaLG4	CaTSNP8858	107.8	scaffold00351	199393
3046	CaLG4	CaTSNP7075	107.8	-	-
3047	CaLG4	CaTSNP6722	107.8	-	-
3048	CaLG4	CaTSNP8032	107.8	-	-
3049	CaLG4	CaSNP54	107.8	-	-
3050	CaLG4	CaTSNP6820	107.8	-	-
3051	CaLG4	CaTSNP7380	107.8	scaffold01111	82599
3052	CaLG4	CaSNP754	107.9	-	-
3053	CaLG4	CaTSNP6473	107.9	scaffold00100	344856
3054	CaLG4	CaTSNP6305	107.9	scaffold00895	102560
3055	CaLG4	CaTSNP6968	107.9	-	-
3056	CaLG4	CaTSNP7025	107.9	9203322	58589
3057	CaLG4	CaTSNP7584	107.9	-	-
3058	CaLG4	CaTSNP8840	107.9	-	-
3059	CaLG4	CaTSNP6363	107.9	scaffold00175	315488
3060	CaLG4	CaSNP908	107.9	-	-
3061	CaLG4	CaTSNP8746	107.9	-	-
3062	CaLG4	CaTSNP7560	107.9	-	-
3063	CaLG4	CaTSNP7908	107.9	-	-
3064	CaLG4	CaTSNP6232	108	-	-
3065	CaLG4	CaTSNP6011	108	9206718	49002
3066	CaLG4	CaTSNP6920	108	-	-
3067	CaLG4	CaTSNP7246	108	-	-
3068	CaLG4	CaTSNP8723	108	-	-
3069	CaLG4	CaTSNP6617	108	scaffold01585	77973

3070	CaLG4	CaTSNP6895	108	scaffold02487	60754
3071	CaLG4	CaTSNP7446	108	scaffold00860	128619
3072	CaLG4	CaTSNP7022	108.1	scaffold00178	267143
3073	CaLG4	CaTSNP7195	108.1	-	-
3074	CaLG4	CaTSNP7007	108.1	scaffold09211	4946
3075	CaLG4	CaTSNP6343	108.1	-	-
3076	CaLG4	CaTSNP7585	108.1	scaffold02912	38899
3077	CaLG4	CaTSNP6105	108.1	-	-
3078	CaLG4	CaTSNP7460	108.1	scaffold10814	3911
3079	CaLG4	CaTSNP7064	108.1	-	-
3080	CaLG4	CaTSNP6046	108.1	scaffold00234	232008
3081	CaLG4	CaTSNP7078	108.1	-	-
3082	CaLG4	CaTSNP8913	108.1	-	-
3083	CaLG4	CaSNP51	108.2	scaffold00744	108591
3084	CaLG4	CaTSNP7090	108.2	-	-
3085	CaLG4	CaTSNP6901	108.2	scaffold01229	81589
3086	CaLG4	CaTSNP6383	108.2	-	-
3087	CaLG4	CaSNP533	108.2	-	-
3088	CaLG4	CaTSNP6016	108.2	scaffold00659	129757
3089	CaLG4	CaTSNP8392	108.2	-	-
3090	CaLG4	CaTSNP6499	108.2	-	-
3091	CaLG4	CaTSNP6481	108.2	-	-
3092	CaLG4	CaTSNP8186	108.2	scaffold00666	126661
3093	CaLG4	CaTSNP7183	108.2	-	-
3094	CaLG4	CaSNP132	108.4	scaffold00196	240961
3095	CaLG4	CaTSNP6402	108.4	-	-
3096	CaLG4	CaTSNP8868	108.4	-	-
3097	CaLG4	CaTSNP6643	108.4	scaffold05492	20893
3098	CaLG4	CaTSNP8803	108.4	-	-

3099	CaLG4	CaTSNP8265	108.4	-	-
3100	CaLG4	CaTSNP7035	108.4	-	-
3101	CaLG4	CaTSNP8535	108.4	scaffold05161	12471
3102	CaLG4	CaTSNP8589	108.4	-	-
3103	CaLG4	CaTSNP8098	108.4	-	-
3104	CaLG4	CaTSNP8263	108.5	-	-
3105	CaLG4	CaTSNP8492	108.5	-	-
3106	CaLG4	CaTSNP8792	108.5	-	-
3107	CaLG4	CaTSNP7103	108.5	scaffold00863	110642
3108	CaLG4	CaTSNP7198	108.5	-	-
3109	CaLG4	CaTSNP6529	108.6	-	-
3110	CaLG4	CaSNP309	108.6	-	-
3111	CaLG4	CaSNP431	108.6	scaffold02503	36471
3112	CaLG4	CaSNP747	108.6	scaffold00087	350568
3113	CaLG4	CaSNP338	108.6	9182217	25467
3114	CaLG4	CaTSNP6972	108.7	-	-
3115	CaLG4	CaTSNP8741	108.8	-	-
3116	CaLG4	CaSNP777	108.8	9183775	25877
3117	CaLG4	CaTSNP8743	108.8	-	-
3118	CaLG4	CaTSNP8976	108.8	-	-
3119	CaLG4	CaTSNP8686	108.8	scaffold00103	309727
3120	CaLG4	CaTSNP9021	108.9	-	-
3121	CaLG4	CaTSNP6204	108.9	-	-
3122	CaLG4	CaTSNP6199	108.9	-	-
3123	CaLG4	CaTSNP6334	108.9	-	-
3124	CaLG4	CaTSNP9046	108.9	-	-
3125	CaLG4	CaSNP176	109.2	-	-
3126	CaLG4	CaSNP831	109.2	-	-
3127	CaLG4	CaSNP38	109.3	-	-

3128	CaLG4	CaSNP129	109.3	scaffold00476	150741
3129	CaLG4	ESNP24	109.4	-	-
3130	CaLG4	CaSNP99	109.4	-	-
3131	CaLG4	CaTSNP6052	109.5	9207820	96412
3132	CaLG4	CaSNP721	109.6	-	-
3133	CaLG4	CaSNP265	109.7	-	-
3134	CaLG4	CaTSNP8747	109.8	-	-
3135	CaLG4	CaGMS30	109.9	-	-
3136	CaLG4	CaTMS556	109.9	-	-
3137	CaLG4	CaSNP712	110	-	-
3138	CaLG4	CaSNP3729	110	-	-
3139	CaLG4	CaSNP693	110.2	-	-
3140	CaLG4	CaTSNP8700	110.5	-	-
3141	CaLG4	CaSNP189	110.5	-	-
3142	CaLG4	CaTSNP7587	110.5	-	-
3143	CaLG4	CaSNP158	110.5	-	-
3144	CaLG4	CaTSNP8612	110.6	9211061	39550
3145	CaLG4	CaSNP709	110.6	-	-
3146	CaLG4	CaSNP29	110.6	-	-
3147	CaLG4	CaSNP677	110.7	-	-
3148	CaLG4	CaSNP736	110.7	-	-
3149	CaLG4	CaSNP264	110.7	-	-
3150	CaLG4	CaSNP272	110.7	-	-
3151	CaLG4	CaSNP311	110.7	-	-
3152	CaLG4	CaSNP282	110.9	-	-
3153	CaLG4	CaSNP6	111	-	-
3154	CaLG4	ESNP5	111.1	-	-
3155	CaLG4	ESNP1	111.1	-	-
3156	CaLG4	CaTSNP7057	111.2	-	-

3157	CaLG4	CaSNP524	111.2	-	-
3158	CaLG4	CaSNP364	111.2	-	-
3159	CaLG4	CaTSNP8985	111.2	-	-
3160	CaLG4	CaTSNP7056	111.2	-	-
3161	CaLG4	CaTSNP7191	111.6	-	-
3162	CaLG4	CaTSNP7632	111.6	-	-
3163	CaLG4	CaTSNP7807	111.6	-	-
3164	CaLG4	CaSNP58	111.7	-	-
3165	CaLG4	CaSNP811	111.8	scaffold00179	250626
3166	CaLG4	CaSNP718	111.8	-	-
3167	CaLG4	CaSNP89	111.8	-	-
3168	CaLG4	CaSNP725	111.9	scaffold00117	304179
3169	CaLG4	CaSNP803	112.1	-	-
3170	CaLG4	CaSNP845	112.1	scaffold02265	44220
3171	CaLG4	CaSNP737	112.2	9122074	74661
3172	CaLG4	CaSNP877	112.2	-	-
3173	CaLG4	CaSNP418	112.2	-	-
3174	CaLG4	CaSNP241	112.3	-	-
3175	CaLG4	CaSNP407	112.3	scaffold00173	246289
3176	CaLG4	CaSNP134	112.5	-	-
3177	CaLG4	CaTSNP7879	112.5	-	-
3178	CaLG4	CaTSNP7039	112.5	-	-
3179	CaLG4	CaTSNP7670	112.8	-	-
3180	CaLG4	CaTSNP8074	113.5	scaffold00737	117360
3181	CaLG4	CaTSNP7202	113.5	-	-
3182	CaLG4	CaTSNP6224	113.5	-	-
3183	CaLG4	CaTSNP7796	113.5	-	-
3184	CaLG4	CaTSNP8837	113.5	9215262	28085
3185	CaLG4	CaTSNP6747	113.5	-	-

3186	CaLG4	CaTSNP6300	113.6	-	-
3187	CaLG4	CaTSNP7043	113.6	9116096	62036
3188	CaLG4	CaTSNP7096	113.6	-	-
3189	CaLG4	CaTSNP7732	113.6	-	-
3190	CaLG4	CaTSNP8084	113.6	-	-
3191	CaLG4	CaTSNP8522	113.6	-	-
3192	CaLG4	CaTSNP8999	113.6	-	-
3193	CaLG4	CaTSNP7250	113.6	-	-
3194	CaLG4	CaTSNP8398	113.6	-	-
3195	CaLG4	CaTSNP6678	113.6	-	-
3196	CaLG4	CaTSNP6682	113.6	-	-
3197	CaLG4	CaTSNP7476	113.6	-	-
3198	CaLG4	CaTSNP7340	113.6	-	-
3199	CaLG4	CaTSNP9086	113.6	-	-
3200	CaLG4	CaTSNP9082	113.7	-	-
3201	CaLG4	CaSNP5054	114.3	-	-
3202	CaLG4	CaGMS1240	114.4	-	-
3203	CaLG4	CaSNP1941	114.4	scaffold00390	191140
3204	CaLG4	CaSNP5130	114.4	9184070	27076
3205	CaLG4	CaSNP2328	114.6	-	-
3206	CaLG4	CaSNP3726	114.7	-	-
3207	CaLG4	CaSNP4089	115.2	-	-
3208	CaLG4	CaTMS751	115.7	-	-
3209	CaLG4	CaSNP2164	115.8	-	-
3210	CaLG4	CaSNP4055	115.9	-	-
3211	CaLG4	CaTSNP6295	115.9	-	-
3212	CaLG4	CaSNP3077	116	scaffold01819	55226
3213	CaLG4	CaTSNP9013	116	-	-
3214	CaLG4	CaTSNP8586	116.3	-	-

3215	CaLG4	CaTSNP8290	116.3	-	-
3216	CaLG4	CaTSNP8247	116.6	-	-
3217	CaLG4	CaSNP2806	116.8	-	-
3218	CaLG4	CaTSNP7109	116.9	-	-
3219	CaLG4	CaTSNP7148	117.3	-	-
3220	CaLG4	CaTSNP8249	117.3	-	-
3221	CaLG4	CaTSNP6094	117.3	-	-
3222	CaLG4	CaTSNP7278	117.3	-	-
3223	CaLG4	CaTSNP6526	117.6	-	-
3224	CaLG4	CaTSNP6887	117.6	-	-
3225	CaLG4	CaSNP2429	117.6	-	-
3226	CaLG4	CaSNP1935	117.7	scaffold06902	7580
3227	CaLG4	CaSNP3670	117.7	-	-
3228	CaLG4	CaTSNP7823	117.9	scaffold00442	164395
3229	CaLG4	CaTSNP7561	118	9185546	21887
3230	CaLG4	CaTSNP8820	118	-	-
3231	CaLG4	CaTSNP7605	118	-	-
3232	CaLG4	CaTSNP8736	118	-	-
3233	CaLG4	CaTSNP6570	118.1	-	-
3234	CaLG4	CaTSNP7661	118.1	-	-
3235	CaLG4	CaTSNP8729	118.1	-	-
3236	CaLG4	CaTSNP6609	118.1	-	-
3237	CaLG4	CaTSNP6718	118.2	-	-
3238	CaLG4	CaTSNP7516	118.3	-	-
3239	CaLG4	CaTSNP7551	118.3	-	-
3240	CaLG4	CaTSNP7221	118.5	-	-
3241	CaLG4	CaTSNP7348	118.5	scaffold00060	429712
3242	CaLG4	CaTSNP6564	118.5	-	-
3243	CaLG4	CaTSNP7290	118.5	-	-

3244	CaLG4	CaTSNP6528	118.5	-	-
3245	CaLG4	CaTSNP7731	118.5	-	-
3246	CaLG4	CaTSNP8968	118.6	-	-
3247	CaLG4	CaTSNP6583	118.6	-	-
3248	CaLG4	CaTSNP6808	118.6	-	-
3249	CaLG4	CaTSNP7449	118.6	scaffold00124	301320
3250	CaLG4	CaTSNP7688	118.6	-	-
3251	CaLG4	CaTSNP7832	118.6	-	-
3252	CaLG4	CaTSNP8149	118.6	-	-
3253	CaLG4	CaTSNP8520	118.6	-	-
3254	CaLG4	CaTSNP6659	118.6	-	-
3255	CaLG4	CaTSNP7425	118.6	-	-
3256	CaLG4	CaTSNP7436	118.6	-	-
3257	CaLG4	CaTSNP6107	118.7	-	-
3258	CaLG4	CaTSNP6218	118.7	-	-
3259	CaLG4	CaTSNP6139	118.7	-	-
3260	CaLG4	CaTSNP6017	118.7	-	-
3261	CaLG4	CaTSNP7363	118.7	-	-
3262	CaLG4	CaTSNP6802	118.7	-	-
3263	CaLG4	CaTSNP7933	118.7	-	-
3264	CaLG4	CaTSNP6372	118.7	9199740	31695
3265	CaLG4	CaTSNP6312	118.7	-	-
3266	CaLG4	CaTSNP6301	118.7	-	-
3267	CaLG4	CaTSNP8453	118.7	-	-
3268	CaLG4	CaTSNP8605	118.7	9137468	59730
3269	CaLG4	CaTSNP9152	118.7	9203698	36041
3270	CaLG4	CaTSNP8548	118.8	9120772	14918
3271	CaLG4	CaTSNP7804	118.8	-	-
3272	CaLG4	CaTSNP8270	118.8	-	-

3273	CaLG4	CaTSNP7619	118.8	-	-
3274	CaLG4	CaTSNP8235	118.8	-	-
3275	CaLG4	CaTSNP7311	118.8	scaffold00021	622492
3276	CaLG4	CaTSNP6671	118.8	scaffold01573	68081
3277	CaLG4	CaTSNP6888	118.8	-	-
3278	CaLG4	CaTSNP7430	118.8	-	-
3279	CaLG4	CaTSNP7611	118.9	-	-
3280	CaLG4	CaTSNP7783	118.9	scaffold06182	19490
3281	CaLG4	CaTSNP8276	118.9	9204203	31447
3282	CaLG4	CaTSNP6568	118.9	scaffold00053	455825
3283	CaLG4	CaTSNP7450	118.9	scaffold00097	338808
3284	CaLG4	CaTSNP7495	118.9	-	-
3285	CaLG4	CaTSNP7422	118.9	-	-
3286	CaLG4	CaTSNP7987	118.9	scaffold01125	96726
3287	CaLG4	CaTSNP8783	118.9	scaffold00537	176994
3288	CaLG4	CaSNP3795	119	scaffold06897	8378
3289	CaLG4	CaSNP1945	119.6	-	-
3290	CaLG4	CaSNP2404	120.1	-	-
3291	CaLG4	CaSNP4501	120.4	-	-
3292	CaLG4	CaSNP3453	120.5	-	-
3293	CaLG4	CaSNP3928	120.5	scaffold05404	18201
3294	CaLG4	CaSNP5082	120.6	-	-
3295	CaLG4	CaSNP2342	120.8	-	-
3296	CaLG4	CaSNP1922	121.6	-	-
3297	CaLG4	CaTMS980	123	-	-
3298	CaLG4	CaTMS574	123.4	-	-
3299	CaLG4	CaTMS785	125.4	-	-
3300	CaLG4	NCPGR21	125.6	scaffold00088	377425
3301	CaLG4	CaTMS1117	126.4	-	-

3302	CaLG4	CaTMS1059	129.8	-	-
3303	CaLG4	CaGMS1136	129.8	-	-
3304	CaLG4	CaTMS1005	129.8	-	-
3305	CaLG4	CaTMS877	129.8	-	-
3306	CaLG4	CaGMS1134	129.8	-	-
3307	CaLG4	CaGMS45	129.8	-	-
3308	CaLG4	CaTMS1015	129.8	-	-
3309	CaLG4	CaTMS852	129.8	-	-
3310	CaLG4	CaTMS926	129.8	-	-
3311	CaLG4	CaTMS995	129.8	-	-
3312	CaLG4	CaGMS1253	129.9	-	-
3313	CaLG4	CaTMS1101	129.9	-	-
3314	CaLG4	CaTMS613	129.9	-	-
3315	CaLG4	CaTMS639	129.9	-	-
3316	CaLG4	CaTMS756	129.9	-	-
3317	CaLG4	CaTMS895	129.9	-	-
3318	CaLG4	CaTMS889	129.9	-	-
3319	CaLG4	CaGMS1282	129.9	-	-
3320	CaLG4	CaTMS591	129.9	-	-
3321	CaLG4	CaTMS642	129.9	-	-
3322	CaLG4	CaTMS924	129.9	-	-
3323	CaLG4	CaTMS1113	130.4	-	-
3324	CaLG4	CaGMS1169	130.5	-	-
3325	CaLG4	NCPGR22	131.9	-	-
3326	CaLG4	CaTMS1006	132.5	-	-
3327	CaLG4	CaTMS770	132.5	-	-
3328	CaLG4	NCPGR140	134.4	scaffold02879	30893
3329	CaLG4	CaTMS683	136.2	-	-
3330	CaLG4	CaTMS991	136.9	-	-

3331	CaLG4	CEST106	137.3	-	-
3332	CaLG4	CaGMS1139	137.8	-	-
3333	CaLG4	CaTMS1087	138.4	-	-
3334	CaLG4	CEST109	140	scaffold00454	165732
3335	CaLG4	CESSR173	140.3	scaffold03737	20756
3336	CaLG4	CESSR122	142.3	-	-
3337	CaLG4	CaTMS644	143.2	-	-
3338	CaLG4	PIP186	146.5	-	-
3339	CaLG4	CESSR141	148.8	scaffold00062	433485
3340	CaLG5	NCPGR273	0	scaffold06259	8918
3341	CaLG5	CaTMS679	1.401	-	-
3342	CaLG5	CaTMS854	2.606	-	-
3343	CaLG5	CaGMS1172	9.877	-	-
3344	CaLG5	CaTMS972	10.33	-	-
3345	CaLG5	CaTMS1012	10.65	-	-
3346	CaLG5	CaTMS617	10.99	-	-
3347	CaLG5	CaTMS730	12.4	-	-
3348	CaLG5	CaTMS894	12.4	-	-
3349	CaLG5	CaTMS848	12.42	-	-
3350	CaLG5	CaTMS780	14.18	-	-
3351	CaLG5	CaTMS984	15	-	-
3352	CaLG5	CaTMS973	15.21	-	-
3353	CaLG5	CaTMS966	15.26	-	-
3354	CaLG5	CaTMS655	16.42	-	-
3355	CaLG5	CaTMS806	16.42	-	-
3356	CaLG5	CaTMS619	16.57	-	-
3357	CaLG5	CaTMS958	16.66	-	-
3358	CaLG5	CaTMS577	16.91	-	-
3359	CaLG5	NCPGR183	19.48	scaffold08178	5962

3360	CaLG5	CaTMS657	20.28	-	-
3361	CaLG5	CaGMS1261	22	-	-
3362	CaLG5	CaTMS1008	22.01	-	-
3363	CaLG5	CaTMS887	22.51	-	-
3364	CaLG5	CaTMS636	22.82	-	-
3365	CaLG5	CaTMS987	23.47	-	-
3366	CaLG5	PIP195	23.6	-	-
3367	CaLG5	CaTMS614	24.04	-	-
3368	CaLG5	CaGMS6	24.09	-	-
3369	CaLG5	CaTMS554	24.97	-	-
3370	CaLG5	CaTMS725	25.11	-	-
3371	CaLG5	CaTMS710	25.67	-	-
3372	CaLG5	CaTMS649	27.02	-	-
3373	CaLG5	CaTMS1065	27.27	-	-
3374	CaLG5	CaTMS621	27.27	-	-
3375	CaLG5	CaTMS1086	27.36	-	-
3376	CaLG5	CaGMS1168	27.98	-	-
3377	CaLG5	CaGMS1294	27.99	-	-
3378	CaLG5	CaGMS1324	28.04	-	-
3379	CaLG5	CaTMS660	28.14	-	-
3380	CaLG5	CaTMS567	28.15	-	-
3381	CaLG5	CaTMS827	28.22	-	-
3382	CaLG5	CaTMS996	28.42	-	-
3383	CaLG5	CaTMS1060	28.91	-	-
3384	CaLG5	CaTMS739	29.24	-	-
3385	CaLG5	CaTMS1121	30.36	-	-
3386	CaLG5	CEST97	32.66	-	-
3387	CaLG5	CaGMS1201	34.87	-	-
3388	CaLG5	CaTMS563	35.06	-	-

3389	CaLG5	NCPGR53	37.16	-	-
3390	CaLG5	CaSNP4827	37.5	-	-
3391	CaLG5	NCPGR153	37.85	scaffold02988	41961
3392	CaLG5	TR29	40.49	-	-
3393	CaLG5	CaTMS913	43.08	-	-
3394	CaLG5	TA39	43.53	-	-
3395	CaLG5	GAA42	44.45	-	-
3396	CaLG5	CEST166	44.94	-	-
3397	CaLG5	TA71	45.86	-	-
3398	CaLG5	STMS7	47.17	-	-
3399	CaLG5	TR59	47.46	-	-
3400	CaLG5	GA4	47.54	-	-
3401	CaLG5	TS53	47.67	-	-
3402	CaLG5	TA179	47.82	-	-
3403	CaLG5	TAAS	47.87	-	-
3404	CaLG5	TS43	48.53	-	-
3405	CaLG5	NCPGR182	50.37	-	-
3406	CaLG5	CEST62	51.74	-	-
3407	CaLG5	NCPGR189	52.11	-	-
3408	CaLG5	NCPGR252	52.21	-	-
3409	CaLG5	NCPGR145	52.25	-	-
3410	CaLG5	NCPGR105	52.33	scaffold06999	7409
3411	CaLG5	H2L102	52.62	-	-
3412	CaLG5	NCPGR5	52.91	-	-
3413	CaLG5	NCPGR210	52.94	scaffold00890	114165
3414	CaLG5	CEST95	53.97	-	-
3415	CaLG5	CaGMS16	54.78	-	-
3416	CaLG5	CaSNP3946	54.9	scaffold01232	80047
3417	CaLG5	CaSNP3942	55.22	scaffold02123	62695

3418	CaLG5	CaTSNP7801	55.3	scaffold01362	76560
3419	CaLG5	CaTSNP8676	55.3	-	-
3420	CaLG5	CaTSNP7477	55.32	scaffold00564	131496
3421	CaLG5	CaTSNP7199	55.33	scaffold00664	127060
3422	CaLG5	CaSNP3299	55.35	scaffold01332	72946
3423	CaLG5	CaSNP2118	55.37	scaffold00279	212657
3424	CaLG5	CaSNP4565	55.38	-	-
3425	CaLG5	CaSNP4071	55.41	scaffold02292	43499
3426	CaLG5	CaSNP2640	55.42	-	-
3427	CaLG5	CaSNP3088	55.43	scaffold02650	34170
3428	CaLG5	CaSNP3245	55.43	scaffold00881	117405
3429	CaLG5	CaSNP4978	55.43	-	-
3430	CaLG5	CaSNP5069	55.43	scaffold01704	71142
3431	CaLG5	CaSNP2316	55.45	scaffold02596	41685
3432	CaLG5	CaSNP2723	55.47	-	-
3433	CaLG5	CaSNP4630	55.48	scaffold03292	25891
3434	CaLG5	CaSNP3954	55.48	scaffold07622	6544
3435	CaLG5	CaSNP3765	55.48	scaffold02008	52365
3436	CaLG5	CaSNP2091	55.48	scaffold01213	87123
3437	CaLG5	CaSNP3547	55.48	scaffold02150	43831
3438	CaLG5	NCPGR217	55.48	-	-
3439	CaLG5	CaSNP4544	55.49	scaffold00652	128990
3440	CaLG5	CaTSNP8753	55.49	scaffold01852	63686
3441	CaLG5	CaSNP3624	55.5	scaffold02270	44363
3442	CaLG5	CaTSNP7132	55.5	-	-
3443	CaLG5	CaSNP2481	55.5	scaffold01024	94833
3444	CaLG5	CaTSNP7368	55.51	9109392	20356
3445	CaLG5	CaTSNP8395	55.52	-	-
3446	CaLG5	CaTSNP8709	55.52	9201121	40449

3447	CaLG5	CaSNP4841	55.52	scaffold00607	137883
3448	CaLG5	CaTSNP6773	55.52	-	-
3449	CaLG5	CaSNP5074	55.53	scaffold01746	70302
3450	CaLG5	CaTSNP6057	55.53	scaffold00317	201238
3451	CaLG5	CaSNP1985	55.53	scaffold00437	164627
3452	CaLG5	CaSNP2893	55.53	scaffold00820	115864
3453	CaLG5	CaTSNP7962	55.54	scaffold00555	144370
3454	CaLG5	CaSNP2379	55.54	9207652	27057
3455	CaLG5	CaSNP4773	55.55	-	-
3456	CaLG5	CaSNP2272	55.55	scaffold00359	195152
3457	CaLG5	CaSNP2209	55.55	scaffold00643	129241
3458	CaLG5	CaSNP3534	55.56	-	-
3459	CaLG5	CaSNP4005	55.56	9215227	26668
3460	CaLG5	CaTSNP6389	55.57	9211460	14145
3461	CaLG5	CaSNP2946	55.57	scaffold01234	95945
3462	CaLG5	CaSNP5030	55.57	-	-
3463	CaLG5	CaSNP2375	55.57	9210526	2788
3464	CaLG5	CaTSNP6508	55.57	-	-
3465	CaLG5	CaTSNP6660	55.57	-	-
3466	CaLG5	CaTSNP7174	55.57	scaffold00246	263909
3467	CaLG5	CaTSNP6051	55.57	scaffold01615	63625
3468	CaLG5	CaTSNP9083	55.57	-	-
3469	CaLG5	CaSNP3474	55.58	scaffold05996	9617
3470	CaLG5	CaTSNP8374	55.58	-	-
3471	CaLG5	CaSNP2466	55.59	-	-
3472	CaLG5	CaSNP3912	55.59	scaffold01317	74869
3473	CaLG5	CaTSNP6881	55.59	-	-
3474	CaLG5	CaTSNP7286	55.59	-	-
3475	CaLG5	CaTSNP7792	55.59	-	-

3476	CaLG5	CaTSNP7471	55.59	-	-
3477	CaLG5	CaTSNP7541	55.59	scaffold01566	89714
3478	CaLG5	CaTSNP7776	55.59	-	-
3479	CaLG5	CaTSNP7212	55.61	scaffold04410	16715
3480	CaLG5	CaTSNP8010	55.61	scaffold01102	87182
3481	CaLG5	CaTSNP8624	55.61	scaffold01705	84868
3482	CaLG5	CaSNP2371	55.61	-	-
3483	CaLG5	CaSNP4116	55.61	-	-
3484	CaLG5	CaTSNP6122	55.63	-	-
3485	CaLG5	CaSNP2616	55.63	scaffold05929	11571
3486	CaLG5	CaSNP2694	55.63	scaffold01666	63947
3487	CaLG5	CaSNP4498	55.65	scaffold06276	8871
3488	CaLG5	CaSNP3452	55.69	9209764	10883
3489	CaLG5	CaSNP2356	55.71	scaffold01329	80420
3490	CaLG5	CaSNP2464	55.71	-	-
3491	CaLG5	CaSNP2597	55.71	-	-
3492	CaLG5	CaSNP3230	55.71	-	-
3493	CaLG5	CaSNP3612	55.71	scaffold00455	165255
3494	CaLG5	CaSNP4700	55.71	scaffold01224	93640
3495	CaLG5	CaSNP4924	55.71	-	-
3496	CaLG5	CaSNP5000	55.71	-	-
3497	CaLG5	CaSNP2945	55.72	-	-
3498	CaLG5	CaSNP2972	55.72	scaffold01514	75327
3499	CaLG5	CaSNP3563	55.72	9214700	1766
3500	CaLG5	CaSNP3297	55.73	-	-
3501	CaLG5	CaSNP4892	55.75	scaffold01917	63797
3502	CaLG5	CaSNP1991	55.87	scaffold00780	125583
3503	CaLG5	CaSNP2217	55.88	scaffold01299	89230
3504	CaLG5	CaSNP2439	55.89	-	-

3505	CaLG5	CaSNP5102	55.89	scaffold01407	130301
3506	CaLG5	CaSNP4342	55.9	9216007	30322
3507	CaLG5	CaSNP2512	55.91	scaffold01808	57909
3508	CaLG5	CaSNP4727	55.91	scaffold01517	90887
3509	CaLG5	CaSNP3718	55.92	scaffold00726	137134
3510	CaLG5	CaSNP3536	55.92	scaffold00674	153405
3511	CaLG5	CaSNP2035	55.93	scaffold00500	150714
3512	CaLG5	CaSNP4837	55.93	scaffold00610	158394
3513	CaLG5	CaSNP5104	55.93	scaffold01558	83978
3514	CaLG5	CaSNP3318	55.94	scaffold00191	286837
3515	CaLG5	CaSNP3332	55.94	-	-
3516	CaLG5	CaSNP4736	55.94	scaffold01142	85865
3517	CaLG5	CaSNP2066	55.94	-	-
3518	CaLG5	CaSNP2352	55.94	scaffold00337	243351
3519	CaLG5	CaSNP3131	55.94	scaffold05135	12262
3520	CaLG5	CaSNP4111	55.94	-	-
3521	CaLG5	CaSNP4772	55.94	-	-
3522	CaLG5	CaSNP4967	55.94	-	-
3523	CaLG5	CaSNP3722	55.95	scaffold00803	117279
3524	CaLG5	CaTSNP6347	55.95	-	-
3525	CaLG5	CaSNP4180	55.96	9205252	22319
3526	CaLG5	CaSNP4475	55.96	scaffold01456	82924
3527	CaLG5	CaTSNP6304	55.96	-	-
3528	CaLG5	CaSNP2998	55.96	scaffold04335	17839
3529	CaLG5	CaTSNP6960	55.98	-	-
3530	CaLG5	CaTSNP7239	55.98	-	-
3531	CaLG5	CaTSNP8699	55.98	scaffold02133	71635
3532	CaLG5	CaTSNP6719	55.98	scaffold02725	34695
3533	CaLG5	CaTSNP7225	55.99	scaffold01703	57781

3534	CaLG5	CaTSNP6240	55.99	scaffold01091	110074
3535	CaLG5	CaSNP4874	55.99	scaffold03187	26858
3536	CaLG5	CaTSNP8847	56	scaffold00994	130394
3537	CaLG5	CaSNP3597	56	scaffold01859	52757
3538	CaLG5	CaSNP3759	56	scaffold02173	52477
3539	CaLG5	CaSNP2953	56.02	scaffold00846	132350
3540	CaLG5	CaSNP2987	56.02	9212278	12457
3541	CaLG5	CaSNP2189	56.03	9211811	25489
3542	CaLG5	CaSNP2359	56.03	scaffold04248	16951
3543	CaLG5	CaSNP3108	56.03	scaffold00728	145683
3544	CaLG5	CaSNP4385	56.03	scaffold02907	32493
3545	CaLG5	CaSNP4398	56.03	scaffold01759	53613
3546	CaLG5	CaSNP4331	56.04	scaffold02374	40791
3547	CaLG5	CaSNP4408	56.05	9213555	10722
3548	CaLG5	CaSNP2153	56.06	scaffold03498	32874
3549	CaLG5	CaSNP2990	56.06	scaffold02726	35324
3550	CaLG5	CaSNP3063	56.06	scaffold01909	59657
3551	CaLG5	CaSNP4766	56.06	-	-
3552	CaLG5	CaSNP3475	56.07	scaffold01662	72734
3553	CaLG5	CaSNP4750	56.07	scaffold03912	26249
3554	CaLG5	CaSNP3356	56.07	scaffold01490	72093
3555	CaLG5	CaSNP3149	56.09	9211123	36791
3556	CaLG5	CaSNP3811	56.09	scaffold02684	33931
3557	CaLG5	CaSNP4210	56.11	scaffold02781	36297
3558	CaLG5	CaSNP4662	56.11	scaffold02415	45099
3559	CaLG5	CaSNP3451	56.12	scaffold00658	155112
3560	CaLG5	CaSNP4194	56.12	scaffold02861	34145
3561	CaLG5	CaSNP4871	56.12	scaffold14115	2619
3562	CaLG5	CaSNP4901	56.13	scaffold01773	59736

3563	CaLG5	CaSNP4429	56.13	scaffold03672	27955
3564	CaLG5	CaSNP2628	56.14	-	-
3565	CaLG5	CaSNP3336	56.14	scaffold01536	74125
3566	CaLG5	CaSNP3339	56.14	scaffold01652	66193
3567	CaLG5	CaSNP3491	56.14	-	-
3568	CaLG5	CaSNP3493	56.14	scaffold02441	47254
3569	CaLG5	CaSNP3581	56.14	scaffold00796	129315
3570	CaLG5	CaSNP3806	56.14	scaffold01643	62918
3571	CaLG5	CaSNP3902	56.14	scaffold00709	124756
3572	CaLG5	CaSNP4730	56.14	scaffold02198	43094
3573	CaLG5	CaSNP4757	56.14	scaffold00496	179197
3574	CaLG5	CaSNP4907	56.14	scaffold03266	27358
3575	CaLG5	CaSNP2955	56.15	scaffold01756	60789
3576	CaLG5	CaSNP3948	56.15	scaffold02530	36421
3577	CaLG5	CaSNP3342	56.17	scaffold02254	45432
3578	CaLG5	CaSNP3459	56.19	scaffold00734	128030
3579	CaLG5	CaSNP4315	56.21	scaffold03739	21678
3580	CaLG5	CaSNP3331	56.22	scaffold01017	114281
3581	CaLG5	CaSNP4648	56.22	scaffold02686	34706
3582	CaLG5	CaSNP3264	56.23	scaffold01349	95243
3583	CaLG5	CaSNP3280	56.23	scaffold01248	93683
3584	CaLG5	CaSNP4189	56.23	9215936	14544
3585	CaLG5	CaSNP3747	56.24	scaffold03563	34441
3586	CaLG5	CaTSNP6983	56.24	scaffold01156	79291
3587	CaLG5	CaSNP3892	56.24	scaffold01305	86771
3588	CaLG5	CaSNP4169	56.24	scaffold03443	24644
3589	CaLG5	CaSNP4711	56.24	scaffold10004	4411
3590	CaLG5	CaSNP2383	56.24	scaffold02491	41788
3591	CaLG5	CaSNP2922	56.24	scaffold05640	10507

3592	CaLG5	CaSNP4106	56.24	-	-
3593	CaLG5	CaSNP3375	56.24	scaffold01916	56561
3594	CaLG5	CaSNP3314	56.24	scaffold02180	46004
3595	CaLG5	CaSNP3403	56.24	scaffold02979	37446
3596	CaLG5	CaSNP3675	56.24	scaffold02073	48816
3597	CaLG5	CaSNP4134	56.24	scaffold01461	90079
3598	CaLG5	CaSNP4413	56.24	scaffold01128	96581
3599	CaLG5	CaSNP4578	56.24	scaffold01967	78173
3600	CaLG5	CaSNP4771	56.24	scaffold02512	55672
3601	CaLG5	CaSNP4792	56.24	scaffold03512	29881
3602	CaLG5	CaSNP4834	56.24	scaffold02263	59601
3603	CaLG5	CaSNP3614	56.24	scaffold02407	54798
3604	CaLG5	CaSNP2366	56.25	9130454	10578
3605	CaLG5	CaTSNP6962	56.25	-	-
3606	CaLG5	CaTSNP7062	56.25	-	-
3607	CaLG5	CaSNP2488	56.25	scaffold00584	137328
3608	CaLG5	CaSNP2667	56.25	9204993	32245
3609	CaLG5	CaSNP3668	56.25	scaffold01757	69425
3610	CaLG5	CaSNP4139	56.25	scaffold02649	35515
3611	CaLG5	CaSNP4552	56.25	scaffold02858	32378
3612	CaLG5	CaSNP4583	56.25	scaffold03330	42457
3613	CaLG5	CaSNP4780	56.25	scaffold03368	31005
3614	CaLG5	CaSNP4801	56.25	scaffold01135	90769
3615	CaLG5	CaSNP3106	56.25	scaffold01889	72690
3616	CaLG5	CaSNP3750	56.25	scaffold01034	111770
3617	CaLG5	CaSNP4653	56.25	scaffold03337	31356
3618	CaLG5	CaSNP4797	56.25	scaffold02327	50412
3619	CaLG5	CaSNP5144	56.25	scaffold01943	56506
3620	CaLG5	CaSNP2151	56.26	-	-

3621	CaLG5	CaSNP2612	56.26	scaffold01474	80788
3622	CaLG5	CaSNP3163	56.26	-	-
3623	CaLG5	CaSNP3907	56.26	9204181	15190
3624	CaLG5	CaSNP4505	56.26	scaffold02006	59869
3625	CaLG5	CaSNP4511	56.26	scaffold02714	42163
3626	CaLG5	CaSNP4582	56.26	scaffold01726	65655
3627	CaLG5	CaSNP2030	56.26	scaffold02720	33951
3628	CaLG5	CaTSNP7237	56.26	-	-
3629	CaLG5	CaTSNP8005	56.27	scaffold01799	56975
3630	CaLG5	CaSNP2483	56.27	-	-
3631	CaLG5	CaSNP3346	56.28	scaffold00335	195572
3632	CaLG5	CaSNP4886	56.28	-	-
3633	CaLG5	CaSNP2843	56.28	-	-
3634	CaLG5	CaSNP4569	56.28	scaffold01483	72773
3635	CaLG5	CaSNP4394	56.28	scaffold02865	33093
3636	CaLG5	CaTSNP8876	56.29	-	-
3637	CaLG5	CaSNP3021	56.29	scaffold02566	56460
3638	CaLG5	CaSNP3224	56.29	scaffold02416	49201
3639	CaLG5	CaSNP4493	56.29	9215657	40411
3640	CaLG5	CaSNP1986	56.3	scaffold01380	79353
3641	CaLG5	CaSNP4114	56.3	-	-
3642	CaLG5	CaSNP3727	56.3	scaffold03009	42739
3643	CaLG5	CaSNP4250	56.3	scaffold10767	3937
3644	CaLG5	CaSNP3190	56.3	-	-
3645	CaLG5	CaSNP2264	56.31	9213860	22973
3646	CaLG5	CaSNP2732	56.31	scaffold01640	65740
3647	CaLG5	CaSNP3755	56.31	scaffold02986	41601
3648	CaLG5	CaSNP2410	56.31	scaffold00615	129503
3649	CaLG5	CaSNP4148	56.32	scaffold00920	101868

3650	CaLG5	CaSNP2760	56.32	scaffold00648	120759
3651	CaLG5	CaSNP3952	56.32	scaffold02440	40205
3652	CaLG5	CaSNP4952	56.32	scaffold01968	55824
3653	CaLG5	CaSNP4065	56.33	scaffold01160	104605
3654	CaLG5	CaSNP4435	56.33	scaffold00838	129404
3655	CaLG5	CaSNP2508	56.34	scaffold00276	210138
3656	CaLG5	CaSNP2674	56.34	scaffold01535	72354
3657	CaLG5	CaSNP3265	56.34	scaffold04474	25958
3658	CaLG5	CaSNP3803	56.34	scaffold01668	77830
3659	CaLG5	CaSNP3991	56.34	scaffold01597	69188
3660	CaLG5	CaSNP4185	56.34	-	-
3661	CaLG5	CaSNP4795	56.34	contig59815	1239
3662	CaLG5	CaSNP3119	56.36	scaffold01107	119161
3663	CaLG5	CaSNP4462	56.36	scaffold02395	53038
3664	CaLG5	CaSNP4338	56.38	scaffold03898	26362
3665	CaLG5	CaSNP2692	56.4	scaffold01861	75880
3666	CaLG5	CaSNP4476	56.4	9208013	24369
3667	CaLG5	CaTSNP7680	56.41	scaffold01884	50209
3668	CaLG5	CaSNP4671	56.57	scaffold02385	53516
3669	CaLG5	CaGMS1137	56.89	9102319	14129
3670	CaLG5	CaGMS42	57	scaffold00204	254094
3671	CaLG5	CaSNP2295	57.36	-	-
3672	CaLG5	CaSNP2639	57.39	scaffold00294	236301
3673	CaLG5	CaSNP5087	57.39	-	-
3674	CaLG5	CaSNP4905	57.45	scaffold00313	217546
3675	CaLG5	CaSNP4913	57.45	scaffold02404	56926
3676	CaLG5	PIP153	57.46	-	-
3677	CaLG5	ESNP98	57.83	9101287	21257
3678	CaLG5	CaSNP385	57.83	-	-

3679	CaLG5	CaSNP720	57.84	-	-
3680	CaLG5	CaSNP762	57.84	-	-
3681	CaLG5	CaSNP261	57.84	scaffold01270	92221
3682	CaLG5	CaSNP582	57.84	-	-
3683	CaLG5	CaSNP72	57.86	scaffold01690	64508
3684	CaLG5	CaSNP839	57.86	-	-
3685	CaLG5	CaSNP4637	57.98	-	-
3686	CaLG5	CaGMS18	58.06	-	-
3687	CaLG5	CaSNP2298	58.09	scaffold00671	127052
3688	CaLG5	CaSNP2871	58.09	-	-
3689	CaLG5	CaSNP2874	58.09	-	-
3690	CaLG5	CaSNP4603	58.09	-	-
3691	CaLG5	CaSNP3567	58.1	scaffold05461	11025
3692	CaLG5	CaSNP4457	58.11	-	-
3693	CaLG5	CaSNP2624	58.14	-	-
3694	CaLG5	CaSNP1978	58.14	-	-
3695	CaLG5	CaSNP4307	58.14	scaffold01295	101017
3696	CaLG5	CaSNP2724	58.17	scaffold00940	115658
3697	CaLG5	CaSNP3939	58.17	scaffold01601	67741
3698	CaLG5	CaSNP2860	58.17	scaffold02699	51909
3699	CaLG5	CaSNP3222	58.17	scaffold01863	51775
3700	CaLG5	CaSNP4868	58.18	scaffold03030	37926
3701	CaLG5	CaSNP4057	58.18	scaffold03729	21523
3702	CaLG5	CaSNP727	58.19	-	-
3703	CaLG5	CaSNP4322	58.19	scaffold02789	32472
3704	CaLG5	CaSNP3075	58.2	scaffold06609	8329
3705	CaLG5	CaSNP3517	58.21	scaffold00903	110559
3706	CaLG5	CaSNP506	58.21	scaffold00834	109949
3707	CaLG5	CaSNP2870	58.21	9207042	31400

3708	CaLG5	CaSNP2261	58.23	9208920	49593
3709	CaLG5	CaSNP913	58.23	9200975	31713
3710	CaLG5	CaSNP2252	58.24	scaffold00542	161261
3711	CaLG5	CaTSNP8758	58.25	9215521	12876
3712	CaLG5	CaSNP4029	58.25	scaffold01014	122073
3713	CaLG5	CaSNP4453	58.25	9207303	15899
3714	CaLG5	CaTSNP7642	58.26	scaffold01104	84396
3715	CaLG5	CaTSNP7318	58.27	scaffold01398	75778
3716	CaLG5	CaTSNP6595	58.28	scaffold01675	69009
3717	CaLG5	CaTSNP7177	58.29	-	-
3718	CaLG5	CaTSNP6531	58.29	scaffold01153	94236
3719	CaLG5	CaTSNP7087	58.29	scaffold01108	99643
3720	CaLG5	CaTSNP7241	58.29	-	-
3721	CaLG5	CaTSNP8618	58.31	scaffold02336	39532
3722	CaLG5	CaSNP2274	58.36	scaffold01336	93355
3723	CaLG5	CaSNP4112	58.36	9207508	14960
3724	CaLG5	CaTSNP6090	58.67	-	-
3725	CaLG5	CaSNP2074	58.89	scaffold00198	250757
3726	CaLG5	CaSNP2793	58.89	-	-
3727	CaLG5	CaSNP2877	58.9	-	-
3728	CaLG5	CaSNP3069	58.9	scaffold00717	135279
3729	CaLG5	CaSNP4988	58.9	scaffold01837	52355
3730	CaLG5	CaSNP4100	58.91	scaffold00746	108567
3731	CaLG5	CaSNP551	58.97	scaffold00171	292705
3732	CaLG5	CaTSNP8893	59	scaffold00909	103845
3733	CaLG5	CaSNP2837	59.05	-	-
3734	CaLG5	CaSNP292	59.15	-	-
3735	CaLG5	CaSNP448	59.15	scaffold01149	100809
3736	CaLG5	CaSNP503	59.15	-	-

3737	CaLG5	CaSNP687	59.17	-	-
3738	CaLG5	CaSNP2158	59.3	scaffold00789	105532
3739	CaLG5	CaSNP2293	59.35	scaffold00348	191539
3740	CaLG5	CaSNP2362	59.35	-	-
3741	CaLG5	CaTSNP7705	59.36	scaffold01987	49981
3742	CaLG5	CaTSNP8121	59.37	-	-
3743	CaLG5	CaTSNP6081	59.39	-	-
3744	CaLG5	CaTSNP6077	59.4	-	-
3745	CaLG5	CaSNP2082	59.4	-	-
3746	CaLG5	CaSNP2218	59.41	scaffold02122	48612
3747	CaLG5	CaTSNP6146	59.41	-	-
3748	CaLG5	CaSNP2887	59.47	scaffold00161	288845
3749	CaLG5	CaSNP4533	59.52	scaffold01769	56755
3750	CaLG5	ESNP73	59.61	-	-
3751	CaLG5	CaSNP557	59.7	-	-
3752	CaLG5	CaSNP330	59.86	-	-
3753	CaLG5	CaTSNP8613	59.87	scaffold01707	58041
3754	CaLG5	CaTSNP6331	59.92	-	-
3755	CaLG5	CaTSNP6755	59.92	-	-
3756	CaLG5	CaTSNP7036	59.92	-	-
3757	CaLG5	CaTMS1055	59.95	-	-
3758	CaLG5	CaSNP5090	59.97	638600	19503
3759	CaLG5	CaSNP2965	60.09	scaffold01449	88200
3760	CaLG5	CaSNP5097	60.09	scaffold06950	7491
3761	CaLG5	CaSNP4559	60.11	scaffold03905	27351
3762	CaLG5	CaSNP2855	60.11	-	-
3763	CaLG5	CaSNP2198	60.17	-	-
3764	CaLG5	CaTSNP6270	60.17	scaffold07919	6237
3765	CaLG5	CaSNP2951	60.2	scaffold01359	85178

3766	CaLG5	CaTSNP7810	60.24	-	-
3767	CaLG5	CaTSNP6706	60.24	-	-
3768	CaLG5	CaTSNP8055	60.24	-	-
3769	CaLG5	CaSNP3372	60.25	-	-
3770	CaLG5	CaSNP771	60.36	-	-
3771	CaLG5	CaTSNP8357	60.42	scaffold00120	326087
3772	CaLG5	CaSNP2435	60.42	-	-
3773	CaLG5	CaTSNP7583	60.43	-	-
3774	CaLG5	CaTSNP9114	60.47	scaffold01385	78023
3775	CaLG5	CaSNP3879	60.49	9207548	1939
3776	CaLG5	CaTSNP6873	60.52	scaffold01912	50813
3777	CaLG5	CaSNP2824	60.55	-	-
3778	CaLG5	CaSNP2840	60.64	scaffold01235	80622
3779	CaLG5	CaSNP3167	60.68	-	-
3780	CaLG5	CaTSNP8905	60.77	-	-
3781	CaLG5	CaTSNP6923	60.99	-	-
3782	CaLG5	CaSNP2028	61.02	-	-
3783	CaLG5	CaSNP2461	61.04	-	-
3784	CaLG5	CaSNP847	61.05	-	-
3785	CaLG5	CaTSNP6903	61.08	-	-
3786	CaLG5	CaTSNP7564	61.13	-	-
3787	CaLG5	CaSNP3815	61.65	scaffold02468	40823
3788	CaLG5	CaSNP4064	61.65	-	-
3789	CaLG5	CaTSNP7379	61.78	-	-
3790	CaLG5	CaSNP2041	62.03	9204474	20528
3791	CaLG5	CaSNP2783	62.03	scaffold00364	190544
3792	CaLG5	CaSNP2542	62.05	scaffold00231	245059
3793	CaLG5	CaTSNP6698	62.22	-	-
3794	CaLG5	CaTMS1056	62.28	-	-

3795	CaLG5	CaSNP1927	62.33	scaffold00635	138134
3796	CaLG5	CaTSNP7606	62.6	-	-
3797	CaLG5	CaSNP2110	62.68	scaffold00582	132297
3798	CaLG5	CaSNP2071	62.72	scaffold00449	164014
3799	CaLG5	CaTSNP8397	62.82	-	-
3800	CaLG5	CaTSNP8053	62.85	-	-
3801	CaLG5	CaTSNP8204	62.86	-	-
3802	CaLG5	CaTSNP8194	62.88	-	-
3803	CaLG5	CaSNP397	62.92	scaffold00469	170371
3804	CaLG5	CaSNP786	62.93	scaffold01494	68419
3805	CaLG5	CaSNP191	62.94	-	-
3806	CaLG5	CaSNP172	63.07	9200217	34100
3807	CaLG5	CaSNP441	63.09	-	-
3808	CaLG5	CaTSNP8810	63.12	-	-
3809	CaLG5	CaTSNP6693	63.24	-	-
3810	CaLG5	CaTSNP8834	63.45	9210321	17779
3811	CaLG5	CaTSNP9089	63.45	scaffold00157	273650
3812	CaLG5	CaTSNP9017	64.18	9204832	42951
3813	CaLG5	CaTSNP8674	65.21	-	-
3814	CaLG5	CaTSNP6161	65.34	scaffold00421	165479
3815	CaLG5	CaTSNP7892	65.38	scaffold02112	60554
3816	CaLG5	CaSNP2727	65.48	scaffold00321	189029
3817	CaLG5	CaTSNP7666	65.5	-	-
3818	CaLG5	CaTSNP6385	65.52	scaffold00762	117692
3819	CaLG5	CaSNP4430	65.7	-	-
3820	CaLG5	CaSNP3863	66.13	-	-
3821	CaLG5	CaTSNP8532	66.33	-	-
3822	CaLG5	CaSNP3989	66.39	-	-
3823	CaLG5	CaSNP2549	66.41	scaffold00533	157056

3824	CaLG5	CaTSNP6197	66.5	scaffold03660	21379
3825	CaLG5	CaTSNP6825	66.5	-	-
3826	CaLG5	CaTMS842	66.51	-	-
3827	CaLG5	CaTSNP6524	66.61	scaffold00486	171337
3828	CaLG5	CaTSNP6823	66.61	-	-
3829	CaLG5	CaTSNP8061	66.61	scaffold00617	134243
3830	CaLG5	CaTSNP8853	66.61	-	-
3831	CaLG5	CaTSNP6727	66.67	-	-
3832	CaLG5	CaTSNP8631	66.67	-	-
3833	CaLG5	CaSNP691	66.7	-	-
3834	CaLG5	CaSNP836	66.7	-	-
3835	CaLG5	CaTSNP7137	66.74	-	-
3836	CaLG5	CaTSNP6432	66.77	-	-
3837	CaLG5	CaTSNP7710	66.8	-	-
3838	CaLG5	CaSNP3401	66.89	scaffold02824	31674
3839	CaLG5	CaTSNP6067	67.07	scaffold00029	575317
3840	CaLG5	CaTSNP6509	67.07	scaffold00339	187741
3841	CaLG5	CaTSNP7405	67.27	-	-
3842	CaLG5	CaTMS551	67.33	-	-
3843	CaLG5	CaTSNP7985	67.48	-	-
3844	CaLG5	CaTSNP9098	67.55	-	-
3845	CaLG5	CaTSNP7549	67.58	-	-
3846	CaLG5	CaTSNP7282	67.63	-	-
3847	CaLG5	CaTSNP7089	67.66	-	-
3848	CaLG5	CaTSNP9140	67.69	-	-
3849	CaLG5	CaSNP1948	67.72	9198384	8960
3850	CaLG5	CaSNP305	67.74	scaffold01022	86271
3851	CaLG5	CaTSNP8680	67.74	-	-
3852	CaLG5	CaSNP2529	67.79	-	-

3853	CaLG5	CaTSNP8658	67.85	-	-
3854	CaLG5	CaTSNP8506	67.93	-	-
3855	CaLG5	CaSNP669	67.94	-	-
3856	CaLG5	CaSNP511	67.97	-	-
3857	CaLG5	CaTSNP6233	68	scaffold03414	24418
3858	CaLG5	CaSNP258	68.08	-	-
3859	CaLG5	ESNP16	68.41	-	-
3860	CaLG5	ESNP22	68.42	-	-
3861	CaLG5	ESNP41	68.42	-	-
3862	CaLG5	CaTSNP9088	68.49	-	-
3863	CaLG5	CaSNP41	68.7	-	-
3864	CaLG5	CaSNP256	68.71	9197401	39036
3865	CaLG5	CaSNP293	68.77	-	-
3866	CaLG5	CaSNP773	68.77	-	-
3867	CaLG5	CaSNP501	68.78	-	-
3868	CaLG5	ESNP86	68.89	-	-
3869	CaLG5	CaSNP59	68.96	-	-
3870	CaLG5	CaSNP608	68.98	-	-
3871	CaLG5	CaSNP599	69.03	-	-
3872	CaLG5	CaSNP481	69.04	-	-
3873	CaLG5	CaTSNP7277	69.37	scaffold00086	355838
3874	CaLG5	CaSNP4	69.42	scaffold01458	64874
3875	CaLG5	CaTMS766	69.43	-	-
3876	CaLG5	CaSNP2092	69.45	-	-
3877	CaLG5	CaSNP2144	69.45	-	-
3878	CaLG5	CaSNP3424	69.47	-	-
3879	CaLG5	CaSNP4091	69.47	-	-
3880	CaLG5	CaSNP4340	69.47	9181147	2727
3881	CaLG5	CaSNP2928	69.5	scaffold01792	63130

3882	CaLG5	CaSNP4024	69.5	-	-
3883	CaLG5	CaTSNP8715	69.5	scaffold00422	175317
3884	CaLG5	CaTSNP8848	69.52	scaffold01112	81608
3885	CaLG5	CaSNP15	69.54	-	-
3886	CaLG5	CaTSNP6192	69.55	-	-
3887	CaLG5	CaTSNP6278	69.55	scaffold02708	33509
3888	CaLG5	CaTSNP6628	69.55	9154689	50421
3889	CaLG5	CaTSNP6666	69.55	9159073	53240
3890	CaLG5	CaTSNP7080	69.55	scaffold01157	78936
3891	CaLG5	CaTSNP8805	69.55	-	-
3892	CaLG5	CaTSNP6150	69.55	scaffold02564	45621
3893	CaLG5	CaTSNP6464	69.61	scaffold00219	254144
3894	CaLG5	CaTSNP7020	69.61	-	-
3895	CaLG5	CaTSNP7305	69.61	-	-
3896	CaLG5	CaTSNP8144	69.61	-	-
3897	CaLG5	CaTSNP8256	69.61	-	-
3898	CaLG5	CaTSNP8716	69.61	scaffold00612	131184
3899	CaLG5	CaTSNP8148	69.63	-	-
3900	CaLG5	CaTSNP8257	69.75	-	-
3901	CaLG5	CaSNP2406	69.78	-	-
3902	CaLG5	CaSNP3030	69.81	-	-
3903	CaLG5	CaSNP4026	69.84	scaffold04372	28867
3904	CaLG5	CaSNP3252	69.9	-	-
3905	CaLG5	CaTSNP8765	69.91	-	-
3906	CaLG5	CaTSNP9065	69.91	-	-
3907	CaLG5	CaTSNP6239	69.91	-	-
3908	CaLG5	CaSNP5029	69.93	scaffold05524	10827
3909	CaLG5	CaTSNP6293	69.97	-	-
3910	CaLG5	CaTSNP6346	69.97	-	-

3911	CaLG5	CaTSNP6993	69.97	scaffold00153	284571
3912	CaLG5	CaTSNP7616	69.99	-	-
3913	CaLG5	CaTSNP8772	70.02	-	-
3914	CaLG5	CaTSNP8830	70.06	-	-
3915	CaLG5	CaTSNP8014	70.08	-	-
3916	CaLG5	CaSNP2473	70.23	-	-
3917	CaLG5	CaSNP1937	70.24	-	-
3918	CaLG5	CaSNP3761	70.38	-	-
3919	CaLG5	CaSNP2509	70.39	-	-
3920	CaLG5	CaSNP2487	70.4	-	-
3921	CaLG5	CaTSNP7094	70.54	-	-
3922	CaLG5	CaTSNP8040	70.54	9210471	49637
3923	CaLG5	CaTSNP8346	70.54	-	-
3924	CaLG5	CaTSNP8412	70.54	scaffold00047	476299
3925	CaLG5	CaTSNP6510	70.54	scaffold00064	429138
3926	CaLG5	CaTSNP8668	70.54	-	-
3927	CaLG5	CaTSNP7553	70.59	-	-
3928	CaLG5	CaTSNP6287	70.59	-	-
3929	CaLG5	CaTSNP6556	70.59	-	-
3930	CaLG5	CaTSNP6741	70.59	-	-
3931	CaLG5	CaTSNP6699	70.6	-	-
3932	CaLG5	CaTSNP7539	70.63	-	-
3933	CaLG5	CaTSNP8623	70.63	-	-
3934	CaLG5	CaTSNP7398	70.69	-	-
3935	CaLG5	CaTSNP7980	70.77	-	-
3936	CaLG5	CaTSNP8845	70.86	-	-
3937	CaLG5	CaTSNP6937	71.05	-	-
3938	CaLG5	CaTSNP7772	71.05	-	-
3939	CaLG5	CaTSNP6281	71.1	scaffold00200	261065

3940	CaLG5	CaTSNP6319	71.1	-	-
3941	CaLG5	CaTSNP7280	71.12	-	-
3942	CaLG5	CaTSNP8011	71.12	-	-
3943	CaLG5	CaTSNP8754	71.12	scaffold00430	166099
3944	CaLG5	CaTSNP6314	71.13	-	-
3945	CaLG5	CaTSNP6581	71.48	scaffold10227	4241
3946	CaLG5	CaTSNP7374	71.5	-	-
3947	CaLG5	CaTSNP8667	71.67	scaffold00426	199188
3948	CaLG5	CaTSNP7994	71.7	scaffold00474	149864
3949	CaLG5	CaTSNP7159	71.75	-	-
3950	CaLG5	CaTSNP8139	71.75	-	-
3951	CaLG5	CaTSNP8450	71.77	-	-
3952	CaLG5	CaTSNP7709	71.79	-	-
3953	CaLG5	CaTSNP8008	71.8	scaffold00331	215396
3954	CaLG5	CaTSNP9040	71.81	-	-
3955	CaLG5	CaTSNP7628	71.88	-	-
3956	CaLG5	CaTSNP7700	71.88	-	-
3957	CaLG5	CaTSNP7308	71.88	-	-
3958	CaLG5	CaSNP2808	72.61	-	-
3959	CaLG5	CaSNP1987	72.67	-	-
3960	CaLG5	CaSNP3321	72.76	-	-
3961	CaLG5	CaSNP2174	72.85	-	-
3962	CaLG5	CaSNP2023	72.89	-	-
3963	CaLG5	CaTSNP6068	72.97	scaffold00112	333150
3964	CaLG5	CaTSNP8661	73.07	-	-
3965	CaLG5	CaTSNP8871	73.15	-	-
3966	CaLG5	CaTSNP6934	73.17	-	-
3967	CaLG5	CaSNP3621	73.2	-	-
3968	CaLG5	CaSNP2554	74.45	scaffold01256	83486

3969	CaLG5	CaSNP2825	74.59	-	-
3970	CaLG5	CaTSNP6554	74.74	-	-
3971	CaLG5	CaTMS668	75.44	-	-
3972	CaLG5	CaTSNP7633	76.38	-	-
3973	CaLG5	CaTSNP8826	76.4	-	-
3974	CaLG5	CaTSNP7966	76.41	scaffold00059	415906
3975	CaLG5	CaTSNP8168	76.41	-	-
3976	CaLG5	CaTSNP8212	76.41	-	-
3977	CaLG5	CaTSNP8595	76.41	-	-
3978	CaLG5	CaTSNP8252	76.41	-	-
3979	CaLG5	CaTSNP6803	76.5	-	-
3980	CaLG5	CaTSNP6491	76.51	-	-
3981	CaLG5	CaTSNP8382	76.7	scaffold00492	145987
3982	CaLG5	CaTSNP7853	77.29	-	-
3983	CaLG5	CaTSNP7072	77.7	-	-
3984	CaLG5	CaTSNP8785	77.7	-	-
3985	CaLG5	CaTSNP6175	77.79	-	-
3986	CaLG5	CaTSNP6290	77.79	-	-
3987	CaLG5	CaTSNP6612	77.79	-	-
3988	CaLG5	CaTSNP6661	77.79	scaffold00367	187994
3989	CaLG5	CaTSNP7172	77.79	scaffold13764	2710
3990	CaLG5	CaTSNP7349	77.79	scaffold08538	5560
3991	CaLG5	CaTSNP7973	77.79	9204158	73634
3992	CaLG5	CaTSNP8530	77.79	-	-
3993	CaLG5	CaTSNP9014	77.79	-	-
3994	CaLG5	CaTSNP9042	77.79	scaffold00154	287602
3995	CaLG5	CaTSNP8823	77.81	-	-
3996	CaLG5	CaTSNP8499	77.81	-	-
3997	CaLG5	CaTSNP6712	77.85	-	-

3998	CaLG5	CaTSNP8846	77.85	-	-
3999	CaLG5	CaTSNP6409	77.88	-	-
4000	CaLG5	CaTSNP8117	77.89	9081662	80447
4001	CaLG5	CaTSNP8396	78.11	scaffold00008	868686
4002	CaLG5	CaTSNP7852	78.32	-	-
4003	CaLG5	CaTSNP8867	78.32	-	-
4004	CaLG5	CaTSNP8523	78.39	-	-
4005	CaLG5	CaTSNP6490	78.78	-	-
4006	CaLG5	CaTSNP6864	78.78	-	-
4007	CaLG5	CaTSNP8283	78.78	-	-
4008	CaLG5	CaTSNP9157	78.78	-	-
4009	CaLG5	CaTSNP9050	78.84	-	-
4010	CaLG5	CaTSNP7021	78.95	-	-
4011	CaLG5	CaTSNP7396	79.6	scaffold00140	276053
4012	CaLG5	CaSNP2492	79.66	-	-
4013	CaLG5	CaSNP2585	79.68	scaffold02478	40613
4014	CaLG5	CaSNP897	79.86	-	-
4015	CaLG5	CaSNP2571	79.89	-	-
4016	CaLG5	CaSNP4600	79.94	-	-
4017	CaLG5	CaTSNP6430	80.79	-	-
4018	CaLG5	CaSNP179	80.94	-	-
4019	CaLG5	CaTSNP6483	80.94	9191285	88622
4020	CaLG5	CaTSNP8363	80.94	-	-
4021	CaLG5	CaSNP553	80.98	-	-
4022	CaLG5	CaTSNP9012	80.99	-	-
4023	CaLG5	CaSNP349	81.07	-	-
4024	CaLG5	CaSNP77	81.11	-	-
4025	CaLG5	CaSNP881	81.17	-	-
4026	CaLG5	CaTSNP6536	81.2	-	-

4027	CaLG5	CaTSNP8637	81.31	-	-
4028	CaLG5	CaTSNP6905	81.77	-	-
4029	CaLG5	CaTSNP8889	81.83	-	-
4030	CaLG5	CaTSNP8923	81.86	-	-
4031	CaLG5	CaTSNP6215	81.9	scaffold00055	452676
4032	CaLG5	CaSNP3432	82.2	-	-
4033	CaLG5	CaTSNP7695	82.35	-	-
4034	CaLG5	CaTSNP8409	82.46	9204082	82020
4035	CaLG5	CaSNP2317	82.62	-	-
4036	CaLG5	CaTSNP8925	83.33	scaffold00209	229734
4037	CaLG5	CaTSNP8843	83.42	9133966	33628
4038	CaLG5	CaTSNP8207	84.45	-	-
4039	CaLG5	CaSNP373	84.59	-	-
4040	CaLG5	CaSNP680	84.59	-	-
4041	CaLG5	CaTSNP7420	84.73	-	-
4042	CaLG5	CaSNP534	84.85	-	-
4043	CaLG5	NCPGR196	85.09	-	-
4044	CaLG5	CaTSNP8017	85.21	-	-
4045	CaLG5	CaSNP826	85.42	-	-
4046	CaLG5	PIP143	85.85	-	-
4047	CaLG5	CaSNP2357	85.92	-	-
4048	CaLG5	CaTSNP7712	86.53	-	-
4049	CaLG5	NCPGR232	86.61	scaffold00631	136946
4050	CaLG5	CaTSNP7145	87.09	-	-
4051	CaLG5	CaSNP4732	88.12	-	-
4052	CaLG5	CaSNP2099	88.85	-	-
4053	CaLG5	CaTSNP6317	89.01	-	-
4054	CaLG5	CaTSNP7809	89.02	scaffold01442	79622
4055	CaLG5	CaTSNP6584	89.06	-	-

4056	CaLG5	CaTSNP6907	89.06	-	-
4057	CaLG5	CaTSNP6548	89.71	-	-
4058	CaLG5	CaTSNP7073	90.3	-	-
4059	CaLG5	CESSR23	91.13	-	-
4060	CaLG5	CaTSNP8768	91.21	-	-
4061	CaLG5	CaTSNP6684	91.23	-	-
4062	CaLG5	CaTSNP8003	91.24	-	-
4063	CaLG5	CaTSNP7967	91.44	scaffold00412	164427
4064	CaLG5	CaTSNP8315	91.44	-	-
4065	CaLG5	CaTSNP7603	91.52	-	-
4066	CaLG5	CaTSNP6326	91.54	-	-
4067	CaLG5	CaTSNP6705	91.56	scaffold02482	51287
4068	CaLG5	CaTMS787	91.56	-	-
4069	CaLG5	CaTSNP7723	91.59	9206340	12532
4070	CaLG5	CaTSNP8277	91.68	scaffold00862	104688
4071	CaLG5	CaTSNP6279	91.92	-	-
4072	CaLG5	CaTSNP8833	92	-	-
4073	CaLG5	CaSNP2934	92.07	-	-
4074	CaLG5	CaTSNP8578	92.07	-	-
4075	CaLG5	CaSNP3208	92.14	-	-
4076	CaLG5	CaSNP2926	92.18	-	-
4077	CaLG5	CaTSNP9085	92.19	-	-
4078	CaLG5	CaTSNP8748	92.19	-	-
4079	CaLG5	CaTSNP8944	92.19	-	-
4080	CaLG5	TS35	92.26	-	-
4081	CaLG5	CaSNP2056	92.28	9091287	17901
4082	CaLG5	CaTSNP7674	92.38	-	-
4083	CaLG5	CaSNP2013	92.42	scaffold00545	144639
4084	CaLG5	CaTSNP6542	92.5	-	-

4085	CaLG5	CaTSNP6323	92.63	-	-
4086	CaLG5	CaTMS715	94.76	-	-
4087	CaLG5	CaTSNP7676	95.3	-	-
4088	CaLG5	CaTSNP8298	95.35	-	-
4089	CaLG5	CaTSNP8439	95.4	-	-
4090	CaLG5	CaTSNP7887	95.41	scaffold00075	404207
4091	CaLG5	CaTSNP7992	95.43	-	-
4092	CaLG5	CaSNP2212	95.96	-	-
4093	CaLG5	CaSNP2541	95.96	-	-
4094	CaLG5	CaTSNP9090	96	-	-
4095	CaLG5	CaTSNP7475	96.12	-	-
4096	CaLG5	CaTSNP7769	96.12	-	-
4097	CaLG5	CaTSNP6071	96.17	-	-
4098	CaLG5	CaGMS23	96.32	-	-
4099	CaLG5	CESSR165	96.85	-	-
4100	CaLG5	CaTSNP9153	97.07	-	-
4101	CaLG5	CaTSNP7904	97.07	-	-
4102	CaLG5	CaTSNP7932	97.07	-	-
4103	CaLG5	CaTSNP8647	97.07	-	-
4104	CaLG5	CaTSNP7394	97.07	-	-
4105	CaLG5	CaTSNP6341	97.09	-	-
4106	CaLG5	CaTSNP6534	97.1	-	-
4107	CaLG5	CaTSNP6637	97.1	-	-
4108	CaLG5	CaTSNP7097	97.1	-	-
4109	CaLG5	CaTSNP9125	97.1	-	-
4110	CaLG5	CaTSNP7982	97.11	-	-
4111	CaLG5	CaTSNP6950	97.19	-	-
4112	CaLG5	CaTSNP6241	97.19	9207601	22382
4113	CaLG5	CaTSNP6816	97.19	-	-

4114	CaLG5	CaTSNP6936	97.19	-	-
4115	CaLG5	CaTSNP7401	97.19	-	-
4116	CaLG5	CaTSNP8446	97.19	-	-
4117	CaLG5	CaTSNP8540	97.19	-	-
4118	CaLG5	CaSNP2155	97.19	-	-
4119	CaLG5	CaTSNP8401	97.21	-	-
4120	CaLG5	CaSNP2020	97.27	-	-
4121	CaLG5	CaSNP5033	97.36	-	-
4122	CaLG5	CaSNP2898	97.36	9191643	56242
4123	CaLG5	CaTSNP7884	97.5	-	-
4124	CaLG5	CaSNP2346	97.52	scaffold00527	142243
4125	CaLG5	CaSNP581	97.52	scaffold00795	110840
4126	CaLG5	CaTSNP8281	97.54	-	-
4127	CaLG5	CaSNP391	97.56	scaffold00092	345607
4128	CaLG5	CaSNP532	97.59	scaffold00387	197952
4129	CaLG5	CaSNP739	97.59	-	-
4130	CaLG5	CaTSNP6145	97.63	-	-
4131	CaLG5	CaTSNP7668	97.63	-	-
4132	CaLG5	CaTSNP8704	97.63	-	-
4133	CaLG5	CaTSNP7558	97.64	-	-
4134	CaLG5	CaTSNP6489	97.67	-	-
4135	CaLG5	CaTSNP8767	97.7	-	-
4136	CaLG5	CaTSNP8970	97.75	-	-
4137	CaLG5	ESNP102	97.75	scaffold00539	157474
4138	CaLG5	CaTSNP7734	97.79	-	-
4139	CaLG5	CaTSNP8472	97.79	-	-
4140	CaLG5	CaTSNP7053	97.81	-	-
4141	CaLG5	CaTSNP7910	97.81	9143161	18632
4142	CaLG5	CaTSNP6262	97.83	scaffold00656	135970

4143	CaLG5	CaTSNP7313	97.83	-	-
4144	CaLG5	CaTSNP8181	97.83	-	-
4145	CaLG5	CaTSNP7686	97.84	-	-
4146	CaLG5	CaTSNP6349	97.85	-	-
4147	CaLG5	CaTSNP8601	97.85	-	-
4148	CaLG5	CaSNP140	97.85	-	-
4149	CaLG5	CaTSNP6425	97.88	-	-
4150	CaLG5	CaTSNP7093	97.88	-	-
4151	CaLG5	CaTSNP8504	97.89	-	-
4152	CaLG5	CaTSNP6812	97.95	-	-
4153	CaLG5	CaTSNP7664	97.95	-	-
4154	CaLG5	CaTSNP8185	97.95	-	-
4155	CaLG5	CaSNP1951	98.02	scaffold00372	184656
4156	CaLG5	CaSNP544	98.08	scaffold00005	930854
4157	CaLG5	CaTSNP7382	98.37	-	-
4158	CaLG5	CESSR111	98.39	-	-
4159	CaLG5	CaTSNP9067	98.44	-	-
4160	CaLG5	CaSNP708	98.76	9180663	99948
4161	CaLG5	CaSNP85	99.47	-	-
4162	CaLG5	CaSNP797	99.5	-	-
4163	CaLG5	CaSNP277	99.54	-	-
4164	CaLG5	CaSNP342	99.91	-	-
4165	CaLG5	CaSNP310	100	scaffold00193	231428
4166	CaLG5	CaSNP21	100.2	scaffold00479	170853
4167	CaLG5	CaSNP598	100.2	scaffold00949	111634
4168	CaLG5	CaSNP435	100.2	scaffold00240	222072
4169	CaLG5	CaSNP896	100.6	-	-
4170	CaLG5	CEST160	100.9	-	-
4171	CaLG5	NCPGR86	101	-	-

4172	CaLG5	NCPGR201	101.1	-	-
4173	CaLG5	CaSNP270	101.4	-	-
4174	CaLG5	PIP176	101.5	-	-
4175	CaLG5	NCPGR59	101.5	scaffold00049	453060
4176	CaLG5	PIP209	101.6	-	-
4177	CaLG5	CaSNP2532	101.8	-	-
4178	CaLG5	CaSNP1955	101.9	-	-
4179	CaLG5	CaTSNP8400	101.9	-	-
4180	CaLG5	CaSNP3173	102	scaffold00514	155231
4181	CaLG5	CaTSNP6500	102	-	-
4182	CaLG5	CaTSNP9160	102	-	-
4183	CaLG5	CaTSNP6479	102	-	-
4184	CaLG5	CaTMS706	102	-	-
4185	CaLG5	CaSNP3901	102.1	-	-
4186	CaLG5	CaTSNP7758	102.1	-	-
4187	CaLG5	CaTSNP8282	102.1	-	-
4188	CaLG5	CaTSNP8436	102.1	-	-
4189	CaLG5	CaSNP679	102.1	scaffold00404	164189
4190	CaLG5	CaSNP543	102.1	-	-
4191	CaLG5	CaSNP486	102.2	scaffold02362	39533
4192	CaLG5	CaTSNP6868	102.2	-	-
4193	CaLG5	CaTSNP6053	102.3	-	-
4194	CaLG5	CaTSNP6767	102.3	-	-
4195	CaLG5	CaTSNP8038	102.4	-	-
4196	CaLG5	CaSNP808	102.4	scaffold00189	240464
4197	CaLG5	CaTSNP6448	102.4	-	-
4198	CaLG5	CEST27	102.6	-	-
4199	CaLG5	CaSNP3897	102.6	-	-
4200	CaLG5	CaTSNP6992	102.9	-	-

4201	CaLG5	CaTSNP7261	102.9	-	-
4202	CaLG5	CaSNP149	102.9	-	-
4203	CaLG5	CaSNP3957	103	scaffold02688	37747
4204	CaLG5	CaSNP377	103.1	scaffold00735	122780
4205	CaLG5	CaSNP255	103.1	scaffold00106	329997
4206	CaLG5	CaSNP4416	103.1	-	-
4207	CaLG5	CaSNP675	103.1	-	-
4208	CaLG5	CaSNP4830	103.2	-	-
4209	CaLG5	CaSSR4	103.4	-	-
4210	CaLG5	CaTSNP7213	103.4	scaffold00165	272492
4211	CaLG5	CaSNP2033	103.5	scaffold00293	194946
4212	CaLG5	CaTSNP7335	103.5	-	-
4213	CaLG5	CaTSNP6460	103.6	-	-
4214	CaLG5	CaTSNP7289	103.7	-	-
4215	CaLG5	CaTSNP8097	103.8	-	-
4216	CaLG5	CaTMS708	103.9	-	-
4217	CaLG5	CaTSNP9064	104.1	-	-
4218	CaLG5	CaTSNP6735	104.1	-	-
4219	CaLG5	CaTSNP9048	104.1	-	-
4220	CaLG5	CaTSNP8549	104.1	-	-
4221	CaLG5	CaTSNP8608	104.2	scaffold06082	9893
4222	CaLG5	CaTSNP6006	104.2	-	-
4223	CaLG5	CaTSNP6549	104.5	-	-
4224	CaLG5	CaTSNP6142	104.5	-	-
4225	CaLG5	CaTSNP8425	104.5	9193831	54768
4226	CaLG5	CaTSNP7231	104.6	-	-
4227	CaLG5	CaTSNP7312	104.7	scaffold01466	64867
4228	CaLG5	CaTSNP8127	105.2	-	-
4229	CaLG5	CaTSNP8490	105.5	-	-

4230	CaLG5	CaTSNP8988	105.5	-	-
4231	CaLG5	CaTSNP9078	105.5	-	-
4232	CaLG5	CaTSNP6632	105.6	-	-
4233	CaLG5	CaTSNP8740	105.6	-	-
4234	CaLG5	CaTSNP6677	106.1	-	-
4235	CaLG5	PIP55	106.6	-	-
4236	CaLG5	CaSNP614	106.9	-	-
4237	CaLG5	TAA169a	107.2	-	-
4238	CaLG5	CaTSNP8269	107.7	-	-
4239	CaLG5	H4F07	107.9	-	-
4240	CaLG5	CaGMS1204	108.1	-	-
4241	CaLG5	CaSNP4701	108.2	scaffold03131	37199
4242	CaLG5	CaTSNP7806	108.8	scaffold16987	2023
4243	CaLG5	CaTSNP6762	109	-	-
4244	CaLG5	CaTSNP7803	109.1	-	-
4245	CaLG5	CaTSNP8722	109.1	-	-
4246	CaLG5	CEST179	109.1	-	-
4247	CaLG5	CaTSNP7322	109.1	9215941	58130
4248	CaLG5	CaTSNP7424	109.1	-	-
4249	CaLG5	CaTSNP8806	109.1	-	-
4250	CaLG5	CaTSNP9019	109.1	-	-
4251	CaLG5	CaTSNP8707	109.2	-	-
4252	CaLG5	CaTSNP6070	109.2	-	-
4253	CaLG5	CaTSNP8296	109.2	-	-
4254	CaLG5	CaTSNP7780	109.2	-	-
4255	CaLG5	CaSNP456	109.3	9191570	60940
4256	CaLG5	CaSNP522	109.8	-	-
4257	CaLG5	CaSNP758	109.8	scaffold00104	322089
4258	CaLG5	CaSNP1929	110.4	-	-

4259	CaLG5	CaSNP4622	110.4	-	-
4260	CaLG5	CESSR432	110.5	-	-
4261	CaLG5	CaSNP127	110.6	-	-
4262	CaLG5	CaSNP52	110.6	-	-
4263	CaLG5	CaSNP92	110.6	-	-
4264	CaLG5	CaSNP636	110.7	9128049	44970
4265	CaLG5	STMS8	110.8	-	-
4266	CaLG5	CaSNP894	110.9	-	-
4267	CaLG5	CaSNP11	110.9	-	-
4268	CaLG5	CaSNP430	112.4	-	-
4269	CaLG5	CaSNP362	112.4	-	-
4270	CaLG5	CaSNP98	113.2	scaffold00002	1228777
4271	CaLG5	CESSR48	113.4	-	-
4272	CaLG5	CaGMS1170	113.7	-	-
4273	CaLG5	CaTMS637	113.7	-	-
4274	CaLG5	CaSNP2528	114.2	9203043	21958
4275	CaLG5	NCPGR56	118.4	scaffold01379	77046
4276	CaLG5	CEST112	124.6	-	-
4277	CaLG5	CEST181	126.8	-	-
4278	CaLG5	PIP218	135.7	-	-
4279	CaLG5	CESSR133	142.2	-	-
4280	CaLG5	PIP154	149.9	-	-
4281	CaLG5	CESSR142	158.8	-	-
4282	CaLG6	CESSR183	0	-	-
4283	CaLG6	CESSR160	3.921	scaffold07169	7161
4284	CaLG6	PIP147	4.433	-	-
4285	CaLG6	NCPGR238	5.745	scaffold00228	283173
4286	CaLG6	CaGMS2	13.92	scaffold01223	88698
4287	CaLG6	CESSR130	17.83	scaffold02069	58169

4288	CaLG6	CaGMS1210	19.13	scaffold01114	88777
4289	CaLG6	H4A07	20.11	-	-
4290	CaLG6	CaTMS599	26.16	-	-
4291	CaLG6	CaTMS667	27.34	-	-
4292	CaLG6	CaTMS635	27.61	-	-
4293	CaLG6	CESSR62	29.67	scaffold02942	39741
4294	CaLG6	CaGMS1241	30.01	-	-
4295	CaLG6	NCPGR46	32.22	-	-
4296	CaLG6	PIP196	35.42	-	-
4297	CaLG6	CaSNP5035	36.29	scaffold00174	268922
4298	CaLG6	CaTMS1118	36.41	-	-
4299	CaLG6	CaSSR2	38.45	-	-
4300	CaLG6	PIP142	39.52	-	-
4301	CaLG6	CaTSNP8503	42.59	-	-
4302	CaLG6	CaSNP235	43.42	scaffold00258	205335
4303	CaLG6	CaSNP734	43.42	9194250	64868
4304	CaLG6	CaGMS278	43.8	-	-
4305	CaLG6	CESSR88	44.7	-	-
4306	CaLG6	CaGMS1198	46	-	-
4307	CaLG6	CaSNP91	47.06	9187459	51767
4308	CaLG6	CaGMS332	47.32	-	-
4309	CaLG6	CaSNP5034	48.24	scaffold00221	220089
4310	CaLG6	CaTSNP9115	48.61	-	-
4311	CaLG6	CaTSNP9158	48.73	-	-
4312	CaLG6	CaTSNP7983	48.78	-	-
4313	CaLG6	CaTSNP7744	48.79	-	-
4314	CaLG6	CaTSNP6708	48.82	-	-
4315	CaLG6	CaTSNP6732	48.84	-	-
4316	CaLG6	CaTSNP7559	49.1	-	-

4317	CaLG6	CaTSNP6804	49.19	scaffold01277	78400
4318	CaLG6	CaTSNP8043	49.2	-	-
4319	CaLG6	CaTSNP7537	49.21	-	-
4320	CaLG6	CaTSNP8019	49.21	-	-
4321	CaLG6	CaTSNP6189	49.41	-	-
4322	CaLG6	CaTSNP8092	49.49	9211044	43590
4323	CaLG6	CaTSNP8334	49.66	-	-
4324	CaLG6	CaTSNP6244	49.77	-	-
4325	CaLG6	CaGMS19	49.92	-	-
4326	CaLG6	CaTSNP7993	50.01	9155483	70406
4327	CaLG6	CaTSNP8784	50.01	-	-
4328	CaLG6	CaTSNP8154	50.01	-	-
4329	CaLG6	CaTSNP6861	50.11	-	-
4330	CaLG6	CaTSNP6401	50.16	-	-
4331	CaLG6	CaTSNP8297	50.19	-	-
4332	CaLG6	CaTSNP6398	50.2	-	-
4333	CaLG6	CaTSNP8933	50.24	-	-
4334	CaLG6	CaTSNP6503	50.3	-	-
4335	CaLG6	CaTSNP7126	50.3	-	-
4336	CaLG6	CaTSNP7492	50.3	-	-
4337	CaLG6	CaSNP3808	50.44	scaffold00992	93267
4338	CaLG6	CaTSNP8776	50.48	-	-
4339	CaLG6	CaSNP5037	50.53	-	-
4340	CaLG6	CaTSNP6436	50.65	scaffold01413	89484
4341	CaLG6	CaTSNP9052	50.65	-	-
4342	CaLG6	CaTSNP8349	50.76	-	-
4343	CaLG6	CaTSNP6650	50.89	scaffold00714	119066
4344	CaLG6	CaTSNP7981	50.89	-	-
4345	CaLG6	CaTSNP6561	50.93	-	-

4346	CaLG6	CaSNP3064	50.99	-	-
4347	CaLG6	CaSNP3193	51.07	scaffold00073	403024
4348	CaLG6	CaSNP2604	51.13	-	-
4349	CaLG6	CaSNP2659	51.16	scaffold01635	58812
4350	CaLG6	CaSNP2251	51.22	scaffold01438	85466
4351	CaLG6	CaSNP3454	51.62	scaffold00077	407345
4352	CaLG6	CaSNP3911	51.62	scaffold00156	291381
4353	CaLG6	CaSNP901	51.65	scaffold00989	94598
4354	CaLG6	CaSNP4663	51.76	scaffold00121	299340
4355	CaLG6	CaSNP504	52.57	9205769	41800
4356	CaLG6	CaSNP442	52.64	9214716	60852
4357	CaLG6	CaSNP651	52.77	scaffold01412	67392
4358	CaLG6	CaSNP157	52.81	scaffold00169	249776
4359	CaLG6	CaSNP43	52.82	9080487	54963
4360	CaLG6	CaSNP1923	53.52	-	-
4361	CaLG6	CaSNP738	54.54	-	-
4362	CaLG6	CaSNP793	54.74	-	-
4363	CaLG6	CaSNP62	54.84	-	-
4364	CaLG6	CaSNP518	54.87	-	-
4365	CaLG6	CaTMS927	55.49	-	-
4366	CaLG6	CaSNP399	55.6	scaffold01001	91619
4367	CaLG6	CaSNP3654	56.08	scaffold02390	48449
4368	CaLG6	CaSNP5154	56.36	-	-
4369	CaLG6	CaSNP2842	56.58	-	-
4370	CaLG6	CaTSNP9025	56.65	scaffold07527	6661
4371	CaLG6	CaTSNP8023	56.82	-	-
4372	CaLG6	CaTSNP6050	56.85	-	-
4373	CaLG6	CaTSNP8953	56.85	-	-
4374	CaLG6	CaTSNP7359	56.95	-	-

4375	CaLG6	CaTSNP7858	56.96	9214764	27095
4376	CaLG6	CaTSNP6422	56.97	scaffold10981	3813
4377	CaLG6	CaTSNP6586	56.98	-	-
4378	CaLG6	CaTSNP7210	56.98	-	-
4379	CaLG6	CaTSNP7955	56.98	-	-
4380	CaLG6	CaTSNP8248	56.98	-	-
4381	CaLG6	CaTSNP8335	56.98	-	-
4382	CaLG6	CaTSNP6048	56.99	-	-
4383	CaLG6	CaTSNP6715	56.99	9212007	57233
4384	CaLG6	CaTSNP9006	56.99	-	-
4385	CaLG6	CaTSNP7384	57	scaffold00956	101430
4386	CaLG6	CaTSNP6606	57.05	scaffold00045	513320
4387	CaLG6	CaTSNP8320	57.05	-	-
4388	CaLG6	CaTSNP6604	57.52	-	-
4389	CaLG6	CaTSNP8897	57.53	-	-
4390	CaLG6	CaTSNP8180	57.54	-	-
4391	CaLG6	CaTSNP8134	57.56	-	-
4392	CaLG6	CaTSNP6302	57.61	9215048	27851
4393	CaLG6	CaTSNP6254	57.61	scaffold00014	722610
4394	CaLG6	CaTSNP7059	57.71	-	-
4395	CaLG6	CaSNP1936	58.52	scaffold00141	289402
4396	CaLG6	CaSNP4101	58.58	-	-
4397	CaLG6	GA26	60.89	-	-
4398	CaLG6	GA21	61.22	-	-
4399	CaLG6	GAA41	61.7	-	-
4400	CaLG6	GAA39	61.85	-	-
4401	CaLG6	CaTMS702	61.96	-	-
4402	CaLG6	TS83	62.55	-	-
4403	CaLG6	NCPGR251	64.12	-	-

4404	CaLG6	CaTMS878	65.15	-	-
4405	CaLG6	NCPGR256	65.51	scaffold00382	169962
4406	CaLG6	CaTMS538	65.52	-	-
4407	CaLG6	CaSNP206	65.7	scaffold00071	401710
4408	CaLG6	CaSNP90	65.7	-	-
4409	CaLG6	CaSNP719	65.73	-	-
4410	CaLG6	ESNP10	65.73	-	-
4411	CaLG6	CaSNP283	65.88	-	-
4412	CaLG6	ESNP85	65.89	scaffold00725	122787
4413	CaLG6	CaSNP2769	65.92	-	-
4414	CaLG6	CaSNP564	66.1	-	-
4415	CaLG6	CaSNP575	66.25	scaffold00114	298061
4416	CaLG6	CaTSNP7922	66.53	scaffold00089	407492
4417	CaLG6	CaTSNP7328	66.56	-	-
4418	CaLG6	CaSNP520	66.62	-	-
4419	CaLG6	CaTSNP6674	66.62	-	-
4420	CaLG6	CaSNP120	66.78	scaffold00460	153837
4421	CaLG6	CaSNP560	66.78	-	-
4422	CaLG6	CaSNP872	66.78	scaffold00727	118270
4423	CaLG6	CaGMS223	66.85	-	-
4424	CaLG6	CaSNP291	66.87	9123736	47483
4425	CaLG6	CaGMS478	67	-	-
4426	CaLG6	CaSNP655	67.01	scaffold00134	290746
4427	CaLG6	CaTSNP7353	67.06	-	-
4428	CaLG6	CaTSNP6296	67.06	-	-
4429	CaLG6	CaSNP2394	67.08	-	-
4430	CaLG6	CaTSNP6967	67.09	-	-
4431	CaLG6	CaTSNP8816	67.09	-	-
4432	CaLG6	CaSNP2060	67.15	-	-

4433	CaLG6	CaTSNP8275	67.16	-	-
4434	CaLG6	CaTSNP8485	67.16	-	-
4435	CaLG6	CaSNP631	67.19	-	-
4436	CaLG6	CaTSNP8829	67.25	-	-
4437	CaLG6	CaSNP4154	67.29	-	-
4438	CaLG6	CaTMS891	67.35	-	-
4439	CaLG6	CaTSNP9032	67.36	-	-
4440	CaLG6	CaTSNP8959	67.38	-	-
4441	CaLG6	CaTSNP8550	67.45	-	-
4442	CaLG6	CaTSNP7851	67.45	-	-
4443	CaLG6	CaTMS865	67.63	-	-
4444	CaLG6	CaSNP24	67.64	scaffold00163	285565
4445	CaLG6	CaSNP497	67.64	scaffold00149	266714
4446	CaLG6	CaSNP2017	67.66	-	-
4447	CaLG6	CaSNP2187	67.66	scaffold00181	252172
4448	CaLG6	CaSNP2899	67.66	-	-
4449	CaLG6	CaSNP715	67.81	-	-
4450	CaLG6	CaSNP633	68.21	scaffold00995	104121
4451	CaLG6	CESSR24	68.23	-	-
4452	CaLG6	CaSNP122	68.47	-	-
4453	CaLG6	CaSNP787	68.47	9167113	13039
4454	CaLG6	CaSNP678	68.7	-	-
4455	CaLG6	CaSNP731	68.7	-	-
4456	CaLG6	CaTSNP8487	68.86	scaffold00202	229632
4457	CaLG6	CaTSNP6344	68.89	-	-
4458	CaLG6	CaTSNP9010	68.89	-	-
4459	CaLG6	CaTSNP8241	68.91	-	-
4460	CaLG6	CaTSNP7006	68.93	-	-
4461	CaLG6	CaSNP186	69.07	-	-

4462	CaLG6	CaTSNP8379	69.22	-	-
4463	CaLG6	CaTSNP8653	69.33	scaffold00592	137372
4464	CaLG6	CaSNP2044	69.38	-	-
4465	CaLG6	CaTSNP8907	69.46	-	-
4466	CaLG6	CaTSNP8583	69.47	-	-
4467	CaLG6	CaSNP4898	69.5	-	-
4468	CaLG6	CaTSNP8979	69.55	-	-
4469	CaLG6	CaSNP4574	69.74	-	-
4470	CaLG6	CaGMS15	69.74	-	-
4471	CaLG6	CaSNP5065	69.83	-	-
4472	CaLG6	CaSNP617	70.12	scaffold00330	184665
4473	CaLG6	CaTMS727	70.25	-	-
4474	CaLG6	STMS2	70.29	-	-
4475	CaLG6	CaSNP4402	70.46	-	-
4476	CaLG6	CaSNP4880	70.69	scaffold03106	38023
4477	CaLG6	CaTSNP7768	70.69	-	-
4478	CaLG6	CaTSNP7825	70.72	-	-
4479	CaLG6	CaSNP635	70.72	scaffold00639	140266
4480	CaLG6	CaTSNP8424	70.75	scaffold03719	22252
4481	CaLG6	CaSNP123	70.79	scaffold00393	174682
4482	CaLG6	CaTSNP8407	70.8	-	-
4483	CaLG6	CaTSNP8937	70.84	-	-
4484	CaLG6	CaTSNP7263	70.92	-	-
4485	CaLG6	CaTSNP6889	71	-	-
4486	CaLG6	NCPGR123	71.07	scaffold00349	196416
4487	CaLG6	CaTSNP7274	71.19	-	-
4488	CaLG6	CaTSNP6027	71.3	-	-
4489	CaLG6	CaSNP4519	71.37	9089380	24113
4490	CaLG6	CaSNP1960	71.44	-	-

4491	CaLG6	CaSNP3515	71.44	scaffold01768	60848
4492	CaLG6	CaTSNP6028	71.44	-	-
4493	CaLG6	CaTMS536	71.48	-	-
4494	CaLG6	CaTSNP8090	71.51	scaffold00199	238000
4495	CaLG6	CaSNP2450	71.56	-	-
4496	CaLG6	CaSNP1963	71.56	-	-
4497	CaLG6	CaTSNP8569	71.68	-	-
4498	CaLG6	CaSNP3295	71.69	-	-
4499	CaLG6	CaTSNP7817	71.7	-	-
4500	CaLG6	CaSNP217	71.71	-	-
4501	CaLG6	CaSNP2924	72.06	-	-
4502	CaLG6	CaTSNP7939	72.27	scaffold07084	7293
4503	CaLG6	CaTSNP7503	72.38	-	-
4504	CaLG6	CaTSNP8927	72.44	-	-
4505	CaLG6	CaTSNP6320	72.47	-	-
4506	CaLG6	CaTSNP7472	72.49	9104880	31207
4507	CaLG6	CaTSNP6315	72.5	-	-
4508	CaLG6	CaTMS851	72.68	-	-
4509	CaLG6	CaSNP4488	72.73	-	-
4510	CaLG6	CaSNP2230	72.77	-	-
4511	CaLG6	CaSNP2133	72.87	-	-
4512	CaLG6	CaTSNP7122	72.88	scaffold01460	79813
4513	CaLG6	CaTSNP6756	72.94	scaffold01462	70417
4514	CaLG6	CaTSNP8494	72.97	-	-
4515	CaLG6	CaTSNP8769	72.97	-	-
4516	CaLG6	CaTMS844	72.98	-	-
4517	CaLG6	CaSNP2809	73	-	-
4518	CaLG6	CaSNP267	73.19	-	-
4519	CaLG6	CaGMS38	73.57	-	-

4520	CaLG6	CaTSNP7111	73.61	scaffold03353	24825
4521	CaLG6	CaSNP4615	73.82	scaffold08814	5299
4522	CaLG6	CaTSNP6186	73.99	-	-
4523	CaLG6	CaSNP2057	74.33	scaffold01099	86005
4524	CaLG6	CaSNP2437	74.33	scaffold00298	202886
4525	CaLG6	CaSNP2447	74.37	-	-
4526	CaLG6	CaSNP4018	74.52	scaffold00588	146251
4527	CaLG6	CaTSNP6335	74.57	scaffold02786	32304
4528	CaLG6	CaSNP2011	74.58	scaffold00067	406311
4529	CaLG6	CaSNP2977	74.58	scaffold01667	63456
4530	CaLG6	CaTSNP6863	74.59	scaffold00883	112607
4531	CaLG6	CaTSNP8559	74.59	-	-
4532	CaLG6	CaTSNP8739	74.59	scaffold02206	43516
4533	CaLG6	STMS15	74.67	-	-
4534	CaLG6	CaSNP2486	74.7	scaffold01238	76649
4535	CaLG6	CaTSNP6959	75.02	-	-
4536	CaLG6	CaSNP647	75.21	scaffold00095	351034
4537	CaLG6	CaTSNP8184	75.31	-	-
4538	CaLG6	CaTSNP8878	75.55	-	-
4539	CaLG6	CaSNP2163	75.55	-	-
4540	CaLG6	CaTSNP6225	75.55	scaffold01328	82573
4541	CaLG6	CaTSNP8650	75.55	-	-
4542	CaLG6	CaTSNP8251	75.62	-	-
4543	CaLG6	CaTSNP6443	75.7	-	-
4544	CaLG6	CaTSNP8371	75.7	-	-
4545	CaLG6	CaTSNP7206	75.74	-	-
4546	CaLG6	CaTSNP8433	75.74	-	-
4547	CaLG6	CaTSNP7613	75.75	scaffold00869	102319
4548	CaLG6	TA14	75.75	-	-

4549	CaLG6	CaTSNP9073	75.81	scaffold00322	183346
4550	CaLG6	CaTSNP8598	75.83	-	-
4551	CaLG6	CaTSNP6911	75.85	-	-
4552	CaLG6	CaSNP2309	75.87	-	-
4553	CaLG6	CaTSNP6354	75.91	scaffold01311	76432
4554	CaLG6	CaTSNP8545	75.91	-	-
4555	CaLG6	CaTSNP6275	75.97	-	-
4556	CaLG6	CaTSNP6407	75.97	scaffold00997	89260
4557	CaLG6	CaTSNP7850	75.97	-	-
4558	CaLG6	CaTSNP7972	75.97	-	-
4559	CaLG6	CaTSNP6750	75.99	-	-
4560	CaLG6	CaTSNP7481	76	-	-
4561	CaLG6	CaTSNP8285	76.04	-	-
4562	CaLG6	CaTSNP8292	76.04	-	-
4563	CaLG6	CaTSNP8864	76.04	-	-
4564	CaLG6	CaTSNP8365	76.05	-	-
4565	CaLG6	CaTSNP9075	76.07	-	-
4566	CaLG6	CaTSNP7898	76.08	-	-
4567	CaLG6	CaTSNP8435	76.09	-	-
4568	CaLG6	CaTSNP7702	76.1	-	-
4569	CaLG6	CaTSNP7105	76.16	-	-
4570	CaLG6	CaTSNP7161	76.17	-	-
4571	CaLG6	CaTSNP7005	76.2	scaffold00450	171423
4572	CaLG6	CaSNP2497	76.21	-	-
4573	CaLG6	CaSNP40	76.32	scaffold01454	81110
4574	CaLG6	CaTSNP7329	76.36	-	-
4575	CaLG6	CaTSNP7351	76.36	-	-
4576	CaLG6	CaTSNP7355	76.36	-	-
4577	CaLG6	CaTSNP8165	76.36	-	-

4578	CaLG6	CaTSNP7725	76.37	-	-
4579	CaLG6	CaSNP1946	76.4	-	-
4580	CaLG6	GA9	76.44	-	-
4581	CaLG6	CaTSNP6291	76.51	-	-
4582	CaLG6	CaSNP113	76.53	scaffold00063	446673
4583	CaLG6	CaSNP509	76.55	scaffold00038	496958
4584	CaLG6	CaSNP30	76.58	scaffold01497	90543
4585	CaLG6	CaSNP605	76.58	-	-
4586	CaLG6	CaTSNP8543	76.65	scaffold00379	178208
4587	CaLG6	CaSNP25	76.71	scaffold00566	137252
4588	CaLG6	CaSNP745	76.71	9207264	68808
4589	CaLG6	CaSNP188	76.72	-	-
4590	CaLG6	CaSNP82	76.81	-	-
4591	CaLG6	CaSNP870	76.81	-	-
4592	CaLG6	TA106	76.88	-	-
4593	CaLG6	NCPGR148	76.9	-	-
4594	CaLG6	CaTSNP8039	77.11	-	-
4595	CaLG6	CaTSNP7662	77.11	-	-
4596	CaLG6	CaTSNP8077	77.15	-	-
4597	CaLG6	CaSNP395	77.37	-	-
4598	CaLG6	H5A04	77.7	-	-
4599	CaLG6	NCPGR187	77.72	-	-
4600	CaLG6	NCPGR221	77.74	-	-
4601	CaLG6	CaSNP4084	77.83	-	-
4602	CaLG6	CaTSNP6414	77.94	scaffold00336	183406
4603	CaLG6	CaSNP829	77.96	scaffold00395	195843
4604	CaLG6	NCPGR4	78.04	-	-
4605	CaLG6	CaSNP367	78.09	-	-
4606	CaLG6	CaTSNP6110	78.2	-	-

4607	CaLG6	CaSNP17	78.22	-	-
4608	CaLG6	CaTSNP7157	78.27	scaffold06790	8525
4609	CaLG6	CaTSNP7303	78.27	scaffold00256	220910
4610	CaLG6	CaTSNP9016	78.27	-	-
4611	CaLG6	CaTSNP7400	78.43	-	-
4612	CaLG6	CaTSNP9069	78.44	-	-
4613	CaLG6	NCPGR202	78.62	-	-
4614	CaLG6	CaTSNP8990	78.65	scaffold00952	95708
4615	CaLG6	GA34	78.74	-	-
4616	CaLG6	CaTSNP7696	78.88	-	-
4617	CaLG6	CaSNP240	78.96	scaffold01337	79461
4618	CaLG6	CaTSNP7284	79.14	scaffold00595	133210
4619	CaLG6	CaTSNP6468	79.19	-	-
4620	CaLG6	CaTSNP9074	79.25	-	-
4621	CaLG6	CaTSNP8028	79.28	-	-
4622	CaLG6	NCPGR200	79.57	-	-
4623	CaLG6	CaSNP4962	79.8	scaffold01920	50879
4624	CaLG6	TA176	79.87	-	-
4625	CaLG6	CaSNP2717	80.16	scaffold01492	63768
4626	CaLG6	CaSNP79	80.93	-	-
4627	CaLG6	CaTMS883	80.96	-	-
4628	CaLG6	CaTSNP7903	80.99	9204062	8839
4629	CaLG6	CaSNP879	81.24	-	-
4630	CaLG6	TR44	81.24	-	-
4631	CaLG6	CaSNP489	81.26	-	-
4632	CaLG6	CaSNP604	81.44	-	-
4633	CaLG6	CaTMS588	81.52	-	-
4634	CaLG6	CaSNP5068	81.61	-	-
4635	CaLG6	CaSNP1971	81.91	scaffold00306	206891

4636	CaLG6	CaSNP3458	81.91	-	-
4637	CaLG6	CaSNP4883	81.91	-	-
4638	CaLG6	CaTSNP8173	82.13	-	-
4639	CaLG6	CaSNP2315	82.2	scaffold00225	227426
4640	CaLG6	NCPGR156	82.21	-	-
4641	CaLG6	CaSNP3200	82.24	9200105	30689
4642	CaLG6	CaSNP2648	82.25	-	-
4643	CaLG6	CaSNP3565	82.25	9198296	23545
4644	CaLG6	CaSNP2097	82.27	scaffold01457	70998
4645	CaLG6	CaSNP4046	82.27	-	-
4646	CaLG6	CaSNP2748	82.3	scaffold00482	174561
4647	CaLG6	CaSNP3656	82.3	-	-
4648	CaLG6	CaSNP2022	82.3	-	-
4649	CaLG6	CaSNP3105	82.31	scaffold00925	105883
4650	CaLG6	CaTSNP7409	82.35	-	-
4651	CaLG6	CaSNP3122	82.37	scaffold01876	58207
4652	CaLG6	CaTSNP6345	82.4	-	-
4653	CaLG6	CaSNP3253	82.41	9196921	17868
4654	CaLG6	CaTSNP8310	82.42	-	-
4655	CaLG6	CaTSNP7500	82.43	-	-
4656	CaLG6	CaTSNP6168	82.43	-	-
4657	CaLG6	CaTMS561	82.45	-	-
4658	CaLG6	CaSNP2112	82.46	-	-
4659	CaLG6	CaTSNP8228	82.46	-	-
4660	CaLG6	CaSNP2076	82.47	-	-
4661	CaLG6	CaTSNP7453	82.48	-	-
4662	CaLG6	CaTSNP8167	82.49	-	-
4663	CaLG6	CaTSNP8949	82.49	scaffold01121	109815
4664	CaLG6	CaTSNP6086	82.5	-	-

4665	CaLG6	CaTSNP7208	82.5	-	-
4666	CaLG6	CaTSNP7512	82.5	-	-
4667	CaLG6	CaTSNP7507	82.52	-	-
4668	CaLG6	CaTSNP6163	82.54	-	-
4669	CaLG6	CaTSNP8673	82.54	-	-
4670	CaLG6	CaTSNP6565	82.55	-	-
4671	CaLG6	CaTSNP6640	82.55	-	-
4672	CaLG6	CaTSNP7646	82.55	-	-
4673	CaLG6	CaTSNP9029	82.57	-	-
4674	CaLG6	CaTSNP7835	82.58	-	-
4675	CaLG6	CaSNP3157	82.58	scaffold00626	136970
4676	CaLG6	CaSNP1969	82.6	scaffold00232	229579
4677	CaLG6	CaSNP1993	82.6	-	-
4678	CaLG6	CaSNP2058	82.6	-	-
4679	CaLG6	CaTSNP6476	82.6	scaffold00253	219505
4680	CaLG6	CaTSNP6894	82.6	-	-
4681	CaLG6	CaSNP2257	82.6	-	-
4682	CaLG6	CaSNP2323	82.6	-	-
4683	CaLG6	CaSNP2938	82.6	-	-
4684	CaLG6	CaTSNP7402	82.61	-	-
4685	CaLG6	CaTSNP8664	82.61	9131385	32715
4686	CaLG6	CaTSNP7542	82.62	scaffold00853	108279
4687	CaLG6	CaTSNP8114	82.62	-	-
4688	CaLG6	CaTSNP8495	82.62	-	-
4689	CaLG6	CaTSNP8939	82.62	-	-
4690	CaLG6	CaTSNP8341	82.62	-	-
4691	CaLG6	CaSNP4364	82.65	-	-
4692	CaLG6	CaSNP2762	82.67	-	-
4693	CaLG6	CaTSNP6947	82.67	-	-

4694	CaLG6	CaSNP1961	82.7	-	-
4695	CaLG6	CaTSNP7162	82.72	-	-
4696	CaLG6	CaTSNP9123	82.72	-	-
4697	CaLG6	CaTSNP8961	82.73	scaffold00603	127406
4698	CaLG6	CaTSNP7383	82.73	-	-
4699	CaLG6	CaTSNP8718	82.76	-	-
4700	CaLG6	CaTSNP7607	82.8	-	-
4701	CaLG6	CaTSNP6115	82.8	-	-
4702	CaLG6	CaTSNP7158	82.8	-	-
4703	CaLG6	CaTSNP8364	82.8	-	-
4704	CaLG6	CaTSNP8712	82.8	-	-
4705	CaLG6	CaTSNP6112	82.81	-	-
4706	CaLG6	CaTSNP6318	82.81	-	-
4707	CaLG6	CaTSNP9041	82.81	-	-
4708	CaLG6	TR7	82.82	-	-
4709	CaLG6	CaTSNP8054	82.84	-	-
4710	CaLG6	CaTSNP8278	82.85	scaffold01729	62700
4711	CaLG6	CaTSNP8964	82.85	-	-
4712	CaLG6	CaTSNP8951	82.91	-	-
4713	CaLG6	CaTSNP6462	82.98	-	-
4714	CaLG6	CaTSNP7900	82.98	-	-
4715	CaLG6	CaSNP5066	83.17	-	-
4716	CaLG6	CaTSNP8662	83.47	-	-
4717	CaLG6	CaSNP591	83.49	-	-
4718	CaLG6	CaTSNP9015	83.54	-	-
4719	CaLG6	CaTSNP6025	83.56	-	-
4720	CaLG6	CaTSNP8155	83.58	scaffold00102	322864
4721	CaLG6	CaTSNP7133	83.61	-	-
4722	CaLG6	CaTSNP8724	83.61	-	-

4723	CaLG6	CaTSNP7233	83.67	-	-
4724	CaLG6	CaSNP3241	83.67	-	-
4725	CaLG6	CaSNP2625	83.69	-	-
4726	CaLG6	CaSNP2735	83.7	-	-
4727	CaLG6	CaSNP2651	83.72	-	-
4728	CaLG6	CaTSNP8223	83.74	-	-
4729	CaLG6	CaTSNP8625	83.74	scaffold04106	17887
4730	CaLG6	CaTSNP6593	83.77	-	-
4731	CaLG6	CaTSNP7938	83.77	-	-
4732	CaLG6	CaSNP4458	83.89	scaffold02727	34889
4733	CaLG6	CaSNP2096	83.96	scaffold00435	185730
4734	CaLG6	CaSNP4557	83.96	-	-
4735	CaLG6	CaSNP2223	83.96	-	-
4736	CaLG6	CaSNP5057	83.96	-	-
4737	CaLG6	CaTSNP7787	83.98	-	-
4738	CaLG6	TA22	84.08	-	-
4739	CaLG6	CaSNP667	84.2	-	-
4740	CaLG6	CaSNP3207	84.22	-	-
4741	CaLG6	CEST25	84.22	-	-
4742	CaLG6	CaTSNP6195	84.34	-	-
4743	CaLG6	CaTSNP7364	84.34	-	-
4744	CaLG6	CaTSNP8355	84.34	-	-
4745	CaLG6	CaTSNP6236	84.34	-	-
4746	CaLG6	CaTSNP6785	84.42	-	-
4747	CaLG6	CaTSNP8989	84.65	-	-
4748	CaLG6	NCPGR177	84.71	-	-
4749	CaLG6	TR1	84.82	-	-
4750	CaLG6	NCPGR93	84.85	scaffold00213	243812
4751	CaLG6	CaTSNP8262	85.15	-	-

4752	CaLG6	CaSNP417	85.15	-	-
4753	CaLG6	NCPGR267	85.16	-	-
4754	CaLG6	CaTSNP6284	85.28	scaffold00776	124641
4755	CaLG6	TA80	85.43	-	-
4756	CaLG6	NCPGR206	85.5	-	-
4757	CaLG6	CaGMS1238	85.51	-	-
4758	CaLG6	CaGMS1247	85.51	-	-
4759	CaLG6	CaTSNP7464	85.77	-	-
4760	CaLG6	CaTSNP7139	85.98	-	-
4761	CaLG6	NCPGR259	86.16	scaffold02522	36231
4762	CaLG6	CaSNP488	86.19	-	-
4763	CaLG6	CaSNP2038	86.24	scaffold01798	54477
4764	CaLG6	NCPGR139	86.3	-	-
4765	CaLG6	CaTSNP7251	86.35	-	-
4766	CaLG6	CaSNP3618	86.37	-	-
4767	CaLG6	CaSNP287	86.37	-	-
4768	CaLG6	CaTMS945	86.53	-	-
4769	CaLG6	NCPGR155	86.56	-	-
4770	CaLG6	CaTSNP6515	86.7	-	-
4771	CaLG6	CaTSNP8266	86.75	scaffold01481	71734
4772	CaLG6	CaSNP902	86.82	-	-
4773	CaLG6	CaTSNP6434	86.86	-	-
4774	CaLG6	NCPGR229	86.93	scaffold02846	47691
4775	CaLG6	CaTSNP8936	87.04	-	-
4776	CaLG6	CaTSNP6843	87.09	-	-
4777	CaLG6	CaTSNP8627	87.1	-	-
4778	CaLG6	CaTSNP6680	87.1	-	-
4779	CaLG6	PIP40	87.15	-	-
4780	CaLG6	CaTSNP8024	87.18	-	-

4781	CaLG6	CaTSNP6118	87.24	-	-
4782	CaLG6	CESSR71	87.29	-	-
4783	CaLG6	CaTSNP6158	87.31	-	-
4784	CaLG6	CaTSNP6758	87.34	-	-
4785	CaLG6	CaSNP3993	87.47	-	-
4786	CaLG6	CaSNP392	87.51	-	-
4787	CaLG6	CaTSNP6733	87.56	scaffold01289	89071
4788	CaLG6	CaTSNP8211	87.59	-	-
4789	CaLG6	CaTSNP7627	87.6	-	-
4790	CaLG6	CaSNP649	87.65	-	-
4791	CaLG6	CaTSNP7571	87.65	-	-
4792	CaLG6	CaTSNP8946	87.89	scaffold01455	65597
4793	CaLG6	CaTSNP8987	87.94	scaffold00411	173754
4794	CaLG6	CaTSNP6377	88	9208135	54675
4795	CaLG6	CaTSNP7480	88	scaffold00886	133080
4796	CaLG6	CaSNP4896	88.04	9106604	36114
4797	CaLG6	CaSNP2273	88.04	scaffold00340	188432
4798	CaLG6	CaTSNP6692	88.05	9111991	37958
4799	CaLG6	CaTSNP6166	88.07	-	-
4800	CaLG6	CaTSNP6591	88.07	-	-
4801	CaLG6	CaTSNP8172	88.07	-	-
4802	CaLG6	CaTSNP7176	88.13	-	-
4803	CaLG6	CaTSNP7019	88.31	-	-
4804	CaLG6	CaTSNP7462	88.31	9115979	12046
4805	CaLG6	CaTSNP7838	88.31	-	-
4806	CaLG6	CaTSNP8986	88.36	-	-
4807	CaLG6	CaTSNP6178	88.51	-	-
4808	CaLG6	CaTSNP6229	88.51	-	-
4809	CaLG6	CaSNP4632	88.6	-	-

4810	CaLG6	CaSNP2159	88.61	scaffold00684	130864
4811	CaLG6	CaSNP313	88.66	scaffold00078	429570
4812	CaLG6	CaSNP3560	88.66	-	-
4813	CaLG6	CaSNP668	88.71	-	-
4814	CaLG6	CaTSNP6009	88.71	-	-
4815	CaLG6	CaSNP1921	88.73	-	-
4816	CaLG6	CaTSNP7510	88.77	-	-
4817	CaLG6	CaTSNP7914	88.78	-	-
4818	CaLG6	CaSNP794	88.78	-	-
4819	CaLG6	CaTSNP6695	88.79	-	-
4820	CaLG6	CaTSNP9039	88.8	-	-
4821	CaLG6	CaTSNP7620	88.81	-	-
4822	CaLG6	CaTSNP6748	88.81	-	-
4823	CaLG6	CaTSNP6228	88.85	-	-
4824	CaLG6	CaTSNP7597	88.85	-	-
4825	CaLG6	CaTSNP7737	88.86	-	-
4826	CaLG6	CaSNP3133	88.87	-	-
4827	CaLG6	CaTSNP7291	88.9	9213386	48801
4828	CaLG6	CaTSNP7691	88.9	-	-
4829	CaLG6	CaSNP178	88.91	-	-
4830	CaLG6	CaSNP4343	88.91	-	-
4831	CaLG6	CaSNP2049	88.93	scaffold00916	97792
4832	CaLG6	CaTSNP7797	89.15	-	-
4833	CaLG6	CaSNP2505	89.23	scaffold01502	68795
4834	CaLG6	CaTSNP7249	89.27	-	-
4835	CaLG6	CaSNP2800	89.28	-	-
4836	CaLG6	CaSNP3770	89.28	scaffold03478	26564
4837	CaLG6	CaSNP2101	89.28	scaffold01183	83807
4838	CaLG6	CaSNP3057	89.28	scaffold00590	140198

4839	CaLG6	CaSNP3900	89.28	-	-
4840	CaLG6	CaSNP4276	89.28	-	-
4841	CaLG6	CaSNP2661	89.3	-	-
4842	CaLG6	CaTSNP6696	89.33	-	-
4843	CaLG6	CaTSNP6620	89.33	-	-
4844	CaLG6	CaTSNP7084	89.33	-	-
4845	CaLG6	CaSNP9	89.37	-	-
4846	CaLG6	CaTSNP6256	89.39	9200797	110926
4847	CaLG6	CaTSNP7779	89.43	scaffold00675	124190
4848	CaLG6	CaTSNP7837	89.43	-	-
4849	CaLG6	CaSNP2337	89.44	scaffold00719	125657
4850	CaLG6	CaTSNP6108	89.44	-	-
4851	CaLG6	CaTSNP7423	89.46	scaffold02005	49380
4852	CaLG6	CaSNP1975	89.47	-	-
4853	CaLG6	CaTSNP8644	89.49	scaffold02223	56969
4854	CaLG6	CaSNP2438	89.5	9093537	18330
4855	CaLG6	CaSNP2234	89.51	scaffold00009	856055
4856	CaLG6	CaTSNP6644	89.52	scaffold00215	238861
4857	CaLG6	CaTSNP7052	89.52	scaffold00508	149335
4858	CaLG6	CaTSNP7147	89.52	-	-
4859	CaLG6	CaTSNP7593	89.52	-	-
4860	CaLG6	CaTSNP8229	89.52	scaffold00135	280082
4861	CaLG6	CaTSNP7414	89.53	-	-
4862	CaLG6	CaTSNP6119	89.54	-	-
4863	CaLG6	CaTSNP7821	89.54	-	-
4864	CaLG6	CaSNP2913	89.58	-	-
4865	CaLG6	CaSNP4037	89.58	-	-
4866	CaLG6	CaTSNP8312	89.61	-	-
4867	CaLG6	CaSNP2142	89.63	-	-

4868	CaLG6	CaSNP3508	89.65	-	-
4869	CaLG6	CaSNP4525	89.65	-	-
4870	CaLG6	CaSNP4216	89.65	-	-
4871	CaLG6	CaSNP3185	89.73	-	-
4872	CaLG6	CaSNP2499	89.88	-	-
4873	CaLG6	CaSNP2	89.88	-	-
4874	CaLG6	CaSNP825	89.91	-	-
4875	CaLG6	CaSNP857	89.91	-	-
4876	CaLG6	CaSNP2267	89.93	-	-
4877	CaLG6	CaSNP2590	89.93	scaffold01575	67407
4878	CaLG6	CaTSNP8423	89.94	-	-
4879	CaLG6	CaSNP2828	89.95	scaffold01335	77470
4880	CaLG6	CaSNP2622	89.97	scaffold00022	621728
4881	CaLG6	CaSNP2988	90.03	scaffold00691	125350
4882	CaLG6	CaSNP32	90.05	-	-
4883	CaLG6	CaSNP638	90.05	-	-
4884	CaLG6	CaSNP914	90.07	scaffold01044	101763
4885	CaLG6	CaTSNP7099	90.23	-	-
4886	CaLG6	CaTSNP8514	90.23	-	-
4887	CaLG6	CaTSNP7107	90.23	-	-
4888	CaLG6	CaSNP3552	90.44	scaffold05769	10168
4889	CaLG6	CaSNP2077	90.49	scaffold00505	161647
4890	CaLG6	CaSNP3325	90.49	scaffold01271	75758
4891	CaLG6	CaSNP723	90.49	-	-
4892	CaLG6	CaTSNP6324	90.55	-	-
4893	CaLG6	CaTMS828	90.56	-	-
4894	CaLG6	CaSNP3931	90.56	-	-
4895	CaLG6	CaTSNP6944	90.58	-	-
4896	CaLG6	CaTSNP7144	90.58	-	-

4897	CaLG6	CaTSNP6824	90.6	9189824	23916
4898	CaLG6	CaTSNP7487	90.6	scaffold00197	248671
4899	CaLG6	CaTSNP8779	90.6	scaffold00261	205662
4900	CaLG6	CaSNP133	90.61	-	-
4901	CaLG6	CaTSNP7745	90.62	-	-
4902	CaLG6	CaTSNP6589	90.62	-	-
4903	CaLG6	CaTSNP6817	90.75	scaffold00363	181949
4904	CaLG6	CaSNP2094	90.75	-	-
4905	CaLG6	CaSNP2959	90.82	-	-
4906	CaLG6	CaSNP3164	90.82	-	-
4907	CaLG6	CaSNP3281	90.82	-	-
4908	CaLG6	CaSNP5115	90.82	scaffold00602	166877
4909	CaLG6	CaSNP780	90.87	scaffold00766	115282
4910	CaLG6	CaSNP2353	90.99	scaffold00579	144533
4911	CaLG6	CaSNP3147	91.11	scaffold01774	66364
4912	CaLG6	CaSNP2582	91.19	-	-
4913	CaLG6	CaSNP3481	91.24	scaffold01251	92889
4914	CaLG6	CaSNP2911	91.28	scaffold00456	181898
4915	CaLG6	CaSNP4751	91.28	scaffold01200	80375
4916	CaLG6	CaSNP3389	91.3	scaffold01037	109524
4917	CaLG6	CaSNP586	91.66	-	-
4918	CaLG6	CaSNP597	91.88	-	-
4919	CaLG6	CaTSNP9105	92.54	scaffold00943	103606
4920	CaLG6	CaTSNP7029	92.62	9214457	21218
4921	CaLG6	CaTSNP7949	92.62	scaffold00247	228431
4922	CaLG6	CaSNP741	92.89	scaffold00596	158197
4923	CaLG6	CaSNP2031	92.92	-	-
4924	CaLG6	CaSNP2716	92.98	9202099	26494
4925	CaLG6	CaSNP3701	93.01	-	-

4926	CaLG6	CaSNP275	93.03	-	-
4927	CaLG6	CaTSNP6433	93.06	-	-
4928	CaLG6	CaTSNP6669	93.1	scaffold00477	158842
4929	CaLG6	CaTSNP8344	93.1	-	-
4930	CaLG6	CaSNP853	93.19	scaffold01664	65602
4931	CaLG6	CaTSNP8467	93.19	-	-
4932	CaLG6	CaSNP252	93.2	-	-
4933	CaLG6	CaSNP548	93.2	-	-
4934	CaLG6	CaSNP865	93.2	-	-
4935	CaLG6	CaSNP388	93.22	-	-
4936	CaLG6	CaSNP885	93.23	-	-
4937	CaLG6	CaSNP302	93.25	-	-
4938	CaLG6	CaTSNP6368	93.26	-	-
4939	CaLG6	CaTSNP8711	93.26	-	-
4940	CaLG6	CaSNP274	93.27	scaffold00647	149324
4941	CaLG6	CaSNP531	93.27	-	-
4942	CaLG6	CaSNP685	93.27	-	-
4943	CaLG6	CaSNP812	93.27	scaffold01140	79669
4944	CaLG6	CaSNP764	93.27	-	-
4945	CaLG6	CaSNP160	93.31	scaffold01824	53557
4946	CaLG6	CaTSNP7844	93.32	scaffold00818	109026
4947	CaLG6	CaSNP34	93.33	scaffold01378	82707
4948	CaLG6	CaSNP639	93.33	-	-
4949	CaLG6	ESNP93	93.33	-	-
4950	CaLG6	CaTSNP7934	93.35	scaffold01354	68854
4951	CaLG6	CaTSNP7194	93.41	scaffold00146	266812
4952	CaLG6	CaTSNP8509	93.41	scaffold01741	64916
4953	CaLG6	CaTSNP6627	93.44	-	-
4954	CaLG6	CaTSNP7625	93.44	scaffold01257	74170

4955	CaLG6	CaTSNP7920	93.44	-	-
4956	CaLG6	CaTSNP8481	93.45	-	-
4957	CaLG6	CaSNP1976	93.47	scaffold00236	279539
4958	CaLG6	CaTSNP6403	93.49	-	-
4959	CaLG6	CaSNP2761	93.5	-	-
4960	CaLG6	CaSNP3419	93.5	-	-
4961	CaLG6	CaSNP4676	93.5	scaffold03453	23429
4962	CaLG6	CaSNP2019	93.51	-	-
4963	CaLG6	CaTSNP8016	93.53	scaffold01281	76639
4964	CaLG6	CaSNP5100	93.79	scaffold06338	8730
4965	CaLG6	CaSNP2341	93.8	-	-
4966	CaLG6	CaTSNP6828	93.83	scaffold01539	78308
4967	CaLG6	CaSNP2620	93.85	-	-
4968	CaLG6	CaTSNP7660	93.85	9206634	18470
4969	CaLG6	CaSNP2190	93.88	-	-
4970	CaLG6	CaTSNP7775	93.89	scaffold00343	211277
4971	CaLG6	CaSNP5126	93.93	-	-
4972	CaLG6	CaTSNP6740	94.08	scaffold00251	233382
4973	CaLG6	CaTSNP6772	94.08	scaffold01847	66206
4974	CaLG6	CaTSNP8693	94.08	-	-
4975	CaLG6	CaTSNP6211	94.08	-	-
4976	CaLG6	CaTSNP6212	94.08	-	-
4977	CaLG6	CaTSNP8360	94.08	-	-
4978	CaLG6	CaTSNP8301	94.08	scaffold01883	51603
4979	CaLG6	CaTSNP7831	94.08	-	-
4980	CaLG6	CaTSNP7979	94.1	9182029	31513
4981	CaLG6	CaTSNP8150	94.1	-	-
4982	CaLG6	CaTSNP9007	94.1	-	-
4983	CaLG6	CaTSNP6111	94.1	-	-

4984	CaLG6	CaTSNP8372	94.1	-	-
4985	CaLG6	CaSNP4293	94.18	-	-
4986	CaLG6	CaSNP3351	94.32	-	-
4987	CaLG6	CaSNP2411	94.43	scaffold03166	27938
4988	CaLG6	CaSNP3118	94.46	-	-
4989	CaLG6	CaSNP4733	94.47	scaffold07313	6963
4990	CaLG6	CaSNP3893	94.48	scaffold02926	30109
4991	CaLG6	CaSNP2531	94.48	scaffold00080	439549
4992	CaLG6	CaSNP2398	94.48	-	-
4993	CaLG6	CaSNP2560	94.48	-	-
4994	CaLG6	CaSNP2788	94.48	scaffold01210	91745
4995	CaLG6	CaSNP2841	94.48	9082934	59150
4996	CaLG6	CaSNP3166	94.48	9215454	34605
4997	CaLG6	CaSNP4708	94.48	-	-
4998	CaLG6	CaSNP2778	94.48	scaffold02446	37612
4999	CaLG6	CaSNP2055	94.49	-	-
5000	CaLG6	CaSNP2181	94.49	-	-
5001	CaLG6	CaSNP4021	94.49	-	-
5002	CaLG6	CaSNP2678	94.81	-	-
5003	CaLG6	CaSNP3764	94.92	-	-
5004	CaLG6	CaSNP2345	94.98	scaffold01159	80248
5005	CaLG6	CaSNP3710	95.01	scaffold01791	58855
5006	CaLG6	CaSNP5141	95.01	scaffold02282	62789
5007	CaLG6	CaSNP3305	95.01	scaffold01032	87953
5008	CaLG6	CaSNP4455	95.03	scaffold01932	49241
5009	CaLG6	CaSNP4580	95.07	-	-
5010	CaLG6	CaSNP4618	95.11	-	-
5011	CaLG6	CaSNP3934	95.11	scaffold01962	78491
5012	CaLG6	CaSNP3538	95.12	scaffold01255	77048

5013	CaLG6	CaSNP4516	95.12	9195961	7136
5014	CaLG6	CaSNP3528	95.13	-	-
5015	CaLG6	CaTSNP6437	95.13	-	-
5016	CaLG6	CaSNP3775	95.13	scaffold01192	102228
5017	CaLG6	CaSNP3065	95.13	scaffold01776	59083
5018	CaLG6	CaSNP3609	95.13	scaffold00678	148643
5019	CaLG6	CaSNP4120	95.13	scaffold02088	55702
5020	CaLG6	CaSNP4594	95.13	scaffold05646	10633
5021	CaLG6	CaSNP4655	95.13	scaffold05893	9846
5022	CaLG6	CaSNP4755	95.13	scaffold02442	62654
5023	CaLG6	CaSNP4786	95.13	scaffold00970	98950
5024	CaLG6	CaSNP4487	95.14	scaffold00855	118071
5025	CaLG6	CaSNP2751	95.15	scaffold00620	136563
5026	CaLG6	CaTSNP7649	95.15	scaffold02939	29904
5027	CaLG6	CaTSNP9011	95.15	-	-
5028	CaLG6	CaSNP3781	95.15	scaffold03773	36503
5029	CaLG6	CaSNP4847	95.15	-	-
5030	CaLG6	CaTSNP8901	95.15	scaffold01093	89294
5031	CaLG6	CaTSNP6844	95.15	-	-
5032	CaLG6	CaTSNP8322	95.15	-	-
5033	CaLG6	CaTSNP6588	95.15	scaffold00552	144278
5034	CaLG6	CaTSNP6745	95.15	-	-
5035	CaLG6	CaSNP3223	95.15	scaffold02422	50353
5036	CaLG6	CaSNP2963	95.16	-	-
5037	CaLG6	CaSNP2418	95.16	scaffold01066	84501
5038	CaLG6	CaSNP2547	95.16	9199582	23699
5039	CaLG6	CaSNP4332	95.17	scaffold01794	52787
5040	CaLG6	CaSNP2220	95.17	scaffold02534	42272
5041	CaLG6	CaSNP1990	95.17	scaffold00619	139561

5042	CaLG6	CaSNP2075	95.17	-	-
5043	CaLG6	CaSNP2157	95.17	scaffold03043	38123
5044	CaLG6	CaSNP2393	95.17	scaffold00578	146719
5045	CaLG6	CaSNP2609	95.17	scaffold00836	102346
5046	CaLG6	CaSNP2699	95.17	scaffold02169	53005
5047	CaLG6	CaSNP2710	95.17	scaffold01355	74915
5048	CaLG6	CaSNP2715	95.17	scaffold01509	65193
5049	CaLG6	CaSNP2786	95.17	-	-
5050	CaLG6	CaSNP3055	95.17	-	-
5051	CaLG6	CaSNP3059	95.17	scaffold01357	77698
5052	CaLG6	CaSNP3066	95.17	-	-
5053	CaLG6	CaSNP3114	95.17	-	-
5054	CaLG6	CaSNP3780	95.17	scaffold01167	87540
5055	CaLG6	CaSNP3992	95.17	scaffold02641	42344
5056	CaLG6	CaSNP4025	95.17	9214727	49433
5057	CaLG6	CaSNP4077	95.17	scaffold03406	28793
5058	CaLG6	CaSNP4096	95.17	-	-
5059	CaLG6	CaSNP4140	95.17	9204852	11118
5060	CaLG6	CaSNP4248	95.17	scaffold03548	32021
5061	CaLG6	CaSNP4639	95.17	scaffold01518	81621
5062	CaLG6	CaSNP4825	95.17	scaffold01120	118823
5063	CaLG6	CaSNP4844	95.17	-	-
5064	CaLG6	CaSNP5046	95.17	scaffold01477	79566
5065	CaLG6	CaSNP5067	95.17	scaffold00094	373169
5066	CaLG6	CaSNP5080	95.17	-	-
5067	CaLG6	CaSNP2113	95.18	scaffold00618	172261
5068	CaLG6	CaSNP3268	95.18	scaffold07657	7757
5069	CaLG6	CaSNP3437	95.18	-	-
5070	CaLG6	CaSNP3728	95.18	scaffold00257	229557

5071	CaLG6	CaSNP4133	95.18	scaffold03004	36389
5072	CaLG6	CaSNP2354	95.18	scaffold01097	87368
5073	CaLG6	CaSNP2823	95.18	scaffold00504	160245
5074	CaLG6	CaSNP2995	95.18	scaffold02694	42994
5075	CaLG6	CaSNP3013	95.18	scaffold02307	49046
5076	CaLG6	CaSNP3296	95.18	9205465	20811
5077	CaLG6	CaSNP3853	95.18	scaffold02318	40359
5078	CaLG6	CaSNP3969	95.18	scaffold01347	80598
5079	CaLG6	CaSNP4007	95.18	scaffold00388	179038
5080	CaLG6	CaSNP4845	95.18	scaffold00758	141769
5081	CaLG6	CaSNP5124	95.18	scaffold00842	136584
5082	CaLG6	CaSNP4601	95.18	-	-
5083	CaLG6	CaSNP4581	95.18	scaffold01331	86312
5084	CaLG6	CaSNP2247	95.18	scaffold00259	234725
5085	CaLG6	CaSNP3685	95.18	9214184	13221
5086	CaLG6	CaSNP4086	95.18	-	-
5087	CaLG6	CaSNP3415	95.18	9203133	22223
5088	CaLG6	CaSNP3382	95.19	9207196	29355
5089	CaLG6	CaSNP3641	95.19	scaffold02401	39224
5090	CaLG6	CaSNP3657	95.19	scaffold02840	40313
5091	CaLG6	CaSNP4222	95.19	scaffold02785	36890
5092	CaLG6	CaSNP4444	95.19	contig66341	1010
5093	CaLG6	CaSNP3529	95.19	-	-
5094	CaLG6	CaTSNP8187	95.19	-	-
5095	CaLG6	CaSNP4290	95.2	9215297	16367
5096	CaLG6	CaSNP3769	95.2	scaffold03024	29500
5097	CaLG6	CaSNP4521	95.2	scaffold03373	33925
5098	CaLG6	CaSNP3687	95.2	scaffold06892	7913
5099	CaLG6	CaSNP4441	95.2	scaffold01734	100035

5100	CaLG6	CaSNP3355	95.2	scaffold01974	56879
5101	CaLG6	CaSNP4993	95.21	scaffold01222	89482
5102	CaLG6	CaSNP3591	95.21	scaffold00118	308465
5103	CaLG6	CaSNP3418	95.21	-	-
5104	CaLG6	CaSNP4743	95.21	scaffold04047	27142
5105	CaLG6	CaSNP2416	95.22	scaffold00809	107332
5106	CaLG6	CaSNP4579	95.22	scaffold01334	93808
5107	CaLG6	CaSNP2521	95.22	scaffold01236	89540
5108	CaLG6	CaSNP4104	95.22	-	-
5109	CaLG6	CaSNP4391	95.22	scaffold01742	71003
5110	CaLG6	CaSNP2456	95.23	scaffold00611	158074
5111	CaLG6	CaSNP2968	95.23	-	-
5112	CaLG6	CaSNP4590	95.24	scaffold01306	76936
5113	CaLG6	CaSNP5001	95.24	scaffold02628	56746
5114	CaLG6	CaSNP3298	95.24	-	-
5115	CaLG6	CaSNP4877	95.24	scaffold01341	80215
5116	CaLG6	CaSNP4765	95.24	scaffold01246	84735
5117	CaLG6	CaSNP2467	95.25	-	-
5118	CaLG6	CaSNP3310	95.25	scaffold01338	73714
5119	CaLG6	CaSNP3741	95.25	scaffold00784	123032
5120	CaLG6	CaSNP5019	95.25	scaffold04405	15881
5121	CaLG6	CaSNP2405	95.25	scaffold00933	95839
5122	CaLG6	CaSNP3602	95.25	scaffold04286	30130
5123	CaLG6	CaSNP2102	95.25	scaffold00712	125672
5124	CaLG6	CaSNP3822	95.25	scaffold02351	47372
5125	CaLG6	CaSNP2338	95.25	scaffold00356	235581
5126	CaLG6	CaSNP2441	95.25	scaffold00896	99033
5127	CaLG6	CaSNP3042	95.25	-	-
5128	CaLG6	CaSNP3340	95.26	9198809	13419

5129	CaLG6	CaSNP2126	95.26	-	-
5130	CaLG6	CaSNP2248	95.26	-	-
5131	CaLG6	CaSNP3007	95.26	scaffold01184	99610
5132	CaLG6	CaSNP3041	95.26	scaffold01006	120297
5133	CaLG6	CaSNP3812	95.26	9206044	32764
5134	CaLG6	CaSNP4335	95.26	-	-
5135	CaLG6	CaSNP4382	95.26	9195006	28632
5136	CaLG6	CaSNP4314	95.26	scaffold02110	64188
5137	CaLG6	CaSNP4891	95.27	scaffold00685	139865
5138	CaLG6	CaSNP2906	95.27	scaffold01434	75678
5139	CaLG6	CaSNP4787	95.27	-	-
5140	CaLG6	CaSNP4688	95.27	scaffold01650	70945
5141	CaLG6	CaSNP3035	95.27	scaffold01048	109479
5142	CaLG6	CaSNP4384	95.27	scaffold00911	123672
5143	CaLG6	CaSNP4023	95.27	scaffold01851	56950
5144	CaLG6	CaSNP4207	95.27	scaffold02035	47614
5145	CaLG6	CaSNP4459	95.28	scaffold01571	79500
5146	CaLG6	CaSNP2763	95.28	scaffold01127	82761
5147	CaLG6	CaSNP2417	95.28	-	-
5148	CaLG6	CaSNP2537	95.28	-	-
5149	CaLG6	CaSNP2971	95.28	scaffold01510	64433
5150	CaLG6	CaSNP3031	95.28	scaffold00892	135072
5151	CaLG6	CaSNP3093	95.28	scaffold00697	143698
5152	CaLG6	CaSNP3308	95.28	-	-
5153	CaLG6	CaSNP3535	95.28	scaffold02396	57951
5154	CaLG6	CaSNP3714	95.28	scaffold01420	84146
5155	CaLG6	CaSNP3833	95.28	scaffold02428	57246
5156	CaLG6	CaSNP4061	95.28	scaffold01813	64301
5157	CaLG6	CaSNP4161	95.28	-	-

5158	CaLG6	CaSNP4236	95.28	scaffold01171	92774
5159	CaLG6	CaSNP4504	95.28	scaffold06441	8492
5160	CaLG6	CaSNP4815	95.28	scaffold02176	44871
5161	CaLG6	CaSNP4832	95.28	-	-
5162	CaLG6	CaSNP4836	95.28	scaffold02917	30902
5163	CaLG6	CaSNP4915	95.28	-	-
5164	CaLG6	CaSNP4937	95.28	scaffold01994	62473
5165	CaLG6	CaSNP5099	95.28	-	-
5166	CaLG6	CaSNP5148	95.29	scaffold03255	45758
5167	CaLG6	CaSNP2297	95.29	scaffold00352	182526
5168	CaLG6	CaSNP2962	95.29	scaffold05085	12476
5169	CaLG6	CaSNP3084	95.29	scaffold01872	55033
5170	CaLG6	CaSNP3171	95.29	scaffold00699	117925
5171	CaLG6	CaSNP3445	95.29	-	-
5172	CaLG6	CaSNP3546	95.29	scaffold05762	10534
5173	CaLG6	CaSNP3596	95.29	-	-
5174	CaLG6	CaSNP3613	95.29	scaffold00813	129783
5175	CaLG6	CaSNP3632	95.29	scaffold02311	43270
5176	CaLG6	CaSNP3640	95.29	scaffold00614	150700
5177	CaLG6	CaSNP3716	95.29	scaffold02130	43703
5178	CaLG6	CaSNP4187	95.29	scaffold02323	49619
5179	CaLG6	CaSNP4270	95.29	-	-
5180	CaLG6	CaSNP4397	95.29	scaffold00138	287076
5181	CaLG6	CaSNP4920	95.29	scaffold02019	49149
5182	CaLG6	CaSNP5078	95.29	scaffold02338	60405
5183	CaLG6	CaSNP4123	95.3	scaffold02032	55980
5184	CaLG6	CaSNP4547	95.3	scaffold03012	43692
5185	CaLG6	CaSNP2895	95.3	scaffold01733	54389
5186	CaLG6	CaSNP4963	95.31	scaffold00893	126707

5187	CaLG6	CaSNP2303	95.36	-	-
5188	CaLG6	CaSNP5089	95.36	scaffold01445	71299
5189	CaLG6	CaSNP3393	95.37	scaffold01899	52177
5190	CaLG6	CaSNP4941	95.37	9202992	14568
5191	CaLG6	CaSNP2425	95.38	scaffold01075	97286
5192	CaLG6	CaSNP2850	95.38	scaffold00560	137081
5193	CaLG6	CaSNP3516	95.38	scaffold02776	41097
5194	CaLG6	CaTSNP6367	95.38	-	-
5195	CaLG6	CaSNP2750	95.39	-	-
5196	CaLG6	CaSNP3229	95.39	-	-
5197	CaLG6	CaSNP3773	95.39	scaffold01671	67549
5198	CaLG6	CaSNP3945	95.39	scaffold02367	40711
5199	CaLG6	CaSNP4121	95.39	scaffold02380	48839
5200	CaLG6	CaSNP4273	95.39	-	-
5201	CaLG6	CaSNP4550	95.39	scaffold02851	35287
5202	CaLG6	CaSNP4586	95.39	-	-
5203	CaLG6	CaSNP2657	95.39	9208091	14358
5204	CaLG6	CaSNP4312	95.39	scaffold01565	65793
5205	CaLG6	CaSNP4950	95.4	-	-
5206	CaLG6	CaTSNP8337	95.4	scaffold01309	91421
5207	CaLG6	CaSNP3965	95.4	scaffold00912	96835
5208	CaLG6	CaSNP2255	95.4	scaffold00509	168228
5209	CaLG6	CaSNP4301	95.4	scaffold01042	89007
5210	CaLG6	CaSNP4098	95.42	scaffold01064	98118
5211	CaLG6	CaSNP4306	95.42	9192557	19500
5212	CaLG6	CaSNP2684	95.43	scaffold01427	72203
5213	CaLG6	CaTSNP8429	95.43	-	-
5214	CaLG6	CaSNP2431	95.43	scaffold01217	78934
5215	CaLG6	CaSNP2768	95.43	scaffold02204	44975

5216	CaLG6	CaSNP2986	95.43	scaffold01468	86259
5217	CaLG6	CaSNP3979	95.43	scaffold01713	65239
5218	CaLG6	CaSNP4257	95.43	scaffold01676	57306
5219	CaLG6	CaSNP4407	95.43	scaffold00654	171334
5220	CaLG6	CaSNP2721	95.43	9191830	26517
5221	CaLG6	CaSNP4495	95.44	scaffold01193	95056
5222	CaLG6	CaSNP4202	95.44	9189576	16550
5223	CaLG6	CaSNP4198	95.44	scaffold01722	73533
5224	CaLG6	CaTSNP7279	95.44	-	-
5225	CaLG6	CaTSNP7317	95.44	scaffold02869	34724
5226	CaLG6	CaTSNP7461	95.44	scaffold01039	96477
5227	CaLG6	CaSNP2116	95.44	-	-
5228	CaLG6	CaSNP2468	95.44	scaffold00568	139865
5229	CaLG6	CaSNP2548	95.44	scaffold01656	69657
5230	CaLG6	CaSNP3112	95.44	-	-
5231	CaLG6	CaSNP3239	95.44	scaffold01959	57750
5232	CaLG6	CaSNP3262	95.44	scaffold07906	6244
5233	CaLG6	CaSNP3482	95.44	scaffold08026	6111
5234	CaLG6	CaSNP3494	95.44	scaffold01045	93525
5235	CaLG6	CaSNP3678	95.44	scaffold01422	71053
5236	CaLG6	CaSNP3870	95.44	-	-
5237	CaLG6	CaSNP3958	95.44	-	-
5238	CaLG6	CaSNP4144	95.44	scaffold04367	16136
5239	CaLG6	CaSNP4178	95.44	scaffold01069	94465
5240	CaLG6	CaSNP4208	95.44	scaffold01685	56278
5241	CaLG6	CaSNP4424	95.44	scaffold01196	102910
5242	CaLG6	CaSNP4684	95.44	scaffold02746	32907
5243	CaLG6	CaSNP4721	95.44	-	-
5244	CaLG6	CaSNP5073	95.44	-	-

5245	CaLG6	CaSNP5131	95.45	-	-
5246	CaLG6	CaSNP4299	95.45	-	-
5247	CaLG6	CaSNP2355	95.46	scaffold00520	160735
5248	CaLG6	CaTSNP8511	95.46	-	-
5249	CaLG6	CaTSNP8714	95.46	-	-
5250	CaLG6	CaSNP4242	95.46	scaffold00513	198287
5251	CaLG6	CaTSNP6179	95.46	scaffold01278	85313
5252	CaLG6	CaTSNP6566	95.46	scaffold00480	151994
5253	CaLG6	CaTSNP6781	95.46	scaffold01205	89436
5254	CaLG6	CaTSNP7100	95.46	scaffold01103	96068
5255	CaLG6	CaTSNP7167	95.46	-	-
5256	CaLG6	CaTSNP7935	95.46	-	-
5257	CaLG6	CaTSNP8691	95.46	scaffold01548	72778
5258	CaLG6	CaTSNP8918	95.46	-	-
5259	CaLG6	CaTSNP9097	95.46	-	-
5260	CaLG6	CaTSNP9139	95.46	scaffold02274	58240
5261	CaLG6	CaTSNP9150	95.46	-	-
5262	CaLG6	CaTSNP6645	95.46	-	-
5263	CaLG6	CaTSNP7042	95.46	scaffold02137	53858
5264	CaLG6	CaTSNP7421	95.46	-	-
5265	CaLG6	CaTSNP7529	95.46	scaffold01344	77607
5266	CaLG6	CaTSNP6130	95.47	scaffold00640	146457
5267	CaLG6	CaTSNP6458	95.47	scaffold02832	31583
5268	CaLG6	CaTSNP6615	95.47	-	-
5269	CaLG6	CaTSNP6697	95.47	-	-
5270	CaLG6	CaTSNP7429	95.47	-	-
5271	CaLG6	CaTSNP7485	95.47	9087496	28333
5272	CaLG6	CaTSNP6205	95.47	-	-
5273	CaLG6	CaTSNP6351	95.47	-	-

5274	CaLG6	CaTSNP7345	95.47	scaffold02213	50747
5275	CaLG6	CaTSNP7860	95.47	scaffold02591	35112
5276	CaLG6	CaTSNP7948	95.47	-	-
5277	CaLG6	CaTSNP7999	95.47	scaffold01011	106232
5278	CaLG6	CaTSNP8081	95.47	-	-
5279	CaLG6	CaTSNP8635	95.47	-	-
5280	CaLG6	CaTSNP8638	95.47	scaffold01206	92599
5281	CaLG6	CaTSNP8861	95.47	scaffold00585	142598
5282	CaLG6	CaSNP3660	95.48	scaffold02617	38922
5283	CaLG6	CaTSNP7181	95.48	-	-
5284	CaLG6	CaTSNP6265	95.48	-	-
5285	CaLG6	CaTSNP6370	95.48	9201473	10518
5286	CaLG6	CaTSNP6480	95.48	-	-
5287	CaLG6	CaTSNP7454	95.48	-	-
5288	CaLG6	CaTSNP7533	95.48	scaffold02554	41409
5289	CaLG6	CaTSNP8675	95.48	-	-
5290	CaLG6	CaTSNP6242	95.49	-	-
5291	CaLG6	CaTSNP8778	95.49	-	-
5292	CaLG6	CaSNP2363	95.5	-	-
5293	CaLG6	CaTSNP6854	95.51	-	-
5294	CaLG6	CaTSNP7915	95.53	scaffold01629	70850
5295	CaLG6	CaTSNP7155	95.55	scaffold03081	28939
5296	CaLG6	CaTSNP7071	95.62	scaffold01853	50950
5297	CaLG6	CaTSNP8630	95.65	-	-
5298	CaLG6	CaTSNP8123	95.66	scaffold06371	8684
5299	CaLG6	CaTSNP7679	95.67	scaffold02704	34820
5300	CaLG6	CaTSNP8218	95.67	-	-
5301	CaLG6	CaSNP3369	95.83	scaffold08692	5416
5302	CaLG6	CaSNP4348	96.05	scaffold01549	63862

5303	CaLG6	CaTSNP6438	96.15	scaffold00921	117148
5304	CaLG6	CaGMS3	96.31	-	-
5305	CaLG6	CaTSNP8376	96.37	scaffold00436	155262
5306	CaLG6	CaTMS600	97.14	-	-
5307	CaLG6	CaTSNP8882	98.8	scaffold04064	30817
5308	CaLG6	CaSNP2306	102.2	scaffold00985	114206
5309	CaLG6	CaGMS1254	105.3	-	-
5310	CaLG6	CaSNP4739	106.7	9212181	5067
5311	CaLG6	PIP175	110.1	-	-
5312	CaLG6	PIP26	113.9	scaffold00547	144598
5313	CaLG6	PIP79	115.4	-	-
5314	CaLG6	PIP144	121.1	-	-
5315	CaLG6	PIP155	123	-	-
5316	CaLG6	CEST180	124.3	-	-
5317	CaLG6	CESSR186	126.3	-	-
5318	CaLG6	PIP85	127.7	-	-
5319	CaLG6	STMS12	131.3	-	-
5320	CaLG6	PIP41	131.9	-	-
5321	CaLG6	PIP30	138.5	-	-
5322	CaLG7	CaSNP2310	0	scaffold01504	71743
5323	CaLG7	CaSNP1983	0.358	scaffold00338	196972
5324	CaLG7	CaSNP2244	5.334	scaffold00405	172504
5325	CaLG7	CaSNP2644	5.532	-	-
5326	CaLG7	CaTSNP6138	7.377	scaffold00188	260221
5327	CaLG7	CaSNP2734	8.135	-	-
5328	CaLG7	CaSNP2350	8.471	9202761	10621
5329	CaLG7	NCPGR32	13.13	-	-
5330	CaLG7	CaSNP3485	13.83	scaffold02435	45364
5331	CaLG7	CaGMS473	13.9	-	-

5332	CaLG7	CaTMS911	14.26	-	-
5333	CaLG7	CaSNP3541	15.69	-	-
5334	CaLG7	CaSNP2305	15.73	scaffold00383	173027
5335	CaLG7	CaSNP3136	16.06	-	-
5336	CaLG7	CaSNP1958	16.1	scaffold00187	245173
5337	CaLG7	CaSNP4597	18.36	-	-
5338	CaLG7	CaSNP341	18.98	-	-
5339	CaLG7	CaSNP768	18.98	-	-
5340	CaLG7	CaSNP4241	19.09	contig53746	1611
5341	CaLG7	CaSNP4809	19.09	scaffold04022	30003
5342	CaLG7	CaSNP716	19.14	scaffold00665	118730
5343	CaLG7	CaSNP4879	19.43	scaffold01599	67792
5344	CaLG7	CaTSNP6217	19.55	-	-
5345	CaLG7	CaSNP642	19.57	scaffold00558	144513
5346	CaLG7	CaSNP2032	19.69	scaffold05407	11211
5347	CaLG7	CaSNP2260	19.69	9189465	85250
5348	CaLG7	CaSNP4415	19.69	scaffold00760	135432
5349	CaLG7	CaSNP4540	19.69	scaffold05667	10460
5350	CaLG7	CaSNP4685	19.69	scaffold00512	155884
5351	CaLG7	CaSNP2672	19.87	scaffold02648	48570
5352	CaLG7	CaSNP2805	19.91	scaffold02925	39282
5353	CaLG7	CaSNP4004	19.94	scaffold01040	95617
5354	CaLG7	CaSNP3924	20.23	scaffold02880	30933
5355	CaLG7	CEST93	20.23	-	-
5356	CaLG7	CaTSNP7665	20.57	scaffold00152	283972
5357	CaLG7	CaSNP3213	20.74	scaffold00945	118916
5358	CaLG7	CaSNP3464	20.74	scaffold00462	164300
5359	CaLG7	CaSNP2047	20.79	scaffold01243	75267
5360	CaLG7	CaSNP4209	20.79	scaffold01282	90022

5361	CaLG7	CaSNP4351	20.82	scaffold01821	52306
5362	CaLG7	CaSNP1995	20.87	9214040	41733
5363	CaLG7	CaSNP2629	20.91	scaffold00361	195950
5364	CaLG7	CaSNP2280	20.99	scaffold00446	165922
5365	CaLG7	CaSNP3143	20.99	-	-
5366	CaLG7	CaSNP3159	20.99	scaffold02179	45353
5367	CaLG7	CaSNP3431	20.99	-	-
5368	CaLG7	CaSNP4069	20.99	scaffold00605	150663
5369	CaLG7	CaSNP2245	21.04	-	-
5370	CaLG7	H2I104	21.05	-	-
5371	CaLG7	CaSNP1938	21.07	scaffold00307	198791
5372	CaLG7	CaSNP3752	21.08	scaffold02286	44293
5373	CaLG7	CaSNP3009	21.13	-	-
5374	CaLG7	CaSNP2385	21.18	scaffold00563	162141
5375	CaLG7	CaSNP2933	21.18	-	-
5376	CaLG7	CaSNP3980	21.2	-	-
5377	CaLG7	TA21	21.37	-	-
5378	CaLG7	CaSNP2083	22.91	scaffold00262	207492
5379	CaLG7	CaSNP3307	23.46	-	-
5380	CaLG7	CaSNP184	23.97	-	-
5381	CaLG7	CaSNP916	23.97	-	-
5382	CaLG7	CaGMS122	25.29	-	-
5383	CaLG7	CaGMS369	25.32	-	-
5384	CaLG7	CaGMS1298	25.63	scaffold11009	3827
5385	CaLG7	CaTMS1084	25.63	-	-
5386	CaLG7	GA102	25.7	-	-
5387	CaLG7	CaSNP2929	25.91	scaffold01424	66192
5388	CaLG7	NCPGR255	26.82	-	-
5389	CaLG7	CaTSNP7238	27.1	-	-

5390	CaLG7	CaTSNP8710	28.09	scaffold01312	83048
5391	CaLG7	CaSNP3488	28.3	scaffold01766	56285
5392	CaLG7	CaSNP802	28.98	-	-
5393	CaLG7	CaGMS286	29.97	scaffold05841	9986
5394	CaLG7	CaTMS762	30.2	-	-
5395	CaLG7	NCPGR130	31.15	scaffold01885	50984
5396	CaLG7	CaTMS939	31.19	-	-
5397	CaLG7	CaGMS190	31.41	-	-
5398	CaLG7	CaTMS767	31.42	-	-
5399	CaLG7	TA78	32	-	-
5400	CaLG7	H5G12	32.52	-	-
5401	CaLG7	CaSNP2039	33.02	9196050	29481
5402	CaLG7	TA18	33.41	-	-
5403	CaLG7	TA28	34.29	-	-
5404	CaLG7	CaSNP2743	34.42	-	-
5405	CaLG7	TS62	35.2	-	-
5406	CaLG7	CaTSNP7350	35.83	scaffold01482	64588
5407	CaLG7	PIP44	36.31	-	-
5408	CaLG7	CaSNP4634	36.56	9214218	29509
5409	CaLG7	TAA59	36.57	-	-
5410	CaLG7	CaSNP2626	36.65	-	-
5411	CaLG7	CaSNP3028	37.04	scaffold01058	89195
5412	CaLG7	TA140	37.3	-	-
5413	CaLG7	NCPGR52	37.43	-	-
5414	CaLG7	CaSNP726	37.67	9213361	21616
5415	CaLG7	TAA58	37.8	-	-
5416	CaLG7	CaSNP4577	37.91	scaffold00325	220224
5417	CaLG7	CaTSNP7514	37.94	scaffold00470	149467
5418	CaLG7	CaSNP2360	38.09	scaffold01955	51640

5419	CaLG7	CaSNP3784	38.31	scaffold03073	39119
5420	CaLG7	CaSNP4426	38.46	scaffold01316	88708
5421	CaLG7	CaSNP1953	39.07	-	-
5422	CaLG7	CaGMS1153	39.17	-	-
5423	CaLG7	CaSNP4319	39.29	scaffold03364	24707
5424	CaLG7	CaTSNP6964	39.39	scaffold01346	69223
5425	CaLG7	CaSNP2052	39.73	-	-
5426	CaLG7	CESSR118	39.86	scaffold00016	731423
5427	CaLG7	CaSNP2370	40.51	scaffold00488	176882
5428	CaLG7	CaSNP3578	40.51	scaffold02285	40508
5429	CaLG7	GAA44	40.89	-	-
5430	CaLG7	CaSNP1928	41.1	scaffold00598	151090
5431	CaLG7	CESSR121	42.22	-	-
5432	CaLG7	CaSNP905	43.06	-	-
5433	CaLG7	CaSNP383	44.86	scaffold00637	144516
5434	CaLG7	CaSNP156	45.03	-	-
5435	CaLG7	CaSNP562	45.32	-	-
5436	CaLG7	CaSNP165	45.65	-	-
5437	CaLG7	CaSNP683	45.81	scaffold00773	108334
5438	CaLG7	CaSNP284	45.89	-	-
5439	CaLG7	CaSNP799	45.97	scaffold00286	200550
5440	CaLG7	CaSNP195	45.98	scaffold01388	80509
5441	CaLG7	CaSNP722	45.98	-	-
5442	CaLG7	CaSNP671	46	scaffold00291	207615
5443	CaLG7	CaSNP31	46.04	9207294	90230
5444	CaLG7	CaSNP182	46.05	-	-
5445	CaLG7	CaSNP222	46.16	scaffold01008	103180
5446	CaLG7	CaSNP246	46.16	scaffold02642	41552
5447	CaLG7	CaSNP296	46.16	scaffold00549	176004

5448	CaLG7	CaSNP42	46.16	-	-
5449	CaLG7	CaSNP67	46.16	-	-
5450	CaLG7	CaSNP835	46.16	-	-
5451	CaLG7	CaSNP846	46.16	-	-
5452	CaLG7	CaSNP57	46.26	scaffold02100	44349
5453	CaLG7	CaSNP3725	46.44	scaffold02242	52436
5454	CaLG7	CaSNP3070	46.49	scaffold06623	9243
5455	CaLG7	CaSNP4642	46.49	-	-
5456	CaLG7	CaSNP694	46.53	scaffold01083	84947
5457	CaLG7	NCPGR249	46.61	scaffold01806	53608
5458	CaLG7	CaSNP3273	47.4	-	-
5459	CaLG7	PIP128	47.86	-	-
5460	CaLG7	CaSNP2572	48.27	scaffold00373	187937
5461	CaLG7	CaSNP4109	48.35	scaffold02207	49551
5462	CaLG7	CaSNP2944	48.37	scaffold01679	58942
5463	CaLG7	CaSNP4124	48.37	scaffold01660	77814
5464	CaLG7	CaSNP3562	48.6	-	-
5465	CaLG7	CaSNP4264	48.75	scaffold01537	73148
5466	CaLG7	CaSNP4405	48.75	-	-
5467	CaLG7	CaSNP3884	48.91	scaffold00790	129942
5468	CaLG7	CaSNP4782	49.15	-	-
5469	CaLG7	CaSNP3610	49.18	scaffold01322	97210
5470	CaLG7	CaSNP2086	49.21	scaffold06238	8968
5471	CaLG7	CaSNP4318	49.23	scaffold02142	50029
5472	CaLG7	CaSNP4373	49.23	-	-
5473	CaLG7	CaSNP5111	49.25	scaffold02141	68409
5474	CaLG7	CaSNP4095	49.25	scaffold00252	265597
5475	CaLG7	CaSNP2596	49.3	scaffold00828	104288
5476	CaLG7	CaSNP2996	49.3	scaffold00583	158908

5477	CaLG7	CaSNP3932	49.31	scaffold01669	65759
5478	CaLG7	CaSNP2635	49.35	-	-
5479	CaLG7	CaSNP4570	49.35	scaffold03119	41154
5480	CaLG7	CaSNP4768	49.35	scaffold01779	76104
5481	CaLG7	CaSNP4009	49.36	scaffold03351	30120
5482	CaLG7	CaSNP2774	49.37	scaffold01924	64455
5483	CaLG7	CaSNP3142	49.38	scaffold01035	106308
5484	CaLG7	CaSNP3039	49.39	scaffold01695	82110
5485	CaLG7	CaSNP3236	49.39	scaffold00978	111054
5486	CaLG7	CaSNP3836	49.39	9216246	36918
5487	CaLG7	CaSNP4378	49.39	9204670	39517
5488	CaLG7	CaSNP4392	49.39	scaffold01657	73278
5489	CaLG7	CaSNP4092	49.39	scaffold01985	64058
5490	CaLG7	CaSNP3335	49.41	scaffold01887	55488
5491	CaLG7	CaSNP2484	49.42	scaffold00400	196798
5492	CaLG7	CaSNP4282	49.42	scaffold03005	34539
5493	CaLG7	CaSNP3468	49.43	scaffold00897	121187
5494	CaLG7	CaSNP2931	49.43	scaffold01055	111392
5495	CaLG7	CaSNP2433	49.43	scaffold00953	102921
5496	CaLG7	CaSNP2726	49.43	scaffold00837	121403
5497	CaLG7	CaSNP4227	49.43	scaffold01124	88709
5498	CaLG7	CaSNP4433	49.43	scaffold02984	46417
5499	CaLG7	CaSNP5107	49.43	scaffold01301	92445
5500	CaLG7	CaSNP2412	49.45	scaffold01244	86938
5501	CaLG7	CaSNP3286	49.45	scaffold04011	18528
5502	CaLG7	CaSNP4131	49.45	scaffold01364	79366
5503	CaLG7	CaSNP4781	49.45	scaffold00662	153177
5504	CaLG7	CaSNP2591	49.45	scaffold01151	88352
5505	CaLG7	CaSNP4137	49.46	-	-

5506	CaLG7	CaSNP3490	49.46	scaffold01394	74024
5507	CaLG7	CaSNP2594	49.47	scaffold00597	164567
5508	CaLG7	CaSNP4861	49.47	scaffold00934	100412
5509	CaLG7	CaSNP2372	49.48	scaffold01324	74394
5510	CaLG7	CaSNP3240	49.48	-	-
5511	CaLG7	CaSNP2857	49.49	scaffold01361	75510
5512	CaLG7	CaSNP3102	49.49	scaffold01971	58571
5513	CaLG7	CaSNP4374	49.49	scaffold01435	71227
5514	CaLG7	CaSNP3865	49.51	scaffold02453	37641
5515	CaLG7	CaSNP3667	49.51	scaffold00586	169258
5516	CaLG7	CaSNP2720	49.51	scaffold00812	122160
5517	CaLG7	CaSNP3377	49.51	scaffold02134	56446
5518	CaLG7	CaSNP2838	49.52	scaffold01228	88020
5519	CaLG7	CaSNP4211	49.52	-	-
5520	CaLG7	CaSNP3788	49.52	scaffold00130	347313
5521	CaLG7	CaSNP4032	49.52	scaffold00116	355765
5522	CaLG7	CaSNP3483	49.52	scaffold02011	57453
5523	CaLG7	CaSNP2867	49.53	-	-
5524	CaLG7	CaSNP2592	49.54	scaffold01339	75344
5525	CaLG7	CaSNP4288	49.54	scaffold01592	82340
5526	CaLG7	CaSNP3195	49.54	9202427	41030
5527	CaLG7	CaSNP2691	49.54	scaffold01176	111793
5528	CaLG7	CaSNP3487	49.54	scaffold02357	39357
5529	CaLG7	CaSNP4548	49.54	scaffold00599	181259
5530	CaLG7	CaSNP2299	49.55	scaffold00898	132042
5531	CaLG7	CaSNP3638	49.55	scaffold05876	10815
5532	CaLG7	CaSNP3645	49.55	scaffold02240	47185
5533	CaLG7	CaSNP4814	49.55	scaffold01164	86163
5534	CaLG7	CaSNP4252	49.56	-	-

5535	CaLG7	CaSNP2396	49.56	9204473	12123
5536	CaLG7	CaSNP4532	49.56	9204723	28815
5537	CaLG7	CaSNP2263	49.57	scaffold00791	106169
5538	CaLG7	CaSNP2671	49.57	-	-
5539	CaLG7	CaSNP3960	49.57	9193511	14118
5540	CaLG7	CaSNP4960	49.57	scaffold01732	68603
5541	CaLG7	CaSNP2700	49.58	scaffold01173	78071
5542	CaLG7	CaSNP4562	49.58	scaffold01833	60987
5543	CaLG7	CaSNP2775	49.58	scaffold01507	76133
5544	CaLG7	CaSNP3176	49.58	scaffold01237	86969
5545	CaLG7	CaSNP3971	49.58	-	-
5546	CaLG7	CaSNP4003	49.59	scaffold00878	122636
5547	CaLG7	CaSNP3409	49.59	-	-
5548	CaLG7	CaSNP3376	49.59	9208817	29549
5549	CaLG7	CaSNP3938	49.59	9211189	17795
5550	CaLG7	CaSNP4034	49.59	scaffold02022	46407
5551	CaLG7	CaSNP4286	49.59	scaffold02821	31714
5552	CaLG7	CaSNP4423	49.59	scaffold03000	29327
5553	CaLG7	CaSNP3720	49.6	scaffold01242	97354
5554	CaLG7	CaSNP4255	49.6	-	-
5555	CaLG7	CaSNP2361	49.6	scaffold01019	110681
5556	CaLG7	CaSNP3467	49.6	scaffold06712	7922
5557	CaLG7	CaSNP4110	49.6	-	-
5558	CaLG7	CaSNP2853	49.61	scaffold00755	118361
5559	CaLG7	CaSNP3702	49.61	scaffold03290	44486
5560	CaLG7	CaSNP3206	49.61	scaffold01199	106715
5561	CaLG7	CaSNP4734	49.61	scaffold01910	65084
5562	CaLG7	CaSNP4445	49.61	-	-
5563	CaLG7	CaSNP3379	49.62	scaffold02267	61722

5564	CaLG7	CaSNP2950	49.62	scaffold05481	10960
5565	CaLG7	CaSNP3426	49.62	-	-
5566	CaLG7	CaSNP4977	49.62	scaffold02372	52125
5567	CaLG7	CaSNP3695	49.62	scaffold02723	33163
5568	CaLG7	CaSNP1967	49.63	-	-
5569	CaLG7	CaSNP3192	49.63	scaffold01100	89713
5570	CaLG7	CaSNP4421	49.63	scaffold02798	33292
5571	CaLG7	CaSNP2530	49.63	scaffold00966	104647
5572	CaLG7	CaSNP2677	49.63	9200986	16875
5573	CaLG7	CaSNP4450	49.63	-	-
5574	CaLG7	CaSNP2324	49.63	scaffold00630	143859
5575	CaLG7	CaSNP3629	49.64	scaffold01801	57182
5576	CaLG7	CaSNP2611	49.64	scaffold01074	88168
5577	CaLG7	CaSNP2740	49.64	scaffold01425	69755
5578	CaLG7	CaSNP4432	49.65	scaffold04229	22733
5579	CaLG7	CaSNP3036	49.65	scaffold00961	110186
5580	CaLG7	CaSNP3943	49.65	9202616	7302
5581	CaLG7	CaSNP5137	49.66	scaffold02195	52025
5582	CaLG7	CaSNP3121	49.67	scaffold00655	132957
5583	CaLG7	CaSNP3188	49.67	scaffold01079	93532
5584	CaLG7	CaSNP4754	49.68	scaffold02972	29485
5585	CaLG7	CaSNP4724	49.68	9199771	17375
5586	CaLG7	CaSNP4873	49.68	9204526	28081
5587	CaLG7	CaSNP4667	49.71	scaffold01428	98732
5588	CaLG7	CaSNP2266	49.72	scaffold00218	253361
5589	CaLG7	CaSNP4981	49.73	scaffold01868	51198
5590	CaLG7	CaSNP4234	49.74	scaffold01051	125306
5591	CaLG7	CaSNP3448	49.77	scaffold01831	59661
5592	CaLG7	CaSNP4076	49.77	-	-

5593	CaLG7	CaSNP3387	49.78	scaffold00688	135637
5594	CaLG7	CaSNP3743	49.79	scaffold01750	54555
5595	CaLG7	CaSNP2081	49.79	scaffold00451	166254
5596	CaLG7	CaSNP2645	49.79	scaffold00360	211070
5597	CaLG7	CaSNP3344	49.79	scaffold02066	48021
5598	CaLG7	CaSNP4388	49.79	scaffold01392	86459
5599	CaLG7	CaSNP4638	49.79	-	-
5600	CaLG7	CaSNP5147	49.8	scaffold04401	16460
5601	CaLG7	CaSNP4454	49.81	-	-
5602	CaLG7	CaSNP2937	49.83	scaffold01796	66829
5603	CaLG7	CaSNP4613	49.87	9203472	21614
5604	CaLG7	CaSNP3384	49.88	-	-
5605	CaLG7	CaSNP2656	49.9	scaffold00874	100579
5606	CaLG7	CaSNP3643	49.91	-	-
5607	CaLG7	CaSNP2072	49.91	scaffold00570	143833
5608	CaLG7	CaSNP5060	49.91	9170741	8631
5609	CaLG7	CaSNP3277	49.91	scaffold00875	108045
5610	CaLG7	CaSNP2442	49.91	scaffold01122	82743
5611	CaLG7	CaSNP3558	49.91	-	-
5612	CaLG7	CaSNP3955	49.91	scaffold02960	42982
5613	CaLG7	CaSNP4174	49.91	-	-
5614	CaLG7	CaSNP4346	49.91	scaffold01018	102650
5615	CaLG7	CaSNP2607	49.92	scaffold01680	71303
5616	CaLG7	CaSNP3287	49.92	scaffold01323	94536
5617	CaLG7	CaSNP3008	49.94	scaffold01106	100260
5618	CaLG7	CaSNP2758	49.95	9208009	16752
5619	CaLG7	CaSNP3813	49.95	scaffold03600	28483
5620	CaLG7	CaSNP3058	49.96	scaffold02519	36539
5621	CaLG7	CaSNP3585	49.96	scaffold01586	75947

5622	CaLG7	CaSNP3586	49.96	scaffold01745	54696
5623	CaLG7	CaSNP3789	49.96	scaffold00550	160950
5624	CaLG7	CaSNP4683	49.96	scaffold00481	169945
5625	CaLG7	CaSNP4921	49.96	-	-
5626	CaLG7	CaSNP5075	49.96	scaffold01155	87990
5627	CaLG7	CaSNP3497	49.96	scaffold02251	49514
5628	CaLG7	CaSNP3634	49.97	scaffold01464	74007
5629	CaLG7	CaSNP2301	49.97	scaffold00267	214561
5630	CaLG7	CaSNP4607	49.99	scaffold00362	183165
5631	CaLG7	CaSNP5146	50.01	scaffold04210	17192
5632	CaLG7	CaSNP4893	50.01	9212802	23319
5633	CaLG7	CaSNP2892	50.03	scaffold01147	96963
5634	CaLG7	CaSNP3003	50.08	scaffold00991	93352
5635	CaLG7	CaSNP4486	50.24	scaffold01835	86945
5636	CaLG7	CaSNP2465	50.34	-	-
5637	CaLG7	CaSNP1949	50.5	scaffold00314	192029
5638	CaLG7	CaSNP3533	50.55	scaffold02782	63340
5639	CaLG7	CaSNP3016	50.58	contig60071	1231
5640	CaLG7	CaSNP4244	50.62	scaffold01291	77132
5641	CaLG7	CaSNP2368	50.96	scaffold03365	24706
5642	CaLG7	CaTSNP6938	51.02	-	-
5643	CaLG7	CaSNP4870	51.02	scaffold00930	93722
5644	CaLG7	CaTMS816	51.48	-	-
5645	CaLG7	CaSNP4135	51.48	scaffold00572	144676
5646	CaLG7	CaSNP3263	51.53	9166962	27561
5647	CaLG7	CaSNP2665	51.83	-	-
5648	CaLG7	CaGMS8	51.85	-	-
5649	CaLG7	CaSNP2238	51.89	-	-
5650	CaLG7	CaSNP4305	51.91	scaffold01590	93355

5651	CaLG7	CaSNP5070	51.92	scaffold00633	125093
5652	CaLG7	CaSNP5153	51.92	-	-
5653	CaLG7	CaSNP2089	51.96	scaffold00192	252495
5654	CaLG7	CaTSNP8582	52.06	-	-
5655	CaLG7	CaTSNP7598	52.13	scaffold06492	8371
5656	CaLG7	CaTSNP8459	52.15	-	-
5657	CaLG7	CaTSNP6896	52.16	scaffold02900	33018
5658	CaLG7	CaSNP5095	52.21	scaffold00913	116874
5659	CaLG7	CaTSNP7802	52.26	-	-
5660	CaLG7	CaTSNP6373	52.26	scaffold03982	19731
5661	CaLG7	CaTSNP6552	52.26	-	-
5662	CaLG7	CaTSNP7258	52.26	-	-
5663	CaLG7	CaTSNP7417	52.28	-	-
5664	CaLG7	CaTSNP7724	52.3	9204662	18975
5665	CaLG7	CaTSNP8158	52.3	-	-
5666	CaLG7	CaTSNP7102	52.31	-	-
5667	CaLG7	CaTSNP7395	52.31	scaffold01606	70059
5668	CaLG7	CaSNP4153	52.34	-	-
5669	CaLG7	CaTSNP6813	52.35	-	-
5670	CaLG7	CaTSNP7136	52.35	-	-
5671	CaLG7	CaSNP4849	52.43	scaffold01513	69771
5672	CaLG7	CaTSNP6282	52.44	-	-
5673	CaLG7	CaTSNP8896	52.44	-	-
5674	CaLG7	CaTSNP6251	52.46	scaffold00398	169372
5675	CaLG7	CaTSNP8079	52.5	-	-
5676	CaLG7	CaTSNP8599	52.5	-	-
5677	CaLG7	CaTSNP8190	52.51	scaffold01285	87310
5678	CaLG7	CaTSNP8060	52.54	-	-
5679	CaLG7	CaTSNP8500	52.6	scaffold01400	83069

5680	CaLG7	CaTSNP7297	52.6	-	-
5681	CaLG7	CaTSNP7531	52.6	-	-
5682	CaLG7	CaTSNP8607	52.66	-	-
5683	CaLG7	CaTSNP6390	52.66	-	-
5684	CaLG7	CaTSNP6252	52.67	scaffold01692	67577
5685	CaLG7	CaTSNP6882	52.67	-	-
5686	CaLG7	CaTSNP9149	52.69	-	-
5687	CaLG7	CaGMS44	52.69	-	-
5688	CaLG7	CaTSNP6083	52.73	scaffold02062	62051
5689	CaLG7	CaSNP2253	52.75	scaffold00128	321318
5690	CaLG7	CaTSNP7129	52.8	-	-
5691	CaLG7	CaTSNP7753	52.83	-	-
5692	CaLG7	CaTSNP6321	52.98	-	-
5693	CaLG7	CaTSNP7918	52.98	-	-
5694	CaLG7	CaTSNP6260	52.99	-	-
5695	CaLG7	CaSNP4090	53.08	-	-
5696	CaLG7	CaTSNP7863	53.14	scaffold01028	116582
5697	CaLG7	CaTSNP8137	53.14	-	-
5698	CaLG7	CaTSNP7486	53.15	-	-
5699	CaLG7	CaTSNP8790	53.15	-	-
5700	CaLG7	CaTSNP7694	54.19	scaffold01016	88418
5701	CaLG7	CaSNP4028	54.66	scaffold01929	51258
5702	CaLG7	CaTSNP8428	55.01	9208937	15146
5703	CaLG7	CaTSNP6098	55.08	-	-
5704	CaLG7	CaTSNP6532	55.08	-	-
5705	CaLG7	CaTSNP9009	55.2	-	-
5706	CaLG7	CaTSNP6266	55.35	scaffold02449	38496
5707	CaLG7	CaTSNP8244	55.38	-	-
5708	CaLG7	CaTSNP6856	55.38	-	-

5709	CaLG7	CaSNP4796	55.68	scaffold01896	55098
5710	CaLG7	CaSNP3435	55.71	9204576	28633
5711	CaLG7	CaSNP2562	55.72	scaffold01653	73811
5712	CaLG7	CaTSNP7344	55.77	-	-
5713	CaLG7	CaSNP2365	55.86	scaffold00466	164081
5714	CaLG7	CaSNP2818	56.33	-	-
5715	CaLG7	CaSNP2179	56.54	9158798	88516
5716	CaLG7	CaTSNP7479	56.54	-	-
5717	CaLG7	CaTSNP7872	56.56	-	-
5718	CaLG7	CaSNP3593	56.57	-	-
5719	CaLG7	CaSNP3349	56.62	scaffold01804	71070
5720	CaLG7	CaTSNP8745	56.64	-	-
5721	CaLG7	CaTSNP8051	56.66	scaffold02783	32407
5722	CaLG7	CaTSNP6981	56.66	-	-
5723	CaLG7	PIP39	56.68	9205487	58823
5724	CaLG7	CaTSNP7068	56.73	-	-
5725	CaLG7	CaSNP2367	56.74	scaffold00475	149272
5726	CaLG7	CaSNP3664	56.82	-	-
5727	CaLG7	CaTSNP6757	56.83	scaffold01758	65513
5728	CaLG7	CaTSNP7016	56.84	-	-
5729	CaLG7	CaTSNP6286	56.85	-	-
5730	CaLG7	CaTSNP6444	56.87	-	-
5731	CaLG7	CaTSNP7788	56.87	-	-
5732	CaLG7	CaTSNP6076	56.89	-	-
5733	CaLG7	CaSNP4820	56.95	-	-
5734	CaLG7	NCPGR14	57.02	-	-
5735	CaLG7	CaSNP2265	57.02	scaffold00401	198681
5736	CaLG7	CaSNP3046	57.04	scaffold00050	477147
5737	CaLG7	CaTSNP6955	57.05	-	-

5738	CaLG7	CaTSNP7046	57.07	scaffold05218	11932
5739	CaLG7	CaTSNP8992	57.1	-	-
5740	CaLG7	CaSNP4070	57.26	-	-
5741	CaLG7	CaSNP2335	57.46	-	-
5742	CaLG7	CaTSNP8914	57.5	-	-
5743	CaLG7	CaSNP1925	57.56	-	-
5744	CaLG7	CaSNP3814	57.58	-	-
5745	CaLG7	CaSNP3260	57.63	-	-
5746	CaLG7	CaTMS888	58.26	-	-
5747	CaLG7	CaTSNP6587	58.45	-	-
5748	CaLG7	CaTSNP8216	59.02	9187252	71777
5749	CaLG7	CaSNP3247	59.18	-	-
5750	CaLG7	CaSNP2477	59.84	-	-
5751	CaLG7	CaSNP3010	60.33	-	-
5752	CaLG7	CaTSNP6200	60.48	-	-
5753	CaLG7	CaTSNP8780	60.5	-	-
5754	CaLG7	CaTSNP8174	60.7	-	-
5755	CaLG7	STMS6	61.54	-	-
5756	CaLG7	CaSNP4360	61.9	scaffold00632	183743
5757	CaLG7	H2B19	62.02	-	-
5758	CaLG7	CaSNP2216	62.71	-	-
5759	CaLG7	CaSNP278	63.05	scaffold00245	223106
5760	CaLG7	CaSNP48	63.16	-	-
5761	CaLG7	CaSNP169	63.26	scaffold01076	84470
5762	CaLG7	CaSNP4151	63.43	1267127	16270
5763	CaLG7	CaSNP508	63.45	-	-
5764	CaLG7	CaSNP2183	63.45	scaffold00300	203502
5765	CaLG7	CaSNP438	63.48	-	-
5766	CaLG7	CaSNP432	63.64	scaffold00222	232171

5767	CaLG7	PIP86	63.85	-	-
5768	CaLG7	CaSNP2054	63.86	9139832	47342
5769	CaLG7	NCPGR268	64.34	-	-
5770	CaLG7	CaTSNP7681	64.38	scaffold01628	60777
5771	CaLG7	CaSNP114	64.48	scaffold01352	71946
5772	CaLG7	CaSNP49	64.53	scaffold00056	447099
5773	CaLG7	CaSNP2208	64.62	scaffold03077	34070
5774	CaLG7	CaSNP1979	64.93	scaffold00556	138718
5775	CaLG7	CaSNP2474	64.93	-	-
5776	CaLG7	CaTSNP8536	65.15	scaffold05181	12092
5777	CaLG7	CaTSNP7044	65.26	-	-
5778	CaLG7	NCPGR41	65.37	scaffold00001	1562097
5779	CaLG7	CaGMS1145	65.38	-	-
5780	CaLG7	CaTSNP6400	65.39	-	-
5781	CaLG7	CaTSNP9135	65.39	-	-
5782	CaLG7	CaTSNP9018	65.47	scaffold00806	116696
5783	CaLG7	CaTSNP7738	65.56	-	-
5784	CaLG7	CaTSNP8984	65.62	-	-
5785	CaLG7	CaSNP5056	65.63	-	-
5786	CaLG7	CaTSNP7447	65.64	-	-
5787	CaLG7	CaSNP552	65.7	scaffold00434	165158
5788	CaLG7	CaTSNP6379	65.72	-	-
5789	CaLG7	CaSNP2801	65.73	-	-
5790	CaLG7	CaTSNP7032	65.81	-	-
5791	CaLG7	CaSNP559	65.93	-	-
5792	CaLG7	CaSNP724	65.97	scaffold00819	119382
5793	CaLG7	CaTSNP8072	65.99	-	-
5794	CaLG7	CaSNP322	66.04	-	-
5795	CaLG7	CaTSNP6488	66.05	-	-

5796	CaLG7	CaTSNP8576	66.06	scaffold13198	2885
5797	CaLG7	CaTSNP8682	66.06	-	-
5798	CaLG7	ESNP38	66.12	-	-
5799	CaLG7	CaTSNP6550	66.13	scaffold03293	25591
5800	CaLG7	CaTSNP9060	66.13	-	-
5801	CaLG7	CaTSNP7562	66.18	-	-
5802	CaLG7	ESNP54	66.21	-	-
5803	CaLG7	ESNP29	66.27	scaffold01480	71820
5804	CaLG7	CaSNP682	66.29	scaffold00015	687920
5805	CaLG7	MtEST235	66.3	-	-
5806	CaLG7	CaSNP907	66.38	-	-
5807	CaLG7	CaSNP627	66.42	-	-
5808	CaLG7	CaSNP109	66.48	-	-
5809	CaLG7	CaSNP600	66.52	-	-
5810	CaLG7	PIP23	66.54	-	-
5811	CaLG7	CaTSNP6366	66.55	-	-
5812	CaLG7	CaTSNP7369	66.55	-	-
5813	CaLG7	CaTSNP8615	66.6	9159958	9557
5814	CaLG7	CaSNP345	66.61	scaffold00673	121786
5815	CaLG7	CaSNP620	66.63	scaffold00260	207131
5816	CaLG7	CaSNP307	66.63	scaffold00491	148219
5817	CaLG7	CaSNP863	66.63	-	-
5818	CaLG7	CaSNP711	66.65	-	-
5819	CaLG7	CaSNP96	66.65	-	-
5820	CaLG7	ESNP51	66.65	scaffold01472	64514
5821	CaLG7	CaSNP862	66.69	-	-
5822	CaLG7	CaSNP257	66.73	-	-
5823	CaLG7	NCPGR98	66.78	-	-
5824	CaLG7	CaSNP221	66.79	-	-

5825	CaLG7	CaSNP2036	67.01	scaffold00172	272460
5826	CaLG7	CaTSNP6701	67.06	-	-
5827	CaLG7	ESNP43	67.54	scaffold00177	248231
5828	CaLG7	CaSNP181	67.73	-	-
5829	CaLG7	CaSNP3350	67.78	-	-
5830	CaLG7	CaSNP4460	67.94	scaffold00861	99747
5831	CaLG7	CaSNP141	67.97	-	-
5832	CaLG7	CaTSNP7839	67.97	-	-
5833	CaLG7	CaSNP219	67.97	9210959	72271
5834	CaLG7	ESNP7	67.97	-	-
5835	CaLG7	ESNP87	67.97	-	-
5836	CaLG7	CaSNP3137	68.05	-	-
5837	CaLG7	CaSNP194	68.07	-	-
5838	CaLG7	CaTMS910	68.1	-	-
5839	CaLG7	CaSNP610	68.1	scaffold00081	389694
5840	CaLG7	CaSNP545	68.14	-	-
5841	CaLG7	CaTSNP6174	68.14	-	-
5842	CaLG7	CaSNP268	68.19	9215096	39195
5843	CaLG7	CaSNP783	68.19	-	-
5844	CaLG7	CaSNP588	68.33	-	-
5845	CaLG7	CaSNP445	68.39	-	-
5846	CaLG7	CaTSNP7134	68.4	-	-
5847	CaLG7	CaSNP220	68.41	-	-
5848	CaLG7	CaSNP27	68.41	-	-
5849	CaLG7	CaSNP2787	68.44	9208103	43349
5850	CaLG7	CaSNP361	68.44	scaffold00835	120797
5851	CaLG7	CaSNP3469	68.48	-	-
5852	CaLG7	CaSNP39	68.53	scaffold00452	159196
5853	CaLG7	ESNP48	68.55	9214662	30022

5854	CaLG7	CaSNP2227	68.59	scaffold00581	129494
5855	CaLG7	CaSNP73	68.62	9207642	107103
5856	CaLG7	CaSNP4060	68.63	scaffold00515	152868
5857	CaLG7	CaSNP4379	68.67	-	-
5858	CaLG7	CaSNP662	68.69	-	-
5859	CaLG7	CaTSNP6935	68.9	-	-
5860	CaLG7	CaTSNP7326	68.93	-	-
5861	CaLG7	CaTSNP8997	68.96	-	-
5862	CaLG7	CaSNP2969	69.29	-	-
5863	CaLG7	CaSNP3165	69.35	-	-
5864	CaLG7	CaTSNP6976	69.38	-	-
5865	CaLG7	CaTSNP8348	69.47	9168355	42610
5866	CaLG7	CaSNP2114	69.64	-	-
5867	CaLG7	CaSNP401	69.68	9202509	42215
5868	CaLG7	CaTSNP9121	69.72	-	-
5869	CaLG7	CaSNP1939	69.74	-	-
5870	CaLG7	CaTSNP8036	69.79	-	-
5871	CaLG7	CaSNP556	69.82	scaffold00027	568460
5872	CaLG7	CaTSNP8942	69.89	-	-
5873	CaLG7	CaTSNP6151	70.21	scaffold04593	17004
5874	CaLG7	CaTSNP7615	70.23	9211145	27939
5875	CaLG7	TA180	70.23	-	-
5876	CaLG7	CaTSNP7123	70.25	scaffold06962	8954
5877	CaLG7	CaTSNP7861	70.27	-	-
5878	CaLG7	CaTSNP9081	70.29	-	-
5879	CaLG7	CaTSNP7343	70.3	-	-
5880	CaLG7	CaTSNP6029	70.3	-	-
5881	CaLG7	CaTSNP8132	70.3	-	-
5882	CaLG7	CaTSNP6411	70.32	9203274	89925

5883	CaLG7	CaTSNP8854	70.32	-	-
5884	CaLG7	CaTSNP8713	70.33	-	-
5885	CaLG7	CaTSNP8881	70.33	9152843	29549
5886	CaLG7	CaTSNP8912	70.33	-	-
5887	CaLG7	CaTSNP7253	70.33	-	-
5888	CaLG7	CaTSNP6592	70.34	-	-
5889	CaLG7	CaTSNP6681	70.34	-	-
5890	CaLG7	CaTSNP6149	70.35	-	-
5891	CaLG7	CaTSNP7714	70.35	-	-
5892	CaLG7	CaTSNP8166	70.36	-	-
5893	CaLG7	CaTSNP8962	70.39	-	-
5894	CaLG7	CaTSNP8899	70.42	-	-
5895	CaLG7	CaTSNP8343	70.44	-	-
5896	CaLG7	CaSNP420	70.47	-	-
5897	CaLG7	CaSNP159	70.52	-	-
5898	CaLG7	CaSNP505	70.7	scaffold00143	284742
5899	CaLG7	CaTSNP7288	70.75	-	-
5900	CaLG7	CaTSNP7621	70.75	-	-
5901	CaLG7	CaTSNP8670	70.75	-	-
5902	CaLG7	CaSNP661	70.76	-	-
5903	CaLG7	CaTSNP8695	70.76	-	-
5904	CaLG7	CaTMS880	70.96	-	-
5905	CaLG7	CaSNP717	71.03	-	-
5906	CaLG7	CaSNP117	71.03	-	-
5907	CaLG7	ESNP69	71.12	scaffold12182	3244
5908	CaLG7	CaTSNP6495	71.2	-	-
5909	CaLG7	CaTSNP7643	71.22	-	-
5910	CaLG7	CaTSNP8242	71.22	-	-
5911	CaLG7	CaSNP778	71.35	-	-

5912	CaLG7	CaSNP837	71.4	scaffold00282	214240
5913	CaLG7	CaSNP462	71.54	scaffold00767	110411
5914	CaLG7	CaTSNP7185	71.62	-	-
5915	CaLG7	CaSNP452	71.66	scaffold02706	33853
5916	CaLG7	CaSNP276	71.71	scaffold00226	222040
5917	CaLG7	CaSNP2449	71.71	scaffold01203	76323
5918	CaLG7	CaSNP208	71.79	scaffold00501	144749
5919	CaLG7	CaSNP2434	71.79	9207619	6957
5920	CaLG7	CaSNP224	71.88	scaffold00649	120090
5921	CaLG7	CaSNP2061	71.94	-	-
5922	CaLG7	ESNP21	71.94	scaffold00463	185295
5923	CaLG7	CaSNP167	71.96	scaffold02055	52501
5924	CaLG7	CaSNP2851	71.99	-	-
5925	CaLG7	CaSNP660	72.06	scaffold01275	73009
5926	CaLG7	CaSNP641	72.07	9109349	23512
5927	CaLG7	CaTSNP6649	72.13	-	-
5928	CaLG7	CaTSNP6123	72.15	-	-
5929	CaLG7	CaTSNP6339	72.17	-	-
5930	CaLG7	CaSNP288	72.19	-	-
5931	CaLG7	CaTSNP6001	72.25	-	-
5932	CaLG7	CaTSNP6418	72.25	-	-
5933	CaLG7	CaTSNP6829	72.25	-	-
5934	CaLG7	CaGMS1239	72.25	-	-
5935	CaLG7	CaTSNP7219	72.31	-	-
5936	CaLG7	CaTSNP7511	72.31	-	-
5937	CaLG7	CaTSNP6475	72.33	-	-
5938	CaLG7	CaTSNP8027	72.33	-	-
5939	CaLG7	CaTSNP8493	72.33	-	-
5940	CaLG7	CaTSNP9084	72.33	-	-

5941	CaLG7	CaTSNP7589	72.49	-	-
5942	CaLG7	CaSNP5121	72.64	-	-
5943	CaLG7	CaSNP2408	72.65	-	-
5944	CaLG7	CaTSNP8418	72.73	-	-
5945	CaLG7	CaTSNP7760	72.8	-	-
5946	CaLG7	CaTSNP8375	72.82	scaffold00661	120151
5947	CaLG7	CaTMS598	72.89	-	-
5948	CaLG7	CaSNP5051	72.98	-	-
5949	CaLG7	CaTMS705	73.03	-	-
5950	CaLG7	CaTSNP8314	73.29	-	-
5951	CaLG7	CaTSNP8593	73.42	-	-
5952	CaLG7	CaTSNP9044	73.42	-	-
5953	CaLG7	CaSNP1966	73.77	-	-
5954	CaLG7	CaTSNP8562	73.78	9215831	98329
5955	CaLG7	CaTSNP8138	73.98	-	-
5956	CaLG7	CaTSNP6360	74.06	-	-
5957	CaLG7	CaTSNP9151	74.31	-	-
5958	CaLG7	CaTSNP7049	74.91	-	-
5959	CaLG7	CaTSNP7373	74.94	-	-
5960	CaLG7	CaTSNP8734	74.96	-	-
5961	CaLG7	CaTSNP8764	74.97	-	-
5962	CaLG7	CaTSNP8640	75.01	-	-
5963	CaLG7	CaTSNP8975	75.02	-	-
5964	CaLG7	CaTSNP6603	75.07	9202491	26534
5965	CaLG7	CaTSNP6397	75.75	-	-
5966	CaLG7	CaTSNP6455	75.79	-	-
5967	CaLG7	CaTSNP8313	75.79	-	-
5968	CaLG7	CaTSNP9047	76.11	-	-
5969	CaLG7	CaTSNP6851	76.15	-	-

5970	CaLG7	CaSNP2701	76.16	-	-
5971	CaLG7	CaTSNP8486	76.16	-	-
5972	CaLG7	CaTSNP8393	76.18	-	-
5973	CaLG7	CaTSNP6374	76.19	-	-
5974	CaLG7	CaTSNP6963	76.2	-	-
5975	CaLG7	CaTSNP7432	76.2	-	-
5976	CaLG7	CaTSNP7438	76.2	-	-
5977	CaLG7	CaTSNP7930	76.2	-	-
5978	CaLG7	CaTSNP8254	76.2	-	-
5979	CaLG7	CaTSNP8273	76.2	-	-
5980	CaLG7	CaTSNP8757	76.22	-	-
5981	CaLG7	CaTSNP6821	76.27	-	-
5982	CaLG7	CaTSNP8124	76.29	-	-
5983	CaLG7	CaSNP2457	76.5	-	-
5984	CaLG7	CaSNP2561	76.55	scaffold01904	57098
5985	CaLG7	CaSNP3330	76.55	-	-
5986	CaLG7	CaTSNP7931	76.56	-	-
5987	CaLG7	CaTSNP6625	76.56	-	-
5988	CaLG7	CaSNP1944	76.58	-	-
5989	CaLG7	CaTSNP6121	76.59	-	-
5990	CaLG7	CaTSNP6276	76.59	-	-
5991	CaLG7	CaTSNP7452	76.59	-	-
5992	CaLG7	CaTSNP6419	76.62	scaffold01082	94638
5993	CaLG7	CaTSNP6886	76.62	scaffold06408	9463
5994	CaLG7	CaTSNP6782	76.64	-	-
5995	CaLG7	CaGMS1132	76.65	-	-
5996	CaLG7	CaTMS1043	76.65	-	-
5997	CaLG7	CaTMS713	76.65	-	-
5998	CaLG7	CaTMS922	76.65	-	-

5999	CaLG7	CaTSNP6945	76.72	-	-
6000	CaLG7	CaTSNP6338	76.74	-	-
6001	CaLG7	CaSNP2188	76.81	-	-
6002	CaLG7	CaTSNP7819	76.82	-	-
6003	CaLG7	CaTSNP6176	76.82	-	-
6004	CaLG7	CaTSNP6763	76.96	-	-
6005	CaLG7	CaTSNP8385	76.96	-	-
6006	CaLG7	CaTSNP8954	76.96	-	-
6007	CaLG7	CaTSNP8161	76.99	9209771	7523
6008	CaLG7	CaTSNP8771	76.99	-	-
6009	CaLG7	CaTSNP7829	77.01	9211313	7178
6010	CaLG7	CaTMS953	77.18	-	-
6011	CaLG7	NCPGR278	77.51	-	-
6012	CaLG7	CaTMS812	78.61	-	-
6013	CaLG7	CaTSNP6511	78.67	-	-
6014	CaLG7	CaTSNP6330	78.69	-	-
6015	CaLG7	CaTSNP7952	78.7	-	-
6016	CaLG7	CaTSNP6563	78.71	-	-
6017	CaLG7	CaTSNP7995	79.13	-	-
6018	CaLG7	CaSNP2010	79.24	-	-
6019	CaLG7	CaTSNP8368	79.39	scaffold00091	339011
6020	CaLG7	CaTMS1031	79.39	-	-
6021	CaLG7	CaTSNP6386	79.41	-	-
6022	CaLG7	CaTSNP8568	79.44	-	-
6023	CaLG7	CaTSNP9109	79.46	-	-
6024	CaLG7	CaSNP3676	80.33	-	-
6025	CaLG7	CaTSNP6478	80.39	-	-
6026	CaLG7	CaTSNP7530	80.41	-	-
6027	CaLG7	CaTSNP8890	80.44	-	-

6028	CaLG7	CaTSNP7272	80.48	-	-
6029	CaLG7	CaTSNP8744	80.54	-	-
6030	CaLG7	CaTSNP6102	80.65	-	-
6031	CaLG7	CaTSNP8151	80.67	-	-
6032	CaLG7	CaTSNP6754	80.77	-	-
6033	CaLG7	CaTSNP8294	80.78	-	-
6034	CaLG7	CaTSNP7547	80.87	-	-
6035	CaLG7	CaTSNP8471	80.87	-	-
6036	CaLG7	CaTSNP8542	80.87	-	-
6037	CaLG7	CaSNP5155	80.88	scaffold00166	250208
6038	CaLG7	CaTSNP7124	80.9	-	-
6039	CaLG7	CaTSNP7178	80.93	scaffold00946	92071
6040	CaLG7	CaTSNP8070	80.95	scaffold00329	192466
6041	CaLG7	CaTSNP6939	80.96	-	-
6042	CaLG7	CaSNP2568	80.96	-	-
6043	CaLG7	CaSNP3674	81.03	scaffold01687	73586
6044	CaLG7	CEST44	81.03	-	-
6045	CaLG7	CaSNP3211	81.19	-	-
6046	CaLG7	CaSNP3519	81.19	-	-
6047	CaLG7	CaTSNP8462	81.46	scaffold00070	409626
6048	CaLG7	CaTSNP8852	81.46	-	-
6049	CaLG7	CaSNP1920	81.54	-	-
6050	CaLG7	CaTSNP6855	81.6	-	-
6051	CaLG7	CaTSNP6753	81.61	-	-
6052	CaLG7	CaTSNP7912	81.66	-	-
6053	CaLG7	CaTSNP6412	81.7	scaffold00023	594304
6054	CaLG7	CaTSNP8402	81.76	-	-
6055	CaLG7	CaTSNP6874	81.87	-	-
6056	CaLG7	CaTSNP8672	81.87	-	-

6057	CaLG7	CaTSNP7950	81.89	-	-
6058	CaLG7	CaTSNP6806	81.89	-	-
6059	CaLG7	CaTSNP9031	81.89	-	-
6060	CaLG7	CaTSNP7152	81.89	-	-
6061	CaLG7	CaTSNP8766	81.94	-	-
6062	CaLG7	CaSNP2008	81.96	scaffold00093	349627
6063	CaLG7	CaTSNP8177	81.98	9153460	48128
6064	CaLG7	CaTSNP6805	82	-	-
6065	CaLG7	CaTSNP7192	82.03	9193975	20324
6066	CaLG7	CaTSNP7408	82.03	scaffold00272	211817
6067	CaLG7	CaTSNP7849	82.03	-	-
6068	CaLG7	CaTSNP8789	82.03	-	-
6069	CaLG7	CaTSNP9096	82.03	-	-
6070	CaLG7	CaTSNP9003	82.03	-	-
6071	CaLG7	CaTMS1076	82.05	-	-
6072	CaLG7	CaTSNP9062	82.06	scaffold01964	59591
6073	CaLG7	CaTSNP6223	82.1	-	-
6074	CaLG7	CaTSNP6257	82.12	-	-
6075	CaLG7	CaTSNP8440	82.12	-	-
6076	CaLG7	CaTSNP7410	82.12	-	-
6077	CaLG7	CaTSNP9001	82.12	-	-
6078	CaLG7	CaTSNP8305	82.14	9211120	50542
6079	CaLG7	CaTSNP9000	82.14	-	-
6080	CaLG7	CaTSNP6779	82.14	-	-
6081	CaLG7	CaTSNP7728	82.14	-	-
6082	CaLG7	CaTSNP7956	82.14	-	-
6083	CaLG7	CaTSNP6310	82.19	-	-
6084	CaLG7	CaTSNP6723	82.21	-	-
6085	CaLG7	CaTSNP7038	82.21	-	-

6086	CaLG7	CaSNP1982	82.51	-	-
6087	CaLG7	CaSNP3132	82.54	scaffold01056	86629
6088	CaLG7	CaGMS1293	82.77	-	-
6089	CaLG7	CaTMS769	82.89	-	-
6090	CaLG7	CaTMS900	82.89	-	-
6091	CaLG7	CaTMS742	82.96	-	-
6092	CaLG7	CaSNP2369	83.04	scaffold00427	169877
6093	CaLG7	CaSNP2413	83.04	-	-
6094	CaLG7	CaSNP1974	83.39	-	-
6095	CaLG7	CaTMS539	88.59	-	-
6096	CaLG7	PIP214	88.78	-	-
6097	CaLG7	CESSR53	90.35	-	-
6098	CaLG7	CaTMS841	90.92	-	-
6099	CaLG7	CaTMS537	93.59	-	-
6100	CaLG7	CaTMS857	96.55	-	-
6101	CaLG7	CaTMS765	98.09	-	-
6102	CaLG7	CaTMS755	98.92	-	-
6103	CaLG7	CaTMS694	98.94	-	-
6104	CaLG7	CaGMS1159	98.94	-	-
6105	CaLG7	CaGMS1262	98.98	-	-
6106	CaLG7	CaGMS1219	98.99	-	-
6107	CaLG7	CaTMS658	98.99	-	-
6108	CaLG7	CaGMS1140	99	-	-
6109	CaLG7	CaGMS1127	99	-	-
6110	CaLG7	CaGMS1141	99	-	-
6111	CaLG7	CaGMS1157	99	-	-
6112	CaLG7	CaGMS1162	99	-	-
6113	CaLG7	CaGMS1177	99	-	-
6114	CaLG7	CaGMS1208	99	-	-

6115	CaLG7	CaTMS1003	99	-	-
6116	CaLG7	CaTMS1026	99	-	-
6117	CaLG7	CaTMS1092	99	-	-
6118	CaLG7	CaTMS1104	99	-	-
6119	CaLG7	CaTMS1109	99	-	-
6120	CaLG7	CaTMS620	99	-	-
6121	CaLG7	CaTMS696	99	-	-
6122	CaLG7	CaTMS718	99	-	-
6123	CaLG7	CaTMS723	99	-	-
6124	CaLG7	CaTMS784	99	-	-
6125	CaLG7	CaTMS789	99	-	-
6126	CaLG7	CaTMS850	99	-	-
6127	CaLG7	CaTMS986	99	-	-
6128	CaLG7	CaTMS994	99	-	-
6129	CaLG7	CaTMS1081	99.01	-	-
6130	CaLG7	CaTMS988	99.01	-	-
6131	CaLG7	CaTMS1099	99.01	-	-
6132	CaLG7	CaTMS1115	99.01	-	-
6133	CaLG7	CaGMS1149	99.01	-	-
6134	CaLG7	CaGMS1152	99.01	-	-
6135	CaLG7	CaGMS1176	99.01	-	-
6136	CaLG7	CaGMS1207	99.01	-	-
6137	CaLG7	CaTMS1002	99.01	-	-
6138	CaLG7	CaTMS1029	99.01	-	-
6139	CaLG7	CaTMS1033	99.01	-	-
6140	CaLG7	CaTMS1096	99.01	-	-
6141	CaLG7	CaTMS1106	99.01	-	-
6142	CaLG7	CaTMS597	99.01	-	-
6143	CaLG7	CaTMS940	99.01	-	-

6144	CaLG7	CaGMS1142	99.01	-	-
6145	CaLG7	CaGMS1155	99.01	-	-
6146	CaLG7	CaTMS1064	99.02	-	-
6147	CaLG7	CaTMS1080	99.02	-	-
6148	CaLG7	CaTMS638	99.02	-	-
6149	CaLG7	CaTMS855	99.02	-	-
6150	CaLG7	CaTMS898	99.02	-	-
6151	CaLG7	CaGMS1138	99.02	-	-
6152	CaLG7	CaGMS1184	99.02	-	-
6153	CaLG7	CaTMS534	99.02	-	-
6154	CaLG7	CaTMS737	99.03	-	-
6155	CaLG7	CaTMS822	99.03	-	-
6156	CaLG7	CaTMS863	99.14	-	-
6157	CaLG7	CaTMS860	99.32	-	-
6158	CaLG7	CaTMS979	99.32	-	-
6159	CaLG7	CaGMS1185	99.33	-	-
6160	CaLG7	CaTMS1102	99.33	-	-
6161	CaLG7	CaTMS833	99.33	-	-
6162	CaLG7	CaTMS1114	99.35	-	-
6163	CaLG7	CaTMS837	99.35	-	-
6164	CaLG7	CaTMS892	99.35	-	-
6165	CaLG7	CaGMS1250	99.36	-	-
6166	CaLG7	CaGMS1200	100.1	-	-
6167	CaLG7	CaGMS1277	100.1	-	-
6168	CaLG7	CaTMS623	100.1	-	-
6169	CaLG7	CaTMS748	100.1	-	-
6170	CaLG7	CaTMS975	100.1	-	-
6171	CaLG7	CaTMS934	100.1	-	-
6172	CaLG7	CaTMS809	100.1	-	-

6173	CaLG7	CaTMS829	100.1	-	-
6174	CaLG7	CaGMS1252	100.1	-	-
6175	CaLG7	CaTMS1023	100.1	-	-
6176	CaLG7	CaTMS1075	100.1	-	-
6177	CaLG7	CaTMS1091	100.1	-	-
6178	CaLG7	CaTMS585	100.1	-	-
6179	CaLG7	CaTMS593	100.1	-	-
6180	CaLG7	CaTMS647	100.1	-	-
6181	CaLG7	CaTMS719	100.1	-	-
6182	CaLG7	CaTMS779	100.1	-	-
6183	CaLG7	CaTMS510	100.1	-	-
6184	CaLG7	CaTMS514	100.1	-	-
6185	CaLG7	CaGMS1167	100.1	-	-
6186	CaLG7	CaTMS1071	100.1	-	-
6187	CaLG7	CaGMS1306	100.1	-	-
6188	CaLG7	CaGMS1309	101.5	-	-
6189	CaLG7	CaTMS882	101.5	-	-
6190	CaLG7	NCPGR34	101.7	scaffold00110	328263
6191	CaLG7	CaGMS35	101.7	-	-
6192	CaLG7	CaTMS1038	101.7	-	-
6193	CaLG7	CaGMS1266	102.1	-	-
6194	CaLG7	CaTMS630	102.2	-	-
6195	CaLG7	CaTMS1018	102.2	-	-
6196	CaLG7	CaTMS564	102.2	-	-
6197	CaLG7	CaTMS799	102.4	-	-
6198	CaLG7	CaTMS546	102.7	-	-
6199	CaLG7	CaTMS552	102.9	-	-
6200	CaLG7	CaTMS950	103.5	-	-
6201	CaLG7	CaTMS736	103.9	-	-

6202	CaLG7	CaTMS906	103.9	-	-
6203	CaLG7	CaTMS1105	103.9	-	-
6204	CaLG7	CaTMS740	103.9	-	-
6205	CaLG7	CaTMS641	104	-	-
6206	CaLG7	CaTMS1010	104.4	-	-
6207	CaLG7	CaGMS40	104.9	-	-
6208	CaLG7	CaTMS677	105.4	-	-
6209	CaLG7	CaTMS678	105.5	-	-
6210	CaLG7	CaTMS689	105.8	-	-
6211	CaLG7	CASTMS9	105.9	-	-
6212	CaLG7	CaTMS670	106.9	-	-
6213	CaLG7	CESSR50	109.8	scaffold00444	157686
6214	CaLG7	CaTMS700	110.8	-	-
6215	CaLG7	CaTMS697	120.5	-	-
6216	CaLG7	CaTMS1025	120.9	-	-
6217	CaLG7	CaTMS876	123.1	-	-
6218	CaLG7	CaTMS825	124	-	-
6219	CaLG8	H3G031	0	-	-
6220	CaLG8	CaTSNP7292	29.67	-	-
6221	CaLG8	GAA46	31.1	-	-
6222	CaLG8	CaGMS1258	32.23	-	-
6223	CaLG8	CaSNP4184	33.18	-	-
6224	CaLG8	CaTSNP7117	33.49	-	-
6225	CaLG8	CaTSNP8619	33.49	-	-
6226	CaLG8	CaSNP2719	33.62	scaffold01158	87335
6227	CaLG8	CaTSNP7240	33.63	-	-
6228	CaLG8	CaTSNP8009	33.64	-	-
6229	CaLG8	CaSNP2904	33.64	scaffold00826	116685
6230	CaLG8	CaTSNP8616	33.74	-	-

6231	CaLG8	CaTSNP8558	33.81	-	-
6232	CaLG8	CaTSNP6928	33.83	-	-
6233	CaLG8	CaTSNP8965	33.87	-	-
6234	CaLG8	CaSNP294	34.15	-	-
6235	CaLG8	CaSNP289	34.22	scaffold00148	270062
6236	CaLG8	CaTSNP8476	34.42	-	-
6237	CaLG8	CaSNP239	34.48	-	-
6238	CaLG8	CaTSNP6277	34.58	-	-
6239	CaLG8	CaTSNP8475	34.58	-	-
6240	CaLG8	CaTSNP7131	34.69	-	-
6241	CaLG8	CaSNP624	35.07	-	-
6242	CaLG8	NCPGR242	35.52	-	-
6243	CaLG8	CaSNP658	36.27	-	-
6244	CaLG8	CaSNP152	36.4	-	-
6245	CaLG8	CaSNP2900	38.13	-	-
6246	CaLG8	CaSNP2621	39.31	-	-
6247	CaLG8	CaSNP838	40.02	scaffold00368	180122
6248	CaLG8	CaTSNP6435	40.28	-	-
6249	CaLG8	CaTSNP6641	40.3	-	-
6250	CaLG8	CaTSNP6198	40.36	-	-
6251	CaLG8	CaTSNP7164	40.42	-	-
6252	CaLG8	CaTSNP7360	40.42	-	-
6253	CaLG8	CaTSNP9063	40.44	-	-
6254	CaLG8	CaTSNP7782	40.5	-	-
6255	CaLG8	CaTSNP9155	40.5	-	-
6256	CaLG8	CaTSNP7406	40.51	-	-
6257	CaLG8	CaTSNP8917	40.52	-	-
6258	CaLG8	CaTSNP7310	40.54	-	-
6259	CaLG8	CaTSNP6545	40.54	-	-

6260	CaLG8	CaTSNP6917	40.54	-	-
6261	CaLG8	CaTSNP7061	40.56	-	-
6262	CaLG8	CaTSNP8626	40.56	9198839	16319
6263	CaLG8	CaTSNP8300	40.57	9207579	60016
6264	CaLG8	CaTSNP6096	40.58	-	-
6265	CaLG8	CaTSNP7535	40.58	-	-
6266	CaLG8	CaTSNP7387	40.59	-	-
6267	CaLG8	CaTSNP8237	40.6	-	-
6268	CaLG8	CaTSNP7377	40.61	-	-
6269	CaLG8	CaTSNP7431	40.63	-	-
6270	CaLG8	CaTSNP8387	40.63	-	-
6271	CaLG8	CaTSNP7256	40.64	-	-
6272	CaLG8	CaTSNP6404	40.66	-	-
6273	CaLG8	CaTSNP7717	40.83	-	-
6274	CaLG8	CaTSNP7468	40.86	-	-
6275	CaLG8	CaTSNP7114	40.87	-	-
6276	CaLG8	CaTSNP6423	41.02	scaffold00567	131522
6277	CaLG8	CaTSNP8239	41.02	-	-
6278	CaLG8	CaTSNP7121	41.25	-	-
6279	CaLG8	CaTSNP7815	41.54	-	-
6280	CaLG8	CaTSNP8706	41.54	-	-
6281	CaLG8	CaTSNP8236	41.55	-	-
6282	CaLG8	CaTSNP6594	41.61	-	-
6283	CaLG8	CaTSNP9005	41.61	-	-
6284	CaLG8	CaTSNP6308	41.76	-	-
6285	CaLG8	CaTSNP6484	42.03	-	-
6286	CaLG8	CaTSNP6072	42.04	-	-
6287	CaLG8	CaTSNP6880	42.04	-	-
6288	CaLG8	CaTSNP7612	42.04	-	-

6289	CaLG8	CaTSNP7901	42.05	-	-
6290	CaLG8	CaTSNP8465	42.05	scaffold06862	7674
6291	CaLG8	CaTSNP9070	42.05	-	-
6292	CaLG8	CaTSNP9104	42.1	-	-
6293	CaLG8	CaTSNP9095	42.22	scaffold04058	18855
6294	CaLG8	CaTSNP6535	42.37	-	-
6295	CaLG8	CaTSNP8056	42.75	-	-
6296	CaLG8	CaTSNP7928	42.79	-	-
6297	CaLG8	CaTSNP9111	42.82	-	-
6298	CaLG8	CaTSNP6539	42.87	-	-
6299	CaLG8	CaTSNP8738	42.91	9184095	17867
6300	CaLG8	CaTSNP7378	42.91	-	-
6301	CaLG8	CaTSNP7677	42.92	-	-
6302	CaLG8	CaTSNP6007	42.94	-	-
6303	CaLG8	CaTSNP7323	42.94	-	-
6304	CaLG8	CaTSNP6988	42.96	9174326	15066
6305	CaLG8	CaTSNP6133	43.04	-	-
6306	CaLG8	CaTSNP7965	43.05	-	-
6307	CaLG8	CaTSNP7919	43.1	scaffold00682	126513
6308	CaLG8	CaTSNP8197	43.11	-	-
6309	CaLG8	CaTSNP7026	43.12	-	-
6310	CaLG8	CaTSNP6912	43.14	-	-
6311	CaLG8	CaSNP2321	43.57	-	-
6312	CaLG8	CaSNP5052	44.4	-	-
6313	CaLG8	CaSNP5062	44.97	-	-
6314	CaLG8	CaSNP3872	45.14	-	-
6315	CaLG8	CaSNP2168	45.21	-	-
6316	CaLG8	CaSNP3584	45.23	-	-
6317	CaLG8	CaSNP2470	45.37	-	-

6318	CaLG8	CaSNP840	45.64	scaffold00249	230849
6319	CaLG8	CaSNP64	46.14	scaffold00041	485748
6320	CaLG8	CaSNP822	46.14	-	-
6321	CaLG8	CaSNP697	46.14	-	-
6322	CaLG8	CaSNP306	46.19	9206806	48282
6323	CaLG8	CaSNP805	46.24	-	-
6324	CaLG8	CaSNP354	46.27	scaffold00670	127104
6325	CaLG8	CaSNP333	46.29	scaffold00263	207362
6326	CaLG8	CaSNP154	46.36	scaffold00004	1136293
6327	CaLG8	CaSNP465	46.38	-	-
6328	CaLG8	CaSNP585	46.38	scaffold00310	186758
6329	CaLG8	CaSNP75	46.49	scaffold00057	432475
6330	CaLG8	CaSNP328	46.59	-	-
6331	CaLG8	CaSNP629	47.02	-	-
6332	CaLG8	TS45	47.49	-	-
6333	CaLG8	CaSNP394	47.63	-	-
6334	CaLG8	CaTSNP7451	47.89	-	-
6335	CaLG8	CaSNP2765	47.9	-	-
6336	CaLG8	CaTSNP7798	48.14	-	-
6337	CaLG8	CaSNP2682	48.15	-	-
6338	CaLG8	CaSNP2390	48.18	-	-
6339	CaLG8	CaSNP2935	48.19	-	-
6340	CaLG8	CaTSNP6207	48.35	-	-
6341	CaLG8	CaTSNP8966	48.35	-	-
6342	CaLG8	CaTSNP6350	48.36	-	-
6343	CaLG8	CaTSNP8484	48.36	-	-
6344	CaLG8	CaTSNP6980	48.4	-	-
6345	CaLG8	CaSNP104	48.52	scaffold00551	141412
6346	CaLG8	CaTSNP8926	48.72	-	-

6347	CaLG8	CaTSNP7341	48.73	-	-
6348	CaLG8	CaSNP2400	49.78	-	-
6349	CaLG8	CaTSNP6060	50.13	-	-
6350	CaLG8	CaTSNP7268	51.1	-	-
6351	CaLG8	CaSNP3068	52.18	-	-
6352	CaLG8	CaTSNP7554	52.93	-	-
6353	CaLG8	CaTSNP7138	52.93	-	-
6354	CaLG8	CESSR18	52.93	scaffold01174	83192
6355	CaLG8	CaTSNP8122	53.03	-	-
6356	CaLG8	CaSNP824	53.27	scaffold00928	102909
6357	CaLG8	CaSNP3104	53.45	scaffold01373	76665
6358	CaLG8	CaTSNP8606	53.5	-	-
6359	CaLG8	CaSNP3186	53.52	scaffold01997	50751
6360	CaLG8	CaTSNP7556	53.55	-	-
6361	CaLG8	CaTSNP8521	53.55	-	-
6362	CaLG8	CaTSNP9100	53.55	-	-
6363	CaLG8	CaTSNP6688	53.59	-	-
6364	CaLG8	CaTSNP6951	53.59	-	-
6365	CaLG8	CaTSNP8082	53.64	-	-
6366	CaLG8	CaSNP478	53.66	scaffold00107	313748
6367	CaLG8	ESNP6	53.66	-	-
6368	CaLG8	CaTMS959	53.96	-	-
6369	CaLG8	CaSNP190	54.13	-	-
6370	CaLG8	CaSNP606	54.19	scaffold00443	171028
6371	CaLG8	CaSNP196	54.64	scaffold00525	140860
6372	CaLG8	NCPGR158	55	-	-
6373	CaLG8	CaGMS1212	55.14	-	-
6374	CaLG8	CaSNP381	55.23	scaffold00764	110283
6375	CaLG8	CaSNP676	55.36	scaffold00290	203324

6376	CaLG8	CaSNP216	55.38	scaffold01353	71046
6377	CaLG8	ESNP62	55.52	scaffold01977	51331
6378	CaLG8	CaSNP443	55.56	-	-
6379	CaLG8	CaTMS805	57.74	-	-
6380	CaLG8	CaTMS672	58.02	-	-
6381	CaLG8	CaTMS978	58.08	-	-
6382	CaLG8	CaTMS1049	58.11	-	-
6383	CaLG8	CaSNP2322	58.16	scaffold00698	141042
6384	CaLG8	CaSNP5151	58.24	-	-
6385	CaLG8	CaSNP2070	58.57	-	-
6386	CaLG8	CaSNP3776	58.74	scaffold02527	37604
6387	CaLG8	CaSNP2073	58.75	-	-
6388	CaLG8	CaTMS524	58.87	-	-
6389	CaLG8	CaSNP2506	58.88	-	-
6390	CaLG8	CaSNP2914	58.88	9154932	22978
6391	CaLG8	CaTSNP8142	59.18	-	-
6392	CaLG8	CaTSNP9055	59.19	scaffold00489	153825
6393	CaLG8	CaTSNP7954	59.2	-	-
6394	CaLG8	CaTSNP7874	59.21	-	-
6395	CaLG8	CaTSNP6216	59.27	-	-
6396	CaLG8	CaTSNP8466	59.27	-	-
6397	CaLG8	CaSNP3711	59.28	scaffold02231	43099
6398	CaLG8	CaTSNP6059	59.29	-	-
6399	CaLG8	CaTSNP8231	59.3	scaffold01694	58140
6400	CaLG8	CaTSNP6533	59.32	-	-
6401	CaLG8	CaTSNP7523	59.32	9206348	21381
6402	CaLG8	CaTSNP8960	59.33	-	-
6403	CaLG8	CaTSNP8062	59.34	-	-
6404	CaLG8	CaTSNP8156	59.34	-	-

6405	CaLG8	CaTSNP7365	59.34	scaffold01778	53144
6406	CaLG8	CaTSNP7631	59.36	scaffold04841	13771
6407	CaLG8	CaTSNP7063	59.38	-	-
6408	CaLG8	TS12	59.46	-	-
6409	CaLG8	CaTSNP7927	59.6	-	-
6410	CaLG8	CaTSNP8307	59.64	scaffold00315	220467
6411	CaLG8	CaTSNP7610	59.65	scaffold03947	18931
6412	CaLG8	CaTSNP7946	59.68	-	-
6413	CaLG8	CaTSNP8883	59.68	-	-
6414	CaLG8	CaTSNP8083	59.69	-	-
6415	CaLG8	CaTSNP8390	59.73	-	-
6416	CaLG8	CaTSNP8915	59.73	-	-
6417	CaLG8	CaTSNP8969	59.73	-	-
6418	CaLG8	CaTSNP6099	59.74	-	-
6419	CaLG8	CaTSNP8726	59.76	-	-
6420	CaLG8	CaTSNP7716	59.77	-	-
6421	CaLG8	CaTSNP6201	59.77	-	-
6422	CaLG8	CaTSNP7730	59.77	-	-
6423	CaLG8	CaTSNP6180	59.84	-	-
6424	CaLG8	CaTSNP9026	59.86	-	-
6425	CaLG8	CaTSNP7150	59.9	-	-
6426	CaLG8	CaTSNP6838	59.94	-	-
6427	CaLG8	CaTSNP8993	59.94	-	-
6428	CaLG8	CaTSNP6353	60.07	-	-
6429	CaLG8	CaTSNP8306	60.08	-	-
6430	CaLG8	CaTSNP8128	60.11	9211715	6758
6431	CaLG8	CaSNP3950	60.16	-	-
6432	CaLG8	CaSNP2084	60.16	scaffold00137	303669
6433	CaLG8	CaSNP5039	60.16	scaffold00375	181242

6434	CaLG8	CaTSNP8553	60.17	-	-
6435	CaLG8	CaSNP3402	60.23	-	-
6436	CaLG8	CaTSNP9002	60.31	-	-
6437	CaLG8	CaSNP2131	60.39	-	-
6438	CaLG8	CaTSNP8919	60.39	-	-
6439	CaLG8	CaSNP3148	60.42	scaffold01280	85055
6440	CaLG8	CaTSNP6899	60.46	-	-
6441	CaLG8	CaSNP2100	60.48	-	-
6442	CaLG8	CaTSNP7739	60.84	-	-
6443	CaLG8	CaTSNP7273	61.41	scaffold00865	99885
6444	CaLG8	NCPGR118	64.43	-	-
6445	CaLG8	NCPGR241	64.99	scaffold00571	164776
6446	CaLG8	CaGMS11	65.42	-	-
6447	CaLG8	CaGMS1269	65.52	-	-
6448	CaLG8	CaTMS1088	65.52	-	-
6449	CaLG8	CaGMS1156	65.82	-	-
6450	CaLG8	CaSNP821	65.88	-	-
6451	CaLG8	CaTMS1052	66	-	-
6452	CaLG8	CaTMS612	66.01	-	-
6453	CaLG8	CaSNP2291	66.11	scaffold01446	74819
6454	CaLG8	NCPGR170	66.14	scaffold00241	219383
6455	CaLG8	CaGMS1144	66.15	-	-
6456	CaLG8	CaTMS604	66.19	-	-
6457	CaLG8	CaTMS1050	66.19	-	-
6458	CaLG8	CaGMS1173	66.21	-	-
6459	CaLG8	CaTMS1011	66.21	-	-
6460	CaLG8	CaTMS1044	66.21	-	-
6461	CaLG8	CaTMS674	66.21	-	-
6462	CaLG8	CaTMS853	66.21	-	-

6463	CaLG8	CaTMS717	66.24	-	-
6464	CaLG8	CaGMS1237	66.3	-	-
6465	CaLG8	CaTMS520	66.57	-	-
6466	CaLG8	CaSNP854	66.96	-	-
6467	CaLG8	CaSNP403	67.26	-	-
6468	CaLG8	CaTMS782	67.56	-	-
6469	CaLG8	CaTMS735	67.92	-	-
6470	CaLG8	NCPGR205	67.94	scaffold16958	2030
6471	CaLG8	CaTMS1048	67.96	-	-
6472	CaLG8	CaTSNP6248	68.73	-	-
6473	CaLG8	CaSNP2246	68.82	-	-
6474	CaLG8	CaSNP4446	69.02	-	-
6475	CaLG8	CaSNP5055	69.39	-	-
6476	CaLG8	CaSNP3080	69.45	scaffold00445	183282
6477	CaLG8	CaSNP4974	69.57	-	-
6478	CaLG8	CaTSNP6656	69.65	-	-
6479	CaLG8	CaSNP63	69.69	-	-
6480	CaLG8	CaTSNP6837	69.81	scaffold00139	303363
6481	CaLG8	CaSNP558	69.85	-	-
6482	CaLG8	CaSNP382	70.1	-	-
6483	CaLG8	CaSNP4473	70.25	-	-
6484	CaLG8	CaSNP2111	70.33	-	-
6485	CaLG8	CaSNP320	70.42	-	-
6486	CaLG8	CaSNP2001	70.88	scaffold00495	147008
6487	CaLG8	CaTMS1042	70.96	-	-
6488	CaLG8	CaTSNP7496	70.99	-	-
6489	CaLG8	CaTSNP8441	71.07	-	-
6490	CaLG8	CaTSNP7392	71.15	scaffold00136	332655
6491	CaLG8	CaSNP4303	71.23	-	-

6492	CaLG8	CaSNP4254	71.26	scaffold00963	97998
6493	CaLG8	CaTSNP6513	71.44	-	-
6494	CaLG8	CaSNP2912	71.54	-	-
6495	CaLG8	CaTSNP8592	71.89	-	-
6496	CaLG8	CaSNP1924	72.11	scaffold00162	268178
6497	CaLG8	CaTSNP7229	72.39	scaffold00848	106588
6498	CaLG8	CaGMS1	72.51	-	-
6499	CaLG8	CaTSNP6668	72.77	-	-
6500	CaLG8	CaTSNP8080	72.89	-	-
6501	CaLG8	CaTSNP8103	72.89	-	-
6502	CaLG8	CaTSNP6941	72.91	-	-
6503	CaLG8	CaTSNP7968	72.91	-	-
6504	CaLG8	CaTSNP8479	72.91	-	-
6505	CaLG8	CaTSNP7698	72.93	-	-
6506	CaLG8	CaTMS632	73.05	-	-
6507	CaLG8	CaTSNP6485	73.15	-	-
6508	CaLG8	CaTSNP6342	73.36	-	-
6509	CaLG8	CaTSNP7466	73.38	scaffold00244	275235
6510	CaLG8	CaTSNP6540	73.4	-	-
6511	CaLG8	CaTSNP7027	73.68	scaffold03781	22517
6512	CaLG8	CaTSNP7149	73.68	-	-
6513	CaLG8	CESSR181	73.91	-	-
6514	CaLG8	CaSNP3724	73.93	9132120	29903
6515	CaLG8	CaSNP1959	73.94	scaffold00186	257841
6516	CaLG8	CaSNP3791	74	scaffold01949	48738
6517	CaLG8	CaSNP4191	74.29	-	-
6518	CaLG8	CaSNP2242	74.31	-	-
6519	CaLG8	CaSNP3715	74.36	scaffold00554	144381
6520	CaLG8	CaSNP2311	75.1	scaffold00749	113803

6521	CaLG8	CaSNP3303	75.26	scaffold01374	73541
6522	CaLG8	CaSNP4693	75.28	scaffold01922	55954
6523	CaLG8	CaSNP2848	75.28	-	-
6524	CaLG8	CaSNP2909	75.28	scaffold01761	75282
6525	CaLG8	CaSNP3730	75.28	scaffold02767	33185
6526	CaLG8	CaSNP2646	75.3	scaffold01258	89485
6527	CaLG8	CaSNP4387	75.3	scaffold01110	111607
6528	CaLG8	CaSNP4078	75.31	scaffold02679	33862
6529	CaLG8	CaSNP4857	75.31	scaffold03013	52996
6530	CaLG8	CaSNP4201	75.31	scaffold00409	206618
6531	CaLG8	CaSNP3255	75.32	-	-
6532	CaLG8	CaSNP3871	75.32	scaffold02675	42738
6533	CaLG8	CaSNP4284	75.32	scaffold01169	125810
6534	CaLG8	CaSNP4705	75.32	-	-
6535	CaLG8	CaSNP4176	75.33	scaffold00822	135612
6536	CaLG8	CaSNP4982	75.33	scaffold02304	44846
6537	CaLG8	CaSNP4651	75.33	scaffold00979	115896
6538	CaLG8	CaSNP4226	75.35	-	-
6539	CaLG8	CaSNP2194	75.35	scaffold00850	111776
6540	CaLG8	CaSNP4035	75.35	scaffold02156	44967
6541	CaLG8	CaSNP4843	75.36	scaffold03117	27759
6542	CaLG8	CaSNP2916	75.39	-	-
6543	CaLG8	CaSNP4219	75.39	-	-
6544	CaLG8	CaSNP2927	75.4	scaffold00289	226831
6545	CaLG8	CaSNP3604	75.4	scaffold00302	219393
6546	CaLG8	CaSNP3930	75.41	scaffold02378	50502
6547	CaLG8	CaSNP4326	75.41	scaffold01873	51346
6548	CaLG8	CaSNP3706	75.44	scaffold02114	65688
6549	CaLG8	CaSNP2202	75.46	scaffold01389	76848

6550	CaLG8	CaSNP4595	75.49	scaffold02862	38819
6551	CaLG8	CaSNP3984	75.49	scaffold01172	94951
6552	CaLG8	CaSNP2535	75.51	scaffold01670	63213
6553	CaLG8	CaSNP3625	75.51	-	-
6554	CaLG8	CaSNP2907	75.55	scaffold01999	47105
6555	CaLG8	CaSNP3271	75.62	scaffold02068	59479
6556	CaLG8	CaSNP3155	75.62	scaffold01928	50485
6557	CaLG8	CaSNP3011	75.63	-	-
6558	CaLG8	CaSNP3359	75.63	-	-
6559	CaLG8	CaSNP3566	75.67	scaffold02228	41806
6560	CaLG8	CaSNP4777	75.7	scaffold00740	125013
6561	CaLG8	CaSNP4668	75.74	scaffold02692	46330
6562	CaLG8	CaSNP2106	75.77	-	-
6563	CaLG8	CaSNP3841	75.87	scaffold00805	110425
6564	CaLG8	CaTSNP6845	76.21	scaffold03092	33282
6565	CaLG8	CaSNP3352	76.22	scaffold01129	90719
6566	CaLG8	CaSNP2643	78.47	scaffold01654	63909
6567	CaLG8	TA125	78.76	-	-
6568	CaLG8	CaTMS1078	85.49	-	-
6569	CaLG8	CaSNP3758	88.07	scaffold03309	34664
6570	CaLG8	H3H022	88.42	-	-
6571	CaLG8	CaTMS974	90.25	-	-
6572	CaLG8	CaTMS760	90.4	-	-
6573	CaLG8	CaGMS17	90.52	-	-
6574	CaLG8	CaTMS818	90.52	-	-
6575	CaLG8	CaTMS603	91.27	-	-
6576	CaLG8	CaTMS609	91.27	-	-
6577	CaLG8	CESSR77	91.82	scaffold00636	132083
6578	CaLG8	CaTMS1037	92.58	-	-

6579	CaLG8	CaTMS1027	93.92	-	-
6580	CaLG8	CaSNP3479	94.76	scaffold01498	93317
6581	CaLG8	CaSNP4002	95	scaffold00879	101783
6582	CaLG8	CaSNP2630	95.29	-	-
6583	CaLG8	CaSNP3081	95.29	scaffold00447	190737
6584	CaLG8	CaSNP3898	95.29	scaffold04350	17332
6585	CaLG8	CaSNP4056	95.29	scaffold03710	23184
6586	CaLG8	CaSNP5128	95.29	scaffold03091	47220
6587	CaLG8	CaSNP3522	95.36	scaffold00540	151054
6588	CaLG8	CaSNP4669	95.36	scaffold00212	245341
6589	CaLG8	CaSNP4807	95.41	scaffold01691	71386
6590	CaLG8	CaSNP2424	95.5	scaffold00346	208431
6591	CaLG8	CaSNP2062	95.52	scaffold00808	121202
6592	CaLG8	CaSNP2595	95.52	scaffold00646	137874
6593	CaLG8	CaSNP4546	95.61	scaffold02402	44551
6594	CaLG8	CaSNP2430	95.93	-	-
6595	CaLG8	CaSNP4633	95.96	scaffold02458	56799
6596	CaLG8	CaGMS1235	96.26	-	-
6597	CaLG8	CaTSNP6769	96.53	9190626	66892
6598	CaLG8	CaTMS1067	97.92	-	-
6599	CaLG8	CaTMS663	101.1	-	-
6600	CaLG8	PIP6	101.1	-	-
6601	CaLG8	CaTMS640	101.8	-	-
6602	CaLG8	CaTMS1122	105.5	-	-
6603	CaLG8	NCPGR50	105.8	-	-
6604	CaLG8	CaTMS747	115.7	-	-
6605	CaLG8	CaTMS628	115.9	-	-
6606	CaLG8	CaTMS1066	116	-	-
6607	CaLG8	CaTMS956	116.1	-	-

6608	CaLG8	CaTMS1019	116.2	-	-
6609	CaLG8	CaTMS881	116.2	-	-
6610	CaLG8	CaTMS540	116.2	-	-
6611	CaLG8	CaTMS745	116.2	-	-
6612	CaLG8	CaTMS771	116.2	-	-
6613	CaLG8	CaGMS1281	116.2	-	-
6614	CaLG8	CaGMS1205	116.2	-	-
6615	CaLG8	CaTMS626	116.2	-	-
6616	CaLG8	CaGMS1178	116.2	-	-
6617	CaLG8	CaTMS570	116.3	-	-
6618	CaLG8	CaGMS1166	116.3	-	-
6619	CaLG8	CaTMS886	116.3	-	-
6620	CaLG8	CaTMS1073	116.3	-	-
6621	CaLG8	CaGMS1131	116.3	-	-
6622	CaLG8	CaGMS1130	116.3	-	-
6623	CaLG8	CaGMS1274	116.3	-	-
6624	CaLG8	CaTMS607	116.3	-	-
6625	CaLG8	CaGMS1267	116.3	-	-
6626	CaLG8	CaTMS808	116.3	-	-
6627	CaLG8	CaTMS1014	116.3	-	-
6628	CaLG8	CaGMS1256	116.3	-	-
6629	CaLG8	CaTMS1021	116.3	-	-
6630	CaLG8	CaTMS669	116.3	-	-
6631	CaLG8	CaGMS1308	116.3	-	-
6632	CaLG8	CaTMS849	116.3	-	-
6633	CaLG8	CaTMS587	116.3	-	-
6634	CaLG8	CaTMS1030	116.3	-	-
6635	CaLG8	CaTMS533	116.3	-	-
6636	CaLG8	CaTMS605	116.3	-	-

6637	CaLG8	CaGMS1319	116.3	-	-
6638	CaLG8	CaTMS943	116.3	-	-
6639	CaLG8	CaGMS1291	116.3	-	-
6640	CaLG8	CaTMS1028	116.3	-	-
6641	CaLG8	CaTMS1093	116.3	-	-
6642	CaLG8	CaTMS602	116.3	-	-
6643	CaLG8	CaTMS836	116.3	-	-
6644	CaLG8	CaTMS509	116.3	-	-
6645	CaLG8	CaTMS1072	116.3	-	-
6646	CaLG8	CaTMS778	116.3	-	-
6647	CaLG8	CaGMS1317	116.3	-	-
6648	CaLG8	CaGMS1270	116.3	-	-
6649	CaLG8	CaGMS1290	116.3	-	-
6650	CaLG8	CaGMS1304	116.3	-	-
6651	CaLG8	CaTMS1016	116.3	-	-
6652	CaLG8	CaTMS1077	116.3	-	-
6653	CaLG8	CaTMS1083	116.3	-	-
6654	CaLG8	CaTMS1103	116.3	-	-
6655	CaLG8	CaTMS578	116.3	-	-
6656	CaLG8	CaTMS610	116.3	-	-
6657	CaLG8	CaTMS611	116.3	-	-
6658	CaLG8	CaTMS659	116.3	-	-
6659	CaLG8	CaTMS777	116.3	-	-
6660	CaLG8	CaTMS897	116.3	-	-
6661	CaLG8	CaTMS907	116.3	-	-
6662	CaLG8	CaTMS948	116.3	-	-
6663	CaLG8	CaGMS27	116.3	-	-
6664	CaLG8	CaGMS1165	116.3	-	-
6665	CaLG8	CaTMS935	116.3	-	-

6666	CaLG8	CaGMS1220	116.3	-	-
6667	CaLG8	CaTMS1120	116.3	-	-
6668	CaLG8	CaTMS947	116.3	-	-
6669	CaLG8	CaGMS29	116.3	-	-
6670	CaLG8	CaGMS1171	116.3	-	-
6671	CaLG8	CaTMS1063	116.3	-	-
6672	CaLG8	CaTMS569	116.3	-	-
6673	CaLG8	CaTMS592	116.3	-	-
6674	CaLG8	CaTMS967	116.3	-	-
6675	CaLG8	CaGMS28	116.3	-	-
6676	CaLG8	CaTMS643	116.3	-	-
6677	CaLG8	CaTMS845	116.3	-	-
6678	CaLG8	CaTMS971	116.4	-	-
6679	CaLG8	CaGMS1129	116.9	-	-
6680	CaLG8	CaTMS923	116.9	-	-
6681	CaLG8	CaTMS814	117.2	-	-
6682	CaLG8	CaTMS793	117.3	-	-
6683	CaLG8	CaTMS645	117.6	-	-
6684	CaLG8	CaTMS826	118	-	-
6685	CaLG8	CaTMS662	118.1	-	-
6686	CaLG8	CaTMS665	118.1	-	-
6687	CaLG8	CaTMS989	118.1	-	-
6688	CaLG8	CaTMS1062	118.2	-	-
6689	CaLG8	PIP194	118.3	-	-
6690	CaLG8	CaTMS625	118.6	-	-
6691	CaLG8	CaTMS553	119.9	-	-
6692	CaLG8	TA196	121.2	-	-
6693	CaLG8	CaTMS653	121.3	-	-
6694	CaLG8	NCPGR137	122.4	-	-

6695	CaLG8	CaTMS646	124.7	-	-
6696	CaLG8	PIP70	139	-	-
6697	CaLG8	CaTMS681	139.2	-	-
6698	CaLG8	PIP126	163.6	-	-

Table S3: Details of synteny analysis of chickpea with *M. truncatula*, *G. max* and *P. vulgaris*

A

	**Mt1**	**Mt2**	**Mt3**	**Mt4**	**Mt5**	**Mt6**	**Mt7**	**Mt8**	**Scaffold**	**Total**
**CaLG1**	11	181	5	11	5	1	3	4	3	**224**
**CaLG2**	3	4	6	5	73	34	6	1	3	**135**
**CaLG3**	22	10	6	9	6	5	194	9	4	**265**
**CaLG4**	219	5	15	13	7	7	12	9	5	**292**
**CaLG5**	9	3	212	7	13	4	10	17	1	**276**
**CaLG6**	7	7	19	143	11	3	9	78	7	**284**
**CaLG7**	20	14	13	118	12	7	10	67	6	**267**
**CaLG8**	14	12	10	15	85	23	17	16	1	**193**
**Total**	**305**	**236**	**286**	**321**	**212**	**84**	**261**	**201**	**30**	**1936**

B

	**Gm1**	**Gm2**	**Gm3**	**Gm4**	**Gm5**	**Gm6**	**Gm7**	**Gm8**	**Gm9**	**Gm10**	**Gm11**	**Gm12**	**Gm13**
**CaLG1**	0	0	0	1	0	5	1	6	14	1	2	17	32
**CaLG2**	2	11	0	0	1	0	1	2	1	0	3	0	4
**CaLG3**	4	8	21	1	2	4	2	9	12	8	3	4	3
**CaLG4**	3	7	6	5	4	6	3	1	3	45	5	1	7
**CaLG5**	2	4	4	29	7	43	2	9	3	2	13	3	7
**CaLG6**	4	2	2	3	22	4	12	28	6	1	11	23	8
**CaLG7**	5	7	3	6	17	5	16	7	6	5	2	4	9
**CaLG8**	17	4	4	2	5	5	5	10	9	1	24	1	4
**Total**	**37**	**43**	**40**	**47**	**58**	**72**	**42**	**72**	**54**	**63**	**63**	**53**	**74**

C

	Pv1	Pv2	Pv3	Pv4	Pv5	Pv6	Pv7	Pv8	Pv9	Pv10	Pv11	Scaffold	Total
**CaLG1**	4	4	1	1	28	21	4	2	11	2	7	0	**85**
**CaLG2**	3	2	4	11	1	1	0	19	2	2	0	1	**46**
**CaLG3**	22	1	5	3	4	7	15	28	5	4	2	1	**97**
**CaLG4**	23	5	8	2	1	1	57	5	5	1	5	0	**113**
**CaLG5**	17	10	5	3	8	13	4	8	46	1	1	0	**116**
**CaLG6**	10	39	3	3	7	5	0	8	2	14	27	0	**118**
**CaLG7**	2	11	45	1	7	7	11	5	9	10	7	3	**118**
**CaLG8**	4	35	10	8	4	5	4	6	4	7	3	0	**90**
**Total**	**85**	**107**	**81**	**32**	**60**	**60**	**95**	**81**	**84**	**41**	**52**	**5**	**783**
